# Causal Relationships Between Leukocyte Subsets and Adverse Fetal Outcomes: A Mendelian Randomization Study

**DOI:** 10.1155/mi/6349687

**Published:** 2024-12-26

**Authors:** Hong Chen, Li-Zhen Shao, Ying-Xiong Wang, Zhi-Jie Han, Yong-Heng Wang, Xia Li, Jing-Yu Chen, Tai-Hang Liu

**Affiliations:** ^1^Department of Bioinformatics, School of Basic Medical Sciences, Chongqing Medical University, Chongqing 400016, China; ^2^Joint International Research Laboratory of Reproduction and Development, Chongqing Medical University, Chongqing 400016, China; ^3^Department of Ultrasound, Children's Hospital of Chongqing Medical University, 136 Zhongshan 2nd Road, Chongqing 400014, China

**Keywords:** adverse fetal outcomes, GWAS, leukocyte, Mendelian randomization

## Abstract

**Background:** The tolerance and dynamic regulation of the maternal immune system during pregnancy are pivotal for ensuring fetal health. Immune cell subsets play a complex and crucial role in this process, closely linked to the neonatal health status. Despite recognizing the significance of dysregulation in the quantity and activity of immune cells in neonatal disease occurrence, their specific roles remain elusive, resulting in a dearth of clinically viable interventions for immune-mediated neonatal diseases.

**Materials and Methods:** Employing two-sample Mendelian randomization (MR) methodology, this study systematically investigated 446 leukocyte features (*N* = 500,675), including leukocyte subsets, absolute cell (AC) counts, and morphological parameters (MP) and their correlation with seven adverse fetal outcomes (*N* = 1,100,458), encompassing fetal growth restriction (FGR), preterm birth (PTB), neonatal jaundice (NNJ), digestive system disorders of fetus and newborn (DSDFN), hemorrhagic and hematological disorders of fetus and newborn (HDFN), respiratory distress of newborn (RDN), and transitory disorders of metabolism specific to fetus and newborn (TDMSFN).

**Results:** The results unveiled significant causal relationships between 301 leukocyte subsets and these seven adverse fetal outcomes, with 259, 245, 15, 44, 11, 32, and 68 pairs of notable associations for each adverse outcome, respectively. Furthermore, the study highlighted potential pathogenic mechanisms underlying the mutual influence among neonatal diseases. MR results indicated FGR as a robustly correlated risk factor for PTB and NNJ and showed a reciprocal causal relationship between NNJ and FGR. PTB exhibited a positive correlation with HDFN.

**Conclusions:** This study provided profound insights into the intricate regulatory mechanisms of leukocyte subsets in neonatal diseases, paving the way for new avenues in the diagnosis and treatment of associated disorders.

## 1. Introduction

Successful pregnancy relies on the delicate equilibrium of the maternal immune system, concurrently ensuring tolerance to the semi-allogeneic fetus and providing effective protection against pathogen invasion. Sustaining this balance involves coordinated changes in various leukocyte subsets. Numerous studies have unequivocally indicated that aberrations in leukocyte activity and quantity can lead to adverse pregnancy outcomes. For instance, the quantity of Regulatory T cells (Tregs) is closely associated with maintaining maternal–fetal tolerance [1–4], yet a reduction in their numbers in the placenta may contribute to the occurrence of gestational diabetes mellitus (GDM) [5]. During pregnancy, the excessive activation of B cells, monocytes, and Natural Killer cells (NK) has been confirmed to be closely correlated with the development of preeclampsia (PE) [6]. Furthermore, decidual NK (dNK) cells, with the ability to modulate the quantity of cytotoxic subsets, a decrease in their numbers or impairment of their immunoregulatory functions is linked to recurrent pregnancy loss (RPL) [7–10]. However, the dysregulation of leukocyte subsets not only triggers maternal pregnancy-related disorders but also significantly impacts fetal health and development. Existing research has indicated that abnormal increases and activation of monocytes and macrophages may lead to adverse fetal outcomes such as fetal growth restriction (FGR), preterm birth (PTB), and metabolic disturbances [11].

Neonatal diseases such as FGR, PTB, and respiratory distress of newborn (RDN), exert profound impacts on both the short-term and long-term health of fetuses, posing potential life-threatening risks. FGR, recognized as a severe obstetric condition, affects 5%–10% of pregnant women [12] and significantly contributes to a substantial proportion of perinatal deaths, accounting for 30% of stillbirths [13]. Moreover, FGR plays a pivotal role in the prevalence of PTB and intrapartum asphyxia [14]. The secondary complications stemming from FGR may persist into adolescence and adulthood, triggering adverse developmental outcomes such as neurodevelopmental impairment, cardiovascular issues, endocrine disruptions, and metabolic disorders [15, 16]. Simultaneously, PTB is a leading cause of global neonatal morbidity and mortality, impacting ~15 million fetuses annually [17]. PTB induces the immature development of various organ systems in fetuses, predisposing them susceptible to multiple health issues, including respiratory distress syndrome [18], gastrointestinal disorders [19], and hematologic diseases [20]. The etiology of these diseases is intricate, with existing research suggesting a correlation between adverse fetal outcomes and disruptions in the immune system homeostasis. For example, RDN may be associated with abnormal immune responses [21]. Ward et al. [22] discovered that neutrophil-driven placental inflammation in the maternal body can impede the normal development of the embryonic heart. Additionally, in PTB, the downregulation of Foxp3 Treg is closely related to the occurrence of necrotizing enterocolitis [23], a pathology often leading to severe sepsis and endangering the lives of neonates [24]. Notably, abnormal activation of macrophages and neutrophils occurs in the early stages of neonatal sepsis [25]. Despite the widely acknowledged importance of the immune system in fetal development, there remains a paucity of systematic research in this field.

With a dedicated focus on this concern, the two-sample Mendelian randomization (MR) method, a robust causal inference approach widely employed in epidemiology and genetics, was employed in this study [26–28]. This method leverages the random distribution of genetic variations to explore causal relationships between specific exposure factors and outcomes, especially in intricate scenarios involving interactions between environmental and genetic factors [29, 30]. Therefore, gene variations linked to 446 leukocyte features were utilized as instrumental variables (IVs), aiming to assess their causal associations with the aforementioned adverse fetal outcomes. By delving into the roles of leukocyte subsets in maternal-fetal interactions, this research aims to gain a more comprehensive understanding of the mechanisms underpinning neonatal health issues. This not only contributes to enhancing early diagnosis of complications such as FGR but also lays a theoretical foundation for the development of more effective preventive and therapeutic strategies in clinical practice.

## 2. Materials and Methods

### 2.1. Study Design

**Figure 1:**
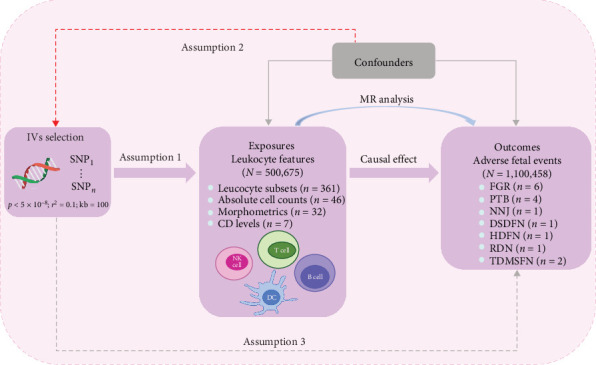
Flowchart depicting the overall design of MR analysis. DSDFN, digestive system disorders of fetus and newborn; FGR, fetal growth restriction; HDFN, hemorrhagic and hematological disorders of fetus and newborn; IVs, instrumental variables; MR, Mendelian randomization; NNJ, neonatal jaundice; PTB, preterm birth; RDN, respiratory distress of newborn; TDMSFN, transitory disorders of metabolism specific to fetus and newborn.

A diverse array of publicly available datasets from genome-wide association studies (GWAS) on risk factors and diseases was harnessed in the two-sample MR study to explore whether exposure demonstrates a causal association with disease occurrence. In this design method, genetic variation was utilized as IVs to address challenges such as confounders and reverse causation inherent in observational studies, leading to more robust causal inferences. The design rested on three foundational assumptions. First, genetic variation was intricately linked to the exposure of interest; second, genetic variation remained unaffected by other confounding factors; and lastly, genetic variation did not directly impact outcomes except through its association with the exposure. Approval from institutional review boards in respective areas was obtained for all studies, obviating the need for additional ethical review. Employing a two-sample MR method, this study investigated the causal relationship between leukocyte subsets and adverse fetal outcomes (Figure 1).

### 2.2. Data Sources

To ensure the absence of misleading associations between genetic variants and results, participants of European ancestry were chosen as the data source, mitigating potential discrepancies in genetic variant frequencies across diverse populations. The GWAS-aggregated data for 446 leucocyte features (*N* = 500,675), comprising leukocyte subsets (*n* = 361), absolute cell (AC) counts (*n* = 46), morphological parameters (MP; *n* = 32), and Cluster of Differentiation (CD) levels (*n* = 7), seven adverse fetal outcomes (*N* = 1,100,458), including FGR (*n* = 5), PTB (*n* = 4), neonatal jaundice (NNJ) (*n* = 1), digestive system disorders of fetus and newborn (DSDFN) (*n* = 1), hemorrhagic and hematological disorders of fetus and newborn (HDFN) (*n* = 1), RDN (*n* = 1) and transitory disorders of metabolism specific to fetus and newborn (TDMSFN) (*n* = 2) were obtained from publicly accessible databases, containing the open GWAS project (https://gwas.mrcieu.ac.uk/datasets/) and GWAS Catalog (https://www.ebi.ac.uk/gwas/downloads/summary-statistics).

### 2.3. Selection Criteria of IVs

The criteria for selecting IVs were implemented through the following steps: First, all single nucleotide polymorphism (SNPs) linked to 446 leukocyte features and seven adverse fetal outcomes were required to attain GWAS significance (*p* < 5 × 10^−8^), this stringent criterion effectively guarded against potential biases stemming from weak IVs. Second, the linkage disequilibrium (LD) coefficient (*r*^2^) was set to 0.1, and the maximum LD region span was established as 100 kb. Finally, the comprehensive information about the SNPs, including rsID, beta value, standard error (SE), *p* − value, effect allele (EA), other allele (OA), and EA frequency (EAF), was systematically summarized for subsequent MR analysis.

### 2.4. Statistical Analysis

A variety of methodologies were employed in two-sample MR analyses to estimate causal effects, primarily utilizing the inverse variance weighting (IVW) method, and to a lesser extent, MR Egger, Wald ratio, and maximum likelihood methods. To mitigate potential false positives, the false discovery rate (FDR) method was applied to correct *p* − values across different trait types and panels. Heterogeneity in SNPs across the two samples was assessed through Cochran's *Q* test, with significance set at *p* < 0.05, indicating substantial heterogeneity. In the absence of heterogeneity or pleiotropy, the IVW approach results were prioritized. In the presence of heterogeneity, the random effects model of IVW or Weighted Median could be employed instead. However, if pleiotropy was detected, priority was given to outcomes obtained from MR-Egger methods [31]. Utilizing these diverse methods for result comparison enhanced result consistency and reliability [32, 33]. All data underwent statistical analyses using R software version R-4.3.1 (https://www.r-project.org/).

## 3. Results

### 3.1. Causal Relationships Between 301 Leukocyte Subsets and Seven Adverse Fetal Outcomes

**Figure 2:**
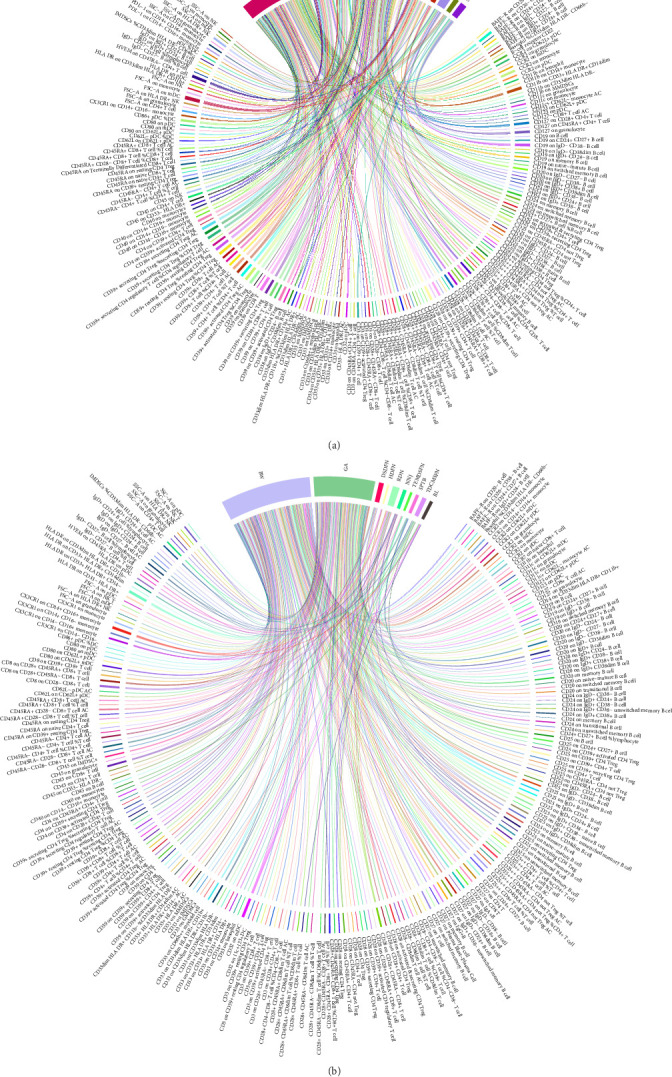
The chordal graph for the positive–negative trend causality between 301 leukocyte subsets and seven adverse fetal outcomes based on MR analysis. (A) Leukocyte subsets positively associated with adverse fetal outcomes. (B) Leukocyte subsets negatively associated with adverse fetal outcomes. Line thickness represents the strength of the correlation. BL, birth length; BW, birth weight; DSDFN, digestive system disorders of fetus and newborn; GA, gestational age; HDFN, hemorrhagic and hematological disorders of fetus and newborn; NNJ, neonatal jaundice; RDN, respiratory distress of newborn; SPTB, spontaneous preterm birth; TDCMSFN, transitory disorders of carbohydrate metabolism specific to fetus and newborn; TEMDSFN, transitory endocrine and metabolic disorders specific to fetus and newborn.

To systematically explore the relationships between various leukocyte subsets and adverse fetal outcomes, GWAS data comprising 446 different features related to leukocytes and seven clinically common adverse fetal outcomes were initially compiled from open GWAS and GWAS Catalog databases. Encompassing leukocyte subsets (*n* = 361), AC counts (*n* = 46), MP (*n* = 32), and CD levels (*n* = 7), the leukocyte features were examined alongside adverse fetal outcomes, including FGR (*n* = 5), PTB (*n* = 4), NNJ (*n* = 1), DSDFN (*n* = 1), HDFN (*n* = 1), RDN (*n* = 1), and TDMSFN (*n* = 2) (Supporting Information 1: Table S1). Subsequently, distinct cell surface markers were employed to classify leukocyte subsets. For instance, T cells were divided into 113 subsets based on CD25, CD28, and CD39; B cells were categorized into 78 subsets using B cell-activating factor receptor (BAFF-R), CD24, and immunoglobulin D (IgD); dendritic cells were classified into 24 subsets using CD80 and CD62L; monocytes into 22 subsets based on CCR2 and CX3CR1; myeloid cells into 31 subsets using markers such as Human Leukocyte Antigen DR (HLA DR) and CD33 (Supporting Information 1: Table S1). In the selection of IVs, stringent criteria (*p* < 5 × 10^−8^, *r*^2^ = 0.1, kb = 100) was applied, resulting in the identification of 9281 SNPs for MR analysis with the aforementioned seven adverse fetal outcomes (Supporting Information 1: Table S2). To mitigate potential false positives, the FDR method for multiple testing correction was employed to further adjust *p*-values in the MR analysis results for different leukocyte subsets and adverse fetal outcomes. Following FDR correction, a total of 674 significant causal associations were identified between 301 leukocyte subsets and the seven adverse fetal outcomes. Among these associations, 332 implied an increased risk for adverse outcomes, while 342 indicated a protective effect against these adverse outcomes (*p*-value after being adjusted by the FDR method (P.FDR)< 0.05, *pval*.*y* > 0.05, Q_*pval* > 0.05; Figure 2A,B).

### 3.2. Causal Relationships Between Leukocyte Subsets and FGR

**Figure 3:**
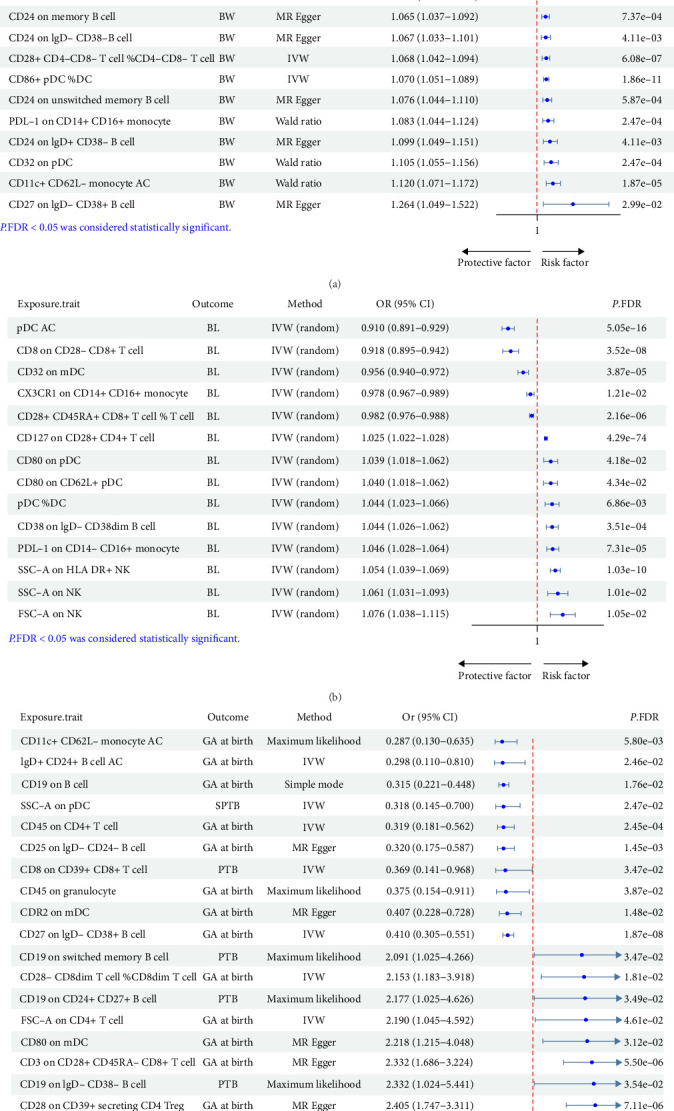
Forest plot for the causal relationships between leukocyte subsets and FGR as well as PTB. (A) Causal effects of the top 10 risk factors and the top 10 protective factors on BW. (B) Casual relationships between leukocyte subsets and BL. (C) Causal effects of the top 10 risk factors and the top 10 protective factors on PTB. BL, birth length; BW, birth weight; CI, confidence interval; GA, gestational age; IVW, Inverse variance weighted; OR, odds ratio; PTB, preterm birth; SPTB, spontaneous preterm birth.

Currently, the clinical assessments of FGR primarily rely on indicators such as fetal birth weight (BW) and birth length (BL) [34]. Through two-sample MR analyses and subsequent FDR correction, significant causal relationships were identified between 245 leukocyte subsets and BW. Of these, 105 subsets were determined to be risk factors, while 140 subsets were identified as protective factors. Predominantly observed in 91 T-cell subsets, followed by 67 B-cell subsets, 26 myeloid cell subsets, 21 monocyte subsets, 24 dendritic cells (DC) subsets, and four NK-cell subsets (Supporting Information 1: Table S3). The top 20 MR results for BW are displayed in Figure 3A. Notably, the strongest significant positive and negative correlations were observed in CD39 on CD39+ CD8+ T cell (odds ratio [OR] = 1.016, 95% confidence interval [CI] = 1.013–1.019, *P*.FDR = 2.40E-42) and IgD+ CD24+ B cell %lymphocyte (OR = 0.960, 95% CI = 0.956–0.965, *P*.FDR = 3.25E-63), respectively. Subsequent MR analyses unveiled discernible causal relationships between 14 leukocyte subsets and BL. Among these, nine subsets were identified as risk factors and five subsets were identified as protective factors, primarily including five DC-cell subsets, followed by three T-cell subsets, three NK-cell subsets, two monocyte subsets, and one B-cell subset. The strongest significant positive and negative correlations were observed in CD127 on CD28+ CD4+ T cell (OR = 1.025, 95% CI = 1.022–1.028, *P*.FDR = 4.29E-74) and plasmacytoid dendritic cell (pDC) AC (OR = 0.910, 95% CI = 0.891–0.929, *P*.FDR = 5.05E-16), respectively (Figure 3B). Notably, CD127 on CD28+ CD4+ T cell, pDC %DC, PDL-1 on CD14- CD16+ monocyte, and SSC-A on HLA DR + NK were identified as risk factors for both BW and BL, whereas pDC AC, CD8 on CD28- CD8+ T cell, CD32 on mDC, and CX3CR1 on CD14+ CD16+ monocyte were protective factors for both outcomes (Table 1). This finding suggested that the occurrence of FGR may involve multiple mechanisms influenced by leukocyte factors.

### 3.3. Causal Relationships Between Leukocyte Subsets and PTB

PTB is a leading cause of global neonatal morbidity and mortality [17]. Following a systematic analysis, a total of 245 leukocyte subsets were found to be significantly associated with PTB (*P*.FDR <0.05; Supporting Information 1: Table S4). Traits within the T-cell panel retained prominence, with a substantial 43% significant pairwise correlation observed among T-cell subsets (105 T-cell pairs vs. 245 total pairs). This was followed by 68 B-cell subsets, 35 myeloid cell subsets, 17 monocyte subsets, 16 DC subsets, and four NK-cell subsets. Within T cells, 54 subsets displayed positive correlations, primarily characterized by CD28-/CD39+, while 51 subsets exhibited negative correlations, mainly associated with CD25+/CD28+. In B cells, 28 subsets demonstrated positive correlations with PTB, primarily involving BAFF-R/IgD on B cell, while 40 subsets showed negative correlations, mainly associated with CD25+/CD38+ B-cell subsets. Among myeloid cells, 11 subsets exhibited positive correlations, primarily characterized by CD33 dim/HLA DR, and 16 subsets displayed negative correlations, mainly associated with CD33+/HLA DR. The top 20 MR results for PTB were presented in Figure 3C. Additionally, CD25++ CD45RA+ CD4 not Treg %CD4+ T cell (OR = 1.214, 95% CI = 1.190–1.239, *P*.FDR = 3.35E-75) and CD45RA− CD4+ T cell %CD4+ T cell (OR = 0.770, 95% CI = 0.746–0.794, *P*.FDR = 2.97E-57) exhibited the strongest significant positive and negative correlations, respectively.

### 3.4. The Exploration of Leukocyte Subsets Associations With Neonatal Disorders: Insights From MR Analysis on NNJ, HDFN, DSDFN, RDN, and Metabolic-Related Diseases

**Figure 4:**
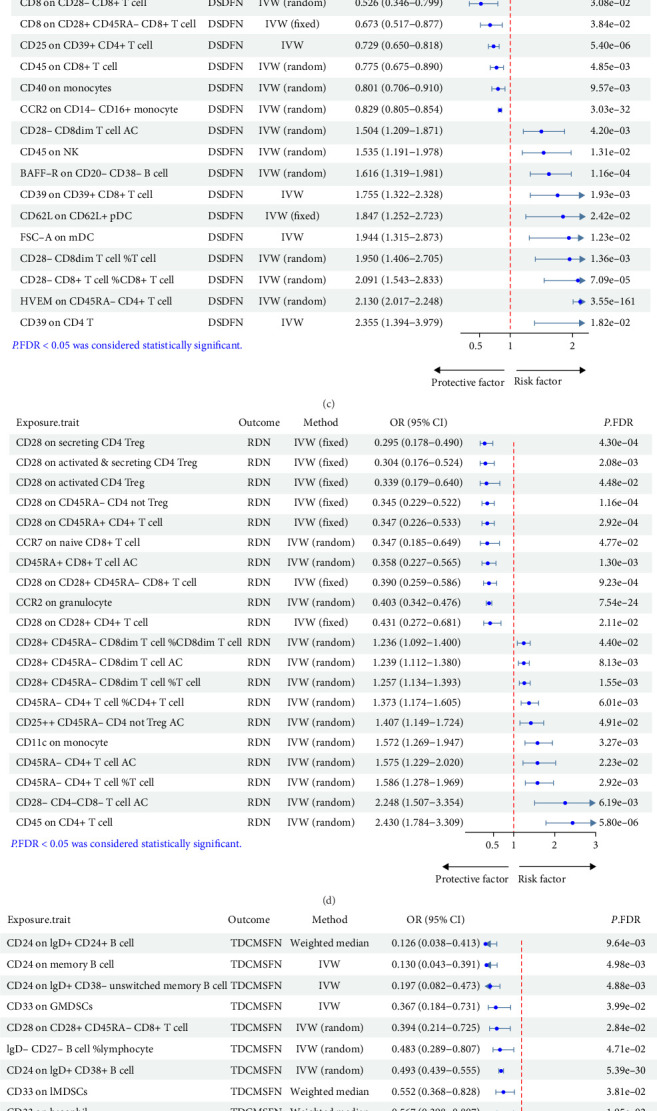
Forest plot depicting the causal relationships between leukocyte subsets and common clinical neonatal diseases. (A and B) Casual relationships between leukocyte subsets and NNJ as well as HDFN, respectively. (C–F) Causal effects of the top 10 risk factors and the top 10 protective factors on DSDFN, RDN, TDCMSFN, and TEMDSFN, respectively. CI, confidence interval; DSDFN, digestive system disorders of fetus and newborn; HDFN, hemorrhagic and hematological disorders of fetus and newborn; IVW, inverse variance weighted; NNJ, neonatal jaundice; OR, odds ratio; RDN, respiratory distress of newborn; TDCMSFN, transitory disorders of carbohydrate metabolism specific to fetus and newborn; TEMDSFN, Transitory endocrine and metabolic disorders specific to fetus and newborn.

Leukocytes, serving as the primary executors in the establishment and regulation of the immune system, play a crucial role in ensuring the development of various organs in fetuses. Jaundice, with an incidence rate as high as 65% in newborns, is primarily caused by insufficient liver capacity in bilirubin metabolism [35]. Among the 15 leukocyte subsets identified with significant causal relationships to NNJ, six subsets were identified as risk factors, while nine subsets were deemed protective factors. This included primarily six T-cell subsets, followed by four B-cell subsets, three myeloid cell subsets, and two monocyte subsets (Figure 4A, Supporting Information 1: Table S5). Interestingly, MR results indicated that CD11b on CD33+ HLA DR + CD14dim (OR = 1.795, 95% CI = 1.653–1.950, *P*.FDR = 7.87E-41) was considered a risk factor for NNJ, while CD11b on basophil (OR = 0.596, 95% CI = 0.565–0.628, *P*.FDR = 3.71E-79) was identified as a protective factor for NNJ, exhibiting the strongest significant positive and negative correlations, respectively. This suggested that immune dysregulation mediated by leukocyte subsets also plays a crucial role in the occurrence of jaundice.

Subsequently, 11 leukocyte subsets were observed to exhibit significant causal relationships with HDFN, including four B-cell subsets, three monocyte subsets, two T-cell subsets, and two myeloid cell subsets (Figure 4B, Supporting Information 1: Table S5). Herpesvirus entry mediator (HVEM) on CD45RA− CD4+ T cell (OR = 0.770, 95% CI = 0.755–0.786, *P*.FDR = 9.28E-148) emerged as the most significantly protective feature among the five protective factors. Moreover, among the six risk factors, CD11b on CD33+ HLA DR + CD14dim (OR = 1.547, 95% CI = 1.447–1.654, *P*.FDR = 3.31E-34) exhibited the strongest significance, indicating a substantial overlap with the results associated with NNJ. This suggested that this feature may possess significant predictive value, potentially extending to the clinical assessment of hemolytic diseases in newborns.

While research on digestive system disorders in neonates remains somewhat limited, their potential risks cannot be underestimated. In the analysis of 285 investigated leukocyte subsets, 44 subsets were found to exhibit significant causal relationships with DSDFN. Of these, 33 subsets were identified as risk factors, and 11 subsets were recognized as protective factors, with T-cell subsets comprising 73% of the dominance (32 T-cell pairs vs. 44 total pairs). This included four DC subsets, three monocyte subsets, two B-cell subsets, two myeloid cell subsets, and one NK-cell subset (Supporting Information 1: Table S5). Notably, the strongest significant positive and negative correlations were observed in HVEM on CD45RA- CD4+ T cell (OR = 2.130, 95% CI = 2.017–2.248, *P*.FDR = 3.55E-161) and CCR2 on CD14- CD16+ monocyte (OR = 0.829, 95% CI = 0.805–0.854, *P*.FDR = 3.03E-32), respectively. The top 20 MR results for DSDFN are displayed in Figure 4C. It is noteworthy that, in the MR analysis results, both resting and activated CD39+ Treg subsets were positively correlated with DSDFN; however, CD28+ Treg-related subsets showed negative correlations with DSDFN (Table 2). This suggested that the quantity and activity levels of Tregs may play a crucial regulatory role in the complex pathogenesis of DSDFN.

Respiratory distress stands as a predominant cause for neonatal transfers to the neonatal intensive care unit (NICU), entailing the peril of hypoxemia and the prospective detriment to essential organs such as the brain and heart, ultimately resulting in unfavorable consequences, including mortality. MR results showed that 32 leukocyte subsets were discerned to exhibit noteworthy causal associations with RDN. Of these, 12 subsets were identified as risk factors, while 20 subsets were protective factors. T-cell subsets, constituting 78% dominance (25 T-cell pairs vs. 32 total pairs), played a predominant role, accompanied by three granulocyte subsets, two monocyte subsets, two B-cell subsets, and one DC subset (Supporting Information 1: Table S5). Noteworthy among these findings were CD28 on CD39+ resting CD4 Treg (OR = 1.178, 95% CI = 1.162–1.193, *P*.FDR = 3.22E-129) and CCR2 on granulocyte (OR = 0.403, 95% CI = 0.342–0.476, *P*.FDR = 7.54E-24), exhibiting the strongest significant positive and negative correlations, respectively. The top 20 MR results for RDN are shown in Figure 4D. Intriguingly, both CD28 on resting CD4 Treg (OR = 0.470, 95% CI = 0.317–0.697, *P*.FDR = 1.31E-02) and CD28 on activated and secreting CD4 Treg (OR = 0.304, 95% CI = 0.176–0.524, *P*.FDR = 2.08E-03) exhibited negative correlations with RDN (Table 3). This implied potential regulatory functions of CD28+ Treg subsets, thereby paving novel avenues for therapeutic interventions in the realm of disease management.

For the MR analysis of neonatal metabolic-related diseases, 42 leukocyte subsets were found to exhibit significant correlations with transitory disorders of carbohydrate metabolism specific to fetus and newborn (TDCMSFN). Among these, 29 leukocyte subsets were identified as risk factors, while 13 subsets were classified as protective factors, encompassing 24 T-cell subsets, five B-cell subsets, five DC subsets, four myeloid-cell subsets, three NK-cell subsets, and one monocyte subset (Supporting Information 1: Table S5). Notably, HVEM on CD45RA- CD4+ T cell (OR = 2.342, 95% CI = 2.165–2.534, *P*.FDR = 1.40E-96) exhibited the strongest significant positive correlation, and CD24 on IgD+ CD38+ B cell (OR = 0.493, 95% CI = 0.439–0.555, *P*.FDR = 5.39E-30) demonstrated the strongest significant negative correlation. The top 20 MR results for RDN are shown in Figure 4E. It is worth noting that HVEM on CD45RA- CD4+ T cell was a risk factor for DSDFN but a protective factor for HDFN. This highlighted the intricate regulation of immune cells in neonatal diseases, necessitating deeper investigation and exploration in future studies. Additionally, 26 leukocyte subsets were investigated to exhibit significant correlations with transitory endocrine and metabolic disorders specific to fetus and newborn (TEMDSFN), including 21 risk factors and five protective factors, mainly comprised of 18 T-cell subsets, two B-cell subsets, two DC subsets, one NK-cell subset, one monocyte subset, and one granulocyte subset (Supporting Information 1: Table S5). The top 20 MR results for RDN are depicted in Figure 4F. Importantly, 14 leukocyte subsets demonstrated significant correlations with both TDCMSFN and TEMDSFN, with the correlations exhibited by each subset remaining consistent (Supporting Information 1: Table S5).

**Figure 5:**
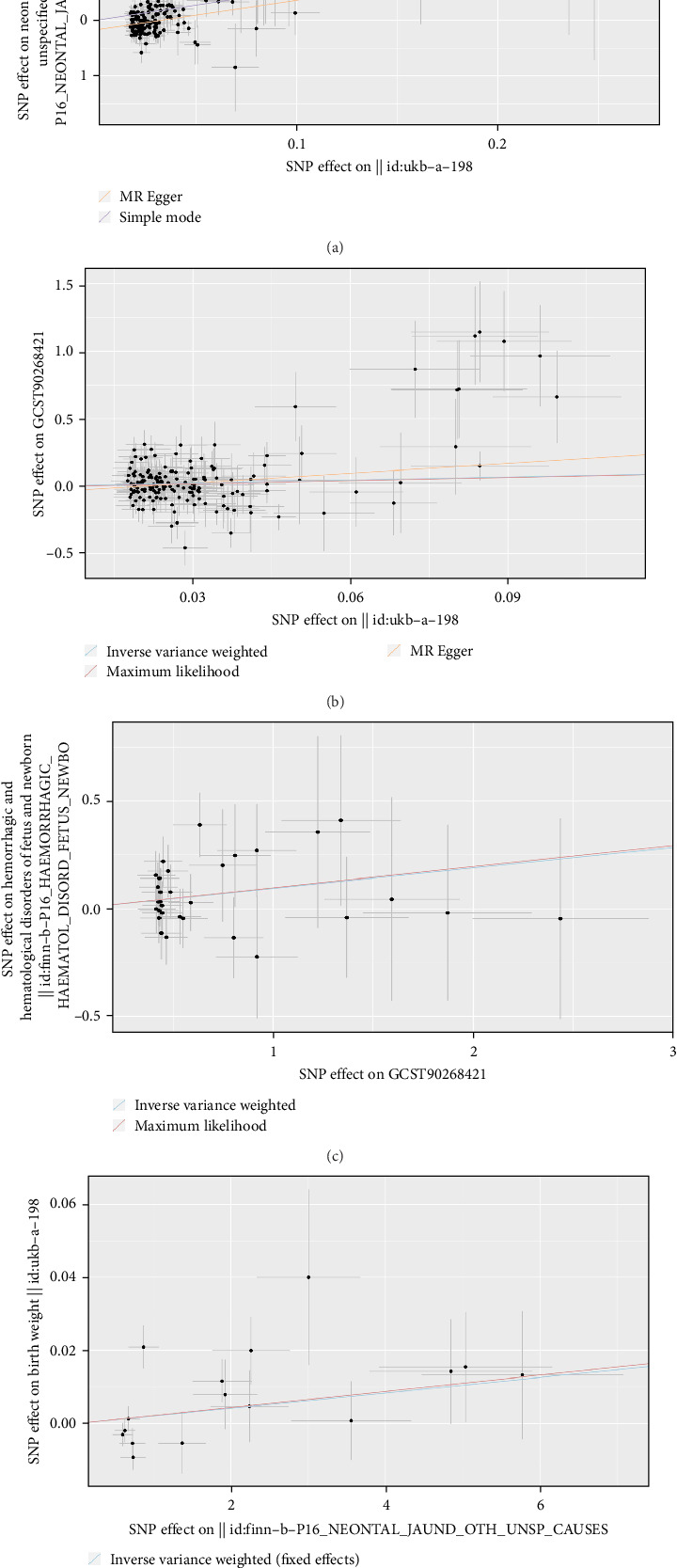
Scatter plot for the relationship between the SNP effect size of causal adverse fetal outcomes (*x*-axis) and another one (*y*-axis). (A and B) BW on NNJ and PTB, respectively. (C) PTB on HDFN. (D) NNJ on BW. BW, birth weight; HDFN, hemorrhagic and hematological disorders of fetus and newborn; NNJ, neonatal jaundice; PTB, preterm birth; SNP, single nucleotide polymorphism.

For further exploration of the genetic variations underlying the associations between FGR and PTB with secondary complications in newborns, MR analysis was employed. The results (Supporting Information 1: Table S6) unveiled a significant positive correlation between BW and NNJ as well as PTB (Figure 5A,B) but exhibited significant negative correlations with DSDFN, HDFN, RDN, TDCMSFN, and TEMDSFN (Supporting Information 2). Notably, PTB demonstrated a significant positive correlation with HDFN (Figure 5C). Of particular interest, reverse MR results revealed a significant positive correlation between NNJ and BW (Figure 5D).

## 4. Discussion

Throughout pregnancy, the maternal immune system undergoes intricate regulation based on fetal development and environmental signals, ensuring tolerance to the embryo while effectively resisting infections [36]. Meanwhile, fetal growth presents a significant challenge to the maternal immune system [37]. Various leukocyte subsets, including T cells, B cells, and NK cells, play crucial roles in maintaining the delicate immune balance at the maternal-fetal interface. Disruption of this balance may result in adverse pregnancy outcomes such as PE, gestational hypertension (GH), and GDM [38, 39]. While the significance of the immune system in neonatal diseases is widely noticed, its specific roles remain elusive. In this study, two-sample MR analysis was employed to explore potential causal relationships between various leukocyte features and neonatal diseases. A total of 285 leukocyte subsets with 178, 232, 15, 44, 11, 32, and 68 pairs of significant causal associations between FGR, PTB, NNJ, DSDFN, HDFN, RDN, and TDMSFN were identified, respectively. These findings stressed the extensive involvement of leukocytes in neonatal diseases.

FGR arises from multiple complex etiologies, with placental insufficiency identified as a predominant pathophysiological factor [40]. A key characteristic of placental insufficiency involves the insufficient remodeling of uterine spiral arteries [41], closely linked to the development of FGR [42]. Notably, existing evidence has highlighted the crucial role of immune cells in regulating spiral artery remodeling. For example, it has been demonstrated that the exhaustion of DCs during the embryo implantation process disrupts the regular vascular remodeling, affecting both vessel permeability and the blood flow directed toward the implantation site [43]. Moreover, decidual Tregs play a crucial role in orchestrating maternal vascular changes essential for the maintenance of spiral artery formation, achieved through their interactions with dNK cells [44]. Notably, during the period of spiral artery remodeling, insufficient activity of NK cells can trigger PE [45], thus significantly elevating the risk of FGR [46]. Additionally, individuals with FGR demonstrated signs of dysregulated immune cell infiltration, including a noteworthy increase in Th17 cells in the peripheral circulation and a significant reduction in CD56+ uNK cells within the placenta [47, 48]. An elevated quantity of FOXP3+ Treg and CD3+ T cells contributed to heightened inflammation, closely associated with the occurrence of FGR [12]. Importantly, this study unveiled significant causal relationships between various leukocyte subsets and FGR, with T-cell subsets playing a predominant role. Despite our limited understanding of immune cell imbalance in FGR, this research provided additional evidence suggesting that the disruption in the quantity or activity of diverse leukocyte subsets may play a pivotal role in contributing to FGR.

The premature activation of pro-inflammatory pathways mediated by maternal leukocyte circulation may disrupt maternal tolerance and contribute to the onset of PTB [49]. In this study, MR results uncovered a significant causal relationship between leukocyte subsets and PTB, with T cells emerging as a predominant factor (43.97%) and 51.96% of T-cell subsets identified as PTB risk factors. This corroborates earlier research that observed the activation of specific T-cell subsets in the maternal circulation during PTB, as evidenced by the establishment of single-cell atlases of the human placenta [50]. Importantly, our findings aligned with evidence that highlighted placental dysfunction as a downstream effect of inflammatory processes [51, 52]. This relationship between inflammation and placental impairment is crucial for understanding the pathogenic pathways leading to PTB. Interactions between immune cells and placental tissues may cause abnormalities in placental development and function, explaining the prematurity outcomes observed in our study. Additionally, studies [53] have demonstrated a strong correlation between leukocyte counts, neutrophil-to-lymphocyte ratios, and cervical length dynamics in predicting early PTB. Current clinically effective interventions to prevent PTB involve the prenatal administration of anti-inflammatory progesterone [54], while corticosteroids and antibiotics offer targeted treatment to enhance neonatal outcomes in preterm pregnancies [55, 56]. Nonetheless, studies have reported a close correlation between reduced PD-L1 expression in trophoblast cells and diminished Treg numbers during pregnancy failure, suggesting that targeting PD-L1 could represent a novel therapeutic approach to prevent miscarriage [57]. Moreover, mesenchymal stem cells have found application in treating various inflammatory diseases like rheumatoid arthritis (RA) and inflammatory bowel disease (IBD). Consequently, the immune cells implicated in the pro-inflammatory disease processes identified in this study may serve as promising targets for more proactive treatment strategies in the future of preventing PTB.

Moreover, this study emphasized the importance of leukocyte subsets in addressing common clinical challenges during the neonatal period. First, jaundice is a prevalent condition in newborns, with pathological NNJ posing a severe threat to the health and even the life of fetuses [58]. T-cell subsets were identified as crucial contributors among the leukocyte subsets associated with NNJ. Additionally, the CD39+ Treg subset was identified as a risk factor for DSDFN, while the CD28+ Treg subset acted as a protective factor for both DSDFN and RDN. This further aligned with existing research highlighting the pivotal role of Tregs in pregnancy by modulating immunity on multiple fronts, ensuring maternal protection for the fetuses [59]. The study also underlined the intricate interplay between neonatal diseases. Numerous studies have indicated that FGR and PTB significantly contribute to the global disease burden [60], with FGR emerging as the most crucial antenatal risk factor for PTB [61]. It is noteworthy that perinatal complications resulting from FGR, such as growth restriction, cerebral palsy, and neurological abnormalities, can profoundly impact the health of newborns, extending into adulthood [62]. MR analysis consistently revealed a robust positive correlation between FGR and PTB, as well as NNJ, demonstrating mutual causality between NNJ and FGR. Furthermore, PTB was identified as a risk factor for HDFN. However, further research is essential to unveil the molecular and biological mechanisms underlying these associations, providing a more comprehensive understanding of the occurrence and development of neonatal diseases.

## 5. Conclusion

Overall, this study, employing MR analysis, investigated for the first time the potential causal relationships between various leukocyte subsets of the maternal immune system and neonatal diseases. Specifically, the research uncovered that the intricate regulation of leukocyte subsets plays a pivotal role in the progression of neonatal diseases during pregnancy. The study revealed diverse regulatory mechanisms of leukocyte subsets in diseases such as FGR, PTB, and NNJ, underscoring the complexity inherent in the pathogenesis of these conditions. Offering a comprehensive perspective on the impact of the immune system during maternal pregnancy on neonatal diseases, the study provided fresh insights for the early diagnosis and treatment of related conditions.

## 6. Advantages and Limitations

This study exhibited several strengths. First, it stood as the pioneering exploration into potential causal relationships between diverse leukocyte features and neonatal diseases through MR analysis. Second, it leveraged a substantial number of IVs and employed various MR analysis methods, ensuring the reliability and robustness of the results. Lastly, the study introduced a fresh perspective on immune dysregulation, contributing to the understanding of the concurrent occurrence of neonatal diseases. However, there were certain limitations to this research. We acknowledge the limitation of our study due to the insufficient GWAS data correlating specific PTB phenotypes, such as unknown etiology, bleeding, infection, suspected FGR, and severe maternal conditions with genetic outcomes [63]. This gap exists because such detailed data are currently absent in existing databases. The discovery of our SNPs and the estimation of their effects within the same population might lead to the potential overestimation of effects, a phenomenon commonly referred to as the “winner's curse” [64]. Additionally, the study was constrained by the limited availability of GWAS data associated with various leukocyte subsets, which limited research on cell types such as macrophages, mast cells, and neutrophils.

## Figures and Tables

**Table 1:** The overlapping leukocyte subsets factors in BL and BW.

Exposure. trait	Panel	Outcome	nSNP	Method	OR	95% CI	*P*.FDR	Heterogeneity *p*-value	Pleiotropy *p*-value
pDC AC	DC	BL	4	IVW (random)	0.910	0.891–0.929	1.60E-02	—	0.725
		BW	6	IVW	0.977	0.963–0.992	5.05E-16	0.451	0.191

CD8 on CD28- CD8+ T cell	T cell	BL	5	IVW (random)	0.918	0.895–0.942	3.52E-08	—	0.689
		BW	11	IVW	0.975	0.960–0.991	1.30E-02	0.309	0.906

CD32 on mDC	DC	BL	3	IVW (random)	0.956	0.940–0.972	3.87E-05	—	0.880
		BW	3	IVW	0.947	0.923–0.972	5.16E-04	0.544	0.469

CX3CR1 on CD14+ CD16+ monocyte	Monocyte	BL	2	IVW (random)	0.978	0.967–0.989	1.21E-02	—	—
		BW	12	IVW (fixed)	0.981	0.974–0.988	3.75E-06	—	0.225

CD127 on CD28+ CD4+ T cell	T cell	BL	2	IVW (random)	1.025	1.022–1.028	4.29E-74	—	—
		BW	3	IVW (random)	1.019	1.007–1.032	1.68E-02	—	0.600

pDC %DC	DC	BL	3	IVW (random)	1.044	1.023–1.066	6.86E-03	—	0.903
		BW	18	IVW (fixed)	1.026	1.019–1.033	3.90E-11	—	0.411

PDL-1 on CD14- CD16+ monocyte	Monocyte	BL	3	IVW (random)	1.046	1.028–1.064	7.31E-05	—	0.714
		BW	11	IVW (fixed)	1.030	1.023–1.038	4.90E-13	—	0.149

SSC-A on HLA DR + NK	NK	BL	3	IVW (random)	1.054	1.039–1.069	1.03E-10	—	0.817
		BW	19	IVW (fixed)	1.021	1.014–1.027	2.16E-08	—	0.064

Abbreviations: BL, birth length; BW, birth weight; CD, cluster of differentiation; CI, confidence interval; DC, dendritic cell; FDR, false discovery rate; HLA DR, human leukocyte antigen DR; IVW, inverse variance weighted; NK, natural killer cells; OR, odds ratio; pDC, plasmacytoid dendritic cell; SNP, single nucleotide polymorphism.

**Table 2:** Correlations of CD39+ Treg/CD28+ Treg subsets with DSDFN.

Exposure. trait	Panel	Outcome	nSNP	Method	OR	95% CI	*P*.FDR	Heterogeneity *p*-value	Pleiotropy *p*-value
CD39 on CD39+ activated CD4 Treg	Treg	DSDFN	170	IVW	1.202	1.104–1.309	5.40E-04	0.051	0.582
CD39 on CD39+ secreting CD4 Treg	Treg	DSDFN	124	IVW	1.212	1.103–1.331	1.28E-03	0.180	0.867
CD39+ activated CD4 Treg %CD4 Treg	Treg	DSDFN	124	IVW	1.356	1.229–1.496	1.96E-07	0.284	0.732
CD39+ activated CD4 Treg AC	Treg	DSDFN	122	IVW	1.397	1.254–1.558	2.16E-07	0.152	0.536
CD39+ resting CD4 Treg %CD4 Treg	Treg	DSDFN	136	IVW	1.227	1.134 −1.329	2.35E-05	0.534	0.250
CD39+ resting CD4 Treg %resting CD4 Treg	Treg	DSDFN	130	IVW	1.274	1.166–1.393	6.56E-06	0.267	0.952
CD39+ resting CD4 Treg AC	Treg	DSDFN	119	IVW	1.233	1.129–1.346	9.87E-05	0.542	0.666
CD39+ secreting CD4 Treg %CD4 Treg	Treg	DSDFN	156	IVW	1.241	1.137–1.354	5.20E-05	0.286	0.362
CD39+ secreting CD4 Treg %secreting CD4 Treg	Treg	DSDFN	156	IVW	1.241	1.138–1.353	4.45E-05	0.213	0.724
CD39+ secreting CD4 Treg AC	Treg	DSDFN	148	IVW	1.320	1.212–1.438	5.63E-08	0.623	0.596
CD28 on activated and secreting CD4 Treg	Treg	DSDFN	6	IVW (random)	0.497	0.313–0.790	3.60E-02	—	0.214
CD28 on resting CD4 Treg	Treg	DSDFN	13	IVW	0.455	0.298–0.695	4.58E-03	0.775	0.264

Abbreviations: CD, cluster of differentiation; CI, confidence interval; DSDFN, digestive system disorders of fetus and newborn; FDR, false discovery rate; IVW, inverse variance weighted; OR, odds ratio; SNP, single nucleotide polymorphism; Treg, regulatory T cells.

**Table 3:** The overlapping leukocyte subsets factors in DSDFN and RDN.

Exposure. trait	Panel	Outcome	nSNP	Method	OR	95% CI	*P*.FDR	Heterogeneity *p*-value	Pleiotropy *p*-value
CD28 on resting CD4 Treg	Treg	DSDFN	13	IVW	0.455	0.298–0.695	4.58E-03	0.775	0.264
		RDN	13	IVW (fixed)	0.470	0.317–0.697	1.31E-02	—	0.714

CD28 on CD28+ CD45RA- CD8+ T cell	T cell	DSDFN	13	IVW	0.470	0.304–0.728	1.06E-02	0.923	0.209
		RDN	13	IVW (fixed)	0.390	0.260–0.586	9.23E-04	—	0.879

CD28 on activated and secreting CD4 Treg	Treg	DSDFN	6	IVW (random)	0.497	0.313–0.790	3.60E-02	—	0.214
		RDN	6	IVW (fixed)	0.304	0.176–0.524	2.08E-03	—	0.801

Abbreviations: CD, cluster of differentiation; CI, confidence interval; DSDFN, digestive system disorders of fetus and newborn; FDR, false discovery rate; IVW, inverse variance weighted; OR, odds ratio; RDN, respiratory distress of newborn; SNP, single nucleotide polymorphism; Treg, regulatory T cells.

## Data Availability

All data are derived from the public database and can be downloaded according to the corresponding number in the supporting information.

## Supporting Information

Supporting Infromation 1 Table S1: GWAS data for 446 leukocyte features and seven adverse fetal outcomes. Table S2: Detailed information about instrumental variables. Table S3: Causality between 259 leukocyte subsets and FGR. Table S4: Causality between 245 leukocyte subsets and PTB. Table S5: Causality between leukocyte subsets and five adverse fetal outcomes. Table S6: Causal relationships between various neonatal diseases.

Supporting Information 2 The scripts for the MR analyses conducted in this study.

Supporting Information 3 Potential causal relationships between various neonatal diseases. Scatter plot for the relationship between the SNP effect size of causal adverse fetal events (*x*-axis) and another one (*y*-axis). (A–E). FGR on DSDFN, HDFN, RDN, TDCMSFN, and TEMDSFN, respectively.
