# Supplementary material for: Causal Relationships Between Leukocyte Subsets and Adverse Fetal Outcomes: A Mendelian Randomization Study
Source: Mediators Inflamm. 2024 Dec 26;2024:6349687. doi: 10.1155/mi/6349687 (PMC11695084; doi:10.1155/mi/6349687)
Supplement: Supporting Information 2 — The scripts for the MR analyses conducted in this study. [file 6349687.f2.docx]

#读取下载内容

setwd("D:\\")#查看R语言当前工作路径，将txt文件放置给文件夹

library('data.table')

a <- fread("finngen_R9_O15_PRE_OR_ECLAMPSIA.gz",header = T)

save(a,file="Finngen.RData")

#获取数据变量

colnames(a)

#筛选强相关的变量：若5E-8筛选出来的变量较少，可适当调大P值（须有文献根据）

ab<-subset(a,pval<5e-8)

ab$phenotype<-"PRE_OR_ECLAMPSIA"

#load("整理.RData")

save(ab,file="整理.RData")

#整理为TwoSampleMR所需要的双样本数据

library(TwoSampleMR)

#暴露数据

exposure<-format_data(ab,

type = "exposure",

snp_col = "rsids",

phenotype_col = "phenotype",

beta_col = "beta",

se_col = "sebeta",

eaf_col="af_alt",

effect_allele_col = "alt",

other_allele_col = "ref",

pval_col = "pval")

#去除连锁不平衡（linkage disequilibrium）

exposure_data<-clump_data(exposure,clump_r2 = 0.001)

#结局数据

outcome<-format_data(ab,

snps=exposure_data$SNP,

type = "outcome",

snp_col = "rsids",

phenotype_col = "phenotype",

beta_col = "beta",

se_col = "sebeta",

eaf_col="af_alt",

effect_allele_col = "alt",

other_allele_col = "ref",

pval_col = "pval")

dat <- harmonise_data(exposure_dat=exposure_data, outcome_dat=outcome,action= 2)

res <- mr(dat,method_list = c("mr_wald_ratio","mr_egger_regression","mr_ivw",

"mr_two_sample_ml","mr_ivw_fe","mr_ivw_mre",

"mr_weighted_median",

"mr_simple_mode","mr_weighted_mode"))

res_or <- generate_odds_ratios(res)

#FDR矫正

library(fdrtool)

res_or <- subset(res_or,pval > 0)

pval <- res_or$pval

fdr = fdrtool(pval, statistic = "pvalue")

my_matrix <- matrix(c(1, 2, 3, 4,5,6,7,8,9,10,11,12,13,14), nrow = 1)

new_col <- c(15)

res_or <- cbind(res_or, new_col) # 在矩阵中添加新列

res_or$new_col <- fdr$qval

####多效性、异质性检验####

heterog <- mr_heterogeneity(dat, method_list=c("mr_egger_regression", "mr_ivw"))

pleio <- mr_pleiotropy_test(dat)

result_heterog = merge(result,heterog,by=c("id.exposure","id.outcome","outcome","exposure","method"),all.x = T)

result_heterog_pleio = merge(result_heterog,pleio,by=c("id.exposure","id.outcome","outcome","exposure"),all.x = T)
