# Supplementary figures and images for: Causal Relationships Between Leukocyte Subsets and Adverse Fetal Outcomes: A Mendelian Randomization Study
Source: Mediators Inflamm. 2024 Dec 26;2024:6349687. doi: 10.1155/mi/6349687 (PMC11695084; doi:10.1155/mi/6349687)

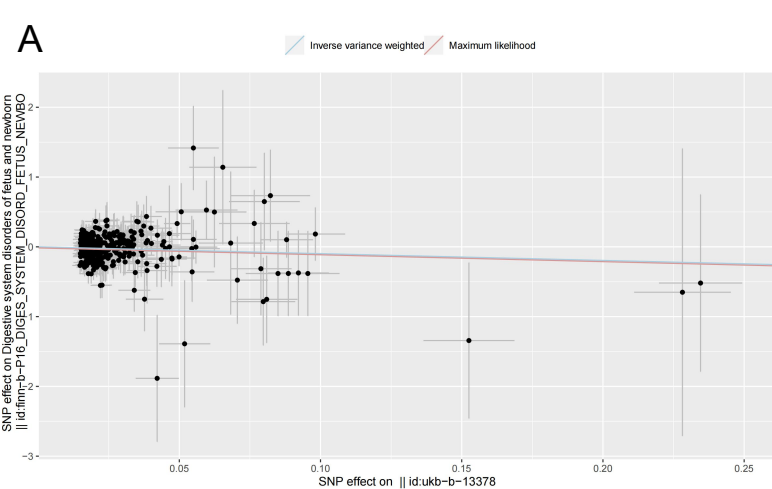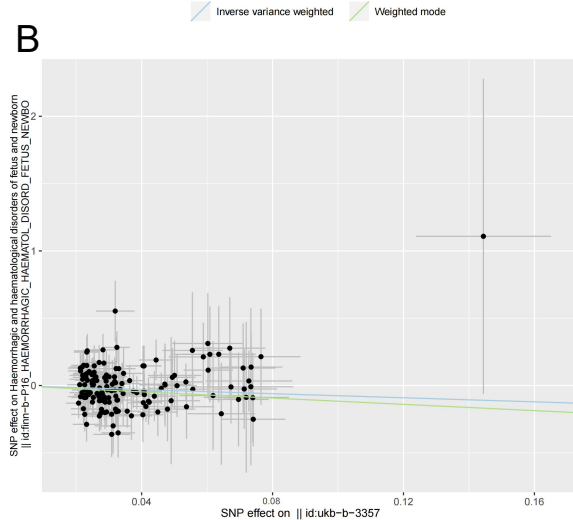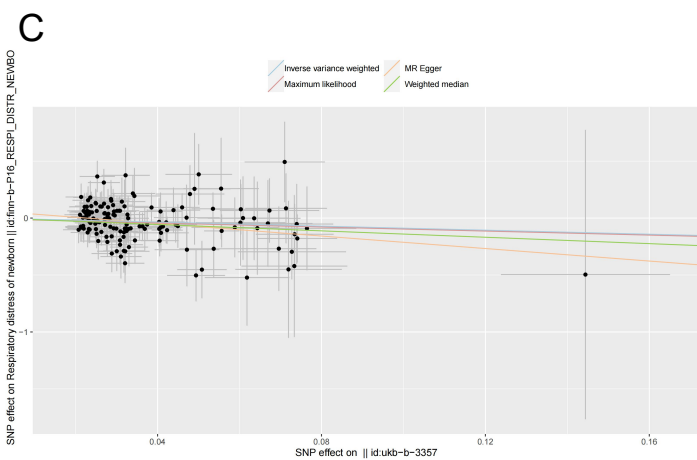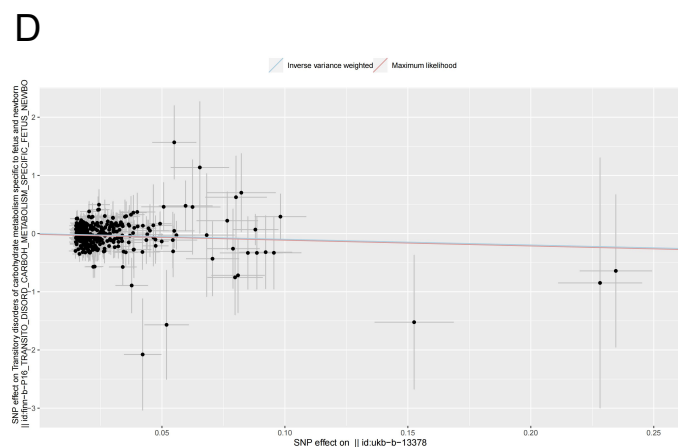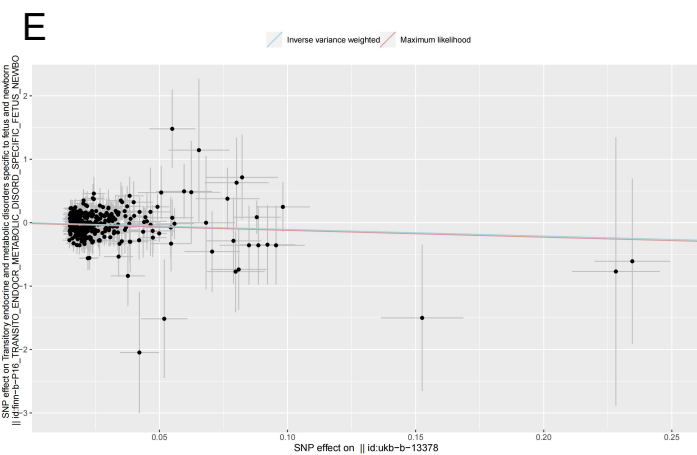

Supplement: Supporting Information 3 — Potential causal relationships between various neonatal diseases. Scatter plot for the relationship between the SNP effect size of causal adverse fetal events (x-axis) and another one (y-axis). (A–E). FGR on DSDFN, HDFN, RDN, TDCMSFN, and TEMDSFN, respectively. [file 6349687.f3.pdf]
