# Supplementary material for: Causal Relationships Between Leukocyte Subsets and Adverse Fetal Outcomes: A Mendelian Randomization Study
Source: Mediators Inflamm. 2024 Dec 26;2024:6349687. doi: 10.1155/mi/6349687 (PMC11695084; doi:10.1155/mi/6349687)
Supplement: Supporting Infromation 1 — Table S1: GWAS data for 446 leukocyte features and seven adverse fetal outcomes. Table S2: Detailed information about instrumental variables. Table S3: Causality between 259 leukocyte subsets and FGR. Table S4: Causality between 245 leukocyte subsets and PTB. Table S5: Causality between leukocyte subsets and five adverse fetal outcomes. Table S6: Causal relationships between various neonatal diseases. [file 6349687.f1.docx]

| **Supplementary Table 1** | GWAS data for 446 leukocyte features and seven adverse fetal outcomes. |  |  |  |  |  |  |  |  |  |  |  |  |  |  |  |  |  |  |  |  |  |  |  |  |  |  |  |  |
| --- | --- | --- | --- | --- | --- | --- | --- | --- | --- | --- | --- | --- | --- | --- | --- | --- | --- | --- | --- | --- | --- | --- | --- | --- | --- | --- | --- | --- | --- |
| **Supplementary Table 2** | Detailed information about instrumental variables. |  |  |  |  |  |  |  |  |  |  |  |  |  |  |  |  |  |  |  |  |  |  |  |  |  |  |  |  |
| **Supplementary Table 3** | Causality between 178 leukocyte subsets and FGR. |  |  |  |  |  |  |  |  |  |  |  |  |  |  |  |  |  |  |  |  |  |  |  |  |  |  |  |  |
| **Supplementary Table 4** | Causality between 232 leukocyte subsets and PTB. |  |  |  |  |  |  |  |  |  |  |  |  |  |  |  |  |  |  |  |  |  |  |  |  |  |  |  |  |
| **Supplementary Table 5** | Causality between leukocyte subsets and five adverse fetal outcomes. |  |  |  |  |  |  |  |  |  |  |  |  |  |  |  |  |  |  |  |  |  |  |  |  |  |  |  |  |
| **Supplementary Table 6** | Causal relationships between various neonatal diseases. |  |  |  |  |  |  |  |  |  |  |  |  |  |  |  |  |  |  |  |  |  |  |  |  |  |  |  |  |
| **Supplementary Table 1: GWAS data for 446 leukocyte features and seven adverse fetal outcomes.** | | | | | | | | | | | | | |  |  |  |  |  |  |  |  |  |  |  |  |  |  |  |  |
| **GWAS data for 446 leukocyte features** | | | | | |  |  |  |  |  |  |  |  |  |  |  |  |  |  |  |  |  |  |  |  |  |  |  |  |
| **ID** | **Trait** | **Consortium** | **Sample size** | **Number of SNPs** | **Year** |  |  |  |  |  |  |  |  |  |  |  |  |  |  |  |  |  |  |  |  |  |  |  |  |
| ebi-a-GCST90001533 | Basophil %CD33dim HLA DR- CD66b- | NA | 1,857 | 14,304,944 | 2020 |  |  |  |  |  |  |  |  |  |  |  |  |  |  |  |  |  |  |  |  |  |  |  |  |
| ebi-a-GCST90002003 | CCR2 on CD14- CD16- | NA | 3,629 | 15,034,296 | 2020 |  |  |  |  |  |  |  |  |  |  |  |  |  |  |  |  |  |  |  |  |  |  |  |  |
| ebi-a-GCST90002096 | CD11b on basophil | NA | 1,556 | 14,113,011 | 2020 |  |  |  |  |  |  |  |  |  |  |  |  |  |  |  |  |  |  |  |  |  |  |  |  |
| ebi-a-GCST90002097 | CD11b on CD33+ HLA DR+ CD14dim | NA | 1,578 | 14,127,355 | 2020 |  |  |  |  |  |  |  |  |  |  |  |  |  |  |  |  |  |  |  |  |  |  |  |  |
| ebi-a-GCST90002095 | CD11b on CD33dim HLA DR- | NA | 1,559 | 14,116,552 | 2020 |  |  |  |  |  |  |  |  |  |  |  |  |  |  |  |  |  |  |  |  |  |  |  |  |
| ebi-a-GCST90002093 | CD11b on CD66b++ myeloid cell | NA | 1,465 | 14,033,866 | 2020 |  |  |  |  |  |  |  |  |  |  |  |  |  |  |  |  |  |  |  |  |  |  |  |  |
| ebi-a-GCST90002094 | CD11b on Monocytic Myeloid-Derived Suppressor Cells | NA | 1,570 | 14,120,805 | 2020 |  |  |  |  |  |  |  |  |  |  |  |  |  |  |  |  |  |  |  |  |  |  |  |  |
| ebi-a-GCST90002020 | CD14 on CD33+ HLA DR+ CD14dim | NA | 1,578 | 14,127,355 | 2020 |  |  |  |  |  |  |  |  |  |  |  |  |  |  |  |  |  |  |  |  |  |  |  |  |
| ebi-a-GCST90002021 | CD14 on CD33dim HLA DR+ CD11b+ | NA | 1,634 | 14,155,482 | 2020 |  |  |  |  |  |  |  |  |  |  |  |  |  |  |  |  |  |  |  |  |  |  |  |  |
| ebi-a-GCST90001739 | CD19 on Plasma Blast-Plasma Cell | NA | 3,657 | 15,049,161 | 2020 |  |  |  |  |  |  |  |  |  |  |  |  |  |  |  |  |  |  |  |  |  |  |  |  |
| ebi-a-GCST90001807 | CD27 on Plasma Blast-Plasma Cell | NA | 3,651 | 15,047,730 | 2020 |  |  |  |  |  |  |  |  |  |  |  |  |  |  |  |  |  |  |  |  |  |  |  |  |
| ebi-a-GCST90001522 | CD33- HLA DR- Absolute Count | NA | 1,858 | 14,304,991 | 2020 |  |  |  |  |  |  |  |  |  |  |  |  |  |  |  |  |  |  |  |  |  |  |  |  |
| ebi-a-GCST90001523 | CD33- HLA DR+ Absolute Count | NA | 1,858 | 14,304,991 | 2020 |  |  |  |  |  |  |  |  |  |  |  |  |  |  |  |  |  |  |  |  |  |  |  |  |
| ebi-a-GCST90001954 | CD33 on basophil | NA | 1,555 | 14,112,929 | 2020 |  |  |  |  |  |  |  |  |  |  |  |  |  |  |  |  |  |  |  |  |  |  |  |  |
| ebi-a-GCST90001956 | CD33 on CD33+ HLA DR+ | NA | 1,579 | 14,129,808 | 2020 |  |  |  |  |  |  |  |  |  |  |  |  |  |  |  |  |  |  |  |  |  |  |  |  |
| ebi-a-GCST90001957 | CD33 on CD33+ HLA DR+ CD14- | NA | 1,578 | 14,129,762 | 2020 |  |  |  |  |  |  |  |  |  |  |  |  |  |  |  |  |  |  |  |  |  |  |  |  |
| ebi-a-GCST90001947 | CD33 on CD33+ HLA DR+ CD14dim | NA | 1,577 | 14,127,271 | 2020 |  |  |  |  |  |  |  |  |  |  |  |  |  |  |  |  |  |  |  |  |  |  |  |  |
| ebi-a-GCST90001953 | CD33 on CD33dim HLA DR- | NA | 1,558 | 14,116,471 | 2020 |  |  |  |  |  |  |  |  |  |  |  |  |  |  |  |  |  |  |  |  |  |  |  |  |
| ebi-a-GCST90001949 | CD33 on CD33dim HLA DR+ CD11b- | NA | 1,634 | 14,155,757 | 2020 |  |  |  |  |  |  |  |  |  |  |  |  |  |  |  |  |  |  |  |  |  |  |  |  |
| ebi-a-GCST90001948 | CD33 on CD33dim HLA DR+ CD11b+ | NA | 1,633 | 14,155,400 | 2020 |  |  |  |  |  |  |  |  |  |  |  |  |  |  |  |  |  |  |  |  |  |  |  |  |
| ebi-a-GCST90001951 | CD33 on CD66b++ myeloid cell | NA | 1,464 | 14,033,777 | 2020 |  |  |  |  |  |  |  |  |  |  |  |  |  |  |  |  |  |  |  |  |  |  |  |  |
| ebi-a-GCST90001955 | CD33 on Immature Myeloid-Derived Suppressor Cells | NA | 1,554 | 14,110,139 | 2020 |  |  |  |  |  |  |  |  |  |  |  |  |  |  |  |  |  |  |  |  |  |  |  |  |
| ebi-a-GCST90001952 | CD33 on Monocytic Myeloid-Derived Suppressor Cells | NA | 1,569 | 14,120,721 | 2020 |  |  |  |  |  |  |  |  |  |  |  |  |  |  |  |  |  |  |  |  |  |  |  |  |
| ebi-a-GCST90001517 | CD33+ HLA DR+ Absolute Count | NA | 1,858 | 14,304,991 | 2020 |  |  |  |  |  |  |  |  |  |  |  |  |  |  |  |  |  |  |  |  |  |  |  |  |
| ebi-a-GCST90001519 | CD33+ HLA DR+ CD14- %CD33+ HLA DR+ | NA | 1,858 | 14,304,991 | 2020 |  |  |  |  |  |  |  |  |  |  |  |  |  |  |  |  |  |  |  |  |  |  |  |  |
| ebi-a-GCST90001518 | CD33+ HLA DR+ CD14- Absolute Count | NA | 1,858 | 14,304,991 | 2020 |  |  |  |  |  |  |  |  |  |  |  |  |  |  |  |  |  |  |  |  |  |  |  |  |
| ebi-a-GCST90001521 | CD33+ HLA DR+ CD14dim %CD33+ HLA DR+ | NA | 1,858 | 14,304,991 | 2020 |  |  |  |  |  |  |  |  |  |  |  |  |  |  |  |  |  |  |  |  |  |  |  |  |
| ebi-a-GCST90001520 | CD33+ HLA DR+ CD14dim Absolute Count | NA | 1,858 | 14,304,991 | 2020 |  |  |  |  |  |  |  |  |  |  |  |  |  |  |  |  |  |  |  |  |  |  |  |  |
| ebi-a-GCST90001531 | CD33dim HLA DR- Absolute Count | NA | 1,856 | 14,304,913 | 2020 |  |  |  |  |  |  |  |  |  |  |  |  |  |  |  |  |  |  |  |  |  |  |  |  |
| ebi-a-GCST90001528 | CD33dim HLA DR+ CD11b- %CD33dim HLA DR+ | NA | 1,858 | 14,304,991 | 2020 |  |  |  |  |  |  |  |  |  |  |  |  |  |  |  |  |  |  |  |  |  |  |  |  |
| ebi-a-GCST90001527 | CD33dim HLA DR+ CD11b- Absolute Count | NA | 1,858 | 14,304,991 | 2020 |  |  |  |  |  |  |  |  |  |  |  |  |  |  |  |  |  |  |  |  |  |  |  |  |
| ebi-a-GCST90001526 | CD33dim HLA DR+ CD11b+ %CD33dim HLA DR+ | NA | 1,858 | 14,304,991 | 2020 |  |  |  |  |  |  |  |  |  |  |  |  |  |  |  |  |  |  |  |  |  |  |  |  |
| ebi-a-GCST90001525 | CD33dim HLA DR+ CD11b+ Absolute Count | NA | 1,858 | 14,304,991 | 2020 |  |  |  |  |  |  |  |  |  |  |  |  |  |  |  |  |  |  |  |  |  |  |  |  |
| ebi-a-GCST90001810 | CD38 on CD3- CD19- | NA | 1,834 | 14,180,911 | 2019 |  |  |  |  |  |  |  |  |  |  |  |  |  |  |  |  |  |  |  |  |  |  |  |  |
| ebi-a-GCST90002051 | CD45 on basophil | NA | 1,556 | 14,113,011 | 2020 |  |  |  |  |  |  |  |  |  |  |  |  |  |  |  |  |  |  |  |  |  |  |  |  |
| ebi-a-GCST90002045 | CD45 on CD33- HLA DR- | NA | 1,635 | 14,155,839 | 2020 |  |  |  |  |  |  |  |  |  |  |  |  |  |  |  |  |  |  |  |  |  |  |  |  |
| ebi-a-GCST90002046 | CD45 on CD33- HLA DR+ | NA | 1,635 | 14,155,839 | 2020 |  |  |  |  |  |  |  |  |  |  |  |  |  |  |  |  |  |  |  |  |  |  |  |  |
| ebi-a-GCST90002053 | CD45 on CD33+ HLA DR+ | NA | 1,580 | 14,129,891 | 2020 |  |  |  |  |  |  |  |  |  |  |  |  |  |  |  |  |  |  |  |  |  |  |  |  |
| ebi-a-GCST90002042 | CD45 on CD33+ HLA DR+ CD14- | NA | 1,579 | 14,129,845 | 2020 |  |  |  |  |  |  |  |  |  |  |  |  |  |  |  |  |  |  |  |  |  |  |  |  |
| ebi-a-GCST90002043 | CD45 on CD33+ HLA DR+ CD14dim | NA | 1,578 | 14,127,355 | 2020 |  |  |  |  |  |  |  |  |  |  |  |  |  |  |  |  |  |  |  |  |  |  |  |  |
| ebi-a-GCST90002050 | CD45 on CD33dim HLA DR- | NA | 1,559 | 14,116,552 | 2020 |  |  |  |  |  |  |  |  |  |  |  |  |  |  |  |  |  |  |  |  |  |  |  |  |
| ebi-a-GCST90002044 | CD45 on CD33dim HLA DR+ CD11b- | NA | 1,635 | 14,155,839 | 2020 |  |  |  |  |  |  |  |  |  |  |  |  |  |  |  |  |  |  |  |  |  |  |  |  |
| ebi-a-GCST90002048 | CD45 on CD66b++ myelod cell | NA | 1,465 | 14,033,866 | 2020 |  |  |  |  |  |  |  |  |  |  |  |  |  |  |  |  |  |  |  |  |  |  |  |  |
| ebi-a-GCST90002052 | CD45 on Immature Myeloid-Derived Suppressor Cells | NA | 1,555 | 14,110,222 | 2020 |  |  |  |  |  |  |  |  |  |  |  |  |  |  |  |  |  |  |  |  |  |  |  |  |
| ebi-a-GCST90001914 | CD45 on lymphocyte | NA | 3,113 | 14,903,739 | 2020 |  |  |  |  |  |  |  |  |  |  |  |  |  |  |  |  |  |  |  |  |  |  |  |  |
| ebi-a-GCST90002041 | CD45 on lymphocyte | NA | 1,635 | 14,155,839 | 2020 |  |  |  |  |  |  |  |  |  |  |  |  |  |  |  |  |  |  |  |  |  |  |  |  |
| ebi-a-GCST90002049 | CD45 on Monocytic Myeloid-Derived Suppressor Cells | NA | 1,570 | 14,120,805 | 2020 |  |  |  |  |  |  |  |  |  |  |  |  |  |  |  |  |  |  |  |  |  |  |  |  |
| ebi-a-GCST90001836 | CD66b on CD66b++ myeloid cell | NA | 1,465 | 14,033,866 | 2020 |  |  |  |  |  |  |  |  |  |  |  |  |  |  |  |  |  |  |  |  |  |  |  |  |
| ebi-a-GCST90001529 | CD66b++ myeloid cell Absolute Count | NA | 1,858 | 14,304,991 | 2020 |  |  |  |  |  |  |  |  |  |  |  |  |  |  |  |  |  |  |  |  |  |  |  |  |
| ebi-a-GCST90001994 | CX3CR1 on CD14- CD16- | NA | 3,590 | 15,023,496 | 2020 |  |  |  |  |  |  |  |  |  |  |  |  |  |  |  |  |  |  |  |  |  |  |  |  |
| ebi-a-GCST90002112 | HLA DR on CD33- HLA DR+ | NA | 1,635 | 14,155,839 | 2020 |  |  |  |  |  |  |  |  |  |  |  |  |  |  |  |  |  |  |  |  |  |  |  |  |
| ebi-a-GCST90002108 | HLA DR on CD33+ HLA DR+ CD14- | NA | 1,579 | 14,129,845 | 2020 |  |  |  |  |  |  |  |  |  |  |  |  |  |  |  |  |  |  |  |  |  |  |  |  |
| ebi-a-GCST90002109 | HLA DR on CD33+ HLA DR+ CD14dim | NA | 1,578 | 14,127,355 | 2020 |  |  |  |  |  |  |  |  |  |  |  |  |  |  |  |  |  |  |  |  |  |  |  |  |
| ebi-a-GCST90002111 | HLA DR on CD33dim HLA DR+ CD11b- | NA | 1,635 | 14,155,839 | 2020 |  |  |  |  |  |  |  |  |  |  |  |  |  |  |  |  |  |  |  |  |  |  |  |  |
| ebi-a-GCST90002110 | HLA DR on CD33dim HLA DR+ CD11b+ | NA | 1,634 | 14,155,482 | 2020 |  |  |  |  |  |  |  |  |  |  |  |  |  |  |  |  |  |  |  |  |  |  |  |  |
| ebi-a-GCST90001516 | Immature Myeloid-Derived Suppressor Cells %CD33dim HLA DR- CD66b- | NA | 1,857 | 14,304,944 | 2020 |  |  |  |  |  |  |  |  |  |  |  |  |  |  |  |  |  |  |  |  |  |  |  |  |
| ebi-a-GCST90002000 | PDL-1 on CD14- CD16- | NA | 3,629 | 15,034,296 | 2020 |  |  |  |  |  |  |  |  |  |  |  |  |  |  |  |  |  |  |  |  |  |  |  |  |
| ebi-a-GCST90001982 | CCR2 on CD14- CD16+ monocyte | NA | 3,621 | 15,029,878 | 2020 |  |  |  |  |  |  |  |  |  |  |  |  |  |  |  |  |  |  |  |  |  |  |  |  |
| ebi-a-GCST90002004 | CCR2 on CD14+ CD16- monocyte | NA | 3,629 | 15,034,296 | 2020 |  |  |  |  |  |  |  |  |  |  |  |  |  |  |  |  |  |  |  |  |  |  |  |  |
| ebi-a-GCST90001992 | CCR2 on CD14+ CD16+ monocyte | NA | 3,618 | 15,030,660 | 2020 |  |  |  |  |  |  |  |  |  |  |  |  |  |  |  |  |  |  |  |  |  |  |  |  |
| ebi-a-GCST90002014 | CCR2 on CD62L+ myeloid Dendritic Cell | NA | 2,870 | 14,824,404 | 2020 |  |  |  |  |  |  |  |  |  |  |  |  |  |  |  |  |  |  |  |  |  |  |  |  |
| ebi-a-GCST90002016 | CCR2 on CD62L+ plasmacytoid Dendritic Cell | NA | 2,871 | 14,826,842 | 2020 |  |  |  |  |  |  |  |  |  |  |  |  |  |  |  |  |  |  |  |  |  |  |  |  |
| ebi-a-GCST90002018 | CCR2 on granulocyte | NA | 2,850 | 14,821,110 | 2020 |  |  |  |  |  |  |  |  |  |  |  |  |  |  |  |  |  |  |  |  |  |  |  |  |
| ebi-a-GCST90002008 | CCR2 on monocyte | NA | 3,629 | 15,034,296 | 2020 |  |  |  |  |  |  |  |  |  |  |  |  |  |  |  |  |  |  |  |  |  |  |  |  |
| ebi-a-GCST90002017 | CCR2 on monocyte | NA | 2,850 | 14,821,110 | 2020 |  |  |  |  |  |  |  |  |  |  |  |  |  |  |  |  |  |  |  |  |  |  |  |  |
| ebi-a-GCST90002013 | CCR2 on myeloid Dendritic Cell | NA | 2,870 | 14,824,404 | 2020 |  |  |  |  |  |  |  |  |  |  |  |  |  |  |  |  |  |  |  |  |  |  |  |  |
| ebi-a-GCST90002015 | CCR2 on plasmacytoid Dendritic Cell | NA | 2,871 | 14,826,842 | 2020 |  |  |  |  |  |  |  |  |  |  |  |  |  |  |  |  |  |  |  |  |  |  |  |  |
| ebi-a-GCST90002091 | CD11b on CD14+ monocyte | NA | 1,635 | 14,155,839 | 2020 |  |  |  |  |  |  |  |  |  |  |  |  |  |  |  |  |  |  |  |  |  |  |  |  |
| ebi-a-GCST90002092 | CD11b on Granulocytic Myeloid-Derived Suppressor Cells | NA | 1,244 | 4,711,770 | 2020 |  |  |  |  |  |  |  |  |  |  |  |  |  |  |  |  |  |  |  |  |  |  |  |  |
| ebi-a-GCST90002088 | CD11c on CD62L+ myeloid Dendritic Cell | NA | 2,872 | 14,829,776 | 2020 |  |  |  |  |  |  |  |  |  |  |  |  |  |  |  |  |  |  |  |  |  |  |  |  |
| ebi-a-GCST90002090 | CD11c on granulocyte | NA | 2,850 | 14,821,110 | 2020 |  |  |  |  |  |  |  |  |  |  |  |  |  |  |  |  |  |  |  |  |  |  |  |  |
| ebi-a-GCST90002089 | CD11c on monocyte | NA | 2,850 | 14,821,110 | 2020 |  |  |  |  |  |  |  |  |  |  |  |  |  |  |  |  |  |  |  |  |  |  |  |  |
| ebi-a-GCST90002087 | CD11c on myeloid Dendritic Cell | NA | 2,872 | 14,829,776 | 2020 |  |  |  |  |  |  |  |  |  |  |  |  |  |  |  |  |  |  |  |  |  |  |  |  |
| ebi-a-GCST90001453 | CD11c+ CD62L- monocyte %monocyte | NA | 3,040 | 14,988,420 | 2020 |  |  |  |  |  |  |  |  |  |  |  |  |  |  |  |  |  |  |  |  |  |  |  |  |
| ebi-a-GCST90001452 | CD11c+ CD62L- monocyte Absolute Count | NA | 3,017 | 14,980,974 | 2020 |  |  |  |  |  |  |  |  |  |  |  |  |  |  |  |  |  |  |  |  |  |  |  |  |
| ebi-a-GCST90001457 | CD11c+ HLA DR++ monocyte %monocyte | NA | 3,042 | 14,988,863 | 2020 |  |  |  |  |  |  |  |  |  |  |  |  |  |  |  |  |  |  |  |  |  |  |  |  |
| ebi-a-GCST90001456 | CD11c+ HLA DR++ monocyte Absolute Count | NA | 3,019 | 14,981,416 | 2020 |  |  |  |  |  |  |  |  |  |  |  |  |  |  |  |  |  |  |  |  |  |  |  |  |
| ebi-a-GCST90001449 | CD11c+ monocyte %monocyte | NA | 3,043 | 14,988,922 | 2020 |  |  |  |  |  |  |  |  |  |  |  |  |  |  |  |  |  |  |  |  |  |  |  |  |
| ebi-a-GCST90001448 | CD11c+ monocyte Absolute Count | NA | 3,020 | 14,981,478 | 2020 |  |  |  |  |  |  |  |  |  |  |  |  |  |  |  |  |  |  |  |  |  |  |  |  |
| ebi-a-GCST90001945 | CD123 on CD62L+ plasmacytoid Dendritic Cell | NA | 2,872 | 14,829,782 | 2020 |  |  |  |  |  |  |  |  |  |  |  |  |  |  |  |  |  |  |  |  |  |  |  |  |
| ebi-a-GCST90001944 | CD123 on plasmacytoid Dendritic Cell | NA | 2,872 | 14,829,782 | 2020 |  |  |  |  |  |  |  |  |  |  |  |  |  |  |  |  |  |  |  |  |  |  |  |  |
| ebi-a-GCST90001926 | CD127 on granulocyte | NA | 2,920 | 14,849,646 | 2020 |  |  |  |  |  |  |  |  |  |  |  |  |  |  |  |  |  |  |  |  |  |  |  |  |
| met-b-162 | CD32 on 11c+123+DC | NA | 497 | 1,623,131 | 2020 |  |  |  |  |  |  |  |  |  |  |  |  |  |  |  |  |  |  |  |  |  |  |  |  |
| met-b-150 | CD32 on mDC | NA | 497 | 1,624,001 | 2020 |  |  |  |  |  |  |  |  |  |  |  |  |  |  |  |  |  |  |  |  |  |  |  |  |
| met-b-143 | CD32 on pDC | NA | 497 | 1,624,619 | 2020 |  |  |  |  |  |  |  |  |  |  |  |  |  |  |  |  |  |  |  |  |  |  |  |  |
| ebi-a-GCST90001946 | CD33 on CD14+ monocyte | NA | 1,634 | 14,155,757 | 2020 |  |  |  |  |  |  |  |  |  |  |  |  |  |  |  |  |  |  |  |  |  |  |  |  |
| ebi-a-GCST90001950 | CD33 on Granulocytic Myeloid-Derived Suppressor Cells | NA | 1,244 | 13,834,170 | 2020 |  |  |  |  |  |  |  |  |  |  |  |  |  |  |  |  |  |  |  |  |  |  |  |  |
| met-b-154 | CD337 on NK early | NA | 497 | 1,623,350 | 2015 |  |  |  |  |  |  |  |  |  |  |  |  |  |  |  |  |  |  |  |  |  |  |  |  |
| ebi-a-GCST90002033 | CD39 on granulocyte | NA | 2,920 | 14,849,646 | 2020 |  |  |  |  |  |  |  |  |  |  |  |  |  |  |  |  |  |  |  |  |  |  |  |  |
| ebi-a-GCST90002034 | CD39 on monocyte | NA | 2,909 | 14,848,657 | 2020 |  |  |  |  |  |  |  |  |  |  |  |  |  |  |  |  |  |  |  |  |  |  |  |  |
| ebi-a-GCST90001989 | CD40 on CD14- CD16+ monocyte | NA | 3,621 | 15,029,878 | 2020 |  |  |  |  |  |  |  |  |  |  |  |  |  |  |  |  |  |  |  |  |  |  |  |  |
| ebi-a-GCST90001980 | CD40 on CD14+ CD16- monocyte | NA | 3,629 | 15,034,296 | 2020 |  |  |  |  |  |  |  |  |  |  |  |  |  |  |  |  |  |  |  |  |  |  |  |  |
| ebi-a-GCST90001981 | CD40 on CD14+ CD16+ monocyte | NA | 3,618 | 15,030,660 | 2020 |  |  |  |  |  |  |  |  |  |  |  |  |  |  |  |  |  |  |  |  |  |  |  |  |
| ebi-a-GCST90001985 | CD40 on monocytes | NA | 3,629 | 15,034,296 | 2020 |  |  |  |  |  |  |  |  |  |  |  |  |  |  |  |  |  |  |  |  |  |  |  |  |
| ebi-a-GCST90001909 | CD45 on CD14+ monocyte | NA | 3,112 | 14,903,652 | 2020 |  |  |  |  |  |  |  |  |  |  |  |  |  |  |  |  |  |  |  |  |  |  |  |  |
| ebi-a-GCST90001913 | CD45 on granulocyte | NA | 3,112 | 14,903,727 | 2020 |  |  |  |  |  |  |  |  |  |  |  |  |  |  |  |  |  |  |  |  |  |  |  |  |
| ebi-a-GCST90002047 | CD45 on Granulocytic Myeloid-Derived Suppressor Cells | NA | 1,244 | 13,834,170 | 2020 |  |  |  |  |  |  |  |  |  |  |  |  |  |  |  |  |  |  |  |  |  |  |  |  |
| ebi-a-GCST90001832 | CD62L on CD62L+ plasmacytoid Dendritic Cell | NA | 2,872 | 14,829,782 | 2020 |  |  |  |  |  |  |  |  |  |  |  |  |  |  |  |  |  |  |  |  |  |  |  |  |
| ebi-a-GCST90001471 | CD62L- plasmacytoid Dendritic Cell %Dendritic Cell | NA | 3,410 | 15,143,298 | 2020 |  |  |  |  |  |  |  |  |  |  |  |  |  |  |  |  |  |  |  |  |  |  |  |  |
| ebi-a-GCST90001470 | CD62L- plasmacytoid Dendritic Cell Absolute Count | NA | 3,374 | 14,710,919 | 2020 |  |  |  |  |  |  |  |  |  |  |  |  |  |  |  |  |  |  |  |  |  |  |  |  |
| ebi-a-GCST90001837 | CD66b on Granulocytic Myeloid-Derived Suppressor Cells | NA | 1,244 | 13,834,170 | 2020 |  |  |  |  |  |  |  |  |  |  |  |  |  |  |  |  |  |  |  |  |  |  |  |  |
| ebi-a-GCST90002036 | CD80 on CD62L+ myeloid Dendritic Cell | NA | 2,871 | 14,829,665 | 2020 |  |  |  |  |  |  |  |  |  |  |  |  |  |  |  |  |  |  |  |  |  |  |  |  |
| ebi-a-GCST90002038 | CD80 on CD62L+ plasmacytoid Dendritic Cell | NA | 2,871 | 14,829,670 | 2020 |  |  |  |  |  |  |  |  |  |  |  |  |  |  |  |  |  |  |  |  |  |  |  |  |
| ebi-a-GCST90002040 | CD80 on granulocyte | NA | 2,850 | 14,821,110 | 2020 |  |  |  |  |  |  |  |  |  |  |  |  |  |  |  |  |  |  |  |  |  |  |  |  |
| ebi-a-GCST90002039 | CD80 on monocyte | NA | 2,850 | 14,821,110 | 2020 |  |  |  |  |  |  |  |  |  |  |  |  |  |  |  |  |  |  |  |  |  |  |  |  |
| ebi-a-GCST90002035 | CD80 on myeloid Dendritic Cell | NA | 2,871 | 14,829,665 | 2020 |  |  |  |  |  |  |  |  |  |  |  |  |  |  |  |  |  |  |  |  |  |  |  |  |
| ebi-a-GCST90002037 | CD80 on plasmacytoid Dendritic Cell | NA | 2,871 | 14,829,670 | 2019 |  |  |  |  |  |  |  |  |  |  |  |  |  |  |  |  |  |  |  |  |  |  |  |  |
| ebi-a-GCST90001467 | CD86+ plasmacytoid Dendritic Cell %Dendritic Cell | NA | 3,410 | 15,143,298 | 2020 |  |  |  |  |  |  |  |  |  |  |  |  |  |  |  |  |  |  |  |  |  |  |  |  |
| ebi-a-GCST90001466 | CD86+ plasmacytoid Dendritic Cell Absolute Count | NA | 3,374 | 15,130,102 | 2020 |  |  |  |  |  |  |  |  |  |  |  |  |  |  |  |  |  |  |  |  |  |  |  |  |
| ebi-a-GCST90002012 | CX3CR1 on CD14- CD16+ monocyte | NA | 3,582 | 15,019,052 | 2020 |  |  |  |  |  |  |  |  |  |  |  |  |  |  |  |  |  |  |  |  |  |  |  |  |
| ebi-a-GCST90001997 | CX3CR1 on CD14+ CD16- monocyte | NA | 3,590 | 15,023,496 | 2020 |  |  |  |  |  |  |  |  |  |  |  |  |  |  |  |  |  |  |  |  |  |  |  |  |
| ebi-a-GCST90001996 | CX3CR1 on CD14+ CD16+ monocyte | NA | 3,579 | 15,019,836 | 2020 |  |  |  |  |  |  |  |  |  |  |  |  |  |  |  |  |  |  |  |  |  |  |  |  |
| ebi-a-GCST90001995 | CX3CR1 on monocyte | NA | 3,590 | 15,023,496 | 2020 |  |  |  |  |  |  |  |  |  |  |  |  |  |  |  |  |  |  |  |  |  |  |  |  |
| ebi-a-GCST90002105 | HLA DR on plasmacytoid Dendritic Cell | NA | 2,872 | 14,829,782 | 2020 |  |  |  |  |  |  |  |  |  |  |  |  |  |  |  |  |  |  |  |  |  |  |  |  |
| ebi-a-GCST90001999 | PDL-1 on CD14- CD16+ monocyte | NA | 3,621 | 15,029,878 | 2020 |  |  |  |  |  |  |  |  |  |  |  |  |  |  |  |  |  |  |  |  |  |  |  |  |
| ebi-a-GCST90001993 | PDL-1 on CD14+ CD16- monocyte | NA | 3,629 | 15,034,296 | 2020 |  |  |  |  |  |  |  |  |  |  |  |  |  |  |  |  |  |  |  |  |  |  |  |  |
| ebi-a-GCST90001998 | PDL-1 on CD14+ CD16+ monocyte | NA | 3,618 | 15,030,660 | 2020 |  |  |  |  |  |  |  |  |  |  |  |  |  |  |  |  |  |  |  |  |  |  |  |  |
| ebi-a-GCST90002002 | PDL-1 on monocyte | NA | 3,629 | 15,034,296 | 2020 |  |  |  |  |  |  |  |  |  |  |  |  |  |  |  |  |  |  |  |  |  |  |  |  |
| ebi-a-GCST90001474 | Plasmacytoid Dendritic Cell %Dendritic Cell | NA | 3,410 | 15,143,298 | 2015 |  |  |  |  |  |  |  |  |  |  |  |  |  |  |  |  |  |  |  |  |  |  |  |  |
| ebi-a-GCST90001460 | Plasmacytoid Dendritic Cell Absolute Count | NA | 3,374 | 15,130,102 | 2015 |  |  |  |  |  |  |  |  |  |  |  |  |  |  |  |  |  |  |  |  |  |  |  |  |
| met-b-146 | CD27 on IgG+B | NA | 497 | 1,623,996 | 2020 |  |  |  |  |  |  |  |  |  |  |  |  |  |  |  |  |  |  |  |  |  |  |  |  |
| met-b-161 | CD3 on 127+DN T | NA | 497 | 1,617,019 | 2015 |  |  |  |  |  |  |  |  |  |  |  |  |  |  |  |  |  |  |  |  |  |  |  |  |
| met-b-160 | CD3 on DN T | NA | 497 | 1,617,019 | 2015 |  |  |  |  |  |  |  |  |  |  |  |  |  |  |  |  |  |  |  |  |  |  |  |  |
| ebi-a-GCST90001971 | FSC-A on lymphocyte | NA | 3,113 | 14,903,739 | 2020 |  |  |  |  |  |  |  |  |  |  |  |  |  |  |  |  |  |  |  |  |  |  |  |  |
| ebi-a-GCST90002079 | SSC-A on lymphocyte | NA | 3,113 | 14,903,739 | 2020 |  |  |  |  |  |  |  |  |  |  |  |  |  |  |  |  |  |  |  |  |  |  |  |  |
| ebi-a-GCST90001830 | BAFF-R on CD20- B cell | NA | 3,648 | 13,966,232 | 2020 |  |  |  |  |  |  |  |  |  |  |  |  |  |  |  |  |  |  |  |  |  |  |  |  |
| ebi-a-GCST90001701 | BAFF-R on CD20- CD38- B cell | NA | 3,592 | 15,034,528 | 2020 |  |  |  |  |  |  |  |  |  |  |  |  |  |  |  |  |  |  |  |  |  |  |  |  |
| ebi-a-GCST90001702 | BAFF-R on CD24+ CD27+ B cell | NA | 3,657 | 15,048,951 | 2020 |  |  |  |  |  |  |  |  |  |  |  |  |  |  |  |  |  |  |  |  |  |  |  |  |
| ebi-a-GCST90001710 | BAFF-R on IgD- CD24- B cell | NA | 3,657 | 15,048,951 | 2020 |  |  |  |  |  |  |  |  |  |  |  |  |  |  |  |  |  |  |  |  |  |  |  |  |
| ebi-a-GCST90001711 | BAFF-R on IgD- CD27- B cell | NA | 3,657 | 15,048,951 | 2020 |  |  |  |  |  |  |  |  |  |  |  |  |  |  |  |  |  |  |  |  |  |  |  |  |
| ebi-a-GCST90001704 | BAFF-R on IgD+ CD24- B cell | NA | 3,657 | 15,048,951 | 2020 |  |  |  |  |  |  |  |  |  |  |  |  |  |  |  |  |  |  |  |  |  |  |  |  |
| ebi-a-GCST90001703 | BAFF-R on IgD+ CD24+ B cell | NA | 3,657 | 15,048,951 | 2020 |  |  |  |  |  |  |  |  |  |  |  |  |  |  |  |  |  |  |  |  |  |  |  |  |
| prot-a-427 | B-cell receptor CD22 | NA | 3,301 | 10,534,735 | 2020 |  |  |  |  |  |  |  |  |  |  |  |  |  |  |  |  |  |  |  |  |  |  |  |  |
| ebi-a-GCST90001907 | CCR7 on naive CD4+ T cell | NA | 2,910 | 14,842,727 | 2020 |  |  |  |  |  |  |  |  |  |  |  |  |  |  |  |  |  |  |  |  |  |  |  |  |
| ebi-a-GCST90001908 | CCR7 on naive CD8+ T cell | NA | 2,910 | 14,842,727 | 2020 |  |  |  |  |  |  |  |  |  |  |  |  |  |  |  |  |  |  |  |  |  |  |  |  |
| ebi-a-GCST90001683 | CD127- CD8+ T cell %CD8+ T cell | NA | 3,403 | 15,134,952 | 2020 |  |  |  |  |  |  |  |  |  |  |  |  |  |  |  |  |  |  |  |  |  |  |  |  |
| ebi-a-GCST90001682 | CD127- CD8+ T cell %T cell | NA | 3,403 | 15,134,952 | 2020 |  |  |  |  |  |  |  |  |  |  |  |  |  |  |  |  |  |  |  |  |  |  |  |  |
| ebi-a-GCST90001684 | CD127- CD8+ T cell Absolute Count | NA | 3,371 | 15,122,502 | 2020 |  |  |  |  |  |  |  |  |  |  |  |  |  |  |  |  |  |  |  |  |  |  |  |  |
| ebi-a-GCST90001930 | CD127 on CD28- CD8+ T cell | NA | 2920 | 14849646 | 2020 |  |  |  |  |  |  |  |  |  |  |  |  |  |  |  |  |  |  |  |  |  |  |  |  |
| ebi-a-GCST90001924 | CD127 on CD28+ CD4+ T cell | NA | 2920 | 14849646 | 2020 |  |  |  |  |  |  |  |  |  |  |  |  |  |  |  |  |  |  |  |  |  |  |  |  |
| ebi-a-GCST90001928 | CD127 on CD28+ CD45RA- CD8+ T cell | NA | 2920 | 14849646 | 2020 |  |  |  |  |  |  |  |  |  |  |  |  |  |  |  |  |  |  |  |  |  |  |  |  |
| ebi-a-GCST90001929 | CD127 on CD28+ CD45RA+ CD8+ T cell | NA | 2920 | 14551148 | 2020 |  |  |  |  |  |  |  |  |  |  |  |  |  |  |  |  |  |  |  |  |  |  |  |  |
| ebi-a-GCST90001925 | CD127 on CD28+ CD4-CD8- T cell | NA | 2918 | 14849609 | 2020 |  |  |  |  |  |  |  |  |  |  |  |  |  |  |  |  |  |  |  |  |  |  |  |  |
| ebi-a-GCST90001931 | CD127 on CD4+ T cell | NA | 2,920 | 4,642,846 | 2015 |  |  |  |  |  |  |  |  |  |  |  |  |  |  |  |  |  |  |  |  |  |  |  |  |
| ebi-a-GCST90001923 | CD127 on CD45RA- CD4 not regulatory T cell | NA | 2,920 | 14,849,646 | 2015 |  |  |  |  |  |  |  |  |  |  |  |  |  |  |  |  |  |  |  |  |  |  |  |  |
| ebi-a-GCST90001932 | CD127 on CD45RA+ CD4+ T cell | NA | 2,920 | 14,849,646 | 2020 |  |  |  |  |  |  |  |  |  |  |  |  |  |  |  |  |  |  |  |  |  |  |  |  |
| ebi-a-GCST90001927 | CD127 on CD8+ T cell | NA | 2,920 | 14,849,646 | 2020 |  |  |  |  |  |  |  |  |  |  |  |  |  |  |  |  |  |  |  |  |  |  |  |  |
| ebi-a-GCST90001922 | CD127 on T cell | NA | 2,920 | 14,849,646 | 2020 |  |  |  |  |  |  |  |  |  |  |  |  |  |  |  |  |  |  |  |  |  |  |  |  |
| ebi-a-GCST90002028 | CD19 on B cell | NA | 3,112 | 14,903,706 | 2020 |  |  |  |  |  |  |  |  |  |  |  |  |  |  |  |  |  |  |  |  |  |  |  |  |
| ebi-a-GCST90001721 | CD19 on CD20- B cell | NA | 3,656 | 15,048,785 | 2020 |  |  |  |  |  |  |  |  |  |  |  |  |  |  |  |  |  |  |  |  |  |  |  |  |
| ebi-a-GCST90001722 | CD19 on CD20- CD38- B cell | NA | 3,595 | 15,036,689 | 2020 |  |  |  |  |  |  |  |  |  |  |  |  |  |  |  |  |  |  |  |  |  |  |  |  |
| ebi-a-GCST90001723 | CD19 on CD24+ CD27+ B cell | NA | 3,656 | 15,048,930 | 2020 |  |  |  |  |  |  |  |  |  |  |  |  |  |  |  |  |  |  |  |  |  |  |  |  |
| ebi-a-GCST90001731 | CD19 on IgD- CD24- B cell | NA | 3,655 | 15,045,985 | 2020 |  |  |  |  |  |  |  |  |  |  |  |  |  |  |  |  |  |  |  |  |  |  |  |  |
| ebi-a-GCST90001732 | CD19 on IgD- CD27- B cell | NA | 3,656 | 15,048,617 | 2020 |  |  |  |  |  |  |  |  |  |  |  |  |  |  |  |  |  |  |  |  |  |  |  |  |
| ebi-a-GCST90001733 | CD19 on IgD- CD38- B cell | NA | 3,657 | 15,048,951 | 2020 |  |  |  |  |  |  |  |  |  |  |  |  |  |  |  |  |  |  |  |  |  |  |  |  |
| ebi-a-GCST90001734 | CD19 on IgD- CD38+ B cell | NA | 3,658 | 15,049,152 | 2020 |  |  |  |  |  |  |  |  |  |  |  |  |  |  |  |  |  |  |  |  |  |  |  |  |
| ebi-a-GCST90001735 | CD19 on IgD- CD38dim B cell | NA | 3,657 | 15,048,951 | 2020 |  |  |  |  |  |  |  |  |  |  |  |  |  |  |  |  |  |  |  |  |  |  |  |  |
| ebi-a-GCST90001741 | CD19 on IgD+ B cell | NA | 3,658 | 15,048,965 | 2020 |  |  |  |  |  |  |  |  |  |  |  |  |  |  |  |  |  |  |  |  |  |  |  |  |
| ebi-a-GCST90001725 | CD19 on IgD+ CD24- B cell | NA | 3,656 | 15,046,318 | 2020 |  |  |  |  |  |  |  |  |  |  |  |  |  |  |  |  |  |  |  |  |  |  |  |  |
| ebi-a-GCST90001724 | CD19 on IgD+ CD24+ B cell | NA | 3,656 | 15,048,930 | 2020 |  |  |  |  |  |  |  |  |  |  |  |  |  |  |  |  |  |  |  |  |  |  |  |  |
| ebi-a-GCST90001726 | CD19 on IgD+ CD38- B cell | NA | 3,645 | 15,045,785 | 2020 |  |  |  |  |  |  |  |  |  |  |  |  |  |  |  |  |  |  |  |  |  |  |  |  |
| ebi-a-GCST90001727 | CD19 on IgD+ CD38- naive B cell | NA | 1,830 | 14,178,548 | 2020 |  |  |  |  |  |  |  |  |  |  |  |  |  |  |  |  |  |  |  |  |  |  |  |  |
| ebi-a-GCST90001728 | CD19 on IgD+ CD38- unswitched memory B cell | NA | 1,833 | 14,184,578 | 2020 |  |  |  |  |  |  |  |  |  |  |  |  |  |  |  |  |  |  |  |  |  |  |  |  |
| ebi-a-GCST90001729 | CD19 on IgD+ CD38+ B cell | NA | 3,657 | 15,048,951 | 2020 |  |  |  |  |  |  |  |  |  |  |  |  |  |  |  |  |  |  |  |  |  |  |  |  |
| ebi-a-GCST90001730 | CD19 on IgD+ CD38dim B cell | NA | 3,657 | 15,048,951 | 2020 |  |  |  |  |  |  |  |  |  |  |  |  |  |  |  |  |  |  |  |  |  |  |  |  |
| ebi-a-GCST90001736 | CD19 on memory B cell | NA | 3,657 | 15,048,951 | 2020 |  |  |  |  |  |  |  |  |  |  |  |  |  |  |  |  |  |  |  |  |  |  |  |  |
| ebi-a-GCST90001737 | CD19 on naive-mature B cell | NA | 3,653 | 15,046,216 | 2020 |  |  |  |  |  |  |  |  |  |  |  |  |  |  |  |  |  |  |  |  |  |  |  |  |
| ebi-a-GCST90001740 | CD19 on switched memory B cell | NA | 3,657 | 15,048,951 | 2020 |  |  |  |  |  |  |  |  |  |  |  |  |  |  |  |  |  |  |  |  |  |  |  |  |
| ebi-a-GCST90001742 | CD19 on transitional B cell | NA | 3,657 | 15,048,951 | 2020 |  |  |  |  |  |  |  |  |  |  |  |  |  |  |  |  |  |  |  |  |  |  |  |  |
| ebi-a-GCST90001738 | CD19 on unswitched memory B cell | NA | 3,656 | 15,048,930 | 2020 |  |  |  |  |  |  |  |  |  |  |  |  |  |  |  |  |  |  |  |  |  |  |  |  |
| ebi-a-GCST90001419 | CD20- B cell %B cell | NA | 3,658 | 15,048,965 | 2020 |  |  |  |  |  |  |  |  |  |  |  |  |  |  |  |  |  |  |  |  |  |  |  |  |
| ebi-a-GCST90001443 | CD20- B cell %lymphocyte | NA | 3,657 | 15,048,951 | 2020 |  |  |  |  |  |  |  |  |  |  |  |  |  |  |  |  |  |  |  |  |  |  |  |  |
| ebi-a-GCST90001421 | CD20- B cell Absolute Count | NA | 3,657 | 15,048,951 | 2020 |  |  |  |  |  |  |  |  |  |  |  |  |  |  |  |  |  |  |  |  |  |  |  |  |
| ebi-a-GCST90001422 | CD20- CD38- B cell %B cell | NA | 3,659 | 15,048,978 | 2020 |  |  |  |  |  |  |  |  |  |  |  |  |  |  |  |  |  |  |  |  |  |  |  |  |
| ebi-a-GCST90001444 | CD20- CD38- B cell %lymphocyte | NA | 3,658 | 15,048,964 | 2020 |  |  |  |  |  |  |  |  |  |  |  |  |  |  |  |  |  |  |  |  |  |  |  |  |
| ebi-a-GCST90001423 | CD20- CD38- B cell Absolute Count | NA | 3,658 | 15,048,964 | 2020 |  |  |  |  |  |  |  |  |  |  |  |  |  |  |  |  |  |  |  |  |  |  |  |  |
| ebi-a-GCST90001743 | CD20 on B cell | NA | 3,660 | 15,049,199 | 2020 |  |  |  |  |  |  |  |  |  |  |  |  |  |  |  |  |  |  |  |  |  |  |  |  |
| ebi-a-GCST90001744 | CD20 on CD20- CD38- B cell | NA | 3,606 | 15,039,196 | 2020 |  |  |  |  |  |  |  |  |  |  |  |  |  |  |  |  |  |  |  |  |  |  |  |  |
| ebi-a-GCST90001745 | CD20 on CD24+ CD27+ B cell | NA | 3,657 | 15,048,951 | 2020 |  |  |  |  |  |  |  |  |  |  |  |  |  |  |  |  |  |  |  |  |  |  |  |  |
| ebi-a-GCST90001753 | CD20 on IgD- CD24- B cell | NA | 3,657 | 15,048,951 | 2020 |  |  |  |  |  |  |  |  |  |  |  |  |  |  |  |  |  |  |  |  |  |  |  |  |
| ebi-a-GCST90001754 | CD20 on IgD- CD27- B cell | NA | 3,657 | 12,797,111 | 2020 |  |  |  |  |  |  |  |  |  |  |  |  |  |  |  |  |  |  |  |  |  |  |  |  |
| ebi-a-GCST90001755 | CD20 on IgD- CD38- B cell | NA | 3,657 | 15,048,951 | 2020 |  |  |  |  |  |  |  |  |  |  |  |  |  |  |  |  |  |  |  |  |  |  |  |  |
| ebi-a-GCST90001756 | CD20 on IgD- CD38+ B cell | NA | 3,659 | 15,049,184 | 2020 |  |  |  |  |  |  |  |  |  |  |  |  |  |  |  |  |  |  |  |  |  |  |  |  |
| ebi-a-GCST90001757 | CD20 on IgD- CD38dim B cell | NA | 3,657 | 14,996,523 | 2020 |  |  |  |  |  |  |  |  |  |  |  |  |  |  |  |  |  |  |  |  |  |  |  |  |
| ebi-a-GCST90001762 | CD20 on IgD+ B cell | NA | 3,658 | 15,048,965 | 2020 |  |  |  |  |  |  |  |  |  |  |  |  |  |  |  |  |  |  |  |  |  |  |  |  |
| ebi-a-GCST90001747 | CD20 on IgD+ CD24- B cell | NA | 3,657 | 15,048,951 | 2020 |  |  |  |  |  |  |  |  |  |  |  |  |  |  |  |  |  |  |  |  |  |  |  |  |
| ebi-a-GCST90001746 | CD20 on IgD+ CD24+ B cell | NA | 3,657 | 15,048,951 | 2020 |  |  |  |  |  |  |  |  |  |  |  |  |  |  |  |  |  |  |  |  |  |  |  |  |
| ebi-a-GCST90001748 | CD20 on IgD+ CD38- B cell | NA | 3,645 | 15,045,785 | 2020 |  |  |  |  |  |  |  |  |  |  |  |  |  |  |  |  |  |  |  |  |  |  |  |  |
| ebi-a-GCST90001749 | CD20 on IgD+ CD38- naive B cell | NA | 1,834 | 14,184,596 | 2020 |  |  |  |  |  |  |  |  |  |  |  |  |  |  |  |  |  |  |  |  |  |  |  |  |
| ebi-a-GCST90001750 | CD20 on IgD+ CD38- unswitched memory B cell | NA | 1,836 | 14,184,873 | 2020 |  |  |  |  |  |  |  |  |  |  |  |  |  |  |  |  |  |  |  |  |  |  |  |  |
| ebi-a-GCST90001751 | CD20 on IgD+ CD38+ B cell | NA | 3,657 | 15,048,951 | 2020 |  |  |  |  |  |  |  |  |  |  |  |  |  |  |  |  |  |  |  |  |  |  |  |  |
| ebi-a-GCST90001752 | CD20 on IgD+ CD38dim B cell | NA | 3,657 | 15,048,951 | 2020 |  |  |  |  |  |  |  |  |  |  |  |  |  |  |  |  |  |  |  |  |  |  |  |  |
| ebi-a-GCST90001758 | CD20 on memory B cell | NA | 3,657 | 15,048,951 | 2020 |  |  |  |  |  |  |  |  |  |  |  |  |  |  |  |  |  |  |  |  |  |  |  |  |
| ebi-a-GCST90001759 | CD20 on naive-mature B cell | NA | 3,657 | 15,048,951 | 2020 |  |  |  |  |  |  |  |  |  |  |  |  |  |  |  |  |  |  |  |  |  |  |  |  |
| ebi-a-GCST90001761 | CD20 on switched memory B cell | NA | 3,657 | 15,048,951 | 2020 |  |  |  |  |  |  |  |  |  |  |  |  |  |  |  |  |  |  |  |  |  |  |  |  |
| ebi-a-GCST90001763 | CD20 on transitional B cell | NA | 3,657 | 15,048,951 | 2020 |  |  |  |  |  |  |  |  |  |  |  |  |  |  |  |  |  |  |  |  |  |  |  |  |
| ebi-a-GCST90001760 | CD20 on unswitched memory B cell | NA | 3,657 | 15,048,951 | 2020 |  |  |  |  |  |  |  |  |  |  |  |  |  |  |  |  |  |  |  |  |  |  |  |  |
| ebi-a-GCST90001764 | CD24 on CD24+ CD27+ B cell | NA | 3,654 | 4,335,912 | 2020 |  |  |  |  |  |  |  |  |  |  |  |  |  |  |  |  |  |  |  |  |  |  |  |  |
| met-b-145 | CD24 on IgA+ B | NA | 497 | 1,626,867 | 2020 |  |  |  |  |  |  |  |  |  |  |  |  |  |  |  |  |  |  |  |  |  |  |  |  |
| ebi-a-GCST90001769 | CD24 on IgD- CD38- B cell | NA | 3,648 | 15,044,894 | 2020 |  |  |  |  |  |  |  |  |  |  |  |  |  |  |  |  |  |  |  |  |  |  |  |  |
| ebi-a-GCST90001770 | CD24 on IgD- CD38dim B cell | NA | 3,649 | 4,520,893 | 2020 |  |  |  |  |  |  |  |  |  |  |  |  |  |  |  |  |  |  |  |  |  |  |  |  |
| ebi-a-GCST90001765 | CD24 on IgD+ CD24+ B cell | NA | 3,655 | 15,048,612 | 2020 |  |  |  |  |  |  |  |  |  |  |  |  |  |  |  |  |  |  |  |  |  |  |  |  |
| ebi-a-GCST90001766 | CD24 on IgD+ CD38- B cell | NA | 3,638 | 15,041,901 | 2020 |  |  |  |  |  |  |  |  |  |  |  |  |  |  |  |  |  |  |  |  |  |  |  |  |
| ebi-a-GCST90001767 | CD24 on IgD+ CD38- unswitched memory B cell | NA | 1,832 | 14,177,072 | 2020 |  |  |  |  |  |  |  |  |  |  |  |  |  |  |  |  |  |  |  |  |  |  |  |  |
| ebi-a-GCST90001768 | CD24 on IgD+ CD38+ B cell | NA | 3,655 | 15,048,617 | 2020 |  |  |  |  |  |  |  |  |  |  |  |  |  |  |  |  |  |  |  |  |  |  |  |  |
| ebi-a-GCST90001771 | CD24 on memory B cell | NA | 3,653 | 15,048,573 | 2020 |  |  |  |  |  |  |  |  |  |  |  |  |  |  |  |  |  |  |  |  |  |  |  |  |
| ebi-a-GCST90001773 | CD24 on switched memory B cell | NA | 3,652 | 15,048,559 | 2020 |  |  |  |  |  |  |  |  |  |  |  |  |  |  |  |  |  |  |  |  |  |  |  |  |
| ebi-a-GCST90001774 | CD24 on transitional B cell | NA | 3,656 | 15,048,923 | 2020 |  |  |  |  |  |  |  |  |  |  |  |  |  |  |  |  |  |  |  |  |  |  |  |  |
| ebi-a-GCST90001772 | CD24 on unswitched memory B cell | NA | 3,655 | 15,048,612 | 2020 |  |  |  |  |  |  |  |  |  |  |  |  |  |  |  |  |  |  |  |  |  |  |  |  |
| ebi-a-GCST90001417 | CD24+ CD27+ B cell %B cell | NA | 3,657 | 15,048,951 | 2020 |  |  |  |  |  |  |  |  |  |  |  |  |  |  |  |  |  |  |  |  |  |  |  |  |
| ebi-a-GCST90001442 | CD24+ CD27+ B cell %lymphocyte | NA | 3,656 | 15,048,937 | 2020 |  |  |  |  |  |  |  |  |  |  |  |  |  |  |  |  |  |  |  |  |  |  |  |  |
| ebi-a-GCST90001418 | CD24+ CD27+ B cell Absolute Count | NA | 3,656 | 15,048,937 | 2020 |  |  |  |  |  |  |  |  |  |  |  |  |  |  |  |  |  |  |  |  |  |  |  |  |
| ebi-a-GCST90001943 | CD25 on activated & secreting CD4 regulatory T cell | NA | 3435 | 15147117 | 2020 |  |  |  |  |  |  |  |  |  |  |  |  |  |  |  |  |  |  |  |  |  |  |  |  |
| ebi-a-GCST90001939 | CD25 on activated CD4 regulatory T cell | NA | 3435 | 15147117 | 2020 |  |  |  |  |  |  |  |  |  |  |  |  |  |  |  |  |  |  |  |  |  |  |  |  |
| ebi-a-GCST90001775 | CD25 on B cell | NA | 3660 | 15049199 | 2020 |  |  |  |  |  |  |  |  |  |  |  |  |  |  |  |  |  |  |  |  |  |  |  |  |
| ebi-a-GCST90001776 | CD25 on CD20- CD38- B cell | NA | 3505 | 15014292 | 2020 |  |  |  |  |  |  |  |  |  |  |  |  |  |  |  |  |  |  |  |  |  |  |  |  |
| ebi-a-GCST90001777 | CD25 on CD24+ CD27+ B cell | NA | 3,657 | 15,048,951 | 2020 |  |  |  |  |  |  |  |  |  |  |  |  |  |  |  |  |  |  |  |  |  |  |  |  |
| ebi-a-GCST90001962 | CD25 on CD28+ CD4+ T cell | NA | 2920 | 4733784 | 2020 |  |  |  |  |  |  |  |  |  |  |  |  |  |  |  |  |  |  |  |  |  |  |  |  |
| ebi-a-GCST90001940 | CD25 on CD39+ activated CD4 regulatory T cell | NA | 3435 | 15147117 | 2020 |  |  |  |  |  |  |  |  |  |  |  |  |  |  |  |  |  |  |  |  |  |  |  |  |
| ebi-a-GCST90001935 | CD25 on CD39+ CD4 regulatory T cell | NA | 3434 | 15146814 | 2020 |  |  |  |  |  |  |  |  |  |  |  |  |  |  |  |  |  |  |  |  |  |  |  |  |
| ebi-a-GCST90001961 | CD25 on CD39+ CD4+ T cell | NA | 2920 | 14849646 | 2020 |  |  |  |  |  |  |  |  |  |  |  |  |  |  |  |  |  |  |  |  |  |  |  |  |
| ebi-a-GCST90001938 | CD25 on CD39+ resting CD4 regulatory T cell | NA | 3118 | 15067285 | 2020 |  |  |  |  |  |  |  |  |  |  |  |  |  |  |  |  |  |  |  |  |  |  |  |  |
| ebi-a-GCST90001942 | CD25 on CD39+ secreting CD4 regulatory T cell | NA | 3435 | 15147117 | 2020 |  |  |  |  |  |  |  |  |  |  |  |  |  |  |  |  |  |  |  |  |  |  |  |  |
| ebi-a-GCST90001936 | CD25 on CD4 regulatory T cell | NA | 3435 | 15147117 | 2020 |  |  |  |  |  |  |  |  |  |  |  |  |  |  |  |  |  |  |  |  |  |  |  |  |
| ebi-a-GCST90001960 | CD25 on CD4+ T cell | NA | 2920 | 14849646 | 2020 |  |  |  |  |  |  |  |  |  |  |  |  |  |  |  |  |  |  |  |  |  |  |  |  |
| ebi-a-GCST90001933 | CD25 on CD45RA- CD4 not regulatory T cell | NA | 3435 | 15147117 | 2020 |  |  |  |  |  |  |  |  |  |  |  |  |  |  |  |  |  |  |  |  |  |  |  |  |
| ebi-a-GCST90001934 | CD25 on CD45RA+ CD4 not regulatory T cell | NA | 3435 | 15147117 | 2020 |  |  |  |  |  |  |  |  |  |  |  |  |  |  |  |  |  |  |  |  |  |  |  |  |
| ebi-a-GCST90001785 | CD25 on IgD- CD24- B cell | NA | 3649 | 15047424 | 2020 |  |  |  |  |  |  |  |  |  |  |  |  |  |  |  |  |  |  |  |  |  |  |  |  |
| ebi-a-GCST90001786 | CD25 on IgD- CD27- B cell | NA | 3,651 | 15,048,044 | 2020 |  |  |  |  |  |  |  |  |  |  |  |  |  |  |  |  |  |  |  |  |  |  |  |  |
| ebi-a-GCST90001787 | CD25 on IgD- CD38- B cell | NA | 3657 | 15048951 | 2020 |  |  |  |  |  |  |  |  |  |  |  |  |  |  |  |  |  |  |  |  |  |  |  |  |
| ebi-a-GCST90001788 | CD25 on IgD- CD38+ B cell | NA | 3656 | 15049126 | 2020 |  |  |  |  |  |  |  |  |  |  |  |  |  |  |  |  |  |  |  |  |  |  |  |  |
| ebi-a-GCST90001789 | CD25 on IgD- CD38dim B cell | NA | 3657 | 15048951 | 2020 |  |  |  |  |  |  |  |  |  |  |  |  |  |  |  |  |  |  |  |  |  |  |  |  |
| ebi-a-GCST90001794 | CD25 on IgD+ B cell | NA | 3658 | 15048965 | 2020 |  |  |  |  |  |  |  |  |  |  |  |  |  |  |  |  |  |  |  |  |  |  |  |  |
| ebi-a-GCST90001779 | CD25 on IgD+ CD24- B cell | NA | 3657 | 15048951 | 2020 |  |  |  |  |  |  |  |  |  |  |  |  |  |  |  |  |  |  |  |  |  |  |  |  |
| ebi-a-GCST90001778 | CD25 on IgD+ CD24+ B cell | NA | 3657 | 15048951 | 2020 |  |  |  |  |  |  |  |  |  |  |  |  |  |  |  |  |  |  |  |  |  |  |  |  |
| ebi-a-GCST90001780 | CD25 on IgD+ CD38- B cell | NA | 3642 | 15045564 | 2015 |  |  |  |  |  |  |  |  |  |  |  |  |  |  |  |  |  |  |  |  |  |  |  |  |
| ebi-a-GCST90001781 | CD25 on IgD+ CD38- naive B cell | NA | 1835 | 14184876 | 2018 |  |  |  |  |  |  |  |  |  |  |  |  |  |  |  |  |  |  |  |  |  |  |  |  |
| ebi-a-GCST90001782 | CD25 on IgD+ CD38- unswitched memory B cell | NA | 1837 | 14185043 | 2019 |  |  |  |  |  |  |  |  |  |  |  |  |  |  |  |  |  |  |  |  |  |  |  |  |
| ebi-a-GCST90001783 | CD25 on IgD+ CD38+ B cell | NA | 3656 | 15048939 | 2020 |  |  |  |  |  |  |  |  |  |  |  |  |  |  |  |  |  |  |  |  |  |  |  |  |
| ebi-a-GCST90001784 | CD25 on IgD+ CD38dim B cell | NA | 3657 | 15048951 | 2015 |  |  |  |  |  |  |  |  |  |  |  |  |  |  |  |  |  |  |  |  |  |  |  |  |
| ebi-a-GCST90001790 | CD25 on memory B cell | NA | 3657 | 15048951 | 2020 |  |  |  |  |  |  |  |  |  |  |  |  |  |  |  |  |  |  |  |  |  |  |  |  |
| ebi-a-GCST90001791 | CD25 on naive-mature B cell | NA | 3657 | 15048951 | 2020 |  |  |  |  |  |  |  |  |  |  |  |  |  |  |  |  |  |  |  |  |  |  |  |  |
| ebi-a-GCST90001937 | CD25 on resting CD4 regulatory T cell | NA | 3434 | 15147104 | 2020 |  |  |  |  |  |  |  |  |  |  |  |  |  |  |  |  |  |  |  |  |  |  |  |  |
| ebi-a-GCST90001941 | CD25 on secreting CD4 regulatory T cell | NA | 3435 | 15147117 | 2020 |  |  |  |  |  |  |  |  |  |  |  |  |  |  |  |  |  |  |  |  |  |  |  |  |
| ebi-a-GCST90001793 | CD25 on switched memory B cell | NA | 3657 | 15048951 | 2020 |  |  |  |  |  |  |  |  |  |  |  |  |  |  |  |  |  |  |  |  |  |  |  |  |
| ebi-a-GCST90001795 | CD25 on transitional B cell | NA | 3657 | 15048951 | 2020 |  |  |  |  |  |  |  |  |  |  |  |  |  |  |  |  |  |  |  |  |  |  |  |  |
| ebi-a-GCST90001792 | CD25 on unswitched memory B cell | NA | 3657 | 15048951 | 2020 |  |  |  |  |  |  |  |  |  |  |  |  |  |  |  |  |  |  |  |  |  |  |  |  |
| ebi-a-GCST90001505 | CD25++ CD4+ T cell %CD4+ T cell | NA | 3437 | 15144196 | 2020 |  |  |  |  |  |  |  |  |  |  |  |  |  |  |  |  |  |  |  |  |  |  |  |  |
| ebi-a-GCST90001506 | CD25++ CD4+ T cell %T cell | NA | 3437 | 15144196 | 2020 |  |  |  |  |  |  |  |  |  |  |  |  |  |  |  |  |  |  |  |  |  |  |  |  |
| ebi-a-GCST90001504 | CD25++ CD4+ T cell Absolute Count | NA | 3405 | 15131843 | 2020 |  |  |  |  |  |  |  |  |  |  |  |  |  |  |  |  |  |  |  |  |  |  |  |  |
| ebi-a-GCST90001511 | CD25++ CD45RA- CD4 not regulatory T cell %CD4+ T cell | NA | 3437 | 15144196 | 2020 |  |  |  |  |  |  |  |  |  |  |  |  |  |  |  |  |  |  |  |  |  |  |  |  |
| ebi-a-GCST90001512 | CD25++ CD45RA- CD4 not regulatory T cell %T cell | NA | 3437 | 15144196 | 2020 |  |  |  |  |  |  |  |  |  |  |  |  |  |  |  |  |  |  |  |  |  |  |  |  |
| ebi-a-GCST90001510 | CD25++ CD45RA- CD4 not regulatory T cell Absolute Count | NA | 3405 | 15131843 | 2020 |  |  |  |  |  |  |  |  |  |  |  |  |  |  |  |  |  |  |  |  |  |  |  |  |
| ebi-a-GCST90001508 | CD25++ CD45RA+ CD4 not regulatory T cell %CD4+ T cell | NA | 3437 | 15144196 | 2020 |  |  |  |  |  |  |  |  |  |  |  |  |  |  |  |  |  |  |  |  |  |  |  |  |
| ebi-a-GCST90001509 | CD25++ CD45RA+ CD4 not regulatory T cell %T cell | NA | 3437 | 15144196 | 2020 |  |  |  |  |  |  |  |  |  |  |  |  |  |  |  |  |  |  |  |  |  |  |  |  |
| ebi-a-GCST90001507 | CD25++ CD45RA+ CD4 not regulatory T cell Absolute Count | NA | 3405 | 15131843 | 2020 |  |  |  |  |  |  |  |  |  |  |  |  |  |  |  |  |  |  |  |  |  |  |  |  |
| ebi-a-GCST90001680 | CD25++ CD8+ T cell %CD8+ T cell | NA | 3162 | 15041080 | 2020 |  |  |  |  |  |  |  |  |  |  |  |  |  |  |  |  |  |  |  |  |  |  |  |  |
| ebi-a-GCST90001679 | CD25++ CD8+ T cell %T cell | NA | 3162 | 15041080 | 2020 |  |  |  |  |  |  |  |  |  |  |  |  |  |  |  |  |  |  |  |  |  |  |  |  |
| ebi-a-GCST90001681 | CD25++ CD8+ T cell Absolute Count | NA | 3130 | 15027914 | 2020 |  |  |  |  |  |  |  |  |  |  |  |  |  |  |  |  |  |  |  |  |  |  |  |  |
| ebi-a-GCST90001796 | CD27 on CD20- B cell | NA | 3,634 | 15,039,158 | 2020 |  |  |  |  |  |  |  |  |  |  |  |  |  |  |  |  |  |  |  |  |  |  |  |  |
| ebi-a-GCST90001797 | CD27 on CD20- CD38- B cell | NA | 3,592 | 15,030,398 | 2020 |  |  |  |  |  |  |  |  |  |  |  |  |  |  |  |  |  |  |  |  |  |  |  |  |
| ebi-a-GCST90001798 | CD27 on CD24+ CD27+ B cell | NA | 3,657 | 15,048,951 | 2020 |  |  |  |  |  |  |  |  |  |  |  |  |  |  |  |  |  |  |  |  |  |  |  |  |
| met-b-155 | CD27 on CD4 T | NA | 497 | 1,623,131 | 2020 |  |  |  |  |  |  |  |  |  |  |  |  |  |  |  |  |  |  |  |  |  |  |  |  |
| met-b-157 | CD27 on CD8 T | NA | 497 | 1,623,131 | 2020 |  |  |  |  |  |  |  |  |  |  |  |  |  |  |  |  |  |  |  |  |  |  |  |  |
| met-b-144 | CD27 on IgA+B | NA | 497 | 1,623,996 | 2020 |  |  |  |  |  |  |  |  |  |  |  |  |  |  |  |  |  |  |  |  |  |  |  |  |
| ebi-a-GCST90001802 | CD27 on IgD- CD38- B cell | NA | 3,657 | 15,048,951 | 2020 |  |  |  |  |  |  |  |  |  |  |  |  |  |  |  |  |  |  |  |  |  |  |  |  |
| ebi-a-GCST90001803 | CD27 on IgD- CD38+ B cell | NA | 3,642 | 15,045,891 | 2020 |  |  |  |  |  |  |  |  |  |  |  |  |  |  |  |  |  |  |  |  |  |  |  |  |
| ebi-a-GCST90001804 | CD27 on IgD- CD38dim B cell | NA | 3,657 | 15,048,951 | 2020 |  |  |  |  |  |  |  |  |  |  |  |  |  |  |  |  |  |  |  |  |  |  |  |  |
| ebi-a-GCST90001800 | CD27 on IgD+ CD24+ B cell | NA | 3,656 | 15,048,938 | 2020 |  |  |  |  |  |  |  |  |  |  |  |  |  |  |  |  |  |  |  |  |  |  |  |  |
| ebi-a-GCST90001801 | CD27 on IgD+ CD38- unswitched memory B cell | NA | 1,836 | 14,185,009 | 2020 |  |  |  |  |  |  |  |  |  |  |  |  |  |  |  |  |  |  |  |  |  |  |  |  |
| ebi-a-GCST90001805 | CD27 on memory B cell | NA | 3,657 | 15,048,951 | 2020 |  |  |  |  |  |  |  |  |  |  |  |  |  |  |  |  |  |  |  |  |  |  |  |  |
| ebi-a-GCST90001808 | CD27 on switched memory B cell | NA | 3,657 | 15,048,951 | 2020 |  |  |  |  |  |  |  |  |  |  |  |  |  |  |  |  |  |  |  |  |  |  |  |  |
| ebi-a-GCST90001799 | CD27 on T cell | NA | 1,838 | 14,185,091 | 2020 |  |  |  |  |  |  |  |  |  |  |  |  |  |  |  |  |  |  |  |  |  |  |  |  |
| ebi-a-GCST90001806 | CD27 on unswitched memory B cell | NA | 3,656 | 15,048,938 | 2020 |  |  |  |  |  |  |  |  |  |  |  |  |  |  |  |  |  |  |  |  |  |  |  |  |
| ebi-a-GCST90001674 | CD28- CD127- CD25++ CD8+ T cell %CD8+ T cell | NA | 3162 | 15041080 | 2020 |  |  |  |  |  |  |  |  |  |  |  |  |  |  |  |  |  |  |  |  |  |  |  |  |
| ebi-a-GCST90001673 | CD28- CD127- CD25++ CD8+ T cell %T cell | NA | 3162 | 15041080 | 2020 |  |  |  |  |  |  |  |  |  |  |  |  |  |  |  |  |  |  |  |  |  |  |  |  |
| ebi-a-GCST90001675 | CD28- CD127- CD25++ CD8+ T cell Absolute Count | NA | 3130 | 15027914 | 2020 |  |  |  |  |  |  |  |  |  |  |  |  |  |  |  |  |  |  |  |  |  |  |  |  |
| ebi-a-GCST90001677 | CD28- CD25++ CD8+ T cell %CD8+ T cell | NA | 3162 | 15041080 | 2020 |  |  |  |  |  |  |  |  |  |  |  |  |  |  |  |  |  |  |  |  |  |  |  |  |
| ebi-a-GCST90001676 | CD28- CD25++ CD8+ T cell %T cell | NA | 3162 | 4376332 | 2020 |  |  |  |  |  |  |  |  |  |  |  |  |  |  |  |  |  |  |  |  |  |  |  |  |
| ebi-a-GCST90001678 | CD28- CD25++ CD8+ T cell Absolute Count | NA | 3130 | 15027914 | 2020 |  |  |  |  |  |  |  |  |  |  |  |  |  |  |  |  |  |  |  |  |  |  |  |  |
| ebi-a-GCST90001653 | CD28- CD4-CD8- T cell %CD4-CD8- T cell | NA | 3440 | 15147619 | 2020 |  |  |  |  |  |  |  |  |  |  |  |  |  |  |  |  |  |  |  |  |  |  |  |  |
| ebi-a-GCST90001694 | CD28- CD4-CD8- T cell %T cell | NA | 3440 | 15147619 | 2020 |  |  |  |  |  |  |  |  |  |  |  |  |  |  |  |  |  |  |  |  |  |  |  |  |
| ebi-a-GCST90001654 | CD28- CD4-CD8- T cell Absolute Count | NA | 3408 | 15135292 | 2020 |  |  |  |  |  |  |  |  |  |  |  |  |  |  |  |  |  |  |  |  |  |  |  |  |
| ebi-a-GCST90001686 | CD28- CD8+ T cell %CD8+ T cell | NA | 3440 | 15147619 | 2020 |  |  |  |  |  |  |  |  |  |  |  |  |  |  |  |  |  |  |  |  |  |  |  |  |
| ebi-a-GCST90001685 | CD28- CD8+ T cell %T cell | NA | 3440 | 15147619 | 2020 |  |  |  |  |  |  |  |  |  |  |  |  |  |  |  |  |  |  |  |  |  |  |  |  |
| ebi-a-GCST90001687 | CD28- CD8+ T cell Absolute Count | NA | 3408 | 15135292 | 2020 |  |  |  |  |  |  |  |  |  |  |  |  |  |  |  |  |  |  |  |  |  |  |  |  |
| ebi-a-GCST90001662 | CD28- CD8dim T cell %CD8dim T cell | NA | 3440 | 15147619 | 2020 |  |  |  |  |  |  |  |  |  |  |  |  |  |  |  |  |  |  |  |  |  |  |  |  |
| ebi-a-GCST90001661 | CD28- CD8dim T cell %T cell | NA | 3440 | 15147619 | 2020 |  |  |  |  |  |  |  |  |  |  |  |  |  |  |  |  |  |  |  |  |  |  |  |  |
| ebi-a-GCST90001663 | CD28- CD8dim T cell Absolute Count | NA | 3408 | 15135292 | 2020 |  |  |  |  |  |  |  |  |  |  |  |  |  |  |  |  |  |  |  |  |  |  |  |  |
| ebi-a-GCST90001889 | CD28 on activated & secreting CD4 regulatory T cell | NA | 2920 | 14849646 | 2020 |  |  |  |  |  |  |  |  |  |  |  |  |  |  |  |  |  |  |  |  |  |  |  |  |
| ebi-a-GCST90001902 | CD28 on activated CD4 regulatory T cell | NA | 2920 | 14849646 | 2020 |  |  |  |  |  |  |  |  |  |  |  |  |  |  |  |  |  |  |  |  |  |  |  |  |
| ebi-a-GCST90001894 | CD28 on CD28+ CD4+ T cell | NA | 2920 | 14849646 | 2020 |  |  |  |  |  |  |  |  |  |  |  |  |  |  |  |  |  |  |  |  |  |  |  |  |
| ebi-a-GCST90001893 | CD28 on CD28+ CD45RA- CD8+ T cell | NA | 2920 | 14849646 | 2020 |  |  |  |  |  |  |  |  |  |  |  |  |  |  |  |  |  |  |  |  |  |  |  |  |
| ebi-a-GCST90001896 | CD28 on CD28+ CD45RA+ CD8+ T cell | NA | 2920 | 14849646 | 2020 |  |  |  |  |  |  |  |  |  |  |  |  |  |  |  |  |  |  |  |  |  |  |  |  |
| ebi-a-GCST90001895 | CD28 on CD28+ CD4-CD8- T cell | NA | 2919 | 929915 | 2020 |  |  |  |  |  |  |  |  |  |  |  |  |  |  |  |  |  |  |  |  |  |  |  |  |
| ebi-a-GCST90001886 | CD28 on CD39+ activated CD4 regulatory T cell | NA | 2920 | 14849646 | 2020 |  |  |  |  |  |  |  |  |  |  |  |  |  |  |  |  |  |  |  |  |  |  |  |  |
| ebi-a-GCST90001892 | CD28 on CD39+ CD4+ T cell | NA | 2920 | 14849646 | 2020 |  |  |  |  |  |  |  |  |  |  |  |  |  |  |  |  |  |  |  |  |  |  |  |  |
| ebi-a-GCST90001897 | CD28 on CD39+ CD8+ T cell | NA | 2914 | 14847481 | 2020 |  |  |  |  |  |  |  |  |  |  |  |  |  |  |  |  |  |  |  |  |  |  |  |  |
| ebi-a-GCST90001901 | CD28 on CD39+ resting CD4 regulatory T cell | NA | 2650 | 14779430 | 2020 |  |  |  |  |  |  |  |  |  |  |  |  |  |  |  |  |  |  |  |  |  |  |  |  |
| ebi-a-GCST90001888 | CD28 on CD39+ secreting CD4 regulatory T cell | NA | 2920 | 14849646 | 2020 |  |  |  |  |  |  |  |  |  |  |  |  |  |  |  |  |  |  |  |  |  |  |  |  |
| ebi-a-GCST90001899 | CD28 on CD4 regulatory T cell | NA | 2920 | 14849646 | 2020 |  |  |  |  |  |  |  |  |  |  |  |  |  |  |  |  |  |  |  |  |  |  |  |  |
| ebi-a-GCST90001898 | CD28 on CD4+ T cell | NA | 2920 | 14849646 | 2020 |  |  |  |  |  |  |  |  |  |  |  |  |  |  |  |  |  |  |  |  |  |  |  |  |
| ebi-a-GCST90001891 | CD28 on CD45RA- CD4 not regulatory T cell | NA | 2920 | 14849646 | 2020 |  |  |  |  |  |  |  |  |  |  |  |  |  |  |  |  |  |  |  |  |  |  |  |  |
| ebi-a-GCST90001890 | CD28 on CD45RA+ CD4+ T cell | NA | 2920 | 13468774 | 2020 |  |  |  |  |  |  |  |  |  |  |  |  |  |  |  |  |  |  |  |  |  |  |  |  |
| ebi-a-GCST90001900 | CD28 on resting CD4 regulatory T cell | NA | 2919 | 4824972 | 2020 |  |  |  |  |  |  |  |  |  |  |  |  |  |  |  |  |  |  |  |  |  |  |  |  |
| ebi-a-GCST90001887 | CD28 on secreting CD4 regulatory T cell | NA | 2920 | 14844808 | 2020 |  |  |  |  |  |  |  |  |  |  |  |  |  |  |  |  |  |  |  |  |  |  |  |  |
| ebi-a-GCST90001692 | CD28+ CD45RA- CD8+ T cell %CD8+ T cell | NA | 3440 | 15147619 | 2020 |  |  |  |  |  |  |  |  |  |  |  |  |  |  |  |  |  |  |  |  |  |  |  |  |
| ebi-a-GCST90001691 | CD28+ CD45RA- CD8+ T cell %T cell | NA | 3440 | 15147619 | 2020 |  |  |  |  |  |  |  |  |  |  |  |  |  |  |  |  |  |  |  |  |  |  |  |  |
| ebi-a-GCST90001693 | CD28+ CD45RA- CD8+ T cell Absolute Count | NA | 3408 | 15135292 | 2020 |  |  |  |  |  |  |  |  |  |  |  |  |  |  |  |  |  |  |  |  |  |  |  |  |
| ebi-a-GCST90001668 | CD28+ CD45RA- CD8dim T cell %CD8dim T cell | NA | 3440 | 15147619 | 2020 |  |  |  |  |  |  |  |  |  |  |  |  |  |  |  |  |  |  |  |  |  |  |  |  |
| ebi-a-GCST90001667 | CD28+ CD45RA- CD8dim T cell %T cell | NA | 3440 | 15147619 | 2020 |  |  |  |  |  |  |  |  |  |  |  |  |  |  |  |  |  |  |  |  |  |  |  |  |
| ebi-a-GCST90001669 | CD28+ CD45RA- CD8dim T cell Absolute Count | NA | 3408 | 15135292 | 2020 |  |  |  |  |  |  |  |  |  |  |  |  |  |  |  |  |  |  |  |  |  |  |  |  |
| ebi-a-GCST90001689 | CD28+ CD45RA+ CD8+ T cell %CD8+ T cell | NA | 3440 | 15147619 | 2020 |  |  |  |  |  |  |  |  |  |  |  |  |  |  |  |  |  |  |  |  |  |  |  |  |
| ebi-a-GCST90001688 | CD28+ CD45RA+ CD8+ T cell %T cell | NA | 3440 | 15147619 | 2020 |  |  |  |  |  |  |  |  |  |  |  |  |  |  |  |  |  |  |  |  |  |  |  |  |
| ebi-a-GCST90001690 | CD28+ CD45RA+ CD8+ T cell Absolute Count | NA | 3408 | 15135292 | 2020 |  |  |  |  |  |  |  |  |  |  |  |  |  |  |  |  |  |  |  |  |  |  |  |  |
| ebi-a-GCST90001665 | CD28+ CD45RA+ CD8dim T cell %CD8dim T cell | NA | 3440 | 15147619 | 2020 |  |  |  |  |  |  |  |  |  |  |  |  |  |  |  |  |  |  |  |  |  |  |  |  |
| ebi-a-GCST90001664 | CD28+ CD45RA+ CD8dim T cell %T cell | NA | 3440 | 15147619 | 2020 |  |  |  |  |  |  |  |  |  |  |  |  |  |  |  |  |  |  |  |  |  |  |  |  |
| ebi-a-GCST90001666 | CD28+ CD45RA+ CD8dim T cell Absolute Count | NA | 3408 | 15135292 | 2020 |  |  |  |  |  |  |  |  |  |  |  |  |  |  |  |  |  |  |  |  |  |  |  |  |
| ebi-a-GCST90001656 | CD28+ CD4-CD8- T cell %CD4-CD8- T cell | NA | 3440 | 15147619 | 2020 |  |  |  |  |  |  |  |  |  |  |  |  |  |  |  |  |  |  |  |  |  |  |  |  |
| ebi-a-GCST90001655 | CD28+ CD4-CD8- T cell %T cell | NA | 3440 | 15147619 | 2020 |  |  |  |  |  |  |  |  |  |  |  |  |  |  |  |  |  |  |  |  |  |  |  |  |
| ebi-a-GCST90001657 | CD28+ CD4-CD8- T cell Absolute Count | NA | 3408 | 15135292 | 2020 |  |  |  |  |  |  |  |  |  |  |  |  |  |  |  |  |  |  |  |  |  |  |  |  |
| ebi-a-GCST90001865 | CD3 on CD28- CD8+ T cell | NA | 2919 | 14849624 | 2020 |  |  |  |  |  |  |  |  |  |  |  |  |  |  |  |  |  |  |  |  |  |  |  |  |
| ebi-a-GCST90001861 | CD3 on CD28+ CD4+ T cell | NA | 2919 | 14849624 | 2020 |  |  |  |  |  |  |  |  |  |  |  |  |  |  |  |  |  |  |  |  |  |  |  |  |
| ebi-a-GCST90001863 | CD3 on CD28+ CD45RA- CD8+ T cell | NA | 2919 | 14849624 | 2020 |  |  |  |  |  |  |  |  |  |  |  |  |  |  |  |  |  |  |  |  |  |  |  |  |
| ebi-a-GCST90001864 | CD3 on CD28+ CD45RA+ CD8+ T cell | NA | 2919 | 14849624 | 2020 |  |  |  |  |  |  |  |  |  |  |  |  |  |  |  |  |  |  |  |  |  |  |  |  |
| ebi-a-GCST90001862 | CD3 on CD28+ CD4-CD8- T cell | NA | 2918 | 14849601 | 2020 |  |  |  |  |  |  |  |  |  |  |  |  |  |  |  |  |  |  |  |  |  |  |  |  |
| ebi-a-GCST90001854 | CD3 on CD39+ activated CD4 regulatory T cell | NA | 2,919 | 14,849,624 | 2020 |  |  |  |  |  |  |  |  |  |  |  |  |  |  |  |  |  |  |  |  |  |  |  |  |
| ebi-a-GCST90001860 | CD3 on CD39+ CD4+ T cell | NA | 2,919 | 14,849,624 | 2020 |  |  |  |  |  |  |  |  |  |  |  |  |  |  |  |  |  |  |  |  |  |  |  |  |
| ebi-a-GCST90001866 | CD3 on CD39+ CD8+ T cell | NA | 2,914 | 14,847,479 | 2020 |  |  |  |  |  |  |  |  |  |  |  |  |  |  |  |  |  |  |  |  |  |  |  |  |
| ebi-a-GCST90001852 | CD3 on CD39+ resting CD4 regulatory T cell | NA | 2,649 | 14,779,400 | 2020 |  |  |  |  |  |  |  |  |  |  |  |  |  |  |  |  |  |  |  |  |  |  |  |  |
| ebi-a-GCST90001856 | CD3 on CD39+ secreting CD4 regulatory T cell | NA | 2,919 | 14,849,624 | 2020 |  |  |  |  |  |  |  |  |  |  |  |  |  |  |  |  |  |  |  |  |  |  |  |  |
| ebi-a-GCST90001845 | CD3 on CD45RA- CD4+ T cell | NA | 2,911 | 14,842,747 | 2020 |  |  |  |  |  |  |  |  |  |  |  |  |  |  |  |  |  |  |  |  |  |  |  |  |
| ebi-a-GCST90001858 | CD3 on CD45RA+ CD4+ T cell | NA | 2,919 | 14,849,624 | 2020 |  |  |  |  |  |  |  |  |  |  |  |  |  |  |  |  |  |  |  |  |  |  |  |  |
| ebi-a-GCST90001809 | CD38 on CD20- B cell | NA | 3,630 | 15,042,909 | 2015 |  |  |  |  |  |  |  |  |  |  |  |  |  |  |  |  |  |  |  |  |  |  |  |  |
| ebi-a-GCST90001815 | CD38 on IgD- CD38dim B cell | NA | 3,657 | 15,048,951 | 2020 |  |  |  |  |  |  |  |  |  |  |  |  |  |  |  |  |  |  |  |  |  |  |  |  |
| ebi-a-GCST90001811 | CD38 on IgD+ CD24- B cell | NA | 3,656 | 15,048,942 | 2020 |  |  |  |  |  |  |  |  |  |  |  |  |  |  |  |  |  |  |  |  |  |  |  |  |
| ebi-a-GCST90002030 | CD39 on CD39+ activated CD4 regulatory T cell | NA | 2,920 | 14,849,646 | 2020 |  |  |  |  |  |  |  |  |  |  |  |  |  |  |  |  |  |  |  |  |  |  |  |  |
| ebi-a-GCST90002032 | CD39 on CD39+ CD4+ T cell | NA | 2,920 | 14,849,646 | 2020 |  |  |  |  |  |  |  |  |  |  |  |  |  |  |  |  |  |  |  |  |  |  |  |  |
| ebi-a-GCST90002029 | CD39 on CD39+ CD8+ T cell | NA | 2,915 | 14,847,499 | 2020 |  |  |  |  |  |  |  |  |  |  |  |  |  |  |  |  |  |  |  |  |  |  |  |  |
| ebi-a-GCST90002031 | CD39 on CD39+ secreting CD4 regulatory T cell | NA | 2,920 | 14,849,646 | 2020 |  |  |  |  |  |  |  |  |  |  |  |  |  |  |  |  |  |  |  |  |  |  |  |  |
| met-b-156 | CD39 on CD4 T | NA | 497 | 1,623,131 | 2020 |  |  |  |  |  |  |  |  |  |  |  |  |  |  |  |  |  |  |  |  |  |  |  |  |
| ebi-a-GCST90001490 | CD39+ activated CD4 regulatory T cell %activated CD4 regulatory T cell | NA | 3437 | 5914718 | 2020 |  |  |  |  |  |  |  |  |  |  |  |  |  |  |  |  |  |  |  |  |  |  |  |  |
| ebi-a-GCST90001491 | CD39+ activated CD4 regulatory T cell %CD4 regulatory T cell | NA | 3,437 | 15,144,196 | 2020 |  |  |  |  |  |  |  |  |  |  |  |  |  |  |  |  |  |  |  |  |  |  |  |  |
| ebi-a-GCST90001489 | CD39+ activated CD4 regulatory T cell Absolute Count | NA | 3,405 | 15,131,843 | 2020 |  |  |  |  |  |  |  |  |  |  |  |  |  |  |  |  |  |  |  |  |  |  |  |  |
| ebi-a-GCST90001659 | CD39+ CD4+ T cell %CD4+ T cell | NA | 3440 | 15147619 | 2020 |  |  |  |  |  |  |  |  |  |  |  |  |  |  |  |  |  |  |  |  |  |  |  |  |
| ebi-a-GCST90001658 | CD39+ CD4+ T cell %T cell | NA | 3440 | 15147619 | 2020 |  |  |  |  |  |  |  |  |  |  |  |  |  |  |  |  |  |  |  |  |  |  |  |  |
| ebi-a-GCST90001660 | CD39+ CD4+ T cell Absolute Count | NA | 3,408 | 15,135,292 | 2020 |  |  |  |  |  |  |  |  |  |  |  |  |  |  |  |  |  |  |  |  |  |  |  |  |
| ebi-a-GCST90001671 | CD39+ CD8+ T cell %CD8+ T cell | NA | 3440 | 15147619 | 2020 |  |  |  |  |  |  |  |  |  |  |  |  |  |  |  |  |  |  |  |  |  |  |  |  |
| ebi-a-GCST90001670 | CD39+ CD8+ T cell %T cell | NA | 3440 | 15147619 | 2020 |  |  |  |  |  |  |  |  |  |  |  |  |  |  |  |  |  |  |  |  |  |  |  |  |
| ebi-a-GCST90001672 | CD39+ CD8+ T cell Absolute Count | NA | 3,408 | 15,135,292 | 2020 |  |  |  |  |  |  |  |  |  |  |  |  |  |  |  |  |  |  |  |  |  |  |  |  |
| ebi-a-GCST90001485 | CD39+ resting CD4 regulatory T cell %CD4 regulatory T cell | NA | 3,437 | 15,144,196 | 2020 |  |  |  |  |  |  |  |  |  |  |  |  |  |  |  |  |  |  |  |  |  |  |  |  |
| ebi-a-GCST90001484 | CD39+ resting CD4 regulatory T cell %resting CD4 regulatory T cell | NA | 3,437 | 15,144,196 | 2020 |  |  |  |  |  |  |  |  |  |  |  |  |  |  |  |  |  |  |  |  |  |  |  |  |
| ebi-a-GCST90001483 | CD39+ resting CD4 regulatory T cell Absolute Count | NA | 3,405 | 15,131,843 | 2020 |  |  |  |  |  |  |  |  |  |  |  |  |  |  |  |  |  |  |  |  |  |  |  |  |
| ebi-a-GCST90001497 | CD39+ secreting CD4 regulatory T cell %CD4 regulatory T cell | NA | 3,437 | 15,144,196 | 2020 |  |  |  |  |  |  |  |  |  |  |  |  |  |  |  |  |  |  |  |  |  |  |  |  |
| ebi-a-GCST90001496 | CD39+ secreting CD4 regulatory T cell %secreting CD4 regulatory T cell | NA | 3,437 | 15,144,196 | 2020 |  |  |  |  |  |  |  |  |  |  |  |  |  |  |  |  |  |  |  |  |  |  |  |  |
| ebi-a-GCST90001495 | CD39+ secreting CD4 regulatory T cell Absolute Count | NA | 3,405 | 15,131,843 | 2020 |  |  |  |  |  |  |  |  |  |  |  |  |  |  |  |  |  |  |  |  |  |  |  |  |
| ebi-a-GCST90002062 | CD4 on CD28+ CD4+ T cell | NA | 2920 | 14849646 | 2020 |  |  |  |  |  |  |  |  |  |  |  |  |  |  |  |  |  |  |  |  |  |  |  |  |
| ebi-a-GCST90002067 | CD4 on CD39+ activated CD4 regulatory T cell | NA | 2,920 | 14,849,646 | 2020 |  |  |  |  |  |  |  |  |  |  |  |  |  |  |  |  |  |  |  |  |  |  |  |  |
| ebi-a-GCST90002061 | CD4 on CD39+ CD4+ T cell | NA | 2,920 | 14,849,646 | 2020 |  |  |  |  |  |  |  |  |  |  |  |  |  |  |  |  |  |  |  |  |  |  |  |  |
| ebi-a-GCST90002065 | CD4 on CD39+ resting CD4 regulatory T cell | NA | 2,650 | 14,779,430 | 2020 |  |  |  |  |  |  |  |  |  |  |  |  |  |  |  |  |  |  |  |  |  |  |  |  |
| ebi-a-GCST90002069 | CD4 on CD39+ secreting CD4 regulatory T cell | NA | 2,920 | 14,849,646 | 2020 |  |  |  |  |  |  |  |  |  |  |  |  |  |  |  |  |  |  |  |  |  |  |  |  |
| ebi-a-GCST90002027 | CD4 on CD45RA+ CD4+ T cell | NA | 2,911 | 14,842,747 | 2020 |  |  |  |  |  |  |  |  |  |  |  |  |  |  |  |  |  |  |  |  |  |  |  |  |
| ebi-a-GCST90001910 | CD45 on B cell | NA | 3,112 | 14,903,706 | 2020 |  |  |  |  |  |  |  |  |  |  |  |  |  |  |  |  |  |  |  |  |  |  |  |  |
| ebi-a-GCST90001916 | CD45 on CD4+ T cell | NA | 3,113 | 14,903,739 | 2020 |  |  |  |  |  |  |  |  |  |  |  |  |  |  |  |  |  |  |  |  |  |  |  |  |
| ebi-a-GCST90001917 | CD45 on CD8+ T cell | NA | 3,113 | 14,903,739 | 2020 |  |  |  |  |  |  |  |  |  |  |  |  |  |  |  |  |  |  |  |  |  |  |  |  |
| ebi-a-GCST90001920 | CD45 on HLA DR+ CD4+ T cell | NA | 3,060 | 14,891,166 | 2020 |  |  |  |  |  |  |  |  |  |  |  |  |  |  |  |  |  |  |  |  |  |  |  |  |
| ebi-a-GCST90001921 | CD45 on HLA DR+ CD8+ T cell | NA | 3,060 | 14,891,166 | 2020 |  |  |  |  |  |  |  |  |  |  |  |  |  |  |  |  |  |  |  |  |  |  |  |  |
| ebi-a-GCST90001912 | CD45 on HLA DR+ Natural Killer | NA | 2,971 | 14,858,184 | 2020 |  |  |  |  |  |  |  |  |  |  |  |  |  |  |  |  |  |  |  |  |  |  |  |  |
| ebi-a-GCST90001918 | CD45 on HLA DR+ T cell | NA | 3,060 | 14,891,166 | 2020 |  |  |  |  |  |  |  |  |  |  |  |  |  |  |  |  |  |  |  |  |  |  |  |  |
| ebi-a-GCST90001911 | CD45 on Natural Killer | NA | 3,113 | 14,903,739 | 2020 |  |  |  |  |  |  |  |  |  |  |  |  |  |  |  |  |  |  |  |  |  |  |  |  |
| ebi-a-GCST90001919 | CD45 on Natural Killer T | NA | 3,113 | 14,903,739 | 2020 |  |  |  |  |  |  |  |  |  |  |  |  |  |  |  |  |  |  |  |  |  |  |  |  |
| ebi-a-GCST90001915 | CD45 on T cell | NA | 3,113 | 14,903,739 | 2020 |  |  |  |  |  |  |  |  |  |  |  |  |  |  |  |  |  |  |  |  |  |  |  |  |
| ebi-a-GCST90001696 | CD45RA- CD28- CD8+ T cell %CD8+ T cell | NA | 3425 | 15138016 | 2020 |  |  |  |  |  |  |  |  |  |  |  |  |  |  |  |  |  |  |  |  |  |  |  |  |
| ebi-a-GCST90001697 | CD45RA- CD28- CD8+ T cell %T cell | NA | 3425 | 15138016 | 2020 |  |  |  |  |  |  |  |  |  |  |  |  |  |  |  |  |  |  |  |  |  |  |  |  |
| ebi-a-GCST90001695 | CD45RA- CD28- CD8+ T cell Absolute Count | NA | 3408 | 15135597 | 2020 |  |  |  |  |  |  |  |  |  |  |  |  |  |  |  |  |  |  |  |  |  |  |  |  |
| ebi-a-GCST90001535 | CD45RA- CD4+ T cell %CD4+ T cell | NA | 3,427 | 15,141,588 | 2020 |  |  |  |  |  |  |  |  |  |  |  |  |  |  |  |  |  |  |  |  |  |  |  |  |
| ebi-a-GCST90001536 | CD45RA- CD4+ T cell %T cell | NA | 3,427 | 15,141,588 | 2020 |  |  |  |  |  |  |  |  |  |  |  |  |  |  |  |  |  |  |  |  |  |  |  |  |
| ebi-a-GCST90001534 | CD45RA- CD4+ T cell Absolute Count | NA | 3,395 | 15,129,224 | 2020 |  |  |  |  |  |  |  |  |  |  |  |  |  |  |  |  |  |  |  |  |  |  |  |  |
| ebi-a-GCST90002103 | CD45RA on CD39+ resting CD4 regulatory T cell | NA | 2,650 | 4,490,814 | 2020 |  |  |  |  |  |  |  |  |  |  |  |  |  |  |  |  |  |  |  |  |  |  |  |  |
| ebi-a-GCST90002098 | CD45RA on naive CD4+ T cell | NA | 2,910 | 14,842,727 | 2020 |  |  |  |  |  |  |  |  |  |  |  |  |  |  |  |  |  |  |  |  |  |  |  |  |
| ebi-a-GCST90002100 | CD45RA on naive CD8+ T cell | NA | 2,910 | 14,842,727 | 2020 |  |  |  |  |  |  |  |  |  |  |  |  |  |  |  |  |  |  |  |  |  |  |  |  |
| ebi-a-GCST90002102 | CD45RA on resting CD4 regulatory T cell | NA | 2,919 | 14,849,628 | 2020 |  |  |  |  |  |  |  |  |  |  |  |  |  |  |  |  |  |  |  |  |  |  |  |  |
| ebi-a-GCST90002101 | CD45RA on Terminally Differentiated CD8+ T cell | NA | 2,911 | 14,842,950 | 2020 |  |  |  |  |  |  |  |  |  |  |  |  |  |  |  |  |  |  |  |  |  |  |  |  |
| ebi-a-GCST90001699 | CD45RA+ CD28- CD8+ T cell %CD8+ T cell | NA | 3425 | 15138016 | 2020 |  |  |  |  |  |  |  |  |  |  |  |  |  |  |  |  |  |  |  |  |  |  |  |  |
| ebi-a-GCST90001700 | CD45RA+ CD28- CD8+ T cell %T cell | NA | 3425 | 15138016 | 2020 |  |  |  |  |  |  |  |  |  |  |  |  |  |  |  |  |  |  |  |  |  |  |  |  |
| ebi-a-GCST90001698 | CD45RA+ CD28- CD8+ T cell Absolute Count | NA | 3408 | 15135597 | 2020 |  |  |  |  |  |  |  |  |  |  |  |  |  |  |  |  |  |  |  |  |  |  |  |  |
| ebi-a-GCST90001561 | CD45RA+ CD8+ T cell %CD8+ T cell | NA | 3,427 | 15,141,588 | 2020 |  |  |  |  |  |  |  |  |  |  |  |  |  |  |  |  |  |  |  |  |  |  |  |  |
| ebi-a-GCST90001562 | CD45RA+ CD8+ T cell %T cell | NA | 3,427 | 15,141,588 | 2020 |  |  |  |  |  |  |  |  |  |  |  |  |  |  |  |  |  |  |  |  |  |  |  |  |
| ebi-a-GCST90001560 | CD45RA+ CD8+ T cell Absolute Count | NA | 3,395 | 15,129,224 | 2020 |  |  |  |  |  |  |  |  |  |  |  |  |  |  |  |  |  |  |  |  |  |  |  |  |
| ebi-a-GCST90002120 | CD8 on CD28- CD8+ T cell | NA | 2920 | 14849646 | 2020 |  |  |  |  |  |  |  |  |  |  |  |  |  |  |  |  |  |  |  |  |  |  |  |  |
| ebi-a-GCST90002118 | CD8 on CD28+ CD45RA- CD8+ T cell | NA | 2920 | 14849646 | 2020 |  |  |  |  |  |  |  |  |  |  |  |  |  |  |  |  |  |  |  |  |  |  |  |  |
| ebi-a-GCST90002119 | CD8 on CD28+ CD45RA+ CD8+ T cell | NA | 2920 | 14849646 | 2018 |  |  |  |  |  |  |  |  |  |  |  |  |  |  |  |  |  |  |  |  |  |  |  |  |
| ebi-a-GCST90002121 | CD8 on CD39+ CD8+ T cell | NA | 2,915 | 14,847,499 | 2020 |  |  |  |  |  |  |  |  |  |  |  |  |  |  |  |  |  |  |  |  |  |  |  |  |
| ebi-a-GCST90001968 | FSC-A on B cell | NA | 3,112 | 14,903,706 | 2020 |  |  |  |  |  |  |  |  |  |  |  |  |  |  |  |  |  |  |  |  |  |  |  |  |
| ebi-a-GCST90001967 | FSC-A on CD14+ monocyte | NA | 3,112 | 14,903,652 | 2020 |  |  |  |  |  |  |  |  |  |  |  |  |  |  |  |  |  |  |  |  |  |  |  |  |
| ebi-a-GCST90001973 | FSC-A on CD4+ T cell | NA | 3,113 | 14,903,739 | 2020 |  |  |  |  |  |  |  |  |  |  |  |  |  |  |  |  |  |  |  |  |  |  |  |  |
| ebi-a-GCST90001974 | FSC-A on CD8+ T cell | NA | 3,113 | 14,903,739 | 2020 |  |  |  |  |  |  |  |  |  |  |  |  |  |  |  |  |  |  |  |  |  |  |  |  |
| ebi-a-GCST90001966 | FSC-A on granulocyte | NA | 2,850 | 14,821,110 | 2020 |  |  |  |  |  |  |  |  |  |  |  |  |  |  |  |  |  |  |  |  |  |  |  |  |
| ebi-a-GCST90001977 | FSC-A on HLA DR+ CD4+ T cell | NA | 3,060 | 14,891,166 | 2020 |  |  |  |  |  |  |  |  |  |  |  |  |  |  |  |  |  |  |  |  |  |  |  |  |
| ebi-a-GCST90001978 | FSC-A on HLA DR+ CD8+ T cell | NA | 3,060 | 14,891,166 | 2020 |  |  |  |  |  |  |  |  |  |  |  |  |  |  |  |  |  |  |  |  |  |  |  |  |
| ebi-a-GCST90001970 | FSC-A on HLA DR+ Natural Killer | NA | 2,971 | 14,858,184 | 2020 |  |  |  |  |  |  |  |  |  |  |  |  |  |  |  |  |  |  |  |  |  |  |  |  |
| ebi-a-GCST90001975 | FSC-A on HLA DR+ T cell | NA | 3,060 | 14,891,166 | 2020 |  |  |  |  |  |  |  |  |  |  |  |  |  |  |  |  |  |  |  |  |  |  |  |  |
| ebi-a-GCST90001965 | FSC-A on monocyte | NA | 2,850 | 14,821,110 | 2020 |  |  |  |  |  |  |  |  |  |  |  |  |  |  |  |  |  |  |  |  |  |  |  |  |
| ebi-a-GCST90001963 | FSC-A on myeloid Dendritic Cell | NA | 2,872 | 14,829,776 | 2020 |  |  |  |  |  |  |  |  |  |  |  |  |  |  |  |  |  |  |  |  |  |  |  |  |
| ebi-a-GCST90001969 | FSC-A on Natural Killer | NA | 3,113 | 14,903,739 | 2020 |  |  |  |  |  |  |  |  |  |  |  |  |  |  |  |  |  |  |  |  |  |  |  |  |
| ebi-a-GCST90001976 | FSC-A on Natural Killer T | NA | 3,113 | 14,903,739 | 2020 |  |  |  |  |  |  |  |  |  |  |  |  |  |  |  |  |  |  |  |  |  |  |  |  |
| ebi-a-GCST90001964 | FSC-A on plasmacytoid Dendritic Cell | NA | 2,872 | 14,829,782 | 2020 |  |  |  |  |  |  |  |  |  |  |  |  |  |  |  |  |  |  |  |  |  |  |  |  |
| ebi-a-GCST90001972 | FSC-A on T cell | NA | 3,113 | 14,903,739 | 2020 |  |  |  |  |  |  |  |  |  |  |  |  |  |  |  |  |  |  |  |  |  |  |  |  |
| ebi-a-GCST90001880 | HVEM on CD45RA- CD4+ T cell | NA | 1,247 | 13,730,810 | 2020 |  |  |  |  |  |  |  |  |  |  |  |  |  |  |  |  |  |  |  |  |  |  |  |  |
| ebi-a-GCST90001413 | IgD- CD24- B cell %B cell | NA | 3,657 | 15,048,951 | 2020 |  |  |  |  |  |  |  |  |  |  |  |  |  |  |  |  |  |  |  |  |  |  |  |  |
| ebi-a-GCST90001440 | IgD- CD24- B cell %lymphocyte | NA | 3,656 | 15,048,937 | 2020 |  |  |  |  |  |  |  |  |  |  |  |  |  |  |  |  |  |  |  |  |  |  |  |  |
| ebi-a-GCST90001414 | IgD- CD24- B cell Absolute Count | NA | 3,656 | 15,048,937 | 2020 |  |  |  |  |  |  |  |  |  |  |  |  |  |  |  |  |  |  |  |  |  |  |  |  |
| ebi-a-GCST90001399 | IgD- CD27- B cell %B cell | NA | 3,657 | 15,048,951 | 2020 |  |  |  |  |  |  |  |  |  |  |  |  |  |  |  |  |  |  |  |  |  |  |  |  |
| ebi-a-GCST90001433 | IgD- CD27- B cell %lymphocyte | NA | 3,656 | 15,048,937 | 2020 |  |  |  |  |  |  |  |  |  |  |  |  |  |  |  |  |  |  |  |  |  |  |  |  |
| ebi-a-GCST90001401 | IgD- CD27- B cell Absolute Count | NA | 3,656 | 15,048,937 | 2020 |  |  |  |  |  |  |  |  |  |  |  |  |  |  |  |  |  |  |  |  |  |  |  |  |
| ebi-a-GCST90001821 | IgD on IgD+ CD24- B cell | NA | 3,656 | 15,048,923 | 2020 |  |  |  |  |  |  |  |  |  |  |  |  |  |  |  |  |  |  |  |  |  |  |  |  |
| ebi-a-GCST90001820 | IgD on IgD+ CD24+ B cell | NA | 3,656 | 15,048,891 | 2020 |  |  |  |  |  |  |  |  |  |  |  |  |  |  |  |  |  |  |  |  |  |  |  |  |
| ebi-a-GCST90001415 | IgD+ CD24- B cell %B cell | NA | 3,657 | 15,048,951 | 2020 |  |  |  |  |  |  |  |  |  |  |  |  |  |  |  |  |  |  |  |  |  |  |  |  |
| ebi-a-GCST90001441 | IgD+ CD24- B cell %lymphocyte | NA | 3,656 | 15,048,937 | 2020 |  |  |  |  |  |  |  |  |  |  |  |  |  |  |  |  |  |  |  |  |  |  |  |  |
| ebi-a-GCST90001416 | IgD+ CD24- B cell Absolute Count | NA | 3,656 | 15,048,937 | 2020 |  |  |  |  |  |  |  |  |  |  |  |  |  |  |  |  |  |  |  |  |  |  |  |  |
| ebi-a-GCST90001411 | IgD+ CD24+ B cell %B cell | NA | 3,657 | 15,048,951 | 2020 |  |  |  |  |  |  |  |  |  |  |  |  |  |  |  |  |  |  |  |  |  |  |  |  |
| ebi-a-GCST90001439 | IgD+ CD24+ B cell %lymphocyte | NA | 3,656 | 15,048,937 | 2020 |  |  |  |  |  |  |  |  |  |  |  |  |  |  |  |  |  |  |  |  |  |  |  |  |
| ebi-a-GCST90001412 | IgD+ CD24+ B cell Absolute Count | NA | 3,656 | 15,048,937 | 2020 |  |  |  |  |  |  |  |  |  |  |  |  |  |  |  |  |  |  |  |  |  |  |  |  |
| ebi-a-GCST90002075 | SSC-A on B cell | NA | 3,112 | 14,903,706 | 2020 |  |  |  |  |  |  |  |  |  |  |  |  |  |  |  |  |  |  |  |  |  |  |  |  |
| ebi-a-GCST90002074 | SSC-A on CD14+ monocyte | NA | 3,112 | 14,903,652 | 2020 |  |  |  |  |  |  |  |  |  |  |  |  |  |  |  |  |  |  |  |  |  |  |  |  |
| ebi-a-GCST90002081 | SSC-A on CD4+ T cell | NA | 3,113 | 14,903,739 | 2020 |  |  |  |  |  |  |  |  |  |  |  |  |  |  |  |  |  |  |  |  |  |  |  |  |
| ebi-a-GCST90002082 | SSC-A on CD8+ T cell | NA | 3,113 | 14,903,739 | 2020 |  |  |  |  |  |  |  |  |  |  |  |  |  |  |  |  |  |  |  |  |  |  |  |  |
| ebi-a-GCST90002078 | SSC-A on granulocyte | NA | 3,112 | 14,903,727 | 2020 |  |  |  |  |  |  |  |  |  |  |  |  |  |  |  |  |  |  |  |  |  |  |  |  |
| ebi-a-GCST90002085 | SSC-A on HLA DR+ CD4+ T cell | NA | 3,060 | 14,891,166 | 2020 |  |  |  |  |  |  |  |  |  |  |  |  |  |  |  |  |  |  |  |  |  |  |  |  |
| ebi-a-GCST90002086 | SSC-A on HLA DR+ CD8+ T cell | NA | 3,060 | 14,891,166 | 2020 |  |  |  |  |  |  |  |  |  |  |  |  |  |  |  |  |  |  |  |  |  |  |  |  |
| ebi-a-GCST90002077 | SSC-A on HLA DR+ Natural Killer | NA | 2,971 | 14,858,184 | 2020 |  |  |  |  |  |  |  |  |  |  |  |  |  |  |  |  |  |  |  |  |  |  |  |  |
| ebi-a-GCST90002083 | SSC-A on HLA DR+ T cell | NA | 3,060 | 14,891,166 | 2020 |  |  |  |  |  |  |  |  |  |  |  |  |  |  |  |  |  |  |  |  |  |  |  |  |
| ebi-a-GCST90002073 | SSC-A on monocyte | NA | 2,850 | 14,821,110 | 2020 |  |  |  |  |  |  |  |  |  |  |  |  |  |  |  |  |  |  |  |  |  |  |  |  |
| ebi-a-GCST90002071 | SSC-A on myeloid Dendritic Cell | NA | 2,872 | 14,829,776 | 2020 |  |  |  |  |  |  |  |  |  |  |  |  |  |  |  |  |  |  |  |  |  |  |  |  |
| ebi-a-GCST90002076 | SSC-A on Natural Killer | NA | 3,113 | 14,903,739 | 2020 |  |  |  |  |  |  |  |  |  |  |  |  |  |  |  |  |  |  |  |  |  |  |  |  |
| ebi-a-GCST90002084 | SSC-A on Natural Killer T | NA | 3,113 | 14,903,739 | 2020 |  |  |  |  |  |  |  |  |  |  |  |  |  |  |  |  |  |  |  |  |  |  |  |  |
| ebi-a-GCST90001885 | CD16-CD56 on HLA DR+ Natural Killer | NA | 2,971 | 14,858,184 | 2020 |  |  |  |  |  |  |  |  |  |  |  |  |  |  |  |  |  |  |  |  |  |  |  |  |
| ebi-a-GCST90002072 | SSC-A on plasmacytoid Dendritic Cell | NA | 2,872 | 14,829,782 | 2020 |  |  |  |  |  |  |  |  |  |  |  |  |  |  |  |  |  |  |  |  |  |  |  |  |
| ebi-a-GCST90002080 | SSC-A on T cell | NA | 3,113 | 14,903,739 | 2020 |  |  |  |  |  |  |  |  |  |  |  |  |  |  |  |  |  |  |  |  |  |  |  |  |
| ebi-a-GCST90000450 | CD244 levels | NA | 982 | 7,883,872 | 2020 |  |  |  |  |  |  |  |  |  |  |  |  |  |  |  |  |  |  |  |  |  |  |  |  |
| ebi-a-GCST90000451 | CD40 levels | NA | 982 | 7,883,849 | 2020 |  |  |  |  |  |  |  |  |  |  |  |  |  |  |  |  |  |  |  |  |  |  |  |  |
| ebi-a-GCST90010113 | CD40 ligand levels | NA | 1,301 | 18,166,693 | 2020 |  |  |  |  |  |  |  |  |  |  |  |  |  |  |  |  |  |  |  |  |  |  |  |  |
| ebi-a-GCST90011997 | CD40 ligand levels | NA | 21,758 | 13,106,328 | 2020 |  |  |  |  |  |  |  |  |  |  |  |  |  |  |  |  |  |  |  |  |  |  |  |  |
| ebi-a-GCST90085714 | CD40 plasma levels | NA | 400 | 5,188,525 | 2020 |  |  |  |  |  |  |  |  |  |  |  |  |  |  |  |  |  |  |  |  |  |  |  |  |
| prot-a-447 | CD40 ligand | NA | 3,301 | 10,534,735 | 2018 |  |  |  |  |  |  |  |  |  |  |  |  |  |  |  |  |  |  |  |  |  |  |  |  |
| prot-b-44 | CD40 ligand | NA | 3,394 | 5,270,646 | 2018 |  |  |  |  |  |  |  |  |  |  |  |  |  |  |  |  |  |  |  |  |  |  |  |  |
| **GWAS data for seven adverse fetal outcomes** | | | | | |  |  |  |  |  |  |  |  |  |  |  |  |  |  |  |  |  |  |  |  |  |  |  |  |
| **ID** | **Trait** | **Consortium** | **Sample size** | **Number of SNPs** | **Year** |  |  |  |  |  |  |  |  |  |  |  |  |  |  |  |  |  |  |  |  |  |  |  |  |
| ukb-b-13378 | Birth weight | MRC-IEU | 261932 | 9851867 | 2018 |  |  |  |  |  |  |  |  |  |  |  |  |  |  |  |  |  |  |  |  |  |  |  |  |
| ukb-a-198 | Birth weight | Neale Lab | 193063 | 10894596 | 2017 |  |  |  |  |  |  |  |  |  |  |  |  |  |  |  |  |  |  |  |  |  |  |  |  |
| ieu-a-1083 | Birth weight | NA | 143677 | 16245524 | 2016 |  |  |  |  |  |  |  |  |  |  |  |  |  |  |  |  |  |  |  |  |  |  |  |  |
| ukb-b-3357 | Birth weight of first child | MRC-IEU | 200272 | 9851867 | 2018 |  |  |  |  |  |  |  |  |  |  |  |  |  |  |  |  |  |  |  |  |  |  |  |  |
| finn-b-P16_NEONTAL_JAUND_OTH_UNSP_CAUSES | Neonatal jaundice from other and unspecified causes | NA | — | 16380466 | 2021 |  |  |  |  |  |  |  |  |  |  |  |  |  |  |  |  |  |  |  |  |  |  |  |  |
| finn-b-P16_DIGES_SYSTEM_DISORD_FETUS_NEWBO | Digestive system disorders of fetus and newborn | NA | — | 16380466 | 2021 |  |  |  |  |  |  |  |  |  |  |  |  |  |  |  |  |  |  |  |  |  |  |  |  |
| finn-b-P16_HAEMORRHAGIC_HAEMATOL_DISORD_FETUS_NEWBO | Haemorrhagic and haematological disorders of fetus and newborn | NA | — | 16380466 | 2021 |  |  |  |  |  |  |  |  |  |  |  |  |  |  |  |  |  |  |  |  |  |  |  |  |
| finn-b-P16_RESPI_DISTR_NEWBO | Respiratory distress of newborn | NA | — | 16380461 | 2021 |  |  |  |  |  |  |  |  |  |  |  |  |  |  |  |  |  |  |  |  |  |  |  |  |
| finn-b-P16_TRANSITO_DISORD_CARBOH_METABOLISM_SPECIFIC_FETUS_NEWBO | Transitory disorders of carbohydrate metabolism specific to fetus and newborn | NA | — | 16380466 | 2021 |  |  |  |  |  |  |  |  |  |  |  |  |  |  |  |  |  |  |  |  |  |  |  |  |
| finn-b-P16_TRANSITO_ENDOCR_METABOLIC_DISORD_SPECIFIC_FETUS_NEWBO | Transitory endocrine and metabolic disorders specific to fetus and newborn | NA | — | 16380466 | 2021 |  |  |  |  |  |  |  |  |  |  |  |  |  |  |  |  |  |  |  |  |  |  |  |  |
| **First Author** | **PubMed ID** | **Study Accession** | **Publication Date** | **Journal** | **Reported Trait** | **technology** | **Trait(s)** | **Trait URI** | **Data Access** | **Sample size** |  |  |  |  |  |  |  |  |  |  |  |  |  |  |  |  |  |  |  |
| Juodakis J | 37195282 | GCST90268419 | 2023/5/17 | Hum Mol Genet | Gestational age at birth (maternal effect) | Genome-wide genotyping array | parental genotype effect measurement, gestational age | <https://ftp.ebi.ac.uk/pub/databases/gwas/summary_statistics/GCST90268001-GCST90269000/GCST90268419/> | FTP Download | 22247 |  |  |  |  |  |  |  |  |  |  |  |  |  |  |  |  |  |  |  |
| Juodakis J | 37195282 | GCST90268421 | 2023/5/17 | Hum Mol Genet | Gestational age at birth (child effect) | Genome-wide genotyping array | gestational age, fetal genotype effect measurement | <https://ftp.ebi.ac.uk/pub/databases/gwas/summary_statistics/GCST90268001-GCST90269000/GCST90268421/> | FTP Download | 21262 |  |  |  |  |  |  |  |  |  |  |  |  |  |  |  |  |  |  |  |
| Supplementary Table 2: Detailed information about instrumental variables. | | | | | | | | | | | | | | |  |  |  |  |  |  |  |  |  |  |  |  |  |  |  |
| **beta.exposure** | **chr.exposure** | **data_source.exposure** | **eaf.exposure** | **effect_allele.exposure** | **exposure** | **id.exposure** | **mr_keep.exposure** | **other_allele.exposure** | **pos.exposure** | **pval.exposure** | **pval_origin.exposure** | **samplesize.exposure** | **se.exposure** | **SNP** |  |  |  |  |  |  |  |  |  |  |  |  |  |  |  |
| -1827 | 4 | igd | 0.9999 | G | \|\| id:ebi-a-GCST90001698 | ebi-a-GCST90001698 | TRUE | A | 167710347 | 6.06E-42 | reported | 3408 | 132.8 | rs2054143 |  |  |  |  |  |  |  |  |  |  |  |  |  |  |  |
| -35.15 | 4 | igd | 0.9999 | G | \|\| id:ebi-a-GCST90001700 | ebi-a-GCST90001700 | TRUE | A | 167710347 | 1.44E-09 | reported | 3425 | 5.793 | rs2054143 |  |  |  |  |  |  |  |  |  |  |  |  |  |  |  |
| -294.6 | 20 | igd | 0.9994 | T | \|\| id:ebi-a-GCST90001695 | ebi-a-GCST90001695 | TRUE | A | 46616280 | 6.96E-09 | reported | 3408 | 50.73 | rs56067013 |  |  |  |  |  |  |  |  |  |  |  |  |  |  |  |
| -659.9 | 22 | igd | 0.9994 | T | \|\| id:ebi-a-GCST90001698 | ebi-a-GCST90001698 | TRUE | C | 39354391 | 7.60E-15 | reported | 3408 | 84.5 | rs4821845 |  |  |  |  |  |  |  |  |  |  |  |  |  |  |  |
| -397.5 | 16 | igd | 0.9991 | T | \|\| id:ebi-a-GCST90001695 | ebi-a-GCST90001695 | TRUE | C | 81462243 | 2.40E-20 | reported | 3408 | 42.74 | rs2615702 |  |  |  |  |  |  |  |  |  |  |  |  |  |  |  |
| -11.3 | 16 | igd | 0.9991 | T | \|\| id:ebi-a-GCST90001697 | ebi-a-GCST90001697 | TRUE | C | 81462243 | 2.21E-08 | reported | 3425 | 2.015 | rs2615702 |  |  |  |  |  |  |  |  |  |  |  |  |  |  |  |
| -548.4 | 21 | igd | 0.999 | C | \|\| id:ebi-a-GCST90001698 | ebi-a-GCST90001698 | TRUE | T | 38156170 | 2.95E-21 | reported | 3408 | 57.56 | rs6517382 |  |  |  |  |  |  |  |  |  |  |  |  |  |  |  |
| -388.2 | 6 | igd | 0.9988 | G | \|\| id:ebi-a-GCST90001698 | ebi-a-GCST90001698 | TRUE | A | 13374132 | 1.11E-14 | reported | 3408 | 50.03 | rs2496150 |  |  |  |  |  |  |  |  |  |  |  |  |  |  |  |
| -455.6 | 20 | igd | 0.9988 | C | \|\| id:ebi-a-GCST90001698 | ebi-a-GCST90001698 | TRUE | G | 41876041 | 3.78E-19 | reported | 3408 | 50.64 | rs1569444 |  |  |  |  |  |  |  |  |  |  |  |  |  |  |  |
| -287.7 | 3 | igd | 0.9987 | G | \|\| id:ebi-a-GCST90001698 | ebi-a-GCST90001698 | TRUE | A | 105611213 | 4.25E-09 | reported | 3408 | 48.86 | rs7613821 |  |  |  |  |  |  |  |  |  |  |  |  |  |  |  |
| -233.6 | 18 | igd | 0.9974 | A | \|\| id:ebi-a-GCST90001698 | ebi-a-GCST90001698 | TRUE | G | 70504468 | 7.08E-09 | reported | 3408 | 40.25 | rs11661882 |  |  |  |  |  |  |  |  |  |  |  |  |  |  |  |
| -201.1 | 9 | igd | 0.9972 | T | \|\| id:ebi-a-GCST90001698 | ebi-a-GCST90001698 | TRUE | C | 135486391 | 8.75E-09 | reported | 3408 | 34.87 | rs421380 |  |  |  |  |  |  |  |  |  |  |  |  |  |  |  |
| -1.139 | 2 | igd | 0.9971 | T | \|\| id:ebi-a-GCST90001561 | ebi-a-GCST90001561 | TRUE | C | 103672986 | 2.52E-08 | reported | 3427 | 0.2039 | rs2463509 |  |  |  |  |  |  |  |  |  |  |  |  |  |  |  |
| -160 | 9 | igd | 0.9956 | C | \|\| id:ebi-a-GCST90001698 | ebi-a-GCST90001698 | TRUE | T | 102458050 | 2.37E-09 | reported | 3408 | 26.73 | rs1312874 |  |  |  |  |  |  |  |  |  |  |  |  |  |  |  |
| -159 | 9 | igd | 0.9955 | G | \|\| id:ebi-a-GCST90001698 | ebi-a-GCST90001698 | TRUE | A | 102642264 | 2.13E-09 | reported | 3408 | 26.49 | rs7872296 |  |  |  |  |  |  |  |  |  |  |  |  |  |  |  |
| -121 | 10 | igd | 0.9921 | G | \|\| id:ebi-a-GCST90001698 | ebi-a-GCST90001698 | TRUE | C | 56635069 | 1.05E-09 | reported | 3408 | 19.77 | rs1840338 |  |  |  |  |  |  |  |  |  |  |  |  |  |  |  |
| -87.22 | 2 | igd | 0.9869 | A | \|\| id:ebi-a-GCST90001698 | ebi-a-GCST90001698 | TRUE | G | 107560107 | 1.55E-08 | reported | 3408 | 15.38 | rs7597139 |  |  |  |  |  |  |  |  |  |  |  |  |  |  |  |
| -0.6378 | 10 | igd | 0.9866 | G | \|\| id:ebi-a-GCST90002030 | ebi-a-GCST90002030 | TRUE | C | 98148557 | 4.26E-08 | reported | 2920 | 0.1161 | rs4919008 |  |  |  |  |  |  |  |  |  |  |  |  |  |  |  |
| 0.5701 | 22 | igd | 0.985 | C | \|\| id:ebi-a-GCST90001702 | ebi-a-GCST90001702 | TRUE | T | 38711124 | 3.26E-08 | reported | 3657 | 0.1029 | rs135765 |  |  |  |  |  |  |  |  |  |  |  |  |  |  |  |
| 0.5855 | 22 | igd | 0.985 | C | \|\| id:ebi-a-GCST90001703 | ebi-a-GCST90001703 | TRUE | T | 38711124 | 1.36E-08 | reported | 3657 | 0.1029 | rs135765 |  |  |  |  |  |  |  |  |  |  |  |  |  |  |  |
| 0.5964 | 22 | igd | 0.985 | C | \|\| id:ebi-a-GCST90001704 | ebi-a-GCST90001704 | TRUE | T | 38711124 | 6.96E-09 | reported | 3657 | 0.1027 | rs135765 |  |  |  |  |  |  |  |  |  |  |  |  |  |  |  |
| -0.7399 | 10 | igd | 0.9839 | C | \|\| id:ebi-a-GCST90002030 | ebi-a-GCST90002030 | TRUE | T | 99336484 | 3.74E-12 | reported | 2920 | 0.1061 | rs4244324 |  |  |  |  |  |  |  |  |  |  |  |  |  |  |  |
| -0.634 | 10 | igd | 0.9839 | C | \|\| id:ebi-a-GCST90002032 | ebi-a-GCST90002032 | TRUE | T | 99336484 | 7.56E-09 | reported | 2920 | 0.1094 | rs4244324 |  |  |  |  |  |  |  |  |  |  |  |  |  |  |  |
| 0.5426 | 22 | igd | 0.9806 | C | \|\| id:ebi-a-GCST90001702 | ebi-a-GCST90001702 | TRUE | T | 38032090 | 3.86E-09 | reported | 3657 | 0.09189 | rs6000850 |  |  |  |  |  |  |  |  |  |  |  |  |  |  |  |
| 0.5349 | 22 | igd | 0.9806 | C | \|\| id:ebi-a-GCST90001703 | ebi-a-GCST90001703 | TRUE | T | 38032090 | 6.31E-09 | reported | 3657 | 0.09188 | rs6000850 |  |  |  |  |  |  |  |  |  |  |  |  |  |  |  |
| 0.5441 | 22 | igd | 0.9806 | C | \|\| id:ebi-a-GCST90001704 | ebi-a-GCST90001704 | TRUE | T | 38032090 | 3.28E-09 | reported | 3657 | 0.09173 | rs6000850 |  |  |  |  |  |  |  |  |  |  |  |  |  |  |  |
| -72.94 | 1 | igd | 0.9795 | T | \|\| id:ebi-a-GCST90001698 | ebi-a-GCST90001698 | TRUE | C | 242844071 | 1.02E-08 | reported | 3408 | 12.7 | rs1556555 |  |  |  |  |  |  |  |  |  |  |  |  |  |  |  |
| 0.853 | 10 | igd | 0.9733 | C | \|\| id:ebi-a-GCST90001961 | ebi-a-GCST90001961 | TRUE | G | 97461206 | 8.77E-23 | reported | 2920 | 0.08609 | rs7085059 |  |  |  |  |  |  |  |  |  |  |  |  |  |  |  |
| -0.7732 | 10 | igd | 0.9733 | C | \|\| id:ebi-a-GCST90002030 | ebi-a-GCST90002030 | TRUE | G | 97461206 | 4.25E-19 | reported | 2920 | 0.08598 | rs7085059 |  |  |  |  |  |  |  |  |  |  |  |  |  |  |  |
| 0.8472 | 10 | igd | 0.9731 | G | \|\| id:ebi-a-GCST90001961 | ebi-a-GCST90001961 | TRUE | A | 97340618 | 1.12E-22 | reported | 2920 | 0.08573 | rs571505 |  |  |  |  |  |  |  |  |  |  |  |  |  |  |  |
| -0.7633 | 10 | igd | 0.9731 | G | \|\| id:ebi-a-GCST90002030 | ebi-a-GCST90002030 | TRUE | A | 97340618 | 7.98E-19 | reported | 2920 | 0.08557 | rs571505 |  |  |  |  |  |  |  |  |  |  |  |  |  |  |  |
| -0.5736 | 10 | igd | 0.9731 | A | \|\| id:ebi-a-GCST90002031 | ebi-a-GCST90002031 | TRUE | G | 97393678 | 7.00E-11 | reported | 2920 | 0.08764 | rs1890187 |  |  |  |  |  |  |  |  |  |  |  |  |  |  |  |
| -1.068 | 10 | igd | 0.973 | C | \|\| id:ebi-a-GCST90001483 | ebi-a-GCST90001483 | TRUE | G | 97461206 | 2.12E-42 | reported | 3405 | 0.0772 | rs7085059 |  |  |  |  |  |  |  |  |  |  |  |  |  |  |  |
| -0.9422 | 10 | igd | 0.973 | C | \|\| id:ebi-a-GCST90001495 | ebi-a-GCST90001495 | TRUE | G | 97461206 | 7.60E-31 | reported | 3405 | 0.08079 | rs7085059 |  |  |  |  |  |  |  |  |  |  |  |  |  |  |  |
| -1.198 | 10 | igd | 0.973 | C | \|\| id:ebi-a-GCST90001660 | ebi-a-GCST90001660 | TRUE | G | 97461206 | 1.85E-50 | reported | 3408 | 0.07886 | rs7085059 |  |  |  |  |  |  |  |  |  |  |  |  |  |  |  |
| -1.1 | 10 | igd | 0.973 | C | \|\| id:ebi-a-GCST90001672 | ebi-a-GCST90001672 | TRUE | G | 97461206 | 8.62E-42 | reported | 3408 | 0.08011 | rs7085059 |  |  |  |  |  |  |  |  |  |  |  |  |  |  |  |
| -1.193 | 10 | igd | 0.9728 | C | \|\| id:ebi-a-GCST90001484 | ebi-a-GCST90001484 | TRUE | G | 97461206 | 7.85E-54 | reported | 3437 | 0.07585 | rs7085059 |  |  |  |  |  |  |  |  |  |  |  |  |  |  |  |
| -0.7781 | 10 | igd | 0.9728 | C | \|\| id:ebi-a-GCST90001491 | ebi-a-GCST90001491 | TRUE | G | 97461206 | 1.81E-22 | reported | 3437 | 0.07924 | rs7085059 |  |  |  |  |  |  |  |  |  |  |  |  |  |  |  |
| -1.081 | 10 | igd | 0.9728 | C | \|\| id:ebi-a-GCST90001496 | ebi-a-GCST90001496 | TRUE | G | 97461206 | 3.12E-41 | reported | 3437 | 0.0793 | rs7085059 |  |  |  |  |  |  |  |  |  |  |  |  |  |  |  |
| -1.009 | 10 | igd | 0.9728 | C | \|\| id:ebi-a-GCST90001497 | ebi-a-GCST90001497 | TRUE | G | 97461206 | 5.88E-37 | reported | 3437 | 0.07853 | rs7085059 |  |  |  |  |  |  |  |  |  |  |  |  |  |  |  |
| -1.051 | 10 | igd | 0.9728 | C | \|\| id:ebi-a-GCST90001670 | ebi-a-GCST90001670 | TRUE | G | 97461206 | 1.22E-39 | reported | 3440 | 0.07875 | rs7085059 |  |  |  |  |  |  |  |  |  |  |  |  |  |  |  |
| -1.075 | 10 | igd | 0.9728 | C | \|\| id:ebi-a-GCST90001671 | ebi-a-GCST90001671 | TRUE | G | 97461206 | 2.74E-41 | reported | 3440 | 0.07885 | rs7085059 |  |  |  |  |  |  |  |  |  |  |  |  |  |  |  |
| -1.047 | 10 | igd | 0.9727 | G | \|\| id:ebi-a-GCST90001483 | ebi-a-GCST90001483 | TRUE | A | 97340618 | 2.91E-41 | reported | 3405 | 0.07681 | rs571505 |  |  |  |  |  |  |  |  |  |  |  |  |  |  |  |
| -0.7429 | 10 | igd | 0.9727 | G | \|\| id:ebi-a-GCST90001489 | ebi-a-GCST90001489 | TRUE | A | 97340618 | 2.86E-20 | reported | 3405 | 0.08003 | rs571505 |  |  |  |  |  |  |  |  |  |  |  |  |  |  |  |
| -0.9331 | 10 | igd | 0.9727 | G | \|\| id:ebi-a-GCST90001495 | ebi-a-GCST90001495 | TRUE | A | 97340618 | 1.31E-30 | reported | 3405 | 0.08034 | rs571505 |  |  |  |  |  |  |  |  |  |  |  |  |  |  |  |
| -1.185 | 10 | igd | 0.9727 | G | \|\| id:ebi-a-GCST90001660 | ebi-a-GCST90001660 | TRUE | A | 97340618 | 5.98E-50 | reported | 3408 | 0.07844 | rs571505 |  |  |  |  |  |  |  |  |  |  |  |  |  |  |  |
| -1.084 | 10 | igd | 0.9727 | G | \|\| id:ebi-a-GCST90001672 | ebi-a-GCST90001672 | TRUE | A | 97340618 | 4.63E-41 | reported | 3408 | 0.07969 | rs571505 |  |  |  |  |  |  |  |  |  |  |  |  |  |  |  |
| -1.176 | 10 | igd | 0.9725 | G | \|\| id:ebi-a-GCST90001484 | ebi-a-GCST90001484 | TRUE | A | 97340618 | 6.16E-53 | reported | 3437 | 0.07547 | rs571505 |  |  |  |  |  |  |  |  |  |  |  |  |  |  |  |
| -1.085 | 10 | igd | 0.9725 | G | \|\| id:ebi-a-GCST90001485 | ebi-a-GCST90001485 | TRUE | A | 97340618 | 3.33E-45 | reported | 3437 | 0.07575 | rs571505 |  |  |  |  |  |  |  |  |  |  |  |  |  |  |  |
| -0.7795 | 10 | igd | 0.9725 | G | \|\| id:ebi-a-GCST90001491 | ebi-a-GCST90001491 | TRUE | A | 97340618 | 8.85E-23 | reported | 3437 | 0.07878 | rs571505 |  |  |  |  |  |  |  |  |  |  |  |  |  |  |  |
| -1.07 | 10 | igd | 0.9725 | G | \|\| id:ebi-a-GCST90001496 | ebi-a-GCST90001496 | TRUE | A | 97340618 | 7.65E-41 | reported | 3437 | 0.07888 | rs571505 |  |  |  |  |  |  |  |  |  |  |  |  |  |  |  |
| -1.001 | 10 | igd | 0.9725 | G | \|\| id:ebi-a-GCST90001497 | ebi-a-GCST90001497 | TRUE | A | 97340618 | 8.40E-37 | reported | 3437 | 0.0781 | rs571505 |  |  |  |  |  |  |  |  |  |  |  |  |  |  |  |
| -1.125 | 10 | igd | 0.9725 | A | \|\| id:ebi-a-GCST90001658 | ebi-a-GCST90001658 | TRUE | G | 97393678 | 1.47E-49 | reported | 3440 | 0.07481 | rs1890187 |  |  |  |  |  |  |  |  |  |  |  |  |  |  |  |
| -1.169 | 10 | igd | 0.9725 | A | \|\| id:ebi-a-GCST90001659 | ebi-a-GCST90001659 | TRUE | G | 97393678 | 1.65E-53 | reported | 3440 | 0.07463 | rs1890187 |  |  |  |  |  |  |  |  |  |  |  |  |  |  |  |
| -1.039 | 10 | igd | 0.9725 | G | \|\| id:ebi-a-GCST90001670 | ebi-a-GCST90001670 | TRUE | A | 97340618 | 3.38E-39 | reported | 3440 | 0.07833 | rs571505 |  |  |  |  |  |  |  |  |  |  |  |  |  |  |  |
| -1.062 | 10 | igd | 0.9725 | G | \|\| id:ebi-a-GCST90001671 | ebi-a-GCST90001671 | TRUE | A | 97340618 | 1.04E-40 | reported | 3440 | 0.07844 | rs571505 |  |  |  |  |  |  |  |  |  |  |  |  |  |  |  |
| 0.5416 | 16 | igd | 0.9713 | G | \|\| id:ebi-a-GCST90002105 | ebi-a-GCST90002105 | TRUE | C | 11004549 | 4.00E-11 | reported | 2872 | 0.0817 | rs7204799 |  |  |  |  |  |  |  |  |  |  |  |  |  |  |  |
| -0.4298 | 10 | igd | 0.9692 | G | \|\| id:ebi-a-GCST90001495 | ebi-a-GCST90001495 | TRUE | A | 100183774 | 1.52E-08 | reported | 3405 | 0.07576 | rs10883093 |  |  |  |  |  |  |  |  |  |  |  |  |  |  |  |
| -0.435 | 10 | igd | 0.9692 | G | \|\| id:ebi-a-GCST90001660 | ebi-a-GCST90001660 | TRUE | A | 100183774 | 6.72E-09 | reported | 3408 | 0.07483 | rs10883093 |  |  |  |  |  |  |  |  |  |  |  |  |  |  |  |
| -0.4377 | 10 | igd | 0.9671 | C | \|\| id:ebi-a-GCST90001484 | ebi-a-GCST90001484 | TRUE | A | 96815772 | 3.63E-10 | reported | 3437 | 0.06961 | rs1341157 |  |  |  |  |  |  |  |  |  |  |  |  |  |  |  |
| -0.4125 | 10 | igd | 0.9671 | C | \|\| id:ebi-a-GCST90001485 | ebi-a-GCST90001485 | TRUE | A | 96815772 | 3.32E-09 | reported | 3437 | 0.06956 | rs1341157 |  |  |  |  |  |  |  |  |  |  |  |  |  |  |  |
| -0.4731 | 10 | igd | 0.9671 | C | \|\| id:ebi-a-GCST90001491 | ebi-a-GCST90001491 | TRUE | A | 96815772 | 2.78E-11 | reported | 3437 | 0.07082 | rs1341157 |  |  |  |  |  |  |  |  |  |  |  |  |  |  |  |
| -0.5079 | 10 | igd | 0.9671 | C | \|\| id:ebi-a-GCST90001496 | ebi-a-GCST90001496 | TRUE | A | 96815772 | 1.72E-12 | reported | 3437 | 0.07171 | rs1341157 |  |  |  |  |  |  |  |  |  |  |  |  |  |  |  |
| -0.5123 | 10 | igd | 0.9671 | C | \|\| id:ebi-a-GCST90001497 | ebi-a-GCST90001497 | TRUE | A | 96815772 | 5.65E-13 | reported | 3437 | 0.07079 | rs1341157 |  |  |  |  |  |  |  |  |  |  |  |  |  |  |  |
| -0.3851 | 10 | igd | 0.967 | C | \|\| id:ebi-a-GCST90001483 | ebi-a-GCST90001483 | TRUE | A | 96815772 | 4.19E-08 | reported | 3405 | 0.07007 | rs1341157 |  |  |  |  |  |  |  |  |  |  |  |  |  |  |  |
| -0.4222 | 10 | igd | 0.967 | C | \|\| id:ebi-a-GCST90001489 | ebi-a-GCST90001489 | TRUE | A | 96815772 | 4.82E-09 | reported | 3405 | 0.07194 | rs1341157 |  |  |  |  |  |  |  |  |  |  |  |  |  |  |  |
| -0.4527 | 10 | igd | 0.967 | C | \|\| id:ebi-a-GCST90001495 | ebi-a-GCST90001495 | TRUE | A | 96815772 | 4.72E-10 | reported | 3405 | 0.07247 | rs1341157 |  |  |  |  |  |  |  |  |  |  |  |  |  |  |  |
| -0.3977 | 10 | igd | 0.967 | C | \|\| id:ebi-a-GCST90001659 | ebi-a-GCST90001659 | TRUE | A | 96815772 | 6.43E-09 | reported | 3440 | 0.06833 | rs1341157 |  |  |  |  |  |  |  |  |  |  |  |  |  |  |  |
| -0.5754 | 6 | igd | 0.9666 | A | \|\| id:ebi-a-GCST90002105 | ebi-a-GCST90002105 | TRUE | T | 32066819 | 8.36E-13 | reported | 2872 | 0.08006 | rs3117182 |  |  |  |  |  |  |  |  |  |  |  |  |  |  |  |
| -0.5281 | 10 | igd | 0.9654 | C | \|\| id:ebi-a-GCST90002030 | ebi-a-GCST90002030 | TRUE | A | 96815772 | 1.94E-12 | reported | 2920 | 0.0747 | rs1341157 |  |  |  |  |  |  |  |  |  |  |  |  |  |  |  |
| -0.4986 | 10 | igd | 0.9654 | C | \|\| id:ebi-a-GCST90002031 | ebi-a-GCST90002031 | TRUE | A | 96815772 | 8.76E-11 | reported | 2920 | 0.07658 | rs1341157 |  |  |  |  |  |  |  |  |  |  |  |  |  |  |  |
| -0.562 | 10 | igd | 0.9654 | C | \|\| id:ebi-a-GCST90002032 | ebi-a-GCST90002032 | TRUE | A | 96815772 | 2.40E-13 | reported | 2920 | 0.07638 | rs1341157 |  |  |  |  |  |  |  |  |  |  |  |  |  |  |  |
| -0.498 | 6 | igd | 0.9643 | A | \|\| id:ebi-a-GCST90002105 | ebi-a-GCST90002105 | TRUE | T | 30972121 | 6.89E-11 | reported | 2872 | 0.07606 | rs2844668 |  |  |  |  |  |  |  |  |  |  |  |  |  |  |  |
| 0.5052 | 1 | igd | 0.9632 | C | \|\| id:ebi-a-GCST90001854 | ebi-a-GCST90001854 | TRUE | A | 165407517 | 1.61E-12 | reported | 2919 | 0.0712 | rs157885 |  |  |  |  |  |  |  |  |  |  |  |  |  |  |  |
| 0.4425 | 1 | igd | 0.9632 | C | \|\| id:ebi-a-GCST90001856 | ebi-a-GCST90001856 | TRUE | A | 165407517 | 8.19E-10 | reported | 2919 | 0.07181 | rs157885 |  |  |  |  |  |  |  |  |  |  |  |  |  |  |  |
| 0.5134 | 1 | igd | 0.9632 | C | \|\| id:ebi-a-GCST90001858 | ebi-a-GCST90001858 | TRUE | A | 165407517 | 1.05E-12 | reported | 2919 | 0.07175 | rs157885 |  |  |  |  |  |  |  |  |  |  |  |  |  |  |  |
| 0.5326 | 1 | igd | 0.9632 | C | \|\| id:ebi-a-GCST90001860 | ebi-a-GCST90001860 | TRUE | A | 165407517 | 1.82E-13 | reported | 2919 | 0.07201 | rs157885 |  |  |  |  |  |  |  |  |  |  |  |  |  |  |  |
| 0.409 | 1 | igd | 0.9632 | C | \|\| id:ebi-a-GCST90001861 | ebi-a-GCST90001861 | TRUE | A | 165407517 | 1.02E-08 | reported | 2919 | 0.07122 | rs157885 |  |  |  |  |  |  |  |  |  |  |  |  |  |  |  |
| 0.4445 | 1 | igd | 0.9547 | T | \|\| id:ebi-a-GCST90001866 | ebi-a-GCST90001866 | TRUE | C | 167428234 | 5.63E-12 | reported | 2914 | 0.06427 | rs4542198 |  |  |  |  |  |  |  |  |  |  |  |  |  |  |  |
| 0.457 | 1 | igd | 0.9546 | T | \|\| id:ebi-a-GCST90001854 | ebi-a-GCST90001854 | TRUE | C | 167428234 | 1.82E-12 | reported | 2919 | 0.06456 | rs4542198 |  |  |  |  |  |  |  |  |  |  |  |  |  |  |  |
| -0.3291 | 6 | igd | 0.9534 | A | \|\| id:ebi-a-GCST90001820 | ebi-a-GCST90001820 | TRUE | G | 43182657 | 3.08E-08 | reported | 3656 | 0.05931 | rs1214714 |  |  |  |  |  |  |  |  |  |  |  |  |  |  |  |
| -0.4048 | 19 | igd | 0.95305 | C | \|\| id:prot-a-439 | prot-a-439 | TRUE | G | 51661468 | 4.07E-12 | reported | 3301 | 0.0584 | rs273671 |  |  |  |  |  |  |  |  |  |  |  |  |  |  |  |
| -0.3159 | 1 | igd | 0.9508 | A | \|\| id:ebi-a-GCST90001981 | ebi-a-GCST90001981 | TRUE | T | 161054223 | 4.36E-08 | reported | 3618 | 0.05757 | rs4233364 |  |  |  |  |  |  |  |  |  |  |  |  |  |  |  |
| -0.3186 | 10 | igd | 0.9478 | T | \|\| id:ebi-a-GCST90001670 | ebi-a-GCST90001670 | TRUE | G | 95565254 | 2.11E-08 | reported | 3440 | 0.05673 | rs1924699 |  |  |  |  |  |  |  |  |  |  |  |  |  |  |  |
| -0.3286 | 10 | igd | 0.9478 | T | \|\| id:ebi-a-GCST90001672 | ebi-a-GCST90001672 | TRUE | G | 95565254 | 1.36E-08 | reported | 3408 | 0.05772 | rs1924699 |  |  |  |  |  |  |  |  |  |  |  |  |  |  |  |
| -0.4313 | 1 | igd | 0.9459 | A | \|\| id:ebi-a-GCST90001526 | ebi-a-GCST90001526 | TRUE | G | 161049358 | 7.11E-09 | reported | 1858 | 0.07416 | rs3820097 |  |  |  |  |  |  |  |  |  |  |  |  |  |  |  |
| 0.4291 | 1 | igd | 0.9459 | A | \|\| id:ebi-a-GCST90001528 | ebi-a-GCST90001528 | TRUE | G | 161049358 | 8.70E-09 | reported | 1858 | 0.07422 | rs3820097 |  |  |  |  |  |  |  |  |  |  |  |  |  |  |  |
| 0.296 | 4 | igd | 0.9398 | A | \|\| id:ebi-a-GCST90001815 | ebi-a-GCST90001815 | TRUE | G | 13909992 | 4.71E-09 | reported | 3657 | 0.05042 | rs2203043 |  |  |  |  |  |  |  |  |  |  |  |  |  |  |  |
| 0.331 | 2 | igd | 0.9397 | A | \|\| id:ebi-a-GCST90002098 | ebi-a-GCST90002098 | TRUE | G | 39618666 | 1.60E-09 | reported | 2910 | 0.05469 | rs4670932 |  |  |  |  |  |  |  |  |  |  |  |  |  |  |  |
| -0.521 | 6 | igd | 0.937 | A | \|\| id:ebi-a-GCST90002112 | ebi-a-GCST90002112 | TRUE | G | 32278635 | 3.23E-11 | reported | 1635 | 0.07797 | rs9268199 |  |  |  |  |  |  |  |  |  |  |  |  |  |  |  |
| -0.3619 | 2 | igd | 0.9338 | A | \|\| id:ebi-a-GCST90002098 | ebi-a-GCST90002098 | TRUE | G | 38156133 | 1.17E-11 | reported | 2910 | 0.05313 | rs7565287 |  |  |  |  |  |  |  |  |  |  |  |  |  |  |  |
| -0.407 | 6 | igd | 0.9248 | G | \|\| id:ebi-a-GCST90002112 | ebi-a-GCST90002112 | TRUE | A | 31597915 | 3.33E-08 | reported | 1635 | 0.07333 | rs3130625 |  |  |  |  |  |  |  |  |  |  |  |  |  |  |  |
| 0.3805 | 11 | igd | 0.9244 | A | \|\| id:ebi-a-GCST90002071 | ebi-a-GCST90002071 | TRUE | G | 5306509 | 2.39E-12 | reported | 2872 | 0.05405 | rs4910742 |  |  |  |  |  |  |  |  |  |  |  |  |  |  |  |
| -0.5638 | 6 | igd | 0.924 | G | \|\| id:ebi-a-GCST90002108 | ebi-a-GCST90002108 | TRUE | A | 31597915 | 2.07E-14 | reported | 1579 | 0.07304 | rs3130625 |  |  |  |  |  |  |  |  |  |  |  |  |  |  |  |
| -0.477 | 6 | igd | 0.9236 | A | \|\| id:ebi-a-GCST90002105 | ebi-a-GCST90002105 | TRUE | G | 31618761 | 5.12E-17 | reported | 2872 | 0.05655 | rs3130050 |  |  |  |  |  |  |  |  |  |  |  |  |  |  |  |
| 0.405 | 22 | igd | 0.9217 | T | \|\| id:ebi-a-GCST90001702 | ebi-a-GCST90001702 | TRUE | C | 40900841 | 2.29E-17 | reported | 3657 | 0.04754 | rs139066 |  |  |  |  |  |  |  |  |  |  |  |  |  |  |  |
| 0.3896 | 22 | igd | 0.9217 | T | \|\| id:ebi-a-GCST90001703 | ebi-a-GCST90001703 | TRUE | C | 40900841 | 3.55E-16 | reported | 3657 | 0.04756 | rs139066 |  |  |  |  |  |  |  |  |  |  |  |  |  |  |  |
| 0.3433 | 22 | igd | 0.9217 | T | \|\| id:ebi-a-GCST90001704 | ebi-a-GCST90001704 | TRUE | C | 40900841 | 6.67E-13 | reported | 3657 | 0.04759 | rs139066 |  |  |  |  |  |  |  |  |  |  |  |  |  |  |  |
| 0.3806 | 22 | igd | 0.9217 | T | \|\| id:ebi-a-GCST90001710 | ebi-a-GCST90001710 | TRUE | C | 40900841 | 1.43E-15 | reported | 3657 | 0.04747 | rs139066 |  |  |  |  |  |  |  |  |  |  |  |  |  |  |  |
| 0.386 | 22 | igd | 0.9217 | T | \|\| id:ebi-a-GCST90001711 | ebi-a-GCST90001711 | TRUE | C | 40900841 | 4.73E-16 | reported | 3657 | 0.04733 | rs139066 |  |  |  |  |  |  |  |  |  |  |  |  |  |  |  |
| -0.3348 | 10 | igd | 0.9204 | T | \|\| id:ebi-a-GCST90001785 | ebi-a-GCST90001785 | TRUE | C | 6042034 | 4.67E-13 | reported | 3649 | 0.0461 | rs10795731 |  |  |  |  |  |  |  |  |  |  |  |  |  |  |  |
| -0.2694 | 10 | igd | 0.9204 | T | \|\| id:ebi-a-GCST90001786 | ebi-a-GCST90001786 | TRUE | C | 6042034 | 4.93E-09 | reported | 3651 | 0.04594 | rs10795731 |  |  |  |  |  |  |  |  |  |  |  |  |  |  |  |
| -0.5369 | 10 | igd | 0.9203 | T | \|\| id:ebi-a-GCST90001777 | ebi-a-GCST90001777 | TRUE | C | 6042034 | 3.40E-32 | reported | 3657 | 0.04502 | rs10795731 |  |  |  |  |  |  |  |  |  |  |  |  |  |  |  |
| -0.419 | 10 | igd | 0.9203 | T | \|\| id:ebi-a-GCST90001787 | ebi-a-GCST90001787 | TRUE | C | 6042034 | 7.01E-20 | reported | 3657 | 0.04564 | rs10795731 |  |  |  |  |  |  |  |  |  |  |  |  |  |  |  |
| -0.529 | 10 | igd | 0.9203 | T | \|\| id:ebi-a-GCST90001789 | ebi-a-GCST90001789 | TRUE | C | 6042034 | 1.08E-31 | reported | 3657 | 0.04473 | rs10795731 |  |  |  |  |  |  |  |  |  |  |  |  |  |  |  |
| -0.5432 | 10 | igd | 0.9203 | T | \|\| id:ebi-a-GCST90001790 | ebi-a-GCST90001790 | TRUE | C | 6042034 | 6.85E-33 | reported | 3657 | 0.04503 | rs10795731 |  |  |  |  |  |  |  |  |  |  |  |  |  |  |  |
| -0.5253 | 10 | igd | 0.9203 | T | \|\| id:ebi-a-GCST90001793 | ebi-a-GCST90001793 | TRUE | C | 6042034 | 2.31E-30 | reported | 3657 | 0.04546 | rs10795731 |  |  |  |  |  |  |  |  |  |  |  |  |  |  |  |
| -0.2835 | 14 | igd | 0.9188 | A | \|\| id:ebi-a-GCST90001821 | ebi-a-GCST90001821 | TRUE | G | 105961529 | 4.43E-10 | reported | 3656 | 0.04532 | rs4983425 |  |  |  |  |  |  |  |  |  |  |  |  |  |  |  |
| -0.2982 | 14 | igd | 0.9186 | A | \|\| id:ebi-a-GCST90001820 | ebi-a-GCST90001820 | TRUE | G | 105961529 | 5.63E-11 | reported | 3656 | 0.04537 | rs4983425 |  |  |  |  |  |  |  |  |  |  |  |  |  |  |  |
| -0.4058 | 10 | igd | 0.9183 | T | \|\| id:ebi-a-GCST90001781 | ebi-a-GCST90001781 | TRUE | C | 6042034 | 1.60E-10 | reported | 1835 | 0.06309 | rs10795731 |  |  |  |  |  |  |  |  |  |  |  |  |  |  |  |
| -0.4436 | 10 | igd | 0.9183 | T | \|\| id:ebi-a-GCST90001782 | ebi-a-GCST90001782 | TRUE | C | 6042034 | 6.12E-13 | reported | 1837 | 0.06119 | rs10795731 |  |  |  |  |  |  |  |  |  |  |  |  |  |  |  |
| -0.2921 | 2 | igd | 0.9156 | A | \|\| id:ebi-a-GCST90002102 | ebi-a-GCST90002102 | TRUE | T | 38132650 | 4.93E-10 | reported | 2919 | 0.0468 | rs2565661 |  |  |  |  |  |  |  |  |  |  |  |  |  |  |  |
| 0.3463 | 19 | igd | 0.9152 | C | \|\| id:ebi-a-GCST90001946 | ebi-a-GCST90001946 | TRUE | A | 52932068 | 3.26E-08 | reported | 1634 | 0.06236 | rs2867975 |  |  |  |  |  |  |  |  |  |  |  |  |  |  |  |
| 0.254 | 20 | igd | 0.9147 | C | \|\| id:ebi-a-GCST90001981 | ebi-a-GCST90001981 | TRUE | T | 44776543 | 2.07E-08 | reported | 3618 | 0.0452 | rs2024570 |  |  |  |  |  |  |  |  |  |  |  |  |  |  |  |
| 0.3622 | 19 | igd | 0.9141 | A | \|\| id:ebi-a-GCST90001947 | ebi-a-GCST90001947 | TRUE | G | 52921906 | 3.40E-08 | reported | 1577 | 0.0653 | rs2059816 |  |  |  |  |  |  |  |  |  |  |  |  |  |  |  |
| 0.2745 | 10 | igd | 0.9068 | A | \|\| id:ebi-a-GCST90001672 | ebi-a-GCST90001672 | TRUE | G | 97055832 | 6.27E-10 | reported | 3408 | 0.04427 | rs10786197 |  |  |  |  |  |  |  |  |  |  |  |  |  |  |  |
| 0.2893 | 10 | igd | 0.9061 | A | \|\| id:ebi-a-GCST90001670 | ebi-a-GCST90001670 | TRUE | G | 97055832 | 3.23E-11 | reported | 3440 | 0.04346 | rs10786197 |  |  |  |  |  |  |  |  |  |  |  |  |  |  |  |
| 0.2554 | 10 | igd | 0.9061 | A | \|\| id:ebi-a-GCST90001671 | ebi-a-GCST90001671 | TRUE | G | 97055832 | 4.88E-09 | reported | 3440 | 0.04354 | rs10786197 |  |  |  |  |  |  |  |  |  |  |  |  |  |  |  |
| -0.4818 | 6 | igd | 0.9049 | G | \|\| id:ebi-a-GCST90002112 | ebi-a-GCST90002112 | TRUE | A | 31480923 | 1.10E-13 | reported | 1635 | 0.0643 | rs3131633 |  |  |  |  |  |  |  |  |  |  |  |  |  |  |  |
| -0.6041 | 6 | igd | 0.9046 | G | \|\| id:ebi-a-GCST90002105 | ebi-a-GCST90002105 | TRUE | A | 31480923 | 2.67E-31 | reported | 2872 | 0.0513 | rs3131633 |  |  |  |  |  |  |  |  |  |  |  |  |  |  |  |
| -0.6209 | 6 | igd | 0.9041 | G | \|\| id:ebi-a-GCST90002108 | ebi-a-GCST90002108 | TRUE | A | 31480923 | 9.45E-22 | reported | 1579 | 0.06384 | rs3131633 |  |  |  |  |  |  |  |  |  |  |  |  |  |  |  |
| -0.4235 | 6 | igd | 0.904 | G | \|\| id:ebi-a-GCST90002109 | ebi-a-GCST90002109 | TRUE | A | 31480923 | 5.68E-11 | reported | 1578 | 0.06419 | rs3131633 |  |  |  |  |  |  |  |  |  |  |  |  |  |  |  |
| -0.2401 | 1 | igd | 0.9022 | G | \|\| id:ebi-a-GCST90001994 | ebi-a-GCST90001994 | TRUE | A | 161874146 | 1.19E-08 | reported | 3590 | 0.04202 | rs7517386 |  |  |  |  |  |  |  |  |  |  |  |  |  |  |  |
| -0.3554 | 19 | igd | 0.9005 | C | \|\| id:ebi-a-GCST90001953 | ebi-a-GCST90001953 | TRUE | T | 51861040 | 1.58E-08 | reported | 1558 | 0.06254 | rs1862496 |  |  |  |  |  |  |  |  |  |  |  |  |  |  |  |
| -0.3512 | 19 | igd | 0.9003 | C | \|\| id:ebi-a-GCST90001954 | ebi-a-GCST90001954 | TRUE | T | 51861040 | 2.33E-08 | reported | 1555 | 0.06256 | rs1862496 |  |  |  |  |  |  |  |  |  |  |  |  |  |  |  |
| -0.3499 | 19 | igd | 0.9003 | C | \|\| id:ebi-a-GCST90001955 | ebi-a-GCST90001955 | TRUE | T | 51861040 | 2.82E-08 | reported | 1554 | 0.0627 | rs1862496 |  |  |  |  |  |  |  |  |  |  |  |  |  |  |  |
| -0.6213 | 6 | igd | 0.9001 | A | \|\| id:ebi-a-GCST90002105 | ebi-a-GCST90002105 | TRUE | G | 32212233 | 3.04E-39 | reported | 2872 | 0.04669 | rs9267948 |  |  |  |  |  |  |  |  |  |  |  |  |  |  |  |
| -0.2306 | 1 | igd | 0.8997 | G | \|\| id:ebi-a-GCST90001994 | ebi-a-GCST90001994 | TRUE | A | 161736035 | 2.76E-08 | reported | 3590 | 0.04142 | rs2298019 |  |  |  |  |  |  |  |  |  |  |  |  |  |  |  |
| -0.3177 | 1 | igd | 0.8979 | G | \|\| id:ebi-a-GCST90002014 | ebi-a-GCST90002014 | TRUE | A | 161763933 | 2.27E-11 | reported | 2870 | 0.04732 | rs2134690 |  |  |  |  |  |  |  |  |  |  |  |  |  |  |  |
| 0.4659 | 3 | igd | 0.8964 | G | \|\| id:ebi-a-GCST90002036 | ebi-a-GCST90002036 | TRUE | A | 52503800 | 1.96E-24 | reported | 2871 | 0.04526 | rs6445484 |  |  |  |  |  |  |  |  |  |  |  |  |  |  |  |
| 0.4055 | 3 | igd | 0.8955 | C | \|\| id:ebi-a-GCST90002035 | ebi-a-GCST90002035 | TRUE | T | 52528000 | 4.79E-19 | reported | 2871 | 0.04516 | rs6787493 |  |  |  |  |  |  |  |  |  |  |  |  |  |  |  |
| -0.4787 | 6 | igd | 0.8942 | G | \|\| id:ebi-a-GCST90002108 | ebi-a-GCST90002108 | TRUE | A | 32636073 | 3.60E-14 | reported | 1579 | 0.06261 | rs3828802 |  |  |  |  |  |  |  |  |  |  |  |  |  |  |  |
| -0.3986 | 6 | igd | 0.8932 | C | \|\| id:ebi-a-GCST90002110 | ebi-a-GCST90002110 | TRUE | T | 32237926 | 3.93E-10 | reported | 1634 | 0.06332 | rs6913182 |  |  |  |  |  |  |  |  |  |  |  |  |  |  |  |
| -0.2541 | 2 | igd | 0.8918 | C | \|\| id:ebi-a-GCST90002098 | ebi-a-GCST90002098 | TRUE | T | 38100259 | 2.40E-09 | reported | 2910 | 0.04244 | rs2707233 |  |  |  |  |  |  |  |  |  |  |  |  |  |  |  |
| 0.3017 | 10 | igd | 0.8875 | C | \|\| id:ebi-a-GCST90002030 | ebi-a-GCST90002030 | TRUE | G | 99596664 | 3.47E-12 | reported | 2920 | 0.04319 | rs4300338 |  |  |  |  |  |  |  |  |  |  |  |  |  |  |  |
| 0.2649 | 10 | igd | 0.8875 | C | \|\| id:ebi-a-GCST90002031 | ebi-a-GCST90002031 | TRUE | G | 99596664 | 2.52E-09 | reported | 2920 | 0.0443 | rs4300338 |  |  |  |  |  |  |  |  |  |  |  |  |  |  |  |
| 0.295 | 10 | igd | 0.8875 | C | \|\| id:ebi-a-GCST90002032 | ebi-a-GCST90002032 | TRUE | G | 99596664 | 2.98E-11 | reported | 2920 | 0.04421 | rs4300338 |  |  |  |  |  |  |  |  |  |  |  |  |  |  |  |
| 0.2212 | 10 | igd | 0.8872 | C | \|\| id:ebi-a-GCST90001658 | ebi-a-GCST90001658 | TRUE | G | 99596664 | 1.18E-08 | reported | 3440 | 0.03869 | rs4300338 |  |  |  |  |  |  |  |  |  |  |  |  |  |  |  |
| 0.2509 | 10 | igd | 0.8871 | C | \|\| id:ebi-a-GCST90001489 | ebi-a-GCST90001489 | TRUE | G | 99596664 | 7.72E-10 | reported | 3405 | 0.04067 | rs4300338 |  |  |  |  |  |  |  |  |  |  |  |  |  |  |  |
| 0.2345 | 10 | igd | 0.8871 | C | \|\| id:ebi-a-GCST90001491 | ebi-a-GCST90001491 | TRUE | G | 99596664 | 5.27E-09 | reported | 3437 | 0.04007 | rs4300338 |  |  |  |  |  |  |  |  |  |  |  |  |  |  |  |
| 0.2581 | 10 | igd | 0.8871 | C | \|\| id:ebi-a-GCST90001495 | ebi-a-GCST90001495 | TRUE | G | 99596664 | 3.32E-10 | reported | 3405 | 0.04095 | rs4300338 |  |  |  |  |  |  |  |  |  |  |  |  |  |  |  |
| 0.2788 | 10 | igd | 0.8871 | C | \|\| id:ebi-a-GCST90001496 | ebi-a-GCST90001496 | TRUE | G | 99596664 | 7.08E-12 | reported | 3437 | 0.04053 | rs4300338 |  |  |  |  |  |  |  |  |  |  |  |  |  |  |  |
| 0.2631 | 10 | igd | 0.8871 | C | \|\| id:ebi-a-GCST90001497 | ebi-a-GCST90001497 | TRUE | G | 99596664 | 5.74E-11 | reported | 3437 | 0.04004 | rs4300338 |  |  |  |  |  |  |  |  |  |  |  |  |  |  |  |
| -0.3348 | 6 | igd | 0.8816 | C | \|\| id:ebi-a-GCST90002108 | ebi-a-GCST90002108 | TRUE | G | 30823075 | 1.69E-08 | reported | 1579 | 0.05904 | rs3130654 |  |  |  |  |  |  |  |  |  |  |  |  |  |  |  |
| -0.6358 | 10 | igd | 0.879 | T | \|\| id:ebi-a-GCST90001485 | ebi-a-GCST90001485 | TRUE | C | 97449273 | 4.23E-63 | reported | 3437 | 0.03714 | rs951418 |  |  |  |  |  |  |  |  |  |  |  |  |  |  |  |
| 0.3114 | 10 | igd | 0.875 | C | \|\| id:ebi-a-GCST90002030 | ebi-a-GCST90002030 | TRUE | T | 100050787 | 5.74E-14 | reported | 2920 | 0.04123 | rs10736128 |  |  |  |  |  |  |  |  |  |  |  |  |  |  |  |
| 0.2776 | 10 | igd | 0.875 | C | \|\| id:ebi-a-GCST90002031 | ebi-a-GCST90002031 | TRUE | T | 100050787 | 5.88E-11 | reported | 2920 | 0.04225 | rs10736128 |  |  |  |  |  |  |  |  |  |  |  |  |  |  |  |
| 0.3085 | 10 | igd | 0.875 | C | \|\| id:ebi-a-GCST90002032 | ebi-a-GCST90002032 | TRUE | T | 100050787 | 3.23E-13 | reported | 2920 | 0.04216 | rs10736128 |  |  |  |  |  |  |  |  |  |  |  |  |  |  |  |
| 0.2082 | 6 | igd | 0.8748 | G | \|\| id:ebi-a-GCST90001746 | ebi-a-GCST90001746 | TRUE | A | 15898884 | 1.85E-08 | reported | 3657 | 0.03692 | rs1569590 |  |  |  |  |  |  |  |  |  |  |  |  |  |  |  |
| 0.2257 | 6 | igd | 0.8748 | G | \|\| id:ebi-a-GCST90001758 | ebi-a-GCST90001758 | TRUE | A | 15898884 | 1.10E-09 | reported | 3657 | 0.03693 | rs1569590 |  |  |  |  |  |  |  |  |  |  |  |  |  |  |  |
| 0.207 | 6 | igd | 0.8748 | G | \|\| id:ebi-a-GCST90001761 | ebi-a-GCST90001761 | TRUE | A | 15898884 | 2.88E-08 | reported | 3657 | 0.03723 | rs1569590 |  |  |  |  |  |  |  |  |  |  |  |  |  |  |  |
| 0.2058 | 6 | igd | 0.8744 | G | \|\| id:ebi-a-GCST90001757 | ebi-a-GCST90001757 | TRUE | T | 15897003 | 3.14E-08 | reported | 3657 | 0.03711 | rs909846 |  |  |  |  |  |  |  |  |  |  |  |  |  |  |  |
| 0.2138 | 6 | igd | 0.8737 | T | \|\| id:ebi-a-GCST90001745 | ebi-a-GCST90001745 | TRUE | C | 15858996 | 7.67E-09 | reported | 3657 | 0.03693 | rs6459417 |  |  |  |  |  |  |  |  |  |  |  |  |  |  |  |
| 0.2282 | 6 | igd | 0.8737 | T | \|\| id:ebi-a-GCST90001760 | ebi-a-GCST90001760 | TRUE | C | 15858996 | 7.25E-10 | reported | 3657 | 0.03694 | rs6459417 |  |  |  |  |  |  |  |  |  |  |  |  |  |  |  |
| 0.4684 | 16 | igd | 0.8668 | C | \|\| id:ebi-a-GCST90002090 | ebi-a-GCST90002090 | TRUE | T | 31379355 | 9.63E-30 | reported | 2850 | 0.04088 | rs8046381 |  |  |  |  |  |  |  |  |  |  |  |  |  |  |  |
| 0.2329 | 1 | igd | 0.864 | T | \|\| id:ebi-a-GCST90001863 | ebi-a-GCST90001863 | TRUE | A | 167406513 | 4.03E-09 | reported | 2919 | 0.03947 | rs2949670 |  |  |  |  |  |  |  |  |  |  |  |  |  |  |  |
| -0.2232 | 1 | igd | 0.8628 | T | \|\| id:ebi-a-GCST90001860 | ebi-a-GCST90001860 | TRUE | G | 167457578 | 4.79E-08 | reported | 2919 | 0.04078 | rs858548 |  |  |  |  |  |  |  |  |  |  |  |  |  |  |  |
| 0.2068 | 5 | igd | 0.8616 | T | \|\| id:ebi-a-GCST90001929 | ebi-a-GCST90001929 | TRUE | C | 35989739 | 4.82E-08 | reported | 2920 | 0.03778 | rs7711442 |  |  |  |  |  |  |  |  |  |  |  |  |  |  |  |
| -0.3794 | 2 | igd | 0.8558 | C | \|\| id:ebi-a-GCST90002098 | ebi-a-GCST90002098 | TRUE | T | 39216773 | 2.53E-24 | reported | 2910 | 0.03694 | rs963730 |  |  |  |  |  |  |  |  |  |  |  |  |  |  |  |
| -0.2235 | 2 | igd | 0.8554 | C | \|\| id:ebi-a-GCST90002102 | ebi-a-GCST90002102 | TRUE | T | 39216773 | 1.74E-09 | reported | 2919 | 0.03701 | rs963730 |  |  |  |  |  |  |  |  |  |  |  |  |  |  |  |
| 0.2635 | 7 | igd | 0.8546 | C | \|\| id:ebi-a-GCST90001966 | ebi-a-GCST90001966 | TRUE | A | 92223518 | 2.11E-11 | reported | 2850 | 0.03918 | rs12154498 |  |  |  |  |  |  |  |  |  |  |  |  |  |  |  |
| 0.3042 | 6 | igd | 0.8522 | T | \|\| id:ebi-a-GCST90002119 | ebi-a-GCST90002119 | TRUE | C | 31322121 | 2.22E-12 | reported | 2920 | 0.04314 | rs2428496 |  |  |  |  |  |  |  |  |  |  |  |  |  |  |  |
| 0.3757 | 19 | igd | 0.8521 | A | \|\| id:ebi-a-GCST90001954 | ebi-a-GCST90001954 | TRUE | G | 51531099 | 7.93E-14 | reported | 1555 | 0.04983 | rs2569457 |  |  |  |  |  |  |  |  |  |  |  |  |  |  |  |
| 0.377 | 19 | igd | 0.8517 | A | \|\| id:ebi-a-GCST90001953 | ebi-a-GCST90001953 | TRUE | G | 51531099 | 5.77E-14 | reported | 1558 | 0.04972 | rs2569457 |  |  |  |  |  |  |  |  |  |  |  |  |  |  |  |
| 0.3448 | 19 | igd | 0.8517 | A | \|\| id:ebi-a-GCST90001955 | ebi-a-GCST90001955 | TRUE | G | 51531099 | 8.11E-12 | reported | 1554 | 0.05005 | rs2569457 |  |  |  |  |  |  |  |  |  |  |  |  |  |  |  |
| -2.908 | 6 | igd | 0.8511 | T | \|\| id:ebi-a-GCST90001696 | ebi-a-GCST90001696 | TRUE | C | 31325380 | 1.38E-09 | reported | 3425 | 0.4786 | rs9266231 |  |  |  |  |  |  |  |  |  |  |  |  |  |  |  |
| 0.312 | 19 | igd | 0.8507 | A | \|\| id:ebi-a-GCST90001947 | ebi-a-GCST90001947 | TRUE | G | 51531099 | 7.23E-10 | reported | 1577 | 0.05032 | rs2569457 |  |  |  |  |  |  |  |  |  |  |  |  |  |  |  |
| 0.3139 | 19 | igd | 0.8505 | A | \|\| id:ebi-a-GCST90001952 | ebi-a-GCST90001952 | TRUE | G | 51531099 | 5.09E-10 | reported | 1569 | 0.05018 | rs2569457 |  |  |  |  |  |  |  |  |  |  |  |  |  |  |  |
| 0.3284 | 19 | igd | 0.8504 | A | \|\| id:ebi-a-GCST90001957 | ebi-a-GCST90001957 | TRUE | G | 51531099 | 7.40E-11 | reported | 1578 | 0.05008 | rs2569457 |  |  |  |  |  |  |  |  |  |  |  |  |  |  |  |
| 0.3246 | 19 | igd | 0.8502 | A | \|\| id:ebi-a-GCST90001956 | ebi-a-GCST90001956 | TRUE | G | 51531099 | 1.21E-10 | reported | 1579 | 0.05008 | rs2569457 |  |  |  |  |  |  |  |  |  |  |  |  |  |  |  |
| -0.244 | 10 | igd | 0.8496 | C | \|\| id:ebi-a-GCST90001497 | ebi-a-GCST90001497 | TRUE | T | 96795524 | 1.15E-11 | reported | 3437 | 0.03583 | rs7087256 |  |  |  |  |  |  |  |  |  |  |  |  |  |  |  |
| -0.2008 | 10 | igd | 0.8494 | A | \|\| id:ebi-a-GCST90001658 | ebi-a-GCST90001658 | TRUE | T | 96801805 | 7.64E-09 | reported | 3440 | 0.03467 | rs1934984 |  |  |  |  |  |  |  |  |  |  |  |  |  |  |  |
| -0.2167 | 10 | igd | 0.8494 | A | \|\| id:ebi-a-GCST90001659 | ebi-a-GCST90001659 | TRUE | T | 96801805 | 4.51E-10 | reported | 3440 | 0.03465 | rs1934984 |  |  |  |  |  |  |  |  |  |  |  |  |  |  |  |
| -0.2142 | 6 | igd | 0.8479 | G | \|\| id:ebi-a-GCST90001768 | ebi-a-GCST90001768 | TRUE | A | 106999132 | 4.36E-10 | reported | 3655 | 0.03423 | rs1676008 |  |  |  |  |  |  |  |  |  |  |  |  |  |  |  |
| -0.2027 | 6 | igd | 0.8478 | G | \|\| id:ebi-a-GCST90001773 | ebi-a-GCST90001773 | TRUE | A | 106999132 | 5.38E-09 | reported | 3652 | 0.03465 | rs1676008 |  |  |  |  |  |  |  |  |  |  |  |  |  |  |  |
| 0.3112 | 19 | igd | 0.8473 | A | \|\| id:ebi-a-GCST90001946 | ebi-a-GCST90001946 | TRUE | G | 51531099 | 1.56E-10 | reported | 1634 | 0.04831 | rs2569457 |  |  |  |  |  |  |  |  |  |  |  |  |  |  |  |
| 0.3096 | 19 | igd | 0.8473 | A | \|\| id:ebi-a-GCST90001949 | ebi-a-GCST90001949 | TRUE | G | 51531099 | 1.30E-10 | reported | 1634 | 0.04786 | rs2569457 |  |  |  |  |  |  |  |  |  |  |  |  |  |  |  |
| 0.3209 | 19 | igd | 0.8472 | A | \|\| id:ebi-a-GCST90001948 | ebi-a-GCST90001948 | TRUE | G | 51531099 | 2.96E-11 | reported | 1633 | 0.04793 | rs2569457 |  |  |  |  |  |  |  |  |  |  |  |  |  |  |  |
| 0.2146 | 2 | igd | 0.8432 | A | \|\| id:ebi-a-GCST90001896 | ebi-a-GCST90001896 | TRUE | G | 204687518 | 2.87E-08 | reported | 2920 | 0.03857 | rs231749 |  |  |  |  |  |  |  |  |  |  |  |  |  |  |  |
| -0.2308 | 10 | igd | 0.8392 | C | \|\| id:ebi-a-GCST90001888 | ebi-a-GCST90001888 | TRUE | G | 97383842 | 1.74E-10 | reported | 2920 | 0.03603 | rs2485643 |  |  |  |  |  |  |  |  |  |  |  |  |  |  |  |
| -0.3008 | 10 | igd | 0.8392 | C | \|\| id:ebi-a-GCST90001892 | ebi-a-GCST90001892 | TRUE | G | 97383842 | 3.00E-16 | reported | 2920 | 0.03659 | rs2485643 |  |  |  |  |  |  |  |  |  |  |  |  |  |  |  |
| -0.27 | 10 | igd | 0.8389 | C | \|\| id:ebi-a-GCST90002029 | ebi-a-GCST90002029 | TRUE | G | 97383842 | 1.11E-13 | reported | 2915 | 0.03618 | rs2485643 |  |  |  |  |  |  |  |  |  |  |  |  |  |  |  |
| 0.4322 | 10 | igd | 0.8387 | G | \|\| id:ebi-a-GCST90001961 | ebi-a-GCST90001961 | TRUE | A | 97366834 | 1.25E-31 | reported | 2920 | 0.0365 | rs496426 |  |  |  |  |  |  |  |  |  |  |  |  |  |  |  |
| -0.7765 | 10 | igd | 0.8387 | G | \|\| id:ebi-a-GCST90002030 | ebi-a-GCST90002030 | TRUE | A | 97366834 | 3.33E-104 | reported | 2920 | 0.03438 | rs496426 |  |  |  |  |  |  |  |  |  |  |  |  |  |  |  |
| -0.6504 | 10 | igd | 0.8387 | G | \|\| id:ebi-a-GCST90002031 | ebi-a-GCST90002031 | TRUE | A | 97366834 | 1.38E-69 | reported | 2920 | 0.03591 | rs496426 |  |  |  |  |  |  |  |  |  |  |  |  |  |  |  |
| -0.6085 | 10 | igd | 0.8387 | G | \|\| id:ebi-a-GCST90002032 | ebi-a-GCST90002032 | TRUE | A | 97366834 | 1.07E-60 | reported | 2920 | 0.03617 | rs496426 |  |  |  |  |  |  |  |  |  |  |  |  |  |  |  |
| -0.5893 | 10 | igd | 0.8382 | C | \|\| id:ebi-a-GCST90001489 | ebi-a-GCST90001489 | TRUE | G | 97383842 | 4.22E-65 | reported | 3405 | 0.03385 | rs2485643 |  |  |  |  |  |  |  |  |  |  |  |  |  |  |  |
| -0.6083 | 10 | igd | 0.8379 | G | \|\| id:ebi-a-GCST90001483 | ebi-a-GCST90001483 | TRUE | A | 97411725 | 1.93E-73 | reported | 3405 | 0.03275 | rs10882646 |  |  |  |  |  |  |  |  |  |  |  |  |  |  |  |
| -0.6918 | 10 | igd | 0.8379 | G | \|\| id:ebi-a-GCST90001660 | ebi-a-GCST90001660 | TRUE | A | 97366834 | 2.14E-90 | reported | 3408 | 0.03329 | rs496426 |  |  |  |  |  |  |  |  |  |  |  |  |  |  |  |
| -0.5975 | 10 | igd | 0.8379 | G | \|\| id:ebi-a-GCST90001672 | ebi-a-GCST90001672 | TRUE | A | 97366834 | 1.48E-65 | reported | 3408 | 0.03419 | rs496426 |  |  |  |  |  |  |  |  |  |  |  |  |  |  |  |
| -0.6396 | 10 | igd | 0.8377 | G | \|\| id:ebi-a-GCST90001495 | ebi-a-GCST90001495 | TRUE | A | 97366834 | 2.86E-75 | reported | 3405 | 0.03398 | rs496426 |  |  |  |  |  |  |  |  |  |  |  |  |  |  |  |
| -0.7066 | 10 | igd | 0.8376 | G | \|\| id:ebi-a-GCST90001658 | ebi-a-GCST90001658 | TRUE | A | 97366834 | 1.89E-104 | reported | 3440 | 0.03144 | rs496426 |  |  |  |  |  |  |  |  |  |  |  |  |  |  |  |
| -0.7086 | 10 | igd | 0.8376 | G | \|\| id:ebi-a-GCST90001659 | ebi-a-GCST90001659 | TRUE | A | 97366834 | 4.66E-105 | reported | 3440 | 0.03144 | rs496426 |  |  |  |  |  |  |  |  |  |  |  |  |  |  |  |
| -0.603 | 10 | igd | 0.8376 | G | \|\| id:ebi-a-GCST90001670 | ebi-a-GCST90001670 | TRUE | A | 97366834 | 1.76E-69 | reported | 3440 | 0.03345 | rs496426 |  |  |  |  |  |  |  |  |  |  |  |  |  |  |  |
| -0.6048 | 10 | igd | 0.8376 | G | \|\| id:ebi-a-GCST90001671 | ebi-a-GCST90001671 | TRUE | A | 97366834 | 1.72E-69 | reported | 3440 | 0.03355 | rs496426 |  |  |  |  |  |  |  |  |  |  |  |  |  |  |  |
| -0.6859 | 10 | igd | 0.8375 | G | \|\| id:ebi-a-GCST90001484 | ebi-a-GCST90001484 | TRUE | A | 97366834 | 3.97E-96 | reported | 3437 | 0.03193 | rs496426 |  |  |  |  |  |  |  |  |  |  |  |  |  |  |  |
| -0.6196 | 10 | igd | 0.8375 | G | \|\| id:ebi-a-GCST90001491 | ebi-a-GCST90001491 | TRUE | A | 97366834 | 3.91E-74 | reported | 3437 | 0.03319 | rs496426 |  |  |  |  |  |  |  |  |  |  |  |  |  |  |  |
| -0.6994 | 10 | igd | 0.8375 | G | \|\| id:ebi-a-GCST90001496 | ebi-a-GCST90001496 | TRUE | A | 97366834 | 1.27E-92 | reported | 3437 | 0.03323 | rs496426 |  |  |  |  |  |  |  |  |  |  |  |  |  |  |  |
| -0.6825 | 10 | igd | 0.8375 | G | \|\| id:ebi-a-GCST90001497 | ebi-a-GCST90001497 | TRUE | A | 97366834 | 2.38E-90 | reported | 3437 | 0.03286 | rs496426 |  |  |  |  |  |  |  |  |  |  |  |  |  |  |  |
| -0.4195 | 10 | igd | 0.8365 | A | \|\| id:ebi-a-GCST90001484 | ebi-a-GCST90001484 | TRUE | G | 97470019 | 6.77E-36 | reported | 3437 | 0.03316 | rs10786231 |  |  |  |  |  |  |  |  |  |  |  |  |  |  |  |
| 0.3448 | 22 | igd | 0.8361 | A | \|\| id:ebi-a-GCST90001702 | ebi-a-GCST90001702 | TRUE | G | 42970242 | 1.21E-23 | reported | 3657 | 0.03416 | rs137056 |  |  |  |  |  |  |  |  |  |  |  |  |  |  |  |
| 0.3453 | 22 | igd | 0.8361 | A | \|\| id:ebi-a-GCST90001703 | ebi-a-GCST90001703 | TRUE | G | 42970242 | 1.01E-23 | reported | 3657 | 0.03415 | rs137056 |  |  |  |  |  |  |  |  |  |  |  |  |  |  |  |
| 0.3214 | 22 | igd | 0.8361 | A | \|\| id:ebi-a-GCST90001704 | ebi-a-GCST90001704 | TRUE | G | 42970242 | 8.82E-21 | reported | 3657 | 0.03417 | rs137056 |  |  |  |  |  |  |  |  |  |  |  |  |  |  |  |
| 0.3296 | 22 | igd | 0.8361 | A | \|\| id:ebi-a-GCST90001710 | ebi-a-GCST90001710 | TRUE | G | 42970242 | 7.76E-22 | reported | 3657 | 0.03411 | rs137056 |  |  |  |  |  |  |  |  |  |  |  |  |  |  |  |
| 0.336 | 22 | igd | 0.8361 | A | \|\| id:ebi-a-GCST90001711 | ebi-a-GCST90001711 | TRUE | G | 42970242 | 9.56E-23 | reported | 3657 | 0.034 | rs137056 |  |  |  |  |  |  |  |  |  |  |  |  |  |  |  |
| 0.1925 | 22 | igd | 0.8328 | G | \|\| id:ebi-a-GCST90001704 | ebi-a-GCST90001704 | TRUE | T | 37777613 | 9.14E-09 | reported | 3657 | 0.03342 | rs4821656 |  |  |  |  |  |  |  |  |  |  |  |  |  |  |  |
| -0.3104 | 6 | igd | 0.8325 | A | \|\| id:ebi-a-GCST90002105 | ebi-a-GCST90002105 | TRUE | G | 29730848 | 5.42E-14 | reported | 2872 | 0.04106 | rs1611284 |  |  |  |  |  |  |  |  |  |  |  |  |  |  |  |
| -0.1833 | 1 | igd | 0.8318 | A | \|\| id:ebi-a-GCST90001982 | ebi-a-GCST90001982 | TRUE | G | 161408397 | 4.16E-08 | reported | 3621 | 0.03335 | rs6427592 |  |  |  |  |  |  |  |  |  |  |  |  |  |  |  |
| -0.4304 | 1 | igd | 0.8313 | A | \|\| id:ebi-a-GCST90001994 | ebi-a-GCST90001994 | TRUE | G | 161408397 | 1.34E-38 | reported | 3590 | 0.03274 | rs6427592 |  |  |  |  |  |  |  |  |  |  |  |  |  |  |  |
| -0.2945 | 6 | igd | 0.8308 | C | \|\| id:ebi-a-GCST90002110 | ebi-a-GCST90002110 | TRUE | T | 32120715 | 9.28E-09 | reported | 1634 | 0.051 | rs1053924 |  |  |  |  |  |  |  |  |  |  |  |  |  |  |  |
| 0.2153 | 22 | igd | 0.8301 | T | \|\| id:ebi-a-GCST90001702 | ebi-a-GCST90001702 | TRUE | G | 37781124 | 9.43E-11 | reported | 3657 | 0.03315 | rs738557 |  |  |  |  |  |  |  |  |  |  |  |  |  |  |  |
| 0.2095 | 22 | igd | 0.8301 | T | \|\| id:ebi-a-GCST90001703 | ebi-a-GCST90001703 | TRUE | G | 37781124 | 2.97E-10 | reported | 3657 | 0.03315 | rs738557 |  |  |  |  |  |  |  |  |  |  |  |  |  |  |  |
| 0.1965 | 22 | igd | 0.8301 | T | \|\| id:ebi-a-GCST90001710 | ebi-a-GCST90001710 | TRUE | G | 37781124 | 3.12E-09 | reported | 3657 | 0.03309 | rs738557 |  |  |  |  |  |  |  |  |  |  |  |  |  |  |  |
| 0.1992 | 22 | igd | 0.8301 | T | \|\| id:ebi-a-GCST90001711 | ebi-a-GCST90001711 | TRUE | G | 37781124 | 1.73E-09 | reported | 3657 | 0.033 | rs738557 |  |  |  |  |  |  |  |  |  |  |  |  |  |  |  |
| -0.2202 | 2 | igd | 0.8292 | T | \|\| id:ebi-a-GCST90001508 | ebi-a-GCST90001508 | TRUE | C | 39165054 | 1.55E-10 | reported | 3437 | 0.0343 | rs10203995 |  |  |  |  |  |  |  |  |  |  |  |  |  |  |  |
| -0.2058 | 2 | igd | 0.8292 | T | \|\| id:ebi-a-GCST90001509 | ebi-a-GCST90001509 | TRUE | C | 39165054 | 2.19E-09 | reported | 3437 | 0.0343 | rs10203995 |  |  |  |  |  |  |  |  |  |  |  |  |  |  |  |
| -0.1986 | 2 | igd | 0.8289 | T | \|\| id:ebi-a-GCST90001507 | ebi-a-GCST90001507 | TRUE | C | 39165054 | 1.47E-08 | reported | 3405 | 0.03498 | rs10203995 |  |  |  |  |  |  |  |  |  |  |  |  |  |  |  |
| -0.2291 | 3 | igd | 0.8288 | G | \|\| id:ebi-a-GCST90001997 | ebi-a-GCST90001997 | TRUE | T | 39246142 | 1.00E-11 | reported | 3590 | 0.03355 | rs2137581 |  |  |  |  |  |  |  |  |  |  |  |  |  |  |  |
| -0.2058 | 3 | igd | 0.8288 | G | \|\| id:ebi-a-GCST90001995 | ebi-a-GCST90001995 | TRUE | T | 39246142 | 7.79E-10 | reported | 3590 | 0.03339 | rs2137581 |  |  |  |  |  |  |  |  |  |  |  |  |  |  |  |
| 0.4384 | 21 | igd | 0.8262 | C | \|\| id:ebi-a-GCST90002095 | ebi-a-GCST90002095 | TRUE | T | 46328099 | 5.30E-20 | reported | 1559 | 0.04722 | rs760462 |  |  |  |  |  |  |  |  |  |  |  |  |  |  |  |
| 0.4516 | 21 | igd | 0.8258 | C | \|\| id:ebi-a-GCST90002096 | ebi-a-GCST90002096 | TRUE | T | 46328099 | 1.85E-21 | reported | 1556 | 0.04677 | rs760462 |  |  |  |  |  |  |  |  |  |  |  |  |  |  |  |
| -0.3148 | 1 | igd | 0.8239 | C | \|\| id:ebi-a-GCST90002013 | ebi-a-GCST90002013 | TRUE | T | 161538507 | 8.43E-18 | reported | 2870 | 0.03639 | rs61801003 |  |  |  |  |  |  |  |  |  |  |  |  |  |  |  |
| -0.2987 | 14 | igd | 0.823 | C | \|\| id:ebi-a-GCST90001821 | ebi-a-GCST90001821 | TRUE | T | 106198083 | 2.69E-15 | reported | 3656 | 0.03762 | rs4271507 |  |  |  |  |  |  |  |  |  |  |  |  |  |  |  |
| -0.2448 | 14 | igd | 0.8229 | C | \|\| id:ebi-a-GCST90001820 | ebi-a-GCST90001820 | TRUE | T | 106198083 | 1.05E-10 | reported | 3656 | 0.03778 | rs4271507 |  |  |  |  |  |  |  |  |  |  |  |  |  |  |  |
| -0.5603 | 10 | igd | 0.8228 | T | \|\| id:ebi-a-GCST90001485 | ebi-a-GCST90001485 | TRUE | C | 97349134 | 6.65E-67 | reported | 3437 | 0.03172 | rs549042 |  |  |  |  |  |  |  |  |  |  |  |  |  |  |  |
| -0.2701 | 1 | igd | 0.8228 | C | \|\| id:ebi-a-GCST90002014 | ebi-a-GCST90002014 | TRUE | G | 161894092 | 3.19E-13 | reported | 2870 | 0.03689 | rs6656937 |  |  |  |  |  |  |  |  |  |  |  |  |  |  |  |
| 0.187 | 6 | igd | 0.8211 | A | \|\| id:ebi-a-GCST90001748 | ebi-a-GCST90001748 | TRUE | G | 15880540 | 6.08E-09 | reported | 3645 | 0.03209 | rs2066399 |  |  |  |  |  |  |  |  |  |  |  |  |  |  |  |
| 0.2255 | 6 | igd | 0.821 | A | \|\| id:ebi-a-GCST90001747 | ebi-a-GCST90001747 | TRUE | G | 15880540 | 2.95E-12 | reported | 3657 | 0.03219 | rs2066399 |  |  |  |  |  |  |  |  |  |  |  |  |  |  |  |
| 0.3353 | 6 | igd | 0.821 | A | \|\| id:ebi-a-GCST90001751 | ebi-a-GCST90001751 | TRUE | G | 15880540 | 1.13E-25 | reported | 3657 | 0.03177 | rs2066399 |  |  |  |  |  |  |  |  |  |  |  |  |  |  |  |
| 0.2375 | 6 | igd | 0.821 | A | \|\| id:ebi-a-GCST90001752 | ebi-a-GCST90001752 | TRUE | G | 15880540 | 1.75E-13 | reported | 3657 | 0.03213 | rs2066399 |  |  |  |  |  |  |  |  |  |  |  |  |  |  |  |
| -0.2534 | 10 | igd | 0.821 | C | \|\| id:ebi-a-GCST90001777 | ebi-a-GCST90001777 | TRUE | A | 6165525 | 1.16E-15 | reported | 3657 | 0.03149 | rs4750000 |  |  |  |  |  |  |  |  |  |  |  |  |  |  |  |
| -0.2193 | 10 | igd | 0.821 | C | \|\| id:ebi-a-GCST90001778 | ebi-a-GCST90001778 | TRUE | A | 6165525 | 5.87E-12 | reported | 3657 | 0.03175 | rs4750000 |  |  |  |  |  |  |  |  |  |  |  |  |  |  |  |
| -0.2117 | 10 | igd | 0.821 | C | \|\| id:ebi-a-GCST90001787 | ebi-a-GCST90001787 | TRUE | A | 6165525 | 3.17E-11 | reported | 3657 | 0.03179 | rs4750000 |  |  |  |  |  |  |  |  |  |  |  |  |  |  |  |
| -0.2482 | 10 | igd | 0.821 | C | \|\| id:ebi-a-GCST90001789 | ebi-a-GCST90001789 | TRUE | A | 6165525 | 2.90E-15 | reported | 3657 | 0.0313 | rs4750000 |  |  |  |  |  |  |  |  |  |  |  |  |  |  |  |
| -0.2377 | 10 | igd | 0.821 | C | \|\| id:ebi-a-GCST90001790 | ebi-a-GCST90001790 | TRUE | A | 6165525 | 6.22E-14 | reported | 3657 | 0.03155 | rs4750000 |  |  |  |  |  |  |  |  |  |  |  |  |  |  |  |
| -0.2413 | 10 | igd | 0.821 | C | \|\| id:ebi-a-GCST90001792 | ebi-a-GCST90001792 | TRUE | A | 6165525 | 2.65E-14 | reported | 3657 | 0.03157 | rs4750000 |  |  |  |  |  |  |  |  |  |  |  |  |  |  |  |
| -0.2706 | 10 | igd | 0.821 | C | \|\| id:ebi-a-GCST90001793 | ebi-a-GCST90001793 | TRUE | A | 6165525 | 2.17E-17 | reported | 3657 | 0.03173 | rs4750000 |  |  |  |  |  |  |  |  |  |  |  |  |  |  |  |
| 0.1788 | 16 | igd | 0.8208 | A | \|\| id:ebi-a-GCST90001752 | ebi-a-GCST90001752 | TRUE | G | 11007193 | 4.23E-08 | reported | 3657 | 0.03256 | rs7404890 |  |  |  |  |  |  |  |  |  |  |  |  |  |  |  |
| 0.1943 | 10 | igd | 0.8199 | G | \|\| id:ebi-a-GCST90001892 | ebi-a-GCST90001892 | TRUE | A | 97678022 | 4.28E-08 | reported | 2920 | 0.03536 | rs4561132 |  |  |  |  |  |  |  |  |  |  |  |  |  |  |  |
| 0.3853 | 10 | igd | 0.8199 | T | \|\| id:ebi-a-GCST90002030 | ebi-a-GCST90002030 | TRUE | G | 97679469 | 2.05E-27 | reported | 2920 | 0.03516 | rs4300337 |  |  |  |  |  |  |  |  |  |  |  |  |  |  |  |
| 0.2832 | 10 | igd | 0.8199 | T | \|\| id:ebi-a-GCST90002032 | ebi-a-GCST90002032 | TRUE | G | 97679469 | 5.51E-15 | reported | 2920 | 0.03604 | rs4300337 |  |  |  |  |  |  |  |  |  |  |  |  |  |  |  |
| 0.2234 | 6 | igd | 0.8198 | G | \|\| id:ebi-a-GCST90001759 | ebi-a-GCST90001759 | TRUE | C | 15914349 | 2.53E-12 | reported | 3657 | 0.0318 | rs6910382 |  |  |  |  |  |  |  |  |  |  |  |  |  |  |  |
| 0.287 | 6 | igd | 0.8198 | G | \|\| id:ebi-a-GCST90001763 | ebi-a-GCST90001763 | TRUE | T | 15897007 | 6.36E-19 | reported | 3657 | 0.03212 | rs879299 |  |  |  |  |  |  |  |  |  |  |  |  |  |  |  |
| 0.2121 | 6 | igd | 0.8197 | G | \|\| id:ebi-a-GCST90001743 | ebi-a-GCST90001743 | TRUE | T | 15913304 | 3.02E-11 | reported | 3660 | 0.03182 | rs12212931 |  |  |  |  |  |  |  |  |  |  |  |  |  |  |  |
| 0.2233 | 6 | igd | 0.8197 | G | \|\| id:ebi-a-GCST90001762 | ebi-a-GCST90001762 | TRUE | T | 15913304 | 2.33E-12 | reported | 3658 | 0.03173 | rs12212931 |  |  |  |  |  |  |  |  |  |  |  |  |  |  |  |
| 0.1924 | 6 | igd | 0.8194 | A | \|\| id:ebi-a-GCST90001754 | ebi-a-GCST90001754 | TRUE | G | 15898371 | 2.24E-09 | reported | 3657 | 0.0321 | rs4716010 |  |  |  |  |  |  |  |  |  |  |  |  |  |  |  |
| 0.286 | 10 | igd | 0.8166 | G | \|\| id:ebi-a-GCST90001489 | ebi-a-GCST90001489 | TRUE | A | 97678022 | 1.01E-17 | reported | 3405 | 0.03318 | rs4561132 |  |  |  |  |  |  |  |  |  |  |  |  |  |  |  |
| 0.3234 | 10 | igd | 0.8166 | G | \|\| id:ebi-a-GCST90001495 | ebi-a-GCST90001495 | TRUE | A | 97678022 | 9.09E-22 | reported | 3405 | 0.03351 | rs4561132 |  |  |  |  |  |  |  |  |  |  |  |  |  |  |  |
| 0.338 | 10 | igd | 0.8166 | G | \|\| id:ebi-a-GCST90001660 | ebi-a-GCST90001660 | TRUE | A | 97678022 | 4.13E-24 | reported | 3408 | 0.03312 | rs4561132 |  |  |  |  |  |  |  |  |  |  |  |  |  |  |  |
| 0.3257 | 10 | igd | 0.8163 | G | \|\| id:ebi-a-GCST90001491 | ebi-a-GCST90001491 | TRUE | A | 97678022 | 4.16E-23 | reported | 3437 | 0.03266 | rs4561132 |  |  |  |  |  |  |  |  |  |  |  |  |  |  |  |
| 0.3686 | 10 | igd | 0.8163 | G | \|\| id:ebi-a-GCST90001496 | ebi-a-GCST90001496 | TRUE | A | 97678022 | 1.68E-28 | reported | 3437 | 0.03298 | rs4561132 |  |  |  |  |  |  |  |  |  |  |  |  |  |  |  |
| 0.3684 | 10 | igd | 0.8163 | G | \|\| id:ebi-a-GCST90001497 | ebi-a-GCST90001497 | TRUE | A | 97678022 | 3.59E-29 | reported | 3437 | 0.03255 | rs4561132 |  |  |  |  |  |  |  |  |  |  |  |  |  |  |  |
| 0.3295 | 10 | igd | 0.8163 | G | \|\| id:ebi-a-GCST90001658 | ebi-a-GCST90001658 | TRUE | A | 97678022 | 3.94E-25 | reported | 3440 | 0.03157 | rs4561132 |  |  |  |  |  |  |  |  |  |  |  |  |  |  |  |
| 0.3462 | 10 | igd | 0.8163 | G | \|\| id:ebi-a-GCST90001659 | ebi-a-GCST90001659 | TRUE | A | 97678022 | 1.28E-27 | reported | 3440 | 0.03152 | rs4561132 |  |  |  |  |  |  |  |  |  |  |  |  |  |  |  |
| 0.3111 | 19 | igd | 0.8156 | C | \|\| id:ebi-a-GCST90001951 | ebi-a-GCST90001951 | TRUE | T | 51473302 | 1.72E-10 | reported | 1464 | 0.04838 | rs11671058 |  |  |  |  |  |  |  |  |  |  |  |  |  |  |  |
| 2.878 | 2 | igd | 0.8145 | A | \|\| id:ebi-a-GCST90001696 | ebi-a-GCST90001696 | TRUE | C | 39177320 | 3.55E-13 | reported | 3425 | 0.3943 | rs408686 |  |  |  |  |  |  |  |  |  |  |  |  |  |  |  |
| 0.9033 | 2 | igd | 0.8145 | A | \|\| id:ebi-a-GCST90001697 | ebi-a-GCST90001697 | TRUE | C | 39177320 | 2.45E-08 | reported | 3425 | 0.1616 | rs408686 |  |  |  |  |  |  |  |  |  |  |  |  |  |  |  |
| 0.1772 | 6 | igd | 0.8127 | G | \|\| id:ebi-a-GCST90001751 | ebi-a-GCST90001751 | TRUE | A | 15851113 | 1.97E-08 | reported | 3657 | 0.03149 | rs220926 |  |  |  |  |  |  |  |  |  |  |  |  |  |  |  |
| -0.2313 | 10 | igd | 0.8122 | C | \|\| id:ebi-a-GCST90001782 | ebi-a-GCST90001782 | TRUE | A | 6165525 | 4.75E-08 | reported | 1837 | 0.04217 | rs4750000 |  |  |  |  |  |  |  |  |  |  |  |  |  |  |  |
| -2.802 | 2 | igd | 0.8117 | A | \|\| id:ebi-a-GCST90001696 | ebi-a-GCST90001696 | TRUE | C | 38884563 | 1.11E-12 | reported | 3425 | 0.3922 | rs6704666 |  |  |  |  |  |  |  |  |  |  |  |  |  |  |  |
| -0.9524 | 2 | igd | 0.8117 | A | \|\| id:ebi-a-GCST90001697 | ebi-a-GCST90001697 | TRUE | C | 38884563 | 3.33E-09 | reported | 3425 | 0.1606 | rs6704666 |  |  |  |  |  |  |  |  |  |  |  |  |  |  |  |
| 0.187 | 2 | igd | 0.8115 | A | \|\| id:ebi-a-GCST90001561 | ebi-a-GCST90001561 | TRUE | C | 38884563 | 2.21E-11 | reported | 3427 | 0.02786 | rs6704666 |  |  |  |  |  |  |  |  |  |  |  |  |  |  |  |
| -0.2651 | 12 | igd | 0.8109 | T | \|\| id:ebi-a-GCST90002074 | ebi-a-GCST90002074 | TRUE | C | 69747177 | 2.96E-15 | reported | 3112 | 0.03341 | rs710794 |  |  |  |  |  |  |  |  |  |  |  |  |  |  |  |
| 0.2825 | 2 | igd | 0.8103 | A | \|\| id:ebi-a-GCST90002098 | ebi-a-GCST90002098 | TRUE | C | 38884563 | 3.74E-17 | reported | 2910 | 0.03334 | rs6704666 |  |  |  |  |  |  |  |  |  |  |  |  |  |  |  |
| -0.3265 | 12 | igd | 0.8102 | T | \|\| id:ebi-a-GCST90002073 | ebi-a-GCST90002073 | TRUE | C | 69747177 | 6.03E-21 | reported | 2850 | 0.0345 | rs710794 |  |  |  |  |  |  |  |  |  |  |  |  |  |  |  |
| -0.4719 | 19 | igd | 0.8051 | G | \|\| id:ebi-a-GCST90001949 | ebi-a-GCST90001949 | TRUE | C | 51644922 | 6.68E-26 | reported | 1634 | 0.04408 | rs1628793 |  |  |  |  |  |  |  |  |  |  |  |  |  |  |  |
| -0.489 | 19 | igd | 0.805 | G | \|\| id:ebi-a-GCST90001948 | ebi-a-GCST90001948 | TRUE | C | 51644922 | 1.04E-27 | reported | 1633 | 0.044 | rs1628793 |  |  |  |  |  |  |  |  |  |  |  |  |  |  |  |
| 0.2518 | 1 | igd | 0.8048 | C | \|\| id:ebi-a-GCST90001471 | ebi-a-GCST90001471 | TRUE | T | 161408369 | 1.27E-15 | reported | 3410 | 0.03133 | rs6427591 |  |  |  |  |  |  |  |  |  |  |  |  |  |  |  |
| -0.3388 | 6 | igd | 0.8048 | G | \|\| id:ebi-a-GCST90002110 | ebi-a-GCST90002110 | TRUE | A | 31919578 | 9.10E-12 | reported | 1634 | 0.04931 | rs2072633 |  |  |  |  |  |  |  |  |  |  |  |  |  |  |  |
| -0.2935 | 6 | igd | 0.8046 | G | \|\| id:ebi-a-GCST90002111 | ebi-a-GCST90002111 | TRUE | A | 31919578 | 2.03E-09 | reported | 1635 | 0.04868 | rs2072633 |  |  |  |  |  |  |  |  |  |  |  |  |  |  |  |
| -0.3124 | 6 | igd | 0.8046 | G | \|\| id:ebi-a-GCST90002112 | ebi-a-GCST90002112 | TRUE | A | 31919578 | 2.74E-10 | reported | 1635 | 0.04918 | rs2072633 |  |  |  |  |  |  |  |  |  |  |  |  |  |  |  |
| -0.3205 | 6 | igd | 0.804 | G | \|\| id:ebi-a-GCST90002105 | ebi-a-GCST90002105 | TRUE | A | 31919578 | 2.19E-16 | reported | 2872 | 0.03881 | rs2072633 |  |  |  |  |  |  |  |  |  |  |  |  |  |  |  |
| 0.4629 | 1 | igd | 0.8038 | G | \|\| id:ebi-a-GCST90001474 | ebi-a-GCST90001474 | TRUE | A | 161410458 | 5.20E-48 | reported | 3410 | 0.0313 | rs12731669 |  |  |  |  |  |  |  |  |  |  |  |  |  |  |  |
| 0.2324 | 1 | igd | 0.8033 | C | \|\| id:ebi-a-GCST90001963 | ebi-a-GCST90001963 | TRUE | T | 161408369 | 1.90E-11 | reported | 2872 | 0.03447 | rs6427591 |  |  |  |  |  |  |  |  |  |  |  |  |  |  |  |
| -0.227 | 22 | igd | 0.803 | T | \|\| id:ebi-a-GCST90001702 | ebi-a-GCST90001702 | TRUE | C | 42087276 | 3.22E-12 | reported | 3657 | 0.03247 | rs5758418 |  |  |  |  |  |  |  |  |  |  |  |  |  |  |  |
| -0.2273 | 22 | igd | 0.803 | T | \|\| id:ebi-a-GCST90001703 | ebi-a-GCST90001703 | TRUE | C | 42087276 | 3.01E-12 | reported | 3657 | 0.03246 | rs5758418 |  |  |  |  |  |  |  |  |  |  |  |  |  |  |  |
| -0.21 | 22 | igd | 0.803 | T | \|\| id:ebi-a-GCST90001704 | ebi-a-GCST90001704 | TRUE | C | 42087276 | 1.09E-10 | reported | 3657 | 0.03245 | rs5758418 |  |  |  |  |  |  |  |  |  |  |  |  |  |  |  |
| -0.2108 | 22 | igd | 0.803 | T | \|\| id:ebi-a-GCST90001710 | ebi-a-GCST90001710 | TRUE | C | 42087276 | 8.84E-11 | reported | 3657 | 0.03241 | rs5758418 |  |  |  |  |  |  |  |  |  |  |  |  |  |  |  |
| -0.2063 | 22 | igd | 0.803 | T | \|\| id:ebi-a-GCST90001711 | ebi-a-GCST90001711 | TRUE | C | 42087276 | 1.99E-10 | reported | 3657 | 0.03233 | rs5758418 |  |  |  |  |  |  |  |  |  |  |  |  |  |  |  |
| -0.3402 | 6 | igd | 0.8024 | G | \|\| id:ebi-a-GCST90002108 | ebi-a-GCST90002108 | TRUE | A | 31919578 | 6.97E-12 | reported | 1579 | 0.04923 | rs2072633 |  |  |  |  |  |  |  |  |  |  |  |  |  |  |  |
| -0.1914 | 10 | igd | 0.8017 | C | \|\| id:ebi-a-GCST90001671 | ebi-a-GCST90001671 | TRUE | G | 97296291 | 2.94E-09 | reported | 3440 | 0.03217 | rs1409708 |  |  |  |  |  |  |  |  |  |  |  |  |  |  |  |
| -0.2349 | 6 | igd | 0.801 | C | \|\| id:ebi-a-GCST90002071 | ebi-a-GCST90002071 | TRUE | T | 32577385 | 6.73E-10 | reported | 2872 | 0.03793 | rs9271147 |  |  |  |  |  |  |  |  |  |  |  |  |  |  |  |
| -0.3181 | 19 | igd | 0.7999 | C | \|\| id:ebi-a-GCST90001951 | ebi-a-GCST90001951 | TRUE | T | 51672114 | 2.63E-11 | reported | 1464 | 0.04735 | rs273655 |  |  |  |  |  |  |  |  |  |  |  |  |  |  |  |
| 0.4323 | 1 | igd | 0.7999 | G | \|\| id:ebi-a-GCST90001999 | ebi-a-GCST90001999 | TRUE | A | 161410458 | 1.25E-43 | reported | 3621 | 0.03079 | rs12731669 |  |  |  |  |  |  |  |  |  |  |  |  |  |  |  |
| 0.3669 | 19 | igd | 0.7997 | A | \|\| id:ebi-a-GCST90001954 | ebi-a-GCST90001954 | TRUE | G | 51475818 | 1.51E-15 | reported | 1555 | 0.04552 | rs2739418 |  |  |  |  |  |  |  |  |  |  |  |  |  |  |  |
| 0.3569 | 19 | igd | 0.7992 | A | \|\| id:ebi-a-GCST90001955 | ebi-a-GCST90001955 | TRUE | G | 51475818 | 9.73E-15 | reported | 1554 | 0.04565 | rs2739418 |  |  |  |  |  |  |  |  |  |  |  |  |  |  |  |
| 0.1722 | 10 | igd | 0.7991 | G | \|\| id:ebi-a-GCST90001491 | ebi-a-GCST90001491 | TRUE | A | 100100503 | 4.72E-08 | reported | 3437 | 0.03145 | rs10748726 |  |  |  |  |  |  |  |  |  |  |  |  |  |  |  |
| 0.1678 | 10 | igd | 0.7991 | G | \|\| id:ebi-a-GCST90001658 | ebi-a-GCST90001658 | TRUE | A | 100100503 | 3.54E-08 | reported | 3440 | 0.03037 | rs10748726 |  |  |  |  |  |  |  |  |  |  |  |  |  |  |  |
| 0.3544 | 19 | igd | 0.7991 | A | \|\| id:ebi-a-GCST90001953 | ebi-a-GCST90001953 | TRUE | G | 51475818 | 1.23E-14 | reported | 1558 | 0.0455 | rs2739418 |  |  |  |  |  |  |  |  |  |  |  |  |  |  |  |
| -0.4683 | 19 | igd | 0.7989 | G | \|\| id:ebi-a-GCST90001956 | ebi-a-GCST90001956 | TRUE | C | 51644922 | 1.73E-24 | reported | 1579 | 0.04509 | rs1628793 |  |  |  |  |  |  |  |  |  |  |  |  |  |  |  |
| -0.4682 | 19 | igd | 0.7988 | G | \|\| id:ebi-a-GCST90001957 | ebi-a-GCST90001957 | TRUE | C | 51644922 | 2.10E-24 | reported | 1578 | 0.04516 | rs1628793 |  |  |  |  |  |  |  |  |  |  |  |  |  |  |  |
| -0.4544 | 19 | igd | 0.7987 | G | \|\| id:ebi-a-GCST90001947 | ebi-a-GCST90001947 | TRUE | C | 51644922 | 4.85E-23 | reported | 1577 | 0.04525 | rs1628793 |  |  |  |  |  |  |  |  |  |  |  |  |  |  |  |
| 0.1825 | 10 | igd | 0.7985 | G | \|\| id:ebi-a-GCST90001489 | ebi-a-GCST90001489 | TRUE | A | 100100503 | 1.14E-08 | reported | 3405 | 0.03189 | rs10748726 |  |  |  |  |  |  |  |  |  |  |  |  |  |  |  |
| -0.2333 | 6 | igd | 0.7985 | A | \|\| id:ebi-a-GCST90001692 | ebi-a-GCST90001692 | TRUE | G | 32581515 | 1.24E-10 | reported | 3440 | 0.03615 | rs3129750 |  |  |  |  |  |  |  |  |  |  |  |  |  |  |  |
| 0.3864 | 19 | igd | 0.7983 | A | \|\| id:ebi-a-GCST90001952 | ebi-a-GCST90001952 | TRUE | G | 51475818 | 5.10E-17 | reported | 1569 | 0.04556 | rs2739418 |  |  |  |  |  |  |  |  |  |  |  |  |  |  |  |
| -0.3883 | 19 | igd | 0.7983 | G | \|\| id:ebi-a-GCST90001952 | ebi-a-GCST90001952 | TRUE | C | 51644922 | 3.92E-17 | reported | 1569 | 0.04561 | rs1628793 |  |  |  |  |  |  |  |  |  |  |  |  |  |  |  |
| -0.4937 | 19 | igd | 0.7965 | G | \|\| id:ebi-a-GCST90001953 | ebi-a-GCST90001953 | TRUE | C | 51644922 | 3.29E-27 | reported | 1558 | 0.04483 | rs1628793 |  |  |  |  |  |  |  |  |  |  |  |  |  |  |  |
| -0.5001 | 19 | igd | 0.7961 | G | \|\| id:ebi-a-GCST90001954 | ebi-a-GCST90001954 | TRUE | C | 51644922 | 7.22E-28 | reported | 1555 | 0.04481 | rs1628793 |  |  |  |  |  |  |  |  |  |  |  |  |  |  |  |
| -0.5041 | 19 | igd | 0.796 | G | \|\| id:ebi-a-GCST90001955 | ebi-a-GCST90001955 | TRUE | C | 51644922 | 2.69E-28 | reported | 1554 | 0.04479 | rs1628793 |  |  |  |  |  |  |  |  |  |  |  |  |  |  |  |
| 0.3955 | 19 | igd | 0.7952 | A | \|\| id:ebi-a-GCST90001947 | ebi-a-GCST90001947 | TRUE | G | 51475818 | 4.01E-18 | reported | 1577 | 0.04502 | rs2739418 |  |  |  |  |  |  |  |  |  |  |  |  |  |  |  |
| 0.4057 | 19 | igd | 0.795 | A | \|\| id:ebi-a-GCST90001957 | ebi-a-GCST90001957 | TRUE | G | 51475818 | 4.26E-19 | reported | 1578 | 0.04486 | rs2739418 |  |  |  |  |  |  |  |  |  |  |  |  |  |  |  |
| 0.4042 | 19 | igd | 0.7948 | A | \|\| id:ebi-a-GCST90001956 | ebi-a-GCST90001956 | TRUE | G | 51475818 | 5.67E-19 | reported | 1579 | 0.04485 | rs2739418 |  |  |  |  |  |  |  |  |  |  |  |  |  |  |  |
| -0.4596 | 19 | igd | 0.7941 | T | \|\| id:ebi-a-GCST90001946 | ebi-a-GCST90001946 | TRUE | C | 51653609 | 3.38E-25 | reported | 1634 | 0.04358 | rs182127 |  |  |  |  |  |  |  |  |  |  |  |  |  |  |  |
| 0.4279 | 19 | igd | 0.7898 | A | \|\| id:ebi-a-GCST90001946 | ebi-a-GCST90001946 | TRUE | G | 51475818 | 2.26E-22 | reported | 1634 | 0.04334 | rs2739418 |  |  |  |  |  |  |  |  |  |  |  |  |  |  |  |
| 0.3888 | 19 | igd | 0.7898 | A | \|\| id:ebi-a-GCST90001949 | ebi-a-GCST90001949 | TRUE | G | 51475818 | 6.13E-19 | reported | 1634 | 0.0432 | rs2739418 |  |  |  |  |  |  |  |  |  |  |  |  |  |  |  |
| 0.4178 | 19 | igd | 0.7897 | A | \|\| id:ebi-a-GCST90001948 | ebi-a-GCST90001948 | TRUE | G | 51475818 | 1.40E-21 | reported | 1633 | 0.04317 | rs2739418 |  |  |  |  |  |  |  |  |  |  |  |  |  |  |  |
| 0.2234 | 6 | igd | 0.7896 | G | \|\| id:ebi-a-GCST90002119 | ebi-a-GCST90002119 | TRUE | T | 31496776 | 3.64E-09 | reported | 2920 | 0.03776 | rs3130054 |  |  |  |  |  |  |  |  |  |  |  |  |  |  |  |
| -0.2465 | 19 | igd | 0.7888 | A | \|\| id:ebi-a-GCST90001531 | ebi-a-GCST90001531 | TRUE | G | 51655950 | 1.82E-09 | reported | 1856 | 0.04079 | rs273660 |  |  |  |  |  |  |  |  |  |  |  |  |  |  |  |
| -0.1846 | 10 | igd | 0.7883 | A | \|\| id:ebi-a-GCST90001672 | ebi-a-GCST90001672 | TRUE | G | 99366733 | 6.61E-09 | reported | 3408 | 0.03174 | rs4638247 |  |  |  |  |  |  |  |  |  |  |  |  |  |  |  |
| -0.2063 | 6 | igd | 0.7872 | C | \|\| id:ebi-a-GCST90001916 | ebi-a-GCST90001916 | TRUE | T | 32600003 | 2.25E-09 | reported | 3113 | 0.03441 | rs3104376 |  |  |  |  |  |  |  |  |  |  |  |  |  |  |  |
| -0.215 | 2 | igd | 0.7872 | A | \|\| id:ebi-a-GCST90002119 | ebi-a-GCST90002119 | TRUE | G | 86857147 | 4.15E-11 | reported | 2920 | 0.03245 | rs308913 |  |  |  |  |  |  |  |  |  |  |  |  |  |  |  |
| -0.2282 | 10 | igd | 0.7863 | C | \|\| id:ebi-a-GCST90002030 | ebi-a-GCST90002030 | TRUE | T | 96928702 | 4.62E-12 | reported | 2920 | 0.03285 | rs2478477 |  |  |  |  |  |  |  |  |  |  |  |  |  |  |  |
| -0.2259 | 10 | igd | 0.7863 | C | \|\| id:ebi-a-GCST90002031 | ebi-a-GCST90002031 | TRUE | T | 96928702 | 2.19E-11 | reported | 2920 | 0.03362 | rs2478477 |  |  |  |  |  |  |  |  |  |  |  |  |  |  |  |
| -0.2332 | 10 | igd | 0.7863 | C | \|\| id:ebi-a-GCST90002032 | ebi-a-GCST90002032 | TRUE | T | 96928702 | 4.64E-12 | reported | 2920 | 0.03358 | rs2478477 |  |  |  |  |  |  |  |  |  |  |  |  |  |  |  |
| 0.2271 | 6 | igd | 0.7824 | A | \|\| id:ebi-a-GCST90001820 | ebi-a-GCST90001820 | TRUE | G | 32765182 | 1.63E-10 | reported | 3656 | 0.03543 | rs7767167 |  |  |  |  |  |  |  |  |  |  |  |  |  |  |  |
| -0.2743 | 6 | igd | 0.7815 | G | \|\| id:ebi-a-GCST90002108 | ebi-a-GCST90002108 | TRUE | A | 29745439 | 1.04E-08 | reported | 1579 | 0.04767 | rs2394685 |  |  |  |  |  |  |  |  |  |  |  |  |  |  |  |
| 0.1769 | 4 | igd | 0.7804 | G | \|\| id:ebi-a-GCST90001471 | ebi-a-GCST90001471 | TRUE | A | 82644251 | 6.53E-09 | reported | 3410 | 0.03041 | rs11931035 |  |  |  |  |  |  |  |  |  |  |  |  |  |  |  |
| 0.1979 | 6 | igd | 0.78 | T | \|\| id:ebi-a-GCST90001684 | ebi-a-GCST90001684 | TRUE | C | 32596309 | 3.87E-08 | reported | 3371 | 0.03593 | rs2036202 |  |  |  |  |  |  |  |  |  |  |  |  |  |  |  |
| -0.3006 | 6 | igd | 0.7779 | C | \|\| id:ebi-a-GCST90002105 | ebi-a-GCST90002105 | TRUE | T | 29929073 | 6.91E-16 | reported | 2872 | 0.03703 | rs2517690 |  |  |  |  |  |  |  |  |  |  |  |  |  |  |  |
| 0.3049 | 6 | igd | 0.7774 | T | \|\| id:ebi-a-GCST90001896 | ebi-a-GCST90001896 | TRUE | C | 90981653 | 4.57E-20 | reported | 2920 | 0.033 | rs9111 |  |  |  |  |  |  |  |  |  |  |  |  |  |  |  |
| -0.2591 | 6 | igd | 0.7772 | T | \|\| id:ebi-a-GCST90002081 | ebi-a-GCST90002081 | TRUE | A | 32589978 | 8.85E-14 | reported | 3113 | 0.03458 | rs9271536 |  |  |  |  |  |  |  |  |  |  |  |  |  |  |  |
| -0.1654 | 9 | igd | 0.776 | T | \|\| id:ebi-a-GCST90001775 | ebi-a-GCST90001775 | TRUE | C | 95886051 | 2.19E-08 | reported | 3660 | 0.0295 | rs3793662 |  |  |  |  |  |  |  |  |  |  |  |  |  |  |  |
| -0.1594 | 9 | igd | 0.7759 | T | \|\| id:ebi-a-GCST90001777 | ebi-a-GCST90001777 | TRUE | C | 95886051 | 3.75E-08 | reported | 3657 | 0.0289 | rs3793662 |  |  |  |  |  |  |  |  |  |  |  |  |  |  |  |
| -0.1898 | 9 | igd | 0.7759 | T | \|\| id:ebi-a-GCST90001779 | ebi-a-GCST90001779 | TRUE | C | 95886051 | 1.11E-10 | reported | 3657 | 0.02934 | rs3793662 |  |  |  |  |  |  |  |  |  |  |  |  |  |  |  |
| -0.1749 | 9 | igd | 0.7759 | T | \|\| id:ebi-a-GCST90001780 | ebi-a-GCST90001780 | TRUE | C | 95886051 | 2.38E-09 | reported | 3642 | 0.02923 | rs3793662 |  |  |  |  |  |  |  |  |  |  |  |  |  |  |  |
| -0.1698 | 9 | igd | 0.7759 | T | \|\| id:ebi-a-GCST90001789 | ebi-a-GCST90001789 | TRUE | C | 95886051 | 3.69E-09 | reported | 3657 | 0.02871 | rs3793662 |  |  |  |  |  |  |  |  |  |  |  |  |  |  |  |
| -0.1626 | 9 | igd | 0.7759 | T | \|\| id:ebi-a-GCST90001790 | ebi-a-GCST90001790 | TRUE | C | 95886051 | 2.05E-08 | reported | 3657 | 0.02894 | rs3793662 |  |  |  |  |  |  |  |  |  |  |  |  |  |  |  |
| -0.1953 | 9 | igd | 0.7759 | T | \|\| id:ebi-a-GCST90001791 | ebi-a-GCST90001791 | TRUE | C | 95886051 | 4.56E-11 | reported | 3657 | 0.02956 | rs3793662 |  |  |  |  |  |  |  |  |  |  |  |  |  |  |  |
| -0.167 | 9 | igd | 0.7759 | T | \|\| id:ebi-a-GCST90001792 | ebi-a-GCST90001792 | TRUE | C | 95886051 | 8.76E-09 | reported | 3657 | 0.02895 | rs3793662 |  |  |  |  |  |  |  |  |  |  |  |  |  |  |  |
| -0.1813 | 9 | igd | 0.7758 | T | \|\| id:ebi-a-GCST90001783 | ebi-a-GCST90001783 | TRUE | C | 95886051 | 4.38E-10 | reported | 3656 | 0.02898 | rs3793662 |  |  |  |  |  |  |  |  |  |  |  |  |  |  |  |
| 0.2608 | 6 | igd | 0.7752 | A | \|\| id:ebi-a-GCST90001932 | ebi-a-GCST90001932 | TRUE | G | 32594275 | 2.23E-13 | reported | 2920 | 0.0354 | rs9271772 |  |  |  |  |  |  |  |  |  |  |  |  |  |  |  |
| -0.1794 | 22 | igd | 0.774 | T | \|\| id:ebi-a-GCST90001702 | ebi-a-GCST90001702 | TRUE | C | 39258078 | 2.36E-09 | reported | 3657 | 0.02997 | rs733107 |  |  |  |  |  |  |  |  |  |  |  |  |  |  |  |
| 0.348 | 6 | igd | 0.7737 | A | \|\| id:ebi-a-GCST90002112 | ebi-a-GCST90002112 | TRUE | G | 32765182 | 2.66E-12 | reported | 1635 | 0.04937 | rs7767167 |  |  |  |  |  |  |  |  |  |  |  |  |  |  |  |
| -0.2179 | 6 | igd | 0.7734 | G | \|\| id:ebi-a-GCST90002027 | ebi-a-GCST90002027 | TRUE | C | 33098896 | 2.34E-10 | reported | 2911 | 0.03427 | rs3129267 |  |  |  |  |  |  |  |  |  |  |  |  |  |  |  |
| 0.3881 | 6 | igd | 0.7733 | A | \|\| id:ebi-a-GCST90002108 | ebi-a-GCST90002108 | TRUE | G | 32765182 | 7.88E-15 | reported | 1579 | 0.04947 | rs7767167 |  |  |  |  |  |  |  |  |  |  |  |  |  |  |  |
| 0.1747 | 6 | igd | 0.7732 | C | \|\| id:ebi-a-GCST90001560 | ebi-a-GCST90001560 | TRUE | T | 32602482 | 1.22E-09 | reported | 3395 | 0.02866 | rs3104369 |  |  |  |  |  |  |  |  |  |  |  |  |  |  |  |
| -0.2187 | 9 | igd | 0.7711 | T | \|\| id:ebi-a-GCST90001781 | ebi-a-GCST90001781 | TRUE | G | 95887320 | 4.81E-08 | reported | 1835 | 0.0399 | rs2275848 |  |  |  |  |  |  |  |  |  |  |  |  |  |  |  |
| -0.1919 | 16 | igd | 0.7709 | A | \|\| id:ebi-a-GCST90001920 | ebi-a-GCST90001920 | TRUE | G | 10973612 | 6.18E-10 | reported | 3060 | 0.03092 | rs72770017 |  |  |  |  |  |  |  |  |  |  |  |  |  |  |  |
| 0.2395 | 6 | igd | 0.7693 | T | \|\| id:ebi-a-GCST90001692 | ebi-a-GCST90001692 | TRUE | C | 31322911 | 8.82E-13 | reported | 3440 | 0.03338 | rs1051488 |  |  |  |  |  |  |  |  |  |  |  |  |  |  |  |
| -0.1833 | 6 | igd | 0.7692 | G | \|\| id:ebi-a-GCST90001973 | ebi-a-GCST90001973 | TRUE | A | 32423237 | 1.39E-08 | reported | 3113 | 0.03222 | rs9268803 |  |  |  |  |  |  |  |  |  |  |  |  |  |  |  |
| -0.1952 | 6 | igd | 0.769 | T | \|\| id:ebi-a-GCST90001916 | ebi-a-GCST90001916 | TRUE | G | 32425487 | 5.37E-09 | reported | 3113 | 0.03336 | rs7744304 |  |  |  |  |  |  |  |  |  |  |  |  |  |  |  |
| -0.2265 | 6 | igd | 0.769 | T | \|\| id:ebi-a-GCST90002081 | ebi-a-GCST90002081 | TRUE | G | 32425487 | 1.21E-11 | reported | 3113 | 0.03328 | rs7744304 |  |  |  |  |  |  |  |  |  |  |  |  |  |  |  |
| 0.1996 | 19 | igd | 0.7669 | T | \|\| id:ebi-a-GCST90001743 | ebi-a-GCST90001743 | TRUE | G | 49841054 | 1.12E-11 | reported | 3660 | 0.0293 | rs354026 |  |  |  |  |  |  |  |  |  |  |  |  |  |  |  |
| 0.2682 | 19 | igd | 0.7669 | T | \|\| id:ebi-a-GCST90001954 | ebi-a-GCST90001954 | TRUE | C | 51548032 | 6.65E-10 | reported | 1555 | 0.04316 | rs11084048 |  |  |  |  |  |  |  |  |  |  |  |  |  |  |  |
| 0.252 | 6 | igd | 0.7669 | G | \|\| id:ebi-a-GCST90002105 | ebi-a-GCST90002105 | TRUE | A | 32545293 | 3.88E-09 | reported | 2872 | 0.04265 | rs9269614 |  |  |  |  |  |  |  |  |  |  |  |  |  |  |  |
| 0.2604 | 19 | igd | 0.7667 | T | \|\| id:ebi-a-GCST90001953 | ebi-a-GCST90001953 | TRUE | C | 51548032 | 1.99E-09 | reported | 1558 | 0.04316 | rs11084048 |  |  |  |  |  |  |  |  |  |  |  |  |  |  |  |
| 0.2395 | 19 | igd | 0.7667 | T | \|\| id:ebi-a-GCST90001955 | ebi-a-GCST90001955 | TRUE | C | 51548032 | 3.96E-08 | reported | 1554 | 0.04339 | rs11084048 |  |  |  |  |  |  |  |  |  |  |  |  |  |  |  |
| -0.2096 | 6 | igd | 0.7652 | G | \|\| id:ebi-a-GCST90001973 | ebi-a-GCST90001973 | TRUE | C | 32592833 | 1.93E-10 | reported | 3113 | 0.03281 | rs9271673 |  |  |  |  |  |  |  |  |  |  |  |  |  |  |  |
| -0.1794 | 1 | igd | 0.7642 | A | \|\| id:ebi-a-GCST90001999 | ebi-a-GCST90001999 | TRUE | T | 161567067 | 2.62E-09 | reported | 3621 | 0.03006 | rs61801824 |  |  |  |  |  |  |  |  |  |  |  |  |  |  |  |
| -0.4813 | 6 | igd | 0.7641 | G | \|\| id:ebi-a-GCST90002110 | ebi-a-GCST90002110 | TRUE | C | 32583005 | 2.31E-26 | reported | 1634 | 0.04452 | rs6917212 |  |  |  |  |  |  |  |  |  |  |  |  |  |  |  |
| -0.4456 | 6 | igd | 0.7639 | G | \|\| id:ebi-a-GCST90002111 | ebi-a-GCST90002111 | TRUE | C | 32583005 | 2.14E-23 | reported | 1635 | 0.04403 | rs6917212 |  |  |  |  |  |  |  |  |  |  |  |  |  |  |  |
| -0.138717 | 1 | igd | 0.7631 | A | \|\| id:met-b-150 | met-b-150 | TRUE | C | 161661489 | 8.27E-09 | reported | 458 | 0.0240705 | rs1340976 |  |  |  |  |  |  |  |  |  |  |  |  |  |  |  |
| -0.1915 | 1 | igd | 0.762 | A | \|\| id:ebi-a-GCST90001970 | ebi-a-GCST90001970 | TRUE | T | 161567067 | 4.18E-09 | reported | 2971 | 0.03249 | rs61801824 |  |  |  |  |  |  |  |  |  |  |  |  |  |  |  |
| 0.1737 | 22 | igd | 0.7586 | T | \|\| id:ebi-a-GCST90001701 | ebi-a-GCST90001701 | TRUE | G | 42416695 | 1.13E-09 | reported | 3592 | 0.02844 | rs133337 |  |  |  |  |  |  |  |  |  |  |  |  |  |  |  |
| 0.5127 | 22 | igd | 0.7583 | T | \|\| id:ebi-a-GCST90001704 | ebi-a-GCST90001704 | TRUE | G | 42416695 | 4.16E-72 | reported | 3657 | 0.02792 | rs133337 |  |  |  |  |  |  |  |  |  |  |  |  |  |  |  |
| 0.248 | 19 | igd | 0.7552 | C | \|\| id:ebi-a-GCST90001947 | ebi-a-GCST90001947 | TRUE | T | 51548192 | 7.73E-09 | reported | 1577 | 0.04271 | rs6509516 |  |  |  |  |  |  |  |  |  |  |  |  |  |  |  |
| 0.2562 | 19 | igd | 0.7551 | C | \|\| id:ebi-a-GCST90001957 | ebi-a-GCST90001957 | TRUE | T | 51548192 | 2.20E-09 | reported | 1578 | 0.04259 | rs6509516 |  |  |  |  |  |  |  |  |  |  |  |  |  |  |  |
| 0.2537 | 19 | igd | 0.7549 | C | \|\| id:ebi-a-GCST90001956 | ebi-a-GCST90001956 | TRUE | T | 51548192 | 3.01E-09 | reported | 1579 | 0.04253 | rs6509516 |  |  |  |  |  |  |  |  |  |  |  |  |  |  |  |
| 0.1628 | 10 | igd | 0.7548 | C | \|\| id:ebi-a-GCST90001659 | ebi-a-GCST90001659 | TRUE | T | 99634541 | 7.51E-09 | reported | 3440 | 0.0281 | rs4919153 |  |  |  |  |  |  |  |  |  |  |  |  |  |  |  |
| 0.1906 | 10 | igd | 0.7547 | C | \|\| id:ebi-a-GCST90001660 | ebi-a-GCST90001660 | TRUE | T | 99634541 | 1.02E-10 | reported | 3408 | 0.0294 | rs4919153 |  |  |  |  |  |  |  |  |  |  |  |  |  |  |  |
| 0.158 | 10 | igd | 0.7546 | C | \|\| id:ebi-a-GCST90001483 | ebi-a-GCST90001483 | TRUE | T | 99634541 | 4.46E-08 | reported | 3405 | 0.02882 | rs4919153 |  |  |  |  |  |  |  |  |  |  |  |  |  |  |  |
| 0.193 | 2 | igd | 0.7533 | C | \|\| id:ebi-a-GCST90001508 | ebi-a-GCST90001508 | TRUE | T | 38964561 | 3.11E-10 | reported | 3437 | 0.03058 | rs1541877 |  |  |  |  |  |  |  |  |  |  |  |  |  |  |  |
| 0.1738 | 2 | igd | 0.7533 | C | \|\| id:ebi-a-GCST90001509 | ebi-a-GCST90001509 | TRUE | T | 38964561 | 1.44E-08 | reported | 3437 | 0.03059 | rs1541877 |  |  |  |  |  |  |  |  |  |  |  |  |  |  |  |
| -0.1878 | 2 | igd | 0.7522 | G | \|\| id:ebi-a-GCST90002119 | ebi-a-GCST90002119 | TRUE | C | 86756209 | 1.05E-09 | reported | 2920 | 0.03069 | rs1036940 |  |  |  |  |  |  |  |  |  |  |  |  |  |  |  |
| 0.2675 | 19 | igd | 0.7491 | C | \|\| id:ebi-a-GCST90001946 | ebi-a-GCST90001946 | TRUE | T | 51548192 | 1.04E-10 | reported | 1634 | 0.04113 | rs6509516 |  |  |  |  |  |  |  |  |  |  |  |  |  |  |  |
| 0.2556 | 19 | igd | 0.7491 | C | \|\| id:ebi-a-GCST90001949 | ebi-a-GCST90001949 | TRUE | T | 51548192 | 5.09E-10 | reported | 1634 | 0.04087 | rs6509516 |  |  |  |  |  |  |  |  |  |  |  |  |  |  |  |
| -0.2102 | 6 | igd | 0.749 | C | \|\| id:ebi-a-GCST90002071 | ebi-a-GCST90002071 | TRUE | T | 32421073 | 1.27E-09 | reported | 2872 | 0.03451 | rs9268791 |  |  |  |  |  |  |  |  |  |  |  |  |  |  |  |
| 0.2519 | 19 | igd | 0.7489 | C | \|\| id:ebi-a-GCST90001948 | ebi-a-GCST90001948 | TRUE | T | 51548192 | 9.90E-10 | reported | 1633 | 0.04098 | rs6509516 |  |  |  |  |  |  |  |  |  |  |  |  |  |  |  |
| -0.1913 | 2 | igd | 0.7479 | G | \|\| id:ebi-a-GCST90002120 | ebi-a-GCST90002120 | TRUE | A | 86869773 | 1.76E-09 | reported | 2920 | 0.03169 | rs308918 |  |  |  |  |  |  |  |  |  |  |  |  |  |  |  |
| -0.2455 | 19 | igd | 0.7477 | G | \|\| id:ebi-a-GCST90001955 | ebi-a-GCST90001955 | TRUE | A | 51600576 | 1.29E-08 | reported | 1554 | 0.04293 | rs2569504 |  |  |  |  |  |  |  |  |  |  |  |  |  |  |  |
| -0.3277 | 19 | igd | 0.7471 | A | \|\| id:ebi-a-GCST90001948 | ebi-a-GCST90001948 | TRUE | C | 51601490 | 9.25E-16 | reported | 1633 | 0.04036 | rs11084054 |  |  |  |  |  |  |  |  |  |  |  |  |  |  |  |
| -0.2649 | 19 | igd | 0.7469 | G | \|\| id:ebi-a-GCST90001954 | ebi-a-GCST90001954 | TRUE | A | 51600576 | 7.06E-10 | reported | 1555 | 0.0427 | rs2569504 |  |  |  |  |  |  |  |  |  |  |  |  |  |  |  |
| -0.3078 | 19 | igd | 0.7466 | A | \|\| id:ebi-a-GCST90001949 | ebi-a-GCST90001949 | TRUE | C | 51601490 | 3.86E-14 | reported | 1634 | 0.04032 | rs11084054 |  |  |  |  |  |  |  |  |  |  |  |  |  |  |  |
| -0.1863 | 2 | igd | 0.7463 | C | \|\| id:ebi-a-GCST90002121 | ebi-a-GCST90002121 | TRUE | T | 86861182 | 3.21E-09 | reported | 2915 | 0.03137 | rs218061 |  |  |  |  |  |  |  |  |  |  |  |  |  |  |  |
| -0.1868 | 2 | igd | 0.7455 | C | \|\| id:ebi-a-GCST90002120 | ebi-a-GCST90002120 | TRUE | T | 86993528 | 3.07E-09 | reported | 2920 | 0.03141 | rs6750263 |  |  |  |  |  |  |  |  |  |  |  |  |  |  |  |
| -0.1772 | 2 | igd | 0.7453 | A | \|\| id:ebi-a-GCST90002121 | ebi-a-GCST90002121 | TRUE | G | 86997548 | 1.25E-08 | reported | 2915 | 0.03104 | rs2271666 |  |  |  |  |  |  |  |  |  |  |  |  |  |  |  |
| -0.3118 | 19 | igd | 0.741 | A | \|\| id:ebi-a-GCST90001956 | ebi-a-GCST90001956 | TRUE | C | 51601490 | 8.25E-14 | reported | 1579 | 0.04139 | rs11084054 |  |  |  |  |  |  |  |  |  |  |  |  |  |  |  |
| 0.1326 | 6 | igd | 0.7405 | C | \|\| id:ebi-a-GCST90001690 | ebi-a-GCST90001690 | TRUE | T | 32592656 | 3.48E-08 | reported | 3408 | 0.02399 | rs9271664 |  |  |  |  |  |  |  |  |  |  |  |  |  |  |  |
| -0.3065 | 19 | igd | 0.7402 | C | \|\| id:ebi-a-GCST90001957 | ebi-a-GCST90001957 | TRUE | G | 51601063 | 2.11E-13 | reported | 1578 | 0.04139 | rs7507331 |  |  |  |  |  |  |  |  |  |  |  |  |  |  |  |
| -0.2967 | 19 | igd | 0.7394 | C | \|\| id:ebi-a-GCST90001947 | ebi-a-GCST90001947 | TRUE | T | 51606016 | 1.29E-12 | reported | 1577 | 0.04148 | rs2569427 |  |  |  |  |  |  |  |  |  |  |  |  |  |  |  |
| 0.2789 | 16 | igd | 0.7368 | A | \|\| id:ebi-a-GCST90002089 | ebi-a-GCST90002089 | TRUE | G | 31361988 | 1.11E-18 | reported | 2850 | 0.03139 | rs4493054 |  |  |  |  |  |  |  |  |  |  |  |  |  |  |  |
| 0.3611 | 16 | igd | 0.7366 | A | \|\| id:ebi-a-GCST90002088 | ebi-a-GCST90002088 | TRUE | G | 31361988 | 1.22E-32 | reported | 2872 | 0.02998 | rs4493054 |  |  |  |  |  |  |  |  |  |  |  |  |  |  |  |
| 0.1628 | 10 | igd | 0.7352 | T | \|\| id:ebi-a-GCST90001483 | ebi-a-GCST90001483 | TRUE | C | 98117061 | 1.03E-08 | reported | 3405 | 0.02836 | rs10748672 |  |  |  |  |  |  |  |  |  |  |  |  |  |  |  |
| 0.2037 | 10 | igd | 0.7352 | T | \|\| id:ebi-a-GCST90001489 | ebi-a-GCST90001489 | TRUE | C | 98117061 | 2.87E-12 | reported | 3405 | 0.02906 | rs10748672 |  |  |  |  |  |  |  |  |  |  |  |  |  |  |  |
| 0.2425 | 10 | igd | 0.7352 | T | \|\| id:ebi-a-GCST90001495 | ebi-a-GCST90001495 | TRUE | C | 98117061 | 1.48E-16 | reported | 3405 | 0.02921 | rs10748672 |  |  |  |  |  |  |  |  |  |  |  |  |  |  |  |
| 0.2582 | 10 | igd | 0.7352 | T | \|\| id:ebi-a-GCST90001660 | ebi-a-GCST90001660 | TRUE | C | 98117061 | 5.52E-19 | reported | 3408 | 0.02883 | rs10748672 |  |  |  |  |  |  |  |  |  |  |  |  |  |  |  |
| 0.302 | 10 | igd | 0.7352 | T | \|\| id:ebi-a-GCST90001672 | ebi-a-GCST90001672 | TRUE | C | 98117061 | 5.07E-25 | reported | 3408 | 0.029 | rs10748672 |  |  |  |  |  |  |  |  |  |  |  |  |  |  |  |
| 0.2534 | 10 | igd | 0.7349 | T | \|\| id:ebi-a-GCST90001658 | ebi-a-GCST90001658 | TRUE | C | 98117061 | 5.05E-20 | reported | 3440 | 0.02749 | rs10748672 |  |  |  |  |  |  |  |  |  |  |  |  |  |  |  |
| 0.2416 | 10 | igd | 0.7349 | T | \|\| id:ebi-a-GCST90001659 | ebi-a-GCST90001659 | TRUE | C | 98117061 | 2.58E-18 | reported | 3440 | 0.02752 | rs10748672 |  |  |  |  |  |  |  |  |  |  |  |  |  |  |  |
| 0.294 | 10 | igd | 0.7349 | T | \|\| id:ebi-a-GCST90001670 | ebi-a-GCST90001670 | TRUE | C | 98117061 | 1.52E-24 | reported | 3440 | 0.02853 | rs10748672 |  |  |  |  |  |  |  |  |  |  |  |  |  |  |  |
| 0.2914 | 10 | igd | 0.7349 | T | \|\| id:ebi-a-GCST90001671 | ebi-a-GCST90001671 | TRUE | C | 98117061 | 4.40E-24 | reported | 3440 | 0.02858 | rs10748672 |  |  |  |  |  |  |  |  |  |  |  |  |  |  |  |
| -0.1994 | 10 | igd | 0.7334 | T | \|\| id:ebi-a-GCST90001961 | ebi-a-GCST90001961 | TRUE | C | 98117061 | 2.17E-10 | reported | 2920 | 0.03129 | rs10748672 |  |  |  |  |  |  |  |  |  |  |  |  |  |  |  |
| 0.2526 | 10 | igd | 0.7334 | T | \|\| id:ebi-a-GCST90002031 | ebi-a-GCST90002031 | TRUE | C | 98117061 | 1.53E-15 | reported | 2920 | 0.03151 | rs10748672 |  |  |  |  |  |  |  |  |  |  |  |  |  |  |  |
| -0.1677 | 6 | igd | 0.7326 | C | \|\| id:ebi-a-GCST90002079 | ebi-a-GCST90002079 | TRUE | T | 32427789 | 2.50E-08 | reported | 3113 | 0.03001 | rs9268832 |  |  |  |  |  |  |  |  |  |  |  |  |  |  |  |
| -0.1734 | 2 | igd | 0.7325 | G | \|\| id:ebi-a-GCST90001460 | ebi-a-GCST90001460 | TRUE | A | 136974687 | 2.41E-10 | reported | 3374 | 0.0273 | rs2176718 |  |  |  |  |  |  |  |  |  |  |  |  |  |  |  |
| -0.1864 | 22 | igd | 0.7317 | T | \|\| id:ebi-a-GCST90001702 | ebi-a-GCST90001702 | TRUE | C | 43606143 | 6.33E-11 | reported | 3657 | 0.02844 | rs5759217 |  |  |  |  |  |  |  |  |  |  |  |  |  |  |  |
| -0.1827 | 22 | igd | 0.7317 | T | \|\| id:ebi-a-GCST90001703 | ebi-a-GCST90001703 | TRUE | C | 43606143 | 1.51E-10 | reported | 3657 | 0.02845 | rs5759217 |  |  |  |  |  |  |  |  |  |  |  |  |  |  |  |
| -0.1776 | 22 | igd | 0.7317 | T | \|\| id:ebi-a-GCST90001704 | ebi-a-GCST90001704 | TRUE | C | 43606143 | 4.54E-10 | reported | 3657 | 0.02842 | rs5759217 |  |  |  |  |  |  |  |  |  |  |  |  |  |  |  |
| -0.317 | 6 | igd | 0.7316 | A | \|\| id:ebi-a-GCST90002110 | ebi-a-GCST90002110 | TRUE | G | 32299592 | 2.52E-13 | reported | 1634 | 0.04296 | rs1003879 |  |  |  |  |  |  |  |  |  |  |  |  |  |  |  |
| -0.2955 | 6 | igd | 0.7315 | A | \|\| id:ebi-a-GCST90002111 | ebi-a-GCST90002111 | TRUE | G | 32299592 | 4.71E-12 | reported | 1635 | 0.04242 | rs1003879 |  |  |  |  |  |  |  |  |  |  |  |  |  |  |  |
| -0.2771 | 6 | igd | 0.7315 | A | \|\| id:ebi-a-GCST90002112 | ebi-a-GCST90002112 | TRUE | G | 32299592 | 1.50E-10 | reported | 1635 | 0.04298 | rs1003879 |  |  |  |  |  |  |  |  |  |  |  |  |  |  |  |
| 0.3807 | 6 | igd | 0.7314 | A | \|\| id:ebi-a-GCST90002105 | ebi-a-GCST90002105 | TRUE | G | 32680122 | 1.38E-24 | reported | 2872 | 0.03685 | rs3135461 |  |  |  |  |  |  |  |  |  |  |  |  |  |  |  |
| 0.1772 | 1 | igd | 0.7312 | C | \|\| id:ebi-a-GCST90001997 | ebi-a-GCST90001997 | TRUE | T | 161388960 | 4.38E-10 | reported | 3590 | 0.02833 | rs4326638 |  |  |  |  |  |  |  |  |  |  |  |  |  |  |  |
| 0.1777 | 1 | igd | 0.7312 | C | \|\| id:ebi-a-GCST90001995 | ebi-a-GCST90001995 | TRUE | T | 161388960 | 3.00E-10 | reported | 3590 | 0.02814 | rs4326638 |  |  |  |  |  |  |  |  |  |  |  |  |  |  |  |
| 0.2158 | 1 | igd | 0.7305 | C | \|\| id:ebi-a-GCST90001996 | ebi-a-GCST90001996 | TRUE | T | 161388960 | 1.88E-14 | reported | 3579 | 0.02806 | rs4326638 |  |  |  |  |  |  |  |  |  |  |  |  |  |  |  |
| -0.1733 | 10 | igd | 0.7302 | C | \|\| id:ebi-a-GCST90001787 | ebi-a-GCST90001787 | TRUE | T | 6079344 | 6.54E-10 | reported | 3657 | 0.02798 | rs11256442 |  |  |  |  |  |  |  |  |  |  |  |  |  |  |  |
| -0.2059 | 10 | igd | 0.7302 | C | \|\| id:ebi-a-GCST90001793 | ebi-a-GCST90001793 | TRUE | T | 6079344 | 2.27E-13 | reported | 3657 | 0.02798 | rs11256442 |  |  |  |  |  |  |  |  |  |  |  |  |  |  |  |
| 0.1759 | 16 | igd | 0.7298 | C | \|\| id:ebi-a-GCST90002087 | ebi-a-GCST90002087 | TRUE | T | 30914626 | 8.79E-09 | reported | 2872 | 0.03049 | rs1046276 |  |  |  |  |  |  |  |  |  |  |  |  |  |  |  |
| 0.1947 | 16 | igd | 0.7298 | C | \|\| id:ebi-a-GCST90002088 | ebi-a-GCST90002088 | TRUE | T | 30914626 | 1.65E-10 | reported | 2872 | 0.03035 | rs1046276 |  |  |  |  |  |  |  |  |  |  |  |  |  |  |  |
| 0.168 | 10 | igd | 0.7288 | G | \|\| id:ebi-a-GCST90001892 | ebi-a-GCST90001892 | TRUE | A | 97062753 | 4.61E-08 | reported | 2920 | 0.03066 | rs7084635 |  |  |  |  |  |  |  |  |  |  |  |  |  |  |  |
| 0.2548 | 10 | igd | 0.7288 | G | \|\| id:ebi-a-GCST90002030 | ebi-a-GCST90002030 | TRUE | A | 97062753 | 4.13E-17 | reported | 2920 | 0.03012 | rs7084635 |  |  |  |  |  |  |  |  |  |  |  |  |  |  |  |
| 0.2376 | 10 | igd | 0.7288 | G | \|\| id:ebi-a-GCST90002031 | ebi-a-GCST90002031 | TRUE | A | 97062753 | 2.43E-14 | reported | 2920 | 0.03101 | rs7084635 |  |  |  |  |  |  |  |  |  |  |  |  |  |  |  |
| 0.273 | 10 | igd | 0.7288 | G | \|\| id:ebi-a-GCST90002032 | ebi-a-GCST90002032 | TRUE | A | 97062753 | 1.58E-18 | reported | 2920 | 0.03087 | rs7084635 |  |  |  |  |  |  |  |  |  |  |  |  |  |  |  |
| -0.154 | 2 | igd | 0.7254 | C | \|\| id:ebi-a-GCST90001562 | ebi-a-GCST90001562 | TRUE | A | 39332093 | 9.58E-10 | reported | 3427 | 0.02511 | rs2168043 |  |  |  |  |  |  |  |  |  |  |  |  |  |  |  |
| 0.2543 | 19 | igd | 0.7241 | C | \|\| id:ebi-a-GCST90001954 | ebi-a-GCST90001954 | TRUE | T | 52161035 | 9.77E-10 | reported | 1555 | 0.04134 | rs12459503 |  |  |  |  |  |  |  |  |  |  |  |  |  |  |  |
| 0.2552 | 19 | igd | 0.724 | C | \|\| id:ebi-a-GCST90001953 | ebi-a-GCST90001953 | TRUE | T | 52161035 | 7.20E-10 | reported | 1558 | 0.04116 | rs12459503 |  |  |  |  |  |  |  |  |  |  |  |  |  |  |  |
| 0.2595 | 19 | igd | 0.7236 | C | \|\| id:ebi-a-GCST90001955 | ebi-a-GCST90001955 | TRUE | T | 52161035 | 4.26E-10 | reported | 1554 | 0.04128 | rs12459503 |  |  |  |  |  |  |  |  |  |  |  |  |  |  |  |
| -0.198 | 2 | igd | 0.7236 | C | \|\| id:ebi-a-GCST90002101 | ebi-a-GCST90002101 | TRUE | A | 39332093 | 7.63E-11 | reported | 2911 | 0.0303 | rs2168043 |  |  |  |  |  |  |  |  |  |  |  |  |  |  |  |
| -0.1812 | 10 | igd | 0.7235 | G | \|\| id:ebi-a-GCST90002032 | ebi-a-GCST90002032 | TRUE | A | 96129098 | 9.29E-09 | reported | 2920 | 0.03145 | rs2764376 |  |  |  |  |  |  |  |  |  |  |  |  |  |  |  |
| 0.1731 | 16 | igd | 0.7235 | C | \|\| id:ebi-a-GCST90002087 | ebi-a-GCST90002087 | TRUE | A | 31420635 | 4.74E-09 | reported | 2872 | 0.02946 | rs11865771 |  |  |  |  |  |  |  |  |  |  |  |  |  |  |  |
| 0.2305 | 19 | igd | 0.7231 | C | \|\| id:ebi-a-GCST90001957 | ebi-a-GCST90001957 | TRUE | T | 52161035 | 2.89E-08 | reported | 1578 | 0.04134 | rs12459503 |  |  |  |  |  |  |  |  |  |  |  |  |  |  |  |
| 0.2294 | 19 | igd | 0.7226 | C | \|\| id:ebi-a-GCST90001956 | ebi-a-GCST90001956 | TRUE | T | 52161035 | 3.17E-08 | reported | 1579 | 0.04126 | rs12459503 |  |  |  |  |  |  |  |  |  |  |  |  |  |  |  |
| -0.1801 | 12 | igd | 0.7218 | A | \|\| id:ebi-a-GCST90002073 | ebi-a-GCST90002073 | TRUE | G | 70561191 | 8.45E-09 | reported | 2850 | 0.03118 | rs10879100 |  |  |  |  |  |  |  |  |  |  |  |  |  |  |  |
| -0.1705 | 12 | igd | 0.7217 | A | \|\| id:ebi-a-GCST90002074 | ebi-a-GCST90002074 | TRUE | G | 70538903 | 1.17E-08 | reported | 3112 | 0.02981 | rs6581921 |  |  |  |  |  |  |  |  |  |  |  |  |  |  |  |
| 0.1984 | 6 | igd | 0.7204 | T | \|\| id:ebi-a-GCST90002119 | ebi-a-GCST90002119 | TRUE | C | 30365210 | 1.42E-09 | reported | 2920 | 0.03266 | rs1264570 |  |  |  |  |  |  |  |  |  |  |  |  |  |  |  |
| -0.2011 | 10 | igd | 0.7181 | A | \|\| id:ebi-a-GCST90001497 | ebi-a-GCST90001497 | TRUE | G | 95959384 | 4.41E-12 | reported | 3437 | 0.02894 | rs2797992 |  |  |  |  |  |  |  |  |  |  |  |  |  |  |  |
| -0.1772 | 10 | igd | 0.718 | A | \|\| id:ebi-a-GCST90001658 | ebi-a-GCST90001658 | TRUE | G | 95959384 | 2.76E-10 | reported | 3440 | 0.028 | rs2797992 |  |  |  |  |  |  |  |  |  |  |  |  |  |  |  |
| -0.1656 | 10 | igd | 0.718 | A | \|\| id:ebi-a-GCST90001671 | ebi-a-GCST90001671 | TRUE | G | 95959384 | 1.55E-08 | reported | 3440 | 0.02922 | rs2797992 |  |  |  |  |  |  |  |  |  |  |  |  |  |  |  |
| -0.1873 | 10 | igd | 0.7179 | T | \|\| id:ebi-a-GCST90001660 | ebi-a-GCST90001660 | TRUE | G | 92872454 | 3.48E-11 | reported | 3408 | 0.02818 | rs920333 |  |  |  |  |  |  |  |  |  |  |  |  |  |  |  |
| -0.1644 | 10 | igd | 0.7177 | T | \|\| id:ebi-a-GCST90001658 | ebi-a-GCST90001658 | TRUE | G | 92872454 | 1.14E-09 | reported | 3440 | 0.02693 | rs920333 |  |  |  |  |  |  |  |  |  |  |  |  |  |  |  |
| -0.17 | 10 | igd | 0.7177 | T | \|\| id:ebi-a-GCST90001659 | ebi-a-GCST90001659 | TRUE | G | 92872454 | 3.02E-10 | reported | 3440 | 0.02692 | rs920333 |  |  |  |  |  |  |  |  |  |  |  |  |  |  |  |
| -0.153 | 10 | igd | 0.7176 | T | \|\| id:ebi-a-GCST90001483 | ebi-a-GCST90001483 | TRUE | G | 92872454 | 3.33E-08 | reported | 3405 | 0.02764 | rs920333 |  |  |  |  |  |  |  |  |  |  |  |  |  |  |  |
| -0.1749 | 10 | igd | 0.7176 | T | \|\| id:ebi-a-GCST90001489 | ebi-a-GCST90001489 | TRUE | G | 92872454 | 7.70E-10 | reported | 3405 | 0.02836 | rs920333 |  |  |  |  |  |  |  |  |  |  |  |  |  |  |  |
| -0.1601 | 10 | igd | 0.7176 | T | \|\| id:ebi-a-GCST90001495 | ebi-a-GCST90001495 | TRUE | G | 92872454 | 2.31E-08 | reported | 3405 | 0.02859 | rs920333 |  |  |  |  |  |  |  |  |  |  |  |  |  |  |  |
| -0.1526 | 10 | igd | 0.7175 | T | \|\| id:ebi-a-GCST90001484 | ebi-a-GCST90001484 | TRUE | G | 92872454 | 2.94E-08 | reported | 3437 | 0.02745 | rs920333 |  |  |  |  |  |  |  |  |  |  |  |  |  |  |  |
| -0.1546 | 10 | igd | 0.7175 | T | \|\| id:ebi-a-GCST90001491 | ebi-a-GCST90001491 | TRUE | G | 92872454 | 3.27E-08 | reported | 3437 | 0.02791 | rs920333 |  |  |  |  |  |  |  |  |  |  |  |  |  |  |  |
| -0.1772 | 10 | igd | 0.7175 | T | \|\| id:ebi-a-GCST90002030 | ebi-a-GCST90002030 | TRUE | G | 92872454 | 4.80E-09 | reported | 2920 | 0.03018 | rs920333 |  |  |  |  |  |  |  |  |  |  |  |  |  |  |  |
| 0.2442 | 19 | igd | 0.7168 | C | \|\| id:ebi-a-GCST90001948 | ebi-a-GCST90001948 | TRUE | T | 52161035 | 1.02E-09 | reported | 1633 | 0.03976 | rs12459503 |  |  |  |  |  |  |  |  |  |  |  |  |  |  |  |
| 0.1813 | 6 | igd | 0.7166 | C | \|\| id:ebi-a-GCST90001932 | ebi-a-GCST90001932 | TRUE | T | 32425983 | 1.20E-08 | reported | 2920 | 0.03171 | rs6457592 |  |  |  |  |  |  |  |  |  |  |  |  |  |  |  |
| 0.2486 | 19 | igd | 0.7163 | C | \|\| id:ebi-a-GCST90001946 | ebi-a-GCST90001946 | TRUE | T | 52161035 | 5.65E-10 | reported | 1634 | 0.03986 | rs12459503 |  |  |  |  |  |  |  |  |  |  |  |  |  |  |  |
| 0.2221 | 19 | igd | 0.7163 | C | \|\| id:ebi-a-GCST90001949 | ebi-a-GCST90001949 | TRUE | T | 52161035 | 2.52E-08 | reported | 1634 | 0.03966 | rs12459503 |  |  |  |  |  |  |  |  |  |  |  |  |  |  |  |
| -0.2518 | 6 | igd | 0.7163 | C | \|\| id:ebi-a-GCST90002108 | ebi-a-GCST90002108 | TRUE | T | 29926597 | 1.10E-08 | reported | 1579 | 0.04384 | rs1632994 |  |  |  |  |  |  |  |  |  |  |  |  |  |  |  |
| -0.2424 | 6 | igd | 0.7156 | C | \|\| id:ebi-a-GCST90002111 | ebi-a-GCST90002111 | TRUE | T | 29927611 | 2.12E-08 | reported | 1635 | 0.04306 | rs2517696 |  |  |  |  |  |  |  |  |  |  |  |  |  |  |  |
| 0.2704 | 19 | igd | 0.7148 | C | \|\| id:ebi-a-GCST90001951 | ebi-a-GCST90001951 | TRUE | T | 52161035 | 1.74E-10 | reported | 1464 | 0.04205 | rs12459503 |  |  |  |  |  |  |  |  |  |  |  |  |  |  |  |
| -0.1897 | 1 | igd | 0.7139 | G | \|\| id:ebi-a-GCST90001474 | ebi-a-GCST90001474 | TRUE | C | 161492644 | 1.52E-11 | reported | 3410 | 0.02802 | rs6678239 |  |  |  |  |  |  |  |  |  |  |  |  |  |  |  |
| -0.1899 | 10 | igd | 0.7113 | C | \|\| id:ebi-a-GCST90002030 | ebi-a-GCST90002030 | TRUE | T | 96150974 | 8.20E-10 | reported | 2920 | 0.03082 | rs2265366 |  |  |  |  |  |  |  |  |  |  |  |  |  |  |  |
| -0.2844 | 6 | igd | 0.7111 | A | \|\| id:ebi-a-GCST90002110 | ebi-a-GCST90002110 | TRUE | G | 32710396 | 4.82E-11 | reported | 1634 | 0.04296 | rs9276410 |  |  |  |  |  |  |  |  |  |  |  |  |  |  |  |
| -0.2528 | 6 | igd | 0.711 | A | \|\| id:ebi-a-GCST90002111 | ebi-a-GCST90002111 | TRUE | G | 32710396 | 3.15E-09 | reported | 1635 | 0.04244 | rs9276410 |  |  |  |  |  |  |  |  |  |  |  |  |  |  |  |
| -0.2823 | 20 | igd | 0.7097 | C | \|\| id:ebi-a-GCST90001801 | ebi-a-GCST90001801 | TRUE | T | 44837987 | 1.07E-13 | reported | 1836 | 0.03769 | rs2425783 |  |  |  |  |  |  |  |  |  |  |  |  |  |  |  |
| -0.2195 | 20 | igd | 0.7081 | C | \|\| id:ebi-a-GCST90001798 | ebi-a-GCST90001798 | TRUE | T | 44837987 | 2.16E-15 | reported | 3657 | 0.02755 | rs2425783 |  |  |  |  |  |  |  |  |  |  |  |  |  |  |  |
| -0.1932 | 20 | igd | 0.7081 | C | \|\| id:ebi-a-GCST90001802 | ebi-a-GCST90001802 | TRUE | T | 44837987 | 5.07E-12 | reported | 3657 | 0.02789 | rs2425783 |  |  |  |  |  |  |  |  |  |  |  |  |  |  |  |
| -0.2207 | 20 | igd | 0.7081 | C | \|\| id:ebi-a-GCST90001804 | ebi-a-GCST90001804 | TRUE | T | 44837987 | 1.15E-15 | reported | 3657 | 0.02743 | rs2425783 |  |  |  |  |  |  |  |  |  |  |  |  |  |  |  |
| -0.1976 | 20 | igd | 0.7081 | C | \|\| id:ebi-a-GCST90001805 | ebi-a-GCST90001805 | TRUE | T | 44837987 | 1.60E-12 | reported | 3657 | 0.02788 | rs2425783 |  |  |  |  |  |  |  |  |  |  |  |  |  |  |  |
| -0.2368 | 20 | igd | 0.7081 | C | \|\| id:ebi-a-GCST90001808 | ebi-a-GCST90001808 | TRUE | T | 44837987 | 1.97E-17 | reported | 3657 | 0.02774 | rs2425783 |  |  |  |  |  |  |  |  |  |  |  |  |  |  |  |
| -0.1556 | 20 | igd | 0.708 | C | \|\| id:ebi-a-GCST90001800 | ebi-a-GCST90001800 | TRUE | T | 44837987 | 3.23E-08 | reported | 3656 | 0.02808 | rs2425783 |  |  |  |  |  |  |  |  |  |  |  |  |  |  |  |
| -0.2151 | 20 | igd | 0.708 | C | \|\| id:ebi-a-GCST90001806 | ebi-a-GCST90001806 | TRUE | T | 44837987 | 1.39E-14 | reported | 3656 | 0.02783 | rs2425783 |  |  |  |  |  |  |  |  |  |  |  |  |  |  |  |
| -0.2199 | 1 | igd | 0.7073 | G | \|\| id:ebi-a-GCST90002077 | ebi-a-GCST90002077 | TRUE | C | 161492644 | 1.71E-13 | reported | 2971 | 0.0297 | rs6678239 |  |  |  |  |  |  |  |  |  |  |  |  |  |  |  |
| -0.1856 | 22 | igd | 0.7069 | A | \|\| id:ebi-a-GCST90001702 | ebi-a-GCST90001702 | TRUE | G | 41511176 | 1.57E-11 | reported | 3657 | 0.02745 | rs738631 |  |  |  |  |  |  |  |  |  |  |  |  |  |  |  |
| -0.1721 | 22 | igd | 0.7069 | A | \|\| id:ebi-a-GCST90001703 | ebi-a-GCST90001703 | TRUE | G | 41511176 | 4.14E-10 | reported | 3657 | 0.02747 | rs738631 |  |  |  |  |  |  |  |  |  |  |  |  |  |  |  |
| -0.1938 | 22 | igd | 0.7069 | A | \|\| id:ebi-a-GCST90001704 | ebi-a-GCST90001704 | TRUE | G | 41511176 | 1.79E-12 | reported | 3657 | 0.02739 | rs738631 |  |  |  |  |  |  |  |  |  |  |  |  |  |  |  |
| -0.1914 | 22 | igd | 0.7069 | A | \|\| id:ebi-a-GCST90001710 | ebi-a-GCST90001710 | TRUE | G | 41511176 | 3.19E-12 | reported | 3657 | 0.02737 | rs738631 |  |  |  |  |  |  |  |  |  |  |  |  |  |  |  |
| -0.1796 | 22 | igd | 0.7069 | A | \|\| id:ebi-a-GCST90001711 | ebi-a-GCST90001711 | TRUE | G | 41511176 | 5.54E-11 | reported | 3657 | 0.02731 | rs738631 |  |  |  |  |  |  |  |  |  |  |  |  |  |  |  |
| -0.1886 | 6 | igd | 0.7067 | C | \|\| id:ebi-a-GCST90001757 | ebi-a-GCST90001757 | TRUE | A | 32577282 | 6.09E-10 | reported | 3657 | 0.0304 | rs9271146 |  |  |  |  |  |  |  |  |  |  |  |  |  |  |  |
| -0.1728 | 6 | igd | 0.7067 | C | \|\| id:ebi-a-GCST90001761 | ebi-a-GCST90001761 | TRUE | A | 32577282 | 1.77E-08 | reported | 3657 | 0.03061 | rs9271146 |  |  |  |  |  |  |  |  |  |  |  |  |  |  |  |
| 0.134426 | 1 | igd | 0.706522 | C | \|\| id:met-b-162 | met-b-162 | TRUE | T | 161651064 | 3.71E-09 | reported | 460 | 0.0227975 | rs723177 |  |  |  |  |  |  |  |  |  |  |  |  |  |  |  |
| -0.1613 | 6 | igd | 0.7042 | C | \|\| id:ebi-a-GCST90001768 | ebi-a-GCST90001768 | TRUE | T | 107161116 | 3.38E-09 | reported | 3655 | 0.02722 | rs2798364 |  |  |  |  |  |  |  |  |  |  |  |  |  |  |  |
| -0.2149 | 6 | igd | 0.7036 | G | \|\| id:ebi-a-GCST90001773 | ebi-a-GCST90001773 | TRUE | C | 107147266 | 4.18E-15 | reported | 3652 | 0.02726 | rs3127663 |  |  |  |  |  |  |  |  |  |  |  |  |  |  |  |
| -0.1701 | 6 | igd | 0.7032 | A | \|\| id:ebi-a-GCST90001774 | ebi-a-GCST90001774 | TRUE | G | 107161532 | 3.26E-10 | reported | 3656 | 0.02698 | rs2642465 |  |  |  |  |  |  |  |  |  |  |  |  |  |  |  |
| 0.267 | 16 | igd | 0.7032 | C | \|\| id:ebi-a-GCST90002087 | ebi-a-GCST90002087 | TRUE | T | 31336519 | 1.81E-19 | reported | 2872 | 0.02938 | rs7206295 |  |  |  |  |  |  |  |  |  |  |  |  |  |  |  |
| 0.1851 | 6 | igd | 0.7024 | C | \|\| id:ebi-a-GCST90002119 | ebi-a-GCST90002119 | TRUE | G | 30160055 | 7.60E-09 | reported | 2920 | 0.03195 | rs2284165 |  |  |  |  |  |  |  |  |  |  |  |  |  |  |  |
| -0.266 | 3 | igd | 0.7022 | T | \|\| id:ebi-a-GCST90002015 | ebi-a-GCST90002015 | TRUE | C | 46462144 | 2.73E-18 | reported | 2871 | 0.03029 | rs4317138 |  |  |  |  |  |  |  |  |  |  |  |  |  |  |  |
| -0.2656 | 3 | igd | 0.7022 | T | \|\| id:ebi-a-GCST90002016 | ebi-a-GCST90002016 | TRUE | C | 46462144 | 2.68E-18 | reported | 2871 | 0.03024 | rs4317138 |  |  |  |  |  |  |  |  |  |  |  |  |  |  |  |
| 0.3155 | 16 | igd | 0.7011 | T | \|\| id:ebi-a-GCST90002090 | ebi-a-GCST90002090 | TRUE | G | 31278991 | 9.42E-25 | reported | 2850 | 0.03042 | rs9938177 |  |  |  |  |  |  |  |  |  |  |  |  |  |  |  |
| 0.1842 | 16 | igd | 0.7007 | G | \|\| id:ebi-a-GCST90002087 | ebi-a-GCST90002087 | TRUE | T | 31162303 | 8.49E-10 | reported | 2872 | 0.02991 | rs9939286 |  |  |  |  |  |  |  |  |  |  |  |  |  |  |  |
| 0.178 | 6 | igd | 0.6997 | G | \|\| id:ebi-a-GCST90001932 | ebi-a-GCST90001932 | TRUE | A | 32200670 | 1.15E-08 | reported | 2920 | 0.0311 | rs6913558 |  |  |  |  |  |  |  |  |  |  |  |  |  |  |  |
| 0.166 | 17 | igd | 0.6986 | A | \|\| id:ebi-a-GCST90001926 | ebi-a-GCST90001926 | TRUE | C | 58074786 | 2.63E-08 | reported | 2920 | 0.02974 | rs17497477 |  |  |  |  |  |  |  |  |  |  |  |  |  |  |  |
| -0.2206 | 2 | igd | 0.6986 | G | \|\| id:ebi-a-GCST90002119 | ebi-a-GCST90002119 | TRUE | A | 87056805 | 5.07E-14 | reported | 2920 | 0.02916 | rs4240205 |  |  |  |  |  |  |  |  |  |  |  |  |  |  |  |
| -0.2032 | 10 | igd | 0.6978 | A | \|\| id:ebi-a-GCST90002030 | ebi-a-GCST90002030 | TRUE | G | 95250825 | 9.08E-12 | reported | 2920 | 0.02968 | rs12221109 |  |  |  |  |  |  |  |  |  |  |  |  |  |  |  |
| -0.1925 | 10 | igd | 0.6978 | A | \|\| id:ebi-a-GCST90002031 | ebi-a-GCST90002031 | TRUE | G | 95250825 | 2.61E-10 | reported | 2920 | 0.03035 | rs12221109 |  |  |  |  |  |  |  |  |  |  |  |  |  |  |  |
| -0.194 | 10 | igd | 0.6978 | A | \|\| id:ebi-a-GCST90002032 | ebi-a-GCST90002032 | TRUE | G | 95250825 | 1.86E-10 | reported | 2920 | 0.03034 | rs12221109 |  |  |  |  |  |  |  |  |  |  |  |  |  |  |  |
| -0.3893 | 6 | igd | 0.6976 | G | \|\| id:ebi-a-GCST90002108 | ebi-a-GCST90002108 | TRUE | T | 32197219 | 8.86E-20 | reported | 1579 | 0.04221 | rs3132948 |  |  |  |  |  |  |  |  |  |  |  |  |  |  |  |
| -0.1892 | 10 | igd | 0.6971 | G | \|\| id:ebi-a-GCST90001961 | ebi-a-GCST90001961 | TRUE | A | 98819317 | 4.62E-10 | reported | 2920 | 0.03025 | rs2817676 |  |  |  |  |  |  |  |  |  |  |  |  |  |  |  |
| 0.2158 | 10 | igd | 0.6971 | G | \|\| id:ebi-a-GCST90002032 | ebi-a-GCST90002032 | TRUE | A | 98819317 | 2.01E-12 | reported | 2920 | 0.03055 | rs2817676 |  |  |  |  |  |  |  |  |  |  |  |  |  |  |  |
| -0.2607 | 6 | igd | 0.696 | A | \|\| id:ebi-a-GCST90002108 | ebi-a-GCST90002108 | TRUE | G | 31319733 | 1.21E-09 | reported | 1579 | 0.04263 | rs9266074 |  |  |  |  |  |  |  |  |  |  |  |  |  |  |  |
| -0.2636 | 6 | igd | 0.6957 | A | \|\| id:ebi-a-GCST90002112 | ebi-a-GCST90002112 | TRUE | G | 31319733 | 5.68E-10 | reported | 1635 | 0.04227 | rs9266074 |  |  |  |  |  |  |  |  |  |  |  |  |  |  |  |
| -0.292 | 6 | igd | 0.6941 | A | \|\| id:ebi-a-GCST90002105 | ebi-a-GCST90002105 | TRUE | G | 31740454 | 1.11E-19 | reported | 2872 | 0.03194 | rs805825 |  |  |  |  |  |  |  |  |  |  |  |  |  |  |  |
| -0.2249 | 6 | igd | 0.692 | C | \|\| id:ebi-a-GCST90002112 | ebi-a-GCST90002112 | TRUE | A | 31319391 | 3.77E-08 | reported | 1635 | 0.04068 | rs9266063 |  |  |  |  |  |  |  |  |  |  |  |  |  |  |  |
| -0.2785 | 6 | igd | 0.6909 | C | \|\| id:ebi-a-GCST90002108 | ebi-a-GCST90002108 | TRUE | A | 31319391 | 1.23E-11 | reported | 1579 | 0.04079 | rs9266063 |  |  |  |  |  |  |  |  |  |  |  |  |  |  |  |
| 0.2454 | 3 | igd | 0.6904 | A | \|\| id:ebi-a-GCST90002004 | ebi-a-GCST90002004 | TRUE | G | 46187707 | 1.17E-20 | reported | 3629 | 0.02618 | rs4683184 |  |  |  |  |  |  |  |  |  |  |  |  |  |  |  |
| 0.2224 | 3 | igd | 0.6904 | A | \|\| id:ebi-a-GCST90002008 | ebi-a-GCST90002008 | TRUE | G | 46187707 | 6.59E-17 | reported | 3629 | 0.0265 | rs4683184 |  |  |  |  |  |  |  |  |  |  |  |  |  |  |  |
| 0.1453 | 10 | igd | 0.6894 | G | \|\| id:ebi-a-GCST90001658 | ebi-a-GCST90001658 | TRUE | A | 100051497 | 2.52E-08 | reported | 3440 | 0.02601 | rs1925637 |  |  |  |  |  |  |  |  |  |  |  |  |  |  |  |
| 0.1781 | 15 | igd | 0.6885 | C | \|\| id:ebi-a-GCST90001821 | ebi-a-GCST90001821 | TRUE | T | 41075944 | 2.52E-11 | reported | 3656 | 0.02661 | rs2412554 |  |  |  |  |  |  |  |  |  |  |  |  |  |  |  |
| -0.2845 | 6 | igd | 0.6885 | A | \|\| id:ebi-a-GCST90002105 | ebi-a-GCST90002105 | TRUE | G | 31319733 | 1.35E-17 | reported | 2872 | 0.03311 | rs9266074 |  |  |  |  |  |  |  |  |  |  |  |  |  |  |  |
| -0.1813 | 6 | igd | 0.6884 | T | \|\| id:ebi-a-GCST90002105 | ebi-a-GCST90002105 | TRUE | A | 32973263 | 5.73E-09 | reported | 2872 | 0.03103 | rs3128936 |  |  |  |  |  |  |  |  |  |  |  |  |  |  |  |
| 0.3253 | 3 | igd | 0.6884 | A | \|\| id:ebi-a-GCST90002017 | ebi-a-GCST90002017 | TRUE | G | 46187707 | 2.69E-27 | reported | 2850 | 0.02975 | rs4683184 |  |  |  |  |  |  |  |  |  |  |  |  |  |  |  |
| -0.1539 | 22 | igd | 0.6864 | G | \|\| id:ebi-a-GCST90001703 | ebi-a-GCST90001703 | TRUE | A | 43563711 | 3.08E-08 | reported | 3657 | 0.02774 | rs107402 |  |  |  |  |  |  |  |  |  |  |  |  |  |  |  |
| -0.1532 | 22 | igd | 0.6864 | G | \|\| id:ebi-a-GCST90001704 | ebi-a-GCST90001704 | TRUE | A | 43563711 | 3.42E-08 | reported | 3657 | 0.0277 | rs107402 |  |  |  |  |  |  |  |  |  |  |  |  |  |  |  |
| -0.1609 | 22 | igd | 0.6864 | G | \|\| id:ebi-a-GCST90001710 | ebi-a-GCST90001710 | TRUE | A | 43563711 | 6.53E-09 | reported | 3657 | 0.02766 | rs107402 |  |  |  |  |  |  |  |  |  |  |  |  |  |  |  |
| -0.1704 | 22 | igd | 0.6864 | G | \|\| id:ebi-a-GCST90001711 | ebi-a-GCST90001711 | TRUE | A | 43563711 | 7.09E-10 | reported | 3657 | 0.02758 | rs107402 |  |  |  |  |  |  |  |  |  |  |  |  |  |  |  |
| 0.1594 | 16 | igd | 0.6859 | G | \|\| id:ebi-a-GCST90002088 | ebi-a-GCST90002088 | TRUE | A | 30812962 | 4.56E-08 | reported | 2872 | 0.02908 | rs4889489 |  |  |  |  |  |  |  |  |  |  |  |  |  |  |  |
| 0.212 | 16 | igd | 0.6847 | G | \|\| id:ebi-a-GCST90002088 | ebi-a-GCST90002088 | TRUE | A | 31261241 | 1.62E-13 | reported | 2872 | 0.0286 | rs8047985 |  |  |  |  |  |  |  |  |  |  |  |  |  |  |  |
| -0.2298 | 20 | igd | 0.6817 | T | \|\| id:ebi-a-GCST90001798 | ebi-a-GCST90001798 | TRUE | A | 44784151 | 3.79E-18 | reported | 3657 | 0.02632 | rs6074045 |  |  |  |  |  |  |  |  |  |  |  |  |  |  |  |
| -0.1923 | 20 | igd | 0.6816 | T | \|\| id:ebi-a-GCST90001800 | ebi-a-GCST90001800 | TRUE | A | 44784151 | 8.28E-13 | reported | 3656 | 0.02677 | rs6074045 |  |  |  |  |  |  |  |  |  |  |  |  |  |  |  |
| -0.1938 | 20 | igd | 0.6816 | T | \|\| id:ebi-a-GCST90001806 | ebi-a-GCST90001806 | TRUE | A | 44784151 | 4.04E-13 | reported | 3656 | 0.02661 | rs6074045 |  |  |  |  |  |  |  |  |  |  |  |  |  |  |  |
| 0.1486 | 22 | igd | 0.6794 | C | \|\| id:ebi-a-GCST90001702 | ebi-a-GCST90001702 | TRUE | T | 41750622 | 3.75E-08 | reported | 3657 | 0.02694 | rs9611560 |  |  |  |  |  |  |  |  |  |  |  |  |  |  |  |
| 0.1542 | 22 | igd | 0.6794 | C | \|\| id:ebi-a-GCST90001710 | ebi-a-GCST90001710 | TRUE | T | 41750622 | 1.01E-08 | reported | 3657 | 0.02686 | rs9611560 |  |  |  |  |  |  |  |  |  |  |  |  |  |  |  |
| -0.1822 | 14 | igd | 0.6794 | T | \|\| id:ebi-a-GCST90001821 | ebi-a-GCST90001821 | TRUE | C | 106096348 | 3.23E-10 | reported | 3656 | 0.02889 | rs61986375 |  |  |  |  |  |  |  |  |  |  |  |  |  |  |  |
| 0.1778 | 6 | igd | 0.6781 | G | \|\| id:ebi-a-GCST90002119 | ebi-a-GCST90002119 | TRUE | T | 29699055 | 3.28E-08 | reported | 2920 | 0.0321 | rs2523403 |  |  |  |  |  |  |  |  |  |  |  |  |  |  |  |
| -0.1614 | 10 | igd | 0.6775 | G | \|\| id:ebi-a-GCST90001670 | ebi-a-GCST90001670 | TRUE | A | 95167741 | 2.97E-09 | reported | 3440 | 0.02713 | rs1617402 |  |  |  |  |  |  |  |  |  |  |  |  |  |  |  |
| -0.1679 | 10 | igd | 0.6775 | G | \|\| id:ebi-a-GCST90001671 | ebi-a-GCST90001671 | TRUE | A | 95167741 | 6.95E-10 | reported | 3440 | 0.02715 | rs1617402 |  |  |  |  |  |  |  |  |  |  |  |  |  |  |  |
| 0.3786 | 6 | igd | 0.6771 | C | \|\| id:ebi-a-GCST90002112 | ebi-a-GCST90002112 | TRUE | T | 32663151 | 4.91E-18 | reported | 1635 | 0.04324 | rs2647021 |  |  |  |  |  |  |  |  |  |  |  |  |  |  |  |
| 0.1617 | 11 | igd | 0.6753 | T | \|\| id:ebi-a-GCST90001757 | ebi-a-GCST90001757 | TRUE | C | 60174250 | 7.80E-10 | reported | 3657 | 0.02624 | rs955019 |  |  |  |  |  |  |  |  |  |  |  |  |  |  |  |
| 0.1857 | 17 | igd | 0.6743 | G | \|\| id:ebi-a-GCST90001654 | ebi-a-GCST90001654 | TRUE | A | 33794697 | 5.64E-12 | reported | 3408 | 0.02686 | rs9896155 |  |  |  |  |  |  |  |  |  |  |  |  |  |  |  |
| 0.1701 | 17 | igd | 0.6741 | G | \|\| id:ebi-a-GCST90001694 | ebi-a-GCST90001694 | TRUE | A | 33794697 | 5.51E-10 | reported | 3440 | 0.02733 | rs9896155 |  |  |  |  |  |  |  |  |  |  |  |  |  |  |  |
| 0.2013 | 6 | igd | 0.6736 | G | \|\| id:ebi-a-GCST90002118 | ebi-a-GCST90002118 | TRUE | A | 30382972 | 3.41E-10 | reported | 2920 | 0.03195 | rs1150769 |  |  |  |  |  |  |  |  |  |  |  |  |  |  |  |
| 0.1765 | 6 | igd | 0.6736 | G | \|\| id:ebi-a-GCST90002121 | ebi-a-GCST90002121 | TRUE | A | 30382972 | 6.36E-09 | reported | 2915 | 0.03031 | rs1150769 |  |  |  |  |  |  |  |  |  |  |  |  |  |  |  |
| -0.3051 | 6 | igd | 0.6734 | C | \|\| id:ebi-a-GCST90002112 | ebi-a-GCST90002112 | TRUE | T | 31407217 | 1.74E-13 | reported | 1635 | 0.04105 | rs3749937 |  |  |  |  |  |  |  |  |  |  |  |  |  |  |  |
| -0.1684 | 10 | igd | 0.6725 | T | \|\| id:ebi-a-GCST90001670 | ebi-a-GCST90001670 | TRUE | A | 95303356 | 6.15E-10 | reported | 3440 | 0.02715 | rs10786112 |  |  |  |  |  |  |  |  |  |  |  |  |  |  |  |
| 0.2029 | 10 | igd | 0.6721 | A | \|\| id:ebi-a-GCST90001658 | ebi-a-GCST90001658 | TRUE | G | 98123188 | 5.10E-15 | reported | 3440 | 0.02582 | rs10736106 |  |  |  |  |  |  |  |  |  |  |  |  |  |  |  |
| 0.2094 | 10 | igd | 0.6721 | A | \|\| id:ebi-a-GCST90001659 | ebi-a-GCST90001659 | TRUE | G | 98123188 | 6.69E-16 | reported | 3440 | 0.0258 | rs10736106 |  |  |  |  |  |  |  |  |  |  |  |  |  |  |  |
| 0.1734 | 10 | igd | 0.6715 | A | \|\| id:ebi-a-GCST90001495 | ebi-a-GCST90001495 | TRUE | G | 98123188 | 2.93E-10 | reported | 3405 | 0.02743 | rs10736106 |  |  |  |  |  |  |  |  |  |  |  |  |  |  |  |
| -0.1643 | 10 | igd | 0.6715 | T | \|\| id:ebi-a-GCST90001672 | ebi-a-GCST90001672 | TRUE | A | 95303356 | 3.00E-09 | reported | 3408 | 0.02762 | rs10786112 |  |  |  |  |  |  |  |  |  |  |  |  |  |  |  |
| 0.2171 | 10 | igd | 0.6712 | G | \|\| id:ebi-a-GCST90001660 | ebi-a-GCST90001660 | TRUE | A | 98122808 | 1.05E-15 | reported | 3408 | 0.02694 | rs10786282 |  |  |  |  |  |  |  |  |  |  |  |  |  |  |  |
| -0.1673 | 2 | igd | 0.6705 | T | \|\| id:ebi-a-GCST90001664 | ebi-a-GCST90001664 | TRUE | G | 39172477 | 2.30E-11 | reported | 3440 | 0.02495 | rs11124656 |  |  |  |  |  |  |  |  |  |  |  |  |  |  |  |
| -0.21 | 2 | igd | 0.6703 | T | \|\| id:ebi-a-GCST90001561 | ebi-a-GCST90001561 | TRUE | G | 39172477 | 5.93E-20 | reported | 3427 | 0.02282 | rs11124656 |  |  |  |  |  |  |  |  |  |  |  |  |  |  |  |
| -0.1946 | 2 | igd | 0.6703 | T | \|\| id:ebi-a-GCST90001562 | ebi-a-GCST90001562 | TRUE | G | 39172477 | 3.20E-16 | reported | 3427 | 0.02371 | rs11124656 |  |  |  |  |  |  |  |  |  |  |  |  |  |  |  |
| 0.1459 | 6 | igd | 0.6702 | G | \|\| id:ebi-a-GCST90001765 | ebi-a-GCST90001765 | TRUE | A | 107141927 | 1.83E-08 | reported | 3655 | 0.02587 | rs3127661 |  |  |  |  |  |  |  |  |  |  |  |  |  |  |  |
| 0.1688 | 6 | igd | 0.6702 | G | \|\| id:ebi-a-GCST90001772 | ebi-a-GCST90001772 | TRUE | A | 107141927 | 8.97E-11 | reported | 3655 | 0.02596 | rs3127661 |  |  |  |  |  |  |  |  |  |  |  |  |  |  |  |
| -0.1607 | 2 | igd | 0.6699 | T | \|\| id:ebi-a-GCST90001666 | ebi-a-GCST90001666 | TRUE | G | 39172477 | 1.40E-11 | reported | 3408 | 0.02369 | rs11124656 |  |  |  |  |  |  |  |  |  |  |  |  |  |  |  |
| -0.1516 | 2 | igd | 0.6697 | T | \|\| id:ebi-a-GCST90001560 | ebi-a-GCST90001560 | TRUE | G | 39172477 | 7.30E-11 | reported | 3395 | 0.02319 | rs11124656 |  |  |  |  |  |  |  |  |  |  |  |  |  |  |  |
| 0.1751 | 10 | igd | 0.6697 | T | \|\| id:ebi-a-GCST90001961 | ebi-a-GCST90001961 | TRUE | A | 95303356 | 2.86E-09 | reported | 2920 | 0.02938 | rs10786112 |  |  |  |  |  |  |  |  |  |  |  |  |  |  |  |
| -0.1822 | 6 | igd | 0.669 | T | \|\| id:ebi-a-GCST90001753 | ebi-a-GCST90001753 | TRUE | G | 32545877 | 1.66E-08 | reported | 3657 | 0.0322 | rs9269642 |  |  |  |  |  |  |  |  |  |  |  |  |  |  |  |
| -0.1649 | 2 | igd | 0.6685 | A | \|\| id:ebi-a-GCST90001460 | ebi-a-GCST90001460 | TRUE | G | 136755684 | 2.77E-10 | reported | 3374 | 0.02605 | rs309134 |  |  |  |  |  |  |  |  |  |  |  |  |  |  |  |
| -0.1949 | 2 | igd | 0.668 | T | \|\| id:ebi-a-GCST90002101 | ebi-a-GCST90002101 | TRUE | G | 39172477 | 1.61E-11 | reported | 2911 | 0.02881 | rs11124656 |  |  |  |  |  |  |  |  |  |  |  |  |  |  |  |
| -0.198 | 10 | igd | 0.6675 | G | \|\| id:ebi-a-GCST90001961 | ebi-a-GCST90001961 | TRUE | A | 98122808 | 7.84E-12 | reported | 2920 | 0.02882 | rs10786282 |  |  |  |  |  |  |  |  |  |  |  |  |  |  |  |
| 0.1789 | 10 | igd | 0.6675 | G | \|\| id:ebi-a-GCST90002061 | ebi-a-GCST90002061 | TRUE | A | 98122808 | 8.40E-10 | reported | 2920 | 0.02905 | rs10786282 |  |  |  |  |  |  |  |  |  |  |  |  |  |  |  |
| 0.1525 | 20 | igd | 0.6669 | C | \|\| id:ebi-a-GCST90001798 | ebi-a-GCST90001798 | TRUE | T | 42019380 | 7.02E-09 | reported | 3657 | 0.02627 | rs9989924 |  |  |  |  |  |  |  |  |  |  |  |  |  |  |  |
| -0.3093 | 6 | igd | 0.6668 | C | \|\| id:ebi-a-GCST90002105 | ebi-a-GCST90002105 | TRUE | G | 31402454 | 1.41E-21 | reported | 2872 | 0.03216 | rs6912659 |  |  |  |  |  |  |  |  |  |  |  |  |  |  |  |
| -0.1777 | 6 | igd | 0.6628 | G | \|\| id:ebi-a-GCST90001752 | ebi-a-GCST90001752 | TRUE | T | 32593220 | 1.54E-08 | reported | 3657 | 0.03135 | rs9271702 |  |  |  |  |  |  |  |  |  |  |  |  |  |  |  |
| 0.1732 | 1 | igd | 0.6622 | G | \|\| id:ebi-a-GCST90001860 | ebi-a-GCST90001860 | TRUE | T | 167500918 | 1.69E-09 | reported | 2919 | 0.02866 | rs858496 |  |  |  |  |  |  |  |  |  |  |  |  |  |  |  |
| -0.2466 | 6 | igd | 0.6586 | G | \|\| id:ebi-a-GCST90002108 | ebi-a-GCST90002108 | TRUE | A | 31402383 | 5.87E-09 | reported | 1579 | 0.04213 | rs55957433 |  |  |  |  |  |  |  |  |  |  |  |  |  |  |  |
| 0.2501 | 10 | igd | 0.6574 | G | \|\| id:ebi-a-GCST90001670 | ebi-a-GCST90001670 | TRUE | A | 98118257 | 1.35E-20 | reported | 3440 | 0.02671 | rs11188736 |  |  |  |  |  |  |  |  |  |  |  |  |  |  |  |
| 0.24 | 10 | igd | 0.6574 | G | \|\| id:ebi-a-GCST90001671 | ebi-a-GCST90001671 | TRUE | A | 98118248 | 5.10E-19 | reported | 3440 | 0.02678 | rs11188735 |  |  |  |  |  |  |  |  |  |  |  |  |  |  |  |
| 0.1648 | 1 | igd | 0.6571 | C | \|\| id:ebi-a-GCST90001854 | ebi-a-GCST90001854 | TRUE | T | 167500815 | 5.65E-09 | reported | 2919 | 0.0282 | rs704859 |  |  |  |  |  |  |  |  |  |  |  |  |  |  |  |
| 0.258 | 10 | igd | 0.657 | C | \|\| id:ebi-a-GCST90001672 | ebi-a-GCST90001672 | TRUE | T | 98116936 | 2.89E-21 | reported | 3408 | 0.02707 | rs10786281 |  |  |  |  |  |  |  |  |  |  |  |  |  |  |  |
| -0.1537 | 3 | igd | 0.657 | T | \|\| id:ebi-a-GCST90001681 | ebi-a-GCST90001681 | TRUE | C | 76674553 | 3.16E-08 | reported | 3130 | 0.02772 | rs2875404 |  |  |  |  |  |  |  |  |  |  |  |  |  |  |  |
| 0.3245 | 6 | igd | 0.6568 | G | \|\| id:ebi-a-GCST90002109 | ebi-a-GCST90002109 | TRUE | A | 32629859 | 4.97E-14 | reported | 1578 | 0.04269 | rs1049130 |  |  |  |  |  |  |  |  |  |  |  |  |  |  |  |
| -0.1879 | 10 | igd | 0.6558 | T | \|\| id:ebi-a-GCST90001496 | ebi-a-GCST90001496 | TRUE | C | 96801753 | 1.09E-11 | reported | 3437 | 0.02756 | rs1934985 |  |  |  |  |  |  |  |  |  |  |  |  |  |  |  |
| -0.1715 | 10 | igd | 0.6555 | T | \|\| id:ebi-a-GCST90001495 | ebi-a-GCST90001495 | TRUE | C | 96801753 | 8.56E-10 | reported | 3405 | 0.02788 | rs1934985 |  |  |  |  |  |  |  |  |  |  |  |  |  |  |  |
| -0.1711 | 10 | igd | 0.6554 | T | \|\| id:ebi-a-GCST90001660 | ebi-a-GCST90001660 | TRUE | C | 96801753 | 6.44E-10 | reported | 3408 | 0.02761 | rs1934985 |  |  |  |  |  |  |  |  |  |  |  |  |  |  |  |
| -0.3156 | 20 | igd | 0.6554 | A | \|\| id:ebi-a-GCST90001985 | ebi-a-GCST90001985 | TRUE | G | 44658296 | 5.41E-33 | reported | 3629 | 0.02612 | rs2868364 |  |  |  |  |  |  |  |  |  |  |  |  |  |  |  |
| -0.317 | 20 | igd | 0.6551 | T | \|\| id:ebi-a-GCST90001981 | ebi-a-GCST90001981 | TRUE | C | 44656949 | 1.21E-33 | reported | 3618 | 0.02596 | rs8125780 |  |  |  |  |  |  |  |  |  |  |  |  |  |  |  |
| -0.3066 | 20 | igd | 0.6551 | A | \|\| id:ebi-a-GCST90001989 | ebi-a-GCST90001989 | TRUE | G | 44658296 | 5.05E-31 | reported | 3621 | 0.02623 | rs2868364 |  |  |  |  |  |  |  |  |  |  |  |  |  |  |  |
| -0.347 | 6 | igd | 0.6536 | C | \|\| id:ebi-a-GCST90002110 | ebi-a-GCST90002110 | TRUE | T | 32707018 | 7.31E-16 | reported | 1634 | 0.04259 | rs9276362 |  |  |  |  |  |  |  |  |  |  |  |  |  |  |  |
| -0.3031 | 6 | igd | 0.6535 | C | \|\| id:ebi-a-GCST90002111 | ebi-a-GCST90002111 | TRUE | T | 32707018 | 9.57E-13 | reported | 1635 | 0.04213 | rs9276362 |  |  |  |  |  |  |  |  |  |  |  |  |  |  |  |
| -0.1864 | 10 | igd | 0.6533 | G | \|\| id:ebi-a-GCST90001659 | ebi-a-GCST90001659 | TRUE | T | 96011865 | 2.35E-12 | reported | 3440 | 0.02648 | rs1547643 |  |  |  |  |  |  |  |  |  |  |  |  |  |  |  |
| -0.1779 | 10 | igd | 0.6527 | G | \|\| id:ebi-a-GCST90001660 | ebi-a-GCST90001660 | TRUE | T | 96011865 | 1.64E-10 | reported | 3408 | 0.02775 | rs1547643 |  |  |  |  |  |  |  |  |  |  |  |  |  |  |  |
| -0.1724 | 10 | igd | 0.6526 | G | \|\| id:ebi-a-GCST90001489 | ebi-a-GCST90001489 | TRUE | T | 96011865 | 5.42E-10 | reported | 3405 | 0.0277 | rs1547643 |  |  |  |  |  |  |  |  |  |  |  |  |  |  |  |
| 0.2667 | 6 | igd | 0.6523 | C | \|\| id:ebi-a-GCST90002108 | ebi-a-GCST90002108 | TRUE | T | 31104698 | 2.16E-10 | reported | 1579 | 0.04174 | rs3130571 |  |  |  |  |  |  |  |  |  |  |  |  |  |  |  |
| 0.1551 | 20 | igd | 0.6505 | T | \|\| id:ebi-a-GCST90001798 | ebi-a-GCST90001798 | TRUE | A | 44953649 | 3.70E-09 | reported | 3657 | 0.02624 | rs1998253 |  |  |  |  |  |  |  |  |  |  |  |  |  |  |  |
| 0.1722 | 20 | igd | 0.6505 | T | \|\| id:ebi-a-GCST90001804 | ebi-a-GCST90001804 | TRUE | A | 44953649 | 4.63E-11 | reported | 3657 | 0.02608 | rs1998253 |  |  |  |  |  |  |  |  |  |  |  |  |  |  |  |
| 0.1496 | 20 | igd | 0.6505 | T | \|\| id:ebi-a-GCST90001805 | ebi-a-GCST90001805 | TRUE | A | 44953649 | 1.81E-08 | reported | 3657 | 0.02651 | rs1998253 |  |  |  |  |  |  |  |  |  |  |  |  |  |  |  |
| 0.1651 | 20 | igd | 0.6505 | T | \|\| id:ebi-a-GCST90001808 | ebi-a-GCST90001808 | TRUE | A | 44953649 | 4.73E-10 | reported | 3657 | 0.02643 | rs1998253 |  |  |  |  |  |  |  |  |  |  |  |  |  |  |  |
| 0.1642 | 20 | igd | 0.6504 | T | \|\| id:ebi-a-GCST90001800 | ebi-a-GCST90001800 | TRUE | A | 44953649 | 7.85E-10 | reported | 3656 | 0.02664 | rs1998253 |  |  |  |  |  |  |  |  |  |  |  |  |  |  |  |
| 0.1545 | 20 | igd | 0.6504 | T | \|\| id:ebi-a-GCST90001806 | ebi-a-GCST90001806 | TRUE | A | 44953649 | 6.21E-09 | reported | 3656 | 0.02652 | rs1998253 |  |  |  |  |  |  |  |  |  |  |  |  |  |  |  |
| 0.2057 | 22 | igd | 0.6503 | G | \|\| id:ebi-a-GCST90001702 | ebi-a-GCST90001702 | TRUE | C | 39758830 | 2.81E-15 | reported | 3657 | 0.02592 | rs2267408 |  |  |  |  |  |  |  |  |  |  |  |  |  |  |  |
| 0.1982 | 22 | igd | 0.6503 | G | \|\| id:ebi-a-GCST90001703 | ebi-a-GCST90001703 | TRUE | C | 39758830 | 2.71E-14 | reported | 3657 | 0.02594 | rs2267408 |  |  |  |  |  |  |  |  |  |  |  |  |  |  |  |
| 0.207 | 22 | igd | 0.6503 | G | \|\| id:ebi-a-GCST90001704 | ebi-a-GCST90001704 | TRUE | C | 39758830 | 1.68E-15 | reported | 3657 | 0.02589 | rs2267408 |  |  |  |  |  |  |  |  |  |  |  |  |  |  |  |
| 0.2045 | 22 | igd | 0.6503 | G | \|\| id:ebi-a-GCST90001710 | ebi-a-GCST90001710 | TRUE | C | 39758830 | 3.42E-15 | reported | 3657 | 0.02585 | rs2267408 |  |  |  |  |  |  |  |  |  |  |  |  |  |  |  |
| 0.2094 | 22 | igd | 0.6503 | G | \|\| id:ebi-a-GCST90001711 | ebi-a-GCST90001711 | TRUE | C | 39758830 | 6.16E-16 | reported | 3657 | 0.02578 | rs2267408 |  |  |  |  |  |  |  |  |  |  |  |  |  |  |  |
| -0.2191 | 10 | igd | 0.6498 | A | \|\| id:ebi-a-GCST90002030 | ebi-a-GCST90002030 | TRUE | C | 96822803 | 3.07E-14 | reported | 2920 | 0.02871 | rs10882523 |  |  |  |  |  |  |  |  |  |  |  |  |  |  |  |
| -0.2172 | 10 | igd | 0.6498 | A | \|\| id:ebi-a-GCST90002031 | ebi-a-GCST90002031 | TRUE | C | 96822803 | 1.38E-13 | reported | 2920 | 0.02922 | rs10882523 |  |  |  |  |  |  |  |  |  |  |  |  |  |  |  |
| 0.358 | 22 | igd | 0.6488 | G | \|\| id:ebi-a-GCST90001702 | ebi-a-GCST90001702 | TRUE | T | 41639728 | 1.34E-41 | reported | 3657 | 0.02616 | rs139496 |  |  |  |  |  |  |  |  |  |  |  |  |  |  |  |
| 0.3527 | 22 | igd | 0.6488 | G | \|\| id:ebi-a-GCST90001703 | ebi-a-GCST90001703 | TRUE | T | 41639728 | 2.00E-40 | reported | 3657 | 0.02618 | rs139496 |  |  |  |  |  |  |  |  |  |  |  |  |  |  |  |
| 0.3386 | 22 | igd | 0.6488 | G | \|\| id:ebi-a-GCST90001704 | ebi-a-GCST90001704 | TRUE | T | 41639728 | 1.98E-37 | reported | 3657 | 0.02619 | rs139496 |  |  |  |  |  |  |  |  |  |  |  |  |  |  |  |
| 0.3436 | 22 | igd | 0.6488 | G | \|\| id:ebi-a-GCST90001710 | ebi-a-GCST90001710 | TRUE | T | 41639728 | 1.39E-38 | reported | 3657 | 0.02614 | rs139496 |  |  |  |  |  |  |  |  |  |  |  |  |  |  |  |
| 0.3245 | 22 | igd | 0.6488 | G | \|\| id:ebi-a-GCST90001711 | ebi-a-GCST90001711 | TRUE | T | 41639728 | 1.05E-34 | reported | 3657 | 0.02614 | rs139496 |  |  |  |  |  |  |  |  |  |  |  |  |  |  |  |
| -0.2458 | 6 | igd | 0.648 | G | \|\| id:ebi-a-GCST90002109 | ebi-a-GCST90002109 | TRUE | A | 32696835 | 2.21E-09 | reported | 1578 | 0.04085 | rs2859124 |  |  |  |  |  |  |  |  |  |  |  |  |  |  |  |
| 2.095 | 6 | igd | 0.6474 | C | \|\| id:ebi-a-GCST90001696 | ebi-a-GCST90001696 | TRUE | T | 29704400 | 1.26E-08 | reported | 3425 | 0.3672 | rs1736913 |  |  |  |  |  |  |  |  |  |  |  |  |  |  |  |
| 0.2205 | 10 | igd | 0.6461 | A | \|\| id:ebi-a-GCST90002031 | ebi-a-GCST90002031 | TRUE | C | 98816373 | 6.23E-14 | reported | 2920 | 0.02924 | rs2255174 |  |  |  |  |  |  |  |  |  |  |  |  |  |  |  |
| 0.1809 | 6 | igd | 0.645 | C | \|\| id:ebi-a-GCST90002067 | ebi-a-GCST90002067 | TRUE | A | 31239577 | 6.05E-09 | reported | 2920 | 0.03101 | rs707911 |  |  |  |  |  |  |  |  |  |  |  |  |  |  |  |
| 0.1621 | 1 | igd | 0.6432 | T | \|\| id:ebi-a-GCST90001864 | ebi-a-GCST90001864 | TRUE | C | 166797260 | 1.74E-08 | reported | 2919 | 0.02867 | rs2312237 |  |  |  |  |  |  |  |  |  |  |  |  |  |  |  |
| -0.1746 | 1 | igd | 0.6426 | T | \|\| id:ebi-a-GCST90002035 | ebi-a-GCST90002035 | TRUE | G | 161700638 | 1.05E-09 | reported | 2871 | 0.02852 | rs1503812 |  |  |  |  |  |  |  |  |  |  |  |  |  |  |  |
| 0.1602 | 12 | igd | 0.6421 | T | \|\| id:ebi-a-GCST90002073 | ebi-a-GCST90002073 | TRUE | A | 70113854 | 3.35E-08 | reported | 2850 | 0.02892 | rs710706 |  |  |  |  |  |  |  |  |  |  |  |  |  |  |  |
| 0.2136 | 22 | igd | 0.6418 | T | \|\| id:ebi-a-GCST90001702 | ebi-a-GCST90001702 | TRUE | C | 39843409 | 2.51E-16 | reported | 3657 | 0.02595 | rs5757678 |  |  |  |  |  |  |  |  |  |  |  |  |  |  |  |
| 0.2077 | 22 | igd | 0.6418 | T | \|\| id:ebi-a-GCST90001703 | ebi-a-GCST90001703 | TRUE | C | 39843409 | 1.61E-15 | reported | 3657 | 0.02596 | rs5757678 |  |  |  |  |  |  |  |  |  |  |  |  |  |  |  |
| 0.2191 | 22 | igd | 0.6418 | T | \|\| id:ebi-a-GCST90001704 | ebi-a-GCST90001704 | TRUE | C | 39843409 | 3.82E-17 | reported | 3657 | 0.0259 | rs5757678 |  |  |  |  |  |  |  |  |  |  |  |  |  |  |  |
| 0.1995 | 22 | igd | 0.6418 | T | \|\| id:ebi-a-GCST90001710 | ebi-a-GCST90001710 | TRUE | C | 39843409 | 1.73E-14 | reported | 3657 | 0.0259 | rs5757678 |  |  |  |  |  |  |  |  |  |  |  |  |  |  |  |
| 0.2006 | 22 | igd | 0.6418 | T | \|\| id:ebi-a-GCST90001711 | ebi-a-GCST90001711 | TRUE | C | 39843409 | 1.08E-14 | reported | 3657 | 0.02584 | rs5757678 |  |  |  |  |  |  |  |  |  |  |  |  |  |  |  |
| -0.229 | 10 | igd | 0.6415 | G | \|\| id:ebi-a-GCST90001777 | ebi-a-GCST90001777 | TRUE | T | 6033301 | 1.43E-19 | reported | 3657 | 0.02516 | rs1323651 |  |  |  |  |  |  |  |  |  |  |  |  |  |  |  |
| -0.203 | 10 | igd | 0.6415 | G | \|\| id:ebi-a-GCST90001778 | ebi-a-GCST90001778 | TRUE | T | 6033301 | 1.64E-15 | reported | 3657 | 0.02538 | rs1323651 |  |  |  |  |  |  |  |  |  |  |  |  |  |  |  |
| -0.1615 | 10 | igd | 0.6415 | G | \|\| id:ebi-a-GCST90001779 | ebi-a-GCST90001779 | TRUE | T | 6033301 | 3.72E-10 | reported | 3657 | 0.02571 | rs1323651 |  |  |  |  |  |  |  |  |  |  |  |  |  |  |  |
| -0.1547 | 10 | igd | 0.6415 | G | \|\| id:ebi-a-GCST90001784 | ebi-a-GCST90001784 | TRUE | T | 6033301 | 2.54E-09 | reported | 3657 | 0.02589 | rs1323651 |  |  |  |  |  |  |  |  |  |  |  |  |  |  |  |
| -0.1811 | 10 | igd | 0.6415 | G | \|\| id:ebi-a-GCST90001787 | ebi-a-GCST90001787 | TRUE | T | 6033301 | 1.31E-12 | reported | 3657 | 0.02545 | rs1323651 |  |  |  |  |  |  |  |  |  |  |  |  |  |  |  |
| -0.2207 | 10 | igd | 0.6415 | G | \|\| id:ebi-a-GCST90001789 | ebi-a-GCST90001789 | TRUE | T | 6033301 | 1.69E-18 | reported | 3657 | 0.02502 | rs1323651 |  |  |  |  |  |  |  |  |  |  |  |  |  |  |  |
| -0.2264 | 10 | igd | 0.6415 | G | \|\| id:ebi-a-GCST90001790 | ebi-a-GCST90001790 | TRUE | T | 6033301 | 3.93E-19 | reported | 3657 | 0.02519 | rs1323651 |  |  |  |  |  |  |  |  |  |  |  |  |  |  |  |
| -0.146 | 10 | igd | 0.6415 | G | \|\| id:ebi-a-GCST90001791 | ebi-a-GCST90001791 | TRUE | T | 6033301 | 1.94E-08 | reported | 3657 | 0.02594 | rs1323651 |  |  |  |  |  |  |  |  |  |  |  |  |  |  |  |
| -0.2166 | 10 | igd | 0.6415 | G | \|\| id:ebi-a-GCST90001792 | ebi-a-GCST90001792 | TRUE | T | 6033301 | 1.35E-17 | reported | 3657 | 0.02523 | rs1323651 |  |  |  |  |  |  |  |  |  |  |  |  |  |  |  |
| -0.2239 | 10 | igd | 0.6415 | G | \|\| id:ebi-a-GCST90001793 | ebi-a-GCST90001793 | TRUE | T | 6033301 | 1.78E-18 | reported | 3657 | 0.02539 | rs1323651 |  |  |  |  |  |  |  |  |  |  |  |  |  |  |  |
| -0.159 | 10 | igd | 0.6415 | G | \|\| id:ebi-a-GCST90001794 | ebi-a-GCST90001794 | TRUE | T | 6033301 | 8.42E-10 | reported | 3658 | 0.02583 | rs1323651 |  |  |  |  |  |  |  |  |  |  |  |  |  |  |  |
| -0.1458 | 6 | igd | 0.6414 | G | \|\| id:ebi-a-GCST90001686 | ebi-a-GCST90001686 | TRUE | A | 31192797 | 3.34E-08 | reported | 3440 | 0.02634 | rs6904670 |  |  |  |  |  |  |  |  |  |  |  |  |  |  |  |
| -0.1632 | 10 | igd | 0.6414 | G | \|\| id:ebi-a-GCST90001775 | ebi-a-GCST90001775 | TRUE | T | 6033301 | 2.96E-10 | reported | 3660 | 0.02583 | rs1323651 |  |  |  |  |  |  |  |  |  |  |  |  |  |  |  |
| -0.1729 | 10 | igd | 0.6414 | G | \|\| id:ebi-a-GCST90001780 | ebi-a-GCST90001780 | TRUE | T | 6033301 | 1.65E-11 | reported | 3642 | 0.0256 | rs1323651 |  |  |  |  |  |  |  |  |  |  |  |  |  |  |  |
| -0.1522 | 12 | igd | 0.6411 | A | \|\| id:ebi-a-GCST90002074 | ebi-a-GCST90002074 | TRUE | G | 69524651 | 3.85E-08 | reported | 3112 | 0.02762 | rs11177513 |  |  |  |  |  |  |  |  |  |  |  |  |  |  |  |
| -0.1853 | 12 | igd | 0.6402 | A | \|\| id:ebi-a-GCST90002073 | ebi-a-GCST90002073 | TRUE | G | 69524651 | 1.12E-10 | reported | 2850 | 0.02863 | rs11177513 |  |  |  |  |  |  |  |  |  |  |  |  |  |  |  |
| 0.16 | 10 | igd | 0.6398 | G | \|\| id:ebi-a-GCST90001660 | ebi-a-GCST90001660 | TRUE | A | 98007727 | 1.79E-09 | reported | 3408 | 0.02653 | rs10882762 |  |  |  |  |  |  |  |  |  |  |  |  |  |  |  |
| 0.1482 | 10 | igd | 0.6398 | G | \|\| id:ebi-a-GCST90001672 | ebi-a-GCST90001672 | TRUE | A | 98007727 | 3.46E-08 | reported | 3408 | 0.02681 | rs10882762 |  |  |  |  |  |  |  |  |  |  |  |  |  |  |  |
| -0.2492 | 19 | igd | 0.6393 | G | \|\| id:ebi-a-GCST90001951 | ebi-a-GCST90001951 | TRUE | A | 51608499 | 4.73E-10 | reported | 1464 | 0.03975 | rs2691252 |  |  |  |  |  |  |  |  |  |  |  |  |  |  |  |
| 0.169 | 10 | igd | 0.6392 | G | \|\| id:ebi-a-GCST90001658 | ebi-a-GCST90001658 | TRUE | A | 98007727 | 2.67E-11 | reported | 3440 | 0.02528 | rs10882762 |  |  |  |  |  |  |  |  |  |  |  |  |  |  |  |
| 0.1589 | 10 | igd | 0.6392 | G | \|\| id:ebi-a-GCST90001659 | ebi-a-GCST90001659 | TRUE | A | 98007727 | 3.79E-10 | reported | 3440 | 0.0253 | rs10882762 |  |  |  |  |  |  |  |  |  |  |  |  |  |  |  |
| 0.1497 | 10 | igd | 0.6392 | G | \|\| id:ebi-a-GCST90001670 | ebi-a-GCST90001670 | TRUE | A | 98007727 | 1.47E-08 | reported | 3440 | 0.02636 | rs10882762 |  |  |  |  |  |  |  |  |  |  |  |  |  |  |  |
| 0.1633 | 10 | igd | 0.6392 | G | \|\| id:ebi-a-GCST90001671 | ebi-a-GCST90001671 | TRUE | A | 98007727 | 6.59E-10 | reported | 3440 | 0.02637 | rs10882762 |  |  |  |  |  |  |  |  |  |  |  |  |  |  |  |
| -0.3022 | 19 | igd | 0.6389 | G | \|\| id:ebi-a-GCST90001946 | ebi-a-GCST90001946 | TRUE | A | 51608499 | 5.74E-16 | reported | 1634 | 0.03695 | rs2691252 |  |  |  |  |  |  |  |  |  |  |  |  |  |  |  |
| 0.165 | 1 | igd | 0.6383 | T | \|\| id:ebi-a-GCST90001985 | ebi-a-GCST90001985 | TRUE | G | 161484175 | 3.62E-10 | reported | 3629 | 0.02625 | rs6427596 |  |  |  |  |  |  |  |  |  |  |  |  |  |  |  |
| 0.2745 | 1 | igd | 0.6382 | T | \|\| id:ebi-a-GCST90001989 | ebi-a-GCST90001989 | TRUE | G | 161484175 | 1.66E-25 | reported | 3621 | 0.0261 | rs6427596 |  |  |  |  |  |  |  |  |  |  |  |  |  |  |  |
| 0.1708 | 1 | igd | 0.6379 | T | \|\| id:ebi-a-GCST90001981 | ebi-a-GCST90001981 | TRUE | G | 161484175 | 6.53E-11 | reported | 3618 | 0.02607 | rs6427596 |  |  |  |  |  |  |  |  |  |  |  |  |  |  |  |
| 0.1746 | 6 | igd | 0.6368 | T | \|\| id:ebi-a-GCST90002118 | ebi-a-GCST90002118 | TRUE | C | 31329494 | 2.85E-08 | reported | 2920 | 0.03136 | rs2523572 |  |  |  |  |  |  |  |  |  |  |  |  |  |  |  |
| 0.1431 | 22 | igd | 0.6365 | T | \|\| id:ebi-a-GCST90001710 | ebi-a-GCST90001710 | TRUE | C | 39032270 | 3.96E-08 | reported | 3657 | 0.026 | rs760645 |  |  |  |  |  |  |  |  |  |  |  |  |  |  |  |
| 0.1635 | 10 | igd | 0.6363 | T | \|\| id:ebi-a-GCST90001489 | ebi-a-GCST90001489 | TRUE | C | 98111279 | 7.89E-10 | reported | 3405 | 0.02652 | rs2146392 |  |  |  |  |  |  |  |  |  |  |  |  |  |  |  |
| 0.169 | 10 | igd | 0.6351 | T | \|\| id:ebi-a-GCST90002030 | ebi-a-GCST90002030 | TRUE | C | 100057707 | 2.23E-09 | reported | 2920 | 0.02817 | rs1925636 |  |  |  |  |  |  |  |  |  |  |  |  |  |  |  |
| 0.1443 | 10 | igd | 0.6348 | T | \|\| id:ebi-a-GCST90001660 | ebi-a-GCST90001660 | TRUE | C | 100057707 | 4.92E-08 | reported | 3408 | 0.0264 | rs1925636 |  |  |  |  |  |  |  |  |  |  |  |  |  |  |  |
| -0.12545 | 1 | igd | 0.633479 | A | \|\| id:met-b-150 | met-b-150 | TRUE | G | 161862555 | 3.99E-09 | reported | 457 | 0.0213191 | rs10918137 |  |  |  |  |  |  |  |  |  |  |  |  |  |  |  |
| -0.126214 | 1 | igd | 0.632675 | G | \|\| id:met-b-150 | met-b-150 | TRUE | A | 161739190 | 3.37E-09 | reported | 456 | 0.0213468 | rs7519514 |  |  |  |  |  |  |  |  |  |  |  |  |  |  |  |
| 0.1857 | 10 | igd | 0.6325 | G | \|\| id:ebi-a-GCST90002030 | ebi-a-GCST90002030 | TRUE | A | 98007727 | 3.95E-11 | reported | 2920 | 0.028 | rs10882762 |  |  |  |  |  |  |  |  |  |  |  |  |  |  |  |
| 0.1694 | 6 | igd | 0.6324 | C | \|\| id:ebi-a-GCST90002118 | ebi-a-GCST90002118 | TRUE | G | 30044388 | 4.51E-08 | reported | 2920 | 0.0309 | rs3132682 |  |  |  |  |  |  |  |  |  |  |  |  |  |  |  |
| 0.1699 | 6 | igd | 0.6324 | C | \|\| id:ebi-a-GCST90002119 | ebi-a-GCST90002119 | TRUE | G | 30044388 | 3.14E-08 | reported | 2920 | 0.03062 | rs3132682 |  |  |  |  |  |  |  |  |  |  |  |  |  |  |  |
| -0.1431 | 20 | igd | 0.6323 | C | \|\| id:ebi-a-GCST90001803 | ebi-a-GCST90001803 | TRUE | T | 44746982 | 3.69E-08 | reported | 3642 | 0.02594 | rs1883832 |  |  |  |  |  |  |  |  |  |  |  |  |  |  |  |
| -0.2585 | 19 | igd | 0.6322 | G | \|\| id:ebi-a-GCST90001952 | ebi-a-GCST90001952 | TRUE | A | 51608499 | 2.05E-11 | reported | 1569 | 0.03829 | rs2691252 |  |  |  |  |  |  |  |  |  |  |  |  |  |  |  |
| -0.1869 | 20 | igd | 0.6318 | C | \|\| id:ebi-a-GCST90001754 | ebi-a-GCST90001754 | TRUE | T | 44746982 | 4.90E-13 | reported | 3657 | 0.02576 | rs1883832 |  |  |  |  |  |  |  |  |  |  |  |  |  |  |  |
| -0.5841 | 20 | igd | 0.6318 | C | \|\| id:ebi-a-GCST90001798 | ebi-a-GCST90001798 | TRUE | T | 44746982 | 8.47E-120 | reported | 3657 | 0.02417 | rs1883832 |  |  |  |  |  |  |  |  |  |  |  |  |  |  |  |
| -0.4707 | 20 | igd | 0.6318 | C | \|\| id:ebi-a-GCST90001802 | ebi-a-GCST90001802 | TRUE | T | 44746982 | 3.83E-75 | reported | 3657 | 0.02507 | rs1883832 |  |  |  |  |  |  |  |  |  |  |  |  |  |  |  |
| -0.6404 | 20 | igd | 0.6318 | C | \|\| id:ebi-a-GCST90001804 | ebi-a-GCST90001804 | TRUE | T | 44746982 | 6.21E-147 | reported | 3657 | 0.02368 | rs1883832 |  |  |  |  |  |  |  |  |  |  |  |  |  |  |  |
| -0.5071 | 20 | igd | 0.6318 | C | \|\| id:ebi-a-GCST90001805 | ebi-a-GCST90001805 | TRUE | T | 44746982 | 1.88E-87 | reported | 3657 | 0.0249 | rs1883832 |  |  |  |  |  |  |  |  |  |  |  |  |  |  |  |
| -0.5853 | 20 | igd | 0.6318 | C | \|\| id:ebi-a-GCST90001808 | ebi-a-GCST90001808 | TRUE | T | 44746982 | 2.53E-118 | reported | 3657 | 0.02439 | rs1883832 |  |  |  |  |  |  |  |  |  |  |  |  |  |  |  |
| -0.4972 | 20 | igd | 0.6317 | C | \|\| id:ebi-a-GCST90001800 | ebi-a-GCST90001800 | TRUE | T | 44746982 | 2.98E-83 | reported | 3656 | 0.02506 | rs1883832 |  |  |  |  |  |  |  |  |  |  |  |  |  |  |  |
| 0.1571 | 10 | igd | 0.6317 | T | \|\| id:ebi-a-GCST90002031 | ebi-a-GCST90002031 | TRUE | C | 98111279 | 4.43E-08 | reported | 2920 | 0.02862 | rs2146392 |  |  |  |  |  |  |  |  |  |  |  |  |  |  |  |
| 0.2306 | 20 | igd | 0.6316 | C | \|\| id:ebi-a-GCST90001786 | ebi-a-GCST90001786 | TRUE | T | 44746982 | 3.11E-19 | reported | 3651 | 0.02558 | rs1883832 |  |  |  |  |  |  |  |  |  |  |  |  |  |  |  |
| 0.3898 | 20 | igd | 0.6314 | G | \|\| id:ebi-a-GCST90001399 | ebi-a-GCST90001399 | TRUE | T | 44747947 | 7.20E-50 | reported | 3657 | 0.02585 | rs4810485 |  |  |  |  |  |  |  |  |  |  |  |  |  |  |  |
| 0.317 | 20 | igd | 0.6313 | G | \|\| id:ebi-a-GCST90001433 | ebi-a-GCST90001433 | TRUE | T | 44747947 | 4.98E-33 | reported | 3656 | 0.02622 | rs4810485 |  |  |  |  |  |  |  |  |  |  |  |  |  |  |  |
| -0.4783 | 20 | igd | 0.6313 | G | \|\| id:ebi-a-GCST90001806 | ebi-a-GCST90001806 | TRUE | T | 44747947 | 5.93E-78 | reported | 3656 | 0.02498 | rs4810485 |  |  |  |  |  |  |  |  |  |  |  |  |  |  |  |
| -0.5719 | 20 | igd | 0.6302 | G | \|\| id:ebi-a-GCST90001801 | ebi-a-GCST90001801 | TRUE | T | 44747947 | 1.33E-62 | reported | 1836 | 0.03295 | rs4810485 |  |  |  |  |  |  |  |  |  |  |  |  |  |  |  |
| -0.2262 | 19 | igd | 0.63 | G | \|\| id:ebi-a-GCST90001953 | ebi-a-GCST90001953 | TRUE | A | 51608499 | 4.25E-09 | reported | 1558 | 0.03829 | rs2691252 |  |  |  |  |  |  |  |  |  |  |  |  |  |  |  |
| 0.1794 | 6 | igd | 0.6272 | G | \|\| id:ebi-a-GCST90002118 | ebi-a-GCST90002118 | TRUE | A | 30613182 | 6.38E-09 | reported | 2920 | 0.03081 | rs3094101 |  |  |  |  |  |  |  |  |  |  |  |  |  |  |  |
| 0.1446 | 4 | igd | 0.623 | C | \|\| id:ebi-a-GCST90001692 | ebi-a-GCST90001692 | TRUE | G | 78946913 | 2.58E-08 | reported | 3440 | 0.0259 | rs4368632 |  |  |  |  |  |  |  |  |  |  |  |  |  |  |  |
| -0.1546 | 20 | igd | 0.6225 | T | \|\| id:ebi-a-GCST90001989 | ebi-a-GCST90001989 | TRUE | G | 42815738 | 3.47E-09 | reported | 3621 | 0.02611 | rs6031444 |  |  |  |  |  |  |  |  |  |  |  |  |  |  |  |
| -0.184 | 10 | igd | 0.6207 | G | \|\| id:ebi-a-GCST90001489 | ebi-a-GCST90001489 | TRUE | C | 96163039 | 1.28E-11 | reported | 3405 | 0.02709 | rs2477534 |  |  |  |  |  |  |  |  |  |  |  |  |  |  |  |
| -0.1894 | 10 | igd | 0.6207 | G | \|\| id:ebi-a-GCST90001495 | ebi-a-GCST90001495 | TRUE | C | 96163039 | 6.13E-12 | reported | 3405 | 0.02744 | rs2477534 |  |  |  |  |  |  |  |  |  |  |  |  |  |  |  |
| -0.211 | 10 | igd | 0.6206 | G | \|\| id:ebi-a-GCST90001660 | ebi-a-GCST90001660 | TRUE | C | 96163039 | 9.58E-15 | reported | 3408 | 0.02712 | rs2477534 |  |  |  |  |  |  |  |  |  |  |  |  |  |  |  |
| -0.1846 | 10 | igd | 0.6205 | G | \|\| id:ebi-a-GCST90001491 | ebi-a-GCST90001491 | TRUE | C | 96163039 | 6.34E-12 | reported | 3437 | 0.02677 | rs2477534 |  |  |  |  |  |  |  |  |  |  |  |  |  |  |  |
| -0.191 | 10 | igd | 0.6205 | G | \|\| id:ebi-a-GCST90001496 | ebi-a-GCST90001496 | TRUE | C | 96163039 | 2.29E-12 | reported | 3437 | 0.02712 | rs2477534 |  |  |  |  |  |  |  |  |  |  |  |  |  |  |  |
| -0.187 | 10 | igd | 0.6205 | G | \|\| id:ebi-a-GCST90001497 | ebi-a-GCST90001497 | TRUE | C | 96163039 | 3.42E-12 | reported | 3437 | 0.02678 | rs2477534 |  |  |  |  |  |  |  |  |  |  |  |  |  |  |  |
| -0.1981 | 10 | igd | 0.6203 | G | \|\| id:ebi-a-GCST90001658 | ebi-a-GCST90001658 | TRUE | C | 96163039 | 2.43E-14 | reported | 3440 | 0.02586 | rs2477534 |  |  |  |  |  |  |  |  |  |  |  |  |  |  |  |
| -0.2162 | 10 | igd | 0.6203 | G | \|\| id:ebi-a-GCST90001659 | ebi-a-GCST90001659 | TRUE | C | 96163039 | 8.10E-17 | reported | 3440 | 0.02583 | rs2477534 |  |  |  |  |  |  |  |  |  |  |  |  |  |  |  |
| 0.1584 | 2 | igd | 0.6199 | A | \|\| id:ebi-a-GCST90002119 | ebi-a-GCST90002119 | TRUE | G | 80961494 | 8.82E-09 | reported | 2920 | 0.02746 | rs1837052 |  |  |  |  |  |  |  |  |  |  |  |  |  |  |  |
| -0.1557 | 8 | igd | 0.6189 | C | \|\| id:ebi-a-GCST90002078 | ebi-a-GCST90002078 | TRUE | T | 6692378 | 1.80E-08 | reported | 3112 | 0.02757 | rs2978898 |  |  |  |  |  |  |  |  |  |  |  |  |  |  |  |
| 0.1586 | 22 | igd | 0.6187 | G | \|\| id:ebi-a-GCST90001710 | ebi-a-GCST90001710 | TRUE | T | 41268925 | 2.06E-09 | reported | 3657 | 0.02639 | rs138352 |  |  |  |  |  |  |  |  |  |  |  |  |  |  |  |
| -0.2362 | 10 | igd | 0.6183 | G | \|\| id:ebi-a-GCST90002030 | ebi-a-GCST90002030 | TRUE | C | 96163039 | 7.69E-17 | reported | 2920 | 0.02817 | rs2477534 |  |  |  |  |  |  |  |  |  |  |  |  |  |  |  |
| -0.1719 | 10 | igd | 0.6183 | G | \|\| id:ebi-a-GCST90002031 | ebi-a-GCST90002031 | TRUE | C | 96163039 | 3.04E-09 | reported | 2920 | 0.0289 | rs2477534 |  |  |  |  |  |  |  |  |  |  |  |  |  |  |  |
| -0.1997 | 10 | igd | 0.6183 | G | \|\| id:ebi-a-GCST90002032 | ebi-a-GCST90002032 | TRUE | C | 96163039 | 5.32E-12 | reported | 2920 | 0.02883 | rs2477534 |  |  |  |  |  |  |  |  |  |  |  |  |  |  |  |
| 0.1566 | 22 | igd | 0.6177 | T | \|\| id:ebi-a-GCST90001702 | ebi-a-GCST90001702 | TRUE | G | 41210827 | 3.80E-09 | reported | 3657 | 0.02652 | rs7290331 |  |  |  |  |  |  |  |  |  |  |  |  |  |  |  |
| 0.1554 | 22 | igd | 0.6177 | T | \|\| id:ebi-a-GCST90001703 | ebi-a-GCST90001703 | TRUE | G | 41210827 | 5.01E-09 | reported | 3657 | 0.02651 | rs7290331 |  |  |  |  |  |  |  |  |  |  |  |  |  |  |  |
| 0.1681 | 22 | igd | 0.6177 | T | \|\| id:ebi-a-GCST90001704 | ebi-a-GCST90001704 | TRUE | G | 41210827 | 2.32E-10 | reported | 3657 | 0.02645 | rs7290331 |  |  |  |  |  |  |  |  |  |  |  |  |  |  |  |
| 0.1811 | 3 | igd | 0.6173 | A | \|\| id:ebi-a-GCST90002078 | ebi-a-GCST90002078 | TRUE | C | 50371432 | 2.24E-10 | reported | 3112 | 0.02845 | rs2236947 |  |  |  |  |  |  |  |  |  |  |  |  |  |  |  |
| 0.3526 | 6 | igd | 0.617 | T | \|\| id:ebi-a-GCST90002027 | ebi-a-GCST90002027 | TRUE | C | 32512048 | 2.08E-11 | reported | 2911 | 0.05242 | rs28584364 |  |  |  |  |  |  |  |  |  |  |  |  |  |  |  |
| -0.4244 | 6 | igd | 0.6165 | G | \|\| id:ebi-a-GCST90002105 | ebi-a-GCST90002105 | TRUE | A | 32587525 | 3.22E-40 | reported | 2872 | 0.03148 | rs9271401 |  |  |  |  |  |  |  |  |  |  |  |  |  |  |  |
| 0.2005 | 12 | igd | 0.6155 | G | \|\| id:ebi-a-GCST90002074 | ebi-a-GCST90002074 | TRUE | T | 69368045 | 1.43E-13 | reported | 3112 | 0.02699 | rs1908667 |  |  |  |  |  |  |  |  |  |  |  |  |  |  |  |
| 0.1979 | 12 | igd | 0.6153 | G | \|\| id:ebi-a-GCST90002073 | ebi-a-GCST90002073 | TRUE | T | 69368045 | 3.61E-12 | reported | 2850 | 0.02835 | rs1908667 |  |  |  |  |  |  |  |  |  |  |  |  |  |  |  |
| -0.1599 | 2 | igd | 0.6147 | T | \|\| id:ebi-a-GCST90001896 | ebi-a-GCST90001896 | TRUE | G | 204477552 | 2.05E-08 | reported | 2920 | 0.02843 | rs7583807 |  |  |  |  |  |  |  |  |  |  |  |  |  |  |  |
| -0.1555 | 10 | igd | 0.6146 | A | \|\| id:ebi-a-GCST90001780 | ebi-a-GCST90001780 | TRUE | G | 6079846 | 1.52E-09 | reported | 3642 | 0.02567 | rs7072398 |  |  |  |  |  |  |  |  |  |  |  |  |  |  |  |
| -0.235 | 10 | igd | 0.6142 | A | \|\| id:ebi-a-GCST90001790 | ebi-a-GCST90001790 | TRUE | G | 6079846 | 2.02E-20 | reported | 3657 | 0.02523 | rs7072398 |  |  |  |  |  |  |  |  |  |  |  |  |  |  |  |
| -0.1432 | 10 | igd | 0.6141 | A | \|\| id:ebi-a-GCST90001775 | ebi-a-GCST90001775 | TRUE | G | 6079846 | 3.59E-08 | reported | 3660 | 0.02593 | rs7072398 |  |  |  |  |  |  |  |  |  |  |  |  |  |  |  |
| -0.1803 | 1 | igd | 0.61344 | C | \|\| id:prot-a-427 | prot-a-427 | TRUE | T | 196671981 | 1.41E-12 | reported | 3301 | 0.0254 | rs368465 |  |  |  |  |  |  |  |  |  |  |  |  |  |  |  |
| 0.3732 | 6 | igd | 0.6131 | T | \|\| id:ebi-a-GCST90002111 | ebi-a-GCST90002111 | TRUE | C | 32662389 | 5.50E-20 | reported | 1635 | 0.04025 | rs9275265 |  |  |  |  |  |  |  |  |  |  |  |  |  |  |  |
| 0.4085 | 6 | igd | 0.6129 | T | \|\| id:ebi-a-GCST90002110 | ebi-a-GCST90002110 | TRUE | C | 32662389 | 5.43E-23 | reported | 1634 | 0.04075 | rs9275265 |  |  |  |  |  |  |  |  |  |  |  |  |  |  |  |
| -0.1537 | 22 | igd | 0.6112 | T | \|\| id:ebi-a-GCST90001702 | ebi-a-GCST90001702 | TRUE | C | 42691238 | 3.80E-09 | reported | 3657 | 0.02603 | rs1548304 |  |  |  |  |  |  |  |  |  |  |  |  |  |  |  |
| -0.1578 | 22 | igd | 0.6112 | T | \|\| id:ebi-a-GCST90001703 | ebi-a-GCST90001703 | TRUE | C | 42691238 | 1.45E-09 | reported | 3657 | 0.02602 | rs1548304 |  |  |  |  |  |  |  |  |  |  |  |  |  |  |  |
| -0.1468 | 22 | igd | 0.6112 | T | \|\| id:ebi-a-GCST90001704 | ebi-a-GCST90001704 | TRUE | C | 42691238 | 1.78E-08 | reported | 3657 | 0.026 | rs1548304 |  |  |  |  |  |  |  |  |  |  |  |  |  |  |  |
| -0.147 | 22 | igd | 0.6112 | T | \|\| id:ebi-a-GCST90001710 | ebi-a-GCST90001710 | TRUE | C | 42691238 | 1.61E-08 | reported | 3657 | 0.02597 | rs1548304 |  |  |  |  |  |  |  |  |  |  |  |  |  |  |  |
| -0.1439 | 22 | igd | 0.6112 | T | \|\| id:ebi-a-GCST90001711 | ebi-a-GCST90001711 | TRUE | C | 42691238 | 3.00E-08 | reported | 3657 | 0.02591 | rs1548304 |  |  |  |  |  |  |  |  |  |  |  |  |  |  |  |
| 0.1475 | 16 | igd | 0.611 | G | \|\| id:ebi-a-GCST90001736 | ebi-a-GCST90001736 | TRUE | A | 28973268 | 7.89E-09 | reported | 3657 | 0.02551 | rs11643297 |  |  |  |  |  |  |  |  |  |  |  |  |  |  |  |
| -0.3523 | 6 | igd | 0.611 | G | \|\| id:ebi-a-GCST90002112 | ebi-a-GCST90002112 | TRUE | A | 32587525 | 4.04E-18 | reported | 1635 | 0.04013 | rs9271401 |  |  |  |  |  |  |  |  |  |  |  |  |  |  |  |
| -0.1799 | 6 | igd | 0.611 | G | \|\| id:ebi-a-GCST90002121 | ebi-a-GCST90002121 | TRUE | A | 29893676 | 1.12E-08 | reported | 2915 | 0.0314 | rs1611434 |  |  |  |  |  |  |  |  |  |  |  |  |  |  |  |
| 0.148 | 16 | igd | 0.6109 | G | \|\| id:ebi-a-GCST90001724 | ebi-a-GCST90001724 | TRUE | A | 28973268 | 7.82E-09 | reported | 3656 | 0.02557 | rs11643297 |  |  |  |  |  |  |  |  |  |  |  |  |  |  |  |
| -0.1614 | 10 | igd | 0.6108 | T | \|\| id:ebi-a-GCST90002030 | ebi-a-GCST90002030 | TRUE | A | 96848147 | 1.30E-08 | reported | 2920 | 0.02831 | rs1326827 |  |  |  |  |  |  |  |  |  |  |  |  |  |  |  |
| -0.1999 | 6 | igd | 0.6108 | G | \|\| id:ebi-a-GCST90002118 | ebi-a-GCST90002118 | TRUE | A | 29893676 | 2.35E-09 | reported | 2920 | 0.03337 | rs1611434 |  |  |  |  |  |  |  |  |  |  |  |  |  |  |  |
| -0.1869 | 6 | igd | 0.6108 | G | \|\| id:ebi-a-GCST90002119 | ebi-a-GCST90002119 | TRUE | A | 29893676 | 1.85E-08 | reported | 2920 | 0.03312 | rs1611434 |  |  |  |  |  |  |  |  |  |  |  |  |  |  |  |
| -0.1958 | 6 | igd | 0.6108 | G | \|\| id:ebi-a-GCST90002120 | ebi-a-GCST90002120 | TRUE | A | 29893676 | 1.69E-09 | reported | 2920 | 0.03239 | rs1611434 |  |  |  |  |  |  |  |  |  |  |  |  |  |  |  |
| -0.1785 | 11 | igd | 0.6102 | T | \|\| id:ebi-a-GCST90001751 | ebi-a-GCST90001751 | TRUE | C | 60266070 | 6.73E-13 | reported | 3657 | 0.02475 | rs7939177 |  |  |  |  |  |  |  |  |  |  |  |  |  |  |  |
| -0.1877 | 11 | igd | 0.6102 | T | \|\| id:ebi-a-GCST90001763 | ebi-a-GCST90001763 | TRUE | C | 60266070 | 7.12E-14 | reported | 3657 | 0.02497 | rs7939177 |  |  |  |  |  |  |  |  |  |  |  |  |  |  |  |
| -0.3893 | 6 | igd | 0.6089 | C | \|\| id:ebi-a-GCST90002110 | ebi-a-GCST90002110 | TRUE | A | 32405865 | 2.80E-22 | reported | 1634 | 0.03952 | rs9268628 |  |  |  |  |  |  |  |  |  |  |  |  |  |  |  |
| -0.3864 | 6 | igd | 0.6089 | C | \|\| id:ebi-a-GCST90002111 | ebi-a-GCST90002111 | TRUE | A | 32405865 | 1.32E-22 | reported | 1635 | 0.03891 | rs9268628 |  |  |  |  |  |  |  |  |  |  |  |  |  |  |  |
| -0.2046 | 11 | igd | 0.6088 | G | \|\| id:ebi-a-GCST90001747 | ebi-a-GCST90001747 | TRUE | A | 60273642 | 2.29E-16 | reported | 3657 | 0.02482 | rs4939384 |  |  |  |  |  |  |  |  |  |  |  |  |  |  |  |
| -0.236 | 11 | igd | 0.6088 | G | \|\| id:ebi-a-GCST90001752 | ebi-a-GCST90001752 | TRUE | A | 60273642 | 2.50E-21 | reported | 3657 | 0.02474 | rs4939384 |  |  |  |  |  |  |  |  |  |  |  |  |  |  |  |
| -0.2011 | 11 | igd | 0.6088 | G | \|\| id:ebi-a-GCST90001762 | ebi-a-GCST90001762 | TRUE | A | 60273642 | 7.90E-16 | reported | 3658 | 0.02485 | rs4939384 |  |  |  |  |  |  |  |  |  |  |  |  |  |  |  |
| -0.1622 | 10 | igd | 0.6085 | C | \|\| id:ebi-a-GCST90001783 | ebi-a-GCST90001783 | TRUE | T | 6098824 | 1.56E-10 | reported | 3656 | 0.02528 | rs706779 |  |  |  |  |  |  |  |  |  |  |  |  |  |  |  |
| -0.1403 | 11 | igd | 0.6084 | T | \|\| id:ebi-a-GCST90001748 | ebi-a-GCST90001748 | TRUE | C | 60261906 | 1.61E-08 | reported | 3645 | 0.02478 | rs4939376 |  |  |  |  |  |  |  |  |  |  |  |  |  |  |  |
| -0.293 | 10 | igd | 0.6084 | C | \|\| id:ebi-a-GCST90001787 | ebi-a-GCST90001787 | TRUE | T | 6098824 | 6.64E-31 | reported | 3657 | 0.02511 | rs706779 |  |  |  |  |  |  |  |  |  |  |  |  |  |  |  |
| -0.3421 | 10 | igd | 0.6084 | C | \|\| id:ebi-a-GCST90001793 | ebi-a-GCST90001793 | TRUE | T | 6098824 | 1.19E-41 | reported | 3657 | 0.02499 | rs706779 |  |  |  |  |  |  |  |  |  |  |  |  |  |  |  |
| -0.1965 | 11 | igd | 0.6081 | G | \|\| id:ebi-a-GCST90001759 | ebi-a-GCST90001759 | TRUE | A | 60268994 | 3.16E-15 | reported | 3657 | 0.02481 | rs1941018 |  |  |  |  |  |  |  |  |  |  |  |  |  |  |  |
| -0.1417 | 11 | igd | 0.608 | C | \|\| id:ebi-a-GCST90001745 | ebi-a-GCST90001745 | TRUE | T | 60267695 | 1.32E-08 | reported | 3657 | 0.02488 | rs7933244 |  |  |  |  |  |  |  |  |  |  |  |  |  |  |  |
| -0.1458 | 11 | igd | 0.608 | C | \|\| id:ebi-a-GCST90001758 | ebi-a-GCST90001758 | TRUE | T | 60267695 | 4.77E-09 | reported | 3657 | 0.02484 | rs7933244 |  |  |  |  |  |  |  |  |  |  |  |  |  |  |  |
| -0.1808 | 11 | igd | 0.6079 | G | \|\| id:ebi-a-GCST90001743 | ebi-a-GCST90001743 | TRUE | A | 60268994 | 4.63E-13 | reported | 3660 | 0.02489 | rs1941018 |  |  |  |  |  |  |  |  |  |  |  |  |  |  |  |
| 0.1732 | 10 | igd | 0.6077 | G | \|\| id:ebi-a-GCST90002030 | ebi-a-GCST90002030 | TRUE | A | 93070898 | 8.35E-10 | reported | 2920 | 0.02812 | rs2648722 |  |  |  |  |  |  |  |  |  |  |  |  |  |  |  |
| 0.2041 | 16 | igd | 0.6074 | C | \|\| id:ebi-a-GCST90002110 | ebi-a-GCST90002110 | TRUE | T | 10935149 | 2.83E-08 | reported | 1634 | 0.03658 | rs11074930 |  |  |  |  |  |  |  |  |  |  |  |  |  |  |  |
| 0.2058 | 16 | igd | 0.6073 | C | \|\| id:ebi-a-GCST90002111 | ebi-a-GCST90002111 | TRUE | T | 10935149 | 1.53E-08 | reported | 1635 | 0.03618 | rs11074930 |  |  |  |  |  |  |  |  |  |  |  |  |  |  |  |
| -0.1477 | 10 | igd | 0.6072 | A | \|\| id:ebi-a-GCST90001506 | ebi-a-GCST90001506 | TRUE | T | 6171689 | 2.24E-08 | reported | 3437 | 0.02636 | rs4291579 |  |  |  |  |  |  |  |  |  |  |  |  |  |  |  |
| -0.161 | 10 | igd | 0.6072 | A | \|\| id:ebi-a-GCST90001672 | ebi-a-GCST90001672 | TRUE | G | 94989859 | 2.85E-09 | reported | 3408 | 0.02703 | rs10786078 |  |  |  |  |  |  |  |  |  |  |  |  |  |  |  |
| -0.1578 | 10 | igd | 0.6068 | A | \|\| id:ebi-a-GCST90001671 | ebi-a-GCST90001671 | TRUE | G | 94989859 | 3.35E-09 | reported | 3440 | 0.02661 | rs10786078 |  |  |  |  |  |  |  |  |  |  |  |  |  |  |  |
| 0.149 | 10 | igd | 0.6067 | G | \|\| id:ebi-a-GCST90001658 | ebi-a-GCST90001658 | TRUE | A | 93070898 | 2.74E-09 | reported | 3440 | 0.02498 | rs2648722 |  |  |  |  |  |  |  |  |  |  |  |  |  |  |  |
| 0.1457 | 10 | igd | 0.6067 | G | \|\| id:ebi-a-GCST90001659 | ebi-a-GCST90001659 | TRUE | A | 93070898 | 6.15E-09 | reported | 3440 | 0.02499 | rs2648722 |  |  |  |  |  |  |  |  |  |  |  |  |  |  |  |
| -0.1691 | 10 | igd | 0.6067 | T | \|\| id:ebi-a-GCST90001670 | ebi-a-GCST90001670 | TRUE | G | 94994118 | 2.07E-10 | reported | 3440 | 0.02652 | rs10786082 |  |  |  |  |  |  |  |  |  |  |  |  |  |  |  |
| -0.1391 | 2 | igd | 0.6052 | T | \|\| id:ebi-a-GCST90001460 | ebi-a-GCST90001460 | TRUE | C | 136022798 | 3.04E-08 | reported | 3374 | 0.02505 | rs935613 |  |  |  |  |  |  |  |  |  |  |  |  |  |  |  |
| -0.1422 | 2 | igd | 0.6042 | C | \|\| id:ebi-a-GCST90001460 | ebi-a-GCST90001460 | TRUE | G | 136140304 | 1.60E-08 | reported | 3374 | 0.0251 | rs6430571 |  |  |  |  |  |  |  |  |  |  |  |  |  |  |  |
| 0.1886 | 1 | igd | 0.6037 | C | \|\| id:ebi-a-GCST90002077 | ebi-a-GCST90002077 | TRUE | G | 161189344 | 9.40E-12 | reported | 2971 | 0.02756 | rs4634925 |  |  |  |  |  |  |  |  |  |  |  |  |  |  |  |
| -0.1766 | 6 | igd | 0.6029 | T | \|\| id:ebi-a-GCST90001762 | ebi-a-GCST90001762 | TRUE | C | 32635435 | 1.15E-08 | reported | 3658 | 0.03088 | rs9274594 |  |  |  |  |  |  |  |  |  |  |  |  |  |  |  |
| -0.1389 | 22 | igd | 0.6024 | T | \|\| id:ebi-a-GCST90001752 | ebi-a-GCST90001752 | TRUE | C | 39781585 | 3.34E-08 | reported | 3657 | 0.02509 | rs1007337 |  |  |  |  |  |  |  |  |  |  |  |  |  |  |  |
| -0.185 | 1 | igd | 0.6023 | C | \|\| id:ebi-a-GCST90002014 | ebi-a-GCST90002014 | TRUE | G | 161189344 | 8.21E-11 | reported | 2870 | 0.02837 | rs4634925 |  |  |  |  |  |  |  |  |  |  |  |  |  |  |  |
| -0.2159 | 6 | igd | 0.6016 | A | \|\| id:ebi-a-GCST90002110 | ebi-a-GCST90002110 | TRUE | G | 33500853 | 2.21E-08 | reported | 1634 | 0.0384 | rs385251 |  |  |  |  |  |  |  |  |  |  |  |  |  |  |  |
| 0.3858 | 2 | igd | 0.601 | G | \|\| id:ebi-a-GCST90001894 | ebi-a-GCST90001894 | TRUE | T | 204631016 | 2.24E-42 | reported | 2920 | 0.02783 | rs1973872 |  |  |  |  |  |  |  |  |  |  |  |  |  |  |  |
| 0.3661 | 2 | igd | 0.601 | G | \|\| id:ebi-a-GCST90001898 | ebi-a-GCST90001898 | TRUE | T | 204631016 | 3.15E-38 | reported | 2920 | 0.02791 | rs1973872 |  |  |  |  |  |  |  |  |  |  |  |  |  |  |  |
| -0.2903 | 6 | igd | 0.6006 | A | \|\| id:ebi-a-GCST90002105 | ebi-a-GCST90002105 | TRUE | T | 32804553 | 1.21E-21 | reported | 2872 | 0.03013 | rs241426 |  |  |  |  |  |  |  |  |  |  |  |  |  |  |  |
| -0.1501 | 20 | igd | 0.6005 | C | \|\| id:ebi-a-GCST90001989 | ebi-a-GCST90001989 | TRUE | T | 44483225 | 6.84E-09 | reported | 3621 | 0.02584 | rs6130947 |  |  |  |  |  |  |  |  |  |  |  |  |  |  |  |
| -0.1568 | 20 | igd | 0.6003 | C | \|\| id:ebi-a-GCST90001985 | ebi-a-GCST90001985 | TRUE | T | 44483225 | 1.31E-09 | reported | 3629 | 0.02578 | rs6130947 |  |  |  |  |  |  |  |  |  |  |  |  |  |  |  |
| 0.2841 | 20 | igd | 0.6002 | C | \|\| id:ebi-a-GCST90001401 | ebi-a-GCST90001401 | TRUE | T | 44749251 | 1.64E-27 | reported | 3656 | 0.02593 | rs4239702 |  |  |  |  |  |  |  |  |  |  |  |  |  |  |  |
| 0.159 | 1 | igd | 0.6002 | G | \|\| id:ebi-a-GCST90001911 | ebi-a-GCST90001911 | TRUE | C | 226420536 | 1.39E-08 | reported | 3113 | 0.02795 | rs2377027 |  |  |  |  |  |  |  |  |  |  |  |  |  |  |  |
| -0.1625 | 20 | igd | 0.5999 | C | \|\| id:ebi-a-GCST90001981 | ebi-a-GCST90001981 | TRUE | T | 44483225 | 2.57E-10 | reported | 3618 | 0.02563 | rs6130947 |  |  |  |  |  |  |  |  |  |  |  |  |  |  |  |
| 0.1396 | 6 | igd | 0.5998 | A | \|\| id:ebi-a-GCST90001766 | ebi-a-GCST90001766 | TRUE | G | 106898421 | 3.86E-08 | reported | 3638 | 0.02534 | rs2236073 |  |  |  |  |  |  |  |  |  |  |  |  |  |  |  |
| 0.1681 | 6 | igd | 0.5997 | A | \|\| id:ebi-a-GCST90001765 | ebi-a-GCST90001765 | TRUE | G | 106898421 | 1.54E-11 | reported | 3655 | 0.02484 | rs2236073 |  |  |  |  |  |  |  |  |  |  |  |  |  |  |  |
| 0.1623 | 6 | igd | 0.5997 | A | \|\| id:ebi-a-GCST90001772 | ebi-a-GCST90001772 | TRUE | G | 106898421 | 9.31E-11 | reported | 3655 | 0.02499 | rs2236073 |  |  |  |  |  |  |  |  |  |  |  |  |  |  |  |
| 0.1709 | 6 | igd | 0.5997 | A | \|\| id:ebi-a-GCST90001773 | ebi-a-GCST90001773 | TRUE | G | 106898421 | 1.46E-11 | reported | 3652 | 0.02524 | rs2236073 |  |  |  |  |  |  |  |  |  |  |  |  |  |  |  |
| 0.1798 | 6 | igd | 0.5996 | A | \|\| id:ebi-a-GCST90001769 | ebi-a-GCST90001769 | TRUE | G | 106898421 | 1.36E-12 | reported | 3648 | 0.02528 | rs2236073 |  |  |  |  |  |  |  |  |  |  |  |  |  |  |  |
| 0.1799 | 6 | igd | 0.5996 | A | \|\| id:ebi-a-GCST90001771 | ebi-a-GCST90001771 | TRUE | G | 106898421 | 6.00E-13 | reported | 3653 | 0.0249 | rs2236073 |  |  |  |  |  |  |  |  |  |  |  |  |  |  |  |
| -0.1776 | 6 | igd | 0.5991 | A | \|\| id:ebi-a-GCST90002105 | ebi-a-GCST90002105 | TRUE | G | 31316482 | 3.28E-08 | reported | 2872 | 0.03206 | rs9265992 |  |  |  |  |  |  |  |  |  |  |  |  |  |  |  |
| -0.2857 | 6 | igd | 0.5991 | A | \|\| id:ebi-a-GCST90002108 | ebi-a-GCST90002108 | TRUE | G | 33500853 | 1.46E-13 | reported | 1579 | 0.03831 | rs385251 |  |  |  |  |  |  |  |  |  |  |  |  |  |  |  |
| -0.1586 | 10 | igd | 0.599 | T | \|\| id:ebi-a-GCST90002030 | ebi-a-GCST90002030 | TRUE | A | 91737647 | 1.53E-08 | reported | 2920 | 0.02795 | rs4933167 |  |  |  |  |  |  |  |  |  |  |  |  |  |  |  |
| 0.1678 | 16 | igd | 0.5987 | G | \|\| id:ebi-a-GCST90002028 | ebi-a-GCST90002028 | TRUE | C | 28944396 | 7.30E-10 | reported | 3112 | 0.02716 | rs2904880 |  |  |  |  |  |  |  |  |  |  |  |  |  |  |  |
| 0.1434 | 16 | igd | 0.5982 | C | \|\| id:ebi-a-GCST90001733 | ebi-a-GCST90001733 | TRUE | T | 28913787 | 1.07E-08 | reported | 3657 | 0.025 | rs7189927 |  |  |  |  |  |  |  |  |  |  |  |  |  |  |  |
| 0.167 | 16 | igd | 0.5982 | C | \|\| id:ebi-a-GCST90001740 | ebi-a-GCST90001740 | TRUE | T | 28913787 | 2.98E-11 | reported | 3657 | 0.02504 | rs7189927 |  |  |  |  |  |  |  |  |  |  |  |  |  |  |  |
| -0.225 | 6 | igd | 0.5982 | G | \|\| id:ebi-a-GCST90002110 | ebi-a-GCST90002110 | TRUE | A | 29935108 | 1.84E-08 | reported | 1634 | 0.03978 | rs9260757 |  |  |  |  |  |  |  |  |  |  |  |  |  |  |  |
| 0.1583 | 16 | igd | 0.5981 | C | \|\| id:ebi-a-GCST90001723 | ebi-a-GCST90001723 | TRUE | T | 28913787 | 2.70E-10 | reported | 3656 | 0.025 | rs7189927 |  |  |  |  |  |  |  |  |  |  |  |  |  |  |  |
| -0.1716 | 1 | igd | 0.5978 | A | \|\| id:ebi-a-GCST90001863 | ebi-a-GCST90001863 | TRUE | G | 167100361 | 3.60E-10 | reported | 2919 | 0.02727 | rs7519927 |  |  |  |  |  |  |  |  |  |  |  |  |  |  |  |
| -0.1609 | 1 | igd | 0.5976 | A | \|\| id:ebi-a-GCST90001866 | ebi-a-GCST90001866 | TRUE | G | 167100361 | 5.20E-09 | reported | 2914 | 0.02747 | rs7519927 |  |  |  |  |  |  |  |  |  |  |  |  |  |  |  |
| -0.1432 | 22 | igd | 0.5975 | T | \|\| id:ebi-a-GCST90001702 | ebi-a-GCST90001702 | TRUE | C | 43145764 | 2.58E-08 | reported | 3657 | 0.02566 | rs1969673 |  |  |  |  |  |  |  |  |  |  |  |  |  |  |  |
| -0.1409 | 22 | igd | 0.5975 | T | \|\| id:ebi-a-GCST90001703 | ebi-a-GCST90001703 | TRUE | C | 43145764 | 4.25E-08 | reported | 3657 | 0.02566 | rs1969673 |  |  |  |  |  |  |  |  |  |  |  |  |  |  |  |
| -0.1414 | 22 | igd | 0.5975 | T | \|\| id:ebi-a-GCST90001704 | ebi-a-GCST90001704 | TRUE | C | 43145764 | 3.65E-08 | reported | 3657 | 0.02563 | rs1969673 |  |  |  |  |  |  |  |  |  |  |  |  |  |  |  |
| -0.1492 | 22 | igd | 0.5975 | T | \|\| id:ebi-a-GCST90001710 | ebi-a-GCST90001710 | TRUE | C | 43145764 | 5.91E-09 | reported | 3657 | 0.02558 | rs1969673 |  |  |  |  |  |  |  |  |  |  |  |  |  |  |  |
| 0.2283 | 19 | igd | 0.5972 | T | \|\| id:ebi-a-GCST90001533 | ebi-a-GCST90001533 | TRUE | C | 51817416 | 1.20E-11 | reported | 1857 | 0.03346 | rs4802781 |  |  |  |  |  |  |  |  |  |  |  |  |  |  |  |
| -0.2199 | 10 | igd | 0.597 | T | \|\| id:ebi-a-GCST90001672 | ebi-a-GCST90001672 | TRUE | C | 98134403 | 3.18E-16 | reported | 3408 | 0.0268 | rs3827862 |  |  |  |  |  |  |  |  |  |  |  |  |  |  |  |
| -0.2006 | 10 | igd | 0.5969 | A | \|\| id:ebi-a-GCST90001782 | ebi-a-GCST90001782 | TRUE | G | 6079846 | 3.07E-09 | reported | 1837 | 0.03368 | rs7072398 |  |  |  |  |  |  |  |  |  |  |  |  |  |  |  |
| 0.1724 | 16 | igd | 0.5968 | G | \|\| id:ebi-a-GCST90001735 | ebi-a-GCST90001735 | TRUE | C | 28944396 | 6.57E-12 | reported | 3657 | 0.02503 | rs2904880 |  |  |  |  |  |  |  |  |  |  |  |  |  |  |  |
| -0.1537 | 22 | igd | 0.5967 | G | \|\| id:ebi-a-GCST90001910 | ebi-a-GCST90001910 | TRUE | A | 39838018 | 4.14E-09 | reported | 3112 | 0.02608 | rs5750829 |  |  |  |  |  |  |  |  |  |  |  |  |  |  |  |
| -0.2089 | 10 | igd | 0.5961 | T | \|\| id:ebi-a-GCST90001670 | ebi-a-GCST90001670 | TRUE | C | 98134403 | 2.95E-15 | reported | 3440 | 0.02634 | rs3827862 |  |  |  |  |  |  |  |  |  |  |  |  |  |  |  |
| -0.1952 | 10 | igd | 0.5961 | T | \|\| id:ebi-a-GCST90001671 | ebi-a-GCST90001671 | TRUE | C | 98134403 | 1.84E-13 | reported | 3440 | 0.02642 | rs3827862 |  |  |  |  |  |  |  |  |  |  |  |  |  |  |  |
| 0.2114 | 6 | igd | 0.5961 | A | \|\| id:ebi-a-GCST90001767 | ebi-a-GCST90001767 | TRUE | G | 106898421 | 2.92E-09 | reported | 1832 | 0.03543 | rs2236073 |  |  |  |  |  |  |  |  |  |  |  |  |  |  |  |
| 0.1592 | 1 | igd | 0.5958 | A | \|\| id:ebi-a-GCST90002035 | ebi-a-GCST90002035 | TRUE | G | 161574548 | 2.05E-08 | reported | 2871 | 0.02831 | rs2305972 |  |  |  |  |  |  |  |  |  |  |  |  |  |  |  |
| -0.1452 | 22 | igd | 0.5953 | A | \|\| id:ebi-a-GCST90001702 | ebi-a-GCST90001702 | TRUE | G | 41325068 | 3.40E-08 | reported | 3657 | 0.02625 | rs2899341 |  |  |  |  |  |  |  |  |  |  |  |  |  |  |  |
| -0.1444 | 22 | igd | 0.5953 | A | \|\| id:ebi-a-GCST90001703 | ebi-a-GCST90001703 | TRUE | G | 41325068 | 4.03E-08 | reported | 3657 | 0.02624 | rs2899341 |  |  |  |  |  |  |  |  |  |  |  |  |  |  |  |
| -0.1458 | 22 | igd | 0.5953 | A | \|\| id:ebi-a-GCST90001704 | ebi-a-GCST90001704 | TRUE | G | 41325068 | 2.79E-08 | reported | 3657 | 0.0262 | rs2899341 |  |  |  |  |  |  |  |  |  |  |  |  |  |  |  |
| 0.26 | 10 | igd | 0.594 | T | \|\| id:ebi-a-GCST90001483 | ebi-a-GCST90001483 | TRUE | C | 98002169 | 1.38E-24 | reported | 3405 | 0.02521 | rs10882760 |  |  |  |  |  |  |  |  |  |  |  |  |  |  |  |
| 0.2667 | 6 | igd | 0.594 | A | \|\| id:ebi-a-GCST90002108 | ebi-a-GCST90002108 | TRUE | G | 32212694 | 7.50E-11 | reported | 1579 | 0.04068 | rs9267952 |  |  |  |  |  |  |  |  |  |  |  |  |  |  |  |
| 0.2688 | 10 | igd | 0.5938 | T | \|\| id:ebi-a-GCST90001485 | ebi-a-GCST90001485 | TRUE | C | 98002169 | 1.03E-26 | reported | 3437 | 0.02492 | rs10882760 |  |  |  |  |  |  |  |  |  |  |  |  |  |  |  |
| -0.3337 | 2 | igd | 0.593 | A | \|\| id:ebi-a-GCST90002098 | ebi-a-GCST90002098 | TRUE | C | 39112166 | 3.95E-36 | reported | 2910 | 0.02623 | rs11124653 |  |  |  |  |  |  |  |  |  |  |  |  |  |  |  |
| -0.2555 | 2 | igd | 0.5925 | A | \|\| id:ebi-a-GCST90002102 | ebi-a-GCST90002102 | TRUE | C | 39112166 | 6.84E-22 | reported | 2919 | 0.02636 | rs11124653 |  |  |  |  |  |  |  |  |  |  |  |  |  |  |  |
| -0.1418 | 2 | igd | 0.5911 | A | \|\| id:ebi-a-GCST90001485 | ebi-a-GCST90001485 | TRUE | C | 39112166 | 3.21E-08 | reported | 3437 | 0.02559 | rs11124653 |  |  |  |  |  |  |  |  |  |  |  |  |  |  |  |
| -0.2138 | 2 | igd | 0.5911 | A | \|\| id:ebi-a-GCST90001508 | ebi-a-GCST90001508 | TRUE | C | 39112166 | 5.23E-16 | reported | 3437 | 0.02624 | rs11124653 |  |  |  |  |  |  |  |  |  |  |  |  |  |  |  |
| -0.1952 | 2 | igd | 0.5911 | A | \|\| id:ebi-a-GCST90001509 | ebi-a-GCST90001509 | TRUE | C | 39112166 | 1.36E-13 | reported | 3437 | 0.02627 | rs11124653 |  |  |  |  |  |  |  |  |  |  |  |  |  |  |  |
| 0.1741 | 10 | igd | 0.5908 | T | \|\| id:ebi-a-GCST90001888 | ebi-a-GCST90001888 | TRUE | A | 98001826 | 1.57E-10 | reported | 2920 | 0.02712 | rs6584060 |  |  |  |  |  |  |  |  |  |  |  |  |  |  |  |
| 0.2321 | 10 | igd | 0.5908 | T | \|\| id:ebi-a-GCST90001892 | ebi-a-GCST90001892 | TRUE | A | 98001826 | 4.71E-17 | reported | 2920 | 0.02748 | rs6584060 |  |  |  |  |  |  |  |  |  |  |  |  |  |  |  |
| 0.2456 | 10 | igd | 0.5908 | T | \|\| id:ebi-a-GCST90002061 | ebi-a-GCST90002061 | TRUE | A | 98001826 | 2.15E-18 | reported | 2920 | 0.02789 | rs6584060 |  |  |  |  |  |  |  |  |  |  |  |  |  |  |  |
| -0.1861 | 2 | igd | 0.5907 | A | \|\| id:ebi-a-GCST90001507 | ebi-a-GCST90001507 | TRUE | C | 39112166 | 3.74E-12 | reported | 3405 | 0.0267 | rs11124653 |  |  |  |  |  |  |  |  |  |  |  |  |  |  |  |
| 0.185 | 6 | igd | 0.5897 | T | \|\| id:ebi-a-GCST90002061 | ebi-a-GCST90002061 | TRUE | C | 31234381 | 1.61E-09 | reported | 2920 | 0.03057 | rs9264532 |  |  |  |  |  |  |  |  |  |  |  |  |  |  |  |
| 0.1487 | 10 | igd | 0.5889 | A | \|\| id:ebi-a-GCST90001484 | ebi-a-GCST90001484 | TRUE | T | 98582573 | 7.73E-09 | reported | 3437 | 0.02569 | rs2790464 |  |  |  |  |  |  |  |  |  |  |  |  |  |  |  |
| -0.195 | 6 | igd | 0.5885 | A | \|\| id:ebi-a-GCST90002118 | ebi-a-GCST90002118 | TRUE | C | 29603136 | 1.15E-10 | reported | 2920 | 0.03014 | rs3095270 |  |  |  |  |  |  |  |  |  |  |  |  |  |  |  |
| -0.1618 | 6 | igd | 0.5885 | A | \|\| id:ebi-a-GCST90002120 | ebi-a-GCST90002120 | TRUE | C | 29603136 | 4.73E-08 | reported | 2920 | 0.02955 | rs3095270 |  |  |  |  |  |  |  |  |  |  |  |  |  |  |  |
| -0.1747 | 6 | igd | 0.5885 | A | \|\| id:ebi-a-GCST90002121 | ebi-a-GCST90002121 | TRUE | C | 29603136 | 1.30E-09 | reported | 2915 | 0.02871 | rs3095270 |  |  |  |  |  |  |  |  |  |  |  |  |  |  |  |
| 0.252841 | 10 | igd | 0.588235 | C | \|\| id:met-b-156 | met-b-156 | TRUE | A | 97624709 | 1.80E-39 | reported | 459 | 0.0192337 | rs2275759 |  |  |  |  |  |  |  |  |  |  |  |  |  |  |  |
| -0.2538 | 6 | igd | 0.5874 | G | \|\| id:ebi-a-GCST90002105 | ebi-a-GCST90002105 | TRUE | C | 31221525 | 9.92E-16 | reported | 2872 | 0.03143 | rs2394948 |  |  |  |  |  |  |  |  |  |  |  |  |  |  |  |
| 0.255123 | 10 | igd | 0.586433 | A | \|\| id:met-b-156 | met-b-156 | TRUE | G | 97508844 | 9.40E-40 | reported | 457 | 0.0193351 | rs7096317 |  |  |  |  |  |  |  |  |  |  |  |  |  |  |  |
| 0.1379 | 22 | igd | 0.5864 | T | \|\| id:ebi-a-GCST90001701 | ebi-a-GCST90001701 | TRUE | C | 42234937 | 3.20E-08 | reported | 3592 | 0.02488 | rs7287010 |  |  |  |  |  |  |  |  |  |  |  |  |  |  |  |
| 0.304 | 22 | igd | 0.5861 | T | \|\| id:ebi-a-GCST90001703 | ebi-a-GCST90001703 | TRUE | C | 42234937 | 1.82E-33 | reported | 3657 | 0.02496 | rs7287010 |  |  |  |  |  |  |  |  |  |  |  |  |  |  |  |
| 0.1951 | 6 | igd | 0.5834 | T | \|\| id:ebi-a-GCST90002105 | ebi-a-GCST90002105 | TRUE | C | 32683324 | 1.13E-09 | reported | 2872 | 0.03193 | rs3104410 |  |  |  |  |  |  |  |  |  |  |  |  |  |  |  |
| -0.1549 | 22 | igd | 0.5816 | T | \|\| id:ebi-a-GCST90001702 | ebi-a-GCST90001702 | TRUE | C | 38906432 | 1.27E-09 | reported | 3657 | 0.02544 | rs138464 |  |  |  |  |  |  |  |  |  |  |  |  |  |  |  |
| -0.1616 | 22 | igd | 0.5816 | T | \|\| id:ebi-a-GCST90001703 | ebi-a-GCST90001703 | TRUE | C | 38906432 | 2.35E-10 | reported | 3657 | 0.02543 | rs138464 |  |  |  |  |  |  |  |  |  |  |  |  |  |  |  |
| -0.1716 | 22 | igd | 0.5816 | T | \|\| id:ebi-a-GCST90001704 | ebi-a-GCST90001704 | TRUE | C | 38906432 | 1.59E-11 | reported | 3657 | 0.02538 | rs138464 |  |  |  |  |  |  |  |  |  |  |  |  |  |  |  |
| -0.1442 | 22 | igd | 0.5816 | T | \|\| id:ebi-a-GCST90001710 | ebi-a-GCST90001710 | TRUE | C | 38906432 | 1.47E-08 | reported | 3657 | 0.02539 | rs138464 |  |  |  |  |  |  |  |  |  |  |  |  |  |  |  |
| -0.1495 | 22 | igd | 0.5816 | T | \|\| id:ebi-a-GCST90001711 | ebi-a-GCST90001711 | TRUE | C | 38906432 | 3.90E-09 | reported | 3657 | 0.02532 | rs138464 |  |  |  |  |  |  |  |  |  |  |  |  |  |  |  |
| -0.182 | 6 | igd | 0.5801 | C | \|\| id:ebi-a-GCST90002081 | ebi-a-GCST90002081 | TRUE | T | 32560741 | 5.63E-09 | reported | 3113 | 0.03114 | rs9270560 |  |  |  |  |  |  |  |  |  |  |  |  |  |  |  |
| -0.2141 | 10 | igd | 0.5779 | T | \|\| id:ebi-a-GCST90002061 | ebi-a-GCST90002061 | TRUE | G | 97358282 | 9.92E-15 | reported | 2920 | 0.02752 | rs525807 |  |  |  |  |  |  |  |  |  |  |  |  |  |  |  |
| -1.737 | 2 | igd | 0.5778 | T | \|\| id:ebi-a-GCST90001696 | ebi-a-GCST90001696 | TRUE | C | 38400597 | 2.14E-08 | reported | 3425 | 0.3094 | rs416497 |  |  |  |  |  |  |  |  |  |  |  |  |  |  |  |
| 0.3004 | 1 | igd | 0.5778 | C | \|\| id:ebi-a-GCST90001880 | ebi-a-GCST90001880 | TRUE | T | 2488608 | 2.17E-14 | reported | 1247 | 0.03886 | rs1886730 |  |  |  |  |  |  |  |  |  |  |  |  |  |  |  |
| 0.1494 | 22 | igd | 0.5777 | A | \|\| id:ebi-a-GCST90001704 | ebi-a-GCST90001704 | TRUE | C | 40849704 | 3.98E-09 | reported | 3657 | 0.02533 | rs4507196 |  |  |  |  |  |  |  |  |  |  |  |  |  |  |  |
| -0.1675 | 1 | igd | 0.5771 | C | \|\| id:ebi-a-GCST90001845 | ebi-a-GCST90001845 | TRUE | A | 167461391 | 1.42E-09 | reported | 2911 | 0.02758 | rs858545 |  |  |  |  |  |  |  |  |  |  |  |  |  |  |  |
| -0.1878 | 1 | igd | 0.5766 | C | \|\| id:ebi-a-GCST90001858 | ebi-a-GCST90001858 | TRUE | A | 167461391 | 9.35E-12 | reported | 2919 | 0.02744 | rs858545 |  |  |  |  |  |  |  |  |  |  |  |  |  |  |  |
| -0.1535 | 1 | igd | 0.5766 | C | \|\| id:ebi-a-GCST90001861 | ebi-a-GCST90001861 | TRUE | A | 167461391 | 1.88E-08 | reported | 2919 | 0.02723 | rs858545 |  |  |  |  |  |  |  |  |  |  |  |  |  |  |  |
| -0.1805 | 1 | igd | 0.5766 | C | \|\| id:ebi-a-GCST90001864 | ebi-a-GCST90001864 | TRUE | A | 167461391 | 5.67E-11 | reported | 2919 | 0.02744 | rs858545 |  |  |  |  |  |  |  |  |  |  |  |  |  |  |  |
| -0.1492 | 22 | igd | 0.5759 | T | \|\| id:ebi-a-GCST90001702 | ebi-a-GCST90001702 | TRUE | C | 39124912 | 4.71E-09 | reported | 3657 | 0.02542 | rs2235229 |  |  |  |  |  |  |  |  |  |  |  |  |  |  |  |
| -0.1545 | 22 | igd | 0.5759 | T | \|\| id:ebi-a-GCST90001703 | ebi-a-GCST90001703 | TRUE | C | 39124912 | 1.31E-09 | reported | 3657 | 0.02541 | rs2235229 |  |  |  |  |  |  |  |  |  |  |  |  |  |  |  |
| -0.1654 | 22 | igd | 0.5759 | T | \|\| id:ebi-a-GCST90001704 | ebi-a-GCST90001704 | TRUE | C | 39124912 | 7.85E-11 | reported | 3657 | 0.02535 | rs2235229 |  |  |  |  |  |  |  |  |  |  |  |  |  |  |  |
| -0.147 | 22 | igd | 0.5759 | T | \|\| id:ebi-a-GCST90001711 | ebi-a-GCST90001711 | TRUE | C | 39124912 | 6.77E-09 | reported | 3657 | 0.02529 | rs2235229 |  |  |  |  |  |  |  |  |  |  |  |  |  |  |  |
| -0.4648 | 10 | igd | 0.5755 | A | \|\| id:ebi-a-GCST90001961 | ebi-a-GCST90001961 | TRUE | G | 97680310 | 2.40E-65 | reported | 2920 | 0.02655 | rs4303172 |  |  |  |  |  |  |  |  |  |  |  |  |  |  |  |
| 0.1772 | 15 | igd | 0.5754 | G | \|\| id:ebi-a-GCST90001821 | ebi-a-GCST90001821 | TRUE | A | 40740713 | 1.71E-12 | reported | 3656 | 0.02503 | rs1659563 |  |  |  |  |  |  |  |  |  |  |  |  |  |  |  |
| -0.8878 | 10 | igd | 0.5738 | C | \|\| id:ebi-a-GCST90001484 | ebi-a-GCST90001484 | TRUE | A | 97475574 | 1.00E-200 | reported | 3437 | 0.02028 | rs4918962 |  |  |  |  |  |  |  |  |  |  |  |  |  |  |  |
| -0.1904 | 17 | igd | 0.5737 | T | \|\| id:ebi-a-GCST90001522 | ebi-a-GCST90001522 | TRUE | G | 33797371 | 1.59E-08 | reported | 1858 | 0.03354 | rs9916257 |  |  |  |  |  |  |  |  |  |  |  |  |  |  |  |
| -0.5992 | 6 | igd | 0.5722 | T | \|\| id:ebi-a-GCST90002108 | ebi-a-GCST90002108 | TRUE | C | 32561327 | 2.14E-49 | reported | 1579 | 0.03915 | rs9270588 |  |  |  |  |  |  |  |  |  |  |  |  |  |  |  |
| -0.4302 | 6 | igd | 0.5722 | T | \|\| id:ebi-a-GCST90002109 | ebi-a-GCST90002109 | TRUE | C | 32561327 | 7.98E-26 | reported | 1578 | 0.04022 | rs9270588 |  |  |  |  |  |  |  |  |  |  |  |  |  |  |  |
| -0.1601 | 10 | igd | 0.5719 | T | \|\| id:ebi-a-GCST90001660 | ebi-a-GCST90001660 | TRUE | G | 99348130 | 6.39E-10 | reported | 3408 | 0.02584 | rs12780691 |  |  |  |  |  |  |  |  |  |  |  |  |  |  |  |
| -0.1582 | 3 | igd | 0.5717 | T | \|\| id:ebi-a-GCST90001457 | ebi-a-GCST90001457 | TRUE | C | 188133439 | 8.47E-09 | reported | 3042 | 0.02739 | rs6808893 |  |  |  |  |  |  |  |  |  |  |  |  |  |  |  |
| -0.1583 | 10 | igd | 0.5717 | T | \|\| id:ebi-a-GCST90001495 | ebi-a-GCST90001495 | TRUE | G | 99348130 | 1.53E-09 | reported | 3405 | 0.02614 | rs12780691 |  |  |  |  |  |  |  |  |  |  |  |  |  |  |  |
| 0.3005 | 2 | igd | 0.5717 | G | \|\| id:ebi-a-GCST90001892 | ebi-a-GCST90001892 | TRUE | A | 204612058 | 1.67E-26 | reported | 2920 | 0.02793 | rs7426056 |  |  |  |  |  |  |  |  |  |  |  |  |  |  |  |
| -0.1491 | 10 | igd | 0.5715 | T | \|\| id:ebi-a-GCST90001658 | ebi-a-GCST90001658 | TRUE | G | 99348130 | 1.63E-09 | reported | 3440 | 0.02466 | rs12780691 |  |  |  |  |  |  |  |  |  |  |  |  |  |  |  |
| -0.1513 | 10 | igd | 0.5715 | T | \|\| id:ebi-a-GCST90001659 | ebi-a-GCST90001659 | TRUE | G | 99348130 | 9.33E-10 | reported | 3440 | 0.02466 | rs12780691 |  |  |  |  |  |  |  |  |  |  |  |  |  |  |  |
| -0.1663 | 10 | igd | 0.5713 | T | \|\| id:ebi-a-GCST90001496 | ebi-a-GCST90001496 | TRUE | G | 99348130 | 1.41E-10 | reported | 3437 | 0.02585 | rs12780691 |  |  |  |  |  |  |  |  |  |  |  |  |  |  |  |
| -0.1635 | 10 | igd | 0.5713 | T | \|\| id:ebi-a-GCST90001497 | ebi-a-GCST90001497 | TRUE | G | 99348130 | 1.70E-10 | reported | 3437 | 0.02553 | rs12780691 |  |  |  |  |  |  |  |  |  |  |  |  |  |  |  |
| 0.6429 | 10 | igd | 0.5703 | C | \|\| id:ebi-a-GCST90001671 | ebi-a-GCST90001671 | TRUE | T | 97681520 | 5.43E-146 | reported | 3440 | 0.02379 | rs10786245 |  |  |  |  |  |  |  |  |  |  |  |  |  |  |  |
| 0.6455 | 10 | igd | 0.5698 | C | \|\| id:ebi-a-GCST90001672 | ebi-a-GCST90001672 | TRUE | T | 97681520 | 1.10E-142 | reported | 3408 | 0.02418 | rs10786245 |  |  |  |  |  |  |  |  |  |  |  |  |  |  |  |
| 0.644 | 10 | igd | 0.5696 | G | \|\| id:ebi-a-GCST90001670 | ebi-a-GCST90001670 | TRUE | A | 97680399 | 2.44E-147 | reported | 3440 | 0.02371 | rs4504994 |  |  |  |  |  |  |  |  |  |  |  |  |  |  |  |
| -0.2003 | 10 | igd | 0.5692 | G | \|\| id:ebi-a-GCST90002030 | ebi-a-GCST90002030 | TRUE | C | 95888687 | 4.54E-13 | reported | 2920 | 0.02755 | rs11187792 |  |  |  |  |  |  |  |  |  |  |  |  |  |  |  |
| -0.1929 | 10 | igd | 0.5692 | G | \|\| id:ebi-a-GCST90002031 | ebi-a-GCST90002031 | TRUE | C | 95888687 | 9.02E-12 | reported | 2920 | 0.02816 | rs11187792 |  |  |  |  |  |  |  |  |  |  |  |  |  |  |  |
| -0.1752 | 10 | igd | 0.5692 | G | \|\| id:ebi-a-GCST90002032 | ebi-a-GCST90002032 | TRUE | C | 95888687 | 5.85E-10 | reported | 2920 | 0.02819 | rs11187792 |  |  |  |  |  |  |  |  |  |  |  |  |  |  |  |
| -0.1537 | 6 | igd | 0.5674 | C | \|\| id:ebi-a-GCST90001820 | ebi-a-GCST90001820 | TRUE | T | 31412961 | 4.99E-08 | reported | 3656 | 0.02814 | rs2596464 |  |  |  |  |  |  |  |  |  |  |  |  |  |  |  |
| -0.1387 | 2 | igd | 0.5673 | G | \|\| id:ebi-a-GCST90001460 | ebi-a-GCST90001460 | TRUE | A | 136421323 | 2.59E-08 | reported | 3374 | 0.02486 | rs1374329 |  |  |  |  |  |  |  |  |  |  |  |  |  |  |  |
| 0.4465 | 22 | igd | 0.5671 | T | \|\| id:ebi-a-GCST90001702 | ebi-a-GCST90001702 | TRUE | C | 42468910 | 1.37E-70 | reported | 3657 | 0.0246 | rs133380 |  |  |  |  |  |  |  |  |  |  |  |  |  |  |  |
| 0.4367 | 22 | igd | 0.5671 | T | \|\| id:ebi-a-GCST90001703 | ebi-a-GCST90001703 | TRUE | C | 42468910 | 1.71E-67 | reported | 3657 | 0.02464 | rs133380 |  |  |  |  |  |  |  |  |  |  |  |  |  |  |  |
| 0.4429 | 22 | igd | 0.5671 | T | \|\| id:ebi-a-GCST90001710 | ebi-a-GCST90001710 | TRUE | C | 42468910 | 8.28E-70 | reported | 3657 | 0.02454 | rs133380 |  |  |  |  |  |  |  |  |  |  |  |  |  |  |  |
| 0.4402 | 22 | igd | 0.5671 | T | \|\| id:ebi-a-GCST90001711 | ebi-a-GCST90001711 | TRUE | C | 42468910 | 2.57E-69 | reported | 3657 | 0.02449 | rs133380 |  |  |  |  |  |  |  |  |  |  |  |  |  |  |  |
| 0.1698 | 1 | igd | 0.5656 | G | \|\| id:ebi-a-GCST90001845 | ebi-a-GCST90001845 | TRUE | A | 166494573 | 1.39E-09 | reported | 2911 | 0.02794 | rs4657567 |  |  |  |  |  |  |  |  |  |  |  |  |  |  |  |
| 0.1502 | 22 | igd | 0.565 | T | \|\| id:ebi-a-GCST90001830 | ebi-a-GCST90001830 | TRUE | C | 42468246 | 9.12E-10 | reported | 3648 | 0.02447 | rs28605764 |  |  |  |  |  |  |  |  |  |  |  |  |  |  |  |
| -0.1526 | 10 | igd | 0.5644 | G | \|\| id:ebi-a-GCST90001496 | ebi-a-GCST90001496 | TRUE | C | 95888687 | 7.59E-09 | reported | 3437 | 0.02635 | rs11187792 |  |  |  |  |  |  |  |  |  |  |  |  |  |  |  |
| 0.303 | 2 | igd | 0.5639 | T | \|\| id:ebi-a-GCST90001886 | ebi-a-GCST90001886 | TRUE | G | 204589281 | 8.44E-30 | reported | 2920 | 0.02642 | rs3116493 |  |  |  |  |  |  |  |  |  |  |  |  |  |  |  |
| 0.2335 | 2 | igd | 0.5635 | C | \|\| id:ebi-a-GCST90001888 | ebi-a-GCST90001888 | TRUE | G | 204586076 | 1.53E-17 | reported | 2920 | 0.02722 | rs3116488 |  |  |  |  |  |  |  |  |  |  |  |  |  |  |  |
| 0.1976 | 2 | igd | 0.5635 | T | \|\| id:ebi-a-GCST90001897 | ebi-a-GCST90001897 | TRUE | G | 204589281 | 7.57E-13 | reported | 2914 | 0.02744 | rs3116493 |  |  |  |  |  |  |  |  |  |  |  |  |  |  |  |
| -0.2225 | 19 | igd | 0.5628 | C | \|\| id:ebi-a-GCST90001948 | ebi-a-GCST90001948 | TRUE | T | 52254351 | 7.66E-10 | reported | 1633 | 0.03595 | rs4801891 |  |  |  |  |  |  |  |  |  |  |  |  |  |  |  |
| 0.142 | 22 | igd | 0.5614 | A | \|\| id:ebi-a-GCST90001710 | ebi-a-GCST90001710 | TRUE | G | 40837822 | 1.71E-08 | reported | 3657 | 0.02513 | rs12170360 |  |  |  |  |  |  |  |  |  |  |  |  |  |  |  |
| 0.1517 | 22 | igd | 0.5602 | G | \|\| id:ebi-a-GCST90001704 | ebi-a-GCST90001704 | TRUE | A | 43520263 | 2.14E-09 | reported | 3657 | 0.02528 | rs2076079 |  |  |  |  |  |  |  |  |  |  |  |  |  |  |  |
| 0.1591 | 22 | igd | 0.5596 | T | \|\| id:ebi-a-GCST90001702 | ebi-a-GCST90001702 | TRUE | C | 43526496 | 3.74E-10 | reported | 3657 | 0.02532 | rs5759176 |  |  |  |  |  |  |  |  |  |  |  |  |  |  |  |
| 0.1589 | 22 | igd | 0.5596 | T | \|\| id:ebi-a-GCST90001703 | ebi-a-GCST90001703 | TRUE | C | 43526496 | 3.85E-10 | reported | 3657 | 0.02532 | rs5759176 |  |  |  |  |  |  |  |  |  |  |  |  |  |  |  |
| -0.1762 | 10 | igd | 0.5587 | C | \|\| id:ebi-a-GCST90001777 | ebi-a-GCST90001777 | TRUE | T | 6178941 | 6.79E-13 | reported | 3657 | 0.02445 | rs11598494 |  |  |  |  |  |  |  |  |  |  |  |  |  |  |  |
| -0.1524 | 10 | igd | 0.5587 | C | \|\| id:ebi-a-GCST90001778 | ebi-a-GCST90001778 | TRUE | T | 6178941 | 6.89E-10 | reported | 3657 | 0.02465 | rs11598494 |  |  |  |  |  |  |  |  |  |  |  |  |  |  |  |
| -0.1458 | 10 | igd | 0.5587 | C | \|\| id:ebi-a-GCST90001789 | ebi-a-GCST90001789 | TRUE | T | 6178941 | 2.34E-09 | reported | 3657 | 0.02435 | rs11598494 |  |  |  |  |  |  |  |  |  |  |  |  |  |  |  |
| -0.1768 | 10 | igd | 0.5587 | C | \|\| id:ebi-a-GCST90001790 | ebi-a-GCST90001790 | TRUE | T | 6178941 | 6.09E-13 | reported | 3657 | 0.02448 | rs11598494 |  |  |  |  |  |  |  |  |  |  |  |  |  |  |  |
| -0.1584 | 10 | igd | 0.5587 | C | \|\| id:ebi-a-GCST90001792 | ebi-a-GCST90001792 | TRUE | T | 6178941 | 1.20E-10 | reported | 3657 | 0.02453 | rs11598494 |  |  |  |  |  |  |  |  |  |  |  |  |  |  |  |
| 0.2681 | 19 | igd | 0.5574 | T | \|\| id:ebi-a-GCST90001951 | ebi-a-GCST90001951 | TRUE | C | 51591138 | 2.62E-12 | reported | 1464 | 0.03799 | rs78949787 |  |  |  |  |  |  |  |  |  |  |  |  |  |  |  |
| -0.1757 | 6 | igd | 0.5553 | A | \|\| id:ebi-a-GCST90002119 | ebi-a-GCST90002119 | TRUE | G | 32337630 | 6.52E-09 | reported | 2920 | 0.03019 | rs2273017 |  |  |  |  |  |  |  |  |  |  |  |  |  |  |  |
| -0.1453 | 20 | igd | 0.555 | A | \|\| id:ebi-a-GCST90001798 | ebi-a-GCST90001798 | TRUE | G | 46785499 | 6.20E-09 | reported | 3657 | 0.02494 | rs162076 |  |  |  |  |  |  |  |  |  |  |  |  |  |  |  |
| -0.143 | 20 | igd | 0.555 | A | \|\| id:ebi-a-GCST90001802 | ebi-a-GCST90001802 | TRUE | G | 46785499 | 1.54E-08 | reported | 3657 | 0.02522 | rs162076 |  |  |  |  |  |  |  |  |  |  |  |  |  |  |  |
| -0.1634 | 20 | igd | 0.555 | A | \|\| id:ebi-a-GCST90001804 | ebi-a-GCST90001804 | TRUE | G | 46785499 | 5.03E-11 | reported | 3657 | 0.02479 | rs162076 |  |  |  |  |  |  |  |  |  |  |  |  |  |  |  |
| -0.1635 | 20 | igd | 0.555 | A | \|\| id:ebi-a-GCST90001808 | ebi-a-GCST90001808 | TRUE | G | 46785499 | 8.52E-11 | reported | 3657 | 0.02512 | rs162076 |  |  |  |  |  |  |  |  |  |  |  |  |  |  |  |
| 0.2135 | 6 | igd | 0.555 | C | \|\| id:ebi-a-GCST90002111 | ebi-a-GCST90002111 | TRUE | T | 32802820 | 2.12E-08 | reported | 1635 | 0.03792 | rs241430 |  |  |  |  |  |  |  |  |  |  |  |  |  |  |  |
| 0.2203 | 6 | igd | 0.5548 | C | \|\| id:ebi-a-GCST90002110 | ebi-a-GCST90002110 | TRUE | T | 32802820 | 1.25E-08 | reported | 1634 | 0.0385 | rs241430 |  |  |  |  |  |  |  |  |  |  |  |  |  |  |  |
| -0.2315 | 6 | igd | 0.5548 | G | \|\| id:ebi-a-GCST90002110 | ebi-a-GCST90002110 | TRUE | T | 31465661 | 6.51E-09 | reported | 1634 | 0.03968 | rs2534671 |  |  |  |  |  |  |  |  |  |  |  |  |  |  |  |
| 0.1624 | 4 | igd | 0.5544 | G | \|\| id:ebi-a-GCST90001811 | ebi-a-GCST90001811 | TRUE | A | 15833532 | 1.16E-10 | reported | 3656 | 0.02513 | rs4425367 |  |  |  |  |  |  |  |  |  |  |  |  |  |  |  |
| 0.2116 | 6 | igd | 0.5535 | C | \|\| id:ebi-a-GCST90002112 | ebi-a-GCST90002112 | TRUE | T | 32798711 | 3.85E-08 | reported | 1635 | 0.0383 | rs117821 |  |  |  |  |  |  |  |  |  |  |  |  |  |  |  |
| 0.276 | 6 | igd | 0.5531 | C | \|\| id:ebi-a-GCST90002105 | ebi-a-GCST90002105 | TRUE | T | 32802820 | 3.51E-20 | reported | 2872 | 0.02977 | rs241430 |  |  |  |  |  |  |  |  |  |  |  |  |  |  |  |
| 0.2373 | 6 | igd | 0.5526 | C | \|\| id:ebi-a-GCST90002108 | ebi-a-GCST90002108 | TRUE | T | 32802820 | 9.15E-10 | reported | 1579 | 0.03852 | rs241430 |  |  |  |  |  |  |  |  |  |  |  |  |  |  |  |
| -0.1602 | 6 | igd | 0.5525 | G | \|\| id:ebi-a-GCST90001743 | ebi-a-GCST90001743 | TRUE | A | 32634341 | 3.69E-09 | reported | 3660 | 0.0271 | rs3189152 |  |  |  |  |  |  |  |  |  |  |  |  |  |  |  |
| 0.1554 | 11 | igd | 0.5524 | C | \|\| id:ebi-a-GCST90002084 | ebi-a-GCST90002084 | TRUE | T | 95852734 | 1.55E-08 | reported | 3113 | 0.02741 | rs10765790 |  |  |  |  |  |  |  |  |  |  |  |  |  |  |  |
| -0.1621 | 1 | igd | 0.5524 | A | \|\| id:ebi-a-GCST90002014 | ebi-a-GCST90002014 | TRUE | G | 161683136 | 8.10E-09 | reported | 2870 | 0.02803 | rs11746 |  |  |  |  |  |  |  |  |  |  |  |  |  |  |  |
| -0.2346 | 10 | igd | 0.5521 | G | \|\| id:ebi-a-GCST90001672 | ebi-a-GCST90001672 | TRUE | A | 96200796 | 4.50E-18 | reported | 3408 | 0.02692 | rs7895305 |  |  |  |  |  |  |  |  |  |  |  |  |  |  |  |
| -0.2292 | 10 | igd | 0.552 | G | \|\| id:ebi-a-GCST90001670 | ebi-a-GCST90001670 | TRUE | A | 96200796 | 5.93E-18 | reported | 3440 | 0.0264 | rs7895305 |  |  |  |  |  |  |  |  |  |  |  |  |  |  |  |
| -0.2072 | 10 | igd | 0.552 | G | \|\| id:ebi-a-GCST90001671 | ebi-a-GCST90001671 | TRUE | A | 96200796 | 7.81E-15 | reported | 3440 | 0.02655 | rs7895305 |  |  |  |  |  |  |  |  |  |  |  |  |  |  |  |
| 0.238 | 1 | igd | 0.552 | C | \|\| id:ebi-a-GCST90002045 | ebi-a-GCST90002045 | TRUE | T | 161508763 | 8.84E-11 | reported | 1635 | 0.03646 | rs10919544 |  |  |  |  |  |  |  |  |  |  |  |  |  |  |  |
| 0.1486 | 10 | igd | 0.5516 | A | \|\| id:ebi-a-GCST90002076 | ebi-a-GCST90002076 | TRUE | G | 126302936 | 4.05E-08 | reported | 3113 | 0.027 | rs7095944 |  |  |  |  |  |  |  |  |  |  |  |  |  |  |  |
| -0.3669 | 6 | igd | 0.5507 | A | \|\| id:ebi-a-GCST90002108 | ebi-a-GCST90002108 | TRUE | G | 32699216 | 4.94E-20 | reported | 1579 | 0.03949 | rs9469257 |  |  |  |  |  |  |  |  |  |  |  |  |  |  |  |
| -0.2102 | 19 | igd | 0.5499 | G | \|\| id:ebi-a-GCST90001948 | ebi-a-GCST90001948 | TRUE | A | 52140628 | 2.78E-08 | reported | 1633 | 0.03766 | rs7249503 |  |  |  |  |  |  |  |  |  |  |  |  |  |  |  |
| -0.1628 | 6 | igd | 0.5487 | C | \|\| id:ebi-a-GCST90001687 | ebi-a-GCST90001687 | TRUE | T | 31382133 | 1.81E-08 | reported | 3408 | 0.02884 | rs7756993 |  |  |  |  |  |  |  |  |  |  |  |  |  |  |  |
| -0.2281 | 10 | igd | 0.5475 | C | \|\| id:ebi-a-GCST90001672 | ebi-a-GCST90001672 | TRUE | T | 96301907 | 3.30E-17 | reported | 3408 | 0.0269 | rs1977289 |  |  |  |  |  |  |  |  |  |  |  |  |  |  |  |
| -0.1509 | 1 | igd | 0.5475 | G | \|\| id:ebi-a-GCST90001969 | ebi-a-GCST90001969 | TRUE | A | 161508617 | 3.03E-08 | reported | 3113 | 0.02717 | rs10919543 |  |  |  |  |  |  |  |  |  |  |  |  |  |  |  |
| -0.2207 | 10 | igd | 0.5474 | C | \|\| id:ebi-a-GCST90001670 | ebi-a-GCST90001670 | TRUE | T | 96301907 | 8.68E-17 | reported | 3440 | 0.02639 | rs1977289 |  |  |  |  |  |  |  |  |  |  |  |  |  |  |  |
| -0.1989 | 10 | igd | 0.5474 | C | \|\| id:ebi-a-GCST90001671 | ebi-a-GCST90001671 | TRUE | T | 96301907 | 8.38E-14 | reported | 3440 | 0.02654 | rs1977289 |  |  |  |  |  |  |  |  |  |  |  |  |  |  |  |
| -0.1575 | 22 | igd | 0.545 | C | \|\| id:ebi-a-GCST90001702 | ebi-a-GCST90001702 | TRUE | G | 42871832 | 8.23E-10 | reported | 3657 | 0.02559 | rs9611804 |  |  |  |  |  |  |  |  |  |  |  |  |  |  |  |
| -0.1561 | 22 | igd | 0.545 | C | \|\| id:ebi-a-GCST90001703 | ebi-a-GCST90001703 | TRUE | G | 42871832 | 1.16E-09 | reported | 3657 | 0.02559 | rs9611804 |  |  |  |  |  |  |  |  |  |  |  |  |  |  |  |
| -0.1578 | 22 | igd | 0.545 | C | \|\| id:ebi-a-GCST90001710 | ebi-a-GCST90001710 | TRUE | G | 42871832 | 6.94E-10 | reported | 3657 | 0.02551 | rs9611804 |  |  |  |  |  |  |  |  |  |  |  |  |  |  |  |
| -0.1664 | 22 | igd | 0.545 | C | \|\| id:ebi-a-GCST90001711 | ebi-a-GCST90001711 | TRUE | G | 42871832 | 6.91E-11 | reported | 3657 | 0.02544 | rs9611804 |  |  |  |  |  |  |  |  |  |  |  |  |  |  |  |
| -0.5579 | 19 | igd | 0.5441 | T | \|\| id:ebi-a-GCST90002052 | ebi-a-GCST90002052 | TRUE | G | 51725824 | 1.22E-50 | reported | 1555 | 0.03593 | rs273640 |  |  |  |  |  |  |  |  |  |  |  |  |  |  |  |
| -0.2275 | 6 | igd | 0.5428 | T | \|\| id:ebi-a-GCST90002109 | ebi-a-GCST90002109 | TRUE | A | 31313148 | 1.01E-08 | reported | 1578 | 0.0395 | rs4398728 |  |  |  |  |  |  |  |  |  |  |  |  |  |  |  |
| -0.2005 | 1 | igd | 0.5427 | C | \|\| id:ebi-a-GCST90001864 | ebi-a-GCST90001864 | TRUE | T | 167116924 | 3.48E-13 | reported | 2919 | 0.02744 | rs12128145 |  |  |  |  |  |  |  |  |  |  |  |  |  |  |  |
| 0.4097 | 19 | igd | 0.54264 | G | \|\| id:prot-a-439 | prot-a-439 | TRUE | T | 51801861 | 1.29E-62 | reported | 3301 | 0.0245 | rs1710355 |  |  |  |  |  |  |  |  |  |  |  |  |  |  |  |
| 0.1615 | 6 | igd | 0.542 | A | \|\| id:ebi-a-GCST90002121 | ebi-a-GCST90002121 | TRUE | G | 31272553 | 1.51E-08 | reported | 2915 | 0.02844 | rs9264904 |  |  |  |  |  |  |  |  |  |  |  |  |  |  |  |
| -0.2186 | 19 | igd | 0.5418 | G | \|\| id:ebi-a-GCST90001954 | ebi-a-GCST90001954 | TRUE | A | 52140628 | 2.02E-08 | reported | 1555 | 0.03875 | rs7249503 |  |  |  |  |  |  |  |  |  |  |  |  |  |  |  |
| 0.2451 | 10 | igd | 0.5413 | A | \|\| id:ebi-a-GCST90001483 | ebi-a-GCST90001483 | TRUE | C | 97980721 | 1.63E-22 | reported | 3405 | 0.02493 | rs2125062 |  |  |  |  |  |  |  |  |  |  |  |  |  |  |  |
| 0.2876 | 10 | igd | 0.5413 | A | \|\| id:ebi-a-GCST90001489 | ebi-a-GCST90001489 | TRUE | C | 97980721 | 5.26E-29 | reported | 3405 | 0.02549 | rs2125062 |  |  |  |  |  |  |  |  |  |  |  |  |  |  |  |
| 0.2991 | 10 | igd | 0.5413 | A | \|\| id:ebi-a-GCST90001495 | ebi-a-GCST90001495 | TRUE | C | 97980721 | 1.11E-30 | reported | 3405 | 0.02572 | rs2125062 |  |  |  |  |  |  |  |  |  |  |  |  |  |  |  |
| 0.2518 | 10 | igd | 0.5412 | A | \|\| id:ebi-a-GCST90001484 | ebi-a-GCST90001484 | TRUE | C | 97980721 | 4.08E-24 | reported | 3437 | 0.02467 | rs2125062 |  |  |  |  |  |  |  |  |  |  |  |  |  |  |  |
| 0.2427 | 10 | igd | 0.5412 | A | \|\| id:ebi-a-GCST90001485 | ebi-a-GCST90001485 | TRUE | C | 97980721 | 1.46E-22 | reported | 3437 | 0.02466 | rs2125062 |  |  |  |  |  |  |  |  |  |  |  |  |  |  |  |
| 0.2886 | 10 | igd | 0.5412 | A | \|\| id:ebi-a-GCST90001491 | ebi-a-GCST90001491 | TRUE | C | 97980721 | 4.41E-30 | reported | 3437 | 0.02509 | rs2125062 |  |  |  |  |  |  |  |  |  |  |  |  |  |  |  |
| 0.2998 | 10 | igd | 0.5412 | A | \|\| id:ebi-a-GCST90001496 | ebi-a-GCST90001496 | TRUE | C | 97980721 | 1.47E-31 | reported | 3437 | 0.0254 | rs2125062 |  |  |  |  |  |  |  |  |  |  |  |  |  |  |  |
| 0.2941 | 10 | igd | 0.5412 | A | \|\| id:ebi-a-GCST90001497 | ebi-a-GCST90001497 | TRUE | C | 97980721 | 3.77E-31 | reported | 3437 | 0.02509 | rs2125062 |  |  |  |  |  |  |  |  |  |  |  |  |  |  |  |
| 0.3295 | 10 | igd | 0.5411 | A | \|\| id:ebi-a-GCST90001660 | ebi-a-GCST90001660 | TRUE | C | 97980721 | 7.00E-38 | reported | 3408 | 0.0253 | rs2125062 |  |  |  |  |  |  |  |  |  |  |  |  |  |  |  |
| 0.2942 | 10 | igd | 0.5411 | A | \|\| id:ebi-a-GCST90001672 | ebi-a-GCST90001672 | TRUE | C | 97980721 | 8.04E-30 | reported | 3408 | 0.02569 | rs2125062 |  |  |  |  |  |  |  |  |  |  |  |  |  |  |  |
| 0.3231 | 10 | igd | 0.541 | A | \|\| id:ebi-a-GCST90001658 | ebi-a-GCST90001658 | TRUE | C | 97980721 | 5.58E-40 | reported | 3440 | 0.0241 | rs2125062 |  |  |  |  |  |  |  |  |  |  |  |  |  |  |  |
| 0.3261 | 10 | igd | 0.541 | A | \|\| id:ebi-a-GCST90001659 | ebi-a-GCST90001659 | TRUE | C | 97980721 | 1.06E-40 | reported | 3440 | 0.0241 | rs2125062 |  |  |  |  |  |  |  |  |  |  |  |  |  |  |  |
| 0.2917 | 10 | igd | 0.541 | A | \|\| id:ebi-a-GCST90001670 | ebi-a-GCST90001670 | TRUE | C | 97980721 | 2.46E-30 | reported | 3440 | 0.02524 | rs2125062 |  |  |  |  |  |  |  |  |  |  |  |  |  |  |  |
| 0.2892 | 10 | igd | 0.541 | A | \|\| id:ebi-a-GCST90001671 | ebi-a-GCST90001671 | TRUE | C | 97980721 | 9.64E-30 | reported | 3440 | 0.0253 | rs2125062 |  |  |  |  |  |  |  |  |  |  |  |  |  |  |  |
| -0.1328 | 2 | igd | 0.54 | G | \|\| id:ebi-a-GCST90001664 | ebi-a-GCST90001664 | TRUE | T | 39334710 | 2.47E-08 | reported | 3440 | 0.02377 | rs963828 |  |  |  |  |  |  |  |  |  |  |  |  |  |  |  |
| -0.126 | 2 | igd | 0.5396 | G | \|\| id:ebi-a-GCST90001666 | ebi-a-GCST90001666 | TRUE | T | 39334710 | 2.52E-08 | reported | 3408 | 0.02256 | rs963828 |  |  |  |  |  |  |  |  |  |  |  |  |  |  |  |
| -0.168 | 1 | igd | 0.5396 | C | \|\| id:ebi-a-GCST90002017 | ebi-a-GCST90002017 | TRUE | G | 161395555 | 1.68E-09 | reported | 2850 | 0.02779 | rs12022054 |  |  |  |  |  |  |  |  |  |  |  |  |  |  |  |
| -0.1502 | 2 | igd | 0.5395 | G | \|\| id:ebi-a-GCST90001561 | ebi-a-GCST90001561 | TRUE | T | 39334710 | 6.62E-12 | reported | 3427 | 0.0218 | rs963828 |  |  |  |  |  |  |  |  |  |  |  |  |  |  |  |
| 1.774 | 2 | igd | 0.5393 | G | \|\| id:ebi-a-GCST90001696 | ebi-a-GCST90001696 | TRUE | T | 39334710 | 8.64E-09 | reported | 3425 | 0.3074 | rs963828 |  |  |  |  |  |  |  |  |  |  |  |  |  |  |  |
| -0.2544 | 2 | igd | 0.5392 | G | \|\| id:ebi-a-GCST90002098 | ebi-a-GCST90002098 | TRUE | T | 39334710 | 2.20E-22 | reported | 2910 | 0.02593 | rs963828 |  |  |  |  |  |  |  |  |  |  |  |  |  |  |  |
| 0.1551 | 1 | igd | 0.5391 | T | \|\| id:ebi-a-GCST90001854 | ebi-a-GCST90001854 | TRUE | G | 169070736 | 8.53E-09 | reported | 2919 | 0.02687 | rs6427176 |  |  |  |  |  |  |  |  |  |  |  |  |  |  |  |
| 0.1811 | 2 | igd | 0.5386 | A | \|\| id:ebi-a-GCST90001963 | ebi-a-GCST90001963 | TRUE | T | 202960847 | 1.72E-10 | reported | 2872 | 0.02827 | rs13419481 |  |  |  |  |  |  |  |  |  |  |  |  |  |  |  |
| -0.2346 | 10 | igd | 0.5385 | A | \|\| id:ebi-a-GCST90001961 | ebi-a-GCST90001961 | TRUE | C | 97980721 | 1.45E-17 | reported | 2920 | 0.02732 | rs2125062 |  |  |  |  |  |  |  |  |  |  |  |  |  |  |  |
| 0.3734 | 10 | igd | 0.5385 | A | \|\| id:ebi-a-GCST90002030 | ebi-a-GCST90002030 | TRUE | C | 97980721 | 2.68E-43 | reported | 2920 | 0.02662 | rs2125062 |  |  |  |  |  |  |  |  |  |  |  |  |  |  |  |
| 0.3056 | 10 | igd | 0.5385 | A | \|\| id:ebi-a-GCST90002031 | ebi-a-GCST90002031 | TRUE | C | 97980721 | 2.90E-28 | reported | 2920 | 0.02743 | rs2125062 |  |  |  |  |  |  |  |  |  |  |  |  |  |  |  |
| 0.2782 | 10 | igd | 0.5385 | A | \|\| id:ebi-a-GCST90002032 | ebi-a-GCST90002032 | TRUE | C | 97980721 | 1.29E-23 | reported | 2920 | 0.02753 | rs2125062 |  |  |  |  |  |  |  |  |  |  |  |  |  |  |  |
| 0.1917 | 10 | igd | 0.5385 | A | \|\| id:ebi-a-GCST90002061 | ebi-a-GCST90002061 | TRUE | C | 97980721 | 4.59E-12 | reported | 2920 | 0.02759 | rs2125062 |  |  |  |  |  |  |  |  |  |  |  |  |  |  |  |
| -0.1602 | 10 | igd | 0.5378 | G | \|\| id:ebi-a-GCST90001505 | ebi-a-GCST90001505 | TRUE | C | 6080794 | 8.02E-10 | reported | 3437 | 0.026 | rs11256457 |  |  |  |  |  |  |  |  |  |  |  |  |  |  |  |
| 0.177 | 6 | igd | 0.5377 | C | \|\| id:ebi-a-GCST90002067 | ebi-a-GCST90002067 | TRUE | G | 31022158 | 3.12E-09 | reported | 2920 | 0.02978 | rs2523855 |  |  |  |  |  |  |  |  |  |  |  |  |  |  |  |
| -0.2998 | 22 | igd | 0.5368 | A | \|\| id:ebi-a-GCST90001702 | ebi-a-GCST90001702 | TRUE | G | 42247932 | 1.89E-33 | reported | 3657 | 0.02463 | rs8140722 |  |  |  |  |  |  |  |  |  |  |  |  |  |  |  |
| -0.3078 | 22 | igd | 0.5368 | A | \|\| id:ebi-a-GCST90001704 | ebi-a-GCST90001704 | TRUE | G | 42247932 | 2.70E-35 | reported | 3657 | 0.02456 | rs8140722 |  |  |  |  |  |  |  |  |  |  |  |  |  |  |  |
| -0.2818 | 22 | igd | 0.5368 | A | \|\| id:ebi-a-GCST90001710 | ebi-a-GCST90001710 | TRUE | G | 42247932 | 7.93E-30 | reported | 3657 | 0.02462 | rs8140722 |  |  |  |  |  |  |  |  |  |  |  |  |  |  |  |
| -0.2766 | 22 | igd | 0.5368 | A | \|\| id:ebi-a-GCST90001711 | ebi-a-GCST90001711 | TRUE | G | 42247932 | 6.47E-29 | reported | 3657 | 0.02457 | rs8140722 |  |  |  |  |  |  |  |  |  |  |  |  |  |  |  |
| 0.1359 | 12 | igd | 0.5341 | G | \|\| id:ebi-a-GCST90001401 | ebi-a-GCST90001401 | TRUE | A | 6553016 | 3.13E-08 | reported | 3656 | 0.02451 | rs2250246 |  |  |  |  |  |  |  |  |  |  |  |  |  |  |  |
| -0.1679 | 12 | igd | 0.534 | G | \|\| id:ebi-a-GCST90001803 | ebi-a-GCST90001803 | TRUE | A | 6553016 | 1.07E-11 | reported | 3642 | 0.02462 | rs2250246 |  |  |  |  |  |  |  |  |  |  |  |  |  |  |  |
| 0.1466 | 12 | igd | 0.5339 | G | \|\| id:ebi-a-GCST90001399 | ebi-a-GCST90001399 | TRUE | A | 6553016 | 2.13E-09 | reported | 3657 | 0.02442 | rs2250246 |  |  |  |  |  |  |  |  |  |  |  |  |  |  |  |
| -0.1386 | 12 | igd | 0.5339 | G | \|\| id:ebi-a-GCST90001798 | ebi-a-GCST90001798 | TRUE | A | 6553016 | 1.51E-08 | reported | 3657 | 0.02442 | rs2250246 |  |  |  |  |  |  |  |  |  |  |  |  |  |  |  |
| -0.2059 | 12 | igd | 0.5339 | G | \|\| id:ebi-a-GCST90001802 | ebi-a-GCST90001802 | TRUE | A | 6553016 | 7.42E-17 | reported | 3657 | 0.02456 | rs2250246 |  |  |  |  |  |  |  |  |  |  |  |  |  |  |  |
| -0.1681 | 12 | igd | 0.5339 | G | \|\| id:ebi-a-GCST90001804 | ebi-a-GCST90001804 | TRUE | A | 6553016 | 4.89E-12 | reported | 3657 | 0.02426 | rs2250246 |  |  |  |  |  |  |  |  |  |  |  |  |  |  |  |
| -0.1465 | 12 | igd | 0.5339 | G | \|\| id:ebi-a-GCST90001805 | ebi-a-GCST90001805 | TRUE | A | 6553016 | 3.11E-09 | reported | 3657 | 0.02466 | rs2250246 |  |  |  |  |  |  |  |  |  |  |  |  |  |  |  |
| -0.1803 | 12 | igd | 0.5339 | G | \|\| id:ebi-a-GCST90001808 | ebi-a-GCST90001808 | TRUE | A | 6553016 | 2.51E-13 | reported | 3657 | 0.02455 | rs2250246 |  |  |  |  |  |  |  |  |  |  |  |  |  |  |  |
| -0.1509 | 12 | igd | 0.5325 | T | \|\| id:ebi-a-GCST90001791 | ebi-a-GCST90001791 | TRUE | C | 31504316 | 7.67E-09 | reported | 3657 | 0.02607 | rs11051398 |  |  |  |  |  |  |  |  |  |  |  |  |  |  |  |
| -0.2086 | 10 | igd | 0.5325 | A | \|\| id:ebi-a-GCST90002030 | ebi-a-GCST90002030 | TRUE | G | 96278395 | 6.33E-14 | reported | 2920 | 0.02768 | rs11187998 |  |  |  |  |  |  |  |  |  |  |  |  |  |  |  |
| -0.1649 | 10 | igd | 0.5325 | A | \|\| id:ebi-a-GCST90002032 | ebi-a-GCST90002032 | TRUE | G | 96278395 | 6.62E-09 | reported | 2920 | 0.02834 | rs11187998 |  |  |  |  |  |  |  |  |  |  |  |  |  |  |  |
| -0.9594 | 10 | igd | 0.532 | A | \|\| id:ebi-a-GCST90001495 | ebi-a-GCST90001495 | TRUE | G | 97474371 | 1.00E-200 | reported | 3405 | 0.02046 | rs10882655 |  |  |  |  |  |  |  |  |  |  |  |  |  |  |  |
| -0.9139 | 10 | igd | 0.5319 | A | \|\| id:ebi-a-GCST90001491 | ebi-a-GCST90001491 | TRUE | G | 97474371 | 1.00E-200 | reported | 3437 | 0.02033 | rs10882655 |  |  |  |  |  |  |  |  |  |  |  |  |  |  |  |
| -0.1533 | 10 | igd | 0.5319 | A | \|\| id:ebi-a-GCST90001495 | ebi-a-GCST90001495 | TRUE | G | 96278395 | 1.66E-08 | reported | 3405 | 0.02709 | rs11187998 |  |  |  |  |  |  |  |  |  |  |  |  |  |  |  |
| -0.1809 | 10 | igd | 0.5318 | A | \|\| id:ebi-a-GCST90001660 | ebi-a-GCST90001660 | TRUE | G | 96278395 | 1.67E-11 | reported | 3408 | 0.02678 | rs11187998 |  |  |  |  |  |  |  |  |  |  |  |  |  |  |  |
| -0.1611 | 10 | igd | 0.5314 | A | \|\| id:ebi-a-GCST90001496 | ebi-a-GCST90001496 | TRUE | G | 96278395 | 1.91E-09 | reported | 3437 | 0.02675 | rs11187998 |  |  |  |  |  |  |  |  |  |  |  |  |  |  |  |
| -0.1638 | 10 | igd | 0.5314 | A | \|\| id:ebi-a-GCST90001497 | ebi-a-GCST90001497 | TRUE | G | 96278395 | 6.19E-10 | reported | 3437 | 0.02641 | rs11187998 |  |  |  |  |  |  |  |  |  |  |  |  |  |  |  |
| -0.1739 | 10 | igd | 0.5314 | A | \|\| id:ebi-a-GCST90001658 | ebi-a-GCST90001658 | TRUE | G | 96278395 | 1.13E-11 | reported | 3440 | 0.02552 | rs11187998 |  |  |  |  |  |  |  |  |  |  |  |  |  |  |  |
| -0.1852 | 10 | igd | 0.5314 | A | \|\| id:ebi-a-GCST90001659 | ebi-a-GCST90001659 | TRUE | G | 96278395 | 4.69E-13 | reported | 3440 | 0.0255 | rs11187998 |  |  |  |  |  |  |  |  |  |  |  |  |  |  |  |
| 0.1955 | 6 | igd | 0.531 | G | \|\| id:ebi-a-GCST90002105 | ebi-a-GCST90002105 | TRUE | A | 33509013 | 1.37E-11 | reported | 2872 | 0.02879 | rs210198 |  |  |  |  |  |  |  |  |  |  |  |  |  |  |  |
| -0.2046 | 10 | igd | 0.5301 | A | \|\| id:ebi-a-GCST90001789 | ebi-a-GCST90001789 | TRUE | G | 6085312 | 7.28E-17 | reported | 3657 | 0.02441 | rs4749926 |  |  |  |  |  |  |  |  |  |  |  |  |  |  |  |
| 0.1763 | 10 | igd | 0.53 | T | \|\| id:ebi-a-GCST90002032 | ebi-a-GCST90002032 | TRUE | C | 95542677 | 4.35E-10 | reported | 2920 | 0.02816 | rs11187665 |  |  |  |  |  |  |  |  |  |  |  |  |  |  |  |
| -0.1617 | 12 | igd | 0.5292 | C | \|\| id:ebi-a-GCST90001534 | ebi-a-GCST90001534 | TRUE | T | 111884608 | 1.22E-08 | reported | 3395 | 0.02832 | rs3184504 |  |  |  |  |  |  |  |  |  |  |  |  |  |  |  |
| -0.1619 | 12 | igd | 0.5287 | T | \|\| id:ebi-a-GCST90001534 | ebi-a-GCST90001534 | TRUE | C | 112007756 | 1.18E-08 | reported | 3395 | 0.02833 | rs653178 |  |  |  |  |  |  |  |  |  |  |  |  |  |  |  |
| 0.7136 | 10 | igd | 0.5276 | A | \|\| id:ebi-a-GCST90001961 | ebi-a-GCST90001961 | TRUE | G | 97474371 | 1.09E-167 | reported | 2920 | 0.02419 | rs10882655 |  |  |  |  |  |  |  |  |  |  |  |  |  |  |  |
| -1.008 | 10 | igd | 0.5276 | A | \|\| id:ebi-a-GCST90002031 | ebi-a-GCST90002031 | TRUE | G | 97474371 | 1.00E-200 | reported | 2920 | 0.02075 | rs10882655 |  |  |  |  |  |  |  |  |  |  |  |  |  |  |  |
| -0.9854 | 10 | igd | 0.5276 | A | \|\| id:ebi-a-GCST90002032 | ebi-a-GCST90002032 | TRUE | G | 97474371 | 1.00E-200 | reported | 2920 | 0.02111 | rs10882655 |  |  |  |  |  |  |  |  |  |  |  |  |  |  |  |
| -0.2945 | 10 | igd | 0.5276 | A | \|\| id:ebi-a-GCST90002069 | ebi-a-GCST90002069 | TRUE | G | 97474371 | 7.84E-28 | reported | 2920 | 0.02665 | rs10882655 |  |  |  |  |  |  |  |  |  |  |  |  |  |  |  |
| 0.2308 | 6 | igd | 0.5272 | C | \|\| id:ebi-a-GCST90002112 | ebi-a-GCST90002112 | TRUE | T | 32174957 | 5.34E-09 | reported | 1635 | 0.03934 | rs3130290 |  |  |  |  |  |  |  |  |  |  |  |  |  |  |  |
| -0.2237 | 6 | igd | 0.5272 | T | \|\| id:ebi-a-GCST90002119 | ebi-a-GCST90002119 | TRUE | C | 29895215 | 1.27E-12 | reported | 2920 | 0.03138 | rs1655938 |  |  |  |  |  |  |  |  |  |  |  |  |  |  |  |
| 0.1526 | 6 | igd | 0.5264 | T | \|\| id:ebi-a-GCST90001887 | ebi-a-GCST90001887 | TRUE | C | 90957463 | 2.34E-08 | reported | 2920 | 0.02725 | rs3757247 |  |  |  |  |  |  |  |  |  |  |  |  |  |  |  |
| -0.5019 | 10 | igd | 0.5264 | A | \|\| id:ebi-a-GCST90002029 | ebi-a-GCST90002029 | TRUE | G | 97484554 | 7.23E-82 | reported | 2915 | 0.02536 | rs11188449 |  |  |  |  |  |  |  |  |  |  |  |  |  |  |  |
| -0.216 | 19 | igd | 0.5263 | C | \|\| id:ebi-a-GCST90001946 | ebi-a-GCST90001946 | TRUE | T | 52253707 | 2.31E-09 | reported | 1634 | 0.03595 | rs11673446 |  |  |  |  |  |  |  |  |  |  |  |  |  |  |  |
| -0.2082 | 19 | igd | 0.5263 | C | \|\| id:ebi-a-GCST90001949 | ebi-a-GCST90001949 | TRUE | T | 52253707 | 6.80E-09 | reported | 1634 | 0.03574 | rs11673446 |  |  |  |  |  |  |  |  |  |  |  |  |  |  |  |
| 0.1606 | 17 | igd | 0.5254 | T | \|\| id:ebi-a-GCST90001969 | ebi-a-GCST90001969 | TRUE | C | 33805730 | 2.34E-09 | reported | 3113 | 0.02681 | rs9912354 |  |  |  |  |  |  |  |  |  |  |  |  |  |  |  |
| -0.157 | 10 | igd | 0.5243 | A | \|\| id:ebi-a-GCST90001780 | ebi-a-GCST90001780 | TRUE | G | 6130226 | 2.44E-10 | reported | 3642 | 0.02474 | rs7911362 |  |  |  |  |  |  |  |  |  |  |  |  |  |  |  |
| -0.1423 | 22 | igd | 0.5241 | C | \|\| id:ebi-a-GCST90001711 | ebi-a-GCST90001711 | TRUE | G | 43114040 | 1.43E-08 | reported | 3657 | 0.02504 | rs2143919 |  |  |  |  |  |  |  |  |  |  |  |  |  |  |  |
| -0.1517 | 10 | igd | 0.5241 | A | \|\| id:ebi-a-GCST90001785 | ebi-a-GCST90001785 | TRUE | G | 6130226 | 9.62E-10 | reported | 3649 | 0.02474 | rs7911362 |  |  |  |  |  |  |  |  |  |  |  |  |  |  |  |
| -0.169 | 10 | igd | 0.5239 | A | \|\| id:ebi-a-GCST90001775 | ebi-a-GCST90001775 | TRUE | G | 6130226 | 1.41E-11 | reported | 3660 | 0.02494 | rs7911362 |  |  |  |  |  |  |  |  |  |  |  |  |  |  |  |
| -0.2553 | 10 | igd | 0.5239 | A | \|\| id:ebi-a-GCST90001777 | ebi-a-GCST90001777 | TRUE | G | 6130226 | 1.33E-25 | reported | 3657 | 0.02422 | rs7911362 |  |  |  |  |  |  |  |  |  |  |  |  |  |  |  |
| -0.2154 | 10 | igd | 0.5239 | A | \|\| id:ebi-a-GCST90001778 | ebi-a-GCST90001778 | TRUE | G | 6130226 | 2.07E-18 | reported | 3657 | 0.02447 | rs7911362 |  |  |  |  |  |  |  |  |  |  |  |  |  |  |  |
| -0.158 | 10 | igd | 0.5239 | A | \|\| id:ebi-a-GCST90001779 | ebi-a-GCST90001779 | TRUE | G | 6130226 | 2.23E-10 | reported | 3657 | 0.02483 | rs7911362 |  |  |  |  |  |  |  |  |  |  |  |  |  |  |  |
| -0.15 | 10 | igd | 0.5239 | A | \|\| id:ebi-a-GCST90001784 | ebi-a-GCST90001784 | TRUE | G | 6130226 | 2.23E-09 | reported | 3657 | 0.02502 | rs7911362 |  |  |  |  |  |  |  |  |  |  |  |  |  |  |  |
| -0.238 | 10 | igd | 0.5239 | A | \|\| id:ebi-a-GCST90001789 | ebi-a-GCST90001789 | TRUE | G | 6130226 | 1.05E-22 | reported | 3657 | 0.02411 | rs7911362 |  |  |  |  |  |  |  |  |  |  |  |  |  |  |  |
| -0.2451 | 10 | igd | 0.5239 | A | \|\| id:ebi-a-GCST90001790 | ebi-a-GCST90001790 | TRUE | G | 6130226 | 1.13E-23 | reported | 3657 | 0.02427 | rs7911362 |  |  |  |  |  |  |  |  |  |  |  |  |  |  |  |
| -0.1589 | 10 | igd | 0.5239 | A | \|\| id:ebi-a-GCST90001791 | ebi-a-GCST90001791 | TRUE | G | 6130226 | 2.37E-10 | reported | 3657 | 0.02502 | rs7911362 |  |  |  |  |  |  |  |  |  |  |  |  |  |  |  |
| -0.2302 | 10 | igd | 0.5239 | A | \|\| id:ebi-a-GCST90001792 | ebi-a-GCST90001792 | TRUE | G | 6130226 | 5.05E-21 | reported | 3657 | 0.02432 | rs7911362 |  |  |  |  |  |  |  |  |  |  |  |  |  |  |  |
| -0.1505 | 10 | igd | 0.5239 | A | \|\| id:ebi-a-GCST90001794 | ebi-a-GCST90001794 | TRUE | G | 6130226 | 1.80E-09 | reported | 3658 | 0.02495 | rs7911362 |  |  |  |  |  |  |  |  |  |  |  |  |  |  |  |
| 0.1737 | 6 | igd | 0.5238 | A | \|\| id:ebi-a-GCST90002119 | ebi-a-GCST90002119 | TRUE | C | 31329135 | 8.29E-09 | reported | 2920 | 0.03005 | rs2428493 |  |  |  |  |  |  |  |  |  |  |  |  |  |  |  |
| -0.1728 | 22 | igd | 0.5235 | G | \|\| id:ebi-a-GCST90001702 | ebi-a-GCST90001702 | TRUE | A | 43714716 | 2.26E-12 | reported | 3657 | 0.02454 | rs5996309 |  |  |  |  |  |  |  |  |  |  |  |  |  |  |  |
| -0.1848 | 22 | igd | 0.5235 | G | \|\| id:ebi-a-GCST90001710 | ebi-a-GCST90001710 | TRUE | A | 43714716 | 5.09E-14 | reported | 3657 | 0.02444 | rs5996309 |  |  |  |  |  |  |  |  |  |  |  |  |  |  |  |
| -0.1814 | 22 | igd | 0.5235 | G | \|\| id:ebi-a-GCST90001711 | ebi-a-GCST90001711 | TRUE | A | 43714716 | 1.27E-13 | reported | 3657 | 0.02439 | rs5996309 |  |  |  |  |  |  |  |  |  |  |  |  |  |  |  |
| 0.1488 | 6 | igd | 0.5224 | G | \|\| id:ebi-a-GCST90001889 | ebi-a-GCST90001889 | TRUE | C | 90958231 | 2.80E-08 | reported | 2920 | 0.02672 | rs11755527 |  |  |  |  |  |  |  |  |  |  |  |  |  |  |  |
| 0.1596 | 6 | igd | 0.5224 | G | \|\| id:ebi-a-GCST90001899 | ebi-a-GCST90001899 | TRUE | C | 90958231 | 9.68E-10 | reported | 2920 | 0.02601 | rs11755527 |  |  |  |  |  |  |  |  |  |  |  |  |  |  |  |
| 0.1662 | 17 | igd | 0.5219 | A | \|\| id:ebi-a-GCST90001663 | ebi-a-GCST90001663 | TRUE | G | 33803592 | 1.05E-10 | reported | 3408 | 0.02565 | rs58745116 |  |  |  |  |  |  |  |  |  |  |  |  |  |  |  |
| -0.2128 | 12 | igd | 0.5218 | T | \|\| id:ebi-a-GCST90002073 | ebi-a-GCST90002073 | TRUE | C | 69592214 | 1.19E-14 | reported | 2850 | 0.02743 | rs10878940 |  |  |  |  |  |  |  |  |  |  |  |  |  |  |  |
| -0.2249 | 19 | igd | 0.52153 | T | \|\| id:prot-a-439 | prot-a-439 | TRUE | C | 51866319 | 4.07E-20 | reported | 3301 | 0.0245 | rs10411180 |  |  |  |  |  |  |  |  |  |  |  |  |  |  |  |
| 0.1462 | 17 | igd | 0.5212 | A | \|\| id:ebi-a-GCST90001661 | ebi-a-GCST90001661 | TRUE | G | 33803592 | 1.10E-08 | reported | 3440 | 0.02553 | rs58745116 |  |  |  |  |  |  |  |  |  |  |  |  |  |  |  |
| -0.1343 | 7 | igd | 0.5205 | C | \|\| id:ebi-a-GCST90001759 | ebi-a-GCST90001759 | TRUE | A | 40190449 | 3.93E-08 | reported | 3657 | 0.0244 | rs388354 |  |  |  |  |  |  |  |  |  |  |  |  |  |  |  |
| -0.1714 | 6 | igd | 0.52 | T | \|\| id:ebi-a-GCST90001932 | ebi-a-GCST90001932 | TRUE | A | 31241761 | 6.60E-09 | reported | 2920 | 0.02947 | rs2524082 |  |  |  |  |  |  |  |  |  |  |  |  |  |  |  |
| -0.2253 | 10 | igd | 0.5199 | A | \|\| id:ebi-a-GCST90001782 | ebi-a-GCST90001782 | TRUE | G | 6130226 | 7.90E-12 | reported | 1837 | 0.03273 | rs7911362 |  |  |  |  |  |  |  |  |  |  |  |  |  |  |  |
| 0.2522 | 19 | igd | 0.5197 | G | \|\| id:ebi-a-GCST90001516 | ebi-a-GCST90001516 | TRUE | A | 51728641 | 1.86E-14 | reported | 1857 | 0.03266 | rs2455069 |  |  |  |  |  |  |  |  |  |  |  |  |  |  |  |
| -0.4103 | 19 | igd | 0.5191 | A | \|\| id:ebi-a-GCST90001523 | ebi-a-GCST90001523 | TRUE | T | 51718317 | 1.73E-35 | reported | 1858 | 0.03231 | rs1697573 |  |  |  |  |  |  |  |  |  |  |  |  |  |  |  |
| -0.1911 | 1 | igd | 0.5185 | T | \|\| id:ebi-a-GCST90001854 | ebi-a-GCST90001854 | TRUE | C | 168708106 | 1.66E-12 | reported | 2919 | 0.02695 | rs609629 |  |  |  |  |  |  |  |  |  |  |  |  |  |  |  |
| -0.1588 | 1 | igd | 0.5185 | T | \|\| id:ebi-a-GCST90001856 | ebi-a-GCST90001856 | TRUE | C | 168708106 | 6.16E-09 | reported | 2919 | 0.02724 | rs609629 |  |  |  |  |  |  |  |  |  |  |  |  |  |  |  |
| -0.2824 | 19 | igd | 0.5165 | A | \|\| id:ebi-a-GCST90001947 | ebi-a-GCST90001947 | TRUE | G | 51595288 | 2.03E-14 | reported | 1577 | 0.03658 | rs34974522 |  |  |  |  |  |  |  |  |  |  |  |  |  |  |  |
| 0.207 | 2 | igd | 0.5164 | A | \|\| id:ebi-a-GCST90002119 | ebi-a-GCST90002119 | TRUE | G | 86908250 | 1.74E-14 | reported | 2920 | 0.02685 | rs4432450 |  |  |  |  |  |  |  |  |  |  |  |  |  |  |  |
| -0.1619 | 1 | igd | 0.5161 | C | \|\| id:ebi-a-GCST90001858 | ebi-a-GCST90001858 | TRUE | T | 168700436 | 3.07E-09 | reported | 2919 | 0.02723 | rs1556792 |  |  |  |  |  |  |  |  |  |  |  |  |  |  |  |
| -0.1748 | 1 | igd | 0.5161 | G | \|\| id:ebi-a-GCST90001860 | ebi-a-GCST90001860 | TRUE | A | 168700562 | 1.80E-10 | reported | 2919 | 0.02731 | rs1556790 |  |  |  |  |  |  |  |  |  |  |  |  |  |  |  |
| -0.1597 | 10 | igd | 0.5156 | C | \|\| id:ebi-a-GCST90001892 | ebi-a-GCST90001892 | TRUE | A | 97741071 | 7.66E-09 | reported | 2920 | 0.02758 | rs10882701 |  |  |  |  |  |  |  |  |  |  |  |  |  |  |  |
| 0.236 | 10 | igd | 0.5156 | C | \|\| id:ebi-a-GCST90001961 | ebi-a-GCST90001961 | TRUE | A | 97741071 | 1.98E-17 | reported | 2920 | 0.02761 | rs10882701 |  |  |  |  |  |  |  |  |  |  |  |  |  |  |  |
| -0.4542 | 10 | igd | 0.5156 | C | \|\| id:ebi-a-GCST90002030 | ebi-a-GCST90002030 | TRUE | A | 97741071 | 4.05E-64 | reported | 2920 | 0.02621 | rs10882701 |  |  |  |  |  |  |  |  |  |  |  |  |  |  |  |
| -0.3988 | 10 | igd | 0.5156 | C | \|\| id:ebi-a-GCST90002031 | ebi-a-GCST90002031 | TRUE | A | 97741071 | 7.28E-47 | reported | 2920 | 0.02725 | rs10882701 |  |  |  |  |  |  |  |  |  |  |  |  |  |  |  |
| -0.3837 | 10 | igd | 0.5156 | C | \|\| id:ebi-a-GCST90002032 | ebi-a-GCST90002032 | TRUE | A | 97741071 | 1.78E-43 | reported | 2920 | 0.0273 | rs10882701 |  |  |  |  |  |  |  |  |  |  |  |  |  |  |  |
| -0.1936 | 10 | igd | 0.5153 | C | \|\| id:ebi-a-GCST90002029 | ebi-a-GCST90002029 | TRUE | A | 97741071 | 1.87E-12 | reported | 2915 | 0.02737 | rs10882701 |  |  |  |  |  |  |  |  |  |  |  |  |  |  |  |
| -0.5102 | 6 | igd | 0.5153 | A | \|\| id:ebi-a-GCST90002110 | ebi-a-GCST90002110 | TRUE | G | 32528256 | 3.48E-08 | reported | 1634 | 0.09207 | rs113508557 |  |  |  |  |  |  |  |  |  |  |  |  |  |  |  |
| -0.1415 | 10 | igd | 0.5148 | C | \|\| id:ebi-a-GCST90001496 | ebi-a-GCST90001496 | TRUE | G | 91716238 | 4.26E-08 | reported | 3437 | 0.02576 | rs2211499 |  |  |  |  |  |  |  |  |  |  |  |  |  |  |  |
| -0.3681 | 10 | igd | 0.5141 | C | \|\| id:ebi-a-GCST90001484 | ebi-a-GCST90001484 | TRUE | A | 97741071 | 6.66E-50 | reported | 3437 | 0.02438 | rs10882701 |  |  |  |  |  |  |  |  |  |  |  |  |  |  |  |
| -0.3593 | 10 | igd | 0.5141 | C | \|\| id:ebi-a-GCST90001485 | ebi-a-GCST90001485 | TRUE | A | 97741071 | 1.08E-47 | reported | 3437 | 0.02438 | rs10882701 |  |  |  |  |  |  |  |  |  |  |  |  |  |  |  |
| -0.3538 | 10 | igd | 0.5141 | C | \|\| id:ebi-a-GCST90001491 | ebi-a-GCST90001491 | TRUE | A | 97741071 | 1.32E-44 | reported | 3437 | 0.02489 | rs10882701 |  |  |  |  |  |  |  |  |  |  |  |  |  |  |  |
| -0.4034 | 10 | igd | 0.5141 | C | \|\| id:ebi-a-GCST90001496 | ebi-a-GCST90001496 | TRUE | A | 97741071 | 2.04E-56 | reported | 3437 | 0.02502 | rs10882701 |  |  |  |  |  |  |  |  |  |  |  |  |  |  |  |
| -0.3962 | 10 | igd | 0.5141 | C | \|\| id:ebi-a-GCST90001497 | ebi-a-GCST90001497 | TRUE | A | 97741071 | 8.03E-56 | reported | 3437 | 0.02472 | rs10882701 |  |  |  |  |  |  |  |  |  |  |  |  |  |  |  |
| -0.3969 | 10 | igd | 0.5141 | C | \|\| id:ebi-a-GCST90001658 | ebi-a-GCST90001658 | TRUE | A | 97741071 | 3.17E-60 | reported | 3440 | 0.02378 | rs10882701 |  |  |  |  |  |  |  |  |  |  |  |  |  |  |  |
| -0.3997 | 10 | igd | 0.5141 | C | \|\| id:ebi-a-GCST90001659 | ebi-a-GCST90001659 | TRUE | A | 97741071 | 4.54E-61 | reported | 3440 | 0.02377 | rs10882701 |  |  |  |  |  |  |  |  |  |  |  |  |  |  |  |
| -0.2934 | 10 | igd | 0.5141 | C | \|\| id:ebi-a-GCST90001670 | ebi-a-GCST90001670 | TRUE | A | 97741071 | 1.28E-30 | reported | 3440 | 0.02526 | rs10882701 |  |  |  |  |  |  |  |  |  |  |  |  |  |  |  |
| -0.3033 | 10 | igd | 0.5141 | C | \|\| id:ebi-a-GCST90001671 | ebi-a-GCST90001671 | TRUE | A | 97741071 | 1.34E-32 | reported | 3440 | 0.02524 | rs10882701 |  |  |  |  |  |  |  |  |  |  |  |  |  |  |  |
| -0.3551 | 10 | igd | 0.514 | C | \|\| id:ebi-a-GCST90001483 | ebi-a-GCST90001483 | TRUE | A | 97741071 | 7.12E-46 | reported | 3405 | 0.0246 | rs10882701 |  |  |  |  |  |  |  |  |  |  |  |  |  |  |  |
| -0.3631 | 10 | igd | 0.514 | C | \|\| id:ebi-a-GCST90001489 | ebi-a-GCST90001489 | TRUE | A | 97741071 | 1.80E-45 | reported | 3405 | 0.02528 | rs10882701 |  |  |  |  |  |  |  |  |  |  |  |  |  |  |  |
| -0.4069 | 10 | igd | 0.514 | C | \|\| id:ebi-a-GCST90001495 | ebi-a-GCST90001495 | TRUE | A | 97741071 | 3.19E-56 | reported | 3405 | 0.02528 | rs10882701 |  |  |  |  |  |  |  |  |  |  |  |  |  |  |  |
| -0.4118 | 10 | igd | 0.5139 | C | \|\| id:ebi-a-GCST90001660 | ebi-a-GCST90001660 | TRUE | A | 97741071 | 3.85E-59 | reported | 3408 | 0.0249 | rs10882701 |  |  |  |  |  |  |  |  |  |  |  |  |  |  |  |
| -0.3048 | 10 | igd | 0.5139 | C | \|\| id:ebi-a-GCST90001672 | ebi-a-GCST90001672 | TRUE | A | 97741071 | 5.04E-32 | reported | 3408 | 0.02561 | rs10882701 |  |  |  |  |  |  |  |  |  |  |  |  |  |  |  |
| 0.1514 | 3 | igd | 0.513 | T | \|\| id:ebi-a-GCST90002004 | ebi-a-GCST90002004 | TRUE | C | 46459490 | 1.28E-09 | reported | 3629 | 0.02488 | rs66484806 |  |  |  |  |  |  |  |  |  |  |  |  |  |  |  |
| -0.2226 | 21 | igd | 0.5129 | A | \|\| id:ebi-a-GCST90002096 | ebi-a-GCST90002096 | TRUE | G | 46340512 | 2.35E-09 | reported | 1556 | 0.03706 | rs760453 |  |  |  |  |  |  |  |  |  |  |  |  |  |  |  |
| -0.1522 | 22 | igd | 0.5126 | C | \|\| id:ebi-a-GCST90001711 | ebi-a-GCST90001711 | TRUE | T | 43573946 | 8.17E-10 | reported | 3657 | 0.02471 | rs6519365 |  |  |  |  |  |  |  |  |  |  |  |  |  |  |  |
| -0.128407 | 1 | igd | 0.511983 | G | \|\| id:met-b-144 | met-b-144 | TRUE | A | 161479745 | 6.48E-11 | reported | 459 | 0.0196573 | rs1801274 |  |  |  |  |  |  |  |  |  |  |  |  |  |  |  |
| -0.135905 | 1 | igd | 0.511983 | G | \|\| id:met-b-146 | met-b-146 | TRUE | A | 161479745 | 5.38E-12 | reported | 459 | 0.0197101 | rs1801274 |  |  |  |  |  |  |  |  |  |  |  |  |  |  |  |
| -0.145366 | 1 | igd | 0.511957 | G | \|\| id:met-b-155 | met-b-155 | TRUE | A | 161479745 | 5.43E-14 | reported | 460 | 0.0193274 | rs1801274 |  |  |  |  |  |  |  |  |  |  |  |  |  |  |  |
| -0.186207 | 1 | igd | 0.511957 | G | \|\| id:met-b-157 | met-b-157 | TRUE | A | 161479745 | 1.26E-21 | reported | 460 | 0.0194914 | rs1801274 |  |  |  |  |  |  |  |  |  |  |  |  |  |  |  |
| 0.219 | 3 | igd | 0.5119 | T | \|\| id:ebi-a-GCST90002017 | ebi-a-GCST90002017 | TRUE | C | 46459490 | 1.48E-14 | reported | 2850 | 0.02832 | rs66484806 |  |  |  |  |  |  |  |  |  |  |  |  |  |  |  |
| 0.1259 | 6 | igd | 0.5109 | G | \|\| id:ebi-a-GCST90001690 | ebi-a-GCST90001690 | TRUE | A | 31415065 | 2.27E-09 | reported | 3408 | 0.021 | rs3131627 |  |  |  |  |  |  |  |  |  |  |  |  |  |  |  |
| 0.1227 | 6 | igd | 0.5105 | G | \|\| id:ebi-a-GCST90001688 | ebi-a-GCST90001688 | TRUE | A | 31415065 | 7.73E-09 | reported | 3440 | 0.0212 | rs3131627 |  |  |  |  |  |  |  |  |  |  |  |  |  |  |  |
| -0.2538 | 19 | igd | 0.5105 | G | \|\| id:ebi-a-GCST90001950 | ebi-a-GCST90001950 | TRUE | A | 51659714 | 1.02E-09 | reported | 1244 | 0.04124 | rs273665 |  |  |  |  |  |  |  |  |  |  |  |  |  |  |  |
| 0.1398 | 6 | igd | 0.5103 | T | \|\| id:ebi-a-GCST90001560 | ebi-a-GCST90001560 | TRUE | G | 31417540 | 2.72E-08 | reported | 3395 | 0.02509 | rs2596459 |  |  |  |  |  |  |  |  |  |  |  |  |  |  |  |
| 0.1901 | 1 | igd | 0.5101 | G | \|\| id:ebi-a-GCST90001970 | ebi-a-GCST90001970 | TRUE | A | 161229651 | 3.64E-12 | reported | 2971 | 0.02723 | rs4078109 |  |  |  |  |  |  |  |  |  |  |  |  |  |  |  |
| 0.1499 | 6 | igd | 0.5099 | T | \|\| id:ebi-a-GCST90001562 | ebi-a-GCST90001562 | TRUE | G | 31417540 | 1.65E-08 | reported | 3427 | 0.02649 | rs2596459 |  |  |  |  |  |  |  |  |  |  |  |  |  |  |  |
| 0.116312 | 1 | igd | 0.509847 | G | \|\| id:met-b-143 | met-b-143 | TRUE | A | 161479745 | 2.91E-09 | reported | 457 | 0.0195919 | rs1801274 |  |  |  |  |  |  |  |  |  |  |  |  |  |  |  |
| -0.153 | 10 | igd | 0.5093 | A | \|\| id:ebi-a-GCST90001670 | ebi-a-GCST90001670 | TRUE | T | 97216354 | 3.57E-09 | reported | 3440 | 0.02585 | rs10786213 |  |  |  |  |  |  |  |  |  |  |  |  |  |  |  |
| -0.1498 | 10 | igd | 0.5093 | A | \|\| id:ebi-a-GCST90001671 | ebi-a-GCST90001671 | TRUE | T | 97216354 | 7.99E-09 | reported | 3440 | 0.0259 | rs10786213 |  |  |  |  |  |  |  |  |  |  |  |  |  |  |  |
| 0.1733 | 2 | igd | 0.5089 | T | \|\| id:ebi-a-GCST90001965 | ebi-a-GCST90001965 | TRUE | G | 203467470 | 1.89E-09 | reported | 2850 | 0.02875 | rs77762937 |  |  |  |  |  |  |  |  |  |  |  |  |  |  |  |
| -0.1574 | 10 | igd | 0.5085 | A | \|\| id:ebi-a-GCST90001672 | ebi-a-GCST90001672 | TRUE | T | 97216354 | 2.34E-09 | reported | 3408 | 0.02628 | rs10786213 |  |  |  |  |  |  |  |  |  |  |  |  |  |  |  |
| 0.2141 | 19 | igd | 0.5083 | G | \|\| id:ebi-a-GCST90001523 | ebi-a-GCST90001523 | TRUE | A | 51659714 | 1.12E-10 | reported | 1858 | 0.03301 | rs273665 |  |  |  |  |  |  |  |  |  |  |  |  |  |  |  |
| 0.2132 | 19 | igd | 0.508 | T | \|\| id:ebi-a-GCST90001954 | ebi-a-GCST90001954 | TRUE | G | 51592564 | 9.44E-09 | reported | 1555 | 0.03694 | rs2091181 |  |  |  |  |  |  |  |  |  |  |  |  |  |  |  |
| 0.208 | 19 | igd | 0.5071 | T | \|\| id:ebi-a-GCST90001953 | ebi-a-GCST90001953 | TRUE | G | 51592564 | 2.05E-08 | reported | 1558 | 0.03689 | rs2091181 |  |  |  |  |  |  |  |  |  |  |  |  |  |  |  |
| 0.1506 | 22 | igd | 0.5068 | C | \|\| id:ebi-a-GCST90001702 | ebi-a-GCST90001702 | TRUE | T | 43066379 | 2.25E-09 | reported | 3657 | 0.02512 | rs130333 |  |  |  |  |  |  |  |  |  |  |  |  |  |  |  |
| 0.1505 | 22 | igd | 0.5068 | C | \|\| id:ebi-a-GCST90001703 | ebi-a-GCST90001703 | TRUE | T | 43066379 | 2.27E-09 | reported | 3657 | 0.02512 | rs130333 |  |  |  |  |  |  |  |  |  |  |  |  |  |  |  |
| 0.1603 | 22 | igd | 0.5068 | C | \|\| id:ebi-a-GCST90001710 | ebi-a-GCST90001710 | TRUE | T | 43066379 | 1.70E-10 | reported | 3657 | 0.02504 | rs130333 |  |  |  |  |  |  |  |  |  |  |  |  |  |  |  |
| 0.1567 | 22 | igd | 0.5068 | C | \|\| id:ebi-a-GCST90001711 | ebi-a-GCST90001711 | TRUE | T | 43066379 | 3.90E-10 | reported | 3657 | 0.02498 | rs130333 |  |  |  |  |  |  |  |  |  |  |  |  |  |  |  |
| -0.2356 | 19 | igd | 0.5068 | A | \|\| id:ebi-a-GCST90002052 | ebi-a-GCST90002052 | TRUE | G | 51818795 | 2.49E-10 | reported | 1555 | 0.037 | rs11668607 |  |  |  |  |  |  |  |  |  |  |  |  |  |  |  |
| -0.3673 | 19 | igd | 0.5061 | G | \|\| id:ebi-a-GCST90001946 | ebi-a-GCST90001946 | TRUE | A | 51821634 | 6.62E-25 | reported | 1634 | 0.03505 | rs2411317 |  |  |  |  |  |  |  |  |  |  |  |  |  |  |  |
| -0.3293 | 19 | igd | 0.5061 | G | \|\| id:ebi-a-GCST90001949 | ebi-a-GCST90001949 | TRUE | A | 51821634 | 1.77E-20 | reported | 1634 | 0.03503 | rs2411317 |  |  |  |  |  |  |  |  |  |  |  |  |  |  |  |
| -0.3465 | 19 | igd | 0.5058 | G | \|\| id:ebi-a-GCST90001948 | ebi-a-GCST90001948 | TRUE | A | 51821634 | 1.88E-22 | reported | 1633 | 0.03502 | rs2411317 |  |  |  |  |  |  |  |  |  |  |  |  |  |  |  |
| 0.2175 | 19 | igd | 0.5054 | T | \|\| id:ebi-a-GCST90001952 | ebi-a-GCST90001952 | TRUE | G | 51592564 | 4.63E-09 | reported | 1569 | 0.03691 | rs2091181 |  |  |  |  |  |  |  |  |  |  |  |  |  |  |  |
| 0.2148 | 21 | igd | 0.5051 | T | \|\| id:ebi-a-GCST90002095 | ebi-a-GCST90002095 | TRUE | A | 46334076 | 8.67E-09 | reported | 1559 | 0.03712 | rs12626729 |  |  |  |  |  |  |  |  |  |  |  |  |  |  |  |
| 0.2368 | 21 | igd | 0.5048 | T | \|\| id:ebi-a-GCST90002096 | ebi-a-GCST90002096 | TRUE | A | 46334076 | 1.74E-10 | reported | 1556 | 0.03685 | rs12626729 |  |  |  |  |  |  |  |  |  |  |  |  |  |  |  |
| 0.3299 | 2 | igd | 0.5047 | G | \|\| id:ebi-a-GCST90001963 | ebi-a-GCST90001963 | TRUE | A | 203591967 | 4.66E-31 | reported | 2872 | 0.02813 | rs72928971 |  |  |  |  |  |  |  |  |  |  |  |  |  |  |  |
| 0.2991 | 16 | igd | 0.5047 | A | \|\| id:ebi-a-GCST90002089 | ebi-a-GCST90002089 | TRUE | T | 31365464 | 8.30E-27 | reported | 2850 | 0.02762 | rs7199570 |  |  |  |  |  |  |  |  |  |  |  |  |  |  |  |
| 0.2346 | 16 | igd | 0.5047 | A | \|\| id:ebi-a-GCST90002090 | ebi-a-GCST90002090 | TRUE | T | 31365464 | 1.36E-16 | reported | 2850 | 0.02821 | rs7199570 |  |  |  |  |  |  |  |  |  |  |  |  |  |  |  |
| 0.164 | 3 | igd | 0.5046 | G | \|\| id:ebi-a-GCST90002017 | ebi-a-GCST90002017 | TRUE | C | 46468641 | 6.07E-09 | reported | 2850 | 0.02811 | rs2097279 |  |  |  |  |  |  |  |  |  |  |  |  |  |  |  |
| 0.2775 | 2 | igd | 0.5045 | T | \|\| id:ebi-a-GCST90002071 | ebi-a-GCST90002071 | TRUE | A | 203545127 | 1.56E-21 | reported | 2872 | 0.02889 | rs113138131 |  |  |  |  |  |  |  |  |  |  |  |  |  |  |  |
| 0.2375 | 19 | igd | 0.5041 | T | \|\| id:ebi-a-GCST90001947 | ebi-a-GCST90001947 | TRUE | G | 51592564 | 1.39E-10 | reported | 1577 | 0.03676 | rs2091181 |  |  |  |  |  |  |  |  |  |  |  |  |  |  |  |
| -0.2873 | 19 | igd | 0.5041 | G | \|\| id:ebi-a-GCST90001951 | ebi-a-GCST90001951 | TRUE | A | 51821634 | 5.90E-14 | reported | 1464 | 0.03788 | rs2411317 |  |  |  |  |  |  |  |  |  |  |  |  |  |  |  |
| 0.3291 | 19 | igd | 0.5038 | A | \|\| id:ebi-a-GCST90001952 | ebi-a-GCST90001952 | TRUE | G | 51818795 | 5.52E-19 | reported | 1569 | 0.0365 | rs11668607 |  |  |  |  |  |  |  |  |  |  |  |  |  |  |  |
| 0.2428 | 19 | igd | 0.5038 | T | \|\| id:ebi-a-GCST90001956 | ebi-a-GCST90001956 | TRUE | G | 51592564 | 4.71E-11 | reported | 1579 | 0.03663 | rs2091181 |  |  |  |  |  |  |  |  |  |  |  |  |  |  |  |
| 0.2415 | 19 | igd | 0.5038 | T | \|\| id:ebi-a-GCST90001957 | ebi-a-GCST90001957 | TRUE | G | 51592564 | 6.11E-11 | reported | 1578 | 0.03666 | rs2091181 |  |  |  |  |  |  |  |  |  |  |  |  |  |  |  |
| 0.2588 | 2 | igd | 0.5036 | G | \|\| id:ebi-a-GCST90002101 | ebi-a-GCST90002101 | TRUE | C | 38921934 | 1.86E-21 | reported | 2911 | 0.02699 | rs7583259 |  |  |  |  |  |  |  |  |  |  |  |  |  |  |  |
| -0.252 | 6 | igd | 0.5035 | T | \|\| id:ebi-a-GCST90002108 | ebi-a-GCST90002108 | TRUE | C | 32899296 | 1.18E-10 | reported | 1579 | 0.03885 | rs154978 |  |  |  |  |  |  |  |  |  |  |  |  |  |  |  |
| 0.2378 | 2 | igd | 0.5023 | G | \|\| id:ebi-a-GCST90001562 | ebi-a-GCST90001562 | TRUE | C | 38921934 | 1.64E-26 | reported | 3427 | 0.02213 | rs7583259 |  |  |  |  |  |  |  |  |  |  |  |  |  |  |  |
| 0.1794 | 2 | igd | 0.5021 | G | \|\| id:ebi-a-GCST90001666 | ebi-a-GCST90001666 | TRUE | C | 38921934 | 8.34E-16 | reported | 3408 | 0.02218 | rs7583259 |  |  |  |  |  |  |  |  |  |  |  |  |  |  |  |
| 0.1951 | 2 | igd | 0.502 | G | \|\| id:ebi-a-GCST90001664 | ebi-a-GCST90001664 | TRUE | C | 38921934 | 8.41E-17 | reported | 3440 | 0.02331 | rs7583259 |  |  |  |  |  |  |  |  |  |  |  |  |  |  |  |
| 0.1741 | 2 | igd | 0.502 | G | \|\| id:ebi-a-GCST90001665 | ebi-a-GCST90001665 | TRUE | C | 38921934 | 2.61E-12 | reported | 3440 | 0.02479 | rs7583259 |  |  |  |  |  |  |  |  |  |  |  |  |  |  |  |
| 0.104 | 2 | igd | 0.502 | G | \|\| id:ebi-a-GCST90001688 | ebi-a-GCST90001688 | TRUE | C | 38921934 | 3.71E-09 | reported | 3440 | 0.01759 | rs7583259 |  |  |  |  |  |  |  |  |  |  |  |  |  |  |  |
| -0.1539 | 10 | igd | 0.5019 | A | \|\| id:ebi-a-GCST90001892 | ebi-a-GCST90001892 | TRUE | C | 98165998 | 2.48E-08 | reported | 2920 | 0.02753 | rs7905873 |  |  |  |  |  |  |  |  |  |  |  |  |  |  |  |
| 0.361 | 19 | igd | 0.5019 | A | \|\| id:ebi-a-GCST90001947 | ebi-a-GCST90001947 | TRUE | G | 51818795 | 8.39E-23 | reported | 1577 | 0.03616 | rs11668607 |  |  |  |  |  |  |  |  |  |  |  |  |  |  |  |
| 0.1786 | 10 | igd | 0.5019 | A | \|\| id:ebi-a-GCST90001961 | ebi-a-GCST90001961 | TRUE | C | 98165998 | 1.40E-10 | reported | 2920 | 0.02774 | rs7905873 |  |  |  |  |  |  |  |  |  |  |  |  |  |  |  |
| 0.3519 | 19 | igd | 0.5016 | A | \|\| id:ebi-a-GCST90001956 | ebi-a-GCST90001956 | TRUE | G | 51818795 | 7.94E-22 | reported | 1579 | 0.03612 | rs11668607 |  |  |  |  |  |  |  |  |  |  |  |  |  |  |  |
| 0.3608 | 6 | igd | 0.5016 | A | \|\| id:ebi-a-GCST90002109 | ebi-a-GCST90002109 | TRUE | G | 32583603 | 4.10E-17 | reported | 1578 | 0.04242 | rs28752515 |  |  |  |  |  |  |  |  |  |  |  |  |  |  |  |
| -0.294 | 6 | igd | 0.5014 | T | \|\| id:ebi-a-GCST90002105 | ebi-a-GCST90002105 | TRUE | C | 32736005 | 8.65E-22 | reported | 2872 | 0.03041 | rs9296042 |  |  |  |  |  |  |  |  |  |  |  |  |  |  |  |
| 0.1387 | 10 | igd | 0.501 | G | \|\| id:ebi-a-GCST90001491 | ebi-a-GCST90001491 | TRUE | T | 92948478 | 3.63E-08 | reported | 3437 | 0.02512 | rs10881900 |  |  |  |  |  |  |  |  |  |  |  |  |  |  |  |
| 0.1574 | 10 | igd | 0.501 | G | \|\| id:ebi-a-GCST90001496 | ebi-a-GCST90001496 | TRUE | T | 92948478 | 6.75E-10 | reported | 3437 | 0.02543 | rs10881900 |  |  |  |  |  |  |  |  |  |  |  |  |  |  |  |
| 0.1471 | 10 | igd | 0.501 | G | \|\| id:ebi-a-GCST90001497 | ebi-a-GCST90001497 | TRUE | T | 92948478 | 5.29E-09 | reported | 3437 | 0.02513 | rs10881900 |  |  |  |  |  |  |  |  |  |  |  |  |  |  |  |
| 0.1594 | 2 | igd | 0.5009 | C | \|\| id:ebi-a-GCST90002073 | ebi-a-GCST90002073 | TRUE | G | 203570761 | 4.32E-08 | reported | 2850 | 0.02902 | rs72928949 |  |  |  |  |  |  |  |  |  |  |  |  |  |  |  |
| 0.142 | 10 | igd | 0.5007 | G | \|\| id:ebi-a-GCST90001659 | ebi-a-GCST90001659 | TRUE | T | 92948478 | 5.13E-09 | reported | 3440 | 0.02424 | rs10881900 |  |  |  |  |  |  |  |  |  |  |  |  |  |  |  |
| 0.1681 | 6 | igd | 0.5003 | A | \|\| id:ebi-a-GCST90002121 | ebi-a-GCST90002121 | TRUE | T | 31072822 | 4.66E-09 | reported | 2915 | 0.0286 | rs9263613 |  |  |  |  |  |  |  |  |  |  |  |  |  |  |  |
| 0.1702 | 6 | igd | 0.5001 | T | \|\| id:ebi-a-GCST90001747 | ebi-a-GCST90001747 | TRUE | C | 32583477 | 4.09E-09 | reported | 3657 | 0.02888 | rs28383223 |  |  |  |  |  |  |  |  |  |  |  |  |  |  |  |
| 0.3426 | 2 | igd | 0.4991 | T | \|\| id:ebi-a-GCST90001963 | ebi-a-GCST90001963 | TRUE | G | 203485086 | 2.21E-33 | reported | 2872 | 0.0281 | rs72926952 |  |  |  |  |  |  |  |  |  |  |  |  |  |  |  |
| -0.2653 | 6 | igd | 0.4991 | T | \|\| id:ebi-a-GCST90002112 | ebi-a-GCST90002112 | TRUE | C | 32454327 | 6.48E-10 | reported | 1635 | 0.04268 | rs9269254 |  |  |  |  |  |  |  |  |  |  |  |  |  |  |  |
| -0.1641 | 10 | igd | 0.499 | T | \|\| id:ebi-a-GCST90001658 | ebi-a-GCST90001658 | TRUE | G | 96012950 | 6.20E-11 | reported | 3440 | 0.02501 | rs7080472 |  |  |  |  |  |  |  |  |  |  |  |  |  |  |  |
| -0.1432 | 10 | igd | 0.499 | T | \|\| id:ebi-a-GCST90001670 | ebi-a-GCST90001670 | TRUE | G | 96012950 | 4.05E-08 | reported | 3440 | 0.02603 | rs7080472 |  |  |  |  |  |  |  |  |  |  |  |  |  |  |  |
| -0.1449 | 10 | igd | 0.499 | T | \|\| id:ebi-a-GCST90001671 | ebi-a-GCST90001671 | TRUE | G | 96012950 | 3.08E-08 | reported | 3440 | 0.02612 | rs7080472 |  |  |  |  |  |  |  |  |  |  |  |  |  |  |  |
| -0.1913 | 10 | igd | 0.4988 | T | \|\| id:ebi-a-GCST90001497 | ebi-a-GCST90001497 | TRUE | G | 96012950 | 1.59E-13 | reported | 3437 | 0.02581 | rs7080472 |  |  |  |  |  |  |  |  |  |  |  |  |  |  |  |
| 0.1659 | 6 | igd | 0.4984 | A | \|\| id:ebi-a-GCST90001759 | ebi-a-GCST90001759 | TRUE | G | 32584153 | 6.07E-09 | reported | 3657 | 0.02845 | rs28383233 |  |  |  |  |  |  |  |  |  |  |  |  |  |  |  |
| -0.3523 | 19 | igd | 0.4984 | G | \|\| id:ebi-a-GCST90001957 | ebi-a-GCST90001957 | TRUE | A | 51821634 | 8.07E-22 | reported | 1578 | 0.03616 | rs2411317 |  |  |  |  |  |  |  |  |  |  |  |  |  |  |  |
| -0.1527 | 10 | igd | 0.4979 | T | \|\| id:ebi-a-GCST90001672 | ebi-a-GCST90001672 | TRUE | G | 96012950 | 8.77E-09 | reported | 3408 | 0.02648 | rs7080472 |  |  |  |  |  |  |  |  |  |  |  |  |  |  |  |
| 0.2423 | 19 | igd | 0.4979 | T | \|\| id:ebi-a-GCST90001946 | ebi-a-GCST90001946 | TRUE | G | 51592564 | 1.53E-11 | reported | 1634 | 0.03567 | rs2091181 |  |  |  |  |  |  |  |  |  |  |  |  |  |  |  |
| 0.253 | 19 | igd | 0.4979 | T | \|\| id:ebi-a-GCST90001949 | ebi-a-GCST90001949 | TRUE | G | 51592564 | 1.32E-12 | reported | 1634 | 0.03539 | rs2091181 |  |  |  |  |  |  |  |  |  |  |  |  |  |  |  |
| -0.1868 | 10 | igd | 0.4978 | T | \|\| id:ebi-a-GCST90001495 | ebi-a-GCST90001495 | TRUE | G | 96012950 | 1.87E-12 | reported | 3405 | 0.02643 | rs7080472 |  |  |  |  |  |  |  |  |  |  |  |  |  |  |  |
| -0.1758 | 1 | igd | 0.4978 | G | \|\| id:ebi-a-GCST90001999 | ebi-a-GCST90001999 | TRUE | A | 161269897 | 2.72E-12 | reported | 3621 | 0.02505 | rs12023218 |  |  |  |  |  |  |  |  |  |  |  |  |  |  |  |
| 0.2547 | 19 | igd | 0.4976 | T | \|\| id:ebi-a-GCST90001948 | ebi-a-GCST90001948 | TRUE | G | 51592564 | 1.08E-12 | reported | 1633 | 0.0355 | rs2091181 |  |  |  |  |  |  |  |  |  |  |  |  |  |  |  |
| -0.1536 | 2 | igd | 0.4963 | T | \|\| id:ebi-a-GCST90001666 | ebi-a-GCST90001666 | TRUE | G | 39022120 | 9.51E-12 | reported | 3408 | 0.02246 | rs34818399 |  |  |  |  |  |  |  |  |  |  |  |  |  |  |  |
| -0.1607 | 2 | igd | 0.4962 | T | \|\| id:ebi-a-GCST90001664 | ebi-a-GCST90001664 | TRUE | G | 39022120 | 1.35E-11 | reported | 3440 | 0.02368 | rs34818399 |  |  |  |  |  |  |  |  |  |  |  |  |  |  |  |
| -0.1522 | 2 | igd | 0.4962 | T | \|\| id:ebi-a-GCST90001665 | ebi-a-GCST90001665 | TRUE | G | 39022120 | 1.46E-09 | reported | 3440 | 0.0251 | rs34818399 |  |  |  |  |  |  |  |  |  |  |  |  |  |  |  |
| 0.1568 | 19 | igd | 0.496 | G | \|\| id:ebi-a-GCST90002090 | ebi-a-GCST90002090 | TRUE | A | 52138563 | 3.66E-08 | reported | 2850 | 0.0284 | rs10419309 |  |  |  |  |  |  |  |  |  |  |  |  |  |  |  |
| 2.316 | 2 | igd | 0.4959 | T | \|\| id:ebi-a-GCST90001696 | ebi-a-GCST90001696 | TRUE | G | 39022120 | 4.60E-14 | reported | 3425 | 0.3057 | rs34818399 |  |  |  |  |  |  |  |  |  |  |  |  |  |  |  |
| 0.7237 | 2 | igd | 0.4959 | T | \|\| id:ebi-a-GCST90001697 | ebi-a-GCST90001697 | TRUE | G | 39022120 | 8.48E-09 | reported | 3425 | 0.1254 | rs34818399 |  |  |  |  |  |  |  |  |  |  |  |  |  |  |  |
| -0.1681 | 2 | igd | 0.4958 | T | \|\| id:ebi-a-GCST90001562 | ebi-a-GCST90001562 | TRUE | G | 39022120 | 1.17E-13 | reported | 3427 | 0.02257 | rs34818399 |  |  |  |  |  |  |  |  |  |  |  |  |  |  |  |
| 0.2125 | 10 | igd | 0.4955 | T | \|\| id:ebi-a-GCST90001888 | ebi-a-GCST90001888 | TRUE | C | 97727916 | 1.90E-15 | reported | 2920 | 0.0266 | rs7921331 |  |  |  |  |  |  |  |  |  |  |  |  |  |  |  |
| 0.2781 | 10 | igd | 0.4955 | T | \|\| id:ebi-a-GCST90002061 | ebi-a-GCST90002061 | TRUE | C | 97727916 | 6.73E-24 | reported | 2920 | 0.02734 | rs7921331 |  |  |  |  |  |  |  |  |  |  |  |  |  |  |  |
| 0.177 | 10 | igd | 0.4955 | T | \|\| id:ebi-a-GCST90002069 | ebi-a-GCST90002069 | TRUE | C | 97727916 | 8.47E-11 | reported | 2920 | 0.02717 | rs7921331 |  |  |  |  |  |  |  |  |  |  |  |  |  |  |  |
| 0.2692 | 19 | igd | 0.4949 | C | \|\| id:ebi-a-GCST90001954 | ebi-a-GCST90001954 | TRUE | T | 51550760 | 5.73E-13 | reported | 1555 | 0.03703 | rs7249762 |  |  |  |  |  |  |  |  |  |  |  |  |  |  |  |
| 0.2616 | 19 | igd | 0.4945 | C | \|\| id:ebi-a-GCST90001953 | ebi-a-GCST90001953 | TRUE | T | 51550760 | 2.52E-12 | reported | 1558 | 0.03706 | rs7249762 |  |  |  |  |  |  |  |  |  |  |  |  |  |  |  |
| 0.258 | 19 | igd | 0.4945 | C | \|\| id:ebi-a-GCST90001955 | ebi-a-GCST90001955 | TRUE | T | 51550760 | 5.32E-12 | reported | 1554 | 0.03712 | rs7249762 |  |  |  |  |  |  |  |  |  |  |  |  |  |  |  |
| -0.3413 | 19 | igd | 0.4939 | G | \|\| id:ebi-a-GCST90001953 | ebi-a-GCST90001953 | TRUE | A | 51821634 | 1.60E-20 | reported | 1558 | 0.03624 | rs2411317 |  |  |  |  |  |  |  |  |  |  |  |  |  |  |  |
| -0.195 | 2 | igd | 0.4935 | T | \|\| id:ebi-a-GCST90002101 | ebi-a-GCST90002101 | TRUE | G | 39022120 | 1.17E-12 | reported | 2911 | 0.02731 | rs34818399 |  |  |  |  |  |  |  |  |  |  |  |  |  |  |  |
| -0.1636 | 2 | igd | 0.4933 | T | \|\| id:ebi-a-GCST90001891 | ebi-a-GCST90001891 | TRUE | C | 204955360 | 3.08E-09 | reported | 2920 | 0.02752 | rs13430191 |  |  |  |  |  |  |  |  |  |  |  |  |  |  |  |
| -0.154 | 2 | igd | 0.4933 | T | \|\| id:ebi-a-GCST90001900 | ebi-a-GCST90001900 | TRUE | C | 204955360 | 2.73E-08 | reported | 2919 | 0.02763 | rs13430191 |  |  |  |  |  |  |  |  |  |  |  |  |  |  |  |
| -0.3452 | 19 | igd | 0.4932 | G | \|\| id:ebi-a-GCST90001954 | ebi-a-GCST90001954 | TRUE | A | 51821634 | 6.64E-21 | reported | 1555 | 0.03628 | rs2411317 |  |  |  |  |  |  |  |  |  |  |  |  |  |  |  |
| -0.3329 | 19 | igd | 0.4932 | G | \|\| id:ebi-a-GCST90001955 | ebi-a-GCST90001955 | TRUE | A | 51821634 | 1.79E-19 | reported | 1554 | 0.03639 | rs2411317 |  |  |  |  |  |  |  |  |  |  |  |  |  |  |  |
| 0.2527 | 19 | igd | 0.4927 | C | \|\| id:ebi-a-GCST90001952 | ebi-a-GCST90001952 | TRUE | T | 51550760 | 1.66E-11 | reported | 1569 | 0.03726 | rs7249762 |  |  |  |  |  |  |  |  |  |  |  |  |  |  |  |
| -0.466 | 6 | igd | 0.4921 | C | \|\| id:ebi-a-GCST90002108 | ebi-a-GCST90002108 | TRUE | T | 32623325 | 2.44E-18 | reported | 1579 | 0.0527 | rs9273333 |  |  |  |  |  |  |  |  |  |  |  |  |  |  |  |
| -0.3499 | 6 | igd | 0.4921 | C | \|\| id:ebi-a-GCST90002109 | ebi-a-GCST90002109 | TRUE | T | 32623325 | 4.62E-11 | reported | 1578 | 0.05278 | rs9273333 |  |  |  |  |  |  |  |  |  |  |  |  |  |  |  |
| 0.1602 | 3 | igd | 0.492 | T | \|\| id:ebi-a-GCST90002078 | ebi-a-GCST90002078 | TRUE | C | 50255663 | 1.02E-08 | reported | 3112 | 0.02789 | rs35926495 |  |  |  |  |  |  |  |  |  |  |  |  |  |  |  |
| 0.2669 | 19 | igd | 0.4911 | C | \|\| id:ebi-a-GCST90001947 | ebi-a-GCST90001947 | TRUE | T | 51550760 | 8.34E-13 | reported | 1577 | 0.03699 | rs7249762 |  |  |  |  |  |  |  |  |  |  |  |  |  |  |  |
| -0.3535 | 6 | igd | 0.4911 | A | \|\| id:ebi-a-GCST90002112 | ebi-a-GCST90002112 | TRUE | C | 32560017 | 1.80E-12 | reported | 1635 | 0.04975 | rs73729339 |  |  |  |  |  |  |  |  |  |  |  |  |  |  |  |
| 0.1368 | 2 | igd | 0.491 | T | \|\| id:ebi-a-GCST90001535 | ebi-a-GCST90001535 | TRUE | C | 39052091 | 1.42E-08 | reported | 3427 | 0.02406 | rs28500497 |  |  |  |  |  |  |  |  |  |  |  |  |  |  |  |
| -0.1296 | 2 | igd | 0.491 | T | \|\| id:ebi-a-GCST90001560 | ebi-a-GCST90001560 | TRUE | C | 39052091 | 4.67E-09 | reported | 3395 | 0.02207 | rs28500497 |  |  |  |  |  |  |  |  |  |  |  |  |  |  |  |
| -0.1946 | 2 | igd | 0.491 | T | \|\| id:ebi-a-GCST90001561 | ebi-a-GCST90001561 | TRUE | C | 39052091 | 5.34E-19 | reported | 3427 | 0.02172 | rs28500497 |  |  |  |  |  |  |  |  |  |  |  |  |  |  |  |
| 0.2757 | 19 | igd | 0.4908 | C | \|\| id:ebi-a-GCST90001956 | ebi-a-GCST90001956 | TRUE | T | 51550760 | 1.23E-13 | reported | 1579 | 0.03687 | rs7249762 |  |  |  |  |  |  |  |  |  |  |  |  |  |  |  |
| 0.2785 | 19 | igd | 0.4908 | C | \|\| id:ebi-a-GCST90001957 | ebi-a-GCST90001957 | TRUE | T | 51550760 | 7.26E-14 | reported | 1578 | 0.03688 | rs7249762 |  |  |  |  |  |  |  |  |  |  |  |  |  |  |  |
| -0.1352 | 17 | igd | 0.4903 | C | \|\| id:ebi-a-GCST90001442 | ebi-a-GCST90001442 | TRUE | A | 2715961 | 1.81E-08 | reported | 3656 | 0.02397 | rs11657759 |  |  |  |  |  |  |  |  |  |  |  |  |  |  |  |
| -0.1758 | 2 | igd | 0.4892 | G | \|\| id:ebi-a-GCST90002118 | ebi-a-GCST90002118 | TRUE | A | 87317017 | 2.20E-10 | reported | 2920 | 0.02761 | rs1460507 |  |  |  |  |  |  |  |  |  |  |  |  |  |  |  |
| 0.1434 | 2 | igd | 0.4879 | T | \|\| id:ebi-a-GCST90001535 | ebi-a-GCST90001535 | TRUE | G | 38948860 | 2.27E-09 | reported | 3427 | 0.02393 | rs12712613 |  |  |  |  |  |  |  |  |  |  |  |  |  |  |  |
| -0.1633 | 17 | igd | 0.4878 | T | \|\| id:ebi-a-GCST90001821 | ebi-a-GCST90001821 | TRUE | G | 62021003 | 6.58E-11 | reported | 3656 | 0.02494 | rs35501848 |  |  |  |  |  |  |  |  |  |  |  |  |  |  |  |
| -0.1922 | 2 | igd | 0.4875 | G | \|\| id:ebi-a-GCST90001891 | ebi-a-GCST90001891 | TRUE | A | 203042344 | 1.92E-12 | reported | 2920 | 0.02718 | rs4513273 |  |  |  |  |  |  |  |  |  |  |  |  |  |  |  |
| -0.205 | 10 | igd | 0.487 | C | \|\| id:ebi-a-GCST90002032 | ebi-a-GCST90002032 | TRUE | T | 96851353 | 4.11E-13 | reported | 2920 | 0.02814 | rs10128455 |  |  |  |  |  |  |  |  |  |  |  |  |  |  |  |
| -0.1532 | 10 | igd | 0.4868 | C | \|\| id:ebi-a-GCST90002029 | ebi-a-GCST90002029 | TRUE | T | 96851353 | 2.34E-08 | reported | 2915 | 0.02736 | rs10128455 |  |  |  |  |  |  |  |  |  |  |  |  |  |  |  |
| 0.179 | 6 | igd | 0.4865 | G | \|\| id:ebi-a-GCST90002061 | ebi-a-GCST90002061 | TRUE | A | 31050348 | 4.60E-09 | reported | 2920 | 0.03045 | rs9263475 |  |  |  |  |  |  |  |  |  |  |  |  |  |  |  |
| 0.1696 | 6 | igd | 0.4865 | G | \|\| id:ebi-a-GCST90002062 | ebi-a-GCST90002062 | TRUE | A | 31050348 | 1.74E-08 | reported | 2920 | 0.03 | rs9263475 |  |  |  |  |  |  |  |  |  |  |  |  |  |  |  |
| 0.2076 | 6 | igd | 0.4865 | G | \|\| id:ebi-a-GCST90002069 | ebi-a-GCST90002069 | TRUE | A | 31050348 | 1.11E-12 | reported | 2920 | 0.02905 | rs9263475 |  |  |  |  |  |  |  |  |  |  |  |  |  |  |  |
| -0.2291 | 1 | igd | 0.4864 | A | \|\| id:ebi-a-GCST90001880 | ebi-a-GCST90001880 | TRUE | C | 2723214 | 6.23E-09 | reported | 1247 | 0.03915 | rs4648450 |  |  |  |  |  |  |  |  |  |  |  |  |  |  |  |
| -0.9175 | 10 | igd | 0.4863 | A | \|\| id:ebi-a-GCST90001658 | ebi-a-GCST90001658 | TRUE | T | 97472570 | 1.00E-200 | reported | 3440 | 0.01908 | rs4918960 |  |  |  |  |  |  |  |  |  |  |  |  |  |  |  |
| -0.9258 | 10 | igd | 0.4863 | A | \|\| id:ebi-a-GCST90001659 | ebi-a-GCST90001659 | TRUE | T | 97472570 | 1.00E-200 | reported | 3440 | 0.01897 | rs4918960 |  |  |  |  |  |  |  |  |  |  |  |  |  |  |  |
| -0.915 | 10 | igd | 0.4863 | A | \|\| id:ebi-a-GCST90001670 | ebi-a-GCST90001670 | TRUE | T | 97472570 | 1.00E-200 | reported | 3440 | 0.02037 | rs4918960 |  |  |  |  |  |  |  |  |  |  |  |  |  |  |  |
| -0.9043 | 10 | igd | 0.4863 | A | \|\| id:ebi-a-GCST90001671 | ebi-a-GCST90001671 | TRUE | T | 97472570 | 1.00E-200 | reported | 3440 | 0.02058 | rs4918960 |  |  |  |  |  |  |  |  |  |  |  |  |  |  |  |
| -0.9412 | 10 | igd | 0.4862 | A | \|\| id:ebi-a-GCST90001496 | ebi-a-GCST90001496 | TRUE | T | 97472570 | 1.00E-200 | reported | 3437 | 0.02027 | rs4918960 |  |  |  |  |  |  |  |  |  |  |  |  |  |  |  |
| -0.9321 | 10 | igd | 0.4862 | A | \|\| id:ebi-a-GCST90001497 | ebi-a-GCST90001497 | TRUE | T | 97472570 | 1.00E-200 | reported | 3437 | 0.01998 | rs4918960 |  |  |  |  |  |  |  |  |  |  |  |  |  |  |  |
| -0.9198 | 10 | igd | 0.4862 | A | \|\| id:ebi-a-GCST90001660 | ebi-a-GCST90001660 | TRUE | T | 97472570 | 1.00E-200 | reported | 3408 | 0.02052 | rs4918960 |  |  |  |  |  |  |  |  |  |  |  |  |  |  |  |
| -0.9143 | 10 | igd | 0.4862 | A | \|\| id:ebi-a-GCST90001672 | ebi-a-GCST90001672 | TRUE | T | 97472570 | 1.00E-200 | reported | 3408 | 0.02091 | rs4918960 |  |  |  |  |  |  |  |  |  |  |  |  |  |  |  |
| -0.1959 | 6 | igd | 0.4856 | T | \|\| id:ebi-a-GCST90002105 | ebi-a-GCST90002105 | TRUE | G | 33091613 | 1.62E-11 | reported | 2872 | 0.02897 | rs9277726 |  |  |  |  |  |  |  |  |  |  |  |  |  |  |  |
| 0.1812 | 1 | igd | 0.4854 | A | \|\| id:ebi-a-GCST90001854 | ebi-a-GCST90001854 | TRUE | G | 167170838 | 2.54E-11 | reported | 2919 | 0.02706 | rs10800291 |  |  |  |  |  |  |  |  |  |  |  |  |  |  |  |
| 0.1576 | 1 | igd | 0.4854 | A | \|\| id:ebi-a-GCST90001858 | ebi-a-GCST90001858 | TRUE | G | 167170838 | 9.38E-09 | reported | 2919 | 0.02737 | rs10800291 |  |  |  |  |  |  |  |  |  |  |  |  |  |  |  |
| 0.1743 | 1 | igd | 0.4854 | A | \|\| id:ebi-a-GCST90001860 | ebi-a-GCST90001860 | TRUE | G | 167170838 | 2.35E-10 | reported | 2919 | 0.02742 | rs10800291 |  |  |  |  |  |  |  |  |  |  |  |  |  |  |  |
| 0.1854 | 1 | igd | 0.4854 | A | \|\| id:ebi-a-GCST90001864 | ebi-a-GCST90001864 | TRUE | G | 167170838 | 1.26E-11 | reported | 2919 | 0.02726 | rs10800291 |  |  |  |  |  |  |  |  |  |  |  |  |  |  |  |
| 0.2797 | 19 | igd | 0.4853 | C | \|\| id:ebi-a-GCST90001948 | ebi-a-GCST90001948 | TRUE | T | 51550760 | 1.02E-14 | reported | 1633 | 0.03581 | rs7249762 |  |  |  |  |  |  |  |  |  |  |  |  |  |  |  |
| -0.1653 | 8 | igd | 0.4853 | A | \|\| id:ebi-a-GCST90002073 | ebi-a-GCST90002073 | TRUE | G | 145003777 | 2.20E-09 | reported | 2850 | 0.02755 | rs11136335 |  |  |  |  |  |  |  |  |  |  |  |  |  |  |  |
| 0.2906 | 19 | igd | 0.485 | C | \|\| id:ebi-a-GCST90001946 | ebi-a-GCST90001946 | TRUE | T | 51550760 | 1.12E-15 | reported | 1634 | 0.0359 | rs7249762 |  |  |  |  |  |  |  |  |  |  |  |  |  |  |  |
| 0.2792 | 19 | igd | 0.485 | C | \|\| id:ebi-a-GCST90001949 | ebi-a-GCST90001949 | TRUE | T | 51550760 | 9.26E-15 | reported | 1634 | 0.0357 | rs7249762 |  |  |  |  |  |  |  |  |  |  |  |  |  |  |  |
| 0.2396 | 1 | igd | 0.4849 | A | \|\| id:ebi-a-GCST90001865 | ebi-a-GCST90001865 | TRUE | G | 167419839 | 1.75E-18 | reported | 2919 | 0.02713 | rs2988279 |  |  |  |  |  |  |  |  |  |  |  |  |  |  |  |
| -0.3823 | 2 | igd | 0.4847 | T | \|\| id:ebi-a-GCST90002098 | ebi-a-GCST90002098 | TRUE | G | 38948860 | 6.73E-50 | reported | 2910 | 0.02525 | rs12712613 |  |  |  |  |  |  |  |  |  |  |  |  |  |  |  |
| -0.1917 | 2 | igd | 0.4847 | T | \|\| id:ebi-a-GCST90002100 | ebi-a-GCST90002100 | TRUE | G | 38948860 | 9.13E-13 | reported | 2910 | 0.02672 | rs12712613 |  |  |  |  |  |  |  |  |  |  |  |  |  |  |  |
| -0.2436 | 2 | igd | 0.4846 | T | \|\| id:ebi-a-GCST90002118 | ebi-a-GCST90002118 | TRUE | G | 87078558 | 1.05E-18 | reported | 2920 | 0.0274 | rs7565893 |  |  |  |  |  |  |  |  |  |  |  |  |  |  |  |
| -0.2387 | 6 | igd | 0.4842 | A | \|\| id:ebi-a-GCST90002108 | ebi-a-GCST90002108 | TRUE | G | 31347798 | 3.60E-09 | reported | 1579 | 0.04022 | rs4713460 |  |  |  |  |  |  |  |  |  |  |  |  |  |  |  |
| -0.1587 | 2 | igd | 0.4839 | A | \|\| id:ebi-a-GCST90001887 | ebi-a-GCST90001887 | TRUE | G | 203084111 | 5.06E-09 | reported | 2920 | 0.02707 | rs3769816 |  |  |  |  |  |  |  |  |  |  |  |  |  |  |  |
| -1.061 | 10 | igd | 0.4839 | A | \|\| id:ebi-a-GCST90002030 | ebi-a-GCST90002030 | TRUE | T | 97472570 | 1.00E-200 | reported | 2920 | 0.01887 | rs4918960 |  |  |  |  |  |  |  |  |  |  |  |  |  |  |  |
| -0.2347 | 10 | igd | 0.4836 | C | \|\| id:ebi-a-GCST90001672 | ebi-a-GCST90001672 | TRUE | T | 95996336 | 8.82E-19 | reported | 3408 | 0.02637 | rs10882412 |  |  |  |  |  |  |  |  |  |  |  |  |  |  |  |
| -0.1486 | 2 | igd | 0.4836 | T | \|\| id:ebi-a-GCST90001886 | ebi-a-GCST90001886 | TRUE | C | 203080294 | 1.54E-08 | reported | 2920 | 0.02619 | rs62196472 |  |  |  |  |  |  |  |  |  |  |  |  |  |  |  |
| 0.1656 | 7 | igd | 0.4826 | A | \|\| id:ebi-a-GCST90001966 | ebi-a-GCST90001966 | TRUE | G | 92647779 | 5.47E-09 | reported | 2850 | 0.0283 | rs10236337 |  |  |  |  |  |  |  |  |  |  |  |  |  |  |  |
| -0.1705 | 2 | igd | 0.482 | G | \|\| id:ebi-a-GCST90001889 | ebi-a-GCST90001889 | TRUE | A | 203033509 | 1.40E-10 | reported | 2920 | 0.02648 | rs9917151 |  |  |  |  |  |  |  |  |  |  |  |  |  |  |  |
| -0.1734 | 2 | igd | 0.482 | G | \|\| id:ebi-a-GCST90001893 | ebi-a-GCST90001893 | TRUE | A | 203033509 | 2.98E-10 | reported | 2920 | 0.02743 | rs9917151 |  |  |  |  |  |  |  |  |  |  |  |  |  |  |  |
| -0.1879 | 2 | igd | 0.482 | G | \|\| id:ebi-a-GCST90001894 | ebi-a-GCST90001894 | TRUE | A | 203033509 | 7.07E-12 | reported | 2920 | 0.02729 | rs9917151 |  |  |  |  |  |  |  |  |  |  |  |  |  |  |  |
| -0.1894 | 2 | igd | 0.482 | G | \|\| id:ebi-a-GCST90001898 | ebi-a-GCST90001898 | TRUE | A | 203033509 | 4.80E-12 | reported | 2920 | 0.02728 | rs9917151 |  |  |  |  |  |  |  |  |  |  |  |  |  |  |  |
| -0.1584 | 2 | igd | 0.482 | G | \|\| id:ebi-a-GCST90001899 | ebi-a-GCST90001899 | TRUE | A | 203033509 | 9.63E-10 | reported | 2920 | 0.02582 | rs9917151 |  |  |  |  |  |  |  |  |  |  |  |  |  |  |  |
| -0.1648 | 2 | igd | 0.482 | G | \|\| id:ebi-a-GCST90001902 | ebi-a-GCST90001902 | TRUE | A | 203033509 | 3.02E-10 | reported | 2920 | 0.02608 | rs9917151 |  |  |  |  |  |  |  |  |  |  |  |  |  |  |  |
| -0.2083 | 19 | igd | 0.481 | A | \|\| id:ebi-a-GCST90001946 | ebi-a-GCST90001946 | TRUE | G | 52052004 | 7.45E-09 | reported | 1634 | 0.03584 | rs11668533 |  |  |  |  |  |  |  |  |  |  |  |  |  |  |  |
| 0.2331 | 1 | igd | 0.4795 | A | \|\| id:ebi-a-GCST90001526 | ebi-a-GCST90001526 | TRUE | G | 161408556 | 1.15E-11 | reported | 1858 | 0.03413 | rs12125079 |  |  |  |  |  |  |  |  |  |  |  |  |  |  |  |
| -0.2322 | 1 | igd | 0.4795 | A | \|\| id:ebi-a-GCST90001528 | ebi-a-GCST90001528 | TRUE | G | 161408556 | 1.42E-11 | reported | 1858 | 0.03415 | rs12125079 |  |  |  |  |  |  |  |  |  |  |  |  |  |  |  |
| 0.1746 | 6 | igd | 0.4793 | T | \|\| id:ebi-a-GCST90002061 | ebi-a-GCST90002061 | TRUE | A | 31247203 | 1.17E-08 | reported | 2920 | 0.03052 | rs2853946 |  |  |  |  |  |  |  |  |  |  |  |  |  |  |  |
| 0.1668 | 6 | igd | 0.4793 | T | \|\| id:ebi-a-GCST90002069 | ebi-a-GCST90002069 | TRUE | A | 31247203 | 1.22E-08 | reported | 2920 | 0.0292 | rs2853946 |  |  |  |  |  |  |  |  |  |  |  |  |  |  |  |
| -0.2289 | 10 | igd | 0.4791 | G | \|\| id:ebi-a-GCST90002032 | ebi-a-GCST90002032 | TRUE | A | 95984276 | 3.13E-16 | reported | 2920 | 0.02786 | rs11187823 |  |  |  |  |  |  |  |  |  |  |  |  |  |  |  |
| 0.1968 | 12 | igd | 0.4788 | G | \|\| id:ebi-a-GCST90002073 | ebi-a-GCST90002073 | TRUE | A | 70369918 | 1.86E-12 | reported | 2850 | 0.02782 | rs710698 |  |  |  |  |  |  |  |  |  |  |  |  |  |  |  |
| 0.1599 | 12 | igd | 0.4786 | G | \|\| id:ebi-a-GCST90002074 | ebi-a-GCST90002074 | TRUE | A | 70369918 | 2.49E-09 | reported | 3112 | 0.02673 | rs710698 |  |  |  |  |  |  |  |  |  |  |  |  |  |  |  |
| -0.1626 | 16 | igd | 0.4771 | A | \|\| id:ebi-a-GCST90001930 | ebi-a-GCST90001930 | TRUE | T | 88618795 | 7.52E-09 | reported | 2920 | 0.02805 | rs8062396 |  |  |  |  |  |  |  |  |  |  |  |  |  |  |  |
| 0.1538 | 10 | igd | 0.4762 | T | \|\| id:ebi-a-GCST90001670 | ebi-a-GCST90001670 | TRUE | C | 96616103 | 6.47E-09 | reported | 3440 | 0.02643 | rs4918690 |  |  |  |  |  |  |  |  |  |  |  |  |  |  |  |
| 0.1703 | 12 | igd | 0.4762 | G | \|\| id:ebi-a-GCST90002074 | ebi-a-GCST90002074 | TRUE | T | 69610415 | 1.42E-10 | reported | 3112 | 0.02646 | rs12228869 |  |  |  |  |  |  |  |  |  |  |  |  |  |  |  |
| 0.1925 | 6 | igd | 0.4762 | T | \|\| id:ebi-a-GCST90002121 | ebi-a-GCST90002121 | TRUE | C | 30285121 | 8.08E-12 | reported | 2915 | 0.02804 | rs9357097 |  |  |  |  |  |  |  |  |  |  |  |  |  |  |  |
| 0.1875 | 6 | igd | 0.476 | T | \|\| id:ebi-a-GCST90002118 | ebi-a-GCST90002118 | TRUE | C | 30285121 | 3.39E-10 | reported | 2920 | 0.02975 | rs9357097 |  |  |  |  |  |  |  |  |  |  |  |  |  |  |  |
| 0.1725 | 6 | igd | 0.476 | T | \|\| id:ebi-a-GCST90002119 | ebi-a-GCST90002119 | TRUE | C | 30285121 | 5.74E-09 | reported | 2920 | 0.02953 | rs9357097 |  |  |  |  |  |  |  |  |  |  |  |  |  |  |  |
| 0.3343 | 2 | igd | 0.4751 | C | \|\| id:ebi-a-GCST90002098 | ebi-a-GCST90002098 | TRUE | T | 38897810 | 7.31E-38 | reported | 2910 | 0.02562 | rs6751481 |  |  |  |  |  |  |  |  |  |  |  |  |  |  |  |
| -0.1428 | 17 | igd | 0.475 | C | \|\| id:ebi-a-GCST90001439 | ebi-a-GCST90001439 | TRUE | T | 2714118 | 3.70E-09 | reported | 3656 | 0.02416 | rs11657118 |  |  |  |  |  |  |  |  |  |  |  |  |  |  |  |
| -0.2159 | 6 | igd | 0.4746 | G | \|\| id:ebi-a-GCST90002110 | ebi-a-GCST90002110 | TRUE | A | 31095007 | 4.67E-08 | reported | 1634 | 0.03933 | rs6931464 |  |  |  |  |  |  |  |  |  |  |  |  |  |  |  |
| -0.2062 | 22 | igd | 0.4743 | G | \|\| id:ebi-a-GCST90001702 | ebi-a-GCST90001702 | TRUE | A | 40376383 | 3.59E-16 | reported | 3657 | 0.02518 | rs114607 |  |  |  |  |  |  |  |  |  |  |  |  |  |  |  |
| -0.2004 | 22 | igd | 0.4743 | G | \|\| id:ebi-a-GCST90001703 | ebi-a-GCST90001703 | TRUE | A | 40376383 | 2.31E-15 | reported | 3657 | 0.02519 | rs114607 |  |  |  |  |  |  |  |  |  |  |  |  |  |  |  |
| -0.1835 | 22 | igd | 0.4743 | G | \|\| id:ebi-a-GCST90001704 | ebi-a-GCST90001704 | TRUE | A | 40376383 | 3.88E-13 | reported | 3657 | 0.02519 | rs114607 |  |  |  |  |  |  |  |  |  |  |  |  |  |  |  |
| -0.195 | 22 | igd | 0.4743 | G | \|\| id:ebi-a-GCST90001710 | ebi-a-GCST90001710 | TRUE | A | 40376383 | 1.10E-14 | reported | 3657 | 0.02513 | rs114607 |  |  |  |  |  |  |  |  |  |  |  |  |  |  |  |
| -0.1986 | 22 | igd | 0.4743 | G | \|\| id:ebi-a-GCST90001711 | ebi-a-GCST90001711 | TRUE | A | 40376383 | 2.96E-15 | reported | 3657 | 0.02506 | rs114607 |  |  |  |  |  |  |  |  |  |  |  |  |  |  |  |
| 0.2384 | 19 | igd | 0.4743 | C | \|\| id:ebi-a-GCST90001950 | ebi-a-GCST90001950 | TRUE | T | 51585259 | 6.34E-09 | reported | 1244 | 0.04076 | rs2691244 |  |  |  |  |  |  |  |  |  |  |  |  |  |  |  |
| 0.2078 | 1 | igd | 0.4741 | C | \|\| id:ebi-a-GCST90001994 | ebi-a-GCST90001994 | TRUE | T | 161590693 | 1.03E-16 | reported | 3590 | 0.02491 | rs34416533 |  |  |  |  |  |  |  |  |  |  |  |  |  |  |  |
| 0.2336 | 12 | igd | 0.4739 | G | \|\| id:ebi-a-GCST90002073 | ebi-a-GCST90002073 | TRUE | T | 69610415 | 2.33E-17 | reported | 2850 | 0.02738 | rs12228869 |  |  |  |  |  |  |  |  |  |  |  |  |  |  |  |
| -0.2906 | 6 | igd | 0.4737 | A | \|\| id:ebi-a-GCST90002110 | ebi-a-GCST90002110 | TRUE | G | 31681160 | 2.42E-13 | reported | 1634 | 0.03935 | rs385306 |  |  |  |  |  |  |  |  |  |  |  |  |  |  |  |
| 0.2329 | 10 | igd | 0.4736 | C | \|\| id:ebi-a-GCST90001897 | ebi-a-GCST90001897 | TRUE | A | 97495005 | 3.33E-18 | reported | 2914 | 0.02659 | rs11188460 |  |  |  |  |  |  |  |  |  |  |  |  |  |  |  |
| -0.2655 | 6 | igd | 0.4734 | A | \|\| id:ebi-a-GCST90002111 | ebi-a-GCST90002111 | TRUE | G | 31681160 | 1.06E-11 | reported | 1635 | 0.03877 | rs385306 |  |  |  |  |  |  |  |  |  |  |  |  |  |  |  |
| 0.4156 | 10 | igd | 0.4733 | C | \|\| id:ebi-a-GCST90001888 | ebi-a-GCST90001888 | TRUE | A | 97601703 | 9.56E-57 | reported | 2920 | 0.02562 | rs10748649 |  |  |  |  |  |  |  |  |  |  |  |  |  |  |  |
| 0.4878 | 10 | igd | 0.4733 | C | \|\| id:ebi-a-GCST90002061 | ebi-a-GCST90002061 | TRUE | A | 97624709 | 2.99E-73 | reported | 2920 | 0.02619 | rs2275759 |  |  |  |  |  |  |  |  |  |  |  |  |  |  |  |
| 0.2341 | 10 | igd | 0.4732 | G | \|\| id:ebi-a-GCST90001897 | ebi-a-GCST90001897 | TRUE | C | 97608114 | 2.09E-18 | reported | 2914 | 0.02657 | rs7088961 |  |  |  |  |  |  |  |  |  |  |  |  |  |  |  |
| 0.502 | 10 | igd | 0.4731 | G | \|\| id:ebi-a-GCST90002029 | ebi-a-GCST90002029 | TRUE | C | 97608114 | 4.04E-82 | reported | 2915 | 0.02533 | rs7088961 |  |  |  |  |  |  |  |  |  |  |  |  |  |  |  |
| 1.177 | 10 | igd | 0.4731 | C | \|\| id:ebi-a-GCST90002030 | ebi-a-GCST90002030 | TRUE | T | 97572962 | 1.00E-200 | reported | 2920 | 0.01658 | rs11188497 |  |  |  |  |  |  |  |  |  |  |  |  |  |  |  |
| 0.1851 | 6 | igd | 0.4731 | C | \|\| id:ebi-a-GCST90002069 | ebi-a-GCST90002069 | TRUE | T | 31313054 | 3.60E-10 | reported | 2920 | 0.02941 | rs9265880 |  |  |  |  |  |  |  |  |  |  |  |  |  |  |  |
| 0.2931 | 10 | igd | 0.4731 | T | \|\| id:ebi-a-GCST90002069 | ebi-a-GCST90002069 | TRUE | A | 97586867 | 1.41E-27 | reported | 2920 | 0.02666 | rs10748648 |  |  |  |  |  |  |  |  |  |  |  |  |  |  |  |
| 0.2213 | 6 | igd | 0.4731 | T | \|\| id:ebi-a-GCST90002119 | ebi-a-GCST90002119 | TRUE | G | 31070884 | 1.20E-13 | reported | 2920 | 0.0297 | rs2517542 |  |  |  |  |  |  |  |  |  |  |  |  |  |  |  |
| -0.7175 | 10 | igd | 0.4729 | T | \|\| id:ebi-a-GCST90001961 | ebi-a-GCST90001961 | TRUE | C | 97574689 | 7.96E-170 | reported | 2920 | 0.02414 | rs4075310 |  |  |  |  |  |  |  |  |  |  |  |  |  |  |  |
| 1.009 | 10 | igd | 0.4729 | T | \|\| id:ebi-a-GCST90002031 | ebi-a-GCST90002031 | TRUE | C | 97574689 | 1.00E-200 | reported | 2920 | 0.02072 | rs4075310 |  |  |  |  |  |  |  |  |  |  |  |  |  |  |  |
| 0.9846 | 10 | igd | 0.4729 | T | \|\| id:ebi-a-GCST90002032 | ebi-a-GCST90002032 | TRUE | C | 97574689 | 1.00E-200 | reported | 2920 | 0.02111 | rs4075310 |  |  |  |  |  |  |  |  |  |  |  |  |  |  |  |
| -0.2955 | 2 | igd | 0.4729 | T | \|\| id:ebi-a-GCST90002119 | ebi-a-GCST90002119 | TRUE | C | 87279109 | 3.00E-28 | reported | 2920 | 0.02653 | rs10205992 |  |  |  |  |  |  |  |  |  |  |  |  |  |  |  |
| 0.4907 | 10 | igd | 0.4728 | G | \|\| id:ebi-a-GCST90002061 | ebi-a-GCST90002061 | TRUE | A | 97507312 | 5.68E-74 | reported | 2920 | 0.0262 | rs7086986 |  |  |  |  |  |  |  |  |  |  |  |  |  |  |  |
| 0.2278 | 3 | igd | 0.4728 | A | \|\| id:ebi-a-GCST90002110 | ebi-a-GCST90002110 | TRUE | T | 188135783 | 2.49E-10 | reported | 1634 | 0.03577 | rs12485444 |  |  |  |  |  |  |  |  |  |  |  |  |  |  |  |
| 0.4153 | 10 | igd | 0.4726 | C | \|\| id:ebi-a-GCST90001888 | ebi-a-GCST90001888 | TRUE | A | 97495005 | 1.72E-56 | reported | 2920 | 0.02566 | rs11188460 |  |  |  |  |  |  |  |  |  |  |  |  |  |  |  |
| 0.1634 | 2 | igd | 0.4723 | G | \|\| id:ebi-a-GCST90002098 | ebi-a-GCST90002098 | TRUE | A | 38670529 | 7.15E-10 | reported | 2910 | 0.02642 | rs10205121 |  |  |  |  |  |  |  |  |  |  |  |  |  |  |  |
| -0.2836 | 6 | igd | 0.4721 | A | \|\| id:ebi-a-GCST90002108 | ebi-a-GCST90002108 | TRUE | G | 32113312 | 6.35E-13 | reported | 1579 | 0.0391 | rs205002 |  |  |  |  |  |  |  |  |  |  |  |  |  |  |  |
| -0.22 | 6 | igd | 0.4721 | A | \|\| id:ebi-a-GCST90002109 | ebi-a-GCST90002109 | TRUE | G | 32113312 | 2.09E-08 | reported | 1578 | 0.03906 | rs205002 |  |  |  |  |  |  |  |  |  |  |  |  |  |  |  |
| 0.2896 | 19 | igd | 0.472 | C | \|\| id:ebi-a-GCST90001955 | ebi-a-GCST90001955 | TRUE | T | 51585259 | 6.04E-15 | reported | 1554 | 0.03674 | rs2691244 |  |  |  |  |  |  |  |  |  |  |  |  |  |  |  |
| 0.2486 | 10 | igd | 0.4719 | T | \|\| id:ebi-a-GCST90001886 | ebi-a-GCST90001886 | TRUE | C | 97519645 | 1.29E-21 | reported | 2920 | 0.02582 | rs1539433 |  |  |  |  |  |  |  |  |  |  |  |  |  |  |  |
| 0.5445 | 10 | igd | 0.4719 | T | \|\| id:ebi-a-GCST90001892 | ebi-a-GCST90001892 | TRUE | C | 97519645 | 5.03E-95 | reported | 2920 | 0.02537 | rs1539433 |  |  |  |  |  |  |  |  |  |  |  |  |  |  |  |
| 0.2209 | 6 | igd | 0.4719 | A | \|\| id:ebi-a-GCST90002067 | ebi-a-GCST90002067 | TRUE | G | 31249127 | 1.42E-13 | reported | 2920 | 0.02974 | rs2253487 |  |  |  |  |  |  |  |  |  |  |  |  |  |  |  |
| 0.316 | 19 | igd | 0.4717 | C | \|\| id:ebi-a-GCST90001954 | ebi-a-GCST90001954 | TRUE | T | 51585259 | 1.25E-17 | reported | 1555 | 0.03653 | rs2691244 |  |  |  |  |  |  |  |  |  |  |  |  |  |  |  |
| 0.1596 | 1 | igd | 0.4717 | T | \|\| id:ebi-a-GCST90001992 | ebi-a-GCST90001992 | TRUE | C | 161589626 | 1.24E-10 | reported | 3618 | 0.02473 | rs61802329 |  |  |  |  |  |  |  |  |  |  |  |  |  |  |  |
| 0.2494 | 10 | igd | 0.4714 | G | \|\| id:ebi-a-GCST90001886 | ebi-a-GCST90001886 | TRUE | A | 97628994 | 9.47E-22 | reported | 2920 | 0.02582 | rs2226163 |  |  |  |  |  |  |  |  |  |  |  |  |  |  |  |
| 0.5439 | 10 | igd | 0.4714 | G | \|\| id:ebi-a-GCST90001892 | ebi-a-GCST90001892 | TRUE | A | 97628994 | 9.14E-95 | reported | 2920 | 0.02538 | rs2226163 |  |  |  |  |  |  |  |  |  |  |  |  |  |  |  |
| 0.306 | 19 | igd | 0.4714 | C | \|\| id:ebi-a-GCST90001953 | ebi-a-GCST90001953 | TRUE | T | 51585259 | 1.33E-16 | reported | 1558 | 0.03658 | rs2691244 |  |  |  |  |  |  |  |  |  |  |  |  |  |  |  |
| -1.689 | 2 | igd | 0.4712 | G | \|\| id:ebi-a-GCST90001696 | ebi-a-GCST90001696 | TRUE | A | 38670529 | 4.96E-08 | reported | 3425 | 0.3091 | rs10205121 |  |  |  |  |  |  |  |  |  |  |  |  |  |  |  |
| -0.1556 | 4 | igd | 0.4709 | T | \|\| id:ebi-a-GCST90002037 | ebi-a-GCST90002037 | TRUE | C | 84027588 | 2.11E-08 | reported | 2871 | 0.0277 | rs9684014 |  |  |  |  |  |  |  |  |  |  |  |  |  |  |  |
| -0.1544 | 4 | igd | 0.4709 | T | \|\| id:ebi-a-GCST90002038 | ebi-a-GCST90002038 | TRUE | C | 84027588 | 2.74E-08 | reported | 2871 | 0.0277 | rs9684014 |  |  |  |  |  |  |  |  |  |  |  |  |  |  |  |
| 0.1547 | 2 | igd | 0.4709 | A | \|\| id:ebi-a-GCST90002118 | ebi-a-GCST90002118 | TRUE | G | 87476941 | 1.92E-08 | reported | 2920 | 0.02745 | rs76222269 |  |  |  |  |  |  |  |  |  |  |  |  |  |  |  |
| -0.1804 | 4 | igd | 0.4708 | T | \|\| id:ebi-a-GCST90001944 | ebi-a-GCST90001944 | TRUE | C | 84027588 | 9.29E-11 | reported | 2872 | 0.02775 | rs9684014 |  |  |  |  |  |  |  |  |  |  |  |  |  |  |  |
| -0.1786 | 4 | igd | 0.4708 | T | \|\| id:ebi-a-GCST90001945 | ebi-a-GCST90001945 | TRUE | C | 84027588 | 1.43E-10 | reported | 2872 | 0.02775 | rs9684014 |  |  |  |  |  |  |  |  |  |  |  |  |  |  |  |
| -0.1515 | 1 | igd | 0.4699 | G | \|\| id:ebi-a-GCST90001864 | ebi-a-GCST90001864 | TRUE | A | 167140959 | 2.89E-08 | reported | 2919 | 0.02724 | rs6691080 |  |  |  |  |  |  |  |  |  |  |  |  |  |  |  |
| 0.2918 | 19 | igd | 0.4697 | C | \|\| id:ebi-a-GCST90001952 | ebi-a-GCST90001952 | TRUE | T | 51585259 | 3.47E-15 | reported | 1569 | 0.0367 | rs2691244 |  |  |  |  |  |  |  |  |  |  |  |  |  |  |  |
| 0.1609 | 2 | igd | 0.4696 | C | \|\| id:ebi-a-GCST90002098 | ebi-a-GCST90002098 | TRUE | G | 40837037 | 1.06E-09 | reported | 2910 | 0.02629 | rs42803 |  |  |  |  |  |  |  |  |  |  |  |  |  |  |  |
| 0.1829 | 10 | igd | 0.4695 | T | \|\| id:ebi-a-GCST90002061 | ebi-a-GCST90002061 | TRUE | A | 97062933 | 3.37E-11 | reported | 2920 | 0.02748 | rs2026682 |  |  |  |  |  |  |  |  |  |  |  |  |  |  |  |
| 1.026 | 10 | igd | 0.4692 | C | \|\| id:ebi-a-GCST90001496 | ebi-a-GCST90001496 | TRUE | T | 97572962 | 1.00E-200 | reported | 3437 | 0.01917 | rs11188497 |  |  |  |  |  |  |  |  |  |  |  |  |  |  |  |
| 1.015 | 10 | igd | 0.4692 | C | \|\| id:ebi-a-GCST90001497 | ebi-a-GCST90001497 | TRUE | T | 97572962 | 1.00E-200 | reported | 3437 | 0.0189 | rs11188497 |  |  |  |  |  |  |  |  |  |  |  |  |  |  |  |
| 0.9121 | 10 | igd | 0.469 | T | \|\| id:ebi-a-GCST90001491 | ebi-a-GCST90001491 | TRUE | C | 97574689 | 1.00E-200 | reported | 3437 | 0.02034 | rs4075310 |  |  |  |  |  |  |  |  |  |  |  |  |  |  |  |
| 1.013 | 10 | igd | 0.469 | C | \|\| id:ebi-a-GCST90001658 | ebi-a-GCST90001658 | TRUE | T | 97572962 | 1.00E-200 | reported | 3440 | 0.01775 | rs11188497 |  |  |  |  |  |  |  |  |  |  |  |  |  |  |  |
| 1.022 | 10 | igd | 0.469 | C | \|\| id:ebi-a-GCST90001659 | ebi-a-GCST90001659 | TRUE | T | 97572962 | 1.00E-200 | reported | 3440 | 0.0176 | rs11188497 |  |  |  |  |  |  |  |  |  |  |  |  |  |  |  |
| 1.01 | 10 | igd | 0.469 | C | \|\| id:ebi-a-GCST90001670 | ebi-a-GCST90001670 | TRUE | T | 97572962 | 1.00E-200 | reported | 3440 | 0.01915 | rs11188497 |  |  |  |  |  |  |  |  |  |  |  |  |  |  |  |
| 1.001 | 10 | igd | 0.469 | C | \|\| id:ebi-a-GCST90001671 | ebi-a-GCST90001671 | TRUE | T | 97572962 | 1.00E-200 | reported | 3440 | 0.01937 | rs11188497 |  |  |  |  |  |  |  |  |  |  |  |  |  |  |  |
| 0.9616 | 10 | igd | 0.4689 | T | \|\| id:ebi-a-GCST90001495 | ebi-a-GCST90001495 | TRUE | C | 97574689 | 1.00E-200 | reported | 3405 | 0.02041 | rs4075310 |  |  |  |  |  |  |  |  |  |  |  |  |  |  |  |
| 1.015 | 10 | igd | 0.4689 | C | \|\| id:ebi-a-GCST90001660 | ebi-a-GCST90001660 | TRUE | T | 97572962 | 1.00E-200 | reported | 3408 | 0.01928 | rs11188497 |  |  |  |  |  |  |  |  |  |  |  |  |  |  |  |
| 1.01 | 10 | igd | 0.4689 | C | \|\| id:ebi-a-GCST90001672 | ebi-a-GCST90001672 | TRUE | T | 97572962 | 1.00E-200 | reported | 3408 | 0.01969 | rs11188497 |  |  |  |  |  |  |  |  |  |  |  |  |  |  |  |
| -0.2246 | 6 | igd | 0.4688 | C | \|\| id:ebi-a-GCST90002111 | ebi-a-GCST90002111 | TRUE | G | 31395062 | 1.84E-08 | reported | 1635 | 0.03972 | rs114559146 |  |  |  |  |  |  |  |  |  |  |  |  |  |  |  |
| 0.8768 | 10 | igd | 0.4687 | T | \|\| id:ebi-a-GCST90001489 | ebi-a-GCST90001489 | TRUE | G | 97607644 | 1.00E-200 | reported | 3405 | 0.02116 | rs7088584 |  |  |  |  |  |  |  |  |  |  |  |  |  |  |  |
| -0.1403 | 13 | igd | 0.4685 | C | \|\| id:ebi-a-GCST90001412 | ebi-a-GCST90001412 | TRUE | T | 109161589 | 1.08E-08 | reported | 3656 | 0.02447 | rs6492117 |  |  |  |  |  |  |  |  |  |  |  |  |  |  |  |
| -0.441 | 6 | igd | 0.4685 | C | \|\| id:ebi-a-GCST90002105 | ebi-a-GCST90002105 | TRUE | T | 32395641 | 3.47E-46 | reported | 2872 | 0.03036 | rs9268576 |  |  |  |  |  |  |  |  |  |  |  |  |  |  |  |
| -0.421 | 6 | igd | 0.4685 | C | \|\| id:ebi-a-GCST90002112 | ebi-a-GCST90002112 | TRUE | T | 32395641 | 5.87E-27 | reported | 1635 | 0.03846 | rs9268576 |  |  |  |  |  |  |  |  |  |  |  |  |  |  |  |
| 0.8751 | 10 | igd | 0.4684 | G | \|\| id:ebi-a-GCST90001489 | ebi-a-GCST90001489 | TRUE | A | 97490998 | 1.00E-200 | reported | 3405 | 0.0212 | rs7094484 |  |  |  |  |  |  |  |  |  |  |  |  |  |  |  |
| -0.238 | 10 | igd | 0.4677 | C | \|\| id:ebi-a-GCST90001935 | ebi-a-GCST90001935 | TRUE | G | 97566052 | 1.67E-21 | reported | 3434 | 0.02482 | rs6584030 |  |  |  |  |  |  |  |  |  |  |  |  |  |  |  |
| -0.2344 | 10 | igd | 0.4677 | C | \|\| id:ebi-a-GCST90001940 | ebi-a-GCST90001940 | TRUE | G | 97566052 | 1.22E-20 | reported | 3435 | 0.02501 | rs6584030 |  |  |  |  |  |  |  |  |  |  |  |  |  |  |  |
| 0.2998 | 19 | igd | 0.4677 | C | \|\| id:ebi-a-GCST90001947 | ebi-a-GCST90001947 | TRUE | T | 51585259 | 5.81E-16 | reported | 1577 | 0.03665 | rs2691244 |  |  |  |  |  |  |  |  |  |  |  |  |  |  |  |
| 0.3132 | 19 | igd | 0.4677 | C | \|\| id:ebi-a-GCST90001957 | ebi-a-GCST90001957 | TRUE | T | 51585259 | 2.19E-17 | reported | 1578 | 0.03649 | rs2691244 |  |  |  |  |  |  |  |  |  |  |  |  |  |  |  |
| 0.1413 | 10 | igd | 0.4676 | T | \|\| id:ebi-a-GCST90001660 | ebi-a-GCST90001660 | TRUE | A | 97062933 | 2.57E-08 | reported | 3408 | 0.02532 | rs2026682 |  |  |  |  |  |  |  |  |  |  |  |  |  |  |  |
| 0.197 | 10 | igd | 0.4676 | T | \|\| id:ebi-a-GCST90001672 | ebi-a-GCST90001672 | TRUE | A | 97062933 | 1.33E-14 | reported | 3408 | 0.02546 | rs2026682 |  |  |  |  |  |  |  |  |  |  |  |  |  |  |  |
| -0.2564 | 6 | igd | 0.4676 | A | \|\| id:ebi-a-GCST90002112 | ebi-a-GCST90002112 | TRUE | G | 31602967 | 5.88E-11 | reported | 1635 | 0.03891 | rs1046089 |  |  |  |  |  |  |  |  |  |  |  |  |  |  |  |
| 0.3128 | 19 | igd | 0.4674 | C | \|\| id:ebi-a-GCST90001956 | ebi-a-GCST90001956 | TRUE | T | 51585259 | 2.27E-17 | reported | 1579 | 0.03647 | rs2691244 |  |  |  |  |  |  |  |  |  |  |  |  |  |  |  |
| 0.1669 | 1 | igd | 0.4673 | T | \|\| id:ebi-a-GCST90002087 | ebi-a-GCST90002087 | TRUE | C | 161589626 | 9.48E-10 | reported | 2872 | 0.02718 | rs61802329 |  |  |  |  |  |  |  |  |  |  |  |  |  |  |  |
| -0.1645 | 6 | igd | 0.4668 | T | \|\| id:ebi-a-GCST90002069 | ebi-a-GCST90002069 | TRUE | C | 31320242 | 2.42E-08 | reported | 2920 | 0.02941 | rs2442721 |  |  |  |  |  |  |  |  |  |  |  |  |  |  |  |
| 0.1621 | 10 | igd | 0.4664 | T | \|\| id:ebi-a-GCST90001658 | ebi-a-GCST90001658 | TRUE | A | 97062933 | 2.12E-11 | reported | 3440 | 0.02412 | rs2026682 |  |  |  |  |  |  |  |  |  |  |  |  |  |  |  |
| 0.1589 | 10 | igd | 0.4664 | T | \|\| id:ebi-a-GCST90001659 | ebi-a-GCST90001659 | TRUE | A | 97062933 | 5.23E-11 | reported | 3440 | 0.02413 | rs2026682 |  |  |  |  |  |  |  |  |  |  |  |  |  |  |  |
| 0.2106 | 10 | igd | 0.4664 | T | \|\| id:ebi-a-GCST90001670 | ebi-a-GCST90001670 | TRUE | A | 97062933 | 5.50E-17 | reported | 3440 | 0.02501 | rs2026682 |  |  |  |  |  |  |  |  |  |  |  |  |  |  |  |
| 0.2029 | 10 | igd | 0.4664 | T | \|\| id:ebi-a-GCST90001671 | ebi-a-GCST90001671 | TRUE | A | 97062933 | 7.77E-16 | reported | 3440 | 0.02506 | rs2026682 |  |  |  |  |  |  |  |  |  |  |  |  |  |  |  |
| 0.1592 | 5 | igd | 0.4659 | G | \|\| id:ebi-a-GCST90001929 | ebi-a-GCST90001929 | TRUE | A | 35831790 | 1.27E-09 | reported | 2920 | 0.02613 | rs6872264 |  |  |  |  |  |  |  |  |  |  |  |  |  |  |  |
| -0.2401 | 6 | igd | 0.4657 | C | \|\| id:ebi-a-GCST90002105 | ebi-a-GCST90002105 | TRUE | T | 31601012 | 2.27E-14 | reported | 2872 | 0.03128 | rs2844466 |  |  |  |  |  |  |  |  |  |  |  |  |  |  |  |
| 0.1557 | 1 | igd | 0.4656 | A | \|\| id:ebi-a-GCST90001981 | ebi-a-GCST90001981 | TRUE | G | 161408556 | 8.08E-10 | reported | 3618 | 0.02528 | rs12125079 |  |  |  |  |  |  |  |  |  |  |  |  |  |  |  |
| -0.1948 | 10 | igd | 0.4655 | C | \|\| id:ebi-a-GCST90001491 | ebi-a-GCST90001491 | TRUE | T | 95990833 | 8.42E-14 | reported | 3437 | 0.02599 | rs2226170 |  |  |  |  |  |  |  |  |  |  |  |  |  |  |  |
| 0.1737 | 1 | igd | 0.4651 | A | \|\| id:ebi-a-GCST90001989 | ebi-a-GCST90001989 | TRUE | G | 161408556 | 9.44E-12 | reported | 3621 | 0.0254 | rs12125079 |  |  |  |  |  |  |  |  |  |  |  |  |  |  |  |
| -0.2534 | 6 | igd | 0.4651 | C | \|\| id:ebi-a-GCST90002110 | ebi-a-GCST90002110 | TRUE | T | 31613739 | 1.93E-10 | reported | 1634 | 0.03955 | rs3130048 |  |  |  |  |  |  |  |  |  |  |  |  |  |  |  |
| -0.2396 | 6 | igd | 0.4648 | C | \|\| id:ebi-a-GCST90002111 | ebi-a-GCST90002111 | TRUE | T | 31613739 | 9.45E-10 | reported | 1635 | 0.03892 | rs3130048 |  |  |  |  |  |  |  |  |  |  |  |  |  |  |  |
| -0.2717 | 6 | igd | 0.4648 | C | \|\| id:ebi-a-GCST90002112 | ebi-a-GCST90002112 | TRUE | T | 31613739 | 6.48E-12 | reported | 1635 | 0.03926 | rs3130048 |  |  |  |  |  |  |  |  |  |  |  |  |  |  |  |
| -0.4145 | 6 | igd | 0.4645 | G | \|\| id:ebi-a-GCST90001692 | ebi-a-GCST90001692 | TRUE | C | 32528323 | 2.71E-09 | reported | 3440 | 0.0695 | rs67792106 |  |  |  |  |  |  |  |  |  |  |  |  |  |  |  |
| 0.1944 | 1 | igd | 0.4645 | A | \|\| id:ebi-a-GCST90001985 | ebi-a-GCST90001985 | TRUE | G | 161408556 | 2.09E-14 | reported | 3629 | 0.02533 | rs12125079 |  |  |  |  |  |  |  |  |  |  |  |  |  |  |  |
| 0.2155 | 6 | igd | 0.4645 | T | \|\| id:ebi-a-GCST90002105 | ebi-a-GCST90002105 | TRUE | C | 31319457 | 4.52E-12 | reported | 2872 | 0.03101 | rs9266064 |  |  |  |  |  |  |  |  |  |  |  |  |  |  |  |
| 1.952 | 6 | igd | 0.4644 | G | \|\| id:ebi-a-GCST90001697 | ebi-a-GCST90001697 | TRUE | C | 32528323 | 1.02E-08 | reported | 3425 | 0.34 | rs67792106 |  |  |  |  |  |  |  |  |  |  |  |  |  |  |  |
| 41.4 | 6 | igd | 0.4642 | G | \|\| id:ebi-a-GCST90001695 | ebi-a-GCST90001695 | TRUE | C | 32528323 | 1.07E-08 | reported | 3408 | 7.219 | rs67792106 |  |  |  |  |  |  |  |  |  |  |  |  |  |  |  |
| 4.714 | 6 | igd | 0.4641 | A | \|\| id:ebi-a-GCST90001696 | ebi-a-GCST90001696 | TRUE | G | 32528293 | 1.31E-08 | reported | 3425 | 0.8273 | rs66777350 |  |  |  |  |  |  |  |  |  |  |  |  |  |  |  |
| -0.2216 | 6 | igd | 0.4633 | G | \|\| id:ebi-a-GCST90002105 | ebi-a-GCST90002105 | TRUE | A | 31096367 | 2.27E-12 | reported | 2872 | 0.03145 | rs3909109 |  |  |  |  |  |  |  |  |  |  |  |  |  |  |  |
| 0.192 | 2 | igd | 0.463 | C | \|\| id:ebi-a-GCST90002119 | ebi-a-GCST90002119 | TRUE | T | 86022747 | 1.37E-12 | reported | 2920 | 0.02696 | rs1356625 |  |  |  |  |  |  |  |  |  |  |  |  |  |  |  |
| 0.4287 | 6 | igd | 0.4627 | A | \|\| id:ebi-a-GCST90001932 | ebi-a-GCST90001932 | TRUE | G | 32528293 | 1.47E-09 | reported | 2920 | 0.07065 | rs66777350 |  |  |  |  |  |  |  |  |  |  |  |  |  |  |  |
| 0.3125 | 1 | igd | 0.4627 | T | \|\| id:ebi-a-GCST90002014 | ebi-a-GCST90002014 | TRUE | G | 161589982 | 1.13E-28 | reported | 2870 | 0.02782 | rs61802333 |  |  |  |  |  |  |  |  |  |  |  |  |  |  |  |
| 0.1369 | 13 | igd | 0.4623 | G | \|\| id:ebi-a-GCST90001439 | ebi-a-GCST90001439 | TRUE | A | 108943034 | 4.03E-08 | reported | 3656 | 0.02489 | rs2893321 |  |  |  |  |  |  |  |  |  |  |  |  |  |  |  |
| 0.1387 | 13 | igd | 0.4623 | G | \|\| id:ebi-a-GCST90001442 | ebi-a-GCST90001442 | TRUE | A | 108943034 | 2.04E-08 | reported | 3656 | 0.02467 | rs2893321 |  |  |  |  |  |  |  |  |  |  |  |  |  |  |  |
| -0.1687 | 6 | igd | 0.4623 | G | \|\| id:ebi-a-GCST90002061 | ebi-a-GCST90002061 | TRUE | C | 31314106 | 3.33E-08 | reported | 2920 | 0.03047 | rs3016018 |  |  |  |  |  |  |  |  |  |  |  |  |  |  |  |
| -0.2045 | 6 | igd | 0.4623 | G | \|\| id:ebi-a-GCST90002067 | ebi-a-GCST90002067 | TRUE | C | 31314106 | 6.73E-12 | reported | 2920 | 0.02968 | rs3016018 |  |  |  |  |  |  |  |  |  |  |  |  |  |  |  |
| 0.1751 | 6 | igd | 0.4623 | G | \|\| id:ebi-a-GCST90002067 | ebi-a-GCST90002067 | TRUE | A | 32197463 | 2.33E-09 | reported | 2920 | 0.02923 | rs495089 |  |  |  |  |  |  |  |  |  |  |  |  |  |  |  |
| 0.3182 | 19 | igd | 0.4614 | C | \|\| id:ebi-a-GCST90001946 | ebi-a-GCST90001946 | TRUE | T | 51585259 | 6.77E-19 | reported | 1634 | 0.03541 | rs2691244 |  |  |  |  |  |  |  |  |  |  |  |  |  |  |  |
| 0.3242 | 19 | igd | 0.4614 | C | \|\| id:ebi-a-GCST90001949 | ebi-a-GCST90001949 | TRUE | T | 51585259 | 8.39E-20 | reported | 1634 | 0.03514 | rs2691244 |  |  |  |  |  |  |  |  |  |  |  |  |  |  |  |
| 0.3259 | 19 | igd | 0.4611 | C | \|\| id:ebi-a-GCST90001948 | ebi-a-GCST90001948 | TRUE | T | 51585259 | 7.23E-20 | reported | 1633 | 0.03526 | rs2691244 |  |  |  |  |  |  |  |  |  |  |  |  |  |  |  |
| -0.2007 | 6 | igd | 0.461 | T | \|\| id:ebi-a-GCST90001747 | ebi-a-GCST90001747 | TRUE | C | 32560752 | 8.12E-11 | reported | 3657 | 0.0308 | rs7768875 |  |  |  |  |  |  |  |  |  |  |  |  |  |  |  |
| -0.1706 | 6 | igd | 0.461 | T | \|\| id:ebi-a-GCST90001751 | ebi-a-GCST90001751 | TRUE | C | 32560752 | 3.05E-08 | reported | 3657 | 0.03073 | rs7768875 |  |  |  |  |  |  |  |  |  |  |  |  |  |  |  |
| -0.2448 | 6 | igd | 0.461 | T | \|\| id:ebi-a-GCST90001752 | ebi-a-GCST90001752 | TRUE | C | 32560752 | 2.89E-15 | reported | 3657 | 0.03087 | rs7768875 |  |  |  |  |  |  |  |  |  |  |  |  |  |  |  |
| -0.1987 | 6 | igd | 0.461 | T | \|\| id:ebi-a-GCST90001759 | ebi-a-GCST90001759 | TRUE | C | 32560752 | 1.08E-10 | reported | 3657 | 0.03069 | rs7768875 |  |  |  |  |  |  |  |  |  |  |  |  |  |  |  |
| -0.204 | 6 | igd | 0.4609 | T | \|\| id:ebi-a-GCST90001762 | ebi-a-GCST90001762 | TRUE | C | 32560752 | 4.86E-11 | reported | 3658 | 0.03094 | rs7768875 |  |  |  |  |  |  |  |  |  |  |  |  |  |  |  |
| -0.2426 | 6 | igd | 0.4596 | C | \|\| id:ebi-a-GCST90002105 | ebi-a-GCST90002105 | TRUE | T | 31613739 | 1.70E-14 | reported | 2872 | 0.03146 | rs3130048 |  |  |  |  |  |  |  |  |  |  |  |  |  |  |  |
| -0.2034 | 6 | igd | 0.4596 | A | \|\| id:ebi-a-GCST90002118 | ebi-a-GCST90002118 | TRUE | G | 29896275 | 1.23E-10 | reported | 2920 | 0.03149 | rs2517758 |  |  |  |  |  |  |  |  |  |  |  |  |  |  |  |
| -0.1788 | 12 | igd | 0.4591 | G | \|\| id:ebi-a-GCST90002073 | ebi-a-GCST90002073 | TRUE | A | 69577200 | 9.19E-11 | reported | 2850 | 0.02749 | rs61926908 |  |  |  |  |  |  |  |  |  |  |  |  |  |  |  |
| -0.1704 | 6 | igd | 0.4586 | T | \|\| id:ebi-a-GCST90002067 | ebi-a-GCST90002067 | TRUE | C | 31326131 | 1.27E-08 | reported | 2920 | 0.02985 | rs73390208 |  |  |  |  |  |  |  |  |  |  |  |  |  |  |  |
| 0.2984 | 22 | igd | 0.458 | G | \|\| id:ebi-a-GCST90001702 | ebi-a-GCST90001702 | TRUE | A | 42812471 | 4.84E-32 | reported | 3657 | 0.02509 | rs735537 |  |  |  |  |  |  |  |  |  |  |  |  |  |  |  |
| 0.2927 | 22 | igd | 0.458 | G | \|\| id:ebi-a-GCST90001703 | ebi-a-GCST90001703 | TRUE | A | 42812471 | 6.96E-31 | reported | 3657 | 0.0251 | rs735537 |  |  |  |  |  |  |  |  |  |  |  |  |  |  |  |
| 0.2844 | 22 | igd | 0.458 | G | \|\| id:ebi-a-GCST90001704 | ebi-a-GCST90001704 | TRUE | A | 42812471 | 2.66E-29 | reported | 3657 | 0.02509 | rs735537 |  |  |  |  |  |  |  |  |  |  |  |  |  |  |  |
| 0.2907 | 22 | igd | 0.458 | G | \|\| id:ebi-a-GCST90001710 | ebi-a-GCST90001710 | TRUE | A | 42812471 | 1.28E-30 | reported | 3657 | 0.02505 | rs735537 |  |  |  |  |  |  |  |  |  |  |  |  |  |  |  |
| 0.2798 | 22 | igd | 0.458 | G | \|\| id:ebi-a-GCST90001711 | ebi-a-GCST90001711 | TRUE | A | 42812471 | 1.34E-28 | reported | 3657 | 0.02501 | rs735537 |  |  |  |  |  |  |  |  |  |  |  |  |  |  |  |
| 0.3265 | 1 | igd | 0.4579 | C | \|\| id:ebi-a-GCST90001852 | ebi-a-GCST90001852 | TRUE | T | 167433056 | 1.57E-31 | reported | 2649 | 0.02759 | rs1723016 |  |  |  |  |  |  |  |  |  |  |  |  |  |  |  |
| 0.1466 | 19 | igd | 0.4572 | C | \|\| id:ebi-a-GCST90001745 | ebi-a-GCST90001745 | TRUE | T | 49462482 | 3.07E-09 | reported | 3657 | 0.02468 | rs7255991 |  |  |  |  |  |  |  |  |  |  |  |  |  |  |  |
| 0.1884 | 6 | igd | 0.4572 | T | \|\| id:ebi-a-GCST90002062 | ebi-a-GCST90002062 | TRUE | C | 31247414 | 9.04E-10 | reported | 2920 | 0.03065 | rs2844608 |  |  |  |  |  |  |  |  |  |  |  |  |  |  |  |
| 0.2196 | 6 | igd | 0.4572 | T | \|\| id:ebi-a-GCST90002119 | ebi-a-GCST90002119 | TRUE | C | 31247414 | 8.61E-13 | reported | 2920 | 0.03057 | rs2844608 |  |  |  |  |  |  |  |  |  |  |  |  |  |  |  |
| 0.3496 | 1 | igd | 0.4569 | G | \|\| id:ebi-a-GCST90001866 | ebi-a-GCST90001866 | TRUE | A | 167433420 | 7.82E-39 | reported | 2914 | 0.02643 | rs1723018 |  |  |  |  |  |  |  |  |  |  |  |  |  |  |  |
| 0.3668 | 1 | igd | 0.4567 | G | \|\| id:ebi-a-GCST90001863 | ebi-a-GCST90001863 | TRUE | A | 167433420 | 7.25E-43 | reported | 2919 | 0.0263 | rs1723018 |  |  |  |  |  |  |  |  |  |  |  |  |  |  |  |
| -0.2427 | 1 | igd | 0.4563 | G | \|\| id:ebi-a-GCST90001880 | ebi-a-GCST90001880 | TRUE | C | 2623180 | 6.89E-10 | reported | 1247 | 0.03903 | rs55713073 |  |  |  |  |  |  |  |  |  |  |  |  |  |  |  |
| -0.6123 | 14 | igd | 0.4558 | T | \|\| id:ebi-a-GCST90001820 | ebi-a-GCST90001820 | TRUE | C | 106306841 | 4.42E-87 | reported | 3656 | 0.03013 | rs709589 |  |  |  |  |  |  |  |  |  |  |  |  |  |  |  |
| -0.183 | 14 | igd | 0.4557 | T | \|\| id:ebi-a-GCST90001779 | ebi-a-GCST90001779 | TRUE | C | 106306841 | 5.98E-09 | reported | 3657 | 0.03138 | rs709589 |  |  |  |  |  |  |  |  |  |  |  |  |  |  |  |
| -0.2486 | 14 | igd | 0.4557 | T | \|\| id:ebi-a-GCST90001783 | ebi-a-GCST90001783 | TRUE | C | 106306841 | 1.19E-15 | reported | 3656 | 0.03091 | rs709589 |  |  |  |  |  |  |  |  |  |  |  |  |  |  |  |
| -0.2255 | 14 | igd | 0.4557 | T | \|\| id:ebi-a-GCST90001795 | ebi-a-GCST90001795 | TRUE | C | 106306841 | 5.41E-13 | reported | 3657 | 0.03114 | rs709589 |  |  |  |  |  |  |  |  |  |  |  |  |  |  |  |
| -0.1755 | 14 | igd | 0.4557 | T | \|\| id:ebi-a-GCST90001806 | ebi-a-GCST90001806 | TRUE | C | 106306841 | 2.78E-08 | reported | 3656 | 0.03152 | rs709589 |  |  |  |  |  |  |  |  |  |  |  |  |  |  |  |
| -0.6633 | 14 | igd | 0.4557 | T | \|\| id:ebi-a-GCST90001821 | ebi-a-GCST90001821 | TRUE | C | 106306841 | 4.57E-103 | reported | 3656 | 0.02979 | rs709589 |  |  |  |  |  |  |  |  |  |  |  |  |  |  |  |
| 0.1767 | 6 | igd | 0.4553 | G | \|\| id:ebi-a-GCST90001743 | ebi-a-GCST90001743 | TRUE | A | 32451834 | 7.68E-10 | reported | 3660 | 0.02864 | rs62405562 |  |  |  |  |  |  |  |  |  |  |  |  |  |  |  |
| 0.1921 | 6 | igd | 0.4552 | G | \|\| id:ebi-a-GCST90001762 | ebi-a-GCST90001762 | TRUE | A | 32451834 | 2.46E-11 | reported | 3658 | 0.02868 | rs62405562 |  |  |  |  |  |  |  |  |  |  |  |  |  |  |  |
| 0.1942 | 6 | igd | 0.455 | G | \|\| id:ebi-a-GCST90001747 | ebi-a-GCST90001747 | TRUE | A | 32451834 | 1.15E-11 | reported | 3657 | 0.02853 | rs62405562 |  |  |  |  |  |  |  |  |  |  |  |  |  |  |  |
| 0.2285 | 6 | igd | 0.455 | G | \|\| id:ebi-a-GCST90001752 | ebi-a-GCST90001752 | TRUE | A | 32451834 | 1.82E-15 | reported | 3657 | 0.02861 | rs62405562 |  |  |  |  |  |  |  |  |  |  |  |  |  |  |  |
| 0.1848 | 6 | igd | 0.455 | G | \|\| id:ebi-a-GCST90001759 | ebi-a-GCST90001759 | TRUE | A | 32451834 | 9.18E-11 | reported | 3657 | 0.02844 | rs62405562 |  |  |  |  |  |  |  |  |  |  |  |  |  |  |  |
| -0.2484 | 14 | igd | 0.4529 | T | \|\| id:ebi-a-GCST90001781 | ebi-a-GCST90001781 | TRUE | C | 106306841 | 1.84E-08 | reported | 1835 | 0.04396 | rs709589 |  |  |  |  |  |  |  |  |  |  |  |  |  |  |  |
| -0.1557 | 10 | igd | 0.4518 | A | \|\| id:ebi-a-GCST90001496 | ebi-a-GCST90001496 | TRUE | G | 98427670 | 1.49E-09 | reported | 3437 | 0.02568 | rs1992057 |  |  |  |  |  |  |  |  |  |  |  |  |  |  |  |
| -0.1566 | 10 | igd | 0.4518 | A | \|\| id:ebi-a-GCST90001497 | ebi-a-GCST90001497 | TRUE | G | 98427670 | 7.34E-10 | reported | 3437 | 0.02536 | rs1992057 |  |  |  |  |  |  |  |  |  |  |  |  |  |  |  |
| 0.1571 | 2 | igd | 0.4514 | C | \|\| id:ebi-a-GCST90002098 | ebi-a-GCST90002098 | TRUE | T | 38418229 | 2.74E-09 | reported | 2910 | 0.02634 | rs232591 |  |  |  |  |  |  |  |  |  |  |  |  |  |  |  |
| 0.2273 | 19 | igd | 0.4498 | C | \|\| id:ebi-a-GCST90001954 | ebi-a-GCST90001954 | TRUE | A | 51882663 | 7.27E-10 | reported | 1555 | 0.03667 | rs2547321 |  |  |  |  |  |  |  |  |  |  |  |  |  |  |  |
| 0.224 | 19 | igd | 0.4498 | C | \|\| id:ebi-a-GCST90001955 | ebi-a-GCST90001955 | TRUE | A | 51882663 | 1.31E-09 | reported | 1554 | 0.03669 | rs2547321 |  |  |  |  |  |  |  |  |  |  |  |  |  |  |  |
| 0.2252 | 19 | igd | 0.4496 | C | \|\| id:ebi-a-GCST90001953 | ebi-a-GCST90001953 | TRUE | A | 51882663 | 1.02E-09 | reported | 1558 | 0.03664 | rs2547321 |  |  |  |  |  |  |  |  |  |  |  |  |  |  |  |
| 0.1783 | 6 | igd | 0.4483 | C | \|\| id:ebi-a-GCST90001932 | ebi-a-GCST90001932 | TRUE | T | 31071758 | 3.29E-09 | reported | 2920 | 0.03004 | rs9263601 |  |  |  |  |  |  |  |  |  |  |  |  |  |  |  |
| 0.2073 | 19 | igd | 0.4483 | C | \|\| id:ebi-a-GCST90001947 | ebi-a-GCST90001947 | TRUE | A | 51882663 | 2.12E-08 | reported | 1577 | 0.03683 | rs2547321 |  |  |  |  |  |  |  |  |  |  |  |  |  |  |  |
| -0.1582 | 10 | igd | 0.4483 | A | \|\| id:ebi-a-GCST90002032 | ebi-a-GCST90002032 | TRUE | G | 98427670 | 1.91E-08 | reported | 2920 | 0.02808 | rs1992057 |  |  |  |  |  |  |  |  |  |  |  |  |  |  |  |
| 0.2002 | 6 | igd | 0.4483 | C | \|\| id:ebi-a-GCST90002118 | ebi-a-GCST90002118 | TRUE | T | 31071758 | 8.61E-11 | reported | 2920 | 0.03074 | rs9263601 |  |  |  |  |  |  |  |  |  |  |  |  |  |  |  |
| 0.1763 | 6 | igd | 0.4483 | C | \|\| id:ebi-a-GCST90002120 | ebi-a-GCST90002120 | TRUE | T | 31071758 | 4.31E-09 | reported | 2920 | 0.02994 | rs9263601 |  |  |  |  |  |  |  |  |  |  |  |  |  |  |  |
| 0.212 | 19 | igd | 0.4481 | C | \|\| id:ebi-a-GCST90001956 | ebi-a-GCST90001956 | TRUE | A | 51882663 | 9.50E-09 | reported | 1579 | 0.03673 | rs2547321 |  |  |  |  |  |  |  |  |  |  |  |  |  |  |  |
| 0.2171 | 19 | igd | 0.448 | C | \|\| id:ebi-a-GCST90001957 | ebi-a-GCST90001957 | TRUE | A | 51882663 | 4.27E-09 | reported | 1578 | 0.03675 | rs2547321 |  |  |  |  |  |  |  |  |  |  |  |  |  |  |  |
| 0.1793 | 6 | igd | 0.4474 | T | \|\| id:ebi-a-GCST90001932 | ebi-a-GCST90001932 | TRUE | C | 32673894 | 3.68E-09 | reported | 2920 | 0.03031 | rs1794269 |  |  |  |  |  |  |  |  |  |  |  |  |  |  |  |
| -0.1859 | 3 | igd | 0.447 | A | \|\| id:ebi-a-GCST90002003 | ebi-a-GCST90002003 | TRUE | G | 46402627 | 9.48E-14 | reported | 3629 | 0.02487 | rs762789 |  |  |  |  |  |  |  |  |  |  |  |  |  |  |  |
| 0.5797 | 1 | igd | 0.4466 | T | \|\| id:ebi-a-GCST90001854 | ebi-a-GCST90001854 | TRUE | C | 167424924 | 5.30E-108 | reported | 2919 | 0.02517 | rs2949661 |  |  |  |  |  |  |  |  |  |  |  |  |  |  |  |
| 0.5477 | 1 | igd | 0.4466 | T | \|\| id:ebi-a-GCST90001856 | ebi-a-GCST90001856 | TRUE | C | 167424924 | 3.54E-94 | reported | 2919 | 0.02564 | rs2949661 |  |  |  |  |  |  |  |  |  |  |  |  |  |  |  |
| 0.6441 | 1 | igd | 0.4466 | T | \|\| id:ebi-a-GCST90001858 | ebi-a-GCST90001858 | TRUE | C | 167424924 | 1.66E-133 | reported | 2919 | 0.02485 | rs2949661 |  |  |  |  |  |  |  |  |  |  |  |  |  |  |  |
| 0.644 | 1 | igd | 0.4466 | T | \|\| id:ebi-a-GCST90001860 | ebi-a-GCST90001860 | TRUE | C | 167424924 | 3.76E-132 | reported | 2919 | 0.02499 | rs2949661 |  |  |  |  |  |  |  |  |  |  |  |  |  |  |  |
| 0.6021 | 1 | igd | 0.4466 | A | \|\| id:ebi-a-GCST90001864 | ebi-a-GCST90001864 | TRUE | G | 167431193 | 1.10E-115 | reported | 2919 | 0.02516 | rs2995089 |  |  |  |  |  |  |  |  |  |  |  |  |  |  |  |
| 0.521 | 1 | igd | 0.4462 | T | \|\| id:ebi-a-GCST90001845 | ebi-a-GCST90001845 | TRUE | C | 167424924 | 1.05E-84 | reported | 2911 | 0.02584 | rs2949661 |  |  |  |  |  |  |  |  |  |  |  |  |  |  |  |
| 0.4749 | 1 | igd | 0.4462 | T | \|\| id:ebi-a-GCST90001861 | ebi-a-GCST90001861 | TRUE | C | 167431352 | 1.61E-71 | reported | 2919 | 0.02583 | rs2988277 |  |  |  |  |  |  |  |  |  |  |  |  |  |  |  |
| 0.2075 | 19 | igd | 0.4455 | C | \|\| id:ebi-a-GCST90001948 | ebi-a-GCST90001948 | TRUE | A | 51882663 | 7.32E-09 | reported | 1633 | 0.03568 | rs2547321 |  |  |  |  |  |  |  |  |  |  |  |  |  |  |  |
| 0.2114 | 19 | igd | 0.4452 | C | \|\| id:ebi-a-GCST90001946 | ebi-a-GCST90001946 | TRUE | A | 51882663 | 4.18E-09 | reported | 1634 | 0.03578 | rs2547321 |  |  |  |  |  |  |  |  |  |  |  |  |  |  |  |
| 0.1728 | 2 | igd | 0.4443 | T | \|\| id:ebi-a-GCST90001900 | ebi-a-GCST90001900 | TRUE | G | 203046418 | 3.97E-10 | reported | 2919 | 0.02753 | rs2177084 |  |  |  |  |  |  |  |  |  |  |  |  |  |  |  |
| -0.265 | 6 | igd | 0.4439 | A | \|\| id:ebi-a-GCST90001752 | ebi-a-GCST90001752 | TRUE | T | 32627105 | 5.36E-13 | reported | 3657 | 0.03659 | rs9273396 |  |  |  |  |  |  |  |  |  |  |  |  |  |  |  |
| -0.2019 | 6 | igd | 0.4439 | A | \|\| id:ebi-a-GCST90001759 | ebi-a-GCST90001759 | TRUE | T | 32627105 | 3.36E-08 | reported | 3657 | 0.03648 | rs9273396 |  |  |  |  |  |  |  |  |  |  |  |  |  |  |  |
| -0.155 | 22 | igd | 0.4428 | G | \|\| id:ebi-a-GCST90001710 | ebi-a-GCST90001710 | TRUE | A | 39415780 | 3.01E-10 | reported | 3657 | 0.02454 | rs5757424 |  |  |  |  |  |  |  |  |  |  |  |  |  |  |  |
| -0.155 | 22 | igd | 0.4428 | G | \|\| id:ebi-a-GCST90001711 | ebi-a-GCST90001711 | TRUE | A | 39415780 | 2.74E-10 | reported | 3657 | 0.02449 | rs5757424 |  |  |  |  |  |  |  |  |  |  |  |  |  |  |  |
| -0.1512 | 22 | igd | 0.4422 | T | \|\| id:ebi-a-GCST90001704 | ebi-a-GCST90001704 | TRUE | G | 39414904 | 8.25E-10 | reported | 3657 | 0.02456 | rs5757423 |  |  |  |  |  |  |  |  |  |  |  |  |  |  |  |
| 0.1995 | 6 | igd | 0.4422 | C | \|\| id:ebi-a-GCST90002035 | ebi-a-GCST90002035 | TRUE | T | 90850164 | 1.17E-12 | reported | 2871 | 0.02793 | rs2325259 |  |  |  |  |  |  |  |  |  |  |  |  |  |  |  |
| 0.2265 | 6 | igd | 0.4422 | G | \|\| id:ebi-a-GCST90002036 | ebi-a-GCST90002036 | TRUE | T | 90852258 | 7.54E-16 | reported | 2871 | 0.02793 | rs12199079 |  |  |  |  |  |  |  |  |  |  |  |  |  |  |  |
| 0.1845 | 2 | igd | 0.4404 | A | \|\| id:ebi-a-GCST90002119 | ebi-a-GCST90002119 | TRUE | G | 86427821 | 2.02E-11 | reported | 2920 | 0.02741 | rs2059352 |  |  |  |  |  |  |  |  |  |  |  |  |  |  |  |
| 0.1619 | 6 | igd | 0.4387 | C | \|\| id:ebi-a-GCST90001901 | ebi-a-GCST90001901 | TRUE | T | 90946479 | 3.44E-09 | reported | 2650 | 0.0273 | rs4707609 |  |  |  |  |  |  |  |  |  |  |  |  |  |  |  |
| 0.156 | 2 | igd | 0.4375 | C | \|\| id:ebi-a-GCST90002118 | ebi-a-GCST90002118 | TRUE | T | 86440436 | 1.72E-08 | reported | 2920 | 0.0276 | rs3087642 |  |  |  |  |  |  |  |  |  |  |  |  |  |  |  |
| -0.5165 | 6 | igd | 0.437 | C | \|\| id:ebi-a-GCST90002108 | ebi-a-GCST90002108 | TRUE | G | 32451788 | 2.61E-37 | reported | 1579 | 0.03942 | rs9269234 |  |  |  |  |  |  |  |  |  |  |  |  |  |  |  |
| -0.3405 | 6 | igd | 0.4369 | C | \|\| id:ebi-a-GCST90002109 | ebi-a-GCST90002109 | TRUE | G | 32451788 | 5.50E-17 | reported | 1578 | 0.0402 | rs9269234 |  |  |  |  |  |  |  |  |  |  |  |  |  |  |  |
| 0.1895 | 10 | igd | 0.4365 | G | \|\| id:ebi-a-GCST90001658 | ebi-a-GCST90001658 | TRUE | A | 97239708 | 4.03E-14 | reported | 3440 | 0.02496 | rs7071071 |  |  |  |  |  |  |  |  |  |  |  |  |  |  |  |
| 0.1915 | 10 | igd | 0.4365 | G | \|\| id:ebi-a-GCST90001659 | ebi-a-GCST90001659 | TRUE | A | 97239708 | 2.20E-14 | reported | 3440 | 0.02496 | rs7071071 |  |  |  |  |  |  |  |  |  |  |  |  |  |  |  |
| 0.2145 | 10 | igd | 0.4361 | G | \|\| id:ebi-a-GCST90001484 | ebi-a-GCST90001484 | TRUE | A | 97239708 | 2.67E-17 | reported | 3437 | 0.02521 | rs7071071 |  |  |  |  |  |  |  |  |  |  |  |  |  |  |  |
| 0.21 | 10 | igd | 0.4361 | G | \|\| id:ebi-a-GCST90001485 | ebi-a-GCST90001485 | TRUE | A | 97239708 | 1.11E-16 | reported | 3437 | 0.02519 | rs7071071 |  |  |  |  |  |  |  |  |  |  |  |  |  |  |  |
| 0.2108 | 10 | igd | 0.4351 | G | \|\| id:ebi-a-GCST90001483 | ebi-a-GCST90001483 | TRUE | A | 97239708 | 1.66E-16 | reported | 3405 | 0.02544 | rs7071071 |  |  |  |  |  |  |  |  |  |  |  |  |  |  |  |
| 0.1434 | 2 | igd | 0.4342 | T | \|\| id:ebi-a-GCST90002102 | ebi-a-GCST90002102 | TRUE | C | 38425644 | 4.36E-08 | reported | 2919 | 0.02613 | rs232584 |  |  |  |  |  |  |  |  |  |  |  |  |  |  |  |
| -0.2187 | 6 | igd | 0.4338 | T | \|\| id:ebi-a-GCST90002105 | ebi-a-GCST90002105 | TRUE | C | 32805307 | 9.32E-13 | reported | 2872 | 0.03049 | rs2071466 |  |  |  |  |  |  |  |  |  |  |  |  |  |  |  |
| -0.5168 | 6 | igd | 0.4338 | G | \|\| id:ebi-a-GCST90002108 | ebi-a-GCST90002108 | TRUE | A | 32594188 | 4.69E-35 | reported | 1579 | 0.04082 | rs9271768 |  |  |  |  |  |  |  |  |  |  |  |  |  |  |  |
| 0.1463 | 10 | igd | 0.4324 | A | \|\| id:ebi-a-GCST90001935 | ebi-a-GCST90001935 | TRUE | G | 6096600 | 9.97E-09 | reported | 3434 | 0.02546 | rs7917726 |  |  |  |  |  |  |  |  |  |  |  |  |  |  |  |
| 0.1419 | 10 | igd | 0.4323 | A | \|\| id:ebi-a-GCST90001940 | ebi-a-GCST90001940 | TRUE | G | 6096600 | 3.46E-08 | reported | 3435 | 0.02567 | rs7917726 |  |  |  |  |  |  |  |  |  |  |  |  |  |  |  |
| 0.2074 | 6 | igd | 0.4309 | A | \|\| id:ebi-a-GCST90002105 | ebi-a-GCST90002105 | TRUE | G | 31326289 | 4.96E-11 | reported | 2872 | 0.03143 | rs9378248 |  |  |  |  |  |  |  |  |  |  |  |  |  |  |  |
| -0.1723 | 1 | igd | 0.4305 | C | \|\| id:ebi-a-GCST90002077 | ebi-a-GCST90002077 | TRUE | T | 160798209 | 4.62E-10 | reported | 2971 | 0.02756 | rs569911 |  |  |  |  |  |  |  |  |  |  |  |  |  |  |  |
| -0.1551 | 1 | igd | 0.43 | G | \|\| id:ebi-a-GCST90001970 | ebi-a-GCST90001970 | TRUE | T | 160797323 | 2.68E-08 | reported | 2971 | 0.02782 | rs577453 |  |  |  |  |  |  |  |  |  |  |  |  |  |  |  |
| 0.1784 | 6 | igd | 0.43 | T | \|\| id:ebi-a-GCST90002119 | ebi-a-GCST90002119 | TRUE | C | 31323677 | 7.92E-09 | reported | 2920 | 0.03082 | rs4081559 |  |  |  |  |  |  |  |  |  |  |  |  |  |  |  |
| 0.2191 | 6 | igd | 0.4296 | A | \|\| id:ebi-a-GCST90002067 | ebi-a-GCST90002067 | TRUE | G | 31323567 | 8.01E-13 | reported | 2920 | 0.03046 | rs4081560 |  |  |  |  |  |  |  |  |  |  |  |  |  |  |  |
| -0.2194 | 10 | igd | 0.4293 | G | \|\| id:ebi-a-GCST90001854 | ebi-a-GCST90001854 | TRUE | A | 97624207 | 7.94E-16 | reported | 2919 | 0.02709 | rs3176881 |  |  |  |  |  |  |  |  |  |  |  |  |  |  |  |
| 0.2125 | 6 | igd | 0.4279 | T | \|\| id:ebi-a-GCST90002067 | ebi-a-GCST90002067 | TRUE | A | 31029401 | 2.10E-12 | reported | 2920 | 0.03011 | rs2517514 |  |  |  |  |  |  |  |  |  |  |  |  |  |  |  |
| 0.1778 | 2 | igd | 0.4279 | G | \|\| id:ebi-a-GCST90002119 | ebi-a-GCST90002119 | TRUE | A | 86326717 | 1.08E-10 | reported | 2920 | 0.02745 | rs72847885 |  |  |  |  |  |  |  |  |  |  |  |  |  |  |  |
| 0.1703 | 6 | igd | 0.4274 | A | \|\| id:ebi-a-GCST90002118 | ebi-a-GCST90002118 | TRUE | G | 31483458 | 3.87E-08 | reported | 2920 | 0.03089 | rs9267444 |  |  |  |  |  |  |  |  |  |  |  |  |  |  |  |
| -0.1967 | 6 | igd | 0.4272 | A | \|\| id:ebi-a-GCST90002105 | ebi-a-GCST90002105 | TRUE | G | 31483458 | 1.39E-09 | reported | 2872 | 0.03236 | rs9267444 |  |  |  |  |  |  |  |  |  |  |  |  |  |  |  |
| -0.2226 | 10 | igd | 0.4269 | C | \|\| id:ebi-a-GCST90001854 | ebi-a-GCST90001854 | TRUE | T | 97510704 | 3.43E-16 | reported | 2919 | 0.02713 | rs10882659 |  |  |  |  |  |  |  |  |  |  |  |  |  |  |  |
| -0.413 | 3 | igd | 0.4269 | A | \|\| id:ebi-a-GCST90002015 | ebi-a-GCST90002015 | TRUE | G | 46278670 | 7.46E-49 | reported | 2871 | 0.02758 | rs6802288 |  |  |  |  |  |  |  |  |  |  |  |  |  |  |  |
| -0.412 | 3 | igd | 0.4269 | A | \|\| id:ebi-a-GCST90002016 | ebi-a-GCST90002016 | TRUE | G | 46278670 | 8.27E-49 | reported | 2871 | 0.02753 | rs6802288 |  |  |  |  |  |  |  |  |  |  |  |  |  |  |  |
| 0.8426 | 10 | igd | 0.4267 | A | \|\| id:ebi-a-GCST90001485 | ebi-a-GCST90001485 | TRUE | G | 97562938 | 1.00E-200 | reported | 3437 | 0.02078 | rs4918969 |  |  |  |  |  |  |  |  |  |  |  |  |  |  |  |
| 0.8878 | 10 | igd | 0.4265 | G | \|\| id:ebi-a-GCST90001484 | ebi-a-GCST90001484 | TRUE | A | 97580944 | 1.00E-200 | reported | 3437 | 0.02026 | rs3176891 |  |  |  |  |  |  |  |  |  |  |  |  |  |  |  |
| 0.8068 | 10 | igd | 0.4264 | G | \|\| id:ebi-a-GCST90001483 | ebi-a-GCST90001483 | TRUE | C | 97569998 | 1.00E-200 | reported | 3405 | 0.02149 | rs4918971 |  |  |  |  |  |  |  |  |  |  |  |  |  |  |  |
| -0.1404 | 16 | igd | 0.4262 | T | \|\| id:ebi-a-GCST90001735 | ebi-a-GCST90001735 | TRUE | C | 28529716 | 2.10E-08 | reported | 3657 | 0.02501 | rs240705 |  |  |  |  |  |  |  |  |  |  |  |  |  |  |  |
| -0.139 | 16 | igd | 0.4262 | T | \|\| id:ebi-a-GCST90001740 | ebi-a-GCST90001740 | TRUE | C | 28529716 | 3.47E-08 | reported | 3657 | 0.02514 | rs240705 |  |  |  |  |  |  |  |  |  |  |  |  |  |  |  |
| -0.1602 | 6 | igd | 0.4243 | T | \|\| id:ebi-a-GCST90001910 | ebi-a-GCST90001910 | TRUE | C | 31029258 | 3.74E-08 | reported | 3112 | 0.02905 | rs2517515 |  |  |  |  |  |  |  |  |  |  |  |  |  |  |  |
| 0.2498 | 6 | igd | 0.4236 | A | \|\| id:ebi-a-GCST90001890 | ebi-a-GCST90001890 | TRUE | G | 90871587 | 1.45E-18 | reported | 2920 | 0.02822 | rs62408206 |  |  |  |  |  |  |  |  |  |  |  |  |  |  |  |
| 0.1709 | 6 | igd | 0.4236 | A | \|\| id:ebi-a-GCST90001894 | ebi-a-GCST90001894 | TRUE | G | 90871587 | 1.22E-09 | reported | 2920 | 0.02804 | rs62408206 |  |  |  |  |  |  |  |  |  |  |  |  |  |  |  |
| 0.3872 | 6 | igd | 0.4236 | A | \|\| id:ebi-a-GCST90001896 | ebi-a-GCST90001896 | TRUE | G | 90871587 | 1.22E-42 | reported | 2920 | 0.02783 | rs62408206 |  |  |  |  |  |  |  |  |  |  |  |  |  |  |  |
| 0.1633 | 6 | igd | 0.4236 | A | \|\| id:ebi-a-GCST90001898 | ebi-a-GCST90001898 | TRUE | G | 90871587 | 6.42E-09 | reported | 2920 | 0.02804 | rs62408206 |  |  |  |  |  |  |  |  |  |  |  |  |  |  |  |
| 0.211789 | 10 | igd | 0.423434 | C | \|\| id:met-b-156 | met-b-156 | TRUE | A | 97408603 | 4.93E-26 | reported | 431 | 0.0200695 | rs4258314 |  |  |  |  |  |  |  |  |  |  |  |  |  |  |  |
| 0.2282 | 6 | igd | 0.4234 | A | \|\| id:ebi-a-GCST90002108 | ebi-a-GCST90002108 | TRUE | G | 31326289 | 1.54E-08 | reported | 1579 | 0.04013 | rs9378248 |  |  |  |  |  |  |  |  |  |  |  |  |  |  |  |
| 0.2065 | 6 | igd | 0.4226 | A | \|\| id:ebi-a-GCST90001752 | ebi-a-GCST90001752 | TRUE | G | 32636364 | 4.83E-12 | reported | 3657 | 0.02978 | rs9274657 |  |  |  |  |  |  |  |  |  |  |  |  |  |  |  |
| 0.174 | 6 | igd | 0.4226 | A | \|\| id:ebi-a-GCST90001757 | ebi-a-GCST90001757 | TRUE | G | 32636364 | 7.35E-09 | reported | 3657 | 0.03002 | rs9274657 |  |  |  |  |  |  |  |  |  |  |  |  |  |  |  |
| 0.17 | 6 | igd | 0.4226 | A | \|\| id:ebi-a-GCST90001761 | ebi-a-GCST90001761 | TRUE | G | 32636364 | 1.96E-08 | reported | 3657 | 0.0302 | rs9274657 |  |  |  |  |  |  |  |  |  |  |  |  |  |  |  |
| 0.1424 | 10 | igd | 0.4224 | T | \|\| id:ebi-a-GCST90001670 | ebi-a-GCST90001670 | TRUE | C | 95680019 | 2.91E-08 | reported | 3440 | 0.02561 | rs12359848 |  |  |  |  |  |  |  |  |  |  |  |  |  |  |  |
| -0.3322 | 2 | igd | 0.4223 | T | \|\| id:ebi-a-GCST90002119 | ebi-a-GCST90002119 | TRUE | C | 87082679 | 7.79E-35 | reported | 2920 | 0.02663 | rs11688534 |  |  |  |  |  |  |  |  |  |  |  |  |  |  |  |
| -0.1746 | 2 | igd | 0.4223 | T | \|\| id:ebi-a-GCST90002121 | ebi-a-GCST90002121 | TRUE | C | 87082679 | 2.31E-10 | reported | 2915 | 0.02745 | rs11688534 |  |  |  |  |  |  |  |  |  |  |  |  |  |  |  |
| -0.1479 | 10 | igd | 0.4221 | C | \|\| id:ebi-a-GCST90001660 | ebi-a-GCST90001660 | TRUE | T | 97963179 | 1.16E-08 | reported | 3408 | 0.02585 | rs7088458 |  |  |  |  |  |  |  |  |  |  |  |  |  |  |  |
| -0.1651 | 10 | igd | 0.4221 | C | \|\| id:ebi-a-GCST90001672 | ebi-a-GCST90001672 | TRUE | T | 97963179 | 2.75E-10 | reported | 3408 | 0.02608 | rs7088458 |  |  |  |  |  |  |  |  |  |  |  |  |  |  |  |
| -0.1754 | 10 | igd | 0.4221 | C | \|\| id:ebi-a-GCST90002030 | ebi-a-GCST90002030 | TRUE | T | 97963179 | 1.23E-10 | reported | 2920 | 0.02715 | rs7088458 |  |  |  |  |  |  |  |  |  |  |  |  |  |  |  |
| -0.1451 | 10 | igd | 0.4219 | C | \|\| id:ebi-a-GCST90001483 | ebi-a-GCST90001483 | TRUE | T | 97963179 | 1.03E-08 | reported | 3405 | 0.02528 | rs7088458 |  |  |  |  |  |  |  |  |  |  |  |  |  |  |  |
| -0.1807 | 6 | igd | 0.4219 | A | \|\| id:ebi-a-GCST90002119 | ebi-a-GCST90002119 | TRUE | G | 32183175 | 1.39E-09 | reported | 2920 | 0.02973 | rs3131290 |  |  |  |  |  |  |  |  |  |  |  |  |  |  |  |
| 0.146 | 10 | igd | 0.4215 | T | \|\| id:ebi-a-GCST90001672 | ebi-a-GCST90001672 | TRUE | C | 95680019 | 2.46E-08 | reported | 3408 | 0.02612 | rs12359848 |  |  |  |  |  |  |  |  |  |  |  |  |  |  |  |
| -0.1653 | 10 | igd | 0.4214 | C | \|\| id:ebi-a-GCST90001658 | ebi-a-GCST90001658 | TRUE | T | 97963179 | 2.18E-11 | reported | 3440 | 0.02461 | rs7088458 |  |  |  |  |  |  |  |  |  |  |  |  |  |  |  |
| -0.1708 | 10 | igd | 0.4214 | C | \|\| id:ebi-a-GCST90001659 | ebi-a-GCST90001659 | TRUE | T | 97963179 | 4.58E-12 | reported | 3440 | 0.02461 | rs7088458 |  |  |  |  |  |  |  |  |  |  |  |  |  |  |  |
| -0.171 | 10 | igd | 0.4214 | C | \|\| id:ebi-a-GCST90001670 | ebi-a-GCST90001670 | TRUE | T | 97963179 | 2.97E-11 | reported | 3440 | 0.02563 | rs7088458 |  |  |  |  |  |  |  |  |  |  |  |  |  |  |  |
| -0.1667 | 10 | igd | 0.4214 | C | \|\| id:ebi-a-GCST90001671 | ebi-a-GCST90001671 | TRUE | T | 97963179 | 9.43E-11 | reported | 3440 | 0.02567 | rs7088458 |  |  |  |  |  |  |  |  |  |  |  |  |  |  |  |
| -0.1694 | 10 | igd | 0.4212 | C | \|\| id:ebi-a-GCST90001484 | ebi-a-GCST90001484 | TRUE | T | 97963179 | 1.60E-11 | reported | 3437 | 0.02505 | rs7088458 |  |  |  |  |  |  |  |  |  |  |  |  |  |  |  |
| -0.1532 | 10 | igd | 0.4212 | C | \|\| id:ebi-a-GCST90001485 | ebi-a-GCST90001485 | TRUE | T | 97963179 | 1.06E-09 | reported | 3437 | 0.02504 | rs7088458 |  |  |  |  |  |  |  |  |  |  |  |  |  |  |  |
| -0.1502 | 10 | igd | 0.4212 | C | \|\| id:ebi-a-GCST90001491 | ebi-a-GCST90001491 | TRUE | T | 97963179 | 4.48E-09 | reported | 3437 | 0.02553 | rs7088458 |  |  |  |  |  |  |  |  |  |  |  |  |  |  |  |
| -0.1602 | 10 | igd | 0.4212 | C | \|\| id:ebi-a-GCST90001496 | ebi-a-GCST90001496 | TRUE | T | 97963179 | 6.59E-10 | reported | 3437 | 0.02586 | rs7088458 |  |  |  |  |  |  |  |  |  |  |  |  |  |  |  |
| -0.1543 | 10 | igd | 0.4212 | C | \|\| id:ebi-a-GCST90001497 | ebi-a-GCST90001497 | TRUE | T | 97963179 | 1.71E-09 | reported | 3437 | 0.02555 | rs7088458 |  |  |  |  |  |  |  |  |  |  |  |  |  |  |  |
| -0.1903 | 2 | igd | 0.4212 | A | \|\| id:ebi-a-GCST90001893 | ebi-a-GCST90001893 | TRUE | G | 204869970 | 8.23E-12 | reported | 2920 | 0.02774 | rs7597547 |  |  |  |  |  |  |  |  |  |  |  |  |  |  |  |
| 0.1491 | 20 | igd | 0.4206 | A | \|\| id:ebi-a-GCST90001798 | ebi-a-GCST90001798 | TRUE | G | 44875873 | 3.36E-09 | reported | 3657 | 0.02516 | rs2425799 |  |  |  |  |  |  |  |  |  |  |  |  |  |  |  |
| 0.1538 | 20 | igd | 0.4206 | A | \|\| id:ebi-a-GCST90001804 | ebi-a-GCST90001804 | TRUE | G | 44875873 | 8.89E-10 | reported | 3657 | 0.02504 | rs2425799 |  |  |  |  |  |  |  |  |  |  |  |  |  |  |  |
| 0.1422 | 20 | igd | 0.4206 | A | \|\| id:ebi-a-GCST90001808 | ebi-a-GCST90001808 | TRUE | G | 44875873 | 2.22E-08 | reported | 3657 | 0.02537 | rs2425799 |  |  |  |  |  |  |  |  |  |  |  |  |  |  |  |
| -0.1506 | 16 | igd | 0.4204 | G | \|\| id:ebi-a-GCST90001735 | ebi-a-GCST90001735 | TRUE | C | 28839735 | 1.92E-09 | reported | 3657 | 0.02502 | rs62036623 |  |  |  |  |  |  |  |  |  |  |  |  |  |  |  |
| -0.2438 | 14 | igd | 0.4199 | T | \|\| id:ebi-a-GCST90001820 | ebi-a-GCST90001820 | TRUE | C | 106875768 | 2.68E-08 | reported | 3656 | 0.04375 | rs11621990 |  |  |  |  |  |  |  |  |  |  |  |  |  |  |  |
| -0.2509 | 2 | igd | 0.4193 | A | \|\| id:ebi-a-GCST90002119 | ebi-a-GCST90002119 | TRUE | G | 87414736 | 4.66E-20 | reported | 2920 | 0.02716 | rs62145197 |  |  |  |  |  |  |  |  |  |  |  |  |  |  |  |
| 0.1376 | 7 | igd | 0.4186 | A | \|\| id:ebi-a-GCST90001821 | ebi-a-GCST90001821 | TRUE | G | 40235407 | 4.04E-08 | reported | 3656 | 0.02501 | rs7808529 |  |  |  |  |  |  |  |  |  |  |  |  |  |  |  |
| 0.1983 | 6 | igd | 0.4185 | A | \|\| id:ebi-a-GCST90002035 | ebi-a-GCST90002035 | TRUE | C | 91008027 | 1.64E-12 | reported | 2871 | 0.02796 | rs6925032 |  |  |  |  |  |  |  |  |  |  |  |  |  |  |  |
| 0.2178 | 6 | igd | 0.4185 | A | \|\| id:ebi-a-GCST90002036 | ebi-a-GCST90002036 | TRUE | C | 91008027 | 9.80E-15 | reported | 2871 | 0.02798 | rs6925032 |  |  |  |  |  |  |  |  |  |  |  |  |  |  |  |
| -0.4405 | 3 | igd | 0.4183 | G | \|\| id:ebi-a-GCST90002015 | ebi-a-GCST90002015 | TRUE | A | 46396938 | 1.45E-55 | reported | 2871 | 0.02745 | rs3092963 |  |  |  |  |  |  |  |  |  |  |  |  |  |  |  |
| -0.4395 | 3 | igd | 0.4183 | G | \|\| id:ebi-a-GCST90002016 | ebi-a-GCST90002016 | TRUE | A | 46396938 | 1.56E-55 | reported | 2871 | 0.0274 | rs3092963 |  |  |  |  |  |  |  |  |  |  |  |  |  |  |  |
| 0.1807 | 1 | igd | 0.4182 | G | \|\| id:ebi-a-GCST90001981 | ebi-a-GCST90001981 | TRUE | A | 161481743 | 1.16E-12 | reported | 3618 | 0.02532 | rs12142755 |  |  |  |  |  |  |  |  |  |  |  |  |  |  |  |
| 0.1734 | 6 | igd | 0.4182 | C | \|\| id:ebi-a-GCST90002118 | ebi-a-GCST90002118 | TRUE | T | 31542308 | 2.76E-08 | reported | 2920 | 0.03112 | rs1799964 |  |  |  |  |  |  |  |  |  |  |  |  |  |  |  |
| -0.1729 | 6 | igd | 0.4181 | A | \|\| id:ebi-a-GCST90001820 | ebi-a-GCST90001820 | TRUE | G | 31483458 | 2.12E-09 | reported | 3656 | 0.0288 | rs9267444 |  |  |  |  |  |  |  |  |  |  |  |  |  |  |  |
| -0.1511 | 1 | igd | 0.4172 | G | \|\| id:ebi-a-GCST90001996 | ebi-a-GCST90001996 | TRUE | A | 161481743 | 3.45E-09 | reported | 3579 | 0.02551 | rs12142755 |  |  |  |  |  |  |  |  |  |  |  |  |  |  |  |
| -0.1445 | 22 | igd | 0.4171 | G | \|\| id:ebi-a-GCST90001704 | ebi-a-GCST90001704 | TRUE | T | 40987730 | 1.46E-08 | reported | 3657 | 0.02544 | rs5758018 |  |  |  |  |  |  |  |  |  |  |  |  |  |  |  |
| -0.1544 | 1 | igd | 0.4157 | G | \|\| id:ebi-a-GCST90001845 | ebi-a-GCST90001845 | TRUE | A | 167317860 | 2.86E-08 | reported | 2911 | 0.02774 | rs4142888 |  |  |  |  |  |  |  |  |  |  |  |  |  |  |  |
| -0.1639 | 10 | igd | 0.4151 | T | \|\| id:ebi-a-GCST90001888 | ebi-a-GCST90001888 | TRUE | C | 98164060 | 2.56E-09 | reported | 2920 | 0.02743 | rs12764493 |  |  |  |  |  |  |  |  |  |  |  |  |  |  |  |
| -0.1736 | 1 | igd | 0.415 | G | \|\| id:ebi-a-GCST90001858 | ebi-a-GCST90001858 | TRUE | A | 167289363 | 3.71E-10 | reported | 2919 | 0.0276 | rs7538951 |  |  |  |  |  |  |  |  |  |  |  |  |  |  |  |
| -0.219 | 1 | igd | 0.4149 | G | \|\| id:ebi-a-GCST90001860 | ebi-a-GCST90001860 | TRUE | A | 167307677 | 3.39E-15 | reported | 2919 | 0.02765 | rs7519945 |  |  |  |  |  |  |  |  |  |  |  |  |  |  |  |
| -0.1671 | 1 | igd | 0.4149 | G | \|\| id:ebi-a-GCST90001864 | ebi-a-GCST90001864 | TRUE | A | 167307677 | 1.63E-09 | reported | 2919 | 0.02761 | rs7519945 |  |  |  |  |  |  |  |  |  |  |  |  |  |  |  |
| -0.1593 | 17 | igd | 0.4148 | T | \|\| id:ebi-a-GCST90001416 | ebi-a-GCST90001416 | TRUE | A | 38038391 | 2.61E-08 | reported | 3656 | 0.02856 | rs62067029 |  |  |  |  |  |  |  |  |  |  |  |  |  |  |  |
| -0.2192 | 1 | igd | 0.4147 | G | \|\| id:ebi-a-GCST90001854 | ebi-a-GCST90001854 | TRUE | T | 167301998 | 1.53E-15 | reported | 2919 | 0.02734 | rs10918683 |  |  |  |  |  |  |  |  |  |  |  |  |  |  |  |
| -0.22 | 1 | igd | 0.4147 | A | \|\| id:ebi-a-GCST90001854 | ebi-a-GCST90001854 | TRUE | T | 167195068 | 1.14E-15 | reported | 2919 | 0.02731 | rs10800293 |  |  |  |  |  |  |  |  |  |  |  |  |  |  |  |
| -0.1937 | 1 | igd | 0.4147 | C | \|\| id:ebi-a-GCST90001856 | ebi-a-GCST90001856 | TRUE | G | 167215996 | 3.00E-12 | reported | 2919 | 0.02764 | rs7418469 |  |  |  |  |  |  |  |  |  |  |  |  |  |  |  |
| -0.2194 | 1 | igd | 0.4147 | A | \|\| id:ebi-a-GCST90001860 | ebi-a-GCST90001860 | TRUE | T | 167195068 | 2.87E-15 | reported | 2919 | 0.02764 | rs10800293 |  |  |  |  |  |  |  |  |  |  |  |  |  |  |  |
| -0.2734 | 10 | igd | 0.4145 | C | \|\| id:ebi-a-GCST90001658 | ebi-a-GCST90001658 | TRUE | A | 97879634 | 7.52E-28 | reported | 3440 | 0.02478 | rs2125060 |  |  |  |  |  |  |  |  |  |  |  |  |  |  |  |
| -0.2763 | 10 | igd | 0.4145 | C | \|\| id:ebi-a-GCST90001659 | ebi-a-GCST90001659 | TRUE | A | 97879634 | 2.12E-28 | reported | 3440 | 0.02477 | rs2125060 |  |  |  |  |  |  |  |  |  |  |  |  |  |  |  |
| -0.2987 | 10 | igd | 0.4143 | C | \|\| id:ebi-a-GCST90001484 | ebi-a-GCST90001484 | TRUE | A | 97879634 | 3.42E-32 | reported | 3437 | 0.02503 | rs2125060 |  |  |  |  |  |  |  |  |  |  |  |  |  |  |  |
| -0.3073 | 10 | igd | 0.4143 | C | \|\| id:ebi-a-GCST90001485 | ebi-a-GCST90001485 | TRUE | A | 97879634 | 4.06E-34 | reported | 3437 | 0.02496 | rs2125060 |  |  |  |  |  |  |  |  |  |  |  |  |  |  |  |
| -0.3148 | 10 | igd | 0.4143 | C | \|\| id:ebi-a-GCST90001496 | ebi-a-GCST90001496 | TRUE | A | 97879634 | 2.39E-33 | reported | 3437 | 0.02588 | rs2125060 |  |  |  |  |  |  |  |  |  |  |  |  |  |  |  |
| -0.2997 | 10 | igd | 0.4143 | C | \|\| id:ebi-a-GCST90001497 | ebi-a-GCST90001497 | TRUE | A | 97879634 | 4.44E-31 | reported | 3437 | 0.0256 | rs2125060 |  |  |  |  |  |  |  |  |  |  |  |  |  |  |  |
| -0.2918 | 10 | igd | 0.4142 | C | \|\| id:ebi-a-GCST90001660 | ebi-a-GCST90001660 | TRUE | A | 97879634 | 9.09E-29 | reported | 3408 | 0.02598 | rs2125060 |  |  |  |  |  |  |  |  |  |  |  |  |  |  |  |
| -0.3803 | 10 | igd | 0.4142 | C | \|\| id:ebi-a-GCST90002030 | ebi-a-GCST90002030 | TRUE | A | 97879634 | 6.78E-43 | reported | 2920 | 0.02725 | rs2125060 |  |  |  |  |  |  |  |  |  |  |  |  |  |  |  |
| -0.3627 | 10 | igd | 0.4142 | C | \|\| id:ebi-a-GCST90002031 | ebi-a-GCST90002031 | TRUE | A | 97879634 | 1.39E-37 | reported | 2920 | 0.02791 | rs2125060 |  |  |  |  |  |  |  |  |  |  |  |  |  |  |  |
| -0.3417 | 10 | igd | 0.4142 | C | \|\| id:ebi-a-GCST90002032 | ebi-a-GCST90002032 | TRUE | A | 97879634 | 1.82E-33 | reported | 2920 | 0.02799 | rs2125060 |  |  |  |  |  |  |  |  |  |  |  |  |  |  |  |
| 0.1803 | 6 | igd | 0.4142 | G | \|\| id:ebi-a-GCST90002067 | ebi-a-GCST90002067 | TRUE | A | 30251314 | 2.06E-09 | reported | 2920 | 0.03 | rs1313929 |  |  |  |  |  |  |  |  |  |  |  |  |  |  |  |
| -0.3045 | 10 | igd | 0.414 | C | \|\| id:ebi-a-GCST90001483 | ebi-a-GCST90001483 | TRUE | A | 97879634 | 8.13E-33 | reported | 3405 | 0.02526 | rs2125060 |  |  |  |  |  |  |  |  |  |  |  |  |  |  |  |
| -0.2696 | 10 | igd | 0.414 | C | \|\| id:ebi-a-GCST90001489 | ebi-a-GCST90001489 | TRUE | A | 97879634 | 1.13E-24 | reported | 3405 | 0.02609 | rs2125060 |  |  |  |  |  |  |  |  |  |  |  |  |  |  |  |
| -0.3093 | 10 | igd | 0.414 | C | \|\| id:ebi-a-GCST90001495 | ebi-a-GCST90001495 | TRUE | A | 97879634 | 1.68E-31 | reported | 3405 | 0.02623 | rs2125060 |  |  |  |  |  |  |  |  |  |  |  |  |  |  |  |
| -0.1953 | 1 | igd | 0.414 | A | \|\| id:ebi-a-GCST90001856 | ebi-a-GCST90001856 | TRUE | G | 167326821 | 2.11E-12 | reported | 2919 | 0.02768 | rs7524664 |  |  |  |  |  |  |  |  |  |  |  |  |  |  |  |
| 0.19 | 1 | igd | 0.4118 | G | \|\| id:ebi-a-GCST90001854 | ebi-a-GCST90001854 | TRUE | A | 167451470 | 3.85E-12 | reported | 2919 | 0.02726 | rs1214605 |  |  |  |  |  |  |  |  |  |  |  |  |  |  |  |
| 0.1611 | 1 | igd | 0.4118 | G | \|\| id:ebi-a-GCST90001856 | ebi-a-GCST90001856 | TRUE | A | 167451470 | 5.83E-09 | reported | 2919 | 0.02758 | rs1214605 |  |  |  |  |  |  |  |  |  |  |  |  |  |  |  |
| 0.1863 | 1 | igd | 0.4118 | G | \|\| id:ebi-a-GCST90001860 | ebi-a-GCST90001860 | TRUE | A | 167451470 | 1.71E-11 | reported | 2919 | 0.02758 | rs1214605 |  |  |  |  |  |  |  |  |  |  |  |  |  |  |  |
| -0.2797 | 22 | igd | 0.4114 | C | \|\| id:ebi-a-GCST90001702 | ebi-a-GCST90001702 | TRUE | A | 41451185 | 1.50E-27 | reported | 3657 | 0.02551 | rs8138132 |  |  |  |  |  |  |  |  |  |  |  |  |  |  |  |
| -0.2746 | 22 | igd | 0.4114 | C | \|\| id:ebi-a-GCST90001703 | ebi-a-GCST90001703 | TRUE | A | 41451185 | 1.32E-26 | reported | 3657 | 0.02552 | rs8138132 |  |  |  |  |  |  |  |  |  |  |  |  |  |  |  |
| -0.2624 | 22 | igd | 0.4114 | C | \|\| id:ebi-a-GCST90001704 | ebi-a-GCST90001704 | TRUE | A | 41451185 | 1.75E-24 | reported | 3657 | 0.02551 | rs8138132 |  |  |  |  |  |  |  |  |  |  |  |  |  |  |  |
| -0.2734 | 22 | igd | 0.4114 | C | \|\| id:ebi-a-GCST90001710 | ebi-a-GCST90001710 | TRUE | A | 41451185 | 1.64E-26 | reported | 3657 | 0.02546 | rs8138132 |  |  |  |  |  |  |  |  |  |  |  |  |  |  |  |
| -0.247 | 22 | igd | 0.4114 | C | \|\| id:ebi-a-GCST90001711 | ebi-a-GCST90001711 | TRUE | A | 41451185 | 5.54E-22 | reported | 3657 | 0.02546 | rs8138132 |  |  |  |  |  |  |  |  |  |  |  |  |  |  |  |
| -0.1667 | 3 | igd | 0.4114 | T | \|\| id:ebi-a-GCST90002003 | ebi-a-GCST90002003 | TRUE | C | 46284558 | 4.26E-11 | reported | 3629 | 0.0252 | rs13081845 |  |  |  |  |  |  |  |  |  |  |  |  |  |  |  |
| -0.1526 | 7 | igd | 0.411 | A | \|\| id:ebi-a-GCST90002033 | ebi-a-GCST90002033 | TRUE | G | 77134132 | 4.55E-08 | reported | 2920 | 0.02783 | rs11766311 |  |  |  |  |  |  |  |  |  |  |  |  |  |  |  |
| -0.1713 | 1 | igd | 0.4106 | C | \|\| id:ebi-a-GCST90001980 | ebi-a-GCST90001980 | TRUE | G | 161451639 | 2.85E-11 | reported | 3629 | 0.02567 | rs9427064 |  |  |  |  |  |  |  |  |  |  |  |  |  |  |  |
| -0.1489 | 22 | igd | 0.4103 | T | \|\| id:ebi-a-GCST90001702 | ebi-a-GCST90001702 | TRUE | G | 41067010 | 6.93E-09 | reported | 3657 | 0.02564 | rs13055075 |  |  |  |  |  |  |  |  |  |  |  |  |  |  |  |
| -0.1494 | 22 | igd | 0.4103 | T | \|\| id:ebi-a-GCST90001703 | ebi-a-GCST90001703 | TRUE | G | 41067010 | 6.02E-09 | reported | 3657 | 0.02563 | rs13055075 |  |  |  |  |  |  |  |  |  |  |  |  |  |  |  |
| 0.1525 | 17 | igd | 0.4102 | T | \|\| id:ebi-a-GCST90001964 | ebi-a-GCST90001964 | TRUE | C | 8677215 | 3.66E-08 | reported | 2872 | 0.02762 | rs722926 |  |  |  |  |  |  |  |  |  |  |  |  |  |  |  |
| 0.1642 | 17 | igd | 0.4102 | T | \|\| id:ebi-a-GCST90002072 | ebi-a-GCST90002072 | TRUE | C | 8677215 | 3.49E-09 | reported | 2872 | 0.02772 | rs722926 |  |  |  |  |  |  |  |  |  |  |  |  |  |  |  |
| -0.1949 | 15 | igd | 0.4099 | T | \|\| id:ebi-a-GCST90001821 | ebi-a-GCST90001821 | TRUE | C | 40962298 | 7.17E-15 | reported | 3656 | 0.02494 | rs8036891 |  |  |  |  |  |  |  |  |  |  |  |  |  |  |  |
| 0.1711 | 6 | igd | 0.4086 | A | \|\| id:ebi-a-GCST90002067 | ebi-a-GCST90002067 | TRUE | G | 31463128 | 3.69E-08 | reported | 2920 | 0.03099 | rs9267352 |  |  |  |  |  |  |  |  |  |  |  |  |  |  |  |
| -0.2027 | 15 | igd | 0.408 | A | \|\| id:ebi-a-GCST90001821 | ebi-a-GCST90001821 | TRUE | G | 40856113 | 5.70E-16 | reported | 3656 | 0.02492 | rs28634695 |  |  |  |  |  |  |  |  |  |  |  |  |  |  |  |
| -0.1607 | 7 | igd | 0.4079 | T | \|\| id:ebi-a-GCST90001944 | ebi-a-GCST90001944 | TRUE | C | 50249013 | 1.80E-08 | reported | 2872 | 0.02845 | rs11761913 |  |  |  |  |  |  |  |  |  |  |  |  |  |  |  |
| -0.1601 | 7 | igd | 0.4079 | T | \|\| id:ebi-a-GCST90001945 | ebi-a-GCST90001945 | TRUE | C | 50249013 | 2.02E-08 | reported | 2872 | 0.02845 | rs11761913 |  |  |  |  |  |  |  |  |  |  |  |  |  |  |  |
| -0.165 | 7 | igd | 0.4079 | T | \|\| id:ebi-a-GCST90002037 | ebi-a-GCST90002037 | TRUE | C | 50249013 | 6.20E-09 | reported | 2871 | 0.02831 | rs11761913 |  |  |  |  |  |  |  |  |  |  |  |  |  |  |  |
| -0.1639 | 7 | igd | 0.4079 | T | \|\| id:ebi-a-GCST90002038 | ebi-a-GCST90002038 | TRUE | C | 50249013 | 7.84E-09 | reported | 2871 | 0.02831 | rs11761913 |  |  |  |  |  |  |  |  |  |  |  |  |  |  |  |
| 0.3431 | 6 | igd | 0.4072 | A | \|\| id:ebi-a-GCST90002108 | ebi-a-GCST90002108 | TRUE | G | 31457344 | 5.94E-16 | reported | 1579 | 0.04196 | rs3130913 |  |  |  |  |  |  |  |  |  |  |  |  |  |  |  |
| 0.2381 | 6 | igd | 0.4068 | A | \|\| id:ebi-a-GCST90002109 | ebi-a-GCST90002109 | TRUE | G | 31457344 | 1.85E-08 | reported | 1578 | 0.04211 | rs3130913 |  |  |  |  |  |  |  |  |  |  |  |  |  |  |  |
| 0.3285 | 6 | igd | 0.4061 | A | \|\| id:ebi-a-GCST90002112 | ebi-a-GCST90002112 | TRUE | G | 31457344 | 6.56E-15 | reported | 1635 | 0.04175 | rs3130913 |  |  |  |  |  |  |  |  |  |  |  |  |  |  |  |
| 0.1738 | 6 | igd | 0.4053 | C | \|\| id:ebi-a-GCST90002105 | ebi-a-GCST90002105 | TRUE | A | 33024654 | 5.26E-09 | reported | 2872 | 0.02968 | rs3135402 |  |  |  |  |  |  |  |  |  |  |  |  |  |  |  |
| 0.1412 | 22 | igd | 0.4047 | T | \|\| id:ebi-a-GCST90001704 | ebi-a-GCST90001704 | TRUE | C | 43159810 | 4.18E-08 | reported | 3657 | 0.02569 | rs5996232 |  |  |  |  |  |  |  |  |  |  |  |  |  |  |  |
| 0.1414 | 22 | igd | 0.4047 | T | \|\| id:ebi-a-GCST90001711 | ebi-a-GCST90001711 | TRUE | C | 43159810 | 3.54E-08 | reported | 3657 | 0.0256 | rs5996232 |  |  |  |  |  |  |  |  |  |  |  |  |  |  |  |
| 0.3774 | 6 | igd | 0.4044 | A | \|\| id:ebi-a-GCST90002105 | ebi-a-GCST90002105 | TRUE | G | 31457344 | 5.98E-31 | reported | 2872 | 0.03224 | rs3130913 |  |  |  |  |  |  |  |  |  |  |  |  |  |  |  |
| -0.2217 | 10 | igd | 0.4041 | C | \|\| id:ebi-a-GCST90001483 | ebi-a-GCST90001483 | TRUE | T | 97071060 | 7.80E-18 | reported | 3405 | 0.02563 | rs1360005 |  |  |  |  |  |  |  |  |  |  |  |  |  |  |  |
| -0.2497 | 10 | igd | 0.4041 | C | \|\| id:ebi-a-GCST90001489 | ebi-a-GCST90001489 | TRUE | T | 97071060 | 3.64E-21 | reported | 3405 | 0.02627 | rs1360005 |  |  |  |  |  |  |  |  |  |  |  |  |  |  |  |
| -0.268 | 10 | igd | 0.4041 | C | \|\| id:ebi-a-GCST90001495 | ebi-a-GCST90001495 | TRUE | T | 97071060 | 1.16E-23 | reported | 3405 | 0.02653 | rs1360005 |  |  |  |  |  |  |  |  |  |  |  |  |  |  |  |
| -0.274 | 10 | igd | 0.404 | C | \|\| id:ebi-a-GCST90001660 | ebi-a-GCST90001660 | TRUE | T | 97071060 | 3.38E-25 | reported | 3408 | 0.02621 | rs1360005 |  |  |  |  |  |  |  |  |  |  |  |  |  |  |  |
| -0.2573 | 10 | igd | 0.404 | C | \|\| id:ebi-a-GCST90001672 | ebi-a-GCST90001672 | TRUE | T | 97071060 | 5.77E-22 | reported | 3408 | 0.02652 | rs1360005 |  |  |  |  |  |  |  |  |  |  |  |  |  |  |  |
| -0.25 | 10 | igd | 0.4037 | C | \|\| id:ebi-a-GCST90001484 | ebi-a-GCST90001484 | TRUE | T | 97071060 | 1.12E-22 | reported | 3437 | 0.02533 | rs1360005 |  |  |  |  |  |  |  |  |  |  |  |  |  |  |  |
| -0.2348 | 10 | igd | 0.4037 | C | \|\| id:ebi-a-GCST90001485 | ebi-a-GCST90001485 | TRUE | T | 97071060 | 3.28E-20 | reported | 3437 | 0.02533 | rs1360005 |  |  |  |  |  |  |  |  |  |  |  |  |  |  |  |
| -0.2659 | 10 | igd | 0.4037 | C | \|\| id:ebi-a-GCST90001491 | ebi-a-GCST90001491 | TRUE | T | 97071060 | 2.12E-24 | reported | 3437 | 0.02589 | rs1360005 |  |  |  |  |  |  |  |  |  |  |  |  |  |  |  |
| -0.2976 | 10 | igd | 0.4037 | C | \|\| id:ebi-a-GCST90001496 | ebi-a-GCST90001496 | TRUE | T | 97071060 | 1.72E-29 | reported | 3437 | 0.02615 | rs1360005 |  |  |  |  |  |  |  |  |  |  |  |  |  |  |  |
| -0.2833 | 10 | igd | 0.4037 | C | \|\| id:ebi-a-GCST90001497 | ebi-a-GCST90001497 | TRUE | T | 97071060 | 1.69E-27 | reported | 3437 | 0.02585 | rs1360005 |  |  |  |  |  |  |  |  |  |  |  |  |  |  |  |
| -0.2745 | 10 | igd | 0.4036 | C | \|\| id:ebi-a-GCST90001658 | ebi-a-GCST90001658 | TRUE | T | 97071060 | 1.30E-27 | reported | 3440 | 0.02499 | rs1360005 |  |  |  |  |  |  |  |  |  |  |  |  |  |  |  |
| -0.2805 | 10 | igd | 0.4036 | C | \|\| id:ebi-a-GCST90001659 | ebi-a-GCST90001659 | TRUE | T | 97071060 | 8.88E-29 | reported | 3440 | 0.02497 | rs1360005 |  |  |  |  |  |  |  |  |  |  |  |  |  |  |  |
| -0.2551 | 10 | igd | 0.4036 | C | \|\| id:ebi-a-GCST90001670 | ebi-a-GCST90001670 | TRUE | T | 97071060 | 2.50E-22 | reported | 3440 | 0.02607 | rs1360005 |  |  |  |  |  |  |  |  |  |  |  |  |  |  |  |
| -0.2564 | 10 | igd | 0.4036 | C | \|\| id:ebi-a-GCST90001671 | ebi-a-GCST90001671 | TRUE | T | 97071060 | 2.00E-22 | reported | 3440 | 0.02613 | rs1360005 |  |  |  |  |  |  |  |  |  |  |  |  |  |  |  |
| 0.1676 | 6 | igd | 0.4026 | C | \|\| id:ebi-a-GCST90002118 | ebi-a-GCST90002118 | TRUE | T | 29717380 | 4.06E-08 | reported | 2920 | 0.03046 | rs12665401 |  |  |  |  |  |  |  |  |  |  |  |  |  |  |  |
| -0.4102 | 6 | igd | 0.4025 | G | \|\| id:ebi-a-GCST90002105 | ebi-a-GCST90002105 | TRUE | C | 32192107 | 4.16E-40 | reported | 2872 | 0.03047 | rs3134929 |  |  |  |  |  |  |  |  |  |  |  |  |  |  |  |
| 0.1578 | 2 | igd | 0.4021 | C | \|\| id:ebi-a-GCST90002119 | ebi-a-GCST90002119 | TRUE | T | 87515293 | 2.17E-08 | reported | 2920 | 0.02811 | rs13423009 |  |  |  |  |  |  |  |  |  |  |  |  |  |  |  |
| -0.2979 | 10 | igd | 0.4019 | C | \|\| id:ebi-a-GCST90002030 | ebi-a-GCST90002030 | TRUE | T | 97071060 | 7.10E-27 | reported | 2920 | 0.02748 | rs1360005 |  |  |  |  |  |  |  |  |  |  |  |  |  |  |  |
| -0.2705 | 10 | igd | 0.4019 | C | \|\| id:ebi-a-GCST90002031 | ebi-a-GCST90002031 | TRUE | T | 97071060 | 1.52E-21 | reported | 2920 | 0.02815 | rs1360005 |  |  |  |  |  |  |  |  |  |  |  |  |  |  |  |
| -0.2573 | 10 | igd | 0.4019 | C | \|\| id:ebi-a-GCST90002032 | ebi-a-GCST90002032 | TRUE | T | 97071060 | 1.24E-19 | reported | 2920 | 0.02819 | rs1360005 |  |  |  |  |  |  |  |  |  |  |  |  |  |  |  |
| 0.1645 | 6 | igd | 0.4013 | G | \|\| id:ebi-a-GCST90002105 | ebi-a-GCST90002105 | TRUE | T | 33330131 | 2.07E-08 | reported | 2872 | 0.02926 | rs211453 |  |  |  |  |  |  |  |  |  |  |  |  |  |  |  |
| 0.183 | 6 | igd | 0.401 | T | \|\| id:ebi-a-GCST90001743 | ebi-a-GCST90001743 | TRUE | C | 32561370 | 3.55E-10 | reported | 3660 | 0.02909 | rs35139284 |  |  |  |  |  |  |  |  |  |  |  |  |  |  |  |
| -0.3 | 6 | igd | 0.4006 | C | \|\| id:ebi-a-GCST90002108 | ebi-a-GCST90002108 | TRUE | T | 31247871 | 8.28E-13 | reported | 1579 | 0.04157 | rs2853943 |  |  |  |  |  |  |  |  |  |  |  |  |  |  |  |
| 0.3901 | 22 | igd | 0.3995 | C | \|\| id:ebi-a-GCST90001711 | ebi-a-GCST90001711 | TRUE | G | 42360833 | 1.67E-53 | reported | 3657 | 0.02492 | rs4822072 |  |  |  |  |  |  |  |  |  |  |  |  |  |  |  |
| -0.1924 | 22 | igd | 0.3994 | G | \|\| id:ebi-a-GCST90001702 | ebi-a-GCST90001702 | TRUE | A | 44374267 | 5.11E-14 | reported | 3657 | 0.02545 | rs5764048 |  |  |  |  |  |  |  |  |  |  |  |  |  |  |  |
| -0.1882 | 22 | igd | 0.3994 | G | \|\| id:ebi-a-GCST90001703 | ebi-a-GCST90001703 | TRUE | A | 44374267 | 1.76E-13 | reported | 3657 | 0.02545 | rs5764048 |  |  |  |  |  |  |  |  |  |  |  |  |  |  |  |
| -0.1836 | 22 | igd | 0.3994 | G | \|\| id:ebi-a-GCST90001704 | ebi-a-GCST90001704 | TRUE | A | 44374267 | 6.32E-13 | reported | 3657 | 0.02543 | rs5764048 |  |  |  |  |  |  |  |  |  |  |  |  |  |  |  |
| -0.1885 | 22 | igd | 0.3994 | G | \|\| id:ebi-a-GCST90001710 | ebi-a-GCST90001710 | TRUE | A | 44374267 | 1.41E-13 | reported | 3657 | 0.02539 | rs5764048 |  |  |  |  |  |  |  |  |  |  |  |  |  |  |  |
| -0.1818 | 22 | igd | 0.3994 | G | \|\| id:ebi-a-GCST90001711 | ebi-a-GCST90001711 | TRUE | A | 44374267 | 8.72E-13 | reported | 3657 | 0.02534 | rs5764048 |  |  |  |  |  |  |  |  |  |  |  |  |  |  |  |
| 0.161975 | 10 | igd | 0.399329 | G | \|\| id:met-b-156 | met-b-156 | TRUE | A | 97729647 | 3.82E-16 | reported | 447 | 0.0198887 | rs17382898 |  |  |  |  |  |  |  |  |  |  |  |  |  |  |  |
| -0.3002 | 6 | igd | 0.3993 | G | \|\| id:ebi-a-GCST90002110 | ebi-a-GCST90002110 | TRUE | C | 32192107 | 2.96E-14 | reported | 1634 | 0.03915 | rs3134929 |  |  |  |  |  |  |  |  |  |  |  |  |  |  |  |
| -0.2266 | 6 | igd | 0.3992 | C | \|\| id:ebi-a-GCST90002105 | ebi-a-GCST90002105 | TRUE | T | 31247871 | 1.58E-11 | reported | 2872 | 0.03348 | rs2853943 |  |  |  |  |  |  |  |  |  |  |  |  |  |  |  |
| 0.1444 | 22 | igd | 0.3991 | G | \|\| id:ebi-a-GCST90001704 | ebi-a-GCST90001704 | TRUE | A | 39006934 | 1.98E-08 | reported | 3657 | 0.02566 | rs5757176 |  |  |  |  |  |  |  |  |  |  |  |  |  |  |  |
| -0.2726 | 6 | igd | 0.3991 | G | \|\| id:ebi-a-GCST90002111 | ebi-a-GCST90002111 | TRUE | C | 32192107 | 2.44E-12 | reported | 1635 | 0.03861 | rs3134929 |  |  |  |  |  |  |  |  |  |  |  |  |  |  |  |
| -0.3326 | 6 | igd | 0.3991 | G | \|\| id:ebi-a-GCST90002112 | ebi-a-GCST90002112 | TRUE | C | 32192107 | 2.24E-17 | reported | 1635 | 0.03878 | rs3134929 |  |  |  |  |  |  |  |  |  |  |  |  |  |  |  |
| 0.2319 | 2 | igd | 0.398 | C | \|\| id:ebi-a-GCST90002071 | ebi-a-GCST90002071 | TRUE | T | 203441224 | 1.63E-15 | reported | 2872 | 0.02895 | rs6435157 |  |  |  |  |  |  |  |  |  |  |  |  |  |  |  |
| -0.2289 | 6 | igd | 0.398 | G | \|\| id:ebi-a-GCST90002109 | ebi-a-GCST90002109 | TRUE | C | 32192107 | 6.11E-09 | reported | 1578 | 0.03916 | rs3134929 |  |  |  |  |  |  |  |  |  |  |  |  |  |  |  |
| -0.2476 | 6 | igd | 0.3979 | C | \|\| id:ebi-a-GCST90002111 | ebi-a-GCST90002111 | TRUE | T | 31503698 | 1.16E-09 | reported | 1635 | 0.04045 | rs929138 |  |  |  |  |  |  |  |  |  |  |  |  |  |  |  |
| -0.3016 | 6 | igd | 0.3979 | C | \|\| id:ebi-a-GCST90002112 | ebi-a-GCST90002112 | TRUE | T | 31503698 | 2.02E-13 | reported | 1635 | 0.04071 | rs929138 |  |  |  |  |  |  |  |  |  |  |  |  |  |  |  |
| -0.3247 | 6 | igd | 0.3974 | C | \|\| id:ebi-a-GCST90002108 | ebi-a-GCST90002108 | TRUE | T | 31503698 | 3.31E-15 | reported | 1579 | 0.04081 | rs929138 |  |  |  |  |  |  |  |  |  |  |  |  |  |  |  |
| -0.246 | 6 | igd | 0.3973 | C | \|\| id:ebi-a-GCST90002109 | ebi-a-GCST90002109 | TRUE | T | 31503698 | 2.00E-09 | reported | 1578 | 0.04078 | rs929138 |  |  |  |  |  |  |  |  |  |  |  |  |  |  |  |
| 0.1806 | 3 | igd | 0.3944 | A | \|\| id:ebi-a-GCST90002017 | ebi-a-GCST90002017 | TRUE | G | 45995118 | 4.02E-10 | reported | 2850 | 0.02877 | rs1552490 |  |  |  |  |  |  |  |  |  |  |  |  |  |  |  |
| 0.2571 | 2 | igd | 0.3922 | A | \|\| id:ebi-a-GCST90001963 | ebi-a-GCST90001963 | TRUE | G | 203226371 | 2.51E-19 | reported | 2872 | 0.0284 | rs72925015 |  |  |  |  |  |  |  |  |  |  |  |  |  |  |  |
| 0.257 | 2 | igd | 0.3922 | G | \|\| id:ebi-a-GCST90001963 | ebi-a-GCST90001963 | TRUE | C | 203357700 | 2.62E-19 | reported | 2872 | 0.0284 | rs72925086 |  |  |  |  |  |  |  |  |  |  |  |  |  |  |  |
| 0.2275 | 2 | igd | 0.3922 | A | \|\| id:ebi-a-GCST90002071 | ebi-a-GCST90002071 | TRUE | G | 203226371 | 5.11E-15 | reported | 2872 | 0.02892 | rs72925015 |  |  |  |  |  |  |  |  |  |  |  |  |  |  |  |
| 0.1554 | 7 | igd | 0.3917 | G | \|\| id:ebi-a-GCST90001821 | ebi-a-GCST90001821 | TRUE | A | 40094641 | 6.86E-10 | reported | 3656 | 0.02512 | rs9639820 |  |  |  |  |  |  |  |  |  |  |  |  |  |  |  |
| 0.2272 | 2 | igd | 0.3915 | C | \|\| id:ebi-a-GCST90002071 | ebi-a-GCST90002071 | TRUE | T | 203328919 | 6.32E-15 | reported | 2872 | 0.02898 | rs45442097 |  |  |  |  |  |  |  |  |  |  |  |  |  |  |  |
| 0.2383 | 22 | igd | 0.3913 | A | \|\| id:ebi-a-GCST90001704 | ebi-a-GCST90001704 | TRUE | T | 42704682 | 2.67E-20 | reported | 3657 | 0.02566 | rs763000 |  |  |  |  |  |  |  |  |  |  |  |  |  |  |  |
| 0.2301 | 22 | igd | 0.3913 | A | \|\| id:ebi-a-GCST90001710 | ebi-a-GCST90001710 | TRUE | T | 42704682 | 4.75E-19 | reported | 3657 | 0.02566 | rs763000 |  |  |  |  |  |  |  |  |  |  |  |  |  |  |  |
| 0.2532 | 22 | igd | 0.3912 | A | \|\| id:ebi-a-GCST90001702 | ebi-a-GCST90001702 | TRUE | G | 42704834 | 1.13E-22 | reported | 3657 | 0.02567 | rs4822107 |  |  |  |  |  |  |  |  |  |  |  |  |  |  |  |
| 0.2542 | 22 | igd | 0.3912 | A | \|\| id:ebi-a-GCST90001703 | ebi-a-GCST90001703 | TRUE | G | 42704834 | 7.45E-23 | reported | 3657 | 0.02566 | rs4822107 |  |  |  |  |  |  |  |  |  |  |  |  |  |  |  |
| 0.2309 | 22 | igd | 0.3912 | A | \|\| id:ebi-a-GCST90001711 | ebi-a-GCST90001711 | TRUE | G | 42704834 | 2.90E-19 | reported | 3657 | 0.02559 | rs4822107 |  |  |  |  |  |  |  |  |  |  |  |  |  |  |  |
| -0.1627 | 22 | igd | 0.3906 | T | \|\| id:ebi-a-GCST90001704 | ebi-a-GCST90001704 | TRUE | C | 41096164 | 4.96E-10 | reported | 3657 | 0.02608 | rs133085 |  |  |  |  |  |  |  |  |  |  |  |  |  |  |  |
| -0.1536 | 22 | igd | 0.3906 | T | \|\| id:ebi-a-GCST90001710 | ebi-a-GCST90001710 | TRUE | C | 41096164 | 4.19E-09 | reported | 3657 | 0.02607 | rs133085 |  |  |  |  |  |  |  |  |  |  |  |  |  |  |  |
| -0.1673 | 1 | igd | 0.39 | C | \|\| id:ebi-a-GCST90001858 | ebi-a-GCST90001858 | TRUE | T | 167393128 | 1.87E-09 | reported | 2919 | 0.02776 | rs4233416 |  |  |  |  |  |  |  |  |  |  |  |  |  |  |  |
| 0.1544 | 7 | igd | 0.3899 | T | \|\| id:ebi-a-GCST90001821 | ebi-a-GCST90001821 | TRUE | C | 39984741 | 8.49E-10 | reported | 3656 | 0.02511 | rs9986997 |  |  |  |  |  |  |  |  |  |  |  |  |  |  |  |
| 0.2419 | 6 | igd | 0.3895 | T | \|\| id:ebi-a-GCST90002108 | ebi-a-GCST90002108 | TRUE | C | 32797620 | 1.72E-09 | reported | 1579 | 0.03994 | rs241438 |  |  |  |  |  |  |  |  |  |  |  |  |  |  |  |
| -0.1558 | 9 | igd | 0.3892 | G | \|\| id:ebi-a-GCST90001748 | ebi-a-GCST90001748 | TRUE | A | 37002142 | 8.43E-10 | reported | 3645 | 0.02531 | rs12554596 |  |  |  |  |  |  |  |  |  |  |  |  |  |  |  |
| -0.197 | 6 | igd | 0.3889 | G | \|\| id:ebi-a-GCST90002121 | ebi-a-GCST90002121 | TRUE | C | 29905628 | 1.38E-10 | reported | 2915 | 0.03058 | rs13190729 |  |  |  |  |  |  |  |  |  |  |  |  |  |  |  |
| 0.1696 | 6 | igd | 0.3888 | C | \|\| id:ebi-a-GCST90001743 | ebi-a-GCST90001743 | TRUE | T | 32585068 | 2.69E-08 | reported | 3660 | 0.03043 | rs28752534 |  |  |  |  |  |  |  |  |  |  |  |  |  |  |  |
| 0.1513 | 10 | igd | 0.3886 | A | \|\| id:ebi-a-GCST90001484 | ebi-a-GCST90001484 | TRUE | G | 93070877 | 1.91E-09 | reported | 3437 | 0.02512 | rs2648723 |  |  |  |  |  |  |  |  |  |  |  |  |  |  |  |
| 0.1604 | 10 | igd | 0.3886 | A | \|\| id:ebi-a-GCST90001496 | ebi-a-GCST90001496 | TRUE | G | 93070877 | 7.09E-10 | reported | 3437 | 0.02595 | rs2648723 |  |  |  |  |  |  |  |  |  |  |  |  |  |  |  |
| 0.1541 | 10 | igd | 0.3886 | A | \|\| id:ebi-a-GCST90001497 | ebi-a-GCST90001497 | TRUE | G | 93070877 | 2.01E-09 | reported | 3437 | 0.02564 | rs2648723 |  |  |  |  |  |  |  |  |  |  |  |  |  |  |  |
| 0.178 | 6 | igd | 0.3886 | C | \|\| id:ebi-a-GCST90001762 | ebi-a-GCST90001762 | TRUE | T | 32585068 | 5.69E-09 | reported | 3658 | 0.03047 | rs28752534 |  |  |  |  |  |  |  |  |  |  |  |  |  |  |  |
| 0.1646 | 1 | igd | 0.3885 | C | \|\| id:ebi-a-GCST90002014 | ebi-a-GCST90002014 | TRUE | T | 161622861 | 4.82E-08 | reported | 2870 | 0.03008 | rs61804163 |  |  |  |  |  |  |  |  |  |  |  |  |  |  |  |
| 0.2079 | 6 | igd | 0.3884 | C | \|\| id:ebi-a-GCST90001752 | ebi-a-GCST90001752 | TRUE | T | 32585068 | 9.57E-12 | reported | 3657 | 0.03042 | rs28752534 |  |  |  |  |  |  |  |  |  |  |  |  |  |  |  |
| 0.1721 | 6 | igd | 0.3884 | C | \|\| id:ebi-a-GCST90001757 | ebi-a-GCST90001757 | TRUE | T | 32585068 | 2.18E-08 | reported | 3657 | 0.03067 | rs28752534 |  |  |  |  |  |  |  |  |  |  |  |  |  |  |  |
| 0.2117 | 6 | igd | 0.3878 | G | \|\| id:ebi-a-GCST90002067 | ebi-a-GCST90002067 | TRUE | C | 31255953 | 1.17E-11 | reported | 2920 | 0.03109 | rs2524049 |  |  |  |  |  |  |  |  |  |  |  |  |  |  |  |
| 0.1427 | 19 | igd | 0.3872 | G | \|\| id:ebi-a-GCST90001755 | ebi-a-GCST90001755 | TRUE | A | 49451217 | 2.00E-08 | reported | 3657 | 0.02537 | rs4802530 |  |  |  |  |  |  |  |  |  |  |  |  |  |  |  |
| 0.1784 | 19 | igd | 0.3872 | G | \|\| id:ebi-a-GCST90001757 | ebi-a-GCST90001757 | TRUE | A | 49451217 | 1.69E-12 | reported | 3657 | 0.02519 | rs4802530 |  |  |  |  |  |  |  |  |  |  |  |  |  |  |  |
| 0.173 | 19 | igd | 0.3872 | G | \|\| id:ebi-a-GCST90001761 | ebi-a-GCST90001761 | TRUE | A | 49451217 | 1.11E-11 | reported | 3657 | 0.0254 | rs4802530 |  |  |  |  |  |  |  |  |  |  |  |  |  |  |  |
| -0.3327 | 6 | igd | 0.3865 | C | \|\| id:ebi-a-GCST90002105 | ebi-a-GCST90002105 | TRUE | T | 31503698 | 3.92E-24 | reported | 2872 | 0.03253 | rs929138 |  |  |  |  |  |  |  |  |  |  |  |  |  |  |  |
| -0.139 | 20 | igd | 0.3858 | G | \|\| id:ebi-a-GCST90001798 | ebi-a-GCST90001798 | TRUE | C | 46840158 | 4.17E-08 | reported | 3657 | 0.02529 | rs852370 |  |  |  |  |  |  |  |  |  |  |  |  |  |  |  |
| -0.1483 | 20 | igd | 0.3858 | G | \|\| id:ebi-a-GCST90001808 | ebi-a-GCST90001808 | TRUE | C | 46840158 | 6.41E-09 | reported | 3657 | 0.02548 | rs852370 |  |  |  |  |  |  |  |  |  |  |  |  |  |  |  |
| 0.161 | 10 | igd | 0.3854 | C | \|\| id:ebi-a-GCST90001941 | ebi-a-GCST90001941 | TRUE | G | 6074451 | 6.04E-10 | reported | 3435 | 0.02594 | rs4747846 |  |  |  |  |  |  |  |  |  |  |  |  |  |  |  |
| 0.1439 | 10 | igd | 0.3854 | C | \|\| id:ebi-a-GCST90001942 | ebi-a-GCST90001942 | TRUE | G | 6074451 | 2.64E-08 | reported | 3435 | 0.0258 | rs4747846 |  |  |  |  |  |  |  |  |  |  |  |  |  |  |  |
| 0.3052 | 10 | igd | 0.3854 | C | \|\| id:ebi-a-GCST90002030 | ebi-a-GCST90002030 | TRUE | T | 96998688 | 1.54E-27 | reported | 2920 | 0.02778 | rs12778797 |  |  |  |  |  |  |  |  |  |  |  |  |  |  |  |
| 0.2849 | 10 | igd | 0.3854 | C | \|\| id:ebi-a-GCST90002031 | ebi-a-GCST90002031 | TRUE | T | 96998688 | 2.24E-23 | reported | 2920 | 0.02836 | rs12778797 |  |  |  |  |  |  |  |  |  |  |  |  |  |  |  |
| 0.1839 | 10 | igd | 0.3851 | A | \|\| id:ebi-a-GCST90002029 | ebi-a-GCST90002029 | TRUE | G | 96998591 | 3.22E-11 | reported | 2915 | 0.02761 | rs2901833 |  |  |  |  |  |  |  |  |  |  |  |  |  |  |  |
| 0.2703 | 10 | igd | 0.3848 | A | \|\| id:ebi-a-GCST90002032 | ebi-a-GCST90002032 | TRUE | G | 96998591 | 3.43E-21 | reported | 2920 | 0.02839 | rs2901833 |  |  |  |  |  |  |  |  |  |  |  |  |  |  |  |
| 0.165 | 6 | igd | 0.3846 | T | \|\| id:ebi-a-GCST90001752 | ebi-a-GCST90001752 | TRUE | C | 32587013 | 3.52E-09 | reported | 3657 | 0.02788 | rs557011 |  |  |  |  |  |  |  |  |  |  |  |  |  |  |  |
| 0.2941 | 10 | igd | 0.3844 | G | \|\| id:ebi-a-GCST90001961 | ebi-a-GCST90001961 | TRUE | A | 98002000 | 7.08E-26 | reported | 2920 | 0.02769 | rs4918998 |  |  |  |  |  |  |  |  |  |  |  |  |  |  |  |
| -0.4186 | 10 | igd | 0.3844 | G | \|\| id:ebi-a-GCST90002030 | ebi-a-GCST90002030 | TRUE | A | 98002000 | 1.21E-52 | reported | 2920 | 0.02686 | rs4918998 |  |  |  |  |  |  |  |  |  |  |  |  |  |  |  |
| -0.3611 | 10 | igd | 0.3844 | G | \|\| id:ebi-a-GCST90002031 | ebi-a-GCST90002031 | TRUE | A | 98002000 | 1.17E-37 | reported | 2920 | 0.02775 | rs4918998 |  |  |  |  |  |  |  |  |  |  |  |  |  |  |  |
| -0.3287 | 10 | igd | 0.3844 | G | \|\| id:ebi-a-GCST90002032 | ebi-a-GCST90002032 | TRUE | A | 98002000 | 2.26E-31 | reported | 2920 | 0.02788 | rs4918998 |  |  |  |  |  |  |  |  |  |  |  |  |  |  |  |
| 0.1557 | 2 | igd | 0.3843 | C | \|\| id:ebi-a-GCST90001485 | ebi-a-GCST90001485 | TRUE | T | 38910595 | 1.23E-09 | reported | 3437 | 0.02556 | rs1469594 |  |  |  |  |  |  |  |  |  |  |  |  |  |  |  |
| 0.2113 | 2 | igd | 0.3843 | C | \|\| id:ebi-a-GCST90001508 | ebi-a-GCST90001508 | TRUE | T | 38910595 | 1.20E-15 | reported | 3437 | 0.02627 | rs1469594 |  |  |  |  |  |  |  |  |  |  |  |  |  |  |  |
| 0.1838 | 2 | igd | 0.3843 | C | \|\| id:ebi-a-GCST90001509 | ebi-a-GCST90001509 | TRUE | T | 38910595 | 3.40E-12 | reported | 3437 | 0.02632 | rs1469594 |  |  |  |  |  |  |  |  |  |  |  |  |  |  |  |
| 0.1568 | 2 | igd | 0.384 | C | \|\| id:ebi-a-GCST90001483 | ebi-a-GCST90001483 | TRUE | T | 38910595 | 1.28E-09 | reported | 3405 | 0.02576 | rs1469594 |  |  |  |  |  |  |  |  |  |  |  |  |  |  |  |
| -0.2149 | 10 | igd | 0.384 | G | \|\| id:ebi-a-GCST90002029 | ebi-a-GCST90002029 | TRUE | A | 98002000 | 7.33E-15 | reported | 2915 | 0.02748 | rs4918998 |  |  |  |  |  |  |  |  |  |  |  |  |  |  |  |
| -0.3006 | 19 | igd | 0.38365 | T | \|\| id:prot-a-439 | prot-a-439 | TRUE | A | 51867170 | 2.75E-29 | reported | 3301 | 0.0268 | rs4802791 |  |  |  |  |  |  |  |  |  |  |  |  |  |  |  |
| 0.1764 | 6 | igd | 0.3826 | T | \|\| id:ebi-a-GCST90001762 | ebi-a-GCST90001762 | TRUE | C | 32582601 | 8.06E-09 | reported | 3658 | 0.03051 | rs34656207 |  |  |  |  |  |  |  |  |  |  |  |  |  |  |  |
| -0.1683 | 16 | igd | 0.3825 | G | \|\| id:ebi-a-GCST90002089 | ebi-a-GCST90002089 | TRUE | T | 48503912 | 3.73E-09 | reported | 2850 | 0.02845 | rs9932817 |  |  |  |  |  |  |  |  |  |  |  |  |  |  |  |
| 0.1449 | 10 | igd | 0.3811 | G | \|\| id:ebi-a-GCST90001658 | ebi-a-GCST90001658 | TRUE | A | 97268195 | 8.85E-09 | reported | 3440 | 0.02514 | rs4917692 |  |  |  |  |  |  |  |  |  |  |  |  |  |  |  |
| 0.1435 | 10 | igd | 0.3811 | G | \|\| id:ebi-a-GCST90001659 | ebi-a-GCST90001659 | TRUE | A | 97268195 | 1.24E-08 | reported | 3440 | 0.02515 | rs4917692 |  |  |  |  |  |  |  |  |  |  |  |  |  |  |  |
| -0.2896 | 10 | igd | 0.3808 | G | \|\| id:ebi-a-GCST90001489 | ebi-a-GCST90001489 | TRUE | A | 98002000 | 3.21E-28 | reported | 3405 | 0.02606 | rs4918998 |  |  |  |  |  |  |  |  |  |  |  |  |  |  |  |
| -0.3399 | 10 | igd | 0.3808 | G | \|\| id:ebi-a-GCST90001495 | ebi-a-GCST90001495 | TRUE | A | 98002000 | 8.97E-38 | reported | 3405 | 0.02614 | rs4918998 |  |  |  |  |  |  |  |  |  |  |  |  |  |  |  |
| -0.284 | 10 | igd | 0.3806 | G | \|\| id:ebi-a-GCST90001484 | ebi-a-GCST90001484 | TRUE | A | 98002000 | 4.24E-29 | reported | 3437 | 0.02513 | rs4918998 |  |  |  |  |  |  |  |  |  |  |  |  |  |  |  |
| -0.297 | 10 | igd | 0.3806 | G | \|\| id:ebi-a-GCST90001491 | ebi-a-GCST90001491 | TRUE | A | 98002000 | 1.59E-30 | reported | 3437 | 0.02561 | rs4918998 |  |  |  |  |  |  |  |  |  |  |  |  |  |  |  |
| -0.3518 | 10 | igd | 0.3806 | G | \|\| id:ebi-a-GCST90001496 | ebi-a-GCST90001496 | TRUE | A | 98002000 | 2.33E-41 | reported | 3437 | 0.02577 | rs4918998 |  |  |  |  |  |  |  |  |  |  |  |  |  |  |  |
| -0.3552 | 10 | igd | 0.3806 | G | \|\| id:ebi-a-GCST90001497 | ebi-a-GCST90001497 | TRUE | A | 98002000 | 3.36E-43 | reported | 3437 | 0.02542 | rs4918998 |  |  |  |  |  |  |  |  |  |  |  |  |  |  |  |
| -0.3615 | 10 | igd | 0.3806 | G | \|\| id:ebi-a-GCST90001660 | ebi-a-GCST90001660 | TRUE | A | 98002000 | 1.37E-43 | reported | 3408 | 0.02574 | rs4918998 |  |  |  |  |  |  |  |  |  |  |  |  |  |  |  |
| -0.3832 | 10 | igd | 0.3806 | G | \|\| id:ebi-a-GCST90001672 | ebi-a-GCST90001672 | TRUE | A | 98002000 | 5.43E-48 | reported | 3408 | 0.02592 | rs4918998 |  |  |  |  |  |  |  |  |  |  |  |  |  |  |  |
| -0.3533 | 10 | igd | 0.3804 | G | \|\| id:ebi-a-GCST90001658 | ebi-a-GCST90001658 | TRUE | A | 98002000 | 8.91E-46 | reported | 3440 | 0.02451 | rs4918998 |  |  |  |  |  |  |  |  |  |  |  |  |  |  |  |
| -0.3558 | 10 | igd | 0.3804 | G | \|\| id:ebi-a-GCST90001659 | ebi-a-GCST90001659 | TRUE | A | 98002000 | 2.09E-46 | reported | 3440 | 0.02451 | rs4918998 |  |  |  |  |  |  |  |  |  |  |  |  |  |  |  |
| -0.3729 | 10 | igd | 0.3804 | G | \|\| id:ebi-a-GCST90001670 | ebi-a-GCST90001670 | TRUE | A | 98002000 | 4.31E-47 | reported | 3440 | 0.02548 | rs4918998 |  |  |  |  |  |  |  |  |  |  |  |  |  |  |  |
| -0.3659 | 10 | igd | 0.3804 | G | \|\| id:ebi-a-GCST90001671 | ebi-a-GCST90001671 | TRUE | A | 98002000 | 3.34E-45 | reported | 3440 | 0.02556 | rs4918998 |  |  |  |  |  |  |  |  |  |  |  |  |  |  |  |
| -0.1749 | 6 | igd | 0.3801 | T | \|\| id:ebi-a-GCST90001820 | ebi-a-GCST90001820 | TRUE | A | 32451760 | 4.13E-08 | reported | 3656 | 0.03181 | rs9269232 |  |  |  |  |  |  |  |  |  |  |  |  |  |  |  |
| 0.2288 | 10 | igd | 0.3798 | C | \|\| id:ebi-a-GCST90001781 | ebi-a-GCST90001781 | TRUE | T | 6106552 | 1.14E-10 | reported | 1835 | 0.03529 | rs7073236 |  |  |  |  |  |  |  |  |  |  |  |  |  |  |  |
| 0.1987 | 10 | igd | 0.3798 | A | \|\| id:ebi-a-GCST90001892 | ebi-a-GCST90001892 | TRUE | C | 97979916 | 7.24E-13 | reported | 2920 | 0.02756 | rs35030481 |  |  |  |  |  |  |  |  |  |  |  |  |  |  |  |
| -0.1854 | 6 | igd | 0.3789 | T | \|\| id:ebi-a-GCST90002067 | ebi-a-GCST90002067 | TRUE | C | 32191620 | 8.86E-10 | reported | 2920 | 0.03015 | rs3134930 |  |  |  |  |  |  |  |  |  |  |  |  |  |  |  |
| 0.2154 | 6 | igd | 0.3788 | T | \|\| id:ebi-a-GCST90002105 | ebi-a-GCST90002105 | TRUE | C | 32191620 | 9.81E-12 | reported | 2872 | 0.03151 | rs3134930 |  |  |  |  |  |  |  |  |  |  |  |  |  |  |  |
| -0.1475 | 16 | igd | 0.3768 | T | \|\| id:ebi-a-GCST90001723 | ebi-a-GCST90001723 | TRUE | A | 28631530 | 7.89E-09 | reported | 3656 | 0.0255 | rs62031607 |  |  |  |  |  |  |  |  |  |  |  |  |  |  |  |
| -0.1541 | 16 | igd | 0.3768 | T | \|\| id:ebi-a-GCST90001735 | ebi-a-GCST90001735 | TRUE | A | 28631530 | 1.51E-09 | reported | 3657 | 0.02543 | rs62031607 |  |  |  |  |  |  |  |  |  |  |  |  |  |  |  |
| -0.1536 | 16 | igd | 0.3768 | T | \|\| id:ebi-a-GCST90001740 | ebi-a-GCST90001740 | TRUE | A | 28631530 | 2.04E-09 | reported | 3657 | 0.02557 | rs62031607 |  |  |  |  |  |  |  |  |  |  |  |  |  |  |  |
| 0.3001 | 6 | igd | 0.3768 | C | \|\| id:ebi-a-GCST90002108 | ebi-a-GCST90002108 | TRUE | T | 32190620 | 7.66E-14 | reported | 1579 | 0.03978 | rs3134931 |  |  |  |  |  |  |  |  |  |  |  |  |  |  |  |
| 0.2005 | 10 | igd | 0.3765 | T | \|\| id:ebi-a-GCST90002030 | ebi-a-GCST90002030 | TRUE | C | 97319637 | 2.17E-12 | reported | 2920 | 0.02842 | rs10882620 |  |  |  |  |  |  |  |  |  |  |  |  |  |  |  |
| -0.2069 | 1 | igd | 0.3765 | A | \|\| id:ebi-a-GCST90002077 | ebi-a-GCST90002077 | TRUE | G | 161244164 | 2.40E-13 | reported | 2971 | 0.02812 | rs4657001 |  |  |  |  |  |  |  |  |  |  |  |  |  |  |  |
| -0.1695 | 6 | igd | 0.3764 | G | \|\| id:ebi-a-GCST90001932 | ebi-a-GCST90001932 | TRUE | A | 32339076 | 1.81E-08 | reported | 2920 | 0.03002 | rs2050190 |  |  |  |  |  |  |  |  |  |  |  |  |  |  |  |
| 0.2028 | 10 | igd | 0.3763 | T | \|\| id:ebi-a-GCST90001658 | ebi-a-GCST90001658 | TRUE | C | 97319637 | 2.67E-15 | reported | 3440 | 0.02553 | rs10882620 |  |  |  |  |  |  |  |  |  |  |  |  |  |  |  |
| 0.1975 | 10 | igd | 0.3763 | T | \|\| id:ebi-a-GCST90001659 | ebi-a-GCST90001659 | TRUE | C | 97319637 | 1.40E-14 | reported | 3440 | 0.02555 | rs10882620 |  |  |  |  |  |  |  |  |  |  |  |  |  |  |  |
| 0.2361 | 10 | igd | 0.3763 | T | \|\| id:ebi-a-GCST90001670 | ebi-a-GCST90001670 | TRUE | C | 97319637 | 9.06E-19 | reported | 3440 | 0.02653 | rs10882620 |  |  |  |  |  |  |  |  |  |  |  |  |  |  |  |
| 0.2334 | 10 | igd | 0.3763 | T | \|\| id:ebi-a-GCST90001671 | ebi-a-GCST90001671 | TRUE | C | 97319637 | 2.37E-18 | reported | 3440 | 0.02656 | rs10882620 |  |  |  |  |  |  |  |  |  |  |  |  |  |  |  |
| 0.1925 | 10 | igd | 0.376 | T | \|\| id:ebi-a-GCST90001660 | ebi-a-GCST90001660 | TRUE | C | 97319637 | 9.13E-13 | reported | 3408 | 0.02685 | rs10882620 |  |  |  |  |  |  |  |  |  |  |  |  |  |  |  |
| 0.2307 | 10 | igd | 0.376 | T | \|\| id:ebi-a-GCST90001672 | ebi-a-GCST90001672 | TRUE | C | 97319637 | 2.11E-17 | reported | 3408 | 0.02703 | rs10882620 |  |  |  |  |  |  |  |  |  |  |  |  |  |  |  |
| 0.2137 | 10 | igd | 0.3759 | T | \|\| id:ebi-a-GCST90001491 | ebi-a-GCST90001491 | TRUE | C | 97319637 | 8.88E-16 | reported | 3437 | 0.02644 | rs10882620 |  |  |  |  |  |  |  |  |  |  |  |  |  |  |  |
| 0.196 | 10 | igd | 0.3759 | T | \|\| id:ebi-a-GCST90001496 | ebi-a-GCST90001496 | TRUE | C | 97319637 | 3.49E-13 | reported | 3437 | 0.02685 | rs10882620 |  |  |  |  |  |  |  |  |  |  |  |  |  |  |  |
| 0.2035 | 10 | igd | 0.3759 | T | \|\| id:ebi-a-GCST90001497 | ebi-a-GCST90001497 | TRUE | C | 97319637 | 2.02E-14 | reported | 3437 | 0.02649 | rs10882620 |  |  |  |  |  |  |  |  |  |  |  |  |  |  |  |
| 0.1591 | 2 | igd | 0.3759 | A | \|\| id:ebi-a-GCST90002119 | ebi-a-GCST90002119 | TRUE | G | 86534521 | 1.08E-08 | reported | 2920 | 0.02774 | rs13024436 |  |  |  |  |  |  |  |  |  |  |  |  |  |  |  |
| 0.2032 | 10 | igd | 0.3756 | T | \|\| id:ebi-a-GCST90001489 | ebi-a-GCST90001489 | TRUE | C | 97319637 | 5.95E-14 | reported | 3405 | 0.02695 | rs10882620 |  |  |  |  |  |  |  |  |  |  |  |  |  |  |  |
| 0.196 | 10 | igd | 0.3756 | T | \|\| id:ebi-a-GCST90001495 | ebi-a-GCST90001495 | TRUE | C | 97319637 | 6.83E-13 | reported | 3405 | 0.02718 | rs10882620 |  |  |  |  |  |  |  |  |  |  |  |  |  |  |  |
| -0.1681 | 6 | igd | 0.3747 | C | \|\| id:ebi-a-GCST90001765 | ebi-a-GCST90001765 | TRUE | T | 107298061 | 3.98E-11 | reported | 3655 | 0.02538 | rs272583 |  |  |  |  |  |  |  |  |  |  |  |  |  |  |  |
| -0.2647 | 22 | igd | 0.3739 | C | \|\| id:ebi-a-GCST90001702 | ebi-a-GCST90001702 | TRUE | G | 42060917 | 4.98E-25 | reported | 3657 | 0.02543 | rs28384790 |  |  |  |  |  |  |  |  |  |  |  |  |  |  |  |
| -0.2688 | 22 | igd | 0.3739 | C | \|\| id:ebi-a-GCST90001703 | ebi-a-GCST90001703 | TRUE | G | 42060917 | 8.77E-26 | reported | 3657 | 0.02541 | rs28384790 |  |  |  |  |  |  |  |  |  |  |  |  |  |  |  |
| 0.141 | 22 | igd | 0.3739 | C | \|\| id:ebi-a-GCST90001704 | ebi-a-GCST90001704 | TRUE | T | 44276105 | 3.62E-08 | reported | 3657 | 0.02554 | rs138129 |  |  |  |  |  |  |  |  |  |  |  |  |  |  |  |
| -0.2607 | 22 | igd | 0.3739 | C | \|\| id:ebi-a-GCST90001704 | ebi-a-GCST90001704 | TRUE | G | 42060917 | 2.24E-24 | reported | 3657 | 0.0254 | rs28384790 |  |  |  |  |  |  |  |  |  |  |  |  |  |  |  |
| -0.2427 | 22 | igd | 0.3739 | C | \|\| id:ebi-a-GCST90001710 | ebi-a-GCST90001710 | TRUE | G | 42060917 | 2.27E-21 | reported | 3657 | 0.02541 | rs28384790 |  |  |  |  |  |  |  |  |  |  |  |  |  |  |  |
| -0.2435 | 22 | igd | 0.3739 | C | \|\| id:ebi-a-GCST90001711 | ebi-a-GCST90001711 | TRUE | G | 42060917 | 1.36E-21 | reported | 3657 | 0.02535 | rs28384790 |  |  |  |  |  |  |  |  |  |  |  |  |  |  |  |
| 0.43 | 22 | igd | 0.3738 | C | \|\| id:ebi-a-GCST90001702 | ebi-a-GCST90001702 | TRUE | A | 42354389 | 3.58E-62 | reported | 3657 | 0.02535 | rs62240996 |  |  |  |  |  |  |  |  |  |  |  |  |  |  |  |
| 0.4233 | 22 | igd | 0.3738 | C | \|\| id:ebi-a-GCST90001703 | ebi-a-GCST90001703 | TRUE | A | 42354389 | 2.72E-60 | reported | 3657 | 0.02537 | rs62240996 |  |  |  |  |  |  |  |  |  |  |  |  |  |  |  |
| 0.4092 | 22 | igd | 0.3738 | C | \|\| id:ebi-a-GCST90001704 | ebi-a-GCST90001704 | TRUE | A | 42354389 | 1.75E-56 | reported | 3657 | 0.0254 | rs62240996 |  |  |  |  |  |  |  |  |  |  |  |  |  |  |  |
| 0.3968 | 22 | igd | 0.3738 | C | \|\| id:ebi-a-GCST90001710 | ebi-a-GCST90001710 | TRUE | A | 42354389 | 3.39E-53 | reported | 3657 | 0.02543 | rs62240996 |  |  |  |  |  |  |  |  |  |  |  |  |  |  |  |
| 0.184 | 10 | igd | 0.3718 | C | \|\| id:ebi-a-GCST90001786 | ebi-a-GCST90001786 | TRUE | T | 6106266 | 3.68E-13 | reported | 3651 | 0.02523 | rs7072793 |  |  |  |  |  |  |  |  |  |  |  |  |  |  |  |
| 0.1894 | 10 | igd | 0.3716 | C | \|\| id:ebi-a-GCST90001785 | ebi-a-GCST90001785 | TRUE | T | 6106266 | 1.10E-13 | reported | 3649 | 0.0254 | rs7072793 |  |  |  |  |  |  |  |  |  |  |  |  |  |  |  |
| -0.2757 | 3 | igd | 0.3712 | G | \|\| id:ebi-a-GCST90002017 | ebi-a-GCST90002017 | TRUE | A | 46339373 | 6.13E-21 | reported | 2850 | 0.02914 | rs6782423 |  |  |  |  |  |  |  |  |  |  |  |  |  |  |  |
| 0.1504 | 22 | igd | 0.3711 | C | \|\| id:ebi-a-GCST90001701 | ebi-a-GCST90001701 | TRUE | T | 42356886 | 3.48E-09 | reported | 3592 | 0.02539 | rs6002560 |  |  |  |  |  |  |  |  |  |  |  |  |  |  |  |
| 0.197 | 2 | igd | 0.371 | C | \|\| id:ebi-a-GCST90001560 | ebi-a-GCST90001560 | TRUE | G | 38925155 | 3.31E-18 | reported | 3395 | 0.02252 | rs11896593 |  |  |  |  |  |  |  |  |  |  |  |  |  |  |  |
| 0.2701 | 2 | igd | 0.3707 | C | \|\| id:ebi-a-GCST90001561 | ebi-a-GCST90001561 | TRUE | G | 38925155 | 1.04E-33 | reported | 3427 | 0.02208 | rs11896593 |  |  |  |  |  |  |  |  |  |  |  |  |  |  |  |
| 0.1051 | 2 | igd | 0.3706 | C | \|\| id:ebi-a-GCST90001690 | ebi-a-GCST90001690 | TRUE | G | 38925155 | 1.17E-08 | reported | 3408 | 0.01838 | rs11896593 |  |  |  |  |  |  |  |  |  |  |  |  |  |  |  |
| 0.1553 | 3 | igd | 0.3705 | T | \|\| id:ebi-a-GCST90002004 | ebi-a-GCST90002004 | TRUE | C | 46130097 | 2.53E-09 | reported | 3629 | 0.02599 | rs4683178 |  |  |  |  |  |  |  |  |  |  |  |  |  |  |  |
| 0.1535 | 3 | igd | 0.3705 | T | \|\| id:ebi-a-GCST90002008 | ebi-a-GCST90002008 | TRUE | C | 46130097 | 5.18E-09 | reported | 3629 | 0.02621 | rs4683178 |  |  |  |  |  |  |  |  |  |  |  |  |  |  |  |
| -0.1513 | 6 | igd | 0.37 | A | \|\| id:ebi-a-GCST90001771 | ebi-a-GCST90001771 | TRUE | G | 107298159 | 3.47E-09 | reported | 3653 | 0.02555 | rs272582 |  |  |  |  |  |  |  |  |  |  |  |  |  |  |  |
| -0.1497 | 6 | igd | 0.37 | A | \|\| id:ebi-a-GCST90001772 | ebi-a-GCST90001772 | TRUE | G | 107298159 | 5.64E-09 | reported | 3655 | 0.02563 | rs272582 |  |  |  |  |  |  |  |  |  |  |  |  |  |  |  |
| -0.2466 | 22 | igd | 0.3698 | C | \|\| id:ebi-a-GCST90001702 | ebi-a-GCST90001702 | TRUE | G | 42873630 | 4.62E-21 | reported | 3657 | 0.02602 | rs35998301 |  |  |  |  |  |  |  |  |  |  |  |  |  |  |  |
| -0.2412 | 22 | igd | 0.3698 | C | \|\| id:ebi-a-GCST90001703 | ebi-a-GCST90001703 | TRUE | G | 42873630 | 3.22E-20 | reported | 3657 | 0.02603 | rs35998301 |  |  |  |  |  |  |  |  |  |  |  |  |  |  |  |
| -0.2156 | 22 | igd | 0.3698 | C | \|\| id:ebi-a-GCST90001704 | ebi-a-GCST90001704 | TRUE | G | 42873630 | 1.80E-16 | reported | 3657 | 0.02606 | rs35998301 |  |  |  |  |  |  |  |  |  |  |  |  |  |  |  |
| -0.246 | 22 | igd | 0.3698 | C | \|\| id:ebi-a-GCST90001710 | ebi-a-GCST90001710 | TRUE | G | 42873630 | 4.40E-21 | reported | 3657 | 0.02595 | rs35998301 |  |  |  |  |  |  |  |  |  |  |  |  |  |  |  |
| -0.2465 | 22 | igd | 0.3698 | C | \|\| id:ebi-a-GCST90001711 | ebi-a-GCST90001711 | TRUE | G | 42873630 | 2.94E-21 | reported | 3657 | 0.02589 | rs35998301 |  |  |  |  |  |  |  |  |  |  |  |  |  |  |  |
| -0.1616 | 7 | igd | 0.3697 | G | \|\| id:ebi-a-GCST90002015 | ebi-a-GCST90002015 | TRUE | A | 1145396 | 3.36E-08 | reported | 2871 | 0.02919 | rs4585652 |  |  |  |  |  |  |  |  |  |  |  |  |  |  |  |
| -0.1613 | 7 | igd | 0.3697 | G | \|\| id:ebi-a-GCST90002016 | ebi-a-GCST90002016 | TRUE | A | 1145396 | 3.41E-08 | reported | 2871 | 0.02914 | rs4585652 |  |  |  |  |  |  |  |  |  |  |  |  |  |  |  |
| 0.1977 | 6 | igd | 0.3685 | A | \|\| id:ebi-a-GCST90002067 | ebi-a-GCST90002067 | TRUE | G | 30933076 | 4.12E-10 | reported | 2920 | 0.03152 | rs2532923 |  |  |  |  |  |  |  |  |  |  |  |  |  |  |  |
| -0.1583 | 6 | igd | 0.368 | C | \|\| id:ebi-a-GCST90001896 | ebi-a-GCST90001896 | TRUE | T | 91028797 | 3.69E-08 | reported | 2920 | 0.02869 | rs285640 |  |  |  |  |  |  |  |  |  |  |  |  |  |  |  |
| 0.1849 | 3 | igd | 0.3672 | A | \|\| id:ebi-a-GCST90002017 | ebi-a-GCST90002017 | TRUE | G | 46155872 | 4.78E-10 | reported | 2850 | 0.0296 | rs13077923 |  |  |  |  |  |  |  |  |  |  |  |  |  |  |  |
| -0.145 | 1 | igd | 0.3672 | C | \|\| id:ebi-a-GCST90001999 | ebi-a-GCST90001999 | TRUE | T | 161937517 | 2.77E-08 | reported | 3621 | 0.02605 | rs2490437 |  |  |  |  |  |  |  |  |  |  |  |  |  |  |  |
| -0.2095 | 6 | igd | 0.3657 | A | \|\| id:ebi-a-GCST90001767 | ebi-a-GCST90001767 | TRUE | G | 107298159 | 4.15E-09 | reported | 1832 | 0.03546 | rs272582 |  |  |  |  |  |  |  |  |  |  |  |  |  |  |  |
| -0.1749 | 3 | igd | 0.3647 | G | \|\| id:ebi-a-GCST90002008 | ebi-a-GCST90002008 | TRUE | A | 46339373 | 1.35E-11 | reported | 3629 | 0.02578 | rs6782423 |  |  |  |  |  |  |  |  |  |  |  |  |  |  |  |
| 0.2874 | 1 | igd | 0.3642 | G | \|\| id:ebi-a-GCST90001832 | ebi-a-GCST90001832 | TRUE | T | 169572947 | 6.82E-26 | reported | 2872 | 0.02705 | rs3917768 |  |  |  |  |  |  |  |  |  |  |  |  |  |  |  |
| 0.1865 | 6 | igd | 0.3641 | C | \|\| id:ebi-a-GCST90001684 | ebi-a-GCST90001684 | TRUE | T | 32443266 | 4.71E-09 | reported | 3371 | 0.03176 | rs9269109 |  |  |  |  |  |  |  |  |  |  |  |  |  |  |  |
| -0.194 | 3 | igd | 0.3641 | A | \|\| id:ebi-a-GCST90002004 | ebi-a-GCST90002004 | TRUE | G | 46339223 | 3.38E-14 | reported | 3629 | 0.02548 | rs6441957 |  |  |  |  |  |  |  |  |  |  |  |  |  |  |  |
| -0.1767 | 6 | igd | 0.3633 | T | \|\| id:ebi-a-GCST90001910 | ebi-a-GCST90001910 | TRUE | C | 31279326 | 4.37E-08 | reported | 3112 | 0.0322 | rs1005249 |  |  |  |  |  |  |  |  |  |  |  |  |  |  |  |
| 0.1897 | 6 | igd | 0.3627 | C | \|\| id:ebi-a-GCST90001687 | ebi-a-GCST90001687 | TRUE | T | 32443266 | 6.01E-10 | reported | 3408 | 0.03055 | rs9269109 |  |  |  |  |  |  |  |  |  |  |  |  |  |  |  |
| 0.161 | 19 | igd | 0.3627 | C | \|\| id:ebi-a-GCST90001753 | ebi-a-GCST90001753 | TRUE | T | 49888009 | 3.09E-10 | reported | 3657 | 0.02551 | rs7254187 |  |  |  |  |  |  |  |  |  |  |  |  |  |  |  |
| 0.1556 | 19 | igd | 0.3627 | C | \|\| id:ebi-a-GCST90001754 | ebi-a-GCST90001754 | TRUE | T | 49888009 | 1.04E-09 | reported | 3657 | 0.02542 | rs7254187 |  |  |  |  |  |  |  |  |  |  |  |  |  |  |  |
| -0.1638 | 4 | igd | 0.3625 | A | \|\| id:ebi-a-GCST90001471 | ebi-a-GCST90001471 | TRUE | G | 84066027 | 2.83E-10 | reported | 3410 | 0.02589 | rs11736172 |  |  |  |  |  |  |  |  |  |  |  |  |  |  |  |
| -0.1816 | 10 | igd | 0.3621 | A | \|\| id:ebi-a-GCST90001670 | ebi-a-GCST90001670 | TRUE | G | 96446632 | 4.40E-11 | reported | 3440 | 0.02748 | rs1010570 |  |  |  |  |  |  |  |  |  |  |  |  |  |  |  |
| -0.1641 | 10 | igd | 0.3621 | A | \|\| id:ebi-a-GCST90001671 | ebi-a-GCST90001671 | TRUE | G | 96446632 | 3.05E-09 | reported | 3440 | 0.0276 | rs1010570 |  |  |  |  |  |  |  |  |  |  |  |  |  |  |  |
| -0.1549 | 4 | igd | 0.3619 | A | \|\| id:ebi-a-GCST90001470 | ebi-a-GCST90001470 | TRUE | G | 84066027 | 1.29E-09 | reported | 3374 | 0.02545 | rs11736172 |  |  |  |  |  |  |  |  |  |  |  |  |  |  |  |
| -0.1745 | 10 | igd | 0.3619 | A | \|\| id:ebi-a-GCST90001672 | ebi-a-GCST90001672 | TRUE | G | 96446632 | 5.14E-10 | reported | 3408 | 0.028 | rs1010570 |  |  |  |  |  |  |  |  |  |  |  |  |  |  |  |
| -0.3637 | 22 | igd | 0.3604 | G | \|\| id:ebi-a-GCST90001702 | ebi-a-GCST90001702 | TRUE | A | 42208672 | 4.16E-43 | reported | 3657 | 0.02608 | rs6002503 |  |  |  |  |  |  |  |  |  |  |  |  |  |  |  |
| -0.3558 | 22 | igd | 0.3604 | G | \|\| id:ebi-a-GCST90001703 | ebi-a-GCST90001703 | TRUE | A | 42208672 | 2.60E-41 | reported | 3657 | 0.0261 | rs6002503 |  |  |  |  |  |  |  |  |  |  |  |  |  |  |  |
| -0.3704 | 22 | igd | 0.3604 | G | \|\| id:ebi-a-GCST90001704 | ebi-a-GCST90001704 | TRUE | A | 42208672 | 7.89E-45 | reported | 3657 | 0.02601 | rs6002503 |  |  |  |  |  |  |  |  |  |  |  |  |  |  |  |
| -0.3565 | 22 | igd | 0.3604 | G | \|\| id:ebi-a-GCST90001710 | ebi-a-GCST90001710 | TRUE | A | 42208672 | 1.17E-41 | reported | 3657 | 0.02603 | rs6002503 |  |  |  |  |  |  |  |  |  |  |  |  |  |  |  |
| -0.347 | 22 | igd | 0.3604 | G | \|\| id:ebi-a-GCST90001711 | ebi-a-GCST90001711 | TRUE | A | 42208672 | 1.03E-39 | reported | 3657 | 0.026 | rs6002503 |  |  |  |  |  |  |  |  |  |  |  |  |  |  |  |
| 0.1789 | 6 | igd | 0.3604 | A | \|\| id:ebi-a-GCST90002067 | ebi-a-GCST90002067 | TRUE | G | 32213008 | 1.79E-08 | reported | 2920 | 0.03168 | rs454875 |  |  |  |  |  |  |  |  |  |  |  |  |  |  |  |
| 0.1597 | 6 | igd | 0.3593 | C | \|\| id:ebi-a-GCST90001678 | ebi-a-GCST90001678 | TRUE | T | 32443266 | 1.04E-08 | reported | 3130 | 0.02782 | rs9269109 |  |  |  |  |  |  |  |  |  |  |  |  |  |  |  |
| 0.1546 | 22 | igd | 0.3593 | A | \|\| id:ebi-a-GCST90001702 | ebi-a-GCST90001702 | TRUE | G | 38007812 | 2.94E-09 | reported | 3657 | 0.02599 | rs6000845 |  |  |  |  |  |  |  |  |  |  |  |  |  |  |  |
| 0.1492 | 22 | igd | 0.3593 | A | \|\| id:ebi-a-GCST90001703 | ebi-a-GCST90001703 | TRUE | G | 38007812 | 1.02E-08 | reported | 3657 | 0.02599 | rs6000845 |  |  |  |  |  |  |  |  |  |  |  |  |  |  |  |
| 0.1429 | 22 | igd | 0.3581 | A | \|\| id:ebi-a-GCST90001704 | ebi-a-GCST90001704 | TRUE | G | 44323074 | 4.12E-08 | reported | 3657 | 0.026 | rs139047 |  |  |  |  |  |  |  |  |  |  |  |  |  |  |  |
| 0.2102 | 1 | igd | 0.3561 | T | \|\| id:ebi-a-GCST90001970 | ebi-a-GCST90001970 | TRUE | C | 161612340 | 2.50E-13 | reported | 2971 | 0.02859 | rs408849 |  |  |  |  |  |  |  |  |  |  |  |  |  |  |  |
| 0.2157 | 1 | igd | 0.3561 | T | \|\| id:ebi-a-GCST90002077 | ebi-a-GCST90002077 | TRUE | C | 161612340 | 3.49E-14 | reported | 2971 | 0.02832 | rs408849 |  |  |  |  |  |  |  |  |  |  |  |  |  |  |  |
| 0.2118 | 6 | igd | 0.356 | G | \|\| id:ebi-a-GCST90002105 | ebi-a-GCST90002105 | TRUE | A | 33432709 | 1.45E-12 | reported | 2872 | 0.02978 | rs4711325 |  |  |  |  |  |  |  |  |  |  |  |  |  |  |  |
| -0.1488 | 22 | igd | 0.3553 | C | \|\| id:ebi-a-GCST90001702 | ebi-a-GCST90001702 | TRUE | T | 44323955 | 1.26E-08 | reported | 3657 | 0.02609 | rs738407 |  |  |  |  |  |  |  |  |  |  |  |  |  |  |  |
| -0.1434 | 22 | igd | 0.3553 | C | \|\| id:ebi-a-GCST90001710 | ebi-a-GCST90001710 | TRUE | T | 44323955 | 3.85E-08 | reported | 3657 | 0.02603 | rs738407 |  |  |  |  |  |  |  |  |  |  |  |  |  |  |  |
| -0.165 | 18 | igd | 0.3544 | A | \|\| id:ebi-a-GCST90002034 | ebi-a-GCST90002034 | TRUE | G | 46483810 | 1.86E-08 | reported | 2909 | 0.02926 | rs2337147 |  |  |  |  |  |  |  |  |  |  |  |  |  |  |  |
| 0.1549 | 2 | igd | 0.3541 | C | \|\| id:ebi-a-GCST90002098 | ebi-a-GCST90002098 | TRUE | T | 39807473 | 1.18E-08 | reported | 2910 | 0.02709 | rs149218800 |  |  |  |  |  |  |  |  |  |  |  |  |  |  |  |
| 0.2172 | 1 | igd | 0.3538 | T | \|\| id:ebi-a-GCST90001474 | ebi-a-GCST90001474 | TRUE | C | 161612340 | 5.03E-16 | reported | 3410 | 0.02664 | rs408849 |  |  |  |  |  |  |  |  |  |  |  |  |  |  |  |
| -0.1924 | 6 | igd | 0.3533 | G | \|\| id:ebi-a-GCST90002120 | ebi-a-GCST90002120 | TRUE | A | 29905782 | 2.17E-09 | reported | 2920 | 0.03205 | rs28894074 |  |  |  |  |  |  |  |  |  |  |  |  |  |  |  |
| 20.08 | 2 | igd | 0.3526 | G | \|\| id:ebi-a-GCST90001695 | ebi-a-GCST90001695 | TRUE | A | 38887964 | 1.18E-12 | reported | 3408 | 2.814 | rs58905133 |  |  |  |  |  |  |  |  |  |  |  |  |  |  |  |
| 3.541 | 2 | igd | 0.3523 | G | \|\| id:ebi-a-GCST90001696 | ebi-a-GCST90001696 | TRUE | A | 38887964 | 2.91E-28 | reported | 3425 | 0.3183 | rs58905133 |  |  |  |  |  |  |  |  |  |  |  |  |  |  |  |
| 1.152 | 2 | igd | 0.3523 | G | \|\| id:ebi-a-GCST90001697 | ebi-a-GCST90001697 | TRUE | A | 38887964 | 2.08E-18 | reported | 3425 | 0.1309 | rs58905133 |  |  |  |  |  |  |  |  |  |  |  |  |  |  |  |
| -0.1469 | 10 | igd | 0.3512 | T | \|\| id:ebi-a-GCST90001985 | ebi-a-GCST90001985 | TRUE | A | 123688152 | 2.60E-08 | reported | 3629 | 0.02633 | rs10887024 |  |  |  |  |  |  |  |  |  |  |  |  |  |  |  |
| 0.2092 | 1 | igd | 0.3509 | T | \|\| id:ebi-a-GCST90001999 | ebi-a-GCST90001999 | TRUE | C | 161612340 | 6.99E-15 | reported | 3621 | 0.02676 | rs408849 |  |  |  |  |  |  |  |  |  |  |  |  |  |  |  |
| -2.745 | 2 | igd | 0.3508 | G | \|\| id:ebi-a-GCST90001699 | ebi-a-GCST90001699 | TRUE | A | 38892207 | 1.96E-13 | reported | 3425 | 0.372 | rs7559619 |  |  |  |  |  |  |  |  |  |  |  |  |  |  |  |
| -0.2094 | 22 | igd | 0.3507 | C | \|\| id:ebi-a-GCST90001702 | ebi-a-GCST90001702 | TRUE | T | 39938303 | 9.38E-16 | reported | 3657 | 0.02594 | rs5757710 |  |  |  |  |  |  |  |  |  |  |  |  |  |  |  |
| -0.2009 | 22 | igd | 0.3507 | C | \|\| id:ebi-a-GCST90001703 | ebi-a-GCST90001703 | TRUE | T | 39938303 | 1.28E-14 | reported | 3657 | 0.02596 | rs5757710 |  |  |  |  |  |  |  |  |  |  |  |  |  |  |  |
| -0.2001 | 22 | igd | 0.3507 | C | \|\| id:ebi-a-GCST90001704 | ebi-a-GCST90001704 | TRUE | T | 39938303 | 1.48E-14 | reported | 3657 | 0.02592 | rs5757710 |  |  |  |  |  |  |  |  |  |  |  |  |  |  |  |
| -0.214 | 22 | igd | 0.3507 | C | \|\| id:ebi-a-GCST90001710 | ebi-a-GCST90001710 | TRUE | T | 39938303 | 1.78E-16 | reported | 3657 | 0.02586 | rs5757710 |  |  |  |  |  |  |  |  |  |  |  |  |  |  |  |
| -0.2043 | 22 | igd | 0.3507 | C | \|\| id:ebi-a-GCST90001711 | ebi-a-GCST90001711 | TRUE | T | 39938303 | 3.29E-15 | reported | 3657 | 0.02582 | rs5757710 |  |  |  |  |  |  |  |  |  |  |  |  |  |  |  |
| 0.1709 | 6 | igd | 0.3506 | A | \|\| id:ebi-a-GCST90001753 | ebi-a-GCST90001753 | TRUE | C | 32633970 | 3.02E-09 | reported | 3657 | 0.02875 | rs35567463 |  |  |  |  |  |  |  |  |  |  |  |  |  |  |  |
| 0.3276 | 16 | igd | 0.3505 | T | \|\| id:ebi-a-GCST90002087 | ebi-a-GCST90002087 | TRUE | C | 31362711 | 7.19E-31 | reported | 2872 | 0.02803 | rs67898294 |  |  |  |  |  |  |  |  |  |  |  |  |  |  |  |
| 0.2257 | 19 | igd | 0.3503 | A | \|\| id:ebi-a-GCST90001745 | ebi-a-GCST90001745 | TRUE | G | 49878196 | 1.22E-18 | reported | 3657 | 0.02547 | rs2288481 |  |  |  |  |  |  |  |  |  |  |  |  |  |  |  |
| 0.1978 | 19 | igd | 0.3503 | A | \|\| id:ebi-a-GCST90001755 | ebi-a-GCST90001755 | TRUE | G | 49878196 | 1.42E-14 | reported | 3657 | 0.0256 | rs2288481 |  |  |  |  |  |  |  |  |  |  |  |  |  |  |  |
| 0.2739 | 19 | igd | 0.3503 | A | \|\| id:ebi-a-GCST90001757 | ebi-a-GCST90001757 | TRUE | G | 49878196 | 7.67E-27 | reported | 3657 | 0.02533 | rs2288481 |  |  |  |  |  |  |  |  |  |  |  |  |  |  |  |
| 0.1979 | 19 | igd | 0.3503 | A | \|\| id:ebi-a-GCST90001758 | ebi-a-GCST90001758 | TRUE | G | 49878196 | 1.10E-14 | reported | 3657 | 0.0255 | rs2288481 |  |  |  |  |  |  |  |  |  |  |  |  |  |  |  |
| 0.2655 | 19 | igd | 0.3503 | A | \|\| id:ebi-a-GCST90001761 | ebi-a-GCST90001761 | TRUE | G | 49878196 | 5.94E-25 | reported | 3657 | 0.02555 | rs2288481 |  |  |  |  |  |  |  |  |  |  |  |  |  |  |  |
| 0.2483 | 10 | igd | 0.35 | T | \|\| id:ebi-a-GCST90001777 | ebi-a-GCST90001777 | TRUE | G | 6075831 | 2.74E-22 | reported | 3657 | 0.02541 | rs7078614 |  |  |  |  |  |  |  |  |  |  |  |  |  |  |  |
| 0.2056 | 10 | igd | 0.35 | T | \|\| id:ebi-a-GCST90001778 | ebi-a-GCST90001778 | TRUE | G | 6075831 | 1.49E-15 | reported | 3657 | 0.02566 | rs7078614 |  |  |  |  |  |  |  |  |  |  |  |  |  |  |  |
| 0.2186 | 10 | igd | 0.35 | T | \|\| id:ebi-a-GCST90001792 | ebi-a-GCST90001792 | TRUE | G | 6075831 | 1.54E-17 | reported | 3657 | 0.02552 | rs7078614 |  |  |  |  |  |  |  |  |  |  |  |  |  |  |  |
| 0.2383 | 10 | igd | 0.35 | T | \|\| id:ebi-a-GCST90001793 | ebi-a-GCST90001793 | TRUE | G | 6075831 | 2.59E-20 | reported | 3657 | 0.02566 | rs7078614 |  |  |  |  |  |  |  |  |  |  |  |  |  |  |  |
| -0.1658 | 1 | igd | 0.3491 | T | \|\| id:ebi-a-GCST90001832 | ebi-a-GCST90001832 | TRUE | G | 169574729 | 4.47E-09 | reported | 2872 | 0.02818 | rs3917761 |  |  |  |  |  |  |  |  |  |  |  |  |  |  |  |
| -0.2876 | 6 | igd | 0.3485 | T | \|\| id:ebi-a-GCST90002109 | ebi-a-GCST90002109 | TRUE | C | 32193564 | 2.02E-11 | reported | 1578 | 0.04258 | rs3134928 |  |  |  |  |  |  |  |  |  |  |  |  |  |  |  |
| -0.1959 | 10 | igd | 0.3481 | C | \|\| id:ebi-a-GCST90001491 | ebi-a-GCST90001491 | TRUE | T | 97214418 | 1.85E-13 | reported | 3437 | 0.02652 | rs10450393 |  |  |  |  |  |  |  |  |  |  |  |  |  |  |  |
| 0.1769 | 22 | igd | 0.3481 | A | \|\| id:ebi-a-GCST90001702 | ebi-a-GCST90001702 | TRUE | G | 44833082 | 1.96E-11 | reported | 3657 | 0.02629 | rs737724 |  |  |  |  |  |  |  |  |  |  |  |  |  |  |  |
| 0.1771 | 22 | igd | 0.3481 | A | \|\| id:ebi-a-GCST90001703 | ebi-a-GCST90001703 | TRUE | G | 44833082 | 1.88E-11 | reported | 3657 | 0.02629 | rs737724 |  |  |  |  |  |  |  |  |  |  |  |  |  |  |  |
| 0.1844 | 22 | igd | 0.3481 | A | \|\| id:ebi-a-GCST90001704 | ebi-a-GCST90001704 | TRUE | G | 44833082 | 2.47E-12 | reported | 3657 | 0.02624 | rs737724 |  |  |  |  |  |  |  |  |  |  |  |  |  |  |  |
| 0.1636 | 22 | igd | 0.3481 | A | \|\| id:ebi-a-GCST90001710 | ebi-a-GCST90001710 | TRUE | G | 44833082 | 4.97E-10 | reported | 3657 | 0.02624 | rs737724 |  |  |  |  |  |  |  |  |  |  |  |  |  |  |  |
| 0.1687 | 22 | igd | 0.3481 | A | \|\| id:ebi-a-GCST90001711 | ebi-a-GCST90001711 | TRUE | G | 44833082 | 1.31E-10 | reported | 3657 | 0.02617 | rs737724 |  |  |  |  |  |  |  |  |  |  |  |  |  |  |  |
| -0.187 | 12 | igd | 0.3479 | T | \|\| id:ebi-a-GCST90002073 | ebi-a-GCST90002073 | TRUE | G | 69166765 | 1.13E-10 | reported | 2850 | 0.02889 | rs11177366 |  |  |  |  |  |  |  |  |  |  |  |  |  |  |  |
| 0.1827 | 6 | igd | 0.3473 | A | \|\| id:ebi-a-GCST90001932 | ebi-a-GCST90001932 | TRUE | G | 31311192 | 2.17E-08 | reported | 2920 | 0.03255 | rs9265821 |  |  |  |  |  |  |  |  |  |  |  |  |  |  |  |
| -0.1908 | 10 | igd | 0.3473 | C | \|\| id:ebi-a-GCST90002031 | ebi-a-GCST90002031 | TRUE | T | 97214418 | 8.52E-11 | reported | 2920 | 0.02929 | rs10450393 |  |  |  |  |  |  |  |  |  |  |  |  |  |  |  |
| -0.3025 | 6 | igd | 0.347 | T | \|\| id:ebi-a-GCST90002110 | ebi-a-GCST90002110 | TRUE | C | 32193564 | 3.00E-12 | reported | 1634 | 0.04302 | rs3134928 |  |  |  |  |  |  |  |  |  |  |  |  |  |  |  |
| -0.2836 | 6 | igd | 0.3468 | T | \|\| id:ebi-a-GCST90002111 | ebi-a-GCST90002111 | TRUE | C | 32193564 | 2.89E-11 | reported | 1635 | 0.04233 | rs3134928 |  |  |  |  |  |  |  |  |  |  |  |  |  |  |  |
| -0.3425 | 6 | igd | 0.3468 | T | \|\| id:ebi-a-GCST90002112 | ebi-a-GCST90002112 | TRUE | C | 32193564 | 1.67E-15 | reported | 1635 | 0.04258 | rs3134928 |  |  |  |  |  |  |  |  |  |  |  |  |  |  |  |
| 0.2237 | 2 | igd | 0.3466 | C | \|\| id:ebi-a-GCST90001911 | ebi-a-GCST90001911 | TRUE | T | 232290157 | 1.34E-15 | reported | 3113 | 0.02785 | rs6721978 |  |  |  |  |  |  |  |  |  |  |  |  |  |  |  |
| -0.207 | 10 | igd | 0.3465 | C | \|\| id:ebi-a-GCST90001483 | ebi-a-GCST90001483 | TRUE | T | 97210264 | 4.11E-15 | reported | 3405 | 0.02625 | rs10786209 |  |  |  |  |  |  |  |  |  |  |  |  |  |  |  |
| -0.1974 | 10 | igd | 0.3465 | C | \|\| id:ebi-a-GCST90001489 | ebi-a-GCST90001489 | TRUE | T | 97210264 | 3.28E-13 | reported | 3405 | 0.027 | rs10786209 |  |  |  |  |  |  |  |  |  |  |  |  |  |  |  |
| -0.2105 | 10 | igd | 0.3462 | G | \|\| id:ebi-a-GCST90002030 | ebi-a-GCST90002030 | TRUE | T | 97193330 | 3.45E-13 | reported | 2920 | 0.02881 | rs4918929 |  |  |  |  |  |  |  |  |  |  |  |  |  |  |  |
| -0.1943 | 10 | igd | 0.3462 | G | \|\| id:ebi-a-GCST90002032 | ebi-a-GCST90002032 | TRUE | T | 97193330 | 4.74E-11 | reported | 2920 | 0.02942 | rs4918929 |  |  |  |  |  |  |  |  |  |  |  |  |  |  |  |
| -0.249 | 6 | igd | 0.3459 | A | \|\| id:ebi-a-GCST90002111 | ebi-a-GCST90002111 | TRUE | G | 30932606 | 1.09E-08 | reported | 1635 | 0.04333 | rs34767257 |  |  |  |  |  |  |  |  |  |  |  |  |  |  |  |
| 0.238 | 6 | igd | 0.3459 | A | \|\| id:ebi-a-GCST90002112 | ebi-a-GCST90002112 | TRUE | G | 32780724 | 4.46E-09 | reported | 1635 | 0.04035 | rs11244 |  |  |  |  |  |  |  |  |  |  |  |  |  |  |  |
| -0.2272 | 10 | igd | 0.3458 | C | \|\| id:ebi-a-GCST90001484 | ebi-a-GCST90001484 | TRUE | T | 97210264 | 3.66E-18 | reported | 3437 | 0.02601 | rs10786209 |  |  |  |  |  |  |  |  |  |  |  |  |  |  |  |
| -0.2248 | 10 | igd | 0.3458 | C | \|\| id:ebi-a-GCST90001485 | ebi-a-GCST90001485 | TRUE | T | 97210264 | 7.59E-18 | reported | 3437 | 0.02598 | rs10786209 |  |  |  |  |  |  |  |  |  |  |  |  |  |  |  |
| -0.5729 | 10 | igd | 0.3458 | T | \|\| id:ebi-a-GCST90001485 | ebi-a-GCST90001485 | TRUE | C | 97438823 | 6.14E-110 | reported | 3437 | 0.02479 | rs10882651 |  |  |  |  |  |  |  |  |  |  |  |  |  |  |  |
| -0.1875 | 10 | igd | 0.3456 | C | \|\| id:ebi-a-GCST90001658 | ebi-a-GCST90001658 | TRUE | T | 97210264 | 3.97E-13 | reported | 3440 | 0.02574 | rs10786209 |  |  |  |  |  |  |  |  |  |  |  |  |  |  |  |
| -0.1922 | 10 | igd | 0.3456 | C | \|\| id:ebi-a-GCST90001659 | ebi-a-GCST90001659 | TRUE | T | 97210264 | 1.03E-13 | reported | 3440 | 0.02574 | rs10786209 |  |  |  |  |  |  |  |  |  |  |  |  |  |  |  |
| 0.1721 | 22 | igd | 0.3456 | A | \|\| id:ebi-a-GCST90001704 | ebi-a-GCST90001704 | TRUE | G | 41709235 | 4.98E-11 | reported | 3657 | 0.02612 | rs1807590 |  |  |  |  |  |  |  |  |  |  |  |  |  |  |  |
| 0.1424 | 2 | igd | 0.345 | G | \|\| id:ebi-a-GCST90001917 | ebi-a-GCST90001917 | TRUE | A | 38892207 | 4.05E-08 | reported | 3113 | 0.02589 | rs7559619 |  |  |  |  |  |  |  |  |  |  |  |  |  |  |  |
| 0.1905 | 6 | igd | 0.3449 | T | \|\| id:ebi-a-GCST90001684 | ebi-a-GCST90001684 | TRUE | C | 31206206 | 9.17E-10 | reported | 3371 | 0.03102 | rs3130685 |  |  |  |  |  |  |  |  |  |  |  |  |  |  |  |
| -0.1603 | 10 | igd | 0.3448 | T | \|\| id:ebi-a-GCST90001489 | ebi-a-GCST90001489 | TRUE | C | 98794615 | 4.21E-09 | reported | 3405 | 0.02722 | rs12268044 |  |  |  |  |  |  |  |  |  |  |  |  |  |  |  |
| -0.17 | 10 | igd | 0.3448 | T | \|\| id:ebi-a-GCST90001495 | ebi-a-GCST90001495 | TRUE | C | 98794615 | 6.72E-10 | reported | 3405 | 0.02746 | rs12268044 |  |  |  |  |  |  |  |  |  |  |  |  |  |  |  |
| -0.3029 | 4 | igd | 0.344 | C | \|\| id:ebi-a-GCST90001471 | ebi-a-GCST90001471 | TRUE | T | 84151427 | 1.35E-31 | reported | 3410 | 0.02564 | rs6535445 |  |  |  |  |  |  |  |  |  |  |  |  |  |  |  |
| -0.1553 | 10 | igd | 0.3439 | T | \|\| id:ebi-a-GCST90001484 | ebi-a-GCST90001484 | TRUE | C | 98794615 | 3.59E-09 | reported | 3437 | 0.02624 | rs12268044 |  |  |  |  |  |  |  |  |  |  |  |  |  |  |  |
| -0.1509 | 10 | igd | 0.3439 | T | \|\| id:ebi-a-GCST90001485 | ebi-a-GCST90001485 | TRUE | C | 98794615 | 9.31E-09 | reported | 3437 | 0.02621 | rs12268044 |  |  |  |  |  |  |  |  |  |  |  |  |  |  |  |
| -0.1886 | 10 | igd | 0.3439 | T | \|\| id:ebi-a-GCST90001491 | ebi-a-GCST90001491 | TRUE | C | 98794615 | 2.05E-12 | reported | 3437 | 0.02672 | rs12268044 |  |  |  |  |  |  |  |  |  |  |  |  |  |  |  |
| -0.1945 | 10 | igd | 0.3439 | T | \|\| id:ebi-a-GCST90001496 | ebi-a-GCST90001496 | TRUE | C | 98794615 | 8.29E-13 | reported | 3437 | 0.02707 | rs12268044 |  |  |  |  |  |  |  |  |  |  |  |  |  |  |  |
| -0.1937 | 10 | igd | 0.3439 | T | \|\| id:ebi-a-GCST90001497 | ebi-a-GCST90001497 | TRUE | C | 98794615 | 5.32E-13 | reported | 3437 | 0.02673 | rs12268044 |  |  |  |  |  |  |  |  |  |  |  |  |  |  |  |
| 0.1894 | 6 | igd | 0.3439 | T | \|\| id:ebi-a-GCST90001687 | ebi-a-GCST90001687 | TRUE | C | 31206206 | 2.56E-10 | reported | 3408 | 0.02987 | rs3130685 |  |  |  |  |  |  |  |  |  |  |  |  |  |  |  |
| -0.2794 | 4 | igd | 0.3435 | C | \|\| id:ebi-a-GCST90001470 | ebi-a-GCST90001470 | TRUE | T | 84151427 | 5.62E-28 | reported | 3374 | 0.02525 | rs6535445 |  |  |  |  |  |  |  |  |  |  |  |  |  |  |  |
| -0.1507 | 10 | igd | 0.3432 | C | \|\| id:ebi-a-GCST90001658 | ebi-a-GCST90001658 | TRUE | T | 91738825 | 1.07E-08 | reported | 3440 | 0.02628 | rs11812465 |  |  |  |  |  |  |  |  |  |  |  |  |  |  |  |
| -0.1473 | 10 | igd | 0.3432 | C | \|\| id:ebi-a-GCST90001659 | ebi-a-GCST90001659 | TRUE | T | 91738825 | 2.29E-08 | reported | 3440 | 0.02629 | rs11812465 |  |  |  |  |  |  |  |  |  |  |  |  |  |  |  |
| -0.2323 | 10 | igd | 0.3432 | T | \|\| id:ebi-a-GCST90002030 | ebi-a-GCST90002030 | TRUE | C | 98794615 | 8.90E-16 | reported | 2920 | 0.02873 | rs12268044 |  |  |  |  |  |  |  |  |  |  |  |  |  |  |  |
| 0.1743 | 10 | igd | 0.3431 | A | \|\| id:ebi-a-GCST90001940 | ebi-a-GCST90001940 | TRUE | G | 97446206 | 9.87E-11 | reported | 3435 | 0.02686 | rs2297788 |  |  |  |  |  |  |  |  |  |  |  |  |  |  |  |
| 0.1828 | 10 | igd | 0.343 | A | \|\| id:ebi-a-GCST90001935 | ebi-a-GCST90001935 | TRUE | G | 97446206 | 8.35E-12 | reported | 3434 | 0.02666 | rs2297788 |  |  |  |  |  |  |  |  |  |  |  |  |  |  |  |
| -0.1703 | 12 | igd | 0.343 | T | \|\| id:ebi-a-GCST90002074 | ebi-a-GCST90002074 | TRUE | G | 69166765 | 1.11E-09 | reported | 3112 | 0.02787 | rs11177366 |  |  |  |  |  |  |  |  |  |  |  |  |  |  |  |
| 0.2608 | 2 | igd | 0.3428 | A | \|\| id:ebi-a-GCST90002045 | ebi-a-GCST90002045 | TRUE | G | 232290640 | 1.68E-12 | reported | 1635 | 0.03665 | rs12614599 |  |  |  |  |  |  |  |  |  |  |  |  |  |  |  |
| 0.2436 | 1 | igd | 0.3421 | T | \|\| id:ebi-a-GCST90001970 | ebi-a-GCST90001970 | TRUE | G | 161374413 | 5.14E-17 | reported | 2971 | 0.02888 | rs12135728 |  |  |  |  |  |  |  |  |  |  |  |  |  |  |  |
| 0.2137 | 1 | igd | 0.3421 | T | \|\| id:ebi-a-GCST90002077 | ebi-a-GCST90002077 | TRUE | G | 161374413 | 1.27E-13 | reported | 2971 | 0.0287 | rs12135728 |  |  |  |  |  |  |  |  |  |  |  |  |  |  |  |
| 0.2046 | 1 | igd | 0.3416 | C | \|\| id:ebi-a-GCST90001970 | ebi-a-GCST90001970 | TRUE | A | 161234623 | 9.74E-13 | reported | 2971 | 0.02856 | rs11265577 |  |  |  |  |  |  |  |  |  |  |  |  |  |  |  |
| 0.182 | 10 | igd | 0.3414 | T | \|\| id:ebi-a-GCST90001935 | ebi-a-GCST90001935 | TRUE | G | 6075831 | 4.31E-12 | reported | 3434 | 0.02618 | rs7078614 |  |  |  |  |  |  |  |  |  |  |  |  |  |  |  |
| 0.1714 | 10 | igd | 0.3413 | T | \|\| id:ebi-a-GCST90001940 | ebi-a-GCST90001940 | TRUE | G | 6075831 | 9.59E-11 | reported | 3435 | 0.0264 | rs7078614 |  |  |  |  |  |  |  |  |  |  |  |  |  |  |  |
| 0.2373 | 10 | igd | 0.3409 | A | \|\| id:ebi-a-GCST90002030 | ebi-a-GCST90002030 | TRUE | C | 95712322 | 3.68E-16 | reported | 2920 | 0.02895 | rs11187737 |  |  |  |  |  |  |  |  |  |  |  |  |  |  |  |
| 0.2082 | 10 | igd | 0.3409 | A | \|\| id:ebi-a-GCST90002031 | ebi-a-GCST90002031 | TRUE | C | 95712322 | 2.86E-12 | reported | 2920 | 0.02969 | rs11187737 |  |  |  |  |  |  |  |  |  |  |  |  |  |  |  |
| 0.252 | 10 | igd | 0.3409 | A | \|\| id:ebi-a-GCST90002032 | ebi-a-GCST90002032 | TRUE | C | 95712322 | 2.40E-17 | reported | 2920 | 0.02956 | rs11187737 |  |  |  |  |  |  |  |  |  |  |  |  |  |  |  |
| -0.2195 | 4 | igd | 0.3407 | G | \|\| id:ebi-a-GCST90002072 | ebi-a-GCST90002072 | TRUE | C | 84157443 | 4.31E-14 | reported | 2872 | 0.02892 | rs2903918 |  |  |  |  |  |  |  |  |  |  |  |  |  |  |  |
| -0.1451 | 10 | igd | 0.34 | T | \|\| id:ebi-a-GCST90001658 | ebi-a-GCST90001658 | TRUE | C | 95521230 | 1.75E-08 | reported | 3440 | 0.02568 | rs12769713 |  |  |  |  |  |  |  |  |  |  |  |  |  |  |  |
| -0.1692 | 10 | igd | 0.34 | T | \|\| id:ebi-a-GCST90001670 | ebi-a-GCST90001670 | TRUE | C | 95521230 | 2.67E-10 | reported | 3440 | 0.02671 | rs12769713 |  |  |  |  |  |  |  |  |  |  |  |  |  |  |  |
| -0.1646 | 10 | igd | 0.34 | T | \|\| id:ebi-a-GCST90001671 | ebi-a-GCST90001671 | TRUE | C | 95521230 | 8.47E-10 | reported | 3440 | 0.02675 | rs12769713 |  |  |  |  |  |  |  |  |  |  |  |  |  |  |  |
| -0.161 | 10 | igd | 0.3396 | T | \|\| id:ebi-a-GCST90001672 | ebi-a-GCST90001672 | TRUE | C | 95521230 | 3.60E-09 | reported | 3408 | 0.0272 | rs12769713 |  |  |  |  |  |  |  |  |  |  |  |  |  |  |  |
| -0.1475 | 1 | igd | 0.3394 | T | \|\| id:ebi-a-GCST90001994 | ebi-a-GCST90001994 | TRUE | G | 161374413 | 1.96E-08 | reported | 3590 | 0.02621 | rs12135728 |  |  |  |  |  |  |  |  |  |  |  |  |  |  |  |
| 0.1537 | 10 | igd | 0.3392 | A | \|\| id:ebi-a-GCST90001497 | ebi-a-GCST90001497 | TRUE | C | 95712322 | 1.76E-08 | reported | 3437 | 0.02722 | rs11187737 |  |  |  |  |  |  |  |  |  |  |  |  |  |  |  |
| 0.146 | 10 | igd | 0.3391 | A | \|\| id:ebi-a-GCST90001659 | ebi-a-GCST90001659 | TRUE | C | 95712322 | 2.99E-08 | reported | 3440 | 0.02629 | rs11187737 |  |  |  |  |  |  |  |  |  |  |  |  |  |  |  |
| 0.194 | 1 | igd | 0.3389 | T | \|\| id:ebi-a-GCST90001999 | ebi-a-GCST90001999 | TRUE | G | 161374413 | 3.36E-13 | reported | 3621 | 0.02656 | rs12135728 |  |  |  |  |  |  |  |  |  |  |  |  |  |  |  |
| 0.1973 | 6 | igd | 0.3382 | T | \|\| id:ebi-a-GCST90002067 | ebi-a-GCST90002067 | TRUE | C | 31867253 | 1.63E-09 | reported | 2920 | 0.03261 | rs9267663 |  |  |  |  |  |  |  |  |  |  |  |  |  |  |  |
| 0.1766 | 6 | igd | 0.338 | G | \|\| id:ebi-a-GCST90001932 | ebi-a-GCST90001932 | TRUE | C | 31862499 | 4.34E-08 | reported | 2920 | 0.03216 | rs9267662 |  |  |  |  |  |  |  |  |  |  |  |  |  |  |  |
| -0.221 | 6 | igd | 0.3368 | C | \|\| id:ebi-a-GCST90001820 | ebi-a-GCST90001820 | TRUE | T | 31297744 | 1.20E-11 | reported | 3656 | 0.03249 | rs9265515 |  |  |  |  |  |  |  |  |  |  |  |  |  |  |  |
| -0.1763 | 10 | igd | 0.3366 | G | \|\| id:ebi-a-GCST90002030 | ebi-a-GCST90002030 | TRUE | C | 97040270 | 2.10E-09 | reported | 2920 | 0.02934 | rs12098298 |  |  |  |  |  |  |  |  |  |  |  |  |  |  |  |
| 0.1996 | 1 | igd | 0.3364 | T | \|\| id:ebi-a-GCST90001474 | ebi-a-GCST90001474 | TRUE | G | 161374413 | 1.31E-13 | reported | 3410 | 0.02685 | rs12135728 |  |  |  |  |  |  |  |  |  |  |  |  |  |  |  |
| -0.3338 | 6 | igd | 0.3363 | T | \|\| id:ebi-a-GCST90002108 | ebi-a-GCST90002108 | TRUE | G | 32338283 | 1.74E-14 | reported | 1579 | 0.04311 | rs3129942 |  |  |  |  |  |  |  |  |  |  |  |  |  |  |  |
| 0.2051 | 10 | igd | 0.3359 | T | \|\| id:ebi-a-GCST90001782 | ebi-a-GCST90001782 | TRUE | C | 6176166 | 7.93E-09 | reported | 1837 | 0.03538 | rs4747886 |  |  |  |  |  |  |  |  |  |  |  |  |  |  |  |
| 0.1433 | 10 | igd | 0.3355 | G | \|\| id:ebi-a-GCST90001484 | ebi-a-GCST90001484 | TRUE | A | 98410170 | 4.48E-08 | reported | 3437 | 0.02613 | rs10748687 |  |  |  |  |  |  |  |  |  |  |  |  |  |  |  |
| 0.1641 | 10 | igd | 0.3352 | T | \|\| id:ebi-a-GCST90001505 | ebi-a-GCST90001505 | TRUE | C | 6176166 | 1.84E-09 | reported | 3437 | 0.02723 | rs4747886 |  |  |  |  |  |  |  |  |  |  |  |  |  |  |  |
| -0.1629 | 10 | igd | 0.3351 | T | \|\| id:ebi-a-GCST90002030 | ebi-a-GCST90002030 | TRUE | C | 97230742 | 1.62E-08 | reported | 2920 | 0.02875 | rs499441 |  |  |  |  |  |  |  |  |  |  |  |  |  |  |  |
| 0.1747 | 10 | igd | 0.3349 | T | \|\| id:ebi-a-GCST90001794 | ebi-a-GCST90001794 | TRUE | C | 6176166 | 7.97E-11 | reported | 3658 | 0.0268 | rs4747886 |  |  |  |  |  |  |  |  |  |  |  |  |  |  |  |
| 0.1841 | 10 | igd | 0.3348 | T | \|\| id:ebi-a-GCST90001775 | ebi-a-GCST90001775 | TRUE | C | 6176166 | 7.31E-12 | reported | 3660 | 0.02678 | rs4747886 |  |  |  |  |  |  |  |  |  |  |  |  |  |  |  |
| 0.2466 | 10 | igd | 0.3348 | T | \|\| id:ebi-a-GCST90001777 | ebi-a-GCST90001777 | TRUE | C | 6176166 | 6.08E-21 | reported | 3657 | 0.0261 | rs4747886 |  |  |  |  |  |  |  |  |  |  |  |  |  |  |  |
| 0.2116 | 10 | igd | 0.3348 | T | \|\| id:ebi-a-GCST90001778 | ebi-a-GCST90001778 | TRUE | C | 6176166 | 1.31E-15 | reported | 3657 | 0.02635 | rs4747886 |  |  |  |  |  |  |  |  |  |  |  |  |  |  |  |
| 0.2231 | 10 | igd | 0.3348 | T | \|\| id:ebi-a-GCST90001779 | ebi-a-GCST90001779 | TRUE | C | 6176166 | 6.68E-17 | reported | 3657 | 0.02658 | rs4747886 |  |  |  |  |  |  |  |  |  |  |  |  |  |  |  |
| 0.1885 | 10 | igd | 0.3348 | T | \|\| id:ebi-a-GCST90001784 | ebi-a-GCST90001784 | TRUE | C | 6176166 | 2.59E-12 | reported | 3657 | 0.02684 | rs4747886 |  |  |  |  |  |  |  |  |  |  |  |  |  |  |  |
| 0.2399 | 10 | igd | 0.3348 | T | \|\| id:ebi-a-GCST90001789 | ebi-a-GCST90001789 | TRUE | C | 6176166 | 3.98E-20 | reported | 3657 | 0.02595 | rs4747886 |  |  |  |  |  |  |  |  |  |  |  |  |  |  |  |
| 0.26 | 10 | igd | 0.3348 | T | \|\| id:ebi-a-GCST90001790 | ebi-a-GCST90001790 | TRUE | C | 6176166 | 4.31E-23 | reported | 3657 | 0.02609 | rs4747886 |  |  |  |  |  |  |  |  |  |  |  |  |  |  |  |
| 0.2123 | 10 | igd | 0.3348 | T | \|\| id:ebi-a-GCST90001791 | ebi-a-GCST90001791 | TRUE | C | 6176166 | 3.24E-15 | reported | 3657 | 0.02682 | rs4747886 |  |  |  |  |  |  |  |  |  |  |  |  |  |  |  |
| 0.2228 | 10 | igd | 0.3348 | T | \|\| id:ebi-a-GCST90001792 | ebi-a-GCST90001792 | TRUE | C | 6176166 | 2.71E-17 | reported | 3657 | 0.02621 | rs4747886 |  |  |  |  |  |  |  |  |  |  |  |  |  |  |  |
| 0.1905 | 10 | igd | 0.3347 | T | \|\| id:ebi-a-GCST90001780 | ebi-a-GCST90001780 | TRUE | C | 6176166 | 8.00E-13 | reported | 3642 | 0.02651 | rs4747886 |  |  |  |  |  |  |  |  |  |  |  |  |  |  |  |
| -0.1484 | 1 | igd | 0.3347 | C | \|\| id:ebi-a-GCST90001807 | ebi-a-GCST90001807 | TRUE | T | 161478859 | 9.69E-09 | reported | 3651 | 0.02581 | rs4657041 |  |  |  |  |  |  |  |  |  |  |  |  |  |  |  |
| -0.1479 | 1 | igd | 0.3344 | C | \|\| id:ebi-a-GCST90001710 | ebi-a-GCST90001710 | TRUE | T | 161478859 | 4.18E-08 | reported | 3657 | 0.02692 | rs4657041 |  |  |  |  |  |  |  |  |  |  |  |  |  |  |  |
| -0.1503 | 1 | igd | 0.3344 | C | \|\| id:ebi-a-GCST90001711 | ebi-a-GCST90001711 | TRUE | T | 161478859 | 2.29E-08 | reported | 3657 | 0.02684 | rs4657041 |  |  |  |  |  |  |  |  |  |  |  |  |  |  |  |
| 0.2075 | 10 | igd | 0.3344 | G | \|\| id:ebi-a-GCST90002030 | ebi-a-GCST90002030 | TRUE | A | 98410170 | 4.29E-13 | reported | 2920 | 0.0285 | rs10748687 |  |  |  |  |  |  |  |  |  |  |  |  |  |  |  |
| 0.2084 | 10 | igd | 0.3344 | G | \|\| id:ebi-a-GCST90002031 | ebi-a-GCST90002031 | TRUE | A | 98410170 | 1.03E-12 | reported | 2920 | 0.02912 | rs10748687 |  |  |  |  |  |  |  |  |  |  |  |  |  |  |  |
| -0.243 | 6 | igd | 0.3343 | T | \|\| id:ebi-a-GCST90002111 | ebi-a-GCST90002111 | TRUE | G | 30014705 | 2.10E-08 | reported | 1635 | 0.04314 | rs78772196 |  |  |  |  |  |  |  |  |  |  |  |  |  |  |  |
| 0.215 | 2 | igd | 0.3336 | G | \|\| id:ebi-a-GCST90001892 | ebi-a-GCST90001892 | TRUE | C | 204573392 | 1.51E-13 | reported | 2920 | 0.02897 | rs35988305 |  |  |  |  |  |  |  |  |  |  |  |  |  |  |  |
| -0.2126 | 6 | igd | 0.3336 | T | \|\| id:ebi-a-GCST90002027 | ebi-a-GCST90002027 | TRUE | C | 32637298 | 3.26E-08 | reported | 2911 | 0.03835 | rs9274712 |  |  |  |  |  |  |  |  |  |  |  |  |  |  |  |
| 0.2617 | 1 | igd | 0.3334 | G | \|\| id:ebi-a-GCST90001525 | ebi-a-GCST90001525 | TRUE | A | 161479745 | 8.34E-14 | reported | 1858 | 0.03479 | rs1801274 |  |  |  |  |  |  |  |  |  |  |  |  |  |  |  |
| 0.5511 | 1 | igd | 0.3334 | G | \|\| id:ebi-a-GCST90001526 | ebi-a-GCST90001526 | TRUE | A | 161479745 | 2.77E-54 | reported | 1858 | 0.03437 | rs1801274 |  |  |  |  |  |  |  |  |  |  |  |  |  |  |  |
| -0.5517 | 1 | igd | 0.3334 | G | \|\| id:ebi-a-GCST90001528 | ebi-a-GCST90001528 | TRUE | A | 161479745 | 2.53E-54 | reported | 1858 | 0.03439 | rs1801274 |  |  |  |  |  |  |  |  |  |  |  |  |  |  |  |
| -0.213 | 6 | igd | 0.3334 | T | \|\| id:ebi-a-GCST90002105 | ebi-a-GCST90002105 | TRUE | C | 30741226 | 1.96E-09 | reported | 2872 | 0.03538 | rs3132604 |  |  |  |  |  |  |  |  |  |  |  |  |  |  |  |
| 0.2427 | 20 | igd | 0.3333 | C | \|\| id:ebi-a-GCST90001801 | ebi-a-GCST90001801 | TRUE | G | 44761377 | 1.54E-11 | reported | 1836 | 0.03576 | rs12481282 |  |  |  |  |  |  |  |  |  |  |  |  |  |  |  |
| -0.1675 | 10 | igd | 0.333 | G | \|\| id:ebi-a-GCST90001491 | ebi-a-GCST90001491 | TRUE | C | 97040270 | 1.12E-09 | reported | 3437 | 0.02743 | rs12098298 |  |  |  |  |  |  |  |  |  |  |  |  |  |  |  |
| -0.1555 | 10 | igd | 0.333 | G | \|\| id:ebi-a-GCST90001496 | ebi-a-GCST90001496 | TRUE | C | 97040270 | 2.43E-08 | reported | 3437 | 0.02782 | rs12098298 |  |  |  |  |  |  |  |  |  |  |  |  |  |  |  |
| -0.1675 | 10 | igd | 0.333 | G | \|\| id:ebi-a-GCST90001497 | ebi-a-GCST90001497 | TRUE | C | 97040270 | 1.17E-09 | reported | 3437 | 0.02745 | rs12098298 |  |  |  |  |  |  |  |  |  |  |  |  |  |  |  |
| -0.1549 | 10 | igd | 0.3328 | G | \|\| id:ebi-a-GCST90001658 | ebi-a-GCST90001658 | TRUE | C | 97040270 | 5.49E-09 | reported | 3440 | 0.0265 | rs12098298 |  |  |  |  |  |  |  |  |  |  |  |  |  |  |  |
| -0.1604 | 10 | igd | 0.3328 | G | \|\| id:ebi-a-GCST90001659 | ebi-a-GCST90001659 | TRUE | C | 97040270 | 1.55E-09 | reported | 3440 | 0.02649 | rs12098298 |  |  |  |  |  |  |  |  |  |  |  |  |  |  |  |
| -0.1564 | 10 | igd | 0.3328 | G | \|\| id:ebi-a-GCST90001670 | ebi-a-GCST90001670 | TRUE | C | 97040270 | 1.56E-08 | reported | 3440 | 0.02758 | rs12098298 |  |  |  |  |  |  |  |  |  |  |  |  |  |  |  |
| -0.1553 | 10 | igd | 0.3328 | G | \|\| id:ebi-a-GCST90001671 | ebi-a-GCST90001671 | TRUE | C | 97040270 | 2.06E-08 | reported | 3440 | 0.02763 | rs12098298 |  |  |  |  |  |  |  |  |  |  |  |  |  |  |  |
| -0.1993 | 10 | igd | 0.3326 | T | \|\| id:ebi-a-GCST90001489 | ebi-a-GCST90001489 | TRUE | C | 97230742 | 3.63E-13 | reported | 3405 | 0.02731 | rs499441 |  |  |  |  |  |  |  |  |  |  |  |  |  |  |  |
| -0.2059 | 10 | igd | 0.3326 | T | \|\| id:ebi-a-GCST90001495 | ebi-a-GCST90001495 | TRUE | C | 97230742 | 9.51E-14 | reported | 3405 | 0.02754 | rs499441 |  |  |  |  |  |  |  |  |  |  |  |  |  |  |  |
| -0.1562 | 10 | igd | 0.3325 | G | \|\| id:ebi-a-GCST90001489 | ebi-a-GCST90001489 | TRUE | C | 97040270 | 2.13E-08 | reported | 3405 | 0.02781 | rs12098298 |  |  |  |  |  |  |  |  |  |  |  |  |  |  |  |
| -0.1523 | 10 | igd | 0.3323 | G | \|\| id:ebi-a-GCST90001660 | ebi-a-GCST90001660 | TRUE | C | 97040270 | 4.47E-08 | reported | 3408 | 0.02777 | rs12098298 |  |  |  |  |  |  |  |  |  |  |  |  |  |  |  |
| -0.2124 | 10 | igd | 0.3323 | T | \|\| id:ebi-a-GCST90001660 | ebi-a-GCST90001660 | TRUE | C | 97230742 | 7.61E-15 | reported | 3408 | 0.0272 | rs499441 |  |  |  |  |  |  |  |  |  |  |  |  |  |  |  |
| -0.1581 | 10 | igd | 0.3323 | T | \|\| id:ebi-a-GCST90001672 | ebi-a-GCST90001672 | TRUE | C | 97230742 | 1.07E-08 | reported | 3408 | 0.02758 | rs499441 |  |  |  |  |  |  |  |  |  |  |  |  |  |  |  |
| -0.154 | 10 | igd | 0.3323 | G | \|\| id:ebi-a-GCST90001672 | ebi-a-GCST90001672 | TRUE | C | 97040270 | 4.31E-08 | reported | 3408 | 0.02804 | rs12098298 |  |  |  |  |  |  |  |  |  |  |  |  |  |  |  |
| -0.1865 | 10 | igd | 0.3321 | T | \|\| id:ebi-a-GCST90001491 | ebi-a-GCST90001491 | TRUE | C | 97230742 | 4.90E-12 | reported | 3437 | 0.0269 | rs499441 |  |  |  |  |  |  |  |  |  |  |  |  |  |  |  |
| -0.1802 | 10 | igd | 0.3321 | T | \|\| id:ebi-a-GCST90001496 | ebi-a-GCST90001496 | TRUE | C | 97230742 | 4.52E-11 | reported | 3437 | 0.02728 | rs499441 |  |  |  |  |  |  |  |  |  |  |  |  |  |  |  |
| -0.1757 | 10 | igd | 0.3321 | T | \|\| id:ebi-a-GCST90001497 | ebi-a-GCST90001497 | TRUE | C | 97230742 | 8.02E-11 | reported | 3437 | 0.02694 | rs499441 |  |  |  |  |  |  |  |  |  |  |  |  |  |  |  |
| -0.2846 | 6 | igd | 0.3315 | A | \|\| id:ebi-a-GCST90002105 | ebi-a-GCST90002105 | TRUE | G | 32326165 | 7.77E-16 | reported | 2872 | 0.03512 | rs9268303 |  |  |  |  |  |  |  |  |  |  |  |  |  |  |  |
| 0.3398 | 20 | igd | 0.3313 | T | \|\| id:ebi-a-GCST90001989 | ebi-a-GCST90001989 | TRUE | C | 44777295 | 1.27E-37 | reported | 3621 | 0.0262 | rs6032678 |  |  |  |  |  |  |  |  |  |  |  |  |  |  |  |
| 0.1833 | 10 | igd | 0.3312 | A | \|\| id:ebi-a-GCST90001897 | ebi-a-GCST90001897 | TRUE | T | 97370989 | 1.42E-10 | reported | 2914 | 0.02847 | rs2296691 |  |  |  |  |  |  |  |  |  |  |  |  |  |  |  |
| 0.2984 | 10 | igd | 0.3312 | A | \|\| id:ebi-a-GCST90002029 | ebi-a-GCST90002029 | TRUE | T | 97370989 | 1.10E-25 | reported | 2915 | 0.02821 | rs2296691 |  |  |  |  |  |  |  |  |  |  |  |  |  |  |  |
| 0.3032 | 2 | igd | 0.3311 | G | \|\| id:ebi-a-GCST90002102 | ebi-a-GCST90002102 | TRUE | A | 38899080 | 2.51E-28 | reported | 2919 | 0.02718 | rs13024705 |  |  |  |  |  |  |  |  |  |  |  |  |  |  |  |
| 0.2998 | 20 | igd | 0.3309 | T | \|\| id:ebi-a-GCST90001980 | ebi-a-GCST90001980 | TRUE | C | 44777295 | 2.73E-30 | reported | 3629 | 0.02598 | rs6032678 |  |  |  |  |  |  |  |  |  |  |  |  |  |  |  |
| 0.339 | 20 | igd | 0.3309 | T | \|\| id:ebi-a-GCST90001985 | ebi-a-GCST90001985 | TRUE | C | 44777295 | 9.05E-38 | reported | 3629 | 0.02608 | rs6032678 |  |  |  |  |  |  |  |  |  |  |  |  |  |  |  |
| -0.3514 | 6 | igd | 0.3309 | T | \|\| id:ebi-a-GCST90002112 | ebi-a-GCST90002112 | TRUE | C | 31749804 | 4.66E-16 | reported | 1635 | 0.04283 | rs743399 |  |  |  |  |  |  |  |  |  |  |  |  |  |  |  |
| 0.461 | 1 | igd | 0.3309 | G | \|\| id:ebi-a-GCST90002094 | ebi-a-GCST90002094 | TRUE | A | 161479745 | 5.24E-32 | reported | 1570 | 0.03828 | rs1801274 |  |  |  |  |  |  |  |  |  |  |  |  |  |  |  |
| 0.4097 | 20 | igd | 0.3308 | T | \|\| id:ebi-a-GCST90001981 | ebi-a-GCST90001981 | TRUE | C | 44777295 | 1.63E-55 | reported | 3618 | 0.02566 | rs6032678 |  |  |  |  |  |  |  |  |  |  |  |  |  |  |  |
| 0.6325 | 10 | igd | 0.3308 | A | \|\| id:ebi-a-GCST90002030 | ebi-a-GCST90002030 | TRUE | T | 97370989 | 7.35E-113 | reported | 2920 | 0.0268 | rs2296691 |  |  |  |  |  |  |  |  |  |  |  |  |  |  |  |
| 0.567 | 10 | igd | 0.3308 | A | \|\| id:ebi-a-GCST90002031 | ebi-a-GCST90002031 | TRUE | T | 97370989 | 3.51E-86 | reported | 2920 | 0.02786 | rs2296691 |  |  |  |  |  |  |  |  |  |  |  |  |  |  |  |
| 0.6031 | 10 | igd | 0.3308 | A | \|\| id:ebi-a-GCST90002032 | ebi-a-GCST90002032 | TRUE | T | 97370989 | 3.77E-98 | reported | 2920 | 0.0276 | rs2296691 |  |  |  |  |  |  |  |  |  |  |  |  |  |  |  |
| 0.2405 | 20 | igd | 0.3307 | C | \|\| id:ebi-a-GCST90001800 | ebi-a-GCST90001800 | TRUE | G | 44761377 | 2.15E-19 | reported | 3656 | 0.02655 | rs12481282 |  |  |  |  |  |  |  |  |  |  |  |  |  |  |  |
| -0.1652 | 20 | igd | 0.3306 | C | \|\| id:ebi-a-GCST90001399 | ebi-a-GCST90001399 | TRUE | G | 44761377 | 8.11E-10 | reported | 3657 | 0.02682 | rs12481282 |  |  |  |  |  |  |  |  |  |  |  |  |  |  |  |
| 0.2421 | 20 | igd | 0.3306 | C | \|\| id:ebi-a-GCST90001798 | ebi-a-GCST90001798 | TRUE | G | 44761377 | 3.80E-20 | reported | 3657 | 0.02618 | rs12481282 |  |  |  |  |  |  |  |  |  |  |  |  |  |  |  |
| 0.2666 | 20 | igd | 0.3306 | C | \|\| id:ebi-a-GCST90001804 | ebi-a-GCST90001804 | TRUE | G | 44761377 | 2.50E-24 | reported | 3657 | 0.02601 | rs12481282 |  |  |  |  |  |  |  |  |  |  |  |  |  |  |  |
| 0.2515 | 20 | igd | 0.3306 | C | \|\| id:ebi-a-GCST90001808 | ebi-a-GCST90001808 | TRUE | G | 44761377 | 2.56E-21 | reported | 3657 | 0.02636 | rs12481282 |  |  |  |  |  |  |  |  |  |  |  |  |  |  |  |
| 0.4941 | 1 | igd | 0.3305 | G | \|\| id:ebi-a-GCST90002097 | ebi-a-GCST90002097 | TRUE | A | 161479745 | 1.43E-38 | reported | 1578 | 0.03702 | rs1801274 |  |  |  |  |  |  |  |  |  |  |  |  |  |  |  |
| 0.2448 | 10 | igd | 0.3304 | A | \|\| id:ebi-a-GCST90001496 | ebi-a-GCST90001496 | TRUE | G | 97243992 | 6.92E-19 | reported | 3437 | 0.02743 | rs76182403 |  |  |  |  |  |  |  |  |  |  |  |  |  |  |  |
| 0.2339 | 10 | igd | 0.3304 | A | \|\| id:ebi-a-GCST90001497 | ebi-a-GCST90001497 | TRUE | G | 97243992 | 9.14E-18 | reported | 3437 | 0.0271 | rs76182403 |  |  |  |  |  |  |  |  |  |  |  |  |  |  |  |
| 0.2069 | 20 | igd | 0.3304 | G | \|\| id:ebi-a-GCST90001806 | ebi-a-GCST90001806 | TRUE | T | 44761485 | 7.40E-15 | reported | 3656 | 0.0265 | rs12480534 |  |  |  |  |  |  |  |  |  |  |  |  |  |  |  |
| 0.624 | 1 | igd | 0.3303 | G | \|\| id:ebi-a-GCST90002091 | ebi-a-GCST90002091 | TRUE | A | 161479745 | 6.27E-61 | reported | 1635 | 0.03633 | rs1801274 |  |  |  |  |  |  |  |  |  |  |  |  |  |  |  |
| -0.3379 | 1 | igd | 0.3302 | G | \|\| id:ebi-a-GCST90002021 | ebi-a-GCST90002021 | TRUE | A | 161479745 | 1.47E-18 | reported | 1634 | 0.03798 | rs1801274 |  |  |  |  |  |  |  |  |  |  |  |  |  |  |  |
| -0.2247 | 1 | igd | 0.33 | A | \|\| id:ebi-a-GCST90002014 | ebi-a-GCST90002014 | TRUE | G | 161478169 | 4.44E-14 | reported | 2870 | 0.02962 | rs60574804 |  |  |  |  |  |  |  |  |  |  |  |  |  |  |  |
| -0.261 | 1 | igd | 0.3299 | G | \|\| id:ebi-a-GCST90001949 | ebi-a-GCST90001949 | TRUE | A | 161479745 | 1.84E-11 | reported | 1634 | 0.03857 | rs1801274 |  |  |  |  |  |  |  |  |  |  |  |  |  |  |  |
| -0.2575 | 1 | igd | 0.3298 | G | \|\| id:ebi-a-GCST90001948 | ebi-a-GCST90001948 | TRUE | A | 161479745 | 3.92E-11 | reported | 1633 | 0.0387 | rs1801274 |  |  |  |  |  |  |  |  |  |  |  |  |  |  |  |
| 0.1907 | 6 | igd | 0.3296 | T | \|\| id:ebi-a-GCST90002067 | ebi-a-GCST90002067 | TRUE | C | 32389375 | 1.55E-08 | reported | 2920 | 0.03363 | rs6912382 |  |  |  |  |  |  |  |  |  |  |  |  |  |  |  |
| -0.2558 | 6 | igd | 0.3296 | G | \|\| id:ebi-a-GCST90002108 | ebi-a-GCST90002108 | TRUE | A | 31322047 | 8.52E-09 | reported | 1579 | 0.04418 | rs1053726 |  |  |  |  |  |  |  |  |  |  |  |  |  |  |  |
| 0.2344 | 10 | igd | 0.3295 | A | \|\| id:ebi-a-GCST90001660 | ebi-a-GCST90001660 | TRUE | G | 97243992 | 1.89E-17 | reported | 3408 | 0.02743 | rs76182403 |  |  |  |  |  |  |  |  |  |  |  |  |  |  |  |
| -0.9416 | 19 | igd | 0.32923 | A | \|\| id:prot-a-439 | prot-a-439 | TRUE | C | 51727962 | 1.00E-200 | reported | 3301 | 0.0209 | rs3865444 |  |  |  |  |  |  |  |  |  |  |  |  |  |  |  |
| 0.2295 | 10 | igd | 0.3292 | A | \|\| id:ebi-a-GCST90001495 | ebi-a-GCST90001495 | TRUE | G | 97243992 | 1.94E-16 | reported | 3405 | 0.02777 | rs76182403 |  |  |  |  |  |  |  |  |  |  |  |  |  |  |  |
| -0.2549 | 6 | igd | 0.329 | C | \|\| id:ebi-a-GCST90002108 | ebi-a-GCST90002108 | TRUE | G | 30741305 | 5.17E-09 | reported | 1579 | 0.04339 | rs3095335 |  |  |  |  |  |  |  |  |  |  |  |  |  |  |  |
| -0.2621 | 19 | igd | 0.3288 | A | \|\| id:ebi-a-GCST90001955 | ebi-a-GCST90001955 | TRUE | G | 53261401 | 2.01E-11 | reported | 1554 | 0.0388 | rs8101912 |  |  |  |  |  |  |  |  |  |  |  |  |  |  |  |
| -0.1834 | 1 | igd | 0.3286 | T | \|\| id:ebi-a-GCST90002013 | ebi-a-GCST90002013 | TRUE | C | 161258765 | 9.95E-10 | reported | 2870 | 0.02992 | rs9651035 |  |  |  |  |  |  |  |  |  |  |  |  |  |  |  |
| 0.1758 | 2 | igd | 0.3283 | A | \|\| id:ebi-a-GCST90001891 | ebi-a-GCST90001891 | TRUE | G | 203143683 | 2.09E-09 | reported | 2920 | 0.02925 | rs72923091 |  |  |  |  |  |  |  |  |  |  |  |  |  |  |  |
| -0.2042 | 6 | igd | 0.3281 | C | \|\| id:ebi-a-GCST90001820 | ebi-a-GCST90001820 | TRUE | G | 30741305 | 8.33E-11 | reported | 3656 | 0.03134 | rs3095335 |  |  |  |  |  |  |  |  |  |  |  |  |  |  |  |
| 0.1722 | 6 | igd | 0.328 | T | \|\| id:ebi-a-GCST90001745 | ebi-a-GCST90001745 | TRUE | C | 29905727 | 4.76E-08 | reported | 3657 | 0.03147 | rs13190846 |  |  |  |  |  |  |  |  |  |  |  |  |  |  |  |
| 0.1821 | 6 | igd | 0.328 | T | \|\| id:ebi-a-GCST90001758 | ebi-a-GCST90001758 | TRUE | C | 29905727 | 7.35E-09 | reported | 3657 | 0.03141 | rs13190846 |  |  |  |  |  |  |  |  |  |  |  |  |  |  |  |
| 0.1884 | 6 | igd | 0.3274 | C | \|\| id:ebi-a-GCST90002119 | ebi-a-GCST90002119 | TRUE | T | 29937971 | 8.59E-09 | reported | 2920 | 0.03263 | rs6939037 |  |  |  |  |  |  |  |  |  |  |  |  |  |  |  |
| 0.1938 | 3 | igd | 0.3274 | A | \|\| id:ebi-a-GCST90002036 | ebi-a-GCST90002036 | TRUE | T | 52686716 | 9.77E-11 | reported | 2871 | 0.02984 | rs2878632 |  |  |  |  |  |  |  |  |  |  |  |  |  |  |  |
| 0.1894 | 1 | igd | 0.3272 | C | \|\| id:ebi-a-GCST90001994 | ebi-a-GCST90001994 | TRUE | T | 161244573 | 1.35E-12 | reported | 3590 | 0.02663 | rs4657003 |  |  |  |  |  |  |  |  |  |  |  |  |  |  |  |
| -0.4628 | 1 | igd | 0.327 | G | \|\| id:ebi-a-GCST90001996 | ebi-a-GCST90001996 | TRUE | A | 161479745 | 2.62E-69 | reported | 3579 | 0.02573 | rs1801274 |  |  |  |  |  |  |  |  |  |  |  |  |  |  |  |
| -0.2388 | 1 | igd | 0.3268 | G | \|\| id:ebi-a-GCST90002012 | ebi-a-GCST90002012 | TRUE | A | 161479745 | 6.57E-20 | reported | 3582 | 0.02599 | rs1801274 |  |  |  |  |  |  |  |  |  |  |  |  |  |  |  |
| 0.4646 | 1 | igd | 0.3267 | G | \|\| id:ebi-a-GCST90001981 | ebi-a-GCST90001981 | TRUE | A | 161479745 | 2.06E-70 | reported | 3618 | 0.02562 | rs1801274 |  |  |  |  |  |  |  |  |  |  |  |  |  |  |  |
| 0.1446 | 1 | igd | 0.3267 | G | \|\| id:ebi-a-GCST90001998 | ebi-a-GCST90001998 | TRUE | A | 161479745 | 3.85E-08 | reported | 3618 | 0.02624 | rs1801274 |  |  |  |  |  |  |  |  |  |  |  |  |  |  |  |
| -0.182 | 16 | igd | 0.3265 | T | \|\| id:ebi-a-GCST90001924 | ebi-a-GCST90001924 | TRUE | C | 423619 | 2.20E-10 | reported | 2920 | 0.02858 | rs1573734 |  |  |  |  |  |  |  |  |  |  |  |  |  |  |  |
| 0.5292 | 1 | igd | 0.3264 | G | \|\| id:ebi-a-GCST90001989 | ebi-a-GCST90001989 | TRUE | A | 161479745 | 1.29E-90 | reported | 3621 | 0.02548 | rs1801274 |  |  |  |  |  |  |  |  |  |  |  |  |  |  |  |
| -0.2069 | 16 | igd | 0.3262 | T | \|\| id:ebi-a-GCST90001932 | ebi-a-GCST90001932 | TRUE | C | 423696 | 4.02E-13 | reported | 2920 | 0.02839 | rs1573735 |  |  |  |  |  |  |  |  |  |  |  |  |  |  |  |
| -0.2582 | 1 | igd | 0.3262 | G | \|\| id:ebi-a-GCST90001994 | ebi-a-GCST90001994 | TRUE | A | 161479745 | 1.06E-22 | reported | 3590 | 0.02615 | rs1801274 |  |  |  |  |  |  |  |  |  |  |  |  |  |  |  |
| -0.4504 | 1 | igd | 0.3262 | G | \|\| id:ebi-a-GCST90001997 | ebi-a-GCST90001997 | TRUE | A | 161479745 | 2.10E-64 | reported | 3590 | 0.02605 | rs1801274 |  |  |  |  |  |  |  |  |  |  |  |  |  |  |  |
| -0.41 | 1 | igd | 0.3262 | G | \|\| id:ebi-a-GCST90001995 | ebi-a-GCST90001995 | TRUE | A | 161479745 | 4.45E-54 | reported | 3590 | 0.02603 | rs1801274 |  |  |  |  |  |  |  |  |  |  |  |  |  |  |  |
| 0.4371 | 1 | igd | 0.3258 | G | \|\| id:ebi-a-GCST90001980 | ebi-a-GCST90001980 | TRUE | A | 161479745 | 1.62E-62 | reported | 3629 | 0.02569 | rs1801274 |  |  |  |  |  |  |  |  |  |  |  |  |  |  |  |
| 0.5089 | 1 | igd | 0.3258 | G | \|\| id:ebi-a-GCST90001985 | ebi-a-GCST90001985 | TRUE | A | 161479745 | 7.00E-84 | reported | 3629 | 0.02554 | rs1801274 |  |  |  |  |  |  |  |  |  |  |  |  |  |  |  |
| -0.2745 | 16 | igd | 0.3258 | G | \|\| id:ebi-a-GCST90001907 | ebi-a-GCST90001907 | TRUE | A | 423423 | 1.65E-21 | reported | 2910 | 0.02859 | rs5020353 |  |  |  |  |  |  |  |  |  |  |  |  |  |  |  |
| -0.209 | 16 | igd | 0.3258 | G | \|\| id:ebi-a-GCST90001908 | ebi-a-GCST90001908 | TRUE | A | 423423 | 3.01E-13 | reported | 2910 | 0.02852 | rs5020353 |  |  |  |  |  |  |  |  |  |  |  |  |  |  |  |
| 0.1963 | 13 | igd | 0.3256 | G | \|\| id:ebi-a-GCST90001412 | ebi-a-GCST90001412 | TRUE | A | 108993494 | 5.62E-14 | reported | 3656 | 0.02601 | rs12874404 |  |  |  |  |  |  |  |  |  |  |  |  |  |  |  |
| 0.1691 | 13 | igd | 0.3256 | G | \|\| id:ebi-a-GCST90001418 | ebi-a-GCST90001418 | TRUE | A | 108993494 | 2.04E-10 | reported | 3656 | 0.02653 | rs12874404 |  |  |  |  |  |  |  |  |  |  |  |  |  |  |  |
| 0.2335 | 13 | igd | 0.3256 | G | \|\| id:ebi-a-GCST90001439 | ebi-a-GCST90001439 | TRUE | A | 108993494 | 1.09E-18 | reported | 3656 | 0.02632 | rs12874404 |  |  |  |  |  |  |  |  |  |  |  |  |  |  |  |
| 0.2114 | 13 | igd | 0.3256 | G | \|\| id:ebi-a-GCST90001442 | ebi-a-GCST90001442 | TRUE | A | 108993494 | 8.09E-16 | reported | 3656 | 0.02614 | rs12874404 |  |  |  |  |  |  |  |  |  |  |  |  |  |  |  |
| 0.2065 | 2 | igd | 0.3256 | A | \|\| id:ebi-a-GCST90001963 | ebi-a-GCST90001963 | TRUE | G | 203032111 | 4.61E-12 | reported | 2872 | 0.02973 | rs72940547 |  |  |  |  |  |  |  |  |  |  |  |  |  |  |  |
| -0.1586 | 1 | igd | 0.3251 | G | \|\| id:ebi-a-GCST90001806 | ebi-a-GCST90001806 | TRUE | A | 161479745 | 3.13E-09 | reported | 3656 | 0.02671 | rs1801274 |  |  |  |  |  |  |  |  |  |  |  |  |  |  |  |
| -0.1805 | 1 | igd | 0.325 | G | \|\| id:ebi-a-GCST90001704 | ebi-a-GCST90001704 | TRUE | A | 161479745 | 2.29E-11 | reported | 3657 | 0.02691 | rs1801274 |  |  |  |  |  |  |  |  |  |  |  |  |  |  |  |
| -0.2208 | 1 | igd | 0.325 | G | \|\| id:ebi-a-GCST90001798 | ebi-a-GCST90001798 | TRUE | A | 161479745 | 7.07E-17 | reported | 3657 | 0.02632 | rs1801274 |  |  |  |  |  |  |  |  |  |  |  |  |  |  |  |
| -0.2111 | 1 | igd | 0.325 | G | \|\| id:ebi-a-GCST90001802 | ebi-a-GCST90001802 | TRUE | A | 161479745 | 2.77E-15 | reported | 3657 | 0.02661 | rs1801274 |  |  |  |  |  |  |  |  |  |  |  |  |  |  |  |
| -0.2036 | 1 | igd | 0.325 | G | \|\| id:ebi-a-GCST90001804 | ebi-a-GCST90001804 | TRUE | A | 161479745 | 1.14E-14 | reported | 3657 | 0.02626 | rs1801274 |  |  |  |  |  |  |  |  |  |  |  |  |  |  |  |
| -0.2933 | 1 | igd | 0.325 | G | \|\| id:ebi-a-GCST90001808 | ebi-a-GCST90001808 | TRUE | A | 161479745 | 2.48E-28 | reported | 3657 | 0.02635 | rs1801274 |  |  |  |  |  |  |  |  |  |  |  |  |  |  |  |
| -0.1811 | 1 | igd | 0.3249 | T | \|\| id:ebi-a-GCST90001474 | ebi-a-GCST90001474 | TRUE | C | 161258765 | 3.99E-11 | reported | 3410 | 0.02733 | rs9651035 |  |  |  |  |  |  |  |  |  |  |  |  |  |  |  |
| 0.1937 | 2 | igd | 0.3244 | C | \|\| id:ebi-a-GCST90001460 | ebi-a-GCST90001460 | TRUE | T | 136580287 | 2.12E-13 | reported | 3374 | 0.02628 | rs2164210 |  |  |  |  |  |  |  |  |  |  |  |  |  |  |  |
| 0.5575 | 10 | igd | 0.3244 | A | \|\| id:ebi-a-GCST90001495 | ebi-a-GCST90001495 | TRUE | T | 97370989 | 1.67E-92 | reported | 3405 | 0.0265 | rs2296691 |  |  |  |  |  |  |  |  |  |  |  |  |  |  |  |
| 0.5243 | 10 | igd | 0.3244 | A | \|\| id:ebi-a-GCST90001660 | ebi-a-GCST90001660 | TRUE | T | 97370989 | 3.46E-83 | reported | 3408 | 0.02639 | rs2296691 |  |  |  |  |  |  |  |  |  |  |  |  |  |  |  |
| 0.5158 | 10 | igd | 0.3243 | A | \|\| id:ebi-a-GCST90001491 | ebi-a-GCST90001491 | TRUE | T | 97370989 | 8.24E-83 | reported | 3437 | 0.02603 | rs2296691 |  |  |  |  |  |  |  |  |  |  |  |  |  |  |  |
| 0.4962 | 10 | igd | 0.3243 | A | \|\| id:ebi-a-GCST90001658 | ebi-a-GCST90001658 | TRUE | T | 97370989 | 6.42E-82 | reported | 3440 | 0.02519 | rs2296691 |  |  |  |  |  |  |  |  |  |  |  |  |  |  |  |
| 0.5069 | 10 | igd | 0.3243 | A | \|\| id:ebi-a-GCST90001659 | ebi-a-GCST90001659 | TRUE | T | 97370989 | 1.46E-85 | reported | 3440 | 0.02514 | rs2296691 |  |  |  |  |  |  |  |  |  |  |  |  |  |  |  |
| 0.4503 | 10 | igd | 0.324 | A | \|\| id:ebi-a-GCST90001484 | ebi-a-GCST90001484 | TRUE | G | 97368696 | 7.93E-66 | reported | 3437 | 0.02571 | rs11591779 |  |  |  |  |  |  |  |  |  |  |  |  |  |  |  |
| 0.5902 | 10 | igd | 0.324 | A | \|\| id:ebi-a-GCST90001496 | ebi-a-GCST90001496 | TRUE | G | 97368696 | 1.05E-106 | reported | 3437 | 0.02596 | rs11591779 |  |  |  |  |  |  |  |  |  |  |  |  |  |  |  |
| 0.5761 | 10 | igd | 0.324 | A | \|\| id:ebi-a-GCST90001497 | ebi-a-GCST90001497 | TRUE | G | 97368696 | 3.80E-104 | reported | 3437 | 0.02568 | rs11591779 |  |  |  |  |  |  |  |  |  |  |  |  |  |  |  |
| -0.269 | 6 | igd | 0.3239 | G | \|\| id:ebi-a-GCST90002108 | ebi-a-GCST90002108 | TRUE | A | 32109979 | 3.91E-10 | reported | 1579 | 0.04271 | rs204999 |  |  |  |  |  |  |  |  |  |  |  |  |  |  |  |
| -0.3136 | 22 | igd | 0.3238 | C | \|\| id:ebi-a-GCST90001702 | ebi-a-GCST90001702 | TRUE | T | 43472999 | 1.61E-31 | reported | 3657 | 0.0266 | rs5759140 |  |  |  |  |  |  |  |  |  |  |  |  |  |  |  |
| -0.3129 | 22 | igd | 0.3238 | C | \|\| id:ebi-a-GCST90001703 | ebi-a-GCST90001703 | TRUE | T | 43472999 | 2.22E-31 | reported | 3657 | 0.0266 | rs5759140 |  |  |  |  |  |  |  |  |  |  |  |  |  |  |  |
| -0.2934 | 22 | igd | 0.3238 | C | \|\| id:ebi-a-GCST90001704 | ebi-a-GCST90001704 | TRUE | T | 43472999 | 8.08E-28 | reported | 3657 | 0.02662 | rs5759140 |  |  |  |  |  |  |  |  |  |  |  |  |  |  |  |
| -0.2863 | 22 | igd | 0.3238 | C | \|\| id:ebi-a-GCST90001710 | ebi-a-GCST90001710 | TRUE | T | 43472999 | 1.33E-26 | reported | 3657 | 0.02661 | rs5759140 |  |  |  |  |  |  |  |  |  |  |  |  |  |  |  |
| -0.2868 | 22 | igd | 0.3238 | C | \|\| id:ebi-a-GCST90001711 | ebi-a-GCST90001711 | TRUE | T | 43472999 | 8.03E-27 | reported | 3657 | 0.02654 | rs5759140 |  |  |  |  |  |  |  |  |  |  |  |  |  |  |  |
| 0.8893 | 10 | igd | 0.3238 | T | \|\| id:ebi-a-GCST90002030 | ebi-a-GCST90002030 | TRUE | G | 97692620 | 1.00E-200 | reported | 2920 | 0.02435 | rs11188526 |  |  |  |  |  |  |  |  |  |  |  |  |  |  |  |
| 0.7702 | 10 | igd | 0.3238 | T | \|\| id:ebi-a-GCST90002031 | ebi-a-GCST90002031 | TRUE | G | 97692620 | 6.25E-164 | reported | 2920 | 0.02644 | rs11188526 |  |  |  |  |  |  |  |  |  |  |  |  |  |  |  |
| 0.7727 | 10 | igd | 0.3238 | T | \|\| id:ebi-a-GCST90002032 | ebi-a-GCST90002032 | TRUE | G | 97692620 | 2.49E-165 | reported | 2920 | 0.0264 | rs11188526 |  |  |  |  |  |  |  |  |  |  |  |  |  |  |  |
| -0.1537 | 3 | igd | 0.3227 | G | \|\| id:ebi-a-GCST90001997 | ebi-a-GCST90001997 | TRUE | C | 39323423 | 1.81E-08 | reported | 3590 | 0.02724 | rs11917223 |  |  |  |  |  |  |  |  |  |  |  |  |  |  |  |
| -0.1815 | 10 | igd | 0.3221 | T | \|\| id:ebi-a-GCST90001935 | ebi-a-GCST90001935 | TRUE | G | 97692620 | 2.47E-11 | reported | 3434 | 0.02711 | rs11188526 |  |  |  |  |  |  |  |  |  |  |  |  |  |  |  |
| -0.1781 | 10 | igd | 0.322 | T | \|\| id:ebi-a-GCST90001940 | ebi-a-GCST90001940 | TRUE | G | 97692620 | 7.79E-11 | reported | 3435 | 0.0273 | rs11188526 |  |  |  |  |  |  |  |  |  |  |  |  |  |  |  |
| 0.6981 | 10 | igd | 0.3218 | T | \|\| id:ebi-a-GCST90001484 | ebi-a-GCST90001484 | TRUE | G | 97692620 | 1.17E-164 | reported | 3437 | 0.02415 | rs11188526 |  |  |  |  |  |  |  |  |  |  |  |  |  |  |  |
| 0.6547 | 10 | igd | 0.3218 | T | \|\| id:ebi-a-GCST90001485 | ebi-a-GCST90001485 | TRUE | G | 97692620 | 1.65E-143 | reported | 3437 | 0.02446 | rs11188526 |  |  |  |  |  |  |  |  |  |  |  |  |  |  |  |
| 0.7007 | 10 | igd | 0.3218 | T | \|\| id:ebi-a-GCST90001491 | ebi-a-GCST90001491 | TRUE | G | 97692620 | 6.93E-159 | reported | 3437 | 0.02473 | rs11188526 |  |  |  |  |  |  |  |  |  |  |  |  |  |  |  |
| 0.804 | 10 | igd | 0.3218 | T | \|\| id:ebi-a-GCST90001496 | ebi-a-GCST90001496 | TRUE | G | 97692620 | 1.00E-200 | reported | 3437 | 0.02422 | rs11188526 |  |  |  |  |  |  |  |  |  |  |  |  |  |  |  |
| 0.79 | 10 | igd | 0.3218 | T | \|\| id:ebi-a-GCST90001497 | ebi-a-GCST90001497 | TRUE | G | 97692620 | 1.00E-200 | reported | 3437 | 0.02396 | rs11188526 |  |  |  |  |  |  |  |  |  |  |  |  |  |  |  |
| 0.7305 | 10 | igd | 0.3217 | T | \|\| id:ebi-a-GCST90001658 | ebi-a-GCST90001658 | TRUE | G | 97692620 | 4.83E-188 | reported | 3440 | 0.02344 | rs11188526 |  |  |  |  |  |  |  |  |  |  |  |  |  |  |  |
| 0.7423 | 10 | igd | 0.3217 | T | \|\| id:ebi-a-GCST90001659 | ebi-a-GCST90001659 | TRUE | G | 97692620 | 7.03E-195 | reported | 3440 | 0.02333 | rs11188526 |  |  |  |  |  |  |  |  |  |  |  |  |  |  |  |
| -0.1947 | 22 | igd | 0.3215 | T | \|\| id:ebi-a-GCST90001830 | ebi-a-GCST90001830 | TRUE | C | 42602139 | 5.86E-14 | reported | 3648 | 0.02582 | rs16986035 |  |  |  |  |  |  |  |  |  |  |  |  |  |  |  |
| 0.6209 | 10 | igd | 0.3214 | T | \|\| id:ebi-a-GCST90001483 | ebi-a-GCST90001483 | TRUE | G | 97692620 | 1.17E-125 | reported | 3405 | 0.02496 | rs11188526 |  |  |  |  |  |  |  |  |  |  |  |  |  |  |  |
| 0.7483 | 10 | igd | 0.3214 | T | \|\| id:ebi-a-GCST90001495 | ebi-a-GCST90001495 | TRUE | G | 97692620 | 1.85E-174 | reported | 3405 | 0.02504 | rs11188526 |  |  |  |  |  |  |  |  |  |  |  |  |  |  |  |
| 0.7348 | 10 | igd | 0.3213 | T | \|\| id:ebi-a-GCST90001660 | ebi-a-GCST90001660 | TRUE | G | 97692620 | 9.02E-172 | reported | 3408 | 0.02481 | rs11188526 |  |  |  |  |  |  |  |  |  |  |  |  |  |  |  |
| -0.1645 | 1 | igd | 0.3213 | T | \|\| id:ebi-a-GCST90001856 | ebi-a-GCST90001856 | TRUE | C | 167143281 | 1.93E-08 | reported | 2919 | 0.02919 | rs12029632 |  |  |  |  |  |  |  |  |  |  |  |  |  |  |  |
| -0.189 | 1 | igd | 0.3213 | T | \|\| id:ebi-a-GCST90001860 | ebi-a-GCST90001860 | TRUE | C | 167143281 | 1.20E-10 | reported | 2919 | 0.02924 | rs12029632 |  |  |  |  |  |  |  |  |  |  |  |  |  |  |  |
| 0.1824 | 10 | igd | 0.3212 | A | \|\| id:ebi-a-GCST90001491 | ebi-a-GCST90001491 | TRUE | G | 95256275 | 3.05E-11 | reported | 3437 | 0.02737 | rs1880263 |  |  |  |  |  |  |  |  |  |  |  |  |  |  |  |
| 0.1944 | 10 | igd | 0.3212 | A | \|\| id:ebi-a-GCST90001496 | ebi-a-GCST90001496 | TRUE | G | 95256275 | 2.80E-12 | reported | 3437 | 0.02772 | rs1880263 |  |  |  |  |  |  |  |  |  |  |  |  |  |  |  |
| 0.1848 | 10 | igd | 0.3212 | A | \|\| id:ebi-a-GCST90001660 | ebi-a-GCST90001660 | TRUE | G | 95256275 | 3.26E-11 | reported | 3408 | 0.02776 | rs1880263 |  |  |  |  |  |  |  |  |  |  |  |  |  |  |  |
| -0.5982 | 22 | igd | 0.3212 | T | \|\| id:ebi-a-GCST90001702 | ebi-a-GCST90001702 | TRUE | C | 42602139 | 2.36E-113 | reported | 3657 | 0.02551 | rs16986035 |  |  |  |  |  |  |  |  |  |  |  |  |  |  |  |
| -0.5934 | 22 | igd | 0.3212 | T | \|\| id:ebi-a-GCST90001703 | ebi-a-GCST90001703 | TRUE | C | 42602139 | 1.59E-111 | reported | 3657 | 0.02554 | rs16986035 |  |  |  |  |  |  |  |  |  |  |  |  |  |  |  |
| -0.5785 | 22 | igd | 0.3212 | T | \|\| id:ebi-a-GCST90001704 | ebi-a-GCST90001704 | TRUE | C | 42602139 | 4.54E-106 | reported | 3657 | 0.02559 | rs16986035 |  |  |  |  |  |  |  |  |  |  |  |  |  |  |  |
| -0.5775 | 22 | igd | 0.3212 | T | \|\| id:ebi-a-GCST90001710 | ebi-a-GCST90001710 | TRUE | C | 42602139 | 8.39E-106 | reported | 3657 | 0.02557 | rs16986035 |  |  |  |  |  |  |  |  |  |  |  |  |  |  |  |
| -0.5798 | 22 | igd | 0.3212 | T | \|\| id:ebi-a-GCST90001711 | ebi-a-GCST90001711 | TRUE | C | 42602139 | 2.28E-107 | reported | 3657 | 0.02547 | rs16986035 |  |  |  |  |  |  |  |  |  |  |  |  |  |  |  |
| -0.1724 | 1 | igd | 0.3212 | T | \|\| id:ebi-a-GCST90001866 | ebi-a-GCST90001866 | TRUE | C | 167143281 | 2.31E-09 | reported | 2914 | 0.02876 | rs12029632 |  |  |  |  |  |  |  |  |  |  |  |  |  |  |  |
| 0.1649 | 10 | igd | 0.3211 | A | \|\| id:ebi-a-GCST90001489 | ebi-a-GCST90001489 | TRUE | G | 95256275 | 3.52E-09 | reported | 3405 | 0.02784 | rs1880263 |  |  |  |  |  |  |  |  |  |  |  |  |  |  |  |
| -0.1823 | 22 | igd | 0.3211 | T | \|\| id:ebi-a-GCST90001701 | ebi-a-GCST90001701 | TRUE | C | 42602139 | 3.89E-12 | reported | 3592 | 0.02618 | rs16986035 |  |  |  |  |  |  |  |  |  |  |  |  |  |  |  |
| -0.4891 | 6 | igd | 0.3211 | A | \|\| id:ebi-a-GCST90002111 | ebi-a-GCST90002111 | TRUE | C | 32479430 | 1.20E-11 | reported | 1635 | 0.07162 | rs114085331 |  |  |  |  |  |  |  |  |  |  |  |  |  |  |  |
| -0.5777 | 6 | igd | 0.321 | A | \|\| id:ebi-a-GCST90002110 | ebi-a-GCST90002110 | TRUE | C | 32479430 | 2.67E-15 | reported | 1634 | 0.07237 | rs114085331 |  |  |  |  |  |  |  |  |  |  |  |  |  |  |  |
| 0.2755 | 1 | igd | 0.3209 | G | \|\| id:ebi-a-GCST90002089 | ebi-a-GCST90002089 | TRUE | A | 161479745 | 1.20E-20 | reported | 2850 | 0.02934 | rs1801274 |  |  |  |  |  |  |  |  |  |  |  |  |  |  |  |
| 0.248 | 1 | igd | 0.3209 | G | \|\| id:ebi-a-GCST90002090 | ebi-a-GCST90002090 | TRUE | A | 161479745 | 1.36E-16 | reported | 2850 | 0.02982 | rs1801274 |  |  |  |  |  |  |  |  |  |  |  |  |  |  |  |
| 0.2581 | 1 | igd | 0.3209 | G | \|\| id:ebi-a-GCST90002017 | ebi-a-GCST90002017 | TRUE | A | 161479745 | 3.69E-18 | reported | 2850 | 0.02951 | rs1801274 |  |  |  |  |  |  |  |  |  |  |  |  |  |  |  |
| -0.2643 | 6 | igd | 0.3207 | A | \|\| id:ebi-a-GCST90002109 | ebi-a-GCST90002109 | TRUE | C | 32298814 | 1.43E-09 | reported | 1578 | 0.04342 | rs3129949 |  |  |  |  |  |  |  |  |  |  |  |  |  |  |  |
| 0.2347 | 6 | igd | 0.3205 | C | \|\| id:ebi-a-GCST90002108 | ebi-a-GCST90002108 | TRUE | T | 32739888 | 1.78E-08 | reported | 1579 | 0.04145 | rs1383265 |  |  |  |  |  |  |  |  |  |  |  |  |  |  |  |
| -0.5121 | 6 | igd | 0.3203 | A | \|\| id:ebi-a-GCST90002105 | ebi-a-GCST90002105 | TRUE | C | 32479430 | 1.20E-18 | reported | 2872 | 0.05771 | rs114085331 |  |  |  |  |  |  |  |  |  |  |  |  |  |  |  |
| 0.3362 | 6 | igd | 0.3203 | G | \|\| id:ebi-a-GCST90002105 | ebi-a-GCST90002105 | TRUE | A | 32353166 | 2.02E-24 | reported | 2872 | 0.03266 | rs9268468 |  |  |  |  |  |  |  |  |  |  |  |  |  |  |  |
| -0.2852 | 6 | igd | 0.3202 | A | \|\| id:ebi-a-GCST90001752 | ebi-a-GCST90001752 | TRUE | C | 32479430 | 3.26E-08 | reported | 3657 | 0.05148 | rs114085331 |  |  |  |  |  |  |  |  |  |  |  |  |  |  |  |
| 0.2846 | 16 | igd | 0.3198 | A | \|\| id:ebi-a-GCST90002088 | ebi-a-GCST90002088 | TRUE | C | 31351340 | 1.07E-22 | reported | 2872 | 0.02878 | rs28821928 |  |  |  |  |  |  |  |  |  |  |  |  |  |  |  |
| -0.2464 | 8 | igd | 0.3192 | T | \|\| id:ebi-a-GCST90002078 | ebi-a-GCST90002078 | TRUE | A | 6826043 | 1.59E-17 | reported | 3112 | 0.02875 | rs2738144 |  |  |  |  |  |  |  |  |  |  |  |  |  |  |  |
| 0.3381 | 6 | igd | 0.319 | T | \|\| id:ebi-a-GCST90002111 | ebi-a-GCST90002111 | TRUE | C | 32445916 | 2.04E-15 | reported | 1635 | 0.04217 | rs28895267 |  |  |  |  |  |  |  |  |  |  |  |  |  |  |  |
| -0.2784 | 6 | igd | 0.3188 | A | \|\| id:ebi-a-GCST90002109 | ebi-a-GCST90002109 | TRUE | G | 31834398 | 3.10E-10 | reported | 1578 | 0.04395 | rs613165 |  |  |  |  |  |  |  |  |  |  |  |  |  |  |  |
| -0.163 | 20 | igd | 0.317 | C | \|\| id:ebi-a-GCST90001980 | ebi-a-GCST90001980 | TRUE | T | 44796128 | 7.60E-10 | reported | 3629 | 0.02642 | rs2425759 |  |  |  |  |  |  |  |  |  |  |  |  |  |  |  |
| -0.303 | 6 | igd | 0.3167 | T | \|\| id:ebi-a-GCST90002110 | ebi-a-GCST90002110 | TRUE | C | 31824315 | 1.25E-11 | reported | 1634 | 0.04441 | rs2395079 |  |  |  |  |  |  |  |  |  |  |  |  |  |  |  |
| -0.3112 | 1 | igd | 0.3166 | G | \|\| id:ebi-a-GCST90001799 | ebi-a-GCST90001799 | TRUE | A | 161479745 | 1.06E-20 | reported | 1838 | 0.03296 | rs1801274 |  |  |  |  |  |  |  |  |  |  |  |  |  |  |  |
| 0.1521 | 6 | igd | 0.3162 | T | \|\| id:ebi-a-GCST90001762 | ebi-a-GCST90001762 | TRUE | C | 14091682 | 1.64E-08 | reported | 3658 | 0.02688 | rs72836542 |  |  |  |  |  |  |  |  |  |  |  |  |  |  |  |
| -0.5404 | 6 | igd | 0.3162 | T | \|\| id:ebi-a-GCST90002105 | ebi-a-GCST90002105 | TRUE | G | 32635998 | 2.13E-52 | reported | 2872 | 0.03476 | rs9274623 |  |  |  |  |  |  |  |  |  |  |  |  |  |  |  |
| 0.1645 | 6 | igd | 0.3161 | T | \|\| id:ebi-a-GCST90001747 | ebi-a-GCST90001747 | TRUE | C | 14091682 | 9.87E-10 | reported | 3657 | 0.02685 | rs72836542 |  |  |  |  |  |  |  |  |  |  |  |  |  |  |  |
| 0.1494 | 6 | igd | 0.3161 | T | \|\| id:ebi-a-GCST90001751 | ebi-a-GCST90001751 | TRUE | C | 14091682 | 2.45E-08 | reported | 3657 | 0.02674 | rs72836542 |  |  |  |  |  |  |  |  |  |  |  |  |  |  |  |
| 0.1752 | 6 | igd | 0.3161 | T | \|\| id:ebi-a-GCST90001752 | ebi-a-GCST90001752 | TRUE | C | 14091682 | 7.20E-11 | reported | 3657 | 0.0268 | rs72836542 |  |  |  |  |  |  |  |  |  |  |  |  |  |  |  |
| 0.1572 | 6 | igd | 0.3161 | T | \|\| id:ebi-a-GCST90001759 | ebi-a-GCST90001759 | TRUE | C | 14091682 | 5.42E-09 | reported | 3657 | 0.02688 | rs72836542 |  |  |  |  |  |  |  |  |  |  |  |  |  |  |  |
| -0.3524 | 6 | igd | 0.3157 | G | \|\| id:ebi-a-GCST90002108 | ebi-a-GCST90002108 | TRUE | A | 31768800 | 1.89E-15 | reported | 1579 | 0.04389 | rs523727 |  |  |  |  |  |  |  |  |  |  |  |  |  |  |  |
| -0.3679 | 6 | igd | 0.3153 | C | \|\| id:ebi-a-GCST90002105 | ebi-a-GCST90002105 | TRUE | T | 32316312 | 3.43E-25 | reported | 2872 | 0.03514 | rs969892 |  |  |  |  |  |  |  |  |  |  |  |  |  |  |  |
| 0.3592 | 6 | igd | 0.3147 | G | \|\| id:ebi-a-GCST90002112 | ebi-a-GCST90002112 | TRUE | A | 32287496 | 1.25E-17 | reported | 1635 | 0.04154 | rs580921 |  |  |  |  |  |  |  |  |  |  |  |  |  |  |  |
| -0.2759 | 6 | igd | 0.3144 | C | \|\| id:ebi-a-GCST90002108 | ebi-a-GCST90002108 | TRUE | T | 31106177 | 2.53E-10 | reported | 1579 | 0.04333 | rs1265098 |  |  |  |  |  |  |  |  |  |  |  |  |  |  |  |
| 0.413 | 6 | igd | 0.3144 | C | \|\| id:ebi-a-GCST90002112 | ebi-a-GCST90002112 | TRUE | G | 32440508 | 2.29E-23 | reported | 1635 | 0.04083 | rs9269072 |  |  |  |  |  |  |  |  |  |  |  |  |  |  |  |
| -0.4554 | 6 | igd | 0.3141 | T | \|\| id:ebi-a-GCST90002112 | ebi-a-GCST90002112 | TRUE | G | 32635998 | 7.00E-25 | reported | 1635 | 0.04349 | rs9274623 |  |  |  |  |  |  |  |  |  |  |  |  |  |  |  |
| 0.236 | 6 | igd | 0.3138 | T | \|\| id:ebi-a-GCST90002108 | ebi-a-GCST90002108 | TRUE | C | 32900787 | 1.52E-08 | reported | 1579 | 0.04148 | rs3129299 |  |  |  |  |  |  |  |  |  |  |  |  |  |  |  |
| -0.285 | 6 | igd | 0.3136 | T | \|\| id:ebi-a-GCST90002110 | ebi-a-GCST90002110 | TRUE | C | 31883679 | 1.22E-10 | reported | 1634 | 0.04399 | rs9267673 |  |  |  |  |  |  |  |  |  |  |  |  |  |  |  |
| 0.1568 | 20 | igd | 0.3131 | G | \|\| id:ebi-a-GCST90001399 | ebi-a-GCST90001399 | TRUE | A | 44650818 | 1.82E-08 | reported | 3657 | 0.0278 | rs6130997 |  |  |  |  |  |  |  |  |  |  |  |  |  |  |  |
| -0.1898 | 20 | igd | 0.3131 | G | \|\| id:ebi-a-GCST90001802 | ebi-a-GCST90001802 | TRUE | A | 44650818 | 2.31E-12 | reported | 3657 | 0.02697 | rs6130997 |  |  |  |  |  |  |  |  |  |  |  |  |  |  |  |
| -0.2376 | 20 | igd | 0.3131 | G | \|\| id:ebi-a-GCST90001804 | ebi-a-GCST90001804 | TRUE | A | 44650818 | 5.11E-19 | reported | 3657 | 0.02652 | rs6130997 |  |  |  |  |  |  |  |  |  |  |  |  |  |  |  |
| -0.2011 | 20 | igd | 0.3131 | G | \|\| id:ebi-a-GCST90001805 | ebi-a-GCST90001805 | TRUE | A | 44650818 | 1.08E-13 | reported | 3657 | 0.02696 | rs6130997 |  |  |  |  |  |  |  |  |  |  |  |  |  |  |  |
| -0.2457 | 20 | igd | 0.3128 | T | \|\| id:ebi-a-GCST90001798 | ebi-a-GCST90001798 | TRUE | C | 44654508 | 3.94E-20 | reported | 3657 | 0.02658 | rs6073990 |  |  |  |  |  |  |  |  |  |  |  |  |  |  |  |
| -0.1904 | 20 | igd | 0.3128 | T | \|\| id:ebi-a-GCST90001800 | ebi-a-GCST90001800 | TRUE | C | 44654508 | 2.42E-12 | reported | 3656 | 0.02708 | rs6073990 |  |  |  |  |  |  |  |  |  |  |  |  |  |  |  |
| -0.1935 | 20 | igd | 0.3128 | T | \|\| id:ebi-a-GCST90001806 | ebi-a-GCST90001806 | TRUE | C | 44654508 | 7.80E-13 | reported | 3656 | 0.02692 | rs6073990 |  |  |  |  |  |  |  |  |  |  |  |  |  |  |  |
| -0.234 | 20 | igd | 0.3128 | T | \|\| id:ebi-a-GCST90001808 | ebi-a-GCST90001808 | TRUE | C | 44654508 | 4.00E-18 | reported | 3657 | 0.02682 | rs6073990 |  |  |  |  |  |  |  |  |  |  |  |  |  |  |  |
| -0.1691 | 16 | igd | 0.3126 | C | \|\| id:ebi-a-GCST90002090 | ebi-a-GCST90002090 | TRUE | G | 48555929 | 2.59E-08 | reported | 2850 | 0.03028 | rs9930644 |  |  |  |  |  |  |  |  |  |  |  |  |  |  |  |
| -0.2807 | 6 | igd | 0.3125 | C | \|\| id:ebi-a-GCST90002111 | ebi-a-GCST90002111 | TRUE | T | 30340678 | 2.70E-10 | reported | 1635 | 0.04418 | rs115316399 |  |  |  |  |  |  |  |  |  |  |  |  |  |  |  |
| -0.1721 | 6 | igd | 0.3117 | A | \|\| id:ebi-a-GCST90001820 | ebi-a-GCST90001820 | TRUE | G | 27767640 | 1.18E-08 | reported | 3656 | 0.03011 | rs200474 |  |  |  |  |  |  |  |  |  |  |  |  |  |  |  |
| 0.2559 | 6 | igd | 0.3116 | T | \|\| id:ebi-a-GCST90002105 | ebi-a-GCST90002105 | TRUE | C | 32900787 | 4.27E-16 | reported | 2872 | 0.03129 | rs3129299 |  |  |  |  |  |  |  |  |  |  |  |  |  |  |  |
| -0.2818 | 6 | igd | 0.3106 | T | \|\| id:ebi-a-GCST90002108 | ebi-a-GCST90002108 | TRUE | C | 31095210 | 1.57E-10 | reported | 1579 | 0.04376 | rs9263706 |  |  |  |  |  |  |  |  |  |  |  |  |  |  |  |
| 0.2221 | 19 | igd | 0.3096 | C | \|\| id:ebi-a-GCST90001954 | ebi-a-GCST90001954 | TRUE | G | 52181678 | 3.57E-08 | reported | 1555 | 0.04011 | rs28616281 |  |  |  |  |  |  |  |  |  |  |  |  |  |  |  |
| -0.1884 | 6 | igd | 0.3095 | T | \|\| id:ebi-a-GCST90001820 | ebi-a-GCST90001820 | TRUE | C | 31824315 | 3.66E-09 | reported | 3656 | 0.03186 | rs2395079 |  |  |  |  |  |  |  |  |  |  |  |  |  |  |  |
| 0.1667 | 19 | igd | 0.3094 | A | \|\| id:ebi-a-GCST90001746 | ebi-a-GCST90001746 | TRUE | G | 49915911 | 2.66E-10 | reported | 3657 | 0.02632 | rs34486765 |  |  |  |  |  |  |  |  |  |  |  |  |  |  |  |
| 0.1684 | 19 | igd | 0.3094 | A | \|\| id:ebi-a-GCST90001751 | ebi-a-GCST90001751 | TRUE | G | 49915911 | 1.75E-10 | reported | 3657 | 0.02631 | rs34486765 |  |  |  |  |  |  |  |  |  |  |  |  |  |  |  |
| 0.167 | 19 | igd | 0.3094 | A | \|\| id:ebi-a-GCST90001752 | ebi-a-GCST90001752 | TRUE | G | 49915911 | 2.90E-10 | reported | 3657 | 0.02642 | rs34486765 |  |  |  |  |  |  |  |  |  |  |  |  |  |  |  |
| 0.1489 | 19 | igd | 0.3094 | A | \|\| id:ebi-a-GCST90001759 | ebi-a-GCST90001759 | TRUE | G | 49915911 | 2.00E-08 | reported | 3657 | 0.02648 | rs34486765 |  |  |  |  |  |  |  |  |  |  |  |  |  |  |  |
| 0.1839 | 19 | igd | 0.3094 | A | \|\| id:ebi-a-GCST90001760 | ebi-a-GCST90001760 | TRUE | G | 49915911 | 3.58E-12 | reported | 3657 | 0.02636 | rs34486765 |  |  |  |  |  |  |  |  |  |  |  |  |  |  |  |
| 0.1471 | 19 | igd | 0.3093 | A | \|\| id:ebi-a-GCST90001762 | ebi-a-GCST90001762 | TRUE | G | 49915911 | 2.95E-08 | reported | 3658 | 0.02647 | rs34486765 |  |  |  |  |  |  |  |  |  |  |  |  |  |  |  |
| 0.1682 | 19 | igd | 0.3092 | A | \|\| id:ebi-a-GCST90001743 | ebi-a-GCST90001743 | TRUE | G | 49915911 | 2.38E-10 | reported | 3660 | 0.02648 | rs34486765 |  |  |  |  |  |  |  |  |  |  |  |  |  |  |  |
| 0.3661 | 6 | igd | 0.3091 | C | \|\| id:ebi-a-GCST90002110 | ebi-a-GCST90002110 | TRUE | T | 32443665 | 1.22E-16 | reported | 1634 | 0.04374 | rs28895241 |  |  |  |  |  |  |  |  |  |  |  |  |  |  |  |
| -0.1769 | 6 | igd | 0.3087 | G | \|\| id:ebi-a-GCST90001820 | ebi-a-GCST90001820 | TRUE | A | 32289594 | 3.96E-08 | reported | 3656 | 0.03214 | rs570963 |  |  |  |  |  |  |  |  |  |  |  |  |  |  |  |
| 0.2396 | 6 | igd | 0.3081 | A | \|\| id:ebi-a-GCST90002110 | ebi-a-GCST90002110 | TRUE | G | 32972887 | 6.01E-09 | reported | 1634 | 0.04098 | rs376892 |  |  |  |  |  |  |  |  |  |  |  |  |  |  |  |
| -0.2529 | 10 | igd | 0.308 | C | \|\| id:ebi-a-GCST90002031 | ebi-a-GCST90002031 | TRUE | T | 97286853 | 1.76E-16 | reported | 2920 | 0.03052 | rs10882610 |  |  |  |  |  |  |  |  |  |  |  |  |  |  |  |
| -0.1949 | 22 | igd | 0.3078 | A | \|\| id:ebi-a-GCST90001702 | ebi-a-GCST90001702 | TRUE | G | 39594580 | 1.99E-12 | reported | 3657 | 0.02761 | rs6001490 |  |  |  |  |  |  |  |  |  |  |  |  |  |  |  |
| -0.1924 | 22 | igd | 0.3078 | A | \|\| id:ebi-a-GCST90001703 | ebi-a-GCST90001703 | TRUE | G | 39594580 | 3.79E-12 | reported | 3657 | 0.0276 | rs6001490 |  |  |  |  |  |  |  |  |  |  |  |  |  |  |  |
| -0.1876 | 22 | igd | 0.3078 | A | \|\| id:ebi-a-GCST90001704 | ebi-a-GCST90001704 | TRUE | G | 39594580 | 1.18E-11 | reported | 3657 | 0.02757 | rs6001490 |  |  |  |  |  |  |  |  |  |  |  |  |  |  |  |
| -0.1967 | 22 | igd | 0.3078 | A | \|\| id:ebi-a-GCST90001710 | ebi-a-GCST90001710 | TRUE | G | 39594580 | 1.07E-12 | reported | 3657 | 0.02753 | rs6001490 |  |  |  |  |  |  |  |  |  |  |  |  |  |  |  |
| -0.1859 | 22 | igd | 0.3078 | A | \|\| id:ebi-a-GCST90001711 | ebi-a-GCST90001711 | TRUE | G | 39594580 | 1.53E-11 | reported | 3657 | 0.02748 | rs6001490 |  |  |  |  |  |  |  |  |  |  |  |  |  |  |  |
| -0.1719 | 16 | igd | 0.3077 | T | \|\| id:ebi-a-GCST90002090 | ebi-a-GCST90002090 | TRUE | A | 48666310 | 1.75E-08 | reported | 2850 | 0.03042 | rs28432694 |  |  |  |  |  |  |  |  |  |  |  |  |  |  |  |
| -0.1647 | 16 | igd | 0.3072 | G | \|\| id:ebi-a-GCST90001929 | ebi-a-GCST90001929 | TRUE | A | 423809 | 8.69E-09 | reported | 2920 | 0.02853 | rs117037628 |  |  |  |  |  |  |  |  |  |  |  |  |  |  |  |
| 0.1658 | 3 | igd | 0.3072 | A | \|\| id:ebi-a-GCST90001997 | ebi-a-GCST90001997 | TRUE | T | 39486923 | 1.45E-09 | reported | 3590 | 0.02734 | rs2088667 |  |  |  |  |  |  |  |  |  |  |  |  |  |  |  |
| 0.1479 | 5 | igd | 0.3067 | T | \|\| id:ebi-a-GCST90001993 | ebi-a-GCST90001993 | TRUE | C | 140004816 | 3.34E-08 | reported | 3629 | 0.02672 | rs778582 |  |  |  |  |  |  |  |  |  |  |  |  |  |  |  |
| 0.3573 | 6 | igd | 0.3061 | T | \|\| id:ebi-a-GCST90002109 | ebi-a-GCST90002109 | TRUE | C | 32376788 | 1.94E-17 | reported | 1578 | 0.04156 | rs3763317 |  |  |  |  |  |  |  |  |  |  |  |  |  |  |  |
| 0.5007 | 6 | igd | 0.3059 | T | \|\| id:ebi-a-GCST90002108 | ebi-a-GCST90002108 | TRUE | C | 32376788 | 6.25E-33 | reported | 1579 | 0.04094 | rs3763317 |  |  |  |  |  |  |  |  |  |  |  |  |  |  |  |
| -0.2436 | 6 | igd | 0.3057 | C | \|\| id:ebi-a-GCST90002105 | ebi-a-GCST90002105 | TRUE | T | 31106177 | 9.29E-12 | reported | 2872 | 0.03559 | rs1265098 |  |  |  |  |  |  |  |  |  |  |  |  |  |  |  |
| -0.2575 | 19 | igd | 0.305 | C | \|\| id:ebi-a-GCST90001951 | ebi-a-GCST90001951 | TRUE | G | 51519346 | 1.15E-09 | reported | 1464 | 0.04203 | rs1061368 |  |  |  |  |  |  |  |  |  |  |  |  |  |  |  |
| -0.3812 | 6 | igd | 0.3037 | G | \|\| id:ebi-a-GCST90002112 | ebi-a-GCST90002112 | TRUE | A | 31938107 | 3.90E-17 | reported | 1635 | 0.0448 | rs474534 |  |  |  |  |  |  |  |  |  |  |  |  |  |  |  |
| -0.2605 | 20 | igd | 0.3034 | T | \|\| id:ebi-a-GCST90001801 | ebi-a-GCST90001801 | TRUE | C | 44654508 | 1.23E-12 | reported | 1836 | 0.03643 | rs6073990 |  |  |  |  |  |  |  |  |  |  |  |  |  |  |  |
| -0.1972 | 6 | igd | 0.3033 | T | \|\| id:ebi-a-GCST90001820 | ebi-a-GCST90001820 | TRUE | G | 32298484 | 7.40E-10 | reported | 3656 | 0.03195 | rs1003880 |  |  |  |  |  |  |  |  |  |  |  |  |  |  |  |
| 0.219 | 6 | igd | 0.3031 | A | \|\| id:ebi-a-GCST90002067 | ebi-a-GCST90002067 | TRUE | G | 30727704 | 1.33E-10 | reported | 2920 | 0.03397 | rs9262169 |  |  |  |  |  |  |  |  |  |  |  |  |  |  |  |
| -0.3773 | 19 | igd | 0.3025 | C | \|\| id:ebi-a-GCST90001948 | ebi-a-GCST90001948 | TRUE | G | 51519346 | 1.64E-21 | reported | 1633 | 0.03905 | rs1061368 |  |  |  |  |  |  |  |  |  |  |  |  |  |  |  |
| -0.2967 | 6 | igd | 0.3024 | G | \|\| id:ebi-a-GCST90002112 | ebi-a-GCST90002112 | TRUE | A | 31740003 | 3.24E-11 | reported | 1635 | 0.04441 | rs707931 |  |  |  |  |  |  |  |  |  |  |  |  |  |  |  |
| -0.3914 | 19 | igd | 0.3023 | C | \|\| id:ebi-a-GCST90001946 | ebi-a-GCST90001946 | TRUE | G | 51519346 | 6.75E-23 | reported | 1634 | 0.03914 | rs1061368 |  |  |  |  |  |  |  |  |  |  |  |  |  |  |  |
| -0.3527 | 19 | igd | 0.3023 | C | \|\| id:ebi-a-GCST90001949 | ebi-a-GCST90001949 | TRUE | G | 51519346 | 5.03E-19 | reported | 1634 | 0.03909 | rs1061368 |  |  |  |  |  |  |  |  |  |  |  |  |  |  |  |
| 0.1823 | 6 | igd | 0.3021 | A | \|\| id:ebi-a-GCST90002081 | ebi-a-GCST90002081 | TRUE | G | 32587717 | 1.08E-08 | reported | 3113 | 0.0318 | rs28707527 |  |  |  |  |  |  |  |  |  |  |  |  |  |  |  |
| 0.1643 | 12 | igd | 0.3019 | T | \|\| id:ebi-a-GCST90002073 | ebi-a-GCST90002073 | TRUE | C | 69855136 | 4.97E-08 | reported | 2850 | 0.03006 | rs11177682 |  |  |  |  |  |  |  |  |  |  |  |  |  |  |  |
| -0.2337 | 10 | igd | 0.3015 | G | \|\| id:ebi-a-GCST90001489 | ebi-a-GCST90001489 | TRUE | A | 97284596 | 2.72E-16 | reported | 3405 | 0.02841 | rs10882608 |  |  |  |  |  |  |  |  |  |  |  |  |  |  |  |
| -0.2722 | 10 | igd | 0.3015 | G | \|\| id:ebi-a-GCST90001495 | ebi-a-GCST90001495 | TRUE | A | 97284596 | 3.54E-21 | reported | 3405 | 0.02862 | rs10882608 |  |  |  |  |  |  |  |  |  |  |  |  |  |  |  |
| 0.2234 | 21 | igd | 0.3015 | G | \|\| id:ebi-a-GCST90002095 | ebi-a-GCST90002095 | TRUE | A | 46340843 | 4.59E-08 | reported | 1559 | 0.04067 | rs2070947 |  |  |  |  |  |  |  |  |  |  |  |  |  |  |  |
| 0.3532 | 19 | igd | 0.30061 | G | \|\| id:prot-a-439 | prot-a-439 | TRUE | A | 51701891 | 5.13E-41 | reported | 3301 | 0.0263 | rs10421385 |  |  |  |  |  |  |  |  |  |  |  |  |  |  |  |
| -0.3091 | 6 | igd | 0.3002 | T | \|\| id:ebi-a-GCST90002108 | ebi-a-GCST90002108 | TRUE | C | 31688200 | 7.97E-12 | reported | 1579 | 0.04485 | rs376510 |  |  |  |  |  |  |  |  |  |  |  |  |  |  |  |
| -0.27 | 22 | igd | 0.2997 | C | \|\| id:ebi-a-GCST90001702 | ebi-a-GCST90001702 | TRUE | G | 43155270 | 9.09E-23 | reported | 3657 | 0.02731 | rs5758918 |  |  |  |  |  |  |  |  |  |  |  |  |  |  |  |
| -0.2679 | 22 | igd | 0.2997 | C | \|\| id:ebi-a-GCST90001703 | ebi-a-GCST90001703 | TRUE | G | 43155270 | 1.92E-22 | reported | 3657 | 0.02731 | rs5758918 |  |  |  |  |  |  |  |  |  |  |  |  |  |  |  |
| -0.2513 | 22 | igd | 0.2997 | C | \|\| id:ebi-a-GCST90001704 | ebi-a-GCST90001704 | TRUE | G | 43155270 | 5.77E-20 | reported | 3657 | 0.02731 | rs5758918 |  |  |  |  |  |  |  |  |  |  |  |  |  |  |  |
| -0.2494 | 22 | igd | 0.2997 | C | \|\| id:ebi-a-GCST90001710 | ebi-a-GCST90001710 | TRUE | G | 43155270 | 9.96E-20 | reported | 3657 | 0.02729 | rs5758918 |  |  |  |  |  |  |  |  |  |  |  |  |  |  |  |
| -0.2511 | 22 | igd | 0.2997 | C | \|\| id:ebi-a-GCST90001711 | ebi-a-GCST90001711 | TRUE | G | 43155270 | 4.51E-20 | reported | 3657 | 0.02721 | rs5758918 |  |  |  |  |  |  |  |  |  |  |  |  |  |  |  |
| -0.1586 | 6 | igd | 0.2996 | C | \|\| id:ebi-a-GCST90001560 | ebi-a-GCST90001560 | TRUE | T | 32666960 | 1.48E-08 | reported | 3395 | 0.02793 | rs114968045 |  |  |  |  |  |  |  |  |  |  |  |  |  |  |  |
| -0.1685 | 16 | igd | 0.2996 | A | \|\| id:ebi-a-GCST90002089 | ebi-a-GCST90002089 | TRUE | G | 31022639 | 3.97E-08 | reported | 2850 | 0.0306 | rs72800847 |  |  |  |  |  |  |  |  |  |  |  |  |  |  |  |
| -0.2988 | 19 | igd | 0.2995 | A | \|\| id:ebi-a-GCST90002074 | ebi-a-GCST90002074 | TRUE | G | 18285944 | 1.82E-25 | reported | 3112 | 0.0284 | rs11554159 |  |  |  |  |  |  |  |  |  |  |  |  |  |  |  |
| -0.1962 | 16 | igd | 0.2994 | A | \|\| id:ebi-a-GCST90002087 | ebi-a-GCST90002087 | TRUE | G | 31022639 | 4.58E-11 | reported | 2872 | 0.02969 | rs72800847 |  |  |  |  |  |  |  |  |  |  |  |  |  |  |  |
| -0.2081 | 16 | igd | 0.2994 | A | \|\| id:ebi-a-GCST90002088 | ebi-a-GCST90002088 | TRUE | G | 31022639 | 2.43E-12 | reported | 2872 | 0.02956 | rs72800847 |  |  |  |  |  |  |  |  |  |  |  |  |  |  |  |
| -0.2165 | 6 | igd | 0.2988 | G | \|\| id:ebi-a-GCST90001820 | ebi-a-GCST90001820 | TRUE | A | 31851234 | 1.58E-11 | reported | 3656 | 0.03202 | rs652888 |  |  |  |  |  |  |  |  |  |  |  |  |  |  |  |
| 0.2369 | 10 | igd | 0.2985 | G | \|\| id:ebi-a-GCST90001961 | ebi-a-GCST90001961 | TRUE | A | 97284596 | 9.66E-15 | reported | 2920 | 0.03044 | rs10882608 |  |  |  |  |  |  |  |  |  |  |  |  |  |  |  |
| -0.2936 | 10 | igd | 0.2985 | G | \|\| id:ebi-a-GCST90002030 | ebi-a-GCST90002030 | TRUE | A | 97284596 | 4.61E-22 | reported | 2920 | 0.03016 | rs10882608 |  |  |  |  |  |  |  |  |  |  |  |  |  |  |  |
| -0.2246 | 10 | igd | 0.2983 | C | \|\| id:ebi-a-GCST90002032 | ebi-a-GCST90002032 | TRUE | T | 97292507 | 4.69E-13 | reported | 2920 | 0.0309 | rs7922126 |  |  |  |  |  |  |  |  |  |  |  |  |  |  |  |
| -0.3745 | 19 | igd | 0.2982 | A | \|\| id:ebi-a-GCST90002073 | ebi-a-GCST90002073 | TRUE | G | 18285944 | 3.14E-36 | reported | 2850 | 0.02938 | rs11554159 |  |  |  |  |  |  |  |  |  |  |  |  |  |  |  |
| 0.201 | 2 | igd | 0.2975 | G | \|\| id:ebi-a-GCST90001485 | ebi-a-GCST90001485 | TRUE | A | 38897249 | 1.83E-13 | reported | 3437 | 0.02719 | rs12712610 |  |  |  |  |  |  |  |  |  |  |  |  |  |  |  |
| 0.1631 | 2 | igd | 0.2974 | G | \|\| id:ebi-a-GCST90001666 | ebi-a-GCST90001666 | TRUE | A | 38897249 | 2.82E-11 | reported | 3408 | 0.02442 | rs12712610 |  |  |  |  |  |  |  |  |  |  |  |  |  |  |  |
| -0.2847 | 2 | igd | 0.2974 | A | \|\| id:ebi-a-GCST90002119 | ebi-a-GCST90002119 | TRUE | C | 86652675 | 4.57E-23 | reported | 2920 | 0.02854 | rs11899750 |  |  |  |  |  |  |  |  |  |  |  |  |  |  |  |
| 0.1912 | 2 | igd | 0.2972 | G | \|\| id:ebi-a-GCST90001483 | ebi-a-GCST90001483 | TRUE | A | 38897249 | 4.16E-12 | reported | 3405 | 0.02749 | rs12712610 |  |  |  |  |  |  |  |  |  |  |  |  |  |  |  |
| 0.2347 | 2 | igd | 0.2972 | G | \|\| id:ebi-a-GCST90001507 | ebi-a-GCST90001507 | TRUE | A | 38897249 | 2.15E-16 | reported | 3405 | 0.02843 | rs12712610 |  |  |  |  |  |  |  |  |  |  |  |  |  |  |  |
| 0.1661 | 10 | igd | 0.2971 | A | \|\| id:ebi-a-GCST90001485 | ebi-a-GCST90001485 | TRUE | G | 98208544 | 7.29E-10 | reported | 3437 | 0.0269 | rs1316860 |  |  |  |  |  |  |  |  |  |  |  |  |  |  |  |
| 0.1735 | 10 | igd | 0.2971 | A | \|\| id:ebi-a-GCST90001491 | ebi-a-GCST90001491 | TRUE | G | 98208544 | 2.76E-10 | reported | 3437 | 0.02741 | rs1316860 |  |  |  |  |  |  |  |  |  |  |  |  |  |  |  |
| -0.1902 | 2 | igd | 0.2969 | G | \|\| id:ebi-a-GCST90001535 | ebi-a-GCST90001535 | TRUE | A | 38897249 | 3.13E-13 | reported | 3427 | 0.02599 | rs12712610 |  |  |  |  |  |  |  |  |  |  |  |  |  |  |  |
| -0.1632 | 2 | igd | 0.2969 | G | \|\| id:ebi-a-GCST90001536 | ebi-a-GCST90001536 | TRUE | A | 38897249 | 7.82E-10 | reported | 3427 | 0.02646 | rs12712610 |  |  |  |  |  |  |  |  |  |  |  |  |  |  |  |
| -0.1725 | 2 | igd | 0.2969 | C | \|\| id:ebi-a-GCST90002118 | ebi-a-GCST90002118 | TRUE | G | 86656646 | 4.89E-09 | reported | 2920 | 0.02939 | rs11127035 |  |  |  |  |  |  |  |  |  |  |  |  |  |  |  |
| 0.1739 | 6 | igd | 0.2967 | T | \|\| id:ebi-a-GCST90001973 | ebi-a-GCST90001973 | TRUE | C | 32583146 | 1.12E-08 | reported | 3113 | 0.03036 | rs4959105 |  |  |  |  |  |  |  |  |  |  |  |  |  |  |  |
| 0.2082 | 6 | igd | 0.2967 | T | \|\| id:ebi-a-GCST90002081 | ebi-a-GCST90002081 | TRUE | C | 32583146 | 3.61E-11 | reported | 3113 | 0.03133 | rs4959105 |  |  |  |  |  |  |  |  |  |  |  |  |  |  |  |
| 0.2212 | 1 | igd | 0.2965 | A | \|\| id:ebi-a-GCST90001999 | ebi-a-GCST90001999 | TRUE | G | 161565479 | 2.48E-15 | reported | 3621 | 0.02782 | rs111504845 |  |  |  |  |  |  |  |  |  |  |  |  |  |  |  |
| -0.2632 | 19 | igd | 0.2964 | A | \|\| id:ebi-a-GCST90001951 | ebi-a-GCST90001951 | TRUE | G | 53264226 | 2.53E-10 | reported | 1464 | 0.04133 | rs4310975 |  |  |  |  |  |  |  |  |  |  |  |  |  |  |  |
| -0.2439 | 6 | igd | 0.2963 | G | \|\| id:ebi-a-GCST90002105 | ebi-a-GCST90002105 | TRUE | A | 31717696 | 2.78E-11 | reported | 2872 | 0.03649 | rs1150793 |  |  |  |  |  |  |  |  |  |  |  |  |  |  |  |
| 0.2594 | 1 | igd | 0.2962 | A | \|\| id:ebi-a-GCST90001970 | ebi-a-GCST90001970 | TRUE | G | 161565479 | 9.29E-18 | reported | 2971 | 0.03003 | rs111504845 |  |  |  |  |  |  |  |  |  |  |  |  |  |  |  |
| 0.2289 | 1 | igd | 0.2962 | A | \|\| id:ebi-a-GCST90002077 | ebi-a-GCST90002077 | TRUE | G | 161565479 | 2.24E-14 | reported | 2971 | 0.02983 | rs111504845 |  |  |  |  |  |  |  |  |  |  |  |  |  |  |  |
| 0.1748 | 3 | igd | 0.2961 | A | \|\| id:ebi-a-GCST90002036 | ebi-a-GCST90002036 | TRUE | C | 52348364 | 9.68E-09 | reported | 2871 | 0.03039 | rs730050 |  |  |  |  |  |  |  |  |  |  |  |  |  |  |  |
| -0.3448 | 6 | igd | 0.2959 | A | \|\| id:ebi-a-GCST90002110 | ebi-a-GCST90002110 | TRUE | G | 32190484 | 3.57E-14 | reported | 1634 | 0.0451 | rs8192569 |  |  |  |  |  |  |  |  |  |  |  |  |  |  |  |
| -0.3039 | 6 | igd | 0.2957 | A | \|\| id:ebi-a-GCST90002111 | ebi-a-GCST90002111 | TRUE | G | 32190484 | 1.14E-11 | reported | 1635 | 0.04446 | rs8192569 |  |  |  |  |  |  |  |  |  |  |  |  |  |  |  |
| -0.39 | 6 | igd | 0.2957 | A | \|\| id:ebi-a-GCST90002112 | ebi-a-GCST90002112 | TRUE | G | 32190484 | 5.42E-18 | reported | 1635 | 0.0446 | rs8192569 |  |  |  |  |  |  |  |  |  |  |  |  |  |  |  |
| -0.1714 | 6 | igd | 0.2957 | G | \|\| id:ebi-a-GCST90002121 | ebi-a-GCST90002121 | TRUE | A | 29760677 | 4.18E-08 | reported | 2915 | 0.03118 | rs1610719 |  |  |  |  |  |  |  |  |  |  |  |  |  |  |  |
| -0.3288 | 19 | igd | 0.295 | A | \|\| id:ebi-a-GCST90001946 | ebi-a-GCST90001946 | TRUE | G | 53264226 | 2.94E-17 | reported | 1634 | 0.03848 | rs4310975 |  |  |  |  |  |  |  |  |  |  |  |  |  |  |  |
| -0.2935 | 19 | igd | 0.295 | A | \|\| id:ebi-a-GCST90001949 | ebi-a-GCST90001949 | TRUE | G | 53264226 | 3.17E-14 | reported | 1634 | 0.03832 | rs4310975 |  |  |  |  |  |  |  |  |  |  |  |  |  |  |  |
| 0.2432 | 3 | igd | 0.2947 | G | \|\| id:ebi-a-GCST90001800 | ebi-a-GCST90001800 | TRUE | C | 32604427 | 1.40E-18 | reported | 3656 | 0.0275 | rs62252804 |  |  |  |  |  |  |  |  |  |  |  |  |  |  |  |
| 0.2423 | 3 | igd | 0.2947 | G | \|\| id:ebi-a-GCST90001806 | ebi-a-GCST90001806 | TRUE | C | 32604427 | 1.25E-18 | reported | 3656 | 0.02735 | rs62252804 |  |  |  |  |  |  |  |  |  |  |  |  |  |  |  |
| 0.2043 | 3 | igd | 0.2946 | G | \|\| id:ebi-a-GCST90001798 | ebi-a-GCST90001798 | TRUE | C | 32604427 | 7.37E-14 | reported | 3657 | 0.0272 | rs62252804 |  |  |  |  |  |  |  |  |  |  |  |  |  |  |  |
| 0.1737 | 3 | igd | 0.2946 | G | \|\| id:ebi-a-GCST90001804 | ebi-a-GCST90001804 | TRUE | C | 32604427 | 1.79E-10 | reported | 3657 | 0.02715 | rs62252804 |  |  |  |  |  |  |  |  |  |  |  |  |  |  |  |
| 0.2105 | 3 | igd | 0.2946 | G | \|\| id:ebi-a-GCST90001805 | ebi-a-GCST90001805 | TRUE | C | 32604427 | 2.24E-14 | reported | 3657 | 0.02745 | rs62252804 |  |  |  |  |  |  |  |  |  |  |  |  |  |  |  |
| 0.165 | 3 | igd | 0.2946 | G | \|\| id:ebi-a-GCST90001808 | ebi-a-GCST90001808 | TRUE | C | 32604427 | 2.15E-09 | reported | 3657 | 0.02749 | rs62252804 |  |  |  |  |  |  |  |  |  |  |  |  |  |  |  |
| 0.1927 | 10 | igd | 0.2946 | T | \|\| id:ebi-a-GCST90001854 | ebi-a-GCST90001854 | TRUE | C | 97394628 | 1.69E-10 | reported | 2919 | 0.03005 | rs3793749 |  |  |  |  |  |  |  |  |  |  |  |  |  |  |  |
| -0.2728 | 10 | igd | 0.2945 | T | \|\| id:ebi-a-GCST90001888 | ebi-a-GCST90001888 | TRUE | C | 97394628 | 3.52E-20 | reported | 2920 | 0.02942 | rs3793749 |  |  |  |  |  |  |  |  |  |  |  |  |  |  |  |
| -0.3509 | 10 | igd | 0.2945 | T | \|\| id:ebi-a-GCST90001892 | ebi-a-GCST90001892 | TRUE | C | 97394628 | 1.89E-31 | reported | 2920 | 0.02972 | rs3793749 |  |  |  |  |  |  |  |  |  |  |  |  |  |  |  |
| -0.3077 | 19 | igd | 0.2945 | A | \|\| id:ebi-a-GCST90001948 | ebi-a-GCST90001948 | TRUE | G | 53264226 | 2.08E-15 | reported | 1633 | 0.03839 | rs4310975 |  |  |  |  |  |  |  |  |  |  |  |  |  |  |  |
| -0.2812 | 10 | igd | 0.2945 | T | \|\| id:ebi-a-GCST90002061 | ebi-a-GCST90002061 | TRUE | C | 97394628 | 4.97E-20 | reported | 2920 | 0.03047 | rs3793749 |  |  |  |  |  |  |  |  |  |  |  |  |  |  |  |
| -0.3162 | 6 | igd | 0.2942 | A | \|\| id:ebi-a-GCST90002108 | ebi-a-GCST90002108 | TRUE | C | 31555392 | 1.46E-12 | reported | 1579 | 0.04432 | rs2256974 |  |  |  |  |  |  |  |  |  |  |  |  |  |  |  |
| -0.2855 | 4 | igd | 0.2941 | A | \|\| id:ebi-a-GCST90002037 | ebi-a-GCST90002037 | TRUE | G | 84160279 | 1.58E-20 | reported | 2871 | 0.03051 | rs7658518 |  |  |  |  |  |  |  |  |  |  |  |  |  |  |  |
| -0.2821 | 4 | igd | 0.2941 | A | \|\| id:ebi-a-GCST90002038 | ebi-a-GCST90002038 | TRUE | G | 84160279 | 4.45E-20 | reported | 2871 | 0.03052 | rs7658518 |  |  |  |  |  |  |  |  |  |  |  |  |  |  |  |
| -0.3048 | 4 | igd | 0.294 | A | \|\| id:ebi-a-GCST90001944 | ebi-a-GCST90001944 | TRUE | G | 84160279 | 4.48E-23 | reported | 2872 | 0.03055 | rs7658518 |  |  |  |  |  |  |  |  |  |  |  |  |  |  |  |
| -0.3012 | 4 | igd | 0.294 | A | \|\| id:ebi-a-GCST90001945 | ebi-a-GCST90001945 | TRUE | G | 84160279 | 1.44E-22 | reported | 2872 | 0.03056 | rs7658518 |  |  |  |  |  |  |  |  |  |  |  |  |  |  |  |
| -0.3034 | 6 | igd | 0.294 | A | \|\| id:ebi-a-GCST90002109 | ebi-a-GCST90002109 | TRUE | G | 32190484 | 1.78E-11 | reported | 1578 | 0.04479 | rs8192569 |  |  |  |  |  |  |  |  |  |  |  |  |  |  |  |
| -0.3443 | 6 | igd | 0.2937 | C | \|\| id:ebi-a-GCST90002109 | ebi-a-GCST90002109 | TRUE | A | 32345429 | 3.73E-14 | reported | 1578 | 0.04506 | rs9268430 |  |  |  |  |  |  |  |  |  |  |  |  |  |  |  |
| -0.4847 | 6 | igd | 0.2935 | C | \|\| id:ebi-a-GCST90002108 | ebi-a-GCST90002108 | TRUE | A | 32345429 | 2.65E-26 | reported | 1579 | 0.04486 | rs9268430 |  |  |  |  |  |  |  |  |  |  |  |  |  |  |  |
| -0.3139 | 6 | igd | 0.2935 | C | \|\| id:ebi-a-GCST90002110 | ebi-a-GCST90002110 | TRUE | T | 31933518 | 5.51E-12 | reported | 1634 | 0.0452 | rs492899 |  |  |  |  |  |  |  |  |  |  |  |  |  |  |  |
| -0.3834 | 19 | igd | 0.2929 | C | \|\| id:ebi-a-GCST90001956 | ebi-a-GCST90001956 | TRUE | G | 51519346 | 1.39E-20 | reported | 1579 | 0.04066 | rs1061368 |  |  |  |  |  |  |  |  |  |  |  |  |  |  |  |
| -0.3824 | 19 | igd | 0.2928 | C | \|\| id:ebi-a-GCST90001957 | ebi-a-GCST90001957 | TRUE | G | 51519346 | 1.86E-20 | reported | 1578 | 0.04069 | rs1061368 |  |  |  |  |  |  |  |  |  |  |  |  |  |  |  |
| -0.2781 | 6 | igd | 0.2928 | T | \|\| id:ebi-a-GCST90002109 | ebi-a-GCST90002109 | TRUE | C | 32194308 | 6.70E-10 | reported | 1578 | 0.04477 | rs499606 |  |  |  |  |  |  |  |  |  |  |  |  |  |  |  |
| -0.3769 | 19 | igd | 0.2926 | C | \|\| id:ebi-a-GCST90001947 | ebi-a-GCST90001947 | TRUE | G | 51519346 | 7.55E-20 | reported | 1577 | 0.04077 | rs1061368 |  |  |  |  |  |  |  |  |  |  |  |  |  |  |  |
| -0.2729 | 6 | igd | 0.2923 | G | \|\| id:ebi-a-GCST90002108 | ebi-a-GCST90002108 | TRUE | C | 32177880 | 2.39E-09 | reported | 1579 | 0.04546 | rs206018 |  |  |  |  |  |  |  |  |  |  |  |  |  |  |  |
| -0.3379 | 19 | igd | 0.2922 | C | \|\| id:ebi-a-GCST90001952 | ebi-a-GCST90001952 | TRUE | G | 51519346 | 4.62E-16 | reported | 1569 | 0.04117 | rs1061368 |  |  |  |  |  |  |  |  |  |  |  |  |  |  |  |
| -0.3649 | 6 | igd | 0.292 | C | \|\| id:ebi-a-GCST90002108 | ebi-a-GCST90002108 | TRUE | T | 31933518 | 1.29E-15 | reported | 1579 | 0.04517 | rs492899 |  |  |  |  |  |  |  |  |  |  |  |  |  |  |  |
| -0.3743 | 6 | igd | 0.2919 | T | \|\| id:ebi-a-GCST90002110 | ebi-a-GCST90002110 | TRUE | C | 32194308 | 1.63E-16 | reported | 1634 | 0.04491 | rs499606 |  |  |  |  |  |  |  |  |  |  |  |  |  |  |  |
| 0.1763 | 22 | igd | 0.2918 | A | \|\| id:ebi-a-GCST90001702 | ebi-a-GCST90001702 | TRUE | G | 40256486 | 2.22E-10 | reported | 3657 | 0.02771 | rs9623081 |  |  |  |  |  |  |  |  |  |  |  |  |  |  |  |
| 0.183 | 22 | igd | 0.2918 | A | \|\| id:ebi-a-GCST90001703 | ebi-a-GCST90001703 | TRUE | G | 40256486 | 4.42E-11 | reported | 3657 | 0.02769 | rs9623081 |  |  |  |  |  |  |  |  |  |  |  |  |  |  |  |
| 0.1642 | 22 | igd | 0.2918 | A | \|\| id:ebi-a-GCST90001704 | ebi-a-GCST90001704 | TRUE | G | 40256486 | 3.29E-09 | reported | 3657 | 0.02769 | rs9623081 |  |  |  |  |  |  |  |  |  |  |  |  |  |  |  |
| 0.1732 | 22 | igd | 0.2918 | A | \|\| id:ebi-a-GCST90001710 | ebi-a-GCST90001710 | TRUE | G | 40256486 | 4.09E-10 | reported | 3657 | 0.02763 | rs9623081 |  |  |  |  |  |  |  |  |  |  |  |  |  |  |  |
| 0.1731 | 22 | igd | 0.2918 | A | \|\| id:ebi-a-GCST90001711 | ebi-a-GCST90001711 | TRUE | G | 40256486 | 3.81E-10 | reported | 3657 | 0.02757 | rs9623081 |  |  |  |  |  |  |  |  |  |  |  |  |  |  |  |
| 0.174 | 7 | igd | 0.2918 | A | \|\| id:ebi-a-GCST90002078 | ebi-a-GCST90002078 | TRUE | T | 92640344 | 1.71E-09 | reported | 3112 | 0.02881 | rs10244152 |  |  |  |  |  |  |  |  |  |  |  |  |  |  |  |
| -0.3504 | 6 | igd | 0.2917 | T | \|\| id:ebi-a-GCST90002111 | ebi-a-GCST90002111 | TRUE | C | 32194308 | 4.02E-15 | reported | 1635 | 0.04419 | rs499606 |  |  |  |  |  |  |  |  |  |  |  |  |  |  |  |
| -0.3994 | 6 | igd | 0.2909 | A | \|\| id:ebi-a-GCST90002105 | ebi-a-GCST90002105 | TRUE | G | 32190484 | 7.67E-28 | reported | 2872 | 0.03614 | rs8192569 |  |  |  |  |  |  |  |  |  |  |  |  |  |  |  |
| -0.1841 | 6 | igd | 0.2908 | G | \|\| id:ebi-a-GCST90001687 | ebi-a-GCST90001687 | TRUE | A | 32677413 | 3.87E-08 | reported | 3408 | 0.03342 | rs17615220 |  |  |  |  |  |  |  |  |  |  |  |  |  |  |  |
| 0.2035 | 6 | igd | 0.2908 | G | \|\| id:ebi-a-GCST90001692 | ebi-a-GCST90001692 | TRUE | A | 32677413 | 1.52E-09 | reported | 3440 | 0.03359 | rs17615220 |  |  |  |  |  |  |  |  |  |  |  |  |  |  |  |
| 0.1582 | 3 | igd | 0.2907 | T | \|\| id:ebi-a-GCST90001805 | ebi-a-GCST90001805 | TRUE | A | 32879823 | 7.95E-09 | reported | 3657 | 0.02735 | rs56131903 |  |  |  |  |  |  |  |  |  |  |  |  |  |  |  |
| -0.3353 | 19 | igd | 0.2905 | C | \|\| id:ebi-a-GCST90001955 | ebi-a-GCST90001955 | TRUE | G | 51519346 | 7.78E-16 | reported | 1554 | 0.04117 | rs1061368 |  |  |  |  |  |  |  |  |  |  |  |  |  |  |  |
| -0.2357 | 16 | igd | 0.2905 | T | \|\| id:ebi-a-GCST90002090 | ebi-a-GCST90002090 | TRUE | C | 31171174 | 3.58E-14 | reported | 2850 | 0.03096 | rs17708638 |  |  |  |  |  |  |  |  |  |  |  |  |  |  |  |
| -0.3453 | 19 | igd | 0.2904 | C | \|\| id:ebi-a-GCST90001953 | ebi-a-GCST90001953 | TRUE | G | 51519346 | 8.99E-17 | reported | 1558 | 0.04105 | rs1061368 |  |  |  |  |  |  |  |  |  |  |  |  |  |  |  |
| -0.3378 | 6 | igd | 0.2901 | T | \|\| id:ebi-a-GCST90002110 | ebi-a-GCST90002110 | TRUE | A | 31824912 | 2.09E-13 | reported | 1634 | 0.04561 | rs9267650 |  |  |  |  |  |  |  |  |  |  |  |  |  |  |  |
| -0.3482 | 19 | igd | 0.29 | C | \|\| id:ebi-a-GCST90001954 | ebi-a-GCST90001954 | TRUE | G | 51519346 | 5.15E-17 | reported | 1555 | 0.04106 | rs1061368 |  |  |  |  |  |  |  |  |  |  |  |  |  |  |  |
| -0.4036 | 6 | igd | 0.29 | C | \|\| id:ebi-a-GCST90002105 | ebi-a-GCST90002105 | TRUE | T | 31933518 | 3.59E-28 | reported | 2872 | 0.03628 | rs492899 |  |  |  |  |  |  |  |  |  |  |  |  |  |  |  |
| 0.2524 | 1 | igd | 0.2899 | A | \|\| id:ebi-a-GCST90001474 | ebi-a-GCST90001474 | TRUE | G | 161565479 | 5.34E-19 | reported | 3410 | 0.02818 | rs111504845 |  |  |  |  |  |  |  |  |  |  |  |  |  |  |  |
| -0.3226 | 6 | igd | 0.2899 | T | \|\| id:ebi-a-GCST90002111 | ebi-a-GCST90002111 | TRUE | A | 31824912 | 9.43E-13 | reported | 1635 | 0.04483 | rs9267650 |  |  |  |  |  |  |  |  |  |  |  |  |  |  |  |
| -0.3988 | 6 | igd | 0.2899 | T | \|\| id:ebi-a-GCST90002112 | ebi-a-GCST90002112 | TRUE | A | 31824912 | 2.05E-18 | reported | 1635 | 0.04502 | rs9267650 |  |  |  |  |  |  |  |  |  |  |  |  |  |  |  |
| 0.1693 | 3 | igd | 0.2893 | G | \|\| id:ebi-a-GCST90001798 | ebi-a-GCST90001798 | TRUE | C | 32634034 | 5.69E-10 | reported | 3657 | 0.02724 | rs35719720 |  |  |  |  |  |  |  |  |  |  |  |  |  |  |  |
| 0.1582 | 3 | igd | 0.2893 | G | \|\| id:ebi-a-GCST90001804 | ebi-a-GCST90001804 | TRUE | C | 32634034 | 6.09E-09 | reported | 3657 | 0.02714 | rs35719720 |  |  |  |  |  |  |  |  |  |  |  |  |  |  |  |
| 0.1579 | 3 | igd | 0.2893 | G | \|\| id:ebi-a-GCST90001808 | ebi-a-GCST90001808 | TRUE | C | 32634034 | 9.97E-09 | reported | 3657 | 0.02748 | rs35719720 |  |  |  |  |  |  |  |  |  |  |  |  |  |  |  |
| -0.3052 | 6 | igd | 0.2893 | T | \|\| id:ebi-a-GCST90002109 | ebi-a-GCST90002109 | TRUE | A | 31824912 | 2.32E-11 | reported | 1578 | 0.04533 | rs9267650 |  |  |  |  |  |  |  |  |  |  |  |  |  |  |  |
| -0.3081 | 6 | igd | 0.2892 | A | \|\| id:ebi-a-GCST90002110 | ebi-a-GCST90002110 | TRUE | G | 31406562 | 1.29E-10 | reported | 1634 | 0.04762 | rs146791096 |  |  |  |  |  |  |  |  |  |  |  |  |  |  |  |
| -0.3979 | 6 | igd | 0.2891 | T | \|\| id:ebi-a-GCST90002108 | ebi-a-GCST90002108 | TRUE | A | 31824912 | 5.33E-18 | reported | 1579 | 0.04547 | rs9267650 |  |  |  |  |  |  |  |  |  |  |  |  |  |  |  |
| -0.2848 | 6 | igd | 0.289 | A | \|\| id:ebi-a-GCST90002109 | ebi-a-GCST90002109 | TRUE | G | 31406562 | 1.58E-09 | reported | 1578 | 0.04691 | rs146791096 |  |  |  |  |  |  |  |  |  |  |  |  |  |  |  |
| -0.3572 | 6 | igd | 0.2888 | A | \|\| id:ebi-a-GCST90002108 | ebi-a-GCST90002108 | TRUE | G | 31406562 | 6.53E-14 | reported | 1579 | 0.04722 | rs146791096 |  |  |  |  |  |  |  |  |  |  |  |  |  |  |  |
| 0.2766 | 3 | igd | 0.2887 | A | \|\| id:ebi-a-GCST90001801 | ebi-a-GCST90001801 | TRUE | G | 32568632 | 2.46E-13 | reported | 1836 | 0.0375 | rs62252794 |  |  |  |  |  |  |  |  |  |  |  |  |  |  |  |
| -0.2127 | 1 | igd | 0.2885 | T | \|\| id:ebi-a-GCST90001989 | ebi-a-GCST90001989 | TRUE | G | 161185058 | 1.92E-14 | reported | 3621 | 0.02768 | rs2070901 |  |  |  |  |  |  |  |  |  |  |  |  |  |  |  |
| -0.5723 | 10 | igd | 0.2883 | C | \|\| id:ebi-a-GCST90001489 | ebi-a-GCST90001489 | TRUE | T | 97376731 | 6.24E-91 | reported | 3405 | 0.02745 | rs1268608 |  |  |  |  |  |  |  |  |  |  |  |  |  |  |  |
| 0.2108 | 2 | igd | 0.2883 | C | \|\| id:ebi-a-GCST90002098 | ebi-a-GCST90002098 | TRUE | G | 41791255 | 2.75E-13 | reported | 2910 | 0.02872 | rs2043011 |  |  |  |  |  |  |  |  |  |  |  |  |  |  |  |
| -0.3903 | 6 | igd | 0.2883 | T | \|\| id:ebi-a-GCST90002105 | ebi-a-GCST90002105 | TRUE | A | 31824912 | 7.31E-26 | reported | 2872 | 0.03676 | rs9267650 |  |  |  |  |  |  |  |  |  |  |  |  |  |  |  |
| -0.2753 | 19 | igd | 0.2878 | A | \|\| id:ebi-a-GCST90001956 | ebi-a-GCST90001956 | TRUE | G | 53264226 | 1.21E-11 | reported | 1579 | 0.04031 | rs4310975 |  |  |  |  |  |  |  |  |  |  |  |  |  |  |  |
| -0.4434 | 6 | igd | 0.2878 | A | \|\| id:ebi-a-GCST90002108 | ebi-a-GCST90002108 | TRUE | G | 32230665 | 4.87E-22 | reported | 1579 | 0.04526 | rs1150762 |  |  |  |  |  |  |  |  |  |  |  |  |  |  |  |
| -0.1928 | 6 | igd | 0.2877 | G | \|\| id:ebi-a-GCST90001932 | ebi-a-GCST90001932 | TRUE | A | 32677413 | 1.02E-08 | reported | 2920 | 0.03357 | rs17615220 |  |  |  |  |  |  |  |  |  |  |  |  |  |  |  |
| -0.1885 | 6 | igd | 0.2877 | C | \|\| id:ebi-a-GCST90001932 | ebi-a-GCST90001932 | TRUE | T | 32583919 | 2.08E-08 | reported | 2920 | 0.03354 | rs33964890 |  |  |  |  |  |  |  |  |  |  |  |  |  |  |  |
| -0.2765 | 19 | igd | 0.2877 | A | \|\| id:ebi-a-GCST90001957 | ebi-a-GCST90001957 | TRUE | G | 53264226 | 9.73E-12 | reported | 1578 | 0.04029 | rs4310975 |  |  |  |  |  |  |  |  |  |  |  |  |  |  |  |
| 0.1769 | 10 | igd | 0.2876 | T | \|\| id:ebi-a-GCST90001660 | ebi-a-GCST90001660 | TRUE | G | 96466078 | 1.75E-09 | reported | 3408 | 0.0293 | rs1409655 |  |  |  |  |  |  |  |  |  |  |  |  |  |  |  |
| -0.1566 | 2 | igd | 0.2875 | A | \|\| id:ebi-a-GCST90001483 | ebi-a-GCST90001483 | TRUE | G | 38745822 | 2.88E-08 | reported | 3405 | 0.02815 | rs11890666 |  |  |  |  |  |  |  |  |  |  |  |  |  |  |  |
| 0.1759 | 10 | igd | 0.2874 | G | \|\| id:ebi-a-GCST90001660 | ebi-a-GCST90001660 | TRUE | A | 96592144 | 2.15E-09 | reported | 3408 | 0.0293 | rs11188090 |  |  |  |  |  |  |  |  |  |  |  |  |  |  |  |
| 0.1767 | 10 | igd | 0.2874 | A | \|\| id:ebi-a-GCST90001672 | ebi-a-GCST90001672 | TRUE | G | 96563757 | 2.61E-09 | reported | 3408 | 0.0296 | rs4494250 |  |  |  |  |  |  |  |  |  |  |  |  |  |  |  |
| -0.2846 | 19 | igd | 0.2874 | A | \|\| id:ebi-a-GCST90001952 | ebi-a-GCST90001952 | TRUE | G | 53264226 | 2.77E-12 | reported | 1569 | 0.0404 | rs4310975 |  |  |  |  |  |  |  |  |  |  |  |  |  |  |  |
| 0.1724 | 10 | igd | 0.2872 | A | \|\| id:ebi-a-GCST90001495 | ebi-a-GCST90001495 | TRUE | C | 96407796 | 6.48E-09 | reported | 3405 | 0.02962 | rs7075182 |  |  |  |  |  |  |  |  |  |  |  |  |  |  |  |
| 0.1774 | 10 | igd | 0.2872 | T | \|\| id:ebi-a-GCST90001496 | ebi-a-GCST90001496 | TRUE | G | 96466078 | 1.54E-09 | reported | 3437 | 0.02928 | rs1409655 |  |  |  |  |  |  |  |  |  |  |  |  |  |  |  |
| 0.1892 | 10 | igd | 0.2872 | T | \|\| id:ebi-a-GCST90001497 | ebi-a-GCST90001497 | TRUE | G | 96466078 | 6.70E-11 | reported | 3437 | 0.02889 | rs1409655 |  |  |  |  |  |  |  |  |  |  |  |  |  |  |  |
| 0.1542 | 10 | igd | 0.2871 | T | \|\| id:ebi-a-GCST90001658 | ebi-a-GCST90001658 | TRUE | G | 96466078 | 3.95E-08 | reported | 3440 | 0.02801 | rs1409655 |  |  |  |  |  |  |  |  |  |  |  |  |  |  |  |
| 0.1757 | 10 | igd | 0.2871 | T | \|\| id:ebi-a-GCST90001659 | ebi-a-GCST90001659 | TRUE | G | 96466078 | 3.79E-10 | reported | 3440 | 0.02798 | rs1409655 |  |  |  |  |  |  |  |  |  |  |  |  |  |  |  |
| -0.1876 | 6 | igd | 0.2871 | G | \|\| id:ebi-a-GCST90001820 | ebi-a-GCST90001820 | TRUE | C | 32177880 | 1.02E-08 | reported | 3656 | 0.03268 | rs206018 |  |  |  |  |  |  |  |  |  |  |  |  |  |  |  |
| 0.1531 | 10 | igd | 0.2869 | G | \|\| id:ebi-a-GCST90001658 | ebi-a-GCST90001658 | TRUE | A | 96592144 | 4.92E-08 | reported | 3440 | 0.02801 | rs11188090 |  |  |  |  |  |  |  |  |  |  |  |  |  |  |  |
| -0.2585 | 19 | igd | 0.2869 | A | \|\| id:ebi-a-GCST90001947 | ebi-a-GCST90001947 | TRUE | G | 53264226 | 2.55E-10 | reported | 1577 | 0.0406 | rs4310975 |  |  |  |  |  |  |  |  |  |  |  |  |  |  |  |
| -0.1612 | 2 | igd | 0.2866 | A | \|\| id:ebi-a-GCST90001485 | ebi-a-GCST90001485 | TRUE | G | 38745822 | 7.94E-09 | reported | 3437 | 0.02787 | rs11890666 |  |  |  |  |  |  |  |  |  |  |  |  |  |  |  |
| 0.2181 | 10 | igd | 0.2865 | A | \|\| id:ebi-a-GCST90002030 | ebi-a-GCST90002030 | TRUE | C | 96407796 | 8.96E-13 | reported | 2920 | 0.03039 | rs7075182 |  |  |  |  |  |  |  |  |  |  |  |  |  |  |  |
| 0.1736 | 10 | igd | 0.2865 | A | \|\| id:ebi-a-GCST90002031 | ebi-a-GCST90002031 | TRUE | C | 96407796 | 2.55E-08 | reported | 2920 | 0.03109 | rs7075182 |  |  |  |  |  |  |  |  |  |  |  |  |  |  |  |
| 0.1912 | 10 | igd | 0.2865 | A | \|\| id:ebi-a-GCST90002032 | ebi-a-GCST90002032 | TRUE | C | 96407796 | 8.38E-10 | reported | 2920 | 0.03105 | rs7075182 |  |  |  |  |  |  |  |  |  |  |  |  |  |  |  |
| -0.1694 | 10 | igd | 0.286 | A | \|\| id:ebi-a-GCST90001892 | ebi-a-GCST90001892 | TRUE | G | 97069472 | 3.15E-08 | reported | 2920 | 0.03054 | rs10786199 |  |  |  |  |  |  |  |  |  |  |  |  |  |  |  |
| 0.1813 | 1 | igd | 0.2859 | A | \|\| id:ebi-a-GCST90001994 | ebi-a-GCST90001994 | TRUE | G | 161704111 | 2.46E-11 | reported | 3590 | 0.02707 | rs11810305 |  |  |  |  |  |  |  |  |  |  |  |  |  |  |  |
| -0.2784 | 19 | igd | 0.2856 | A | \|\| id:ebi-a-GCST90001953 | ebi-a-GCST90001953 | TRUE | G | 53264226 | 6.78E-12 | reported | 1558 | 0.04026 | rs4310975 |  |  |  |  |  |  |  |  |  |  |  |  |  |  |  |
| -0.2849 | 19 | igd | 0.2855 | A | \|\| id:ebi-a-GCST90001954 | ebi-a-GCST90001954 | TRUE | G | 53264226 | 2.38E-12 | reported | 1555 | 0.04032 | rs4310975 |  |  |  |  |  |  |  |  |  |  |  |  |  |  |  |
| 0.1804 | 10 | igd | 0.2848 | T | \|\| id:ebi-a-GCST90001496 | ebi-a-GCST90001496 | TRUE | C | 96600732 | 8.08E-10 | reported | 3437 | 0.02927 | rs12416196 |  |  |  |  |  |  |  |  |  |  |  |  |  |  |  |
| 0.1903 | 10 | igd | 0.2848 | T | \|\| id:ebi-a-GCST90001497 | ebi-a-GCST90001497 | TRUE | C | 96600732 | 5.17E-11 | reported | 3437 | 0.02888 | rs12416196 |  |  |  |  |  |  |  |  |  |  |  |  |  |  |  |
| 0.1749 | 10 | igd | 0.2847 | T | \|\| id:ebi-a-GCST90001659 | ebi-a-GCST90001659 | TRUE | C | 96600732 | 4.49E-10 | reported | 3440 | 0.02797 | rs12416196 |  |  |  |  |  |  |  |  |  |  |  |  |  |  |  |
| -0.248 | 6 | igd | 0.2845 | G | \|\| id:ebi-a-GCST90002105 | ebi-a-GCST90002105 | TRUE | C | 32177880 | 2.74E-11 | reported | 2872 | 0.0371 | rs206018 |  |  |  |  |  |  |  |  |  |  |  |  |  |  |  |
| 0.1621 | 2 | igd | 0.2837 | A | \|\| id:ebi-a-GCST90002098 | ebi-a-GCST90002098 | TRUE | G | 38051089 | 3.31E-08 | reported | 2910 | 0.02926 | rs13027679 |  |  |  |  |  |  |  |  |  |  |  |  |  |  |  |
| 0.173 | 1 | igd | 0.2834 | T | \|\| id:ebi-a-GCST90001913 | ebi-a-GCST90001913 | TRUE | G | 161185058 | 6.37E-09 | reported | 3112 | 0.02971 | rs2070901 |  |  |  |  |  |  |  |  |  |  |  |  |  |  |  |
| -0.1573 | 22 | igd | 0.2832 | A | \|\| id:ebi-a-GCST90001702 | ebi-a-GCST90001702 | TRUE | G | 44293352 | 2.22E-08 | reported | 3657 | 0.02805 | rs28495372 |  |  |  |  |  |  |  |  |  |  |  |  |  |  |  |
| -0.1537 | 22 | igd | 0.2832 | A | \|\| id:ebi-a-GCST90001703 | ebi-a-GCST90001703 | TRUE | G | 44293352 | 4.56E-08 | reported | 3657 | 0.02805 | rs28495372 |  |  |  |  |  |  |  |  |  |  |  |  |  |  |  |
| -0.1604 | 2 | igd | 0.2827 | G | \|\| id:ebi-a-GCST90001661 | ebi-a-GCST90001661 | TRUE | A | 111808558 | 1.54E-08 | reported | 3440 | 0.02829 | rs12986962 |  |  |  |  |  |  |  |  |  |  |  |  |  |  |  |
| -0.1568 | 2 | igd | 0.2826 | G | \|\| id:ebi-a-GCST90001663 | ebi-a-GCST90001663 | TRUE | A | 111808558 | 3.59E-08 | reported | 3408 | 0.0284 | rs12986962 |  |  |  |  |  |  |  |  |  |  |  |  |  |  |  |
| -0.4994 | 10 | igd | 0.2823 | G | \|\| id:ebi-a-GCST90001670 | ebi-a-GCST90001670 | TRUE | C | 97361716 | 4.36E-71 | reported | 3440 | 0.02736 | rs543672 |  |  |  |  |  |  |  |  |  |  |  |  |  |  |  |
| -0.487 | 10 | igd | 0.2823 | G | \|\| id:ebi-a-GCST90001671 | ebi-a-GCST90001671 | TRUE | C | 97361716 | 3.70E-67 | reported | 3440 | 0.02751 | rs543672 |  |  |  |  |  |  |  |  |  |  |  |  |  |  |  |
| -0.5011 | 10 | igd | 0.2823 | G | \|\| id:ebi-a-GCST90001672 | ebi-a-GCST90001672 | TRUE | C | 97361716 | 4.04E-69 | reported | 3408 | 0.02787 | rs543672 |  |  |  |  |  |  |  |  |  |  |  |  |  |  |  |
| -0.225 | 2 | igd | 0.2823 | C | \|\| id:ebi-a-GCST90002098 | ebi-a-GCST90002098 | TRUE | A | 38510608 | 8.94E-15 | reported | 2910 | 0.02887 | rs6743640 |  |  |  |  |  |  |  |  |  |  |  |  |  |  |  |
| -0.1706 | 2 | igd | 0.2823 | C | \|\| id:ebi-a-GCST90002100 | ebi-a-GCST90002100 | TRUE | A | 38510608 | 1.06E-08 | reported | 2910 | 0.02972 | rs6743640 |  |  |  |  |  |  |  |  |  |  |  |  |  |  |  |
| -0.1888 | 2 | igd | 0.2816 | C | \|\| id:ebi-a-GCST90002102 | ebi-a-GCST90002102 | TRUE | A | 38510608 | 5.57E-11 | reported | 2919 | 0.0287 | rs6743640 |  |  |  |  |  |  |  |  |  |  |  |  |  |  |  |
| 0.3138 | 6 | igd | 0.281 | A | \|\| id:ebi-a-GCST90002105 | ebi-a-GCST90002105 | TRUE | G | 31102241 | 1.31E-20 | reported | 2872 | 0.03346 | rs9468874 |  |  |  |  |  |  |  |  |  |  |  |  |  |  |  |
| 0.4175 | 10 | igd | 0.2807 | G | \|\| id:ebi-a-GCST90001961 | ebi-a-GCST90001961 | TRUE | C | 97361716 | 4.94E-43 | reported | 2920 | 0.02987 | rs543672 |  |  |  |  |  |  |  |  |  |  |  |  |  |  |  |
| 0.1822 | 10 | igd | 0.2805 | T | \|\| id:ebi-a-GCST90001495 | ebi-a-GCST90001495 | TRUE | C | 95966698 | 1.02E-09 | reported | 3405 | 0.02975 | rs11187814 |  |  |  |  |  |  |  |  |  |  |  |  |  |  |  |
| 0.1723 | 2 | igd | 0.2801 | C | \|\| id:ebi-a-GCST90001893 | ebi-a-GCST90001893 | TRUE | T | 204421284 | 4.39E-08 | reported | 2920 | 0.03139 | rs1174604 |  |  |  |  |  |  |  |  |  |  |  |  |  |  |  |
| 0.188 | 22 | igd | 0.2799 | C | \|\| id:ebi-a-GCST90001710 | ebi-a-GCST90001710 | TRUE | T | 43599917 | 2.00E-11 | reported | 3657 | 0.02795 | rs138983 |  |  |  |  |  |  |  |  |  |  |  |  |  |  |  |
| 0.1661 | 10 | igd | 0.2786 | A | \|\| id:ebi-a-GCST90001484 | ebi-a-GCST90001484 | TRUE | G | 95966271 | 5.61E-09 | reported | 3437 | 0.02842 | rs2860746 |  |  |  |  |  |  |  |  |  |  |  |  |  |  |  |
| 0.1744 | 10 | igd | 0.2786 | A | \|\| id:ebi-a-GCST90001485 | ebi-a-GCST90001485 | TRUE | G | 95966271 | 8.77E-10 | reported | 3437 | 0.02837 | rs2860746 |  |  |  |  |  |  |  |  |  |  |  |  |  |  |  |
| 0.1812 | 10 | igd | 0.2784 | A | \|\| id:ebi-a-GCST90001483 | ebi-a-GCST90001483 | TRUE | G | 95966271 | 2.98E-10 | reported | 3405 | 0.02867 | rs2860746 |  |  |  |  |  |  |  |  |  |  |  |  |  |  |  |
| -0.1905 | 6 | igd | 0.2783 | T | \|\| id:ebi-a-GCST90002119 | ebi-a-GCST90002119 | TRUE | C | 29936634 | 2.16E-08 | reported | 2920 | 0.03392 | rs2523973 |  |  |  |  |  |  |  |  |  |  |  |  |  |  |  |
| -0.1759 | 10 | igd | 0.2781 | G | \|\| id:ebi-a-GCST90001892 | ebi-a-GCST90001892 | TRUE | T | 97862580 | 7.69E-09 | reported | 2920 | 0.03038 | rs11188604 |  |  |  |  |  |  |  |  |  |  |  |  |  |  |  |
| 0.2009 | 2 | igd | 0.2774 | T | \|\| id:ebi-a-GCST90001899 | ebi-a-GCST90001899 | TRUE | C | 204570092 | 3.39E-12 | reported | 2920 | 0.02874 | rs3181096 |  |  |  |  |  |  |  |  |  |  |  |  |  |  |  |
| -0.2809 | 19 | igd | 0.2774 | C | \|\| id:ebi-a-GCST90001948 | ebi-a-GCST90001948 | TRUE | T | 51340188 | 3.57E-12 | reported | 1633 | 0.0401 | rs2569745 |  |  |  |  |  |  |  |  |  |  |  |  |  |  |  |
| 0.2034 | 10 | igd | 0.2773 | C | \|\| id:ebi-a-GCST90001787 | ebi-a-GCST90001787 | TRUE | T | 6074634 | 2.10E-13 | reported | 3657 | 0.02759 | rs7920946 |  |  |  |  |  |  |  |  |  |  |  |  |  |  |  |
| 0.2483 | 10 | igd | 0.2773 | C | \|\| id:ebi-a-GCST90001790 | ebi-a-GCST90001790 | TRUE | T | 6074634 | 1.66E-19 | reported | 3657 | 0.02733 | rs7920946 |  |  |  |  |  |  |  |  |  |  |  |  |  |  |  |
| -0.2735 | 19 | igd | 0.2772 | C | \|\| id:ebi-a-GCST90001946 | ebi-a-GCST90001946 | TRUE | T | 51340188 | 1.49E-11 | reported | 1634 | 0.04024 | rs2569745 |  |  |  |  |  |  |  |  |  |  |  |  |  |  |  |
| -0.2828 | 19 | igd | 0.2772 | C | \|\| id:ebi-a-GCST90001949 | ebi-a-GCST90001949 | TRUE | T | 51340188 | 2.22E-12 | reported | 1634 | 0.03997 | rs2569745 |  |  |  |  |  |  |  |  |  |  |  |  |  |  |  |
| 0.1681 | 10 | igd | 0.2769 | C | \|\| id:ebi-a-GCST90001961 | ebi-a-GCST90001961 | TRUE | T | 97856899 | 4.72E-08 | reported | 2920 | 0.0307 | rs11188601 |  |  |  |  |  |  |  |  |  |  |  |  |  |  |  |
| 0.2392 | 6 | igd | 0.2769 | G | \|\| id:ebi-a-GCST90002110 | ebi-a-GCST90002110 | TRUE | A | 32965062 | 9.70E-09 | reported | 1634 | 0.04149 | rs3128947 |  |  |  |  |  |  |  |  |  |  |  |  |  |  |  |
| 0.1789 | 6 | igd | 0.2767 | A | \|\| id:ebi-a-GCST90001675 | ebi-a-GCST90001675 | TRUE | G | 32446459 | 4.52E-08 | reported | 3130 | 0.03263 | rs17203563 |  |  |  |  |  |  |  |  |  |  |  |  |  |  |  |
| -0.2107 | 6 | igd | 0.2766 | T | \|\| id:ebi-a-GCST90002105 | ebi-a-GCST90002105 | TRUE | C | 33000150 | 2.30E-10 | reported | 2872 | 0.03311 | rs493158 |  |  |  |  |  |  |  |  |  |  |  |  |  |  |  |
| 0.3139 | 2 | igd | 0.2763 | C | \|\| id:ebi-a-GCST90001900 | ebi-a-GCST90001900 | TRUE | T | 204571909 | 7.60E-25 | reported | 2919 | 0.03022 | rs34453488 |  |  |  |  |  |  |  |  |  |  |  |  |  |  |  |
| -0.1548 | 4 | igd | 0.2762 | A | \|\| id:ebi-a-GCST90001692 | ebi-a-GCST90001692 | TRUE | C | 78710538 | 4.50E-08 | reported | 3440 | 0.02824 | rs16996915 |  |  |  |  |  |  |  |  |  |  |  |  |  |  |  |
| 0.2658 | 2 | igd | 0.2762 | T | \|\| id:ebi-a-GCST90001890 | ebi-a-GCST90001890 | TRUE | A | 204572057 | 8.41E-18 | reported | 2920 | 0.03074 | rs3181102 |  |  |  |  |  |  |  |  |  |  |  |  |  |  |  |
| 0.2473 | 6 | igd | 0.2761 | A | \|\| id:ebi-a-GCST90002108 | ebi-a-GCST90002108 | TRUE | G | 31102241 | 1.32E-08 | reported | 1579 | 0.04328 | rs9468874 |  |  |  |  |  |  |  |  |  |  |  |  |  |  |  |
| -0.3543 | 6 | igd | 0.2761 | A | \|\| id:ebi-a-GCST90002112 | ebi-a-GCST90002112 | TRUE | G | 31880480 | 2.64E-14 | reported | 1635 | 0.0461 | rs9267671 |  |  |  |  |  |  |  |  |  |  |  |  |  |  |  |
| -0.2698 | 6 | igd | 0.276 | T | \|\| id:ebi-a-GCST90002109 | ebi-a-GCST90002109 | TRUE | C | 32682135 | 8.20E-09 | reported | 1578 | 0.04656 | rs3957147 |  |  |  |  |  |  |  |  |  |  |  |  |  |  |  |
| 0.2305 | 2 | igd | 0.2757 | A | \|\| id:ebi-a-GCST90001887 | ebi-a-GCST90001887 | TRUE | G | 204570826 | 3.37E-14 | reported | 2920 | 0.03024 | rs35593994 |  |  |  |  |  |  |  |  |  |  |  |  |  |  |  |
| 0.2421 | 2 | igd | 0.2757 | A | \|\| id:ebi-a-GCST90001889 | ebi-a-GCST90001889 | TRUE | G | 204570826 | 3.48E-16 | reported | 2920 | 0.02952 | rs35593994 |  |  |  |  |  |  |  |  |  |  |  |  |  |  |  |
| 0.2846 | 2 | igd | 0.2757 | A | \|\| id:ebi-a-GCST90001891 | ebi-a-GCST90001891 | TRUE | G | 204570826 | 9.79E-21 | reported | 2920 | 0.03025 | rs35593994 |  |  |  |  |  |  |  |  |  |  |  |  |  |  |  |
| 0.2642 | 2 | igd | 0.2757 | A | \|\| id:ebi-a-GCST90001893 | ebi-a-GCST90001893 | TRUE | G | 204570826 | 8.23E-18 | reported | 2920 | 0.03054 | rs35593994 |  |  |  |  |  |  |  |  |  |  |  |  |  |  |  |
| 0.2323 | 2 | igd | 0.2757 | A | \|\| id:ebi-a-GCST90001902 | ebi-a-GCST90001902 | TRUE | G | 204570826 | 1.92E-15 | reported | 2920 | 0.02908 | rs35593994 |  |  |  |  |  |  |  |  |  |  |  |  |  |  |  |
| 0.2531 | 6 | igd | 0.2757 | A | \|\| id:ebi-a-GCST90002118 | ebi-a-GCST90002118 | TRUE | G | 29930939 | 6.75E-13 | reported | 2920 | 0.03508 | rs56262516 |  |  |  |  |  |  |  |  |  |  |  |  |  |  |  |
| 0.23 | 6 | igd | 0.2757 | A | \|\| id:ebi-a-GCST90002120 | ebi-a-GCST90002120 | TRUE | G | 29930939 | 1.24E-11 | reported | 2920 | 0.03381 | rs56262516 |  |  |  |  |  |  |  |  |  |  |  |  |  |  |  |
| 0.1977 | 6 | igd | 0.2756 | A | \|\| id:ebi-a-GCST90002121 | ebi-a-GCST90002121 | TRUE | G | 29930939 | 1.59E-09 | reported | 2915 | 0.03266 | rs56262516 |  |  |  |  |  |  |  |  |  |  |  |  |  |  |  |
| -0.345 | 6 | igd | 0.2755 | T | \|\| id:ebi-a-GCST90002108 | ebi-a-GCST90002108 | TRUE | C | 31920873 | 1.84E-13 | reported | 1579 | 0.04646 | rs4151664 |  |  |  |  |  |  |  |  |  |  |  |  |  |  |  |
| -0.3141 | 6 | igd | 0.2755 | T | \|\| id:ebi-a-GCST90002111 | ebi-a-GCST90002111 | TRUE | C | 32682135 | 1.31E-11 | reported | 1635 | 0.04608 | rs3957147 |  |  |  |  |  |  |  |  |  |  |  |  |  |  |  |
| 0.3394 | 6 | igd | 0.2749 | A | \|\| id:ebi-a-GCST90002112 | ebi-a-GCST90002112 | TRUE | G | 31102241 | 3.33E-15 | reported | 1635 | 0.04267 | rs9468874 |  |  |  |  |  |  |  |  |  |  |  |  |  |  |  |
| -0.1636 | 2 | igd | 0.2743 | A | \|\| id:ebi-a-GCST90001886 | ebi-a-GCST90001886 | TRUE | T | 204328474 | 3.98E-08 | reported | 2920 | 0.02972 | rs77594357 |  |  |  |  |  |  |  |  |  |  |  |  |  |  |  |
| -0.1723 | 2 | igd | 0.2742 | A | \|\| id:ebi-a-GCST90001900 | ebi-a-GCST90001900 | TRUE | T | 204328474 | 3.18E-08 | reported | 2919 | 0.03107 | rs77594357 |  |  |  |  |  |  |  |  |  |  |  |  |  |  |  |
| 0.339 | 1 | igd | 0.2737 | G | \|\| id:ebi-a-GCST90001989 | ebi-a-GCST90001989 | TRUE | T | 161515638 | 7.56E-33 | reported | 3621 | 0.02812 | rs6687275 |  |  |  |  |  |  |  |  |  |  |  |  |  |  |  |
| -0.2887 | 10 | igd | 0.2736 | G | \|\| id:ebi-a-GCST90002030 | ebi-a-GCST90002030 | TRUE | A | 95988042 | 2.85E-20 | reported | 2920 | 0.03106 | rs11187826 |  |  |  |  |  |  |  |  |  |  |  |  |  |  |  |
| -0.2396 | 10 | igd | 0.2736 | G | \|\| id:ebi-a-GCST90002031 | ebi-a-GCST90002031 | TRUE | A | 95988042 | 6.99E-14 | reported | 2920 | 0.03184 | rs11187826 |  |  |  |  |  |  |  |  |  |  |  |  |  |  |  |
| 0.1903 | 14 | igd | 0.2732 | C | \|\| id:ebi-a-GCST90001821 | ebi-a-GCST90001821 | TRUE | T | 105870108 | 7.53E-12 | reported | 3656 | 0.0277 | rs8016766 |  |  |  |  |  |  |  |  |  |  |  |  |  |  |  |
| 0.2991 | 20 | igd | 0.2723 | C | \|\| id:ebi-a-GCST90001801 | ebi-a-GCST90001801 | TRUE | G | 44781215 | 3.59E-15 | reported | 1836 | 0.03769 | rs6074042 |  |  |  |  |  |  |  |  |  |  |  |  |  |  |  |
| -0.2756 | 19 | igd | 0.2722 | G | \|\| id:ebi-a-GCST90001533 | ebi-a-GCST90001533 | TRUE | A | 51574038 | 1.37E-13 | reported | 1857 | 0.03696 | rs7351079 |  |  |  |  |  |  |  |  |  |  |  |  |  |  |  |
| -0.3068 | 6 | igd | 0.2722 | G | \|\| id:ebi-a-GCST90002112 | ebi-a-GCST90002112 | TRUE | A | 31401095 | 8.68E-11 | reported | 1635 | 0.04697 | rs72848724 |  |  |  |  |  |  |  |  |  |  |  |  |  |  |  |
| -0.2641 | 19 | igd | 0.2711 | C | \|\| id:ebi-a-GCST90001956 | ebi-a-GCST90001956 | TRUE | T | 51340188 | 3.10E-10 | reported | 1579 | 0.0417 | rs2569745 |  |  |  |  |  |  |  |  |  |  |  |  |  |  |  |
| -0.3895 | 19 | igd | 0.271 | G | \|\| id:ebi-a-GCST90001948 | ebi-a-GCST90001948 | TRUE | A | 51574038 | 8.18E-22 | reported | 1633 | 0.04 | rs7351079 |  |  |  |  |  |  |  |  |  |  |  |  |  |  |  |
| -0.2647 | 19 | igd | 0.2709 | C | \|\| id:ebi-a-GCST90001957 | ebi-a-GCST90001957 | TRUE | T | 51340188 | 2.94E-10 | reported | 1578 | 0.04173 | rs2569745 |  |  |  |  |  |  |  |  |  |  |  |  |  |  |  |
| -0.4007 | 19 | igd | 0.2708 | G | \|\| id:ebi-a-GCST90001946 | ebi-a-GCST90001946 | TRUE | A | 51574038 | 7.97E-23 | reported | 1634 | 0.04013 | rs7351079 |  |  |  |  |  |  |  |  |  |  |  |  |  |  |  |
| -0.2673 | 19 | igd | 0.2708 | C | \|\| id:ebi-a-GCST90001947 | ebi-a-GCST90001947 | TRUE | T | 51340188 | 2.04E-10 | reported | 1577 | 0.04176 | rs2569745 |  |  |  |  |  |  |  |  |  |  |  |  |  |  |  |
| -0.3832 | 19 | igd | 0.2708 | G | \|\| id:ebi-a-GCST90001949 | ebi-a-GCST90001949 | TRUE | A | 51574038 | 2.91E-21 | reported | 1634 | 0.03992 | rs7351079 |  |  |  |  |  |  |  |  |  |  |  |  |  |  |  |
| -0.2949 | 6 | igd | 0.2706 | A | \|\| id:ebi-a-GCST90002111 | ebi-a-GCST90002111 | TRUE | T | 31819421 | 2.44E-10 | reported | 1635 | 0.04629 | rs2021007 |  |  |  |  |  |  |  |  |  |  |  |  |  |  |  |
| -0.3552 | 6 | igd | 0.2705 | T | \|\| id:ebi-a-GCST90002105 | ebi-a-GCST90002105 | TRUE | C | 31920873 | 3.80E-20 | reported | 2872 | 0.03835 | rs4151664 |  |  |  |  |  |  |  |  |  |  |  |  |  |  |  |
| 0.2482 | 1 | igd | 0.2702 | T | \|\| id:ebi-a-GCST90001832 | ebi-a-GCST90001832 | TRUE | C | 169851579 | 1.18E-17 | reported | 2872 | 0.02883 | rs6678351 |  |  |  |  |  |  |  |  |  |  |  |  |  |  |  |
| -0.3103 | 19 | igd | 0.2702 | G | \|\| id:ebi-a-GCST90001951 | ebi-a-GCST90001951 | TRUE | A | 51574038 | 1.04E-12 | reported | 1464 | 0.04316 | rs7351079 |  |  |  |  |  |  |  |  |  |  |  |  |  |  |  |
| -0.2341 | 19 | igd | 0.2702 | C | \|\| id:ebi-a-GCST90001952 | ebi-a-GCST90001952 | TRUE | T | 51340188 | 2.88E-08 | reported | 1569 | 0.04198 | rs2569745 |  |  |  |  |  |  |  |  |  |  |  |  |  |  |  |
| -0.1585 | 20 | igd | 0.2698 | C | \|\| id:ebi-a-GCST90001786 | ebi-a-GCST90001786 | TRUE | G | 44781215 | 1.71E-08 | reported | 3651 | 0.02805 | rs6074042 |  |  |  |  |  |  |  |  |  |  |  |  |  |  |  |
| -0.3517 | 6 | igd | 0.2698 | A | \|\| id:ebi-a-GCST90002108 | ebi-a-GCST90002108 | TRUE | C | 31820469 | 1.14E-13 | reported | 1579 | 0.04696 | rs495699 |  |  |  |  |  |  |  |  |  |  |  |  |  |  |  |
| -0.1919 | 20 | igd | 0.2697 | C | \|\| id:ebi-a-GCST90001433 | ebi-a-GCST90001433 | TRUE | G | 44781215 | 2.87E-11 | reported | 3656 | 0.02876 | rs6074042 |  |  |  |  |  |  |  |  |  |  |  |  |  |  |  |
| -0.2384 | 20 | igd | 0.2696 | C | \|\| id:ebi-a-GCST90001399 | ebi-a-GCST90001399 | TRUE | G | 44781215 | 9.71E-17 | reported | 3657 | 0.02856 | rs6074042 |  |  |  |  |  |  |  |  |  |  |  |  |  |  |  |
| 0.3372 | 20 | igd | 0.2696 | C | \|\| id:ebi-a-GCST90001798 | ebi-a-GCST90001798 | TRUE | G | 44781215 | 2.50E-33 | reported | 3657 | 0.02775 | rs6074042 |  |  |  |  |  |  |  |  |  |  |  |  |  |  |  |
| 0.2877 | 20 | igd | 0.2696 | C | \|\| id:ebi-a-GCST90001800 | ebi-a-GCST90001800 | TRUE | T | 44780038 | 5.86E-24 | reported | 3656 | 0.0283 | rs13037526 |  |  |  |  |  |  |  |  |  |  |  |  |  |  |  |
| 0.2727 | 20 | igd | 0.2696 | T | \|\| id:ebi-a-GCST90001802 | ebi-a-GCST90001802 | TRUE | C | 44781380 | 7.65E-22 | reported | 3657 | 0.02821 | rs6074043 |  |  |  |  |  |  |  |  |  |  |  |  |  |  |  |
| 0.3535 | 20 | igd | 0.2696 | T | \|\| id:ebi-a-GCST90001804 | ebi-a-GCST90001804 | TRUE | C | 44781380 | 7.16E-37 | reported | 3657 | 0.02756 | rs6074043 |  |  |  |  |  |  |  |  |  |  |  |  |  |  |  |
| 0.2773 | 20 | igd | 0.2696 | C | \|\| id:ebi-a-GCST90001805 | ebi-a-GCST90001805 | TRUE | G | 44781215 | 1.56E-22 | reported | 3657 | 0.02821 | rs6074042 |  |  |  |  |  |  |  |  |  |  |  |  |  |  |  |
| 0.2701 | 20 | igd | 0.2696 | C | \|\| id:ebi-a-GCST90001806 | ebi-a-GCST90001806 | TRUE | T | 44780038 | 1.74E-21 | reported | 3656 | 0.02819 | rs13037526 |  |  |  |  |  |  |  |  |  |  |  |  |  |  |  |
| 0.334 | 20 | igd | 0.2696 | T | \|\| id:ebi-a-GCST90001808 | ebi-a-GCST90001808 | TRUE | C | 44781380 | 2.90E-32 | reported | 3657 | 0.02797 | rs6074043 |  |  |  |  |  |  |  |  |  |  |  |  |  |  |  |
| -0.2382 | 19 | igd | 0.2696 | C | \|\| id:ebi-a-GCST90001953 | ebi-a-GCST90001953 | TRUE | T | 51340188 | 1.60E-08 | reported | 1558 | 0.04193 | rs2569745 |  |  |  |  |  |  |  |  |  |  |  |  |  |  |  |
| -0.1755 | 10 | igd | 0.2693 | G | \|\| id:ebi-a-GCST90001484 | ebi-a-GCST90001484 | TRUE | A | 95988042 | 1.46E-09 | reported | 3437 | 0.02894 | rs11187826 |  |  |  |  |  |  |  |  |  |  |  |  |  |  |  |
| -0.1625 | 10 | igd | 0.2693 | G | \|\| id:ebi-a-GCST90001485 | ebi-a-GCST90001485 | TRUE | A | 95988042 | 2.09E-08 | reported | 3437 | 0.02892 | rs11187826 |  |  |  |  |  |  |  |  |  |  |  |  |  |  |  |
| -0.2537 | 10 | igd | 0.2693 | G | \|\| id:ebi-a-GCST90001496 | ebi-a-GCST90001496 | TRUE | A | 95988042 | 3.14E-17 | reported | 3437 | 0.02989 | rs11187826 |  |  |  |  |  |  |  |  |  |  |  |  |  |  |  |
| -0.2657 | 10 | igd | 0.2693 | G | \|\| id:ebi-a-GCST90001497 | ebi-a-GCST90001497 | TRUE | A | 95988042 | 3.24E-19 | reported | 3437 | 0.02948 | rs11187826 |  |  |  |  |  |  |  |  |  |  |  |  |  |  |  |
| -0.2671 | 10 | igd | 0.2693 | G | \|\| id:ebi-a-GCST90001658 | ebi-a-GCST90001658 | TRUE | A | 95988042 | 1.18E-20 | reported | 3440 | 0.02848 | rs11187826 |  |  |  |  |  |  |  |  |  |  |  |  |  |  |  |
| -0.2819 | 10 | igd | 0.2693 | G | \|\| id:ebi-a-GCST90001659 | ebi-a-GCST90001659 | TRUE | A | 95988042 | 7.46E-23 | reported | 3440 | 0.02844 | rs11187826 |  |  |  |  |  |  |  |  |  |  |  |  |  |  |  |
| -0.259 | 10 | igd | 0.2693 | G | \|\| id:ebi-a-GCST90001670 | ebi-a-GCST90001670 | TRUE | A | 95988042 | 3.65E-18 | reported | 3440 | 0.02964 | rs11187826 |  |  |  |  |  |  |  |  |  |  |  |  |  |  |  |
| -0.2479 | 10 | igd | 0.2693 | G | \|\| id:ebi-a-GCST90001671 | ebi-a-GCST90001671 | TRUE | A | 95988042 | 1.16E-16 | reported | 3440 | 0.02976 | rs11187826 |  |  |  |  |  |  |  |  |  |  |  |  |  |  |  |
| -0.2501 | 19 | igd | 0.2693 | C | \|\| id:ebi-a-GCST90001955 | ebi-a-GCST90001955 | TRUE | T | 51340188 | 3.25E-09 | reported | 1554 | 0.04202 | rs2569745 |  |  |  |  |  |  |  |  |  |  |  |  |  |  |  |
| -0.2362 | 19 | igd | 0.2691 | C | \|\| id:ebi-a-GCST90001954 | ebi-a-GCST90001954 | TRUE | T | 51340188 | 2.26E-08 | reported | 1555 | 0.04203 | rs2569745 |  |  |  |  |  |  |  |  |  |  |  |  |  |  |  |
| -0.1752 | 10 | igd | 0.2686 | G | \|\| id:ebi-a-GCST90001483 | ebi-a-GCST90001483 | TRUE | A | 95988042 | 2.34E-09 | reported | 3405 | 0.02925 | rs11187826 |  |  |  |  |  |  |  |  |  |  |  |  |  |  |  |
| -0.2254 | 10 | igd | 0.2686 | G | \|\| id:ebi-a-GCST90001489 | ebi-a-GCST90001489 | TRUE | A | 95988042 | 6.79E-14 | reported | 3405 | 0.02996 | rs11187826 |  |  |  |  |  |  |  |  |  |  |  |  |  |  |  |
| -0.2608 | 10 | igd | 0.2686 | G | \|\| id:ebi-a-GCST90001495 | ebi-a-GCST90001495 | TRUE | A | 95988042 | 1.01E-17 | reported | 3405 | 0.03025 | rs11187826 |  |  |  |  |  |  |  |  |  |  |  |  |  |  |  |
| -0.2723 | 10 | igd | 0.2686 | G | \|\| id:ebi-a-GCST90001660 | ebi-a-GCST90001660 | TRUE | A | 95988042 | 1.44E-19 | reported | 3408 | 0.0299 | rs11187826 |  |  |  |  |  |  |  |  |  |  |  |  |  |  |  |
| 0.2427 | 6 | igd | 0.2684 | A | \|\| id:ebi-a-GCST90002110 | ebi-a-GCST90002110 | TRUE | G | 31102143 | 3.53E-08 | reported | 1634 | 0.04382 | rs10947140 |  |  |  |  |  |  |  |  |  |  |  |  |  |  |  |
| -0.1781 | 6 | igd | 0.2683 | A | \|\| id:ebi-a-GCST90002105 | ebi-a-GCST90002105 | TRUE | G | 33638657 | 1.54E-08 | reported | 2872 | 0.0314 | rs2274198 |  |  |  |  |  |  |  |  |  |  |  |  |  |  |  |
| 0.2391 | 3 | igd | 0.2681 | A | \|\| id:ebi-a-GCST90001995 | ebi-a-GCST90001995 | TRUE | G | 39355919 | 6.28E-17 | reported | 3590 | 0.02846 | rs72865957 |  |  |  |  |  |  |  |  |  |  |  |  |  |  |  |
| 0.1582 | 3 | igd | 0.2677 | T | \|\| id:ebi-a-GCST90001996 | ebi-a-GCST90001996 | TRUE | C | 39352960 | 3.44E-08 | reported | 3579 | 0.02861 | rs34253405 |  |  |  |  |  |  |  |  |  |  |  |  |  |  |  |
| 0.2836 | 3 | igd | 0.2675 | T | \|\| id:ebi-a-GCST90001997 | ebi-a-GCST90001997 | TRUE | C | 39352960 | 5.81E-23 | reported | 3590 | 0.02854 | rs34253405 |  |  |  |  |  |  |  |  |  |  |  |  |  |  |  |
| -0.227 | 10 | igd | 0.267 | A | \|\| id:ebi-a-GCST90001892 | ebi-a-GCST90001892 | TRUE | G | 97700813 | 2.52E-13 | reported | 2920 | 0.03087 | rs4561134 |  |  |  |  |  |  |  |  |  |  |  |  |  |  |  |
| 0.1398 | 2 | igd | 0.2664 | A | \|\| id:ebi-a-GCST90001666 | ebi-a-GCST90001666 | TRUE | C | 87061070 | 2.72E-08 | reported | 3408 | 0.02509 | rs61038907 |  |  |  |  |  |  |  |  |  |  |  |  |  |  |  |
| 0.1645 | 19 | igd | 0.26609 | A | \|\| id:prot-a-439 | prot-a-439 | TRUE | G | 51578855 | 6.76E-09 | reported | 3301 | 0.0284 | rs2569487 |  |  |  |  |  |  |  |  |  |  |  |  |  |  |  |
| -0.3256 | 6 | igd | 0.2657 | A | \|\| id:ebi-a-GCST90002105 | ebi-a-GCST90002105 | TRUE | C | 31820469 | 6.21E-17 | reported | 2872 | 0.0387 | rs495699 |  |  |  |  |  |  |  |  |  |  |  |  |  |  |  |
| -0.1799 | 10 | igd | 0.2656 | T | \|\| id:ebi-a-GCST90001491 | ebi-a-GCST90001491 | TRUE | A | 97794477 | 3.87E-10 | reported | 3437 | 0.02866 | rs10786262 |  |  |  |  |  |  |  |  |  |  |  |  |  |  |  |
| -0.1933 | 10 | igd | 0.2656 | T | \|\| id:ebi-a-GCST90001672 | ebi-a-GCST90001672 | TRUE | A | 97794477 | 4.58E-11 | reported | 3408 | 0.02926 | rs10786262 |  |  |  |  |  |  |  |  |  |  |  |  |  |  |  |
| -0.1985 | 10 | igd | 0.2654 | T | \|\| id:ebi-a-GCST90001670 | ebi-a-GCST90001670 | TRUE | A | 97794477 | 6.41E-12 | reported | 3440 | 0.02879 | rs10786262 |  |  |  |  |  |  |  |  |  |  |  |  |  |  |  |
| -0.2027 | 10 | igd | 0.2653 | A | \|\| id:ebi-a-GCST90001671 | ebi-a-GCST90001671 | TRUE | G | 97805074 | 2.42E-12 | reported | 3440 | 0.02882 | rs915506 |  |  |  |  |  |  |  |  |  |  |  |  |  |  |  |
| -0.182 | 6 | igd | 0.265 | G | \|\| id:ebi-a-GCST90001920 | ebi-a-GCST90001920 | TRUE | A | 33100189 | 1.00E-08 | reported | 3060 | 0.03168 | rs2092305 |  |  |  |  |  |  |  |  |  |  |  |  |  |  |  |
| 2.424 | 2 | igd | 0.2648 | T | \|\| id:ebi-a-GCST90001699 | ebi-a-GCST90001699 | TRUE | C | 38897823 | 1.36E-09 | reported | 3425 | 0.3988 | rs6736380 |  |  |  |  |  |  |  |  |  |  |  |  |  |  |  |
| 0.3454 | 2 | igd | 0.2648 | A | \|\| id:ebi-a-GCST90002102 | ebi-a-GCST90002102 | TRUE | T | 38895920 | 1.57E-32 | reported | 2919 | 0.02873 | rs13024840 |  |  |  |  |  |  |  |  |  |  |  |  |  |  |  |
| -0.1835 | 7 | igd | 0.2645 | C | \|\| id:ebi-a-GCST90001474 | ebi-a-GCST90001474 | TRUE | T | 50307710 | 2.25E-10 | reported | 3410 | 0.02885 | rs876036 |  |  |  |  |  |  |  |  |  |  |  |  |  |  |  |
| -0.1686 | 22 | igd | 0.264 | G | \|\| id:ebi-a-GCST90001702 | ebi-a-GCST90001702 | TRUE | A | 44323219 | 3.77E-09 | reported | 3657 | 0.02853 | rs9625961 |  |  |  |  |  |  |  |  |  |  |  |  |  |  |  |
| -0.1603 | 22 | igd | 0.264 | G | \|\| id:ebi-a-GCST90001703 | ebi-a-GCST90001703 | TRUE | A | 44323219 | 2.12E-08 | reported | 3657 | 0.02854 | rs9625961 |  |  |  |  |  |  |  |  |  |  |  |  |  |  |  |
| -0.1587 | 22 | igd | 0.264 | G | \|\| id:ebi-a-GCST90001704 | ebi-a-GCST90001704 | TRUE | A | 44323219 | 2.80E-08 | reported | 3657 | 0.02851 | rs9625961 |  |  |  |  |  |  |  |  |  |  |  |  |  |  |  |
| -0.1603 | 22 | igd | 0.264 | G | \|\| id:ebi-a-GCST90001710 | ebi-a-GCST90001710 | TRUE | A | 44323219 | 1.90E-08 | reported | 3657 | 0.02845 | rs9625961 |  |  |  |  |  |  |  |  |  |  |  |  |  |  |  |
| -0.1592 | 22 | igd | 0.264 | G | \|\| id:ebi-a-GCST90001711 | ebi-a-GCST90001711 | TRUE | A | 44323219 | 2.23E-08 | reported | 3657 | 0.02841 | rs9625961 |  |  |  |  |  |  |  |  |  |  |  |  |  |  |  |
| -0.2465 | 10 | igd | 0.2639 | A | \|\| id:ebi-a-GCST90002030 | ebi-a-GCST90002030 | TRUE | C | 96127448 | 9.85E-15 | reported | 2920 | 0.03167 | rs113544984 |  |  |  |  |  |  |  |  |  |  |  |  |  |  |  |
| -0.2023 | 10 | igd | 0.2639 | T | \|\| id:ebi-a-GCST90002032 | ebi-a-GCST90002032 | TRUE | C | 96125361 | 4.85E-10 | reported | 2920 | 0.03239 | rs11187907 |  |  |  |  |  |  |  |  |  |  |  |  |  |  |  |
| -0.1643 | 10 | igd | 0.2638 | T | \|\| id:ebi-a-GCST90001672 | ebi-a-GCST90001672 | TRUE | C | 97211736 | 2.13E-08 | reported | 3408 | 0.02926 | rs10786211 |  |  |  |  |  |  |  |  |  |  |  |  |  |  |  |
| 0.1697 | 2 | igd | 0.2636 | A | \|\| id:ebi-a-GCST90002100 | ebi-a-GCST90002100 | TRUE | T | 38895920 | 2.65E-08 | reported | 2910 | 0.03042 | rs13024840 |  |  |  |  |  |  |  |  |  |  |  |  |  |  |  |
| -0.2207 | 6 | igd | 0.2636 | C | \|\| id:ebi-a-GCST90002105 | ebi-a-GCST90002105 | TRUE | G | 31537296 | 6.95E-09 | reported | 2872 | 0.03798 | rs9267499 |  |  |  |  |  |  |  |  |  |  |  |  |  |  |  |
| -0.2533 | 7 | igd | 0.2635 | C | \|\| id:ebi-a-GCST90001523 | ebi-a-GCST90001523 | TRUE | T | 50307710 | 1.49E-11 | reported | 1858 | 0.03729 | rs876036 |  |  |  |  |  |  |  |  |  |  |  |  |  |  |  |
| -0.1862 | 10 | igd | 0.2635 | A | \|\| id:ebi-a-GCST90002031 | ebi-a-GCST90002031 | TRUE | T | 96124838 | 1.18E-08 | reported | 2920 | 0.03255 | rs11187906 |  |  |  |  |  |  |  |  |  |  |  |  |  |  |  |
| -0.31 | 6 | igd | 0.2634 | G | \|\| id:ebi-a-GCST90002105 | ebi-a-GCST90002105 | TRUE | A | 31401095 | 3.54E-15 | reported | 2872 | 0.03917 | rs72848724 |  |  |  |  |  |  |  |  |  |  |  |  |  |  |  |
| -0.2382 | 6 | igd | 0.2633 | A | \|\| id:ebi-a-GCST90001820 | ebi-a-GCST90001820 | TRUE | C | 31780594 | 3.13E-12 | reported | 3656 | 0.03405 | rs481825 |  |  |  |  |  |  |  |  |  |  |  |  |  |  |  |
| -0.1907 | 6 | igd | 0.2631 | C | \|\| id:ebi-a-GCST90001820 | ebi-a-GCST90001820 | TRUE | G | 31537296 | 1.36E-08 | reported | 3656 | 0.03351 | rs9267499 |  |  |  |  |  |  |  |  |  |  |  |  |  |  |  |
| -0.2898 | 6 | igd | 0.263 | A | \|\| id:ebi-a-GCST90002109 | ebi-a-GCST90002109 | TRUE | G | 30955359 | 4.37E-10 | reported | 1578 | 0.04615 | rs2844677 |  |  |  |  |  |  |  |  |  |  |  |  |  |  |  |
| -0.2874 | 6 | igd | 0.2629 | A | \|\| id:ebi-a-GCST90002110 | ebi-a-GCST90002110 | TRUE | G | 30955359 | 1.20E-09 | reported | 1634 | 0.047 | rs2844677 |  |  |  |  |  |  |  |  |  |  |  |  |  |  |  |
| -0.3632 | 6 | igd | 0.2628 | A | \|\| id:ebi-a-GCST90002108 | ebi-a-GCST90002108 | TRUE | G | 30955359 | 9.76E-15 | reported | 1579 | 0.04646 | rs2844677 |  |  |  |  |  |  |  |  |  |  |  |  |  |  |  |
| -0.2936 | 6 | igd | 0.2627 | A | \|\| id:ebi-a-GCST90002111 | ebi-a-GCST90002111 | TRUE | G | 30955359 | 2.39E-10 | reported | 1635 | 0.04606 | rs2844677 |  |  |  |  |  |  |  |  |  |  |  |  |  |  |  |
| 0.186 | 2 | igd | 0.2624 | T | \|\| id:ebi-a-GCST90001664 | ebi-a-GCST90001664 | TRUE | C | 38896631 | 4.50E-12 | reported | 3440 | 0.02679 | rs11688977 |  |  |  |  |  |  |  |  |  |  |  |  |  |  |  |
| 0.59 | 10 | igd | 0.2623 | T | \|\| id:ebi-a-GCST90001483 | ebi-a-GCST90001483 | TRUE | C | 97440812 | 2.29E-99 | reported | 3405 | 0.02697 | rs7073010 |  |  |  |  |  |  |  |  |  |  |  |  |  |  |  |
| 0.1804 | 22 | igd | 0.2621 | T | \|\| id:ebi-a-GCST90001702 | ebi-a-GCST90001702 | TRUE | C | 40415140 | 3.47E-10 | reported | 3657 | 0.02867 | rs2071771 |  |  |  |  |  |  |  |  |  |  |  |  |  |  |  |
| 0.1725 | 22 | igd | 0.2621 | T | \|\| id:ebi-a-GCST90001703 | ebi-a-GCST90001703 | TRUE | C | 40415140 | 1.96E-09 | reported | 3657 | 0.02867 | rs2071771 |  |  |  |  |  |  |  |  |  |  |  |  |  |  |  |
| 0.1624 | 22 | igd | 0.2621 | T | \|\| id:ebi-a-GCST90001704 | ebi-a-GCST90001704 | TRUE | C | 40415140 | 1.54E-08 | reported | 3657 | 0.02865 | rs2071771 |  |  |  |  |  |  |  |  |  |  |  |  |  |  |  |
| 0.1642 | 22 | igd | 0.2621 | T | \|\| id:ebi-a-GCST90001710 | ebi-a-GCST90001710 | TRUE | C | 40415140 | 1.03E-08 | reported | 3657 | 0.02862 | rs2071771 |  |  |  |  |  |  |  |  |  |  |  |  |  |  |  |
| 0.1592 | 22 | igd | 0.2621 | T | \|\| id:ebi-a-GCST90001711 | ebi-a-GCST90001711 | TRUE | C | 40415140 | 2.63E-08 | reported | 3657 | 0.02855 | rs2071771 |  |  |  |  |  |  |  |  |  |  |  |  |  |  |  |
| -16.83 | 2 | igd | 0.262 | T | \|\| id:ebi-a-GCST90001695 | ebi-a-GCST90001695 | TRUE | C | 38896631 | 3.57E-08 | reported | 3408 | 3.047 | rs11688977 |  |  |  |  |  |  |  |  |  |  |  |  |  |  |  |
| -3.154 | 2 | igd | 0.262 | T | \|\| id:ebi-a-GCST90001696 | ebi-a-GCST90001696 | TRUE | C | 38896631 | 1.15E-19 | reported | 3425 | 0.3455 | rs11688977 |  |  |  |  |  |  |  |  |  |  |  |  |  |  |  |
| -1.024 | 2 | igd | 0.262 | T | \|\| id:ebi-a-GCST90001697 | ebi-a-GCST90001697 | TRUE | C | 38896631 | 6.31E-13 | reported | 3425 | 0.1418 | rs11688977 |  |  |  |  |  |  |  |  |  |  |  |  |  |  |  |
| -0.1971 | 7 | igd | 0.2619 | C | \|\| id:ebi-a-GCST90001460 | ebi-a-GCST90001460 | TRUE | G | 50308811 | 9.51E-13 | reported | 3374 | 0.02752 | rs876039 |  |  |  |  |  |  |  |  |  |  |  |  |  |  |  |
| -0.3366 | 19 | igd | 0.2619 | G | \|\| id:ebi-a-GCST90001956 | ebi-a-GCST90001956 | TRUE | A | 51574038 | 2.54E-15 | reported | 1579 | 0.04212 | rs7351079 |  |  |  |  |  |  |  |  |  |  |  |  |  |  |  |
| -0.3413 | 19 | igd | 0.2617 | G | \|\| id:ebi-a-GCST90001957 | ebi-a-GCST90001957 | TRUE | A | 51574038 | 1.07E-15 | reported | 1578 | 0.04213 | rs7351079 |  |  |  |  |  |  |  |  |  |  |  |  |  |  |  |
| -0.3464 | 6 | igd | 0.2617 | C | \|\| id:ebi-a-GCST90002105 | ebi-a-GCST90002105 | TRUE | G | 31461492 | 1.61E-19 | reported | 2872 | 0.03805 | rs9267325 |  |  |  |  |  |  |  |  |  |  |  |  |  |  |  |
| -0.415 | 6 | igd | 0.2616 | T | \|\| id:ebi-a-GCST90002108 | ebi-a-GCST90002108 | TRUE | C | 32666358 | 4.72E-18 | reported | 1579 | 0.04734 | rs1794283 |  |  |  |  |  |  |  |  |  |  |  |  |  |  |  |
| 0.1874 | 1 | igd | 0.2615 | T | \|\| id:ebi-a-GCST90001866 | ebi-a-GCST90001866 | TRUE | C | 167451115 | 1.16E-09 | reported | 2914 | 0.03069 | rs12737372 |  |  |  |  |  |  |  |  |  |  |  |  |  |  |  |
| -0.2113 | 20 | igd | 0.2615 | C | \|\| id:ebi-a-GCST90001985 | ebi-a-GCST90001985 | TRUE | G | 44797583 | 5.26E-14 | reported | 3629 | 0.02796 | rs1320999 |  |  |  |  |  |  |  |  |  |  |  |  |  |  |  |
| -0.286 | 6 | igd | 0.2615 | C | \|\| id:ebi-a-GCST90002111 | ebi-a-GCST90002111 | TRUE | G | 31461492 | 1.15E-09 | reported | 1635 | 0.04671 | rs9267325 |  |  |  |  |  |  |  |  |  |  |  |  |  |  |  |
| -0.3324 | 6 | igd | 0.2615 | C | \|\| id:ebi-a-GCST90002112 | ebi-a-GCST90002112 | TRUE | G | 31461492 | 2.48E-12 | reported | 1635 | 0.0471 | rs9267325 |  |  |  |  |  |  |  |  |  |  |  |  |  |  |  |
| -0.326 | 19 | igd | 0.2613 | G | \|\| id:ebi-a-GCST90001947 | ebi-a-GCST90001947 | TRUE | A | 51574038 | 2.43E-14 | reported | 1577 | 0.04234 | rs7351079 |  |  |  |  |  |  |  |  |  |  |  |  |  |  |  |
| 0.204 | 1 | igd | 0.261 | T | \|\| id:ebi-a-GCST90001863 | ebi-a-GCST90001863 | TRUE | C | 167451115 | 2.41E-11 | reported | 2919 | 0.03042 | rs12737372 |  |  |  |  |  |  |  |  |  |  |  |  |  |  |  |
| -0.2013 | 20 | igd | 0.261 | C | \|\| id:ebi-a-GCST90001989 | ebi-a-GCST90001989 | TRUE | G | 44797583 | 8.99E-13 | reported | 3621 | 0.02807 | rs1320999 |  |  |  |  |  |  |  |  |  |  |  |  |  |  |  |
| 0.2665 | 3 | igd | 0.2609 | G | \|\| id:ebi-a-GCST90001798 | ebi-a-GCST90001798 | TRUE | A | 32526818 | 3.30E-21 | reported | 3657 | 0.02802 | rs342766 |  |  |  |  |  |  |  |  |  |  |  |  |  |  |  |
| 0.2022 | 3 | igd | 0.2609 | G | \|\| id:ebi-a-GCST90001802 | ebi-a-GCST90001802 | TRUE | A | 32526818 | 1.46E-12 | reported | 3657 | 0.02846 | rs342766 |  |  |  |  |  |  |  |  |  |  |  |  |  |  |  |
| 0.2337 | 3 | igd | 0.2609 | G | \|\| id:ebi-a-GCST90001804 | ebi-a-GCST90001804 | TRUE | A | 32526818 | 9.26E-17 | reported | 3657 | 0.02797 | rs342766 |  |  |  |  |  |  |  |  |  |  |  |  |  |  |  |
| 0.1987 | 3 | igd | 0.2609 | G | \|\| id:ebi-a-GCST90001805 | ebi-a-GCST90001805 | TRUE | A | 32526818 | 3.46E-12 | reported | 3657 | 0.02846 | rs342766 |  |  |  |  |  |  |  |  |  |  |  |  |  |  |  |
| 0.2453 | 3 | igd | 0.2609 | G | \|\| id:ebi-a-GCST90001808 | ebi-a-GCST90001808 | TRUE | A | 32526818 | 6.56E-18 | reported | 3657 | 0.0283 | rs342766 |  |  |  |  |  |  |  |  |  |  |  |  |  |  |  |
| 0.2386 | 3 | igd | 0.2608 | G | \|\| id:ebi-a-GCST90001800 | ebi-a-GCST90001800 | TRUE | A | 32526818 | 8.18E-17 | reported | 3656 | 0.02852 | rs342766 |  |  |  |  |  |  |  |  |  |  |  |  |  |  |  |
| 0.2329 | 3 | igd | 0.2608 | G | \|\| id:ebi-a-GCST90001806 | ebi-a-GCST90001806 | TRUE | A | 32526818 | 3.09E-16 | reported | 3656 | 0.02838 | rs342766 |  |  |  |  |  |  |  |  |  |  |  |  |  |  |  |
| 0.1835 | 3 | igd | 0.2606 | T | \|\| id:ebi-a-GCST90002013 | ebi-a-GCST90002013 | TRUE | G | 46306130 | 9.78E-09 | reported | 2870 | 0.03191 | rs3091250 |  |  |  |  |  |  |  |  |  |  |  |  |  |  |  |
| -0.3677 | 19 | igd | 0.2604 | G | \|\| id:ebi-a-GCST90001952 | ebi-a-GCST90001952 | TRUE | A | 51574038 | 9.03E-18 | reported | 1569 | 0.04232 | rs7351079 |  |  |  |  |  |  |  |  |  |  |  |  |  |  |  |
| 0.1482 | 2 | igd | 0.2601 | A | \|\| id:ebi-a-GCST90001666 | ebi-a-GCST90001666 | TRUE | G | 38821513 | 4.98E-09 | reported | 3408 | 0.02528 | rs36057155 |  |  |  |  |  |  |  |  |  |  |  |  |  |  |  |
| 0.246 | 16 | igd | 0.2598 | T | \|\| id:ebi-a-GCST90002089 | ebi-a-GCST90002089 | TRUE | C | 31164675 | 1.43E-14 | reported | 2850 | 0.0318 | rs137879778 |  |  |  |  |  |  |  |  |  |  |  |  |  |  |  |
| 0.2263 | 2 | igd | 0.2597 | A | \|\| id:ebi-a-GCST90002101 | ebi-a-GCST90002101 | TRUE | G | 38821513 | 2.00E-13 | reported | 2911 | 0.03064 | rs36057155 |  |  |  |  |  |  |  |  |  |  |  |  |  |  |  |
| 0.2257 | 19 | igd | 0.2596 | T | \|\| id:ebi-a-GCST90002073 | ebi-a-GCST90002073 | TRUE | C | 18282335 | 1.19E-12 | reported | 2850 | 0.03162 | rs72999501 |  |  |  |  |  |  |  |  |  |  |  |  |  |  |  |
| -0.2439 | 6 | igd | 0.2594 | G | \|\| id:ebi-a-GCST90001820 | ebi-a-GCST90001820 | TRUE | T | 32671207 | 6.87E-13 | reported | 3656 | 0.03383 | rs1794511 |  |  |  |  |  |  |  |  |  |  |  |  |  |  |  |
| 0.2556 | 16 | igd | 0.2594 | T | \|\| id:ebi-a-GCST90002087 | ebi-a-GCST90002087 | TRUE | C | 31164675 | 1.97E-16 | reported | 2872 | 0.0309 | rs137879778 |  |  |  |  |  |  |  |  |  |  |  |  |  |  |  |
| 0.2444 | 16 | igd | 0.2594 | T | \|\| id:ebi-a-GCST90002088 | ebi-a-GCST90002088 | TRUE | C | 31164675 | 3.17E-15 | reported | 2872 | 0.03083 | rs137879778 |  |  |  |  |  |  |  |  |  |  |  |  |  |  |  |
| 0.1762 | 2 | igd | 0.2593 | A | \|\| id:ebi-a-GCST90001664 | ebi-a-GCST90001664 | TRUE | G | 38821513 | 4.36E-11 | reported | 3440 | 0.02665 | rs36057155 |  |  |  |  |  |  |  |  |  |  |  |  |  |  |  |
| 0.2098 | 19 | igd | 0.259 | T | \|\| id:ebi-a-GCST90002074 | ebi-a-GCST90002074 | TRUE | C | 18282335 | 5.48E-12 | reported | 3112 | 0.03031 | rs72999501 |  |  |  |  |  |  |  |  |  |  |  |  |  |  |  |
| -0.3338 | 6 | igd | 0.259 | T | \|\| id:ebi-a-GCST90002111 | ebi-a-GCST90002111 | TRUE | G | 31937866 | 3.41E-12 | reported | 1635 | 0.0476 | rs9267786 |  |  |  |  |  |  |  |  |  |  |  |  |  |  |  |
| -0.2858 | 6 | igd | 0.2589 | T | \|\| id:ebi-a-GCST90002109 | ebi-a-GCST90002109 | TRUE | G | 31937866 | 3.14E-09 | reported | 1578 | 0.04797 | rs9267786 |  |  |  |  |  |  |  |  |  |  |  |  |  |  |  |
| 0.1655 | 7 | igd | 0.2589 | C | \|\| id:ebi-a-GCST90001994 | ebi-a-GCST90001994 | TRUE | T | 50307710 | 5.07E-09 | reported | 3590 | 0.02824 | rs876036 |  |  |  |  |  |  |  |  |  |  |  |  |  |  |  |
| -0.2043 | 10 | igd | 0.2587 | A | \|\| id:ebi-a-GCST90001496 | ebi-a-GCST90001496 | TRUE | C | 96127448 | 2.81E-11 | reported | 3437 | 0.03059 | rs113544984 |  |  |  |  |  |  |  |  |  |  |  |  |  |  |  |
| -0.2163 | 10 | igd | 0.2587 | A | \|\| id:ebi-a-GCST90001497 | ebi-a-GCST90001497 | TRUE | C | 96127448 | 9.27E-13 | reported | 3437 | 0.03018 | rs113544984 |  |  |  |  |  |  |  |  |  |  |  |  |  |  |  |
| 0.2764 | 2 | igd | 0.2587 | A | \|\| id:ebi-a-GCST90001508 | ebi-a-GCST90001508 | TRUE | T | 38895920 | 4.05E-21 | reported | 3437 | 0.02911 | rs13024840 |  |  |  |  |  |  |  |  |  |  |  |  |  |  |  |
| 0.2465 | 2 | igd | 0.2587 | A | \|\| id:ebi-a-GCST90001509 | ebi-a-GCST90001509 | TRUE | T | 38895920 | 4.22E-17 | reported | 3437 | 0.02917 | rs13024840 |  |  |  |  |  |  |  |  |  |  |  |  |  |  |  |
| -0.2223 | 10 | igd | 0.2587 | A | \|\| id:ebi-a-GCST90001658 | ebi-a-GCST90001658 | TRUE | C | 96127448 | 3.13E-14 | reported | 3440 | 0.02916 | rs113544984 |  |  |  |  |  |  |  |  |  |  |  |  |  |  |  |
| -0.2314 | 10 | igd | 0.2587 | A | \|\| id:ebi-a-GCST90001659 | ebi-a-GCST90001659 | TRUE | C | 96127448 | 2.67E-15 | reported | 3440 | 0.02914 | rs113544984 |  |  |  |  |  |  |  |  |  |  |  |  |  |  |  |
| -0.1856 | 7 | igd | 0.2587 | T | \|\| id:ebi-a-GCST90001832 | ebi-a-GCST90001832 | TRUE | C | 50271499 | 7.29E-10 | reported | 2872 | 0.03003 | rs11773763 |  |  |  |  |  |  |  |  |  |  |  |  |  |  |  |
| 0.1914 | 1 | igd | 0.2586 | T | \|\| id:ebi-a-GCST90001852 | ebi-a-GCST90001852 | TRUE | C | 167451115 | 2.36E-09 | reported | 2649 | 0.03195 | rs12737372 |  |  |  |  |  |  |  |  |  |  |  |  |  |  |  |
| -0.2185 | 10 | igd | 0.2584 | G | \|\| id:ebi-a-GCST90001660 | ebi-a-GCST90001660 | TRUE | A | 96106603 | 1.15E-12 | reported | 3408 | 0.03061 | rs11187897 |  |  |  |  |  |  |  |  |  |  |  |  |  |  |  |
| -0.1839 | 10 | igd | 0.2583 | G | \|\| id:ebi-a-GCST90001489 | ebi-a-GCST90001489 | TRUE | A | 96106603 | 2.02E-09 | reported | 3405 | 0.03059 | rs11187897 |  |  |  |  |  |  |  |  |  |  |  |  |  |  |  |
| 0.2874 | 3 | igd | 0.2582 | G | \|\| id:ebi-a-GCST90001801 | ebi-a-GCST90001801 | TRUE | A | 32526818 | 8.45E-14 | reported | 1836 | 0.03822 | rs342766 |  |  |  |  |  |  |  |  |  |  |  |  |  |  |  |
| -0.2117 | 10 | igd | 0.2581 | A | \|\| id:ebi-a-GCST90001495 | ebi-a-GCST90001495 | TRUE | T | 96124838 | 1.02E-11 | reported | 3405 | 0.031 | rs11187906 |  |  |  |  |  |  |  |  |  |  |  |  |  |  |  |
| 0.1718 | 2 | igd | 0.2581 | T | \|\| id:ebi-a-GCST90001665 | ebi-a-GCST90001665 | TRUE | A | 38895918 | 1.74E-09 | reported | 3440 | 0.02847 | rs12998415 |  |  |  |  |  |  |  |  |  |  |  |  |  |  |  |
| -0.2934 | 6 | igd | 0.2581 | G | \|\| id:ebi-a-GCST90002108 | ebi-a-GCST90002108 | TRUE | A | 30376365 | 6.59E-10 | reported | 1579 | 0.04721 | rs9261798 |  |  |  |  |  |  |  |  |  |  |  |  |  |  |  |
| -0.3645 | 19 | igd | 0.258 | G | \|\| id:ebi-a-GCST90001953 | ebi-a-GCST90001953 | TRUE | A | 51574038 | 1.15E-17 | reported | 1558 | 0.04209 | rs7351079 |  |  |  |  |  |  |  |  |  |  |  |  |  |  |  |
| -0.3351 | 19 | igd | 0.258 | G | \|\| id:ebi-a-GCST90001955 | ebi-a-GCST90001955 | TRUE | A | 51574038 | 5.07E-15 | reported | 1554 | 0.0424 | rs7351079 |  |  |  |  |  |  |  |  |  |  |  |  |  |  |  |
| -0.3642 | 19 | igd | 0.2579 | G | \|\| id:ebi-a-GCST90001954 | ebi-a-GCST90001954 | TRUE | A | 51574038 | 1.40E-17 | reported | 1555 | 0.04217 | rs7351079 |  |  |  |  |  |  |  |  |  |  |  |  |  |  |  |
| -0.1971 | 22 | igd | 0.2577 | A | \|\| id:ebi-a-GCST90001702 | ebi-a-GCST90001702 | TRUE | C | 40716488 | 1.49E-11 | reported | 3657 | 0.02911 | rs4470425 |  |  |  |  |  |  |  |  |  |  |  |  |  |  |  |
| -0.1933 | 22 | igd | 0.2577 | A | \|\| id:ebi-a-GCST90001703 | ebi-a-GCST90001703 | TRUE | C | 40716488 | 3.57E-11 | reported | 3657 | 0.02911 | rs4470425 |  |  |  |  |  |  |  |  |  |  |  |  |  |  |  |
| -0.1921 | 22 | igd | 0.2577 | A | \|\| id:ebi-a-GCST90001704 | ebi-a-GCST90001704 | TRUE | C | 40716488 | 4.49E-11 | reported | 3657 | 0.02908 | rs4470425 |  |  |  |  |  |  |  |  |  |  |  |  |  |  |  |
| -0.1903 | 22 | igd | 0.2577 | A | \|\| id:ebi-a-GCST90001710 | ebi-a-GCST90001710 | TRUE | C | 40716488 | 6.54E-11 | reported | 3657 | 0.02904 | rs4470425 |  |  |  |  |  |  |  |  |  |  |  |  |  |  |  |
| -0.1912 | 22 | igd | 0.2577 | A | \|\| id:ebi-a-GCST90001711 | ebi-a-GCST90001711 | TRUE | C | 40716488 | 4.71E-11 | reported | 3657 | 0.02897 | rs4470425 |  |  |  |  |  |  |  |  |  |  |  |  |  |  |  |
| -0.2186 | 10 | igd | 0.2573 | A | \|\| id:ebi-a-GCST90001672 | ebi-a-GCST90001672 | TRUE | G | 96122857 | 1.97E-12 | reported | 3408 | 0.03095 | rs7101207 |  |  |  |  |  |  |  |  |  |  |  |  |  |  |  |
| -0.2241 | 6 | igd | 0.2568 | T | \|\| id:ebi-a-GCST90002105 | ebi-a-GCST90002105 | TRUE | C | 32917544 | 4.04E-11 | reported | 2872 | 0.03381 | rs1063478 |  |  |  |  |  |  |  |  |  |  |  |  |  |  |  |
| -0.2162 | 22 | igd | 0.2565 | A | \|\| id:ebi-a-GCST90001703 | ebi-a-GCST90001703 | TRUE | G | 43715288 | 9.52E-15 | reported | 3657 | 0.02779 | rs4822279 |  |  |  |  |  |  |  |  |  |  |  |  |  |  |  |
| -0.2146 | 22 | igd | 0.2565 | A | \|\| id:ebi-a-GCST90001704 | ebi-a-GCST90001704 | TRUE | G | 43715288 | 1.39E-14 | reported | 3657 | 0.02776 | rs4822279 |  |  |  |  |  |  |  |  |  |  |  |  |  |  |  |
| -0.1876 | 6 | igd | 0.2565 | A | \|\| id:ebi-a-GCST90002119 | ebi-a-GCST90002119 | TRUE | G | 31580539 | 3.04E-08 | reported | 2920 | 0.03378 | rs2471960 |  |  |  |  |  |  |  |  |  |  |  |  |  |  |  |
| -0.1714 | 2 | igd | 0.2562 | C | \|\| id:ebi-a-GCST90001889 | ebi-a-GCST90001889 | TRUE | T | 204862688 | 1.73E-08 | reported | 2920 | 0.03032 | rs11682773 |  |  |  |  |  |  |  |  |  |  |  |  |  |  |  |
| -0.1751 | 2 | igd | 0.2562 | C | \|\| id:ebi-a-GCST90001890 | ebi-a-GCST90001890 | TRUE | T | 204862688 | 3.29E-08 | reported | 2920 | 0.03161 | rs11682773 |  |  |  |  |  |  |  |  |  |  |  |  |  |  |  |
| -0.18 | 10 | igd | 0.2557 | T | \|\| id:ebi-a-GCST90002076 | ebi-a-GCST90002076 | TRUE | C | 126383857 | 4.79E-09 | reported | 3113 | 0.03066 | rs11597613 |  |  |  |  |  |  |  |  |  |  |  |  |  |  |  |
| -0.265 | 6 | igd | 0.2557 | A | \|\| id:ebi-a-GCST90002105 | ebi-a-GCST90002105 | TRUE | G | 30955359 | 1.13E-11 | reported | 2872 | 0.03887 | rs2844677 |  |  |  |  |  |  |  |  |  |  |  |  |  |  |  |
| 0.2471 | 6 | igd | 0.2557 | A | \|\| id:ebi-a-GCST90002112 | ebi-a-GCST90002112 | TRUE | C | 32965889 | 8.06E-09 | reported | 1635 | 0.04263 | rs4713602 |  |  |  |  |  |  |  |  |  |  |  |  |  |  |  |
| -0.2523 | 7 | igd | 0.255 | T | \|\| id:ebi-a-GCST90002046 | ebi-a-GCST90002046 | TRUE | G | 50318308 | 8.80E-10 | reported | 1635 | 0.04091 | rs17552904 |  |  |  |  |  |  |  |  |  |  |  |  |  |  |  |
| 0.2313 | 6 | igd | 0.2549 | A | \|\| id:ebi-a-GCST90002105 | ebi-a-GCST90002105 | TRUE | C | 32965889 | 3.22E-12 | reported | 2872 | 0.03305 | rs4713602 |  |  |  |  |  |  |  |  |  |  |  |  |  |  |  |
| 0.1762 | 7 | igd | 0.2548 | G | \|\| id:ebi-a-GCST90002098 | ebi-a-GCST90002098 | TRUE | T | 28172739 | 4.26E-09 | reported | 2910 | 0.0299 | rs917116 |  |  |  |  |  |  |  |  |  |  |  |  |  |  |  |
| -0.1849 | 2 | igd | 0.2546 | A | \|\| id:ebi-a-GCST90001887 | ebi-a-GCST90001887 | TRUE | C | 204852795 | 2.94E-09 | reported | 2920 | 0.03106 | rs16840486 |  |  |  |  |  |  |  |  |  |  |  |  |  |  |  |
| 0.1862 | 10 | igd | 0.2544 | C | \|\| id:ebi-a-GCST90001658 | ebi-a-GCST90001658 | TRUE | A | 95252188 | 3.38E-11 | reported | 3440 | 0.028 | rs11187462 |  |  |  |  |  |  |  |  |  |  |  |  |  |  |  |
| 0.205 | 10 | igd | 0.2544 | C | \|\| id:ebi-a-GCST90001659 | ebi-a-GCST90001659 | TRUE | A | 95252188 | 2.80E-13 | reported | 3440 | 0.02796 | rs11187462 |  |  |  |  |  |  |  |  |  |  |  |  |  |  |  |
| -0.2821 | 19 | igd | 0.2544 | G | \|\| id:ebi-a-GCST90001950 | ebi-a-GCST90001950 | TRUE | A | 51574038 | 5.38E-09 | reported | 1244 | 0.048 | rs7351079 |  |  |  |  |  |  |  |  |  |  |  |  |  |  |  |
| 0.2038 | 10 | igd | 0.2541 | C | \|\| id:ebi-a-GCST90001497 | ebi-a-GCST90001497 | TRUE | A | 95252188 | 2.41E-12 | reported | 3437 | 0.02897 | rs11187462 |  |  |  |  |  |  |  |  |  |  |  |  |  |  |  |
| -0.236 | 6 | igd | 0.254 | T | \|\| id:ebi-a-GCST90001820 | ebi-a-GCST90001820 | TRUE | C | 32030700 | 2.19E-11 | reported | 3656 | 0.03515 | rs9267797 |  |  |  |  |  |  |  |  |  |  |  |  |  |  |  |
| 0.2121 | 10 | igd | 0.2531 | C | \|\| id:ebi-a-GCST90001484 | ebi-a-GCST90001484 | TRUE | A | 95253744 | 9.82E-14 | reported | 3437 | 0.02837 | rs12251594 |  |  |  |  |  |  |  |  |  |  |  |  |  |  |  |
| 0.1791 | 10 | igd | 0.2531 | C | \|\| id:ebi-a-GCST90001485 | ebi-a-GCST90001485 | TRUE | A | 95253744 | 3.21E-10 | reported | 3437 | 0.0284 | rs12251594 |  |  |  |  |  |  |  |  |  |  |  |  |  |  |  |
| -0.2422 | 6 | igd | 0.253 | G | \|\| id:ebi-a-GCST90001820 | ebi-a-GCST90001820 | TRUE | A | 31918464 | 7.04E-12 | reported | 3656 | 0.0352 | rs4151659 |  |  |  |  |  |  |  |  |  |  |  |  |  |  |  |
| -0.2389 | 7 | igd | 0.2529 | T | \|\| id:ebi-a-GCST90002078 | ebi-a-GCST90002078 | TRUE | A | 92237396 | 5.55E-15 | reported | 3112 | 0.03042 | rs42031 |  |  |  |  |  |  |  |  |  |  |  |  |  |  |  |
| -0.2728 | 22 | igd | 0.2518 | G | \|\| id:ebi-a-GCST90001704 | ebi-a-GCST90001704 | TRUE | A | 44300847 | 2.01E-21 | reported | 3657 | 0.02853 | rs11090614 |  |  |  |  |  |  |  |  |  |  |  |  |  |  |  |
| -0.3054 | 20 | igd | 0.2516 | G | \|\| id:ebi-a-GCST90001801 | ebi-a-GCST90001801 | TRUE | T | 44779073 | 3.96E-15 | reported | 1836 | 0.03854 | rs56287761 |  |  |  |  |  |  |  |  |  |  |  |  |  |  |  |
| -0.1688 | 6 | igd | 0.2511 | A | \|\| id:ebi-a-GCST90001774 | ebi-a-GCST90001774 | TRUE | G | 106897047 | 3.07E-09 | reported | 3656 | 0.0284 | rs4141622 |  |  |  |  |  |  |  |  |  |  |  |  |  |  |  |
| 0.2245 | 16 | igd | 0.2509 | G | \|\| id:ebi-a-GCST90002105 | ebi-a-GCST90002105 | TRUE | A | 10970476 | 5.36E-13 | reported | 2872 | 0.03096 | rs12596540 |  |  |  |  |  |  |  |  |  |  |  |  |  |  |  |
| -0.2224 | 16 | igd | 0.2507 | C | \|\| id:ebi-a-GCST90001907 | ebi-a-GCST90001907 | TRUE | G | 401096 | 1.07E-12 | reported | 2910 | 0.03108 | rs9921342 |  |  |  |  |  |  |  |  |  |  |  |  |  |  |  |
| -0.1707 | 16 | igd | 0.2507 | C | \|\| id:ebi-a-GCST90001908 | ebi-a-GCST90001908 | TRUE | G | 401096 | 4.08E-08 | reported | 2910 | 0.03102 | rs9921342 |  |  |  |  |  |  |  |  |  |  |  |  |  |  |  |
| -0.3974 | 6 | igd | 0.2505 | C | \|\| id:ebi-a-GCST90002112 | ebi-a-GCST90002112 | TRUE | T | 32290650 | 1.54E-16 | reported | 1635 | 0.04764 | rs2143603 |  |  |  |  |  |  |  |  |  |  |  |  |  |  |  |
| -0.2525 | 2 | igd | 0.2498 | G | \|\| id:ebi-a-GCST90001896 | ebi-a-GCST90001896 | TRUE | A | 204581195 | 5.77E-15 | reported | 2920 | 0.03216 | rs17533594 |  |  |  |  |  |  |  |  |  |  |  |  |  |  |  |
| -0.3003 | 6 | igd | 0.2498 | C | \|\| id:ebi-a-GCST90002108 | ebi-a-GCST90002108 | TRUE | T | 31081205 | 8.86E-10 | reported | 1579 | 0.0487 | rs2233956 |  |  |  |  |  |  |  |  |  |  |  |  |  |  |  |
| -0.1916 | 6 | igd | 0.2497 | T | \|\| id:ebi-a-GCST90001692 | ebi-a-GCST90001692 | TRUE | C | 31138377 | 4.20E-09 | reported | 3440 | 0.03253 | rs2077010 |  |  |  |  |  |  |  |  |  |  |  |  |  |  |  |
| -0.388 | 6 | igd | 0.2497 | G | \|\| id:ebi-a-GCST90002110 | ebi-a-GCST90002110 | TRUE | A | 32309386 | 1.76E-15 | reported | 1634 | 0.04828 | rs9268255 |  |  |  |  |  |  |  |  |  |  |  |  |  |  |  |
| -0.3805 | 6 | igd | 0.2495 | G | \|\| id:ebi-a-GCST90002111 | ebi-a-GCST90002111 | TRUE | A | 32309386 | 1.89E-15 | reported | 1635 | 0.0474 | rs9268255 |  |  |  |  |  |  |  |  |  |  |  |  |  |  |  |
| -0.2677 | 6 | igd | 0.2495 | C | \|\| id:ebi-a-GCST90002111 | ebi-a-GCST90002111 | TRUE | T | 31081205 | 3.08E-08 | reported | 1635 | 0.04811 | rs2233956 |  |  |  |  |  |  |  |  |  |  |  |  |  |  |  |
| -0.2822 | 6 | igd | 0.2495 | C | \|\| id:ebi-a-GCST90002112 | ebi-a-GCST90002112 | TRUE | T | 31081205 | 7.98E-09 | reported | 1635 | 0.04867 | rs2233956 |  |  |  |  |  |  |  |  |  |  |  |  |  |  |  |
| -0.1984 | 7 | igd | 0.2495 | T | \|\| id:ebi-a-GCST90002018 | ebi-a-GCST90002018 | TRUE | A | 92237396 | 5.84E-10 | reported | 2850 | 0.03192 | rs42031 |  |  |  |  |  |  |  |  |  |  |  |  |  |  |  |
| 0.1952 | 22 | igd | 0.2494 | A | \|\| id:ebi-a-GCST90001702 | ebi-a-GCST90001702 | TRUE | G | 37847465 | 2.52E-11 | reported | 3657 | 0.02916 | rs6519064 |  |  |  |  |  |  |  |  |  |  |  |  |  |  |  |
| 0.1843 | 22 | igd | 0.2494 | A | \|\| id:ebi-a-GCST90001703 | ebi-a-GCST90001703 | TRUE | G | 37847465 | 3.02E-10 | reported | 3657 | 0.02918 | rs6519064 |  |  |  |  |  |  |  |  |  |  |  |  |  |  |  |
| 0.1872 | 22 | igd | 0.2494 | A | \|\| id:ebi-a-GCST90001704 | ebi-a-GCST90001704 | TRUE | G | 37847465 | 1.48E-10 | reported | 3657 | 0.02913 | rs6519064 |  |  |  |  |  |  |  |  |  |  |  |  |  |  |  |
| 0.192 | 22 | igd | 0.2494 | A | \|\| id:ebi-a-GCST90001710 | ebi-a-GCST90001710 | TRUE | G | 37847465 | 4.65E-11 | reported | 3657 | 0.02908 | rs6519064 |  |  |  |  |  |  |  |  |  |  |  |  |  |  |  |
| 0.1759 | 22 | igd | 0.2494 | A | \|\| id:ebi-a-GCST90001711 | ebi-a-GCST90001711 | TRUE | G | 37847465 | 1.53E-09 | reported | 3657 | 0.02904 | rs6519064 |  |  |  |  |  |  |  |  |  |  |  |  |  |  |  |
| -0.3273 | 6 | igd | 0.2494 | A | \|\| id:ebi-a-GCST90002110 | ebi-a-GCST90002110 | TRUE | G | 31573110 | 5.31E-11 | reported | 1634 | 0.04954 | rs9267517 |  |  |  |  |  |  |  |  |  |  |  |  |  |  |  |
| 0.1914 | 20 | igd | 0.2492 | G | \|\| id:ebi-a-GCST90001399 | ebi-a-GCST90001399 | TRUE | T | 44779073 | 4.35E-11 | reported | 3657 | 0.02895 | rs56287761 |  |  |  |  |  |  |  |  |  |  |  |  |  |  |  |
| -0.2639 | 20 | igd | 0.2492 | G | \|\| id:ebi-a-GCST90001798 | ebi-a-GCST90001798 | TRUE | T | 44779073 | 2.90E-20 | reported | 3657 | 0.02845 | rs56287761 |  |  |  |  |  |  |  |  |  |  |  |  |  |  |  |
| -0.2585 | 20 | igd | 0.2492 | G | \|\| id:ebi-a-GCST90001804 | ebi-a-GCST90001804 | TRUE | T | 44779073 | 1.28E-19 | reported | 3657 | 0.02836 | rs56287761 |  |  |  |  |  |  |  |  |  |  |  |  |  |  |  |
| -0.2298 | 20 | igd | 0.2492 | G | \|\| id:ebi-a-GCST90001805 | ebi-a-GCST90001805 | TRUE | T | 44779073 | 1.95E-15 | reported | 3657 | 0.0288 | rs56287761 |  |  |  |  |  |  |  |  |  |  |  |  |  |  |  |
| -0.2354 | 20 | igd | 0.2492 | G | \|\| id:ebi-a-GCST90001808 | ebi-a-GCST90001808 | TRUE | T | 44779073 | 3.57E-16 | reported | 3657 | 0.02875 | rs56287761 |  |  |  |  |  |  |  |  |  |  |  |  |  |  |  |
| -0.3257 | 6 | igd | 0.2492 | A | \|\| id:ebi-a-GCST90002111 | ebi-a-GCST90002111 | TRUE | G | 31573110 | 2.67E-11 | reported | 1635 | 0.04855 | rs9267517 |  |  |  |  |  |  |  |  |  |  |  |  |  |  |  |
| -0.3861 | 6 | igd | 0.2492 | A | \|\| id:ebi-a-GCST90002112 | ebi-a-GCST90002112 | TRUE | G | 31573110 | 5.21E-15 | reported | 1635 | 0.0489 | rs9267517 |  |  |  |  |  |  |  |  |  |  |  |  |  |  |  |
| -0.2076 | 10 | igd | 0.249 | G | \|\| id:ebi-a-GCST90001782 | ebi-a-GCST90001782 | TRUE | A | 6225119 | 3.86E-08 | reported | 1837 | 0.0376 | rs55768419 |  |  |  |  |  |  |  |  |  |  |  |  |  |  |  |
| -0.224 | 20 | igd | 0.249 | G | \|\| id:ebi-a-GCST90001800 | ebi-a-GCST90001800 | TRUE | T | 44779073 | 1.27E-14 | reported | 3656 | 0.02894 | rs56287761 |  |  |  |  |  |  |  |  |  |  |  |  |  |  |  |
| -0.2321 | 20 | igd | 0.249 | G | \|\| id:ebi-a-GCST90001806 | ebi-a-GCST90001806 | TRUE | T | 44779073 | 9.34E-16 | reported | 3656 | 0.02875 | rs56287761 |  |  |  |  |  |  |  |  |  |  |  |  |  |  |  |
| -0.0764 | 8 | igd | 0.2489 | G | \|\| id:ebi-a-GCST90011997 | ebi-a-GCST90011997 | TRUE | A | 106590706 | 1.36E-08 | reported | 21758 | 0.0135 | rs4602861 |  |  |  |  |  |  |  |  |  |  |  |  |  |  |  |
| -0.2881 | 6 | igd | 0.2487 | A | \|\| id:ebi-a-GCST90002109 | ebi-a-GCST90002109 | TRUE | G | 31573110 | 4.50E-09 | reported | 1578 | 0.04885 | rs9267517 |  |  |  |  |  |  |  |  |  |  |  |  |  |  |  |
| -0.3854 | 6 | igd | 0.2486 | A | \|\| id:ebi-a-GCST90002108 | ebi-a-GCST90002108 | TRUE | G | 31573110 | 9.21E-15 | reported | 1579 | 0.04925 | rs9267517 |  |  |  |  |  |  |  |  |  |  |  |  |  |  |  |
| -0.1748 | 10 | igd | 0.2485 | G | \|\| id:ebi-a-GCST90001775 | ebi-a-GCST90001775 | TRUE | A | 6225119 | 1.33E-09 | reported | 3660 | 0.02875 | rs55768419 |  |  |  |  |  |  |  |  |  |  |  |  |  |  |  |
| -0.3728 | 6 | igd | 0.2485 | A | \|\| id:ebi-a-GCST90002110 | ebi-a-GCST90002110 | TRUE | G | 32089274 | 3.33E-14 | reported | 1634 | 0.04871 | rs2284180 |  |  |  |  |  |  |  |  |  |  |  |  |  |  |  |
| -0.3097 | 6 | igd | 0.2484 | A | \|\| id:ebi-a-GCST90002109 | ebi-a-GCST90002109 | TRUE | G | 32089274 | 1.68E-10 | reported | 1578 | 0.04817 | rs2284180 |  |  |  |  |  |  |  |  |  |  |  |  |  |  |  |
| -0.217 | 10 | igd | 0.2483 | G | \|\| id:ebi-a-GCST90001777 | ebi-a-GCST90001777 | TRUE | A | 6225119 | 1.39E-14 | reported | 3657 | 0.02807 | rs55768419 |  |  |  |  |  |  |  |  |  |  |  |  |  |  |  |
| -0.1935 | 10 | igd | 0.2483 | G | \|\| id:ebi-a-GCST90001778 | ebi-a-GCST90001778 | TRUE | A | 6225119 | 9.67E-12 | reported | 3657 | 0.02832 | rs55768419 |  |  |  |  |  |  |  |  |  |  |  |  |  |  |  |
| -0.186 | 10 | igd | 0.2483 | G | \|\| id:ebi-a-GCST90001779 | ebi-a-GCST90001779 | TRUE | A | 6225119 | 9.84E-11 | reported | 3657 | 0.02866 | rs55768419 |  |  |  |  |  |  |  |  |  |  |  |  |  |  |  |
| -0.1891 | 10 | igd | 0.2483 | G | \|\| id:ebi-a-GCST90001784 | ebi-a-GCST90001784 | TRUE | A | 6225119 | 5.99E-11 | reported | 3657 | 0.0288 | rs55768419 |  |  |  |  |  |  |  |  |  |  |  |  |  |  |  |
| -0.2146 | 10 | igd | 0.2483 | G | \|\| id:ebi-a-GCST90001789 | ebi-a-GCST90001789 | TRUE | A | 6225119 | 2.00E-14 | reported | 3657 | 0.02793 | rs55768419 |  |  |  |  |  |  |  |  |  |  |  |  |  |  |  |
| -0.2046 | 10 | igd | 0.2483 | G | \|\| id:ebi-a-GCST90001790 | ebi-a-GCST90001790 | TRUE | A | 6225119 | 4.58E-13 | reported | 3657 | 0.02816 | rs55768419 |  |  |  |  |  |  |  |  |  |  |  |  |  |  |  |
| -0.1914 | 10 | igd | 0.2483 | G | \|\| id:ebi-a-GCST90001791 | ebi-a-GCST90001791 | TRUE | A | 6225119 | 3.90E-11 | reported | 3657 | 0.02888 | rs55768419 |  |  |  |  |  |  |  |  |  |  |  |  |  |  |  |
| -0.1947 | 10 | igd | 0.2483 | G | \|\| id:ebi-a-GCST90001792 | ebi-a-GCST90001792 | TRUE | A | 6225119 | 6.02E-12 | reported | 3657 | 0.02821 | rs55768419 |  |  |  |  |  |  |  |  |  |  |  |  |  |  |  |
| -0.2059 | 10 | igd | 0.2483 | G | \|\| id:ebi-a-GCST90001793 | ebi-a-GCST90001793 | TRUE | A | 6225119 | 4.65E-13 | reported | 3657 | 0.02836 | rs55768419 |  |  |  |  |  |  |  |  |  |  |  |  |  |  |  |
| -0.4268 | 6 | igd | 0.2483 | A | \|\| id:ebi-a-GCST90002108 | ebi-a-GCST90002108 | TRUE | G | 32089274 | 2.82E-18 | reported | 1579 | 0.04836 | rs2284180 |  |  |  |  |  |  |  |  |  |  |  |  |  |  |  |
| -0.3599 | 6 | igd | 0.2483 | A | \|\| id:ebi-a-GCST90002111 | ebi-a-GCST90002111 | TRUE | G | 32089274 | 8.28E-14 | reported | 1635 | 0.04779 | rs2284180 |  |  |  |  |  |  |  |  |  |  |  |  |  |  |  |
| -0.4113 | 6 | igd | 0.2483 | A | \|\| id:ebi-a-GCST90002112 | ebi-a-GCST90002112 | TRUE | G | 32089274 | 2.97E-17 | reported | 1635 | 0.04815 | rs2284180 |  |  |  |  |  |  |  |  |  |  |  |  |  |  |  |
| -0.1655 | 10 | igd | 0.2482 | G | \|\| id:ebi-a-GCST90001794 | ebi-a-GCST90001794 | TRUE | A | 6225119 | 9.59E-09 | reported | 3658 | 0.02878 | rs55768419 |  |  |  |  |  |  |  |  |  |  |  |  |  |  |  |
| 0.1856 | 19 | igd | 0.248 | C | \|\| id:ebi-a-GCST90001757 | ebi-a-GCST90001757 | TRUE | T | 49768180 | 5.03E-11 | reported | 3657 | 0.02816 | rs9304682 |  |  |  |  |  |  |  |  |  |  |  |  |  |  |  |
| 0.18 | 19 | igd | 0.248 | C | \|\| id:ebi-a-GCST90001761 | ebi-a-GCST90001761 | TRUE | T | 49768180 | 2.55E-10 | reported | 3657 | 0.02838 | rs9304682 |  |  |  |  |  |  |  |  |  |  |  |  |  |  |  |
| -0.2872 | 6 | igd | 0.2476 | C | \|\| id:ebi-a-GCST90002105 | ebi-a-GCST90002105 | TRUE | T | 31081205 | 9.11E-13 | reported | 2872 | 0.04002 | rs2233956 |  |  |  |  |  |  |  |  |  |  |  |  |  |  |  |
| -0.5168 | 2 | igd | 0.2476 | G | \|\| id:ebi-a-GCST90002119 | ebi-a-GCST90002119 | TRUE | A | 87009488 | 2.91E-65 | reported | 2920 | 0.02954 | rs938487 |  |  |  |  |  |  |  |  |  |  |  |  |  |  |  |
| 0.222 | 20 | igd | 0.2475 | C | \|\| id:ebi-a-GCST90001801 | ebi-a-GCST90001801 | TRUE | T | 44797562 | 6.44E-09 | reported | 1836 | 0.03806 | rs2425761 |  |  |  |  |  |  |  |  |  |  |  |  |  |  |  |
| 0.2616 | 20 | igd | 0.2472 | A | \|\| id:ebi-a-GCST90001804 | ebi-a-GCST90001804 | TRUE | G | 44800835 | 1.35E-20 | reported | 3657 | 0.02795 | rs2425765 |  |  |  |  |  |  |  |  |  |  |  |  |  |  |  |
| 0.2043 | 20 | igd | 0.2472 | A | \|\| id:ebi-a-GCST90001805 | ebi-a-GCST90001805 | TRUE | G | 44800835 | 8.79E-13 | reported | 3657 | 0.02848 | rs2425765 |  |  |  |  |  |  |  |  |  |  |  |  |  |  |  |
| 0.2495 | 20 | igd | 0.2472 | A | \|\| id:ebi-a-GCST90001808 | ebi-a-GCST90001808 | TRUE | G | 44800835 | 1.98E-18 | reported | 3657 | 0.02833 | rs2425765 |  |  |  |  |  |  |  |  |  |  |  |  |  |  |  |
| 0.2025 | 20 | igd | 0.2469 | A | \|\| id:ebi-a-GCST90001802 | ebi-a-GCST90001802 | TRUE | G | 44788633 | 1.53E-12 | reported | 3657 | 0.02854 | rs2425754 |  |  |  |  |  |  |  |  |  |  |  |  |  |  |  |
| 0.2023 | 10 | igd | 0.2467 | G | \|\| id:ebi-a-GCST90002029 | ebi-a-GCST90002029 | TRUE | T | 97982751 | 8.98E-11 | reported | 2915 | 0.03109 | rs4917725 |  |  |  |  |  |  |  |  |  |  |  |  |  |  |  |
| -0.2032 | 12 | igd | 0.2466 | A | \|\| id:ebi-a-GCST90002062 | ebi-a-GCST90002062 | TRUE | G | 6903631 | 1.71E-10 | reported | 2920 | 0.03171 | rs11615628 |  |  |  |  |  |  |  |  |  |  |  |  |  |  |  |
| -0.2062 | 12 | igd | 0.2466 | A | \|\| id:ebi-a-GCST90002067 | ebi-a-GCST90002067 | TRUE | G | 6903631 | 5.49E-11 | reported | 2920 | 0.03133 | rs11615628 |  |  |  |  |  |  |  |  |  |  |  |  |  |  |  |
| -0.3264 | 12 | igd | 0.2465 | A | \|\| id:ebi-a-GCST90002027 | ebi-a-GCST90002027 | TRUE | G | 6903631 | 6.46E-25 | reported | 2911 | 0.03137 | rs11615628 |  |  |  |  |  |  |  |  |  |  |  |  |  |  |  |
| 0.1716 | 2 | igd | 0.2464 | C | \|\| id:ebi-a-GCST90001665 | ebi-a-GCST90001665 | TRUE | T | 87009742 | 2.85E-09 | reported | 3440 | 0.02881 | rs2367376 |  |  |  |  |  |  |  |  |  |  |  |  |  |  |  |
| -0.5526 | 1 | igd | 0.2461 | G | \|\| id:ebi-a-GCST90001999 | ebi-a-GCST90001999 | TRUE | T | 161533055 | 3.82E-82 | reported | 3621 | 0.02805 | rs71632989 |  |  |  |  |  |  |  |  |  |  |  |  |  |  |  |
| -0.3617 | 6 | igd | 0.246 | A | \|\| id:ebi-a-GCST90002105 | ebi-a-GCST90002105 | TRUE | G | 31573110 | 1.28E-18 | reported | 2872 | 0.04079 | rs9267517 |  |  |  |  |  |  |  |  |  |  |  |  |  |  |  |
| -0.435 | 6 | igd | 0.246 | C | \|\| id:ebi-a-GCST90002105 | ebi-a-GCST90002105 | TRUE | T | 32290650 | 8.63E-28 | reported | 2872 | 0.0394 | rs2143603 |  |  |  |  |  |  |  |  |  |  |  |  |  |  |  |
| 0.1979 | 6 | igd | 0.2459 | G | \|\| id:ebi-a-GCST90001692 | ebi-a-GCST90001692 | TRUE | A | 32451888 | 5.75E-09 | reported | 3440 | 0.03389 | rs2395200 |  |  |  |  |  |  |  |  |  |  |  |  |  |  |  |
| -0.2868 | 10 | igd | 0.2457 | T | \|\| id:ebi-a-GCST90001780 | ebi-a-GCST90001780 | TRUE | C | 6113782 | 3.86E-24 | reported | 3642 | 0.02809 | rs1887027 |  |  |  |  |  |  |  |  |  |  |  |  |  |  |  |
| 0.3323 | 1 | igd | 0.2457 | G | \|\| id:ebi-a-GCST90002035 | ebi-a-GCST90002035 | TRUE | A | 161526397 | 1.06E-25 | reported | 2871 | 0.0314 | rs71632979 |  |  |  |  |  |  |  |  |  |  |  |  |  |  |  |
| -0.2405 | 6 | igd | 0.2455 | T | \|\| id:ebi-a-GCST90002118 | ebi-a-GCST90002118 | TRUE | C | 29750672 | 1.89E-10 | reported | 2920 | 0.03762 | rs1611250 |  |  |  |  |  |  |  |  |  |  |  |  |  |  |  |
| -0.2809 | 10 | igd | 0.2454 | T | \|\| id:ebi-a-GCST90001775 | ebi-a-GCST90001775 | TRUE | C | 6113782 | 8.87E-23 | reported | 3660 | 0.0284 | rs1887027 |  |  |  |  |  |  |  |  |  |  |  |  |  |  |  |
| -0.4244 | 10 | igd | 0.2454 | T | \|\| id:ebi-a-GCST90001777 | ebi-a-GCST90001777 | TRUE | C | 6113782 | 8.06E-53 | reported | 3657 | 0.0273 | rs1887027 |  |  |  |  |  |  |  |  |  |  |  |  |  |  |  |
| -0.3962 | 10 | igd | 0.2454 | T | \|\| id:ebi-a-GCST90001778 | ebi-a-GCST90001778 | TRUE | C | 6113782 | 1.71E-45 | reported | 3657 | 0.0276 | rs1887027 |  |  |  |  |  |  |  |  |  |  |  |  |  |  |  |
| -0.295 | 10 | igd | 0.2454 | T | \|\| id:ebi-a-GCST90001779 | ebi-a-GCST90001779 | TRUE | C | 6113782 | 3.46E-25 | reported | 3657 | 0.02824 | rs1887027 |  |  |  |  |  |  |  |  |  |  |  |  |  |  |  |
| -0.2827 | 10 | igd | 0.2454 | T | \|\| id:ebi-a-GCST90001784 | ebi-a-GCST90001784 | TRUE | C | 6113782 | 5.71E-23 | reported | 3657 | 0.02846 | rs1887027 |  |  |  |  |  |  |  |  |  |  |  |  |  |  |  |
| -0.362 | 10 | igd | 0.2454 | T | \|\| id:ebi-a-GCST90001789 | ebi-a-GCST90001789 | TRUE | C | 6113782 | 4.91E-39 | reported | 3657 | 0.02738 | rs1887027 |  |  |  |  |  |  |  |  |  |  |  |  |  |  |  |
| -0.4203 | 10 | igd | 0.2454 | T | \|\| id:ebi-a-GCST90001790 | ebi-a-GCST90001790 | TRUE | C | 6113782 | 1.16E-51 | reported | 3657 | 0.02736 | rs1887027 |  |  |  |  |  |  |  |  |  |  |  |  |  |  |  |
| -0.2878 | 10 | igd | 0.2454 | T | \|\| id:ebi-a-GCST90001791 | ebi-a-GCST90001791 | TRUE | C | 6113782 | 1.10E-23 | reported | 3657 | 0.02849 | rs1887027 |  |  |  |  |  |  |  |  |  |  |  |  |  |  |  |
| -0.4047 | 10 | igd | 0.2454 | T | \|\| id:ebi-a-GCST90001792 | ebi-a-GCST90001792 | TRUE | C | 6113782 | 7.76E-48 | reported | 3657 | 0.02745 | rs1887027 |  |  |  |  |  |  |  |  |  |  |  |  |  |  |  |
| -0.267 | 10 | igd | 0.2454 | T | \|\| id:ebi-a-GCST90001794 | ebi-a-GCST90001794 | TRUE | C | 6113782 | 1.07E-20 | reported | 3658 | 0.02845 | rs1887027 |  |  |  |  |  |  |  |  |  |  |  |  |  |  |  |
| -0.1669 | 10 | igd | 0.2454 | T | \|\| id:ebi-a-GCST90001795 | ebi-a-GCST90001795 | TRUE | C | 6113782 | 4.07E-09 | reported | 3657 | 0.02831 | rs1887027 |  |  |  |  |  |  |  |  |  |  |  |  |  |  |  |
| -0.1762 | 20 | igd | 0.2453 | A | \|\| id:ebi-a-GCST90001399 | ebi-a-GCST90001399 | TRUE | T | 44796921 | 9.51E-10 | reported | 3657 | 0.02872 | rs2425760 |  |  |  |  |  |  |  |  |  |  |  |  |  |  |  |
| 0.2025 | 22 | igd | 0.2451 | A | \|\| id:ebi-a-GCST90001702 | ebi-a-GCST90001702 | TRUE | G | 40359877 | 9.94E-12 | reported | 3657 | 0.02964 | rs141791242 |  |  |  |  |  |  |  |  |  |  |  |  |  |  |  |
| 0.1916 | 22 | igd | 0.2451 | A | \|\| id:ebi-a-GCST90001703 | ebi-a-GCST90001703 | TRUE | G | 40359877 | 1.17E-10 | reported | 3657 | 0.02966 | rs141791242 |  |  |  |  |  |  |  |  |  |  |  |  |  |  |  |
| 0.1979 | 22 | igd | 0.2451 | A | \|\| id:ebi-a-GCST90001710 | ebi-a-GCST90001710 | TRUE | G | 40359877 | 2.55E-11 | reported | 3657 | 0.02957 | rs141791242 |  |  |  |  |  |  |  |  |  |  |  |  |  |  |  |
| 0.1901 | 22 | igd | 0.2451 | A | \|\| id:ebi-a-GCST90001711 | ebi-a-GCST90001711 | TRUE | G | 40359877 | 1.32E-10 | reported | 3657 | 0.02951 | rs141791242 |  |  |  |  |  |  |  |  |  |  |  |  |  |  |  |
| -0.1851 | 7 | igd | 0.245 | T | \|\| id:ebi-a-GCST90002072 | ebi-a-GCST90002072 | TRUE | G | 50318308 | 1.20E-08 | reported | 2872 | 0.03237 | rs17552904 |  |  |  |  |  |  |  |  |  |  |  |  |  |  |  |
| -0.2391 | 6 | igd | 0.2448 | A | \|\| id:ebi-a-GCST90001820 | ebi-a-GCST90001820 | TRUE | G | 32281784 | 7.83E-12 | reported | 3656 | 0.03483 | rs9268211 |  |  |  |  |  |  |  |  |  |  |  |  |  |  |  |
| -0.1969 | 14 | igd | 0.2447 | A | \|\| id:ebi-a-GCST90001821 | ebi-a-GCST90001821 | TRUE | G | 105840287 | 6.05E-11 | reported | 3656 | 0.03 | rs12891236 |  |  |  |  |  |  |  |  |  |  |  |  |  |  |  |
| -0.2523 | 6 | igd | 0.2445 | A | \|\| id:ebi-a-GCST90001820 | ebi-a-GCST90001820 | TRUE | G | 31573110 | 2.37E-12 | reported | 3656 | 0.03586 | rs9267517 |  |  |  |  |  |  |  |  |  |  |  |  |  |  |  |
| -0.2419 | 6 | igd | 0.2444 | T | \|\| id:ebi-a-GCST90001820 | ebi-a-GCST90001820 | TRUE | C | 32395942 | 7.22E-12 | reported | 3656 | 0.03519 | rs9268577 |  |  |  |  |  |  |  |  |  |  |  |  |  |  |  |
| -0.4292 | 6 | igd | 0.2443 | A | \|\| id:ebi-a-GCST90002105 | ebi-a-GCST90002105 | TRUE | G | 32089274 | 1.65E-26 | reported | 2872 | 0.03988 | rs2284180 |  |  |  |  |  |  |  |  |  |  |  |  |  |  |  |
| -0.5887 | 1 | igd | 0.2441 | T | \|\| id:ebi-a-GCST90001474 | ebi-a-GCST90001474 | TRUE | C | 161520931 | 1.97E-92 | reported | 3410 | 0.02799 | rs56199187 |  |  |  |  |  |  |  |  |  |  |  |  |  |  |  |
| -0.211 | 6 | igd | 0.2441 | C | \|\| id:ebi-a-GCST90001820 | ebi-a-GCST90001820 | TRUE | T | 31081205 | 3.37E-09 | reported | 3656 | 0.0356 | rs2233956 |  |  |  |  |  |  |  |  |  |  |  |  |  |  |  |
| 0.3259 | 1 | igd | 0.2441 | T | \|\| id:ebi-a-GCST90001832 | ebi-a-GCST90001832 | TRUE | G | 169665551 | 4.42E-28 | reported | 2872 | 0.02935 | rs4987358 |  |  |  |  |  |  |  |  |  |  |  |  |  |  |  |
| 0.1708 | 10 | igd | 0.244 | C | \|\| id:ebi-a-GCST90001888 | ebi-a-GCST90001888 | TRUE | G | 97980648 | 3.93E-08 | reported | 2920 | 0.03101 | rs17111461 |  |  |  |  |  |  |  |  |  |  |  |  |  |  |  |
| -0.4537 | 10 | igd | 0.2439 | T | \|\| id:ebi-a-GCST90001782 | ebi-a-GCST90001782 | TRUE | C | 6114010 | 6.15E-33 | reported | 1837 | 0.03721 | rs10905716 |  |  |  |  |  |  |  |  |  |  |  |  |  |  |  |
| 0.3386 | 6 | igd | 0.2433 | T | \|\| id:ebi-a-GCST90001687 | ebi-a-GCST90001687 | TRUE | G | 32617889 | 2.15E-08 | reported | 3408 | 0.06032 | rs112984211 |  |  |  |  |  |  |  |  |  |  |  |  |  |  |  |
| -0.248 | 19 | igd | 0.2433 | G | \|\| id:ebi-a-GCST90001946 | ebi-a-GCST90001946 | TRUE | A | 53248249 | 1.68E-09 | reported | 1634 | 0.04091 | rs28669361 |  |  |  |  |  |  |  |  |  |  |  |  |  |  |  |
| -0.2358 | 2 | igd | 0.2431 | T | \|\| id:ebi-a-GCST90001900 | ebi-a-GCST90001900 | TRUE | C | 204842532 | 1.40E-13 | reported | 2919 | 0.03173 | rs62177554 |  |  |  |  |  |  |  |  |  |  |  |  |  |  |  |
| 0.1726 | 2 | igd | 0.243 | A | \|\| id:ebi-a-GCST90001664 | ebi-a-GCST90001664 | TRUE | G | 87058580 | 2.21E-10 | reported | 3440 | 0.02712 | rs4832054 |  |  |  |  |  |  |  |  |  |  |  |  |  |  |  |
| -0.2095 | 2 | igd | 0.243 | T | \|\| id:ebi-a-GCST90001891 | ebi-a-GCST90001891 | TRUE | C | 204842532 | 4.91E-11 | reported | 2920 | 0.03175 | rs62177554 |  |  |  |  |  |  |  |  |  |  |  |  |  |  |  |
| -0.1818 | 2 | igd | 0.243 | T | \|\| id:ebi-a-GCST90001898 | ebi-a-GCST90001898 | TRUE | C | 204842532 | 1.30E-08 | reported | 2920 | 0.03188 | rs62177554 |  |  |  |  |  |  |  |  |  |  |  |  |  |  |  |
| -0.2339 | 6 | igd | 0.243 | A | \|\| id:ebi-a-GCST90002105 | ebi-a-GCST90002105 | TRUE | C | 29921291 | 2.45E-08 | reported | 2872 | 0.04183 | rs139723994 |  |  |  |  |  |  |  |  |  |  |  |  |  |  |  |
| -0.2192 | 7 | igd | 0.2429 | A | \|\| id:ebi-a-GCST90001944 | ebi-a-GCST90001944 | TRUE | G | 50309890 | 4.68E-11 | reported | 2872 | 0.03318 | rs62447171 |  |  |  |  |  |  |  |  |  |  |  |  |  |  |  |
| -0.2184 | 7 | igd | 0.2429 | A | \|\| id:ebi-a-GCST90001945 | ebi-a-GCST90001945 | TRUE | G | 50309890 | 5.49E-11 | reported | 2872 | 0.03318 | rs62447171 |  |  |  |  |  |  |  |  |  |  |  |  |  |  |  |
| -0.2297 | 6 | igd | 0.2426 | C | \|\| id:ebi-a-GCST90001820 | ebi-a-GCST90001820 | TRUE | G | 32135878 | 7.62E-11 | reported | 3656 | 0.03519 | rs205000 |  |  |  |  |  |  |  |  |  |  |  |  |  |  |  |
| -0.2093 | 7 | igd | 0.2426 | A | \|\| id:ebi-a-GCST90002037 | ebi-a-GCST90002037 | TRUE | G | 50309890 | 2.84E-10 | reported | 2871 | 0.03307 | rs62447171 |  |  |  |  |  |  |  |  |  |  |  |  |  |  |  |
| -0.2089 | 7 | igd | 0.2426 | A | \|\| id:ebi-a-GCST90002038 | ebi-a-GCST90002038 | TRUE | G | 50309890 | 3.09E-10 | reported | 2871 | 0.03307 | rs62447171 |  |  |  |  |  |  |  |  |  |  |  |  |  |  |  |
| -0.283 | 19 | igd | 0.2425 | C | \|\| id:ebi-a-GCST90001745 | ebi-a-GCST90001745 | TRUE | T | 49872521 | 8.18E-23 | reported | 3657 | 0.0286 | rs55809481 |  |  |  |  |  |  |  |  |  |  |  |  |  |  |  |
| -0.2125 | 19 | igd | 0.2425 | C | \|\| id:ebi-a-GCST90001746 | ebi-a-GCST90001746 | TRUE | T | 49872521 | 1.61E-13 | reported | 3657 | 0.0287 | rs55809481 |  |  |  |  |  |  |  |  |  |  |  |  |  |  |  |
| -0.2424 | 19 | igd | 0.2425 | C | \|\| id:ebi-a-GCST90001755 | ebi-a-GCST90001755 | TRUE | T | 49872521 | 5.44E-17 | reported | 3657 | 0.02879 | rs55809481 |  |  |  |  |  |  |  |  |  |  |  |  |  |  |  |
| -0.3192 | 19 | igd | 0.2425 | C | \|\| id:ebi-a-GCST90001757 | ebi-a-GCST90001757 | TRUE | T | 49872521 | 9.01E-29 | reported | 3657 | 0.02843 | rs55809481 |  |  |  |  |  |  |  |  |  |  |  |  |  |  |  |
| -0.3215 | 19 | igd | 0.2425 | C | \|\| id:ebi-a-GCST90001761 | ebi-a-GCST90001761 | TRUE | T | 49872521 | 9.31E-29 | reported | 3657 | 0.02865 | rs55809481 |  |  |  |  |  |  |  |  |  |  |  |  |  |  |  |
| 0.3515 | 10 | igd | 0.2417 | G | \|\| id:ebi-a-GCST90002029 | ebi-a-GCST90002029 | TRUE | A | 97729647 | 1.48E-29 | reported | 2915 | 0.03079 | rs17382898 |  |  |  |  |  |  |  |  |  |  |  |  |  |  |  |
| 0.2386 | 10 | igd | 0.2414 | G | \|\| id:ebi-a-GCST90001892 | ebi-a-GCST90001892 | TRUE | A | 97729647 | 4.54E-14 | reported | 2920 | 0.03147 | rs17382898 |  |  |  |  |  |  |  |  |  |  |  |  |  |  |  |
| -0.3391 | 6 | igd | 0.2413 | T | \|\| id:ebi-a-GCST90002108 | ebi-a-GCST90002108 | TRUE | C | 31005464 | 1.19E-11 | reported | 1579 | 0.04964 | rs9262555 |  |  |  |  |  |  |  |  |  |  |  |  |  |  |  |
| -0.2695 | 6 | igd | 0.241 | T | \|\| id:ebi-a-GCST90002111 | ebi-a-GCST90002111 | TRUE | C | 30264933 | 1.82E-08 | reported | 1635 | 0.04764 | rs261956 |  |  |  |  |  |  |  |  |  |  |  |  |  |  |  |
| -0.3039 | 6 | igd | 0.241 | A | \|\| id:ebi-a-GCST90002112 | ebi-a-GCST90002112 | TRUE | G | 31230137 | 1.05E-09 | reported | 1635 | 0.0495 | rs9264419 |  |  |  |  |  |  |  |  |  |  |  |  |  |  |  |
| -0.3226 | 6 | igd | 0.241 | T | \|\| id:ebi-a-GCST90002112 | ebi-a-GCST90002112 | TRUE | C | 31005464 | 1.12E-10 | reported | 1635 | 0.0497 | rs9262555 |  |  |  |  |  |  |  |  |  |  |  |  |  |  |  |
| 0.1618 | 10 | igd | 0.2402 | T | \|\| id:ebi-a-GCST90001483 | ebi-a-GCST90001483 | TRUE | C | 95260506 | 3.74E-08 | reported | 3405 | 0.02934 | rs17108835 |  |  |  |  |  |  |  |  |  |  |  |  |  |  |  |
| 0.2783 | 19 | igd | 0.2402 | A | \|\| id:ebi-a-GCST90001954 | ebi-a-GCST90001954 | TRUE | T | 51525121 | 1.96E-10 | reported | 1555 | 0.04343 | rs4417638 |  |  |  |  |  |  |  |  |  |  |  |  |  |  |  |
| -0.5493 | 1 | igd | 0.2402 | G | \|\| id:ebi-a-GCST90001970 | ebi-a-GCST90001970 | TRUE | A | 161526397 | 3.58E-69 | reported | 2971 | 0.03044 | rs71632979 |  |  |  |  |  |  |  |  |  |  |  |  |  |  |  |
| -0.5652 | 1 | igd | 0.2402 | G | \|\| id:ebi-a-GCST90002077 | ebi-a-GCST90002077 | TRUE | A | 161526397 | 1.98E-74 | reported | 2971 | 0.0301 | rs71632979 |  |  |  |  |  |  |  |  |  |  |  |  |  |  |  |
| -0.1934 | 6 | igd | 0.2399 | A | \|\| id:ebi-a-GCST90001820 | ebi-a-GCST90001820 | TRUE | G | 29487535 | 6.09E-09 | reported | 3656 | 0.03319 | rs9468541 |  |  |  |  |  |  |  |  |  |  |  |  |  |  |  |
| 0.278 | 19 | igd | 0.2397 | A | \|\| id:ebi-a-GCST90001953 | ebi-a-GCST90001953 | TRUE | T | 51525121 | 2.01E-10 | reported | 1558 | 0.04341 | rs4417638 |  |  |  |  |  |  |  |  |  |  |  |  |  |  |  |
| 0.5549 | 10 | igd | 0.2396 | C | \|\| id:ebi-a-GCST90001489 | ebi-a-GCST90001489 | TRUE | A | 97715871 | 8.54E-80 | reported | 3405 | 0.02857 | rs10882698 |  |  |  |  |  |  |  |  |  |  |  |  |  |  |  |
| 0.1932 | 10 | igd | 0.2391 | G | \|\| id:ebi-a-GCST90001495 | ebi-a-GCST90001495 | TRUE | A | 95264878 | 2.47E-10 | reported | 3405 | 0.03044 | rs34081505 |  |  |  |  |  |  |  |  |  |  |  |  |  |  |  |
| 0.2962 | 6 | igd | 0.2391 | A | \|\| id:ebi-a-GCST90002108 | ebi-a-GCST90002108 | TRUE | G | 32191041 | 5.09E-11 | reported | 1579 | 0.04479 | rs434841 |  |  |  |  |  |  |  |  |  |  |  |  |  |  |  |
| 0.2708 | 19 | igd | 0.2387 | A | \|\| id:ebi-a-GCST90001947 | ebi-a-GCST90001947 | TRUE | T | 51525121 | 6.19E-10 | reported | 1577 | 0.0435 | rs4417638 |  |  |  |  |  |  |  |  |  |  |  |  |  |  |  |
| 0.2635 | 19 | igd | 0.2386 | A | \|\| id:ebi-a-GCST90001957 | ebi-a-GCST90001957 | TRUE | T | 51525121 | 1.73E-09 | reported | 1578 | 0.0435 | rs4417638 |  |  |  |  |  |  |  |  |  |  |  |  |  |  |  |
| 0.2603 | 19 | igd | 0.2384 | A | \|\| id:ebi-a-GCST90001952 | ebi-a-GCST90001952 | TRUE | T | 51525121 | 2.83E-09 | reported | 1569 | 0.04356 | rs4417638 |  |  |  |  |  |  |  |  |  |  |  |  |  |  |  |
| 0.2685 | 19 | igd | 0.2384 | A | \|\| id:ebi-a-GCST90001956 | ebi-a-GCST90001956 | TRUE | T | 51525121 | 7.97E-10 | reported | 1579 | 0.04343 | rs4417638 |  |  |  |  |  |  |  |  |  |  |  |  |  |  |  |
| 0.3699 | 6 | igd | 0.2381 | A | \|\| id:ebi-a-GCST90002110 | ebi-a-GCST90002110 | TRUE | G | 32191041 | 1.91E-16 | reported | 1634 | 0.04449 | rs434841 |  |  |  |  |  |  |  |  |  |  |  |  |  |  |  |
| 0.3349 | 6 | igd | 0.2379 | A | \|\| id:ebi-a-GCST90002111 | ebi-a-GCST90002111 | TRUE | G | 32191041 | 4.02E-14 | reported | 1635 | 0.04391 | rs434841 |  |  |  |  |  |  |  |  |  |  |  |  |  |  |  |
| 0.1943 | 6 | igd | 0.2376 | C | \|\| id:ebi-a-GCST90002121 | ebi-a-GCST90002121 | TRUE | T | 30565101 | 1.70E-08 | reported | 2915 | 0.03435 | rs1264429 |  |  |  |  |  |  |  |  |  |  |  |  |  |  |  |
| 0.2085 | 6 | igd | 0.2375 | C | \|\| id:ebi-a-GCST90002067 | ebi-a-GCST90002067 | TRUE | T | 30565101 | 1.37E-08 | reported | 2920 | 0.03663 | rs1264429 |  |  |  |  |  |  |  |  |  |  |  |  |  |  |  |
| -0.2761 | 1 | igd | 0.2371 | T | \|\| id:ebi-a-GCST90001963 | ebi-a-GCST90001963 | TRUE | C | 161515326 | 1.22E-17 | reported | 2872 | 0.03208 | rs55971447 |  |  |  |  |  |  |  |  |  |  |  |  |  |  |  |
| -0.4101 | 1 | igd | 0.2369 | T | \|\| id:ebi-a-GCST90002013 | ebi-a-GCST90002013 | TRUE | C | 161515326 | 2.59E-37 | reported | 2870 | 0.03167 | rs55971447 |  |  |  |  |  |  |  |  |  |  |  |  |  |  |  |
| 0.2537 | 6 | igd | 0.2368 | A | \|\| id:ebi-a-GCST90002105 | ebi-a-GCST90002105 | TRUE | G | 32191041 | 6.54E-13 | reported | 2872 | 0.03513 | rs434841 |  |  |  |  |  |  |  |  |  |  |  |  |  |  |  |
| 0.1855 | 1 | igd | 0.2366 | A | \|\| id:ebi-a-GCST90001924 | ebi-a-GCST90001924 | TRUE | G | 173848808 | 8.94E-09 | reported | 2920 | 0.03217 | rs28680958 |  |  |  |  |  |  |  |  |  |  |  |  |  |  |  |
| 0.1835 | 22 | igd | 0.2363 | C | \|\| id:ebi-a-GCST90001703 | ebi-a-GCST90001703 | TRUE | T | 39302783 | 8.37E-10 | reported | 3657 | 0.02982 | rs132526 |  |  |  |  |  |  |  |  |  |  |  |  |  |  |  |
| 0.1706 | 22 | igd | 0.2363 | C | \|\| id:ebi-a-GCST90001711 | ebi-a-GCST90001711 | TRUE | T | 39302783 | 9.91E-09 | reported | 3657 | 0.02969 | rs132526 |  |  |  |  |  |  |  |  |  |  |  |  |  |  |  |
| -0.2463 | 6 | igd | 0.2361 | G | \|\| id:ebi-a-GCST90001932 | ebi-a-GCST90001932 | TRUE | A | 32451730 | 1.81E-12 | reported | 2920 | 0.0348 | rs13195237 |  |  |  |  |  |  |  |  |  |  |  |  |  |  |  |
| 0.1838 | 2 | igd | 0.2353 | A | \|\| id:ebi-a-GCST90001911 | ebi-a-GCST90001911 | TRUE | G | 232446772 | 7.10E-09 | reported | 3113 | 0.03166 | rs12468411 |  |  |  |  |  |  |  |  |  |  |  |  |  |  |  |
| -0.3011 | 1 | igd | 0.2352 | T | \|\| id:ebi-a-GCST90001471 | ebi-a-GCST90001471 | TRUE | C | 161515326 | 6.70E-25 | reported | 3410 | 0.02899 | rs55971447 |  |  |  |  |  |  |  |  |  |  |  |  |  |  |  |
| -0.3662 | 6 | igd | 0.235 | T | \|\| id:ebi-a-GCST90002110 | ebi-a-GCST90002110 | TRUE | C | 32831661 | 1.66E-14 | reported | 1634 | 0.04728 | rs4959116 |  |  |  |  |  |  |  |  |  |  |  |  |  |  |  |
| -0.342 | 6 | igd | 0.2349 | T | \|\| id:ebi-a-GCST90002111 | ebi-a-GCST90002111 | TRUE | C | 32831661 | 3.55E-13 | reported | 1635 | 0.04664 | rs4959116 |  |  |  |  |  |  |  |  |  |  |  |  |  |  |  |
| -0.3203 | 22 | igd | 0.2346 | T | \|\| id:ebi-a-GCST90001702 | ebi-a-GCST90001702 | TRUE | A | 44297686 | 9.65E-28 | reported | 3657 | 0.0291 | rs9614288 |  |  |  |  |  |  |  |  |  |  |  |  |  |  |  |
| -0.3097 | 22 | igd | 0.2346 | T | \|\| id:ebi-a-GCST90001703 | ebi-a-GCST90001703 | TRUE | A | 44297686 | 5.05E-26 | reported | 3657 | 0.02913 | rs9614288 |  |  |  |  |  |  |  |  |  |  |  |  |  |  |  |
| -0.2871 | 22 | igd | 0.2346 | T | \|\| id:ebi-a-GCST90001710 | ebi-a-GCST90001710 | TRUE | A | 44297686 | 1.20E-22 | reported | 3657 | 0.02912 | rs9614288 |  |  |  |  |  |  |  |  |  |  |  |  |  |  |  |
| -0.2875 | 22 | igd | 0.2346 | T | \|\| id:ebi-a-GCST90001711 | ebi-a-GCST90001711 | TRUE | A | 44297686 | 8.00E-23 | reported | 3657 | 0.02904 | rs9614288 |  |  |  |  |  |  |  |  |  |  |  |  |  |  |  |
| -0.2739 | 1 | igd | 0.2345 | T | \|\| id:ebi-a-GCST90001467 | ebi-a-GCST90001467 | TRUE | C | 161507448 | 1.98E-20 | reported | 3410 | 0.02938 | rs3845548 |  |  |  |  |  |  |  |  |  |  |  |  |  |  |  |
| 0.2435 | 6 | igd | 0.2343 | T | \|\| id:ebi-a-GCST90002105 | ebi-a-GCST90002105 | TRUE | C | 31869673 | 9.17E-13 | reported | 2872 | 0.03394 | rs2844455 |  |  |  |  |  |  |  |  |  |  |  |  |  |  |  |
| 0.1834 | 7 | igd | 0.2342 | C | \|\| id:ebi-a-GCST90001927 | ebi-a-GCST90001927 | TRUE | T | 31163003 | 1.63E-09 | reported | 2920 | 0.03032 | rs7801342 |  |  |  |  |  |  |  |  |  |  |  |  |  |  |  |
| 0.2331 | 1 | igd | 0.2341 | T | \|\| id:ebi-a-GCST90001970 | ebi-a-GCST90001970 | TRUE | C | 161168004 | 6.28E-13 | reported | 2971 | 0.03226 | rs33941127 |  |  |  |  |  |  |  |  |  |  |  |  |  |  |  |
| -0.3826 | 6 | igd | 0.2337 | A | \|\| id:ebi-a-GCST90002108 | ebi-a-GCST90002108 | TRUE | G | 32851432 | 1.73E-15 | reported | 1579 | 0.04758 | rs2857219 |  |  |  |  |  |  |  |  |  |  |  |  |  |  |  |
| -0.3239 | 6 | igd | 0.2337 | T | \|\| id:ebi-a-GCST90002108 | ebi-a-GCST90002108 | TRUE | C | 31336994 | 1.85E-10 | reported | 1579 | 0.05048 | rs9266417 |  |  |  |  |  |  |  |  |  |  |  |  |  |  |  |
| -0.196 | 19 | igd | 0.2335 | C | \|\| id:ebi-a-GCST90001753 | ebi-a-GCST90001753 | TRUE | T | 49847916 | 3.03E-11 | reported | 3657 | 0.02941 | rs30006 |  |  |  |  |  |  |  |  |  |  |  |  |  |  |  |
| -0.1907 | 19 | igd | 0.2335 | C | \|\| id:ebi-a-GCST90001754 | ebi-a-GCST90001754 | TRUE | T | 49847916 | 8.55E-11 | reported | 3657 | 0.02929 | rs30006 |  |  |  |  |  |  |  |  |  |  |  |  |  |  |  |
| 0.2049 | 3 | igd | 0.2335 | A | \|\| id:ebi-a-GCST90001806 | ebi-a-GCST90001806 | TRUE | G | 32499535 | 2.55E-12 | reported | 3656 | 0.02917 | rs56284874 |  |  |  |  |  |  |  |  |  |  |  |  |  |  |  |
| -0.2743 | 6 | igd | 0.2335 | A | \|\| id:ebi-a-GCST90002109 | ebi-a-GCST90002109 | TRUE | G | 32851432 | 9.66E-09 | reported | 1578 | 0.04756 | rs2857219 |  |  |  |  |  |  |  |  |  |  |  |  |  |  |  |
| 0.3042 | 6 | igd | 0.2334 | T | \|\| id:ebi-a-GCST90002108 | ebi-a-GCST90002108 | TRUE | C | 31869673 | 1.73E-12 | reported | 1579 | 0.04278 | rs2844455 |  |  |  |  |  |  |  |  |  |  |  |  |  |  |  |
| 0.2344 | 6 | igd | 0.2332 | T | \|\| id:ebi-a-GCST90002109 | ebi-a-GCST90002109 | TRUE | C | 31869673 | 4.80E-08 | reported | 1578 | 0.04274 | rs2844455 |  |  |  |  |  |  |  |  |  |  |  |  |  |  |  |
| -0.2404 | 19 | igd | 0.2323 | T | \|\| id:ebi-a-GCST90001760 | ebi-a-GCST90001760 | TRUE | C | 49853881 | 2.09E-16 | reported | 3657 | 0.02912 | rs6509428 |  |  |  |  |  |  |  |  |  |  |  |  |  |  |  |
| 0.2873 | 19 | igd | 0.2321 | A | \|\| id:ebi-a-GCST90001948 | ebi-a-GCST90001948 | TRUE | T | 51525121 | 1.76E-11 | reported | 1633 | 0.04242 | rs4417638 |  |  |  |  |  |  |  |  |  |  |  |  |  |  |  |
| 0.3016 | 6 | igd | 0.2321 | T | \|\| id:ebi-a-GCST90002108 | ebi-a-GCST90002108 | TRUE | C | 33730095 | 1.36E-11 | reported | 1579 | 0.04427 | rs1887340 |  |  |  |  |  |  |  |  |  |  |  |  |  |  |  |
| 0.2383 | 10 | igd | 0.232 | C | \|\| id:ebi-a-GCST90001489 | ebi-a-GCST90001489 | TRUE | A | 97072727 | 8.10E-15 | reported | 3405 | 0.03055 | rs1053417 |  |  |  |  |  |  |  |  |  |  |  |  |  |  |  |
| 0.2779 | 10 | igd | 0.232 | C | \|\| id:ebi-a-GCST90001495 | ebi-a-GCST90001495 | TRUE | A | 97072727 | 2.18E-19 | reported | 3405 | 0.03068 | rs1053417 |  |  |  |  |  |  |  |  |  |  |  |  |  |  |  |
| 0.295 | 10 | igd | 0.232 | C | \|\| id:ebi-a-GCST90001660 | ebi-a-GCST90001660 | TRUE | A | 97072727 | 3.27E-22 | reported | 3408 | 0.03022 | rs1053417 |  |  |  |  |  |  |  |  |  |  |  |  |  |  |  |
| 0.2981 | 19 | igd | 0.2319 | A | \|\| id:ebi-a-GCST90001946 | ebi-a-GCST90001946 | TRUE | T | 51525121 | 3.57E-12 | reported | 1634 | 0.04254 | rs4417638 |  |  |  |  |  |  |  |  |  |  |  |  |  |  |  |
| 0.2704 | 19 | igd | 0.2319 | A | \|\| id:ebi-a-GCST90001949 | ebi-a-GCST90001949 | TRUE | T | 51525121 | 2.34E-10 | reported | 1634 | 0.0424 | rs4417638 |  |  |  |  |  |  |  |  |  |  |  |  |  |  |  |
| -0.27 | 6 | igd | 0.2319 | T | \|\| id:ebi-a-GCST90002105 | ebi-a-GCST90002105 | TRUE | C | 32452587 | 2.18E-12 | reported | 2872 | 0.03827 | rs28368843 |  |  |  |  |  |  |  |  |  |  |  |  |  |  |  |
| 0.1948 | 1 | igd | 0.2318 | T | \|\| id:ebi-a-GCST90001474 | ebi-a-GCST90001474 | TRUE | C | 161168004 | 1.14E-10 | reported | 3410 | 0.03012 | rs33941127 |  |  |  |  |  |  |  |  |  |  |  |  |  |  |  |
| -0.2188 | 2 | igd | 0.2317 | G | \|\| id:ebi-a-GCST90002120 | ebi-a-GCST90002120 | TRUE | A | 87056480 | 1.18E-11 | reported | 2920 | 0.03213 | rs7425430 |  |  |  |  |  |  |  |  |  |  |  |  |  |  |  |
| 0.197 | 22 | igd | 0.2316 | T | \|\| id:ebi-a-GCST90001702 | ebi-a-GCST90001702 | TRUE | G | 39437591 | 4.13E-11 | reported | 3657 | 0.02975 | rs9611084 |  |  |  |  |  |  |  |  |  |  |  |  |  |  |  |
| 0.1996 | 22 | igd | 0.2316 | T | \|\| id:ebi-a-GCST90001703 | ebi-a-GCST90001703 | TRUE | G | 39437591 | 2.25E-11 | reported | 3657 | 0.02974 | rs9611084 |  |  |  |  |  |  |  |  |  |  |  |  |  |  |  |
| 0.2658 | 6 | igd | 0.2315 | T | \|\| id:ebi-a-GCST90002112 | ebi-a-GCST90002112 | TRUE | C | 31869673 | 6.17E-10 | reported | 1635 | 0.04271 | rs2844455 |  |  |  |  |  |  |  |  |  |  |  |  |  |  |  |
| -0.2725 | 1 | igd | 0.2313 | G | \|\| id:ebi-a-GCST90002002 | ebi-a-GCST90002002 | TRUE | C | 161492175 | 1.53E-20 | reported | 3629 | 0.02916 | rs9427401 |  |  |  |  |  |  |  |  |  |  |  |  |  |  |  |
| 0.2583 | 10 | igd | 0.2311 | C | \|\| id:ebi-a-GCST90001658 | ebi-a-GCST90001658 | TRUE | A | 97072727 | 5.81E-19 | reported | 3440 | 0.02886 | rs1053417 |  |  |  |  |  |  |  |  |  |  |  |  |  |  |  |
| -0.1809 | 1 | igd | 0.231 | T | \|\| id:ebi-a-GCST90001452 | ebi-a-GCST90001452 | TRUE | C | 161515326 | 4.95E-09 | reported | 3017 | 0.03084 | rs55971447 |  |  |  |  |  |  |  |  |  |  |  |  |  |  |  |
| 0.223 | 2 | igd | 0.231 | A | \|\| id:ebi-a-GCST90001561 | ebi-a-GCST90001561 | TRUE | G | 38901086 | 4.24E-18 | reported | 3427 | 0.02557 | rs7600227 |  |  |  |  |  |  |  |  |  |  |  |  |  |  |  |
| 0.2016 | 1 | igd | 0.2308 | G | \|\| id:ebi-a-GCST90002071 | ebi-a-GCST90002071 | TRUE | A | 161563554 | 1.68E-09 | reported | 2872 | 0.03334 | rs139529340 |  |  |  |  |  |  |  |  |  |  |  |  |  |  |  |
| 0.1787 | 22 | igd | 0.2307 | G | \|\| id:ebi-a-GCST90001702 | ebi-a-GCST90001702 | TRUE | A | 41884954 | 2.29E-09 | reported | 3657 | 0.02982 | rs738140 |  |  |  |  |  |  |  |  |  |  |  |  |  |  |  |
| 0.1681 | 22 | igd | 0.2307 | G | \|\| id:ebi-a-GCST90001703 | ebi-a-GCST90001703 | TRUE | A | 41884954 | 1.87E-08 | reported | 3657 | 0.02983 | rs738140 |  |  |  |  |  |  |  |  |  |  |  |  |  |  |  |
| 0.1918 | 22 | igd | 0.2307 | G | \|\| id:ebi-a-GCST90001704 | ebi-a-GCST90001704 | TRUE | A | 41884954 | 1.29E-10 | reported | 3657 | 0.02975 | rs738140 |  |  |  |  |  |  |  |  |  |  |  |  |  |  |  |
| 0.1916 | 22 | igd | 0.2307 | G | \|\| id:ebi-a-GCST90001710 | ebi-a-GCST90001710 | TRUE | A | 41884954 | 1.30E-10 | reported | 3657 | 0.02972 | rs738140 |  |  |  |  |  |  |  |  |  |  |  |  |  |  |  |
| 0.1821 | 22 | igd | 0.2307 | G | \|\| id:ebi-a-GCST90001711 | ebi-a-GCST90001711 | TRUE | A | 41884954 | 9.17E-10 | reported | 3657 | 0.02966 | rs738140 |  |  |  |  |  |  |  |  |  |  |  |  |  |  |  |
| 0.1652 | 2 | igd | 0.2306 | G | \|\| id:ebi-a-GCST90001560 | ebi-a-GCST90001560 | TRUE | A | 38902624 | 2.13E-10 | reported | 3395 | 0.02593 | rs67145759 |  |  |  |  |  |  |  |  |  |  |  |  |  |  |  |
| -0.2717 | 19 | igd | 0.2304 | C | \|\| id:ebi-a-GCST90001758 | ebi-a-GCST90001758 | TRUE | T | 49846398 | 1.71E-20 | reported | 3657 | 0.02911 | rs30003 |  |  |  |  |  |  |  |  |  |  |  |  |  |  |  |
| -0.1667 | 19 | igd | 0.2304 | C | \|\| id:ebi-a-GCST90001759 | ebi-a-GCST90001759 | TRUE | T | 49846398 | 1.67E-08 | reported | 3657 | 0.02948 | rs30003 |  |  |  |  |  |  |  |  |  |  |  |  |  |  |  |
| -0.1948 | 19 | igd | 0.2303 | C | \|\| id:ebi-a-GCST90001748 | ebi-a-GCST90001748 | TRUE | T | 49846398 | 3.22E-11 | reported | 3645 | 0.02926 | rs30003 |  |  |  |  |  |  |  |  |  |  |  |  |  |  |  |
| -0.1786 | 19 | igd | 0.2303 | C | \|\| id:ebi-a-GCST90001762 | ebi-a-GCST90001762 | TRUE | T | 49846398 | 1.42E-09 | reported | 3658 | 0.02943 | rs30003 |  |  |  |  |  |  |  |  |  |  |  |  |  |  |  |
| 0.1825 | 8 | igd | 0.2303 | G | \|\| id:ebi-a-GCST90001926 | ebi-a-GCST90001926 | TRUE | C | 6891971 | 4.90E-08 | reported | 2920 | 0.03337 | rs4433170 |  |  |  |  |  |  |  |  |  |  |  |  |  |  |  |
| -0.2128 | 6 | igd | 0.2301 | C | \|\| id:ebi-a-GCST90002067 | ebi-a-GCST90002067 | TRUE | A | 31292582 | 1.44E-08 | reported | 2920 | 0.03743 | rs9405064 |  |  |  |  |  |  |  |  |  |  |  |  |  |  |  |
| -0.2448 | 10 | igd | 0.23 | T | \|\| id:ebi-a-GCST90001961 | ebi-a-GCST90001961 | TRUE | C | 97069849 | 9.19E-14 | reported | 2920 | 0.03269 | rs17110604 |  |  |  |  |  |  |  |  |  |  |  |  |  |  |  |
| 0.2972 | 6 | igd | 0.23 | C | \|\| id:ebi-a-GCST90002109 | ebi-a-GCST90002109 | TRUE | A | 31292582 | 3.89E-09 | reported | 1578 | 0.05018 | rs9405064 |  |  |  |  |  |  |  |  |  |  |  |  |  |  |  |
| -0.3828 | 6 | igd | 0.2298 | T | \|\| id:ebi-a-GCST90002105 | ebi-a-GCST90002105 | TRUE | G | 32811629 | 1.81E-23 | reported | 2872 | 0.03801 | rs2071543 |  |  |  |  |  |  |  |  |  |  |  |  |  |  |  |
| 0.1867 | 10 | igd | 0.2295 | A | \|\| id:ebi-a-GCST90001672 | ebi-a-GCST90001672 | TRUE | G | 99696325 | 2.39E-09 | reported | 3408 | 0.0312 | rs57933561 |  |  |  |  |  |  |  |  |  |  |  |  |  |  |  |
| -0.1646 | 22 | igd | 0.2289 | T | \|\| id:ebi-a-GCST90001702 | ebi-a-GCST90001702 | TRUE | C | 39343188 | 3.96E-08 | reported | 3657 | 0.02991 | rs7289804 |  |  |  |  |  |  |  |  |  |  |  |  |  |  |  |
| -0.1654 | 22 | igd | 0.2289 | T | \|\| id:ebi-a-GCST90001703 | ebi-a-GCST90001703 | TRUE | C | 39343188 | 3.38E-08 | reported | 3657 | 0.0299 | rs7289804 |  |  |  |  |  |  |  |  |  |  |  |  |  |  |  |
| -0.2695 | 6 | igd | 0.2289 | G | \|\| id:ebi-a-GCST90002105 | ebi-a-GCST90002105 | TRUE | A | 31349809 | 3.30E-10 | reported | 2872 | 0.04274 | rs9266720 |  |  |  |  |  |  |  |  |  |  |  |  |  |  |  |
| -0.2249 | 20 | igd | 0.2287 | G | \|\| id:ebi-a-GCST90001981 | ebi-a-GCST90001981 | TRUE | A | 44812788 | 2.76E-14 | reported | 3618 | 0.02944 | rs1977114 |  |  |  |  |  |  |  |  |  |  |  |  |  |  |  |
| -0.3668 | 6 | igd | 0.2284 | A | \|\| id:ebi-a-GCST90002112 | ebi-a-GCST90002112 | TRUE | G | 32809848 | 3.37E-14 | reported | 1635 | 0.04794 | rs9357155 |  |  |  |  |  |  |  |  |  |  |  |  |  |  |  |
| -0.3319 | 10 | igd | 0.2283 | G | \|\| id:ebi-a-GCST90001485 | ebi-a-GCST90001485 | TRUE | A | 97335609 | 8.38E-30 | reported | 3437 | 0.02899 | rs6584009 |  |  |  |  |  |  |  |  |  |  |  |  |  |  |  |
| 0.2884 | 6 | igd | 0.228 | T | \|\| id:ebi-a-GCST90002108 | ebi-a-GCST90002108 | TRUE | C | 32853277 | 4.78E-10 | reported | 1579 | 0.04602 | rs7767732 |  |  |  |  |  |  |  |  |  |  |  |  |  |  |  |
| -0.2551 | 2 | igd | 0.228 | C | \|\| id:ebi-a-GCST90002121 | ebi-a-GCST90002121 | TRUE | T | 87055981 | 1.80E-15 | reported | 2915 | 0.03189 | rs72847706 |  |  |  |  |  |  |  |  |  |  |  |  |  |  |  |
| 0.2738 | 6 | igd | 0.2277 | T | \|\| id:ebi-a-GCST90002110 | ebi-a-GCST90002110 | TRUE | C | 32853277 | 3.02E-09 | reported | 1634 | 0.04591 | rs7767732 |  |  |  |  |  |  |  |  |  |  |  |  |  |  |  |
| -0.1773 | 6 | igd | 0.2275 | G | \|\| id:ebi-a-GCST90001773 | ebi-a-GCST90001773 | TRUE | A | 107297924 | 2.42E-09 | reported | 3652 | 0.02964 | rs11153030 |  |  |  |  |  |  |  |  |  |  |  |  |  |  |  |
| -0.326 | 6 | igd | 0.2272 | T | \|\| id:ebi-a-GCST90002111 | ebi-a-GCST90002111 | TRUE | G | 31386094 | 1.99E-10 | reported | 1635 | 0.05091 | rs9266852 |  |  |  |  |  |  |  |  |  |  |  |  |  |  |  |
| -0.3811 | 6 | igd | 0.2271 | G | \|\| id:ebi-a-GCST90002110 | ebi-a-GCST90002110 | TRUE | A | 32700917 | 6.85E-14 | reported | 1634 | 0.05043 | rs12664812 |  |  |  |  |  |  |  |  |  |  |  |  |  |  |  |
| 0.1771 | 8 | igd | 0.227 | G | \|\| id:ebi-a-GCST90002078 | ebi-a-GCST90002078 | TRUE | A | 6888565 | 4.77E-08 | reported | 3112 | 0.03236 | rs55740316 |  |  |  |  |  |  |  |  |  |  |  |  |  |  |  |
| -0.3161 | 6 | igd | 0.2269 | A | \|\| id:ebi-a-GCST90002109 | ebi-a-GCST90002109 | TRUE | G | 32733390 | 2.64E-10 | reported | 1578 | 0.0497 | rs17840184 |  |  |  |  |  |  |  |  |  |  |  |  |  |  |  |
| -0.3633 | 6 | igd | 0.2269 | G | \|\| id:ebi-a-GCST90002111 | ebi-a-GCST90002111 | TRUE | A | 32700917 | 3.68E-13 | reported | 1635 | 0.04958 | rs12664812 |  |  |  |  |  |  |  |  |  |  |  |  |  |  |  |
| -0.3883 | 6 | igd | 0.2269 | G | \|\| id:ebi-a-GCST90002112 | ebi-a-GCST90002112 | TRUE | A | 32700917 | 1.56E-14 | reported | 1635 | 0.05008 | rs12664812 |  |  |  |  |  |  |  |  |  |  |  |  |  |  |  |
| 0.2599 | 6 | igd | 0.2268 | T | \|\| id:ebi-a-GCST90002105 | ebi-a-GCST90002105 | TRUE | C | 32853277 | 4.37E-13 | reported | 2872 | 0.03571 | rs7767732 |  |  |  |  |  |  |  |  |  |  |  |  |  |  |  |
| 0.1754 | 1 | igd | 0.2267 | T | \|\| id:ebi-a-GCST90001474 | ebi-a-GCST90001474 | TRUE | A | 161444369 | 7.50E-09 | reported | 3410 | 0.03027 | rs12120358 |  |  |  |  |  |  |  |  |  |  |  |  |  |  |  |
| -0.4373 | 6 | igd | 0.2267 | A | \|\| id:ebi-a-GCST90002108 | ebi-a-GCST90002108 | TRUE | G | 32733390 | 4.02E-18 | reported | 1579 | 0.04978 | rs17840184 |  |  |  |  |  |  |  |  |  |  |  |  |  |  |  |
| -0.2766 | 10 | igd | 0.2265 | T | \|\| id:ebi-a-GCST90001484 | ebi-a-GCST90001484 | TRUE | C | 97469109 | 1.07E-19 | reported | 3437 | 0.03028 | rs7073937 |  |  |  |  |  |  |  |  |  |  |  |  |  |  |  |
| 0.2877 | 16 | igd | 0.2263 | C | \|\| id:ebi-a-GCST90001918 | ebi-a-GCST90001918 | TRUE | G | 10974423 | 4.55E-20 | reported | 3060 | 0.03115 | rs12924236 |  |  |  |  |  |  |  |  |  |  |  |  |  |  |  |
| 0.2199 | 16 | igd | 0.2263 | C | \|\| id:ebi-a-GCST90002083 | ebi-a-GCST90002083 | TRUE | G | 10974423 | 2.36E-12 | reported | 3060 | 0.03124 | rs12924236 |  |  |  |  |  |  |  |  |  |  |  |  |  |  |  |
| -0.2193 | 6 | igd | 0.2262 | G | \|\| id:ebi-a-GCST90001820 | ebi-a-GCST90001820 | TRUE | A | 31349809 | 5.33E-09 | reported | 3656 | 0.03748 | rs9266720 |  |  |  |  |  |  |  |  |  |  |  |  |  |  |  |
| -0.2226 | 10 | igd | 0.2261 | G | \|\| id:ebi-a-GCST90001660 | ebi-a-GCST90001660 | TRUE | C | 98801814 | 6.53E-13 | reported | 3408 | 0.03084 | rs59838893 |  |  |  |  |  |  |  |  |  |  |  |  |  |  |  |
| -0.2421 | 10 | igd | 0.2261 | G | \|\| id:ebi-a-GCST90001672 | ebi-a-GCST90001672 | TRUE | C | 98801814 | 9.23E-15 | reported | 3408 | 0.0311 | rs59838893 |  |  |  |  |  |  |  |  |  |  |  |  |  |  |  |
| -0.2237 | 8 | igd | 0.2261 | G | \|\| id:ebi-a-GCST90002078 | ebi-a-GCST90002078 | TRUE | A | 6880925 | 1.96E-12 | reported | 3112 | 0.03167 | rs7826487 |  |  |  |  |  |  |  |  |  |  |  |  |  |  |  |
| 0.1851 | 10 | igd | 0.2257 | T | \|\| id:ebi-a-GCST90002031 | ebi-a-GCST90002031 | TRUE | C | 105527521 | 3.91E-08 | reported | 2920 | 0.0336 | rs12778775 |  |  |  |  |  |  |  |  |  |  |  |  |  |  |  |
| 0.2289 | 6 | igd | 0.2255 | T | \|\| id:ebi-a-GCST90002105 | ebi-a-GCST90002105 | TRUE | C | 33730095 | 2.66E-11 | reported | 2872 | 0.03421 | rs1887340 |  |  |  |  |  |  |  |  |  |  |  |  |  |  |  |
| -0.293 | 6 | igd | 0.2252 | T | \|\| id:ebi-a-GCST90002110 | ebi-a-GCST90002110 | TRUE | C | 31231988 | 2.36E-08 | reported | 1634 | 0.05222 | rs9264475 |  |  |  |  |  |  |  |  |  |  |  |  |  |  |  |
| -0.3221 | 6 | igd | 0.2251 | T | \|\| id:ebi-a-GCST90002111 | ebi-a-GCST90002111 | TRUE | C | 31231988 | 3.41E-10 | reported | 1635 | 0.05098 | rs9264475 |  |  |  |  |  |  |  |  |  |  |  |  |  |  |  |
| -0.2033 | 10 | igd | 0.225 | G | \|\| id:ebi-a-GCST90001658 | ebi-a-GCST90001658 | TRUE | C | 98801814 | 5.94E-12 | reported | 3440 | 0.02944 | rs59838893 |  |  |  |  |  |  |  |  |  |  |  |  |  |  |  |
| -0.2287 | 10 | igd | 0.225 | G | \|\| id:ebi-a-GCST90001659 | ebi-a-GCST90001659 | TRUE | C | 98801814 | 9.47E-15 | reported | 3440 | 0.02939 | rs59838893 |  |  |  |  |  |  |  |  |  |  |  |  |  |  |  |
| -0.2419 | 10 | igd | 0.225 | G | \|\| id:ebi-a-GCST90001670 | ebi-a-GCST90001670 | TRUE | C | 98801814 | 3.42E-15 | reported | 3440 | 0.03058 | rs59838893 |  |  |  |  |  |  |  |  |  |  |  |  |  |  |  |
| -0.2345 | 10 | igd | 0.225 | G | \|\| id:ebi-a-GCST90001671 | ebi-a-GCST90001671 | TRUE | C | 98801814 | 2.55E-14 | reported | 3440 | 0.03064 | rs59838893 |  |  |  |  |  |  |  |  |  |  |  |  |  |  |  |
| -0.32 | 6 | igd | 0.2248 | C | \|\| id:ebi-a-GCST90002111 | ebi-a-GCST90002111 | TRUE | T | 31081672 | 3.52E-10 | reported | 1635 | 0.05069 | rs9263634 |  |  |  |  |  |  |  |  |  |  |  |  |  |  |  |
| -0.2791 | 6 | igd | 0.2247 | G | \|\| id:ebi-a-GCST90001932 | ebi-a-GCST90001932 | TRUE | A | 32636433 | 4.36E-13 | reported | 2920 | 0.03835 | rs35800511 |  |  |  |  |  |  |  |  |  |  |  |  |  |  |  |
| 0.2711 | 10 | igd | 0.2247 | T | \|\| id:ebi-a-GCST90001961 | ebi-a-GCST90001961 | TRUE | C | 97469109 | 1.69E-15 | reported | 2920 | 0.03385 | rs7073937 |  |  |  |  |  |  |  |  |  |  |  |  |  |  |  |
| -0.2043 | 10 | igd | 0.2247 | T | \|\| id:ebi-a-GCST90002033 | ebi-a-GCST90002033 | TRUE | C | 97469109 | 2.45E-09 | reported | 2920 | 0.03414 | rs7073937 |  |  |  |  |  |  |  |  |  |  |  |  |  |  |  |
| 0.1934 | 1 | igd | 0.2246 | G | \|\| id:ebi-a-GCST90001832 | ebi-a-GCST90001832 | TRUE | C | 169968420 | 6.27E-10 | reported | 2872 | 0.03118 | rs541896 |  |  |  |  |  |  |  |  |  |  |  |  |  |  |  |
| -0.3517 | 6 | igd | 0.2245 | T | \|\| id:ebi-a-GCST90002108 | ebi-a-GCST90002108 | TRUE | C | 31231988 | 1.38E-11 | reported | 1579 | 0.05164 | rs9264475 |  |  |  |  |  |  |  |  |  |  |  |  |  |  |  |
| 0.3118 | 19 | igd | 0.2244 | T | \|\| id:ebi-a-GCST90001951 | ebi-a-GCST90001951 | TRUE | C | 51699811 | 2.29E-11 | reported | 1464 | 0.04627 | rs10419722 |  |  |  |  |  |  |  |  |  |  |  |  |  |  |  |
| 0.2004 | 10 | igd | 0.2238 | A | \|\| id:ebi-a-GCST90002032 | ebi-a-GCST90002032 | TRUE | G | 99558738 | 3.26E-09 | reported | 2920 | 0.03376 | rs10509714 |  |  |  |  |  |  |  |  |  |  |  |  |  |  |  |
| -0.3128 | 19 | igd | 0.2236 | A | \|\| id:ebi-a-GCST90001519 | ebi-a-GCST90001519 | TRUE | G | 51731176 | 2.63E-15 | reported | 1858 | 0.03922 | rs7245846 |  |  |  |  |  |  |  |  |  |  |  |  |  |  |  |
| -0.1847 | 19 | igd | 0.2234 | G | \|\| id:ebi-a-GCST90001752 | ebi-a-GCST90001752 | TRUE | C | 49896752 | 6.85E-10 | reported | 3657 | 0.02986 | rs11083972 |  |  |  |  |  |  |  |  |  |  |  |  |  |  |  |
| 0.18 | 10 | igd | 0.2233 | A | \|\| id:ebi-a-GCST90001489 | ebi-a-GCST90001489 | TRUE | G | 98382554 | 3.04E-09 | reported | 3405 | 0.03028 | rs12571884 |  |  |  |  |  |  |  |  |  |  |  |  |  |  |  |
| 0.2064 | 10 | igd | 0.2233 | A | \|\| id:ebi-a-GCST90001495 | ebi-a-GCST90001495 | TRUE | G | 98382554 | 1.55E-11 | reported | 3405 | 0.03051 | rs12571884 |  |  |  |  |  |  |  |  |  |  |  |  |  |  |  |
| 0.197 | 6 | igd | 0.2233 | A | \|\| id:ebi-a-GCST90001752 | ebi-a-GCST90001752 | TRUE | G | 32198527 | 1.20E-09 | reported | 3657 | 0.03232 | rs6901175 |  |  |  |  |  |  |  |  |  |  |  |  |  |  |  |
| -0.2047 | 6 | igd | 0.2232 | G | \|\| id:ebi-a-GCST90001820 | ebi-a-GCST90001820 | TRUE | A | 32861920 | 2.29E-09 | reported | 3656 | 0.03417 | rs241409 |  |  |  |  |  |  |  |  |  |  |  |  |  |  |  |
| -0.224 | 6 | igd | 0.2229 | A | \|\| id:ebi-a-GCST90001687 | ebi-a-GCST90001687 | TRUE | G | 32636477 | 5.18E-09 | reported | 3408 | 0.03825 | rs35571040 |  |  |  |  |  |  |  |  |  |  |  |  |  |  |  |
| 0.2022 | 10 | igd | 0.2228 | A | \|\| id:ebi-a-GCST90002030 | ebi-a-GCST90002030 | TRUE | G | 95401292 | 9.14E-10 | reported | 2920 | 0.03291 | rs1018634 |  |  |  |  |  |  |  |  |  |  |  |  |  |  |  |
| 0.2216 | 1 | igd | 0.2228 | T | \|\| id:ebi-a-GCST90002013 | ebi-a-GCST90002013 | TRUE | A | 161444369 | 5.64E-11 | reported | 2870 | 0.03369 | rs12120358 |  |  |  |  |  |  |  |  |  |  |  |  |  |  |  |
| 0.1902 | 10 | igd | 0.2226 | A | \|\| id:ebi-a-GCST90001496 | ebi-a-GCST90001496 | TRUE | G | 98382554 | 3.57E-10 | reported | 3437 | 0.03023 | rs12571884 |  |  |  |  |  |  |  |  |  |  |  |  |  |  |  |
| -22.53 | 6 | igd | 0.2226 | A | \|\| id:ebi-a-GCST90001695 | ebi-a-GCST90001695 | TRUE | G | 32636477 | 1.51E-08 | reported | 3408 | 3.971 | rs35571040 |  |  |  |  |  |  |  |  |  |  |  |  |  |  |  |
| -2.666 | 6 | igd | 0.2226 | A | \|\| id:ebi-a-GCST90001696 | ebi-a-GCST90001696 | TRUE | G | 32636477 | 6.83E-09 | reported | 3425 | 0.4589 | rs35571040 |  |  |  |  |  |  |  |  |  |  |  |  |  |  |  |
| -0.3081 | 6 | igd | 0.2226 | T | \|\| id:ebi-a-GCST90002108 | ebi-a-GCST90002108 | TRUE | C | 30830667 | 2.51E-09 | reported | 1579 | 0.0514 | rs9262257 |  |  |  |  |  |  |  |  |  |  |  |  |  |  |  |
| -0.1864 | 6 | igd | 0.2224 | A | \|\| id:ebi-a-GCST90001686 | ebi-a-GCST90001686 | TRUE | G | 32636477 | 4.40E-08 | reported | 3440 | 0.03397 | rs35571040 |  |  |  |  |  |  |  |  |  |  |  |  |  |  |  |
| 0.2374 | 6 | igd | 0.2224 | A | \|\| id:ebi-a-GCST90001692 | ebi-a-GCST90001692 | TRUE | G | 32636282 | 4.67E-10 | reported | 3440 | 0.038 | rs36058358 |  |  |  |  |  |  |  |  |  |  |  |  |  |  |  |
| -0.1875 | 6 | igd | 0.2224 | C | \|\| id:ebi-a-GCST90001920 | ebi-a-GCST90001920 | TRUE | G | 33083197 | 3.96E-08 | reported | 3060 | 0.03406 | rs6928350 |  |  |  |  |  |  |  |  |  |  |  |  |  |  |  |
| 0.2591 | 3 | igd | 0.2222 | G | \|\| id:ebi-a-GCST90001801 | ebi-a-GCST90001801 | TRUE | A | 32830171 | 3.64E-10 | reported | 1836 | 0.04111 | rs12488435 |  |  |  |  |  |  |  |  |  |  |  |  |  |  |  |
| -0.2272 | 3 | igd | 0.2221 | G | \|\| id:ebi-a-GCST90001997 | ebi-a-GCST90001997 | TRUE | C | 38869361 | 6.14E-14 | reported | 3590 | 0.03015 | rs6799972 |  |  |  |  |  |  |  |  |  |  |  |  |  |  |  |
| -0.2214 | 3 | igd | 0.2221 | G | \|\| id:ebi-a-GCST90001995 | ebi-a-GCST90001995 | TRUE | C | 38869361 | 1.74E-13 | reported | 3590 | 0.02993 | rs6799972 |  |  |  |  |  |  |  |  |  |  |  |  |  |  |  |
| -0.1657 | 9 | igd | 0.2216 | T | \|\| id:ebi-a-GCST90001787 | ebi-a-GCST90001787 | TRUE | C | 4327960 | 2.25E-08 | reported | 3657 | 0.02956 | rs12342539 |  |  |  |  |  |  |  |  |  |  |  |  |  |  |  |
| -0.2802 | 22 | igd | 0.2215 | C | \|\| id:ebi-a-GCST90001702 | ebi-a-GCST90001702 | TRUE | G | 39863326 | 1.16E-20 | reported | 3657 | 0.02988 | rs5750840 |  |  |  |  |  |  |  |  |  |  |  |  |  |  |  |
| -0.27 | 22 | igd | 0.2215 | C | \|\| id:ebi-a-GCST90001703 | ebi-a-GCST90001703 | TRUE | G | 39863326 | 2.77E-19 | reported | 3657 | 0.0299 | rs5750840 |  |  |  |  |  |  |  |  |  |  |  |  |  |  |  |
| -0.2668 | 22 | igd | 0.2215 | C | \|\| id:ebi-a-GCST90001704 | ebi-a-GCST90001704 | TRUE | G | 39863326 | 6.52E-19 | reported | 3657 | 0.02987 | rs5750840 |  |  |  |  |  |  |  |  |  |  |  |  |  |  |  |
| -0.2771 | 22 | igd | 0.2215 | C | \|\| id:ebi-a-GCST90001710 | ebi-a-GCST90001710 | TRUE | G | 39863326 | 2.40E-20 | reported | 3657 | 0.02981 | rs5750840 |  |  |  |  |  |  |  |  |  |  |  |  |  |  |  |
| -0.2786 | 22 | igd | 0.2215 | C | \|\| id:ebi-a-GCST90001711 | ebi-a-GCST90001711 | TRUE | G | 39863326 | 1.22E-20 | reported | 3657 | 0.02973 | rs5750840 |  |  |  |  |  |  |  |  |  |  |  |  |  |  |  |
| 0.225 | 1 | igd | 0.2212 | A | \|\| id:ebi-a-GCST90001845 | ebi-a-GCST90001845 | TRUE | G | 167538127 | 1.14E-11 | reported | 2911 | 0.03301 | rs116628565 |  |  |  |  |  |  |  |  |  |  |  |  |  |  |  |
| -0.4308 | 6 | igd | 0.2211 | G | \|\| id:ebi-a-GCST90002105 | ebi-a-GCST90002105 | TRUE | A | 32700917 | 7.91E-26 | reported | 2872 | 0.0406 | rs12664812 |  |  |  |  |  |  |  |  |  |  |  |  |  |  |  |
| 0.361 | 15 | igd | 0.2209 | T | \|\| id:ebi-a-GCST90001526 | ebi-a-GCST90001526 | TRUE | C | 80263217 | 7.44E-19 | reported | 1858 | 0.04027 | rs3826007 |  |  |  |  |  |  |  |  |  |  |  |  |  |  |  |
| -0.3414 | 15 | igd | 0.2209 | A | \|\| id:ebi-a-GCST90001527 | ebi-a-GCST90001527 | TRUE | G | 80260872 | 2.20E-19 | reported | 1858 | 0.0375 | rs67760360 |  |  |  |  |  |  |  |  |  |  |  |  |  |  |  |
| -0.3541 | 15 | igd | 0.2209 | T | \|\| id:ebi-a-GCST90001528 | ebi-a-GCST90001528 | TRUE | C | 80263217 | 3.71E-18 | reported | 1858 | 0.04034 | rs3826007 |  |  |  |  |  |  |  |  |  |  |  |  |  |  |  |
| -0.1799 | 2 | igd | 0.2209 | T | \|\| id:ebi-a-GCST90002119 | ebi-a-GCST90002119 | TRUE | A | 87741458 | 3.74E-08 | reported | 2920 | 0.03261 | rs76542489 |  |  |  |  |  |  |  |  |  |  |  |  |  |  |  |
| -0.3339 | 1 | igd | 0.2209 | A | \|\| id:ebi-a-GCST90002013 | ebi-a-GCST90002013 | TRUE | G | 161390516 | 5.23E-24 | reported | 2870 | 0.03274 | rs9427039 |  |  |  |  |  |  |  |  |  |  |  |  |  |  |  |
| 0.252 | 1 | igd | 0.2208 | A | \|\| id:ebi-a-GCST90001858 | ebi-a-GCST90001858 | TRUE | G | 167538127 | 2.52E-14 | reported | 2919 | 0.0329 | rs116628565 |  |  |  |  |  |  |  |  |  |  |  |  |  |  |  |
| 0.2006 | 1 | igd | 0.2208 | A | \|\| id:ebi-a-GCST90001860 | ebi-a-GCST90001860 | TRUE | G | 167538127 | 1.68E-09 | reported | 2919 | 0.03319 | rs116628565 |  |  |  |  |  |  |  |  |  |  |  |  |  |  |  |
| 0.2233 | 1 | igd | 0.2208 | A | \|\| id:ebi-a-GCST90001861 | ebi-a-GCST90001861 | TRUE | G | 167538127 | 9.32E-12 | reported | 2919 | 0.03263 | rs116628565 |  |  |  |  |  |  |  |  |  |  |  |  |  |  |  |
| 0.1872 | 1 | igd | 0.2208 | A | \|\| id:ebi-a-GCST90001863 | ebi-a-GCST90001863 | TRUE | G | 167538127 | 9.31E-09 | reported | 2919 | 0.0325 | rs116628565 |  |  |  |  |  |  |  |  |  |  |  |  |  |  |  |
| 0.2662 | 1 | igd | 0.2208 | A | \|\| id:ebi-a-GCST90001864 | ebi-a-GCST90001864 | TRUE | G | 167538127 | 7.63E-16 | reported | 2919 | 0.03284 | rs116628565 |  |  |  |  |  |  |  |  |  |  |  |  |  |  |  |
| 0.1897 | 1 | igd | 0.2208 | A | \|\| id:ebi-a-GCST90001866 | ebi-a-GCST90001866 | TRUE | G | 167538127 | 6.62E-09 | reported | 2914 | 0.03261 | rs116628565 |  |  |  |  |  |  |  |  |  |  |  |  |  |  |  |
| 0.1982 | 10 | igd | 0.2205 | A | \|\| id:ebi-a-GCST90002030 | ebi-a-GCST90002030 | TRUE | G | 98382554 | 8.60E-10 | reported | 2920 | 0.03221 | rs12571884 |  |  |  |  |  |  |  |  |  |  |  |  |  |  |  |
| -0.213 | 22 | igd | 0.2204 | A | \|\| id:ebi-a-GCST90001702 | ebi-a-GCST90001702 | TRUE | G | 40000442 | 1.79E-12 | reported | 3657 | 0.0301 | rs5757746 |  |  |  |  |  |  |  |  |  |  |  |  |  |  |  |
| -0.2094 | 22 | igd | 0.2204 | A | \|\| id:ebi-a-GCST90001703 | ebi-a-GCST90001703 | TRUE | G | 40000442 | 4.15E-12 | reported | 3657 | 0.0301 | rs5757746 |  |  |  |  |  |  |  |  |  |  |  |  |  |  |  |
| -0.2057 | 22 | igd | 0.2204 | A | \|\| id:ebi-a-GCST90001704 | ebi-a-GCST90001704 | TRUE | G | 40000442 | 9.21E-12 | reported | 3657 | 0.03007 | rs5757746 |  |  |  |  |  |  |  |  |  |  |  |  |  |  |  |
| -0.1989 | 22 | igd | 0.2204 | A | \|\| id:ebi-a-GCST90001710 | ebi-a-GCST90001710 | TRUE | G | 40000442 | 4.12E-11 | reported | 3657 | 0.03004 | rs5757746 |  |  |  |  |  |  |  |  |  |  |  |  |  |  |  |
| -0.1933 | 22 | igd | 0.2204 | A | \|\| id:ebi-a-GCST90001711 | ebi-a-GCST90001711 | TRUE | G | 40000442 | 1.30E-10 | reported | 3657 | 0.02998 | rs5757746 |  |  |  |  |  |  |  |  |  |  |  |  |  |  |  |
| 0.3002 | 19 | igd | 0.2203 | T | \|\| id:ebi-a-GCST90001954 | ebi-a-GCST90001954 | TRUE | C | 51498131 | 9.89E-12 | reported | 1555 | 0.04375 | rs11667107 |  |  |  |  |  |  |  |  |  |  |  |  |  |  |  |
| -0.2245 | 6 | igd | 0.2199 | G | \|\| id:ebi-a-GCST90001820 | ebi-a-GCST90001820 | TRUE | A | 31450153 | 2.47E-09 | reported | 3656 | 0.03755 | rs2904774 |  |  |  |  |  |  |  |  |  |  |  |  |  |  |  |
| 0.298 | 19 | igd | 0.2198 | T | \|\| id:ebi-a-GCST90001953 | ebi-a-GCST90001953 | TRUE | C | 51498131 | 1.37E-11 | reported | 1558 | 0.04374 | rs11667107 |  |  |  |  |  |  |  |  |  |  |  |  |  |  |  |
| -0.2905 | 6 | igd | 0.2197 | T | \|\| id:ebi-a-GCST90002105 | ebi-a-GCST90002105 | TRUE | C | 31231988 | 3.85E-11 | reported | 2872 | 0.04379 | rs9264475 |  |  |  |  |  |  |  |  |  |  |  |  |  |  |  |
| -0.2105 | 10 | igd | 0.2195 | C | \|\| id:ebi-a-GCST90001892 | ebi-a-GCST90001892 | TRUE | A | 98205222 | 4.12E-10 | reported | 2920 | 0.03356 | rs11188757 |  |  |  |  |  |  |  |  |  |  |  |  |  |  |  |
| 0.2117 | 10 | igd | 0.2195 | C | \|\| id:ebi-a-GCST90001961 | ebi-a-GCST90001961 | TRUE | A | 98205222 | 4.65E-10 | reported | 2920 | 0.03387 | rs11188757 |  |  |  |  |  |  |  |  |  |  |  |  |  |  |  |
| -0.2758 | 6 | igd | 0.2194 | C | \|\| id:ebi-a-GCST90002105 | ebi-a-GCST90002105 | TRUE | T | 31081672 | 2.64E-10 | reported | 2872 | 0.0435 | rs9263634 |  |  |  |  |  |  |  |  |  |  |  |  |  |  |  |
| -0.3589 | 16 | igd | 0.2194 | T | \|\| id:ebi-a-GCST90002110 | ebi-a-GCST90002110 | TRUE | C | 10979440 | 1.36E-17 | reported | 1634 | 0.04157 | rs11074934 |  |  |  |  |  |  |  |  |  |  |  |  |  |  |  |
| -0.4126 | 16 | igd | 0.2193 | T | \|\| id:ebi-a-GCST90002111 | ebi-a-GCST90002111 | TRUE | C | 10979440 | 2.14E-23 | reported | 1635 | 0.04076 | rs11074934 |  |  |  |  |  |  |  |  |  |  |  |  |  |  |  |
| 22.81 | 6 | igd | 0.2188 | A | \|\| id:ebi-a-GCST90001695 | ebi-a-GCST90001695 | TRUE | G | 31116210 | 1.74E-10 | reported | 3408 | 3.563 | rs130071 |  |  |  |  |  |  |  |  |  |  |  |  |  |  |  |
| 0.3006 | 19 | igd | 0.2188 | T | \|\| id:ebi-a-GCST90001955 | ebi-a-GCST90001955 | TRUE | C | 51503285 | 1.15E-11 | reported | 1554 | 0.04396 | rs16988799 |  |  |  |  |  |  |  |  |  |  |  |  |  |  |  |
| 2.545 | 6 | igd | 0.2185 | A | \|\| id:ebi-a-GCST90001696 | ebi-a-GCST90001696 | TRUE | G | 31116210 | 7.93E-10 | reported | 3425 | 0.4129 | rs130071 |  |  |  |  |  |  |  |  |  |  |  |  |  |  |  |
| 1.086 | 6 | igd | 0.2185 | A | \|\| id:ebi-a-GCST90001697 | ebi-a-GCST90001697 | TRUE | G | 31116210 | 1.37E-10 | reported | 3425 | 0.1686 | rs130071 |  |  |  |  |  |  |  |  |  |  |  |  |  |  |  |
| -0.2444 | 6 | igd | 0.2181 | G | \|\| id:ebi-a-GCST90001820 | ebi-a-GCST90001820 | TRUE | A | 32700917 | 1.54E-11 | reported | 3656 | 0.03613 | rs12664812 |  |  |  |  |  |  |  |  |  |  |  |  |  |  |  |
| 0.2817 | 19 | igd | 0.218 | T | \|\| id:ebi-a-GCST90001952 | ebi-a-GCST90001952 | TRUE | C | 51498131 | 2.07E-10 | reported | 1569 | 0.04403 | rs11667107 |  |  |  |  |  |  |  |  |  |  |  |  |  |  |  |
| 0.2273 | 6 | igd | 0.2178 | G | \|\| id:ebi-a-GCST90002105 | ebi-a-GCST90002105 | TRUE | C | 31381637 | 4.04E-10 | reported | 2872 | 0.03623 | rs2256328 |  |  |  |  |  |  |  |  |  |  |  |  |  |  |  |
| -0.2252 | 18 | igd | 0.2177 | T | \|\| id:ebi-a-GCST90001520 | ebi-a-GCST90001520 | TRUE | C | 5858361 | 1.45E-08 | reported | 1858 | 0.03955 | rs74253876 |  |  |  |  |  |  |  |  |  |  |  |  |  |  |  |
| 0.2814 | 19 | igd | 0.2175 | T | \|\| id:ebi-a-GCST90001947 | ebi-a-GCST90001947 | TRUE | C | 51503285 | 2.33E-10 | reported | 1577 | 0.04411 | rs16988799 |  |  |  |  |  |  |  |  |  |  |  |  |  |  |  |
| 0.2842 | 19 | igd | 0.2174 | T | \|\| id:ebi-a-GCST90001957 | ebi-a-GCST90001957 | TRUE | C | 51503285 | 1.58E-10 | reported | 1578 | 0.04413 | rs16988799 |  |  |  |  |  |  |  |  |  |  |  |  |  |  |  |
| -0.140049 | 1 | igd | 0.217391 | G | \|\| id:met-b-162 | met-b-162 | TRUE | A | 161700679 | 4.58E-08 | reported | 460 | 0.025617 | rs1503813 |  |  |  |  |  |  |  |  |  |  |  |  |  |  |  |
| 0.1769 | 4 | igd | 0.2173 | C | \|\| id:ebi-a-GCST90001815 | ebi-a-GCST90001815 | TRUE | T | 15786324 | 1.50E-09 | reported | 3657 | 0.02919 | rs7663941 |  |  |  |  |  |  |  |  |  |  |  |  |  |  |  |
| -0.1807 | 10 | igd | 0.2172 | G | \|\| id:ebi-a-GCST90001491 | ebi-a-GCST90001491 | TRUE | A | 96760622 | 1.56E-08 | reported | 3437 | 0.03187 | rs1113644 |  |  |  |  |  |  |  |  |  |  |  |  |  |  |  |
| 0.2865 | 19 | igd | 0.2172 | T | \|\| id:ebi-a-GCST90001956 | ebi-a-GCST90001956 | TRUE | C | 51503285 | 9.83E-11 | reported | 1579 | 0.04399 | rs16988799 |  |  |  |  |  |  |  |  |  |  |  |  |  |  |  |
| -0.2444 | 6 | igd | 0.2171 | C | \|\| id:ebi-a-GCST90002119 | ebi-a-GCST90002119 | TRUE | A | 31236250 | 1.07E-11 | reported | 2920 | 0.03581 | rs9394047 |  |  |  |  |  |  |  |  |  |  |  |  |  |  |  |
| 0.206 | 3 | igd | 0.217 | G | \|\| id:ebi-a-GCST90001798 | ebi-a-GCST90001798 | TRUE | A | 32830171 | 9.80E-12 | reported | 3657 | 0.03016 | rs12488435 |  |  |  |  |  |  |  |  |  |  |  |  |  |  |  |
| 0.1892 | 3 | igd | 0.217 | G | \|\| id:ebi-a-GCST90001800 | ebi-a-GCST90001800 | TRUE | A | 32830171 | 7.06E-10 | reported | 3656 | 0.03061 | rs12488435 |  |  |  |  |  |  |  |  |  |  |  |  |  |  |  |
| 0.1765 | 3 | igd | 0.217 | G | \|\| id:ebi-a-GCST90001804 | ebi-a-GCST90001804 | TRUE | A | 32830171 | 4.88E-09 | reported | 3657 | 0.0301 | rs12488435 |  |  |  |  |  |  |  |  |  |  |  |  |  |  |  |
| 0.1984 | 3 | igd | 0.217 | G | \|\| id:ebi-a-GCST90001808 | ebi-a-GCST90001808 | TRUE | A | 32830171 | 7.83E-11 | reported | 3657 | 0.03042 | rs12488435 |  |  |  |  |  |  |  |  |  |  |  |  |  |  |  |
| -0.1901 | 12 | igd | 0.2164 | A | \|\| id:ebi-a-GCST90002074 | ebi-a-GCST90002074 | TRUE | C | 69818080 | 3.40E-09 | reported | 3112 | 0.03207 | rs12367916 |  |  |  |  |  |  |  |  |  |  |  |  |  |  |  |
| 0.3189 | 6 | igd | 0.2164 | A | \|\| id:ebi-a-GCST90002105 | ebi-a-GCST90002105 | TRUE | G | 32795706 | 1.06E-18 | reported | 2872 | 0.03587 | rs55781756 |  |  |  |  |  |  |  |  |  |  |  |  |  |  |  |
| -0.281 | 6 | igd | 0.2162 | C | \|\| id:ebi-a-GCST90002111 | ebi-a-GCST90002111 | TRUE | T | 29914686 | 1.85E-08 | reported | 1635 | 0.0497 | rs6901707 |  |  |  |  |  |  |  |  |  |  |  |  |  |  |  |
| -0.2096 | 6 | igd | 0.2161 | A | \|\| id:ebi-a-GCST90002105 | ebi-a-GCST90002105 | TRUE | G | 32421272 | 1.41E-08 | reported | 2872 | 0.03684 | rs3129896 |  |  |  |  |  |  |  |  |  |  |  |  |  |  |  |
| -0.239 | 12 | igd | 0.2158 | C | \|\| id:ebi-a-GCST90002073 | ebi-a-GCST90002073 | TRUE | T | 69831984 | 1.01E-12 | reported | 2850 | 0.03337 | rs11177671 |  |  |  |  |  |  |  |  |  |  |  |  |  |  |  |
| -0.1837 | 12 | igd | 0.2158 | C | \|\| id:ebi-a-GCST90002074 | ebi-a-GCST90002074 | TRUE | T | 69852009 | 1.78E-08 | reported | 3112 | 0.03253 | rs61927226 |  |  |  |  |  |  |  |  |  |  |  |  |  |  |  |
| -0.2393 | 12 | igd | 0.2156 | A | \|\| id:ebi-a-GCST90002073 | ebi-a-GCST90002073 | TRUE | G | 69818346 | 9.30E-13 | reported | 2850 | 0.03336 | rs11177661 |  |  |  |  |  |  |  |  |  |  |  |  |  |  |  |
| -0.1865 | 22 | igd | 0.2155 | G | \|\| id:ebi-a-GCST90001704 | ebi-a-GCST90001704 | TRUE | A | 40262482 | 6.86E-10 | reported | 3657 | 0.03015 | rs6001697 |  |  |  |  |  |  |  |  |  |  |  |  |  |  |  |
| -0.2386 | 6 | igd | 0.2154 | C | \|\| id:ebi-a-GCST90001820 | ebi-a-GCST90001820 | TRUE | T | 31081672 | 4.24E-10 | reported | 3656 | 0.0381 | rs9263634 |  |  |  |  |  |  |  |  |  |  |  |  |  |  |  |
| 0.2052 | 10 | igd | 0.2153 | T | \|\| id:ebi-a-GCST90001495 | ebi-a-GCST90001495 | TRUE | C | 97087522 | 1.35E-10 | reported | 3405 | 0.03186 | rs7085635 |  |  |  |  |  |  |  |  |  |  |  |  |  |  |  |
| -0.2762 | 6 | igd | 0.2153 | T | \|\| id:ebi-a-GCST90002111 | ebi-a-GCST90002111 | TRUE | C | 28205826 | 1.21E-08 | reported | 1635 | 0.04822 | rs112421400 |  |  |  |  |  |  |  |  |  |  |  |  |  |  |  |
| 0.2379 | 10 | igd | 0.2151 | T | \|\| id:ebi-a-GCST90001660 | ebi-a-GCST90001660 | TRUE | C | 97087522 | 4.36E-14 | reported | 3408 | 0.03138 | rs7085635 |  |  |  |  |  |  |  |  |  |  |  |  |  |  |  |
| -0.2143 | 20 | igd | 0.2151 | A | \|\| id:ebi-a-GCST90001802 | ebi-a-GCST90001802 | TRUE | T | 44729408 | 3.88E-12 | reported | 3657 | 0.03077 | rs6032659 |  |  |  |  |  |  |  |  |  |  |  |  |  |  |  |
| -0.2974 | 20 | igd | 0.2151 | A | \|\| id:ebi-a-GCST90001808 | ebi-a-GCST90001808 | TRUE | T | 44729408 | 3.96E-22 | reported | 3657 | 0.03055 | rs6032659 |  |  |  |  |  |  |  |  |  |  |  |  |  |  |  |
| -0.4747 | 19 | igd | 0.215 | C | \|\| id:ebi-a-GCST90001531 | ebi-a-GCST90001531 | TRUE | G | 51736383 | 3.74E-33 | reported | 1856 | 0.0388 | rs33978622 |  |  |  |  |  |  |  |  |  |  |  |  |  |  |  |
| 0.1916 | 10 | igd | 0.215 | T | \|\| id:ebi-a-GCST90001658 | ebi-a-GCST90001658 | TRUE | C | 97087522 | 1.89E-10 | reported | 3440 | 0.02999 | rs7085635 |  |  |  |  |  |  |  |  |  |  |  |  |  |  |  |
| 0.2207 | 10 | igd | 0.2148 | A | \|\| id:ebi-a-GCST90002029 | ebi-a-GCST90002029 | TRUE | G | 97990028 | 2.78E-11 | reported | 2915 | 0.03303 | rs61856986 |  |  |  |  |  |  |  |  |  |  |  |  |  |  |  |
| 0.223 | 10 | igd | 0.2147 | A | \|\| id:ebi-a-GCST90001892 | ebi-a-GCST90001892 | TRUE | G | 97990028 | 2.94E-11 | reported | 2920 | 0.0334 | rs61856986 |  |  |  |  |  |  |  |  |  |  |  |  |  |  |  |
| -0.2332 | 10 | igd | 0.2147 | A | \|\| id:ebi-a-GCST90001961 | ebi-a-GCST90001961 | TRUE | G | 97990028 | 5.59E-12 | reported | 2920 | 0.03371 | rs61856986 |  |  |  |  |  |  |  |  |  |  |  |  |  |  |  |
| 0.3939 | 10 | igd | 0.2147 | A | \|\| id:ebi-a-GCST90002030 | ebi-a-GCST90002030 | TRUE | G | 97990028 | 3.22E-32 | reported | 2920 | 0.03294 | rs61856986 |  |  |  |  |  |  |  |  |  |  |  |  |  |  |  |
| 0.3079 | 10 | igd | 0.2147 | A | \|\| id:ebi-a-GCST90002031 | ebi-a-GCST90002031 | TRUE | G | 97990028 | 1.98E-19 | reported | 2920 | 0.03392 | rs61856986 |  |  |  |  |  |  |  |  |  |  |  |  |  |  |  |
| 0.3356 | 10 | igd | 0.2147 | A | \|\| id:ebi-a-GCST90002032 | ebi-a-GCST90002032 | TRUE | G | 97990028 | 7.76E-23 | reported | 2920 | 0.03383 | rs61856986 |  |  |  |  |  |  |  |  |  |  |  |  |  |  |  |
| 0.196 | 10 | igd | 0.2147 | A | \|\| id:ebi-a-GCST90002061 | ebi-a-GCST90002061 | TRUE | G | 97990028 | 8.95E-09 | reported | 2920 | 0.03399 | rs61856986 |  |  |  |  |  |  |  |  |  |  |  |  |  |  |  |
| 0.3043 | 10 | igd | 0.2145 | A | \|\| id:ebi-a-GCST90001658 | ebi-a-GCST90001658 | TRUE | G | 97990028 | 8.39E-24 | reported | 3440 | 0.03003 | rs61856986 |  |  |  |  |  |  |  |  |  |  |  |  |  |  |  |
| 0.296 | 10 | igd | 0.2145 | A | \|\| id:ebi-a-GCST90001659 | ebi-a-GCST90001659 | TRUE | G | 97990028 | 1.38E-22 | reported | 3440 | 0.03006 | rs61856986 |  |  |  |  |  |  |  |  |  |  |  |  |  |  |  |
| 0.3203 | 10 | igd | 0.2145 | A | \|\| id:ebi-a-GCST90001660 | ebi-a-GCST90001660 | TRUE | G | 97990028 | 5.11E-24 | reported | 3408 | 0.03144 | rs61856986 |  |  |  |  |  |  |  |  |  |  |  |  |  |  |  |
| 0.3408 | 10 | igd | 0.2145 | A | \|\| id:ebi-a-GCST90001670 | ebi-a-GCST90001670 | TRUE | G | 97990028 | 2.12E-27 | reported | 3440 | 0.03116 | rs61856986 |  |  |  |  |  |  |  |  |  |  |  |  |  |  |  |
| 0.3365 | 10 | igd | 0.2145 | A | \|\| id:ebi-a-GCST90001671 | ebi-a-GCST90001671 | TRUE | G | 97990028 | 1.27E-26 | reported | 3440 | 0.03124 | rs61856986 |  |  |  |  |  |  |  |  |  |  |  |  |  |  |  |
| 0.3567 | 10 | igd | 0.2145 | A | \|\| id:ebi-a-GCST90001672 | ebi-a-GCST90001672 | TRUE | G | 97990028 | 5.95E-29 | reported | 3408 | 0.03165 | rs61856986 |  |  |  |  |  |  |  |  |  |  |  |  |  |  |  |
| 0.1973 | 10 | igd | 0.2144 | A | \|\| id:ebi-a-GCST90001483 | ebi-a-GCST90001483 | TRUE | G | 97990028 | 1.91E-10 | reported | 3405 | 0.03089 | rs61856986 |  |  |  |  |  |  |  |  |  |  |  |  |  |  |  |
| 0.2092 | 10 | igd | 0.2144 | A | \|\| id:ebi-a-GCST90001484 | ebi-a-GCST90001484 | TRUE | G | 97990028 | 9.97E-12 | reported | 3437 | 0.03063 | rs61856986 |  |  |  |  |  |  |  |  |  |  |  |  |  |  |  |
| 0.2028 | 10 | igd | 0.2144 | A | \|\| id:ebi-a-GCST90001485 | ebi-a-GCST90001485 | TRUE | G | 97990028 | 3.93E-11 | reported | 3437 | 0.0306 | rs61856986 |  |  |  |  |  |  |  |  |  |  |  |  |  |  |  |
| 0.2872 | 10 | igd | 0.2144 | A | \|\| id:ebi-a-GCST90001489 | ebi-a-GCST90001489 | TRUE | G | 97990028 | 1.45E-19 | reported | 3405 | 0.03154 | rs61856986 |  |  |  |  |  |  |  |  |  |  |  |  |  |  |  |
| 0.269 | 10 | igd | 0.2144 | A | \|\| id:ebi-a-GCST90001491 | ebi-a-GCST90001491 | TRUE | G | 97990028 | 9.50E-18 | reported | 3437 | 0.03118 | rs61856986 |  |  |  |  |  |  |  |  |  |  |  |  |  |  |  |
| 0.3156 | 10 | igd | 0.2144 | A | \|\| id:ebi-a-GCST90001495 | ebi-a-GCST90001495 | TRUE | G | 97990028 | 6.89E-23 | reported | 3405 | 0.03181 | rs61856986 |  |  |  |  |  |  |  |  |  |  |  |  |  |  |  |
| 0.2969 | 10 | igd | 0.2144 | A | \|\| id:ebi-a-GCST90001496 | ebi-a-GCST90001496 | TRUE | G | 97990028 | 8.42E-21 | reported | 3437 | 0.03154 | rs61856986 |  |  |  |  |  |  |  |  |  |  |  |  |  |  |  |
| 0.2962 | 10 | igd | 0.2144 | A | \|\| id:ebi-a-GCST90001497 | ebi-a-GCST90001497 | TRUE | G | 97990028 | 3.36E-21 | reported | 3437 | 0.03113 | rs61856986 |  |  |  |  |  |  |  |  |  |  |  |  |  |  |  |
| -0.2979 | 6 | igd | 0.2144 | A | \|\| id:ebi-a-GCST90002112 | ebi-a-GCST90002112 | TRUE | G | 30693497 | 9.89E-09 | reported | 1635 | 0.05169 | rs148511179 |  |  |  |  |  |  |  |  |  |  |  |  |  |  |  |
| -0.1816 | 22 | igd | 0.2142 | G | \|\| id:ebi-a-GCST90001702 | ebi-a-GCST90001702 | TRUE | T | 40215996 | 2.24E-09 | reported | 3657 | 0.03029 | rs6001688 |  |  |  |  |  |  |  |  |  |  |  |  |  |  |  |
| -0.187 | 22 | igd | 0.2142 | G | \|\| id:ebi-a-GCST90001703 | ebi-a-GCST90001703 | TRUE | T | 40215996 | 7.24E-10 | reported | 3657 | 0.03028 | rs6001688 |  |  |  |  |  |  |  |  |  |  |  |  |  |  |  |
| 0.2439 | 7 | igd | 0.2142 | T | \|\| id:ebi-a-GCST90002015 | ebi-a-GCST90002015 | TRUE | C | 1095701 | 9.17E-13 | reported | 2871 | 0.03399 | rs143407337 |  |  |  |  |  |  |  |  |  |  |  |  |  |  |  |
| 0.2427 | 7 | igd | 0.2142 | T | \|\| id:ebi-a-GCST90002016 | ebi-a-GCST90002016 | TRUE | C | 1095701 | 1.10E-12 | reported | 2871 | 0.03394 | rs143407337 |  |  |  |  |  |  |  |  |  |  |  |  |  |  |  |
| -0.3063 | 6 | igd | 0.2141 | T | \|\| id:ebi-a-GCST90002111 | ebi-a-GCST90002111 | TRUE | C | 30724807 | 2.34E-09 | reported | 1635 | 0.05099 | rs9262167 |  |  |  |  |  |  |  |  |  |  |  |  |  |  |  |
| 0.2533 | 22 | igd | 0.214 | G | \|\| id:ebi-a-GCST90001702 | ebi-a-GCST90001702 | TRUE | A | 42688532 | 4.55E-16 | reported | 3657 | 0.03104 | rs17002947 |  |  |  |  |  |  |  |  |  |  |  |  |  |  |  |
| 0.2422 | 22 | igd | 0.214 | G | \|\| id:ebi-a-GCST90001703 | ebi-a-GCST90001703 | TRUE | A | 42688532 | 7.98E-15 | reported | 3657 | 0.03105 | rs17002947 |  |  |  |  |  |  |  |  |  |  |  |  |  |  |  |
| -0.2215 | 6 | igd | 0.2139 | A | \|\| id:ebi-a-GCST90001820 | ebi-a-GCST90001820 | TRUE | G | 30973636 | 5.93E-09 | reported | 3656 | 0.03798 | rs373858 |  |  |  |  |  |  |  |  |  |  |  |  |  |  |  |
| 0.3661 | 19 | igd | 0.2138 | C | \|\| id:ebi-a-GCST90002046 | ebi-a-GCST90002046 | TRUE | G | 51736383 | 2.90E-17 | reported | 1635 | 0.04284 | rs33978622 |  |  |  |  |  |  |  |  |  |  |  |  |  |  |  |
| -0.339 | 6 | igd | 0.2134 | T | \|\| id:ebi-a-GCST90002108 | ebi-a-GCST90002108 | TRUE | C | 30724807 | 6.96E-11 | reported | 1579 | 0.05163 | rs9262167 |  |  |  |  |  |  |  |  |  |  |  |  |  |  |  |
| -0.5183 | 19 | igd | 0.2132 | A | \|\| id:ebi-a-GCST90001533 | ebi-a-GCST90001533 | TRUE | C | 51727962 | 1.14E-40 | reported | 1857 | 0.03788 | rs3865444 |  |  |  |  |  |  |  |  |  |  |  |  |  |  |  |
| -0.3696 | 19 | igd | 0.2131 | A | \|\| id:ebi-a-GCST90001517 | ebi-a-GCST90001517 | TRUE | C | 51727962 | 4.36E-21 | reported | 1858 | 0.03875 | rs3865444 |  |  |  |  |  |  |  |  |  |  |  |  |  |  |  |
| -0.3767 | 19 | igd | 0.2131 | A | \|\| id:ebi-a-GCST90001518 | ebi-a-GCST90001518 | TRUE | C | 51727962 | 2.13E-21 | reported | 1858 | 0.03917 | rs3865444 |  |  |  |  |  |  |  |  |  |  |  |  |  |  |  |
| -0.3278 | 19 | igd | 0.2131 | A | \|\| id:ebi-a-GCST90001520 | ebi-a-GCST90001520 | TRUE | C | 51727962 | 1.08E-16 | reported | 1858 | 0.03915 | rs3865444 |  |  |  |  |  |  |  |  |  |  |  |  |  |  |  |
| 0.2894 | 19 | igd | 0.2128 | T | \|\| id:ebi-a-GCST90001948 | ebi-a-GCST90001948 | TRUE | C | 51498131 | 2.02E-11 | reported | 1633 | 0.04286 | rs11667107 |  |  |  |  |  |  |  |  |  |  |  |  |  |  |  |
| 0.2001 | 6 | igd | 0.2128 | G | \|\| id:ebi-a-GCST90001973 | ebi-a-GCST90001973 | TRUE | C | 32452142 | 5.80E-09 | reported | 3113 | 0.03427 | rs1974459 |  |  |  |  |  |  |  |  |  |  |  |  |  |  |  |
| 0.2479 | 6 | igd | 0.2128 | G | \|\| id:ebi-a-GCST90002081 | ebi-a-GCST90002081 | TRUE | C | 32452142 | 3.00E-12 | reported | 3113 | 0.03538 | rs1974459 |  |  |  |  |  |  |  |  |  |  |  |  |  |  |  |
| 0.3015 | 19 | igd | 0.2127 | T | \|\| id:ebi-a-GCST90001946 | ebi-a-GCST90001946 | TRUE | C | 51498131 | 3.46E-12 | reported | 1634 | 0.043 | rs11667107 |  |  |  |  |  |  |  |  |  |  |  |  |  |  |  |
| 0.2798 | 19 | igd | 0.2127 | T | \|\| id:ebi-a-GCST90001949 | ebi-a-GCST90001949 | TRUE | C | 51498729 | 9.16E-11 | reported | 1634 | 0.04289 | rs11084045 |  |  |  |  |  |  |  |  |  |  |  |  |  |  |  |
| -1.139 | 19 | igd | 0.2122 | A | \|\| id:ebi-a-GCST90001948 | ebi-a-GCST90001948 | TRUE | C | 51727962 | 2.38E-191 | reported | 1633 | 0.03358 | rs3865444 |  |  |  |  |  |  |  |  |  |  |  |  |  |  |  |
| -1.143 | 19 | igd | 0.2121 | A | \|\| id:ebi-a-GCST90001946 | ebi-a-GCST90001946 | TRUE | C | 51727962 | 1.05E-190 | reported | 1634 | 0.03378 | rs3865444 |  |  |  |  |  |  |  |  |  |  |  |  |  |  |  |
| -1.119 | 19 | igd | 0.2121 | A | \|\| id:ebi-a-GCST90001949 | ebi-a-GCST90001949 | TRUE | C | 51727962 | 2.40E-183 | reported | 1634 | 0.03392 | rs3865444 |  |  |  |  |  |  |  |  |  |  |  |  |  |  |  |
| -0.2089 | 1 | igd | 0.2115 | T | \|\| id:ebi-a-GCST90001832 | ebi-a-GCST90001832 | TRUE | A | 111334781 | 5.18E-11 | reported | 2872 | 0.03169 | rs343813 |  |  |  |  |  |  |  |  |  |  |  |  |  |  |  |
| 0.3531 | 6 | igd | 0.2114 | T | \|\| id:ebi-a-GCST90002110 | ebi-a-GCST90002110 | TRUE | C | 32590735 | 1.85E-13 | reported | 1634 | 0.04758 | rs35062987 |  |  |  |  |  |  |  |  |  |  |  |  |  |  |  |
| -0.1845 | 20 | igd | 0.2113 | T | \|\| id:ebi-a-GCST90001800 | ebi-a-GCST90001800 | TRUE | C | 44558739 | 2.60E-09 | reported | 3656 | 0.03091 | rs113963381 |  |  |  |  |  |  |  |  |  |  |  |  |  |  |  |
| 0.3347 | 6 | igd | 0.2113 | T | \|\| id:ebi-a-GCST90002111 | ebi-a-GCST90002111 | TRUE | C | 32590735 | 1.45E-12 | reported | 1635 | 0.04691 | rs35062987 |  |  |  |  |  |  |  |  |  |  |  |  |  |  |  |
| -0.2239 | 20 | igd | 0.2112 | T | \|\| id:ebi-a-GCST90001798 | ebi-a-GCST90001798 | TRUE | C | 44558739 | 2.32E-13 | reported | 3657 | 0.03043 | rs113963381 |  |  |  |  |  |  |  |  |  |  |  |  |  |  |  |
| -0.1973 | 20 | igd | 0.2112 | T | \|\| id:ebi-a-GCST90001802 | ebi-a-GCST90001802 | TRUE | C | 44558739 | 1.56E-10 | reported | 3657 | 0.03075 | rs113963381 |  |  |  |  |  |  |  |  |  |  |  |  |  |  |  |
| -0.2529 | 20 | igd | 0.2112 | T | \|\| id:ebi-a-GCST90001804 | ebi-a-GCST90001804 | TRUE | C | 44558739 | 8.96E-17 | reported | 3657 | 0.03026 | rs113963381 |  |  |  |  |  |  |  |  |  |  |  |  |  |  |  |
| -0.1767 | 20 | igd | 0.2112 | T | \|\| id:ebi-a-GCST90001805 | ebi-a-GCST90001805 | TRUE | C | 44558739 | 1.04E-08 | reported | 3657 | 0.0308 | rs113963381 |  |  |  |  |  |  |  |  |  |  |  |  |  |  |  |
| -0.2294 | 20 | igd | 0.2112 | T | \|\| id:ebi-a-GCST90001808 | ebi-a-GCST90001808 | TRUE | C | 44558739 | 9.11E-14 | reported | 3657 | 0.03066 | rs113963381 |  |  |  |  |  |  |  |  |  |  |  |  |  |  |  |
| 0.1801 | 20 | igd | 0.211 | G | \|\| id:ebi-a-GCST90001399 | ebi-a-GCST90001399 | TRUE | A | 44733811 | 9.37E-09 | reported | 3657 | 0.0313 | rs6032661 |  |  |  |  |  |  |  |  |  |  |  |  |  |  |  |
| -0.2772 | 20 | igd | 0.211 | T | \|\| id:ebi-a-GCST90001798 | ebi-a-GCST90001798 | TRUE | C | 44745945 | 3.63E-19 | reported | 3657 | 0.03081 | rs6032666 |  |  |  |  |  |  |  |  |  |  |  |  |  |  |  |
| -0.2409 | 20 | igd | 0.211 | G | \|\| id:ebi-a-GCST90001800 | ebi-a-GCST90001800 | TRUE | A | 44733811 | 1.77E-14 | reported | 3656 | 0.0313 | rs6032661 |  |  |  |  |  |  |  |  |  |  |  |  |  |  |  |
| -0.3159 | 20 | igd | 0.211 | T | \|\| id:ebi-a-GCST90001804 | ebi-a-GCST90001804 | TRUE | C | 44745945 | 1.21E-24 | reported | 3657 | 0.0306 | rs6032666 |  |  |  |  |  |  |  |  |  |  |  |  |  |  |  |
| -0.2263 | 20 | igd | 0.211 | T | \|\| id:ebi-a-GCST90001805 | ebi-a-GCST90001805 | TRUE | C | 44745945 | 5.03E-13 | reported | 3657 | 0.03122 | rs6032666 |  |  |  |  |  |  |  |  |  |  |  |  |  |  |  |
| -0.2305 | 20 | igd | 0.211 | G | \|\| id:ebi-a-GCST90001806 | ebi-a-GCST90001806 | TRUE | A | 44733811 | 1.66E-13 | reported | 3656 | 0.03114 | rs6032661 |  |  |  |  |  |  |  |  |  |  |  |  |  |  |  |
| -0.1692 | 1 | igd | 0.2109 | T | \|\| id:ebi-a-GCST90001994 | ebi-a-GCST90001994 | TRUE | C | 161410253 | 4.91E-08 | reported | 3590 | 0.03096 | rs115805162 |  |  |  |  |  |  |  |  |  |  |  |  |  |  |  |
| -0.171 | 1 | igd | 0.2106 | G | \|\| id:ebi-a-GCST90001992 | ebi-a-GCST90001992 | TRUE | C | 161409023 | 2.00E-08 | reported | 3618 | 0.0304 | rs74124976 |  |  |  |  |  |  |  |  |  |  |  |  |  |  |  |
| 0.2202 | 1 | igd | 0.2104 | G | \|\| id:ebi-a-GCST90002012 | ebi-a-GCST90002012 | TRUE | A | 161502849 | 1.10E-12 | reported | 3582 | 0.03082 | rs79568124 |  |  |  |  |  |  |  |  |  |  |  |  |  |  |  |
| -0.2189 | 1 | igd | 0.2103 | A | \|\| id:ebi-a-GCST90001858 | ebi-a-GCST90001858 | TRUE | T | 167532878 | 8.60E-11 | reported | 2919 | 0.0336 | rs12145204 |  |  |  |  |  |  |  |  |  |  |  |  |  |  |  |
| -0.2012 | 1 | igd | 0.2103 | A | \|\| id:ebi-a-GCST90001861 | ebi-a-GCST90001861 | TRUE | T | 167532878 | 1.72E-09 | reported | 2919 | 0.03331 | rs12145204 |  |  |  |  |  |  |  |  |  |  |  |  |  |  |  |
| -0.1935 | 1 | igd | 0.2103 | A | \|\| id:ebi-a-GCST90001863 | ebi-a-GCST90001863 | TRUE | T | 167532878 | 6.03E-09 | reported | 2919 | 0.03318 | rs12145204 |  |  |  |  |  |  |  |  |  |  |  |  |  |  |  |
| -0.2322 | 1 | igd | 0.2103 | A | \|\| id:ebi-a-GCST90001864 | ebi-a-GCST90001864 | TRUE | T | 167532878 | 5.73E-12 | reported | 2919 | 0.03358 | rs12145204 |  |  |  |  |  |  |  |  |  |  |  |  |  |  |  |
| 0.2041 | 2 | igd | 0.2103 | C | \|\| id:ebi-a-GCST90001963 | ebi-a-GCST90001963 | TRUE | A | 204364150 | 3.00E-09 | reported | 2872 | 0.03431 | rs10432517 |  |  |  |  |  |  |  |  |  |  |  |  |  |  |  |
| -0.1935 | 1 | igd | 0.2102 | A | \|\| id:ebi-a-GCST90001845 | ebi-a-GCST90001845 | TRUE | T | 167532878 | 1.14E-08 | reported | 2911 | 0.0338 | rs12145204 |  |  |  |  |  |  |  |  |  |  |  |  |  |  |  |
| -0.1982 | 6 | igd | 0.2101 | T | \|\| id:ebi-a-GCST90001820 | ebi-a-GCST90001820 | TRUE | C | 28205826 | 1.70E-08 | reported | 3656 | 0.03505 | rs112421400 |  |  |  |  |  |  |  |  |  |  |  |  |  |  |  |
| 0.1993 | 3 | igd | 0.2096 | G | \|\| id:ebi-a-GCST90001808 | ebi-a-GCST90001808 | TRUE | A | 32714530 | 7.42E-11 | reported | 3657 | 0.03052 | rs6550148 |  |  |  |  |  |  |  |  |  |  |  |  |  |  |  |
| 0.2535 | 1 | igd | 0.2095 | G | \|\| id:ebi-a-GCST90002077 | ebi-a-GCST90002077 | TRUE | C | 161409023 | 5.24E-14 | reported | 2971 | 0.03352 | rs74124976 |  |  |  |  |  |  |  |  |  |  |  |  |  |  |  |
| -0.9497 | 19 | igd | 0.2094 | A | \|\| id:ebi-a-GCST90001951 | ebi-a-GCST90001951 | TRUE | C | 51727962 | 2.39E-108 | reported | 1464 | 0.0394 | rs3865444 |  |  |  |  |  |  |  |  |  |  |  |  |  |  |  |
| 0.2304 | 3 | igd | 0.2093 | A | \|\| id:ebi-a-GCST90001858 | ebi-a-GCST90001858 | TRUE | G | 45947066 | 1.23E-11 | reported | 2919 | 0.03386 | rs79179998 |  |  |  |  |  |  |  |  |  |  |  |  |  |  |  |
| 0.2126 | 22 | igd | 0.2092 | G | \|\| id:ebi-a-GCST90001702 | ebi-a-GCST90001702 | TRUE | C | 39647580 | 5.04E-12 | reported | 3657 | 0.03069 | rs9607627 |  |  |  |  |  |  |  |  |  |  |  |  |  |  |  |
| 0.209 | 22 | igd | 0.2092 | G | \|\| id:ebi-a-GCST90001703 | ebi-a-GCST90001703 | TRUE | C | 39647580 | 1.15E-11 | reported | 3657 | 0.03069 | rs9607627 |  |  |  |  |  |  |  |  |  |  |  |  |  |  |  |
| 0.2043 | 22 | igd | 0.2092 | G | \|\| id:ebi-a-GCST90001704 | ebi-a-GCST90001704 | TRUE | C | 39647580 | 3.11E-11 | reported | 3657 | 0.03066 | rs9607627 |  |  |  |  |  |  |  |  |  |  |  |  |  |  |  |
| 0.2081 | 22 | igd | 0.2092 | G | \|\| id:ebi-a-GCST90001710 | ebi-a-GCST90001710 | TRUE | C | 39647580 | 1.24E-11 | reported | 3657 | 0.03061 | rs9607627 |  |  |  |  |  |  |  |  |  |  |  |  |  |  |  |
| 0.1965 | 22 | igd | 0.2092 | G | \|\| id:ebi-a-GCST90001711 | ebi-a-GCST90001711 | TRUE | C | 39647580 | 1.44E-10 | reported | 3657 | 0.03056 | rs9607627 |  |  |  |  |  |  |  |  |  |  |  |  |  |  |  |
| 0.2048 | 2 | igd | 0.209 | C | \|\| id:ebi-a-GCST90002102 | ebi-a-GCST90002102 | TRUE | A | 41650674 | 1.77E-10 | reported | 2919 | 0.03198 | rs75884004 |  |  |  |  |  |  |  |  |  |  |  |  |  |  |  |
| -0.3023 | 6 | igd | 0.2089 | T | \|\| id:ebi-a-GCST90002112 | ebi-a-GCST90002112 | TRUE | C | 30421463 | 4.42E-09 | reported | 1635 | 0.05124 | rs184489107 |  |  |  |  |  |  |  |  |  |  |  |  |  |  |  |
| 0.1935 | 10 | igd | 0.2087 | G | \|\| id:ebi-a-GCST90001483 | ebi-a-GCST90001483 | TRUE | A | 97267538 | 5.25E-10 | reported | 3405 | 0.03106 | rs7896439 |  |  |  |  |  |  |  |  |  |  |  |  |  |  |  |
| 0.211 | 10 | igd | 0.2083 | G | \|\| id:ebi-a-GCST90001484 | ebi-a-GCST90001484 | TRUE | A | 97267538 | 8.93E-12 | reported | 3437 | 0.03082 | rs7896439 |  |  |  |  |  |  |  |  |  |  |  |  |  |  |  |
| 0.1891 | 10 | igd | 0.2083 | G | \|\| id:ebi-a-GCST90001485 | ebi-a-GCST90001485 | TRUE | A | 97267538 | 9.37E-10 | reported | 3437 | 0.03082 | rs7896439 |  |  |  |  |  |  |  |  |  |  |  |  |  |  |  |
| 0.1922 | 10 | igd | 0.2083 | G | \|\| id:ebi-a-GCST90001491 | ebi-a-GCST90001491 | TRUE | A | 97267538 | 1.14E-09 | reported | 3437 | 0.03148 | rs7896439 |  |  |  |  |  |  |  |  |  |  |  |  |  |  |  |
| 0.2281 | 10 | igd | 0.2083 | A | \|\| id:ebi-a-GCST90001934 | ebi-a-GCST90001934 | TRUE | G | 6115171 | 2.56E-14 | reported | 3435 | 0.02982 | rs10905719 |  |  |  |  |  |  |  |  |  |  |  |  |  |  |  |
| 0.1841 | 10 | igd | 0.2082 | A | \|\| id:ebi-a-GCST90001508 | ebi-a-GCST90001508 | TRUE | G | 6115171 | 7.06E-09 | reported | 3437 | 0.03172 | rs10905719 |  |  |  |  |  |  |  |  |  |  |  |  |  |  |  |
| 0.1734 | 10 | igd | 0.2082 | A | \|\| id:ebi-a-GCST90001509 | ebi-a-GCST90001509 | TRUE | G | 6115171 | 4.88E-08 | reported | 3437 | 0.03172 | rs10905719 |  |  |  |  |  |  |  |  |  |  |  |  |  |  |  |
| 0.2329 | 2 | igd | 0.2081 | C | \|\| id:ebi-a-GCST90002098 | ebi-a-GCST90002098 | TRUE | A | 41650674 | 7.54E-13 | reported | 2910 | 0.03234 | rs75884004 |  |  |  |  |  |  |  |  |  |  |  |  |  |  |  |
| -0.3224 | 6 | igd | 0.208 | T | \|\| id:ebi-a-GCST90002108 | ebi-a-GCST90002108 | TRUE | C | 30421463 | 4.31E-10 | reported | 1579 | 0.05131 | rs184489107 |  |  |  |  |  |  |  |  |  |  |  |  |  |  |  |
| -0.4466 | 10 | igd | 0.2078 | T | \|\| id:ebi-a-GCST90001483 | ebi-a-GCST90001483 | TRUE | G | 97430857 | 1.07E-47 | reported | 3405 | 0.0303 | rs7908923 |  |  |  |  |  |  |  |  |  |  |  |  |  |  |  |
| -0.1993 | 6 | igd | 0.2078 | A | \|\| id:ebi-a-GCST90001692 | ebi-a-GCST90001692 | TRUE | G | 31424778 | 9.91E-09 | reported | 3440 | 0.03468 | rs3130475 |  |  |  |  |  |  |  |  |  |  |  |  |  |  |  |
| 0.3306 | 22 | igd | 0.2078 | A | \|\| id:ebi-a-GCST90001702 | ebi-a-GCST90001702 | TRUE | G | 42419459 | 1.38E-26 | reported | 3657 | 0.03073 | rs74538360 |  |  |  |  |  |  |  |  |  |  |  |  |  |  |  |
| 0.339 | 22 | igd | 0.2078 | A | \|\| id:ebi-a-GCST90001703 | ebi-a-GCST90001703 | TRUE | G | 42419459 | 6.79E-28 | reported | 3657 | 0.0307 | rs74538360 |  |  |  |  |  |  |  |  |  |  |  |  |  |  |  |
| 0.3113 | 22 | igd | 0.2078 | A | \|\| id:ebi-a-GCST90001704 | ebi-a-GCST90001704 | TRUE | G | 42419459 | 8.66E-24 | reported | 3657 | 0.03074 | rs74538360 |  |  |  |  |  |  |  |  |  |  |  |  |  |  |  |
| 0.3029 | 22 | igd | 0.2078 | A | \|\| id:ebi-a-GCST90001710 | ebi-a-GCST90001710 | TRUE | G | 42419459 | 1.22E-22 | reported | 3657 | 0.03073 | rs74538360 |  |  |  |  |  |  |  |  |  |  |  |  |  |  |  |
| 0.3211 | 22 | igd | 0.2078 | A | \|\| id:ebi-a-GCST90001711 | ebi-a-GCST90001711 | TRUE | G | 42419459 | 2.09E-25 | reported | 3657 | 0.0306 | rs74538360 |  |  |  |  |  |  |  |  |  |  |  |  |  |  |  |
| 0.2188 | 3 | igd | 0.2074 | G | \|\| id:ebi-a-GCST90002017 | ebi-a-GCST90002017 | TRUE | C | 46264059 | 2.34E-10 | reported | 2850 | 0.0344 | rs34673873 |  |  |  |  |  |  |  |  |  |  |  |  |  |  |  |
| -0.2949 | 6 | igd | 0.2073 | C | \|\| id:ebi-a-GCST90002111 | ebi-a-GCST90002111 | TRUE | T | 30147694 | 1.06E-08 | reported | 1635 | 0.05129 | rs181874186 |  |  |  |  |  |  |  |  |  |  |  |  |  |  |  |
| -0.2211 | 6 | igd | 0.2072 | G | \|\| id:ebi-a-GCST90001820 | ebi-a-GCST90001820 | TRUE | A | 30761343 | 8.48E-09 | reported | 3656 | 0.03831 | rs9262205 |  |  |  |  |  |  |  |  |  |  |  |  |  |  |  |
| -0.2553 | 20 | igd | 0.207 | G | \|\| id:ebi-a-GCST90001801 | ebi-a-GCST90001801 | TRUE | A | 44733811 | 2.00E-09 | reported | 1836 | 0.04234 | rs6032661 |  |  |  |  |  |  |  |  |  |  |  |  |  |  |  |
| -0.2665 | 1 | igd | 0.2067 | G | \|\| id:ebi-a-GCST90002091 | ebi-a-GCST90002091 | TRUE | A | 161499460 | 7.21E-09 | reported | 1635 | 0.04582 | rs11578245 |  |  |  |  |  |  |  |  |  |  |  |  |  |  |  |
| -0.2459 | 6 | igd | 0.206 | T | \|\| id:ebi-a-GCST90002105 | ebi-a-GCST90002105 | TRUE | C | 30421463 | 1.44E-08 | reported | 2872 | 0.04325 | rs184489107 |  |  |  |  |  |  |  |  |  |  |  |  |  |  |  |
| 0.2112 | 6 | igd | 0.2058 | G | \|\| id:ebi-a-GCST90002081 | ebi-a-GCST90002081 | TRUE | C | 32338885 | 1.58E-09 | reported | 3113 | 0.03489 | rs7763192 |  |  |  |  |  |  |  |  |  |  |  |  |  |  |  |
| -0.1753 | 2 | igd | 0.2057 | G | \|\| id:ebi-a-GCST90002098 | ebi-a-GCST90002098 | TRUE | A | 38083416 | 4.81E-08 | reported | 2910 | 0.03202 | rs934209 |  |  |  |  |  |  |  |  |  |  |  |  |  |  |  |
| -0.2022 | 6 | igd | 0.2055 | G | \|\| id:ebi-a-GCST90001932 | ebi-a-GCST90001932 | TRUE | C | 32338885 | 1.55E-08 | reported | 2920 | 0.03564 | rs7763192 |  |  |  |  |  |  |  |  |  |  |  |  |  |  |  |
| -0.1773 | 22 | igd | 0.2054 | A | \|\| id:ebi-a-GCST90001710 | ebi-a-GCST90001710 | TRUE | C | 40206811 | 8.34E-09 | reported | 3657 | 0.0307 | rs6001686 |  |  |  |  |  |  |  |  |  |  |  |  |  |  |  |
| -0.1847 | 22 | igd | 0.2054 | A | \|\| id:ebi-a-GCST90001711 | ebi-a-GCST90001711 | TRUE | C | 40206811 | 1.78E-09 | reported | 3657 | 0.03061 | rs6001686 |  |  |  |  |  |  |  |  |  |  |  |  |  |  |  |
| -0.28 | 6 | igd | 0.2053 | T | \|\| id:ebi-a-GCST90002110 | ebi-a-GCST90002110 | TRUE | C | 27700822 | 4.11E-08 | reported | 1634 | 0.0508 | rs11969118 |  |  |  |  |  |  |  |  |  |  |  |  |  |  |  |
| -0.186 | 4 | igd | 0.2052 | G | \|\| id:ebi-a-GCST90001865 | ebi-a-GCST90001865 | TRUE | C | 6388191 | 4.21E-08 | reported | 2919 | 0.03384 | rs4689428 |  |  |  |  |  |  |  |  |  |  |  |  |  |  |  |
| -0.203 | 6 | igd | 0.2045 | A | \|\| id:ebi-a-GCST90001687 | ebi-a-GCST90001687 | TRUE | G | 32595682 | 2.53E-08 | reported | 3408 | 0.03634 | rs6922257 |  |  |  |  |  |  |  |  |  |  |  |  |  |  |  |
| 0.2057 | 3 | igd | 0.2045 | A | \|\| id:ebi-a-GCST90001806 | ebi-a-GCST90001806 | TRUE | G | 32838168 | 4.50E-11 | reported | 3656 | 0.03113 | rs62251900 |  |  |  |  |  |  |  |  |  |  |  |  |  |  |  |
| 0.2552 | 6 | igd | 0.2039 | A | \|\| id:ebi-a-GCST90001692 | ebi-a-GCST90001692 | TRUE | G | 32595682 | 2.95E-12 | reported | 3440 | 0.03643 | rs6922257 |  |  |  |  |  |  |  |  |  |  |  |  |  |  |  |
| -0.276 | 20 | igd | 0.2039 | A | \|\| id:ebi-a-GCST90001980 | ebi-a-GCST90001980 | TRUE | G | 44756891 | 1.29E-18 | reported | 3629 | 0.03117 | rs3765456 |  |  |  |  |  |  |  |  |  |  |  |  |  |  |  |
| -0.3374 | 20 | igd | 0.2039 | T | \|\| id:ebi-a-GCST90001985 | ebi-a-GCST90001985 | TRUE | G | 44755111 | 9.61E-27 | reported | 3629 | 0.03126 | rs3746821 |  |  |  |  |  |  |  |  |  |  |  |  |  |  |  |
| -0.2044 | 16 | igd | 0.2038 | C | \|\| id:ebi-a-GCST90001858 | ebi-a-GCST90001858 | TRUE | T | 430304 | 1.49E-09 | reported | 2919 | 0.03371 | rs12921174 |  |  |  |  |  |  |  |  |  |  |  |  |  |  |  |
| 0.2382 | 1 | igd | 0.2038 | A | \|\| id:ebi-a-GCST90001999 | ebi-a-GCST90001999 | TRUE | G | 161478235 | 3.75E-14 | reported | 3621 | 0.03134 | rs35855160 |  |  |  |  |  |  |  |  |  |  |  |  |  |  |  |
| -0.3709 | 20 | igd | 0.2034 | A | \|\| id:ebi-a-GCST90001981 | ebi-a-GCST90001981 | TRUE | G | 44756891 | 2.51E-32 | reported | 3618 | 0.03103 | rs3765456 |  |  |  |  |  |  |  |  |  |  |  |  |  |  |  |
| -0.3107 | 20 | igd | 0.2034 | T | \|\| id:ebi-a-GCST90001989 | ebi-a-GCST90001989 | TRUE | G | 44755111 | 9.93E-23 | reported | 3621 | 0.03145 | rs3746821 |  |  |  |  |  |  |  |  |  |  |  |  |  |  |  |
| -0.6699 | 10 | igd | 0.2031 | A | \|\| id:ebi-a-GCST90002030 | ebi-a-GCST90002030 | TRUE | G | 97681174 | 1.26E-90 | reported | 2920 | 0.03203 | rs76712236 |  |  |  |  |  |  |  |  |  |  |  |  |  |  |  |
| -0.6058 | 10 | igd | 0.2031 | A | \|\| id:ebi-a-GCST90002031 | ebi-a-GCST90002031 | TRUE | G | 97681174 | 2.81E-70 | reported | 2920 | 0.03326 | rs76712236 |  |  |  |  |  |  |  |  |  |  |  |  |  |  |  |
| -0.567 | 10 | igd | 0.2031 | A | \|\| id:ebi-a-GCST90002032 | ebi-a-GCST90002032 | TRUE | G | 97681174 | 1.91E-61 | reported | 2920 | 0.03348 | rs76712236 |  |  |  |  |  |  |  |  |  |  |  |  |  |  |  |
| 0.1894 | 10 | igd | 0.203 | A | \|\| id:ebi-a-GCST90001761 | ebi-a-GCST90001761 | TRUE | G | 94522438 | 2.00E-09 | reported | 3657 | 0.0315 | rs35039517 |  |  |  |  |  |  |  |  |  |  |  |  |  |  |  |
| -0.3587 | 22 | igd | 0.2029 | C | \|\| id:ebi-a-GCST90001702 | ebi-a-GCST90001702 | TRUE | T | 41410922 | 5.41E-30 | reported | 3657 | 0.03124 | rs12484084 |  |  |  |  |  |  |  |  |  |  |  |  |  |  |  |
| -0.3547 | 22 | igd | 0.2029 | C | \|\| id:ebi-a-GCST90001703 | ebi-a-GCST90001703 | TRUE | T | 41410922 | 2.29E-29 | reported | 3657 | 0.03125 | rs12484084 |  |  |  |  |  |  |  |  |  |  |  |  |  |  |  |
| -0.3313 | 22 | igd | 0.2029 | C | \|\| id:ebi-a-GCST90001704 | ebi-a-GCST90001704 | TRUE | T | 41410922 | 7.77E-26 | reported | 3657 | 0.03128 | rs12484084 |  |  |  |  |  |  |  |  |  |  |  |  |  |  |  |
| -0.3232 | 22 | igd | 0.2029 | C | \|\| id:ebi-a-GCST90001710 | ebi-a-GCST90001710 | TRUE | T | 41410922 | 1.01E-24 | reported | 3657 | 0.03126 | rs12484084 |  |  |  |  |  |  |  |  |  |  |  |  |  |  |  |
| -0.3197 | 22 | igd | 0.2029 | C | \|\| id:ebi-a-GCST90001711 | ebi-a-GCST90001711 | TRUE | T | 41410922 | 2.54E-24 | reported | 3657 | 0.03119 | rs12484084 |  |  |  |  |  |  |  |  |  |  |  |  |  |  |  |
| -0.4069 | 10 | igd | 0.2026 | A | \|\| id:ebi-a-GCST90001483 | ebi-a-GCST90001483 | TRUE | G | 97681174 | 2.13E-38 | reported | 3405 | 0.03101 | rs76712236 |  |  |  |  |  |  |  |  |  |  |  |  |  |  |  |
| -0.51 | 10 | igd | 0.2026 | A | \|\| id:ebi-a-GCST90001495 | ebi-a-GCST90001495 | TRUE | G | 97681174 | 7.71E-56 | reported | 3405 | 0.0318 | rs76712236 |  |  |  |  |  |  |  |  |  |  |  |  |  |  |  |
| -0.53 | 10 | igd | 0.2025 | A | \|\| id:ebi-a-GCST90001660 | ebi-a-GCST90001660 | TRUE | G | 97681174 | 1.12E-61 | reported | 3408 | 0.03133 | rs76712236 |  |  |  |  |  |  |  |  |  |  |  |  |  |  |  |
| 0.1966 | 6 | igd | 0.2024 | G | \|\| id:ebi-a-GCST90001745 | ebi-a-GCST90001745 | TRUE | A | 30735105 | 1.62E-08 | reported | 3657 | 0.03473 | rs12210947 |  |  |  |  |  |  |  |  |  |  |  |  |  |  |  |
| -0.4446 | 10 | igd | 0.2021 | A | \|\| id:ebi-a-GCST90001484 | ebi-a-GCST90001484 | TRUE | G | 97681174 | 1.91E-46 | reported | 3437 | 0.03061 | rs76712236 |  |  |  |  |  |  |  |  |  |  |  |  |  |  |  |
| -0.4306 | 10 | igd | 0.2021 | A | \|\| id:ebi-a-GCST90001485 | ebi-a-GCST90001485 | TRUE | G | 97681174 | 1.01E-43 | reported | 3437 | 0.03062 | rs76712236 |  |  |  |  |  |  |  |  |  |  |  |  |  |  |  |
| -0.4861 | 10 | igd | 0.2021 | A | \|\| id:ebi-a-GCST90001491 | ebi-a-GCST90001491 | TRUE | G | 97681174 | 3.03E-53 | reported | 3437 | 0.0311 | rs76712236 |  |  |  |  |  |  |  |  |  |  |  |  |  |  |  |
| -0.5592 | 10 | igd | 0.2021 | A | \|\| id:ebi-a-GCST90001496 | ebi-a-GCST90001496 | TRUE | G | 97681174 | 9.32E-69 | reported | 3437 | 0.03119 | rs76712236 |  |  |  |  |  |  |  |  |  |  |  |  |  |  |  |
| -0.5459 | 10 | igd | 0.2021 | A | \|\| id:ebi-a-GCST90001497 | ebi-a-GCST90001497 | TRUE | G | 97681174 | 3.55E-67 | reported | 3437 | 0.03084 | rs76712236 |  |  |  |  |  |  |  |  |  |  |  |  |  |  |  |
| 0.2572 | 2 | igd | 0.2021 | G | \|\| id:ebi-a-GCST90002119 | ebi-a-GCST90002119 | TRUE | C | 87124134 | 2.49E-14 | reported | 2920 | 0.03358 | rs113998866 |  |  |  |  |  |  |  |  |  |  |  |  |  |  |  |
| -0.5336 | 10 | igd | 0.2019 | A | \|\| id:ebi-a-GCST90001658 | ebi-a-GCST90001658 | TRUE | G | 97681174 | 8.06E-69 | reported | 3440 | 0.02975 | rs76712236 |  |  |  |  |  |  |  |  |  |  |  |  |  |  |  |
| -0.5408 | 10 | igd | 0.2019 | A | \|\| id:ebi-a-GCST90001659 | ebi-a-GCST90001659 | TRUE | G | 97681174 | 1.08E-70 | reported | 3440 | 0.02972 | rs76712236 |  |  |  |  |  |  |  |  |  |  |  |  |  |  |  |
| 0.2944 | 19 | igd | 0.2017 | C | \|\| id:ebi-a-GCST90001955 | ebi-a-GCST90001955 | TRUE | T | 51521876 | 2.58E-10 | reported | 1554 | 0.04625 | rs7255834 |  |  |  |  |  |  |  |  |  |  |  |  |  |  |  |
| 0.198 | 6 | igd | 0.2016 | C | \|\| id:ebi-a-GCST90001684 | ebi-a-GCST90001684 | TRUE | T | 32190217 | 4.75E-08 | reported | 3371 | 0.03619 | rs915895 |  |  |  |  |  |  |  |  |  |  |  |  |  |  |  |
| 0.225 | 1 | igd | 0.2016 | A | \|\| id:ebi-a-GCST90002013 | ebi-a-GCST90002013 | TRUE | G | 161478235 | 1.61E-10 | reported | 2870 | 0.03505 | rs35855160 |  |  |  |  |  |  |  |  |  |  |  |  |  |  |  |
| 0.2811 | 22 | igd | 0.2015 | A | \|\| id:ebi-a-GCST90001702 | ebi-a-GCST90001702 | TRUE | G | 42546852 | 6.85E-19 | reported | 3657 | 0.03149 | rs59390354 |  |  |  |  |  |  |  |  |  |  |  |  |  |  |  |
| 0.2677 | 22 | igd | 0.2015 | A | \|\| id:ebi-a-GCST90001703 | ebi-a-GCST90001703 | TRUE | G | 42546852 | 2.86E-17 | reported | 3657 | 0.03152 | rs59390354 |  |  |  |  |  |  |  |  |  |  |  |  |  |  |  |
| 0.277 | 22 | igd | 0.2015 | A | \|\| id:ebi-a-GCST90001704 | ebi-a-GCST90001704 | TRUE | G | 42546852 | 1.94E-18 | reported | 3657 | 0.03145 | rs59390354 |  |  |  |  |  |  |  |  |  |  |  |  |  |  |  |
| 0.2752 | 22 | igd | 0.2015 | A | \|\| id:ebi-a-GCST90001710 | ebi-a-GCST90001710 | TRUE | G | 42546852 | 3.05E-18 | reported | 3657 | 0.03143 | rs59390354 |  |  |  |  |  |  |  |  |  |  |  |  |  |  |  |
| 0.2589 | 22 | igd | 0.2015 | A | \|\| id:ebi-a-GCST90001711 | ebi-a-GCST90001711 | TRUE | G | 42546852 | 2.14E-16 | reported | 3657 | 0.03138 | rs59390354 |  |  |  |  |  |  |  |  |  |  |  |  |  |  |  |
| -0.3163 | 6 | igd | 0.2015 | T | \|\| id:ebi-a-GCST90002111 | ebi-a-GCST90002111 | TRUE | C | 30614900 | 1.43E-09 | reported | 1635 | 0.05197 | rs1110445 |  |  |  |  |  |  |  |  |  |  |  |  |  |  |  |
| -0.2153 | 6 | igd | 0.2012 | A | \|\| id:ebi-a-GCST90002119 | ebi-a-GCST90002119 | TRUE | G | 32749805 | 1.47E-08 | reported | 2920 | 0.0379 | rs2857188 |  |  |  |  |  |  |  |  |  |  |  |  |  |  |  |
| 0.1993 | 6 | igd | 0.2011 | T | \|\| id:ebi-a-GCST90001758 | ebi-a-GCST90001758 | TRUE | G | 30730446 | 1.13E-08 | reported | 3657 | 0.03483 | rs139847598 |  |  |  |  |  |  |  |  |  |  |  |  |  |  |  |
| 0.3407 | 20 | igd | 0.201 | T | \|\| id:ebi-a-GCST90001980 | ebi-a-GCST90001980 | TRUE | C | 44652397 | 3.54E-28 | reported | 3629 | 0.03069 | rs62215576 |  |  |  |  |  |  |  |  |  |  |  |  |  |  |  |
| -0.317 | 6 | igd | 0.2008 | A | \|\| id:ebi-a-GCST90002108 | ebi-a-GCST90002108 | TRUE | G | 30612495 | 2.34E-09 | reported | 1579 | 0.05278 | rs3094102 |  |  |  |  |  |  |  |  |  |  |  |  |  |  |  |
| -0.3124 | 6 | igd | 0.2006 | G | \|\| id:ebi-a-GCST90002111 | ebi-a-GCST90002111 | TRUE | C | 30442167 | 2.52E-09 | reported | 1635 | 0.05212 | rs151310747 |  |  |  |  |  |  |  |  |  |  |  |  |  |  |  |
| -0.2279 | 19 | igd | 0.2002 | T | \|\| id:prot-a-439 | prot-a-439 | TRUE | C | 51883001 | 3.98E-13 | reported | 3301 | 0.0314 | rs147321547 |  |  |  |  |  |  |  |  |  |  |  |  |  |  |  |
| 0.1846 | 10 | igd | 0.1998 | T | \|\| id:ebi-a-GCST90001660 | ebi-a-GCST90001660 | TRUE | A | 99644605 | 1.32E-08 | reported | 3408 | 0.0324 | rs77507167 |  |  |  |  |  |  |  |  |  |  |  |  |  |  |  |
| 0.2003 | 2 | igd | 0.1996 | A | \|\| id:ebi-a-GCST90002102 | ebi-a-GCST90002102 | TRUE | T | 41546342 | 1.06E-09 | reported | 2919 | 0.03273 | rs17027464 |  |  |  |  |  |  |  |  |  |  |  |  |  |  |  |
| 0.3417 | 19 | igd | 0.1995 | A | \|\| id:ebi-a-GCST90001955 | ebi-a-GCST90001955 | TRUE | G | 51544665 | 1.66E-13 | reported | 1554 | 0.04593 | rs138712525 |  |  |  |  |  |  |  |  |  |  |  |  |  |  |  |
| 0.346 | 19 | igd | 0.1994 | A | \|\| id:ebi-a-GCST90001954 | ebi-a-GCST90001954 | TRUE | G | 51544665 | 7.09E-14 | reported | 1555 | 0.0458 | rs138712525 |  |  |  |  |  |  |  |  |  |  |  |  |  |  |  |
| 0.1756 | 10 | igd | 0.1993 | T | \|\| id:ebi-a-GCST90001745 | ebi-a-GCST90001745 | TRUE | C | 94516008 | 3.07E-08 | reported | 3657 | 0.03165 | rs11187166 |  |  |  |  |  |  |  |  |  |  |  |  |  |  |  |
| 0.1757 | 10 | igd | 0.1993 | T | \|\| id:ebi-a-GCST90001757 | ebi-a-GCST90001757 | TRUE | C | 94516008 | 2.97E-08 | reported | 3657 | 0.03163 | rs11187166 |  |  |  |  |  |  |  |  |  |  |  |  |  |  |  |
| -0.2437 | 6 | igd | 0.1993 | T | \|\| id:ebi-a-GCST90001932 | ebi-a-GCST90001932 | TRUE | C | 32584581 | 1.54E-11 | reported | 2920 | 0.036 | rs73730372 |  |  |  |  |  |  |  |  |  |  |  |  |  |  |  |
| 0.1921 | 2 | igd | 0.1991 | A | \|\| id:ebi-a-GCST90001654 | ebi-a-GCST90001654 | TRUE | G | 87047145 | 1.45E-09 | reported | 3408 | 0.03166 | rs6710361 |  |  |  |  |  |  |  |  |  |  |  |  |  |  |  |
| 0.2097 | 6 | igd | 0.1991 | A | \|\| id:ebi-a-GCST90001773 | ebi-a-GCST90001773 | TRUE | G | 107178044 | 1.54E-11 | reported | 3652 | 0.03099 | rs9486451 |  |  |  |  |  |  |  |  |  |  |  |  |  |  |  |
| 0.1917 | 6 | igd | 0.199 | A | \|\| id:ebi-a-GCST90001765 | ebi-a-GCST90001765 | TRUE | G | 107178044 | 3.80E-10 | reported | 3655 | 0.03054 | rs9486451 |  |  |  |  |  |  |  |  |  |  |  |  |  |  |  |
| 0.1877 | 6 | igd | 0.199 | A | \|\| id:ebi-a-GCST90001771 | ebi-a-GCST90001771 | TRUE | G | 107178044 | 9.91E-10 | reported | 3653 | 0.03063 | rs9486451 |  |  |  |  |  |  |  |  |  |  |  |  |  |  |  |
| 0.1812 | 6 | igd | 0.199 | A | \|\| id:ebi-a-GCST90001772 | ebi-a-GCST90001772 | TRUE | G | 107178044 | 3.95E-09 | reported | 3655 | 0.03071 | rs9486451 |  |  |  |  |  |  |  |  |  |  |  |  |  |  |  |
| 0.3653 | 6 | igd | 0.199 | A | \|\| id:ebi-a-GCST90001890 | ebi-a-GCST90001890 | TRUE | G | 90976768 | 2.25E-26 | reported | 2920 | 0.03405 | rs72928038 |  |  |  |  |  |  |  |  |  |  |  |  |  |  |  |
| -0.4805 | 2 | igd | 0.199 | G | \|\| id:ebi-a-GCST90001891 | ebi-a-GCST90001891 | TRUE | A | 204596729 | 5.57E-45 | reported | 2920 | 0.03356 | rs13390252 |  |  |  |  |  |  |  |  |  |  |  |  |  |  |  |
| 0.2512 | 6 | igd | 0.199 | A | \|\| id:ebi-a-GCST90001894 | ebi-a-GCST90001894 | TRUE | G | 90976768 | 1.84E-13 | reported | 2920 | 0.03397 | rs72928038 |  |  |  |  |  |  |  |  |  |  |  |  |  |  |  |
| 0.5885 | 6 | igd | 0.199 | A | \|\| id:ebi-a-GCST90001896 | ebi-a-GCST90001896 | TRUE | G | 90976768 | 5.13E-67 | reported | 2920 | 0.03315 | rs72928038 |  |  |  |  |  |  |  |  |  |  |  |  |  |  |  |
| 0.233 | 6 | igd | 0.199 | A | \|\| id:ebi-a-GCST90001898 | ebi-a-GCST90001898 | TRUE | G | 90976768 | 9.00E-12 | reported | 2920 | 0.03402 | rs72928038 |  |  |  |  |  |  |  |  |  |  |  |  |  |  |  |
| 0.3426 | 19 | igd | 0.199 | A | \|\| id:ebi-a-GCST90001953 | ebi-a-GCST90001953 | TRUE | G | 51544665 | 1.22E-13 | reported | 1558 | 0.04579 | rs138712525 |  |  |  |  |  |  |  |  |  |  |  |  |  |  |  |
| 0.1931 | 22 | igd | 0.1989 | C | \|\| id:ebi-a-GCST90001702 | ebi-a-GCST90001702 | TRUE | T | 41947716 | 9.26E-10 | reported | 3657 | 0.03146 | rs13056537 |  |  |  |  |  |  |  |  |  |  |  |  |  |  |  |
| 0.1837 | 22 | igd | 0.1989 | C | \|\| id:ebi-a-GCST90001703 | ebi-a-GCST90001703 | TRUE | T | 41947716 | 5.77E-09 | reported | 3657 | 0.03147 | rs13056537 |  |  |  |  |  |  |  |  |  |  |  |  |  |  |  |
| 0.1817 | 22 | igd | 0.1989 | C | \|\| id:ebi-a-GCST90001710 | ebi-a-GCST90001710 | TRUE | T | 41947716 | 7.60E-09 | reported | 3657 | 0.03139 | rs13056537 |  |  |  |  |  |  |  |  |  |  |  |  |  |  |  |
| 0.1881 | 22 | igd | 0.1989 | C | \|\| id:ebi-a-GCST90001711 | ebi-a-GCST90001711 | TRUE | T | 41947716 | 2.06E-09 | reported | 3657 | 0.03131 | rs13056537 |  |  |  |  |  |  |  |  |  |  |  |  |  |  |  |
| 0.1846 | 6 | igd | 0.1989 | A | \|\| id:ebi-a-GCST90001769 | ebi-a-GCST90001769 | TRUE | G | 107178044 | 3.29E-09 | reported | 3648 | 0.03113 | rs9486451 |  |  |  |  |  |  |  |  |  |  |  |  |  |  |  |
| 0.2478 | 6 | igd | 0.1988 | G | \|\| id:ebi-a-GCST90001692 | ebi-a-GCST90001692 | TRUE | A | 32584880 | 4.12E-11 | reported | 3440 | 0.03742 | rs73730377 |  |  |  |  |  |  |  |  |  |  |  |  |  |  |  |
| 0.2243 | 6 | igd | 0.1988 | G | \|\| id:ebi-a-GCST90002071 | ebi-a-GCST90002071 | TRUE | A | 32584880 | 1.21E-08 | reported | 2872 | 0.03925 | rs73730377 |  |  |  |  |  |  |  |  |  |  |  |  |  |  |  |
| -0.5184 | 2 | igd | 0.1987 | C | \|\| id:ebi-a-GCST90001900 | ebi-a-GCST90001900 | TRUE | T | 204623481 | 7.38E-52 | reported | 2919 | 0.03354 | rs62184016 |  |  |  |  |  |  |  |  |  |  |  |  |  |  |  |
| 0.2968 | 19 | igd | 0.1987 | A | \|\| id:ebi-a-GCST90001957 | ebi-a-GCST90001957 | TRUE | G | 51544665 | 1.77E-10 | reported | 1578 | 0.04622 | rs138712525 |  |  |  |  |  |  |  |  |  |  |  |  |  |  |  |
| -0.4171 | 2 | igd | 0.1986 | C | \|\| id:ebi-a-GCST90001890 | ebi-a-GCST90001890 | TRUE | T | 204623481 | 5.77E-33 | reported | 2920 | 0.03444 | rs62184016 |  |  |  |  |  |  |  |  |  |  |  |  |  |  |  |
| 0.1909 | 2 | igd | 0.1986 | A | \|\| id:ebi-a-GCST90002098 | ebi-a-GCST90002098 | TRUE | T | 41546342 | 1.02E-08 | reported | 2910 | 0.03323 | rs17027464 |  |  |  |  |  |  |  |  |  |  |  |  |  |  |  |
| 0.2998 | 6 | igd | 0.1986 | A | \|\| id:ebi-a-GCST90002105 | ebi-a-GCST90002105 | TRUE | G | 31428500 | 6.43E-15 | reported | 2872 | 0.03825 | rs73728583 |  |  |  |  |  |  |  |  |  |  |  |  |  |  |  |
| 0.3025 | 19 | igd | 0.1985 | A | \|\| id:ebi-a-GCST90001956 | ebi-a-GCST90001956 | TRUE | G | 51544665 | 7.50E-11 | reported | 1579 | 0.04615 | rs138712525 |  |  |  |  |  |  |  |  |  |  |  |  |  |  |  |
| -0.4161 | 2 | igd | 0.1983 | T | \|\| id:ebi-a-GCST90001887 | ebi-a-GCST90001887 | TRUE | C | 204609402 | 3.31E-34 | reported | 2920 | 0.03368 | rs62183989 |  |  |  |  |  |  |  |  |  |  |  |  |  |  |  |
| -0.4077 | 2 | igd | 0.1983 | T | \|\| id:ebi-a-GCST90001889 | ebi-a-GCST90001889 | TRUE | C | 204609402 | 2.32E-34 | reported | 2920 | 0.03293 | rs62183989 |  |  |  |  |  |  |  |  |  |  |  |  |  |  |  |
| -0.3589 | 2 | igd | 0.1983 | T | \|\| id:ebi-a-GCST90001902 | ebi-a-GCST90001902 | TRUE | C | 204609402 | 1.13E-27 | reported | 2920 | 0.03258 | rs62183989 |  |  |  |  |  |  |  |  |  |  |  |  |  |  |  |
| 0.1952 | 6 | igd | 0.1981 | G | \|\| id:ebi-a-GCST90001745 | ebi-a-GCST90001745 | TRUE | T | 32584977 | 2.96E-08 | reported | 3657 | 0.03514 | rs113016235 |  |  |  |  |  |  |  |  |  |  |  |  |  |  |  |
| -0.1887 | 16 | igd | 0.1981 | T | \|\| id:ebi-a-GCST90002087 | ebi-a-GCST90002087 | TRUE | C | 30610876 | 2.92E-08 | reported | 2872 | 0.03392 | rs35661204 |  |  |  |  |  |  |  |  |  |  |  |  |  |  |  |
| -0.1992 | 16 | igd | 0.1981 | T | \|\| id:ebi-a-GCST90002088 | ebi-a-GCST90002088 | TRUE | C | 30610876 | 4.22E-09 | reported | 2872 | 0.0338 | rs35661204 |  |  |  |  |  |  |  |  |  |  |  |  |  |  |  |
| 0.2823 | 6 | igd | 0.1979 | A | \|\| id:ebi-a-GCST90002108 | ebi-a-GCST90002108 | TRUE | G | 31428500 | 1.66E-08 | reported | 1579 | 0.04975 | rs73728583 |  |  |  |  |  |  |  |  |  |  |  |  |  |  |  |
| 0.2904 | 6 | igd | 0.1976 | A | \|\| id:ebi-a-GCST90001767 | ebi-a-GCST90001767 | TRUE | G | 107178044 | 2.07E-11 | reported | 1832 | 0.04307 | rs9486451 |  |  |  |  |  |  |  |  |  |  |  |  |  |  |  |
| -0.4779 | 2 | igd | 0.1976 | G | \|\| id:ebi-a-GCST90001893 | ebi-a-GCST90001893 | TRUE | A | 204631712 | 9.69E-44 | reported | 2920 | 0.03389 | rs56216383 |  |  |  |  |  |  |  |  |  |  |  |  |  |  |  |
| -0.3545 | 2 | igd | 0.1976 | C | \|\| id:ebi-a-GCST90001899 | ebi-a-GCST90001899 | TRUE | T | 204627500 | 1.68E-27 | reported | 2920 | 0.0323 | rs56209416 |  |  |  |  |  |  |  |  |  |  |  |  |  |  |  |
| -0.2204 | 6 | igd | 0.1976 | T | \|\| id:ebi-a-GCST90002119 | ebi-a-GCST90002119 | TRUE | G | 30730446 | 4.90E-09 | reported | 2920 | 0.03755 | rs139847598 |  |  |  |  |  |  |  |  |  |  |  |  |  |  |  |
| 0.4475 | 7 | igd | 0.1975 | T | \|\| id:ebi-a-GCST90001966 | ebi-a-GCST90001966 | TRUE | C | 92408370 | 3.45E-38 | reported | 2850 | 0.03413 | rs445 |  |  |  |  |  |  |  |  |  |  |  |  |  |  |  |
| 0.2088 | 7 | igd | 0.1975 | T | \|\| id:ebi-a-GCST90002073 | ebi-a-GCST90002073 | TRUE | C | 92408370 | 1.45E-09 | reported | 2850 | 0.03441 | rs445 |  |  |  |  |  |  |  |  |  |  |  |  |  |  |  |
| 0.2826 | 7 | igd | 0.1975 | T | \|\| id:ebi-a-GCST90002018 | ebi-a-GCST90002018 | TRUE | C | 92408370 | 2.41E-16 | reported | 2850 | 0.03426 | rs445 |  |  |  |  |  |  |  |  |  |  |  |  |  |  |  |
| 0.3156 | 7 | igd | 0.1974 | T | \|\| id:ebi-a-GCST90001926 | ebi-a-GCST90001926 | TRUE | C | 92408370 | 4.02E-20 | reported | 2920 | 0.03411 | rs445 |  |  |  |  |  |  |  |  |  |  |  |  |  |  |  |
| 0.2094 | 7 | igd | 0.1974 | T | \|\| id:ebi-a-GCST90002033 | ebi-a-GCST90002033 | TRUE | C | 92408370 | 1.06E-09 | reported | 2920 | 0.03421 | rs445 |  |  |  |  |  |  |  |  |  |  |  |  |  |  |  |
| 0.229 | 7 | igd | 0.1968 | T | \|\| id:ebi-a-GCST90002074 | ebi-a-GCST90002074 | TRUE | C | 92408370 | 4.94E-12 | reported | 3112 | 0.03302 | rs445 |  |  |  |  |  |  |  |  |  |  |  |  |  |  |  |
| -0.2381 | 2 | igd | 0.1966 | C | \|\| id:ebi-a-GCST90001894 | ebi-a-GCST90001894 | TRUE | T | 204513610 | 8.67E-12 | reported | 2920 | 0.03473 | rs3925039 |  |  |  |  |  |  |  |  |  |  |  |  |  |  |  |
| -0.2258 | 2 | igd | 0.1966 | C | \|\| id:ebi-a-GCST90001898 | ebi-a-GCST90001898 | TRUE | T | 204513610 | 9.66E-11 | reported | 2920 | 0.03477 | rs3925039 |  |  |  |  |  |  |  |  |  |  |  |  |  |  |  |
| 0.2892 | 6 | igd | 0.1966 | A | \|\| id:ebi-a-GCST90002112 | ebi-a-GCST90002112 | TRUE | G | 31428500 | 6.56E-09 | reported | 1635 | 0.04959 | rs73728583 |  |  |  |  |  |  |  |  |  |  |  |  |  |  |  |
| 0.2448 | 22 | igd | 0.1965 | C | \|\| id:ebi-a-GCST90001702 | ebi-a-GCST90001702 | TRUE | T | 43725421 | 7.91E-15 | reported | 3657 | 0.03138 | rs950553 |  |  |  |  |  |  |  |  |  |  |  |  |  |  |  |
| 0.2341 | 22 | igd | 0.1965 | C | \|\| id:ebi-a-GCST90001710 | ebi-a-GCST90001710 | TRUE | T | 43725421 | 9.50E-14 | reported | 3657 | 0.03131 | rs950553 |  |  |  |  |  |  |  |  |  |  |  |  |  |  |  |
| 0.2357 | 22 | igd | 0.1965 | C | \|\| id:ebi-a-GCST90001711 | ebi-a-GCST90001711 | TRUE | T | 43725421 | 5.64E-14 | reported | 3657 | 0.03123 | rs950553 |  |  |  |  |  |  |  |  |  |  |  |  |  |  |  |
| 0.4371 | 7 | igd | 0.1965 | T | \|\| id:ebi-a-GCST90002078 | ebi-a-GCST90002078 | TRUE | C | 92408370 | 3.84E-40 | reported | 3112 | 0.03249 | rs445 |  |  |  |  |  |  |  |  |  |  |  |  |  |  |  |
| 0.2336 | 6 | igd | 0.1964 | C | \|\| id:ebi-a-GCST90002105 | ebi-a-GCST90002105 | TRUE | T | 33480738 | 3.61E-10 | reported | 2872 | 0.03713 | rs68191 |  |  |  |  |  |  |  |  |  |  |  |  |  |  |  |
| -0.2428 | 6 | igd | 0.1963 | A | \|\| id:ebi-a-GCST90001820 | ebi-a-GCST90001820 | TRUE | C | 30451167 | 2.97E-08 | reported | 3656 | 0.0437 | rs138339691 |  |  |  |  |  |  |  |  |  |  |  |  |  |  |  |
| 0.1905 | 1 | igd | 0.1962 | C | \|\| id:ebi-a-GCST90002077 | ebi-a-GCST90002077 | TRUE | A | 161481752 | 2.14E-08 | reported | 2971 | 0.03392 | rs12142756 |  |  |  |  |  |  |  |  |  |  |  |  |  |  |  |
| -0.2507 | 6 | igd | 0.196 | T | \|\| id:ebi-a-GCST90002105 | ebi-a-GCST90002105 | TRUE | C | 32205922 | 1.49E-10 | reported | 2872 | 0.03899 | rs114356017 |  |  |  |  |  |  |  |  |  |  |  |  |  |  |  |
| 0.2116 | 3 | igd | 0.1954 | T | \|\| id:ebi-a-GCST90001798 | ebi-a-GCST90001798 | TRUE | G | 32497574 | 6.96E-12 | reported | 3657 | 0.03075 | rs3888882 |  |  |  |  |  |  |  |  |  |  |  |  |  |  |  |
| 0.1747 | 3 | igd | 0.1954 | T | \|\| id:ebi-a-GCST90001804 | ebi-a-GCST90001804 | TRUE | G | 32497574 | 1.36E-08 | reported | 3657 | 0.03069 | rs3888882 |  |  |  |  |  |  |  |  |  |  |  |  |  |  |  |
| 0.1805 | 3 | igd | 0.1954 | T | \|\| id:ebi-a-GCST90001805 | ebi-a-GCST90001805 | TRUE | G | 32497574 | 7.05E-09 | reported | 3657 | 0.03111 | rs3888882 |  |  |  |  |  |  |  |  |  |  |  |  |  |  |  |
| 0.1912 | 1 | igd | 0.1954 | T | \|\| id:ebi-a-GCST90002077 | ebi-a-GCST90002077 | TRUE | C | 161235597 | 1.68E-08 | reported | 2971 | 0.03379 | rs75213764 |  |  |  |  |  |  |  |  |  |  |  |  |  |  |  |
| 0.2254 | 3 | igd | 0.1953 | T | \|\| id:ebi-a-GCST90001800 | ebi-a-GCST90001800 | TRUE | G | 32497574 | 5.72E-13 | reported | 3656 | 0.03117 | rs3888882 |  |  |  |  |  |  |  |  |  |  |  |  |  |  |  |
| -0.4345 | 1 | igd | 0.1952 | C | \|\| id:ebi-a-GCST90001970 | ebi-a-GCST90001970 | TRUE | T | 161399920 | 1.43E-37 | reported | 2971 | 0.03345 | rs9427372 |  |  |  |  |  |  |  |  |  |  |  |  |  |  |  |
| -0.4617 | 1 | igd | 0.1952 | C | \|\| id:ebi-a-GCST90002077 | ebi-a-GCST90002077 | TRUE | T | 161399920 | 5.80E-43 | reported | 2971 | 0.03307 | rs9427372 |  |  |  |  |  |  |  |  |  |  |  |  |  |  |  |
| 0.2545 | 6 | igd | 0.1947 | G | \|\| id:ebi-a-GCST90001745 | ebi-a-GCST90001745 | TRUE | A | 32640351 | 1.38E-08 | reported | 3657 | 0.04474 | rs111259171 |  |  |  |  |  |  |  |  |  |  |  |  |  |  |  |
| 0.1971 | 6 | igd | 0.1945 | C | \|\| id:ebi-a-GCST90001973 | ebi-a-GCST90001973 | TRUE | G | 32627042 | 3.02E-08 | reported | 3113 | 0.03549 | rs9273390 |  |  |  |  |  |  |  |  |  |  |  |  |  |  |  |
| 0.2364 | 6 | igd | 0.1945 | C | \|\| id:ebi-a-GCST90002081 | ebi-a-GCST90002081 | TRUE | G | 32627042 | 1.35E-10 | reported | 3113 | 0.03669 | rs9273390 |  |  |  |  |  |  |  |  |  |  |  |  |  |  |  |
| 0.2444 | 6 | igd | 0.1942 | T | \|\| id:ebi-a-GCST90002081 | ebi-a-GCST90002081 | TRUE | G | 32626267 | 7.80E-12 | reported | 3113 | 0.03559 | rs35139945 |  |  |  |  |  |  |  |  |  |  |  |  |  |  |  |
| 0.2357 | 2 | igd | 0.1941 | A | \|\| id:ebi-a-GCST90001507 | ebi-a-GCST90001507 | TRUE | G | 38900088 | 1.18E-12 | reported | 3405 | 0.03303 | rs13032255 |  |  |  |  |  |  |  |  |  |  |  |  |  |  |  |
| -1.131 | 19 | igd | 0.1939 | A | \|\| id:ebi-a-GCST90001954 | ebi-a-GCST90001954 | TRUE | G | 51731176 | 1.81E-150 | reported | 1555 | 0.03861 | rs7245846 |  |  |  |  |  |  |  |  |  |  |  |  |  |  |  |
| -1.219 | 19 | igd | 0.1938 | A | \|\| id:ebi-a-GCST90001956 | ebi-a-GCST90001956 | TRUE | C | 51727962 | 2.81E-187 | reported | 1579 | 0.03627 | rs3865444 |  |  |  |  |  |  |  |  |  |  |  |  |  |  |  |
| -1.223 | 19 | igd | 0.1936 | A | \|\| id:ebi-a-GCST90001957 | ebi-a-GCST90001957 | TRUE | C | 51727962 | 9.18E-189 | reported | 1578 | 0.0362 | rs3865444 |  |  |  |  |  |  |  |  |  |  |  |  |  |  |  |
| -1.214 | 19 | igd | 0.1934 | A | \|\| id:ebi-a-GCST90001947 | ebi-a-GCST90001947 | TRUE | C | 51727962 | 7.83E-184 | reported | 1577 | 0.03655 | rs3865444 |  |  |  |  |  |  |  |  |  |  |  |  |  |  |  |
| -1.115 | 19 | igd | 0.1934 | A | \|\| id:ebi-a-GCST90001955 | ebi-a-GCST90001955 | TRUE | G | 51731176 | 6.08E-143 | reported | 1554 | 0.0393 | rs7245846 |  |  |  |  |  |  |  |  |  |  |  |  |  |  |  |
| 0.223 | 6 | igd | 0.1934 | C | \|\| id:ebi-a-GCST90002071 | ebi-a-GCST90002071 | TRUE | G | 32627042 | 1.62E-08 | reported | 2872 | 0.03937 | rs9273390 |  |  |  |  |  |  |  |  |  |  |  |  |  |  |  |
| 0.1748 | 6 | igd | 0.1933 | T | \|\| id:ebi-a-GCST90001686 | ebi-a-GCST90001686 | TRUE | A | 31419065 | 4.48E-08 | reported | 3440 | 0.03189 | rs3130901 |  |  |  |  |  |  |  |  |  |  |  |  |  |  |  |
| -0.2466 | 6 | igd | 0.1933 | C | \|\| id:ebi-a-GCST90001932 | ebi-a-GCST90001932 | TRUE | G | 32627042 | 6.18E-11 | reported | 2920 | 0.03757 | rs9273390 |  |  |  |  |  |  |  |  |  |  |  |  |  |  |  |
| 0.2385 | 6 | igd | 0.1932 | C | \|\| id:ebi-a-GCST90001692 | ebi-a-GCST90001692 | TRUE | G | 32627042 | 2.01E-10 | reported | 3440 | 0.03739 | rs9273390 |  |  |  |  |  |  |  |  |  |  |  |  |  |  |  |
| -0.1963 | 10 | igd | 0.1931 | G | \|\| id:ebi-a-GCST90001672 | ebi-a-GCST90001672 | TRUE | C | 95015801 | 6.27E-09 | reported | 3408 | 0.03371 | rs11596416 |  |  |  |  |  |  |  |  |  |  |  |  |  |  |  |
| 21 | 6 | igd | 0.1931 | T | \|\| id:ebi-a-GCST90001695 | ebi-a-GCST90001695 | TRUE | A | 31419065 | 1.89E-08 | reported | 3408 | 3.727 | rs3130901 |  |  |  |  |  |  |  |  |  |  |  |  |  |  |  |
| -0.1781 | 10 | igd | 0.193 | G | \|\| id:ebi-a-GCST90001659 | ebi-a-GCST90001659 | TRUE | C | 95015801 | 2.46E-08 | reported | 3440 | 0.03187 | rs11596416 |  |  |  |  |  |  |  |  |  |  |  |  |  |  |  |
| -0.1942 | 10 | igd | 0.193 | G | \|\| id:ebi-a-GCST90001670 | ebi-a-GCST90001670 | TRUE | C | 95015801 | 5.04E-09 | reported | 3440 | 0.03314 | rs11596416 |  |  |  |  |  |  |  |  |  |  |  |  |  |  |  |
| 0.2129 | 6 | igd | 0.193 | T | \|\| id:ebi-a-GCST90001684 | ebi-a-GCST90001684 | TRUE | A | 31419065 | 1.15E-08 | reported | 3371 | 0.03721 | rs3130901 |  |  |  |  |  |  |  |  |  |  |  |  |  |  |  |
| 0.2218 | 6 | igd | 0.1929 | T | \|\| id:ebi-a-GCST90001687 | ebi-a-GCST90001687 | TRUE | A | 31419065 | 6.90E-10 | reported | 3408 | 0.03586 | rs3130901 |  |  |  |  |  |  |  |  |  |  |  |  |  |  |  |
| 0.3107 | 19 | igd | 0.1929 | A | \|\| id:ebi-a-GCST90001948 | ebi-a-GCST90001948 | TRUE | G | 51544665 | 6.19E-12 | reported | 1633 | 0.04486 | rs138712525 |  |  |  |  |  |  |  |  |  |  |  |  |  |  |  |
| 0.3048 | 19 | igd | 0.1928 | A | \|\| id:ebi-a-GCST90001946 | ebi-a-GCST90001946 | TRUE | G | 51544665 | 2.07E-11 | reported | 1634 | 0.04517 | rs138712525 |  |  |  |  |  |  |  |  |  |  |  |  |  |  |  |
| 0.3012 | 19 | igd | 0.1928 | A | \|\| id:ebi-a-GCST90001949 | ebi-a-GCST90001949 | TRUE | G | 51544665 | 2.45E-11 | reported | 1634 | 0.0448 | rs138712525 |  |  |  |  |  |  |  |  |  |  |  |  |  |  |  |
| 0.1659 | 2 | igd | 0.1927 | A | \|\| id:ebi-a-GCST90001655 | ebi-a-GCST90001655 | TRUE | G | 87021659 | 8.92E-09 | reported | 3440 | 0.02878 | rs34546581 |  |  |  |  |  |  |  |  |  |  |  |  |  |  |  |
| -0.2325 | 10 | igd | 0.1923 | A | \|\| id:ebi-a-GCST90001484 | ebi-a-GCST90001484 | TRUE | C | 97146820 | 9.46E-14 | reported | 3437 | 0.03109 | rs10159602 |  |  |  |  |  |  |  |  |  |  |  |  |  |  |  |
| -0.1961 | 10 | igd | 0.1923 | A | \|\| id:ebi-a-GCST90001485 | ebi-a-GCST90001485 | TRUE | C | 97146820 | 3.34E-10 | reported | 3437 | 0.03112 | rs10159602 |  |  |  |  |  |  |  |  |  |  |  |  |  |  |  |
| -0.1842 | 10 | igd | 0.1922 | A | \|\| id:ebi-a-GCST90001659 | ebi-a-GCST90001659 | TRUE | C | 97146820 | 2.12E-09 | reported | 3440 | 0.03067 | rs10159602 |  |  |  |  |  |  |  |  |  |  |  |  |  |  |  |
| 0.1981 | 6 | igd | 0.1922 | C | \|\| id:ebi-a-GCST90001745 | ebi-a-GCST90001745 | TRUE | G | 32627042 | 2.60E-08 | reported | 3657 | 0.03552 | rs9273390 |  |  |  |  |  |  |  |  |  |  |  |  |  |  |  |
| -0.204 | 2 | igd | 0.192 | A | \|\| id:ebi-a-GCST90001893 | ebi-a-GCST90001893 | TRUE | G | 204800126 | 6.36E-09 | reported | 2920 | 0.03503 | rs11571305 |  |  |  |  |  |  |  |  |  |  |  |  |  |  |  |
| -0.1986 | 10 | igd | 0.1919 | A | \|\| id:ebi-a-GCST90001483 | ebi-a-GCST90001483 | TRUE | C | 97146820 | 2.96E-10 | reported | 3405 | 0.03142 | rs10159602 |  |  |  |  |  |  |  |  |  |  |  |  |  |  |  |
| -0.1931 | 10 | igd | 0.1919 | A | \|\| id:ebi-a-GCST90001489 | ebi-a-GCST90001489 | TRUE | C | 97146820 | 2.48E-09 | reported | 3405 | 0.03229 | rs10159602 |  |  |  |  |  |  |  |  |  |  |  |  |  |  |  |
| -0.1951 | 10 | igd | 0.1919 | C | \|\| id:ebi-a-GCST90001491 | ebi-a-GCST90001491 | TRUE | T | 97130450 | 9.31E-10 | reported | 3437 | 0.03178 | rs12269572 |  |  |  |  |  |  |  |  |  |  |  |  |  |  |  |
| 0.2517 | 22 | igd | 0.1917 | A | \|\| id:ebi-a-GCST90001711 | ebi-a-GCST90001711 | TRUE | G | 42349912 | 8.80E-15 | reported | 3657 | 0.03232 | rs55903228 |  |  |  |  |  |  |  |  |  |  |  |  |  |  |  |
| 0.2611 | 1 | igd | 0.1917 | T | \|\| id:ebi-a-GCST90002035 | ebi-a-GCST90002035 | TRUE | C | 161404553 | 8.54E-14 | reported | 2871 | 0.03482 | rs9427373 |  |  |  |  |  |  |  |  |  |  |  |  |  |  |  |
| 0.1948 | 6 | igd | 0.1911 | T | \|\| id:ebi-a-GCST90001743 | ebi-a-GCST90001743 | TRUE | C | 32583054 | 3.97E-08 | reported | 3660 | 0.0354 | rs111411953 |  |  |  |  |  |  |  |  |  |  |  |  |  |  |  |
| 0.2153 | 6 | igd | 0.191 | T | \|\| id:ebi-a-GCST90001752 | ebi-a-GCST90001752 | TRUE | C | 32583054 | 1.32E-09 | reported | 3657 | 0.03542 | rs111411953 |  |  |  |  |  |  |  |  |  |  |  |  |  |  |  |
| 0.2094 | 6 | igd | 0.191 | T | \|\| id:ebi-a-GCST90001757 | ebi-a-GCST90001757 | TRUE | C | 32583054 | 4.75E-09 | reported | 3657 | 0.03568 | rs111411953 |  |  |  |  |  |  |  |  |  |  |  |  |  |  |  |
| 0.2024 | 6 | igd | 0.191 | T | \|\| id:ebi-a-GCST90001973 | ebi-a-GCST90001973 | TRUE | C | 32627885 | 7.89E-09 | reported | 3113 | 0.03497 | rs113664950 |  |  |  |  |  |  |  |  |  |  |  |  |  |  |  |
| -0.2663 | 1 | igd | 0.1909 | T | \|\| id:ebi-a-GCST90001467 | ebi-a-GCST90001467 | TRUE | C | 161404553 | 1.91E-16 | reported | 3410 | 0.03221 | rs9427373 |  |  |  |  |  |  |  |  |  |  |  |  |  |  |  |
| -0.2341 | 10 | igd | 0.1907 | G | \|\| id:ebi-a-GCST90001672 | ebi-a-GCST90001672 | TRUE | A | 98244903 | 4.31E-12 | reported | 3408 | 0.03368 | rs11597763 |  |  |  |  |  |  |  |  |  |  |  |  |  |  |  |
| -0.2817 | 6 | igd | 0.1905 | A | \|\| id:ebi-a-GCST90002111 | ebi-a-GCST90002111 | TRUE | T | 27742258 | 4.79E-08 | reported | 1635 | 0.05137 | rs111828532 |  |  |  |  |  |  |  |  |  |  |  |  |  |  |  |
| -0.2244 | 10 | igd | 0.1904 | G | \|\| id:ebi-a-GCST90001670 | ebi-a-GCST90001670 | TRUE | A | 98244903 | 1.48E-11 | reported | 3440 | 0.03314 | rs11597763 |  |  |  |  |  |  |  |  |  |  |  |  |  |  |  |
| -0.237 | 10 | igd | 0.1903 | G | \|\| id:ebi-a-GCST90001497 | ebi-a-GCST90001497 | TRUE | A | 98244903 | 7.94E-13 | reported | 3437 | 0.03296 | rs11597763 |  |  |  |  |  |  |  |  |  |  |  |  |  |  |  |
| -1.151 | 19 | igd | 0.1896 | A | \|\| id:ebi-a-GCST90001952 | ebi-a-GCST90001952 | TRUE | C | 51727962 | 1.71E-153 | reported | 1569 | 0.03884 | rs3865444 |  |  |  |  |  |  |  |  |  |  |  |  |  |  |  |
| -0.2257 | 6 | igd | 0.1896 | T | \|\| id:ebi-a-GCST90002105 | ebi-a-GCST90002105 | TRUE | C | 32627885 | 1.02E-08 | reported | 2872 | 0.03929 | rs113664950 |  |  |  |  |  |  |  |  |  |  |  |  |  |  |  |
| 0.2527 | 6 | igd | 0.1895 | T | \|\| id:ebi-a-GCST90001692 | ebi-a-GCST90001692 | TRUE | C | 32453474 | 5.41E-11 | reported | 3440 | 0.0384 | rs144039739 |  |  |  |  |  |  |  |  |  |  |  |  |  |  |  |
| 0.2159 | 2 | igd | 0.189 | G | \|\| id:ebi-a-GCST90001694 | ebi-a-GCST90001694 | TRUE | T | 87035076 | 5.00E-11 | reported | 3440 | 0.03275 | rs6755452 |  |  |  |  |  |  |  |  |  |  |  |  |  |  |  |
| 0.2138 | 2 | igd | 0.1886 | A | \|\| id:ebi-a-GCST90002101 | ebi-a-GCST90002101 | TRUE | C | 38910402 | 1.04E-09 | reported | 2911 | 0.03491 | rs4670885 |  |  |  |  |  |  |  |  |  |  |  |  |  |  |  |
| 0.2368 | 6 | igd | 0.1885 | T | \|\| id:ebi-a-GCST90002105 | ebi-a-GCST90002105 | TRUE | C | 32179896 | 3.40E-10 | reported | 2872 | 0.03758 | rs2071286 |  |  |  |  |  |  |  |  |  |  |  |  |  |  |  |
| -0.186 | 1 | igd | 0.1884 | A | \|\| id:ebi-a-GCST90001938 | ebi-a-GCST90001938 | TRUE | G | 119421209 | 2.73E-08 | reported | 3118 | 0.03337 | rs6681289 |  |  |  |  |  |  |  |  |  |  |  |  |  |  |  |
| -0.378 | 6 | igd | 0.1872 | C | \|\| id:ebi-a-GCST90001932 | ebi-a-GCST90001932 | TRUE | T | 32505536 | 7.64E-13 | reported | 2920 | 0.0525 | rs28572744 |  |  |  |  |  |  |  |  |  |  |  |  |  |  |  |
| 0.2366 | 1 | igd | 0.1872 | C | \|\| id:ebi-a-GCST90001996 | ebi-a-GCST90001996 | TRUE | T | 161500875 | 4.49E-13 | reported | 3579 | 0.03256 | rs10919347 |  |  |  |  |  |  |  |  |  |  |  |  |  |  |  |
| 0.4963 | 2 | igd | 0.1871 | T | \|\| id:ebi-a-GCST90002098 | ebi-a-GCST90002098 | TRUE | C | 38933040 | 5.75E-51 | reported | 2910 | 0.03241 | rs34558770 |  |  |  |  |  |  |  |  |  |  |  |  |  |  |  |
| 0.34 | 6 | igd | 0.1868 | G | \|\| id:ebi-a-GCST90002071 | ebi-a-GCST90002071 | TRUE | T | 32501627 | 4.35E-10 | reported | 2872 | 0.05429 | rs115780495 |  |  |  |  |  |  |  |  |  |  |  |  |  |  |  |
| 0.2281 | 1 | igd | 0.1866 | C | \|\| id:ebi-a-GCST90001997 | ebi-a-GCST90001997 | TRUE | T | 161500875 | 5.75E-12 | reported | 3590 | 0.03302 | rs10919347 |  |  |  |  |  |  |  |  |  |  |  |  |  |  |  |
| 0.2089 | 1 | igd | 0.1866 | C | \|\| id:ebi-a-GCST90001995 | ebi-a-GCST90001995 | TRUE | T | 161500875 | 2.09E-10 | reported | 3590 | 0.03278 | rs10919347 |  |  |  |  |  |  |  |  |  |  |  |  |  |  |  |
| 0.5039 | 6 | igd | 0.1865 | T | \|\| id:ebi-a-GCST90002111 | ebi-a-GCST90002111 | TRUE | C | 32550431 | 2.60E-17 | reported | 1635 | 0.05888 | rs36101847 |  |  |  |  |  |  |  |  |  |  |  |  |  |  |  |
| 0.1827 | 2 | igd | 0.1864 | G | \|\| id:ebi-a-GCST90002102 | ebi-a-GCST90002102 | TRUE | A | 39365307 | 2.64E-08 | reported | 2919 | 0.03274 | rs62142833 |  |  |  |  |  |  |  |  |  |  |  |  |  |  |  |
| 0.5467 | 6 | igd | 0.1864 | T | \|\| id:ebi-a-GCST90002110 | ebi-a-GCST90002110 | TRUE | C | 32550431 | 1.38E-19 | reported | 1634 | 0.05961 | rs36101847 |  |  |  |  |  |  |  |  |  |  |  |  |  |  |  |
| 0.2181 | 2 | igd | 0.1862 | A | \|\| id:ebi-a-GCST90001562 | ebi-a-GCST90001562 | TRUE | C | 38910402 | 4.33E-14 | reported | 3427 | 0.02876 | rs4670885 |  |  |  |  |  |  |  |  |  |  |  |  |  |  |  |
| 0.5825 | 6 | igd | 0.1862 | T | \|\| id:ebi-a-GCST90002108 | ebi-a-GCST90002108 | TRUE | C | 32673272 | 1.11E-30 | reported | 1579 | 0.04952 | rs9275489 |  |  |  |  |  |  |  |  |  |  |  |  |  |  |  |
| -0.5422 | 10 | igd | 0.1859 | G | \|\| id:ebi-a-GCST90001489 | ebi-a-GCST90001489 | TRUE | T | 97366817 | 4.82E-60 | reported | 3405 | 0.03252 | rs11592809 |  |  |  |  |  |  |  |  |  |  |  |  |  |  |  |
| -0.5814 | 10 | igd | 0.1859 | G | \|\| id:ebi-a-GCST90001495 | ebi-a-GCST90001495 | TRUE | T | 97366817 | 1.06E-67 | reported | 3405 | 0.0327 | rs11592809 |  |  |  |  |  |  |  |  |  |  |  |  |  |  |  |
| -0.6221 | 10 | igd | 0.1859 | G | \|\| id:ebi-a-GCST90001658 | ebi-a-GCST90001658 | TRUE | T | 97366817 | 1.95E-87 | reported | 3440 | 0.03049 | rs11592809 |  |  |  |  |  |  |  |  |  |  |  |  |  |  |  |
| -0.6294 | 10 | igd | 0.1859 | T | \|\| id:ebi-a-GCST90001660 | ebi-a-GCST90001660 | TRUE | C | 97464060 | 3.10E-80 | reported | 3408 | 0.03231 | rs12779833 |  |  |  |  |  |  |  |  |  |  |  |  |  |  |  |
| -0.6326 | 10 | igd | 0.1859 | T | \|\| id:ebi-a-GCST90001672 | ebi-a-GCST90001672 | TRUE | C | 97464060 | 1.88E-79 | reported | 3408 | 0.03264 | rs12779833 |  |  |  |  |  |  |  |  |  |  |  |  |  |  |  |
| -0.2296 | 6 | igd | 0.1858 | C | \|\| id:ebi-a-GCST90002119 | ebi-a-GCST90002119 | TRUE | T | 30914552 | 1.69E-09 | reported | 2920 | 0.03799 | rs3130781 |  |  |  |  |  |  |  |  |  |  |  |  |  |  |  |
| -0.4347 | 10 | igd | 0.1856 | T | \|\| id:ebi-a-GCST90001483 | ebi-a-GCST90001483 | TRUE | C | 97464060 | 4.00E-40 | reported | 3405 | 0.03236 | rs12779833 |  |  |  |  |  |  |  |  |  |  |  |  |  |  |  |
| -0.4666 | 10 | igd | 0.1856 | G | \|\| id:ebi-a-GCST90001484 | ebi-a-GCST90001484 | TRUE | T | 97366817 | 9.86E-48 | reported | 3437 | 0.03166 | rs11592809 |  |  |  |  |  |  |  |  |  |  |  |  |  |  |  |
| -0.5537 | 10 | igd | 0.1856 | G | \|\| id:ebi-a-GCST90001491 | ebi-a-GCST90001491 | TRUE | T | 97366817 | 2.38E-64 | reported | 3437 | 0.03201 | rs11592809 |  |  |  |  |  |  |  |  |  |  |  |  |  |  |  |
| -0.5857 | 10 | igd | 0.1856 | C | \|\| id:ebi-a-GCST90001497 | ebi-a-GCST90001497 | TRUE | G | 97370942 | 4.84E-72 | reported | 3437 | 0.03187 | rs2296690 |  |  |  |  |  |  |  |  |  |  |  |  |  |  |  |
| -0.6235 | 10 | igd | 0.1856 | T | \|\| id:ebi-a-GCST90001659 | ebi-a-GCST90001659 | TRUE | C | 97464060 | 2.47E-86 | reported | 3440 | 0.03077 | rs12779833 |  |  |  |  |  |  |  |  |  |  |  |  |  |  |  |
| -0.6317 | 10 | igd | 0.1856 | T | \|\| id:ebi-a-GCST90001670 | ebi-a-GCST90001670 | TRUE | C | 97464060 | 7.81E-82 | reported | 3440 | 0.03209 | rs12779833 |  |  |  |  |  |  |  |  |  |  |  |  |  |  |  |
| -0.6266 | 10 | igd | 0.1856 | T | \|\| id:ebi-a-GCST90001671 | ebi-a-GCST90001671 | TRUE | C | 97464060 | 4.18E-80 | reported | 3440 | 0.0322 | rs12779833 |  |  |  |  |  |  |  |  |  |  |  |  |  |  |  |
| -0.2619 | 10 | igd | 0.1853 | C | \|\| id:ebi-a-GCST90001483 | ebi-a-GCST90001483 | TRUE | T | 97329435 | 7.43E-16 | reported | 3405 | 0.03232 | rs78153419 |  |  |  |  |  |  |  |  |  |  |  |  |  |  |  |
| -0.5977 | 10 | igd | 0.1853 | T | \|\| id:ebi-a-GCST90001496 | ebi-a-GCST90001496 | TRUE | C | 97464060 | 5.85E-72 | reported | 3437 | 0.03254 | rs12779833 |  |  |  |  |  |  |  |  |  |  |  |  |  |  |  |
| -0.8358 | 19 | igd | 0.1853 | A | \|\| id:ebi-a-GCST90001950 | ebi-a-GCST90001950 | TRUE | G | 51731176 | 1.02E-58 | reported | 1244 | 0.04903 | rs7245846 |  |  |  |  |  |  |  |  |  |  |  |  |  |  |  |
| -0.1838 | 1 | igd | 0.1852 | A | \|\| id:ebi-a-GCST90001980 | ebi-a-GCST90001980 | TRUE | G | 161234796 | 7.93E-09 | reported | 3629 | 0.03178 | rs2501880 |  |  |  |  |  |  |  |  |  |  |  |  |  |  |  |
| -0.4442 | 10 | igd | 0.185 | G | \|\| id:ebi-a-GCST90001485 | ebi-a-GCST90001485 | TRUE | A | 97459003 | 1.33E-42 | reported | 3437 | 0.03202 | rs11593038 |  |  |  |  |  |  |  |  |  |  |  |  |  |  |  |
| 0.2117 | 10 | igd | 0.1849 | T | \|\| id:ebi-a-GCST90002029 | ebi-a-GCST90002029 | TRUE | C | 97801295 | 6.58E-10 | reported | 2915 | 0.03416 | rs149019898 |  |  |  |  |  |  |  |  |  |  |  |  |  |  |  |
| -1.138 | 19 | igd | 0.1845 | A | \|\| id:ebi-a-GCST90001953 | ebi-a-GCST90001953 | TRUE | C | 51727962 | 2.60E-145 | reported | 1558 | 0.03974 | rs3865444 |  |  |  |  |  |  |  |  |  |  |  |  |  |  |  |
| -0.3122 | 19 | igd | 0.1842 | C | \|\| id:ebi-a-GCST90001947 | ebi-a-GCST90001947 | TRUE | T | 51589473 | 8.08E-11 | reported | 1577 | 0.0477 | rs4802765 |  |  |  |  |  |  |  |  |  |  |  |  |  |  |  |
| 0.2009 | 6 | igd | 0.184 | G | \|\| id:ebi-a-GCST90001752 | ebi-a-GCST90001752 | TRUE | A | 31755776 | 4.14E-08 | reported | 3657 | 0.03655 | rs9469053 |  |  |  |  |  |  |  |  |  |  |  |  |  |  |  |
| 0.2372 | 6 | igd | 0.184 | G | \|\| id:ebi-a-GCST90001757 | ebi-a-GCST90001757 | TRUE | A | 31755776 | 1.47E-10 | reported | 3657 | 0.03691 | rs9469053 |  |  |  |  |  |  |  |  |  |  |  |  |  |  |  |
| 0.2584 | 6 | igd | 0.184 | C | \|\| id:ebi-a-GCST90002071 | ebi-a-GCST90002071 | TRUE | T | 32666487 | 3.41E-09 | reported | 2872 | 0.04358 | rs114170505 |  |  |  |  |  |  |  |  |  |  |  |  |  |  |  |
| -0.213 | 2 | igd | 0.184 | C | \|\| id:ebi-a-GCST90002102 | ebi-a-GCST90002102 | TRUE | T | 39127247 | 1.71E-10 | reported | 2919 | 0.03324 | rs896251 |  |  |  |  |  |  |  |  |  |  |  |  |  |  |  |
| 0.1995 | 6 | igd | 0.1839 | A | \|\| id:ebi-a-GCST90001755 | ebi-a-GCST90001755 | TRUE | G | 31753526 | 4.32E-08 | reported | 3657 | 0.03634 | rs11751198 |  |  |  |  |  |  |  |  |  |  |  |  |  |  |  |
| 0.2182 | 6 | igd | 0.1839 | A | \|\| id:ebi-a-GCST90001761 | ebi-a-GCST90001761 | TRUE | G | 31753526 | 4.52E-09 | reported | 3657 | 0.03712 | rs11751198 |  |  |  |  |  |  |  |  |  |  |  |  |  |  |  |
| -0.2033 | 1 | igd | 0.1835 | G | \|\| id:ebi-a-GCST90001963 | ebi-a-GCST90001963 | TRUE | A | 161306465 | 1.36E-08 | reported | 2872 | 0.03569 | rs55864322 |  |  |  |  |  |  |  |  |  |  |  |  |  |  |  |
| 0.1864 | 9 | igd | 0.1835 | C | \|\| id:ebi-a-GCST90001980 | ebi-a-GCST90001980 | TRUE | T | 95889160 | 4.55E-09 | reported | 3629 | 0.03172 | rs60629714 |  |  |  |  |  |  |  |  |  |  |  |  |  |  |  |
| -0.2238 | 10 | igd | 0.1834 | G | \|\| id:ebi-a-GCST90001886 | ebi-a-GCST90001886 | TRUE | T | 97366817 | 7.89E-11 | reported | 2920 | 0.03429 | rs11592809 |  |  |  |  |  |  |  |  |  |  |  |  |  |  |  |
| -0.2913 | 10 | igd | 0.1834 | G | \|\| id:ebi-a-GCST90001888 | ebi-a-GCST90001888 | TRUE | T | 97366817 | 9.62E-17 | reported | 2920 | 0.03485 | rs11592809 |  |  |  |  |  |  |  |  |  |  |  |  |  |  |  |
| -0.388 | 10 | igd | 0.1834 | G | \|\| id:ebi-a-GCST90001892 | ebi-a-GCST90001892 | TRUE | T | 97366817 | 1.03E-27 | reported | 2920 | 0.0352 | rs11592809 |  |  |  |  |  |  |  |  |  |  |  |  |  |  |  |
| 0.4189 | 10 | igd | 0.1834 | G | \|\| id:ebi-a-GCST90001961 | ebi-a-GCST90001961 | TRUE | T | 97366817 | 1.52E-31 | reported | 2920 | 0.03543 | rs11592809 |  |  |  |  |  |  |  |  |  |  |  |  |  |  |  |
| -0.6897 | 10 | igd | 0.1834 | G | \|\| id:ebi-a-GCST90002030 | ebi-a-GCST90002030 | TRUE | T | 97366817 | 1.10E-87 | reported | 2920 | 0.03358 | rs11592809 |  |  |  |  |  |  |  |  |  |  |  |  |  |  |  |
| -0.5757 | 10 | igd | 0.1834 | G | \|\| id:ebi-a-GCST90002031 | ebi-a-GCST90002031 | TRUE | T | 97366817 | 7.05E-58 | reported | 2920 | 0.03511 | rs11592809 |  |  |  |  |  |  |  |  |  |  |  |  |  |  |  |
| -0.5946 | 10 | igd | 0.1834 | G | \|\| id:ebi-a-GCST90002032 | ebi-a-GCST90002032 | TRUE | T | 97366817 | 8.01E-62 | reported | 2920 | 0.03499 | rs11592809 |  |  |  |  |  |  |  |  |  |  |  |  |  |  |  |
| -0.2216 | 10 | igd | 0.1834 | G | \|\| id:ebi-a-GCST90002069 | ebi-a-GCST90002069 | TRUE | T | 97366817 | 5.76E-10 | reported | 2920 | 0.03564 | rs11592809 |  |  |  |  |  |  |  |  |  |  |  |  |  |  |  |
| -0.259 | 22 | igd | 0.1831 | C | \|\| id:ebi-a-GCST90001702 | ebi-a-GCST90001702 | TRUE | T | 40355729 | 2.95E-15 | reported | 3657 | 0.03268 | rs118033792 |  |  |  |  |  |  |  |  |  |  |  |  |  |  |  |
| -0.2627 | 22 | igd | 0.1831 | C | \|\| id:ebi-a-GCST90001703 | ebi-a-GCST90001703 | TRUE | T | 40355729 | 1.17E-15 | reported | 3657 | 0.03266 | rs118033792 |  |  |  |  |  |  |  |  |  |  |  |  |  |  |  |
| -0.2565 | 22 | igd | 0.1831 | C | \|\| id:ebi-a-GCST90001704 | ebi-a-GCST90001704 | TRUE | T | 40355729 | 5.07E-15 | reported | 3657 | 0.03263 | rs118033792 |  |  |  |  |  |  |  |  |  |  |  |  |  |  |  |
| -0.227 | 22 | igd | 0.1831 | C | \|\| id:ebi-a-GCST90001710 | ebi-a-GCST90001710 | TRUE | T | 40355729 | 4.20E-12 | reported | 3657 | 0.03265 | rs118033792 |  |  |  |  |  |  |  |  |  |  |  |  |  |  |  |
| -0.2316 | 22 | igd | 0.1831 | C | \|\| id:ebi-a-GCST90001711 | ebi-a-GCST90001711 | TRUE | T | 40355729 | 1.36E-12 | reported | 3657 | 0.03256 | rs118033792 |  |  |  |  |  |  |  |  |  |  |  |  |  |  |  |
| -0.2218 | 1 | igd | 0.1827 | A | \|\| id:ebi-a-GCST90001467 | ebi-a-GCST90001467 | TRUE | G | 161301818 | 1.22E-11 | reported | 3410 | 0.03262 | rs36059328 |  |  |  |  |  |  |  |  |  |  |  |  |  |  |  |
| 0.2507 | 10 | igd | 0.1827 | C | \|\| id:ebi-a-GCST90002061 | ebi-a-GCST90002061 | TRUE | G | 98152689 | 6.16E-12 | reported | 2920 | 0.03631 | rs35132272 |  |  |  |  |  |  |  |  |  |  |  |  |  |  |  |
| -0.2219 | 6 | igd | 0.1827 | T | \|\| id:ebi-a-GCST90002121 | ebi-a-GCST90002121 | TRUE | C | 30994024 | 6.70E-09 | reported | 2915 | 0.03816 | rs552487972 |  |  |  |  |  |  |  |  |  |  |  |  |  |  |  |
| -0.2044 | 10 | igd | 0.1825 | G | \|\| id:ebi-a-GCST90002030 | ebi-a-GCST90002030 | TRUE | A | 101413863 | 1.18E-08 | reported | 2920 | 0.03574 | rs2038563 |  |  |  |  |  |  |  |  |  |  |  |  |  |  |  |
| 0.2308 | 10 | igd | 0.1824 | G | \|\| id:ebi-a-GCST90002030 | ebi-a-GCST90002030 | TRUE | A | 96634439 | 1.33E-10 | reported | 2920 | 0.0358 | rs61888362 |  |  |  |  |  |  |  |  |  |  |  |  |  |  |  |
| -0.1962 | 1 | igd | 0.1821 | T | \|\| id:ebi-a-GCST90002002 | ebi-a-GCST90002002 | TRUE | C | 161278778 | 1.36E-09 | reported | 3629 | 0.03229 | rs72714962 |  |  |  |  |  |  |  |  |  |  |  |  |  |  |  |
| -0.299 | 19 | igd | 0.1816 | A | \|\| id:ebi-a-GCST90001948 | ebi-a-GCST90001948 | TRUE | G | 51594078 | 7.92E-11 | reported | 1633 | 0.04568 | rs34368430 |  |  |  |  |  |  |  |  |  |  |  |  |  |  |  |
| -0.2753 | 19 | igd | 0.1815 | A | \|\| id:ebi-a-GCST90001946 | ebi-a-GCST90001946 | TRUE | G | 51594078 | 2.82E-09 | reported | 1634 | 0.04607 | rs34368430 |  |  |  |  |  |  |  |  |  |  |  |  |  |  |  |
| -0.2819 | 19 | igd | 0.1815 | A | \|\| id:ebi-a-GCST90001949 | ebi-a-GCST90001949 | TRUE | G | 51594078 | 8.38E-10 | reported | 1634 | 0.04566 | rs34368430 |  |  |  |  |  |  |  |  |  |  |  |  |  |  |  |
| 0.2416 | 6 | igd | 0.1815 | A | \|\| id:ebi-a-GCST90002081 | ebi-a-GCST90002081 | TRUE | G | 32343979 | 7.66E-11 | reported | 3113 | 0.03699 | rs17202190 |  |  |  |  |  |  |  |  |  |  |  |  |  |  |  |
| 0.2083 | 6 | igd | 0.1813 | T | \|\| id:ebi-a-GCST90001896 | ebi-a-GCST90001896 | TRUE | C | 91080200 | 3.80E-09 | reported | 2920 | 0.03523 | rs7741944 |  |  |  |  |  |  |  |  |  |  |  |  |  |  |  |
| 0.2138 | 6 | igd | 0.181 | C | \|\| id:ebi-a-GCST90001692 | ebi-a-GCST90001692 | TRUE | G | 32214901 | 1.96E-08 | reported | 3440 | 0.03799 | rs146727227 |  |  |  |  |  |  |  |  |  |  |  |  |  |  |  |
| -0.2441 | 6 | igd | 0.1808 | A | \|\| id:ebi-a-GCST90001932 | ebi-a-GCST90001932 | TRUE | G | 32343979 | 1.08E-10 | reported | 2920 | 0.03768 | rs17202190 |  |  |  |  |  |  |  |  |  |  |  |  |  |  |  |
| -0.2622 | 3 | igd | 0.1806 | C | \|\| id:ebi-a-GCST90001997 | ebi-a-GCST90001997 | TRUE | G | 38980559 | 1.58E-15 | reported | 3590 | 0.03274 | rs11129812 |  |  |  |  |  |  |  |  |  |  |  |  |  |  |  |
| -0.2472 | 3 | igd | 0.1806 | C | \|\| id:ebi-a-GCST90001995 | ebi-a-GCST90001995 | TRUE | G | 38980559 | 3.85E-14 | reported | 3590 | 0.03254 | rs11129812 |  |  |  |  |  |  |  |  |  |  |  |  |  |  |  |
| 0.2857 | 2 | igd | 0.1805 | A | \|\| id:ebi-a-GCST90001896 | ebi-a-GCST90001896 | TRUE | G | 204709936 | 3.11E-14 | reported | 2920 | 0.03744 | rs11887591 |  |  |  |  |  |  |  |  |  |  |  |  |  |  |  |
| -0.2697 | 6 | igd | 0.1805 | A | \|\| id:ebi-a-GCST90002119 | ebi-a-GCST90002119 | TRUE | T | 30763714 | 3.18E-11 | reported | 2920 | 0.04047 | rs3131062 |  |  |  |  |  |  |  |  |  |  |  |  |  |  |  |
| 0.2066 | 6 | igd | 0.1802 | A | \|\| id:ebi-a-GCST90001752 | ebi-a-GCST90001752 | TRUE | G | 32343979 | 7.56E-09 | reported | 3657 | 0.03568 | rs17202190 |  |  |  |  |  |  |  |  |  |  |  |  |  |  |  |
| 0.2084 | 6 | igd | 0.1802 | A | \|\| id:ebi-a-GCST90001757 | ebi-a-GCST90001757 | TRUE | G | 32343979 | 7.44E-09 | reported | 3657 | 0.03596 | rs17202190 |  |  |  |  |  |  |  |  |  |  |  |  |  |  |  |
| 0.2687 | 6 | igd | 0.1801 | G | \|\| id:ebi-a-GCST90002112 | ebi-a-GCST90002112 | TRUE | A | 33772761 | 2.83E-08 | reported | 1635 | 0.04817 | rs3763253 |  |  |  |  |  |  |  |  |  |  |  |  |  |  |  |
| -0.35 | 10 | igd | 0.1799 | C | \|\| id:ebi-a-GCST90002029 | ebi-a-GCST90002029 | TRUE | T | 97377901 | 1.02E-22 | reported | 2915 | 0.03539 | rs3793751 |  |  |  |  |  |  |  |  |  |  |  |  |  |  |  |
| 0.2378 | 6 | igd | 0.1799 | T | \|\| id:ebi-a-GCST90002081 | ebi-a-GCST90002081 | TRUE | C | 32238501 | 1.80E-10 | reported | 3113 | 0.03716 | rs9368709 |  |  |  |  |  |  |  |  |  |  |  |  |  |  |  |
| 0.2257 | 10 | igd | 0.1796 | G | \|\| id:ebi-a-GCST90001775 | ebi-a-GCST90001775 | TRUE | A | 6134869 | 1.39E-11 | reported | 3660 | 0.03328 | rs1571025 |  |  |  |  |  |  |  |  |  |  |  |  |  |  |  |
| 0.2137 | 10 | igd | 0.1796 | G | \|\| id:ebi-a-GCST90001794 | ebi-a-GCST90001794 | TRUE | A | 6134869 | 1.55E-10 | reported | 3658 | 0.03329 | rs1571025 |  |  |  |  |  |  |  |  |  |  |  |  |  |  |  |
| -0.1938 | 2 | igd | 0.1796 | A | \|\| id:ebi-a-GCST90002098 | ebi-a-GCST90002098 | TRUE | G | 39777712 | 1.71E-08 | reported | 2910 | 0.03428 | rs4670286 |  |  |  |  |  |  |  |  |  |  |  |  |  |  |  |
| 0.1879 | 22 | igd | 0.1795 | A | \|\| id:ebi-a-GCST90001702 | ebi-a-GCST90001702 | TRUE | C | 46348684 | 1.34E-08 | reported | 3657 | 0.033 | rs10448601 |  |  |  |  |  |  |  |  |  |  |  |  |  |  |  |
| 0.1876 | 22 | igd | 0.1795 | A | \|\| id:ebi-a-GCST90001704 | ebi-a-GCST90001704 | TRUE | C | 46348684 | 1.37E-08 | reported | 3657 | 0.03296 | rs10448601 |  |  |  |  |  |  |  |  |  |  |  |  |  |  |  |
| 0.1911 | 22 | igd | 0.1795 | A | \|\| id:ebi-a-GCST90001710 | ebi-a-GCST90001710 | TRUE | C | 46348684 | 6.87E-09 | reported | 3657 | 0.0329 | rs10448601 |  |  |  |  |  |  |  |  |  |  |  |  |  |  |  |
| 0.1807 | 22 | igd | 0.1795 | A | \|\| id:ebi-a-GCST90001711 | ebi-a-GCST90001711 | TRUE | C | 46348684 | 4.08E-08 | reported | 3657 | 0.03285 | rs10448601 |  |  |  |  |  |  |  |  |  |  |  |  |  |  |  |
| 0.2909 | 10 | igd | 0.1795 | G | \|\| id:ebi-a-GCST90001777 | ebi-a-GCST90001777 | TRUE | A | 6134869 | 5.19E-19 | reported | 3657 | 0.03247 | rs1571025 |  |  |  |  |  |  |  |  |  |  |  |  |  |  |  |
| 0.24 | 10 | igd | 0.1795 | G | \|\| id:ebi-a-GCST90001778 | ebi-a-GCST90001778 | TRUE | A | 6134869 | 3.00E-13 | reported | 3657 | 0.03277 | rs1571025 |  |  |  |  |  |  |  |  |  |  |  |  |  |  |  |
| 0.2741 | 10 | igd | 0.1795 | G | \|\| id:ebi-a-GCST90001779 | ebi-a-GCST90001779 | TRUE | A | 6134869 | 1.39E-16 | reported | 3657 | 0.033 | rs1571025 |  |  |  |  |  |  |  |  |  |  |  |  |  |  |  |
| 0.2356 | 10 | igd | 0.1795 | G | \|\| id:ebi-a-GCST90001784 | ebi-a-GCST90001784 | TRUE | A | 6134869 | 1.88E-12 | reported | 3657 | 0.03333 | rs1571025 |  |  |  |  |  |  |  |  |  |  |  |  |  |  |  |
| 0.227 | 10 | igd | 0.1795 | G | \|\| id:ebi-a-GCST90001787 | ebi-a-GCST90001787 | TRUE | A | 6134869 | 5.23E-12 | reported | 3657 | 0.0328 | rs1571025 |  |  |  |  |  |  |  |  |  |  |  |  |  |  |  |
| 0.2933 | 10 | igd | 0.1795 | G | \|\| id:ebi-a-GCST90001789 | ebi-a-GCST90001789 | TRUE | A | 6134869 | 1.50E-19 | reported | 3657 | 0.03224 | rs1571025 |  |  |  |  |  |  |  |  |  |  |  |  |  |  |  |
| 0.2902 | 10 | igd | 0.1795 | G | \|\| id:ebi-a-GCST90001790 | ebi-a-GCST90001790 | TRUE | A | 6134869 | 6.54E-19 | reported | 3657 | 0.03249 | rs1571025 |  |  |  |  |  |  |  |  |  |  |  |  |  |  |  |
| 0.2525 | 10 | igd | 0.1795 | G | \|\| id:ebi-a-GCST90001791 | ebi-a-GCST90001791 | TRUE | A | 6134869 | 4.39E-14 | reported | 3657 | 0.03331 | rs1571025 |  |  |  |  |  |  |  |  |  |  |  |  |  |  |  |
| 0.2586 | 10 | igd | 0.1795 | G | \|\| id:ebi-a-GCST90001792 | ebi-a-GCST90001792 | TRUE | A | 6134869 | 2.72E-15 | reported | 3657 | 0.03258 | rs1571025 |  |  |  |  |  |  |  |  |  |  |  |  |  |  |  |
| 0.29 | 10 | igd | 0.1795 | G | \|\| id:ebi-a-GCST90001793 | ebi-a-GCST90001793 | TRUE | A | 6134869 | 1.31E-18 | reported | 3657 | 0.03276 | rs1571025 |  |  |  |  |  |  |  |  |  |  |  |  |  |  |  |
| 0.2201 | 6 | igd | 0.1794 | G | \|\| id:ebi-a-GCST90001692 | ebi-a-GCST90001692 | TRUE | A | 32329150 | 7.39E-09 | reported | 3440 | 0.03797 | rs137931178 |  |  |  |  |  |  |  |  |  |  |  |  |  |  |  |
| 0.2143 | 10 | igd | 0.1794 | G | \|\| id:ebi-a-GCST90001780 | ebi-a-GCST90001780 | TRUE | A | 6134869 | 8.92E-11 | reported | 3642 | 0.03295 | rs1571025 |  |  |  |  |  |  |  |  |  |  |  |  |  |  |  |
| 0.3017 | 10 | igd | 0.1793 | C | \|\| id:ebi-a-GCST90002030 | ebi-a-GCST90002030 | TRUE | T | 96267404 | 2.07E-16 | reported | 2920 | 0.0365 | rs56175117 |  |  |  |  |  |  |  |  |  |  |  |  |  |  |  |
| 0.2317 | 10 | igd | 0.1793 | C | \|\| id:ebi-a-GCST90002031 | ebi-a-GCST90002031 | TRUE | T | 96267404 | 5.61E-10 | reported | 2920 | 0.03724 | rs56175117 |  |  |  |  |  |  |  |  |  |  |  |  |  |  |  |
| 0.285 | 10 | igd | 0.1793 | C | \|\| id:ebi-a-GCST90002032 | ebi-a-GCST90002032 | TRUE | T | 96267404 | 2.23E-14 | reported | 2920 | 0.03713 | rs56175117 |  |  |  |  |  |  |  |  |  |  |  |  |  |  |  |
| -0.3628 | 10 | igd | 0.1793 | C | \|\| id:ebi-a-GCST90002061 | ebi-a-GCST90002061 | TRUE | A | 97372669 | 6.53E-23 | reported | 2920 | 0.03651 | rs1409710 |  |  |  |  |  |  |  |  |  |  |  |  |  |  |  |
| -0.2443 | 6 | igd | 0.1791 | T | \|\| id:ebi-a-GCST90001932 | ebi-a-GCST90001932 | TRUE | C | 32238501 | 1.23E-10 | reported | 2920 | 0.03782 | rs9368709 |  |  |  |  |  |  |  |  |  |  |  |  |  |  |  |
| 0.2047 | 6 | igd | 0.1787 | T | \|\| id:ebi-a-GCST90001752 | ebi-a-GCST90001752 | TRUE | C | 32238501 | 1.27E-08 | reported | 3657 | 0.03589 | rs9368709 |  |  |  |  |  |  |  |  |  |  |  |  |  |  |  |
| 0.2083 | 6 | igd | 0.1787 | T | \|\| id:ebi-a-GCST90001757 | ebi-a-GCST90001757 | TRUE | C | 32238501 | 9.16E-09 | reported | 3657 | 0.03618 | rs9368709 |  |  |  |  |  |  |  |  |  |  |  |  |  |  |  |
| 0.1962 | 2 | igd | 0.1779 | T | \|\| id:ebi-a-GCST90001891 | ebi-a-GCST90001891 | TRUE | C | 39003961 | 4.70E-08 | reported | 2920 | 0.03583 | rs59639671 |  |  |  |  |  |  |  |  |  |  |  |  |  |  |  |
| 0.2359 | 10 | igd | 0.1776 | T | \|\| id:ebi-a-GCST90002032 | ebi-a-GCST90002032 | TRUE | G | 96721181 | 2.40E-10 | reported | 2920 | 0.03712 | rs61886796 |  |  |  |  |  |  |  |  |  |  |  |  |  |  |  |
| -0.299 | 19 | igd | 0.177 | A | \|\| id:ebi-a-GCST90001956 | ebi-a-GCST90001956 | TRUE | G | 51594078 | 4.58E-10 | reported | 1579 | 0.04768 | rs34368430 |  |  |  |  |  |  |  |  |  |  |  |  |  |  |  |
| -0.235 | 3 | igd | 0.1768 | A | \|\| id:ebi-a-GCST90002036 | ebi-a-GCST90002036 | TRUE | G | 52877270 | 1.54E-10 | reported | 2871 | 0.03657 | rs11720228 |  |  |  |  |  |  |  |  |  |  |  |  |  |  |  |
| 0.2106 | 10 | igd | 0.1767 | C | \|\| id:ebi-a-GCST90001483 | ebi-a-GCST90001483 | TRUE | A | 96267414 | 8.37E-10 | reported | 3405 | 0.03421 | rs56259239 |  |  |  |  |  |  |  |  |  |  |  |  |  |  |  |
| 0.2284 | 10 | igd | 0.1767 | C | \|\| id:ebi-a-GCST90001489 | ebi-a-GCST90001489 | TRUE | A | 96267414 | 9.11E-11 | reported | 3405 | 0.03512 | rs56259239 |  |  |  |  |  |  |  |  |  |  |  |  |  |  |  |
| 0.2518 | 10 | igd | 0.1767 | C | \|\| id:ebi-a-GCST90001495 | ebi-a-GCST90001495 | TRUE | A | 96267414 | 1.81E-12 | reported | 3405 | 0.03559 | rs56259239 |  |  |  |  |  |  |  |  |  |  |  |  |  |  |  |
| -0.2856 | 10 | igd | 0.1766 | C | \|\| id:ebi-a-GCST90001660 | ebi-a-GCST90001660 | TRUE | T | 98206884 | 1.38E-16 | reported | 3408 | 0.03438 | rs12413182 |  |  |  |  |  |  |  |  |  |  |  |  |  |  |  |
| 0.2757 | 2 | igd | 0.1766 | A | \|\| id:ebi-a-GCST90002102 | ebi-a-GCST90002102 | TRUE | G | 39008949 | 5.10E-16 | reported | 2919 | 0.03381 | rs1056104 |  |  |  |  |  |  |  |  |  |  |  |  |  |  |  |
| 0.2219 | 10 | igd | 0.1765 | C | \|\| id:ebi-a-GCST90001484 | ebi-a-GCST90001484 | TRUE | T | 96267404 | 6.55E-11 | reported | 3437 | 0.03387 | rs56175117 |  |  |  |  |  |  |  |  |  |  |  |  |  |  |  |
| 0.2055 | 10 | igd | 0.1765 | C | \|\| id:ebi-a-GCST90001485 | ebi-a-GCST90001485 | TRUE | A | 96267414 | 1.42E-09 | reported | 3437 | 0.03385 | rs56259239 |  |  |  |  |  |  |  |  |  |  |  |  |  |  |  |
| 0.2007 | 10 | igd | 0.1765 | C | \|\| id:ebi-a-GCST90001491 | ebi-a-GCST90001491 | TRUE | T | 96267404 | 9.45E-09 | reported | 3437 | 0.03487 | rs56175117 |  |  |  |  |  |  |  |  |  |  |  |  |  |  |  |
| 0.2322 | 10 | igd | 0.1765 | C | \|\| id:ebi-a-GCST90001496 | ebi-a-GCST90001496 | TRUE | A | 96267414 | 5.44E-11 | reported | 3437 | 0.03529 | rs56259239 |  |  |  |  |  |  |  |  |  |  |  |  |  |  |  |
| 0.2351 | 10 | igd | 0.1765 | C | \|\| id:ebi-a-GCST90001497 | ebi-a-GCST90001497 | TRUE | A | 96267414 | 1.74E-11 | reported | 3437 | 0.03483 | rs56259239 |  |  |  |  |  |  |  |  |  |  |  |  |  |  |  |
| 0.2622 | 10 | igd | 0.1765 | C | \|\| id:ebi-a-GCST90001660 | ebi-a-GCST90001660 | TRUE | A | 96267414 | 1.26E-13 | reported | 3408 | 0.03524 | rs56259239 |  |  |  |  |  |  |  |  |  |  |  |  |  |  |  |
| -0.298 | 19 | igd | 0.1765 | A | \|\| id:ebi-a-GCST90001957 | ebi-a-GCST90001957 | TRUE | G | 51594078 | 6.04E-10 | reported | 1578 | 0.04785 | rs34368430 |  |  |  |  |  |  |  |  |  |  |  |  |  |  |  |
| 0.2413 | 10 | igd | 0.1763 | C | \|\| id:ebi-a-GCST90001658 | ebi-a-GCST90001658 | TRUE | T | 96267404 | 9.62E-13 | reported | 3440 | 0.03369 | rs56175117 |  |  |  |  |  |  |  |  |  |  |  |  |  |  |  |
| 0.2607 | 10 | igd | 0.1763 | C | \|\| id:ebi-a-GCST90001659 | ebi-a-GCST90001659 | TRUE | T | 96267404 | 1.23E-14 | reported | 3440 | 0.03365 | rs56175117 |  |  |  |  |  |  |  |  |  |  |  |  |  |  |  |
| 0.229 | 2 | igd | 0.1761 | T | \|\| id:ebi-a-GCST90001507 | ebi-a-GCST90001507 | TRUE | C | 39003961 | 3.59E-11 | reported | 3405 | 0.03448 | rs59639671 |  |  |  |  |  |  |  |  |  |  |  |  |  |  |  |
| -0.372 | 1 | igd | 0.176 | T | \|\| id:ebi-a-GCST90001970 | ebi-a-GCST90001970 | TRUE | C | 161278778 | 1.83E-25 | reported | 2971 | 0.03534 | rs72714962 |  |  |  |  |  |  |  |  |  |  |  |  |  |  |  |
| -0.1815 | 10 | igd | 0.1759 | G | \|\| id:ebi-a-GCST90001659 | ebi-a-GCST90001659 | TRUE | A | 101413863 | 4.87E-08 | reported | 3440 | 0.03319 | rs2038563 |  |  |  |  |  |  |  |  |  |  |  |  |  |  |  |
| 0.2669 | 2 | igd | 0.1756 | T | \|\| id:ebi-a-GCST90001508 | ebi-a-GCST90001508 | TRUE | C | 39003961 | 3.99E-15 | reported | 3437 | 0.03382 | rs59639671 |  |  |  |  |  |  |  |  |  |  |  |  |  |  |  |
| 0.2386 | 2 | igd | 0.1756 | T | \|\| id:ebi-a-GCST90001509 | ebi-a-GCST90001509 | TRUE | C | 39003961 | 2.22E-12 | reported | 3437 | 0.03386 | rs59639671 |  |  |  |  |  |  |  |  |  |  |  |  |  |  |  |
| -0.2881 | 19 | igd | 0.1755 | A | \|\| id:ebi-a-GCST90001953 | ebi-a-GCST90001953 | TRUE | G | 51594078 | 2.62E-09 | reported | 1558 | 0.0481 | rs34368430 |  |  |  |  |  |  |  |  |  |  |  |  |  |  |  |
| -0.3069 | 19 | igd | 0.1754 | A | \|\| id:ebi-a-GCST90001955 | ebi-a-GCST90001955 | TRUE | G | 51594078 | 2.69E-10 | reported | 1554 | 0.04827 | rs34368430 |  |  |  |  |  |  |  |  |  |  |  |  |  |  |  |
| -0.202 | 4 | igd | 0.1753 | G | \|\| id:ebi-a-GCST90001815 | ebi-a-GCST90001815 | TRUE | A | 15899742 | 2.09E-10 | reported | 3657 | 0.03169 | rs11731825 |  |  |  |  |  |  |  |  |  |  |  |  |  |  |  |
| -0.2994 | 19 | igd | 0.1752 | A | \|\| id:ebi-a-GCST90001954 | ebi-a-GCST90001954 | TRUE | G | 51594078 | 6.55E-10 | reported | 1555 | 0.04817 | rs34368430 |  |  |  |  |  |  |  |  |  |  |  |  |  |  |  |
| -0.1913 | 10 | igd | 0.1749 | A | \|\| id:ebi-a-GCST90001483 | ebi-a-GCST90001483 | TRUE | T | 98210930 | 1.94E-08 | reported | 3405 | 0.03398 | rs17111879 |  |  |  |  |  |  |  |  |  |  |  |  |  |  |  |
| -0.2629 | 10 | igd | 0.1749 | A | \|\| id:ebi-a-GCST90001495 | ebi-a-GCST90001495 | TRUE | T | 98210930 | 9.38E-14 | reported | 3405 | 0.03515 | rs17111879 |  |  |  |  |  |  |  |  |  |  |  |  |  |  |  |
| -0.2153 | 10 | igd | 0.1747 | A | \|\| id:ebi-a-GCST90002031 | ebi-a-GCST90002031 | TRUE | T | 98210930 | 1.10E-08 | reported | 2920 | 0.03757 | rs17111879 |  |  |  |  |  |  |  |  |  |  |  |  |  |  |  |
| -0.2782 | 10 | igd | 0.1746 | A | \|\| id:ebi-a-GCST90001658 | ebi-a-GCST90001658 | TRUE | T | 98210930 | 5.80E-17 | reported | 3440 | 0.03307 | rs17111879 |  |  |  |  |  |  |  |  |  |  |  |  |  |  |  |
| -0.2826 | 10 | igd | 0.1746 | A | \|\| id:ebi-a-GCST90001659 | ebi-a-GCST90001659 | TRUE | T | 98210930 | 1.89E-17 | reported | 3440 | 0.03306 | rs17111879 |  |  |  |  |  |  |  |  |  |  |  |  |  |  |  |
| -0.2329 | 10 | igd | 0.1746 | A | \|\| id:ebi-a-GCST90001671 | ebi-a-GCST90001671 | TRUE | T | 98210930 | 1.93E-11 | reported | 3440 | 0.03459 | rs17111879 |  |  |  |  |  |  |  |  |  |  |  |  |  |  |  |
| -0.2352 | 20 | igd | 0.1746 | A | \|\| id:ebi-a-GCST90001802 | ebi-a-GCST90001802 | TRUE | G | 44757407 | 7.20E-13 | reported | 3657 | 0.03266 | rs3765459 |  |  |  |  |  |  |  |  |  |  |  |  |  |  |  |
| -0.2536 | 20 | igd | 0.1746 | A | \|\| id:ebi-a-GCST90001805 | ebi-a-GCST90001805 | TRUE | G | 44757407 | 1.05E-14 | reported | 3657 | 0.03265 | rs3765459 |  |  |  |  |  |  |  |  |  |  |  |  |  |  |  |
| -0.2097 | 10 | igd | 0.1744 | A | \|\| id:ebi-a-GCST90001484 | ebi-a-GCST90001484 | TRUE | T | 98210930 | 5.13E-10 | reported | 3437 | 0.03365 | rs17111879 |  |  |  |  |  |  |  |  |  |  |  |  |  |  |  |
| -0.1857 | 10 | igd | 0.1744 | A | \|\| id:ebi-a-GCST90001485 | ebi-a-GCST90001485 | TRUE | T | 98210930 | 3.62E-08 | reported | 3437 | 0.03364 | rs17111879 |  |  |  |  |  |  |  |  |  |  |  |  |  |  |  |
| -0.2334 | 10 | igd | 0.1744 | A | \|\| id:ebi-a-GCST90001496 | ebi-a-GCST90001496 | TRUE | T | 98210930 | 2.35E-11 | reported | 3437 | 0.03481 | rs17111879 |  |  |  |  |  |  |  |  |  |  |  |  |  |  |  |
| 0.4111 | 19 | igd | 0.1741 | T | \|\| id:ebi-a-GCST90001955 | ebi-a-GCST90001955 | TRUE | C | 51739227 | 3.34E-17 | reported | 1554 | 0.04817 | rs146995981 |  |  |  |  |  |  |  |  |  |  |  |  |  |  |  |
| 0.3917 | 19 | igd | 0.174 | T | \|\| id:ebi-a-GCST90001954 | ebi-a-GCST90001954 | TRUE | C | 51739227 | 8.78E-16 | reported | 1555 | 0.04819 | rs146995981 |  |  |  |  |  |  |  |  |  |  |  |  |  |  |  |
| -0.2659 | 10 | igd | 0.1739 | A | \|\| id:ebi-a-GCST90001489 | ebi-a-GCST90001489 | TRUE | G | 98206115 | 3.16E-14 | reported | 3405 | 0.03487 | rs11188758 |  |  |  |  |  |  |  |  |  |  |  |  |  |  |  |
| -0.2143 | 16 | igd | 0.1737 | T | \|\| id:ebi-a-GCST90001964 | ebi-a-GCST90001964 | TRUE | C | 89045233 | 5.27E-09 | reported | 2872 | 0.0366 | rs62045817 |  |  |  |  |  |  |  |  |  |  |  |  |  |  |  |
| 0.2057 | 2 | igd | 0.1737 | G | \|\| id:ebi-a-GCST90002102 | ebi-a-GCST90002102 | TRUE | A | 41899246 | 2.46E-09 | reported | 2919 | 0.03438 | rs4952496 |  |  |  |  |  |  |  |  |  |  |  |  |  |  |  |
| 0.3807 | 19 | igd | 0.1736 | T | \|\| id:ebi-a-GCST90001953 | ebi-a-GCST90001953 | TRUE | C | 51739227 | 5.53E-15 | reported | 1558 | 0.04823 | rs146995981 |  |  |  |  |  |  |  |  |  |  |  |  |  |  |  |
| -0.2648 | 10 | igd | 0.1736 | A | \|\| id:ebi-a-GCST90002030 | ebi-a-GCST90002030 | TRUE | G | 98206115 | 7.84E-13 | reported | 2920 | 0.03679 | rs11188758 |  |  |  |  |  |  |  |  |  |  |  |  |  |  |  |
| -0.2279 | 16 | igd | 0.1736 | G | \|\| id:ebi-a-GCST90002088 | ebi-a-GCST90002088 | TRUE | A | 31453496 | 7.75E-11 | reported | 2872 | 0.03489 | rs113634117 |  |  |  |  |  |  |  |  |  |  |  |  |  |  |  |
| 0.5108 | 1 | igd | 0.1735 | G | \|\| id:ebi-a-GCST90001994 | ebi-a-GCST90001994 | TRUE | C | 161510516 | 5.51E-57 | reported | 3590 | 0.03154 | rs144126567 |  |  |  |  |  |  |  |  |  |  |  |  |  |  |  |
| -0.2381 | 10 | igd | 0.1734 | A | \|\| id:ebi-a-GCST90001491 | ebi-a-GCST90001491 | TRUE | G | 98206115 | 5.52E-12 | reported | 3437 | 0.03443 | rs11188758 |  |  |  |  |  |  |  |  |  |  |  |  |  |  |  |
| 0.2147 | 2 | igd | 0.1734 | G | \|\| id:ebi-a-GCST90002098 | ebi-a-GCST90002098 | TRUE | A | 41899246 | 8.09E-10 | reported | 2910 | 0.03483 | rs4952496 |  |  |  |  |  |  |  |  |  |  |  |  |  |  |  |
| 0.2415 | 6 | igd | 0.1726 | A | \|\| id:ebi-a-GCST90002067 | ebi-a-GCST90002067 | TRUE | T | 31383130 | 3.03E-08 | reported | 2920 | 0.04348 | rs9266834 |  |  |  |  |  |  |  |  |  |  |  |  |  |  |  |
| -0.2225 | 1 | igd | 0.1724 | C | \|\| id:ebi-a-GCST90001471 | ebi-a-GCST90001471 | TRUE | T | 161615395 | 8.20E-12 | reported | 3410 | 0.03243 | rs56091027 |  |  |  |  |  |  |  |  |  |  |  |  |  |  |  |
| -0.4487 | 6 | igd | 0.1724 | C | \|\| id:ebi-a-GCST90002105 | ebi-a-GCST90002105 | TRUE | G | 32520731 | 1.39E-11 | reported | 2872 | 0.06613 | rs57658648 |  |  |  |  |  |  |  |  |  |  |  |  |  |  |  |
| 0.3828 | 19 | igd | 0.1721 | T | \|\| id:ebi-a-GCST90001952 | ebi-a-GCST90001952 | TRUE | C | 51739227 | 4.97E-15 | reported | 1569 | 0.04842 | rs146995981 |  |  |  |  |  |  |  |  |  |  |  |  |  |  |  |
| 0.2691 | 1 | igd | 0.1721 | G | \|\| id:ebi-a-GCST90001982 | ebi-a-GCST90001982 | TRUE | C | 161510516 | 2.01E-16 | reported | 3621 | 0.03257 | rs144126567 |  |  |  |  |  |  |  |  |  |  |  |  |  |  |  |
| 0.2853 | 3 | igd | 0.172 | G | \|\| id:ebi-a-GCST90002014 | ebi-a-GCST90002014 | TRUE | A | 46334167 | 1.25E-14 | reported | 2870 | 0.0368 | rs4683201 |  |  |  |  |  |  |  |  |  |  |  |  |  |  |  |
| 0.2214 | 4 | igd | 0.1716 | T | \|\| id:ebi-a-GCST90001470 | ebi-a-GCST90001470 | TRUE | C | 84151064 | 7.58E-12 | reported | 3374 | 0.03223 | rs17352615 |  |  |  |  |  |  |  |  |  |  |  |  |  |  |  |
| 0.3121 | 19 | igd | 0.1716 | T | \|\| id:ebi-a-GCST90001950 | ebi-a-GCST90001950 | TRUE | C | 51739227 | 9.49E-09 | reported | 1244 | 0.05401 | rs146995981 |  |  |  |  |  |  |  |  |  |  |  |  |  |  |  |
| 0.4073 | 19 | igd | 0.1715 | T | \|\| id:ebi-a-GCST90001947 | ebi-a-GCST90001947 | TRUE | C | 51739227 | 7.63E-17 | reported | 1577 | 0.04831 | rs146995981 |  |  |  |  |  |  |  |  |  |  |  |  |  |  |  |
| 0.4448 | 19 | igd | 0.1714 | T | \|\| id:ebi-a-GCST90001957 | ebi-a-GCST90001957 | TRUE | C | 51739227 | 7.22E-20 | reported | 1578 | 0.04809 | rs146995981 |  |  |  |  |  |  |  |  |  |  |  |  |  |  |  |
| 0.4375 | 19 | igd | 0.1713 | T | \|\| id:ebi-a-GCST90001956 | ebi-a-GCST90001956 | TRUE | C | 51739227 | 2.53E-19 | reported | 1579 | 0.04805 | rs146995981 |  |  |  |  |  |  |  |  |  |  |  |  |  |  |  |
| 0.1995 | 2 | igd | 0.1712 | A | \|\| id:ebi-a-GCST90001887 | ebi-a-GCST90001887 | TRUE | G | 38935236 | 3.17E-08 | reported | 2920 | 0.03596 | rs34460712 |  |  |  |  |  |  |  |  |  |  |  |  |  |  |  |
| 0.2293 | 4 | igd | 0.1711 | T | \|\| id:ebi-a-GCST90001471 | ebi-a-GCST90001471 | TRUE | C | 84151064 | 3.58E-12 | reported | 3410 | 0.03286 | rs17352615 |  |  |  |  |  |  |  |  |  |  |  |  |  |  |  |
| 0.4937 | 6 | igd | 0.1706 | A | \|\| id:ebi-a-GCST90002112 | ebi-a-GCST90002112 | TRUE | C | 32614419 | 8.77E-12 | reported | 1635 | 0.0718 | rs72852237 |  |  |  |  |  |  |  |  |  |  |  |  |  |  |  |
| -0.2307 | 6 | igd | 0.1704 | G | \|\| id:ebi-a-GCST90002119 | ebi-a-GCST90002119 | TRUE | C | 32130329 | 6.25E-09 | reported | 2920 | 0.03959 | rs34129552 |  |  |  |  |  |  |  |  |  |  |  |  |  |  |  |
| 0.2104 | 10 | igd | 0.1702 | C | \|\| id:ebi-a-GCST90001495 | ebi-a-GCST90001495 | TRUE | T | 97278123 | 1.75E-09 | reported | 3405 | 0.03486 | rs943347 |  |  |  |  |  |  |  |  |  |  |  |  |  |  |  |
| -0.2655 | 22 | igd | 0.1695 | A | \|\| id:ebi-a-GCST90001702 | ebi-a-GCST90001702 | TRUE | G | 43572420 | 3.88E-15 | reported | 3657 | 0.03364 | rs9612019 |  |  |  |  |  |  |  |  |  |  |  |  |  |  |  |
| -0.2559 | 22 | igd | 0.1695 | A | \|\| id:ebi-a-GCST90001703 | ebi-a-GCST90001703 | TRUE | G | 43572420 | 3.60E-14 | reported | 3657 | 0.03365 | rs9612019 |  |  |  |  |  |  |  |  |  |  |  |  |  |  |  |
| -0.2432 | 22 | igd | 0.1695 | A | \|\| id:ebi-a-GCST90001704 | ebi-a-GCST90001704 | TRUE | G | 43572420 | 5.87E-13 | reported | 3657 | 0.03363 | rs9612019 |  |  |  |  |  |  |  |  |  |  |  |  |  |  |  |
| -0.2656 | 22 | igd | 0.1695 | A | \|\| id:ebi-a-GCST90001710 | ebi-a-GCST90001710 | TRUE | G | 43572420 | 3.19E-15 | reported | 3657 | 0.03355 | rs9612019 |  |  |  |  |  |  |  |  |  |  |  |  |  |  |  |
| -0.2668 | 22 | igd | 0.1695 | A | \|\| id:ebi-a-GCST90001711 | ebi-a-GCST90001711 | TRUE | G | 43572420 | 2.02E-15 | reported | 3657 | 0.03346 | rs9612019 |  |  |  |  |  |  |  |  |  |  |  |  |  |  |  |
| -0.1904 | 20 | igd | 0.1694 | C | \|\| id:ebi-a-GCST90001798 | ebi-a-GCST90001798 | TRUE | G | 44828738 | 1.09E-08 | reported | 3657 | 0.03324 | rs112971251 |  |  |  |  |  |  |  |  |  |  |  |  |  |  |  |
| 0.1978 | 10 | igd | 0.1693 | A | \|\| id:ebi-a-GCST90001660 | ebi-a-GCST90001660 | TRUE | G | 97269577 | 1.13E-08 | reported | 3408 | 0.03456 | rs4917694 |  |  |  |  |  |  |  |  |  |  |  |  |  |  |  |
| 0.27 | 19 | igd | 0.1693 | A | \|\| id:ebi-a-GCST90001947 | ebi-a-GCST90001947 | TRUE | G | 51683579 | 3.44E-08 | reported | 1577 | 0.04869 | rs62115989 |  |  |  |  |  |  |  |  |  |  |  |  |  |  |  |
| -0.4221 | 19 | igd | 0.1693 | C | \|\| id:ebi-a-GCST90001948 | ebi-a-GCST90001948 | TRUE | T | 51594543 | 1.18E-18 | reported | 1633 | 0.0473 | rs7343094 |  |  |  |  |  |  |  |  |  |  |  |  |  |  |  |
| 0.2234 | 10 | igd | 0.1692 | A | \|\| id:ebi-a-GCST90001496 | ebi-a-GCST90001496 | TRUE | G | 97269577 | 1.14E-10 | reported | 3437 | 0.03454 | rs4917694 |  |  |  |  |  |  |  |  |  |  |  |  |  |  |  |
| 0.2117 | 10 | igd | 0.1692 | A | \|\| id:ebi-a-GCST90001497 | ebi-a-GCST90001497 | TRUE | G | 97269577 | 6.19E-10 | reported | 3437 | 0.03412 | rs4917694 |  |  |  |  |  |  |  |  |  |  |  |  |  |  |  |
| -0.4197 | 19 | igd | 0.1692 | C | \|\| id:ebi-a-GCST90001946 | ebi-a-GCST90001946 | TRUE | T | 51594543 | 2.55E-18 | reported | 1634 | 0.04751 | rs7343094 |  |  |  |  |  |  |  |  |  |  |  |  |  |  |  |
| -0.3942 | 19 | igd | 0.1692 | C | \|\| id:ebi-a-GCST90001949 | ebi-a-GCST90001949 | TRUE | T | 51594543 | 1.68E-16 | reported | 1634 | 0.04732 | rs7343094 |  |  |  |  |  |  |  |  |  |  |  |  |  |  |  |
| 0.2926 | 19 | igd | 0.1692 | A | \|\| id:ebi-a-GCST90001957 | ebi-a-GCST90001957 | TRUE | G | 51683579 | 1.90E-09 | reported | 1578 | 0.04843 | rs62115989 |  |  |  |  |  |  |  |  |  |  |  |  |  |  |  |
| 0.2903 | 19 | igd | 0.1691 | A | \|\| id:ebi-a-GCST90001956 | ebi-a-GCST90001956 | TRUE | G | 51683579 | 2.57E-09 | reported | 1579 | 0.04845 | rs62115989 |  |  |  |  |  |  |  |  |  |  |  |  |  |  |  |
| 0.2574 | 1 | igd | 0.1689 | A | \|\| id:ebi-a-GCST90001992 | ebi-a-GCST90001992 | TRUE | G | 161531340 | 4.42E-15 | reported | 3618 | 0.03268 | rs115383270 |  |  |  |  |  |  |  |  |  |  |  |  |  |  |  |
| -0.2444 | 10 | igd | 0.1688 | A | \|\| id:ebi-a-GCST90001961 | ebi-a-GCST90001961 | TRUE | G | 95166455 | 3.53E-11 | reported | 2920 | 0.03676 | rs1746489 |  |  |  |  |  |  |  |  |  |  |  |  |  |  |  |
| 0.2061 | 10 | igd | 0.1688 | A | \|\| id:ebi-a-GCST90002061 | ebi-a-GCST90002061 | TRUE | G | 95166455 | 3.02E-08 | reported | 2920 | 0.03709 | rs1746489 |  |  |  |  |  |  |  |  |  |  |  |  |  |  |  |
| -0.352 | 19 | igd | 0.1687 | C | \|\| id:ebi-a-GCST90001951 | ebi-a-GCST90001951 | TRUE | T | 51594543 | 7.39E-12 | reported | 1464 | 0.05097 | rs7343094 |  |  |  |  |  |  |  |  |  |  |  |  |  |  |  |
| -0.2287 | 10 | igd | 0.1685 | G | \|\| id:ebi-a-GCST90001670 | ebi-a-GCST90001670 | TRUE | T | 96701035 | 1.50E-10 | reported | 3440 | 0.03559 | rs113242967 |  |  |  |  |  |  |  |  |  |  |  |  |  |  |  |
| -0.2102 | 10 | igd | 0.1685 | G | \|\| id:ebi-a-GCST90001671 | ebi-a-GCST90001671 | TRUE | T | 96701035 | 4.40E-09 | reported | 3440 | 0.03572 | rs113242967 |  |  |  |  |  |  |  |  |  |  |  |  |  |  |  |
| -0.2256 | 10 | igd | 0.1683 | G | \|\| id:ebi-a-GCST90001672 | ebi-a-GCST90001672 | TRUE | T | 96701035 | 5.41E-10 | reported | 3408 | 0.03624 | rs113242967 |  |  |  |  |  |  |  |  |  |  |  |  |  |  |  |
| 0.3397 | 1 | igd | 0.1683 | A | \|\| id:ebi-a-GCST90002014 | ebi-a-GCST90002014 | TRUE | G | 161531340 | 7.20E-20 | reported | 2870 | 0.03695 | rs115383270 |  |  |  |  |  |  |  |  |  |  |  |  |  |  |  |
| -0.2933 | 1 | igd | 0.1683 | T | \|\| id:ebi-a-GCST90002013 | ebi-a-GCST90002013 | TRUE | C | 161289441 | 2.75E-15 | reported | 2870 | 0.03691 | rs66535289 |  |  |  |  |  |  |  |  |  |  |  |  |  |  |  |
| -0.2326 | 6 | igd | 0.1678 | T | \|\| id:ebi-a-GCST90002119 | ebi-a-GCST90002119 | TRUE | A | 29602717 | 6.57E-09 | reported | 2920 | 0.03997 | rs28749531 |  |  |  |  |  |  |  |  |  |  |  |  |  |  |  |
| -0.215 | 19 | igd | 0.1677 | T | \|\| id:ebi-a-GCST90002090 | ebi-a-GCST90002090 | TRUE | C | 52129097 | 6.42E-09 | reported | 2850 | 0.03692 | rs113861918 |  |  |  |  |  |  |  |  |  |  |  |  |  |  |  |
| -0.2854 | 12 | igd | 0.1672 | A | \|\| id:ebi-a-GCST90001801 | ebi-a-GCST90001801 | TRUE | G | 6554628 | 9.11E-10 | reported | 1836 | 0.04636 | rs25680 |  |  |  |  |  |  |  |  |  |  |  |  |  |  |  |
| -0.2616 | 12 | igd | 0.1671 | A | \|\| id:ebi-a-GCST90001798 | ebi-a-GCST90001798 | TRUE | G | 6554628 | 2.86E-15 | reported | 3657 | 0.03299 | rs25680 |  |  |  |  |  |  |  |  |  |  |  |  |  |  |  |
| -0.2558 | 12 | igd | 0.1671 | A | \|\| id:ebi-a-GCST90001802 | ebi-a-GCST90001802 | TRUE | G | 6554628 | 2.38E-14 | reported | 3657 | 0.0334 | rs25680 |  |  |  |  |  |  |  |  |  |  |  |  |  |  |  |
| -0.2122 | 12 | igd | 0.1671 | A | \|\| id:ebi-a-GCST90001804 | ebi-a-GCST90001804 | TRUE | G | 6554628 | 1.29E-10 | reported | 3657 | 0.03291 | rs25680 |  |  |  |  |  |  |  |  |  |  |  |  |  |  |  |
| -0.213 | 12 | igd | 0.1671 | A | \|\| id:ebi-a-GCST90001805 | ebi-a-GCST90001805 | TRUE | G | 6554628 | 2.13E-10 | reported | 3657 | 0.03344 | rs25680 |  |  |  |  |  |  |  |  |  |  |  |  |  |  |  |
| -0.3147 | 12 | igd | 0.1671 | A | \|\| id:ebi-a-GCST90001808 | ebi-a-GCST90001808 | TRUE | G | 6554628 | 3.76E-21 | reported | 3657 | 0.03313 | rs25680 |  |  |  |  |  |  |  |  |  |  |  |  |  |  |  |
| -0.3587 | 10 | igd | 0.167 | A | \|\| id:ebi-a-GCST90001489 | ebi-a-GCST90001489 | TRUE | G | 97707267 | 8.29E-25 | reported | 3405 | 0.0346 | rs113514451 |  |  |  |  |  |  |  |  |  |  |  |  |  |  |  |
| -0.1925 | 12 | igd | 0.167 | A | \|\| id:ebi-a-GCST90001800 | ebi-a-GCST90001800 | TRUE | G | 6554628 | 1.15E-08 | reported | 3656 | 0.03366 | rs25680 |  |  |  |  |  |  |  |  |  |  |  |  |  |  |  |
| -0.2612 | 12 | igd | 0.167 | A | \|\| id:ebi-a-GCST90001806 | ebi-a-GCST90001806 | TRUE | G | 6554628 | 6.60E-15 | reported | 3656 | 0.03338 | rs25680 |  |  |  |  |  |  |  |  |  |  |  |  |  |  |  |
| -0.2477 | 19 | igd | 0.1668 | T | \|\| id:ebi-a-GCST90001523 | ebi-a-GCST90001523 | TRUE | C | 51746918 | 3.06E-08 | reported | 1858 | 0.04454 | rs62115996 |  |  |  |  |  |  |  |  |  |  |  |  |  |  |  |
| 0.2459 | 19 | igd | 0.1668 | T | \|\| id:ebi-a-GCST90001531 | ebi-a-GCST90001531 | TRUE | C | 51739227 | 3.30E-08 | reported | 1856 | 0.04433 | rs146995981 |  |  |  |  |  |  |  |  |  |  |  |  |  |  |  |
| 0.2163 | 3 | igd | 0.1668 | A | \|\| id:ebi-a-GCST90001858 | ebi-a-GCST90001858 | TRUE | G | 46086145 | 5.14E-09 | reported | 2919 | 0.03691 | rs114289482 |  |  |  |  |  |  |  |  |  |  |  |  |  |  |  |
| -0.2073 | 3 | igd | 0.1665 | A | \|\| id:ebi-a-GCST90002036 | ebi-a-GCST90002036 | TRUE | C | 52997945 | 3.42E-08 | reported | 2871 | 0.03745 | rs35385050 |  |  |  |  |  |  |  |  |  |  |  |  |  |  |  |
| 0.4779 | 19 | igd | 0.166 | T | \|\| id:ebi-a-GCST90001948 | ebi-a-GCST90001948 | TRUE | C | 51739227 | 9.02E-24 | reported | 1633 | 0.0468 | rs146995981 |  |  |  |  |  |  |  |  |  |  |  |  |  |  |  |
| 0.4503 | 19 | igd | 0.1659 | T | \|\| id:ebi-a-GCST90001946 | ebi-a-GCST90001946 | TRUE | C | 51739227 | 5.15E-21 | reported | 1634 | 0.04722 | rs146995981 |  |  |  |  |  |  |  |  |  |  |  |  |  |  |  |
| 0.4639 | 19 | igd | 0.1659 | T | \|\| id:ebi-a-GCST90001949 | ebi-a-GCST90001949 | TRUE | C | 51739227 | 1.54E-22 | reported | 1634 | 0.04679 | rs146995981 |  |  |  |  |  |  |  |  |  |  |  |  |  |  |  |
| -0.3481 | 19 | igd | 0.1657 | C | \|\| id:ebi-a-GCST90001955 | ebi-a-GCST90001955 | TRUE | T | 51594543 | 2.82E-12 | reported | 1554 | 0.04942 | rs7343094 |  |  |  |  |  |  |  |  |  |  |  |  |  |  |  |
| 0.1943 | 2 | igd | 0.1656 | T | \|\| id:ebi-a-GCST90002098 | ebi-a-GCST90002098 | TRUE | C | 38741112 | 4.65E-08 | reported | 2910 | 0.03546 | rs77066249 |  |  |  |  |  |  |  |  |  |  |  |  |  |  |  |
| -0.2768 | 6 | igd | 0.1656 | G | \|\| id:ebi-a-GCST90002119 | ebi-a-GCST90002119 | TRUE | A | 29820182 | 1.41E-10 | reported | 2920 | 0.043 | rs9258608 |  |  |  |  |  |  |  |  |  |  |  |  |  |  |  |
| -0.2027 | 1 | igd | 0.1655 | A | \|\| id:ebi-a-GCST90001858 | ebi-a-GCST90001858 | TRUE | G | 167138747 | 2.68E-08 | reported | 2919 | 0.03634 | rs77126537 |  |  |  |  |  |  |  |  |  |  |  |  |  |  |  |
| 0.2899 | 22 | igd | 0.1653 | C | \|\| id:ebi-a-GCST90001704 | ebi-a-GCST90001704 | TRUE | A | 42651535 | 3.06E-16 | reported | 3657 | 0.03532 | rs56128552 |  |  |  |  |  |  |  |  |  |  |  |  |  |  |  |
| 0.2951 | 22 | igd | 0.1653 | C | \|\| id:ebi-a-GCST90001710 | ebi-a-GCST90001710 | TRUE | A | 42651535 | 8.36E-17 | reported | 3657 | 0.03527 | rs56128552 |  |  |  |  |  |  |  |  |  |  |  |  |  |  |  |
| 0.2736 | 22 | igd | 0.1653 | C | \|\| id:ebi-a-GCST90001711 | ebi-a-GCST90001711 | TRUE | A | 42651535 | 1.02E-14 | reported | 3657 | 0.03522 | rs56128552 |  |  |  |  |  |  |  |  |  |  |  |  |  |  |  |
| -0.4013 | 19 | igd | 0.1653 | C | \|\| id:ebi-a-GCST90001953 | ebi-a-GCST90001953 | TRUE | T | 51594543 | 6.32E-16 | reported | 1558 | 0.04912 | rs7343094 |  |  |  |  |  |  |  |  |  |  |  |  |  |  |  |
| 0.2504 | 19 | igd | 0.1652 | A | \|\| id:ebi-a-GCST90001523 | ebi-a-GCST90001523 | TRUE | T | 51644501 | 3.51E-08 | reported | 1858 | 0.04521 | rs1710384 |  |  |  |  |  |  |  |  |  |  |  |  |  |  |  |
| -0.4008 | 19 | igd | 0.1651 | C | \|\| id:ebi-a-GCST90001952 | ebi-a-GCST90001952 | TRUE | T | 51594543 | 8.70E-16 | reported | 1569 | 0.0493 | rs7343094 |  |  |  |  |  |  |  |  |  |  |  |  |  |  |  |
| -0.5974 | 10 | igd | 0.1645 | G | \|\| id:ebi-a-GCST90001660 | ebi-a-GCST90001660 | TRUE | C | 97348958 | 3.82E-67 | reported | 3408 | 0.03375 | rs12761112 |  |  |  |  |  |  |  |  |  |  |  |  |  |  |  |
| -0.5948 | 10 | igd | 0.1644 | G | \|\| id:ebi-a-GCST90001659 | ebi-a-GCST90001659 | TRUE | C | 97348958 | 3.78E-73 | reported | 3440 | 0.0321 | rs12761112 |  |  |  |  |  |  |  |  |  |  |  |  |  |  |  |
| -0.6122 | 10 | igd | 0.1644 | G | \|\| id:ebi-a-GCST90001671 | ebi-a-GCST90001671 | TRUE | C | 97348958 | 2.70E-71 | reported | 3440 | 0.03349 | rs12761112 |  |  |  |  |  |  |  |  |  |  |  |  |  |  |  |
| 0.2239 | 2 | igd | 0.1644 | C | \|\| id:ebi-a-GCST90002102 | ebi-a-GCST90002102 | TRUE | G | 39263755 | 7.87E-11 | reported | 2919 | 0.0343 | rs77278214 |  |  |  |  |  |  |  |  |  |  |  |  |  |  |  |
| -0.6131 | 10 | igd | 0.1643 | G | \|\| id:ebi-a-GCST90001672 | ebi-a-GCST90001672 | TRUE | A | 97324501 | 3.66E-69 | reported | 3408 | 0.03409 | rs11593693 |  |  |  |  |  |  |  |  |  |  |  |  |  |  |  |
| -0.4035 | 19 | igd | 0.1643 | C | \|\| id:ebi-a-GCST90001954 | ebi-a-GCST90001954 | TRUE | T | 51594543 | 6.73E-16 | reported | 1555 | 0.04943 | rs7343094 |  |  |  |  |  |  |  |  |  |  |  |  |  |  |  |
| -0.3891 | 19 | igd | 0.1643 | C | \|\| id:ebi-a-GCST90001956 | ebi-a-GCST90001956 | TRUE | T | 51594543 | 4.77E-15 | reported | 1579 | 0.04918 | rs7343094 |  |  |  |  |  |  |  |  |  |  |  |  |  |  |  |
| -0.2194 | 6 | igd | 0.1643 | A | \|\| id:ebi-a-GCST90002121 | ebi-a-GCST90002121 | TRUE | G | 31122500 | 1.10E-08 | reported | 2915 | 0.03829 | rs130065 |  |  |  |  |  |  |  |  |  |  |  |  |  |  |  |
| -0.4138 | 10 | igd | 0.1642 | G | \|\| id:ebi-a-GCST90001483 | ebi-a-GCST90001483 | TRUE | A | 97324501 | 6.43E-34 | reported | 3405 | 0.03371 | rs11593693 |  |  |  |  |  |  |  |  |  |  |  |  |  |  |  |
| -0.6152 | 10 | igd | 0.1642 | G | \|\| id:ebi-a-GCST90001670 | ebi-a-GCST90001670 | TRUE | A | 97324501 | 4.03E-72 | reported | 3440 | 0.03345 | rs11593693 |  |  |  |  |  |  |  |  |  |  |  |  |  |  |  |
| -0.3127 | 6 | igd | 0.1642 | A | \|\| id:ebi-a-GCST90002119 | ebi-a-GCST90002119 | TRUE | G | 31122500 | 1.42E-14 | reported | 2920 | 0.04043 | rs130065 |  |  |  |  |  |  |  |  |  |  |  |  |  |  |  |
| -0.4233 | 10 | igd | 0.1641 | G | \|\| id:ebi-a-GCST90001485 | ebi-a-GCST90001485 | TRUE | A | 97324501 | 3.26E-36 | reported | 3437 | 0.0333 | rs11593693 |  |  |  |  |  |  |  |  |  |  |  |  |  |  |  |
| -0.5637 | 10 | igd | 0.1641 | G | \|\| id:ebi-a-GCST90001496 | ebi-a-GCST90001496 | TRUE | A | 97324501 | 1.67E-59 | reported | 3437 | 0.03398 | rs11593693 |  |  |  |  |  |  |  |  |  |  |  |  |  |  |  |
| 0.3239 | 19 | igd | 0.1641 | T | \|\| id:ebi-a-GCST90001948 | ebi-a-GCST90001948 | TRUE | C | 51683580 | 8.12E-12 | reported | 1633 | 0.04704 | rs62115990 |  |  |  |  |  |  |  |  |  |  |  |  |  |  |  |
| -0.3868 | 19 | igd | 0.1641 | C | \|\| id:ebi-a-GCST90001957 | ebi-a-GCST90001957 | TRUE | T | 51594543 | 7.24E-15 | reported | 1578 | 0.04923 | rs7343094 |  |  |  |  |  |  |  |  |  |  |  |  |  |  |  |
| 0.3298 | 19 | igd | 0.164 | A | \|\| id:ebi-a-GCST90001946 | ebi-a-GCST90001946 | TRUE | G | 51683579 | 4.93E-12 | reported | 1634 | 0.04739 | rs62115989 |  |  |  |  |  |  |  |  |  |  |  |  |  |  |  |
| 0.3164 | 19 | igd | 0.164 | T | \|\| id:ebi-a-GCST90001949 | ebi-a-GCST90001949 | TRUE | C | 51683580 | 2.30E-11 | reported | 1634 | 0.04699 | rs62115990 |  |  |  |  |  |  |  |  |  |  |  |  |  |  |  |
| -0.1903 | 10 | igd | 0.1639 | A | \|\| id:ebi-a-GCST90001660 | ebi-a-GCST90001660 | TRUE | G | 99204791 | 2.70E-08 | reported | 3408 | 0.03415 | rs17415112 |  |  |  |  |  |  |  |  |  |  |  |  |  |  |  |
| -0.1941 | 10 | igd | 0.1639 | A | \|\| id:ebi-a-GCST90001672 | ebi-a-GCST90001672 | TRUE | G | 99204791 | 1.96E-08 | reported | 3408 | 0.03449 | rs17415112 |  |  |  |  |  |  |  |  |  |  |  |  |  |  |  |
| 0.3284 | 19 | igd | 0.1639 | G | \|\| id:ebi-a-GCST90001947 | ebi-a-GCST90001947 | TRUE | C | 51530741 | 7.82E-11 | reported | 1577 | 0.05014 | rs61752567 |  |  |  |  |  |  |  |  |  |  |  |  |  |  |  |
| 0.4362 | 19 | igd | 0.1639 | T | \|\| id:ebi-a-GCST90001951 | ebi-a-GCST90001951 | TRUE | C | 51739227 | 8.48E-18 | reported | 1464 | 0.05011 | rs146995981 |  |  |  |  |  |  |  |  |  |  |  |  |  |  |  |
| 0.4211 | 6 | igd | 0.1637 | A | \|\| id:ebi-a-GCST90002110 | ebi-a-GCST90002110 | TRUE | C | 33024032 | 8.52E-15 | reported | 1634 | 0.05376 | rs6934244 |  |  |  |  |  |  |  |  |  |  |  |  |  |  |  |
| 0.3775 | 6 | igd | 0.1636 | A | \|\| id:ebi-a-GCST90002111 | ebi-a-GCST90002111 | TRUE | C | 33024032 | 1.76E-12 | reported | 1635 | 0.05312 | rs6934244 |  |  |  |  |  |  |  |  |  |  |  |  |  |  |  |
| 0.2065 | 6 | igd | 0.1635 | C | \|\| id:ebi-a-GCST90001973 | ebi-a-GCST90001973 | TRUE | T | 30913475 | 4.51E-08 | reported | 3113 | 0.03765 | rs7745305 |  |  |  |  |  |  |  |  |  |  |  |  |  |  |  |
| 0.2391 | 6 | igd | 0.1635 | C | \|\| id:ebi-a-GCST90002081 | ebi-a-GCST90002081 | TRUE | T | 30913475 | 1.06E-09 | reported | 3113 | 0.03907 | rs7745305 |  |  |  |  |  |  |  |  |  |  |  |  |  |  |  |
| -0.2125 | 10 | igd | 0.1633 | T | \|\| id:ebi-a-GCST90001495 | ebi-a-GCST90001495 | TRUE | C | 98244928 | 1.35E-09 | reported | 3405 | 0.03496 | rs80018342 |  |  |  |  |  |  |  |  |  |  |  |  |  |  |  |
| 0.2156 | 10 | igd | 0.1633 | C | \|\| id:ebi-a-GCST90001672 | ebi-a-GCST90001672 | TRUE | G | 95359968 | 8.14E-10 | reported | 3408 | 0.035 | rs10882282 |  |  |  |  |  |  |  |  |  |  |  |  |  |  |  |
| -0.2289 | 10 | igd | 0.1631 | T | \|\| id:ebi-a-GCST90001660 | ebi-a-GCST90001660 | TRUE | C | 98244928 | 4.05E-11 | reported | 3408 | 0.03455 | rs80018342 |  |  |  |  |  |  |  |  |  |  |  |  |  |  |  |
| 0.2006 | 10 | igd | 0.1631 | C | \|\| id:ebi-a-GCST90001670 | ebi-a-GCST90001670 | TRUE | G | 95359968 | 6.17E-09 | reported | 3440 | 0.03442 | rs10882282 |  |  |  |  |  |  |  |  |  |  |  |  |  |  |  |
| -0.2506 | 10 | igd | 0.1631 | T | \|\| id:ebi-a-GCST90001672 | ebi-a-GCST90001672 | TRUE | C | 98244928 | 7.86E-13 | reported | 3408 | 0.03485 | rs80018342 |  |  |  |  |  |  |  |  |  |  |  |  |  |  |  |
| -0.2759 | 10 | igd | 0.1628 | G | \|\| id:ebi-a-GCST90001484 | ebi-a-GCST90001484 | TRUE | A | 97988866 | 2.44E-16 | reported | 3437 | 0.03349 | rs11812812 |  |  |  |  |  |  |  |  |  |  |  |  |  |  |  |
| -0.2624 | 10 | igd | 0.1628 | G | \|\| id:ebi-a-GCST90001485 | ebi-a-GCST90001485 | TRUE | A | 97988866 | 6.03E-15 | reported | 3437 | 0.03347 | rs11812812 |  |  |  |  |  |  |  |  |  |  |  |  |  |  |  |
| -0.2174 | 10 | igd | 0.1628 | G | \|\| id:ebi-a-GCST90001491 | ebi-a-GCST90001491 | TRUE | T | 96880523 | 5.23E-10 | reported | 3437 | 0.0349 | rs61280152 |  |  |  |  |  |  |  |  |  |  |  |  |  |  |  |
| -0.2134 | 10 | igd | 0.1628 | G | \|\| id:ebi-a-GCST90001496 | ebi-a-GCST90001496 | TRUE | T | 96880523 | 1.79E-09 | reported | 3437 | 0.03538 | rs61280152 |  |  |  |  |  |  |  |  |  |  |  |  |  |  |  |
| -0.2086 | 10 | igd | 0.1628 | G | \|\| id:ebi-a-GCST90001497 | ebi-a-GCST90001497 | TRUE | T | 96880523 | 2.63E-09 | reported | 3437 | 0.03494 | rs61280152 |  |  |  |  |  |  |  |  |  |  |  |  |  |  |  |
| -0.2247 | 10 | igd | 0.1626 | T | \|\| id:ebi-a-GCST90001658 | ebi-a-GCST90001658 | TRUE | C | 98244928 | 1.16E-11 | reported | 3440 | 0.03301 | rs80018342 |  |  |  |  |  |  |  |  |  |  |  |  |  |  |  |
| -0.2212 | 10 | igd | 0.1626 | G | \|\| id:ebi-a-GCST90001658 | ebi-a-GCST90001658 | TRUE | T | 96880523 | 5.86E-11 | reported | 3440 | 0.03368 | rs61280152 |  |  |  |  |  |  |  |  |  |  |  |  |  |  |  |
| -0.2152 | 10 | igd | 0.1626 | G | \|\| id:ebi-a-GCST90001659 | ebi-a-GCST90001659 | TRUE | T | 96880523 | 1.95E-10 | reported | 3440 | 0.0337 | rs61280152 |  |  |  |  |  |  |  |  |  |  |  |  |  |  |  |
| -0.2347 | 10 | igd | 0.1626 | T | \|\| id:ebi-a-GCST90001659 | ebi-a-GCST90001659 | TRUE | C | 98244928 | 1.37E-12 | reported | 3440 | 0.03299 | rs80018342 |  |  |  |  |  |  |  |  |  |  |  |  |  |  |  |
| -0.2436 | 10 | igd | 0.1626 | T | \|\| id:ebi-a-GCST90001670 | ebi-a-GCST90001670 | TRUE | C | 98244928 | 1.50E-12 | reported | 3440 | 0.03431 | rs80018342 |  |  |  |  |  |  |  |  |  |  |  |  |  |  |  |
| -0.2368 | 10 | igd | 0.1626 | T | \|\| id:ebi-a-GCST90001671 | ebi-a-GCST90001671 | TRUE | C | 98244928 | 6.86E-12 | reported | 3440 | 0.0344 | rs80018342 |  |  |  |  |  |  |  |  |  |  |  |  |  |  |  |
| 0.3233 | 19 | igd | 0.1625 | G | \|\| id:ebi-a-GCST90001952 | ebi-a-GCST90001952 | TRUE | C | 51530741 | 1.71E-10 | reported | 1569 | 0.0503 | rs61752567 |  |  |  |  |  |  |  |  |  |  |  |  |  |  |  |
| 0.3464 | 6 | igd | 0.1624 | C | \|\| id:ebi-a-GCST90002108 | ebi-a-GCST90002108 | TRUE | A | 32408735 | 1.13E-10 | reported | 1579 | 0.05336 | rs3129878 |  |  |  |  |  |  |  |  |  |  |  |  |  |  |  |
| 0.421 | 6 | igd | 0.1624 | A | \|\| id:ebi-a-GCST90002108 | ebi-a-GCST90002108 | TRUE | C | 33024032 | 1.51E-14 | reported | 1579 | 0.05425 | rs6934244 |  |  |  |  |  |  |  |  |  |  |  |  |  |  |  |
| -0.214 | 10 | igd | 0.1623 | G | \|\| id:ebi-a-GCST90001489 | ebi-a-GCST90001489 | TRUE | T | 96880523 | 1.68E-09 | reported | 3405 | 0.03542 | rs61280152 |  |  |  |  |  |  |  |  |  |  |  |  |  |  |  |
| -0.2047 | 10 | igd | 0.1623 | G | \|\| id:ebi-a-GCST90001495 | ebi-a-GCST90001495 | TRUE | T | 96880523 | 1.17E-08 | reported | 3405 | 0.0358 | rs61280152 |  |  |  |  |  |  |  |  |  |  |  |  |  |  |  |
| 0.5507 | 6 | igd | 0.1623 | A | \|\| id:ebi-a-GCST90002105 | ebi-a-GCST90002105 | TRUE | C | 32614419 | 2.07E-21 | reported | 2872 | 0.0575 | rs72852237 |  |  |  |  |  |  |  |  |  |  |  |  |  |  |  |
| 0.315 | 6 | igd | 0.1622 | A | \|\| id:ebi-a-GCST90002109 | ebi-a-GCST90002109 | TRUE | C | 33024032 | 8.19E-09 | reported | 1578 | 0.05435 | rs6934244 |  |  |  |  |  |  |  |  |  |  |  |  |  |  |  |
| -0.2033 | 10 | igd | 0.1621 | G | \|\| id:ebi-a-GCST90001660 | ebi-a-GCST90001660 | TRUE | T | 96880523 | 1.02E-08 | reported | 3408 | 0.03541 | rs61280152 |  |  |  |  |  |  |  |  |  |  |  |  |  |  |  |
| -0.2086 | 10 | igd | 0.1616 | A | \|\| id:ebi-a-GCST90002031 | ebi-a-GCST90002031 | TRUE | C | 102447647 | 4.40E-08 | reported | 2920 | 0.03801 | rs41310284 |  |  |  |  |  |  |  |  |  |  |  |  |  |  |  |
| -0.3246 | 1 | igd | 0.1613 | C | \|\| id:ebi-a-GCST90001474 | ebi-a-GCST90001474 | TRUE | G | 161623025 | 3.58E-21 | reported | 3410 | 0.03414 | rs61804164 |  |  |  |  |  |  |  |  |  |  |  |  |  |  |  |
| -0.2405 | 22 | igd | 0.1612 | G | \|\| id:ebi-a-GCST90001830 | ebi-a-GCST90001830 | TRUE | A | 42210119 | 2.67E-13 | reported | 3648 | 0.03278 | rs73163358 |  |  |  |  |  |  |  |  |  |  |  |  |  |  |  |
| -0.2107 | 2 | igd | 0.1612 | C | \|\| id:ebi-a-GCST90002102 | ebi-a-GCST90002102 | TRUE | T | 38900569 | 4.02E-09 | reported | 2919 | 0.0357 | rs113504153 |  |  |  |  |  |  |  |  |  |  |  |  |  |  |  |
| 0.3666 | 6 | igd | 0.1606 | C | \|\| id:ebi-a-GCST90002110 | ebi-a-GCST90002110 | TRUE | A | 32408735 | 7.51E-12 | reported | 1634 | 0.05315 | rs3129878 |  |  |  |  |  |  |  |  |  |  |  |  |  |  |  |
| 0.3324 | 6 | igd | 0.1606 | C | \|\| id:ebi-a-GCST90002111 | ebi-a-GCST90002111 | TRUE | A | 32408735 | 3.10E-10 | reported | 1635 | 0.05248 | rs3129878 |  |  |  |  |  |  |  |  |  |  |  |  |  |  |  |
| -0.2316 | 2 | igd | 0.1605 | C | \|\| id:ebi-a-GCST90001483 | ebi-a-GCST90001483 | TRUE | T | 38900569 | 2.75E-11 | reported | 3405 | 0.03466 | rs113504153 |  |  |  |  |  |  |  |  |  |  |  |  |  |  |  |
| -0.2412 | 2 | igd | 0.1603 | C | \|\| id:ebi-a-GCST90001485 | ebi-a-GCST90001485 | TRUE | T | 38900569 | 2.58E-12 | reported | 3437 | 0.03434 | rs113504153 |  |  |  |  |  |  |  |  |  |  |  |  |  |  |  |
| 0.5649 | 6 | igd | 0.1602 | C | \|\| id:ebi-a-GCST90002105 | ebi-a-GCST90002105 | TRUE | T | 32639909 | 6.06E-26 | reported | 2872 | 0.05311 | rs41316211 |  |  |  |  |  |  |  |  |  |  |  |  |  |  |  |
| 2.625 | 6 | igd | 0.1601 | A | \|\| id:ebi-a-GCST90001696 | ebi-a-GCST90001696 | TRUE | G | 31413809 | 2.03E-08 | reported | 3425 | 0.467 | rs9267091 |  |  |  |  |  |  |  |  |  |  |  |  |  |  |  |
| 0.2248 | 3 | igd | 0.16 | A | \|\| id:ebi-a-GCST90002015 | ebi-a-GCST90002015 | TRUE | T | 46352908 | 3.13E-09 | reported | 2871 | 0.03783 | rs62244806 |  |  |  |  |  |  |  |  |  |  |  |  |  |  |  |
| 0.2249 | 3 | igd | 0.16 | A | \|\| id:ebi-a-GCST90002016 | ebi-a-GCST90002016 | TRUE | T | 46352908 | 2.93E-09 | reported | 2871 | 0.03776 | rs62244806 |  |  |  |  |  |  |  |  |  |  |  |  |  |  |  |
| -0.2253 | 1 | igd | 0.1596 | T | \|\| id:ebi-a-GCST90002014 | ebi-a-GCST90002014 | TRUE | C | 161646807 | 3.83E-09 | reported | 2870 | 0.03812 | rs11799952 |  |  |  |  |  |  |  |  |  |  |  |  |  |  |  |
| 0.2587 | 22 | igd | 0.1591 | T | \|\| id:ebi-a-GCST90001702 | ebi-a-GCST90001702 | TRUE | G | 42848354 | 4.37E-14 | reported | 3657 | 0.03413 | rs5758727 |  |  |  |  |  |  |  |  |  |  |  |  |  |  |  |
| 0.2685 | 22 | igd | 0.1591 | T | \|\| id:ebi-a-GCST90001703 | ebi-a-GCST90001703 | TRUE | G | 42848354 | 4.54E-15 | reported | 3657 | 0.03411 | rs5758727 |  |  |  |  |  |  |  |  |  |  |  |  |  |  |  |
| 0.2422 | 22 | igd | 0.1591 | T | \|\| id:ebi-a-GCST90001704 | ebi-a-GCST90001704 | TRUE | G | 42848354 | 1.49E-12 | reported | 3657 | 0.03411 | rs5758727 |  |  |  |  |  |  |  |  |  |  |  |  |  |  |  |
| 0.2459 | 22 | igd | 0.1591 | T | \|\| id:ebi-a-GCST90001710 | ebi-a-GCST90001710 | TRUE | G | 42848354 | 6.29E-13 | reported | 3657 | 0.03406 | rs5758727 |  |  |  |  |  |  |  |  |  |  |  |  |  |  |  |
| 0.2514 | 22 | igd | 0.1591 | T | \|\| id:ebi-a-GCST90001711 | ebi-a-GCST90001711 | TRUE | G | 42848354 | 1.69E-13 | reported | 3657 | 0.03397 | rs5758727 |  |  |  |  |  |  |  |  |  |  |  |  |  |  |  |
| -0.1874 | 10 | igd | 0.159 | A | \|\| id:ebi-a-GCST90001659 | ebi-a-GCST90001659 | TRUE | C | 102447647 | 2.35E-08 | reported | 3440 | 0.03349 | rs41310284 |  |  |  |  |  |  |  |  |  |  |  |  |  |  |  |
| -0.1974 | 10 | igd | 0.1589 | A | \|\| id:ebi-a-GCST90001491 | ebi-a-GCST90001491 | TRUE | C | 102447647 | 1.34E-08 | reported | 3437 | 0.03466 | rs41310284 |  |  |  |  |  |  |  |  |  |  |  |  |  |  |  |
| 0.1947 | 20 | igd | 0.1584 | C | \|\| id:ebi-a-GCST90001800 | ebi-a-GCST90001800 | TRUE | T | 44622238 | 2.11E-08 | reported | 3656 | 0.03467 | rs73128584 |  |  |  |  |  |  |  |  |  |  |  |  |  |  |  |
| 0.2217 | 20 | igd | 0.1583 | C | \|\| id:ebi-a-GCST90001798 | ebi-a-GCST90001798 | TRUE | T | 44622238 | 9.81E-11 | reported | 3657 | 0.03416 | rs73128584 |  |  |  |  |  |  |  |  |  |  |  |  |  |  |  |
| 0.2295 | 20 | igd | 0.1583 | C | \|\| id:ebi-a-GCST90001804 | ebi-a-GCST90001804 | TRUE | T | 44622238 | 1.77E-11 | reported | 3657 | 0.03403 | rs73128584 |  |  |  |  |  |  |  |  |  |  |  |  |  |  |  |
| 0.2089 | 20 | igd | 0.1583 | C | \|\| id:ebi-a-GCST90001805 | ebi-a-GCST90001805 | TRUE | T | 44622238 | 1.54E-09 | reported | 3657 | 0.0345 | rs73128584 |  |  |  |  |  |  |  |  |  |  |  |  |  |  |  |
| 0.2261 | 20 | igd | 0.1583 | C | \|\| id:ebi-a-GCST90001808 | ebi-a-GCST90001808 | TRUE | T | 44622238 | 5.78E-11 | reported | 3657 | 0.03443 | rs73128584 |  |  |  |  |  |  |  |  |  |  |  |  |  |  |  |
| 0.3488 | 19 | igd | 0.158 | T | \|\| id:ebi-a-GCST90001950 | ebi-a-GCST90001950 | TRUE | C | 51695139 | 9.90E-10 | reported | 1244 | 0.05665 | rs12461866 |  |  |  |  |  |  |  |  |  |  |  |  |  |  |  |
| 0.5175 | 6 | igd | 0.1578 | C | \|\| id:ebi-a-GCST90002112 | ebi-a-GCST90002112 | TRUE | T | 32639909 | 1.03E-13 | reported | 1635 | 0.06898 | rs41316211 |  |  |  |  |  |  |  |  |  |  |  |  |  |  |  |
| 0.2085 | 2 | igd | 0.1567 | G | \|\| id:ebi-a-GCST90002098 | ebi-a-GCST90002098 | TRUE | A | 42004997 | 1.22E-08 | reported | 2910 | 0.03649 | rs719593 |  |  |  |  |  |  |  |  |  |  |  |  |  |  |  |
| 0.3715 | 6 | igd | 0.1565 | A | \|\| id:ebi-a-GCST90002105 | ebi-a-GCST90002105 | TRUE | C | 33024032 | 8.68E-18 | reported | 2872 | 0.04296 | rs6934244 |  |  |  |  |  |  |  |  |  |  |  |  |  |  |  |
| 0.3366 | 6 | igd | 0.1561 | T | \|\| id:ebi-a-GCST90002108 | ebi-a-GCST90002108 | TRUE | C | 32340070 | 5.63E-10 | reported | 1579 | 0.05395 | rs1980496 |  |  |  |  |  |  |  |  |  |  |  |  |  |  |  |
| 0.3186 | 6 | igd | 0.1555 | G | \|\| id:ebi-a-GCST90002105 | ebi-a-GCST90002105 | TRUE | C | 29589124 | 4.83E-15 | reported | 2872 | 0.04046 | rs29221 |  |  |  |  |  |  |  |  |  |  |  |  |  |  |  |
| -0.2434 | 6 | igd | 0.1555 | G | \|\| id:ebi-a-GCST90002118 | ebi-a-GCST90002118 | TRUE | A | 29531818 | 5.82E-09 | reported | 2920 | 0.04168 | rs362527 |  |  |  |  |  |  |  |  |  |  |  |  |  |  |  |
| 0.4026 | 19 | igd | 0.1553 | T | \|\| id:ebi-a-GCST90001954 | ebi-a-GCST90001954 | TRUE | C | 51695139 | 8.94E-15 | reported | 1555 | 0.05142 | rs12461866 |  |  |  |  |  |  |  |  |  |  |  |  |  |  |  |
| -0.1916 | 22 | igd | 0.1552 | C | \|\| id:ebi-a-GCST90001704 | ebi-a-GCST90001704 | TRUE | T | 44610112 | 2.62E-08 | reported | 3657 | 0.03435 | rs139207 |  |  |  |  |  |  |  |  |  |  |  |  |  |  |  |
| -0.3741 | 1 | igd | 0.1552 | T | \|\| id:ebi-a-GCST90001858 | ebi-a-GCST90001858 | TRUE | C | 167449608 | 2.11E-24 | reported | 2919 | 0.03637 | rs1214609 |  |  |  |  |  |  |  |  |  |  |  |  |  |  |  |
| -0.3277 | 1 | igd | 0.1552 | T | \|\| id:ebi-a-GCST90001861 | ebi-a-GCST90001861 | TRUE | C | 167449608 | 2.28E-19 | reported | 2919 | 0.03616 | rs1214609 |  |  |  |  |  |  |  |  |  |  |  |  |  |  |  |
| -0.2171 | 1 | igd | 0.1552 | T | \|\| id:ebi-a-GCST90001863 | ebi-a-GCST90001863 | TRUE | C | 167449608 | 2.51E-09 | reported | 2919 | 0.0363 | rs1214609 |  |  |  |  |  |  |  |  |  |  |  |  |  |  |  |
| -0.3407 | 1 | igd | 0.1552 | T | \|\| id:ebi-a-GCST90001864 | ebi-a-GCST90001864 | TRUE | C | 167449608 | 1.97E-20 | reported | 2919 | 0.0365 | rs1214609 |  |  |  |  |  |  |  |  |  |  |  |  |  |  |  |
| 0.3825 | 19 | igd | 0.1551 | T | \|\| id:ebi-a-GCST90001955 | ebi-a-GCST90001955 | TRUE | C | 51695139 | 2.09E-13 | reported | 1554 | 0.05162 | rs12461866 |  |  |  |  |  |  |  |  |  |  |  |  |  |  |  |
| 0.4003 | 19 | igd | 0.155 | T | \|\| id:ebi-a-GCST90001953 | ebi-a-GCST90001953 | TRUE | C | 51695139 | 1.30E-14 | reported | 1558 | 0.05144 | rs12461866 |  |  |  |  |  |  |  |  |  |  |  |  |  |  |  |
| 0.2328 | 1 | igd | 0.1547 | T | \|\| id:ebi-a-GCST90001854 | ebi-a-GCST90001854 | TRUE | G | 167123828 | 5.00E-10 | reported | 2919 | 0.03731 | rs12138032 |  |  |  |  |  |  |  |  |  |  |  |  |  |  |  |
| 0.2798 | 1 | igd | 0.1547 | T | \|\| id:ebi-a-GCST90001858 | ebi-a-GCST90001858 | TRUE | G | 167123828 | 1.07E-13 | reported | 2919 | 0.03747 | rs12138032 |  |  |  |  |  |  |  |  |  |  |  |  |  |  |  |
| 0.2864 | 1 | igd | 0.1547 | T | \|\| id:ebi-a-GCST90001860 | ebi-a-GCST90001860 | TRUE | G | 167123828 | 3.65E-14 | reported | 2919 | 0.03763 | rs12138032 |  |  |  |  |  |  |  |  |  |  |  |  |  |  |  |
| 0.2815 | 19 | igd | 0.1547 | A | \|\| id:ebi-a-GCST90001954 | ebi-a-GCST90001954 | TRUE | G | 54847587 | 3.96E-08 | reported | 1555 | 0.05099 | rs17836364 |  |  |  |  |  |  |  |  |  |  |  |  |  |  |  |
| 0.7607 | 6 | igd | 0.1546 | G | \|\| id:ebi-a-GCST90002105 | ebi-a-GCST90002105 | TRUE | A | 32433067 | 3.95E-76 | reported | 2872 | 0.03998 | rs9268926 |  |  |  |  |  |  |  |  |  |  |  |  |  |  |  |
| 0.2713 | 1 | igd | 0.1545 | G | \|\| id:ebi-a-GCST90001970 | ebi-a-GCST90001970 | TRUE | T | 161508180 | 3.61E-13 | reported | 2971 | 0.03715 | rs332396 |  |  |  |  |  |  |  |  |  |  |  |  |  |  |  |
| -0.2158 | 1 | igd | 0.1545 | T | \|\| id:ebi-a-GCST90002036 | ebi-a-GCST90002036 | TRUE | C | 92661882 | 3.05E-08 | reported | 2871 | 0.03886 | rs80105455 |  |  |  |  |  |  |  |  |  |  |  |  |  |  |  |
| 0.283 | 19 | igd | 0.1544 | A | \|\| id:ebi-a-GCST90001955 | ebi-a-GCST90001955 | TRUE | G | 54847587 | 3.61E-08 | reported | 1554 | 0.05112 | rs17836364 |  |  |  |  |  |  |  |  |  |  |  |  |  |  |  |
| 0.3188 | 1 | igd | 0.1541 | T | \|\| id:ebi-a-GCST90001999 | ebi-a-GCST90001999 | TRUE | C | 161489353 | 4.15E-20 | reported | 3621 | 0.0345 | rs1542042 |  |  |  |  |  |  |  |  |  |  |  |  |  |  |  |
| 0.2338 | 4 | igd | 0.154 | C | \|\| id:ebi-a-GCST90002037 | ebi-a-GCST90002037 | TRUE | T | 84177774 | 2.75E-09 | reported | 2871 | 0.0392 | rs145702441 |  |  |  |  |  |  |  |  |  |  |  |  |  |  |  |
| 0.2327 | 4 | igd | 0.154 | C | \|\| id:ebi-a-GCST90002038 | ebi-a-GCST90002038 | TRUE | T | 84177774 | 3.27E-09 | reported | 2871 | 0.0392 | rs145702441 |  |  |  |  |  |  |  |  |  |  |  |  |  |  |  |
| 0.2372 | 4 | igd | 0.1539 | C | \|\| id:ebi-a-GCST90001944 | ebi-a-GCST90001944 | TRUE | T | 84177774 | 1.81E-09 | reported | 2872 | 0.03931 | rs145702441 |  |  |  |  |  |  |  |  |  |  |  |  |  |  |  |
| 0.2345 | 4 | igd | 0.1539 | C | \|\| id:ebi-a-GCST90001945 | ebi-a-GCST90001945 | TRUE | T | 84177774 | 2.72E-09 | reported | 2872 | 0.03931 | rs145702441 |  |  |  |  |  |  |  |  |  |  |  |  |  |  |  |
| 0.41 | 19 | igd | 0.1539 | T | \|\| id:ebi-a-GCST90001952 | ebi-a-GCST90001952 | TRUE | C | 51695139 | 3.65E-15 | reported | 1569 | 0.0516 | rs12461866 |  |  |  |  |  |  |  |  |  |  |  |  |  |  |  |
| 0.6711 | 6 | igd | 0.1536 | G | \|\| id:ebi-a-GCST90002108 | ebi-a-GCST90002108 | TRUE | C | 32501241 | 5.29E-17 | reported | 1579 | 0.07917 | rs115032171 |  |  |  |  |  |  |  |  |  |  |  |  |  |  |  |
| -0.171836 | 10 | igd | 0.153509 | A | \|\| id:met-b-156 | met-b-156 | TRUE | G | 97396245 | 1.42E-10 | reported | 456 | 0.0267906 | rs1933164 |  |  |  |  |  |  |  |  |  |  |  |  |  |  |  |
| 0.3785 | 19 | igd | 0.1535 | T | \|\| id:ebi-a-GCST90001947 | ebi-a-GCST90001947 | TRUE | C | 51695139 | 4.16E-13 | reported | 1577 | 0.05177 | rs12461866 |  |  |  |  |  |  |  |  |  |  |  |  |  |  |  |
| -0.2487 | 10 | igd | 0.1535 | C | \|\| id:ebi-a-GCST90002029 | ebi-a-GCST90002029 | TRUE | A | 97740223 | 5.40E-11 | reported | 2915 | 0.03777 | rs11599105 |  |  |  |  |  |  |  |  |  |  |  |  |  |  |  |
| 0.4173 | 19 | igd | 0.1534 | T | \|\| id:ebi-a-GCST90001957 | ebi-a-GCST90001957 | TRUE | C | 51695139 | 1.11E-15 | reported | 1578 | 0.05153 | rs12461866 |  |  |  |  |  |  |  |  |  |  |  |  |  |  |  |
| 0.4399 | 6 | igd | 0.1534 | G | \|\| id:ebi-a-GCST90002109 | ebi-a-GCST90002109 | TRUE | C | 32501241 | 3.89E-08 | reported | 1578 | 0.07964 | rs115032171 |  |  |  |  |  |  |  |  |  |  |  |  |  |  |  |
| -0.3267 | 10 | igd | 0.1533 | G | \|\| id:ebi-a-GCST90001485 | ebi-a-GCST90001485 | TRUE | A | 97772358 | 7.44E-21 | reported | 3437 | 0.03466 | rs17383738 |  |  |  |  |  |  |  |  |  |  |  |  |  |  |  |
| 0.4096 | 19 | igd | 0.1533 | T | \|\| id:ebi-a-GCST90001956 | ebi-a-GCST90001956 | TRUE | C | 51695139 | 3.49E-15 | reported | 1579 | 0.05151 | rs12461866 |  |  |  |  |  |  |  |  |  |  |  |  |  |  |  |
| -0.328 | 10 | igd | 0.153 | G | \|\| id:ebi-a-GCST90001483 | ebi-a-GCST90001483 | TRUE | A | 97772358 | 1.52E-20 | reported | 3405 | 0.03507 | rs17383738 |  |  |  |  |  |  |  |  |  |  |  |  |  |  |  |
| -0.2439 | 6 | igd | 0.153 | G | \|\| id:ebi-a-GCST90002121 | ebi-a-GCST90002121 | TRUE | A | 29730390 | 1.31E-09 | reported | 2915 | 0.04008 | rs9500976 |  |  |  |  |  |  |  |  |  |  |  |  |  |  |  |
| 0.2985 | 10 | igd | 0.1529 | T | \|\| id:ebi-a-GCST90002030 | ebi-a-GCST90002030 | TRUE | C | 96517572 | 1.77E-14 | reported | 2920 | 0.03874 | rs61886219 |  |  |  |  |  |  |  |  |  |  |  |  |  |  |  |
| 0.2302 | 10 | igd | 0.1529 | T | \|\| id:ebi-a-GCST90002031 | ebi-a-GCST90002031 | TRUE | C | 96517572 | 6.62E-09 | reported | 2920 | 0.03957 | rs61886219 |  |  |  |  |  |  |  |  |  |  |  |  |  |  |  |
| 0.328 | 10 | igd | 0.1529 | T | \|\| id:ebi-a-GCST90002032 | ebi-a-GCST90002032 | TRUE | C | 96517572 | 1.14E-16 | reported | 2920 | 0.03934 | rs61886219 |  |  |  |  |  |  |  |  |  |  |  |  |  |  |  |
| -0.2695 | 6 | igd | 0.1529 | G | \|\| id:ebi-a-GCST90002118 | ebi-a-GCST90002118 | TRUE | A | 29730390 | 3.04E-10 | reported | 2920 | 0.04264 | rs9500976 |  |  |  |  |  |  |  |  |  |  |  |  |  |  |  |
| -0.2727 | 6 | igd | 0.1529 | G | \|\| id:ebi-a-GCST90002119 | ebi-a-GCST90002119 | TRUE | A | 29730390 | 1.33E-10 | reported | 2920 | 0.0423 | rs9500976 |  |  |  |  |  |  |  |  |  |  |  |  |  |  |  |
| 0.2234 | 1 | igd | 0.1528 | G | \|\| id:ebi-a-GCST90001858 | ebi-a-GCST90001858 | TRUE | A | 166785415 | 6.42E-09 | reported | 2919 | 0.03836 | rs11583960 |  |  |  |  |  |  |  |  |  |  |  |  |  |  |  |
| -0.2166 | 6 | igd | 0.1528 | T | \|\| id:ebi-a-GCST90002121 | ebi-a-GCST90002121 | TRUE | C | 32029183 | 2.48E-08 | reported | 2915 | 0.03875 | rs2269429 |  |  |  |  |  |  |  |  |  |  |  |  |  |  |  |
| 0.2541 | 19 | igd | 0.1527 | T | \|\| id:ebi-a-GCST90001533 | ebi-a-GCST90001533 | TRUE | C | 8786913 | 3.85E-08 | reported | 1857 | 0.04602 | rs4499342 |  |  |  |  |  |  |  |  |  |  |  |  |  |  |  |
| 0.2941 | 19 | igd | 0.1527 | A | \|\| id:ebi-a-GCST90001946 | ebi-a-GCST90001946 | TRUE | G | 54847587 | 4.25E-09 | reported | 1634 | 0.0498 | rs17836364 |  |  |  |  |  |  |  |  |  |  |  |  |  |  |  |
| 0.2271 | 10 | igd | 0.1517 | T | \|\| id:ebi-a-GCST90001495 | ebi-a-GCST90001495 | TRUE | C | 96517572 | 2.21E-09 | reported | 3405 | 0.03786 | rs61886219 |  |  |  |  |  |  |  |  |  |  |  |  |  |  |  |
| 0.3062 | 6 | igd | 0.1517 | A | \|\| id:ebi-a-GCST90002111 | ebi-a-GCST90002111 | TRUE | G | 32812801 | 7.72E-09 | reported | 1635 | 0.05275 | rs113215190 |  |  |  |  |  |  |  |  |  |  |  |  |  |  |  |
| -0.1949 | 20 | igd | 0.1516 | T | \|\| id:ebi-a-GCST90001981 | ebi-a-GCST90001981 | TRUE | C | 44511627 | 2.26E-08 | reported | 3618 | 0.03478 | rs11086984 |  |  |  |  |  |  |  |  |  |  |  |  |  |  |  |
| -0.1942 | 20 | igd | 0.1516 | T | \|\| id:ebi-a-GCST90001985 | ebi-a-GCST90001985 | TRUE | C | 44511627 | 3.04E-08 | reported | 3629 | 0.03498 | rs11086984 |  |  |  |  |  |  |  |  |  |  |  |  |  |  |  |
| 0.3517 | 6 | igd | 0.1515 | A | \|\| id:ebi-a-GCST90002110 | ebi-a-GCST90002110 | TRUE | G | 32812801 | 6.32E-11 | reported | 1634 | 0.05345 | rs113215190 |  |  |  |  |  |  |  |  |  |  |  |  |  |  |  |
| 0.256 | 20 | igd | 0.1514 | T | \|\| id:ebi-a-GCST90001798 | ebi-a-GCST90001798 | TRUE | C | 44511627 | 1.30E-13 | reported | 3657 | 0.03444 | rs11086984 |  |  |  |  |  |  |  |  |  |  |  |  |  |  |  |
| 0.2106 | 20 | igd | 0.1514 | T | \|\| id:ebi-a-GCST90001802 | ebi-a-GCST90001802 | TRUE | C | 44511627 | 1.67E-09 | reported | 3657 | 0.03486 | rs11086984 |  |  |  |  |  |  |  |  |  |  |  |  |  |  |  |
| 0.2742 | 20 | igd | 0.1514 | T | \|\| id:ebi-a-GCST90001804 | ebi-a-GCST90001804 | TRUE | C | 44511627 | 1.59E-15 | reported | 3657 | 0.03426 | rs11086984 |  |  |  |  |  |  |  |  |  |  |  |  |  |  |  |
| 0.199 | 20 | igd | 0.1514 | T | \|\| id:ebi-a-GCST90001805 | ebi-a-GCST90001805 | TRUE | C | 44511627 | 1.25E-08 | reported | 3657 | 0.03488 | rs11086984 |  |  |  |  |  |  |  |  |  |  |  |  |  |  |  |
| 0.2761 | 20 | igd | 0.1514 | T | \|\| id:ebi-a-GCST90001808 | ebi-a-GCST90001808 | TRUE | C | 44511627 | 2.23E-15 | reported | 3657 | 0.03467 | rs11086984 |  |  |  |  |  |  |  |  |  |  |  |  |  |  |  |
| 0.1988 | 20 | igd | 0.1513 | T | \|\| id:ebi-a-GCST90001806 | ebi-a-GCST90001806 | TRUE | C | 44511627 | 1.25E-08 | reported | 3656 | 0.03485 | rs11086984 |  |  |  |  |  |  |  |  |  |  |  |  |  |  |  |
| 0.6619 | 6 | igd | 0.1508 | G | \|\| id:ebi-a-GCST90002112 | ebi-a-GCST90002112 | TRUE | T | 32402778 | 3.63E-35 | reported | 1635 | 0.05224 | rs9268614 |  |  |  |  |  |  |  |  |  |  |  |  |  |  |  |
| -0.4906 | 10 | igd | 0.1502 | C | \|\| id:ebi-a-GCST90001495 | ebi-a-GCST90001495 | TRUE | G | 97365622 | 2.12E-41 | reported | 3405 | 0.03591 | rs11188395 |  |  |  |  |  |  |  |  |  |  |  |  |  |  |  |
| -0.5031 | 10 | igd | 0.1502 | C | \|\| id:ebi-a-GCST90001660 | ebi-a-GCST90001660 | TRUE | G | 97365622 | 1.63E-44 | reported | 3408 | 0.03543 | rs11188395 |  |  |  |  |  |  |  |  |  |  |  |  |  |  |  |
| -0.5592 | 10 | igd | 0.1501 | C | \|\| id:ebi-a-GCST90001496 | ebi-a-GCST90001496 | TRUE | G | 97365622 | 5.46E-55 | reported | 3437 | 0.03517 | rs11188395 |  |  |  |  |  |  |  |  |  |  |  |  |  |  |  |
| -0.5346 | 10 | igd | 0.1501 | C | \|\| id:ebi-a-GCST90001497 | ebi-a-GCST90001497 | TRUE | G | 97365622 | 1.49E-51 | reported | 3437 | 0.0348 | rs11188395 |  |  |  |  |  |  |  |  |  |  |  |  |  |  |  |
| -0.4372 | 10 | igd | 0.15 | C | \|\| id:ebi-a-GCST90001484 | ebi-a-GCST90001484 | TRUE | T | 97374858 | 2.88E-36 | reported | 3437 | 0.03436 | rs11188402 |  |  |  |  |  |  |  |  |  |  |  |  |  |  |  |
| -0.4954 | 10 | igd | 0.1499 | A | \|\| id:ebi-a-GCST90001658 | ebi-a-GCST90001658 | TRUE | G | 97368990 | 1.82E-47 | reported | 3440 | 0.03371 | rs10882639 |  |  |  |  |  |  |  |  |  |  |  |  |  |  |  |
| -0.5049 | 10 | igd | 0.1499 | A | \|\| id:ebi-a-GCST90001659 | ebi-a-GCST90001659 | TRUE | G | 97368990 | 2.77E-49 | reported | 3440 | 0.03367 | rs10882639 |  |  |  |  |  |  |  |  |  |  |  |  |  |  |  |
| -0.4733 | 10 | igd | 0.1498 | A | \|\| id:ebi-a-GCST90001491 | ebi-a-GCST90001491 | TRUE | G | 97368990 | 1.50E-40 | reported | 3437 | 0.03504 | rs10882639 |  |  |  |  |  |  |  |  |  |  |  |  |  |  |  |
| 0.2125 | 2 | igd | 0.1494 | G | \|\| id:ebi-a-GCST90001900 | ebi-a-GCST90001900 | TRUE | A | 204133565 | 2.41E-08 | reported | 2919 | 0.03798 | rs55939578 |  |  |  |  |  |  |  |  |  |  |  |  |  |  |  |
| 0.403 | 1 | igd | 0.1494 | T | \|\| id:ebi-a-GCST90001994 | ebi-a-GCST90001994 | TRUE | C | 161291228 | 1.87E-31 | reported | 3590 | 0.03421 | rs72714967 |  |  |  |  |  |  |  |  |  |  |  |  |  |  |  |
| 0.2175 | 1 | igd | 0.1494 | T | \|\| id:ebi-a-GCST90001997 | ebi-a-GCST90001997 | TRUE | C | 161291228 | 1.07E-09 | reported | 3590 | 0.03556 | rs72714967 |  |  |  |  |  |  |  |  |  |  |  |  |  |  |  |
| 0.2199 | 2 | igd | 0.1493 | G | \|\| id:ebi-a-GCST90001893 | ebi-a-GCST90001893 | TRUE | A | 204133565 | 8.79E-09 | reported | 2920 | 0.03811 | rs55939578 |  |  |  |  |  |  |  |  |  |  |  |  |  |  |  |
| -0.2612 | 6 | igd | 0.1491 | A | \|\| id:ebi-a-GCST90002119 | ebi-a-GCST90002119 | TRUE | G | 30648817 | 3.41E-09 | reported | 2920 | 0.04406 | rs151155542 |  |  |  |  |  |  |  |  |  |  |  |  |  |  |  |
| -0.2735 | 6 | igd | 0.149 | A | \|\| id:ebi-a-GCST90002119 | ebi-a-GCST90002119 | TRUE | G | 30762645 | 7.51E-10 | reported | 2920 | 0.04428 | rs188964110 |  |  |  |  |  |  |  |  |  |  |  |  |  |  |  |
| -0.3679 | 1 | igd | 0.1487 | T | \|\| id:ebi-a-GCST90001999 | ebi-a-GCST90001999 | TRUE | C | 161291228 | 2.21E-25 | reported | 3621 | 0.03507 | rs72714967 |  |  |  |  |  |  |  |  |  |  |  |  |  |  |  |
| 0.2238 | 10 | igd | 0.1485 | T | \|\| id:ebi-a-GCST90001497 | ebi-a-GCST90001497 | TRUE | C | 98382882 | 2.25E-10 | reported | 3437 | 0.03518 | rs4917744 |  |  |  |  |  |  |  |  |  |  |  |  |  |  |  |
| 0.2184 | 6 | igd | 0.1482 | T | \|\| id:ebi-a-GCST90001761 | ebi-a-GCST90001761 | TRUE | G | 31392047 | 3.68E-08 | reported | 3657 | 0.03959 | rs9501107 |  |  |  |  |  |  |  |  |  |  |  |  |  |  |  |
| -0.2588 | 1 | igd | 0.1482 | A | \|\| id:ebi-a-GCST90001854 | ebi-a-GCST90001854 | TRUE | G | 166464476 | 1.41E-11 | reported | 2919 | 0.03815 | rs12138291 |  |  |  |  |  |  |  |  |  |  |  |  |  |  |  |
| -0.2363 | 1 | igd | 0.1482 | A | \|\| id:ebi-a-GCST90001856 | ebi-a-GCST90001856 | TRUE | G | 166464476 | 1.02E-09 | reported | 2919 | 0.03857 | rs12138291 |  |  |  |  |  |  |  |  |  |  |  |  |  |  |  |
| -0.2467 | 1 | igd | 0.1482 | A | \|\| id:ebi-a-GCST90001858 | ebi-a-GCST90001858 | TRUE | G | 166464476 | 1.47E-10 | reported | 2919 | 0.03836 | rs12138291 |  |  |  |  |  |  |  |  |  |  |  |  |  |  |  |
| -0.2773 | 1 | igd | 0.1482 | A | \|\| id:ebi-a-GCST90001860 | ebi-a-GCST90001860 | TRUE | G | 166464476 | 7.97E-13 | reported | 2919 | 0.03854 | rs12138291 |  |  |  |  |  |  |  |  |  |  |  |  |  |  |  |
| -0.2709 | 1 | igd | 0.1482 | A | \|\| id:ebi-a-GCST90001864 | ebi-a-GCST90001864 | TRUE | G | 166464476 | 1.93E-12 | reported | 2919 | 0.03832 | rs12138291 |  |  |  |  |  |  |  |  |  |  |  |  |  |  |  |
| 0.4847 | 19 | igd | 0.1482 | T | \|\| id:ebi-a-GCST90001948 | ebi-a-GCST90001948 | TRUE | C | 51695139 | 1.24E-21 | reported | 1633 | 0.05001 | rs12461866 |  |  |  |  |  |  |  |  |  |  |  |  |  |  |  |
| 0.4592 | 19 | igd | 0.1481 | T | \|\| id:ebi-a-GCST90001946 | ebi-a-GCST90001946 | TRUE | C | 51695139 | 2.34E-19 | reported | 1634 | 0.05041 | rs12461866 |  |  |  |  |  |  |  |  |  |  |  |  |  |  |  |
| 0.4781 | 19 | igd | 0.1481 | T | \|\| id:ebi-a-GCST90001949 | ebi-a-GCST90001949 | TRUE | C | 51695139 | 3.60E-21 | reported | 1634 | 0.04993 | rs12461866 |  |  |  |  |  |  |  |  |  |  |  |  |  |  |  |
| 0.2287 | 6 | igd | 0.1481 | G | \|\| id:ebi-a-GCST90002081 | ebi-a-GCST90002081 | TRUE | C | 31405825 | 2.31E-08 | reported | 3113 | 0.04083 | rs79493037 |  |  |  |  |  |  |  |  |  |  |  |  |  |  |  |
| -0.2895 | 1 | igd | 0.148 | A | \|\| id:ebi-a-GCST90001981 | ebi-a-GCST90001981 | TRUE | T | 161466549 | 6.25E-16 | reported | 3618 | 0.03564 | rs115438555 |  |  |  |  |  |  |  |  |  |  |  |  |  |  |  |
| -0.2369 | 6 | igd | 0.148 | C | \|\| id:ebi-a-GCST90002121 | ebi-a-GCST90002121 | TRUE | T | 29932257 | 6.51E-09 | reported | 2915 | 0.0407 | rs73416612 |  |  |  |  |  |  |  |  |  |  |  |  |  |  |  |
| 0.2091 | 10 | igd | 0.1478 | G | \|\| id:ebi-a-GCST90001491 | ebi-a-GCST90001491 | TRUE | C | 102113278 | 3.86E-09 | reported | 3437 | 0.0354 | rs3793765 |  |  |  |  |  |  |  |  |  |  |  |  |  |  |  |
| 0.1993 | 10 | igd | 0.1478 | G | \|\| id:ebi-a-GCST90001496 | ebi-a-GCST90001496 | TRUE | C | 102113278 | 3.05E-08 | reported | 3437 | 0.0359 | rs3793765 |  |  |  |  |  |  |  |  |  |  |  |  |  |  |  |
| -0.2578 | 6 | igd | 0.1478 | C | \|\| id:ebi-a-GCST90002118 | ebi-a-GCST90002118 | TRUE | G | 31321238 | 2.73E-09 | reported | 2920 | 0.04322 | rs6922309 |  |  |  |  |  |  |  |  |  |  |  |  |  |  |  |
| -0.297 | 6 | igd | 0.1478 | C | \|\| id:ebi-a-GCST90002119 | ebi-a-GCST90002119 | TRUE | G | 31321238 | 4.56E-12 | reported | 2920 | 0.04275 | rs6922309 |  |  |  |  |  |  |  |  |  |  |  |  |  |  |  |
| -0.2792 | 22 | igd | 0.1471 | T | \|\| id:ebi-a-GCST90001704 | ebi-a-GCST90001704 | TRUE | A | 40614675 | 2.36E-15 | reported | 3657 | 0.0351 | rs78666753 |  |  |  |  |  |  |  |  |  |  |  |  |  |  |  |
| -0.2556 | 22 | igd | 0.1471 | T | \|\| id:ebi-a-GCST90001711 | ebi-a-GCST90001711 | TRUE | A | 40614675 | 3.56E-13 | reported | 3657 | 0.03502 | rs78666753 |  |  |  |  |  |  |  |  |  |  |  |  |  |  |  |
| 0.3047 | 1 | igd | 0.1471 | A | \|\| id:ebi-a-GCST90001970 | ebi-a-GCST90001970 | TRUE | G | 161410349 | 4.75E-15 | reported | 2971 | 0.03869 | rs146718864 |  |  |  |  |  |  |  |  |  |  |  |  |  |  |  |
| -0.6321 | 10 | igd | 0.1471 | C | \|\| id:ebi-a-GCST90002030 | ebi-a-GCST90002030 | TRUE | G | 97365622 | 1.19E-62 | reported | 2920 | 0.03693 | rs11188395 |  |  |  |  |  |  |  |  |  |  |  |  |  |  |  |
| -0.6164 | 10 | igd | 0.1471 | C | \|\| id:ebi-a-GCST90002031 | ebi-a-GCST90002031 | TRUE | G | 97365622 | 9.23E-57 | reported | 2920 | 0.03799 | rs11188395 |  |  |  |  |  |  |  |  |  |  |  |  |  |  |  |
| -0.5398 | 10 | igd | 0.1471 | C | \|\| id:ebi-a-GCST90002032 | ebi-a-GCST90002032 | TRUE | G | 97365622 | 1.42E-43 | reported | 2920 | 0.03836 | rs11188395 |  |  |  |  |  |  |  |  |  |  |  |  |  |  |  |
| -0.2664 | 10 | igd | 0.1467 | A | \|\| id:ebi-a-GCST90002029 | ebi-a-GCST90002029 | TRUE | G | 97368990 | 5.37E-12 | reported | 2915 | 0.03847 | rs10882639 |  |  |  |  |  |  |  |  |  |  |  |  |  |  |  |
| -0.2507 | 10 | igd | 0.1465 | A | \|\| id:ebi-a-GCST90001484 | ebi-a-GCST90001484 | TRUE | C | 96990275 | 2.10E-12 | reported | 3437 | 0.03554 | rs72820690 |  |  |  |  |  |  |  |  |  |  |  |  |  |  |  |
| -0.2259 | 10 | igd | 0.1465 | A | \|\| id:ebi-a-GCST90001485 | ebi-a-GCST90001485 | TRUE | C | 96990275 | 2.34E-10 | reported | 3437 | 0.03554 | rs72820690 |  |  |  |  |  |  |  |  |  |  |  |  |  |  |  |
| -0.2701 | 10 | igd | 0.1465 | A | \|\| id:ebi-a-GCST90001491 | ebi-a-GCST90001491 | TRUE | C | 96990275 | 1.24E-13 | reported | 3437 | 0.03629 | rs72820690 |  |  |  |  |  |  |  |  |  |  |  |  |  |  |  |
| -0.2804 | 10 | igd | 0.1465 | A | \|\| id:ebi-a-GCST90001496 | ebi-a-GCST90001496 | TRUE | C | 96990275 | 3.10E-14 | reported | 3437 | 0.03676 | rs72820690 |  |  |  |  |  |  |  |  |  |  |  |  |  |  |  |
| -0.2779 | 10 | igd | 0.1465 | A | \|\| id:ebi-a-GCST90001497 | ebi-a-GCST90001497 | TRUE | C | 96990275 | 2.46E-14 | reported | 3437 | 0.0363 | rs72820690 |  |  |  |  |  |  |  |  |  |  |  |  |  |  |  |
| -0.287 | 10 | igd | 0.1464 | A | \|\| id:ebi-a-GCST90001658 | ebi-a-GCST90001658 | TRUE | C | 96990275 | 3.35E-16 | reported | 3440 | 0.035 | rs72820690 |  |  |  |  |  |  |  |  |  |  |  |  |  |  |  |
| -0.2854 | 10 | igd | 0.1464 | A | \|\| id:ebi-a-GCST90001659 | ebi-a-GCST90001659 | TRUE | C | 96990275 | 4.88E-16 | reported | 3440 | 0.03501 | rs72820690 |  |  |  |  |  |  |  |  |  |  |  |  |  |  |  |
| -0.24 | 10 | igd | 0.1464 | A | \|\| id:ebi-a-GCST90001670 | ebi-a-GCST90001670 | TRUE | C | 96990275 | 5.81E-11 | reported | 3440 | 0.03653 | rs72820690 |  |  |  |  |  |  |  |  |  |  |  |  |  |  |  |
| -0.2376 | 10 | igd | 0.1464 | A | \|\| id:ebi-a-GCST90001671 | ebi-a-GCST90001671 | TRUE | C | 96990275 | 9.80E-11 | reported | 3440 | 0.03661 | rs72820690 |  |  |  |  |  |  |  |  |  |  |  |  |  |  |  |
| 0.7089 | 20 | igd | 0.1464 | A | \|\| id:ebi-a-GCST90001989 | ebi-a-GCST90001989 | TRUE | G | 44747086 | 5.03E-94 | reported | 3621 | 0.03346 | rs745307 |  |  |  |  |  |  |  |  |  |  |  |  |  |  |  |
| 0.209 | 1 | igd | 0.1462 | G | \|\| id:ebi-a-GCST90001919 | ebi-a-GCST90001919 | TRUE | A | 198636736 | 3.88E-08 | reported | 3113 | 0.03794 | rs12145132 |  |  |  |  |  |  |  |  |  |  |  |  |  |  |  |
| -0.2379 | 1 | igd | 0.1461 | A | \|\| id:ebi-a-GCST90001858 | ebi-a-GCST90001858 | TRUE | C | 167104790 | 3.85E-10 | reported | 2919 | 0.03788 | rs12077809 |  |  |  |  |  |  |  |  |  |  |  |  |  |  |  |
| -0.2105 | 1 | igd | 0.1461 | A | \|\| id:ebi-a-GCST90001861 | ebi-a-GCST90001861 | TRUE | C | 167104790 | 2.26E-08 | reported | 2919 | 0.03755 | rs12077809 |  |  |  |  |  |  |  |  |  |  |  |  |  |  |  |
| -0.2162 | 1 | igd | 0.1461 | C | \|\| id:ebi-a-GCST90001864 | ebi-a-GCST90001864 | TRUE | G | 167400081 | 2.58E-08 | reported | 2919 | 0.03873 | rs1052230 |  |  |  |  |  |  |  |  |  |  |  |  |  |  |  |
| 0.6978 | 20 | igd | 0.1461 | A | \|\| id:ebi-a-GCST90001981 | ebi-a-GCST90001981 | TRUE | G | 44747086 | 3.78E-92 | reported | 3618 | 0.03329 | rs745307 |  |  |  |  |  |  |  |  |  |  |  |  |  |  |  |
| -0.2119 | 10 | igd | 0.146 | A | \|\| id:ebi-a-GCST90001483 | ebi-a-GCST90001483 | TRUE | C | 96990275 | 4.12E-09 | reported | 3405 | 0.03595 | rs72820690 |  |  |  |  |  |  |  |  |  |  |  |  |  |  |  |
| -0.2667 | 10 | igd | 0.146 | A | \|\| id:ebi-a-GCST90001489 | ebi-a-GCST90001489 | TRUE | C | 96990275 | 5.44E-13 | reported | 3405 | 0.03683 | rs72820690 |  |  |  |  |  |  |  |  |  |  |  |  |  |  |  |
| -0.2672 | 10 | igd | 0.146 | A | \|\| id:ebi-a-GCST90001495 | ebi-a-GCST90001495 | TRUE | C | 96990275 | 8.73E-13 | reported | 3405 | 0.03723 | rs72820690 |  |  |  |  |  |  |  |  |  |  |  |  |  |  |  |
| 0.6636 | 20 | igd | 0.146 | A | \|\| id:ebi-a-GCST90001980 | ebi-a-GCST90001980 | TRUE | G | 44747086 | 2.07E-83 | reported | 3629 | 0.03341 | rs745307 |  |  |  |  |  |  |  |  |  |  |  |  |  |  |  |
| 0.715 | 20 | igd | 0.146 | A | \|\| id:ebi-a-GCST90001985 | ebi-a-GCST90001985 | TRUE | G | 44747086 | 7.30E-96 | reported | 3629 | 0.0334 | rs745307 |  |  |  |  |  |  |  |  |  |  |  |  |  |  |  |
| -0.2675 | 10 | igd | 0.1458 | A | \|\| id:ebi-a-GCST90001660 | ebi-a-GCST90001660 | TRUE | C | 96990275 | 4.64E-13 | reported | 3408 | 0.03682 | rs72820690 |  |  |  |  |  |  |  |  |  |  |  |  |  |  |  |
| -0.2326 | 10 | igd | 0.1458 | A | \|\| id:ebi-a-GCST90001672 | ebi-a-GCST90001672 | TRUE | C | 96990275 | 4.85E-10 | reported | 3408 | 0.03726 | rs72820690 |  |  |  |  |  |  |  |  |  |  |  |  |  |  |  |
| -0.2197 | 1 | igd | 0.1457 | G | \|\| id:ebi-a-GCST90001471 | ebi-a-GCST90001471 | TRUE | A | 161302354 | 5.26E-10 | reported | 3410 | 0.03527 | rs72714978 |  |  |  |  |  |  |  |  |  |  |  |  |  |  |  |
| -0.405 | 1 | igd | 0.1457 | G | \|\| id:ebi-a-GCST90001474 | ebi-a-GCST90001474 | TRUE | A | 161302354 | 3.32E-29 | reported | 3410 | 0.03576 | rs72714978 |  |  |  |  |  |  |  |  |  |  |  |  |  |  |  |
| -0.3883 | 19 | igd | 0.1456 | T | \|\| id:ebi-a-GCST90001523 | ebi-a-GCST90001523 | TRUE | C | 51695139 | 3.86E-16 | reported | 1858 | 0.04725 | rs12461866 |  |  |  |  |  |  |  |  |  |  |  |  |  |  |  |
| 0.2579 | 1 | igd | 0.1455 | A | \|\| id:ebi-a-GCST90001474 | ebi-a-GCST90001474 | TRUE | G | 161410349 | 1.94E-12 | reported | 3410 | 0.0365 | rs146718864 |  |  |  |  |  |  |  |  |  |  |  |  |  |  |  |
| 0.2931 | 6 | igd | 0.145 | A | \|\| id:ebi-a-GCST90002105 | ebi-a-GCST90002105 | TRUE | G | 32812801 | 1.53E-11 | reported | 2872 | 0.04327 | rs113215190 |  |  |  |  |  |  |  |  |  |  |  |  |  |  |  |
| 0.2538 | 2 | igd | 0.1447 | T | \|\| id:ebi-a-GCST90002102 | ebi-a-GCST90002102 | TRUE | C | 41792322 | 9.09E-12 | reported | 2919 | 0.03706 | rs57865541 |  |  |  |  |  |  |  |  |  |  |  |  |  |  |  |
| -0.2796 | 3 | igd | 0.1442 | A | \|\| id:ebi-a-GCST90002036 | ebi-a-GCST90002036 | TRUE | G | 52743854 | 2.45E-12 | reported | 2871 | 0.03973 | rs34182518 |  |  |  |  |  |  |  |  |  |  |  |  |  |  |  |
| -0.2394 | 1 | igd | 0.1439 | G | \|\| id:ebi-a-GCST90001863 | ebi-a-GCST90001863 | TRUE | C | 166485891 | 3.64E-10 | reported | 2919 | 0.03806 | rs6684311 |  |  |  |  |  |  |  |  |  |  |  |  |  |  |  |
| -0.219 | 6 | igd | 0.1434 | A | \|\| id:ebi-a-GCST90001752 | ebi-a-GCST90001752 | TRUE | C | 32435823 | 7.03E-09 | reported | 3657 | 0.03774 | rs41291806 |  |  |  |  |  |  |  |  |  |  |  |  |  |  |  |
| -0.2869 | 3 | igd | 0.1433 | C | \|\| id:ebi-a-GCST90002036 | ebi-a-GCST90002036 | TRUE | G | 52618362 | 7.02E-13 | reported | 2871 | 0.03978 | rs35272104 |  |  |  |  |  |  |  |  |  |  |  |  |  |  |  |
| -0.2446 | 3 | igd | 0.1432 | A | \|\| id:ebi-a-GCST90002035 | ebi-a-GCST90002035 | TRUE | C | 52716943 | 1.11E-09 | reported | 2871 | 0.04001 | rs71299614 |  |  |  |  |  |  |  |  |  |  |  |  |  |  |  |
| 0.2256 | 1 | igd | 0.1429 | C | \|\| id:ebi-a-GCST90001858 | ebi-a-GCST90001858 | TRUE | T | 166946072 | 1.10E-08 | reported | 2919 | 0.03937 | rs6699159 |  |  |  |  |  |  |  |  |  |  |  |  |  |  |  |
| -0.4367 | 1 | igd | 0.1428 | C | \|\| id:ebi-a-GCST90001989 | ebi-a-GCST90001989 | TRUE | T | 161470274 | 3.83E-33 | reported | 3621 | 0.03605 | rs111994823 |  |  |  |  |  |  |  |  |  |  |  |  |  |  |  |
| -0.2824 | 1 | igd | 0.1426 | C | \|\| id:ebi-a-GCST90001985 | ebi-a-GCST90001985 | TRUE | T | 161470274 | 1.03E-14 | reported | 3629 | 0.03635 | rs111994823 |  |  |  |  |  |  |  |  |  |  |  |  |  |  |  |
| -0.2303 | 6 | igd | 0.1423 | A | \|\| id:ebi-a-GCST90002119 | ebi-a-GCST90002119 | TRUE | G | 32097876 | 4.47E-08 | reported | 2920 | 0.04198 | rs169494 |  |  |  |  |  |  |  |  |  |  |  |  |  |  |  |
| -0.2293 | 1 | igd | 0.1422 | G | \|\| id:ebi-a-GCST90001858 | ebi-a-GCST90001858 | TRUE | A | 167464442 | 5.86E-09 | reported | 2919 | 0.03928 | rs35307794 |  |  |  |  |  |  |  |  |  |  |  |  |  |  |  |
| -0.2146 | 10 | igd | 0.142 | G | \|\| id:ebi-a-GCST90001496 | ebi-a-GCST90001496 | TRUE | T | 94855135 | 4.93E-09 | reported | 3437 | 0.0366 | rs11187277 |  |  |  |  |  |  |  |  |  |  |  |  |  |  |  |
| -0.2062 | 10 | igd | 0.142 | G | \|\| id:ebi-a-GCST90001497 | ebi-a-GCST90001497 | TRUE | T | 94855135 | 1.26E-08 | reported | 3437 | 0.03615 | rs11187277 |  |  |  |  |  |  |  |  |  |  |  |  |  |  |  |
| 0.289 | 10 | igd | 0.1419 | A | \|\| id:ebi-a-GCST90001670 | ebi-a-GCST90001670 | TRUE | G | 95163883 | 2.22E-15 | reported | 3440 | 0.03628 | rs56041929 |  |  |  |  |  |  |  |  |  |  |  |  |  |  |  |
| 0.2888 | 10 | igd | 0.1419 | A | \|\| id:ebi-a-GCST90001671 | ebi-a-GCST90001671 | TRUE | G | 95163883 | 2.50E-15 | reported | 3440 | 0.03632 | rs56041929 |  |  |  |  |  |  |  |  |  |  |  |  |  |  |  |
| 0.2404 | 2 | igd | 0.1417 | A | \|\| id:ebi-a-GCST90002102 | ebi-a-GCST90002102 | TRUE | G | 39000659 | 6.04E-11 | reported | 2919 | 0.03661 | rs3112194 |  |  |  |  |  |  |  |  |  |  |  |  |  |  |  |
| -0.3402 | 22 | igd | 0.1416 | A | \|\| id:ebi-a-GCST90001702 | ebi-a-GCST90001702 | TRUE | G | 42874781 | 4.12E-21 | reported | 3657 | 0.03585 | rs2142827 |  |  |  |  |  |  |  |  |  |  |  |  |  |  |  |
| -0.3265 | 22 | igd | 0.1416 | A | \|\| id:ebi-a-GCST90001703 | ebi-a-GCST90001703 | TRUE | G | 42874781 | 1.48E-19 | reported | 3657 | 0.03588 | rs2142827 |  |  |  |  |  |  |  |  |  |  |  |  |  |  |  |
| -0.2915 | 22 | igd | 0.1416 | A | \|\| id:ebi-a-GCST90001704 | ebi-a-GCST90001704 | TRUE | G | 42874781 | 6.59E-16 | reported | 3657 | 0.03592 | rs2142827 |  |  |  |  |  |  |  |  |  |  |  |  |  |  |  |
| -0.3216 | 22 | igd | 0.1416 | A | \|\| id:ebi-a-GCST90001710 | ebi-a-GCST90001710 | TRUE | G | 42874781 | 4.11E-19 | reported | 3657 | 0.0358 | rs2142827 |  |  |  |  |  |  |  |  |  |  |  |  |  |  |  |
| -0.3302 | 22 | igd | 0.1416 | A | \|\| id:ebi-a-GCST90001711 | ebi-a-GCST90001711 | TRUE | G | 42874781 | 3.66E-20 | reported | 3657 | 0.03569 | rs2142827 |  |  |  |  |  |  |  |  |  |  |  |  |  |  |  |
| 0.2682 | 3 | igd | 0.1415 | A | \|\| id:ebi-a-GCST90001798 | ebi-a-GCST90001798 | TRUE | G | 32652184 | 6.11E-14 | reported | 3657 | 0.03559 | rs62250779 |  |  |  |  |  |  |  |  |  |  |  |  |  |  |  |
| 0.2947 | 3 | igd | 0.1415 | A | \|\| id:ebi-a-GCST90001800 | ebi-a-GCST90001800 | TRUE | G | 32652184 | 4.13E-16 | reported | 3656 | 0.03607 | rs62250779 |  |  |  |  |  |  |  |  |  |  |  |  |  |  |  |
| 0.2224 | 3 | igd | 0.1415 | A | \|\| id:ebi-a-GCST90001804 | ebi-a-GCST90001804 | TRUE | G | 32652184 | 4.23E-10 | reported | 3657 | 0.03552 | rs62250779 |  |  |  |  |  |  |  |  |  |  |  |  |  |  |  |
| 0.2501 | 3 | igd | 0.1415 | A | \|\| id:ebi-a-GCST90001805 | ebi-a-GCST90001805 | TRUE | G | 32652184 | 4.31E-12 | reported | 3657 | 0.03598 | rs62250779 |  |  |  |  |  |  |  |  |  |  |  |  |  |  |  |
| 0.2927 | 3 | igd | 0.1415 | A | \|\| id:ebi-a-GCST90001806 | ebi-a-GCST90001806 | TRUE | G | 32652184 | 4.69E-16 | reported | 3656 | 0.03588 | rs62250779 |  |  |  |  |  |  |  |  |  |  |  |  |  |  |  |
| 0.2343 | 3 | igd | 0.1415 | C | \|\| id:ebi-a-GCST90002013 | ebi-a-GCST90002013 | TRUE | T | 46406578 | 8.78E-09 | reported | 2870 | 0.04061 | rs2373227 |  |  |  |  |  |  |  |  |  |  |  |  |  |  |  |
| 0.2875 | 10 | igd | 0.1413 | A | \|\| id:ebi-a-GCST90001672 | ebi-a-GCST90001672 | TRUE | G | 95163883 | 9.50E-15 | reported | 3408 | 0.03696 | rs56041929 |  |  |  |  |  |  |  |  |  |  |  |  |  |  |  |
| -0.4204 | 1 | igd | 0.1412 | T | \|\| id:ebi-a-GCST90002077 | ebi-a-GCST90002077 | TRUE | C | 161291228 | 7.12E-28 | reported | 2971 | 0.03803 | rs72714967 |  |  |  |  |  |  |  |  |  |  |  |  |  |  |  |
| -0.3317 | 10 | igd | 0.1408 | A | \|\| id:ebi-a-GCST90001660 | ebi-a-GCST90001660 | TRUE | G | 97988763 | 1.76E-19 | reported | 3408 | 0.03652 | rs11188673 |  |  |  |  |  |  |  |  |  |  |  |  |  |  |  |
| -0.4144 | 10 | igd | 0.1408 | A | \|\| id:ebi-a-GCST90002030 | ebi-a-GCST90002030 | TRUE | G | 97988763 | 4.01E-26 | reported | 2920 | 0.03882 | rs11188673 |  |  |  |  |  |  |  |  |  |  |  |  |  |  |  |
| -0.3471 | 10 | igd | 0.1408 | A | \|\| id:ebi-a-GCST90002031 | ebi-a-GCST90002031 | TRUE | G | 97988763 | 7.59E-18 | reported | 2920 | 0.04007 | rs11188673 |  |  |  |  |  |  |  |  |  |  |  |  |  |  |  |
| -0.368 | 10 | igd | 0.1408 | A | \|\| id:ebi-a-GCST90002032 | ebi-a-GCST90002032 | TRUE | G | 97988763 | 6.24E-20 | reported | 2920 | 0.03997 | rs11188673 |  |  |  |  |  |  |  |  |  |  |  |  |  |  |  |
| -0.2902 | 2 | igd | 0.1408 | T | \|\| id:ebi-a-GCST90002121 | ebi-a-GCST90002121 | TRUE | G | 86872244 | 1.29E-13 | reported | 2915 | 0.03899 | rs1437615 |  |  |  |  |  |  |  |  |  |  |  |  |  |  |  |
| -0.2695 | 10 | igd | 0.1407 | A | \|\| id:ebi-a-GCST90001483 | ebi-a-GCST90001483 | TRUE | G | 97988763 | 8.07E-14 | reported | 3405 | 0.03593 | rs11188673 |  |  |  |  |  |  |  |  |  |  |  |  |  |  |  |
| -0.3252 | 10 | igd | 0.1407 | A | \|\| id:ebi-a-GCST90001489 | ebi-a-GCST90001489 | TRUE | G | 97988763 | 1.51E-18 | reported | 3405 | 0.03679 | rs11188673 |  |  |  |  |  |  |  |  |  |  |  |  |  |  |  |
| -0.3468 | 10 | igd | 0.1407 | A | \|\| id:ebi-a-GCST90001495 | ebi-a-GCST90001495 | TRUE | G | 97988763 | 1.20E-20 | reported | 3405 | 0.03698 | rs11188673 |  |  |  |  |  |  |  |  |  |  |  |  |  |  |  |
| -0.4528 | 22 | igd | 0.1406 | A | \|\| id:ebi-a-GCST90001702 | ebi-a-GCST90001702 | TRUE | G | 43390319 | 1.47E-35 | reported | 3657 | 0.03599 | rs2018340 |  |  |  |  |  |  |  |  |  |  |  |  |  |  |  |
| -0.4511 | 22 | igd | 0.1406 | A | \|\| id:ebi-a-GCST90001703 | ebi-a-GCST90001703 | TRUE | G | 43393437 | 2.63E-35 | reported | 3657 | 0.036 | rs5759079 |  |  |  |  |  |  |  |  |  |  |  |  |  |  |  |
| -0.4296 | 22 | igd | 0.1406 | A | \|\| id:ebi-a-GCST90001704 | ebi-a-GCST90001704 | TRUE | G | 43393437 | 3.27E-32 | reported | 3657 | 0.03601 | rs5759079 |  |  |  |  |  |  |  |  |  |  |  |  |  |  |  |
| -0.4454 | 22 | igd | 0.1406 | A | \|\| id:ebi-a-GCST90001710 | ebi-a-GCST90001710 | TRUE | G | 43390319 | 1.34E-34 | reported | 3657 | 0.03593 | rs2018340 |  |  |  |  |  |  |  |  |  |  |  |  |  |  |  |
| -0.4443 | 22 | igd | 0.1406 | A | \|\| id:ebi-a-GCST90001711 | ebi-a-GCST90001711 | TRUE | G | 43393437 | 1.27E-34 | reported | 3657 | 0.03583 | rs5759079 |  |  |  |  |  |  |  |  |  |  |  |  |  |  |  |
| 0.3727 | 3 | igd | 0.1405 | A | \|\| id:ebi-a-GCST90001801 | ebi-a-GCST90001801 | TRUE | G | 32652184 | 1.70E-14 | reported | 1836 | 0.04819 | rs62250779 |  |  |  |  |  |  |  |  |  |  |  |  |  |  |  |
| -0.3308 | 10 | igd | 0.1403 | A | \|\| id:ebi-a-GCST90001658 | ebi-a-GCST90001658 | TRUE | G | 97988763 | 4.13E-21 | reported | 3440 | 0.03485 | rs11188673 |  |  |  |  |  |  |  |  |  |  |  |  |  |  |  |
| -0.3295 | 10 | igd | 0.1403 | A | \|\| id:ebi-a-GCST90001659 | ebi-a-GCST90001659 | TRUE | G | 97988763 | 6.01E-21 | reported | 3440 | 0.03486 | rs11188673 |  |  |  |  |  |  |  |  |  |  |  |  |  |  |  |
| -0.3208 | 10 | igd | 0.1401 | A | \|\| id:ebi-a-GCST90001491 | ebi-a-GCST90001491 | TRUE | G | 97988763 | 1.16E-18 | reported | 3437 | 0.03617 | rs11188673 |  |  |  |  |  |  |  |  |  |  |  |  |  |  |  |
| -0.3523 | 10 | igd | 0.1401 | A | \|\| id:ebi-a-GCST90001496 | ebi-a-GCST90001496 | TRUE | G | 97988763 | 1.13E-21 | reported | 3437 | 0.03659 | rs11188673 |  |  |  |  |  |  |  |  |  |  |  |  |  |  |  |
| -0.3437 | 10 | igd | 0.1401 | A | \|\| id:ebi-a-GCST90001497 | ebi-a-GCST90001497 | TRUE | G | 97988763 | 3.44E-21 | reported | 3437 | 0.03614 | rs11188673 |  |  |  |  |  |  |  |  |  |  |  |  |  |  |  |
| -0.291 | 16 | igd | 0.1398 | T | \|\| id:ebi-a-GCST90002105 | ebi-a-GCST90002105 | TRUE | C | 10863414 | 1.10E-13 | reported | 2872 | 0.03898 | rs11644019 |  |  |  |  |  |  |  |  |  |  |  |  |  |  |  |
| -0.4419 | 2 | igd | 0.1397 | G | \|\| id:ebi-a-GCST90002118 | ebi-a-GCST90002118 | TRUE | A | 86874374 | 1.39E-29 | reported | 2920 | 0.03869 | rs6547701 |  |  |  |  |  |  |  |  |  |  |  |  |  |  |  |
| -0.2527 | 2 | igd | 0.1397 | G | \|\| id:ebi-a-GCST90002120 | ebi-a-GCST90002120 | TRUE | A | 86874374 | 1.92E-10 | reported | 2920 | 0.03954 | rs6547701 |  |  |  |  |  |  |  |  |  |  |  |  |  |  |  |
| -0.2868 | 22 | igd | 0.1396 | A | \|\| id:ebi-a-GCST90001702 | ebi-a-GCST90001702 | TRUE | G | 40596384 | 3.00E-15 | reported | 3657 | 0.03619 | rs142949835 |  |  |  |  |  |  |  |  |  |  |  |  |  |  |  |
| -0.2926 | 22 | igd | 0.1396 | A | \|\| id:ebi-a-GCST90001703 | ebi-a-GCST90001703 | TRUE | G | 40596384 | 8.09E-16 | reported | 3657 | 0.03618 | rs142949835 |  |  |  |  |  |  |  |  |  |  |  |  |  |  |  |
| -0.2667 | 22 | igd | 0.1396 | A | \|\| id:ebi-a-GCST90001710 | ebi-a-GCST90001710 | TRUE | G | 40596384 | 1.92E-13 | reported | 3657 | 0.03614 | rs142949835 |  |  |  |  |  |  |  |  |  |  |  |  |  |  |  |
| 0.2069 | 10 | igd | 0.1395 | A | \|\| id:ebi-a-GCST90001672 | ebi-a-GCST90001672 | TRUE | G | 93654244 | 4.95E-08 | reported | 3408 | 0.03785 | rs117332124 |  |  |  |  |  |  |  |  |  |  |  |  |  |  |  |
| -0.6351 | 2 | igd | 0.1394 | G | \|\| id:ebi-a-GCST90002119 | ebi-a-GCST90002119 | TRUE | A | 86877960 | 6.50E-63 | reported | 2920 | 0.03702 | rs61286004 |  |  |  |  |  |  |  |  |  |  |  |  |  |  |  |
| 0.1977 | 2 | igd | 0.1392 | G | \|\| id:ebi-a-GCST90001665 | ebi-a-GCST90001665 | TRUE | A | 86890742 | 3.95E-08 | reported | 3440 | 0.03592 | rs113822514 |  |  |  |  |  |  |  |  |  |  |  |  |  |  |  |
| -0.2124 | 2 | igd | 0.1387 | T | \|\| id:ebi-a-GCST90002100 | ebi-a-GCST90002100 | TRUE | A | 38433680 | 4.72E-08 | reported | 2910 | 0.03879 | rs10865135 |  |  |  |  |  |  |  |  |  |  |  |  |  |  |  |
| -0.195 | 3 | igd | 0.1383 | C | \|\| id:ebi-a-GCST90001994 | ebi-a-GCST90001994 | TRUE | T | 39182259 | 3.89E-08 | reported | 3590 | 0.0354 | rs7611953 |  |  |  |  |  |  |  |  |  |  |  |  |  |  |  |
| -0.3076 | 3 | igd | 0.1383 | C | \|\| id:ebi-a-GCST90001997 | ebi-a-GCST90001997 | TRUE | T | 39182259 | 1.82E-17 | reported | 3590 | 0.03599 | rs7611953 |  |  |  |  |  |  |  |  |  |  |  |  |  |  |  |
| -0.2912 | 3 | igd | 0.1383 | C | \|\| id:ebi-a-GCST90001995 | ebi-a-GCST90001995 | TRUE | T | 39182259 | 5.37E-16 | reported | 3590 | 0.03577 | rs7611953 |  |  |  |  |  |  |  |  |  |  |  |  |  |  |  |
| -0.2062 | 3 | igd | 0.1382 | C | \|\| id:ebi-a-GCST90001996 | ebi-a-GCST90001996 | TRUE | T | 39182259 | 1.03E-08 | reported | 3579 | 0.03593 | rs7611953 |  |  |  |  |  |  |  |  |  |  |  |  |  |  |  |
| -0.2686 | 6 | igd | 0.1378 | T | \|\| id:ebi-a-GCST90002118 | ebi-a-GCST90002118 | TRUE | G | 29765452 | 3.02E-09 | reported | 2920 | 0.04515 | rs9461556 |  |  |  |  |  |  |  |  |  |  |  |  |  |  |  |
| -0.2328 | 3 | igd | 0.1375 | C | \|\| id:ebi-a-GCST90001996 | ebi-a-GCST90001996 | TRUE | G | 39248253 | 1.62E-10 | reported | 3579 | 0.0363 | rs4676613 |  |  |  |  |  |  |  |  |  |  |  |  |  |  |  |
| 0.2832 | 3 | igd | 0.1374 | T | \|\| id:ebi-a-GCST90002015 | ebi-a-GCST90002015 | TRUE | C | 46350538 | 1.94E-12 | reported | 2871 | 0.04006 | rs12494200 |  |  |  |  |  |  |  |  |  |  |  |  |  |  |  |
| 0.2821 | 3 | igd | 0.1374 | T | \|\| id:ebi-a-GCST90002016 | ebi-a-GCST90002016 | TRUE | C | 46350538 | 2.16E-12 | reported | 2871 | 0.03999 | rs12494200 |  |  |  |  |  |  |  |  |  |  |  |  |  |  |  |
| -0.2731 | 6 | igd | 0.1374 | T | \|\| id:ebi-a-GCST90002121 | ebi-a-GCST90002121 | TRUE | G | 31019810 | 5.91E-11 | reported | 2915 | 0.04156 | rs12214258 |  |  |  |  |  |  |  |  |  |  |  |  |  |  |  |
| -0.2562 | 6 | igd | 0.1373 | T | \|\| id:ebi-a-GCST90001932 | ebi-a-GCST90001932 | TRUE | G | 31019810 | 3.59E-09 | reported | 2920 | 0.04328 | rs12214258 |  |  |  |  |  |  |  |  |  |  |  |  |  |  |  |
| -0.3256 | 6 | igd | 0.1373 | T | \|\| id:ebi-a-GCST90002119 | ebi-a-GCST90002119 | TRUE | G | 31019810 | 2.01E-13 | reported | 2920 | 0.04411 | rs12214258 |  |  |  |  |  |  |  |  |  |  |  |  |  |  |  |
| 0.3325 | 6 | igd | 0.1371 | T | \|\| id:ebi-a-GCST90002108 | ebi-a-GCST90002108 | TRUE | C | 32814456 | 7.73E-09 | reported | 1579 | 0.05727 | rs59989068 |  |  |  |  |  |  |  |  |  |  |  |  |  |  |  |
| 0.2416 | 1 | igd | 0.137 | G | \|\| id:ebi-a-GCST90002077 | ebi-a-GCST90002077 | TRUE | A | 161184097 | 7.46E-10 | reported | 2971 | 0.03912 | rs1136224 |  |  |  |  |  |  |  |  |  |  |  |  |  |  |  |
| -0.2258 | 1 | igd | 0.1369 | T | \|\| id:ebi-a-GCST90001845 | ebi-a-GCST90001845 | TRUE | C | 167160080 | 1.85E-08 | reported | 2911 | 0.04002 | rs10918663 |  |  |  |  |  |  |  |  |  |  |  |  |  |  |  |
| -0.2301 | 1 | igd | 0.1365 | T | \|\| id:ebi-a-GCST90001854 | ebi-a-GCST90001854 | TRUE | C | 167160080 | 7.27E-09 | reported | 2919 | 0.03967 | rs10918663 |  |  |  |  |  |  |  |  |  |  |  |  |  |  |  |
| -0.2782 | 1 | igd | 0.1365 | T | \|\| id:ebi-a-GCST90001858 | ebi-a-GCST90001858 | TRUE | C | 167160080 | 3.74E-12 | reported | 2919 | 0.03988 | rs10918663 |  |  |  |  |  |  |  |  |  |  |  |  |  |  |  |
| -0.2464 | 1 | igd | 0.1365 | T | \|\| id:ebi-a-GCST90001860 | ebi-a-GCST90001860 | TRUE | C | 167160080 | 9.23E-10 | reported | 2919 | 0.04012 | rs10918663 |  |  |  |  |  |  |  |  |  |  |  |  |  |  |  |
| -0.2532 | 1 | igd | 0.1365 | T | \|\| id:ebi-a-GCST90001861 | ebi-a-GCST90001861 | TRUE | C | 167160080 | 1.71E-10 | reported | 2919 | 0.03951 | rs10918663 |  |  |  |  |  |  |  |  |  |  |  |  |  |  |  |
| -0.2281 | 1 | igd | 0.1365 | T | \|\| id:ebi-a-GCST90001863 | ebi-a-GCST90001863 | TRUE | C | 167160080 | 7.01E-09 | reported | 2919 | 0.03928 | rs10918663 |  |  |  |  |  |  |  |  |  |  |  |  |  |  |  |
| -0.3048 | 1 | igd | 0.1365 | T | \|\| id:ebi-a-GCST90001864 | ebi-a-GCST90001864 | TRUE | C | 167160080 | 2.41E-14 | reported | 2919 | 0.03976 | rs10918663 |  |  |  |  |  |  |  |  |  |  |  |  |  |  |  |
| 0.3472 | 6 | igd | 0.1365 | C | \|\| id:ebi-a-GCST90002110 | ebi-a-GCST90002110 | TRUE | T | 32685344 | 1.70E-09 | reported | 1634 | 0.05731 | rs112357706 |  |  |  |  |  |  |  |  |  |  |  |  |  |  |  |
| 0.2592 | 10 | igd | 0.1363 | C | \|\| id:ebi-a-GCST90001483 | ebi-a-GCST90001483 | TRUE | T | 95782925 | 9.33E-13 | reported | 3405 | 0.03616 | rs1935960 |  |  |  |  |  |  |  |  |  |  |  |  |  |  |  |
| 0.2769 | 10 | igd | 0.1363 | T | \|\| id:ebi-a-GCST90001496 | ebi-a-GCST90001496 | TRUE | A | 95969913 | 2.05E-13 | reported | 3437 | 0.03754 | rs10509669 |  |  |  |  |  |  |  |  |  |  |  |  |  |  |  |
| 0.2918 | 10 | igd | 0.1362 | C | \|\| id:ebi-a-GCST90001485 | ebi-a-GCST90001485 | TRUE | T | 95782925 | 4.85E-16 | reported | 3437 | 0.03579 | rs1935960 |  |  |  |  |  |  |  |  |  |  |  |  |  |  |  |
| 0.2002 | 22 | igd | 0.1356 | G | \|\| id:ebi-a-GCST90001702 | ebi-a-GCST90001702 | TRUE | A | 44310518 | 3.49E-08 | reported | 3657 | 0.03623 | rs74739848 |  |  |  |  |  |  |  |  |  |  |  |  |  |  |  |
| 0.2021 | 22 | igd | 0.1356 | G | \|\| id:ebi-a-GCST90001703 | ebi-a-GCST90001703 | TRUE | A | 44310518 | 2.60E-08 | reported | 3657 | 0.03622 | rs74739848 |  |  |  |  |  |  |  |  |  |  |  |  |  |  |  |
| 0.2061 | 10 | igd | 0.1356 | A | \|\| id:ebi-a-GCST90001976 | ebi-a-GCST90001976 | TRUE | G | 126319724 | 4.68E-08 | reported | 3113 | 0.03764 | rs75360179 |  |  |  |  |  |  |  |  |  |  |  |  |  |  |  |
| 0.3498 | 6 | igd | 0.1355 | C | \|\| id:ebi-a-GCST90002108 | ebi-a-GCST90002108 | TRUE | T | 32685344 | 1.59E-09 | reported | 1579 | 0.05762 | rs112357706 |  |  |  |  |  |  |  |  |  |  |  |  |  |  |  |
| 0.2648 | 6 | igd | 0.1354 | C | \|\| id:ebi-a-GCST90002105 | ebi-a-GCST90002105 | TRUE | T | 31327757 | 3.63E-09 | reported | 2872 | 0.04473 | rs28695590 |  |  |  |  |  |  |  |  |  |  |  |  |  |  |  |
| -0.225 | 1 | igd | 0.1352 | A | \|\| id:ebi-a-GCST90001989 | ebi-a-GCST90001989 | TRUE | G | 161570541 | 3.41E-09 | reported | 3621 | 0.03797 | rs143450775 |  |  |  |  |  |  |  |  |  |  |  |  |  |  |  |
| -0.2176 | 10 | igd | 0.135 | C | \|\| id:ebi-a-GCST90001670 | ebi-a-GCST90001670 | TRUE | T | 93344254 | 5.79E-09 | reported | 3440 | 0.03727 | rs10881926 |  |  |  |  |  |  |  |  |  |  |  |  |  |  |  |
| -0.2181 | 10 | igd | 0.135 | C | \|\| id:ebi-a-GCST90001671 | ebi-a-GCST90001671 | TRUE | T | 93344254 | 5.78E-09 | reported | 3440 | 0.03735 | rs10881926 |  |  |  |  |  |  |  |  |  |  |  |  |  |  |  |
| 0.599 | 10 | igd | 0.1347 | A | \|\| id:ebi-a-GCST90001485 | ebi-a-GCST90001485 | TRUE | G | 97667422 | 1.71E-62 | reported | 3437 | 0.03518 | rs10882685 |  |  |  |  |  |  |  |  |  |  |  |  |  |  |  |
| 0.2713 | 6 | igd | 0.1347 | C | \|\| id:ebi-a-GCST90002105 | ebi-a-GCST90002105 | TRUE | T | 32685344 | 3.40E-09 | reported | 2872 | 0.04576 | rs112357706 |  |  |  |  |  |  |  |  |  |  |  |  |  |  |  |
| -0.223 | 10 | igd | 0.1346 | C | \|\| id:ebi-a-GCST90002029 | ebi-a-GCST90002029 | TRUE | G | 97961811 | 1.87E-08 | reported | 2915 | 0.03954 | rs12779295 |  |  |  |  |  |  |  |  |  |  |  |  |  |  |  |
| -0.3992 | 10 | igd | 0.1343 | G | \|\| id:ebi-a-GCST90001485 | ebi-a-GCST90001485 | TRUE | A | 97671022 | 2.60E-27 | reported | 3437 | 0.03656 | rs11596697 |  |  |  |  |  |  |  |  |  |  |  |  |  |  |  |
| -0.2103 | 10 | igd | 0.1343 | G | \|\| id:ebi-a-GCST90001491 | ebi-a-GCST90001491 | TRUE | A | 97805908 | 2.13E-08 | reported | 3437 | 0.03745 | rs57334361 |  |  |  |  |  |  |  |  |  |  |  |  |  |  |  |
| -0.2435 | 17 | igd | 0.1343 | C | \|\| id:ebi-a-GCST90001653 | ebi-a-GCST90001653 | TRUE | T | 33814758 | 6.13E-11 | reported | 3440 | 0.0371 | rs4796089 |  |  |  |  |  |  |  |  |  |  |  |  |  |  |  |
| 0.2435 | 17 | igd | 0.1343 | C | \|\| id:ebi-a-GCST90001656 | ebi-a-GCST90001656 | TRUE | T | 33814758 | 6.13E-11 | reported | 3440 | 0.0371 | rs4796089 |  |  |  |  |  |  |  |  |  |  |  |  |  |  |  |
| -0.2225 | 17 | igd | 0.1343 | C | \|\| id:ebi-a-GCST90001661 | ebi-a-GCST90001661 | TRUE | T | 33814758 | 3.91E-09 | reported | 3440 | 0.0377 | rs4796089 |  |  |  |  |  |  |  |  |  |  |  |  |  |  |  |
| -0.2283 | 17 | igd | 0.1343 | C | \|\| id:ebi-a-GCST90001662 | ebi-a-GCST90001662 | TRUE | T | 33814758 | 4.32E-10 | reported | 3440 | 0.03646 | rs4796089 |  |  |  |  |  |  |  |  |  |  |  |  |  |  |  |
| -0.2034 | 17 | igd | 0.1343 | C | \|\| id:ebi-a-GCST90001686 | ebi-a-GCST90001686 | TRUE | T | 33814758 | 1.35E-09 | reported | 3440 | 0.03346 | rs4796089 |  |  |  |  |  |  |  |  |  |  |  |  |  |  |  |
| -0.3974 | 10 | igd | 0.1342 | G | \|\| id:ebi-a-GCST90001483 | ebi-a-GCST90001483 | TRUE | A | 97671022 | 1.54E-26 | reported | 3405 | 0.03696 | rs11596697 |  |  |  |  |  |  |  |  |  |  |  |  |  |  |  |
| -0.3062 | 16 | igd | 0.1342 | C | \|\| id:ebi-a-GCST90002090 | ebi-a-GCST90002090 | TRUE | T | 31059325 | 3.77E-13 | reported | 2850 | 0.04195 | rs72785511 |  |  |  |  |  |  |  |  |  |  |  |  |  |  |  |
| -0.4439 | 10 | igd | 0.134 | T | \|\| id:ebi-a-GCST90001484 | ebi-a-GCST90001484 | TRUE | C | 97370600 | 2.13E-34 | reported | 3437 | 0.0359 | rs483920 |  |  |  |  |  |  |  |  |  |  |  |  |  |  |  |
| -0.4985 | 10 | igd | 0.134 | T | \|\| id:ebi-a-GCST90001491 | ebi-a-GCST90001491 | TRUE | C | 97370600 | 3.48E-41 | reported | 3437 | 0.0366 | rs483920 |  |  |  |  |  |  |  |  |  |  |  |  |  |  |  |
| -0.5623 | 10 | igd | 0.134 | T | \|\| id:ebi-a-GCST90001496 | ebi-a-GCST90001496 | TRUE | C | 97370600 | 6.63E-51 | reported | 3437 | 0.03685 | rs483920 |  |  |  |  |  |  |  |  |  |  |  |  |  |  |  |
| -0.561 | 10 | igd | 0.134 | T | \|\| id:ebi-a-GCST90001497 | ebi-a-GCST90001497 | TRUE | C | 97370600 | 5.58E-52 | reported | 3437 | 0.03636 | rs483920 |  |  |  |  |  |  |  |  |  |  |  |  |  |  |  |
| -0.5551 | 10 | igd | 0.1339 | T | \|\| id:ebi-a-GCST90001658 | ebi-a-GCST90001658 | TRUE | C | 97370600 | 2.08E-54 | reported | 3440 | 0.03511 | rs483920 |  |  |  |  |  |  |  |  |  |  |  |  |  |  |  |
| -0.5622 | 10 | igd | 0.1339 | T | \|\| id:ebi-a-GCST90001659 | ebi-a-GCST90001659 | TRUE | C | 97370600 | 8.57E-56 | reported | 3440 | 0.03508 | rs483920 |  |  |  |  |  |  |  |  |  |  |  |  |  |  |  |
| -0.2344 | 17 | igd | 0.1339 | C | \|\| id:ebi-a-GCST90001663 | ebi-a-GCST90001663 | TRUE | T | 33814758 | 7.21E-10 | reported | 3408 | 0.03793 | rs4796089 |  |  |  |  |  |  |  |  |  |  |  |  |  |  |  |
| -0.5656 | 10 | igd | 0.1339 | T | \|\| id:ebi-a-GCST90001670 | ebi-a-GCST90001670 | TRUE | C | 97370600 | 2.68E-52 | reported | 3440 | 0.03654 | rs483920 |  |  |  |  |  |  |  |  |  |  |  |  |  |  |  |
| -0.5567 | 10 | igd | 0.1339 | T | \|\| id:ebi-a-GCST90001671 | ebi-a-GCST90001671 | TRUE | C | 97370600 | 2.30E-50 | reported | 3440 | 0.03669 | rs483920 |  |  |  |  |  |  |  |  |  |  |  |  |  |  |  |
| -0.2103 | 17 | igd | 0.1339 | C | \|\| id:ebi-a-GCST90001687 | ebi-a-GCST90001687 | TRUE | T | 33814758 | 1.90E-08 | reported | 3408 | 0.03733 | rs4796089 |  |  |  |  |  |  |  |  |  |  |  |  |  |  |  |
| 0.6376 | 10 | igd | 0.1337 | T | \|\| id:ebi-a-GCST90001484 | ebi-a-GCST90001484 | TRUE | A | 97685914 | 3.33E-70 | reported | 3437 | 0.03517 | rs11819376 |  |  |  |  |  |  |  |  |  |  |  |  |  |  |  |
| 0.6051 | 10 | igd | 0.1337 | T | \|\| id:ebi-a-GCST90001491 | ebi-a-GCST90001491 | TRUE | A | 97685914 | 3.28E-60 | reported | 3437 | 0.03625 | rs11819376 |  |  |  |  |  |  |  |  |  |  |  |  |  |  |  |
| 0.687 | 10 | igd | 0.1337 | T | \|\| id:ebi-a-GCST90001496 | ebi-a-GCST90001496 | TRUE | A | 97685914 | 7.59E-76 | reported | 3437 | 0.03635 | rs11819376 |  |  |  |  |  |  |  |  |  |  |  |  |  |  |  |
| 0.6667 | 10 | igd | 0.1337 | T | \|\| id:ebi-a-GCST90001497 | ebi-a-GCST90001497 | TRUE | A | 97685914 | 3.31E-73 | reported | 3437 | 0.03596 | rs11819376 |  |  |  |  |  |  |  |  |  |  |  |  |  |  |  |
| 0.6623 | 10 | igd | 0.1337 | T | \|\| id:ebi-a-GCST90001658 | ebi-a-GCST90001658 | TRUE | A | 97685914 | 1.48E-77 | reported | 3440 | 0.03463 | rs11819376 |  |  |  |  |  |  |  |  |  |  |  |  |  |  |  |
| 0.6726 | 10 | igd | 0.1337 | T | \|\| id:ebi-a-GCST90001659 | ebi-a-GCST90001659 | TRUE | A | 97685914 | 4.95E-80 | reported | 3440 | 0.03458 | rs11819376 |  |  |  |  |  |  |  |  |  |  |  |  |  |  |  |
| 0.5531 | 10 | igd | 0.1337 | T | \|\| id:ebi-a-GCST90001670 | ebi-a-GCST90001670 | TRUE | A | 97685914 | 7.86E-50 | reported | 3440 | 0.03667 | rs11819376 |  |  |  |  |  |  |  |  |  |  |  |  |  |  |  |
| 0.5634 | 10 | igd | 0.1337 | T | \|\| id:ebi-a-GCST90001671 | ebi-a-GCST90001671 | TRUE | A | 97685914 | 2.07E-51 | reported | 3440 | 0.03673 | rs11819376 |  |  |  |  |  |  |  |  |  |  |  |  |  |  |  |
| -0.3988 | 10 | igd | 0.1336 | T | \|\| id:ebi-a-GCST90001483 | ebi-a-GCST90001483 | TRUE | C | 97370600 | 1.92E-27 | reported | 3405 | 0.03642 | rs483920 |  |  |  |  |  |  |  |  |  |  |  |  |  |  |  |
| -0.53 | 10 | igd | 0.1336 | T | \|\| id:ebi-a-GCST90001495 | ebi-a-GCST90001495 | TRUE | C | 97370600 | 3.00E-44 | reported | 3405 | 0.03744 | rs483920 |  |  |  |  |  |  |  |  |  |  |  |  |  |  |  |
| -0.5645 | 10 | igd | 0.1335 | T | \|\| id:ebi-a-GCST90001660 | ebi-a-GCST90001660 | TRUE | C | 97370600 | 3.56E-51 | reported | 3408 | 0.03689 | rs483920 |  |  |  |  |  |  |  |  |  |  |  |  |  |  |  |
| -0.5654 | 10 | igd | 0.1335 | T | \|\| id:ebi-a-GCST90001672 | ebi-a-GCST90001672 | TRUE | C | 97370600 | 2.40E-50 | reported | 3408 | 0.03727 | rs483920 |  |  |  |  |  |  |  |  |  |  |  |  |  |  |  |
| 0.6635 | 10 | igd | 0.1334 | T | \|\| id:ebi-a-GCST90001660 | ebi-a-GCST90001660 | TRUE | A | 97685914 | 1.59E-70 | reported | 3408 | 0.03651 | rs11819376 |  |  |  |  |  |  |  |  |  |  |  |  |  |  |  |
| 0.5577 | 10 | igd | 0.1334 | T | \|\| id:ebi-a-GCST90001672 | ebi-a-GCST90001672 | TRUE | A | 97685914 | 9.86E-49 | reported | 3408 | 0.03741 | rs11819376 |  |  |  |  |  |  |  |  |  |  |  |  |  |  |  |
| 0.566 | 10 | igd | 0.1333 | T | \|\| id:ebi-a-GCST90001483 | ebi-a-GCST90001483 | TRUE | A | 97685914 | 4.52E-54 | reported | 3405 | 0.03591 | rs11819376 |  |  |  |  |  |  |  |  |  |  |  |  |  |  |  |
| 0.6116 | 10 | igd | 0.1333 | T | \|\| id:ebi-a-GCST90001495 | ebi-a-GCST90001495 | TRUE | A | 97685914 | 1.70E-58 | reported | 3405 | 0.0372 | rs11819376 |  |  |  |  |  |  |  |  |  |  |  |  |  |  |  |
| -0.3226 | 22 | igd | 0.1333 | C | \|\| id:ebi-a-GCST90001702 | ebi-a-GCST90001702 | TRUE | T | 42983217 | 1.17E-18 | reported | 3657 | 0.03639 | rs76940643 |  |  |  |  |  |  |  |  |  |  |  |  |  |  |  |
| -0.3146 | 22 | igd | 0.1333 | C | \|\| id:ebi-a-GCST90001703 | ebi-a-GCST90001703 | TRUE | T | 42983217 | 8.03E-18 | reported | 3657 | 0.0364 | rs76940643 |  |  |  |  |  |  |  |  |  |  |  |  |  |  |  |
| -0.2804 | 22 | igd | 0.1333 | C | \|\| id:ebi-a-GCST90001704 | ebi-a-GCST90001704 | TRUE | T | 42983217 | 1.77E-14 | reported | 3657 | 0.03643 | rs76940643 |  |  |  |  |  |  |  |  |  |  |  |  |  |  |  |
| -0.3262 | 22 | igd | 0.1333 | C | \|\| id:ebi-a-GCST90001710 | ebi-a-GCST90001710 | TRUE | T | 42983217 | 3.78E-19 | reported | 3657 | 0.03628 | rs76940643 |  |  |  |  |  |  |  |  |  |  |  |  |  |  |  |
| -0.3264 | 22 | igd | 0.1333 | C | \|\| id:ebi-a-GCST90001711 | ebi-a-GCST90001711 | TRUE | T | 42983217 | 2.96E-19 | reported | 3657 | 0.03619 | rs76940643 |  |  |  |  |  |  |  |  |  |  |  |  |  |  |  |
| 0.2411 | 3 | igd | 0.1331 | A | \|\| id:ebi-a-GCST90001997 | ebi-a-GCST90001997 | TRUE | T | 38951040 | 1.48E-10 | reported | 3590 | 0.03752 | rs4676585 |  |  |  |  |  |  |  |  |  |  |  |  |  |  |  |
| 0.3045 | 6 | igd | 0.133 | G | \|\| id:ebi-a-GCST90002105 | ebi-a-GCST90002105 | TRUE | C | 31009508 | 2.79E-12 | reported | 2872 | 0.04339 | rs2517544 |  |  |  |  |  |  |  |  |  |  |  |  |  |  |  |
| -0.3055 | 14 | igd | 0.1327 | T | \|\| id:ebi-a-GCST90001820 | ebi-a-GCST90001820 | TRUE | C | 106305025 | 8.25E-13 | reported | 3656 | 0.04253 | rs28431803 |  |  |  |  |  |  |  |  |  |  |  |  |  |  |  |
| -0.3389 | 14 | igd | 0.1327 | T | \|\| id:ebi-a-GCST90001821 | ebi-a-GCST90001821 | TRUE | C | 106305025 | 1.80E-15 | reported | 3656 | 0.04242 | rs28431803 |  |  |  |  |  |  |  |  |  |  |  |  |  |  |  |
| -0.2566 | 22 | igd | 0.1326 | A | \|\| id:ebi-a-GCST90001702 | ebi-a-GCST90001702 | TRUE | T | 38548552 | 6.89E-12 | reported | 3657 | 0.03729 | rs132945 |  |  |  |  |  |  |  |  |  |  |  |  |  |  |  |
| -0.2492 | 22 | igd | 0.1326 | A | \|\| id:ebi-a-GCST90001704 | ebi-a-GCST90001704 | TRUE | T | 38548552 | 2.58E-11 | reported | 3657 | 0.03725 | rs132945 |  |  |  |  |  |  |  |  |  |  |  |  |  |  |  |
| -0.23 | 22 | igd | 0.1326 | A | \|\| id:ebi-a-GCST90001710 | ebi-a-GCST90001710 | TRUE | T | 38548552 | 7.25E-10 | reported | 3657 | 0.03723 | rs132945 |  |  |  |  |  |  |  |  |  |  |  |  |  |  |  |
| 0.224 | 10 | igd | 0.1326 | A | \|\| id:ebi-a-GCST90001787 | ebi-a-GCST90001787 | TRUE | G | 6168290 | 5.06E-10 | reported | 3657 | 0.03594 | rs77347828 |  |  |  |  |  |  |  |  |  |  |  |  |  |  |  |
| -0.2417 | 6 | igd | 0.1325 | G | \|\| id:ebi-a-GCST90002119 | ebi-a-GCST90002119 | TRUE | A | 32734304 | 4.80E-08 | reported | 2920 | 0.04416 | rs9276601 |  |  |  |  |  |  |  |  |  |  |  |  |  |  |  |
| 0.4214 | 10 | igd | 0.1322 | T | \|\| id:ebi-a-GCST90001961 | ebi-a-GCST90001961 | TRUE | C | 97370600 | 1.64E-25 | reported | 2920 | 0.03998 | rs483920 |  |  |  |  |  |  |  |  |  |  |  |  |  |  |  |
| -0.6167 | 10 | igd | 0.1322 | T | \|\| id:ebi-a-GCST90002030 | ebi-a-GCST90002030 | TRUE | C | 97370600 | 2.14E-54 | reported | 2920 | 0.03888 | rs483920 |  |  |  |  |  |  |  |  |  |  |  |  |  |  |  |
| -0.4859 | 10 | igd | 0.1322 | T | \|\| id:ebi-a-GCST90002031 | ebi-a-GCST90002031 | TRUE | C | 97370600 | 8.88E-33 | reported | 2920 | 0.04025 | rs483920 |  |  |  |  |  |  |  |  |  |  |  |  |  |  |  |
| -0.491 | 10 | igd | 0.1322 | T | \|\| id:ebi-a-GCST90002032 | ebi-a-GCST90002032 | TRUE | C | 97370600 | 1.90E-33 | reported | 2920 | 0.04023 | rs483920 |  |  |  |  |  |  |  |  |  |  |  |  |  |  |  |
| -0.2245 | 10 | igd | 0.1321 | T | \|\| id:ebi-a-GCST90002029 | ebi-a-GCST90002029 | TRUE | C | 97370600 | 1.83E-08 | reported | 2915 | 0.03978 | rs483920 |  |  |  |  |  |  |  |  |  |  |  |  |  |  |  |
| -0.2524 | 16 | igd | 0.1321 | A | \|\| id:ebi-a-GCST90002088 | ebi-a-GCST90002088 | TRUE | G | 31154345 | 4.52E-10 | reported | 2872 | 0.04034 | rs79912852 |  |  |  |  |  |  |  |  |  |  |  |  |  |  |  |
| -0.3271 | 3 | igd | 0.132 | G | \|\| id:ebi-a-GCST90001996 | ebi-a-GCST90001996 | TRUE | C | 39293757 | 5.15E-19 | reported | 3579 | 0.03651 | rs9823718 |  |  |  |  |  |  |  |  |  |  |  |  |  |  |  |
| -0.2149 | 3 | igd | 0.1319 | G | \|\| id:ebi-a-GCST90001994 | ebi-a-GCST90001994 | TRUE | C | 39293757 | 3.24E-09 | reported | 3590 | 0.03622 | rs9823718 |  |  |  |  |  |  |  |  |  |  |  |  |  |  |  |
| -0.4122 | 3 | igd | 0.1319 | G | \|\| id:ebi-a-GCST90001997 | ebi-a-GCST90001997 | TRUE | C | 39293757 | 5.78E-29 | reported | 3590 | 0.03658 | rs9823718 |  |  |  |  |  |  |  |  |  |  |  |  |  |  |  |
| -0.4083 | 3 | igd | 0.1319 | G | \|\| id:ebi-a-GCST90001995 | ebi-a-GCST90001995 | TRUE | C | 39293757 | 7.85E-29 | reported | 3590 | 0.03633 | rs9823718 |  |  |  |  |  |  |  |  |  |  |  |  |  |  |  |
| 0.3935 | 10 | igd | 0.1317 | G | \|\| id:ebi-a-GCST90001961 | ebi-a-GCST90001961 | TRUE | A | 97679939 | 1.53E-21 | reported | 2920 | 0.04095 | rs36103691 |  |  |  |  |  |  |  |  |  |  |  |  |  |  |  |
| -0.4142 | 10 | igd | 0.1317 | T | \|\| id:ebi-a-GCST90001961 | ebi-a-GCST90001961 | TRUE | A | 97685914 | 6.60E-25 | reported | 2920 | 0.03982 | rs11819376 |  |  |  |  |  |  |  |  |  |  |  |  |  |  |  |
| -0.6005 | 10 | igd | 0.1317 | G | \|\| id:ebi-a-GCST90002030 | ebi-a-GCST90002030 | TRUE | A | 97679939 | 9.85E-50 | reported | 2920 | 0.03974 | rs36103691 |  |  |  |  |  |  |  |  |  |  |  |  |  |  |  |
| 0.7399 | 10 | igd | 0.1317 | T | \|\| id:ebi-a-GCST90002030 | ebi-a-GCST90002030 | TRUE | A | 97685914 | 8.82E-80 | reported | 2920 | 0.03792 | rs11819376 |  |  |  |  |  |  |  |  |  |  |  |  |  |  |  |
| 0.6068 | 10 | igd | 0.1317 | T | \|\| id:ebi-a-GCST90002031 | ebi-a-GCST90002031 | TRUE | A | 97685914 | 2.62E-51 | reported | 2920 | 0.03949 | rs11819376 |  |  |  |  |  |  |  |  |  |  |  |  |  |  |  |
| -0.5254 | 10 | igd | 0.1317 | A | \|\| id:ebi-a-GCST90002031 | ebi-a-GCST90002031 | TRUE | G | 97684234 | 1.08E-36 | reported | 2920 | 0.04095 | rs34963139 |  |  |  |  |  |  |  |  |  |  |  |  |  |  |  |
| -0.5086 | 10 | igd | 0.1317 | A | \|\| id:ebi-a-GCST90002032 | ebi-a-GCST90002032 | TRUE | G | 97684234 | 1.89E-34 | reported | 2920 | 0.04102 | rs34963139 |  |  |  |  |  |  |  |  |  |  |  |  |  |  |  |
| 0.5669 | 10 | igd | 0.1317 | T | \|\| id:ebi-a-GCST90002032 | ebi-a-GCST90002032 | TRUE | A | 97685914 | 8.48E-45 | reported | 2920 | 0.03969 | rs11819376 |  |  |  |  |  |  |  |  |  |  |  |  |  |  |  |
| 0.55 | 6 | igd | 0.1315 | G | \|\| id:ebi-a-GCST90002109 | ebi-a-GCST90002109 | TRUE | A | 32479493 | 4.42E-11 | reported | 1578 | 0.08287 | rs79905009 |  |  |  |  |  |  |  |  |  |  |  |  |  |  |  |
| 0.8073 | 6 | igd | 0.1314 | G | \|\| id:ebi-a-GCST90002108 | ebi-a-GCST90002108 | TRUE | A | 32479493 | 3.73E-22 | reported | 1579 | 0.08216 | rs79905009 |  |  |  |  |  |  |  |  |  |  |  |  |  |  |  |
| -0.3236 | 6 | igd | 0.1313 | T | \|\| id:ebi-a-GCST90002119 | ebi-a-GCST90002119 | TRUE | C | 31451848 | 1.61E-12 | reported | 2920 | 0.04561 | rs3094010 |  |  |  |  |  |  |  |  |  |  |  |  |  |  |  |
| 0.3219 | 10 | igd | 0.1312 | T | \|\| id:ebi-a-GCST90002030 | ebi-a-GCST90002030 | TRUE | A | 95969913 | 1.34E-15 | reported | 2920 | 0.04006 | rs10509669 |  |  |  |  |  |  |  |  |  |  |  |  |  |  |  |
| 0.2808 | 10 | igd | 0.1312 | T | \|\| id:ebi-a-GCST90002031 | ebi-a-GCST90002031 | TRUE | A | 95969913 | 1.06E-11 | reported | 2920 | 0.04115 | rs10509669 |  |  |  |  |  |  |  |  |  |  |  |  |  |  |  |
| 0.3108 | 10 | igd | 0.1312 | T | \|\| id:ebi-a-GCST90002032 | ebi-a-GCST90002032 | TRUE | A | 95969913 | 4.92E-14 | reported | 2920 | 0.04104 | rs10509669 |  |  |  |  |  |  |  |  |  |  |  |  |  |  |  |
| 0.3481 | 6 | igd | 0.1311 | C | \|\| id:ebi-a-GCST90002108 | ebi-a-GCST90002108 | TRUE | T | 33045500 | 1.42E-09 | reported | 1579 | 0.05716 | rs2856816 |  |  |  |  |  |  |  |  |  |  |  |  |  |  |  |
| -0.2559 | 10 | igd | 0.131 | A | \|\| id:ebi-a-GCST90001961 | ebi-a-GCST90001961 | TRUE | G | 98188719 | 5.85E-10 | reported | 2920 | 0.04116 | rs12765987 |  |  |  |  |  |  |  |  |  |  |  |  |  |  |  |
| -0.2845 | 10 | igd | 0.1308 | A | \|\| id:ebi-a-GCST90001670 | ebi-a-GCST90001670 | TRUE | G | 96138064 | 2.71E-13 | reported | 3440 | 0.03878 | rs77346846 |  |  |  |  |  |  |  |  |  |  |  |  |  |  |  |
| -0.2737 | 10 | igd | 0.1308 | A | \|\| id:ebi-a-GCST90001671 | ebi-a-GCST90001671 | TRUE | G | 96138064 | 2.37E-12 | reported | 3440 | 0.0389 | rs77346846 |  |  |  |  |  |  |  |  |  |  |  |  |  |  |  |
| 0.2452 | 1 | igd | 0.1307 | T | \|\| id:ebi-a-GCST90001864 | ebi-a-GCST90001864 | TRUE | A | 166820284 | 1.91E-09 | reported | 2919 | 0.0407 | rs3820387 |  |  |  |  |  |  |  |  |  |  |  |  |  |  |  |
| 0.3804 | 19 | igd | 0.1306 | T | \|\| id:ebi-a-GCST90001955 | ebi-a-GCST90001955 | TRUE | G | 51592949 | 4.42E-12 | reported | 1554 | 0.05451 | rs117439272 |  |  |  |  |  |  |  |  |  |  |  |  |  |  |  |
| -0.56 | 10 | igd | 0.1305 | G | \|\| id:ebi-a-GCST90001658 | ebi-a-GCST90001658 | TRUE | A | 97679939 | 8.13E-53 | reported | 3440 | 0.03598 | rs36103691 |  |  |  |  |  |  |  |  |  |  |  |  |  |  |  |
| -0.5481 | 10 | igd | 0.1305 | G | \|\| id:ebi-a-GCST90001659 | ebi-a-GCST90001659 | TRUE | A | 97679939 | 1.35E-50 | reported | 3440 | 0.03604 | rs36103691 |  |  |  |  |  |  |  |  |  |  |  |  |  |  |  |
| -0.5652 | 10 | igd | 0.1305 | G | \|\| id:ebi-a-GCST90001670 | ebi-a-GCST90001670 | TRUE | A | 97679939 | 9.10E-50 | reported | 3440 | 0.03749 | rs36103691 |  |  |  |  |  |  |  |  |  |  |  |  |  |  |  |
| -0.5596 | 10 | igd | 0.1305 | G | \|\| id:ebi-a-GCST90001671 | ebi-a-GCST90001671 | TRUE | A | 97679939 | 1.48E-48 | reported | 3440 | 0.03761 | rs36103691 |  |  |  |  |  |  |  |  |  |  |  |  |  |  |  |
| 0.41 | 19 | igd | 0.1305 | T | \|\| id:ebi-a-GCST90001954 | ebi-a-GCST90001954 | TRUE | G | 51592949 | 7.70E-14 | reported | 1555 | 0.05435 | rs117439272 |  |  |  |  |  |  |  |  |  |  |  |  |  |  |  |
| 0.7309 | 6 | igd | 0.1305 | T | \|\| id:ebi-a-GCST90002108 | ebi-a-GCST90002108 | TRUE | C | 32516329 | 2.25E-15 | reported | 1579 | 0.09128 | rs71534588 |  |  |  |  |  |  |  |  |  |  |  |  |  |  |  |
| -0.5723 | 10 | igd | 0.1304 | G | \|\| id:ebi-a-GCST90001660 | ebi-a-GCST90001660 | TRUE | A | 97679939 | 2.66E-50 | reported | 3408 | 0.03774 | rs36103691 |  |  |  |  |  |  |  |  |  |  |  |  |  |  |  |
| -0.5688 | 10 | igd | 0.1304 | G | \|\| id:ebi-a-GCST90001672 | ebi-a-GCST90001672 | TRUE | A | 97679939 | 9.57E-49 | reported | 3408 | 0.03815 | rs36103691 |  |  |  |  |  |  |  |  |  |  |  |  |  |  |  |
| 0.4056 | 19 | igd | 0.1303 | T | \|\| id:ebi-a-GCST90001953 | ebi-a-GCST90001953 | TRUE | G | 51592949 | 1.40E-13 | reported | 1558 | 0.05434 | rs117439272 |  |  |  |  |  |  |  |  |  |  |  |  |  |  |  |
| -0.4171 | 10 | igd | 0.1302 | C | \|\| id:ebi-a-GCST90001484 | ebi-a-GCST90001484 | TRUE | T | 97681714 | 5.63E-29 | reported | 3437 | 0.037 | rs11594853 |  |  |  |  |  |  |  |  |  |  |  |  |  |  |  |
| -0.4754 | 10 | igd | 0.1302 | G | \|\| id:ebi-a-GCST90001491 | ebi-a-GCST90001491 | TRUE | A | 97679939 | 9.51E-36 | reported | 3437 | 0.03765 | rs36103691 |  |  |  |  |  |  |  |  |  |  |  |  |  |  |  |
| -0.5455 | 10 | igd | 0.1302 | G | \|\| id:ebi-a-GCST90001496 | ebi-a-GCST90001496 | TRUE | A | 97679939 | 1.25E-45 | reported | 3437 | 0.03791 | rs36103691 |  |  |  |  |  |  |  |  |  |  |  |  |  |  |  |
| -0.5457 | 10 | igd | 0.1302 | G | \|\| id:ebi-a-GCST90001497 | ebi-a-GCST90001497 | TRUE | A | 97679939 | 7.92E-47 | reported | 3437 | 0.0374 | rs36103691 |  |  |  |  |  |  |  |  |  |  |  |  |  |  |  |
| -0.553 | 10 | igd | 0.1301 | G | \|\| id:ebi-a-GCST90001495 | ebi-a-GCST90001495 | TRUE | A | 97679939 | 7.79E-46 | reported | 3405 | 0.03833 | rs36103691 |  |  |  |  |  |  |  |  |  |  |  |  |  |  |  |
| 0.2596 | 6 | igd | 0.1299 | A | \|\| id:ebi-a-GCST90002105 | ebi-a-GCST90002105 | TRUE | G | 28941688 | 9.47E-09 | reported | 2872 | 0.04509 | rs6933672 |  |  |  |  |  |  |  |  |  |  |  |  |  |  |  |
| 0.2287 | 22 | igd | 0.1295 | G | \|\| id:ebi-a-GCST90001702 | ebi-a-GCST90001702 | TRUE | T | 40676672 | 6.04E-10 | reported | 3657 | 0.03685 | rs28360630 |  |  |  |  |  |  |  |  |  |  |  |  |  |  |  |
| 0.2209 | 22 | igd | 0.1295 | G | \|\| id:ebi-a-GCST90001703 | ebi-a-GCST90001703 | TRUE | T | 40676672 | 2.24E-09 | reported | 3657 | 0.03686 | rs28360630 |  |  |  |  |  |  |  |  |  |  |  |  |  |  |  |
| 0.2173 | 22 | igd | 0.1295 | G | \|\| id:ebi-a-GCST90001704 | ebi-a-GCST90001704 | TRUE | T | 40676672 | 3.93E-09 | reported | 3657 | 0.03682 | rs28360630 |  |  |  |  |  |  |  |  |  |  |  |  |  |  |  |
| 0.2127 | 22 | igd | 0.1295 | G | \|\| id:ebi-a-GCST90001710 | ebi-a-GCST90001710 | TRUE | T | 40676672 | 7.86E-09 | reported | 3657 | 0.03677 | rs28360630 |  |  |  |  |  |  |  |  |  |  |  |  |  |  |  |
| 0.2119 | 22 | igd | 0.1295 | G | \|\| id:ebi-a-GCST90001711 | ebi-a-GCST90001711 | TRUE | T | 40676672 | 8.24E-09 | reported | 3657 | 0.03669 | rs28360630 |  |  |  |  |  |  |  |  |  |  |  |  |  |  |  |
| 0.2486 | 10 | igd | 0.1293 | T | \|\| id:ebi-a-GCST90001892 | ebi-a-GCST90001892 | TRUE | C | 98176319 | 1.46E-09 | reported | 2920 | 0.04097 | rs12762576 |  |  |  |  |  |  |  |  |  |  |  |  |  |  |  |
| 0.3651 | 19 | igd | 0.1291 | T | \|\| id:ebi-a-GCST90001952 | ebi-a-GCST90001952 | TRUE | G | 51592949 | 3.86E-11 | reported | 1569 | 0.05485 | rs117439272 |  |  |  |  |  |  |  |  |  |  |  |  |  |  |  |
| 0.3435 | 19 | igd | 0.129 | T | \|\| id:ebi-a-GCST90001947 | ebi-a-GCST90001947 | TRUE | G | 51592949 | 5.10E-10 | reported | 1577 | 0.05491 | rs117439272 |  |  |  |  |  |  |  |  |  |  |  |  |  |  |  |
| 0.3924 | 19 | igd | 0.129 | T | \|\| id:ebi-a-GCST90001957 | ebi-a-GCST90001957 | TRUE | G | 51592949 | 1.03E-12 | reported | 1578 | 0.05461 | rs117439272 |  |  |  |  |  |  |  |  |  |  |  |  |  |  |  |
| 0.3786 | 19 | igd | 0.1289 | T | \|\| id:ebi-a-GCST90001956 | ebi-a-GCST90001956 | TRUE | G | 51592949 | 6.11E-12 | reported | 1579 | 0.05463 | rs117439272 |  |  |  |  |  |  |  |  |  |  |  |  |  |  |  |
| 0.2469 | 10 | igd | 0.1288 | T | \|\| id:ebi-a-GCST90001670 | ebi-a-GCST90001670 | TRUE | C | 98176319 | 1.21E-10 | reported | 3440 | 0.03823 | rs12762576 |  |  |  |  |  |  |  |  |  |  |  |  |  |  |  |
| 0.2398 | 10 | igd | 0.1288 | T | \|\| id:ebi-a-GCST90001671 | ebi-a-GCST90001671 | TRUE | C | 98176319 | 4.36E-10 | reported | 3440 | 0.03831 | rs12762576 |  |  |  |  |  |  |  |  |  |  |  |  |  |  |  |
| 0.26 | 22 | igd | 0.1287 | C | \|\| id:ebi-a-GCST90001702 | ebi-a-GCST90001702 | TRUE | T | 42622369 | 2.53E-12 | reported | 3657 | 0.03701 | rs9623543 |  |  |  |  |  |  |  |  |  |  |  |  |  |  |  |
| 0.2593 | 22 | igd | 0.1287 | C | \|\| id:ebi-a-GCST90001703 | ebi-a-GCST90001703 | TRUE | T | 42622369 | 2.90E-12 | reported | 3657 | 0.03701 | rs9623543 |  |  |  |  |  |  |  |  |  |  |  |  |  |  |  |
| 0.2322 | 22 | igd | 0.1287 | C | \|\| id:ebi-a-GCST90001704 | ebi-a-GCST90001704 | TRUE | T | 42622369 | 3.93E-10 | reported | 3657 | 0.03701 | rs9623543 |  |  |  |  |  |  |  |  |  |  |  |  |  |  |  |
| 0.2541 | 22 | igd | 0.1287 | C | \|\| id:ebi-a-GCST90001710 | ebi-a-GCST90001710 | TRUE | T | 42622369 | 6.89E-12 | reported | 3657 | 0.03691 | rs9623543 |  |  |  |  |  |  |  |  |  |  |  |  |  |  |  |
| 0.2683 | 22 | igd | 0.1287 | C | \|\| id:ebi-a-GCST90001711 | ebi-a-GCST90001711 | TRUE | T | 42622369 | 3.82E-13 | reported | 3657 | 0.03681 | rs9623543 |  |  |  |  |  |  |  |  |  |  |  |  |  |  |  |
| -0.2363 | 16 | igd | 0.1287 | G | \|\| id:ebi-a-GCST90001785 | ebi-a-GCST90001785 | TRUE | A | 81916912 | 1.47E-10 | reported | 3649 | 0.03676 | rs11548656 |  |  |  |  |  |  |  |  |  |  |  |  |  |  |  |
| -0.5931 | 6 | igd | 0.1286 | G | \|\| id:ebi-a-GCST90002108 | ebi-a-GCST90002108 | TRUE | A | 32575735 | 2.56E-12 | reported | 1579 | 0.08406 | rs9461776 |  |  |  |  |  |  |  |  |  |  |  |  |  |  |  |
| 0.2188 | 2 | igd | 0.128 | C | \|\| id:ebi-a-GCST90002098 | ebi-a-GCST90002098 | TRUE | T | 38634844 | 2.62E-08 | reported | 2910 | 0.0392 | rs13392984 |  |  |  |  |  |  |  |  |  |  |  |  |  |  |  |
| 0.3626 | 6 | igd | 0.1279 | G | \|\| id:ebi-a-GCST90002108 | ebi-a-GCST90002108 | TRUE | C | 31009508 | 1.53E-10 | reported | 1579 | 0.05626 | rs2517544 |  |  |  |  |  |  |  |  |  |  |  |  |  |  |  |
| 0.3013 | 19 | igd | 0.1277 | G | \|\| id:ebi-a-GCST90001957 | ebi-a-GCST90001957 | TRUE | A | 51451043 | 2.75E-08 | reported | 1578 | 0.05395 | rs2411333 |  |  |  |  |  |  |  |  |  |  |  |  |  |  |  |
| 0.2584 | 10 | igd | 0.1276 | T | \|\| id:ebi-a-GCST90001672 | ebi-a-GCST90001672 | TRUE | C | 98176319 | 3.97E-11 | reported | 3408 | 0.03899 | rs12762576 |  |  |  |  |  |  |  |  |  |  |  |  |  |  |  |
| 0.3044 | 19 | igd | 0.1276 | G | \|\| id:ebi-a-GCST90001956 | ebi-a-GCST90001956 | TRUE | A | 51451043 | 1.88E-08 | reported | 1579 | 0.05385 | rs2411333 |  |  |  |  |  |  |  |  |  |  |  |  |  |  |  |
| 0.3056 | 19 | igd | 0.1275 | G | \|\| id:ebi-a-GCST90001947 | ebi-a-GCST90001947 | TRUE | A | 51451043 | 1.79E-08 | reported | 1577 | 0.05399 | rs2411333 |  |  |  |  |  |  |  |  |  |  |  |  |  |  |  |
| -0.2769 | 6 | igd | 0.1271 | C | \|\| id:ebi-a-GCST90002118 | ebi-a-GCST90002118 | TRUE | T | 30756887 | 4.76E-08 | reported | 2920 | 0.05059 | rs568802587 |  |  |  |  |  |  |  |  |  |  |  |  |  |  |  |
| 0.2371 | 6 | igd | 0.127 | T | \|\| id:ebi-a-GCST90001758 | ebi-a-GCST90001758 | TRUE | G | 31052632 | 2.81E-08 | reported | 3657 | 0.0426 | rs28732092 |  |  |  |  |  |  |  |  |  |  |  |  |  |  |  |
| 0.2482 | 22 | igd | 0.1269 | T | \|\| id:ebi-a-GCST90001702 | ebi-a-GCST90001702 | TRUE | G | 39768080 | 9.00E-11 | reported | 3657 | 0.03818 | rs11704158 |  |  |  |  |  |  |  |  |  |  |  |  |  |  |  |
| 0.2435 | 22 | igd | 0.1269 | T | \|\| id:ebi-a-GCST90001703 | ebi-a-GCST90001703 | TRUE | G | 39768080 | 2.02E-10 | reported | 3657 | 0.03818 | rs11704158 |  |  |  |  |  |  |  |  |  |  |  |  |  |  |  |
| 0.2474 | 22 | igd | 0.1269 | T | \|\| id:ebi-a-GCST90001704 | ebi-a-GCST90001704 | TRUE | G | 39768080 | 9.82E-11 | reported | 3657 | 0.03812 | rs11704158 |  |  |  |  |  |  |  |  |  |  |  |  |  |  |  |
| 0.2438 | 22 | igd | 0.1269 | T | \|\| id:ebi-a-GCST90001710 | ebi-a-GCST90001710 | TRUE | G | 39768080 | 1.72E-10 | reported | 3657 | 0.03808 | rs11704158 |  |  |  |  |  |  |  |  |  |  |  |  |  |  |  |
| 0.2557 | 22 | igd | 0.1269 | T | \|\| id:ebi-a-GCST90001711 | ebi-a-GCST90001711 | TRUE | G | 39768080 | 1.90E-11 | reported | 3657 | 0.03797 | rs11704158 |  |  |  |  |  |  |  |  |  |  |  |  |  |  |  |
| -0.3411 | 6 | igd | 0.1269 | A | \|\| id:ebi-a-GCST90002119 | ebi-a-GCST90002119 | TRUE | G | 31168029 | 1.49E-13 | reported | 2920 | 0.04595 | rs6903896 |  |  |  |  |  |  |  |  |  |  |  |  |  |  |  |
| 0.3381 | 6 | igd | 0.1267 | A | \|\| id:ebi-a-GCST90002108 | ebi-a-GCST90002108 | TRUE | G | 30978746 | 1.85E-09 | reported | 1579 | 0.05592 | rs12203410 |  |  |  |  |  |  |  |  |  |  |  |  |  |  |  |
| 0.3368 | 10 | igd | 0.1266 | T | \|\| id:ebi-a-GCST90001660 | ebi-a-GCST90001660 | TRUE | G | 98269497 | 2.63E-19 | reported | 3408 | 0.03727 | rs57194010 |  |  |  |  |  |  |  |  |  |  |  |  |  |  |  |
| 0.3211 | 10 | igd | 0.1266 | T | \|\| id:ebi-a-GCST90001672 | ebi-a-GCST90001672 | TRUE | G | 98269497 | 2.34E-17 | reported | 3408 | 0.03769 | rs57194010 |  |  |  |  |  |  |  |  |  |  |  |  |  |  |  |
| 0.2115 | 20 | igd | 0.1266 | T | \|\| id:ebi-a-GCST90001989 | ebi-a-GCST90001989 | TRUE | C | 44082071 | 2.17E-08 | reported | 3621 | 0.0377 | rs6094142 |  |  |  |  |  |  |  |  |  |  |  |  |  |  |  |
| -0.2839 | 6 | igd | 0.1266 | T | \|\| id:ebi-a-GCST90002121 | ebi-a-GCST90002121 | TRUE | C | 31138472 | 7.21E-11 | reported | 2915 | 0.04341 | rs17197199 |  |  |  |  |  |  |  |  |  |  |  |  |  |  |  |
| 0.2263 | 20 | igd | 0.1265 | A | \|\| id:ebi-a-GCST90001798 | ebi-a-GCST90001798 | TRUE | C | 44981692 | 2.14E-09 | reported | 3657 | 0.0377 | rs45456397 |  |  |  |  |  |  |  |  |  |  |  |  |  |  |  |
| 0.2366 | 20 | igd | 0.1265 | A | \|\| id:ebi-a-GCST90001804 | ebi-a-GCST90001804 | TRUE | C | 44981692 | 3.24E-10 | reported | 3657 | 0.03753 | rs45456397 |  |  |  |  |  |  |  |  |  |  |  |  |  |  |  |
| 0.2214 | 20 | igd | 0.1265 | A | \|\| id:ebi-a-GCST90001808 | ebi-a-GCST90001808 | TRUE | C | 44981692 | 6.28E-09 | reported | 3657 | 0.03802 | rs45456397 |  |  |  |  |  |  |  |  |  |  |  |  |  |  |  |
| -0.2895 | 6 | igd | 0.1265 | C | \|\| id:ebi-a-GCST90002118 | ebi-a-GCST90002118 | TRUE | T | 31178203 | 5.54E-10 | reported | 2920 | 0.04651 | rs61283711 |  |  |  |  |  |  |  |  |  |  |  |  |  |  |  |
| 0.2488 | 10 | igd | 0.1264 | T | \|\| id:ebi-a-GCST90001489 | ebi-a-GCST90001489 | TRUE | G | 98269497 | 4.41E-11 | reported | 3405 | 0.03764 | rs57194010 |  |  |  |  |  |  |  |  |  |  |  |  |  |  |  |
| 0.3077 | 10 | igd | 0.1264 | T | \|\| id:ebi-a-GCST90001495 | ebi-a-GCST90001495 | TRUE | G | 98269497 | 5.72E-16 | reported | 3405 | 0.03782 | rs57194010 |  |  |  |  |  |  |  |  |  |  |  |  |  |  |  |
| 0.2905 | 10 | igd | 0.1263 | T | \|\| id:ebi-a-GCST90001658 | ebi-a-GCST90001658 | TRUE | G | 98269497 | 5.51E-16 | reported | 3440 | 0.03569 | rs57194010 |  |  |  |  |  |  |  |  |  |  |  |  |  |  |  |
| 0.2945 | 10 | igd | 0.1263 | T | \|\| id:ebi-a-GCST90001659 | ebi-a-GCST90001659 | TRUE | G | 98269497 | 2.21E-16 | reported | 3440 | 0.03569 | rs57194010 |  |  |  |  |  |  |  |  |  |  |  |  |  |  |  |
| 0.2985 | 10 | igd | 0.1263 | T | \|\| id:ebi-a-GCST90001670 | ebi-a-GCST90001670 | TRUE | G | 98269497 | 1.29E-15 | reported | 3440 | 0.03716 | rs57194010 |  |  |  |  |  |  |  |  |  |  |  |  |  |  |  |
| 0.3018 | 10 | igd | 0.1263 | T | \|\| id:ebi-a-GCST90001671 | ebi-a-GCST90001671 | TRUE | G | 98269497 | 6.80E-16 | reported | 3440 | 0.0372 | rs57194010 |  |  |  |  |  |  |  |  |  |  |  |  |  |  |  |
| -0.4065 | 10 | igd | 0.1262 | G | \|\| id:ebi-a-GCST90002030 | ebi-a-GCST90002030 | TRUE | A | 98146121 | 8.10E-23 | reported | 2920 | 0.04099 | rs72829522 |  |  |  |  |  |  |  |  |  |  |  |  |  |  |  |
| -0.3225 | 10 | igd | 0.1262 | G | \|\| id:ebi-a-GCST90002032 | ebi-a-GCST90002032 | TRUE | A | 98146121 | 2.51E-14 | reported | 2920 | 0.0421 | rs72829522 |  |  |  |  |  |  |  |  |  |  |  |  |  |  |  |
| 0.291 | 10 | igd | 0.1261 | T | \|\| id:ebi-a-GCST90001496 | ebi-a-GCST90001496 | TRUE | G | 98269497 | 1.11E-14 | reported | 3437 | 0.03749 | rs57194010 |  |  |  |  |  |  |  |  |  |  |  |  |  |  |  |
| 0.301 | 10 | igd | 0.1261 | T | \|\| id:ebi-a-GCST90001497 | ebi-a-GCST90001497 | TRUE | G | 98269497 | 5.56E-16 | reported | 3437 | 0.03699 | rs57194010 |  |  |  |  |  |  |  |  |  |  |  |  |  |  |  |
| 0.2157 | 10 | igd | 0.1258 | A | \|\| id:ebi-a-GCST90001483 | ebi-a-GCST90001483 | TRUE | G | 98270396 | 4.85E-09 | reported | 3405 | 0.03676 | rs7922821 |  |  |  |  |  |  |  |  |  |  |  |  |  |  |  |
| -0.2585 | 12 | igd | 0.1258 | A | \|\| id:ebi-a-GCST90001798 | ebi-a-GCST90001798 | TRUE | G | 6558575 | 1.43E-12 | reported | 3657 | 0.03638 | rs61916429 |  |  |  |  |  |  |  |  |  |  |  |  |  |  |  |
| -0.2224 | 12 | igd | 0.1258 | A | \|\| id:ebi-a-GCST90001804 | ebi-a-GCST90001804 | TRUE | G | 6558575 | 9.47E-10 | reported | 3657 | 0.03626 | rs61916429 |  |  |  |  |  |  |  |  |  |  |  |  |  |  |  |
| -0.2881 | 12 | igd | 0.1258 | A | \|\| id:ebi-a-GCST90001808 | ebi-a-GCST90001808 | TRUE | G | 6558575 | 4.76E-15 | reported | 3657 | 0.03662 | rs61916429 |  |  |  |  |  |  |  |  |  |  |  |  |  |  |  |
| -0.2138 | 12 | igd | 0.1257 | A | \|\| id:ebi-a-GCST90001806 | ebi-a-GCST90001806 | TRUE | G | 6558575 | 7.50E-09 | reported | 3656 | 0.03691 | rs61916429 |  |  |  |  |  |  |  |  |  |  |  |  |  |  |  |
| -0.1956 | 2 | igd | 0.1256 | C | \|\| id:ebi-a-GCST90001664 | ebi-a-GCST90001664 | TRUE | T | 38691445 | 3.23E-08 | reported | 3440 | 0.03531 | rs35518093 |  |  |  |  |  |  |  |  |  |  |  |  |  |  |  |
| -0.225 | 12 | igd | 0.1256 | T | \|\| id:ebi-a-GCST90001802 | ebi-a-GCST90001802 | TRUE | A | 6558720 | 1.19E-09 | reported | 3657 | 0.03691 | rs11064198 |  |  |  |  |  |  |  |  |  |  |  |  |  |  |  |
| -0.2231 | 12 | igd | 0.1255 | A | \|\| id:ebi-a-GCST90002074 | ebi-a-GCST90002074 | TRUE | G | 69323569 | 2.33E-08 | reported | 3112 | 0.03983 | rs6581839 |  |  |  |  |  |  |  |  |  |  |  |  |  |  |  |
| -0.2869 | 12 | igd | 0.1253 | G | \|\| id:ebi-a-GCST90002073 | ebi-a-GCST90002073 | TRUE | C | 69323066 | 4.10E-12 | reported | 2850 | 0.0412 | rs2047481 |  |  |  |  |  |  |  |  |  |  |  |  |  |  |  |
| -0.236 | 19 | igd | 0.12506 | G | \|\| id:prot-a-439 | prot-a-439 | TRUE | C | 51640330 | 1.41E-08 | reported | 3301 | 0.0416 | rs34681424 |  |  |  |  |  |  |  |  |  |  |  |  |  |  |  |
| -0.3081 | 22 | igd | 0.125 | A | \|\| id:ebi-a-GCST90001702 | ebi-a-GCST90001702 | TRUE | G | 43569380 | 9.07E-16 | reported | 3657 | 0.03816 | rs118162361 |  |  |  |  |  |  |  |  |  |  |  |  |  |  |  |
| -0.3057 | 22 | igd | 0.125 | A | \|\| id:ebi-a-GCST90001703 | ebi-a-GCST90001703 | TRUE | G | 43569380 | 1.52E-15 | reported | 3657 | 0.03816 | rs118162361 |  |  |  |  |  |  |  |  |  |  |  |  |  |  |  |
| -0.2921 | 22 | igd | 0.125 | A | \|\| id:ebi-a-GCST90001704 | ebi-a-GCST90001704 | TRUE | G | 43569380 | 2.34E-14 | reported | 3657 | 0.03813 | rs118162361 |  |  |  |  |  |  |  |  |  |  |  |  |  |  |  |
| -0.315 | 22 | igd | 0.125 | A | \|\| id:ebi-a-GCST90001710 | ebi-a-GCST90001710 | TRUE | G | 43569380 | 1.70E-16 | reported | 3657 | 0.03804 | rs118162361 |  |  |  |  |  |  |  |  |  |  |  |  |  |  |  |
| -0.3155 | 22 | igd | 0.125 | A | \|\| id:ebi-a-GCST90001711 | ebi-a-GCST90001711 | TRUE | G | 43569380 | 1.27E-16 | reported | 3657 | 0.03794 | rs118162361 |  |  |  |  |  |  |  |  |  |  |  |  |  |  |  |
| -0.2558 | 6 | igd | 0.1249 | A | \|\| id:ebi-a-GCST90001692 | ebi-a-GCST90001692 | TRUE | G | 31327660 | 7.24E-09 | reported | 3440 | 0.04411 | rs1811197 |  |  |  |  |  |  |  |  |  |  |  |  |  |  |  |
| 0.4377 | 19 | igd | 0.1249 | T | \|\| id:ebi-a-GCST90001948 | ebi-a-GCST90001948 | TRUE | G | 51592949 | 3.93E-16 | reported | 1633 | 0.05321 | rs117439272 |  |  |  |  |  |  |  |  |  |  |  |  |  |  |  |
| 0.4077 | 19 | igd | 0.1248 | T | \|\| id:ebi-a-GCST90001946 | ebi-a-GCST90001946 | TRUE | G | 51592949 | 5.24E-14 | reported | 1634 | 0.05369 | rs117439272 |  |  |  |  |  |  |  |  |  |  |  |  |  |  |  |
| 0.4312 | 19 | igd | 0.1248 | T | \|\| id:ebi-a-GCST90001949 | ebi-a-GCST90001949 | TRUE | G | 51592949 | 9.97E-16 | reported | 1634 | 0.05317 | rs117439272 |  |  |  |  |  |  |  |  |  |  |  |  |  |  |  |
| -0.2661 | 10 | igd | 0.1248 | T | \|\| id:ebi-a-GCST90001961 | ebi-a-GCST90001961 | TRUE | G | 98269497 | 5.88E-11 | reported | 2920 | 0.0405 | rs57194010 |  |  |  |  |  |  |  |  |  |  |  |  |  |  |  |
| 0.3008 | 10 | igd | 0.1248 | T | \|\| id:ebi-a-GCST90002030 | ebi-a-GCST90002030 | TRUE | G | 98269497 | 5.42E-14 | reported | 2920 | 0.0398 | rs57194010 |  |  |  |  |  |  |  |  |  |  |  |  |  |  |  |
| 0.3485 | 6 | igd | 0.1247 | T | \|\| id:ebi-a-GCST90002105 | ebi-a-GCST90002105 | TRUE | C | 29530868 | 3.09E-14 | reported | 2872 | 0.04566 | rs362529 |  |  |  |  |  |  |  |  |  |  |  |  |  |  |  |
| 0.2876 | 3 | igd | 0.1246 | A | \|\| id:ebi-a-GCST90002014 | ebi-a-GCST90002014 | TRUE | G | 46468470 | 3.64E-11 | reported | 2870 | 0.04328 | rs2097278 |  |  |  |  |  |  |  |  |  |  |  |  |  |  |  |
| 0.201 | 4 | igd | 0.1244 | T | \|\| id:ebi-a-GCST90001815 | ebi-a-GCST90001815 | TRUE | C | 15910047 | 4.10E-08 | reported | 3657 | 0.03656 | rs56219219 |  |  |  |  |  |  |  |  |  |  |  |  |  |  |  |
| 0.2181 | 22 | igd | 0.1243 | A | \|\| id:ebi-a-GCST90001702 | ebi-a-GCST90001702 | TRUE | G | 40537011 | 9.33E-09 | reported | 3657 | 0.03789 | rs34301841 |  |  |  |  |  |  |  |  |  |  |  |  |  |  |  |
| 0.2111 | 22 | igd | 0.1243 | A | \|\| id:ebi-a-GCST90001703 | ebi-a-GCST90001703 | TRUE | G | 40537011 | 2.74E-08 | reported | 3657 | 0.0379 | rs34301841 |  |  |  |  |  |  |  |  |  |  |  |  |  |  |  |
| 0.2097 | 22 | igd | 0.1243 | A | \|\| id:ebi-a-GCST90001704 | ebi-a-GCST90001704 | TRUE | G | 40537011 | 3.23E-08 | reported | 3657 | 0.03785 | rs34301841 |  |  |  |  |  |  |  |  |  |  |  |  |  |  |  |
| 0.2932 | 19 | igd | 0.1243 | G | \|\| id:ebi-a-GCST90001948 | ebi-a-GCST90001948 | TRUE | A | 51451043 | 3.18E-08 | reported | 1633 | 0.05276 | rs2411333 |  |  |  |  |  |  |  |  |  |  |  |  |  |  |  |
| 0.3101 | 19 | igd | 0.1242 | G | \|\| id:ebi-a-GCST90001946 | ebi-a-GCST90001946 | TRUE | A | 51451043 | 6.00E-09 | reported | 1634 | 0.05303 | rs2411333 |  |  |  |  |  |  |  |  |  |  |  |  |  |  |  |
| 0.3097 | 19 | igd | 0.1242 | G | \|\| id:ebi-a-GCST90001949 | ebi-a-GCST90001949 | TRUE | A | 51451043 | 4.69E-09 | reported | 1634 | 0.05258 | rs2411333 |  |  |  |  |  |  |  |  |  |  |  |  |  |  |  |
| 0.3694 | 19 | igd | 0.124 | T | \|\| id:ebi-a-GCST90001951 | ebi-a-GCST90001951 | TRUE | G | 51592949 | 1.53E-10 | reported | 1464 | 0.05728 | rs117439272 |  |  |  |  |  |  |  |  |  |  |  |  |  |  |  |
| -0.226 | 10 | igd | 0.1238 | G | \|\| id:ebi-a-GCST90001888 | ebi-a-GCST90001888 | TRUE | T | 97635721 | 3.69E-08 | reported | 2920 | 0.04094 | rs78679271 |  |  |  |  |  |  |  |  |  |  |  |  |  |  |  |
| -0.2895 | 10 | igd | 0.1238 | G | \|\| id:ebi-a-GCST90001892 | ebi-a-GCST90001892 | TRUE | T | 97635721 | 3.80E-12 | reported | 2920 | 0.04151 | rs78679271 |  |  |  |  |  |  |  |  |  |  |  |  |  |  |  |
| -0.2786 | 10 | igd | 0.1238 | G | \|\| id:ebi-a-GCST90002029 | ebi-a-GCST90002029 | TRUE | T | 97635721 | 1.91E-11 | reported | 2915 | 0.04134 | rs78679271 |  |  |  |  |  |  |  |  |  |  |  |  |  |  |  |
| -0.2974 | 10 | igd | 0.1238 | G | \|\| id:ebi-a-GCST90002061 | ebi-a-GCST90002061 | TRUE | T | 97635721 | 2.18E-12 | reported | 2920 | 0.04217 | rs78679271 |  |  |  |  |  |  |  |  |  |  |  |  |  |  |  |
| 0.3751 | 10 | igd | 0.1235 | A | \|\| id:ebi-a-GCST90001969 | ebi-a-GCST90001969 | TRUE | G | 126464649 | 5.97E-21 | reported | 3113 | 0.03966 | rs112140735 |  |  |  |  |  |  |  |  |  |  |  |  |  |  |  |
| 0.3961 | 10 | igd | 0.1235 | A | \|\| id:ebi-a-GCST90002076 | ebi-a-GCST90002076 | TRUE | G | 126464649 | 1.29E-22 | reported | 3113 | 0.04016 | rs112140735 |  |  |  |  |  |  |  |  |  |  |  |  |  |  |  |
| 0.3268 | 6 | igd | 0.1235 | C | \|\| id:ebi-a-GCST90002108 | ebi-a-GCST90002108 | TRUE | T | 33692368 | 2.37E-08 | reported | 1579 | 0.05824 | rs7744811 |  |  |  |  |  |  |  |  |  |  |  |  |  |  |  |
| -0.2533 | 10 | igd | 0.1231 | G | \|\| id:ebi-a-GCST90001484 | ebi-a-GCST90001484 | TRUE | A | 98146121 | 3.61E-11 | reported | 3437 | 0.03814 | rs72829522 |  |  |  |  |  |  |  |  |  |  |  |  |  |  |  |
| -0.2232 | 10 | igd | 0.1231 | G | \|\| id:ebi-a-GCST90001485 | ebi-a-GCST90001485 | TRUE | A | 98146121 | 5.30E-09 | reported | 3437 | 0.03814 | rs72829522 |  |  |  |  |  |  |  |  |  |  |  |  |  |  |  |
| -0.279 | 10 | igd | 0.1231 | G | \|\| id:ebi-a-GCST90001491 | ebi-a-GCST90001491 | TRUE | A | 98146121 | 9.23E-13 | reported | 3437 | 0.03891 | rs72829522 |  |  |  |  |  |  |  |  |  |  |  |  |  |  |  |
| -0.316 | 10 | igd | 0.1231 | G | \|\| id:ebi-a-GCST90001496 | ebi-a-GCST90001496 | TRUE | A | 98146121 | 1.36E-15 | reported | 3437 | 0.03936 | rs72829522 |  |  |  |  |  |  |  |  |  |  |  |  |  |  |  |
| -0.329 | 10 | igd | 0.1231 | G | \|\| id:ebi-a-GCST90001497 | ebi-a-GCST90001497 | TRUE | A | 98146121 | 3.49E-17 | reported | 3437 | 0.03883 | rs72829522 |  |  |  |  |  |  |  |  |  |  |  |  |  |  |  |
| -0.2512 | 7 | igd | 0.1228 | G | \|\| id:ebi-a-GCST90001966 | ebi-a-GCST90001966 | TRUE | A | 92485039 | 5.90E-09 | reported | 2850 | 0.04303 | rs7793983 |  |  |  |  |  |  |  |  |  |  |  |  |  |  |  |
| 0.2229 | 1 | igd | 0.1228 | T | \|\| id:ebi-a-GCST90001994 | ebi-a-GCST90001994 | TRUE | C | 161078538 | 6.55E-09 | reported | 3590 | 0.03833 | rs78241797 |  |  |  |  |  |  |  |  |  |  |  |  |  |  |  |
| -0.4617 | 10 | igd | 0.1226 | G | \|\| id:ebi-a-GCST90001489 | ebi-a-GCST90001489 | TRUE | T | 97635721 | 2.87E-32 | reported | 3405 | 0.03864 | rs78679271 |  |  |  |  |  |  |  |  |  |  |  |  |  |  |  |
| 0.3352 | 10 | igd | 0.1225 | C | \|\| id:ebi-a-GCST90001483 | ebi-a-GCST90001483 | TRUE | T | 98165797 | 5.13E-19 | reported | 3405 | 0.0374 | rs11599403 |  |  |  |  |  |  |  |  |  |  |  |  |  |  |  |
| 0.3212 | 10 | igd | 0.1225 | C | \|\| id:ebi-a-GCST90001489 | ebi-a-GCST90001489 | TRUE | T | 98165797 | 9.96E-17 | reported | 3405 | 0.03848 | rs11599403 |  |  |  |  |  |  |  |  |  |  |  |  |  |  |  |
| 0.384 | 10 | igd | 0.1225 | C | \|\| id:ebi-a-GCST90001495 | ebi-a-GCST90001495 | TRUE | T | 98165797 | 5.16E-23 | reported | 3405 | 0.03859 | rs11599403 |  |  |  |  |  |  |  |  |  |  |  |  |  |  |  |
| 0.2257 | 19 | igd | 0.12242 | A | \|\| id:prot-a-439 | prot-a-439 | TRUE | G | 51828848 | 3.63E-09 | reported | 3301 | 0.0383 | rs12979633 |  |  |  |  |  |  |  |  |  |  |  |  |  |  |  |
| 0.4134 | 10 | igd | 0.1224 | C | \|\| id:ebi-a-GCST90001660 | ebi-a-GCST90001660 | TRUE | T | 98165797 | 4.13E-27 | reported | 3408 | 0.03801 | rs11599403 |  |  |  |  |  |  |  |  |  |  |  |  |  |  |  |
| 0.3934 | 10 | igd | 0.1224 | C | \|\| id:ebi-a-GCST90001672 | ebi-a-GCST90001672 | TRUE | T | 98165797 | 3.25E-24 | reported | 3408 | 0.03846 | rs11599403 |  |  |  |  |  |  |  |  |  |  |  |  |  |  |  |
| 0.2579 | 12 | igd | 0.1224 | T | \|\| id:ebi-a-GCST90001798 | ebi-a-GCST90001798 | TRUE | C | 6605299 | 5.23E-12 | reported | 3657 | 0.03725 | rs147714617 |  |  |  |  |  |  |  |  |  |  |  |  |  |  |  |
| 0.264 | 12 | igd | 0.1224 | T | \|\| id:ebi-a-GCST90001804 | ebi-a-GCST90001804 | TRUE | C | 6605299 | 1.33E-12 | reported | 3657 | 0.03709 | rs147714617 |  |  |  |  |  |  |  |  |  |  |  |  |  |  |  |
| 0.2563 | 12 | igd | 0.1224 | T | \|\| id:ebi-a-GCST90001805 | ebi-a-GCST90001805 | TRUE | C | 6605299 | 1.11E-11 | reported | 3657 | 0.03761 | rs147714617 |  |  |  |  |  |  |  |  |  |  |  |  |  |  |  |
| 0.2484 | 12 | igd | 0.1224 | T | \|\| id:ebi-a-GCST90001806 | ebi-a-GCST90001806 | TRUE | C | 6605299 | 4.48E-11 | reported | 3656 | 0.03759 | rs147714617 |  |  |  |  |  |  |  |  |  |  |  |  |  |  |  |
| 0.299 | 12 | igd | 0.1224 | T | \|\| id:ebi-a-GCST90001808 | ebi-a-GCST90001808 | TRUE | C | 6605299 | 1.93E-15 | reported | 3657 | 0.03747 | rs147714617 |  |  |  |  |  |  |  |  |  |  |  |  |  |  |  |
| 0.389 | 10 | igd | 0.1224 | T | \|\| id:ebi-a-GCST90001969 | ebi-a-GCST90001969 | TRUE | C | 126346603 | 5.08E-22 | reported | 3113 | 0.04003 | rs1006368 |  |  |  |  |  |  |  |  |  |  |  |  |  |  |  |
| 0.4144 | 10 | igd | 0.1224 | T | \|\| id:ebi-a-GCST90002076 | ebi-a-GCST90002076 | TRUE | C | 126346603 | 3.61E-24 | reported | 3113 | 0.04052 | rs1006368 |  |  |  |  |  |  |  |  |  |  |  |  |  |  |  |
| -0.267 | 6 | igd | 0.1224 | A | \|\| id:ebi-a-GCST90002119 | ebi-a-GCST90002119 | TRUE | G | 29774691 | 1.98E-08 | reported | 2920 | 0.04743 | rs2523411 |  |  |  |  |  |  |  |  |  |  |  |  |  |  |  |
| 0.2594 | 3 | igd | 0.1224 | C | \|\| id:ebi-a-GCST90001997 | ebi-a-GCST90001997 | TRUE | T | 39160399 | 2.74E-11 | reported | 3590 | 0.03882 | rs1877564 |  |  |  |  |  |  |  |  |  |  |  |  |  |  |  |
| 0.3776 | 10 | igd | 0.1223 | C | \|\| id:ebi-a-GCST90001484 | ebi-a-GCST90001484 | TRUE | T | 98165797 | 4.30E-24 | reported | 3437 | 0.03702 | rs11599403 |  |  |  |  |  |  |  |  |  |  |  |  |  |  |  |
| 0.3493 | 10 | igd | 0.1223 | C | \|\| id:ebi-a-GCST90001485 | ebi-a-GCST90001485 | TRUE | T | 98165797 | 7.30E-21 | reported | 3437 | 0.03704 | rs11599403 |  |  |  |  |  |  |  |  |  |  |  |  |  |  |  |
| 0.3447 | 10 | igd | 0.1223 | C | \|\| id:ebi-a-GCST90001491 | ebi-a-GCST90001491 | TRUE | T | 98165797 | 1.11E-19 | reported | 3437 | 0.03774 | rs11599403 |  |  |  |  |  |  |  |  |  |  |  |  |  |  |  |
| 0.4281 | 10 | igd | 0.1223 | C | \|\| id:ebi-a-GCST90001496 | ebi-a-GCST90001496 | TRUE | T | 98165797 | 6.50E-29 | reported | 3437 | 0.03802 | rs11599403 |  |  |  |  |  |  |  |  |  |  |  |  |  |  |  |
| 0.4111 | 10 | igd | 0.1223 | C | \|\| id:ebi-a-GCST90001497 | ebi-a-GCST90001497 | TRUE | T | 98165797 | 2.05E-27 | reported | 3437 | 0.03758 | rs11599403 |  |  |  |  |  |  |  |  |  |  |  |  |  |  |  |
| 0.4277 | 10 | igd | 0.1222 | C | \|\| id:ebi-a-GCST90001658 | ebi-a-GCST90001658 | TRUE | T | 98165797 | 1.07E-31 | reported | 3440 | 0.03614 | rs11599403 |  |  |  |  |  |  |  |  |  |  |  |  |  |  |  |
| 0.447 | 10 | igd | 0.1222 | C | \|\| id:ebi-a-GCST90001659 | ebi-a-GCST90001659 | TRUE | T | 98165797 | 1.59E-34 | reported | 3440 | 0.03608 | rs11599403 |  |  |  |  |  |  |  |  |  |  |  |  |  |  |  |
| 0.4068 | 10 | igd | 0.1222 | C | \|\| id:ebi-a-GCST90001670 | ebi-a-GCST90001670 | TRUE | T | 98165797 | 1.27E-26 | reported | 3440 | 0.03778 | rs11599403 |  |  |  |  |  |  |  |  |  |  |  |  |  |  |  |
| 0.4155 | 10 | igd | 0.1222 | C | \|\| id:ebi-a-GCST90001671 | ebi-a-GCST90001671 | TRUE | T | 98165797 | 1.10E-27 | reported | 3440 | 0.03777 | rs11599403 |  |  |  |  |  |  |  |  |  |  |  |  |  |  |  |
| 0.2607 | 10 | igd | 0.1221 | C | \|\| id:ebi-a-GCST90001484 | ebi-a-GCST90001484 | TRUE | T | 98269285 | 2.59E-12 | reported | 3437 | 0.03712 | rs74479493 |  |  |  |  |  |  |  |  |  |  |  |  |  |  |  |
| -0.5552 | 22 | igd | 0.122 | A | \|\| id:ebi-a-GCST90001702 | ebi-a-GCST90001702 | TRUE | G | 42348062 | 2.10E-47 | reported | 3657 | 0.03784 | rs28722907 |  |  |  |  |  |  |  |  |  |  |  |  |  |  |  |
| -0.5329 | 22 | igd | 0.122 | A | \|\| id:ebi-a-GCST90001703 | ebi-a-GCST90001703 | TRUE | G | 42348062 | 9.57E-44 | reported | 3657 | 0.03791 | rs28722907 |  |  |  |  |  |  |  |  |  |  |  |  |  |  |  |
| -0.4573 | 22 | igd | 0.122 | A | \|\| id:ebi-a-GCST90001704 | ebi-a-GCST90001704 | TRUE | G | 42348062 | 1.52E-32 | reported | 3657 | 0.03812 | rs28722907 |  |  |  |  |  |  |  |  |  |  |  |  |  |  |  |
| -0.5391 | 22 | igd | 0.122 | A | \|\| id:ebi-a-GCST90001710 | ebi-a-GCST90001710 | TRUE | G | 42348062 | 6.11E-45 | reported | 3657 | 0.03781 | rs28722907 |  |  |  |  |  |  |  |  |  |  |  |  |  |  |  |
| -0.5214 | 22 | igd | 0.122 | A | \|\| id:ebi-a-GCST90001711 | ebi-a-GCST90001711 | TRUE | G | 42348062 | 2.41E-42 | reported | 3657 | 0.03775 | rs28722907 |  |  |  |  |  |  |  |  |  |  |  |  |  |  |  |
| 0.206 | 12 | igd | 0.1216 | T | \|\| id:ebi-a-GCST90001803 | ebi-a-GCST90001803 | TRUE | C | 6605299 | 4.21E-08 | reported | 3642 | 0.03751 | rs147714617 |  |  |  |  |  |  |  |  |  |  |  |  |  |  |  |
| -0.2746 | 3 | igd | 0.1216 | T | \|\| id:ebi-a-GCST90001997 | ebi-a-GCST90001997 | TRUE | A | 39081337 | 7.43E-13 | reported | 3590 | 0.03816 | rs55677214 |  |  |  |  |  |  |  |  |  |  |  |  |  |  |  |
| -0.2436 | 3 | igd | 0.1216 | T | \|\| id:ebi-a-GCST90001995 | ebi-a-GCST90001995 | TRUE | A | 39081337 | 1.54E-10 | reported | 3590 | 0.03794 | rs55677214 |  |  |  |  |  |  |  |  |  |  |  |  |  |  |  |
| 0.2092 | 14 | igd | 0.1214 | A | \|\| id:ebi-a-GCST90001821 | ebi-a-GCST90001821 | TRUE | C | 104133530 | 3.36E-08 | reported | 3656 | 0.03781 | rs151199076 |  |  |  |  |  |  |  |  |  |  |  |  |  |  |  |
| 0.3417 | 6 | igd | 0.121 | A | \|\| id:ebi-a-GCST90002108 | ebi-a-GCST90002108 | TRUE | G | 30783294 | 1.18E-08 | reported | 1579 | 0.0596 | rs71541426 |  |  |  |  |  |  |  |  |  |  |  |  |  |  |  |
| -0.2721 | 10 | igd | 0.1206 | A | \|\| id:ebi-a-GCST90002029 | ebi-a-GCST90002029 | TRUE | G | 97494858 | 9.07E-11 | reported | 2915 | 0.04182 | rs112850881 |  |  |  |  |  |  |  |  |  |  |  |  |  |  |  |
| -0.2674 | 1 | igd | 0.1206 | C | \|\| id:ebi-a-GCST90002002 | ebi-a-GCST90002002 | TRUE | G | 161380701 | 2.60E-12 | reported | 3629 | 0.03809 | rs143029017 |  |  |  |  |  |  |  |  |  |  |  |  |  |  |  |
| -0.2615 | 10 | igd | 0.1205 | A | \|\| id:ebi-a-GCST90001892 | ebi-a-GCST90001892 | TRUE | G | 97494858 | 5.75E-10 | reported | 2920 | 0.04206 | rs112850881 |  |  |  |  |  |  |  |  |  |  |  |  |  |  |  |
| 0.4335 | 10 | igd | 0.1205 | A | \|\| id:ebi-a-GCST90001961 | ebi-a-GCST90001961 | TRUE | G | 97494858 | 1.22E-24 | reported | 2920 | 0.04192 | rs112850881 |  |  |  |  |  |  |  |  |  |  |  |  |  |  |  |
| -0.6653 | 10 | igd | 0.1205 | A | \|\| id:ebi-a-GCST90002030 | ebi-a-GCST90002030 | TRUE | G | 97494858 | 7.96E-59 | reported | 2920 | 0.04022 | rs112850881 |  |  |  |  |  |  |  |  |  |  |  |  |  |  |  |
| -0.583 | 10 | igd | 0.1205 | A | \|\| id:ebi-a-GCST90002031 | ebi-a-GCST90002031 | TRUE | G | 97494858 | 5.83E-43 | reported | 2920 | 0.04174 | rs112850881 |  |  |  |  |  |  |  |  |  |  |  |  |  |  |  |
| -0.5405 | 10 | igd | 0.1205 | A | \|\| id:ebi-a-GCST90002032 | ebi-a-GCST90002032 | TRUE | G | 97494858 | 4.59E-37 | reported | 2920 | 0.0419 | rs112850881 |  |  |  |  |  |  |  |  |  |  |  |  |  |  |  |
| -0.2832 | 10 | igd | 0.1205 | A | \|\| id:ebi-a-GCST90002061 | ebi-a-GCST90002061 | TRUE | G | 97494858 | 3.94E-11 | reported | 2920 | 0.0427 | rs112850881 |  |  |  |  |  |  |  |  |  |  |  |  |  |  |  |
| 0.2446 | 12 | igd | 0.1205 | G | \|\| id:ebi-a-GCST90002073 | ebi-a-GCST90002073 | TRUE | A | 69633070 | 5.33E-09 | reported | 2850 | 0.04178 | rs2231689 |  |  |  |  |  |  |  |  |  |  |  |  |  |  |  |
| 0.5528 | 6 | igd | 0.1205 | T | \|\| id:ebi-a-GCST90002111 | ebi-a-GCST90002111 | TRUE | A | 32560385 | 1.48E-21 | reported | 1635 | 0.05716 | rs34291045 |  |  |  |  |  |  |  |  |  |  |  |  |  |  |  |
| 0.5258 | 6 | igd | 0.1203 | T | \|\| id:ebi-a-GCST90002110 | ebi-a-GCST90002110 | TRUE | A | 32560385 | 4.67E-19 | reported | 1634 | 0.05823 | rs34291045 |  |  |  |  |  |  |  |  |  |  |  |  |  |  |  |
| 0.2286 | 12 | igd | 0.1202 | T | \|\| id:ebi-a-GCST90002074 | ebi-a-GCST90002074 | TRUE | C | 69618568 | 1.28E-08 | reported | 3112 | 0.04007 | rs73141971 |  |  |  |  |  |  |  |  |  |  |  |  |  |  |  |
| 1.049 | 6 | igd | 0.1202 | A | \|\| id:ebi-a-GCST90002112 | ebi-a-GCST90002112 | TRUE | G | 32565201 | 2.44E-60 | reported | 1635 | 0.06137 | rs2760980 |  |  |  |  |  |  |  |  |  |  |  |  |  |  |  |
| -0.2653 | 20 | igd | 0.12 | T | \|\| id:ebi-a-GCST90001802 | ebi-a-GCST90001802 | TRUE | A | 44738625 | 3.48E-12 | reported | 3657 | 0.038 | rs78121022 |  |  |  |  |  |  |  |  |  |  |  |  |  |  |  |
| -0.2751 | 17 | igd | 0.12 | C | \|\| id:ebi-a-GCST90001964 | ebi-a-GCST90001964 | TRUE | T | 62181990 | 5.73E-11 | reported | 2872 | 0.04183 | rs57891429 |  |  |  |  |  |  |  |  |  |  |  |  |  |  |  |
| 0.4857 | 19 | igd | 0.1199 | C | \|\| id:ebi-a-GCST90001951 | ebi-a-GCST90001951 | TRUE | G | 51704651 | 1.02E-16 | reported | 1464 | 0.0578 | rs273620 |  |  |  |  |  |  |  |  |  |  |  |  |  |  |  |
| -0.2635 | 10 | igd | 0.1199 | A | \|\| id:ebi-a-GCST90002030 | ebi-a-GCST90002030 | TRUE | C | 92354492 | 2.78E-10 | reported | 2920 | 0.0416 | rs75666171 |  |  |  |  |  |  |  |  |  |  |  |  |  |  |  |
| 0.3639 | 1 | igd | 0.1198 | C | \|\| id:ebi-a-GCST90001866 | ebi-a-GCST90001866 | TRUE | T | 167413475 | 5.06E-19 | reported | 2914 | 0.04056 | rs74524218 |  |  |  |  |  |  |  |  |  |  |  |  |  |  |  |
| 0.4889 | 1 | igd | 0.1197 | C | \|\| id:ebi-a-GCST90001845 | ebi-a-GCST90001845 | TRUE | T | 167413475 | 1.99E-32 | reported | 2911 | 0.04073 | rs74524218 |  |  |  |  |  |  |  |  |  |  |  |  |  |  |  |
| 0.3903 | 1 | igd | 0.1197 | C | \|\| id:ebi-a-GCST90001854 | ebi-a-GCST90001854 | TRUE | T | 167413475 | 1.83E-21 | reported | 2919 | 0.0407 | rs74524218 |  |  |  |  |  |  |  |  |  |  |  |  |  |  |  |
| 0.3478 | 1 | igd | 0.1197 | C | \|\| id:ebi-a-GCST90001856 | ebi-a-GCST90001856 | TRUE | T | 167413475 | 4.92E-17 | reported | 2919 | 0.04121 | rs74524218 |  |  |  |  |  |  |  |  |  |  |  |  |  |  |  |
| 0.5526 | 1 | igd | 0.1197 | C | \|\| id:ebi-a-GCST90001858 | ebi-a-GCST90001858 | TRUE | T | 167413475 | 1.46E-41 | reported | 2919 | 0.04028 | rs74524218 |  |  |  |  |  |  |  |  |  |  |  |  |  |  |  |
| 0.4446 | 1 | igd | 0.1197 | C | \|\| id:ebi-a-GCST90001860 | ebi-a-GCST90001860 | TRUE | T | 167413475 | 6.86E-27 | reported | 2919 | 0.041 | rs74524218 |  |  |  |  |  |  |  |  |  |  |  |  |  |  |  |
| 0.4472 | 1 | igd | 0.1197 | C | \|\| id:ebi-a-GCST90001861 | ebi-a-GCST90001861 | TRUE | T | 167413475 | 4.90E-28 | reported | 2919 | 0.04031 | rs74524218 |  |  |  |  |  |  |  |  |  |  |  |  |  |  |  |
| 0.4435 | 1 | igd | 0.1197 | C | \|\| id:ebi-a-GCST90001863 | ebi-a-GCST90001863 | TRUE | T | 167413475 | 7.46E-28 | reported | 2919 | 0.04012 | rs74524218 |  |  |  |  |  |  |  |  |  |  |  |  |  |  |  |
| 0.606 | 1 | igd | 0.1197 | C | \|\| id:ebi-a-GCST90001864 | ebi-a-GCST90001864 | TRUE | T | 167413475 | 6.52E-50 | reported | 2919 | 0.04002 | rs74524218 |  |  |  |  |  |  |  |  |  |  |  |  |  |  |  |
| 0.2821 | 1 | igd | 0.1197 | C | \|\| id:ebi-a-GCST90001865 | ebi-a-GCST90001865 | TRUE | T | 167413475 | 1.41E-11 | reported | 2919 | 0.04157 | rs74524218 |  |  |  |  |  |  |  |  |  |  |  |  |  |  |  |
| 0.2964 | 12 | igd | 0.1195 | C | \|\| id:ebi-a-GCST90001802 | ebi-a-GCST90001802 | TRUE | A | 6615199 | 8.33E-15 | reported | 3657 | 0.03803 | rs146983427 |  |  |  |  |  |  |  |  |  |  |  |  |  |  |  |
| 0.2823 | 1 | igd | 0.1195 | C | \|\| id:ebi-a-GCST90001852 | ebi-a-GCST90001852 | TRUE | T | 167413475 | 3.07E-11 | reported | 2649 | 0.04231 | rs74524218 |  |  |  |  |  |  |  |  |  |  |  |  |  |  |  |
| 0.4428 | 10 | igd | 0.1193 | C | \|\| id:ebi-a-GCST90002030 | ebi-a-GCST90002030 | TRUE | T | 98165797 | 5.35E-27 | reported | 2920 | 0.04075 | rs11599403 |  |  |  |  |  |  |  |  |  |  |  |  |  |  |  |
| 0.3936 | 10 | igd | 0.1193 | C | \|\| id:ebi-a-GCST90002031 | ebi-a-GCST90002031 | TRUE | T | 98165797 | 1.25E-20 | reported | 2920 | 0.04194 | rs11599403 |  |  |  |  |  |  |  |  |  |  |  |  |  |  |  |
| 0.3315 | 10 | igd | 0.1193 | C | \|\| id:ebi-a-GCST90002032 | ebi-a-GCST90002032 | TRUE | T | 98165797 | 4.80E-15 | reported | 2920 | 0.0421 | rs11599403 |  |  |  |  |  |  |  |  |  |  |  |  |  |  |  |
| -0.6882 | 6 | igd | 0.1193 | A | \|\| id:ebi-a-GCST90002110 | ebi-a-GCST90002110 | TRUE | G | 32623528 | 2.09E-12 | reported | 1634 | 0.09717 | rs73732214 |  |  |  |  |  |  |  |  |  |  |  |  |  |  |  |
| -0.573 | 6 | igd | 0.1193 | A | \|\| id:ebi-a-GCST90002111 | ebi-a-GCST90002111 | TRUE | G | 32623528 | 3.22E-09 | reported | 1635 | 0.09626 | rs73732214 |  |  |  |  |  |  |  |  |  |  |  |  |  |  |  |
| -0.4587 | 10 | igd | 0.1189 | A | \|\| id:ebi-a-GCST90001489 | ebi-a-GCST90001489 | TRUE | G | 97494858 | 5.43E-31 | reported | 3405 | 0.03923 | rs112850881 |  |  |  |  |  |  |  |  |  |  |  |  |  |  |  |
| -0.5195 | 10 | igd | 0.1189 | A | \|\| id:ebi-a-GCST90001495 | ebi-a-GCST90001495 | TRUE | G | 97494858 | 7.69E-39 | reported | 3405 | 0.03935 | rs112850881 |  |  |  |  |  |  |  |  |  |  |  |  |  |  |  |
| -0.2714 | 7 | igd | 0.1189 | T | \|\| id:ebi-a-GCST90002078 | ebi-a-GCST90002078 | TRUE | C | 92374091 | 5.12E-11 | reported | 3112 | 0.04118 | rs73232041 |  |  |  |  |  |  |  |  |  |  |  |  |  |  |  |
| -0.5271 | 10 | igd | 0.1188 | A | \|\| id:ebi-a-GCST90001660 | ebi-a-GCST90001660 | TRUE | G | 97494858 | 7.02E-41 | reported | 3408 | 0.03885 | rs112850881 |  |  |  |  |  |  |  |  |  |  |  |  |  |  |  |
| -0.5251 | 10 | igd | 0.1188 | A | \|\| id:ebi-a-GCST90001672 | ebi-a-GCST90001672 | TRUE | G | 97494858 | 8.47E-40 | reported | 3408 | 0.03926 | rs112850881 |  |  |  |  |  |  |  |  |  |  |  |  |  |  |  |
| -0.4223 | 10 | igd | 0.1187 | A | \|\| id:ebi-a-GCST90001484 | ebi-a-GCST90001484 | TRUE | G | 97494858 | 3.60E-28 | reported | 3437 | 0.03803 | rs112850881 |  |  |  |  |  |  |  |  |  |  |  |  |  |  |  |
| -0.462 | 10 | igd | 0.1187 | A | \|\| id:ebi-a-GCST90001491 | ebi-a-GCST90001491 | TRUE | G | 97494858 | 2.43E-32 | reported | 3437 | 0.03862 | rs112850881 |  |  |  |  |  |  |  |  |  |  |  |  |  |  |  |
| -0.5457 | 10 | igd | 0.1187 | A | \|\| id:ebi-a-GCST90001496 | ebi-a-GCST90001496 | TRUE | G | 97494858 | 1.25E-43 | reported | 3437 | 0.03884 | rs112850881 |  |  |  |  |  |  |  |  |  |  |  |  |  |  |  |
| -0.5524 | 10 | igd | 0.1187 | A | \|\| id:ebi-a-GCST90001497 | ebi-a-GCST90001497 | TRUE | G | 97494858 | 7.89E-46 | reported | 3437 | 0.0383 | rs112850881 |  |  |  |  |  |  |  |  |  |  |  |  |  |  |  |
| -0.5364 | 10 | igd | 0.1186 | A | \|\| id:ebi-a-GCST90001658 | ebi-a-GCST90001658 | TRUE | G | 97494858 | 2.11E-46 | reported | 3440 | 0.03695 | rs112850881 |  |  |  |  |  |  |  |  |  |  |  |  |  |  |  |
| -0.549 | 10 | igd | 0.1186 | A | \|\| id:ebi-a-GCST90001659 | ebi-a-GCST90001659 | TRUE | G | 97494858 | 1.45E-48 | reported | 3440 | 0.0369 | rs112850881 |  |  |  |  |  |  |  |  |  |  |  |  |  |  |  |
| -0.5292 | 10 | igd | 0.1186 | A | \|\| id:ebi-a-GCST90001670 | ebi-a-GCST90001670 | TRUE | G | 97494858 | 1.00E-41 | reported | 3440 | 0.03858 | rs112850881 |  |  |  |  |  |  |  |  |  |  |  |  |  |  |  |
| -0.5303 | 10 | igd | 0.1186 | A | \|\| id:ebi-a-GCST90001671 | ebi-a-GCST90001671 | TRUE | G | 97494858 | 8.67E-42 | reported | 3440 | 0.03863 | rs112850881 |  |  |  |  |  |  |  |  |  |  |  |  |  |  |  |
| -0.2906 | 1 | igd | 0.1185 | G | \|\| id:ebi-a-GCST90001845 | ebi-a-GCST90001845 | TRUE | A | 167217843 | 6.41E-12 | reported | 2911 | 0.04213 | rs72693612 |  |  |  |  |  |  |  |  |  |  |  |  |  |  |  |
| -0.2384 | 20 | igd | 0.1184 | T | \|\| id:ebi-a-GCST90001804 | ebi-a-GCST90001804 | TRUE | C | 44814996 | 7.01E-10 | reported | 3657 | 0.03856 | rs74533257 |  |  |  |  |  |  |  |  |  |  |  |  |  |  |  |
| -0.2384 | 20 | igd | 0.1184 | T | \|\| id:ebi-a-GCST90001808 | ebi-a-GCST90001808 | TRUE | C | 44814996 | 1.14E-09 | reported | 3657 | 0.03906 | rs74533257 |  |  |  |  |  |  |  |  |  |  |  |  |  |  |  |
| 0.3101 | 6 | igd | 0.1184 | A | \|\| id:ebi-a-GCST90002105 | ebi-a-GCST90002105 | TRUE | G | 30783294 | 7.59E-11 | reported | 2872 | 0.04746 | rs71541426 |  |  |  |  |  |  |  |  |  |  |  |  |  |  |  |
| 0.2439 | 20 | igd | 0.118 | C | \|\| id:ebi-a-GCST90001798 | ebi-a-GCST90001798 | TRUE | T | 45095161 | 4.71E-10 | reported | 3657 | 0.03905 | rs6011967 |  |  |  |  |  |  |  |  |  |  |  |  |  |  |  |
| 0.2509 | 20 | igd | 0.118 | C | \|\| id:ebi-a-GCST90001804 | ebi-a-GCST90001804 | TRUE | T | 45095161 | 1.24E-10 | reported | 3657 | 0.03888 | rs6011967 |  |  |  |  |  |  |  |  |  |  |  |  |  |  |  |
| 0.2385 | 20 | igd | 0.118 | C | \|\| id:ebi-a-GCST90001808 | ebi-a-GCST90001808 | TRUE | T | 45095161 | 1.52E-09 | reported | 3657 | 0.03937 | rs6011967 |  |  |  |  |  |  |  |  |  |  |  |  |  |  |  |
| 0.2842 | 10 | igd | 0.1177 | A | \|\| id:ebi-a-GCST90001777 | ebi-a-GCST90001777 | TRUE | C | 6078079 | 6.75E-14 | reported | 3657 | 0.03778 | rs6602392 |  |  |  |  |  |  |  |  |  |  |  |  |  |  |  |
| 0.2309 | 10 | igd | 0.1177 | A | \|\| id:ebi-a-GCST90001778 | ebi-a-GCST90001778 | TRUE | C | 6078079 | 1.51E-09 | reported | 3657 | 0.03811 | rs6602392 |  |  |  |  |  |  |  |  |  |  |  |  |  |  |  |
| 0.2856 | 10 | igd | 0.1177 | A | \|\| id:ebi-a-GCST90001790 | ebi-a-GCST90001790 | TRUE | C | 6078079 | 5.30E-14 | reported | 3657 | 0.03781 | rs6602392 |  |  |  |  |  |  |  |  |  |  |  |  |  |  |  |
| 0.2479 | 10 | igd | 0.1177 | A | \|\| id:ebi-a-GCST90001792 | ebi-a-GCST90001792 | TRUE | C | 6078079 | 6.98E-11 | reported | 3657 | 0.03791 | rs6602392 |  |  |  |  |  |  |  |  |  |  |  |  |  |  |  |
| 0.2751 | 10 | igd | 0.1177 | A | \|\| id:ebi-a-GCST90001793 | ebi-a-GCST90001793 | TRUE | C | 6078079 | 6.56E-13 | reported | 3657 | 0.03813 | rs6602392 |  |  |  |  |  |  |  |  |  |  |  |  |  |  |  |
| -0.2753 | 7 | igd | 0.1175 | T | \|\| id:ebi-a-GCST90001966 | ebi-a-GCST90001966 | TRUE | C | 92374091 | 2.83E-10 | reported | 2850 | 0.0435 | rs73232041 |  |  |  |  |  |  |  |  |  |  |  |  |  |  |  |
| 0.2375 | 12 | igd | 0.1175 | G | \|\| id:ebi-a-GCST90002073 | ebi-a-GCST90002073 | TRUE | C | 69733190 | 1.90E-08 | reported | 2850 | 0.04213 | rs73145927 |  |  |  |  |  |  |  |  |  |  |  |  |  |  |  |
| 0.4121 | 6 | igd | 0.1175 | C | \|\| id:ebi-a-GCST90002110 | ebi-a-GCST90002110 | TRUE | T | 32209943 | 1.67E-11 | reported | 1634 | 0.06078 | rs75057504 |  |  |  |  |  |  |  |  |  |  |  |  |  |  |  |
| -0.4974 | 2 | igd | 0.1175 | G | \|\| id:ebi-a-GCST90002118 | ebi-a-GCST90002118 | TRUE | C | 86982504 | 5.63E-32 | reported | 2920 | 0.04176 | rs13004867 |  |  |  |  |  |  |  |  |  |  |  |  |  |  |  |
| -0.206996 | 10 | igd | 0.11745 | G | \|\| id:met-b-156 | met-b-156 | TRUE | T | 97406816 | 3.12E-13 | reported | 447 | 0.0283978 | rs7897091 |  |  |  |  |  |  |  |  |  |  |  |  |  |  |  |
| 0.3576 | 6 | igd | 0.1174 | C | \|\| id:ebi-a-GCST90002111 | ebi-a-GCST90002111 | TRUE | T | 32209943 | 3.14E-09 | reported | 1635 | 0.06003 | rs75057504 |  |  |  |  |  |  |  |  |  |  |  |  |  |  |  |
| 1.217 | 6 | igd | 0.1173 | G | \|\| id:ebi-a-GCST90002105 | ebi-a-GCST90002105 | TRUE | T | 32570311 | 6.25E-146 | reported | 2872 | 0.0446 | rs34039593 |  |  |  |  |  |  |  |  |  |  |  |  |  |  |  |
| -0.2355 | 17 | igd | 0.1172 | T | \|\| id:ebi-a-GCST90001654 | ebi-a-GCST90001654 | TRUE | C | 33793208 | 2.77E-09 | reported | 3408 | 0.03952 | rs75361910 |  |  |  |  |  |  |  |  |  |  |  |  |  |  |  |
| -0.301 | 6 | igd | 0.1168 | C | \|\| id:ebi-a-GCST90002119 | ebi-a-GCST90002119 | TRUE | A | 30343272 | 9.61E-10 | reported | 2920 | 0.04905 | rs3132634 |  |  |  |  |  |  |  |  |  |  |  |  |  |  |  |
| 0.2557 | 6 | igd | 0.1168 | T | \|\| id:ebi-a-GCST90002119 | ebi-a-GCST90002119 | TRUE | C | 29756800 | 1.96E-08 | reported | 2920 | 0.0454 | rs2394172 |  |  |  |  |  |  |  |  |  |  |  |  |  |  |  |
| 0.2356 | 1 | igd | 0.116 | C | \|\| id:ebi-a-GCST90001864 | ebi-a-GCST90001864 | TRUE | G | 166922731 | 4.26E-08 | reported | 2919 | 0.04288 | rs2142615 |  |  |  |  |  |  |  |  |  |  |  |  |  |  |  |
| -0.2467 | 7 | igd | 0.1154 | T | \|\| id:ebi-a-GCST90002078 | ebi-a-GCST90002078 | TRUE | G | 92073947 | 3.54E-09 | reported | 3112 | 0.04166 | rs78168358 |  |  |  |  |  |  |  |  |  |  |  |  |  |  |  |
| -0.2814 | 22 | igd | 0.1153 | C | \|\| id:ebi-a-GCST90001703 | ebi-a-GCST90001703 | TRUE | A | 38526882 | 1.21E-11 | reported | 3657 | 0.04137 | rs201113645 |  |  |  |  |  |  |  |  |  |  |  |  |  |  |  |
| -0.2616 | 22 | igd | 0.1153 | C | \|\| id:ebi-a-GCST90001711 | ebi-a-GCST90001711 | TRUE | A | 38526882 | 2.42E-10 | reported | 3657 | 0.0412 | rs201113645 |  |  |  |  |  |  |  |  |  |  |  |  |  |  |  |
| -0.2255 | 22 | igd | 0.1144 | C | \|\| id:ebi-a-GCST90001702 | ebi-a-GCST90001702 | TRUE | G | 41293117 | 2.47E-08 | reported | 3657 | 0.04036 | rs71325197 |  |  |  |  |  |  |  |  |  |  |  |  |  |  |  |
| -0.2507 | 10 | igd | 0.114 | G | \|\| id:ebi-a-GCST90002030 | ebi-a-GCST90002030 | TRUE | T | 95850334 | 5.42E-09 | reported | 2920 | 0.04285 | rs79016143 |  |  |  |  |  |  |  |  |  |  |  |  |  |  |  |
| -0.2458 | 10 | igd | 0.114 | G | \|\| id:ebi-a-GCST90002031 | ebi-a-GCST90002031 | TRUE | T | 95850334 | 2.17E-08 | reported | 2920 | 0.04379 | rs79016143 |  |  |  |  |  |  |  |  |  |  |  |  |  |  |  |
| -0.2218 | 11 | igd | 0.1139 | T | \|\| id:ebi-a-GCST90001752 | ebi-a-GCST90001752 | TRUE | G | 60361184 | 1.93E-08 | reported | 3657 | 0.03939 | rs74593111 |  |  |  |  |  |  |  |  |  |  |  |  |  |  |  |
| -0.2257 | 11 | igd | 0.1139 | T | \|\| id:ebi-a-GCST90001761 | ebi-a-GCST90001761 | TRUE | G | 60361184 | 1.24E-08 | reported | 3657 | 0.03955 | rs74593111 |  |  |  |  |  |  |  |  |  |  |  |  |  |  |  |
| 0.2941 | 2 | igd | 0.1139 | A | \|\| id:ebi-a-GCST90001894 | ebi-a-GCST90001894 | TRUE | G | 204546960 | 9.27E-12 | reported | 2920 | 0.04297 | rs75329808 |  |  |  |  |  |  |  |  |  |  |  |  |  |  |  |
| 0.3074 | 2 | igd | 0.1139 | A | \|\| id:ebi-a-GCST90001898 | ebi-a-GCST90001898 | TRUE | G | 204546960 | 1.04E-12 | reported | 2920 | 0.04296 | rs75329808 |  |  |  |  |  |  |  |  |  |  |  |  |  |  |  |
| 0.4688 | 3 | igd | 0.1138 | A | \|\| id:ebi-a-GCST90001801 | ebi-a-GCST90001801 | TRUE | T | 32792072 | 3.45E-18 | reported | 1836 | 0.05335 | rs62251884 |  |  |  |  |  |  |  |  |  |  |  |  |  |  |  |
| 0.2196 | 3 | igd | 0.1136 | A | \|\| id:ebi-a-GCST90001798 | ebi-a-GCST90001798 | TRUE | G | 32302456 | 2.67E-08 | reported | 3657 | 0.03939 | rs7621492 |  |  |  |  |  |  |  |  |  |  |  |  |  |  |  |
| 0.2435 | 3 | igd | 0.1136 | A | \|\| id:ebi-a-GCST90001800 | ebi-a-GCST90001800 | TRUE | G | 32302456 | 1.18E-09 | reported | 3656 | 0.03994 | rs7621492 |  |  |  |  |  |  |  |  |  |  |  |  |  |  |  |
| 0.2178 | 3 | igd | 0.1136 | A | \|\| id:ebi-a-GCST90001806 | ebi-a-GCST90001806 | TRUE | G | 32302456 | 4.66E-08 | reported | 3656 | 0.03977 | rs7621492 |  |  |  |  |  |  |  |  |  |  |  |  |  |  |  |
| -0.429 | 22 | igd | 0.1133 | T | \|\| id:ebi-a-GCST90001702 | ebi-a-GCST90001702 | TRUE | C | 43092556 | 2.92E-27 | reported | 3657 | 0.03935 | rs6002907 |  |  |  |  |  |  |  |  |  |  |  |  |  |  |  |
| -0.4109 | 22 | igd | 0.1133 | T | \|\| id:ebi-a-GCST90001703 | ebi-a-GCST90001703 | TRUE | C | 43092556 | 3.98E-25 | reported | 3657 | 0.03939 | rs6002907 |  |  |  |  |  |  |  |  |  |  |  |  |  |  |  |
| -0.3815 | 22 | igd | 0.1133 | T | \|\| id:ebi-a-GCST90001704 | ebi-a-GCST90001704 | TRUE | C | 43092556 | 6.88E-22 | reported | 3657 | 0.03942 | rs6002907 |  |  |  |  |  |  |  |  |  |  |  |  |  |  |  |
| -0.4206 | 22 | igd | 0.1133 | T | \|\| id:ebi-a-GCST90001710 | ebi-a-GCST90001710 | TRUE | C | 43092556 | 2.19E-26 | reported | 3657 | 0.03926 | rs6002907 |  |  |  |  |  |  |  |  |  |  |  |  |  |  |  |
| -0.4128 | 22 | igd | 0.1133 | T | \|\| id:ebi-a-GCST90001711 | ebi-a-GCST90001711 | TRUE | C | 43092556 | 1.36E-25 | reported | 3657 | 0.03918 | rs6002907 |  |  |  |  |  |  |  |  |  |  |  |  |  |  |  |
| -0.175679 | 10 | igd | 0.113043 | C | \|\| id:met-b-156 | met-b-156 | TRUE | A | 97372669 | 2.37E-08 | reported | 460 | 0.0314676 | rs1409710 |  |  |  |  |  |  |  |  |  |  |  |  |  |  |  |
| 0.3839 | 6 | igd | 0.1128 | T | \|\| id:ebi-a-GCST90002112 | ebi-a-GCST90002112 | TRUE | C | 33667660 | 1.72E-10 | reported | 1635 | 0.05974 | rs73743305 |  |  |  |  |  |  |  |  |  |  |  |  |  |  |  |
| 0.2627 | 6 | igd | 0.1127 | T | \|\| id:ebi-a-GCST90001765 | ebi-a-GCST90001765 | TRUE | C | 107087063 | 5.70E-12 | reported | 3655 | 0.03802 | rs112925176 |  |  |  |  |  |  |  |  |  |  |  |  |  |  |  |
| 0.2852 | 6 | igd | 0.1127 | T | \|\| id:ebi-a-GCST90001772 | ebi-a-GCST90001772 | TRUE | C | 107087063 | 9.93E-14 | reported | 3655 | 0.03817 | rs112925176 |  |  |  |  |  |  |  |  |  |  |  |  |  |  |  |
| 0.2587 | 6 | igd | 0.1126 | T | \|\| id:ebi-a-GCST90001771 | ebi-a-GCST90001771 | TRUE | C | 107087063 | 1.35E-11 | reported | 3653 | 0.03813 | rs112925176 |  |  |  |  |  |  |  |  |  |  |  |  |  |  |  |
| -0.2272 | 10 | igd | 0.1125 | T | \|\| id:ebi-a-GCST90001484 | ebi-a-GCST90001484 | TRUE | C | 98160099 | 1.39E-08 | reported | 3437 | 0.03993 | rs72640002 |  |  |  |  |  |  |  |  |  |  |  |  |  |  |  |
| -0.2549 | 10 | igd | 0.1125 | T | \|\| id:ebi-a-GCST90001496 | ebi-a-GCST90001496 | TRUE | C | 98160099 | 6.49E-10 | reported | 3437 | 0.04114 | rs72640002 |  |  |  |  |  |  |  |  |  |  |  |  |  |  |  |
| -0.2572 | 10 | igd | 0.1125 | T | \|\| id:ebi-a-GCST90001497 | ebi-a-GCST90001497 | TRUE | C | 98160099 | 2.75E-10 | reported | 3437 | 0.04062 | rs72640002 |  |  |  |  |  |  |  |  |  |  |  |  |  |  |  |
| 0.2543 | 10 | igd | 0.1124 | A | \|\| id:ebi-a-GCST90001658 | ebi-a-GCST90001658 | TRUE | G | 98388573 | 4.79E-11 | reported | 3440 | 0.03854 | rs118087712 |  |  |  |  |  |  |  |  |  |  |  |  |  |  |  |
| 0.2619 | 10 | igd | 0.1124 | A | \|\| id:ebi-a-GCST90001659 | ebi-a-GCST90001659 | TRUE | G | 98388573 | 1.26E-11 | reported | 3440 | 0.03853 | rs118087712 |  |  |  |  |  |  |  |  |  |  |  |  |  |  |  |
| 0.2904 | 10 | igd | 0.1124 | A | \|\| id:ebi-a-GCST90001660 | ebi-a-GCST90001660 | TRUE | G | 98388573 | 7.54E-13 | reported | 3408 | 0.04035 | rs118087712 |  |  |  |  |  |  |  |  |  |  |  |  |  |  |  |
| 0.2723 | 10 | igd | 0.1124 | A | \|\| id:ebi-a-GCST90001670 | ebi-a-GCST90001670 | TRUE | G | 98388573 | 1.33E-11 | reported | 3440 | 0.04011 | rs118087712 |  |  |  |  |  |  |  |  |  |  |  |  |  |  |  |
| 0.2688 | 10 | igd | 0.1124 | A | \|\| id:ebi-a-GCST90001671 | ebi-a-GCST90001671 | TRUE | G | 98388573 | 2.54E-11 | reported | 3440 | 0.04016 | rs118087712 |  |  |  |  |  |  |  |  |  |  |  |  |  |  |  |
| 0.2875 | 10 | igd | 0.1124 | A | \|\| id:ebi-a-GCST90001672 | ebi-a-GCST90001672 | TRUE | G | 98388573 | 2.09E-12 | reported | 3408 | 0.04076 | rs118087712 |  |  |  |  |  |  |  |  |  |  |  |  |  |  |  |
| 0.2943 | 6 | igd | 0.1124 | T | \|\| id:ebi-a-GCST90001745 | ebi-a-GCST90001745 | TRUE | C | 29765925 | 1.32E-08 | reported | 3657 | 0.05167 | rs9461557 |  |  |  |  |  |  |  |  |  |  |  |  |  |  |  |
| 0.2163 | 10 | igd | 0.1122 | A | \|\| id:ebi-a-GCST90001483 | ebi-a-GCST90001483 | TRUE | G | 98388573 | 4.86E-08 | reported | 3405 | 0.03956 | rs118087712 |  |  |  |  |  |  |  |  |  |  |  |  |  |  |  |
| 0.2558 | 10 | igd | 0.1122 | A | \|\| id:ebi-a-GCST90001484 | ebi-a-GCST90001484 | TRUE | G | 98388573 | 7.77E-11 | reported | 3437 | 0.0392 | rs118087712 |  |  |  |  |  |  |  |  |  |  |  |  |  |  |  |
| 0.2288 | 10 | igd | 0.1122 | A | \|\| id:ebi-a-GCST90001485 | ebi-a-GCST90001485 | TRUE | G | 98388573 | 5.78E-09 | reported | 3437 | 0.0392 | rs118087712 |  |  |  |  |  |  |  |  |  |  |  |  |  |  |  |
| -0.3435 | 10 | igd | 0.112 | T | \|\| id:ebi-a-GCST90002030 | ebi-a-GCST90002030 | TRUE | C | 98160099 | 2.57E-15 | reported | 2920 | 0.04319 | rs72640002 |  |  |  |  |  |  |  |  |  |  |  |  |  |  |  |
| -0.3194 | 10 | igd | 0.112 | T | \|\| id:ebi-a-GCST90002032 | ebi-a-GCST90002032 | TRUE | C | 98160099 | 7.28E-13 | reported | 2920 | 0.04432 | rs72640002 |  |  |  |  |  |  |  |  |  |  |  |  |  |  |  |
| 0.3377 | 19 | igd | 0.1119 | G | \|\| id:ebi-a-GCST90001947 | ebi-a-GCST90001947 | TRUE | A | 51818606 | 1.96E-08 | reported | 1577 | 0.05983 | rs10419464 |  |  |  |  |  |  |  |  |  |  |  |  |  |  |  |
| 0.3618 | 19 | igd | 0.1119 | G | \|\| id:ebi-a-GCST90001957 | ebi-a-GCST90001957 | TRUE | A | 51818606 | 1.59E-09 | reported | 1578 | 0.0596 | rs10419464 |  |  |  |  |  |  |  |  |  |  |  |  |  |  |  |
| 0.3474 | 6 | igd | 0.1119 | G | \|\| id:ebi-a-GCST90002105 | ebi-a-GCST90002105 | TRUE | A | 33689299 | 6.52E-14 | reported | 2872 | 0.04611 | rs73743310 |  |  |  |  |  |  |  |  |  |  |  |  |  |  |  |
| 0.3479 | 19 | igd | 0.1118 | G | \|\| id:ebi-a-GCST90001956 | ebi-a-GCST90001956 | TRUE | A | 51818606 | 6.54E-09 | reported | 1579 | 0.05964 | rs10419464 |  |  |  |  |  |  |  |  |  |  |  |  |  |  |  |
| -0.2597 | 10 | igd | 0.1118 | A | \|\| id:ebi-a-GCST90001961 | ebi-a-GCST90001961 | TRUE | G | 98388573 | 2.82E-09 | reported | 2920 | 0.04358 | rs118087712 |  |  |  |  |  |  |  |  |  |  |  |  |  |  |  |
| 0.2769 | 6 | igd | 0.1118 | G | \|\| id:ebi-a-GCST90001973 | ebi-a-GCST90001973 | TRUE | A | 31009094 | 1.73E-09 | reported | 3113 | 0.04585 | rs13191523 |  |  |  |  |  |  |  |  |  |  |  |  |  |  |  |
| 0.2943 | 6 | igd | 0.1118 | G | \|\| id:ebi-a-GCST90002081 | ebi-a-GCST90002081 | TRUE | A | 31009094 | 8.66E-10 | reported | 3113 | 0.04784 | rs13191523 |  |  |  |  |  |  |  |  |  |  |  |  |  |  |  |
| 0.2835 | 10 | igd | 0.1117 | T | \|\| id:ebi-a-GCST90001483 | ebi-a-GCST90001483 | TRUE | C | 97070990 | 1.98E-12 | reported | 3405 | 0.04015 | rs117200502 |  |  |  |  |  |  |  |  |  |  |  |  |  |  |  |
| 0.3592 | 10 | igd | 0.1116 | T | \|\| id:ebi-a-GCST90001672 | ebi-a-GCST90001672 | TRUE | C | 97070990 | 5.51E-18 | reported | 3408 | 0.04133 | rs117200502 |  |  |  |  |  |  |  |  |  |  |  |  |  |  |  |
| -0.3505 | 10 | igd | 0.1115 | G | \|\| id:ebi-a-GCST90001672 | ebi-a-GCST90001672 | TRUE | A | 97908387 | 6.22E-18 | reported | 3408 | 0.04039 | rs75588805 |  |  |  |  |  |  |  |  |  |  |  |  |  |  |  |
| 0.3215 | 10 | igd | 0.1114 | T | \|\| id:ebi-a-GCST90001484 | ebi-a-GCST90001484 | TRUE | C | 97070990 | 8.40E-16 | reported | 3437 | 0.03975 | rs117200502 |  |  |  |  |  |  |  |  |  |  |  |  |  |  |  |
| 0.3016 | 10 | igd | 0.1114 | T | \|\| id:ebi-a-GCST90001485 | ebi-a-GCST90001485 | TRUE | C | 97070990 | 4.11E-14 | reported | 3437 | 0.03974 | rs117200502 |  |  |  |  |  |  |  |  |  |  |  |  |  |  |  |
| 0.293 | 10 | igd | 0.1114 | T | \|\| id:ebi-a-GCST90001491 | ebi-a-GCST90001491 | TRUE | C | 97070990 | 6.42E-13 | reported | 3437 | 0.04059 | rs117200502 |  |  |  |  |  |  |  |  |  |  |  |  |  |  |  |
| 0.4259 | 10 | igd | 0.1114 | T | \|\| id:ebi-a-GCST90001496 | ebi-a-GCST90001496 | TRUE | C | 97070990 | 3.91E-25 | reported | 3437 | 0.0408 | rs117200502 |  |  |  |  |  |  |  |  |  |  |  |  |  |  |  |
| 0.4056 | 10 | igd | 0.1114 | T | \|\| id:ebi-a-GCST90001497 | ebi-a-GCST90001497 | TRUE | C | 97070990 | 1.80E-23 | reported | 3437 | 0.04033 | rs117200502 |  |  |  |  |  |  |  |  |  |  |  |  |  |  |  |
| 0.3358 | 10 | igd | 0.1113 | T | \|\| id:ebi-a-GCST90001659 | ebi-a-GCST90001659 | TRUE | C | 97070990 | 1.13E-17 | reported | 3440 | 0.03902 | rs117200502 |  |  |  |  |  |  |  |  |  |  |  |  |  |  |  |
| 0.3458 | 10 | igd | 0.1113 | T | \|\| id:ebi-a-GCST90001670 | ebi-a-GCST90001670 | TRUE | C | 97070990 | 2.51E-17 | reported | 3440 | 0.04063 | rs117200502 |  |  |  |  |  |  |  |  |  |  |  |  |  |  |  |
| 0.3582 | 10 | igd | 0.1113 | T | \|\| id:ebi-a-GCST90001671 | ebi-a-GCST90001671 | TRUE | C | 97070990 | 1.88E-18 | reported | 3440 | 0.04065 | rs117200502 |  |  |  |  |  |  |  |  |  |  |  |  |  |  |  |
| 0.2406 | 6 | igd | 0.1113 | T | \|\| id:ebi-a-GCST90001769 | ebi-a-GCST90001769 | TRUE | C | 107092498 | 7.50E-10 | reported | 3648 | 0.03899 | rs113577045 |  |  |  |  |  |  |  |  |  |  |  |  |  |  |  |
| -0.416 | 10 | igd | 0.1111 | G | \|\| id:ebi-a-GCST90001491 | ebi-a-GCST90001491 | TRUE | A | 97908387 | 9.77E-26 | reported | 3437 | 0.03935 | rs75588805 |  |  |  |  |  |  |  |  |  |  |  |  |  |  |  |
| 0.2207 | 6 | igd | 0.1111 | T | \|\| id:ebi-a-GCST90001766 | ebi-a-GCST90001766 | TRUE | C | 107092498 | 1.65E-08 | reported | 3638 | 0.03901 | rs113577045 |  |  |  |  |  |  |  |  |  |  |  |  |  |  |  |
| -0.3374 | 10 | igd | 0.111 | G | \|\| id:ebi-a-GCST90001670 | ebi-a-GCST90001670 | TRUE | A | 97908387 | 3.36E-17 | reported | 3440 | 0.03979 | rs75588805 |  |  |  |  |  |  |  |  |  |  |  |  |  |  |  |
| -0.3364 | 10 | igd | 0.111 | G | \|\| id:ebi-a-GCST90001671 | ebi-a-GCST90001671 | TRUE | A | 97908387 | 4.28E-17 | reported | 3440 | 0.03981 | rs75588805 |  |  |  |  |  |  |  |  |  |  |  |  |  |  |  |
| 0.2181 | 1 | igd | 0.1109 | G | \|\| id:ebi-a-GCST90001989 | ebi-a-GCST90001989 | TRUE | A | 161246613 | 3.51E-08 | reported | 3621 | 0.03947 | rs113063605 |  |  |  |  |  |  |  |  |  |  |  |  |  |  |  |
| 0.3443 | 10 | igd | 0.1108 | T | \|\| id:ebi-a-GCST90002030 | ebi-a-GCST90002030 | TRUE | C | 97070990 | 2.13E-15 | reported | 2920 | 0.04316 | rs117200502 |  |  |  |  |  |  |  |  |  |  |  |  |  |  |  |
| 0.2911 | 10 | igd | 0.1108 | T | \|\| id:ebi-a-GCST90002031 | ebi-a-GCST90002031 | TRUE | C | 97070990 | 6.47E-11 | reported | 2920 | 0.0444 | rs117200502 |  |  |  |  |  |  |  |  |  |  |  |  |  |  |  |
| 0.2178 | 1 | igd | 0.1106 | G | \|\| id:ebi-a-GCST90001985 | ebi-a-GCST90001985 | TRUE | A | 161246613 | 3.83E-08 | reported | 3629 | 0.03952 | rs113063605 |  |  |  |  |  |  |  |  |  |  |  |  |  |  |  |
| 0.4083 | 6 | igd | 0.1105 | T | \|\| id:ebi-a-GCST90001767 | ebi-a-GCST90001767 | TRUE | C | 107090222 | 7.46E-14 | reported | 1832 | 0.05417 | rs112184735 |  |  |  |  |  |  |  |  |  |  |  |  |  |  |  |
| 0.3593 | 19 | igd | 0.1104 | T | \|\| id:ebi-a-GCST90001955 | ebi-a-GCST90001955 | TRUE | G | 51858800 | 7.73E-10 | reported | 1554 | 0.05805 | rs139724399 |  |  |  |  |  |  |  |  |  |  |  |  |  |  |  |
| -0.2887 | 2 | igd | 0.1103 | A | \|\| id:ebi-a-GCST90002120 | ebi-a-GCST90002120 | TRUE | C | 87003846 | 6.65E-11 | reported | 2920 | 0.04406 | rs11547281 |  |  |  |  |  |  |  |  |  |  |  |  |  |  |  |
| -0.2507 | 5 | igd | 0.1101 | G | \|\| id:ebi-a-GCST90001923 | ebi-a-GCST90001923 | TRUE | C | 35886731 | 5.30E-09 | reported | 2920 | 0.04281 | rs16899999 |  |  |  |  |  |  |  |  |  |  |  |  |  |  |  |
| -0.2835 | 5 | igd | 0.1101 | G | \|\| id:ebi-a-GCST90001924 | ebi-a-GCST90001924 | TRUE | C | 35886731 | 4.67E-11 | reported | 2920 | 0.04291 | rs16899999 |  |  |  |  |  |  |  |  |  |  |  |  |  |  |  |
| 0.347 | 19 | igd | 0.1101 | T | \|\| id:ebi-a-GCST90001953 | ebi-a-GCST90001953 | TRUE | G | 51858800 | 2.72E-09 | reported | 1558 | 0.058 | rs139724399 |  |  |  |  |  |  |  |  |  |  |  |  |  |  |  |
| -0.3349 | 6 | igd | 0.1101 | T | \|\| id:ebi-a-GCST90002119 | ebi-a-GCST90002119 | TRUE | C | 29765925 | 1.84E-09 | reported | 2920 | 0.05553 | rs9461557 |  |  |  |  |  |  |  |  |  |  |  |  |  |  |  |
| 0.3182 | 3 | igd | 0.11 | T | \|\| id:ebi-a-GCST90001800 | ebi-a-GCST90001800 | TRUE | G | 32633585 | 1.77E-15 | reported | 3656 | 0.03982 | rs62252808 |  |  |  |  |  |  |  |  |  |  |  |  |  |  |  |
| 0.334 | 3 | igd | 0.11 | T | \|\| id:ebi-a-GCST90001806 | ebi-a-GCST90001806 | TRUE | G | 32633585 | 4.69E-17 | reported | 3656 | 0.0396 | rs62252808 |  |  |  |  |  |  |  |  |  |  |  |  |  |  |  |
| 0.3487 | 19 | igd | 0.11 | T | \|\| id:ebi-a-GCST90001954 | ebi-a-GCST90001954 | TRUE | G | 51858800 | 2.41E-09 | reported | 1555 | 0.05809 | rs139724399 |  |  |  |  |  |  |  |  |  |  |  |  |  |  |  |
| 0.3438 | 3 | igd | 0.1099 | T | \|\| id:ebi-a-GCST90001798 | ebi-a-GCST90001798 | TRUE | G | 32633585 | 2.31E-18 | reported | 3657 | 0.03912 | rs62252808 |  |  |  |  |  |  |  |  |  |  |  |  |  |  |  |
| 0.2542 | 3 | igd | 0.1099 | T | \|\| id:ebi-a-GCST90001802 | ebi-a-GCST90001802 | TRUE | G | 32633585 | 1.84E-10 | reported | 3657 | 0.03977 | rs62252808 |  |  |  |  |  |  |  |  |  |  |  |  |  |  |  |
| 0.2876 | 3 | igd | 0.1099 | T | \|\| id:ebi-a-GCST90001804 | ebi-a-GCST90001804 | TRUE | G | 32633585 | 2.14E-13 | reported | 3657 | 0.03904 | rs62252808 |  |  |  |  |  |  |  |  |  |  |  |  |  |  |  |
| 0.2935 | 3 | igd | 0.1099 | T | \|\| id:ebi-a-GCST90001805 | ebi-a-GCST90001805 | TRUE | G | 32633585 | 1.65E-13 | reported | 3657 | 0.03965 | rs62252808 |  |  |  |  |  |  |  |  |  |  |  |  |  |  |  |
| 0.2973 | 3 | igd | 0.1099 | T | \|\| id:ebi-a-GCST90001808 | ebi-a-GCST90001808 | TRUE | G | 32633585 | 6.93E-14 | reported | 3657 | 0.03955 | rs62252808 |  |  |  |  |  |  |  |  |  |  |  |  |  |  |  |
| 0.3505 | 3 | igd | 0.1098 | A | \|\| id:ebi-a-GCST90001800 | ebi-a-GCST90001800 | TRUE | T | 32792072 | 4.39E-18 | reported | 3656 | 0.04022 | rs62251884 |  |  |  |  |  |  |  |  |  |  |  |  |  |  |  |
| 0.2703 | 3 | igd | 0.1098 | A | \|\| id:ebi-a-GCST90001802 | ebi-a-GCST90001802 | TRUE | T | 32792072 | 2.06E-11 | reported | 3657 | 0.04021 | rs62251884 |  |  |  |  |  |  |  |  |  |  |  |  |  |  |  |
| 0.3023 | 3 | igd | 0.1098 | A | \|\| id:ebi-a-GCST90001805 | ebi-a-GCST90001805 | TRUE | T | 32792072 | 6.46E-14 | reported | 3657 | 0.04015 | rs62251884 |  |  |  |  |  |  |  |  |  |  |  |  |  |  |  |
| 0.3746 | 3 | igd | 0.1098 | A | \|\| id:ebi-a-GCST90001806 | ebi-a-GCST90001806 | TRUE | T | 32792072 | 1.14E-20 | reported | 3656 | 0.03994 | rs62251884 |  |  |  |  |  |  |  |  |  |  |  |  |  |  |  |
| -0.3252 | 2 | igd | 0.1098 | G | \|\| id:ebi-a-GCST90002121 | ebi-a-GCST90002121 | TRUE | T | 87000387 | 1.14E-13 | reported | 2915 | 0.0436 | rs67856656 |  |  |  |  |  |  |  |  |  |  |  |  |  |  |  |
| 0.2622 | 10 | igd | 0.1095 | A | \|\| id:ebi-a-GCST90001484 | ebi-a-GCST90001484 | TRUE | C | 97091926 | 7.35E-11 | reported | 3437 | 0.04013 | rs79729192 |  |  |  |  |  |  |  |  |  |  |  |  |  |  |  |
| 0.2611 | 10 | igd | 0.1095 | A | \|\| id:ebi-a-GCST90001485 | ebi-a-GCST90001485 | TRUE | C | 97091926 | 8.31E-11 | reported | 3437 | 0.04007 | rs79729192 |  |  |  |  |  |  |  |  |  |  |  |  |  |  |  |
| -0.2571 | 10 | igd | 0.1094 | G | \|\| id:ebi-a-GCST90002029 | ebi-a-GCST90002029 | TRUE | A | 97908387 | 3.91E-09 | reported | 2915 | 0.04353 | rs75588805 |  |  |  |  |  |  |  |  |  |  |  |  |  |  |  |
| 0.2087 | 2 | igd | 0.1093 | A | \|\| id:ebi-a-GCST90001664 | ebi-a-GCST90001664 | TRUE | C | 87003846 | 3.48E-08 | reported | 3440 | 0.03776 | rs11547281 |  |  |  |  |  |  |  |  |  |  |  |  |  |  |  |
| 0.2266 | 22 | igd | 0.1092 | A | \|\| id:ebi-a-GCST90001703 | ebi-a-GCST90001703 | TRUE | G | 41415939 | 2.24E-08 | reported | 3657 | 0.04043 | rs8136385 |  |  |  |  |  |  |  |  |  |  |  |  |  |  |  |
| 0.2321 | 22 | igd | 0.1091 | T | \|\| id:ebi-a-GCST90001702 | ebi-a-GCST90001702 | TRUE | C | 41429823 | 1.09E-08 | reported | 3657 | 0.04051 | rs34272897 |  |  |  |  |  |  |  |  |  |  |  |  |  |  |  |
| 0.2361 | 10 | igd | 0.109 | A | \|\| id:ebi-a-GCST90001483 | ebi-a-GCST90001483 | TRUE | C | 97091926 | 6.37E-09 | reported | 3405 | 0.04056 | rs79729192 |  |  |  |  |  |  |  |  |  |  |  |  |  |  |  |
| -0.2495 | 1 | igd | 0.1089 | T | \|\| id:ebi-a-GCST90001860 | ebi-a-GCST90001860 | TRUE | C | 167596497 | 1.53E-08 | reported | 2919 | 0.04397 | rs1311452 |  |  |  |  |  |  |  |  |  |  |  |  |  |  |  |
| 0.3803 | 3 | igd | 0.1086 | G | \|\| id:ebi-a-GCST90001798 | ebi-a-GCST90001798 | TRUE | A | 32774472 | 2.39E-21 | reported | 3657 | 0.03984 | rs139451424 |  |  |  |  |  |  |  |  |  |  |  |  |  |  |  |
| 0.3159 | 3 | igd | 0.1086 | G | \|\| id:ebi-a-GCST90001804 | ebi-a-GCST90001804 | TRUE | A | 32774472 | 2.87E-15 | reported | 3657 | 0.03983 | rs139451424 |  |  |  |  |  |  |  |  |  |  |  |  |  |  |  |
| 0.3373 | 3 | igd | 0.1086 | G | \|\| id:ebi-a-GCST90001808 | ebi-a-GCST90001808 | TRUE | A | 32774472 | 7.74E-17 | reported | 3657 | 0.04027 | rs139451424 |  |  |  |  |  |  |  |  |  |  |  |  |  |  |  |
| 0.3965 | 19 | igd | 0.1086 | G | \|\| id:ebi-a-GCST90001946 | ebi-a-GCST90001946 | TRUE | A | 51818606 | 1.78E-11 | reported | 1634 | 0.05855 | rs10419464 |  |  |  |  |  |  |  |  |  |  |  |  |  |  |  |
| 0.39 | 19 | igd | 0.1086 | G | \|\| id:ebi-a-GCST90001949 | ebi-a-GCST90001949 | TRUE | A | 51818606 | 2.63E-11 | reported | 1634 | 0.05809 | rs10419464 |  |  |  |  |  |  |  |  |  |  |  |  |  |  |  |
| -0.2784 | 16 | igd | 0.1083 | C | \|\| id:ebi-a-GCST90002087 | ebi-a-GCST90002087 | TRUE | T | 31187688 | 2.62E-10 | reported | 2872 | 0.04389 | rs72785572 |  |  |  |  |  |  |  |  |  |  |  |  |  |  |  |
| -0.3488 | 3 | igd | 0.1082 | G | \|\| id:ebi-a-GCST90002035 | ebi-a-GCST90002035 | TRUE | T | 52261468 | 6.96E-15 | reported | 2871 | 0.04456 | rs352144 |  |  |  |  |  |  |  |  |  |  |  |  |  |  |  |
| -0.3851 | 3 | igd | 0.1082 | G | \|\| id:ebi-a-GCST90002036 | ebi-a-GCST90002036 | TRUE | T | 52261468 | 9.16E-18 | reported | 2871 | 0.04457 | rs352144 |  |  |  |  |  |  |  |  |  |  |  |  |  |  |  |
| 0.2629 | 6 | igd | 0.1081 | C | \|\| id:ebi-a-GCST90001758 | ebi-a-GCST90001758 | TRUE | G | 31209854 | 2.98E-08 | reported | 3657 | 0.04733 | rs28572463 |  |  |  |  |  |  |  |  |  |  |  |  |  |  |  |
| 0.299 | 17 | igd | 0.1081 | A | \|\| id:ebi-a-GCST90001907 | ebi-a-GCST90001907 | TRUE | G | 38722200 | 1.68E-11 | reported | 2910 | 0.04424 | rs62066594 |  |  |  |  |  |  |  |  |  |  |  |  |  |  |  |
| 0.2755 | 11 | igd | 0.108 | G | \|\| id:ebi-a-GCST90001762 | ebi-a-GCST90001762 | TRUE | T | 60288804 | 7.12E-12 | reported | 3658 | 0.04005 | rs149695230 |  |  |  |  |  |  |  |  |  |  |  |  |  |  |  |
| 0.2622 | 11 | igd | 0.1079 | G | \|\| id:ebi-a-GCST90001747 | ebi-a-GCST90001747 | TRUE | T | 60288804 | 6.71E-11 | reported | 3657 | 0.04006 | rs149695230 |  |  |  |  |  |  |  |  |  |  |  |  |  |  |  |
| 0.3022 | 11 | igd | 0.1079 | G | \|\| id:ebi-a-GCST90001752 | ebi-a-GCST90001752 | TRUE | T | 60288804 | 5.08E-14 | reported | 3657 | 0.03998 | rs149695230 |  |  |  |  |  |  |  |  |  |  |  |  |  |  |  |
| 0.2628 | 11 | igd | 0.1079 | G | \|\| id:ebi-a-GCST90001759 | ebi-a-GCST90001759 | TRUE | T | 60288804 | 6.13E-11 | reported | 3657 | 0.04005 | rs149695230 |  |  |  |  |  |  |  |  |  |  |  |  |  |  |  |
| -0.2906 | 16 | igd | 0.1079 | C | \|\| id:ebi-a-GCST90002089 | ebi-a-GCST90002089 | TRUE | T | 31187688 | 1.77E-10 | reported | 2850 | 0.04539 | rs72785572 |  |  |  |  |  |  |  |  |  |  |  |  |  |  |  |
| 0.2726 | 6 | igd | 0.1078 | C | \|\| id:ebi-a-GCST90001973 | ebi-a-GCST90001973 | TRUE | T | 31148873 | 8.65E-09 | reported | 3113 | 0.04724 | rs148031710 |  |  |  |  |  |  |  |  |  |  |  |  |  |  |  |
| 0.2272 | 10 | igd | 0.1078 | T | \|\| id:ebi-a-GCST90001976 | ebi-a-GCST90001976 | TRUE | C | 126551228 | 3.87E-08 | reported | 3113 | 0.04123 | rs60499622 |  |  |  |  |  |  |  |  |  |  |  |  |  |  |  |
| 0.2974 | 6 | igd | 0.1078 | C | \|\| id:ebi-a-GCST90002081 | ebi-a-GCST90002081 | TRUE | T | 31148873 | 1.91E-09 | reported | 3113 | 0.04937 | rs148031710 |  |  |  |  |  |  |  |  |  |  |  |  |  |  |  |
| 0.4838 | 10 | igd | 0.1077 | C | \|\| id:ebi-a-GCST90001775 | ebi-a-GCST90001775 | TRUE | T | 6106621 | 1.68E-32 | reported | 3660 | 0.04036 | rs41294937 |  |  |  |  |  |  |  |  |  |  |  |  |  |  |  |
| 0.476 | 10 | igd | 0.1076 | C | \|\| id:ebi-a-GCST90001794 | ebi-a-GCST90001794 | TRUE | T | 6106621 | 1.74E-31 | reported | 3658 | 0.04039 | rs41294937 |  |  |  |  |  |  |  |  |  |  |  |  |  |  |  |
| 0.5514 | 10 | igd | 0.1075 | C | \|\| id:ebi-a-GCST90001777 | ebi-a-GCST90001777 | TRUE | T | 6106621 | 1.25E-43 | reported | 3657 | 0.03929 | rs41294937 |  |  |  |  |  |  |  |  |  |  |  |  |  |  |  |
| 0.5847 | 10 | igd | 0.1075 | A | \|\| id:ebi-a-GCST90001779 | ebi-a-GCST90001779 | TRUE | C | 6115678 | 1.32E-47 | reported | 3657 | 0.03976 | rs62626323 |  |  |  |  |  |  |  |  |  |  |  |  |  |  |  |
| 0.5433 | 10 | igd | 0.1075 | A | \|\| id:ebi-a-GCST90001784 | ebi-a-GCST90001784 | TRUE | C | 6115678 | 1.25E-40 | reported | 3657 | 0.04021 | rs62626323 |  |  |  |  |  |  |  |  |  |  |  |  |  |  |  |
| 0.5734 | 10 | igd | 0.1075 | C | \|\| id:ebi-a-GCST90001789 | ebi-a-GCST90001789 | TRUE | T | 6106621 | 9.24E-48 | reported | 3657 | 0.03893 | rs41294937 |  |  |  |  |  |  |  |  |  |  |  |  |  |  |  |
| 0.5481 | 10 | igd | 0.1075 | C | \|\| id:ebi-a-GCST90001790 | ebi-a-GCST90001790 | TRUE | T | 6106621 | 4.74E-43 | reported | 3657 | 0.03933 | rs41294937 |  |  |  |  |  |  |  |  |  |  |  |  |  |  |  |
| 0.5626 | 10 | igd | 0.1075 | C | \|\| id:ebi-a-GCST90001791 | ebi-a-GCST90001791 | TRUE | T | 6106621 | 2.21E-43 | reported | 3657 | 0.04021 | rs41294937 |  |  |  |  |  |  |  |  |  |  |  |  |  |  |  |
| 0.5555 | 10 | igd | 0.1075 | C | \|\| id:ebi-a-GCST90001793 | ebi-a-GCST90001793 | TRUE | T | 6106621 | 1.57E-43 | reported | 3657 | 0.03963 | rs41294937 |  |  |  |  |  |  |  |  |  |  |  |  |  |  |  |
| 0.2969 | 10 | igd | 0.1075 | A | \|\| id:ebi-a-GCST90001795 | ebi-a-GCST90001795 | TRUE | C | 6115678 | 1.78E-13 | reported | 3657 | 0.04016 | rs62626323 |  |  |  |  |  |  |  |  |  |  |  |  |  |  |  |
| 0.349 | 10 | igd | 0.1074 | A | \|\| id:ebi-a-GCST90001783 | ebi-a-GCST90001783 | TRUE | C | 6115678 | 3.54E-18 | reported | 3656 | 0.03994 | rs62626323 |  |  |  |  |  |  |  |  |  |  |  |  |  |  |  |
| 0.3718 | 10 | igd | 0.1073 | C | \|\| id:ebi-a-GCST90001785 | ebi-a-GCST90001785 | TRUE | T | 6106621 | 5.23E-20 | reported | 3649 | 0.04036 | rs41294937 |  |  |  |  |  |  |  |  |  |  |  |  |  |  |  |
| -0.2818 | 12 | igd | 0.1072 | G | \|\| id:ebi-a-GCST90002074 | ebi-a-GCST90002074 | TRUE | A | 70112707 | 5.65E-11 | reported | 3112 | 0.04285 | rs775451 |  |  |  |  |  |  |  |  |  |  |  |  |  |  |  |
| 0.2674 | 6 | igd | 0.1071 | C | \|\| id:ebi-a-GCST90001687 | ebi-a-GCST90001687 | TRUE | T | 32343369 | 7.25E-09 | reported | 3408 | 0.0461 | rs3117106 |  |  |  |  |  |  |  |  |  |  |  |  |  |  |  |
| -0.2453 | 10 | igd | 0.107 | T | \|\| id:ebi-a-GCST90001489 | ebi-a-GCST90001489 | TRUE | A | 98008283 | 3.12E-09 | reported | 3405 | 0.04129 | rs72811038 |  |  |  |  |  |  |  |  |  |  |  |  |  |  |  |
| -0.2777 | 10 | igd | 0.107 | T | \|\| id:ebi-a-GCST90001495 | ebi-a-GCST90001495 | TRUE | A | 98008283 | 2.72E-11 | reported | 3405 | 0.04155 | rs72811038 |  |  |  |  |  |  |  |  |  |  |  |  |  |  |  |
| -0.3128 | 10 | igd | 0.107 | T | \|\| id:ebi-a-GCST90001660 | ebi-a-GCST90001660 | TRUE | A | 98008283 | 2.93E-14 | reported | 3408 | 0.04097 | rs72811038 |  |  |  |  |  |  |  |  |  |  |  |  |  |  |  |
| -0.3571 | 10 | igd | 0.107 | T | \|\| id:ebi-a-GCST90001672 | ebi-a-GCST90001672 | TRUE | A | 98008283 | 7.65E-18 | reported | 3408 | 0.04128 | rs72811038 |  |  |  |  |  |  |  |  |  |  |  |  |  |  |  |
| 0.4151 | 6 | igd | 0.107 | A | \|\| id:ebi-a-GCST90002112 | ebi-a-GCST90002112 | TRUE | G | 33053609 | 2.23E-11 | reported | 1635 | 0.06162 | rs11551421 |  |  |  |  |  |  |  |  |  |  |  |  |  |  |  |
| 0.2537 | 6 | igd | 0.1069 | A | \|\| id:ebi-a-GCST90001758 | ebi-a-GCST90001758 | TRUE | G | 31809504 | 3.93E-08 | reported | 3657 | 0.04609 | rs112405902 |  |  |  |  |  |  |  |  |  |  |  |  |  |  |  |
| 0.2604 | 6 | igd | 0.1069 | A | \|\| id:ebi-a-GCST90001760 | ebi-a-GCST90001760 | TRUE | G | 31809504 | 1.75E-08 | reported | 3657 | 0.0461 | rs112405902 |  |  |  |  |  |  |  |  |  |  |  |  |  |  |  |
| 0.4768 | 10 | igd | 0.1069 | A | \|\| id:ebi-a-GCST90001778 | ebi-a-GCST90001778 | TRUE | G | 6120173 | 2.17E-32 | reported | 3657 | 0.03985 | rs41295093 |  |  |  |  |  |  |  |  |  |  |  |  |  |  |  |
| 0.4428 | 10 | igd | 0.1069 | A | \|\| id:ebi-a-GCST90001787 | ebi-a-GCST90001787 | TRUE | G | 6120173 | 4.61E-28 | reported | 3657 | 0.03998 | rs41295093 |  |  |  |  |  |  |  |  |  |  |  |  |  |  |  |
| 0.5105 | 10 | igd | 0.1069 | A | \|\| id:ebi-a-GCST90001792 | ebi-a-GCST90001792 | TRUE | G | 6120173 | 2.59E-37 | reported | 3657 | 0.03955 | rs41295093 |  |  |  |  |  |  |  |  |  |  |  |  |  |  |  |
| 0.4244 | 10 | igd | 0.1068 | A | \|\| id:ebi-a-GCST90001780 | ebi-a-GCST90001780 | TRUE | G | 6120173 | 1.11E-25 | reported | 3642 | 0.0402 | rs41295093 |  |  |  |  |  |  |  |  |  |  |  |  |  |  |  |
| 0.4909 | 10 | igd | 0.1068 | C | \|\| id:ebi-a-GCST90001781 | ebi-a-GCST90001781 | TRUE | T | 6106621 | 1.67E-18 | reported | 1835 | 0.05533 | rs41294937 |  |  |  |  |  |  |  |  |  |  |  |  |  |  |  |
| 0.3186 | 10 | igd | 0.1067 | A | \|\| id:ebi-a-GCST90001786 | ebi-a-GCST90001786 | TRUE | G | 6120173 | 3.50E-15 | reported | 3651 | 0.0403 | rs41295093 |  |  |  |  |  |  |  |  |  |  |  |  |  |  |  |
| -0.2335 | 10 | igd | 0.1066 | T | \|\| id:ebi-a-GCST90001484 | ebi-a-GCST90001484 | TRUE | A | 98008283 | 5.53E-09 | reported | 3437 | 0.03995 | rs72811038 |  |  |  |  |  |  |  |  |  |  |  |  |  |  |  |
| -0.2511 | 10 | igd | 0.1066 | T | \|\| id:ebi-a-GCST90001491 | ebi-a-GCST90001491 | TRUE | A | 98008283 | 7.03E-10 | reported | 3437 | 0.04062 | rs72811038 |  |  |  |  |  |  |  |  |  |  |  |  |  |  |  |
| -0.268 | 10 | igd | 0.1066 | T | \|\| id:ebi-a-GCST90001496 | ebi-a-GCST90001496 | TRUE | A | 98008283 | 8.31E-11 | reported | 3437 | 0.04114 | rs72811038 |  |  |  |  |  |  |  |  |  |  |  |  |  |  |  |
| -0.2743 | 10 | igd | 0.1066 | T | \|\| id:ebi-a-GCST90001497 | ebi-a-GCST90001497 | TRUE | A | 98008283 | 1.67E-11 | reported | 3437 | 0.0406 | rs72811038 |  |  |  |  |  |  |  |  |  |  |  |  |  |  |  |
| 0.4446 | 1 | igd | 0.1066 | A | \|\| id:ebi-a-GCST90001526 | ebi-a-GCST90001526 | TRUE | C | 161520472 | 2.77E-15 | reported | 1858 | 0.05579 | rs139755165 |  |  |  |  |  |  |  |  |  |  |  |  |  |  |  |
| -0.4395 | 1 | igd | 0.1066 | A | \|\| id:ebi-a-GCST90001528 | ebi-a-GCST90001528 | TRUE | C | 161520472 | 5.97E-15 | reported | 1858 | 0.05585 | rs139755165 |  |  |  |  |  |  |  |  |  |  |  |  |  |  |  |
| 0.2323 | 20 | igd | 0.1066 | A | \|\| id:ebi-a-GCST90002012 | ebi-a-GCST90002012 | TRUE | G | 48892942 | 7.69E-09 | reported | 3582 | 0.04013 | rs41314914 |  |  |  |  |  |  |  |  |  |  |  |  |  |  |  |
| -0.2753 | 10 | igd | 0.1065 | T | \|\| id:ebi-a-GCST90001658 | ebi-a-GCST90001658 | TRUE | A | 98008283 | 2.55E-12 | reported | 3440 | 0.03919 | rs72811038 |  |  |  |  |  |  |  |  |  |  |  |  |  |  |  |
| -0.2873 | 10 | igd | 0.1065 | T | \|\| id:ebi-a-GCST90001659 | ebi-a-GCST90001659 | TRUE | A | 98008283 | 2.74E-13 | reported | 3440 | 0.03917 | rs72811038 |  |  |  |  |  |  |  |  |  |  |  |  |  |  |  |
| -0.3325 | 10 | igd | 0.1065 | T | \|\| id:ebi-a-GCST90001670 | ebi-a-GCST90001670 | TRUE | A | 98008283 | 4.38E-16 | reported | 3440 | 0.04071 | rs72811038 |  |  |  |  |  |  |  |  |  |  |  |  |  |  |  |
| -0.3089 | 10 | igd | 0.1065 | T | \|\| id:ebi-a-GCST90001671 | ebi-a-GCST90001671 | TRUE | A | 98008283 | 4.74E-14 | reported | 3440 | 0.0408 | rs72811038 |  |  |  |  |  |  |  |  |  |  |  |  |  |  |  |
| 0.5217 | 10 | igd | 0.1062 | A | \|\| id:ebi-a-GCST90001782 | ebi-a-GCST90001782 | TRUE | G | 6120173 | 7.18E-22 | reported | 1837 | 0.05359 | rs41295093 |  |  |  |  |  |  |  |  |  |  |  |  |  |  |  |
| 0.2896 | 10 | igd | 0.1062 | A | \|\| id:ebi-a-GCST90001892 | ebi-a-GCST90001892 | TRUE | T | 97960335 | 3.26E-11 | reported | 2920 | 0.04348 | rs74151285 |  |  |  |  |  |  |  |  |  |  |  |  |  |  |  |
| 0.4593 | 19 | igd | 0.1062 | G | \|\| id:ebi-a-GCST90001955 | ebi-a-GCST90001955 | TRUE | A | 51677700 | 3.80E-14 | reported | 1554 | 0.06013 | rs17728644 |  |  |  |  |  |  |  |  |  |  |  |  |  |  |  |
| 0.2448 | 10 | igd | 0.1062 | T | \|\| id:ebi-a-GCST90001961 | ebi-a-GCST90001961 | TRUE | A | 98008283 | 4.05E-08 | reported | 2920 | 0.04448 | rs72811038 |  |  |  |  |  |  |  |  |  |  |  |  |  |  |  |
| 0.2587 | 20 | igd | 0.1062 | G | \|\| id:ebi-a-GCST90001989 | ebi-a-GCST90001989 | TRUE | A | 44184498 | 1.41E-10 | reported | 3621 | 0.0402 | rs2272955 |  |  |  |  |  |  |  |  |  |  |  |  |  |  |  |
| -0.3561 | 10 | igd | 0.1062 | T | \|\| id:ebi-a-GCST90002030 | ebi-a-GCST90002030 | TRUE | A | 98008283 | 3.79E-16 | reported | 2920 | 0.04346 | rs72811038 |  |  |  |  |  |  |  |  |  |  |  |  |  |  |  |
| 0.6116 | 10 | igd | 0.1062 | A | \|\| id:ebi-a-GCST90002030 | ebi-a-GCST90002030 | TRUE | T | 97960335 | 1.94E-45 | reported | 2920 | 0.04249 | rs74151285 |  |  |  |  |  |  |  |  |  |  |  |  |  |  |  |
| 0.4951 | 10 | igd | 0.1062 | A | \|\| id:ebi-a-GCST90002031 | ebi-a-GCST90002031 | TRUE | T | 97960335 | 5.65E-29 | reported | 2920 | 0.04385 | rs74151285 |  |  |  |  |  |  |  |  |  |  |  |  |  |  |  |
| -0.3185 | 10 | igd | 0.1062 | T | \|\| id:ebi-a-GCST90002031 | ebi-a-GCST90002031 | TRUE | A | 98008283 | 1.36E-12 | reported | 2920 | 0.04474 | rs72811038 |  |  |  |  |  |  |  |  |  |  |  |  |  |  |  |
| -0.3076 | 10 | igd | 0.1062 | T | \|\| id:ebi-a-GCST90002032 | ebi-a-GCST90002032 | TRUE | A | 98008283 | 7.38E-12 | reported | 2920 | 0.04472 | rs72811038 |  |  |  |  |  |  |  |  |  |  |  |  |  |  |  |
| 0.534 | 10 | igd | 0.1062 | T | \|\| id:ebi-a-GCST90002032 | ebi-a-GCST90002032 | TRUE | G | 97960955 | 1.51E-33 | reported | 2920 | 0.04369 | rs7075831 |  |  |  |  |  |  |  |  |  |  |  |  |  |  |  |
| 0.2793 | 1 | igd | 0.1061 | A | \|\| id:ebi-a-GCST90001845 | ebi-a-GCST90001845 | TRUE | G | 167136405 | 2.66E-10 | reported | 2911 | 0.04405 | rs61814886 |  |  |  |  |  |  |  |  |  |  |  |  |  |  |  |
| 0.4829 | 19 | igd | 0.1061 | G | \|\| id:ebi-a-GCST90001954 | ebi-a-GCST90001954 | TRUE | A | 51677700 | 1.52E-15 | reported | 1555 | 0.05992 | rs17728644 |  |  |  |  |  |  |  |  |  |  |  |  |  |  |  |
| 0.3211 | 1 | igd | 0.1061 | A | \|\| id:ebi-a-GCST90002097 | ebi-a-GCST90002097 | TRUE | G | 161506889 | 3.83E-08 | reported | 1578 | 0.05811 | rs79277331 |  |  |  |  |  |  |  |  |  |  |  |  |  |  |  |
| 0.4626 | 3 | igd | 0.1059 | T | \|\| id:ebi-a-GCST90001801 | ebi-a-GCST90001801 | TRUE | G | 32633585 | 4.41E-17 | reported | 1836 | 0.05452 | rs62252808 |  |  |  |  |  |  |  |  |  |  |  |  |  |  |  |
| 0.2885 | 1 | igd | 0.1059 | A | \|\| id:ebi-a-GCST90001856 | ebi-a-GCST90001856 | TRUE | G | 167136405 | 6.97E-11 | reported | 2919 | 0.04408 | rs61814886 |  |  |  |  |  |  |  |  |  |  |  |  |  |  |  |
| 0.4881 | 19 | igd | 0.1059 | G | \|\| id:ebi-a-GCST90001953 | ebi-a-GCST90001953 | TRUE | A | 51677700 | 7.36E-16 | reported | 1558 | 0.05988 | rs17728644 |  |  |  |  |  |  |  |  |  |  |  |  |  |  |  |
| -0.2703 | 1 | igd | 0.1058 | C | \|\| id:ebi-a-GCST90001845 | ebi-a-GCST90001845 | TRUE | G | 167437263 | 1.36E-09 | reported | 2911 | 0.04446 | rs7523351 |  |  |  |  |  |  |  |  |  |  |  |  |  |  |  |
| 0.4493 | 1 | igd | 0.1058 | G | \|\| id:ebi-a-GCST90002091 | ebi-a-GCST90002091 | TRUE | A | 161511433 | 6.57E-14 | reported | 1635 | 0.05942 | rs74387071 |  |  |  |  |  |  |  |  |  |  |  |  |  |  |  |
| 0.2511 | 10 | igd | 0.1057 | T | \|\| id:ebi-a-GCST90002032 | ebi-a-GCST90002032 | TRUE | G | 95543430 | 4.77E-08 | reported | 2920 | 0.04587 | rs17109405 |  |  |  |  |  |  |  |  |  |  |  |  |  |  |  |
| -0.2424 | 22 | igd | 0.1056 | A | \|\| id:ebi-a-GCST90001702 | ebi-a-GCST90001702 | TRUE | G | 43473405 | 1.35E-09 | reported | 3657 | 0.03989 | rs73179040 |  |  |  |  |  |  |  |  |  |  |  |  |  |  |  |
| -0.2395 | 22 | igd | 0.1056 | A | \|\| id:ebi-a-GCST90001703 | ebi-a-GCST90001703 | TRUE | G | 43473405 | 2.12E-09 | reported | 3657 | 0.03989 | rs73179040 |  |  |  |  |  |  |  |  |  |  |  |  |  |  |  |
| -0.2189 | 22 | igd | 0.1056 | A | \|\| id:ebi-a-GCST90001711 | ebi-a-GCST90001711 | TRUE | G | 43473405 | 3.85E-08 | reported | 3657 | 0.03973 | rs73179040 |  |  |  |  |  |  |  |  |  |  |  |  |  |  |  |
| -0.3406 | 1 | igd | 0.1055 | C | \|\| id:ebi-a-GCST90001858 | ebi-a-GCST90001858 | TRUE | G | 167437263 | 1.60E-14 | reported | 2919 | 0.04413 | rs7523351 |  |  |  |  |  |  |  |  |  |  |  |  |  |  |  |
| -0.2454 | 1 | igd | 0.1055 | C | \|\| id:ebi-a-GCST90001863 | ebi-a-GCST90001863 | TRUE | G | 167437263 | 2.22E-08 | reported | 2919 | 0.04375 | rs7523351 |  |  |  |  |  |  |  |  |  |  |  |  |  |  |  |
| -0.3178 | 1 | igd | 0.1055 | C | \|\| id:ebi-a-GCST90001864 | ebi-a-GCST90001864 | TRUE | G | 167437263 | 8.34E-13 | reported | 2919 | 0.04422 | rs7523351 |  |  |  |  |  |  |  |  |  |  |  |  |  |  |  |
| 0.4628 | 10 | igd | 0.1052 | A | \|\| id:ebi-a-GCST90001484 | ebi-a-GCST90001484 | TRUE | G | 97961789 | 8.58E-31 | reported | 3437 | 0.03972 | rs7099132 |  |  |  |  |  |  |  |  |  |  |  |  |  |  |  |
| 0.4547 | 10 | igd | 0.1052 | A | \|\| id:ebi-a-GCST90001485 | ebi-a-GCST90001485 | TRUE | G | 97961789 | 7.53E-30 | reported | 3437 | 0.03969 | rs7099132 |  |  |  |  |  |  |  |  |  |  |  |  |  |  |  |
| 0.4896 | 10 | igd | 0.1052 | A | \|\| id:ebi-a-GCST90001491 | ebi-a-GCST90001491 | TRUE | G | 97961789 | 7.24E-33 | reported | 3437 | 0.04057 | rs7099132 |  |  |  |  |  |  |  |  |  |  |  |  |  |  |  |
| 0.4752 | 10 | igd | 0.1052 | A | \|\| id:ebi-a-GCST90001496 | ebi-a-GCST90001496 | TRUE | G | 97961789 | 3.08E-30 | reported | 3437 | 0.04119 | rs7099132 |  |  |  |  |  |  |  |  |  |  |  |  |  |  |  |
| 0.4448 | 10 | igd | 0.1052 | A | \|\| id:ebi-a-GCST90001497 | ebi-a-GCST90001497 | TRUE | T | 97960335 | 2.78E-27 | reported | 3437 | 0.04076 | rs74151285 |  |  |  |  |  |  |  |  |  |  |  |  |  |  |  |
| 0.4646 | 10 | igd | 0.1051 | T | \|\| id:ebi-a-GCST90001658 | ebi-a-GCST90001658 | TRUE | G | 97960955 | 1.10E-31 | reported | 3440 | 0.03927 | rs7075831 |  |  |  |  |  |  |  |  |  |  |  |  |  |  |  |
| 0.4625 | 10 | igd | 0.1051 | T | \|\| id:ebi-a-GCST90001659 | ebi-a-GCST90001659 | TRUE | G | 97960955 | 2.14E-31 | reported | 3440 | 0.03928 | rs7075831 |  |  |  |  |  |  |  |  |  |  |  |  |  |  |  |
| 0.3991 | 10 | igd | 0.1051 | A | \|\| id:ebi-a-GCST90001670 | ebi-a-GCST90001670 | TRUE | T | 97960335 | 5.06E-22 | reported | 3440 | 0.04109 | rs74151285 |  |  |  |  |  |  |  |  |  |  |  |  |  |  |  |
| 0.4037 | 10 | igd | 0.1051 | T | \|\| id:ebi-a-GCST90001671 | ebi-a-GCST90001671 | TRUE | G | 97960955 | 2.18E-22 | reported | 3440 | 0.04118 | rs7075831 |  |  |  |  |  |  |  |  |  |  |  |  |  |  |  |
| -0.3173 | 5 | igd | 0.1051 | A | \|\| id:ebi-a-GCST90001929 | ebi-a-GCST90001929 | TRUE | T | 35840724 | 3.00E-14 | reported | 2920 | 0.04155 | rs66570838 |  |  |  |  |  |  |  |  |  |  |  |  |  |  |  |
| -0.2975 | 5 | igd | 0.105 | A | \|\| id:ebi-a-GCST90001932 | ebi-a-GCST90001932 | TRUE | T | 35872190 | 8.08E-12 | reported | 2920 | 0.04334 | rs11567754 |  |  |  |  |  |  |  |  |  |  |  |  |  |  |  |
| 0.243 | 2 | igd | 0.105 | G | \|\| id:ebi-a-GCST90001970 | ebi-a-GCST90001970 | TRUE | C | 130608376 | 4.83E-08 | reported | 2971 | 0.0444 | rs11891318 |  |  |  |  |  |  |  |  |  |  |  |  |  |  |  |
| 0.3007 | 10 | igd | 0.105 | A | \|\| id:ebi-a-GCST90002030 | ebi-a-GCST90002030 | TRUE | G | 98820067 | 3.00E-11 | reported | 2920 | 0.04507 | rs80347374 |  |  |  |  |  |  |  |  |  |  |  |  |  |  |  |
| 0.269 | 10 | igd | 0.105 | A | \|\| id:ebi-a-GCST90002031 | ebi-a-GCST90002031 | TRUE | G | 98820067 | 6.31E-09 | reported | 2920 | 0.04618 | rs80347374 |  |  |  |  |  |  |  |  |  |  |  |  |  |  |  |
| 0.3107 | 10 | igd | 0.105 | A | \|\| id:ebi-a-GCST90002032 | ebi-a-GCST90002032 | TRUE | G | 98820067 | 1.85E-11 | reported | 2920 | 0.04607 | rs80347374 |  |  |  |  |  |  |  |  |  |  |  |  |  |  |  |
| 0.2836 | 6 | igd | 0.105 | C | \|\| id:ebi-a-GCST90002105 | ebi-a-GCST90002105 | TRUE | T | 33891382 | 9.80E-10 | reported | 2872 | 0.04625 | rs13219222 |  |  |  |  |  |  |  |  |  |  |  |  |  |  |  |
| -0.4591 | 6 | igd | 0.1049 | G | \|\| id:ebi-a-GCST90001752 | ebi-a-GCST90001752 | TRUE | T | 32625802 | 4.13E-10 | reported | 3657 | 0.07326 | rs149503020 |  |  |  |  |  |  |  |  |  |  |  |  |  |  |  |
| 0.2351 | 11 | igd | 0.1048 | A | \|\| id:ebi-a-GCST90001743 | ebi-a-GCST90001743 | TRUE | G | 60312126 | 7.80E-09 | reported | 3660 | 0.04063 | rs72928780 |  |  |  |  |  |  |  |  |  |  |  |  |  |  |  |
| 0.3917 | 19 | igd | 0.1048 | G | \|\| id:ebi-a-GCST90001952 | ebi-a-GCST90001952 | TRUE | A | 51677700 | 1.61E-10 | reported | 1569 | 0.06086 | rs17728644 |  |  |  |  |  |  |  |  |  |  |  |  |  |  |  |
| 0.4273 | 10 | igd | 0.1047 | A | \|\| id:ebi-a-GCST90001483 | ebi-a-GCST90001483 | TRUE | G | 97961789 | 8.17E-26 | reported | 3405 | 0.04034 | rs7099132 |  |  |  |  |  |  |  |  |  |  |  |  |  |  |  |
| 0.4398 | 10 | igd | 0.1047 | A | \|\| id:ebi-a-GCST90001489 | ebi-a-GCST90001489 | TRUE | G | 97961789 | 6.62E-26 | reported | 3405 | 0.04144 | rs7099132 |  |  |  |  |  |  |  |  |  |  |  |  |  |  |  |
| 0.4142 | 10 | igd | 0.1047 | A | \|\| id:ebi-a-GCST90001495 | ebi-a-GCST90001495 | TRUE | T | 97960335 | 1.23E-22 | reported | 3405 | 0.042 | rs74151285 |  |  |  |  |  |  |  |  |  |  |  |  |  |  |  |
| 0.4076 | 10 | igd | 0.1046 | A | \|\| id:ebi-a-GCST90001660 | ebi-a-GCST90001660 | TRUE | T | 97960335 | 2.13E-22 | reported | 3408 | 0.04157 | rs74151285 |  |  |  |  |  |  |  |  |  |  |  |  |  |  |  |
| 0.3744 | 10 | igd | 0.1046 | A | \|\| id:ebi-a-GCST90001672 | ebi-a-GCST90001672 | TRUE | T | 97960335 | 9.23E-19 | reported | 3408 | 0.04208 | rs74151285 |  |  |  |  |  |  |  |  |  |  |  |  |  |  |  |
| 0.3958 | 19 | igd | 0.1046 | G | \|\| id:ebi-a-GCST90001947 | ebi-a-GCST90001947 | TRUE | A | 51677700 | 9.76E-11 | reported | 1577 | 0.06075 | rs17728644 |  |  |  |  |  |  |  |  |  |  |  |  |  |  |  |
| 0.4047 | 19 | igd | 0.1046 | G | \|\| id:ebi-a-GCST90001957 | ebi-a-GCST90001957 | TRUE | A | 51677700 | 3.38E-11 | reported | 1578 | 0.06062 | rs17728644 |  |  |  |  |  |  |  |  |  |  |  |  |  |  |  |
| 0.4022 | 19 | igd | 0.1045 | G | \|\| id:ebi-a-GCST90001956 | ebi-a-GCST90001956 | TRUE | A | 51677700 | 4.39E-11 | reported | 1579 | 0.0606 | rs17728644 |  |  |  |  |  |  |  |  |  |  |  |  |  |  |  |
| -0.5989 | 6 | igd | 0.1043 | G | \|\| id:ebi-a-GCST90002110 | ebi-a-GCST90002110 | TRUE | T | 32625802 | 1.51E-08 | reported | 1634 | 0.1053 | rs149503020 |  |  |  |  |  |  |  |  |  |  |  |  |  |  |  |
| -0.2781 | 6 | igd | 0.1043 | C | \|\| id:ebi-a-GCST90002118 | ebi-a-GCST90002118 | TRUE | T | 31774788 | 4.39E-08 | reported | 2920 | 0.05066 | rs113065708 |  |  |  |  |  |  |  |  |  |  |  |  |  |  |  |
| -0.3397 | 6 | igd | 0.1043 | C | \|\| id:ebi-a-GCST90002119 | ebi-a-GCST90002119 | TRUE | T | 31774788 | 1.67E-11 | reported | 2920 | 0.05025 | rs113065708 |  |  |  |  |  |  |  |  |  |  |  |  |  |  |  |
| 0.3135 | 10 | igd | 0.1041 | G | \|\| id:ebi-a-GCST90001775 | ebi-a-GCST90001775 | TRUE | A | 6073929 | 1.34E-14 | reported | 3660 | 0.04054 | rs10905644 |  |  |  |  |  |  |  |  |  |  |  |  |  |  |  |
| 0.2564 | 10 | igd | 0.1041 | G | \|\| id:ebi-a-GCST90001780 | ebi-a-GCST90001780 | TRUE | A | 6073929 | 2.11E-10 | reported | 3642 | 0.04024 | rs10905644 |  |  |  |  |  |  |  |  |  |  |  |  |  |  |  |
| 0.3017 | 10 | igd | 0.104 | G | \|\| id:ebi-a-GCST90001794 | ebi-a-GCST90001794 | TRUE | A | 6073929 | 1.26E-13 | reported | 3658 | 0.04056 | rs10905644 |  |  |  |  |  |  |  |  |  |  |  |  |  |  |  |
| -0.3764 | 22 | igd | 0.1039 | T | \|\| id:ebi-a-GCST90001704 | ebi-a-GCST90001704 | TRUE | C | 42802543 | 4.43E-19 | reported | 3657 | 0.04194 | rs11704106 |  |  |  |  |  |  |  |  |  |  |  |  |  |  |  |
| -0.4713 | 22 | igd | 0.1039 | T | \|\| id:ebi-a-GCST90001710 | ebi-a-GCST90001710 | TRUE | C | 42802543 | 3.41E-29 | reported | 3657 | 0.04166 | rs11704106 |  |  |  |  |  |  |  |  |  |  |  |  |  |  |  |
| -0.4485 | 22 | igd | 0.1039 | T | \|\| id:ebi-a-GCST90001711 | ebi-a-GCST90001711 | TRUE | C | 42802543 | 1.03E-26 | reported | 3657 | 0.04159 | rs11704106 |  |  |  |  |  |  |  |  |  |  |  |  |  |  |  |
| 0.3621 | 10 | igd | 0.1039 | G | \|\| id:ebi-a-GCST90001779 | ebi-a-GCST90001779 | TRUE | A | 6073929 | 3.62E-19 | reported | 3657 | 0.04025 | rs10905644 |  |  |  |  |  |  |  |  |  |  |  |  |  |  |  |
| 0.2253 | 10 | igd | 0.1039 | G | \|\| id:ebi-a-GCST90001783 | ebi-a-GCST90001783 | TRUE | A | 6073929 | 1.85E-08 | reported | 3656 | 0.03996 | rs10905644 |  |  |  |  |  |  |  |  |  |  |  |  |  |  |  |
| 0.3411 | 10 | igd | 0.1039 | G | \|\| id:ebi-a-GCST90001784 | ebi-a-GCST90001784 | TRUE | A | 6073929 | 5.92E-17 | reported | 3657 | 0.04057 | rs10905644 |  |  |  |  |  |  |  |  |  |  |  |  |  |  |  |
| 0.2292 | 10 | igd | 0.1039 | G | \|\| id:ebi-a-GCST90001785 | ebi-a-GCST90001785 | TRUE | A | 6073929 | 1.56E-08 | reported | 3649 | 0.04043 | rs10905644 |  |  |  |  |  |  |  |  |  |  |  |  |  |  |  |
| 0.3541 | 10 | igd | 0.1039 | G | \|\| id:ebi-a-GCST90001789 | ebi-a-GCST90001789 | TRUE | A | 6073929 | 3.72E-19 | reported | 3657 | 0.03936 | rs10905644 |  |  |  |  |  |  |  |  |  |  |  |  |  |  |  |
| 0.3493 | 10 | igd | 0.1039 | G | \|\| id:ebi-a-GCST90001791 | ebi-a-GCST90001791 | TRUE | A | 6073929 | 1.12E-17 | reported | 3657 | 0.0406 | rs10905644 |  |  |  |  |  |  |  |  |  |  |  |  |  |  |  |
| -0.5316 | 6 | igd | 0.1038 | A | \|\| id:ebi-a-GCST90002105 | ebi-a-GCST90002105 | TRUE | C | 32624017 | 2.34E-14 | reported | 2872 | 0.0693 | rs145607970 |  |  |  |  |  |  |  |  |  |  |  |  |  |  |  |
| -0.5536 | 6 | igd | 0.1034 | A | \|\| id:ebi-a-GCST90002112 | ebi-a-GCST90002112 | TRUE | C | 32624017 | 4.91E-10 | reported | 1635 | 0.08844 | rs145607970 |  |  |  |  |  |  |  |  |  |  |  |  |  |  |  |
| 0.3719 | 19 | igd | 0.10332 | T | \|\| id:prot-a-439 | prot-a-439 | TRUE | C | 51739227 | 2.45E-17 | reported | 3301 | 0.0439 | rs146995981 |  |  |  |  |  |  |  |  |  |  |  |  |  |  |  |
| 0.2377 | 10 | igd | 0.1033 | T | \|\| id:ebi-a-GCST90001497 | ebi-a-GCST90001497 | TRUE | G | 95543430 | 1.84E-08 | reported | 3437 | 0.04214 | rs17109405 |  |  |  |  |  |  |  |  |  |  |  |  |  |  |  |
| -0.7058 | 6 | igd | 0.1032 | A | \|\| id:ebi-a-GCST90002108 | ebi-a-GCST90002108 | TRUE | C | 32624017 | 3.09E-15 | reported | 1579 | 0.08858 | rs145607970 |  |  |  |  |  |  |  |  |  |  |  |  |  |  |  |
| 0.227 | 22 | igd | 0.1025 | A | \|\| id:ebi-a-GCST90001702 | ebi-a-GCST90001702 | TRUE | C | 43141391 | 3.50E-08 | reported | 3657 | 0.04108 | rs9611872 |  |  |  |  |  |  |  |  |  |  |  |  |  |  |  |
| 0.2262 | 22 | igd | 0.1025 | A | \|\| id:ebi-a-GCST90001703 | ebi-a-GCST90001703 | TRUE | C | 43141391 | 3.90E-08 | reported | 3657 | 0.04108 | rs9611872 |  |  |  |  |  |  |  |  |  |  |  |  |  |  |  |
| 0.2263 | 22 | igd | 0.1025 | A | \|\| id:ebi-a-GCST90001704 | ebi-a-GCST90001704 | TRUE | C | 43141391 | 3.69E-08 | reported | 3657 | 0.04102 | rs9611872 |  |  |  |  |  |  |  |  |  |  |  |  |  |  |  |
| 0.2325 | 22 | igd | 0.1025 | A | \|\| id:ebi-a-GCST90001710 | ebi-a-GCST90001710 | TRUE | C | 43141391 | 1.48E-08 | reported | 3657 | 0.04095 | rs9611872 |  |  |  |  |  |  |  |  |  |  |  |  |  |  |  |
| 0.238 | 22 | igd | 0.1025 | A | \|\| id:ebi-a-GCST90001711 | ebi-a-GCST90001711 | TRUE | C | 43141391 | 6.17E-09 | reported | 3657 | 0.04085 | rs9611872 |  |  |  |  |  |  |  |  |  |  |  |  |  |  |  |
| 0.2896 | 6 | igd | 0.1025 | G | \|\| id:ebi-a-GCST90001758 | ebi-a-GCST90001758 | TRUE | A | 29778271 | 8.25E-09 | reported | 3657 | 0.05014 | rs9461562 |  |  |  |  |  |  |  |  |  |  |  |  |  |  |  |
| -0.3645 | 22 | igd | 0.1024 | C | \|\| id:ebi-a-GCST90001702 | ebi-a-GCST90001702 | TRUE | A | 43573834 | 1.01E-18 | reported | 3657 | 0.04103 | rs66536338 |  |  |  |  |  |  |  |  |  |  |  |  |  |  |  |
| -0.3697 | 22 | igd | 0.1024 | C | \|\| id:ebi-a-GCST90001703 | ebi-a-GCST90001703 | TRUE | A | 43573834 | 3.13E-19 | reported | 3657 | 0.04101 | rs66536338 |  |  |  |  |  |  |  |  |  |  |  |  |  |  |  |
| -0.337 | 22 | igd | 0.1024 | C | \|\| id:ebi-a-GCST90001704 | ebi-a-GCST90001704 | TRUE | A | 43573834 | 2.94E-16 | reported | 3657 | 0.04103 | rs66536338 |  |  |  |  |  |  |  |  |  |  |  |  |  |  |  |
| -0.3685 | 22 | igd | 0.1024 | C | \|\| id:ebi-a-GCST90001710 | ebi-a-GCST90001710 | TRUE | A | 43573834 | 3.33E-19 | reported | 3657 | 0.04091 | rs66536338 |  |  |  |  |  |  |  |  |  |  |  |  |  |  |  |
| -0.3617 | 22 | igd | 0.1024 | C | \|\| id:ebi-a-GCST90001711 | ebi-a-GCST90001711 | TRUE | A | 43573834 | 1.23E-18 | reported | 3657 | 0.04082 | rs66536338 |  |  |  |  |  |  |  |  |  |  |  |  |  |  |  |
| -0.4052 | 3 | igd | 0.1022 | A | \|\| id:ebi-a-GCST90002035 | ebi-a-GCST90002035 | TRUE | G | 52362580 | 9.55E-19 | reported | 2871 | 0.04552 | rs34628357 |  |  |  |  |  |  |  |  |  |  |  |  |  |  |  |
| -0.4661 | 3 | igd | 0.1022 | A | \|\| id:ebi-a-GCST90002036 | ebi-a-GCST90002036 | TRUE | G | 52362580 | 2.90E-24 | reported | 2871 | 0.04545 | rs34628357 |  |  |  |  |  |  |  |  |  |  |  |  |  |  |  |
| -0.2516 | 1 | igd | 0.1021 | A | \|\| id:ebi-a-GCST90001996 | ebi-a-GCST90001996 | TRUE | T | 161501445 | 1.41E-09 | reported | 3579 | 0.04144 | rs12120973 |  |  |  |  |  |  |  |  |  |  |  |  |  |  |  |
| -0.2169 | 5 | igd | 0.1019 | A | \|\| id:ebi-a-GCST90001686 | ebi-a-GCST90001686 | TRUE | G | 59819941 | 9.95E-09 | reported | 3440 | 0.03774 | rs7728865 |  |  |  |  |  |  |  |  |  |  |  |  |  |  |  |
| 0.3897 | 1 | igd | 0.1019 | A | \|\| id:ebi-a-GCST90001981 | ebi-a-GCST90001981 | TRUE | T | 161501445 | 3.77E-21 | reported | 3618 | 0.04103 | rs12120973 |  |  |  |  |  |  |  |  |  |  |  |  |  |  |  |
| 0.2701 | 10 | igd | 0.1019 | A | \|\| id:ebi-a-GCST90002061 | ebi-a-GCST90002061 | TRUE | G | 97235508 | 2.06E-09 | reported | 2920 | 0.04493 | rs7085831 |  |  |  |  |  |  |  |  |  |  |  |  |  |  |  |
| 0.2833 | 6 | igd | 0.1018 | A | \|\| id:ebi-a-GCST90002081 | ebi-a-GCST90002081 | TRUE | T | 31761201 | 1.58E-08 | reported | 3113 | 0.04999 | rs111359007 |  |  |  |  |  |  |  |  |  |  |  |  |  |  |  |
| 0.4848 | 19 | igd | 0.1017 | G | \|\| id:ebi-a-GCST90001948 | ebi-a-GCST90001948 | TRUE | A | 51677700 | 3.72E-16 | reported | 1633 | 0.05889 | rs17728644 |  |  |  |  |  |  |  |  |  |  |  |  |  |  |  |
| 0.4408 | 19 | igd | 0.1016 | G | \|\| id:ebi-a-GCST90001946 | ebi-a-GCST90001946 | TRUE | A | 51677700 | 1.97E-13 | reported | 1634 | 0.05946 | rs17728644 |  |  |  |  |  |  |  |  |  |  |  |  |  |  |  |
| 0.4405 | 19 | igd | 0.1016 | G | \|\| id:ebi-a-GCST90001949 | ebi-a-GCST90001949 | TRUE | A | 51677700 | 1.35E-13 | reported | 1634 | 0.05902 | rs17728644 |  |  |  |  |  |  |  |  |  |  |  |  |  |  |  |
| 0.2973 | 1 | igd | 0.1015 | A | \|\| id:ebi-a-GCST90001980 | ebi-a-GCST90001980 | TRUE | T | 161501445 | 6.30E-13 | reported | 3629 | 0.04118 | rs12120973 |  |  |  |  |  |  |  |  |  |  |  |  |  |  |  |
| -0.2802 | 6 | igd | 0.1015 | A | \|\| id:ebi-a-GCST90002121 | ebi-a-GCST90002121 | TRUE | T | 31761201 | 5.34E-09 | reported | 2915 | 0.04787 | rs111359007 |  |  |  |  |  |  |  |  |  |  |  |  |  |  |  |
| 0.3174 | 12 | igd | 0.1014 | T | \|\| id:ebi-a-GCST90002073 | ebi-a-GCST90002073 | TRUE | A | 69630522 | 3.75E-12 | reported | 2850 | 0.04549 | rs77817030 |  |  |  |  |  |  |  |  |  |  |  |  |  |  |  |
| -0.3122 | 1 | igd | 0.1013 | A | \|\| id:ebi-a-GCST90001997 | ebi-a-GCST90001997 | TRUE | G | 161506889 | 1.62E-13 | reported | 3590 | 0.04216 | rs79277331 |  |  |  |  |  |  |  |  |  |  |  |  |  |  |  |
| -0.285 | 1 | igd | 0.1013 | A | \|\| id:ebi-a-GCST90001995 | ebi-a-GCST90001995 | TRUE | G | 161506889 | 1.20E-11 | reported | 3590 | 0.0419 | rs79277331 |  |  |  |  |  |  |  |  |  |  |  |  |  |  |  |
| 0.3004 | 22 | igd | 0.1012 | C | \|\| id:ebi-a-GCST90001703 | ebi-a-GCST90001703 | TRUE | T | 43730300 | 5.28E-13 | reported | 3657 | 0.04147 | rs9612042 |  |  |  |  |  |  |  |  |  |  |  |  |  |  |  |
| 0.2836 | 22 | igd | 0.1012 | C | \|\| id:ebi-a-GCST90001704 | ebi-a-GCST90001704 | TRUE | T | 43730300 | 9.10E-12 | reported | 3657 | 0.04145 | rs9612042 |  |  |  |  |  |  |  |  |  |  |  |  |  |  |  |
| 0.3426 | 10 | igd | 0.1012 | G | \|\| id:ebi-a-GCST90002030 | ebi-a-GCST90002030 | TRUE | T | 98243844 | 1.08E-14 | reported | 2920 | 0.04409 | rs11188786 |  |  |  |  |  |  |  |  |  |  |  |  |  |  |  |
| 0.3217 | 10 | igd | 0.1012 | G | \|\| id:ebi-a-GCST90002032 | ebi-a-GCST90002032 | TRUE | T | 98243844 | 1.17E-12 | reported | 2920 | 0.04506 | rs11188786 |  |  |  |  |  |  |  |  |  |  |  |  |  |  |  |
| -0.3335 | 12 | igd | 0.1011 | G | \|\| id:ebi-a-GCST90002073 | ebi-a-GCST90002073 | TRUE | A | 70104692 | 4.43E-13 | reported | 2850 | 0.04583 | rs775459 |  |  |  |  |  |  |  |  |  |  |  |  |  |  |  |
| 0.4441 | 10 | igd | 0.1009 | A | \|\| id:ebi-a-GCST90001658 | ebi-a-GCST90001658 | TRUE | G | 97009657 | 4.50E-28 | reported | 3440 | 0.04007 | rs10882573 |  |  |  |  |  |  |  |  |  |  |  |  |  |  |  |
| 0.462 | 10 | igd | 0.1009 | A | \|\| id:ebi-a-GCST90001659 | ebi-a-GCST90001659 | TRUE | G | 97009657 | 2.81E-30 | reported | 3440 | 0.04002 | rs10882573 |  |  |  |  |  |  |  |  |  |  |  |  |  |  |  |
| 0.4844 | 10 | igd | 0.1009 | A | \|\| id:ebi-a-GCST90001660 | ebi-a-GCST90001660 | TRUE | G | 97009657 | 2.80E-30 | reported | 3408 | 0.04195 | rs10882573 |  |  |  |  |  |  |  |  |  |  |  |  |  |  |  |
| 0.4401 | 10 | igd | 0.1009 | A | \|\| id:ebi-a-GCST90001670 | ebi-a-GCST90001670 | TRUE | G | 97009657 | 1.42E-25 | reported | 3440 | 0.04177 | rs10882573 |  |  |  |  |  |  |  |  |  |  |  |  |  |  |  |
| 0.4462 | 10 | igd | 0.1009 | A | \|\| id:ebi-a-GCST90001671 | ebi-a-GCST90001671 | TRUE | G | 97009657 | 3.61E-26 | reported | 3440 | 0.04182 | rs10882573 |  |  |  |  |  |  |  |  |  |  |  |  |  |  |  |
| 0.462 | 10 | igd | 0.1009 | A | \|\| id:ebi-a-GCST90001672 | ebi-a-GCST90001672 | TRUE | G | 97009657 | 3.93E-27 | reported | 3408 | 0.04246 | rs10882573 |  |  |  |  |  |  |  |  |  |  |  |  |  |  |  |
| -0.2663 | 22 | igd | 0.1009 | T | \|\| id:ebi-a-GCST90001702 | ebi-a-GCST90001702 | TRUE | C | 40303934 | 4.47E-10 | reported | 3657 | 0.04259 | rs137963 |  |  |  |  |  |  |  |  |  |  |  |  |  |  |  |
| -0.2681 | 22 | igd | 0.1009 | T | \|\| id:ebi-a-GCST90001703 | ebi-a-GCST90001703 | TRUE | C | 40303934 | 3.35E-10 | reported | 3657 | 0.04256 | rs137963 |  |  |  |  |  |  |  |  |  |  |  |  |  |  |  |
| -0.2466 | 22 | igd | 0.1009 | T | \|\| id:ebi-a-GCST90001710 | ebi-a-GCST90001710 | TRUE | C | 40303934 | 7.21E-09 | reported | 3657 | 0.04252 | rs137963 |  |  |  |  |  |  |  |  |  |  |  |  |  |  |  |
| -0.2502 | 22 | igd | 0.1009 | T | \|\| id:ebi-a-GCST90001711 | ebi-a-GCST90001711 | TRUE | C | 40303934 | 3.83E-09 | reported | 3657 | 0.04237 | rs137963 |  |  |  |  |  |  |  |  |  |  |  |  |  |  |  |
| 0.3586 | 10 | igd | 0.1007 | A | \|\| id:ebi-a-GCST90001483 | ebi-a-GCST90001483 | TRUE | G | 97009657 | 5.53E-18 | reported | 3405 | 0.04127 | rs10882573 |  |  |  |  |  |  |  |  |  |  |  |  |  |  |  |
| 0.3885 | 10 | igd | 0.1007 | A | \|\| id:ebi-a-GCST90001484 | ebi-a-GCST90001484 | TRUE | G | 97009657 | 3.55E-21 | reported | 3437 | 0.04086 | rs10882573 |  |  |  |  |  |  |  |  |  |  |  |  |  |  |  |
| 0.381 | 10 | igd | 0.1007 | A | \|\| id:ebi-a-GCST90001485 | ebi-a-GCST90001485 | TRUE | G | 97009657 | 1.77E-20 | reported | 3437 | 0.04082 | rs10882573 |  |  |  |  |  |  |  |  |  |  |  |  |  |  |  |
| 0.4019 | 10 | igd | 0.1007 | A | \|\| id:ebi-a-GCST90001489 | ebi-a-GCST90001489 | TRUE | G | 97009657 | 3.86E-21 | reported | 3405 | 0.04231 | rs10882573 |  |  |  |  |  |  |  |  |  |  |  |  |  |  |  |
| 0.433 | 10 | igd | 0.1007 | A | \|\| id:ebi-a-GCST90001491 | ebi-a-GCST90001491 | TRUE | G | 97009657 | 5.13E-25 | reported | 3437 | 0.04159 | rs10882573 |  |  |  |  |  |  |  |  |  |  |  |  |  |  |  |
| 0.4553 | 10 | igd | 0.1007 | A | \|\| id:ebi-a-GCST90001495 | ebi-a-GCST90001495 | TRUE | G | 97009657 | 2.76E-26 | reported | 3405 | 0.04256 | rs10882573 |  |  |  |  |  |  |  |  |  |  |  |  |  |  |  |
| 0.4987 | 10 | igd | 0.1007 | A | \|\| id:ebi-a-GCST90001496 | ebi-a-GCST90001496 | TRUE | G | 97009657 | 5.73E-32 | reported | 3437 | 0.04195 | rs10882573 |  |  |  |  |  |  |  |  |  |  |  |  |  |  |  |
| 0.4948 | 10 | igd | 0.1007 | A | \|\| id:ebi-a-GCST90001497 | ebi-a-GCST90001497 | TRUE | G | 97009657 | 2.95E-32 | reported | 3437 | 0.04142 | rs10882573 |  |  |  |  |  |  |  |  |  |  |  |  |  |  |  |
| 0.2247 | 10 | igd | 0.1007 | G | \|\| id:ebi-a-GCST90001658 | ebi-a-GCST90001658 | TRUE | T | 98243844 | 1.43E-08 | reported | 3440 | 0.03954 | rs11188786 |  |  |  |  |  |  |  |  |  |  |  |  |  |  |  |
| 0.2199 | 10 | igd | 0.1007 | G | \|\| id:ebi-a-GCST90001659 | ebi-a-GCST90001659 | TRUE | T | 98243844 | 2.92E-08 | reported | 3440 | 0.03955 | rs11188786 |  |  |  |  |  |  |  |  |  |  |  |  |  |  |  |
| -0.2864 | 10 | igd | 0.1006 | G | \|\| id:ebi-a-GCST90001483 | ebi-a-GCST90001483 | TRUE | A | 97074129 | 4.58E-12 | reported | 3405 | 0.04125 | rs17849148 |  |  |  |  |  |  |  |  |  |  |  |  |  |  |  |
| -0.3056 | 10 | igd | 0.1004 | G | \|\| id:ebi-a-GCST90001484 | ebi-a-GCST90001484 | TRUE | A | 97074129 | 9.99E-14 | reported | 3437 | 0.04091 | rs17849148 |  |  |  |  |  |  |  |  |  |  |  |  |  |  |  |
| -0.2901 | 10 | igd | 0.1004 | G | \|\| id:ebi-a-GCST90001485 | ebi-a-GCST90001485 | TRUE | A | 97074129 | 1.55E-12 | reported | 3437 | 0.04088 | rs17849148 |  |  |  |  |  |  |  |  |  |  |  |  |  |  |  |
| 0.2249 | 10 | igd | 0.1003 | T | \|\| id:ebi-a-GCST90001658 | ebi-a-GCST90001658 | TRUE | C | 91880989 | 3.55E-08 | reported | 3440 | 0.04071 | rs141656130 |  |  |  |  |  |  |  |  |  |  |  |  |  |  |  |
| 0.2337 | 10 | igd | 0.1003 | T | \|\| id:ebi-a-GCST90001659 | ebi-a-GCST90001659 | TRUE | C | 91880989 | 1.02E-08 | reported | 3440 | 0.04071 | rs141656130 |  |  |  |  |  |  |  |  |  |  |  |  |  |  |  |
| -0.2485 | 19 | igd | 0.10014 | T | \|\| id:prot-a-439 | prot-a-439 | TRUE | C | 51575009 | 4.68E-09 | reported | 3301 | 0.0424 | rs62114140 |  |  |  |  |  |  |  |  |  |  |  |  |  |  |  |
| 0.2439 | 10 | igd | 0.1001 | T | \|\| id:ebi-a-GCST90001484 | ebi-a-GCST90001484 | TRUE | C | 91880989 | 3.62E-09 | reported | 3437 | 0.04122 | rs141656130 |  |  |  |  |  |  |  |  |  |  |  |  |  |  |  |
| 0.291 | 1 | igd | 0.1001 | G | \|\| id:ebi-a-GCST90001832 | ebi-a-GCST90001832 | TRUE | A | 170154115 | 1.37E-11 | reported | 2872 | 0.04287 | rs75994182 |  |  |  |  |  |  |  |  |  |  |  |  |  |  |  |
| -0.2612 | 1 | igd | 0.0999 | A | \|\| id:ebi-a-GCST90001860 | ebi-a-GCST90001860 | TRUE | G | 165193662 | 1.23E-08 | reported | 2919 | 0.04572 | rs12742947 |  |  |  |  |  |  |  |  |  |  |  |  |  |  |  |
| 0.2839 | 12 | igd | 0.0999 | T | \|\| id:ebi-a-GCST90002074 | ebi-a-GCST90002074 | TRUE | A | 69630522 | 1.08E-10 | reported | 3112 | 0.04382 | rs77817030 |  |  |  |  |  |  |  |  |  |  |  |  |  |  |  |
| -0.4215 | 22 | igd | 0.0998 | G | \|\| id:ebi-a-GCST90001830 | ebi-a-GCST90001830 | TRUE | A | 42321591 | 1.03E-25 | reported | 3648 | 0.0399 | rs5996087 |  |  |  |  |  |  |  |  |  |  |  |  |  |  |  |
| 0.2334 | 10 | igd | 0.0997 | C | \|\| id:ebi-a-GCST90001779 | ebi-a-GCST90001779 | TRUE | T | 5827901 | 2.29E-08 | reported | 3657 | 0.04168 | rs17403348 |  |  |  |  |  |  |  |  |  |  |  |  |  |  |  |
| 0.2314 | 10 | igd | 0.0997 | C | \|\| id:ebi-a-GCST90001791 | ebi-a-GCST90001791 | TRUE | T | 5827901 | 3.86E-08 | reported | 3657 | 0.04201 | rs17403348 |  |  |  |  |  |  |  |  |  |  |  |  |  |  |  |
| -0.2408 | 22 | igd | 0.0995 | G | \|\| id:ebi-a-GCST90001702 | ebi-a-GCST90001702 | TRUE | C | 39757623 | 7.04E-09 | reported | 3657 | 0.04149 | rs142952844 |  |  |  |  |  |  |  |  |  |  |  |  |  |  |  |
| -0.228 | 22 | igd | 0.0995 | G | \|\| id:ebi-a-GCST90001703 | ebi-a-GCST90001703 | TRUE | C | 39757623 | 4.22E-08 | reported | 3657 | 0.04151 | rs142952844 |  |  |  |  |  |  |  |  |  |  |  |  |  |  |  |
| -0.2364 | 22 | igd | 0.0995 | G | \|\| id:ebi-a-GCST90001704 | ebi-a-GCST90001704 | TRUE | C | 39757623 | 1.28E-08 | reported | 3657 | 0.04145 | rs142952844 |  |  |  |  |  |  |  |  |  |  |  |  |  |  |  |
| -0.2264 | 22 | igd | 0.0995 | G | \|\| id:ebi-a-GCST90001711 | ebi-a-GCST90001711 | TRUE | C | 39757623 | 4.57E-08 | reported | 3657 | 0.04131 | rs142952844 |  |  |  |  |  |  |  |  |  |  |  |  |  |  |  |
| 0.4038 | 1 | igd | 0.0995 | A | \|\| id:ebi-a-GCST90001985 | ebi-a-GCST90001985 | TRUE | C | 161520472 | 6.01E-21 | reported | 3629 | 0.04274 | rs139755165 |  |  |  |  |  |  |  |  |  |  |  |  |  |  |  |
| 0.2335 | 19 | igd | 0.0993 | T | \|\| id:ebi-a-GCST90001745 | ebi-a-GCST90001745 | TRUE | G | 49768214 | 1.12E-08 | reported | 3657 | 0.04078 | rs12986007 |  |  |  |  |  |  |  |  |  |  |  |  |  |  |  |
| 0.2386 | 3 | igd | 0.0993 | T | \|\| id:ebi-a-GCST90001798 | ebi-a-GCST90001798 | TRUE | C | 31889065 | 8.35E-09 | reported | 3657 | 0.04132 | rs111428221 |  |  |  |  |  |  |  |  |  |  |  |  |  |  |  |
| 0.4574 | 10 | igd | 0.0993 | C | \|\| id:ebi-a-GCST90002030 | ebi-a-GCST90002030 | TRUE | T | 97318089 | 6.43E-24 | reported | 2920 | 0.04496 | rs35302565 |  |  |  |  |  |  |  |  |  |  |  |  |  |  |  |
| 0.3957 | 10 | igd | 0.0993 | C | \|\| id:ebi-a-GCST90002032 | ebi-a-GCST90002032 | TRUE | T | 97318089 | 1.71E-17 | reported | 2920 | 0.0462 | rs35302565 |  |  |  |  |  |  |  |  |  |  |  |  |  |  |  |
| 0.3249 | 6 | igd | 0.0991 | A | \|\| id:ebi-a-GCST90002105 | ebi-a-GCST90002105 | TRUE | C | 33791725 | 5.86E-11 | reported | 2872 | 0.04944 | rs767894 |  |  |  |  |  |  |  |  |  |  |  |  |  |  |  |
| 0.398 | 6 | igd | 0.0991 | T | \|\| id:ebi-a-GCST90002105 | ebi-a-GCST90002105 | TRUE | G | 32228496 | 2.84E-15 | reported | 2872 | 0.05011 | rs145458341 |  |  |  |  |  |  |  |  |  |  |  |  |  |  |  |
| -1.493 | 22 | igd | 0.099 | T | \|\| id:ebi-a-GCST90001702 | ebi-a-GCST90001702 | TRUE | C | 42315371 | 1.00E-200 | reported | 3657 | 0.03391 | rs73165129 |  |  |  |  |  |  |  |  |  |  |  |  |  |  |  |
| -1.487 | 22 | igd | 0.099 | T | \|\| id:ebi-a-GCST90001703 | ebi-a-GCST90001703 | TRUE | C | 42315371 | 1.00E-200 | reported | 3657 | 0.03398 | rs73165129 |  |  |  |  |  |  |  |  |  |  |  |  |  |  |  |
| -1.51 | 22 | igd | 0.099 | T | \|\| id:ebi-a-GCST90001704 | ebi-a-GCST90001704 | TRUE | C | 42315371 | 1.00E-200 | reported | 3657 | 0.03363 | rs73165129 |  |  |  |  |  |  |  |  |  |  |  |  |  |  |  |
| -1.427 | 22 | igd | 0.099 | T | \|\| id:ebi-a-GCST90001710 | ebi-a-GCST90001710 | TRUE | C | 42315371 | 1.00E-200 | reported | 3657 | 0.03453 | rs73165129 |  |  |  |  |  |  |  |  |  |  |  |  |  |  |  |
| -1.439 | 22 | igd | 0.099 | T | \|\| id:ebi-a-GCST90001711 | ebi-a-GCST90001711 | TRUE | C | 42315371 | 1.00E-200 | reported | 3657 | 0.03429 | rs73165129 |  |  |  |  |  |  |  |  |  |  |  |  |  |  |  |
| -0.2468 | 1 | igd | 0.0989 | T | \|\| id:ebi-a-GCST90001989 | ebi-a-GCST90001989 | TRUE | C | 161269415 | 5.56E-09 | reported | 3621 | 0.04224 | rs111516661 |  |  |  |  |  |  |  |  |  |  |  |  |  |  |  |
| 0.3127 | 10 | igd | 0.0988 | C | \|\| id:ebi-a-GCST90002031 | ebi-a-GCST90002031 | TRUE | T | 98244494 | 1.00E-11 | reported | 2920 | 0.04576 | rs9325483 |  |  |  |  |  |  |  |  |  |  |  |  |  |  |  |
| -0.5191 | 22 | igd | 0.0987 | T | \|\| id:ebi-a-GCST90001701 | ebi-a-GCST90001701 | TRUE | C | 42315371 | 6.75E-37 | reported | 3592 | 0.04044 | rs73165129 |  |  |  |  |  |  |  |  |  |  |  |  |  |  |  |
| 0.3437 | 10 | igd | 0.0986 | C | \|\| id:ebi-a-GCST90001496 | ebi-a-GCST90001496 | TRUE | T | 97318089 | 1.38E-15 | reported | 3437 | 0.04283 | rs35302565 |  |  |  |  |  |  |  |  |  |  |  |  |  |  |  |
| 0.3419 | 10 | igd | 0.0986 | C | \|\| id:ebi-a-GCST90001497 | ebi-a-GCST90001497 | TRUE | T | 97318089 | 8.53E-16 | reported | 3437 | 0.04228 | rs35302565 |  |  |  |  |  |  |  |  |  |  |  |  |  |  |  |
| 0.3088 | 10 | igd | 0.0985 | C | \|\| id:ebi-a-GCST90001658 | ebi-a-GCST90001658 | TRUE | T | 97318089 | 5.06E-14 | reported | 3440 | 0.04084 | rs35302565 |  |  |  |  |  |  |  |  |  |  |  |  |  |  |  |
| 0.3087 | 10 | igd | 0.0985 | C | \|\| id:ebi-a-GCST90001659 | ebi-a-GCST90001659 | TRUE | T | 97318089 | 5.20E-14 | reported | 3440 | 0.04084 | rs35302565 |  |  |  |  |  |  |  |  |  |  |  |  |  |  |  |
| 0.266 | 10 | igd | 0.0985 | C | \|\| id:ebi-a-GCST90001670 | ebi-a-GCST90001670 | TRUE | T | 97318089 | 4.88E-10 | reported | 3440 | 0.04263 | rs35302565 |  |  |  |  |  |  |  |  |  |  |  |  |  |  |  |
| 0.3678 | 10 | igd | 0.0985 | T | \|\| id:ebi-a-GCST90002031 | ebi-a-GCST90002031 | TRUE | C | 97318185 | 4.30E-15 | reported | 2920 | 0.04662 | rs35932840 |  |  |  |  |  |  |  |  |  |  |  |  |  |  |  |
| 0.4007 | 6 | igd | 0.0985 | C | \|\| id:ebi-a-GCST90002112 | ebi-a-GCST90002112 | TRUE | A | 30943178 | 1.83E-10 | reported | 1635 | 0.06245 | rs6926507 |  |  |  |  |  |  |  |  |  |  |  |  |  |  |  |
| 0.293 | 10 | igd | 0.0982 | C | \|\| id:ebi-a-GCST90001660 | ebi-a-GCST90001660 | TRUE | T | 97318089 | 1.05E-11 | reported | 3408 | 0.04294 | rs35302565 |  |  |  |  |  |  |  |  |  |  |  |  |  |  |  |
| 0.2562 | 10 | igd | 0.0982 | C | \|\| id:ebi-a-GCST90001672 | ebi-a-GCST90001672 | TRUE | T | 97318089 | 4.06E-09 | reported | 3408 | 0.04344 | rs35302565 |  |  |  |  |  |  |  |  |  |  |  |  |  |  |  |
| -0.2525 | 11 | igd | 0.0982 | T | \|\| id:ebi-a-GCST90001760 | ebi-a-GCST90001760 | TRUE | C | 60249734 | 1.45E-09 | reported | 3657 | 0.04163 | rs4447195 |  |  |  |  |  |  |  |  |  |  |  |  |  |  |  |
| 0.2766 | 22 | igd | 0.0979 | C | \|\| id:ebi-a-GCST90001702 | ebi-a-GCST90001702 | TRUE | T | 42680201 | 1.56E-10 | reported | 3657 | 0.0431 | rs76462269 |  |  |  |  |  |  |  |  |  |  |  |  |  |  |  |
| 0.2958 | 22 | igd | 0.0979 | C | \|\| id:ebi-a-GCST90001703 | ebi-a-GCST90001703 | TRUE | T | 42680201 | 7.46E-12 | reported | 3657 | 0.04305 | rs76462269 |  |  |  |  |  |  |  |  |  |  |  |  |  |  |  |
| 0.2775 | 22 | igd | 0.0979 | C | \|\| id:ebi-a-GCST90001704 | ebi-a-GCST90001704 | TRUE | T | 42680201 | 1.27E-10 | reported | 3657 | 0.04302 | rs76462269 |  |  |  |  |  |  |  |  |  |  |  |  |  |  |  |
| 0.2565 | 13 | igd | 0.0979 | A | \|\| id:ebi-a-GCST90001907 | ebi-a-GCST90001907 | TRUE | G | 106399307 | 4.36E-08 | reported | 2910 | 0.04671 | rs61968809 |  |  |  |  |  |  |  |  |  |  |  |  |  |  |  |
| 0.2412 | 10 | igd | 0.0978 | C | \|\| id:ebi-a-GCST90001489 | ebi-a-GCST90001489 | TRUE | T | 95946164 | 2.29E-08 | reported | 3405 | 0.04306 | rs61886324 |  |  |  |  |  |  |  |  |  |  |  |  |  |  |  |
| 0.2692 | 10 | igd | 0.0977 | C | \|\| id:ebi-a-GCST90001660 | ebi-a-GCST90001660 | TRUE | T | 95946164 | 4.17E-10 | reported | 3408 | 0.04296 | rs61886324 |  |  |  |  |  |  |  |  |  |  |  |  |  |  |  |
| 0.3733 | 10 | igd | 0.0977 | C | \|\| id:ebi-a-GCST90001672 | ebi-a-GCST90001672 | TRUE | T | 95946164 | 7.85E-18 | reported | 3408 | 0.04316 | rs61886324 |  |  |  |  |  |  |  |  |  |  |  |  |  |  |  |
| -0.3673 | 1 | igd | 0.0976 | T | \|\| id:ebi-a-GCST90001845 | ebi-a-GCST90001845 | TRUE | C | 167442107 | 4.27E-16 | reported | 2911 | 0.04491 | rs61810481 |  |  |  |  |  |  |  |  |  |  |  |  |  |  |  |
| 0.3557 | 10 | igd | 0.0975 | T | \|\| id:ebi-a-GCST90001484 | ebi-a-GCST90001484 | TRUE | C | 97318185 | 2.36E-17 | reported | 3437 | 0.04175 | rs35932840 |  |  |  |  |  |  |  |  |  |  |  |  |  |  |  |
| 0.3196 | 10 | igd | 0.0975 | T | \|\| id:ebi-a-GCST90001485 | ebi-a-GCST90001485 | TRUE | C | 97318185 | 2.60E-14 | reported | 3437 | 0.04177 | rs35932840 |  |  |  |  |  |  |  |  |  |  |  |  |  |  |  |
| 0.3561 | 10 | igd | 0.0975 | T | \|\| id:ebi-a-GCST90001491 | ebi-a-GCST90001491 | TRUE | C | 97318185 | 7.71E-17 | reported | 3437 | 0.0425 | rs35932840 |  |  |  |  |  |  |  |  |  |  |  |  |  |  |  |
| 0.2706 | 10 | igd | 0.0975 | C | \|\| id:ebi-a-GCST90001658 | ebi-a-GCST90001658 | TRUE | T | 95946164 | 4.87E-11 | reported | 3440 | 0.04102 | rs61886324 |  |  |  |  |  |  |  |  |  |  |  |  |  |  |  |
| 0.2613 | 10 | igd | 0.0975 | C | \|\| id:ebi-a-GCST90001659 | ebi-a-GCST90001659 | TRUE | T | 95946164 | 2.21E-10 | reported | 3440 | 0.04104 | rs61886324 |  |  |  |  |  |  |  |  |  |  |  |  |  |  |  |
| 0.3744 | 10 | igd | 0.0975 | C | \|\| id:ebi-a-GCST90001670 | ebi-a-GCST90001670 | TRUE | T | 95946164 | 1.87E-18 | reported | 3440 | 0.04247 | rs61886324 |  |  |  |  |  |  |  |  |  |  |  |  |  |  |  |
| 0.3691 | 10 | igd | 0.0975 | C | \|\| id:ebi-a-GCST90001671 | ebi-a-GCST90001671 | TRUE | T | 95946164 | 6.52E-18 | reported | 3440 | 0.04256 | rs61886324 |  |  |  |  |  |  |  |  |  |  |  |  |  |  |  |
| -0.2586 | 1 | igd | 0.0973 | G | \|\| id:ebi-a-GCST90001856 | ebi-a-GCST90001856 | TRUE | A | 167425846 | 2.36E-08 | reported | 2919 | 0.04618 | rs1891257 |  |  |  |  |  |  |  |  |  |  |  |  |  |  |  |
| -0.2928 | 1 | igd | 0.0973 | G | \|\| id:ebi-a-GCST90001860 | ebi-a-GCST90001860 | TRUE | A | 167425846 | 2.54E-10 | reported | 2919 | 0.04613 | rs1891257 |  |  |  |  |  |  |  |  |  |  |  |  |  |  |  |
| 0.2363 | 19 | igd | 0.0972 | T | \|\| id:ebi-a-GCST90001754 | ebi-a-GCST90001754 | TRUE | C | 50940778 | 2.51E-08 | reported | 3657 | 0.04232 | rs61731791 |  |  |  |  |  |  |  |  |  |  |  |  |  |  |  |
| 0.2328 | 19 | igd | 0.0972 | T | \|\| id:ebi-a-GCST90001755 | ebi-a-GCST90001755 | TRUE | C | 50940778 | 3.86E-08 | reported | 3657 | 0.04226 | rs61731791 |  |  |  |  |  |  |  |  |  |  |  |  |  |  |  |
| 0.2688 | 19 | igd | 0.0972 | T | \|\| id:ebi-a-GCST90001757 | ebi-a-GCST90001757 | TRUE | C | 50940778 | 1.75E-10 | reported | 3657 | 0.042 | rs61731791 |  |  |  |  |  |  |  |  |  |  |  |  |  |  |  |
| 0.2358 | 19 | igd | 0.0972 | T | \|\| id:ebi-a-GCST90001761 | ebi-a-GCST90001761 | TRUE | C | 50940778 | 2.83E-08 | reported | 3657 | 0.04238 | rs61731791 |  |  |  |  |  |  |  |  |  |  |  |  |  |  |  |
| 0.2824 | 10 | igd | 0.0971 | T | \|\| id:ebi-a-GCST90001483 | ebi-a-GCST90001483 | TRUE | C | 97318185 | 2.71E-11 | reported | 3405 | 0.04225 | rs35932840 |  |  |  |  |  |  |  |  |  |  |  |  |  |  |  |
| 0.3153 | 10 | igd | 0.0971 | T | \|\| id:ebi-a-GCST90001489 | ebi-a-GCST90001489 | TRUE | C | 97318185 | 4.31E-13 | reported | 3405 | 0.04335 | rs35932840 |  |  |  |  |  |  |  |  |  |  |  |  |  |  |  |
| 0.3087 | 10 | igd | 0.0971 | T | \|\| id:ebi-a-GCST90001495 | ebi-a-GCST90001495 | TRUE | C | 97318185 | 1.97E-12 | reported | 3405 | 0.04371 | rs35932840 |  |  |  |  |  |  |  |  |  |  |  |  |  |  |  |
| -0.255 | 11 | igd | 0.0971 | T | \|\| id:ebi-a-GCST90001755 | ebi-a-GCST90001755 | TRUE | C | 60164527 | 5.41E-10 | reported | 3657 | 0.04098 | rs10501383 |  |  |  |  |  |  |  |  |  |  |  |  |  |  |  |
| -0.2991 | 11 | igd | 0.0971 | T | \|\| id:ebi-a-GCST90001761 | ebi-a-GCST90001761 | TRUE | C | 60164527 | 3.61E-13 | reported | 3657 | 0.04099 | rs10501383 |  |  |  |  |  |  |  |  |  |  |  |  |  |  |  |
| -0.4523 | 1 | igd | 0.0971 | T | \|\| id:ebi-a-GCST90001854 | ebi-a-GCST90001854 | TRUE | C | 167442107 | 5.62E-24 | reported | 2919 | 0.04439 | rs61810481 |  |  |  |  |  |  |  |  |  |  |  |  |  |  |  |
| -0.3873 | 1 | igd | 0.0971 | T | \|\| id:ebi-a-GCST90001856 | ebi-a-GCST90001856 | TRUE | C | 167442107 | 1.25E-17 | reported | 2919 | 0.04501 | rs61810481 |  |  |  |  |  |  |  |  |  |  |  |  |  |  |  |
| -0.479 | 1 | igd | 0.0971 | T | \|\| id:ebi-a-GCST90001860 | ebi-a-GCST90001860 | TRUE | C | 167442107 | 3.58E-26 | reported | 2919 | 0.04483 | rs61810481 |  |  |  |  |  |  |  |  |  |  |  |  |  |  |  |
| 0.4789 | 1 | igd | 0.0969 | C | \|\| id:ebi-a-GCST90001526 | ebi-a-GCST90001526 | TRUE | T | 161477028 | 7.90E-17 | reported | 1858 | 0.05693 | rs4657040 |  |  |  |  |  |  |  |  |  |  |  |  |  |  |  |
| -0.4775 | 1 | igd | 0.0969 | C | \|\| id:ebi-a-GCST90001528 | ebi-a-GCST90001528 | TRUE | T | 161477028 | 1.03E-16 | reported | 1858 | 0.05698 | rs4657040 |  |  |  |  |  |  |  |  |  |  |  |  |  |  |  |
| 0.3857 | 6 | igd | 0.0969 | A | \|\| id:ebi-a-GCST90002108 | ebi-a-GCST90002108 | TRUE | C | 33796411 | 3.71E-09 | reported | 1579 | 0.06503 | rs73747332 |  |  |  |  |  |  |  |  |  |  |  |  |  |  |  |
| 0.458 | 1 | igd | 0.0969 | C | \|\| id:ebi-a-GCST90002091 | ebi-a-GCST90002091 | TRUE | T | 161477028 | 1.62E-13 | reported | 1635 | 0.06155 | rs4657040 |  |  |  |  |  |  |  |  |  |  |  |  |  |  |  |
| -0.515 | 22 | igd | 0.0967 | T | \|\| id:ebi-a-GCST90001702 | ebi-a-GCST90001702 | TRUE | C | 42761285 | 1.23E-32 | reported | 3657 | 0.04287 | rs116900029 |  |  |  |  |  |  |  |  |  |  |  |  |  |  |  |
| -0.4955 | 22 | igd | 0.0967 | T | \|\| id:ebi-a-GCST90001703 | ebi-a-GCST90001703 | TRUE | C | 42761285 | 2.54E-30 | reported | 3657 | 0.04291 | rs116900029 |  |  |  |  |  |  |  |  |  |  |  |  |  |  |  |
| -0.2687 | 11 | igd | 0.0967 | T | \|\| id:ebi-a-GCST90001743 | ebi-a-GCST90001743 | TRUE | C | 60230531 | 1.66E-10 | reported | 3660 | 0.04193 | rs2070770 |  |  |  |  |  |  |  |  |  |  |  |  |  |  |  |
| -0.2571 | 11 | igd | 0.0967 | T | \|\| id:ebi-a-GCST90001748 | ebi-a-GCST90001748 | TRUE | C | 60230531 | 7.49E-10 | reported | 3645 | 0.04166 | rs2070770 |  |  |  |  |  |  |  |  |  |  |  |  |  |  |  |
| 0.278 | 10 | igd | 0.0967 | C | \|\| id:ebi-a-GCST90001961 | ebi-a-GCST90001961 | TRUE | T | 95393031 | 4.36E-09 | reported | 2920 | 0.04722 | rs141535732 |  |  |  |  |  |  |  |  |  |  |  |  |  |  |  |
| -0.2888 | 10 | igd | 0.0967 | C | \|\| id:ebi-a-GCST90002030 | ebi-a-GCST90002030 | TRUE | T | 95393031 | 7.59E-10 | reported | 2920 | 0.04677 | rs141535732 |  |  |  |  |  |  |  |  |  |  |  |  |  |  |  |
| -0.2563 | 11 | igd | 0.0966 | T | \|\| id:ebi-a-GCST90001762 | ebi-a-GCST90001762 | TRUE | C | 60230531 | 1.04E-09 | reported | 3658 | 0.04188 | rs2070770 |  |  |  |  |  |  |  |  |  |  |  |  |  |  |  |
| 0.2823 | 10 | igd | 0.0966 | T | \|\| id:ebi-a-GCST90001775 | ebi-a-GCST90001775 | TRUE | C | 6167124 | 3.51E-11 | reported | 3660 | 0.04249 | rs10905856 |  |  |  |  |  |  |  |  |  |  |  |  |  |  |  |
| 0.2391 | 10 | igd | 0.0965 | C | \|\| id:ebi-a-GCST90001495 | ebi-a-GCST90001495 | TRUE | G | 97983918 | 4.75E-08 | reported | 3405 | 0.04369 | rs72811013 |  |  |  |  |  |  |  |  |  |  |  |  |  |  |  |
| -0.2664 | 11 | igd | 0.0965 | T | \|\| id:ebi-a-GCST90001747 | ebi-a-GCST90001747 | TRUE | C | 60230531 | 2.25E-10 | reported | 3657 | 0.04188 | rs2070770 |  |  |  |  |  |  |  |  |  |  |  |  |  |  |  |
| -0.295 | 11 | igd | 0.0965 | T | \|\| id:ebi-a-GCST90001752 | ebi-a-GCST90001752 | TRUE | C | 60230531 | 1.99E-12 | reported | 3657 | 0.04178 | rs2070770 |  |  |  |  |  |  |  |  |  |  |  |  |  |  |  |
| -0.2657 | 11 | igd | 0.0965 | T | \|\| id:ebi-a-GCST90001757 | ebi-a-GCST90001757 | TRUE | C | 60230531 | 2.09E-10 | reported | 3657 | 0.0417 | rs2070770 |  |  |  |  |  |  |  |  |  |  |  |  |  |  |  |
| -0.2717 | 11 | igd | 0.0965 | T | \|\| id:ebi-a-GCST90001758 | ebi-a-GCST90001758 | TRUE | C | 60230531 | 8.13E-11 | reported | 3657 | 0.04169 | rs2070770 |  |  |  |  |  |  |  |  |  |  |  |  |  |  |  |
| -0.2612 | 11 | igd | 0.0965 | T | \|\| id:ebi-a-GCST90001759 | ebi-a-GCST90001759 | TRUE | C | 60230531 | 5.15E-10 | reported | 3657 | 0.04191 | rs2070770 |  |  |  |  |  |  |  |  |  |  |  |  |  |  |  |
| 0.2742 | 10 | igd | 0.0965 | T | \|\| id:ebi-a-GCST90001794 | ebi-a-GCST90001794 | TRUE | C | 6167124 | 1.25E-10 | reported | 3658 | 0.0425 | rs10905856 |  |  |  |  |  |  |  |  |  |  |  |  |  |  |  |
| 0.3953 | 19 | igd | 0.0965 | A | \|\| id:ebi-a-GCST90001954 | ebi-a-GCST90001954 | TRUE | G | 51785550 | 6.86E-10 | reported | 1555 | 0.06368 | rs1710386 |  |  |  |  |  |  |  |  |  |  |  |  |  |  |  |
| 0.383 | 19 | igd | 0.0965 | A | \|\| id:ebi-a-GCST90001955 | ebi-a-GCST90001955 | TRUE | G | 51785550 | 2.38E-09 | reported | 1554 | 0.06377 | rs1710386 |  |  |  |  |  |  |  |  |  |  |  |  |  |  |  |
| -0.2845 | 10 | igd | 0.0964 | C | \|\| id:ebi-a-GCST90001660 | ebi-a-GCST90001660 | TRUE | T | 95393031 | 1.03E-10 | reported | 3408 | 0.04388 | rs141535732 |  |  |  |  |  |  |  |  |  |  |  |  |  |  |  |
| 0.2609 | 10 | igd | 0.0964 | C | \|\| id:ebi-a-GCST90001660 | ebi-a-GCST90001660 | TRUE | G | 97983918 | 1.55E-09 | reported | 3408 | 0.04309 | rs72811013 |  |  |  |  |  |  |  |  |  |  |  |  |  |  |  |
| -0.2697 | 10 | igd | 0.0964 | C | \|\| id:ebi-a-GCST90001672 | ebi-a-GCST90001672 | TRUE | T | 95393031 | 1.33E-09 | reported | 3408 | 0.04435 | rs141535732 |  |  |  |  |  |  |  |  |  |  |  |  |  |  |  |
| 0.3302 | 10 | igd | 0.0964 | T | \|\| id:ebi-a-GCST90001779 | ebi-a-GCST90001779 | TRUE | C | 6167124 | 6.67E-15 | reported | 3657 | 0.0422 | rs10905856 |  |  |  |  |  |  |  |  |  |  |  |  |  |  |  |
| 0.3078 | 10 | igd | 0.0964 | T | \|\| id:ebi-a-GCST90001784 | ebi-a-GCST90001784 | TRUE | C | 6167124 | 5.68E-13 | reported | 3657 | 0.04255 | rs10905856 |  |  |  |  |  |  |  |  |  |  |  |  |  |  |  |
| 0.3054 | 10 | igd | 0.0964 | T | \|\| id:ebi-a-GCST90001791 | ebi-a-GCST90001791 | TRUE | C | 6167124 | 9.01E-13 | reported | 3657 | 0.04259 | rs10905856 |  |  |  |  |  |  |  |  |  |  |  |  |  |  |  |
| -0.2469 | 10 | igd | 0.0963 | C | \|\| id:ebi-a-GCST90001495 | ebi-a-GCST90001495 | TRUE | T | 95393031 | 2.99E-08 | reported | 3405 | 0.04444 | rs141535732 |  |  |  |  |  |  |  |  |  |  |  |  |  |  |  |
| 0.4072 | 19 | igd | 0.0963 | A | \|\| id:ebi-a-GCST90001953 | ebi-a-GCST90001953 | TRUE | G | 51785550 | 2.03E-10 | reported | 1558 | 0.06361 | rs1710386 |  |  |  |  |  |  |  |  |  |  |  |  |  |  |  |
| -0.2903 | 6 | igd | 0.0962 | T | \|\| id:ebi-a-GCST90001692 | ebi-a-GCST90001692 | TRUE | C | 31022928 | 1.66E-08 | reported | 3440 | 0.05132 | rs62401699 |  |  |  |  |  |  |  |  |  |  |  |  |  |  |  |
| -0.2917 | 10 | igd | 0.0961 | C | \|\| id:ebi-a-GCST90001658 | ebi-a-GCST90001658 | TRUE | T | 95393031 | 3.86E-12 | reported | 3440 | 0.04187 | rs141535732 |  |  |  |  |  |  |  |  |  |  |  |  |  |  |  |
| -0.2775 | 10 | igd | 0.0961 | C | \|\| id:ebi-a-GCST90001659 | ebi-a-GCST90001659 | TRUE | T | 95393031 | 4.05E-11 | reported | 3440 | 0.0419 | rs141535732 |  |  |  |  |  |  |  |  |  |  |  |  |  |  |  |
| -0.2771 | 10 | igd | 0.0961 | C | \|\| id:ebi-a-GCST90001670 | ebi-a-GCST90001670 | TRUE | T | 95393031 | 2.38E-10 | reported | 3440 | 0.04361 | rs141535732 |  |  |  |  |  |  |  |  |  |  |  |  |  |  |  |
| -0.2827 | 10 | igd | 0.0961 | C | \|\| id:ebi-a-GCST90001671 | ebi-a-GCST90001671 | TRUE | T | 95393031 | 1.11E-10 | reported | 3440 | 0.04368 | rs141535732 |  |  |  |  |  |  |  |  |  |  |  |  |  |  |  |
| -0.2406 | 10 | igd | 0.096 | C | \|\| id:ebi-a-GCST90001497 | ebi-a-GCST90001497 | TRUE | T | 95393031 | 3.28E-08 | reported | 3437 | 0.04345 | rs141535732 |  |  |  |  |  |  |  |  |  |  |  |  |  |  |  |
| 0.3751 | 6 | igd | 0.096 | T | \|\| id:ebi-a-GCST90002109 | ebi-a-GCST90002109 | TRUE | G | 32228496 | 8.28E-09 | reported | 1578 | 0.06473 | rs145458341 |  |  |  |  |  |  |  |  |  |  |  |  |  |  |  |
| 0.5623 | 6 | igd | 0.0959 | T | \|\| id:ebi-a-GCST90002108 | ebi-a-GCST90002108 | TRUE | G | 32228496 | 6.06E-18 | reported | 1579 | 0.06436 | rs145458341 |  |  |  |  |  |  |  |  |  |  |  |  |  |  |  |
| 0.2186 | 14 | igd | 0.0957 | C | \|\| id:ebi-a-GCST90001974 | ebi-a-GCST90001974 | TRUE | T | 29178686 | 4.71E-08 | reported | 3113 | 0.03992 | rs77789294 |  |  |  |  |  |  |  |  |  |  |  |  |  |  |  |
| 0.2544 | 1 | igd | 0.0956 | T | \|\| id:ebi-a-GCST90001474 | ebi-a-GCST90001474 | TRUE | C | 161269415 | 3.03E-09 | reported | 3410 | 0.04279 | rs111516661 |  |  |  |  |  |  |  |  |  |  |  |  |  |  |  |
| -0.3595 | 10 | igd | 0.0955 | C | \|\| id:ebi-a-GCST90002030 | ebi-a-GCST90002030 | TRUE | T | 96178235 | 5.43E-15 | reported | 2920 | 0.04575 | rs78415565 |  |  |  |  |  |  |  |  |  |  |  |  |  |  |  |
| -0.3027 | 10 | igd | 0.0955 | C | \|\| id:ebi-a-GCST90002031 | ebi-a-GCST90002031 | TRUE | T | 96178235 | 1.36E-10 | reported | 2920 | 0.04697 | rs78415565 |  |  |  |  |  |  |  |  |  |  |  |  |  |  |  |
| -0.3666 | 10 | igd | 0.0955 | C | \|\| id:ebi-a-GCST90002032 | ebi-a-GCST90002032 | TRUE | T | 96178235 | 6.50E-15 | reported | 2920 | 0.04679 | rs78415565 |  |  |  |  |  |  |  |  |  |  |  |  |  |  |  |
| -0.2896 | 10 | igd | 0.0954 | C | \|\| id:ebi-a-GCST90002031 | ebi-a-GCST90002031 | TRUE | T | 96301156 | 7.23E-10 | reported | 2920 | 0.04686 | rs114795233 |  |  |  |  |  |  |  |  |  |  |  |  |  |  |  |
| -0.3496 | 10 | igd | 0.0954 | C | \|\| id:ebi-a-GCST90002032 | ebi-a-GCST90002032 | TRUE | T | 96301156 | 9.14E-14 | reported | 2920 | 0.04669 | rs114795233 |  |  |  |  |  |  |  |  |  |  |  |  |  |  |  |
| -0.3631 | 1 | igd | 0.0954 | C | \|\| id:ebi-a-GCST90001996 | ebi-a-GCST90001996 | TRUE | T | 161477028 | 1.88E-17 | reported | 3579 | 0.04249 | rs4657040 |  |  |  |  |  |  |  |  |  |  |  |  |  |  |  |
| 0.2689 | 14 | igd | 0.0952 | C | \|\| id:ebi-a-GCST90001820 | ebi-a-GCST90001820 | TRUE | G | 105445412 | 1.38E-10 | reported | 3656 | 0.04177 | rs2819455 |  |  |  |  |  |  |  |  |  |  |  |  |  |  |  |
| -0.4067 | 10 | igd | 0.0952 | G | \|\| id:ebi-a-GCST90002030 | ebi-a-GCST90002030 | TRUE | A | 96946279 | 3.16E-18 | reported | 2920 | 0.04641 | rs142170794 |  |  |  |  |  |  |  |  |  |  |  |  |  |  |  |
| -0.3471 | 10 | igd | 0.0952 | G | \|\| id:ebi-a-GCST90002031 | ebi-a-GCST90002031 | TRUE | A | 96946279 | 4.63E-13 | reported | 2920 | 0.04775 | rs142170794 |  |  |  |  |  |  |  |  |  |  |  |  |  |  |  |
| -0.3511 | 10 | igd | 0.0952 | G | \|\| id:ebi-a-GCST90002032 | ebi-a-GCST90002032 | TRUE | A | 96946279 | 2.36E-13 | reported | 2920 | 0.0477 | rs142170794 |  |  |  |  |  |  |  |  |  |  |  |  |  |  |  |
| 0.2604 | 10 | igd | 0.0951 | C | \|\| id:ebi-a-GCST90001658 | ebi-a-GCST90001658 | TRUE | T | 98484769 | 1.44E-10 | reported | 3440 | 0.04049 | rs688972 |  |  |  |  |  |  |  |  |  |  |  |  |  |  |  |
| 0.2763 | 10 | igd | 0.0951 | C | \|\| id:ebi-a-GCST90001659 | ebi-a-GCST90001659 | TRUE | T | 98484769 | 1.03E-11 | reported | 3440 | 0.04047 | rs688972 |  |  |  |  |  |  |  |  |  |  |  |  |  |  |  |
| 0.354 | 1 | igd | 0.0951 | C | \|\| id:ebi-a-GCST90001981 | ebi-a-GCST90001981 | TRUE | T | 161477028 | 8.51E-17 | reported | 3618 | 0.04232 | rs4657040 |  |  |  |  |  |  |  |  |  |  |  |  |  |  |  |
| 0.3759 | 6 | igd | 0.0951 | A | \|\| id:ebi-a-GCST90002112 | ebi-a-GCST90002112 | TRUE | C | 33796411 | 7.46E-09 | reported | 1635 | 0.06469 | rs73747332 |  |  |  |  |  |  |  |  |  |  |  |  |  |  |  |
| -0.3397 | 1 | igd | 0.0951 | C | \|\| id:ebi-a-GCST90001997 | ebi-a-GCST90001997 | TRUE | T | 161477028 | 3.03E-15 | reported | 3590 | 0.04286 | rs4657040 |  |  |  |  |  |  |  |  |  |  |  |  |  |  |  |
| -0.3163 | 1 | igd | 0.0951 | C | \|\| id:ebi-a-GCST90001995 | ebi-a-GCST90001995 | TRUE | T | 161477028 | 1.45E-13 | reported | 3590 | 0.04263 | rs4657040 |  |  |  |  |  |  |  |  |  |  |  |  |  |  |  |
| 0.3462 | 10 | igd | 0.095 | C | \|\| id:ebi-a-GCST90001484 | ebi-a-GCST90001484 | TRUE | T | 98484769 | 4.51E-17 | reported | 3437 | 0.04101 | rs688972 |  |  |  |  |  |  |  |  |  |  |  |  |  |  |  |
| 0.3333 | 10 | igd | 0.095 | C | \|\| id:ebi-a-GCST90001485 | ebi-a-GCST90001485 | TRUE | T | 98484769 | 5.76E-16 | reported | 3437 | 0.04098 | rs688972 |  |  |  |  |  |  |  |  |  |  |  |  |  |  |  |
| 0.2537 | 10 | igd | 0.095 | C | \|\| id:ebi-a-GCST90001491 | ebi-a-GCST90001491 | TRUE | T | 98484769 | 1.67E-09 | reported | 3437 | 0.04197 | rs688972 |  |  |  |  |  |  |  |  |  |  |  |  |  |  |  |
| 0.3021 | 10 | igd | 0.095 | C | \|\| id:ebi-a-GCST90001496 | ebi-a-GCST90001496 | TRUE | T | 98484769 | 1.34E-12 | reported | 3437 | 0.04245 | rs688972 |  |  |  |  |  |  |  |  |  |  |  |  |  |  |  |
| 0.2655 | 10 | igd | 0.095 | C | \|\| id:ebi-a-GCST90001497 | ebi-a-GCST90001497 | TRUE | T | 98484769 | 2.88E-10 | reported | 3437 | 0.04198 | rs688972 |  |  |  |  |  |  |  |  |  |  |  |  |  |  |  |
| 0.4001 | 1 | igd | 0.095 | C | \|\| id:ebi-a-GCST90001989 | ebi-a-GCST90001989 | TRUE | T | 161477028 | 6.81E-21 | reported | 3621 | 0.04241 | rs4657040 |  |  |  |  |  |  |  |  |  |  |  |  |  |  |  |
| 0.3731 | 6 | igd | 0.095 | A | \|\| id:ebi-a-GCST90002108 | ebi-a-GCST90002108 | TRUE | T | 33302666 | 2.25E-08 | reported | 1579 | 0.06638 | rs6900824 |  |  |  |  |  |  |  |  |  |  |  |  |  |  |  |
| 0.3025 | 10 | igd | 0.0949 | C | \|\| id:ebi-a-GCST90001483 | ebi-a-GCST90001483 | TRUE | T | 98484769 | 3.72E-13 | reported | 3405 | 0.04147 | rs688972 |  |  |  |  |  |  |  |  |  |  |  |  |  |  |  |
| 0.2528 | 10 | igd | 0.0949 | C | \|\| id:ebi-a-GCST90001660 | ebi-a-GCST90001660 | TRUE | T | 98484769 | 3.06E-09 | reported | 3408 | 0.04253 | rs688972 |  |  |  |  |  |  |  |  |  |  |  |  |  |  |  |
| -0.2334 | 22 | igd | 0.0949 | T | \|\| id:ebi-a-GCST90001704 | ebi-a-GCST90001704 | TRUE | C | 44760501 | 3.85E-08 | reported | 3657 | 0.04236 | rs5765777 |  |  |  |  |  |  |  |  |  |  |  |  |  |  |  |
| -0.4376 | 12 | igd | 0.0949 | G | \|\| id:ebi-a-GCST90002073 | ebi-a-GCST90002073 | TRUE | T | 69303098 | 2.23E-20 | reported | 2850 | 0.04694 | rs61926289 |  |  |  |  |  |  |  |  |  |  |  |  |  |  |  |
| -0.4496 | 10 | igd | 0.0948 | A | \|\| id:ebi-a-GCST90001672 | ebi-a-GCST90001672 | TRUE | G | 97124665 | 1.26E-24 | reported | 3408 | 0.04355 | rs1556054 |  |  |  |  |  |  |  |  |  |  |  |  |  |  |  |
| 0.3787 | 1 | igd | 0.0948 | C | \|\| id:ebi-a-GCST90001980 | ebi-a-GCST90001980 | TRUE | T | 161477028 | 4.76E-19 | reported | 3629 | 0.04223 | rs4657040 |  |  |  |  |  |  |  |  |  |  |  |  |  |  |  |
| 0.4358 | 1 | igd | 0.0948 | C | \|\| id:ebi-a-GCST90001985 | ebi-a-GCST90001985 | TRUE | T | 161477028 | 1.74E-24 | reported | 3629 | 0.04236 | rs4657040 |  |  |  |  |  |  |  |  |  |  |  |  |  |  |  |
| -0.4095 | 10 | igd | 0.0947 | A | \|\| id:ebi-a-GCST90001491 | ebi-a-GCST90001491 | TRUE | G | 97124665 | 1.49E-21 | reported | 3437 | 0.04265 | rs1556054 |  |  |  |  |  |  |  |  |  |  |  |  |  |  |  |
| -0.4128 | 10 | igd | 0.0947 | A | \|\| id:ebi-a-GCST90001496 | ebi-a-GCST90001496 | TRUE | G | 97124665 | 2.41E-21 | reported | 3437 | 0.04323 | rs1556054 |  |  |  |  |  |  |  |  |  |  |  |  |  |  |  |
| -0.4178 | 10 | igd | 0.0947 | A | \|\| id:ebi-a-GCST90001497 | ebi-a-GCST90001497 | TRUE | G | 97124665 | 2.35E-22 | reported | 3437 | 0.04266 | rs1556054 |  |  |  |  |  |  |  |  |  |  |  |  |  |  |  |
| -0.2485 | 2 | igd | 0.0946 | A | \|\| id:ebi-a-GCST90001664 | ebi-a-GCST90001664 | TRUE | G | 39029499 | 8.84E-10 | reported | 3440 | 0.04043 | rs55722804 |  |  |  |  |  |  |  |  |  |  |  |  |  |  |  |
| -0.4302 | 10 | igd | 0.0946 | A | \|\| id:ebi-a-GCST90001670 | ebi-a-GCST90001670 | TRUE | G | 97124665 | 2.12E-23 | reported | 3440 | 0.04285 | rs1556054 |  |  |  |  |  |  |  |  |  |  |  |  |  |  |  |
| -0.4156 | 10 | igd | 0.0946 | A | \|\| id:ebi-a-GCST90001671 | ebi-a-GCST90001671 | TRUE | G | 97124665 | 7.91E-22 | reported | 3440 | 0.043 | rs1556054 |  |  |  |  |  |  |  |  |  |  |  |  |  |  |  |
| -0.2235 | 2 | igd | 0.0944 | A | \|\| id:ebi-a-GCST90001561 | ebi-a-GCST90001561 | TRUE | G | 39029499 | 2.16E-09 | reported | 3427 | 0.03725 | rs55722804 |  |  |  |  |  |  |  |  |  |  |  |  |  |  |  |
| -0.2476 | 20 | igd | 0.0944 | G | \|\| id:ebi-a-GCST90001806 | ebi-a-GCST90001806 | TRUE | C | 46242688 | 1.14E-08 | reported | 3656 | 0.04328 | rs72645230 |  |  |  |  |  |  |  |  |  |  |  |  |  |  |  |
| -0.5385 | 10 | igd | 0.0943 | A | \|\| id:ebi-a-GCST90002030 | ebi-a-GCST90002030 | TRUE | G | 97124665 | 2.76E-31 | reported | 2920 | 0.04575 | rs1556054 |  |  |  |  |  |  |  |  |  |  |  |  |  |  |  |
| -0.4669 | 10 | igd | 0.0943 | A | \|\| id:ebi-a-GCST90002031 | ebi-a-GCST90002031 | TRUE | G | 97124665 | 5.95E-23 | reported | 2920 | 0.04694 | rs1556054 |  |  |  |  |  |  |  |  |  |  |  |  |  |  |  |
| -0.4269 | 10 | igd | 0.0943 | A | \|\| id:ebi-a-GCST90002032 | ebi-a-GCST90002032 | TRUE | G | 97124665 | 2.09E-19 | reported | 2920 | 0.04706 | rs1556054 |  |  |  |  |  |  |  |  |  |  |  |  |  |  |  |
| 0.3791 | 6 | igd | 0.0942 | T | \|\| id:ebi-a-GCST90002112 | ebi-a-GCST90002112 | TRUE | G | 32228496 | 6.67E-09 | reported | 1635 | 0.06502 | rs145458341 |  |  |  |  |  |  |  |  |  |  |  |  |  |  |  |
| 0.4281 | 19 | igd | 0.094 | A | \|\| id:ebi-a-GCST90001948 | ebi-a-GCST90001948 | TRUE | G | 51785550 | 1.03E-11 | reported | 1633 | 0.06248 | rs1710386 |  |  |  |  |  |  |  |  |  |  |  |  |  |  |  |
| 0.3571 | 19 | igd | 0.0939 | A | \|\| id:ebi-a-GCST90001946 | ebi-a-GCST90001946 | TRUE | G | 51785550 | 1.76E-08 | reported | 1634 | 0.06307 | rs1710386 |  |  |  |  |  |  |  |  |  |  |  |  |  |  |  |
| 0.3644 | 19 | igd | 0.0939 | A | \|\| id:ebi-a-GCST90001949 | ebi-a-GCST90001949 | TRUE | G | 51785550 | 7.26E-09 | reported | 1634 | 0.06266 | rs1710386 |  |  |  |  |  |  |  |  |  |  |  |  |  |  |  |
| -0.3245 | 12 | igd | 0.0937 | G | \|\| id:ebi-a-GCST90002074 | ebi-a-GCST90002074 | TRUE | T | 69303098 | 8.40E-13 | reported | 3112 | 0.04516 | rs61926289 |  |  |  |  |  |  |  |  |  |  |  |  |  |  |  |
| -0.2787 | 2 | igd | 0.0935 | T | \|\| id:ebi-a-GCST90001894 | ebi-a-GCST90001894 | TRUE | C | 204795203 | 3.52E-09 | reported | 2920 | 0.04704 | rs60326917 |  |  |  |  |  |  |  |  |  |  |  |  |  |  |  |
| -0.2609 | 2 | igd | 0.0935 | T | \|\| id:ebi-a-GCST90001898 | ebi-a-GCST90001898 | TRUE | C | 204795203 | 3.21E-08 | reported | 2920 | 0.04705 | rs60326917 |  |  |  |  |  |  |  |  |  |  |  |  |  |  |  |
| -0.3101 | 2 | igd | 0.0935 | T | \|\| id:ebi-a-GCST90001900 | ebi-a-GCST90001900 | TRUE | C | 204795203 | 5.25E-11 | reported | 2919 | 0.04707 | rs60326917 |  |  |  |  |  |  |  |  |  |  |  |  |  |  |  |
| 0.5136 | 6 | igd | 0.0934 | G | \|\| id:ebi-a-GCST90002108 | ebi-a-GCST90002108 | TRUE | A | 32545612 | 3.30E-09 | reported | 1579 | 0.08632 | rs79001177 |  |  |  |  |  |  |  |  |  |  |  |  |  |  |  |
| 0.3003 | 2 | igd | 0.093 | A | \|\| id:ebi-a-GCST90001886 | ebi-a-GCST90001886 | TRUE | G | 204586515 | 5.87E-11 | reported | 2920 | 0.0457 | rs55686954 |  |  |  |  |  |  |  |  |  |  |  |  |  |  |  |
| -0.1685 | 15 | igd | 0.0929 | T | \|\| id:ebi-a-GCST90001690 | ebi-a-GCST90001690 | TRUE | C | 36456429 | 3.80E-08 | reported | 3408 | 0.03056 | rs12901644 |  |  |  |  |  |  |  |  |  |  |  |  |  |  |  |
| -0.2624 | 10 | igd | 0.0928 | A | \|\| id:ebi-a-GCST90001496 | ebi-a-GCST90001496 | TRUE | G | 96131474 | 6.48E-09 | reported | 3437 | 0.0451 | rs35469099 |  |  |  |  |  |  |  |  |  |  |  |  |  |  |  |
| 0.6038 | 6 | igd | 0.0927 | G | \|\| id:ebi-a-GCST90002110 | ebi-a-GCST90002110 | TRUE | A | 32545612 | 3.63E-12 | reported | 1634 | 0.08621 | rs79001177 |  |  |  |  |  |  |  |  |  |  |  |  |  |  |  |
| 0.481 | 6 | igd | 0.0927 | G | \|\| id:ebi-a-GCST90002111 | ebi-a-GCST90002111 | TRUE | A | 32545612 | 1.95E-08 | reported | 1635 | 0.08522 | rs79001177 |  |  |  |  |  |  |  |  |  |  |  |  |  |  |  |
| -0.299 | 22 | igd | 0.0926 | C | \|\| id:ebi-a-GCST90001702 | ebi-a-GCST90001702 | TRUE | G | 40539525 | 4.92E-12 | reported | 3657 | 0.04314 | rs12483853 |  |  |  |  |  |  |  |  |  |  |  |  |  |  |  |
| -0.307 | 22 | igd | 0.0926 | C | \|\| id:ebi-a-GCST90001703 | ebi-a-GCST90001703 | TRUE | G | 40539525 | 1.29E-12 | reported | 3657 | 0.04312 | rs12483853 |  |  |  |  |  |  |  |  |  |  |  |  |  |  |  |
| -0.3261 | 22 | igd | 0.0926 | C | \|\| id:ebi-a-GCST90001704 | ebi-a-GCST90001704 | TRUE | G | 40539525 | 4.36E-14 | reported | 3657 | 0.04303 | rs12483853 |  |  |  |  |  |  |  |  |  |  |  |  |  |  |  |
| -0.2897 | 22 | igd | 0.0926 | C | \|\| id:ebi-a-GCST90001710 | ebi-a-GCST90001710 | TRUE | G | 40539525 | 1.95E-11 | reported | 3657 | 0.04304 | rs12483853 |  |  |  |  |  |  |  |  |  |  |  |  |  |  |  |
| -0.285 | 22 | igd | 0.0926 | C | \|\| id:ebi-a-GCST90001711 | ebi-a-GCST90001711 | TRUE | G | 40539525 | 3.67E-11 | reported | 3657 | 0.04295 | rs12483853 |  |  |  |  |  |  |  |  |  |  |  |  |  |  |  |
| 0.2983 | 10 | igd | 0.0926 | C | \|\| id:ebi-a-GCST90002061 | ebi-a-GCST90002061 | TRUE | T | 95946164 | 2.86E-10 | reported | 2920 | 0.04714 | rs61886324 |  |  |  |  |  |  |  |  |  |  |  |  |  |  |  |
| -0.3515 | 1 | igd | 0.0925 | G | \|\| id:ebi-a-GCST90001858 | ebi-a-GCST90001858 | TRUE | A | 167171571 | 3.20E-14 | reported | 2919 | 0.04607 | rs12127503 |  |  |  |  |  |  |  |  |  |  |  |  |  |  |  |
| -0.2639 | 1 | igd | 0.0925 | G | \|\| id:ebi-a-GCST90001861 | ebi-a-GCST90001861 | TRUE | A | 167171571 | 9.13E-09 | reported | 2919 | 0.04579 | rs12127503 |  |  |  |  |  |  |  |  |  |  |  |  |  |  |  |
| -0.2717 | 1 | igd | 0.0925 | G | \|\| id:ebi-a-GCST90001863 | ebi-a-GCST90001863 | TRUE | A | 167171571 | 2.63E-09 | reported | 2919 | 0.0455 | rs12127503 |  |  |  |  |  |  |  |  |  |  |  |  |  |  |  |
| -0.3809 | 1 | igd | 0.0925 | G | \|\| id:ebi-a-GCST90001864 | ebi-a-GCST90001864 | TRUE | A | 167171571 | 1.75E-16 | reported | 2919 | 0.04596 | rs12127503 |  |  |  |  |  |  |  |  |  |  |  |  |  |  |  |
| -0.3152 | 6 | igd | 0.0925 | A | \|\| id:ebi-a-GCST90002119 | ebi-a-GCST90002119 | TRUE | G | 32068885 | 1.86E-09 | reported | 2920 | 0.05227 | rs113251500 |  |  |  |  |  |  |  |  |  |  |  |  |  |  |  |
| 0.285 | 6 | igd | 0.0923 | T | \|\| id:ebi-a-GCST90002105 | ebi-a-GCST90002105 | TRUE | C | 32785841 | 4.28E-08 | reported | 2872 | 0.05189 | rs73410764 |  |  |  |  |  |  |  |  |  |  |  |  |  |  |  |
| -0.2835 | 1 | igd | 0.0921 | G | \|\| id:ebi-a-GCST90001845 | ebi-a-GCST90001845 | TRUE | A | 167171571 | 1.06E-09 | reported | 2911 | 0.04633 | rs12127503 |  |  |  |  |  |  |  |  |  |  |  |  |  |  |  |
| -0.2904 | 6 | igd | 0.092 | T | \|\| id:ebi-a-GCST90001751 | ebi-a-GCST90001751 | TRUE | C | 15939080 | 7.67E-12 | reported | 3657 | 0.04229 | rs78737825 |  |  |  |  |  |  |  |  |  |  |  |  |  |  |  |
| -0.2621 | 6 | igd | 0.092 | T | \|\| id:ebi-a-GCST90001763 | ebi-a-GCST90001763 | TRUE | C | 15939080 | 1.02E-09 | reported | 3657 | 0.04281 | rs78737825 |  |  |  |  |  |  |  |  |  |  |  |  |  |  |  |
| -0.2869 | 1 | igd | 0.0918 | G | \|\| id:ebi-a-GCST90001854 | ebi-a-GCST90001854 | TRUE | A | 167170270 | 5.26E-10 | reported | 2919 | 0.04604 | rs16858712 |  |  |  |  |  |  |  |  |  |  |  |  |  |  |  |
| -0.2927 | 1 | igd | 0.0918 | G | \|\| id:ebi-a-GCST90001856 | ebi-a-GCST90001856 | TRUE | A | 167170270 | 3.37E-10 | reported | 2919 | 0.04643 | rs16858712 |  |  |  |  |  |  |  |  |  |  |  |  |  |  |  |
| -0.3525 | 1 | igd | 0.0918 | G | \|\| id:ebi-a-GCST90001860 | ebi-a-GCST90001860 | TRUE | A | 167170270 | 4.20E-14 | reported | 2919 | 0.04643 | rs16858712 |  |  |  |  |  |  |  |  |  |  |  |  |  |  |  |
| 0.2687 | 2 | igd | 0.0918 | C | \|\| id:ebi-a-GCST90001892 | ebi-a-GCST90001892 | TRUE | A | 204628795 | 2.18E-08 | reported | 2920 | 0.04788 | rs117701653 |  |  |  |  |  |  |  |  |  |  |  |  |  |  |  |
| -0.271 | 2 | igd | 0.0918 | T | \|\| id:ebi-a-GCST90002098 | ebi-a-GCST90002098 | TRUE | C | 39375730 | 1.54E-09 | reported | 2910 | 0.04472 | rs146350898 |  |  |  |  |  |  |  |  |  |  |  |  |  |  |  |
| -0.2878 | 11 | igd | 0.0917 | C | \|\| id:ebi-a-GCST90001745 | ebi-a-GCST90001745 | TRUE | T | 60166613 | 1.03E-11 | reported | 3657 | 0.04216 | rs74621542 |  |  |  |  |  |  |  |  |  |  |  |  |  |  |  |
| 0.2619 | 10 | igd | 0.0916 | A | \|\| id:ebi-a-GCST90001659 | ebi-a-GCST90001659 | TRUE | G | 95950828 | 6.46E-10 | reported | 3440 | 0.04227 | rs35173192 |  |  |  |  |  |  |  |  |  |  |  |  |  |  |  |
| 0.4484 | 1 | igd | 0.0915 | C | \|\| id:ebi-a-GCST90001994 | ebi-a-GCST90001994 | TRUE | T | 161612233 | 1.15E-25 | reported | 3590 | 0.04249 | rs7554873 |  |  |  |  |  |  |  |  |  |  |  |  |  |  |  |
| 0.2692 | 10 | igd | 0.0914 | T | \|\| id:ebi-a-GCST90002030 | ebi-a-GCST90002030 | TRUE | C | 97064619 | 1.55E-08 | reported | 2920 | 0.04747 | rs77251666 |  |  |  |  |  |  |  |  |  |  |  |  |  |  |  |
| 0.3063 | 10 | igd | 0.0913 | A | \|\| id:ebi-a-GCST90001672 | ebi-a-GCST90001672 | TRUE | G | 97166923 | 1.90E-11 | reported | 3408 | 0.04547 | rs7068867 |  |  |  |  |  |  |  |  |  |  |  |  |  |  |  |
| 0.378 | 6 | igd | 0.0912 | T | \|\| id:ebi-a-GCST90002108 | ebi-a-GCST90002108 | TRUE | C | 32785841 | 1.75E-08 | reported | 1579 | 0.06674 | rs73410764 |  |  |  |  |  |  |  |  |  |  |  |  |  |  |  |
| 0.2552 | 10 | igd | 0.0911 | A | \|\| id:ebi-a-GCST90001660 | ebi-a-GCST90001660 | TRUE | G | 95950828 | 1.01E-08 | reported | 3408 | 0.04443 | rs35173192 |  |  |  |  |  |  |  |  |  |  |  |  |  |  |  |
| 0.471 | 2 | igd | 0.0911 | T | \|\| id:ebi-a-GCST90002098 | ebi-a-GCST90002098 | TRUE | G | 39020877 | 3.08E-25 | reported | 2910 | 0.04496 | rs116758203 |  |  |  |  |  |  |  |  |  |  |  |  |  |  |  |
| 0.2827 | 10 | igd | 0.0909 | C | \|\| id:ebi-a-GCST90001892 | ebi-a-GCST90001892 | TRUE | G | 97856158 | 1.86E-09 | reported | 2920 | 0.04689 | rs11188600 |  |  |  |  |  |  |  |  |  |  |  |  |  |  |  |
| 0.5696 | 10 | igd | 0.0909 | C | \|\| id:ebi-a-GCST90002030 | ebi-a-GCST90002030 | TRUE | G | 97856158 | 5.31E-34 | reported | 2920 | 0.04626 | rs11188600 |  |  |  |  |  |  |  |  |  |  |  |  |  |  |  |
| 0.4607 | 10 | igd | 0.0909 | C | \|\| id:ebi-a-GCST90002031 | ebi-a-GCST90002031 | TRUE | G | 97856158 | 6.72E-22 | reported | 2920 | 0.04752 | rs11188600 |  |  |  |  |  |  |  |  |  |  |  |  |  |  |  |
| 0.5039 | 10 | igd | 0.0909 | C | \|\| id:ebi-a-GCST90002032 | ebi-a-GCST90002032 | TRUE | G | 97856158 | 5.97E-26 | reported | 2920 | 0.04737 | rs11188600 |  |  |  |  |  |  |  |  |  |  |  |  |  |  |  |
| 0.284 | 10 | igd | 0.0907 | T | \|\| id:ebi-a-GCST90001659 | ebi-a-GCST90001659 | TRUE | C | 97165619 | 4.52E-11 | reported | 3440 | 0.04298 | rs17849168 |  |  |  |  |  |  |  |  |  |  |  |  |  |  |  |
| 0.2938 | 10 | igd | 0.0907 | A | \|\| id:ebi-a-GCST90001670 | ebi-a-GCST90001670 | TRUE | G | 97166923 | 5.96E-11 | reported | 3440 | 0.04474 | rs7068867 |  |  |  |  |  |  |  |  |  |  |  |  |  |  |  |
| 0.2891 | 10 | igd | 0.0907 | T | \|\| id:ebi-a-GCST90001671 | ebi-a-GCST90001671 | TRUE | C | 97165619 | 1.25E-10 | reported | 3440 | 0.04481 | rs17849168 |  |  |  |  |  |  |  |  |  |  |  |  |  |  |  |
| 0.2687 | 10 | igd | 0.0906 | T | \|\| id:ebi-a-GCST90001496 | ebi-a-GCST90001496 | TRUE | C | 97165619 | 2.94E-09 | reported | 3437 | 0.04516 | rs17849168 |  |  |  |  |  |  |  |  |  |  |  |  |  |  |  |
| 0.2678 | 10 | igd | 0.0906 | T | \|\| id:ebi-a-GCST90001497 | ebi-a-GCST90001497 | TRUE | C | 97165619 | 2.11E-09 | reported | 3437 | 0.04459 | rs17849168 |  |  |  |  |  |  |  |  |  |  |  |  |  |  |  |
| -0.4329 | 6 | igd | 0.0904 | C | \|\| id:ebi-a-GCST90001752 | ebi-a-GCST90001752 | TRUE | T | 32625820 | 1.78E-08 | reported | 3657 | 0.0767 | rs143133357 |  |  |  |  |  |  |  |  |  |  |  |  |  |  |  |
| -0.3839 | 19 | igd | 0.0902 | C | \|\| id:ebi-a-GCST90001951 | ebi-a-GCST90001951 | TRUE | T | 52094863 | 1.18E-08 | reported | 1464 | 0.06692 | rs11883251 |  |  |  |  |  |  |  |  |  |  |  |  |  |  |  |
| 0.3498 | 19 | igd | 0.0902 | T | \|\| id:ebi-a-GCST90001952 | ebi-a-GCST90001952 | TRUE | C | 51709137 | 3.77E-08 | reported | 1569 | 0.06327 | rs77396601 |  |  |  |  |  |  |  |  |  |  |  |  |  |  |  |
| 0.2434 | 10 | igd | 0.0899 | A | \|\| id:ebi-a-GCST90001484 | ebi-a-GCST90001484 | TRUE | C | 107757380 | 1.98E-08 | reported | 3437 | 0.04326 | rs4293064 |  |  |  |  |  |  |  |  |  |  |  |  |  |  |  |
| 0.2497 | 2 | igd | 0.0896 | T | \|\| id:ebi-a-GCST90001561 | ebi-a-GCST90001561 | TRUE | G | 39020877 | 5.02E-11 | reported | 3427 | 0.03789 | rs116758203 |  |  |  |  |  |  |  |  |  |  |  |  |  |  |  |
| -3.204 | 2 | igd | 0.0896 | T | \|\| id:ebi-a-GCST90001696 | ebi-a-GCST90001696 | TRUE | G | 39020877 | 2.09E-09 | reported | 3425 | 0.5333 | rs116758203 |  |  |  |  |  |  |  |  |  |  |  |  |  |  |  |
| 0.2403 | 20 | igd | 0.0896 | C | \|\| id:ebi-a-GCST90001989 | ebi-a-GCST90001989 | TRUE | G | 42185211 | 4.60E-08 | reported | 3621 | 0.04386 | rs77508942 |  |  |  |  |  |  |  |  |  |  |  |  |  |  |  |
| -0.2843 | 1 | igd | 0.0894 | A | \|\| id:ebi-a-GCST90001861 | ebi-a-GCST90001861 | TRUE | T | 167435660 | 2.21E-09 | reported | 2919 | 0.04738 | rs12063588 |  |  |  |  |  |  |  |  |  |  |  |  |  |  |  |
| 0.4255 | 10 | igd | 0.0893 | C | \|\| id:ebi-a-GCST90001484 | ebi-a-GCST90001484 | TRUE | G | 97856158 | 1.33E-22 | reported | 3437 | 0.04319 | rs11188600 |  |  |  |  |  |  |  |  |  |  |  |  |  |  |  |
| 0.4071 | 10 | igd | 0.0893 | C | \|\| id:ebi-a-GCST90001485 | ebi-a-GCST90001485 | TRUE | G | 97856158 | 7.35E-21 | reported | 3437 | 0.04317 | rs11188600 |  |  |  |  |  |  |  |  |  |  |  |  |  |  |  |
| 0.4651 | 10 | igd | 0.0893 | C | \|\| id:ebi-a-GCST90001491 | ebi-a-GCST90001491 | TRUE | G | 97856158 | 1.36E-25 | reported | 3437 | 0.04412 | rs11188600 |  |  |  |  |  |  |  |  |  |  |  |  |  |  |  |
| 0.438 | 10 | igd | 0.0893 | C | \|\| id:ebi-a-GCST90001496 | ebi-a-GCST90001496 | TRUE | G | 97856158 | 2.84E-22 | reported | 3437 | 0.04481 | rs11188600 |  |  |  |  |  |  |  |  |  |  |  |  |  |  |  |
| 0.419 | 10 | igd | 0.0893 | C | \|\| id:ebi-a-GCST90001497 | ebi-a-GCST90001497 | TRUE | G | 97856158 | 5.43E-21 | reported | 3437 | 0.04428 | rs11188600 |  |  |  |  |  |  |  |  |  |  |  |  |  |  |  |
| 0.4204 | 10 | igd | 0.0892 | C | \|\| id:ebi-a-GCST90001658 | ebi-a-GCST90001658 | TRUE | G | 97856158 | 1.64E-22 | reported | 3440 | 0.04277 | rs11188600 |  |  |  |  |  |  |  |  |  |  |  |  |  |  |  |
| 0.4199 | 10 | igd | 0.0892 | C | \|\| id:ebi-a-GCST90001659 | ebi-a-GCST90001659 | TRUE | G | 97856158 | 1.89E-22 | reported | 3440 | 0.04277 | rs11188600 |  |  |  |  |  |  |  |  |  |  |  |  |  |  |  |
| 0.3517 | 10 | igd | 0.0892 | C | \|\| id:ebi-a-GCST90001670 | ebi-a-GCST90001670 | TRUE | G | 97856158 | 4.58E-15 | reported | 3440 | 0.04467 | rs11188600 |  |  |  |  |  |  |  |  |  |  |  |  |  |  |  |
| 0.354 | 10 | igd | 0.0892 | C | \|\| id:ebi-a-GCST90001671 | ebi-a-GCST90001671 | TRUE | G | 97856158 | 3.65E-15 | reported | 3440 | 0.04479 | rs11188600 |  |  |  |  |  |  |  |  |  |  |  |  |  |  |  |
| -0.3033 | 10 | igd | 0.0892 | A | \|\| id:ebi-a-GCST90002031 | ebi-a-GCST90002031 | TRUE | G | 96674834 | 4.17E-10 | reported | 2920 | 0.04838 | rs118030161 |  |  |  |  |  |  |  |  |  |  |  |  |  |  |  |
| -0.3815 | 10 | igd | 0.0892 | A | \|\| id:ebi-a-GCST90002032 | ebi-a-GCST90002032 | TRUE | G | 96674834 | 3.27E-15 | reported | 2920 | 0.04815 | rs118030161 |  |  |  |  |  |  |  |  |  |  |  |  |  |  |  |
| 0.3877 | 10 | igd | 0.0888 | C | \|\| id:ebi-a-GCST90001483 | ebi-a-GCST90001483 | TRUE | G | 97856158 | 1.67E-18 | reported | 3405 | 0.04391 | rs11188600 |  |  |  |  |  |  |  |  |  |  |  |  |  |  |  |
| 0.4264 | 10 | igd | 0.0888 | C | \|\| id:ebi-a-GCST90001489 | ebi-a-GCST90001489 | TRUE | G | 97856158 | 5.21E-21 | reported | 3405 | 0.04504 | rs11188600 |  |  |  |  |  |  |  |  |  |  |  |  |  |  |  |
| 0.3961 | 10 | igd | 0.0888 | C | \|\| id:ebi-a-GCST90001495 | ebi-a-GCST90001495 | TRUE | G | 97856158 | 6.23E-18 | reported | 3405 | 0.04565 | rs11188600 |  |  |  |  |  |  |  |  |  |  |  |  |  |  |  |
| 0.387 | 10 | igd | 0.0888 | C | \|\| id:ebi-a-GCST90001660 | ebi-a-GCST90001660 | TRUE | G | 97856158 | 1.66E-17 | reported | 3408 | 0.04521 | rs11188600 |  |  |  |  |  |  |  |  |  |  |  |  |  |  |  |
| 0.3399 | 10 | igd | 0.0888 | C | \|\| id:ebi-a-GCST90001672 | ebi-a-GCST90001672 | TRUE | G | 97856158 | 1.40E-13 | reported | 3408 | 0.04577 | rs11188600 |  |  |  |  |  |  |  |  |  |  |  |  |  |  |  |
| -0.2765 | 19 | igd | 0.0888 | T | \|\| id:ebi-a-GCST90002084 | ebi-a-GCST90002084 | TRUE | C | 8789227 | 3.60E-09 | reported | 3113 | 0.04672 | rs10422871 |  |  |  |  |  |  |  |  |  |  |  |  |  |  |  |
| 0.2522 | 19 | igd | 0.0887 | A | \|\| id:ebi-a-GCST90001757 | ebi-a-GCST90001757 | TRUE | G | 50957677 | 1.33E-08 | reported | 3657 | 0.04429 | rs112268715 |  |  |  |  |  |  |  |  |  |  |  |  |  |  |  |
| 0.2667 | 10 | igd | 0.0885 | T | \|\| id:ebi-a-GCST90001670 | ebi-a-GCST90001670 | TRUE | C | 101633310 | 2.48E-09 | reported | 3440 | 0.0446 | rs10509739 |  |  |  |  |  |  |  |  |  |  |  |  |  |  |  |
| 0.2534 | 10 | igd | 0.0885 | T | \|\| id:ebi-a-GCST90001671 | ebi-a-GCST90001671 | TRUE | C | 101633310 | 1.56E-08 | reported | 3440 | 0.0447 | rs10509739 |  |  |  |  |  |  |  |  |  |  |  |  |  |  |  |
| 0.3004 | 10 | igd | 0.0885 | T | \|\| id:ebi-a-GCST90001672 | ebi-a-GCST90001672 | TRUE | C | 101633310 | 4.01E-11 | reported | 3408 | 0.04534 | rs10509739 |  |  |  |  |  |  |  |  |  |  |  |  |  |  |  |
| -0.4564 | 19 | igd | 0.0882 | C | \|\| id:ebi-a-GCST90001948 | ebi-a-GCST90001948 | TRUE | T | 52094863 | 7.38E-13 | reported | 1633 | 0.06313 | rs11883251 |  |  |  |  |  |  |  |  |  |  |  |  |  |  |  |
| -0.4314 | 19 | igd | 0.0881 | C | \|\| id:ebi-a-GCST90001946 | ebi-a-GCST90001946 | TRUE | T | 52094863 | 1.38E-11 | reported | 1634 | 0.06337 | rs11883251 |  |  |  |  |  |  |  |  |  |  |  |  |  |  |  |
| -0.4519 | 19 | igd | 0.0881 | C | \|\| id:ebi-a-GCST90001949 | ebi-a-GCST90001949 | TRUE | T | 52094863 | 1.07E-12 | reported | 1634 | 0.06295 | rs11883251 |  |  |  |  |  |  |  |  |  |  |  |  |  |  |  |
| -0.2856 | 10 | igd | 0.088 | T | \|\| id:ebi-a-GCST90001961 | ebi-a-GCST90001961 | TRUE | C | 97165619 | 4.89E-09 | reported | 2920 | 0.04867 | rs17849168 |  |  |  |  |  |  |  |  |  |  |  |  |  |  |  |
| 0.2832 | 22 | igd | 0.0879 | C | \|\| id:ebi-a-GCST90001702 | ebi-a-GCST90001702 | TRUE | T | 41769083 | 1.02E-10 | reported | 3657 | 0.04367 | rs11090046 |  |  |  |  |  |  |  |  |  |  |  |  |  |  |  |
| 0.2676 | 22 | igd | 0.0879 | C | \|\| id:ebi-a-GCST90001703 | ebi-a-GCST90001703 | TRUE | T | 41769083 | 1.01E-09 | reported | 3657 | 0.0437 | rs11090046 |  |  |  |  |  |  |  |  |  |  |  |  |  |  |  |
| 0.3032 | 22 | igd | 0.0879 | C | \|\| id:ebi-a-GCST90001710 | ebi-a-GCST90001710 | TRUE | T | 41769083 | 3.81E-12 | reported | 3657 | 0.04351 | rs11090046 |  |  |  |  |  |  |  |  |  |  |  |  |  |  |  |
| 0.2835 | 22 | igd | 0.0879 | C | \|\| id:ebi-a-GCST90001711 | ebi-a-GCST90001711 | TRUE | T | 41769083 | 7.80E-11 | reported | 3657 | 0.04345 | rs11090046 |  |  |  |  |  |  |  |  |  |  |  |  |  |  |  |
| 0.3257 | 22 | igd | 0.0872 | A | \|\| id:ebi-a-GCST90001702 | ebi-a-GCST90001702 | TRUE | G | 42682786 | 1.02E-12 | reported | 3657 | 0.04554 | rs4822100 |  |  |  |  |  |  |  |  |  |  |  |  |  |  |  |
| 0.3395 | 22 | igd | 0.0872 | A | \|\| id:ebi-a-GCST90001703 | ebi-a-GCST90001703 | TRUE | G | 42682786 | 1.05E-13 | reported | 3657 | 0.04549 | rs4822100 |  |  |  |  |  |  |  |  |  |  |  |  |  |  |  |
| 0.3163 | 22 | igd | 0.0872 | A | \|\| id:ebi-a-GCST90001704 | ebi-a-GCST90001704 | TRUE | G | 42682786 | 4.12E-12 | reported | 3657 | 0.04547 | rs4822100 |  |  |  |  |  |  |  |  |  |  |  |  |  |  |  |
| 0.2983 | 22 | igd | 0.0872 | A | \|\| id:ebi-a-GCST90001710 | ebi-a-GCST90001710 | TRUE | G | 42682786 | 6.14E-11 | reported | 3657 | 0.04547 | rs4822100 |  |  |  |  |  |  |  |  |  |  |  |  |  |  |  |
| 0.3206 | 6 | igd | 0.0872 | C | \|\| id:ebi-a-GCST90002105 | ebi-a-GCST90002105 | TRUE | A | 32332661 | 1.76E-09 | reported | 2872 | 0.0531 | rs34673422 |  |  |  |  |  |  |  |  |  |  |  |  |  |  |  |
| -0.2783 | 10 | igd | 0.0871 | A | \|\| id:ebi-a-GCST90001489 | ebi-a-GCST90001489 | TRUE | G | 99955651 | 5.80E-10 | reported | 3405 | 0.04479 | rs117069940 |  |  |  |  |  |  |  |  |  |  |  |  |  |  |  |
| -0.2478 | 10 | igd | 0.0871 | A | \|\| id:ebi-a-GCST90001660 | ebi-a-GCST90001660 | TRUE | G | 99955651 | 3.02E-08 | reported | 3408 | 0.04463 | rs117069940 |  |  |  |  |  |  |  |  |  |  |  |  |  |  |  |
| -0.2537 | 10 | igd | 0.0871 | A | \|\| id:ebi-a-GCST90001672 | ebi-a-GCST90001672 | TRUE | G | 99955651 | 1.94E-08 | reported | 3408 | 0.04506 | rs117069940 |  |  |  |  |  |  |  |  |  |  |  |  |  |  |  |
| 0.3917 | 10 | igd | 0.087 | G | \|\| id:ebi-a-GCST90001660 | ebi-a-GCST90001660 | TRUE | A | 96902688 | 5.91E-18 | reported | 3408 | 0.04512 | rs10882550 |  |  |  |  |  |  |  |  |  |  |  |  |  |  |  |
| 0.3863 | 10 | igd | 0.087 | G | \|\| id:ebi-a-GCST90001672 | ebi-a-GCST90001672 | TRUE | A | 96902688 | 3.45E-17 | reported | 3408 | 0.04558 | rs10882550 |  |  |  |  |  |  |  |  |  |  |  |  |  |  |  |
| -0.5949 | 22 | igd | 0.087 | T | \|\| id:ebi-a-GCST90001710 | ebi-a-GCST90001710 | TRUE | G | 42566101 | 1.63E-39 | reported | 3657 | 0.04469 | rs5751233 |  |  |  |  |  |  |  |  |  |  |  |  |  |  |  |
| 0.3566 | 10 | igd | 0.087 | C | \|\| id:ebi-a-GCST90002029 | ebi-a-GCST90002029 | TRUE | G | 97497130 | 9.11E-14 | reported | 2915 | 0.04761 | rs7478275 |  |  |  |  |  |  |  |  |  |  |  |  |  |  |  |
| 0.2505 | 10 | igd | 0.0869 | C | \|\| id:ebi-a-GCST90001483 | ebi-a-GCST90001483 | TRUE | A | 96857031 | 1.91E-08 | reported | 3405 | 0.04446 | rs11188180 |  |  |  |  |  |  |  |  |  |  |  |  |  |  |  |
| 0.3448 | 10 | igd | 0.0869 | G | \|\| id:ebi-a-GCST90001495 | ebi-a-GCST90001495 | TRUE | A | 96902688 | 6.53E-14 | reported | 3405 | 0.04579 | rs10882550 |  |  |  |  |  |  |  |  |  |  |  |  |  |  |  |
| 0.3638 | 10 | igd | 0.0869 | G | \|\| id:ebi-a-GCST90001658 | ebi-a-GCST90001658 | TRUE | A | 96902688 | 4.38E-17 | reported | 3440 | 0.04307 | rs10882550 |  |  |  |  |  |  |  |  |  |  |  |  |  |  |  |
| 0.375 | 10 | igd | 0.0869 | G | \|\| id:ebi-a-GCST90001659 | ebi-a-GCST90001659 | TRUE | A | 96902688 | 4.61E-18 | reported | 3440 | 0.04305 | rs10882550 |  |  |  |  |  |  |  |  |  |  |  |  |  |  |  |
| 0.3691 | 10 | igd | 0.0869 | G | \|\| id:ebi-a-GCST90001670 | ebi-a-GCST90001670 | TRUE | A | 96902688 | 2.53E-16 | reported | 3440 | 0.04483 | rs10882550 |  |  |  |  |  |  |  |  |  |  |  |  |  |  |  |
| -0.2468 | 10 | igd | 0.0869 | A | \|\| id:ebi-a-GCST90001670 | ebi-a-GCST90001670 | TRUE | G | 99955651 | 2.78E-08 | reported | 3440 | 0.04432 | rs117069940 |  |  |  |  |  |  |  |  |  |  |  |  |  |  |  |
| 0.3796 | 10 | igd | 0.0869 | G | \|\| id:ebi-a-GCST90001671 | ebi-a-GCST90001671 | TRUE | A | 96902688 | 4.07E-17 | reported | 3440 | 0.0449 | rs10882550 |  |  |  |  |  |  |  |  |  |  |  |  |  |  |  |
| 0.2771 | 10 | igd | 0.0868 | C | \|\| id:ebi-a-GCST90001484 | ebi-a-GCST90001484 | TRUE | A | 96857031 | 3.53E-10 | reported | 3437 | 0.04404 | rs11188180 |  |  |  |  |  |  |  |  |  |  |  |  |  |  |  |
| 0.2669 | 10 | igd | 0.0868 | C | \|\| id:ebi-a-GCST90001485 | ebi-a-GCST90001485 | TRUE | A | 96857031 | 1.46E-09 | reported | 3437 | 0.044 | rs11188180 |  |  |  |  |  |  |  |  |  |  |  |  |  |  |  |
| 0.2476 | 10 | igd | 0.0868 | T | \|\| id:ebi-a-GCST90001485 | ebi-a-GCST90001485 | TRUE | C | 107757069 | 3.07E-08 | reported | 3437 | 0.04461 | rs35103389 |  |  |  |  |  |  |  |  |  |  |  |  |  |  |  |
| 0.2901 | 10 | igd | 0.0868 | C | \|\| id:ebi-a-GCST90001489 | ebi-a-GCST90001489 | TRUE | T | 96864355 | 2.25E-10 | reported | 3405 | 0.0456 | rs11188187 |  |  |  |  |  |  |  |  |  |  |  |  |  |  |  |
| -0.2775 | 10 | igd | 0.0868 | A | \|\| id:ebi-a-GCST90001491 | ebi-a-GCST90001491 | TRUE | G | 99955651 | 3.26E-10 | reported | 3437 | 0.04401 | rs117069940 |  |  |  |  |  |  |  |  |  |  |  |  |  |  |  |
| 0.3175 | 10 | igd | 0.0868 | C | \|\| id:ebi-a-GCST90001491 | ebi-a-GCST90001491 | TRUE | A | 96857031 | 1.96E-12 | reported | 3437 | 0.04494 | rs11188180 |  |  |  |  |  |  |  |  |  |  |  |  |  |  |  |
| 0.3905 | 10 | igd | 0.0868 | G | \|\| id:ebi-a-GCST90001496 | ebi-a-GCST90001496 | TRUE | A | 96902688 | 8.53E-18 | reported | 3437 | 0.04519 | rs10882550 |  |  |  |  |  |  |  |  |  |  |  |  |  |  |  |
| 0.3881 | 10 | igd | 0.0868 | G | \|\| id:ebi-a-GCST90001497 | ebi-a-GCST90001497 | TRUE | A | 96902688 | 5.17E-18 | reported | 3437 | 0.04462 | rs10882550 |  |  |  |  |  |  |  |  |  |  |  |  |  |  |  |
| 0.3303 | 22 | igd | 0.0868 | T | \|\| id:ebi-a-GCST90001702 | ebi-a-GCST90001702 | TRUE | C | 42581248 | 5.07E-13 | reported | 3657 | 0.04556 | rs2142695 |  |  |  |  |  |  |  |  |  |  |  |  |  |  |  |
| 0.342 | 22 | igd | 0.0868 | T | \|\| id:ebi-a-GCST90001703 | ebi-a-GCST90001703 | TRUE | C | 42581248 | 7.25E-14 | reported | 3657 | 0.04552 | rs2142695 |  |  |  |  |  |  |  |  |  |  |  |  |  |  |  |
| 0.3214 | 22 | igd | 0.0868 | T | \|\| id:ebi-a-GCST90001704 | ebi-a-GCST90001704 | TRUE | C | 42581248 | 1.92E-12 | reported | 3657 | 0.0455 | rs2142695 |  |  |  |  |  |  |  |  |  |  |  |  |  |  |  |
| 0.3025 | 22 | igd | 0.0868 | T | \|\| id:ebi-a-GCST90001710 | ebi-a-GCST90001710 | TRUE | C | 42581248 | 3.39E-11 | reported | 3657 | 0.0455 | rs2142695 |  |  |  |  |  |  |  |  |  |  |  |  |  |  |  |
| 0.3014 | 10 | igd | 0.0868 | C | \|\| id:ebi-a-GCST90001892 | ebi-a-GCST90001892 | TRUE | G | 97497130 | 5.25E-10 | reported | 2920 | 0.04836 | rs7478275 |  |  |  |  |  |  |  |  |  |  |  |  |  |  |  |
| -0.3428 | 10 | igd | 0.0868 | A | \|\| id:ebi-a-GCST90001961 | ebi-a-GCST90001961 | TRUE | G | 96892257 | 2.12E-12 | reported | 2920 | 0.04858 | rs12355731 |  |  |  |  |  |  |  |  |  |  |  |  |  |  |  |
| -0.3687 | 10 | igd | 0.0868 | C | \|\| id:ebi-a-GCST90001961 | ebi-a-GCST90001961 | TRUE | G | 97497130 | 4.95E-14 | reported | 2920 | 0.0487 | rs7478275 |  |  |  |  |  |  |  |  |  |  |  |  |  |  |  |
| 0.7674 | 10 | igd | 0.0868 | C | \|\| id:ebi-a-GCST90002030 | ebi-a-GCST90002030 | TRUE | G | 97497130 | 2.71E-58 | reported | 2920 | 0.04661 | rs7478275 |  |  |  |  |  |  |  |  |  |  |  |  |  |  |  |
| 0.6857 | 10 | igd | 0.0868 | C | \|\| id:ebi-a-GCST90002031 | ebi-a-GCST90002031 | TRUE | G | 97497130 | 1.39E-44 | reported | 2920 | 0.04812 | rs7478275 |  |  |  |  |  |  |  |  |  |  |  |  |  |  |  |
| 0.7423 | 10 | igd | 0.0868 | C | \|\| id:ebi-a-GCST90002032 | ebi-a-GCST90002032 | TRUE | G | 97497130 | 2.81E-52 | reported | 2920 | 0.04782 | rs7478275 |  |  |  |  |  |  |  |  |  |  |  |  |  |  |  |
| -0.2664 | 4 | igd | 0.0867 | G | \|\| id:ebi-a-GCST90001471 | ebi-a-GCST90001471 | TRUE | A | 82502357 | 1.72E-09 | reported | 3410 | 0.04411 | rs191779135 |  |  |  |  |  |  |  |  |  |  |  |  |  |  |  |
| -0.2314 | 2 | igd | 0.0866 | G | \|\| id:ebi-a-GCST90001664 | ebi-a-GCST90001664 | TRUE | A | 38925610 | 3.37E-08 | reported | 3440 | 0.04182 | rs12465156 |  |  |  |  |  |  |  |  |  |  |  |  |  |  |  |
| -0.2628 | 4 | igd | 0.0865 | G | \|\| id:ebi-a-GCST90001470 | ebi-a-GCST90001470 | TRUE | A | 82502357 | 1.38E-09 | reported | 3374 | 0.04325 | rs191779135 |  |  |  |  |  |  |  |  |  |  |  |  |  |  |  |
| -0.6124 | 22 | igd | 0.0865 | T | \|\| id:ebi-a-GCST90001710 | ebi-a-GCST90001710 | TRUE | G | 42448761 | 2.72E-41 | reported | 3657 | 0.04494 | rs5758563 |  |  |  |  |  |  |  |  |  |  |  |  |  |  |  |
| 0.2696 | 18 | igd | 0.0864 | T | \|\| id:ebi-a-GCST90001681 | ebi-a-GCST90001681 | TRUE | C | 30263492 | 8.82E-09 | reported | 3130 | 0.04674 | rs1403755 |  |  |  |  |  |  |  |  |  |  |  |  |  |  |  |
| -0.2975 | 17 | igd | 0.0863 | T | \|\| id:ebi-a-GCST90001926 | ebi-a-GCST90001926 | TRUE | A | 56227116 | 1.06E-09 | reported | 2920 | 0.04862 | rs36089182 |  |  |  |  |  |  |  |  |  |  |  |  |  |  |  |
| 0.4008 | 19 | igd | 0.0863 | T | \|\| id:ebi-a-GCST90001946 | ebi-a-GCST90001946 | TRUE | C | 51715345 | 1.56E-10 | reported | 1634 | 0.06222 | rs61730558 |  |  |  |  |  |  |  |  |  |  |  |  |  |  |  |
| 0.4064 | 19 | igd | 0.0863 | G | \|\| id:ebi-a-GCST90001948 | ebi-a-GCST90001948 | TRUE | T | 51716784 | 5.48E-11 | reported | 1633 | 0.06156 | rs78901355 |  |  |  |  |  |  |  |  |  |  |  |  |  |  |  |
| 0.4006 | 19 | igd | 0.0863 | G | \|\| id:ebi-a-GCST90001949 | ebi-a-GCST90001949 | TRUE | T | 51716784 | 9.51E-11 | reported | 1634 | 0.06147 | rs78901355 |  |  |  |  |  |  |  |  |  |  |  |  |  |  |  |
| 0.5235 | 10 | igd | 0.0857 | C | \|\| id:ebi-a-GCST90001484 | ebi-a-GCST90001484 | TRUE | G | 97497130 | 1.29E-32 | reported | 3437 | 0.04357 | rs7478275 |  |  |  |  |  |  |  |  |  |  |  |  |  |  |  |
| 0.4993 | 10 | igd | 0.0857 | C | \|\| id:ebi-a-GCST90001485 | ebi-a-GCST90001485 | TRUE | G | 97497130 | 7.51E-30 | reported | 3437 | 0.04358 | rs7478275 |  |  |  |  |  |  |  |  |  |  |  |  |  |  |  |
| 0.5597 | 10 | igd | 0.0857 | C | \|\| id:ebi-a-GCST90001491 | ebi-a-GCST90001491 | TRUE | G | 97497130 | 1.17E-35 | reported | 3437 | 0.0444 | rs7478275 |  |  |  |  |  |  |  |  |  |  |  |  |  |  |  |
| 0.6542 | 10 | igd | 0.0857 | C | \|\| id:ebi-a-GCST90001496 | ebi-a-GCST90001496 | TRUE | G | 97497130 | 3.33E-47 | reported | 3437 | 0.04465 | rs7478275 |  |  |  |  |  |  |  |  |  |  |  |  |  |  |  |
| 0.6319 | 10 | igd | 0.0857 | C | \|\| id:ebi-a-GCST90001497 | ebi-a-GCST90001497 | TRUE | G | 97497130 | 3.47E-45 | reported | 3437 | 0.04415 | rs7478275 |  |  |  |  |  |  |  |  |  |  |  |  |  |  |  |
| 0.585 | 10 | igd | 0.0856 | C | \|\| id:ebi-a-GCST90001658 | ebi-a-GCST90001658 | TRUE | G | 97497130 | 1.46E-41 | reported | 3440 | 0.04274 | rs7478275 |  |  |  |  |  |  |  |  |  |  |  |  |  |  |  |
| 0.6055 | 10 | igd | 0.0856 | C | \|\| id:ebi-a-GCST90001659 | ebi-a-GCST90001659 | TRUE | G | 97497130 | 1.77E-44 | reported | 3440 | 0.04266 | rs7478275 |  |  |  |  |  |  |  |  |  |  |  |  |  |  |  |
| 0.4359 | 10 | igd | 0.0856 | C | \|\| id:ebi-a-GCST90001670 | ebi-a-GCST90001670 | TRUE | G | 97497130 | 7.28E-22 | reported | 3440 | 0.04505 | rs7478275 |  |  |  |  |  |  |  |  |  |  |  |  |  |  |  |
| 0.4201 | 10 | igd | 0.0856 | C | \|\| id:ebi-a-GCST90001671 | ebi-a-GCST90001671 | TRUE | G | 97497130 | 2.46E-20 | reported | 3440 | 0.04518 | rs7478275 |  |  |  |  |  |  |  |  |  |  |  |  |  |  |  |
| -0.5138 | 19 | igd | 0.0856 | C | \|\| id:ebi-a-GCST90001957 | ebi-a-GCST90001957 | TRUE | T | 52094863 | 1.81E-14 | reported | 1578 | 0.06641 | rs11883251 |  |  |  |  |  |  |  |  |  |  |  |  |  |  |  |
| -0.6259 | 22 | igd | 0.0855 | T | \|\| id:ebi-a-GCST90001702 | ebi-a-GCST90001702 | TRUE | C | 42616172 | 1.80E-42 | reported | 3657 | 0.04524 | rs118040307 |  |  |  |  |  |  |  |  |  |  |  |  |  |  |  |
| -0.6022 | 22 | igd | 0.0855 | T | \|\| id:ebi-a-GCST90001703 | ebi-a-GCST90001703 | TRUE | C | 42616172 | 2.12E-39 | reported | 3657 | 0.04531 | rs118040307 |  |  |  |  |  |  |  |  |  |  |  |  |  |  |  |
| 0.4473 | 19 | igd | 0.0855 | G | \|\| id:ebi-a-GCST90001954 | ebi-a-GCST90001954 | TRUE | A | 51811788 | 3.43E-11 | reported | 1555 | 0.06701 | rs10407329 |  |  |  |  |  |  |  |  |  |  |  |  |  |  |  |
| -0.5034 | 19 | igd | 0.0855 | C | \|\| id:ebi-a-GCST90001956 | ebi-a-GCST90001956 | TRUE | T | 52094863 | 5.65E-14 | reported | 1579 | 0.06637 | rs11883251 |  |  |  |  |  |  |  |  |  |  |  |  |  |  |  |
| -0.4376 | 19 | igd | 0.0854 | C | \|\| id:ebi-a-GCST90001952 | ebi-a-GCST90001952 | TRUE | T | 52094863 | 5.97E-11 | reported | 1569 | 0.06639 | rs11883251 |  |  |  |  |  |  |  |  |  |  |  |  |  |  |  |
| 0.437 | 19 | igd | 0.0854 | G | \|\| id:ebi-a-GCST90001953 | ebi-a-GCST90001953 | TRUE | A | 51811788 | 9.15E-11 | reported | 1558 | 0.06697 | rs10407329 |  |  |  |  |  |  |  |  |  |  |  |  |  |  |  |
| 0.4808 | 10 | igd | 0.0853 | C | \|\| id:ebi-a-GCST90001483 | ebi-a-GCST90001483 | TRUE | G | 97497130 | 3.84E-27 | reported | 3405 | 0.04417 | rs7478275 |  |  |  |  |  |  |  |  |  |  |  |  |  |  |  |
| 0.5491 | 10 | igd | 0.0853 | C | \|\| id:ebi-a-GCST90001489 | ebi-a-GCST90001489 | TRUE | G | 97497130 | 2.78E-33 | reported | 3405 | 0.04519 | rs7478275 |  |  |  |  |  |  |  |  |  |  |  |  |  |  |  |
| 0.6236 | 10 | igd | 0.0853 | C | \|\| id:ebi-a-GCST90001495 | ebi-a-GCST90001495 | TRUE | G | 97497130 | 7.65E-42 | reported | 3405 | 0.04539 | rs7478275 |  |  |  |  |  |  |  |  |  |  |  |  |  |  |  |
| -0.4903 | 19 | igd | 0.0853 | C | \|\| id:ebi-a-GCST90001947 | ebi-a-GCST90001947 | TRUE | T | 52094863 | 2.99E-13 | reported | 1577 | 0.06663 | rs11883251 |  |  |  |  |  |  |  |  |  |  |  |  |  |  |  |
| 0.4227 | 19 | igd | 0.0853 | G | \|\| id:ebi-a-GCST90001955 | ebi-a-GCST90001955 | TRUE | A | 51811788 | 4.28E-10 | reported | 1554 | 0.06727 | rs10407329 |  |  |  |  |  |  |  |  |  |  |  |  |  |  |  |
| 0.5924 | 10 | igd | 0.0852 | C | \|\| id:ebi-a-GCST90001660 | ebi-a-GCST90001660 | TRUE | G | 97497130 | 1.12E-38 | reported | 3408 | 0.04498 | rs7478275 |  |  |  |  |  |  |  |  |  |  |  |  |  |  |  |
| 0.448 | 10 | igd | 0.0852 | C | \|\| id:ebi-a-GCST90001672 | ebi-a-GCST90001672 | TRUE | G | 97497130 | 3.41E-22 | reported | 3408 | 0.04593 | rs7478275 |  |  |  |  |  |  |  |  |  |  |  |  |  |  |  |
| -0.5208 | 22 | igd | 0.0852 | T | \|\| id:ebi-a-GCST90001704 | ebi-a-GCST90001704 | TRUE | C | 42667552 | 1.38E-29 | reported | 3657 | 0.0457 | rs117168709 |  |  |  |  |  |  |  |  |  |  |  |  |  |  |  |
| -0.6159 | 22 | igd | 0.0852 | T | \|\| id:ebi-a-GCST90001710 | ebi-a-GCST90001710 | TRUE | C | 42667552 | 5.73E-41 | reported | 3657 | 0.04538 | rs117168709 |  |  |  |  |  |  |  |  |  |  |  |  |  |  |  |
| -0.5949 | 22 | igd | 0.0852 | T | \|\| id:ebi-a-GCST90001711 | ebi-a-GCST90001711 | TRUE | C | 42667552 | 1.48E-38 | reported | 3657 | 0.04528 | rs117168709 |  |  |  |  |  |  |  |  |  |  |  |  |  |  |  |
| -0.8685 | 6 | igd | 0.0852 | T | \|\| id:ebi-a-GCST90002108 | ebi-a-GCST90002108 | TRUE | A | 32624001 | 1.71E-16 | reported | 1579 | 0.1042 | rs55866704 |  |  |  |  |  |  |  |  |  |  |  |  |  |  |  |
| -0.6798 | 6 | igd | 0.0852 | T | \|\| id:ebi-a-GCST90002109 | ebi-a-GCST90002109 | TRUE | A | 32624001 | 1.20E-10 | reported | 1578 | 0.1049 | rs55866704 |  |  |  |  |  |  |  |  |  |  |  |  |  |  |  |
| 0.4535 | 6 | igd | 0.0851 | A | \|\| id:ebi-a-GCST90002105 | ebi-a-GCST90002105 | TRUE | G | 28850808 | 7.97E-17 | reported | 2872 | 0.0541 | rs146328993 |  |  |  |  |  |  |  |  |  |  |  |  |  |  |  |
| 0.264 | 10 | igd | 0.085 | G | \|\| id:ebi-a-GCST90001658 | ebi-a-GCST90001658 | TRUE | A | 97174164 | 2.60E-09 | reported | 3440 | 0.04422 | rs3818539 |  |  |  |  |  |  |  |  |  |  |  |  |  |  |  |
| 0.3957 | 19 | igd | 0.0848 | G | \|\| id:ebi-a-GCST90001952 | ebi-a-GCST90001952 | TRUE | A | 51811788 | 5.89E-09 | reported | 1569 | 0.06761 | rs10407329 |  |  |  |  |  |  |  |  |  |  |  |  |  |  |  |
| -0.2623 | 22 | igd | 0.0845 | T | \|\| id:ebi-a-GCST90001710 | ebi-a-GCST90001710 | TRUE | C | 41292821 | 1.10E-08 | reported | 3657 | 0.04579 | rs13056402 |  |  |  |  |  |  |  |  |  |  |  |  |  |  |  |
| -1.048 | 12 | igd | 0.0845 | A | \|\| id:ebi-a-GCST90002074 | ebi-a-GCST90002074 | TRUE | C | 69744014 | 3.30E-114 | reported | 3112 | 0.04423 | rs1800973 |  |  |  |  |  |  |  |  |  |  |  |  |  |  |  |
| -0.2824 | 12 | igd | 0.0844 | A | \|\| id:ebi-a-GCST90001965 | ebi-a-GCST90001965 | TRUE | C | 69744014 | 1.61E-08 | reported | 2850 | 0.04984 | rs1800973 |  |  |  |  |  |  |  |  |  |  |  |  |  |  |  |
| -1.218 | 12 | igd | 0.0844 | A | \|\| id:ebi-a-GCST90002073 | ebi-a-GCST90002073 | TRUE | C | 69744014 | 1.71E-145 | reported | 2850 | 0.04471 | rs1800973 |  |  |  |  |  |  |  |  |  |  |  |  |  |  |  |
| -0.6317 | 22 | igd | 0.0842 | A | \|\| id:ebi-a-GCST90001702 | ebi-a-GCST90001702 | TRUE | G | 42470736 | 9.08E-43 | reported | 3657 | 0.04549 | rs78725400 |  |  |  |  |  |  |  |  |  |  |  |  |  |  |  |
| -0.7736 | 22 | igd | 0.0842 | A | \|\| id:ebi-a-GCST90001702 | ebi-a-GCST90001702 | TRUE | G | 41665995 | 8.64E-64 | reported | 3657 | 0.04499 | rs139764778 |  |  |  |  |  |  |  |  |  |  |  |  |  |  |  |
| -0.7593 | 22 | igd | 0.0842 | A | \|\| id:ebi-a-GCST90001703 | ebi-a-GCST90001703 | TRUE | G | 41665995 | 1.77E-61 | reported | 3657 | 0.04503 | rs139764778 |  |  |  |  |  |  |  |  |  |  |  |  |  |  |  |
| -0.6088 | 22 | igd | 0.0842 | A | \|\| id:ebi-a-GCST90001703 | ebi-a-GCST90001703 | TRUE | G | 42470736 | 8.11E-40 | reported | 3657 | 0.04555 | rs78725400 |  |  |  |  |  |  |  |  |  |  |  |  |  |  |  |
| -0.7077 | 22 | igd | 0.0842 | A | \|\| id:ebi-a-GCST90001704 | ebi-a-GCST90001704 | TRUE | G | 41665995 | 1.47E-53 | reported | 3657 | 0.04519 | rs139764778 |  |  |  |  |  |  |  |  |  |  |  |  |  |  |  |
| -0.5232 | 22 | igd | 0.0842 | A | \|\| id:ebi-a-GCST90001704 | ebi-a-GCST90001704 | TRUE | G | 42470736 | 9.12E-30 | reported | 3657 | 0.04576 | rs78725400 |  |  |  |  |  |  |  |  |  |  |  |  |  |  |  |
| -0.7524 | 22 | igd | 0.0842 | A | \|\| id:ebi-a-GCST90001710 | ebi-a-GCST90001710 | TRUE | G | 41665995 | 1.34E-60 | reported | 3657 | 0.04497 | rs139764778 |  |  |  |  |  |  |  |  |  |  |  |  |  |  |  |
| -0.597 | 22 | igd | 0.0842 | A | \|\| id:ebi-a-GCST90001711 | ebi-a-GCST90001711 | TRUE | G | 42470736 | 1.03E-38 | reported | 3657 | 0.04534 | rs78725400 |  |  |  |  |  |  |  |  |  |  |  |  |  |  |  |
| -0.7477 | 22 | igd | 0.0842 | A | \|\| id:ebi-a-GCST90001711 | ebi-a-GCST90001711 | TRUE | G | 41665995 | 3.15E-60 | reported | 3657 | 0.04484 | rs139764778 |  |  |  |  |  |  |  |  |  |  |  |  |  |  |  |
| -0.38 | 22 | igd | 0.0842 | G | \|\| id:ebi-a-GCST90001830 | ebi-a-GCST90001830 | TRUE | C | 42529366 | 3.82E-18 | reported | 3648 | 0.04352 | rs118017929 |  |  |  |  |  |  |  |  |  |  |  |  |  |  |  |
| 0.233145 | 21 | igd | 0.0841232 | G | \|\| id:met-b-162 | met-b-162 | TRUE | A | 36364598 | 2.28E-11 | reported | 422 | 0.0348653 | rs8130985 |  |  |  |  |  |  |  |  |  |  |  |  |  |  |  |
| -0.2928 | 22 | igd | 0.0841 | A | \|\| id:ebi-a-GCST90001701 | ebi-a-GCST90001701 | TRUE | G | 41665995 | 6.78E-11 | reported | 3592 | 0.04474 | rs139764778 |  |  |  |  |  |  |  |  |  |  |  |  |  |  |  |
| -0.6004 | 19 | igd | 0.0841 | A | \|\| id:ebi-a-GCST90001946 | ebi-a-GCST90001946 | TRUE | C | 51897760 | 1.84E-20 | reported | 1634 | 0.0639 | rs111695695 |  |  |  |  |  |  |  |  |  |  |  |  |  |  |  |
| -0.5582 | 19 | igd | 0.0841 | A | \|\| id:ebi-a-GCST90001949 | ebi-a-GCST90001949 | TRUE | C | 51897760 | 3.87E-18 | reported | 1634 | 0.06354 | rs111695695 |  |  |  |  |  |  |  |  |  |  |  |  |  |  |  |
| -0.3487 | 12 | igd | 0.0841 | A | \|\| id:ebi-a-GCST90002071 | ebi-a-GCST90002071 | TRUE | C | 69744014 | 6.14E-12 | reported | 2872 | 0.0505 | rs1800973 |  |  |  |  |  |  |  |  |  |  |  |  |  |  |  |
| 0.2617 | 3 | igd | 0.084 | T | \|\| id:ebi-a-GCST90001994 | ebi-a-GCST90001994 | TRUE | C | 39315901 | 4.29E-09 | reported | 3590 | 0.04446 | rs56391246 |  |  |  |  |  |  |  |  |  |  |  |  |  |  |  |
| -1.427 | 22 | igd | 0.0839 | G | \|\| id:ebi-a-GCST90001702 | ebi-a-GCST90001702 | TRUE | C | 42529366 | 1.00E-200 | reported | 3657 | 0.03874 | rs118017929 |  |  |  |  |  |  |  |  |  |  |  |  |  |  |  |
| -1.421 | 22 | igd | 0.0839 | G | \|\| id:ebi-a-GCST90001703 | ebi-a-GCST90001703 | TRUE | C | 42529366 | 1.00E-200 | reported | 3657 | 0.0388 | rs118017929 |  |  |  |  |  |  |  |  |  |  |  |  |  |  |  |
| -1.451 | 22 | igd | 0.0839 | G | \|\| id:ebi-a-GCST90001704 | ebi-a-GCST90001704 | TRUE | C | 42529366 | 1.00E-200 | reported | 3657 | 0.03844 | rs118017929 |  |  |  |  |  |  |  |  |  |  |  |  |  |  |  |
| -1.371 | 22 | igd | 0.0839 | G | \|\| id:ebi-a-GCST90001710 | ebi-a-GCST90001710 | TRUE | C | 42529366 | 1.00E-200 | reported | 3657 | 0.03914 | rs118017929 |  |  |  |  |  |  |  |  |  |  |  |  |  |  |  |
| -1.382 | 22 | igd | 0.0839 | G | \|\| id:ebi-a-GCST90001711 | ebi-a-GCST90001711 | TRUE | C | 42529366 | 1.00E-200 | reported | 3657 | 0.03893 | rs118017929 |  |  |  |  |  |  |  |  |  |  |  |  |  |  |  |
| -0.4706 | 22 | igd | 0.0838 | G | \|\| id:ebi-a-GCST90001701 | ebi-a-GCST90001701 | TRUE | C | 42529366 | 3.51E-26 | reported | 3592 | 0.04411 | rs118017929 |  |  |  |  |  |  |  |  |  |  |  |  |  |  |  |
| -0.5693 | 19 | igd | 0.0836 | A | \|\| id:ebi-a-GCST90001948 | ebi-a-GCST90001948 | TRUE | C | 51897760 | 1.54E-18 | reported | 1633 | 0.06402 | rs111695695 |  |  |  |  |  |  |  |  |  |  |  |  |  |  |  |
| 0.2748 | 2 | igd | 0.0831 | A | \|\| id:ebi-a-GCST90001900 | ebi-a-GCST90001900 | TRUE | G | 204661197 | 4.53E-08 | reported | 2919 | 0.05011 | rs10932022 |  |  |  |  |  |  |  |  |  |  |  |  |  |  |  |
| 0.2834 | 6 | igd | 0.083 | T | \|\| id:ebi-a-GCST90002105 | ebi-a-GCST90002105 | TRUE | C | 27398131 | 3.81E-08 | reported | 2872 | 0.05138 | rs116073868 |  |  |  |  |  |  |  |  |  |  |  |  |  |  |  |
| 0.5843 | 6 | igd | 0.083 | C | \|\| id:ebi-a-GCST90002108 | ebi-a-GCST90002108 | TRUE | A | 32332661 | 5.21E-17 | reported | 1579 | 0.06892 | rs34673422 |  |  |  |  |  |  |  |  |  |  |  |  |  |  |  |
| 0.3938 | 6 | igd | 0.083 | C | \|\| id:ebi-a-GCST90002109 | ebi-a-GCST90002109 | TRUE | A | 32332661 | 1.48E-08 | reported | 1578 | 0.06917 | rs34673422 |  |  |  |  |  |  |  |  |  |  |  |  |  |  |  |
| -0.289 | 10 | igd | 0.0829 | G | \|\| id:ebi-a-GCST90001496 | ebi-a-GCST90001496 | TRUE | T | 98048411 | 5.24E-10 | reported | 3437 | 0.04638 | rs78573017 |  |  |  |  |  |  |  |  |  |  |  |  |  |  |  |
| -0.3014 | 10 | igd | 0.0829 | G | \|\| id:ebi-a-GCST90001497 | ebi-a-GCST90001497 | TRUE | T | 98048411 | 5.24E-11 | reported | 3437 | 0.04577 | rs78573017 |  |  |  |  |  |  |  |  |  |  |  |  |  |  |  |
| -0.4685 | 19 | igd | 0.0828 | C | \|\| id:ebi-a-GCST90001953 | ebi-a-GCST90001953 | TRUE | T | 52094863 | 5.03E-12 | reported | 1558 | 0.06732 | rs11883251 |  |  |  |  |  |  |  |  |  |  |  |  |  |  |  |
| -0.2569 | 10 | igd | 0.0827 | A | \|\| id:ebi-a-GCST90001672 | ebi-a-GCST90001672 | TRUE | C | 95535240 | 3.54E-08 | reported | 3408 | 0.04649 | rs10882332 |  |  |  |  |  |  |  |  |  |  |  |  |  |  |  |
| -0.4754 | 19 | igd | 0.0826 | C | \|\| id:ebi-a-GCST90001954 | ebi-a-GCST90001954 | TRUE | T | 52094863 | 2.72E-12 | reported | 1555 | 0.06745 | rs11883251 |  |  |  |  |  |  |  |  |  |  |  |  |  |  |  |
| -0.373 | 10 | igd | 0.0825 | G | \|\| id:ebi-a-GCST90002030 | ebi-a-GCST90002030 | TRUE | T | 98048411 | 4.46E-14 | reported | 2920 | 0.04918 | rs78573017 |  |  |  |  |  |  |  |  |  |  |  |  |  |  |  |
| -0.3316 | 10 | igd | 0.0825 | G | \|\| id:ebi-a-GCST90002032 | ebi-a-GCST90002032 | TRUE | T | 98048411 | 6.01E-11 | reported | 2920 | 0.05048 | rs78573017 |  |  |  |  |  |  |  |  |  |  |  |  |  |  |  |
| -0.279 | 16 | igd | 0.0825 | T | \|\| id:ebi-a-GCST90002087 | ebi-a-GCST90002087 | TRUE | C | 31402042 | 7.22E-09 | reported | 2872 | 0.04807 | rs79298699 |  |  |  |  |  |  |  |  |  |  |  |  |  |  |  |
| -0.4489 | 19 | igd | 0.0824 | C | \|\| id:ebi-a-GCST90001955 | ebi-a-GCST90001955 | TRUE | T | 52094863 | 4.65E-11 | reported | 1554 | 0.06771 | rs11883251 |  |  |  |  |  |  |  |  |  |  |  |  |  |  |  |
| -0.2556 | 10 | igd | 0.0821 | A | \|\| id:ebi-a-GCST90001670 | ebi-a-GCST90001670 | TRUE | C | 95535240 | 2.65E-08 | reported | 3440 | 0.04585 | rs10882332 |  |  |  |  |  |  |  |  |  |  |  |  |  |  |  |
| -0.4418 | 10 | igd | 0.082 | T | \|\| id:ebi-a-GCST90001671 | ebi-a-GCST90001671 | TRUE | C | 97735403 | 1.98E-21 | reported | 3440 | 0.04617 | rs17111253 |  |  |  |  |  |  |  |  |  |  |  |  |  |  |  |
| 0.274 | 10 | igd | 0.082 | T | \|\| id:ebi-a-GCST90002032 | ebi-a-GCST90002032 | TRUE | C | 98395623 | 3.56E-08 | reported | 2920 | 0.04958 | rs147924010 |  |  |  |  |  |  |  |  |  |  |  |  |  |  |  |
| 0.4867 | 19 | igd | 0.0818 | G | \|\| id:ebi-a-GCST90001948 | ebi-a-GCST90001948 | TRUE | A | 51811788 | 2.35E-13 | reported | 1633 | 0.06587 | rs10407329 |  |  |  |  |  |  |  |  |  |  |  |  |  |  |  |
| -0.2654 | 4 | igd | 0.0817 | T | \|\| id:ebi-a-GCST90001471 | ebi-a-GCST90001471 | TRUE | A | 82424430 | 4.22E-09 | reported | 3410 | 0.04505 | rs114951960 |  |  |  |  |  |  |  |  |  |  |  |  |  |  |  |
| 0.4422 | 6 | igd | 0.0817 | G | \|\| id:ebi-a-GCST90002108 | ebi-a-GCST90002108 | TRUE | A | 32709352 | 1.11E-09 | reported | 1579 | 0.07214 | rs28577241 |  |  |  |  |  |  |  |  |  |  |  |  |  |  |  |
| -0.3303 | 2 | igd | 0.0815 | C | \|\| id:ebi-a-GCST90002119 | ebi-a-GCST90002119 | TRUE | T | 87168684 | 4.90E-11 | reported | 2920 | 0.05005 | rs188069130 |  |  |  |  |  |  |  |  |  |  |  |  |  |  |  |
| 0.2614 | 20 | igd | 0.0812 | A | \|\| id:ebi-a-GCST90001804 | ebi-a-GCST90001804 | TRUE | G | 46644731 | 5.79E-09 | reported | 3657 | 0.04478 | rs77441335 |  |  |  |  |  |  |  |  |  |  |  |  |  |  |  |
| 0.2588 | 20 | igd | 0.0812 | A | \|\| id:ebi-a-GCST90001808 | ebi-a-GCST90001808 | TRUE | G | 46644731 | 1.28E-08 | reported | 3657 | 0.04539 | rs77441335 |  |  |  |  |  |  |  |  |  |  |  |  |  |  |  |
| -0.2806 | 10 | igd | 0.081 | A | \|\| id:ebi-a-GCST90002030 | ebi-a-GCST90002030 | TRUE | C | 95535240 | 1.54E-08 | reported | 2920 | 0.04946 | rs10882332 |  |  |  |  |  |  |  |  |  |  |  |  |  |  |  |
| -0.2827 | 10 | igd | 0.0808 | T | \|\| id:ebi-a-GCST90002029 | ebi-a-GCST90002029 | TRUE | C | 97735403 | 1.68E-08 | reported | 2915 | 0.04997 | rs17111253 |  |  |  |  |  |  |  |  |  |  |  |  |  |  |  |
| 0.3889 | 2 | igd | 0.0808 | C | \|\| id:ebi-a-GCST90002098 | ebi-a-GCST90002098 | TRUE | T | 39232035 | 6.84E-16 | reported | 2910 | 0.0479 | rs72799435 |  |  |  |  |  |  |  |  |  |  |  |  |  |  |  |
| 0.2918 | 2 | igd | 0.0808 | C | \|\| id:ebi-a-GCST90002102 | ebi-a-GCST90002102 | TRUE | T | 39232035 | 1.06E-09 | reported | 2919 | 0.04767 | rs72799435 |  |  |  |  |  |  |  |  |  |  |  |  |  |  |  |
| 0.4152 | 10 | igd | 0.0806 | A | \|\| id:ebi-a-GCST90001775 | ebi-a-GCST90001775 | TRUE | G | 6177494 | 1.62E-19 | reported | 3660 | 0.04569 | rs57069153 |  |  |  |  |  |  |  |  |  |  |  |  |  |  |  |
| 0.4417 | 10 | igd | 0.0805 | A | \|\| id:ebi-a-GCST90001775 | ebi-a-GCST90001775 | TRUE | G | 6050933 | 1.56E-21 | reported | 3660 | 0.04605 | rs34181124 |  |  |  |  |  |  |  |  |  |  |  |  |  |  |  |
| 0.3959 | 10 | igd | 0.0805 | A | \|\| id:ebi-a-GCST90001794 | ebi-a-GCST90001794 | TRUE | G | 6177494 | 7.18E-18 | reported | 3658 | 0.04574 | rs57069153 |  |  |  |  |  |  |  |  |  |  |  |  |  |  |  |
| 0.4901 | 10 | igd | 0.0804 | A | \|\| id:ebi-a-GCST90001779 | ebi-a-GCST90001779 | TRUE | G | 6177494 | 6.47E-27 | reported | 3657 | 0.04526 | rs57069153 |  |  |  |  |  |  |  |  |  |  |  |  |  |  |  |
| 0.2835 | 10 | igd | 0.0804 | A | \|\| id:ebi-a-GCST90001783 | ebi-a-GCST90001783 | TRUE | G | 6177478 | 3.48E-10 | reported | 3656 | 0.04505 | rs112613053 |  |  |  |  |  |  |  |  |  |  |  |  |  |  |  |
| 0.4482 | 10 | igd | 0.0804 | A | \|\| id:ebi-a-GCST90001784 | ebi-a-GCST90001784 | TRUE | G | 6177494 | 2.02E-22 | reported | 3657 | 0.04572 | rs57069153 |  |  |  |  |  |  |  |  |  |  |  |  |  |  |  |
| 0.4718 | 10 | igd | 0.0804 | A | \|\| id:ebi-a-GCST90001791 | ebi-a-GCST90001791 | TRUE | G | 6177494 | 1.14E-24 | reported | 3657 | 0.04568 | rs57069153 |  |  |  |  |  |  |  |  |  |  |  |  |  |  |  |
| 0.4276 | 10 | igd | 0.0804 | A | \|\| id:ebi-a-GCST90001794 | ebi-a-GCST90001794 | TRUE | G | 6050933 | 2.94E-20 | reported | 3658 | 0.0461 | rs34181124 |  |  |  |  |  |  |  |  |  |  |  |  |  |  |  |
| 0.4463 | 10 | igd | 0.0803 | A | \|\| id:ebi-a-GCST90001778 | ebi-a-GCST90001778 | TRUE | G | 6050933 | 1.54E-22 | reported | 3657 | 0.04539 | rs34181124 |  |  |  |  |  |  |  |  |  |  |  |  |  |  |  |
| 0.5101 | 10 | igd | 0.0803 | A | \|\| id:ebi-a-GCST90001779 | ebi-a-GCST90001779 | TRUE | G | 6050933 | 1.53E-28 | reported | 3657 | 0.04564 | rs34181124 |  |  |  |  |  |  |  |  |  |  |  |  |  |  |  |
| 0.3821 | 10 | igd | 0.0803 | A | \|\| id:ebi-a-GCST90001780 | ebi-a-GCST90001780 | TRUE | G | 6050933 | 9.27E-17 | reported | 3642 | 0.04574 | rs34181124 |  |  |  |  |  |  |  |  |  |  |  |  |  |  |  |
| 0.4448 | 10 | igd | 0.0803 | T | \|\| id:ebi-a-GCST90001782 | ebi-a-GCST90001782 | TRUE | C | 6183022 | 5.81E-13 | reported | 1837 | 0.06129 | rs10905892 |  |  |  |  |  |  |  |  |  |  |  |  |  |  |  |
| 0.4752 | 10 | igd | 0.0803 | A | \|\| id:ebi-a-GCST90001784 | ebi-a-GCST90001784 | TRUE | G | 6050933 | 1.35E-24 | reported | 3657 | 0.04608 | rs34181124 |  |  |  |  |  |  |  |  |  |  |  |  |  |  |  |
| 0.4865 | 10 | igd | 0.0803 | A | \|\| id:ebi-a-GCST90001791 | ebi-a-GCST90001791 | TRUE | G | 6050933 | 1.08E-25 | reported | 3657 | 0.04608 | rs34181124 |  |  |  |  |  |  |  |  |  |  |  |  |  |  |  |
| 0.4937 | 10 | igd | 0.0803 | A | \|\| id:ebi-a-GCST90001792 | ebi-a-GCST90001792 | TRUE | G | 6050933 | 1.56E-27 | reported | 3657 | 0.04504 | rs34181124 |  |  |  |  |  |  |  |  |  |  |  |  |  |  |  |
| -0.4077 | 10 | igd | 0.0803 | G | \|\| id:ebi-a-GCST90001961 | ebi-a-GCST90001961 | TRUE | A | 96997820 | 5.63E-16 | reported | 2920 | 0.05006 | rs1049814 |  |  |  |  |  |  |  |  |  |  |  |  |  |  |  |
| 0.4293 | 10 | igd | 0.0801 | A | \|\| id:ebi-a-GCST90002030 | ebi-a-GCST90002030 | TRUE | G | 98674197 | 6.19E-17 | reported | 2920 | 0.05102 | rs113614971 |  |  |  |  |  |  |  |  |  |  |  |  |  |  |  |
| 0.3501 | 10 | igd | 0.0801 | A | \|\| id:ebi-a-GCST90002031 | ebi-a-GCST90002031 | TRUE | G | 98674197 | 2.56E-11 | reported | 2920 | 0.05229 | rs113614971 |  |  |  |  |  |  |  |  |  |  |  |  |  |  |  |
| 0.4258 | 10 | igd | 0.0801 | A | \|\| id:ebi-a-GCST90002032 | ebi-a-GCST90002032 | TRUE | G | 98674197 | 4.37E-16 | reported | 2920 | 0.05209 | rs113614971 |  |  |  |  |  |  |  |  |  |  |  |  |  |  |  |
| 0.3017 | 10 | igd | 0.08 | A | \|\| id:ebi-a-GCST90001489 | ebi-a-GCST90001489 | TRUE | G | 98674197 | 6.23E-10 | reported | 3405 | 0.04864 | rs113614971 |  |  |  |  |  |  |  |  |  |  |  |  |  |  |  |
| 0.3237 | 10 | igd | 0.08 | A | \|\| id:ebi-a-GCST90001495 | ebi-a-GCST90001495 | TRUE | G | 98674197 | 4.85E-11 | reported | 3405 | 0.04907 | rs113614971 |  |  |  |  |  |  |  |  |  |  |  |  |  |  |  |
| 0.3005 | 10 | igd | 0.08 | A | \|\| id:ebi-a-GCST90001660 | ebi-a-GCST90001660 | TRUE | G | 98674197 | 6.84E-10 | reported | 3408 | 0.04856 | rs113614971 |  |  |  |  |  |  |  |  |  |  |  |  |  |  |  |
| -0.3273 | 2 | igd | 0.08 | T | \|\| id:ebi-a-GCST90002118 | ebi-a-GCST90002118 | TRUE | A | 87034294 | 6.00E-11 | reported | 2920 | 0.04984 | rs2367375 |  |  |  |  |  |  |  |  |  |  |  |  |  |  |  |
| -0.351 | 19 | igd | 0.07999 | C | \|\| id:prot-a-439 | prot-a-439 | TRUE | G | 51646140 | 8.13E-13 | reported | 3301 | 0.049 | rs140185670 |  |  |  |  |  |  |  |  |  |  |  |  |  |  |  |
| 0.2715 | 10 | igd | 0.0799 | A | \|\| id:ebi-a-GCST90001484 | ebi-a-GCST90001484 | TRUE | G | 98674197 | 7.84E-09 | reported | 3437 | 0.04692 | rs113614971 |  |  |  |  |  |  |  |  |  |  |  |  |  |  |  |
| 0.325 | 10 | igd | 0.0799 | A | \|\| id:ebi-a-GCST90001491 | ebi-a-GCST90001491 | TRUE | G | 98674197 | 1.23E-11 | reported | 3437 | 0.04779 | rs113614971 |  |  |  |  |  |  |  |  |  |  |  |  |  |  |  |
| 0.3527 | 6 | igd | 0.0799 | G | \|\| id:ebi-a-GCST90002105 | ebi-a-GCST90002105 | TRUE | A | 32709352 | 9.67E-10 | reported | 2872 | 0.05748 | rs28577241 |  |  |  |  |  |  |  |  |  |  |  |  |  |  |  |
| 0.2558 | 10 | igd | 0.0798 | A | \|\| id:ebi-a-GCST90001659 | ebi-a-GCST90001659 | TRUE | G | 98674197 | 3.58E-08 | reported | 3440 | 0.04631 | rs113614971 |  |  |  |  |  |  |  |  |  |  |  |  |  |  |  |
| -0.2492 | 12 | igd | 0.0798 | A | \|\| id:ebi-a-GCST90002003 | ebi-a-GCST90002003 | TRUE | C | 69744014 | 2.82E-08 | reported | 3629 | 0.04479 | rs1800973 |  |  |  |  |  |  |  |  |  |  |  |  |  |  |  |
| -0.2797 | 12 | igd | 0.0798 | A | \|\| id:ebi-a-GCST90002000 | ebi-a-GCST90002000 | TRUE | C | 69744014 | 2.37E-10 | reported | 3629 | 0.04402 | rs1800973 |  |  |  |  |  |  |  |  |  |  |  |  |  |  |  |
| 0.3371 | 10 | igd | 0.0796 | T | \|\| id:ebi-a-GCST90001496 | ebi-a-GCST90001496 | TRUE | C | 98597402 | 4.24E-12 | reported | 3437 | 0.04848 | rs2790466 |  |  |  |  |  |  |  |  |  |  |  |  |  |  |  |
| 0.3554 | 10 | igd | 0.0796 | T | \|\| id:ebi-a-GCST90001497 | ebi-a-GCST90001497 | TRUE | C | 98597402 | 1.36E-13 | reported | 3437 | 0.04783 | rs2790466 |  |  |  |  |  |  |  |  |  |  |  |  |  |  |  |
| 0.9394 | 6 | igd | 0.0796 | C | \|\| id:ebi-a-GCST90002105 | ebi-a-GCST90002105 | TRUE | T | 32643328 | 9.81E-39 | reported | 2872 | 0.0711 | rs9275028 |  |  |  |  |  |  |  |  |  |  |  |  |  |  |  |
| -0.3003 | 10 | igd | 0.0793 | T | \|\| id:ebi-a-GCST90001961 | ebi-a-GCST90001961 | TRUE | C | 95966358 | 3.02E-09 | reported | 2920 | 0.05048 | rs7908334 |  |  |  |  |  |  |  |  |  |  |  |  |  |  |  |
| -0.3441 | 22 | igd | 0.0792 | C | \|\| id:ebi-a-GCST90001702 | ebi-a-GCST90001702 | TRUE | T | 43597066 | 2.81E-13 | reported | 3657 | 0.04694 | rs9612025 |  |  |  |  |  |  |  |  |  |  |  |  |  |  |  |
| -0.3448 | 22 | igd | 0.0792 | C | \|\| id:ebi-a-GCST90001710 | ebi-a-GCST90001710 | TRUE | T | 43597066 | 2.18E-13 | reported | 3657 | 0.04681 | rs9612025 |  |  |  |  |  |  |  |  |  |  |  |  |  |  |  |
| 0.4125 | 6 | igd | 0.0792 | A | \|\| id:ebi-a-GCST90002105 | ebi-a-GCST90002105 | TRUE | G | 32071008 | 5.65E-15 | reported | 2872 | 0.05252 | rs41268902 |  |  |  |  |  |  |  |  |  |  |  |  |  |  |  |
| 0.5775 | 10 | igd | 0.0791 | G | \|\| id:ebi-a-GCST90001489 | ebi-a-GCST90001489 | TRUE | C | 97655268 | 4.48E-35 | reported | 3405 | 0.04621 | rs7909917 |  |  |  |  |  |  |  |  |  |  |  |  |  |  |  |
| 0.4081 | 10 | igd | 0.0791 | A | \|\| id:ebi-a-GCST90001961 | ebi-a-GCST90001961 | TRUE | G | 97913520 | 1.50E-15 | reported | 2920 | 0.05088 | rs78407012 |  |  |  |  |  |  |  |  |  |  |  |  |  |  |  |
| -0.2795 | 10 | igd | 0.0789 | A | \|\| id:ebi-a-GCST90001489 | ebi-a-GCST90001489 | TRUE | G | 98404462 | 5.82E-09 | reported | 3405 | 0.04789 | rs117362004 |  |  |  |  |  |  |  |  |  |  |  |  |  |  |  |
| -0.2945 | 10 | igd | 0.0789 | A | \|\| id:ebi-a-GCST90001495 | ebi-a-GCST90001495 | TRUE | G | 98404462 | 1.31E-09 | reported | 3405 | 0.04841 | rs117362004 |  |  |  |  |  |  |  |  |  |  |  |  |  |  |  |
| -0.3443 | 10 | igd | 0.0789 | A | \|\| id:ebi-a-GCST90002030 | ebi-a-GCST90002030 | TRUE | G | 98404462 | 9.30E-12 | reported | 2920 | 0.05031 | rs117362004 |  |  |  |  |  |  |  |  |  |  |  |  |  |  |  |
| -0.3209 | 10 | igd | 0.0788 | A | \|\| id:ebi-a-GCST90001660 | ebi-a-GCST90001660 | TRUE | G | 98404462 | 2.33E-11 | reported | 3408 | 0.04786 | rs117362004 |  |  |  |  |  |  |  |  |  |  |  |  |  |  |  |
| -0.3341 | 10 | igd | 0.0788 | A | \|\| id:ebi-a-GCST90001672 | ebi-a-GCST90001672 | TRUE | G | 98404462 | 5.52E-12 | reported | 3408 | 0.04831 | rs117362004 |  |  |  |  |  |  |  |  |  |  |  |  |  |  |  |
| 0.4117 | 6 | igd | 0.0788 | C | \|\| id:ebi-a-GCST90002108 | ebi-a-GCST90002108 | TRUE | T | 32933029 | 1.81E-08 | reported | 1579 | 0.07274 | rs16871255 |  |  |  |  |  |  |  |  |  |  |  |  |  |  |  |
| 0.4253 | 10 | igd | 0.0785 | A | \|\| id:ebi-a-GCST90001781 | ebi-a-GCST90001781 | TRUE | C | 6171591 | 3.11E-11 | reported | 1835 | 0.06365 | rs11256929 |  |  |  |  |  |  |  |  |  |  |  |  |  |  |  |
| -0.273 | 10 | igd | 0.0784 | A | \|\| id:ebi-a-GCST90001497 | ebi-a-GCST90001497 | TRUE | G | 98404462 | 8.83E-09 | reported | 3437 | 0.04734 | rs117362004 |  |  |  |  |  |  |  |  |  |  |  |  |  |  |  |
| -0.3019 | 10 | igd | 0.0783 | A | \|\| id:ebi-a-GCST90001658 | ebi-a-GCST90001658 | TRUE | G | 98404462 | 4.49E-11 | reported | 3440 | 0.04568 | rs117362004 |  |  |  |  |  |  |  |  |  |  |  |  |  |  |  |
| -0.3061 | 10 | igd | 0.0783 | A | \|\| id:ebi-a-GCST90001659 | ebi-a-GCST90001659 | TRUE | G | 98404462 | 2.41E-11 | reported | 3440 | 0.04568 | rs117362004 |  |  |  |  |  |  |  |  |  |  |  |  |  |  |  |
| -0.314 | 10 | igd | 0.0783 | A | \|\| id:ebi-a-GCST90001670 | ebi-a-GCST90001670 | TRUE | G | 98404462 | 4.44E-11 | reported | 3440 | 0.0475 | rs117362004 |  |  |  |  |  |  |  |  |  |  |  |  |  |  |  |
| -0.3249 | 10 | igd | 0.0783 | A | \|\| id:ebi-a-GCST90001671 | ebi-a-GCST90001671 | TRUE | G | 98404462 | 1.02E-11 | reported | 3440 | 0.0476 | rs117362004 |  |  |  |  |  |  |  |  |  |  |  |  |  |  |  |
| -0.2551 | 3 | igd | 0.0783 | T | \|\| id:ebi-a-GCST90002004 | ebi-a-GCST90002004 | TRUE | C | 45153051 | 1.60E-08 | reported | 3629 | 0.04506 | rs35605067 |  |  |  |  |  |  |  |  |  |  |  |  |  |  |  |
| -0.2989 | 11 | igd | 0.0782 | T | \|\| id:ebi-a-GCST90001909 | ebi-a-GCST90001909 | TRUE | A | 5403108 | 3.68E-09 | reported | 3112 | 0.05053 | rs12364299 |  |  |  |  |  |  |  |  |  |  |  |  |  |  |  |
| -0.3725 | 1 | igd | 0.0782 | A | \|\| id:ebi-a-GCST90002035 | ebi-a-GCST90002035 | TRUE | G | 93035991 | 2.32E-12 | reported | 2871 | 0.05288 | rs116359246 |  |  |  |  |  |  |  |  |  |  |  |  |  |  |  |
| -0.3835 | 1 | igd | 0.0782 | A | \|\| id:ebi-a-GCST90002036 | ebi-a-GCST90002036 | TRUE | G | 93035991 | 5.99E-13 | reported | 2871 | 0.05302 | rs116359246 |  |  |  |  |  |  |  |  |  |  |  |  |  |  |  |
| -0.2747 | 2 | igd | 0.0781 | G | \|\| id:ebi-a-GCST90001886 | ebi-a-GCST90001886 | TRUE | A | 204698763 | 1.90E-08 | reported | 2920 | 0.04873 | rs113047912 |  |  |  |  |  |  |  |  |  |  |  |  |  |  |  |
| -0.384 | 2 | igd | 0.0781 | G | \|\| id:ebi-a-GCST90001891 | ebi-a-GCST90001891 | TRUE | A | 204698763 | 3.58E-14 | reported | 2920 | 0.05043 | rs113047912 |  |  |  |  |  |  |  |  |  |  |  |  |  |  |  |
| -0.5372 | 19 | igd | 0.0781 | A | \|\| id:ebi-a-GCST90001952 | ebi-a-GCST90001952 | TRUE | C | 51897760 | 7.14E-15 | reported | 1569 | 0.06836 | rs111695695 |  |  |  |  |  |  |  |  |  |  |  |  |  |  |  |
| 0.4362 | 6 | igd | 0.0777 | A | \|\| id:ebi-a-GCST90002112 | ebi-a-GCST90002112 | TRUE | G | 32071008 | 2.46E-10 | reported | 1635 | 0.06849 | rs41268902 |  |  |  |  |  |  |  |  |  |  |  |  |  |  |  |
| -0.275 | 10 | igd | 0.0776 | T | \|\| id:ebi-a-GCST90001660 | ebi-a-GCST90001660 | TRUE | A | 99461284 | 2.12E-08 | reported | 3408 | 0.04897 | rs1002938 |  |  |  |  |  |  |  |  |  |  |  |  |  |  |  |
| -0.3143 | 22 | igd | 0.0775 | G | \|\| id:ebi-a-GCST90001702 | ebi-a-GCST90001702 | TRUE | A | 40098459 | 6.98E-11 | reported | 3657 | 0.04806 | rs35969023 |  |  |  |  |  |  |  |  |  |  |  |  |  |  |  |
| -0.2929 | 22 | igd | 0.0775 | G | \|\| id:ebi-a-GCST90001703 | ebi-a-GCST90001703 | TRUE | A | 40098459 | 1.23E-09 | reported | 3657 | 0.04808 | rs35969023 |  |  |  |  |  |  |  |  |  |  |  |  |  |  |  |
| -0.3033 | 22 | igd | 0.0775 | G | \|\| id:ebi-a-GCST90001710 | ebi-a-GCST90001710 | TRUE | A | 40098459 | 2.88E-10 | reported | 3657 | 0.04797 | rs35969023 |  |  |  |  |  |  |  |  |  |  |  |  |  |  |  |
| -0.2944 | 22 | igd | 0.0775 | G | \|\| id:ebi-a-GCST90001711 | ebi-a-GCST90001711 | TRUE | A | 40098459 | 8.20E-10 | reported | 3657 | 0.04782 | rs35969023 |  |  |  |  |  |  |  |  |  |  |  |  |  |  |  |
| -0.5879 | 19 | igd | 0.07741 | C | \|\| id:prot-a-439 | prot-a-439 | TRUE | G | 51691761 | 8.13E-33 | reported | 3301 | 0.0493 | rs35413640 |  |  |  |  |  |  |  |  |  |  |  |  |  |  |  |
| 0.4587 | 10 | igd | 0.0774 | A | \|\| id:ebi-a-GCST90001483 | ebi-a-GCST90001483 | TRUE | G | 97299035 | 2.73E-23 | reported | 3405 | 0.0458 | rs11188349 |  |  |  |  |  |  |  |  |  |  |  |  |  |  |  |
| -0.281 | 10 | igd | 0.0774 | T | \|\| id:ebi-a-GCST90001489 | ebi-a-GCST90001489 | TRUE | A | 99461284 | 1.22E-08 | reported | 3405 | 0.04919 | rs1002938 |  |  |  |  |  |  |  |  |  |  |  |  |  |  |  |
| -0.2711 | 10 | igd | 0.0774 | C | \|\| id:ebi-a-GCST90001491 | ebi-a-GCST90001491 | TRUE | T | 96389285 | 3.34E-08 | reported | 3437 | 0.04897 | rs187722828 |  |  |  |  |  |  |  |  |  |  |  |  |  |  |  |
| -0.2906 | 10 | igd | 0.0774 | C | \|\| id:ebi-a-GCST90001496 | ebi-a-GCST90001496 | TRUE | T | 96389285 | 5.07E-09 | reported | 3437 | 0.0496 | rs187722828 |  |  |  |  |  |  |  |  |  |  |  |  |  |  |  |
| -0.2779 | 10 | igd | 0.0774 | C | \|\| id:ebi-a-GCST90001497 | ebi-a-GCST90001497 | TRUE | T | 96389285 | 1.52E-08 | reported | 3437 | 0.04899 | rs187722828 |  |  |  |  |  |  |  |  |  |  |  |  |  |  |  |
| -0.5626 | 22 | igd | 0.0774 | C | \|\| id:ebi-a-GCST90001711 | ebi-a-GCST90001711 | TRUE | G | 43268311 | 1.45E-32 | reported | 3657 | 0.04689 | rs80273835 |  |  |  |  |  |  |  |  |  |  |  |  |  |  |  |
| 0.4239 | 10 | igd | 0.0774 | T | \|\| id:ebi-a-GCST90001778 | ebi-a-GCST90001778 | TRUE | C | 6183022 | 5.39E-20 | reported | 3657 | 0.04603 | rs10905892 |  |  |  |  |  |  |  |  |  |  |  |  |  |  |  |
| 0.348 | 10 | igd | 0.0774 | T | \|\| id:ebi-a-GCST90001780 | ebi-a-GCST90001780 | TRUE | C | 6183022 | 7.90E-14 | reported | 3642 | 0.0464 | rs10905892 |  |  |  |  |  |  |  |  |  |  |  |  |  |  |  |
| 0.391 | 10 | igd | 0.0774 | T | \|\| id:ebi-a-GCST90001787 | ebi-a-GCST90001787 | TRUE | C | 6183022 | 3.32E-17 | reported | 3657 | 0.04613 | rs10905892 |  |  |  |  |  |  |  |  |  |  |  |  |  |  |  |
| 0.4526 | 10 | igd | 0.0774 | T | \|\| id:ebi-a-GCST90001792 | ebi-a-GCST90001792 | TRUE | C | 6183022 | 8.63E-23 | reported | 3657 | 0.04575 | rs10905892 |  |  |  |  |  |  |  |  |  |  |  |  |  |  |  |
| -0.2596 | 10 | igd | 0.0773 | T | \|\| id:ebi-a-GCST90001658 | ebi-a-GCST90001658 | TRUE | A | 99461284 | 2.98E-08 | reported | 3440 | 0.04673 | rs1002938 |  |  |  |  |  |  |  |  |  |  |  |  |  |  |  |
| -0.2622 | 10 | igd | 0.0773 | C | \|\| id:ebi-a-GCST90001659 | ebi-a-GCST90001659 | TRUE | T | 96389285 | 3.28E-08 | reported | 3440 | 0.04734 | rs187722828 |  |  |  |  |  |  |  |  |  |  |  |  |  |  |  |
| 0.5037 | 10 | igd | 0.0773 | A | \|\| id:ebi-a-GCST90001660 | ebi-a-GCST90001660 | TRUE | G | 97299035 | 1.23E-26 | reported | 3408 | 0.04676 | rs11188349 |  |  |  |  |  |  |  |  |  |  |  |  |  |  |  |
| 0.4521 | 10 | igd | 0.0773 | A | \|\| id:ebi-a-GCST90001672 | ebi-a-GCST90001672 | TRUE | G | 97299035 | 2.60E-21 | reported | 3408 | 0.04738 | rs11188349 |  |  |  |  |  |  |  |  |  |  |  |  |  |  |  |
| 0.3101 | 1 | igd | 0.0773 | C | \|\| id:ebi-a-GCST90001854 | ebi-a-GCST90001854 | TRUE | A | 167360046 | 1.18E-09 | reported | 2919 | 0.05082 | rs139450566 |  |  |  |  |  |  |  |  |  |  |  |  |  |  |  |
| 0.2912 | 1 | igd | 0.0773 | C | \|\| id:ebi-a-GCST90001856 | ebi-a-GCST90001856 | TRUE | A | 167360046 | 1.41E-08 | reported | 2919 | 0.05119 | rs139450566 |  |  |  |  |  |  |  |  |  |  |  |  |  |  |  |
| 0.325 | 1 | igd | 0.0773 | C | \|\| id:ebi-a-GCST90001858 | ebi-a-GCST90001858 | TRUE | A | 167360046 | 2.52E-10 | reported | 2919 | 0.05119 | rs139450566 |  |  |  |  |  |  |  |  |  |  |  |  |  |  |  |
| 0.3378 | 1 | igd | 0.0773 | C | \|\| id:ebi-a-GCST90001860 | ebi-a-GCST90001860 | TRUE | A | 167360046 | 5.75E-11 | reported | 2919 | 0.05138 | rs139450566 |  |  |  |  |  |  |  |  |  |  |  |  |  |  |  |
| 0.3325 | 1 | igd | 0.0773 | C | \|\| id:ebi-a-GCST90001863 | ebi-a-GCST90001863 | TRUE | A | 167360046 | 4.41E-11 | reported | 2919 | 0.05027 | rs139450566 |  |  |  |  |  |  |  |  |  |  |  |  |  |  |  |
| 0.4326 | 1 | igd | 0.0773 | C | \|\| id:ebi-a-GCST90001864 | ebi-a-GCST90001864 | TRUE | A | 167360046 | 2.87E-17 | reported | 2919 | 0.05086 | rs139450566 |  |  |  |  |  |  |  |  |  |  |  |  |  |  |  |
| -0.5399 | 19 | igd | 0.0773 | A | \|\| id:ebi-a-GCST90001953 | ebi-a-GCST90001953 | TRUE | C | 51897760 | 3.59E-15 | reported | 1558 | 0.06793 | rs111695695 |  |  |  |  |  |  |  |  |  |  |  |  |  |  |  |
| 0.5863 | 10 | igd | 0.0772 | A | \|\| id:ebi-a-GCST90001484 | ebi-a-GCST90001484 | TRUE | G | 97299035 | 8.18E-38 | reported | 3437 | 0.04506 | rs11188349 |  |  |  |  |  |  |  |  |  |  |  |  |  |  |  |
| 0.5116 | 10 | igd | 0.0772 | A | \|\| id:ebi-a-GCST90001485 | ebi-a-GCST90001485 | TRUE | G | 97299035 | 4.10E-29 | reported | 3437 | 0.04526 | rs11188349 |  |  |  |  |  |  |  |  |  |  |  |  |  |  |  |
| 0.4158 | 10 | igd | 0.0772 | A | \|\| id:ebi-a-GCST90001491 | ebi-a-GCST90001491 | TRUE | G | 97299035 | 5.73E-19 | reported | 3437 | 0.04646 | rs11188349 |  |  |  |  |  |  |  |  |  |  |  |  |  |  |  |
| 0.5106 | 10 | igd | 0.0772 | A | \|\| id:ebi-a-GCST90001496 | ebi-a-GCST90001496 | TRUE | G | 97299035 | 3.05E-27 | reported | 3437 | 0.04683 | rs11188349 |  |  |  |  |  |  |  |  |  |  |  |  |  |  |  |
| 0.5071 | 10 | igd | 0.0772 | A | \|\| id:ebi-a-GCST90001497 | ebi-a-GCST90001497 | TRUE | G | 97299035 | 1.55E-27 | reported | 3437 | 0.04623 | rs11188349 |  |  |  |  |  |  |  |  |  |  |  |  |  |  |  |
| 0.4995 | 10 | igd | 0.0772 | A | \|\| id:ebi-a-GCST90001658 | ebi-a-GCST90001658 | TRUE | G | 97299035 | 1.24E-28 | reported | 3440 | 0.04459 | rs11188349 |  |  |  |  |  |  |  |  |  |  |  |  |  |  |  |
| 0.5215 | 10 | igd | 0.0772 | A | \|\| id:ebi-a-GCST90001659 | ebi-a-GCST90001659 | TRUE | G | 97299035 | 4.24E-31 | reported | 3440 | 0.04453 | rs11188349 |  |  |  |  |  |  |  |  |  |  |  |  |  |  |  |
| 0.4491 | 10 | igd | 0.0772 | A | \|\| id:ebi-a-GCST90001670 | ebi-a-GCST90001670 | TRUE | G | 97299035 | 1.14E-21 | reported | 3440 | 0.04665 | rs11188349 |  |  |  |  |  |  |  |  |  |  |  |  |  |  |  |
| 0.4595 | 10 | igd | 0.0772 | A | \|\| id:ebi-a-GCST90001671 | ebi-a-GCST90001671 | TRUE | G | 97299035 | 1.43E-22 | reported | 3440 | 0.04667 | rs11188349 |  |  |  |  |  |  |  |  |  |  |  |  |  |  |  |
| 0.2741 | 10 | igd | 0.0772 | T | \|\| id:ebi-a-GCST90001786 | ebi-a-GCST90001786 | TRUE | C | 6183022 | 3.65E-09 | reported | 3651 | 0.04635 | rs10905892 |  |  |  |  |  |  |  |  |  |  |  |  |  |  |  |
| -0.5454 | 19 | igd | 0.0772 | A | \|\| id:ebi-a-GCST90001954 | ebi-a-GCST90001954 | TRUE | C | 51897760 | 2.04E-15 | reported | 1555 | 0.06799 | rs111695695 |  |  |  |  |  |  |  |  |  |  |  |  |  |  |  |
| -0.5128 | 19 | igd | 0.0772 | A | \|\| id:ebi-a-GCST90001955 | ebi-a-GCST90001955 | TRUE | C | 51897760 | 1.06E-13 | reported | 1554 | 0.06836 | rs111695695 |  |  |  |  |  |  |  |  |  |  |  |  |  |  |  |
| 0.3963 | 19 | igd | 0.0772 | A | \|\| id:ebi-a-GCST90002052 | ebi-a-GCST90002052 | TRUE | C | 51897760 | 1.38E-08 | reported | 1555 | 0.06945 | rs111695695 |  |  |  |  |  |  |  |  |  |  |  |  |  |  |  |
| 0.3234 | 1 | igd | 0.0771 | C | \|\| id:ebi-a-GCST90001845 | ebi-a-GCST90001845 | TRUE | A | 167360046 | 3.28E-10 | reported | 2911 | 0.05128 | rs139450566 |  |  |  |  |  |  |  |  |  |  |  |  |  |  |  |
| 0.3051 | 1 | igd | 0.0771 | T | \|\| id:ebi-a-GCST90001854 | ebi-a-GCST90001854 | TRUE | C | 167181536 | 1.98E-09 | reported | 2919 | 0.05069 | rs77013340 |  |  |  |  |  |  |  |  |  |  |  |  |  |  |  |
| 0.2916 | 1 | igd | 0.0771 | T | \|\| id:ebi-a-GCST90001856 | ebi-a-GCST90001856 | TRUE | C | 167181536 | 1.21E-08 | reported | 2919 | 0.05103 | rs77013340 |  |  |  |  |  |  |  |  |  |  |  |  |  |  |  |
| 0.3195 | 1 | igd | 0.0771 | T | \|\| id:ebi-a-GCST90001858 | ebi-a-GCST90001858 | TRUE | C | 167181536 | 4.54E-10 | reported | 2919 | 0.05108 | rs77013340 |  |  |  |  |  |  |  |  |  |  |  |  |  |  |  |
| 0.335 | 1 | igd | 0.0771 | T | \|\| id:ebi-a-GCST90001860 | ebi-a-GCST90001860 | TRUE | C | 167181536 | 7.39E-11 | reported | 2919 | 0.05125 | rs77013340 |  |  |  |  |  |  |  |  |  |  |  |  |  |  |  |
| 0.3293 | 1 | igd | 0.0771 | T | \|\| id:ebi-a-GCST90001863 | ebi-a-GCST90001863 | TRUE | C | 167181536 | 5.96E-11 | reported | 2919 | 0.05013 | rs77013340 |  |  |  |  |  |  |  |  |  |  |  |  |  |  |  |
| 0.4237 | 1 | igd | 0.0771 | T | \|\| id:ebi-a-GCST90001864 | ebi-a-GCST90001864 | TRUE | C | 167181536 | 1.05E-16 | reported | 2919 | 0.05075 | rs77013340 |  |  |  |  |  |  |  |  |  |  |  |  |  |  |  |
| -0.3024 | 12 | igd | 0.0771 | C | \|\| id:ebi-a-GCST90002071 | ebi-a-GCST90002071 | TRUE | G | 69612262 | 1.14E-08 | reported | 2872 | 0.05283 | rs79242862 |  |  |  |  |  |  |  |  |  |  |  |  |  |  |  |
| -0.9675 | 12 | igd | 0.077 | C | \|\| id:ebi-a-GCST90002073 | ebi-a-GCST90002073 | TRUE | G | 69612262 | 4.14E-80 | reported | 2850 | 0.04944 | rs79242862 |  |  |  |  |  |  |  |  |  |  |  |  |  |  |  |
| 0.3165 | 1 | igd | 0.0769 | T | \|\| id:ebi-a-GCST90001845 | ebi-a-GCST90001845 | TRUE | C | 167181536 | 7.07E-10 | reported | 2911 | 0.05116 | rs77013340 |  |  |  |  |  |  |  |  |  |  |  |  |  |  |  |
| 0.2564 | 22 | igd | 0.0767 | T | \|\| id:ebi-a-GCST90001703 | ebi-a-GCST90001703 | TRUE | C | 44290480 | 3.57E-08 | reported | 3657 | 0.04643 | rs56152442 |  |  |  |  |  |  |  |  |  |  |  |  |  |  |  |
| 0.3047 | 10 | igd | 0.0767 | C | \|\| id:ebi-a-GCST90001783 | ebi-a-GCST90001783 | TRUE | G | 6033846 | 6.21E-11 | reported | 3656 | 0.04645 | rs71479745 |  |  |  |  |  |  |  |  |  |  |  |  |  |  |  |
| -0.432 | 10 | igd | 0.0765 | T | \|\| id:ebi-a-GCST90001489 | ebi-a-GCST90001489 | TRUE | G | 97667582 | 4.12E-19 | reported | 3405 | 0.04807 | rs41291578 |  |  |  |  |  |  |  |  |  |  |  |  |  |  |  |
| -0.3019 | 10 | igd | 0.0765 | C | \|\| id:ebi-a-GCST90002030 | ebi-a-GCST90002030 | TRUE | T | 96389285 | 8.60E-09 | reported | 2920 | 0.05229 | rs187722828 |  |  |  |  |  |  |  |  |  |  |  |  |  |  |  |
| -0.4095 | 10 | igd | 0.0764 | T | \|\| id:ebi-a-GCST90001483 | ebi-a-GCST90001483 | TRUE | C | 97570310 | 4.35E-18 | reported | 3405 | 0.04697 | rs117163620 |  |  |  |  |  |  |  |  |  |  |  |  |  |  |  |
| -0.4429 | 10 | igd | 0.0764 | T | \|\| id:ebi-a-GCST90001495 | ebi-a-GCST90001495 | TRUE | C | 97570310 | 1.31E-19 | reported | 3405 | 0.04859 | rs117163620 |  |  |  |  |  |  |  |  |  |  |  |  |  |  |  |
| 0.7053 | 6 | igd | 0.0764 | A | \|\| id:ebi-a-GCST90002105 | ebi-a-GCST90002105 | TRUE | G | 31105672 | 4.51E-33 | reported | 2872 | 0.05814 | rs2233954 |  |  |  |  |  |  |  |  |  |  |  |  |  |  |  |
| -0.4857 | 10 | igd | 0.0763 | T | \|\| id:ebi-a-GCST90001660 | ebi-a-GCST90001660 | TRUE | C | 97570310 | 7.74E-24 | reported | 3408 | 0.04789 | rs117163620 |  |  |  |  |  |  |  |  |  |  |  |  |  |  |  |
| -0.4729 | 10 | igd | 0.0762 | G | \|\| id:ebi-a-GCST90001671 | ebi-a-GCST90001671 | TRUE | A | 97584544 | 6.15E-23 | reported | 3440 | 0.04761 | rs17111156 |  |  |  |  |  |  |  |  |  |  |  |  |  |  |  |
| 0.2825 | 22 | igd | 0.0762 | A | \|\| id:ebi-a-GCST90001702 | ebi-a-GCST90001702 | TRUE | G | 41386788 | 6.18E-09 | reported | 3657 | 0.04849 | rs117990764 |  |  |  |  |  |  |  |  |  |  |  |  |  |  |  |
| 0.3352 | 22 | igd | 0.0762 | A | \|\| id:ebi-a-GCST90001702 | ebi-a-GCST90001702 | TRUE | G | 41733262 | 1.21E-12 | reported | 3657 | 0.04702 | rs138818147 |  |  |  |  |  |  |  |  |  |  |  |  |  |  |  |
| 0.2676 | 22 | igd | 0.0762 | A | \|\| id:ebi-a-GCST90001703 | ebi-a-GCST90001703 | TRUE | G | 41386788 | 3.69E-08 | reported | 3657 | 0.0485 | rs117990764 |  |  |  |  |  |  |  |  |  |  |  |  |  |  |  |
| 0.3313 | 22 | igd | 0.0762 | A | \|\| id:ebi-a-GCST90001703 | ebi-a-GCST90001703 | TRUE | G | 41733262 | 2.18E-12 | reported | 3657 | 0.04702 | rs138818147 |  |  |  |  |  |  |  |  |  |  |  |  |  |  |  |
| 0.3021 | 22 | igd | 0.0762 | A | \|\| id:ebi-a-GCST90001704 | ebi-a-GCST90001704 | TRUE | G | 41733262 | 1.48E-10 | reported | 3657 | 0.04701 | rs138818147 |  |  |  |  |  |  |  |  |  |  |  |  |  |  |  |
| 0.2858 | 22 | igd | 0.0762 | A | \|\| id:ebi-a-GCST90001710 | ebi-a-GCST90001710 | TRUE | G | 41733262 | 1.30E-09 | reported | 3657 | 0.04697 | rs138818147 |  |  |  |  |  |  |  |  |  |  |  |  |  |  |  |
| 0.303 | 22 | igd | 0.0762 | A | \|\| id:ebi-a-GCST90001711 | ebi-a-GCST90001711 | TRUE | G | 41733262 | 1.12E-10 | reported | 3657 | 0.04684 | rs138818147 |  |  |  |  |  |  |  |  |  |  |  |  |  |  |  |
| -0.4586 | 10 | igd | 0.0761 | T | \|\| id:ebi-a-GCST90001484 | ebi-a-GCST90001484 | TRUE | C | 97570310 | 1.30E-22 | reported | 3437 | 0.04653 | rs117163620 |  |  |  |  |  |  |  |  |  |  |  |  |  |  |  |
| -0.4591 | 10 | igd | 0.0761 | T | \|\| id:ebi-a-GCST90001485 | ebi-a-GCST90001485 | TRUE | C | 97570310 | 1.01E-22 | reported | 3437 | 0.04646 | rs117163620 |  |  |  |  |  |  |  |  |  |  |  |  |  |  |  |
| -0.4658 | 10 | igd | 0.0761 | T | \|\| id:ebi-a-GCST90001491 | ebi-a-GCST90001491 | TRUE | C | 97570310 | 1.62E-22 | reported | 3437 | 0.04737 | rs117163620 |  |  |  |  |  |  |  |  |  |  |  |  |  |  |  |
| -0.5175 | 10 | igd | 0.0761 | T | \|\| id:ebi-a-GCST90001496 | ebi-a-GCST90001496 | TRUE | C | 97570310 | 8.19E-27 | reported | 3437 | 0.04787 | rs117163620 |  |  |  |  |  |  |  |  |  |  |  |  |  |  |  |
| -0.4846 | 10 | igd | 0.0761 | T | \|\| id:ebi-a-GCST90001497 | ebi-a-GCST90001497 | TRUE | C | 97570310 | 3.10E-24 | reported | 3437 | 0.04735 | rs117163620 |  |  |  |  |  |  |  |  |  |  |  |  |  |  |  |
| -0.4773 | 10 | igd | 0.076 | T | \|\| id:ebi-a-GCST90001658 | ebi-a-GCST90001658 | TRUE | C | 97570310 | 3.53E-25 | reported | 3440 | 0.04568 | rs117163620 |  |  |  |  |  |  |  |  |  |  |  |  |  |  |  |
| -0.4752 | 10 | igd | 0.076 | T | \|\| id:ebi-a-GCST90001659 | ebi-a-GCST90001659 | TRUE | C | 97570310 | 5.70E-25 | reported | 3440 | 0.04569 | rs117163620 |  |  |  |  |  |  |  |  |  |  |  |  |  |  |  |
| -0.2892 | 1 | igd | 0.0759 | C | \|\| id:ebi-a-GCST90001860 | ebi-a-GCST90001860 | TRUE | T | 166494226 | 4.13E-08 | reported | 2919 | 0.05258 | rs77114151 |  |  |  |  |  |  |  |  |  |  |  |  |  |  |  |
| -0.3125 | 1 | igd | 0.0758 | A | \|\| id:ebi-a-GCST90001989 | ebi-a-GCST90001989 | TRUE | G | 161847528 | 6.85E-11 | reported | 3621 | 0.04775 | rs139376787 |  |  |  |  |  |  |  |  |  |  |  |  |  |  |  |
| -0.3919 | 22 | igd | 0.0757 | C | \|\| id:ebi-a-GCST90001702 | ebi-a-GCST90001702 | TRUE | A | 40549441 | 6.80E-16 | reported | 3657 | 0.04831 | rs62236804 |  |  |  |  |  |  |  |  |  |  |  |  |  |  |  |
| -0.3325 | 22 | igd | 0.0757 | C | \|\| id:ebi-a-GCST90001704 | ebi-a-GCST90001704 | TRUE | A | 40549441 | 7.22E-12 | reported | 3657 | 0.04835 | rs62236804 |  |  |  |  |  |  |  |  |  |  |  |  |  |  |  |
| -0.3677 | 22 | igd | 0.0757 | C | \|\| id:ebi-a-GCST90001710 | ebi-a-GCST90001710 | TRUE | A | 40549441 | 3.15E-14 | reported | 3657 | 0.04824 | rs62236804 |  |  |  |  |  |  |  |  |  |  |  |  |  |  |  |
| -0.3744 | 22 | igd | 0.0757 | C | \|\| id:ebi-a-GCST90001711 | ebi-a-GCST90001711 | TRUE | A | 40549441 | 8.96E-15 | reported | 3657 | 0.04809 | rs62236804 |  |  |  |  |  |  |  |  |  |  |  |  |  |  |  |
| -0.4743 | 10 | igd | 0.0756 | G | \|\| id:ebi-a-GCST90001672 | ebi-a-GCST90001672 | TRUE | C | 97653866 | 2.80E-22 | reported | 3408 | 0.04852 | rs139350575 |  |  |  |  |  |  |  |  |  |  |  |  |  |  |  |
| -0.5799 | 22 | igd | 0.0756 | A | \|\| id:ebi-a-GCST90001702 | ebi-a-GCST90001702 | TRUE | G | 43233960 | 2.02E-33 | reported | 3657 | 0.04765 | rs76712051 |  |  |  |  |  |  |  |  |  |  |  |  |  |  |  |
| -0.5653 | 22 | igd | 0.0756 | A | \|\| id:ebi-a-GCST90001703 | ebi-a-GCST90001703 | TRUE | G | 43233960 | 7.66E-32 | reported | 3657 | 0.04768 | rs76712051 |  |  |  |  |  |  |  |  |  |  |  |  |  |  |  |
| -0.2936 | 11 | igd | 0.0756 | T | \|\| id:ebi-a-GCST90002034 | ebi-a-GCST90002034 | TRUE | C | 5268498 | 4.79E-08 | reported | 2909 | 0.05364 | rs77346511 |  |  |  |  |  |  |  |  |  |  |  |  |  |  |  |
| 0.2896 | 10 | igd | 0.0755 | T | \|\| id:ebi-a-GCST90001961 | ebi-a-GCST90001961 | TRUE | G | 97667582 | 2.72E-08 | reported | 2920 | 0.05197 | rs41291578 |  |  |  |  |  |  |  |  |  |  |  |  |  |  |  |
| 0.2832 | 10 | igd | 0.0754 | A | \|\| id:ebi-a-GCST90001670 | ebi-a-GCST90001670 | TRUE | G | 100569678 | 3.40E-09 | reported | 3440 | 0.04779 | rs12220844 |  |  |  |  |  |  |  |  |  |  |  |  |  |  |  |
| 0.269 | 10 | igd | 0.0754 | A | \|\| id:ebi-a-GCST90001671 | ebi-a-GCST90001671 | TRUE | G | 100569678 | 2.05E-08 | reported | 3440 | 0.04786 | rs12220844 |  |  |  |  |  |  |  |  |  |  |  |  |  |  |  |
| -0.8062 | 12 | igd | 0.0754 | C | \|\| id:ebi-a-GCST90002074 | ebi-a-GCST90002074 | TRUE | G | 69612262 | 6.25E-59 | reported | 3112 | 0.04875 | rs79242862 |  |  |  |  |  |  |  |  |  |  |  |  |  |  |  |
| -0.4689 | 10 | igd | 0.0753 | G | \|\| id:ebi-a-GCST90001670 | ebi-a-GCST90001670 | TRUE | C | 97653866 | 1.80E-22 | reported | 3440 | 0.04775 | rs139350575 |  |  |  |  |  |  |  |  |  |  |  |  |  |  |  |
| -0.27 | 19 | igd | 0.0753 | C | \|\| id:ebi-a-GCST90001757 | ebi-a-GCST90001757 | TRUE | G | 49764961 | 1.37E-08 | reported | 3657 | 0.04745 | rs117372956 |  |  |  |  |  |  |  |  |  |  |  |  |  |  |  |
| 0.5127 | 10 | igd | 0.0753 | T | \|\| id:ebi-a-GCST90001777 | ebi-a-GCST90001777 | TRUE | C | 6170605 | 3.98E-28 | reported | 3657 | 0.04624 | rs77511007 |  |  |  |  |  |  |  |  |  |  |  |  |  |  |  |
| 0.5286 | 10 | igd | 0.0753 | T | \|\| id:ebi-a-GCST90001789 | ebi-a-GCST90001789 | TRUE | C | 6170605 | 3.27E-30 | reported | 3657 | 0.04587 | rs77511007 |  |  |  |  |  |  |  |  |  |  |  |  |  |  |  |
| 0.5124 | 10 | igd | 0.0753 | T | \|\| id:ebi-a-GCST90001790 | ebi-a-GCST90001790 | TRUE | C | 6170605 | 4.62E-28 | reported | 3657 | 0.04626 | rs77511007 |  |  |  |  |  |  |  |  |  |  |  |  |  |  |  |
| 0.5208 | 10 | igd | 0.0753 | T | \|\| id:ebi-a-GCST90001793 | ebi-a-GCST90001793 | TRUE | C | 6170605 | 1.63E-28 | reported | 3657 | 0.04662 | rs77511007 |  |  |  |  |  |  |  |  |  |  |  |  |  |  |  |
| -0.5968 | 10 | igd | 0.0753 | T | \|\| id:ebi-a-GCST90002030 | ebi-a-GCST90002030 | TRUE | C | 97570310 | 1.51E-31 | reported | 2920 | 0.05047 | rs117163620 |  |  |  |  |  |  |  |  |  |  |  |  |  |  |  |
| -0.6042 | 10 | igd | 0.0753 | T | \|\| id:ebi-a-GCST90002031 | ebi-a-GCST90002031 | TRUE | C | 97570310 | 9.28E-31 | reported | 2920 | 0.0518 | rs117163620 |  |  |  |  |  |  |  |  |  |  |  |  |  |  |  |
| -0.5689 | 10 | igd | 0.0753 | T | \|\| id:ebi-a-GCST90002032 | ebi-a-GCST90002032 | TRUE | C | 97570310 | 1.94E-27 | reported | 2920 | 0.05189 | rs117163620 |  |  |  |  |  |  |  |  |  |  |  |  |  |  |  |
| 0.2715 | 10 | igd | 0.0752 | T | \|\| id:ebi-a-GCST90001505 | ebi-a-GCST90001505 | TRUE | C | 6183022 | 2.04E-08 | reported | 3437 | 0.04829 | rs10905892 |  |  |  |  |  |  |  |  |  |  |  |  |  |  |  |
| -0.5253 | 22 | igd | 0.0752 | A | \|\| id:ebi-a-GCST90001704 | ebi-a-GCST90001704 | TRUE | G | 43212437 | 1.32E-27 | reported | 3657 | 0.04785 | rs78647462 |  |  |  |  |  |  |  |  |  |  |  |  |  |  |  |
| -0.5866 | 22 | igd | 0.0752 | A | \|\| id:ebi-a-GCST90001710 | ebi-a-GCST90001710 | TRUE | G | 43212437 | 3.55E-34 | reported | 3657 | 0.04764 | rs78647462 |  |  |  |  |  |  |  |  |  |  |  |  |  |  |  |
| 0.2831 | 10 | igd | 0.0752 | C | \|\| id:ebi-a-GCST90002030 | ebi-a-GCST90002030 | TRUE | G | 97209800 | 4.56E-08 | reported | 2920 | 0.05164 | rs117812273 |  |  |  |  |  |  |  |  |  |  |  |  |  |  |  |
| 0.3742 | 2 | igd | 0.0752 | T | \|\| id:ebi-a-GCST90002102 | ebi-a-GCST90002102 | TRUE | A | 38895956 | 2.02E-14 | reported | 2919 | 0.04867 | rs190580469 |  |  |  |  |  |  |  |  |  |  |  |  |  |  |  |
| -0.6392 | 2 | igd | 0.0752 | G | \|\| id:ebi-a-GCST90002119 | ebi-a-GCST90002119 | TRUE | T | 87056351 | 3.71E-38 | reported | 2920 | 0.04878 | rs113958550 |  |  |  |  |  |  |  |  |  |  |  |  |  |  |  |
| 0.2735 | 10 | igd | 0.0751 | A | \|\| id:ebi-a-GCST90001672 | ebi-a-GCST90001672 | TRUE | G | 100569678 | 2.02E-08 | reported | 3408 | 0.04864 | rs12220844 |  |  |  |  |  |  |  |  |  |  |  |  |  |  |  |
| 0.2871 | 22 | igd | 0.0751 | A | \|\| id:ebi-a-GCST90001704 | ebi-a-GCST90001704 | TRUE | G | 42566417 | 2.69E-09 | reported | 3657 | 0.04813 | rs73167200 |  |  |  |  |  |  |  |  |  |  |  |  |  |  |  |
| 0.3449 | 10 | igd | 0.0751 | A | \|\| id:ebi-a-GCST90001785 | ebi-a-GCST90001785 | TRUE | C | 6171591 | 2.98E-13 | reported | 3649 | 0.0471 | rs11256929 |  |  |  |  |  |  |  |  |  |  |  |  |  |  |  |
| -0.2927 | 10 | igd | 0.0751 | T | \|\| id:ebi-a-GCST90002029 | ebi-a-GCST90002029 | TRUE | C | 97570310 | 1.51E-08 | reported | 2915 | 0.05156 | rs117163620 |  |  |  |  |  |  |  |  |  |  |  |  |  |  |  |
| 0.2778 | 2 | igd | 0.0751 | T | \|\| id:ebi-a-GCST90002100 | ebi-a-GCST90002100 | TRUE | A | 38895956 | 4.41E-08 | reported | 2910 | 0.05061 | rs190580469 |  |  |  |  |  |  |  |  |  |  |  |  |  |  |  |
| 0.2682 | 22 | igd | 0.0749 | C | \|\| id:ebi-a-GCST90001711 | ebi-a-GCST90001711 | TRUE | G | 42611834 | 2.54E-08 | reported | 3657 | 0.04804 | rs73169123 |  |  |  |  |  |  |  |  |  |  |  |  |  |  |  |
| -0.2734 | 10 | igd | 0.0748 | A | \|\| id:ebi-a-GCST90001484 | ebi-a-GCST90001484 | TRUE | G | 98553063 | 1.24E-08 | reported | 3437 | 0.0479 | rs142616230 |  |  |  |  |  |  |  |  |  |  |  |  |  |  |  |
| -0.2772 | 10 | igd | 0.0748 | A | \|\| id:ebi-a-GCST90001496 | ebi-a-GCST90001496 | TRUE | G | 98553063 | 2.00E-08 | reported | 3437 | 0.04928 | rs142616230 |  |  |  |  |  |  |  |  |  |  |  |  |  |  |  |
| -0.2808 | 10 | igd | 0.0748 | A | \|\| id:ebi-a-GCST90001497 | ebi-a-GCST90001497 | TRUE | G | 98553063 | 8.51E-09 | reported | 3437 | 0.04865 | rs142616230 |  |  |  |  |  |  |  |  |  |  |  |  |  |  |  |
| 0.2746 | 10 | igd | 0.0748 | C | \|\| id:ebi-a-GCST90001660 | ebi-a-GCST90001660 | TRUE | T | 91995838 | 1.18E-08 | reported | 3408 | 0.04803 | rs146265359 |  |  |  |  |  |  |  |  |  |  |  |  |  |  |  |
| 0.277 | 10 | igd | 0.0748 | C | \|\| id:ebi-a-GCST90001672 | ebi-a-GCST90001672 | TRUE | T | 91995838 | 1.21E-08 | reported | 3408 | 0.0485 | rs146265359 |  |  |  |  |  |  |  |  |  |  |  |  |  |  |  |
| -0.3525 | 17 | igd | 0.0748 | A | \|\| id:ebi-a-GCST90001926 | ebi-a-GCST90001926 | TRUE | G | 57940703 | 1.43E-11 | reported | 2920 | 0.05197 | rs75440302 |  |  |  |  |  |  |  |  |  |  |  |  |  |  |  |
| 0.2872 | 10 | igd | 0.0747 | C | \|\| id:ebi-a-GCST90001483 | ebi-a-GCST90001483 | TRUE | T | 91995838 | 9.13E-10 | reported | 3405 | 0.04677 | rs146265359 |  |  |  |  |  |  |  |  |  |  |  |  |  |  |  |
| -0.5729 | 22 | igd | 0.0747 | T | \|\| id:ebi-a-GCST90001702 | ebi-a-GCST90001702 | TRUE | C | 41776453 | 8.31E-34 | reported | 3657 | 0.04679 | rs41281285 |  |  |  |  |  |  |  |  |  |  |  |  |  |  |  |
| -0.5603 | 22 | igd | 0.0747 | T | \|\| id:ebi-a-GCST90001703 | ebi-a-GCST90001703 | TRUE | C | 41776453 | 2.18E-32 | reported | 3657 | 0.04683 | rs41281285 |  |  |  |  |  |  |  |  |  |  |  |  |  |  |  |
| -0.5278 | 22 | igd | 0.0747 | T | \|\| id:ebi-a-GCST90001704 | ebi-a-GCST90001704 | TRUE | C | 41776453 | 6.07E-29 | reported | 3657 | 0.04687 | rs41281285 |  |  |  |  |  |  |  |  |  |  |  |  |  |  |  |
| -0.5268 | 22 | igd | 0.0747 | T | \|\| id:ebi-a-GCST90001710 | ebi-a-GCST90001710 | TRUE | C | 41776453 | 6.44E-29 | reported | 3657 | 0.0468 | rs41281285 |  |  |  |  |  |  |  |  |  |  |  |  |  |  |  |
| -0.5283 | 22 | igd | 0.0747 | T | \|\| id:ebi-a-GCST90001711 | ebi-a-GCST90001711 | TRUE | C | 41776453 | 3.39E-29 | reported | 3657 | 0.04669 | rs41281285 |  |  |  |  |  |  |  |  |  |  |  |  |  |  |  |
| -0.34 | 17 | igd | 0.0747 | C | \|\| id:ebi-a-GCST90001926 | ebi-a-GCST90001926 | TRUE | A | 58052124 | 7.43E-11 | reported | 2920 | 0.05203 | rs143669204 |  |  |  |  |  |  |  |  |  |  |  |  |  |  |  |
| 0.3127 | 10 | igd | 0.0746 | C | \|\| id:ebi-a-GCST90001658 | ebi-a-GCST90001658 | TRUE | T | 91995838 | 1.03E-11 | reported | 3440 | 0.04582 | rs146265359 |  |  |  |  |  |  |  |  |  |  |  |  |  |  |  |
| 0.3303 | 10 | igd | 0.0746 | C | \|\| id:ebi-a-GCST90001659 | ebi-a-GCST90001659 | TRUE | T | 91995838 | 6.67E-13 | reported | 3440 | 0.04579 | rs146265359 |  |  |  |  |  |  |  |  |  |  |  |  |  |  |  |
| 0.3036 | 10 | igd | 0.0746 | C | \|\| id:ebi-a-GCST90001670 | ebi-a-GCST90001670 | TRUE | T | 91995838 | 2.23E-10 | reported | 3440 | 0.0477 | rs146265359 |  |  |  |  |  |  |  |  |  |  |  |  |  |  |  |
| 0.3118 | 10 | igd | 0.0746 | C | \|\| id:ebi-a-GCST90001671 | ebi-a-GCST90001671 | TRUE | T | 91995838 | 7.78E-11 | reported | 3440 | 0.04779 | rs146265359 |  |  |  |  |  |  |  |  |  |  |  |  |  |  |  |
| 0.3376 | 10 | igd | 0.0745 | C | \|\| id:ebi-a-GCST90001484 | ebi-a-GCST90001484 | TRUE | T | 91995838 | 4.25E-13 | reported | 3437 | 0.04641 | rs146265359 |  |  |  |  |  |  |  |  |  |  |  |  |  |  |  |
| 0.3128 | 10 | igd | 0.0745 | C | \|\| id:ebi-a-GCST90001485 | ebi-a-GCST90001485 | TRUE | T | 91995838 | 1.82E-11 | reported | 3437 | 0.04639 | rs146265359 |  |  |  |  |  |  |  |  |  |  |  |  |  |  |  |
| 0.2965 | 10 | igd | 0.0745 | C | \|\| id:ebi-a-GCST90001496 | ebi-a-GCST90001496 | TRUE | T | 91995838 | 8.21E-10 | reported | 3437 | 0.04815 | rs146265359 |  |  |  |  |  |  |  |  |  |  |  |  |  |  |  |
| 0.2843 | 10 | igd | 0.0745 | C | \|\| id:ebi-a-GCST90001497 | ebi-a-GCST90001497 | TRUE | T | 91995838 | 2.48E-09 | reported | 3437 | 0.04756 | rs146265359 |  |  |  |  |  |  |  |  |  |  |  |  |  |  |  |
| -0.2771 | 10 | igd | 0.0745 | A | \|\| id:ebi-a-GCST90001672 | ebi-a-GCST90001672 | TRUE | T | 92023686 | 2.04E-08 | reported | 3408 | 0.04929 | rs117621776 |  |  |  |  |  |  |  |  |  |  |  |  |  |  |  |
| 0.4295 | 6 | igd | 0.0745 | A | \|\| id:ebi-a-GCST90002109 | ebi-a-GCST90002109 | TRUE | G | 31105672 | 1.24E-08 | reported | 1578 | 0.07503 | rs2233954 |  |  |  |  |  |  |  |  |  |  |  |  |  |  |  |
| -0.4362 | 22 | igd | 0.0744 | C | \|\| id:ebi-a-GCST90001702 | ebi-a-GCST90001702 | TRUE | T | 41013857 | 4.59E-19 | reported | 3657 | 0.04862 | rs112057490 |  |  |  |  |  |  |  |  |  |  |  |  |  |  |  |
| -0.4205 | 22 | igd | 0.0744 | C | \|\| id:ebi-a-GCST90001703 | ebi-a-GCST90001703 | TRUE | T | 41013857 | 7.91E-18 | reported | 3657 | 0.04864 | rs112057490 |  |  |  |  |  |  |  |  |  |  |  |  |  |  |  |
| -0.3713 | 22 | igd | 0.0744 | C | \|\| id:ebi-a-GCST90001704 | ebi-a-GCST90001704 | TRUE | T | 41013857 | 3.07E-14 | reported | 3657 | 0.04868 | rs112057490 |  |  |  |  |  |  |  |  |  |  |  |  |  |  |  |
| -0.4059 | 22 | igd | 0.0744 | C | \|\| id:ebi-a-GCST90001710 | ebi-a-GCST90001710 | TRUE | T | 41013857 | 8.85E-17 | reported | 3657 | 0.04856 | rs112057490 |  |  |  |  |  |  |  |  |  |  |  |  |  |  |  |
| -0.4135 | 22 | igd | 0.0744 | C | \|\| id:ebi-a-GCST90001711 | ebi-a-GCST90001711 | TRUE | T | 41013857 | 1.92E-17 | reported | 3657 | 0.04841 | rs112057490 |  |  |  |  |  |  |  |  |  |  |  |  |  |  |  |
| -0.2867 | 12 | igd | 0.0744 | A | \|\| id:ebi-a-GCST90002073 | ebi-a-GCST90002073 | TRUE | G | 68147533 | 3.58E-08 | reported | 2850 | 0.05188 | rs77837300 |  |  |  |  |  |  |  |  |  |  |  |  |  |  |  |
| 0.6243 | 6 | igd | 0.0744 | A | \|\| id:ebi-a-GCST90002108 | ebi-a-GCST90002108 | TRUE | G | 31105672 | 1.75E-16 | reported | 1579 | 0.07495 | rs2233954 |  |  |  |  |  |  |  |  |  |  |  |  |  |  |  |
| -0.3567 | 17 | igd | 0.0743 | A | \|\| id:ebi-a-GCST90001926 | ebi-a-GCST90001926 | TRUE | G | 57788608 | 8.06E-12 | reported | 2920 | 0.05196 | rs77913816 |  |  |  |  |  |  |  |  |  |  |  |  |  |  |  |
| -0.4787 | 22 | igd | 0.0742 | A | \|\| id:ebi-a-GCST90001702 | ebi-a-GCST90001702 | TRUE | G | 43468137 | 3.95E-23 | reported | 3657 | 0.048 | rs141435567 |  |  |  |  |  |  |  |  |  |  |  |  |  |  |  |
| -0.4685 | 22 | igd | 0.0742 | A | \|\| id:ebi-a-GCST90001703 | ebi-a-GCST90001703 | TRUE | G | 43468137 | 3.17E-22 | reported | 3657 | 0.04801 | rs141435567 |  |  |  |  |  |  |  |  |  |  |  |  |  |  |  |
| -0.381 | 22 | igd | 0.0742 | T | \|\| id:ebi-a-GCST90001703 | ebi-a-GCST90001703 | TRUE | C | 40567196 | 1.15E-14 | reported | 3657 | 0.04914 | rs113530150 |  |  |  |  |  |  |  |  |  |  |  |  |  |  |  |
| -0.4294 | 22 | igd | 0.0742 | A | \|\| id:ebi-a-GCST90001704 | ebi-a-GCST90001704 | TRUE | G | 43468137 | 6.15E-19 | reported | 3657 | 0.04804 | rs141435567 |  |  |  |  |  |  |  |  |  |  |  |  |  |  |  |
| -0.4622 | 22 | igd | 0.0742 | A | \|\| id:ebi-a-GCST90001710 | ebi-a-GCST90001710 | TRUE | G | 43468137 | 9.50E-22 | reported | 3657 | 0.04793 | rs141435567 |  |  |  |  |  |  |  |  |  |  |  |  |  |  |  |
| -0.4667 | 22 | igd | 0.0742 | A | \|\| id:ebi-a-GCST90001711 | ebi-a-GCST90001711 | TRUE | G | 43468137 | 2.81E-22 | reported | 3657 | 0.04776 | rs141435567 |  |  |  |  |  |  |  |  |  |  |  |  |  |  |  |
| 0.3092 | 10 | igd | 0.0741 | A | \|\| id:ebi-a-GCST90002029 | ebi-a-GCST90002029 | TRUE | G | 97614026 | 1.33E-09 | reported | 2915 | 0.05083 | rs189315256 |  |  |  |  |  |  |  |  |  |  |  |  |  |  |  |
| 0.3086 | 10 | igd | 0.074 | A | \|\| id:ebi-a-GCST90001892 | ebi-a-GCST90001892 | TRUE | G | 97614026 | 1.93E-09 | reported | 2920 | 0.05124 | rs189315256 |  |  |  |  |  |  |  |  |  |  |  |  |  |  |  |
| 0.3222 | 19 | igd | 0.07383 | G | \|\| id:prot-a-439 | prot-a-439 | TRUE | A | 51818606 | 8.51E-12 | reported | 3301 | 0.0472 | rs10419464 |  |  |  |  |  |  |  |  |  |  |  |  |  |  |  |
| -0.2975 | 10 | igd | 0.0738 | A | \|\| id:ebi-a-GCST90001671 | ebi-a-GCST90001671 | TRUE | T | 92023686 | 1.06E-09 | reported | 3440 | 0.04862 | rs117621776 |  |  |  |  |  |  |  |  |  |  |  |  |  |  |  |
| -0.281 | 11 | igd | 0.0738 | A | \|\| id:ebi-a-GCST90001752 | ebi-a-GCST90001752 | TRUE | G | 60125440 | 1.76E-09 | reported | 3657 | 0.04657 | rs17154838 |  |  |  |  |  |  |  |  |  |  |  |  |  |  |  |
| 0.2839 | 16 | igd | 0.0735 | C | \|\| id:ebi-a-GCST90001863 | ebi-a-GCST90001863 | TRUE | T | 50264054 | 3.92E-08 | reported | 2919 | 0.05153 | rs62028293 |  |  |  |  |  |  |  |  |  |  |  |  |  |  |  |
| 0.5046 | 19 | igd | 0.0734 | T | \|\| id:ebi-a-GCST90001955 | ebi-a-GCST90001955 | TRUE | C | 51868443 | 5.21E-12 | reported | 1554 | 0.07256 | rs58483275 |  |  |  |  |  |  |  |  |  |  |  |  |  |  |  |
| 0.6163 | 6 | igd | 0.0734 | A | \|\| id:ebi-a-GCST90002112 | ebi-a-GCST90002112 | TRUE | G | 31105672 | 4.02E-16 | reported | 1635 | 0.07496 | rs2233954 |  |  |  |  |  |  |  |  |  |  |  |  |  |  |  |
| -0.3203 | 22 | igd | 0.0733 | T | \|\| id:ebi-a-GCST90001704 | ebi-a-GCST90001704 | TRUE | C | 39738379 | 1.17E-10 | reported | 3657 | 0.04956 | rs117458132 |  |  |  |  |  |  |  |  |  |  |  |  |  |  |  |
| -0.3838 | 22 | igd | 0.0733 | T | \|\| id:ebi-a-GCST90001711 | ebi-a-GCST90001711 | TRUE | C | 39738379 | 8.47E-15 | reported | 3657 | 0.04925 | rs117458132 |  |  |  |  |  |  |  |  |  |  |  |  |  |  |  |
| 0.5176 | 19 | igd | 0.0733 | T | \|\| id:ebi-a-GCST90001954 | ebi-a-GCST90001954 | TRUE | C | 51868443 | 1.33E-12 | reported | 1555 | 0.07238 | rs58483275 |  |  |  |  |  |  |  |  |  |  |  |  |  |  |  |
| 0.3152 | 2 | igd | 0.0732 | T | \|\| id:ebi-a-GCST90001508 | ebi-a-GCST90001508 | TRUE | A | 38895956 | 1.67E-10 | reported | 3437 | 0.04919 | rs190580469 |  |  |  |  |  |  |  |  |  |  |  |  |  |  |  |
| 0.3036 | 2 | igd | 0.0732 | T | \|\| id:ebi-a-GCST90001509 | ebi-a-GCST90001509 | TRUE | A | 38895956 | 7.46E-10 | reported | 3437 | 0.04918 | rs190580469 |  |  |  |  |  |  |  |  |  |  |  |  |  |  |  |
| 0.2691 | 2 | igd | 0.0732 | T | \|\| id:ebi-a-GCST90001561 | ebi-a-GCST90001561 | TRUE | A | 38895956 | 9.65E-11 | reported | 3427 | 0.04144 | rs190580469 |  |  |  |  |  |  |  |  |  |  |  |  |  |  |  |
| 0.5135 | 19 | igd | 0.0732 | T | \|\| id:ebi-a-GCST90001953 | ebi-a-GCST90001953 | TRUE | C | 51868443 | 1.97E-12 | reported | 1558 | 0.07239 | rs58483275 |  |  |  |  |  |  |  |  |  |  |  |  |  |  |  |
| 0.2784 | 10 | igd | 0.0731 | A | \|\| id:ebi-a-GCST90001658 | ebi-a-GCST90001658 | TRUE | C | 93057020 | 5.86E-09 | reported | 3440 | 0.0477 | rs2631687 |  |  |  |  |  |  |  |  |  |  |  |  |  |  |  |
| 0.3234 | 10 | igd | 0.0731 | A | \|\| id:ebi-a-GCST90001659 | ebi-a-GCST90001659 | TRUE | C | 93057020 | 1.31E-11 | reported | 3440 | 0.04763 | rs2631687 |  |  |  |  |  |  |  |  |  |  |  |  |  |  |  |
| 0.3109 | 10 | igd | 0.0731 | A | \|\| id:ebi-a-GCST90001660 | ebi-a-GCST90001660 | TRUE | C | 93057020 | 5.86E-10 | reported | 3408 | 0.05005 | rs2631687 |  |  |  |  |  |  |  |  |  |  |  |  |  |  |  |
| -0.267 | 2 | igd | 0.0731 | T | \|\| id:ebi-a-GCST90001664 | ebi-a-GCST90001664 | TRUE | G | 38431267 | 3.34E-09 | reported | 3440 | 0.04503 | rs79827501 |  |  |  |  |  |  |  |  |  |  |  |  |  |  |  |
| -3.732 | 2 | igd | 0.0731 | T | \|\| id:ebi-a-GCST90001696 | ebi-a-GCST90001696 | TRUE | A | 38895956 | 1.75E-10 | reported | 3425 | 0.583 | rs190580469 |  |  |  |  |  |  |  |  |  |  |  |  |  |  |  |
| 0.3028 | 7 | igd | 0.0731 | T | \|\| id:ebi-a-GCST90001926 | ebi-a-GCST90001926 | TRUE | C | 92300568 | 7.97E-09 | reported | 2920 | 0.05233 | rs3731332 |  |  |  |  |  |  |  |  |  |  |  |  |  |  |  |
| -0.4764 | 19 | igd | 0.0731 | G | \|\| id:ebi-a-GCST90001946 | ebi-a-GCST90001946 | TRUE | T | 52897142 | 3.79E-12 | reported | 1634 | 0.06809 | rs11882720 |  |  |  |  |  |  |  |  |  |  |  |  |  |  |  |
| -0.4228 | 19 | igd | 0.0731 | G | \|\| id:ebi-a-GCST90001949 | ebi-a-GCST90001949 | TRUE | T | 52897142 | 5.93E-10 | reported | 1634 | 0.06787 | rs11882720 |  |  |  |  |  |  |  |  |  |  |  |  |  |  |  |
| 0.2749 | 10 | igd | 0.073 | A | \|\| id:ebi-a-GCST90001483 | ebi-a-GCST90001483 | TRUE | C | 93057020 | 2.21E-08 | reported | 3405 | 0.04902 | rs2631687 |  |  |  |  |  |  |  |  |  |  |  |  |  |  |  |
| 0.364 | 10 | igd | 0.073 | A | \|\| id:ebi-a-GCST90001484 | ebi-a-GCST90001484 | TRUE | C | 93057020 | 6.96E-14 | reported | 3437 | 0.04841 | rs2631687 |  |  |  |  |  |  |  |  |  |  |  |  |  |  |  |
| 0.3175 | 10 | igd | 0.073 | A | \|\| id:ebi-a-GCST90001485 | ebi-a-GCST90001485 | TRUE | C | 93057020 | 6.38E-11 | reported | 3437 | 0.04843 | rs2631687 |  |  |  |  |  |  |  |  |  |  |  |  |  |  |  |
| 0.4863 | 10 | igd | 0.073 | A | \|\| id:ebi-a-GCST90001489 | ebi-a-GCST90001489 | TRUE | G | 97614026 | 7.37E-23 | reported | 3405 | 0.04905 | rs189315256 |  |  |  |  |  |  |  |  |  |  |  |  |  |  |  |
| 0.2976 | 10 | igd | 0.073 | A | \|\| id:ebi-a-GCST90001495 | ebi-a-GCST90001495 | TRUE | C | 93057020 | 4.89E-09 | reported | 3405 | 0.05073 | rs2631687 |  |  |  |  |  |  |  |  |  |  |  |  |  |  |  |
| 0.3678 | 10 | igd | 0.073 | A | \|\| id:ebi-a-GCST90001496 | ebi-a-GCST90001496 | TRUE | C | 93057020 | 2.24E-13 | reported | 3437 | 0.04994 | rs2631687 |  |  |  |  |  |  |  |  |  |  |  |  |  |  |  |
| 0.3665 | 10 | igd | 0.073 | A | \|\| id:ebi-a-GCST90001497 | ebi-a-GCST90001497 | TRUE | C | 93057020 | 1.34E-13 | reported | 3437 | 0.04931 | rs2631687 |  |  |  |  |  |  |  |  |  |  |  |  |  |  |  |
| 0.2762 | 3 | igd | 0.073 | C | \|\| id:ebi-a-GCST90001798 | ebi-a-GCST90001798 | TRUE | T | 33189782 | 1.58E-08 | reported | 3657 | 0.04874 | rs79991185 |  |  |  |  |  |  |  |  |  |  |  |  |  |  |  |
| 0.2834 | 3 | igd | 0.073 | C | \|\| id:ebi-a-GCST90001806 | ebi-a-GCST90001806 | TRUE | T | 33189782 | 8.84E-09 | reported | 3656 | 0.04916 | rs79991185 |  |  |  |  |  |  |  |  |  |  |  |  |  |  |  |
| 0.3121 | 20 | igd | 0.073 | C | \|\| id:ebi-a-GCST90001981 | ebi-a-GCST90001981 | TRUE | T | 44615889 | 3.89E-11 | reported | 3618 | 0.04708 | rs74616372 |  |  |  |  |  |  |  |  |  |  |  |  |  |  |  |
| -0.347 | 1 | igd | 0.0729 | T | \|\| id:ebi-a-GCST90001999 | ebi-a-GCST90001999 | TRUE | G | 161570970 | 6.02E-12 | reported | 3621 | 0.05027 | rs546183406 |  |  |  |  |  |  |  |  |  |  |  |  |  |  |  |
| 0.4369 | 7 | igd | 0.0728 | T | \|\| id:ebi-a-GCST90001966 | ebi-a-GCST90001966 | TRUE | C | 92300568 | 2.06E-16 | reported | 2850 | 0.05284 | rs3731332 |  |  |  |  |  |  |  |  |  |  |  |  |  |  |  |
| 0.3191 | 20 | igd | 0.0728 | C | \|\| id:ebi-a-GCST90001989 | ebi-a-GCST90001989 | TRUE | T | 44615889 | 1.76E-11 | reported | 3621 | 0.0473 | rs74616372 |  |  |  |  |  |  |  |  |  |  |  |  |  |  |  |
| 0.4939 | 6 | igd | 0.0728 | A | \|\| id:ebi-a-GCST90002110 | ebi-a-GCST90002110 | TRUE | T | 32438751 | 4.97E-08 | reported | 1634 | 0.09016 | rs180843960 |  |  |  |  |  |  |  |  |  |  |  |  |  |  |  |
| -0.2744 | 11 | igd | 0.0727 | G | \|\| id:ebi-a-GCST90001743 | ebi-a-GCST90001743 | TRUE | T | 60066539 | 8.88E-09 | reported | 3660 | 0.0476 | rs12146430 |  |  |  |  |  |  |  |  |  |  |  |  |  |  |  |
| -0.2854 | 11 | igd | 0.0727 | G | \|\| id:ebi-a-GCST90001762 | ebi-a-GCST90001762 | TRUE | T | 60066539 | 2.01E-09 | reported | 3658 | 0.04747 | rs12146430 |  |  |  |  |  |  |  |  |  |  |  |  |  |  |  |
| 0.3759 | 20 | igd | 0.0727 | C | \|\| id:ebi-a-GCST90001985 | ebi-a-GCST90001985 | TRUE | T | 44615889 | 2.26E-15 | reported | 3629 | 0.04722 | rs74616372 |  |  |  |  |  |  |  |  |  |  |  |  |  |  |  |
| -0.3345 | 22 | igd | 0.0726 | A | \|\| id:ebi-a-GCST90001702 | ebi-a-GCST90001702 | TRUE | G | 44073254 | 6.55E-12 | reported | 3657 | 0.04855 | rs112374923 |  |  |  |  |  |  |  |  |  |  |  |  |  |  |  |
| -0.3886 | 22 | igd | 0.0726 | A | \|\| id:ebi-a-GCST90001702 | ebi-a-GCST90001702 | TRUE | G | 39704126 | 1.14E-14 | reported | 3657 | 0.05011 | rs149688578 |  |  |  |  |  |  |  |  |  |  |  |  |  |  |  |
| -0.3284 | 22 | igd | 0.0726 | A | \|\| id:ebi-a-GCST90001703 | ebi-a-GCST90001703 | TRUE | G | 44073254 | 1.56E-11 | reported | 3657 | 0.04855 | rs112374923 |  |  |  |  |  |  |  |  |  |  |  |  |  |  |  |
| -0.3632 | 22 | igd | 0.0726 | A | \|\| id:ebi-a-GCST90001703 | ebi-a-GCST90001703 | TRUE | G | 39704126 | 5.37E-13 | reported | 3657 | 0.05015 | rs149688578 |  |  |  |  |  |  |  |  |  |  |  |  |  |  |  |
| -0.31 | 22 | igd | 0.0726 | A | \|\| id:ebi-a-GCST90001704 | ebi-a-GCST90001704 | TRUE | G | 44073254 | 1.87E-10 | reported | 3657 | 0.04852 | rs112374923 |  |  |  |  |  |  |  |  |  |  |  |  |  |  |  |
| -0.4003 | 22 | igd | 0.0726 | A | \|\| id:ebi-a-GCST90001710 | ebi-a-GCST90001710 | TRUE | G | 39704126 | 1.51E-15 | reported | 3657 | 0.04997 | rs149688578 |  |  |  |  |  |  |  |  |  |  |  |  |  |  |  |
| -0.3151 | 22 | igd | 0.0726 | A | \|\| id:ebi-a-GCST90001710 | ebi-a-GCST90001710 | TRUE | G | 44073254 | 8.93E-11 | reported | 3657 | 0.04846 | rs112374923 |  |  |  |  |  |  |  |  |  |  |  |  |  |  |  |
| -0.3296 | 22 | igd | 0.0726 | A | \|\| id:ebi-a-GCST90001711 | ebi-a-GCST90001711 | TRUE | G | 44073254 | 1.03E-11 | reported | 3657 | 0.0483 | rs112374923 |  |  |  |  |  |  |  |  |  |  |  |  |  |  |  |
| -0.2605 | 11 | igd | 0.0726 | G | \|\| id:ebi-a-GCST90001745 | ebi-a-GCST90001745 | TRUE | T | 60066539 | 4.13E-08 | reported | 3657 | 0.04739 | rs12146430 |  |  |  |  |  |  |  |  |  |  |  |  |  |  |  |
| -0.2941 | 11 | igd | 0.0726 | G | \|\| id:ebi-a-GCST90001747 | ebi-a-GCST90001747 | TRUE | T | 60066539 | 6.52E-10 | reported | 3657 | 0.04749 | rs12146430 |  |  |  |  |  |  |  |  |  |  |  |  |  |  |  |
| -0.3213 | 11 | igd | 0.0726 | G | \|\| id:ebi-a-GCST90001752 | ebi-a-GCST90001752 | TRUE | T | 60066539 | 1.38E-11 | reported | 3657 | 0.04738 | rs12146430 |  |  |  |  |  |  |  |  |  |  |  |  |  |  |  |
| -0.2975 | 11 | igd | 0.0726 | G | \|\| id:ebi-a-GCST90001759 | ebi-a-GCST90001759 | TRUE | T | 60066539 | 4.25E-10 | reported | 3657 | 0.04751 | rs12146430 |  |  |  |  |  |  |  |  |  |  |  |  |  |  |  |
| -0.3452 | 2 | igd | 0.0726 | T | \|\| id:ebi-a-GCST90001890 | ebi-a-GCST90001890 | TRUE | C | 203835790 | 1.51E-10 | reported | 2920 | 0.05371 | rs62183744 |  |  |  |  |  |  |  |  |  |  |  |  |  |  |  |
| -0.3151 | 2 | igd | 0.0726 | T | \|\| id:ebi-a-GCST90001894 | ebi-a-GCST90001894 | TRUE | C | 203835790 | 3.38E-09 | reported | 2920 | 0.05314 | rs62183744 |  |  |  |  |  |  |  |  |  |  |  |  |  |  |  |
| -0.3098 | 2 | igd | 0.0726 | T | \|\| id:ebi-a-GCST90001898 | ebi-a-GCST90001898 | TRUE | C | 203835790 | 6.29E-09 | reported | 2920 | 0.05318 | rs62183744 |  |  |  |  |  |  |  |  |  |  |  |  |  |  |  |
| -0.366 | 2 | igd | 0.0726 | T | \|\| id:ebi-a-GCST90001900 | ebi-a-GCST90001900 | TRUE | C | 203835790 | 6.24E-12 | reported | 2919 | 0.05303 | rs62183744 |  |  |  |  |  |  |  |  |  |  |  |  |  |  |  |
| -0.4484 | 19 | igd | 0.0726 | G | \|\| id:ebi-a-GCST90001948 | ebi-a-GCST90001948 | TRUE | T | 52897142 | 7.43E-11 | reported | 1633 | 0.06841 | rs11882720 |  |  |  |  |  |  |  |  |  |  |  |  |  |  |  |
| -0.6251 | 6 | igd | 0.0726 | T | \|\| id:ebi-a-GCST90002105 | ebi-a-GCST90002105 | TRUE | C | 32592796 | 5.73E-29 | reported | 2872 | 0.05535 | rs28383322 |  |  |  |  |  |  |  |  |  |  |  |  |  |  |  |
| 0.3158 | 10 | igd | 0.0723 | G | \|\| id:ebi-a-GCST90001660 | ebi-a-GCST90001660 | TRUE | A | 95796101 | 6.88E-11 | reported | 3408 | 0.04826 | rs143635041 |  |  |  |  |  |  |  |  |  |  |  |  |  |  |  |
| 0.3076 | 10 | igd | 0.0723 | G | \|\| id:ebi-a-GCST90001672 | ebi-a-GCST90001672 | TRUE | A | 95796101 | 3.18E-10 | reported | 3408 | 0.04876 | rs143635041 |  |  |  |  |  |  |  |  |  |  |  |  |  |  |  |
| -0.4422 | 22 | igd | 0.0723 | T | \|\| id:ebi-a-GCST90001702 | ebi-a-GCST90001702 | TRUE | C | 40742686 | 5.41E-19 | reported | 3657 | 0.0494 | rs2228415 |  |  |  |  |  |  |  |  |  |  |  |  |  |  |  |
| -0.4207 | 22 | igd | 0.0723 | T | \|\| id:ebi-a-GCST90001703 | ebi-a-GCST90001703 | TRUE | C | 40742686 | 2.48E-17 | reported | 3657 | 0.04944 | rs2228415 |  |  |  |  |  |  |  |  |  |  |  |  |  |  |  |
| -0.3705 | 22 | igd | 0.0723 | T | \|\| id:ebi-a-GCST90001704 | ebi-a-GCST90001704 | TRUE | C | 40742686 | 8.68E-14 | reported | 3657 | 0.04947 | rs2228415 |  |  |  |  |  |  |  |  |  |  |  |  |  |  |  |
| -0.4028 | 22 | igd | 0.0723 | T | \|\| id:ebi-a-GCST90001710 | ebi-a-GCST90001710 | TRUE | C | 40742686 | 4.58E-16 | reported | 3657 | 0.04936 | rs2228415 |  |  |  |  |  |  |  |  |  |  |  |  |  |  |  |
| -0.41 | 22 | igd | 0.0723 | T | \|\| id:ebi-a-GCST90001711 | ebi-a-GCST90001711 | TRUE | C | 40742686 | 1.11E-16 | reported | 3657 | 0.0492 | rs2228415 |  |  |  |  |  |  |  |  |  |  |  |  |  |  |  |
| -0.3419 | 1 | igd | 0.0723 | C | \|\| id:ebi-a-GCST90001864 | ebi-a-GCST90001864 | TRUE | G | 167138220 | 3.10E-11 | reported | 2919 | 0.05128 | rs75015075 |  |  |  |  |  |  |  |  |  |  |  |  |  |  |  |
| 0.3137 | 10 | igd | 0.0722 | G | \|\| id:ebi-a-GCST90001495 | ebi-a-GCST90001495 | TRUE | A | 95796101 | 1.62E-10 | reported | 3405 | 0.04892 | rs143635041 |  |  |  |  |  |  |  |  |  |  |  |  |  |  |  |
| -0.2603 | 2 | igd | 0.0722 | C | \|\| id:ebi-a-GCST90001664 | ebi-a-GCST90001664 | TRUE | G | 38582808 | 1.09E-08 | reported | 3440 | 0.04542 | rs111804320 |  |  |  |  |  |  |  |  |  |  |  |  |  |  |  |
| 0.4023 | 19 | igd | 0.0722 | T | \|\| id:ebi-a-GCST90001957 | ebi-a-GCST90001957 | TRUE | A | 51868925 | 4.58E-08 | reported | 1578 | 0.07324 | rs73560177 |  |  |  |  |  |  |  |  |  |  |  |  |  |  |  |
| -0.3276 | 10 | igd | 0.0721 | T | \|\| id:ebi-a-GCST90001934 | ebi-a-GCST90001934 | TRUE | C | 6102012 | 9.86E-12 | reported | 3435 | 0.04795 | rs12722489 |  |  |  |  |  |  |  |  |  |  |  |  |  |  |  |
| 0.4015 | 10 | igd | 0.0719 | G | \|\| id:ebi-a-GCST90001484 | ebi-a-GCST90001484 | TRUE | A | 95796101 | 1.63E-17 | reported | 3437 | 0.04688 | rs143635041 |  |  |  |  |  |  |  |  |  |  |  |  |  |  |  |
| 0.2824 | 10 | igd | 0.0719 | G | \|\| id:ebi-a-GCST90001491 | ebi-a-GCST90001491 | TRUE | A | 95796101 | 3.80E-09 | reported | 3437 | 0.0478 | rs143635041 |  |  |  |  |  |  |  |  |  |  |  |  |  |  |  |
| 0.3958 | 10 | igd | 0.0719 | G | \|\| id:ebi-a-GCST90001496 | ebi-a-GCST90001496 | TRUE | A | 95796101 | 3.14E-16 | reported | 3437 | 0.04821 | rs143635041 |  |  |  |  |  |  |  |  |  |  |  |  |  |  |  |
| 0.3928 | 10 | igd | 0.0719 | G | \|\| id:ebi-a-GCST90001497 | ebi-a-GCST90001497 | TRUE | A | 95796101 | 2.22E-16 | reported | 3437 | 0.0476 | rs143635041 |  |  |  |  |  |  |  |  |  |  |  |  |  |  |  |
| 0.3355 | 10 | igd | 0.0719 | G | \|\| id:ebi-a-GCST90001658 | ebi-a-GCST90001658 | TRUE | A | 95796101 | 3.55E-13 | reported | 3440 | 0.04596 | rs143635041 |  |  |  |  |  |  |  |  |  |  |  |  |  |  |  |
| 0.3626 | 10 | igd | 0.0719 | G | \|\| id:ebi-a-GCST90001659 | ebi-a-GCST90001659 | TRUE | A | 95796101 | 3.79E-15 | reported | 3440 | 0.04591 | rs143635041 |  |  |  |  |  |  |  |  |  |  |  |  |  |  |  |
| 0.3129 | 10 | igd | 0.0719 | G | \|\| id:ebi-a-GCST90001670 | ebi-a-GCST90001670 | TRUE | A | 95796101 | 8.03E-11 | reported | 3440 | 0.04799 | rs143635041 |  |  |  |  |  |  |  |  |  |  |  |  |  |  |  |
| 0.3046 | 10 | igd | 0.0719 | G | \|\| id:ebi-a-GCST90001671 | ebi-a-GCST90001671 | TRUE | A | 95796101 | 2.49E-10 | reported | 3440 | 0.048 | rs143635041 |  |  |  |  |  |  |  |  |  |  |  |  |  |  |  |
| -0.2985 | 16 | igd | 0.0719 | T | \|\| id:ebi-a-GCST90002087 | ebi-a-GCST90002087 | TRUE | C | 31624520 | 1.59E-08 | reported | 2872 | 0.05267 | rs147258092 |  |  |  |  |  |  |  |  |  |  |  |  |  |  |  |
| -0.3132 | 16 | igd | 0.0719 | T | \|\| id:ebi-a-GCST90002088 | ebi-a-GCST90002088 | TRUE | C | 31624520 | 2.69E-09 | reported | 2872 | 0.05248 | rs147258092 |  |  |  |  |  |  |  |  |  |  |  |  |  |  |  |
| -0.2955 | 1 | igd | 0.0718 | T | \|\| id:ebi-a-GCST90001467 | ebi-a-GCST90001467 | TRUE | G | 161570970 | 3.19E-09 | reported | 3410 | 0.04978 | rs546183406 |  |  |  |  |  |  |  |  |  |  |  |  |  |  |  |
| -0.4855 | 1 | igd | 0.0718 | T | \|\| id:ebi-a-GCST90001474 | ebi-a-GCST90001474 | TRUE | G | 161570970 | 3.20E-22 | reported | 3410 | 0.04974 | rs546183406 |  |  |  |  |  |  |  |  |  |  |  |  |  |  |  |
| 0.2712 | 3 | igd | 0.0718 | G | \|\| id:ebi-a-GCST90001806 | ebi-a-GCST90001806 | TRUE | A | 36349691 | 3.82E-08 | reported | 3656 | 0.04922 | rs144642376 |  |  |  |  |  |  |  |  |  |  |  |  |  |  |  |
| 0.2781 | 3 | igd | 0.0718 | G | \|\| id:ebi-a-GCST90001808 | ebi-a-GCST90001808 | TRUE | A | 36349691 | 1.69E-08 | reported | 3657 | 0.0492 | rs144642376 |  |  |  |  |  |  |  |  |  |  |  |  |  |  |  |
| 0.3548 | 1 | igd | 0.0718 | T | \|\| id:ebi-a-GCST90002035 | ebi-a-GCST90002035 | TRUE | G | 161570970 | 7.00E-11 | reported | 2871 | 0.0542 | rs546183406 |  |  |  |  |  |  |  |  |  |  |  |  |  |  |  |
| -0.2659 | 1 | igd | 0.0718 | A | \|\| id:ebi-a-GCST90001999 | ebi-a-GCST90001999 | TRUE | G | 161659801 | 3.16E-08 | reported | 3621 | 0.04797 | rs114931935 |  |  |  |  |  |  |  |  |  |  |  |  |  |  |  |
| 0.267 | 20 | igd | 0.0716 | T | \|\| id:ebi-a-GCST90001985 | ebi-a-GCST90001985 | TRUE | G | 46701225 | 4.83E-08 | reported | 3629 | 0.04883 | rs11699356 |  |  |  |  |  |  |  |  |  |  |  |  |  |  |  |
| 0.533 | 10 | igd | 0.0716 | G | \|\| id:ebi-a-GCST90002030 | ebi-a-GCST90002030 | TRUE | A | 95796101 | 4.81E-25 | reported | 2920 | 0.05108 | rs143635041 |  |  |  |  |  |  |  |  |  |  |  |  |  |  |  |
| 0.512 | 10 | igd | 0.0716 | G | \|\| id:ebi-a-GCST90002031 | ebi-a-GCST90002031 | TRUE | A | 95796101 | 4.45E-22 | reported | 2920 | 0.05257 | rs143635041 |  |  |  |  |  |  |  |  |  |  |  |  |  |  |  |
| 0.4532 | 10 | igd | 0.0716 | G | \|\| id:ebi-a-GCST90002032 | ebi-a-GCST90002032 | TRUE | A | 95796101 | 1.29E-17 | reported | 2920 | 0.0527 | rs143635041 |  |  |  |  |  |  |  |  |  |  |  |  |  |  |  |
| -0.3128 | 2 | igd | 0.0716 | A | \|\| id:ebi-a-GCST90002098 | ebi-a-GCST90002098 | TRUE | G | 38930440 | 1.22E-09 | reported | 2910 | 0.05131 | rs189477959 |  |  |  |  |  |  |  |  |  |  |  |  |  |  |  |
| -0.3619 | 1 | igd | 0.0714 | T | \|\| id:ebi-a-GCST90002013 | ebi-a-GCST90002013 | TRUE | G | 161570970 | 5.33E-11 | reported | 2870 | 0.05494 | rs546183406 |  |  |  |  |  |  |  |  |  |  |  |  |  |  |  |
| -0.3923 | 19 | igd | 0.0712 | C | \|\| id:ebi-a-GCST90001956 | ebi-a-GCST90001956 | TRUE | T | 52094516 | 3.15E-08 | reported | 1579 | 0.07054 | rs6509556 |  |  |  |  |  |  |  |  |  |  |  |  |  |  |  |
| -0.5042 | 1 | igd | 0.071 | T | \|\| id:ebi-a-GCST90001970 | ebi-a-GCST90001970 | TRUE | G | 161570970 | 1.01E-20 | reported | 2971 | 0.05361 | rs546183406 |  |  |  |  |  |  |  |  |  |  |  |  |  |  |  |
| -0.4881 | 1 | igd | 0.071 | T | \|\| id:ebi-a-GCST90002077 | ebi-a-GCST90002077 | TRUE | G | 161570970 | 8.31E-20 | reported | 2971 | 0.0532 | rs546183406 |  |  |  |  |  |  |  |  |  |  |  |  |  |  |  |
| 0.3863 | 7 | igd | 0.071 | T | \|\| id:ebi-a-GCST90002078 | ebi-a-GCST90002078 | TRUE | C | 92300568 | 2.14E-14 | reported | 3112 | 0.05031 | rs3731332 |  |  |  |  |  |  |  |  |  |  |  |  |  |  |  |
| -0.3037 | 2 | igd | 0.071 | T | \|\| id:ebi-a-GCST90002098 | ebi-a-GCST90002098 | TRUE | C | 39035295 | 4.54E-09 | reported | 2910 | 0.05165 | rs182431516 |  |  |  |  |  |  |  |  |  |  |  |  |  |  |  |
| -0.3327 | 2 | igd | 0.0709 | C | \|\| id:ebi-a-GCST90001887 | ebi-a-GCST90001887 | TRUE | T | 204826646 | 3.51E-10 | reported | 2920 | 0.05284 | rs62177481 |  |  |  |  |  |  |  |  |  |  |  |  |  |  |  |
| -0.3043 | 2 | igd | 0.0709 | C | \|\| id:ebi-a-GCST90001889 | ebi-a-GCST90001889 | TRUE | T | 204826646 | 4.43E-09 | reported | 2920 | 0.0517 | rs62177481 |  |  |  |  |  |  |  |  |  |  |  |  |  |  |  |
| -0.3084 | 2 | igd | 0.0709 | C | \|\| id:ebi-a-GCST90001890 | ebi-a-GCST90001890 | TRUE | T | 204826646 | 1.22E-08 | reported | 2920 | 0.05398 | rs62177481 |  |  |  |  |  |  |  |  |  |  |  |  |  |  |  |
| -0.3018 | 2 | igd | 0.0709 | C | \|\| id:ebi-a-GCST90001893 | ebi-a-GCST90001893 | TRUE | T | 204826646 | 1.95E-08 | reported | 2920 | 0.05358 | rs62177481 |  |  |  |  |  |  |  |  |  |  |  |  |  |  |  |
| -0.3193 | 2 | igd | 0.0709 | C | \|\| id:ebi-a-GCST90001894 | ebi-a-GCST90001894 | TRUE | T | 204826646 | 2.35E-09 | reported | 2920 | 0.0533 | rs62177481 |  |  |  |  |  |  |  |  |  |  |  |  |  |  |  |
| 0.2737 | 10 | igd | 0.0708 | T | \|\| id:ebi-a-GCST90001491 | ebi-a-GCST90001491 | TRUE | C | 102230537 | 3.06E-08 | reported | 3437 | 0.04932 | rs140766344 |  |  |  |  |  |  |  |  |  |  |  |  |  |  |  |
| 0.2895 | 10 | igd | 0.0707 | T | \|\| id:ebi-a-GCST90001505 | ebi-a-GCST90001505 | TRUE | C | 6054765 | 6.16E-09 | reported | 3437 | 0.04968 | rs12722600 |  |  |  |  |  |  |  |  |  |  |  |  |  |  |  |
| 0.2774 | 20 | igd | 0.0707 | A | \|\| id:ebi-a-GCST90001804 | ebi-a-GCST90001804 | TRUE | T | 45162484 | 1.08E-08 | reported | 3657 | 0.0484 | rs73305523 |  |  |  |  |  |  |  |  |  |  |  |  |  |  |  |
| -0.4395 | 19 | igd | 0.0707 | G | \|\| id:ebi-a-GCST90001947 | ebi-a-GCST90001947 | TRUE | T | 52897142 | 1.16E-09 | reported | 1577 | 0.07179 | rs11882720 |  |  |  |  |  |  |  |  |  |  |  |  |  |  |  |
| 0.319 | 6 | igd | 0.0707 | G | \|\| id:ebi-a-GCST90002105 | ebi-a-GCST90002105 | TRUE | A | 27505721 | 7.23E-09 | reported | 2872 | 0.05498 | rs62401375 |  |  |  |  |  |  |  |  |  |  |  |  |  |  |  |
| -0.3088 | 10 | igd | 0.0706 | C | \|\| id:ebi-a-GCST90001489 | ebi-a-GCST90001489 | TRUE | G | 99532911 | 6.76E-10 | reported | 3405 | 0.0499 | rs142762435 |  |  |  |  |  |  |  |  |  |  |  |  |  |  |  |
| -0.2851 | 10 | igd | 0.0706 | C | \|\| id:ebi-a-GCST90001495 | ebi-a-GCST90001495 | TRUE | G | 99532911 | 1.59E-08 | reported | 3405 | 0.05032 | rs142762435 |  |  |  |  |  |  |  |  |  |  |  |  |  |  |  |
| -0.2948 | 10 | igd | 0.0706 | C | \|\| id:ebi-a-GCST90001660 | ebi-a-GCST90001660 | TRUE | G | 99532911 | 3.25E-09 | reported | 3408 | 0.04969 | rs142762435 |  |  |  |  |  |  |  |  |  |  |  |  |  |  |  |
| -0.2788 | 10 | igd | 0.0706 | C | \|\| id:ebi-a-GCST90001672 | ebi-a-GCST90001672 | TRUE | G | 99532911 | 3.04E-08 | reported | 3408 | 0.05021 | rs142762435 |  |  |  |  |  |  |  |  |  |  |  |  |  |  |  |
| -0.4413 | 19 | igd | 0.0706 | G | \|\| id:ebi-a-GCST90001956 | ebi-a-GCST90001956 | TRUE | T | 52897142 | 9.25E-10 | reported | 1579 | 0.07165 | rs11882720 |  |  |  |  |  |  |  |  |  |  |  |  |  |  |  |
| 0.289 | 20 | igd | 0.0705 | T | \|\| id:ebi-a-GCST90001798 | ebi-a-GCST90001798 | TRUE | A | 45168093 | 3.27E-09 | reported | 3657 | 0.04871 | rs6066014 |  |  |  |  |  |  |  |  |  |  |  |  |  |  |  |
| 0.2938 | 20 | igd | 0.0705 | T | \|\| id:ebi-a-GCST90001808 | ebi-a-GCST90001808 | TRUE | A | 45168093 | 2.37E-09 | reported | 3657 | 0.0491 | rs6066014 |  |  |  |  |  |  |  |  |  |  |  |  |  |  |  |
| 0.3212 | 10 | igd | 0.0705 | T | \|\| id:ebi-a-GCST90001937 | ebi-a-GCST90001937 | TRUE | C | 6054765 | 1.09E-10 | reported | 3434 | 0.04962 | rs12722600 |  |  |  |  |  |  |  |  |  |  |  |  |  |  |  |
| -0.2711 | 10 | igd | 0.0704 | C | \|\| id:ebi-a-GCST90001484 | ebi-a-GCST90001484 | TRUE | G | 99532911 | 2.10E-08 | reported | 3437 | 0.04826 | rs142762435 |  |  |  |  |  |  |  |  |  |  |  |  |  |  |  |
| -0.2673 | 10 | igd | 0.0704 | C | \|\| id:ebi-a-GCST90001485 | ebi-a-GCST90001485 | TRUE | G | 99532911 | 3.13E-08 | reported | 3437 | 0.0482 | rs142762435 |  |  |  |  |  |  |  |  |  |  |  |  |  |  |  |
| -0.2902 | 10 | igd | 0.0704 | C | \|\| id:ebi-a-GCST90001491 | ebi-a-GCST90001491 | TRUE | G | 99532911 | 3.63E-09 | reported | 3437 | 0.04906 | rs142762435 |  |  |  |  |  |  |  |  |  |  |  |  |  |  |  |
| -0.2748 | 10 | igd | 0.0704 | C | \|\| id:ebi-a-GCST90001496 | ebi-a-GCST90001496 | TRUE | G | 99532911 | 3.59E-08 | reported | 3437 | 0.04975 | rs142762435 |  |  |  |  |  |  |  |  |  |  |  |  |  |  |  |
| 0.4387 | 19 | igd | 0.0704 | T | \|\| id:ebi-a-GCST90001946 | ebi-a-GCST90001946 | TRUE | C | 51868443 | 1.39E-09 | reported | 1634 | 0.07202 | rs58483275 |  |  |  |  |  |  |  |  |  |  |  |  |  |  |  |
| 0.4941 | 19 | igd | 0.0704 | T | \|\| id:ebi-a-GCST90001948 | ebi-a-GCST90001948 | TRUE | C | 51868443 | 6.18E-12 | reported | 1633 | 0.07133 | rs58483275 |  |  |  |  |  |  |  |  |  |  |  |  |  |  |  |
| 0.4452 | 19 | igd | 0.0704 | T | \|\| id:ebi-a-GCST90001949 | ebi-a-GCST90001949 | TRUE | A | 51868925 | 5.87E-10 | reported | 1634 | 0.07145 | rs73560177 |  |  |  |  |  |  |  |  |  |  |  |  |  |  |  |
| 0.346 | 1 | igd | 0.0704 | T | \|\| id:ebi-a-GCST90002035 | ebi-a-GCST90002035 | TRUE | C | 161653554 | 1.92E-10 | reported | 2871 | 0.05414 | rs114384494 |  |  |  |  |  |  |  |  |  |  |  |  |  |  |  |
| -0.2996 | 10 | igd | 0.0703 | C | \|\| id:ebi-a-GCST90001658 | ebi-a-GCST90001658 | TRUE | G | 99532911 | 2.77E-10 | reported | 3440 | 0.04734 | rs142762435 |  |  |  |  |  |  |  |  |  |  |  |  |  |  |  |
| -0.292 | 10 | igd | 0.0703 | C | \|\| id:ebi-a-GCST90001659 | ebi-a-GCST90001659 | TRUE | G | 99532911 | 7.87E-10 | reported | 3440 | 0.04736 | rs142762435 |  |  |  |  |  |  |  |  |  |  |  |  |  |  |  |
| -0.2852 | 10 | igd | 0.0703 | C | \|\| id:ebi-a-GCST90001670 | ebi-a-GCST90001670 | TRUE | G | 99532911 | 8.22E-09 | reported | 3440 | 0.04935 | rs142762435 |  |  |  |  |  |  |  |  |  |  |  |  |  |  |  |
| -0.3084 | 10 | igd | 0.0703 | C | \|\| id:ebi-a-GCST90001671 | ebi-a-GCST90001671 | TRUE | G | 99532911 | 4.61E-10 | reported | 3440 | 0.04935 | rs142762435 |  |  |  |  |  |  |  |  |  |  |  |  |  |  |  |
| -0.4468 | 19 | igd | 0.0703 | G | \|\| id:ebi-a-GCST90001957 | ebi-a-GCST90001957 | TRUE | T | 52897142 | 6.36E-10 | reported | 1578 | 0.07183 | rs11882720 |  |  |  |  |  |  |  |  |  |  |  |  |  |  |  |
| -0.3345 | 1 | igd | 0.0703 | A | \|\| id:ebi-a-GCST90001989 | ebi-a-GCST90001989 | TRUE | G | 161949163 | 2.05E-11 | reported | 3621 | 0.04974 | rs140749884 |  |  |  |  |  |  |  |  |  |  |  |  |  |  |  |
| 0.3652 | 6 | igd | 0.0703 | C | \|\| id:ebi-a-GCST90002105 | ebi-a-GCST90002105 | TRUE | T | 26299874 | 5.13E-10 | reported | 2872 | 0.05856 | rs111628921 |  |  |  |  |  |  |  |  |  |  |  |  |  |  |  |
| 0.31 | 10 | igd | 0.0702 | C | \|\| id:ebi-a-GCST90001961 | ebi-a-GCST90001961 | TRUE | T | 97616428 | 1.28E-08 | reported | 2920 | 0.05434 | rs189455847 |  |  |  |  |  |  |  |  |  |  |  |  |  |  |  |
| -0.6103 | 10 | igd | 0.0702 | C | \|\| id:ebi-a-GCST90002030 | ebi-a-GCST90002030 | TRUE | T | 97616428 | 1.04E-30 | reported | 2920 | 0.05237 | rs189455847 |  |  |  |  |  |  |  |  |  |  |  |  |  |  |  |
| -0.4903 | 10 | igd | 0.0702 | C | \|\| id:ebi-a-GCST90002031 | ebi-a-GCST90002031 | TRUE | T | 97616428 | 3.38E-19 | reported | 2920 | 0.05437 | rs189455847 |  |  |  |  |  |  |  |  |  |  |  |  |  |  |  |
| -0.5308 | 10 | igd | 0.0702 | C | \|\| id:ebi-a-GCST90002032 | ebi-a-GCST90002032 | TRUE | T | 97616428 | 2.48E-22 | reported | 2920 | 0.05417 | rs189455847 |  |  |  |  |  |  |  |  |  |  |  |  |  |  |  |
| 0.443 | 1 | igd | 0.0702 | T | \|\| id:ebi-a-GCST90002014 | ebi-a-GCST90002014 | TRUE | C | 161653554 | 8.37E-16 | reported | 2870 | 0.05473 | rs114384494 |  |  |  |  |  |  |  |  |  |  |  |  |  |  |  |
| -0.2691 | 20 | igd | 0.07 | G | \|\| id:ebi-a-GCST90001806 | ebi-a-GCST90001806 | TRUE | T | 45732275 | 4.95E-08 | reported | 3656 | 0.04925 | rs6066198 |  |  |  |  |  |  |  |  |  |  |  |  |  |  |  |
| 0.3804 | 10 | igd | 0.0699 | C | \|\| id:ebi-a-GCST90001961 | ebi-a-GCST90001961 | TRUE | T | 97070395 | 3.11E-12 | reported | 2920 | 0.05432 | rs117983078 |  |  |  |  |  |  |  |  |  |  |  |  |  |  |  |
| 0.3351 | 6 | igd | 0.0699 | A | \|\| id:ebi-a-GCST90002118 | ebi-a-GCST90002118 | TRUE | C | 29823191 | 7.72E-09 | reported | 2920 | 0.05786 | rs16896139 |  |  |  |  |  |  |  |  |  |  |  |  |  |  |  |
| -0.2766 | 1 | igd | 0.0696 | C | \|\| id:ebi-a-GCST90001474 | ebi-a-GCST90001474 | TRUE | G | 161730198 | 4.44E-08 | reported | 3410 | 0.05043 | rs12756470 |  |  |  |  |  |  |  |  |  |  |  |  |  |  |  |
| -0.3997 | 19 | igd | 0.0695 | G | \|\| id:ebi-a-GCST90001954 | ebi-a-GCST90001954 | TRUE | T | 52897142 | 4.02E-08 | reported | 1555 | 0.07243 | rs11882720 |  |  |  |  |  |  |  |  |  |  |  |  |  |  |  |
| -0.4252 | 19 | igd | 0.0695 | G | \|\| id:ebi-a-GCST90001955 | ebi-a-GCST90001955 | TRUE | T | 52897142 | 5.45E-09 | reported | 1554 | 0.07248 | rs11882720 |  |  |  |  |  |  |  |  |  |  |  |  |  |  |  |
| -0.5396 | 10 | igd | 0.0694 | C | \|\| id:ebi-a-GCST90001660 | ebi-a-GCST90001660 | TRUE | T | 97070395 | 1.62E-26 | reported | 3408 | 0.05021 | rs117983078 |  |  |  |  |  |  |  |  |  |  |  |  |  |  |  |
| -0.4777 | 1 | igd | 0.0694 | T | \|\| id:ebi-a-GCST90001845 | ebi-a-GCST90001845 | TRUE | C | 167405064 | 2.30E-19 | reported | 2911 | 0.05272 | rs115697342 |  |  |  |  |  |  |  |  |  |  |  |  |  |  |  |
| 0.2808 | 10 | igd | 0.0693 | G | \|\| id:ebi-a-GCST90001483 | ebi-a-GCST90001483 | TRUE | T | 97155873 | 2.25E-08 | reported | 3405 | 0.0501 | rs57682907 |  |  |  |  |  |  |  |  |  |  |  |  |  |  |  |
| -0.4784 | 10 | igd | 0.0693 | C | \|\| id:ebi-a-GCST90001489 | ebi-a-GCST90001489 | TRUE | T | 97070395 | 3.94E-21 | reported | 3405 | 0.05037 | rs117983078 |  |  |  |  |  |  |  |  |  |  |  |  |  |  |  |
| -0.4849 | 10 | igd | 0.0693 | C | \|\| id:ebi-a-GCST90001495 | ebi-a-GCST90001495 | TRUE | T | 97070395 | 3.14E-21 | reported | 3405 | 0.05092 | rs117983078 |  |  |  |  |  |  |  |  |  |  |  |  |  |  |  |
| -0.4858 | 10 | igd | 0.0693 | C | \|\| id:ebi-a-GCST90001658 | ebi-a-GCST90001658 | TRUE | T | 97070395 | 1.03E-23 | reported | 3440 | 0.04803 | rs117983078 |  |  |  |  |  |  |  |  |  |  |  |  |  |  |  |
| -0.4857 | 10 | igd | 0.0693 | C | \|\| id:ebi-a-GCST90001659 | ebi-a-GCST90001659 | TRUE | T | 97070395 | 1.07E-23 | reported | 3440 | 0.04804 | rs117983078 |  |  |  |  |  |  |  |  |  |  |  |  |  |  |  |
| 0.2732 | 10 | igd | 0.0693 | T | \|\| id:ebi-a-GCST90001659 | ebi-a-GCST90001659 | TRUE | G | 102192591 | 1.56E-08 | reported | 3440 | 0.04821 | rs117628976 |  |  |  |  |  |  |  |  |  |  |  |  |  |  |  |
| -0.6197 | 22 | igd | 0.0693 | A | \|\| id:ebi-a-GCST90001702 | ebi-a-GCST90001702 | TRUE | G | 43132180 | 1.07E-34 | reported | 3657 | 0.04992 | rs117321107 |  |  |  |  |  |  |  |  |  |  |  |  |  |  |  |
| -0.6033 | 22 | igd | 0.0693 | A | \|\| id:ebi-a-GCST90001703 | ebi-a-GCST90001703 | TRUE | G | 43132180 | 5.81E-33 | reported | 3657 | 0.04995 | rs117321107 |  |  |  |  |  |  |  |  |  |  |  |  |  |  |  |
| -0.605 | 22 | igd | 0.0693 | A | \|\| id:ebi-a-GCST90001711 | ebi-a-GCST90001711 | TRUE | G | 43132180 | 1.78E-33 | reported | 3657 | 0.04967 | rs117321107 |  |  |  |  |  |  |  |  |  |  |  |  |  |  |  |
| -0.3761 | 10 | igd | 0.0693 | C | \|\| id:ebi-a-GCST90002030 | ebi-a-GCST90002030 | TRUE | G | 99532911 | 1.18E-12 | reported | 2920 | 0.05268 | rs142762435 |  |  |  |  |  |  |  |  |  |  |  |  |  |  |  |
| -0.3492 | 10 | igd | 0.0693 | C | \|\| id:ebi-a-GCST90002031 | ebi-a-GCST90002031 | TRUE | G | 99532911 | 1.27E-10 | reported | 2920 | 0.05412 | rs142762435 |  |  |  |  |  |  |  |  |  |  |  |  |  |  |  |
| -0.2986 | 10 | igd | 0.0693 | C | \|\| id:ebi-a-GCST90002032 | ebi-a-GCST90002032 | TRUE | G | 99532911 | 3.87E-08 | reported | 2920 | 0.05418 | rs142762435 |  |  |  |  |  |  |  |  |  |  |  |  |  |  |  |
| 0.7176 | 6 | igd | 0.0693 | A | \|\| id:ebi-a-GCST90002105 | ebi-a-GCST90002105 | TRUE | T | 31207991 | 1.38E-30 | reported | 2872 | 0.0617 | rs144184221 |  |  |  |  |  |  |  |  |  |  |  |  |  |  |  |
| 0.3251 | 10 | igd | 0.069 | G | \|\| id:ebi-a-GCST90001484 | ebi-a-GCST90001484 | TRUE | T | 97155873 | 6.80E-11 | reported | 3437 | 0.04966 | rs57682907 |  |  |  |  |  |  |  |  |  |  |  |  |  |  |  |
| 0.2746 | 10 | igd | 0.069 | G | \|\| id:ebi-a-GCST90001485 | ebi-a-GCST90001485 | TRUE | T | 97155873 | 3.50E-08 | reported | 3437 | 0.04968 | rs57682907 |  |  |  |  |  |  |  |  |  |  |  |  |  |  |  |
| -0.5932 | 1 | igd | 0.069 | T | \|\| id:ebi-a-GCST90001854 | ebi-a-GCST90001854 | TRUE | C | 167405064 | 1.80E-29 | reported | 2919 | 0.05205 | rs115697342 |  |  |  |  |  |  |  |  |  |  |  |  |  |  |  |
| -0.518 | 1 | igd | 0.069 | T | \|\| id:ebi-a-GCST90001856 | ebi-a-GCST90001856 | TRUE | C | 167405064 | 2.39E-22 | reported | 2919 | 0.05284 | rs115697342 |  |  |  |  |  |  |  |  |  |  |  |  |  |  |  |
| -0.555 | 1 | igd | 0.069 | T | \|\| id:ebi-a-GCST90001858 | ebi-a-GCST90001858 | TRUE | C | 167405064 | 1.10E-25 | reported | 2919 | 0.05247 | rs115697342 |  |  |  |  |  |  |  |  |  |  |  |  |  |  |  |
| -0.6263 | 1 | igd | 0.069 | T | \|\| id:ebi-a-GCST90001860 | ebi-a-GCST90001860 | TRUE | C | 167405064 | 5.01E-32 | reported | 2919 | 0.05254 | rs115697342 |  |  |  |  |  |  |  |  |  |  |  |  |  |  |  |
| -0.4776 | 1 | igd | 0.069 | T | \|\| id:ebi-a-GCST90001861 | ebi-a-GCST90001861 | TRUE | C | 167405064 | 1.05E-19 | reported | 2919 | 0.0522 | rs115697342 |  |  |  |  |  |  |  |  |  |  |  |  |  |  |  |
| -0.4215 | 1 | igd | 0.069 | T | \|\| id:ebi-a-GCST90001863 | ebi-a-GCST90001863 | TRUE | C | 167405064 | 8.81E-16 | reported | 2919 | 0.05212 | rs115697342 |  |  |  |  |  |  |  |  |  |  |  |  |  |  |  |
| -0.525 | 1 | igd | 0.069 | T | \|\| id:ebi-a-GCST90001864 | ebi-a-GCST90001864 | TRUE | C | 167405064 | 4.25E-23 | reported | 2919 | 0.05259 | rs115697342 |  |  |  |  |  |  |  |  |  |  |  |  |  |  |  |
| -0.3721 | 1 | igd | 0.069 | T | \|\| id:ebi-a-GCST90001865 | ebi-a-GCST90001865 | TRUE | C | 167405064 | 4.44E-12 | reported | 2919 | 0.05353 | rs115697342 |  |  |  |  |  |  |  |  |  |  |  |  |  |  |  |
| -0.3974 | 10 | igd | 0.0689 | T | \|\| id:ebi-a-GCST90001489 | ebi-a-GCST90001489 | TRUE | C | 97516612 | 4.27E-15 | reported | 3405 | 0.05041 | rs74671500 |  |  |  |  |  |  |  |  |  |  |  |  |  |  |  |
| 0.3184 | 2 | igd | 0.0689 | T | \|\| id:ebi-a-GCST90002098 | ebi-a-GCST90002098 | TRUE | C | 41757305 | 1.59E-09 | reported | 2910 | 0.0526 | rs79747818 |  |  |  |  |  |  |  |  |  |  |  |  |  |  |  |
| -0.4459 | 10 | igd | 0.0688 | T | \|\| id:ebi-a-GCST90001672 | ebi-a-GCST90001672 | TRUE | C | 97516612 | 2.11E-18 | reported | 3408 | 0.05066 | rs74671500 |  |  |  |  |  |  |  |  |  |  |  |  |  |  |  |
| -0.3894 | 1 | igd | 0.0688 | T | \|\| id:ebi-a-GCST90001866 | ebi-a-GCST90001866 | TRUE | C | 167405064 | 1.71E-13 | reported | 2914 | 0.05258 | rs115697342 |  |  |  |  |  |  |  |  |  |  |  |  |  |  |  |
| 0.5136 | 10 | igd | 0.0688 | C | \|\| id:ebi-a-GCST90002030 | ebi-a-GCST90002030 | TRUE | G | 98140314 | 1.82E-21 | reported | 2920 | 0.05355 | rs188163491 |  |  |  |  |  |  |  |  |  |  |  |  |  |  |  |
| 0.4729 | 10 | igd | 0.0688 | C | \|\| id:ebi-a-GCST90002031 | ebi-a-GCST90002031 | TRUE | G | 98140314 | 1.10E-17 | reported | 2920 | 0.05487 | rs188163491 |  |  |  |  |  |  |  |  |  |  |  |  |  |  |  |
| 0.5063 | 10 | igd | 0.0688 | C | \|\| id:ebi-a-GCST90002032 | ebi-a-GCST90002032 | TRUE | G | 98140314 | 4.32E-20 | reported | 2920 | 0.05475 | rs188163491 |  |  |  |  |  |  |  |  |  |  |  |  |  |  |  |
| 0.727 | 6 | igd | 0.0688 | C | \|\| id:ebi-a-GCST90002105 | ebi-a-GCST90002105 | TRUE | T | 31339393 | 6.08E-31 | reported | 2872 | 0.06212 | rs16899340 |  |  |  |  |  |  |  |  |  |  |  |  |  |  |  |
| 0.7328 | 6 | igd | 0.0688 | C | \|\| id:ebi-a-GCST90002105 | ebi-a-GCST90002105 | TRUE | T | 31463832 | 1.82E-32 | reported | 2872 | 0.061 | rs73390402 |  |  |  |  |  |  |  |  |  |  |  |  |  |  |  |
| 0.4578 | 6 | igd | 0.0688 | C | \|\| id:ebi-a-GCST90002109 | ebi-a-GCST90002109 | TRUE | T | 31463832 | 3.36E-09 | reported | 1578 | 0.07698 | rs73390402 |  |  |  |  |  |  |  |  |  |  |  |  |  |  |  |
| 0.657 | 6 | igd | 0.0687 | C | \|\| id:ebi-a-GCST90002108 | ebi-a-GCST90002108 | TRUE | T | 31463832 | 2.98E-17 | reported | 1579 | 0.07689 | rs73390402 |  |  |  |  |  |  |  |  |  |  |  |  |  |  |  |
| 0.6059 | 6 | igd | 0.0687 | A | \|\| id:ebi-a-GCST90002108 | ebi-a-GCST90002108 | TRUE | T | 31207991 | 1.45E-14 | reported | 1579 | 0.07803 | rs144184221 |  |  |  |  |  |  |  |  |  |  |  |  |  |  |  |
| -0.4383 | 10 | igd | 0.0686 | T | \|\| id:ebi-a-GCST90001670 | ebi-a-GCST90001670 | TRUE | C | 97516612 | 2.08E-18 | reported | 3440 | 0.04979 | rs74671500 |  |  |  |  |  |  |  |  |  |  |  |  |  |  |  |
| 0.751 | 6 | igd | 0.0686 | G | \|\| id:ebi-a-GCST90002105 | ebi-a-GCST90002105 | TRUE | A | 31569745 | 7.61E-34 | reported | 2872 | 0.06113 | rs116007826 |  |  |  |  |  |  |  |  |  |  |  |  |  |  |  |
| -0.3163 | 2 | igd | 0.0685 | G | \|\| id:ebi-a-GCST90001890 | ebi-a-GCST90001890 | TRUE | C | 205015903 | 1.09E-08 | reported | 2920 | 0.05519 | rs147990121 |  |  |  |  |  |  |  |  |  |  |  |  |  |  |  |
| -0.3318 | 2 | igd | 0.0685 | G | \|\| id:ebi-a-GCST90001891 | ebi-a-GCST90001891 | TRUE | C | 205015903 | 1.23E-09 | reported | 2920 | 0.05442 | rs147990121 |  |  |  |  |  |  |  |  |  |  |  |  |  |  |  |
| 0.2951 | 10 | igd | 0.0685 | A | \|\| id:ebi-a-GCST90001892 | ebi-a-GCST90001892 | TRUE | G | 95281076 | 4.70E-08 | reported | 2920 | 0.05388 | rs112358299 |  |  |  |  |  |  |  |  |  |  |  |  |  |  |  |
| -0.3026 | 2 | igd | 0.0685 | G | \|\| id:ebi-a-GCST90001894 | ebi-a-GCST90001894 | TRUE | C | 205015903 | 3.20E-08 | reported | 2920 | 0.05457 | rs147990121 |  |  |  |  |  |  |  |  |  |  |  |  |  |  |  |
| -0.3359 | 2 | igd | 0.0685 | G | \|\| id:ebi-a-GCST90001900 | ebi-a-GCST90001900 | TRUE | C | 205015903 | 8.02E-10 | reported | 2919 | 0.05449 | rs147990121 |  |  |  |  |  |  |  |  |  |  |  |  |  |  |  |
| -0.4093 | 10 | igd | 0.0685 | A | \|\| id:ebi-a-GCST90001961 | ebi-a-GCST90001961 | TRUE | G | 95281076 | 6.15E-14 | reported | 2920 | 0.05427 | rs112358299 |  |  |  |  |  |  |  |  |  |  |  |  |  |  |  |
| 0.3101 | 10 | igd | 0.0685 | A | \|\| id:ebi-a-GCST90002030 | ebi-a-GCST90002030 | TRUE | G | 95281076 | 1.24E-08 | reported | 2920 | 0.05431 | rs112358299 |  |  |  |  |  |  |  |  |  |  |  |  |  |  |  |
| 0.3095 | 10 | igd | 0.0685 | A | \|\| id:ebi-a-GCST90002031 | ebi-a-GCST90002031 | TRUE | G | 95281076 | 2.33E-08 | reported | 2920 | 0.05525 | rs112358299 |  |  |  |  |  |  |  |  |  |  |  |  |  |  |  |
| -0.3822 | 2 | igd | 0.0685 | A | \|\| id:ebi-a-GCST90002119 | ebi-a-GCST90002119 | TRUE | G | 86174229 | 6.01E-13 | reported | 2920 | 0.05284 | rs79409842 |  |  |  |  |  |  |  |  |  |  |  |  |  |  |  |
| 0.3134 | 10 | igd | 0.0683 | C | \|\| id:ebi-a-GCST90001483 | ebi-a-GCST90001483 | TRUE | G | 98140314 | 4.76E-10 | reported | 3405 | 0.05019 | rs188163491 |  |  |  |  |  |  |  |  |  |  |  |  |  |  |  |
| 0.4142 | 10 | igd | 0.0683 | C | \|\| id:ebi-a-GCST90001489 | ebi-a-GCST90001489 | TRUE | G | 98140314 | 1.01E-15 | reported | 3405 | 0.05136 | rs188163491 |  |  |  |  |  |  |  |  |  |  |  |  |  |  |  |
| 0.4462 | 10 | igd | 0.0683 | C | \|\| id:ebi-a-GCST90001495 | ebi-a-GCST90001495 | TRUE | G | 98140314 | 1.01E-17 | reported | 3405 | 0.05177 | rs188163491 |  |  |  |  |  |  |  |  |  |  |  |  |  |  |  |
| -0.313 | 17 | igd | 0.0683 | C | \|\| id:ebi-a-GCST90001926 | ebi-a-GCST90001926 | TRUE | T | 56491186 | 1.88E-08 | reported | 2920 | 0.05552 | rs117507353 |  |  |  |  |  |  |  |  |  |  |  |  |  |  |  |
| 0.309 | 10 | igd | 0.0683 | T | \|\| id:ebi-a-GCST90002061 | ebi-a-GCST90002061 | TRUE | G | 107778954 | 2.07E-08 | reported | 2920 | 0.05496 | rs140895566 |  |  |  |  |  |  |  |  |  |  |  |  |  |  |  |
| 0.3982 | 10 | igd | 0.0682 | C | \|\| id:ebi-a-GCST90001660 | ebi-a-GCST90001660 | TRUE | G | 98140314 | 1.06E-14 | reported | 3408 | 0.05127 | rs188163491 |  |  |  |  |  |  |  |  |  |  |  |  |  |  |  |
| 0.312 | 10 | igd | 0.0682 | C | \|\| id:ebi-a-GCST90001672 | ebi-a-GCST90001672 | TRUE | G | 98140314 | 2.10E-09 | reported | 3408 | 0.05196 | rs188163491 |  |  |  |  |  |  |  |  |  |  |  |  |  |  |  |
| 0.2693 | 11 | igd | 0.0682 | A | \|\| id:ebi-a-GCST90001752 | ebi-a-GCST90001752 | TRUE | G | 60126844 | 4.23E-08 | reported | 3657 | 0.04903 | rs117762171 |  |  |  |  |  |  |  |  |  |  |  |  |  |  |  |
| 0.3161 | 6 | igd | 0.0682 | A | \|\| id:ebi-a-GCST90002105 | ebi-a-GCST90002105 | TRUE | G | 33300969 | 9.39E-09 | reported | 2872 | 0.05489 | rs115138315 |  |  |  |  |  |  |  |  |  |  |  |  |  |  |  |
| -0.4983 | 6 | igd | 0.0682 | G | \|\| id:ebi-a-GCST90002110 | ebi-a-GCST90002110 | TRUE | A | 32606912 | 5.97E-10 | reported | 1634 | 0.07999 | rs28383364 |  |  |  |  |  |  |  |  |  |  |  |  |  |  |  |
| -0.4434 | 6 | igd | 0.0682 | G | \|\| id:ebi-a-GCST90002111 | ebi-a-GCST90002111 | TRUE | A | 32606912 | 2.42E-08 | reported | 1635 | 0.07909 | rs28383364 |  |  |  |  |  |  |  |  |  |  |  |  |  |  |  |
| -0.4796 | 6 | igd | 0.0682 | T | \|\| id:ebi-a-GCST90002112 | ebi-a-GCST90002112 | TRUE | C | 32592796 | 7.95E-11 | reported | 1635 | 0.07328 | rs28383322 |  |  |  |  |  |  |  |  |  |  |  |  |  |  |  |
| 0.3246 | 10 | igd | 0.0681 | C | \|\| id:ebi-a-GCST90001484 | ebi-a-GCST90001484 | TRUE | G | 98140314 | 7.88E-11 | reported | 3437 | 0.04977 | rs188163491 |  |  |  |  |  |  |  |  |  |  |  |  |  |  |  |
| 0.3168 | 10 | igd | 0.0681 | C | \|\| id:ebi-a-GCST90001485 | ebi-a-GCST90001485 | TRUE | G | 98140314 | 2.10E-10 | reported | 3437 | 0.04971 | rs188163491 |  |  |  |  |  |  |  |  |  |  |  |  |  |  |  |
| 0.3986 | 10 | igd | 0.0681 | C | \|\| id:ebi-a-GCST90001491 | ebi-a-GCST90001491 | TRUE | G | 98140314 | 4.49E-15 | reported | 3437 | 0.05061 | rs188163491 |  |  |  |  |  |  |  |  |  |  |  |  |  |  |  |
| 0.3241 | 10 | igd | 0.0681 | T | \|\| id:ebi-a-GCST90001495 | ebi-a-GCST90001495 | TRUE | C | 98548954 | 3.21E-10 | reported | 3405 | 0.05139 | rs111852518 |  |  |  |  |  |  |  |  |  |  |  |  |  |  |  |
| 0.4016 | 10 | igd | 0.0681 | C | \|\| id:ebi-a-GCST90001496 | ebi-a-GCST90001496 | TRUE | G | 98140314 | 6.47E-15 | reported | 3437 | 0.0513 | rs188163491 |  |  |  |  |  |  |  |  |  |  |  |  |  |  |  |
| 0.2905 | 10 | igd | 0.0681 | T | \|\| id:ebi-a-GCST90001496 | ebi-a-GCST90001496 | TRUE | C | 98548954 | 1.30E-08 | reported | 3437 | 0.05097 | rs111852518 |  |  |  |  |  |  |  |  |  |  |  |  |  |  |  |
| 0.2893 | 10 | igd | 0.0681 | T | \|\| id:ebi-a-GCST90001497 | ebi-a-GCST90001497 | TRUE | C | 98548954 | 9.81E-09 | reported | 3437 | 0.05032 | rs111852518 |  |  |  |  |  |  |  |  |  |  |  |  |  |  |  |
| 0.4156 | 10 | igd | 0.0681 | C | \|\| id:ebi-a-GCST90001497 | ebi-a-GCST90001497 | TRUE | G | 98140314 | 3.02E-16 | reported | 3437 | 0.05061 | rs188163491 |  |  |  |  |  |  |  |  |  |  |  |  |  |  |  |
| 0.3945 | 10 | igd | 0.0681 | T | \|\| id:ebi-a-GCST90001660 | ebi-a-GCST90001660 | TRUE | C | 98548954 | 8.48E-15 | reported | 3408 | 0.05061 | rs111852518 |  |  |  |  |  |  |  |  |  |  |  |  |  |  |  |
| 0.3558 | 10 | igd | 0.0681 | T | \|\| id:ebi-a-GCST90001672 | ebi-a-GCST90001672 | TRUE | C | 98548954 | 4.42E-12 | reported | 3408 | 0.0512 | rs111852518 |  |  |  |  |  |  |  |  |  |  |  |  |  |  |  |
| 0.6643 | 6 | igd | 0.0681 | G | \|\| id:ebi-a-GCST90002108 | ebi-a-GCST90002108 | TRUE | A | 31569745 | 1.75E-17 | reported | 1579 | 0.07716 | rs116007826 |  |  |  |  |  |  |  |  |  |  |  |  |  |  |  |
| 0.4645 | 6 | igd | 0.0681 | G | \|\| id:ebi-a-GCST90002109 | ebi-a-GCST90002109 | TRUE | A | 31569745 | 2.25E-09 | reported | 1578 | 0.07723 | rs116007826 |  |  |  |  |  |  |  |  |  |  |  |  |  |  |  |
| 0.3747 | 10 | igd | 0.068 | C | \|\| id:ebi-a-GCST90001658 | ebi-a-GCST90001658 | TRUE | G | 98140314 | 2.45E-14 | reported | 3440 | 0.04893 | rs188163491 |  |  |  |  |  |  |  |  |  |  |  |  |  |  |  |
| 0.3796 | 10 | igd | 0.068 | C | \|\| id:ebi-a-GCST90001659 | ebi-a-GCST90001659 | TRUE | G | 98140314 | 1.13E-14 | reported | 3440 | 0.04893 | rs188163491 |  |  |  |  |  |  |  |  |  |  |  |  |  |  |  |
| 0.3526 | 10 | igd | 0.068 | T | \|\| id:ebi-a-GCST90001659 | ebi-a-GCST90001659 | TRUE | C | 98548954 | 4.01E-13 | reported | 3440 | 0.04841 | rs111852518 |  |  |  |  |  |  |  |  |  |  |  |  |  |  |  |
| 0.3117 | 10 | igd | 0.068 | T | \|\| id:ebi-a-GCST90001670 | ebi-a-GCST90001670 | TRUE | C | 98548954 | 7.53E-10 | reported | 3440 | 0.05051 | rs111852518 |  |  |  |  |  |  |  |  |  |  |  |  |  |  |  |
| 0.3019 | 10 | igd | 0.068 | C | \|\| id:ebi-a-GCST90001670 | ebi-a-GCST90001670 | TRUE | G | 98140314 | 3.81E-09 | reported | 3440 | 0.0511 | rs188163491 |  |  |  |  |  |  |  |  |  |  |  |  |  |  |  |
| 0.3159 | 10 | igd | 0.068 | C | \|\| id:ebi-a-GCST90001671 | ebi-a-GCST90001671 | TRUE | G | 98140314 | 7.34E-10 | reported | 3440 | 0.05115 | rs188163491 |  |  |  |  |  |  |  |  |  |  |  |  |  |  |  |
| 0.3675 | 10 | igd | 0.068 | T | \|\| id:ebi-a-GCST90001671 | ebi-a-GCST90001671 | TRUE | C | 98548954 | 3.96E-13 | reported | 3440 | 0.05045 | rs111852518 |  |  |  |  |  |  |  |  |  |  |  |  |  |  |  |
| 0.2851 | 22 | igd | 0.068 | T | \|\| id:ebi-a-GCST90001711 | ebi-a-GCST90001711 | TRUE | C | 43363377 | 1.50E-08 | reported | 3657 | 0.05024 | rs148598719 |  |  |  |  |  |  |  |  |  |  |  |  |  |  |  |
| -0.3034 | 1 | igd | 0.068 | A | \|\| id:ebi-a-GCST90001970 | ebi-a-GCST90001970 | TRUE | G | 161659801 | 3.43E-08 | reported | 2971 | 0.05484 | rs114931935 |  |  |  |  |  |  |  |  |  |  |  |  |  |  |  |
| -0.3163 | 1 | igd | 0.068 | A | \|\| id:ebi-a-GCST90002077 | ebi-a-GCST90002077 | TRUE | G | 161659801 | 6.84E-09 | reported | 2971 | 0.05442 | rs114931935 |  |  |  |  |  |  |  |  |  |  |  |  |  |  |  |
| 0.3446 | 6 | igd | 0.068 | A | \|\| id:ebi-a-GCST90002118 | ebi-a-GCST90002118 | TRUE | G | 30031456 | 4.67E-09 | reported | 2920 | 0.05864 | rs56270681 |  |  |  |  |  |  |  |  |  |  |  |  |  |  |  |
| -0.3214 | 2 | igd | 0.068 | T | \|\| id:ebi-a-GCST90002119 | ebi-a-GCST90002119 | TRUE | C | 87276659 | 2.91E-09 | reported | 2920 | 0.05396 | rs186660701 |  |  |  |  |  |  |  |  |  |  |  |  |  |  |  |
| 0.5738 | 6 | igd | 0.0679 | T | \|\| id:ebi-a-GCST90002105 | ebi-a-GCST90002105 | TRUE | C | 32948426 | 7.77E-26 | reported | 2872 | 0.05406 | rs2071876 |  |  |  |  |  |  |  |  |  |  |  |  |  |  |  |
| 0.5819 | 6 | igd | 0.0679 | C | \|\| id:ebi-a-GCST90002112 | ebi-a-GCST90002112 | TRUE | T | 31463832 | 7.51E-14 | reported | 1635 | 0.07713 | rs73390402 |  |  |  |  |  |  |  |  |  |  |  |  |  |  |  |
| 0.5549 | 6 | igd | 0.0679 | A | \|\| id:ebi-a-GCST90002112 | ebi-a-GCST90002112 | TRUE | T | 31207991 | 1.78E-12 | reported | 1635 | 0.07808 | rs144184221 |  |  |  |  |  |  |  |  |  |  |  |  |  |  |  |
| -0.439 | 3 | igd | 0.0679 | A | \|\| id:ebi-a-GCST90002017 | ebi-a-GCST90002017 | TRUE | G | 46292339 | 3.51E-14 | reported | 2850 | 0.05763 | rs34272024 |  |  |  |  |  |  |  |  |  |  |  |  |  |  |  |
| 0.6234 | 6 | igd | 0.0678 | C | \|\| id:ebi-a-GCST90002108 | ebi-a-GCST90002108 | TRUE | T | 31339393 | 4.39E-15 | reported | 1579 | 0.0787 | rs16899340 |  |  |  |  |  |  |  |  |  |  |  |  |  |  |  |
| 0.3048 | 20 | igd | 0.0677 | G | \|\| id:ebi-a-GCST90001989 | ebi-a-GCST90001989 | TRUE | A | 44863608 | 1.53E-09 | reported | 3621 | 0.05032 | rs78199910 |  |  |  |  |  |  |  |  |  |  |  |  |  |  |  |
| 0.5939 | 6 | igd | 0.0676 | G | \|\| id:ebi-a-GCST90002112 | ebi-a-GCST90002112 | TRUE | A | 31569745 | 2.66E-14 | reported | 1635 | 0.0773 | rs116007826 |  |  |  |  |  |  |  |  |  |  |  |  |  |  |  |
| 0.3373 | 10 | igd | 0.0675 | T | \|\| id:ebi-a-GCST90001489 | ebi-a-GCST90001489 | TRUE | C | 97294154 | 4.74E-11 | reported | 3405 | 0.0511 | rs72809762 |  |  |  |  |  |  |  |  |  |  |  |  |  |  |  |
| 0.4322 | 10 | igd | 0.0675 | T | \|\| id:ebi-a-GCST90001495 | ebi-a-GCST90001495 | TRUE | C | 97294154 | 4.98E-17 | reported | 3405 | 0.05126 | rs72809762 |  |  |  |  |  |  |  |  |  |  |  |  |  |  |  |
| 0.4486 | 10 | igd | 0.0675 | T | \|\| id:ebi-a-GCST90001660 | ebi-a-GCST90001660 | TRUE | C | 97294154 | 1.12E-18 | reported | 3408 | 0.05056 | rs72809762 |  |  |  |  |  |  |  |  |  |  |  |  |  |  |  |
| 0.3581 | 6 | igd | 0.0675 | T | \|\| id:ebi-a-GCST90002105 | ebi-a-GCST90002105 | TRUE | A | 32803999 | 4.53E-10 | reported | 2872 | 0.05723 | rs9378275 |  |  |  |  |  |  |  |  |  |  |  |  |  |  |  |
| -0.3552 | 10 | igd | 0.0674 | C | \|\| id:ebi-a-GCST90001483 | ebi-a-GCST90001483 | TRUE | T | 97616428 | 1.84E-12 | reported | 3405 | 0.05023 | rs189455847 |  |  |  |  |  |  |  |  |  |  |  |  |  |  |  |
| -0.4454 | 10 | igd | 0.0674 | C | \|\| id:ebi-a-GCST90001495 | ebi-a-GCST90001495 | TRUE | T | 97616428 | 1.13E-17 | reported | 3405 | 0.05175 | rs189455847 |  |  |  |  |  |  |  |  |  |  |  |  |  |  |  |
| -0.5108 | 19 | igd | 0.0673 | T | \|\| id:ebi-a-GCST90001531 | ebi-a-GCST90001531 | TRUE | C | 33754548 | 5.44E-14 | reported | 1856 | 0.06739 | rs78744187 |  |  |  |  |  |  |  |  |  |  |  |  |  |  |  |
| 0.277 | 10 | igd | 0.0673 | A | \|\| id:ebi-a-GCST90001659 | ebi-a-GCST90001659 | TRUE | G | 95281076 | 1.02E-08 | reported | 3440 | 0.04824 | rs112358299 |  |  |  |  |  |  |  |  |  |  |  |  |  |  |  |
| -0.4332 | 10 | igd | 0.0673 | C | \|\| id:ebi-a-GCST90001660 | ebi-a-GCST90001660 | TRUE | T | 97616428 | 3.41E-17 | reported | 3408 | 0.05111 | rs189455847 |  |  |  |  |  |  |  |  |  |  |  |  |  |  |  |
| 0.2818 | 10 | igd | 0.0673 | A | \|\| id:ebi-a-GCST90001660 | ebi-a-GCST90001660 | TRUE | G | 95281076 | 2.70E-08 | reported | 3408 | 0.05056 | rs112358299 |  |  |  |  |  |  |  |  |  |  |  |  |  |  |  |
| 0.3442 | 10 | igd | 0.0673 | A | \|\| id:ebi-a-GCST90001670 | ebi-a-GCST90001670 | TRUE | G | 95281076 | 7.56E-12 | reported | 3440 | 0.0501 | rs112358299 |  |  |  |  |  |  |  |  |  |  |  |  |  |  |  |
| 0.3041 | 10 | igd | 0.0673 | A | \|\| id:ebi-a-GCST90001671 | ebi-a-GCST90001671 | TRUE | G | 95281076 | 1.58E-09 | reported | 3440 | 0.05025 | rs112358299 |  |  |  |  |  |  |  |  |  |  |  |  |  |  |  |
| -0.3834 | 10 | igd | 0.0673 | C | \|\| id:ebi-a-GCST90001672 | ebi-a-GCST90001672 | TRUE | T | 97616428 | 1.58E-13 | reported | 3408 | 0.05173 | rs189455847 |  |  |  |  |  |  |  |  |  |  |  |  |  |  |  |
| 0.3523 | 10 | igd | 0.0673 | A | \|\| id:ebi-a-GCST90001672 | ebi-a-GCST90001672 | TRUE | G | 95281076 | 5.51E-12 | reported | 3408 | 0.05093 | rs112358299 |  |  |  |  |  |  |  |  |  |  |  |  |  |  |  |
| -0.4178 | 10 | igd | 0.0672 | C | \|\| id:ebi-a-GCST90001484 | ebi-a-GCST90001484 | TRUE | T | 97616428 | 7.83E-17 | reported | 3437 | 0.04987 | rs189455847 |  |  |  |  |  |  |  |  |  |  |  |  |  |  |  |
| -0.3516 | 10 | igd | 0.0672 | C | \|\| id:ebi-a-GCST90001485 | ebi-a-GCST90001485 | TRUE | T | 97616428 | 2.33E-12 | reported | 3437 | 0.04995 | rs189455847 |  |  |  |  |  |  |  |  |  |  |  |  |  |  |  |
| -0.4176 | 10 | igd | 0.0672 | C | \|\| id:ebi-a-GCST90001491 | ebi-a-GCST90001491 | TRUE | T | 97616428 | 2.36E-16 | reported | 3437 | 0.05066 | rs189455847 |  |  |  |  |  |  |  |  |  |  |  |  |  |  |  |
| -0.4626 | 10 | igd | 0.0672 | C | \|\| id:ebi-a-GCST90001496 | ebi-a-GCST90001496 | TRUE | T | 97616428 | 2.88E-19 | reported | 3437 | 0.05124 | rs189455847 |  |  |  |  |  |  |  |  |  |  |  |  |  |  |  |
| -0.485 | 10 | igd | 0.0672 | C | \|\| id:ebi-a-GCST90001497 | ebi-a-GCST90001497 | TRUE | T | 97616428 | 1.49E-21 | reported | 3437 | 0.05052 | rs189455847 |  |  |  |  |  |  |  |  |  |  |  |  |  |  |  |
| -0.4536 | 10 | igd | 0.0672 | C | \|\| id:ebi-a-GCST90001658 | ebi-a-GCST90001658 | TRUE | T | 97616428 | 2.36E-20 | reported | 3440 | 0.04876 | rs189455847 |  |  |  |  |  |  |  |  |  |  |  |  |  |  |  |
| -0.4693 | 10 | igd | 0.0672 | C | \|\| id:ebi-a-GCST90001659 | ebi-a-GCST90001659 | TRUE | T | 97616428 | 1.09E-21 | reported | 3440 | 0.04872 | rs189455847 |  |  |  |  |  |  |  |  |  |  |  |  |  |  |  |
| -0.3862 | 10 | igd | 0.0672 | C | \|\| id:ebi-a-GCST90001670 | ebi-a-GCST90001670 | TRUE | T | 97616428 | 4.77E-14 | reported | 3440 | 0.05101 | rs189455847 |  |  |  |  |  |  |  |  |  |  |  |  |  |  |  |
| -0.403 | 10 | igd | 0.0672 | C | \|\| id:ebi-a-GCST90001671 | ebi-a-GCST90001671 | TRUE | T | 97616428 | 3.65E-15 | reported | 3440 | 0.05099 | rs189455847 |  |  |  |  |  |  |  |  |  |  |  |  |  |  |  |
| -0.3094 | 10 | igd | 0.0671 | T | \|\| id:ebi-a-GCST90001491 | ebi-a-GCST90001491 | TRUE | C | 92106512 | 1.53E-09 | reported | 3437 | 0.05107 | rs113837062 |  |  |  |  |  |  |  |  |  |  |  |  |  |  |  |
| 0.3263 | 10 | igd | 0.067 | T | \|\| id:ebi-a-GCST90001483 | ebi-a-GCST90001483 | TRUE | C | 98546713 | 7.47E-11 | reported | 3405 | 0.04996 | rs12573061 |  |  |  |  |  |  |  |  |  |  |  |  |  |  |  |
| 0.6604 | 10 | igd | 0.067 | C | \|\| id:ebi-a-GCST90002030 | ebi-a-GCST90002030 | TRUE | T | 97827487 | 6.16E-32 | reported | 2920 | 0.05548 | rs12098340 |  |  |  |  |  |  |  |  |  |  |  |  |  |  |  |
| 0.5298 | 10 | igd | 0.067 | A | \|\| id:ebi-a-GCST90002031 | ebi-a-GCST90002031 | TRUE | G | 97837887 | 3.16E-20 | reported | 2920 | 0.05708 | rs60218681 |  |  |  |  |  |  |  |  |  |  |  |  |  |  |  |
| 0.429 | 10 | igd | 0.067 | C | \|\| id:ebi-a-GCST90002032 | ebi-a-GCST90002032 | TRUE | T | 97827487 | 9.71E-14 | reported | 2920 | 0.05735 | rs12098340 |  |  |  |  |  |  |  |  |  |  |  |  |  |  |  |
| 0.5619 | 6 | igd | 0.067 | C | \|\| id:ebi-a-GCST90002112 | ebi-a-GCST90002112 | TRUE | T | 31339393 | 1.47E-12 | reported | 1635 | 0.07879 | rs16899340 |  |  |  |  |  |  |  |  |  |  |  |  |  |  |  |
| -0.3323 | 3 | igd | 0.067 | G | \|\| id:ebi-a-GCST90001997 | ebi-a-GCST90001997 | TRUE | A | 39510601 | 7.07E-10 | reported | 3590 | 0.05375 | rs13067113 |  |  |  |  |  |  |  |  |  |  |  |  |  |  |  |
| -0.3138 | 3 | igd | 0.067 | G | \|\| id:ebi-a-GCST90001995 | ebi-a-GCST90001995 | TRUE | A | 39510601 | 4.65E-09 | reported | 3590 | 0.05343 | rs13067113 |  |  |  |  |  |  |  |  |  |  |  |  |  |  |  |
| 0.3242 | 10 | igd | 0.0668 | T | \|\| id:ebi-a-GCST90001484 | ebi-a-GCST90001484 | TRUE | C | 98546713 | 7.94E-11 | reported | 3437 | 0.04971 | rs12573061 |  |  |  |  |  |  |  |  |  |  |  |  |  |  |  |
| 0.3045 | 10 | igd | 0.0668 | T | \|\| id:ebi-a-GCST90001485 | ebi-a-GCST90001485 | TRUE | C | 98546713 | 9.78E-10 | reported | 3437 | 0.04967 | rs12573061 |  |  |  |  |  |  |  |  |  |  |  |  |  |  |  |
| -0.3994 | 19 | igd | 0.0668 | T | \|\| id:ebi-a-GCST90001531 | ebi-a-GCST90001531 | TRUE | G | 51685133 | 3.07E-09 | reported | 1856 | 0.06705 | rs116960573 |  |  |  |  |  |  |  |  |  |  |  |  |  |  |  |
| -0.5319 | 19 | igd | 0.0668 | T | \|\| id:ebi-a-GCST90001533 | ebi-a-GCST90001533 | TRUE | G | 51685133 | 8.52E-16 | reported | 1857 | 0.06552 | rs116960573 |  |  |  |  |  |  |  |  |  |  |  |  |  |  |  |
| 0.6959 | 10 | igd | 0.0668 | C | \|\| id:ebi-a-GCST90001660 | ebi-a-GCST90001660 | TRUE | T | 97881031 | 5.38E-40 | reported | 3408 | 0.0519 | rs17111341 |  |  |  |  |  |  |  |  |  |  |  |  |  |  |  |
| 0.6424 | 10 | igd | 0.0668 | C | \|\| id:ebi-a-GCST90001672 | ebi-a-GCST90001672 | TRUE | T | 97881031 | 1.45E-33 | reported | 3408 | 0.05263 | rs17111341 |  |  |  |  |  |  |  |  |  |  |  |  |  |  |  |
| 0.5388 | 10 | igd | 0.0668 | C | \|\| id:ebi-a-GCST90002031 | ebi-a-GCST90002031 | TRUE | T | 97945485 | 6.95E-21 | reported | 2920 | 0.05704 | rs112945882 |  |  |  |  |  |  |  |  |  |  |  |  |  |  |  |
| 0.5436 | 10 | igd | 0.0667 | C | \|\| id:ebi-a-GCST90001489 | ebi-a-GCST90001489 | TRUE | T | 97881031 | 1.04E-24 | reported | 3405 | 0.05255 | rs17111341 |  |  |  |  |  |  |  |  |  |  |  |  |  |  |  |
| 0.6371 | 10 | igd | 0.0667 | C | \|\| id:ebi-a-GCST90001495 | ebi-a-GCST90001495 | TRUE | T | 97881031 | 6.78E-33 | reported | 3405 | 0.05277 | rs17111341 |  |  |  |  |  |  |  |  |  |  |  |  |  |  |  |
| 0.3523 | 10 | igd | 0.0667 | T | \|\| id:ebi-a-GCST90001658 | ebi-a-GCST90001658 | TRUE | C | 98546713 | 6.09E-13 | reported | 3440 | 0.04875 | rs12573061 |  |  |  |  |  |  |  |  |  |  |  |  |  |  |  |
| 0.6657 | 10 | igd | 0.0667 | C | \|\| id:ebi-a-GCST90001658 | ebi-a-GCST90001658 | TRUE | T | 97881031 | 3.74E-40 | reported | 3440 | 0.04954 | rs17111341 |  |  |  |  |  |  |  |  |  |  |  |  |  |  |  |
| 0.6919 | 10 | igd | 0.0667 | C | \|\| id:ebi-a-GCST90001659 | ebi-a-GCST90001659 | TRUE | T | 97881031 | 2.57E-43 | reported | 3440 | 0.04944 | rs17111341 |  |  |  |  |  |  |  |  |  |  |  |  |  |  |  |
| 0.6237 | 10 | igd | 0.0667 | C | \|\| id:ebi-a-GCST90001670 | ebi-a-GCST90001670 | TRUE | T | 97881031 | 9.80E-33 | reported | 3440 | 0.0518 | rs17111341 |  |  |  |  |  |  |  |  |  |  |  |  |  |  |  |
| 0.6402 | 10 | igd | 0.0667 | C | \|\| id:ebi-a-GCST90001671 | ebi-a-GCST90001671 | TRUE | T | 97881031 | 2.59E-34 | reported | 3440 | 0.05184 | rs17111341 |  |  |  |  |  |  |  |  |  |  |  |  |  |  |  |
| -1.016 | 19 | igd | 0.0667 | T | \|\| id:ebi-a-GCST90001946 | ebi-a-GCST90001946 | TRUE | G | 51685133 | 3.22E-46 | reported | 1634 | 0.06894 | rs116960573 |  |  |  |  |  |  |  |  |  |  |  |  |  |  |  |
| -1.007 | 19 | igd | 0.0667 | T | \|\| id:ebi-a-GCST90001948 | ebi-a-GCST90001948 | TRUE | G | 51685133 | 1.20E-45 | reported | 1633 | 0.06882 | rs116960573 |  |  |  |  |  |  |  |  |  |  |  |  |  |  |  |
| -0.4036 | 19 | igd | 0.0667 | G | \|\| id:ebi-a-GCST90001948 | ebi-a-GCST90001948 | TRUE | A | 52081138 | 1.58E-08 | reported | 1633 | 0.07104 | rs62113338 |  |  |  |  |  |  |  |  |  |  |  |  |  |  |  |
| -0.9928 | 19 | igd | 0.0667 | T | \|\| id:ebi-a-GCST90001949 | ebi-a-GCST90001949 | TRUE | G | 51685133 | 1.35E-44 | reported | 1634 | 0.06873 | rs116960573 |  |  |  |  |  |  |  |  |  |  |  |  |  |  |  |
| -0.3918 | 19 | igd | 0.0667 | G | \|\| id:ebi-a-GCST90001949 | ebi-a-GCST90001949 | TRUE | A | 52081138 | 3.89E-08 | reported | 1634 | 0.07096 | rs62113338 |  |  |  |  |  |  |  |  |  |  |  |  |  |  |  |
| -0.3192 | 3 | igd | 0.0667 | C | \|\| id:ebi-a-GCST90001997 | ebi-a-GCST90001997 | TRUE | T | 38928831 | 1.90E-10 | reported | 3590 | 0.04997 | rs145135505 |  |  |  |  |  |  |  |  |  |  |  |  |  |  |  |
| -0.2745 | 3 | igd | 0.0667 | C | \|\| id:ebi-a-GCST90001995 | ebi-a-GCST90001995 | TRUE | T | 38928831 | 3.56E-08 | reported | 3590 | 0.0497 | rs145135505 |  |  |  |  |  |  |  |  |  |  |  |  |  |  |  |
| 0.574 | 10 | igd | 0.0666 | C | \|\| id:ebi-a-GCST90001491 | ebi-a-GCST90001491 | TRUE | T | 97881031 | 3.68E-28 | reported | 3437 | 0.0517 | rs17111341 |  |  |  |  |  |  |  |  |  |  |  |  |  |  |  |
| 0.6958 | 10 | igd | 0.0666 | C | \|\| id:ebi-a-GCST90001496 | ebi-a-GCST90001496 | TRUE | T | 97881031 | 7.39E-40 | reported | 3437 | 0.05199 | rs17111341 |  |  |  |  |  |  |  |  |  |  |  |  |  |  |  |
| 0.6714 | 10 | igd | 0.0666 | C | \|\| id:ebi-a-GCST90001497 | ebi-a-GCST90001497 | TRUE | T | 97881031 | 4.28E-38 | reported | 3437 | 0.0514 | rs17111341 |  |  |  |  |  |  |  |  |  |  |  |  |  |  |  |
| -0.4201 | 10 | igd | 0.0666 | C | \|\| id:ebi-a-GCST90001961 | ebi-a-GCST90001961 | TRUE | T | 97881031 | 1.78E-13 | reported | 2920 | 0.05677 | rs17111341 |  |  |  |  |  |  |  |  |  |  |  |  |  |  |  |
| 0.3742 | 22 | igd | 0.0664 | A | \|\| id:ebi-a-GCST90001702 | ebi-a-GCST90001702 | TRUE | G | 42480421 | 7.45E-13 | reported | 3657 | 0.052 | rs139043947 |  |  |  |  |  |  |  |  |  |  |  |  |  |  |  |
| 0.3806 | 22 | igd | 0.0664 | A | \|\| id:ebi-a-GCST90001703 | ebi-a-GCST90001703 | TRUE | G | 42480421 | 2.95E-13 | reported | 3657 | 0.05197 | rs139043947 |  |  |  |  |  |  |  |  |  |  |  |  |  |  |  |
| 0.3538 | 22 | igd | 0.0664 | A | \|\| id:ebi-a-GCST90001704 | ebi-a-GCST90001704 | TRUE | G | 42480421 | 1.13E-11 | reported | 3657 | 0.05194 | rs139043947 |  |  |  |  |  |  |  |  |  |  |  |  |  |  |  |
| 0.3445 | 22 | igd | 0.0664 | A | \|\| id:ebi-a-GCST90001710 | ebi-a-GCST90001710 | TRUE | G | 42480421 | 3.74E-11 | reported | 3657 | 0.05193 | rs139043947 |  |  |  |  |  |  |  |  |  |  |  |  |  |  |  |
| 0.6752 | 10 | igd | 0.0663 | C | \|\| id:ebi-a-GCST90002030 | ebi-a-GCST90002030 | TRUE | T | 97930955 | 8.63E-33 | reported | 2920 | 0.05592 | rs2125059 |  |  |  |  |  |  |  |  |  |  |  |  |  |  |  |
| 0.4431 | 10 | igd | 0.0663 | C | \|\| id:ebi-a-GCST90002032 | ebi-a-GCST90002032 | TRUE | T | 97930955 | 2.40E-14 | reported | 2920 | 0.0578 | rs2125059 |  |  |  |  |  |  |  |  |  |  |  |  |  |  |  |
| -0.3748 | 1 | igd | 0.066 | G | \|\| id:ebi-a-GCST90001989 | ebi-a-GCST90001989 | TRUE | T | 162125242 | 1.49E-13 | reported | 3621 | 0.05053 | rs368510411 |  |  |  |  |  |  |  |  |  |  |  |  |  |  |  |
| 0.3166 | 20 | igd | 0.0659 | A | \|\| id:ebi-a-GCST90001989 | ebi-a-GCST90001989 | TRUE | G | 42214463 | 1.08E-09 | reported | 3621 | 0.05179 | rs149811612 |  |  |  |  |  |  |  |  |  |  |  |  |  |  |  |
| -0.5299 | 6 | igd | 0.0659 | A | \|\| id:ebi-a-GCST90002109 | ebi-a-GCST90002109 | TRUE | G | 32625051 | 1.96E-08 | reported | 1578 | 0.09388 | rs28479136 |  |  |  |  |  |  |  |  |  |  |  |  |  |  |  |
| -0.28 | 1 | igd | 0.0658 | G | \|\| id:ebi-a-GCST90001981 | ebi-a-GCST90001981 | TRUE | T | 162125242 | 2.92E-08 | reported | 3618 | 0.05038 | rs368510411 |  |  |  |  |  |  |  |  |  |  |  |  |  |  |  |
| 0.6774 | 10 | igd | 0.0658 | G | \|\| id:ebi-a-GCST90002030 | ebi-a-GCST90002030 | TRUE | T | 97824758 | 2.22E-32 | reported | 2920 | 0.05648 | rs74151252 |  |  |  |  |  |  |  |  |  |  |  |  |  |  |  |
| 0.5363 | 10 | igd | 0.0658 | G | \|\| id:ebi-a-GCST90002031 | ebi-a-GCST90002031 | TRUE | T | 97824758 | 4.78E-20 | reported | 2920 | 0.05806 | rs74151252 |  |  |  |  |  |  |  |  |  |  |  |  |  |  |  |
| 0.3895 | 10 | igd | 0.0658 | G | \|\| id:ebi-a-GCST90002032 | ebi-a-GCST90002032 | TRUE | T | 97824758 | 3.24E-11 | reported | 2920 | 0.05847 | rs74151252 |  |  |  |  |  |  |  |  |  |  |  |  |  |  |  |
| 0.4834 | 6 | igd | 0.0657 | T | \|\| id:ebi-a-GCST90002112 | ebi-a-GCST90002112 | TRUE | C | 32948426 | 2.81E-11 | reported | 1635 | 0.07212 | rs2071876 |  |  |  |  |  |  |  |  |  |  |  |  |  |  |  |
| -0.634 | 19 | igd | 0.06568 | A | \|\| id:prot-a-439 | prot-a-439 | TRUE | C | 51815081 | 6.46E-31 | reported | 3301 | 0.0548 | rs564957276 |  |  |  |  |  |  |  |  |  |  |  |  |  |  |  |
| 0.7301 | 10 | igd | 0.0656 | G | \|\| id:ebi-a-GCST90001658 | ebi-a-GCST90001658 | TRUE | T | 97824758 | 2.23E-46 | reported | 3440 | 0.0503 | rs74151252 |  |  |  |  |  |  |  |  |  |  |  |  |  |  |  |
| 0.7638 | 10 | igd | 0.0656 | G | \|\| id:ebi-a-GCST90001659 | ebi-a-GCST90001659 | TRUE | T | 97824758 | 1.00E-50 | reported | 3440 | 0.05016 | rs74151252 |  |  |  |  |  |  |  |  |  |  |  |  |  |  |  |
| 0.6787 | 10 | igd | 0.0656 | G | \|\| id:ebi-a-GCST90001670 | ebi-a-GCST90001670 | TRUE | T | 97824758 | 3.53E-37 | reported | 3440 | 0.05264 | rs74151252 |  |  |  |  |  |  |  |  |  |  |  |  |  |  |  |
| 0.6817 | 10 | igd | 0.0656 | G | \|\| id:ebi-a-GCST90001671 | ebi-a-GCST90001671 | TRUE | T | 97824758 | 2.40E-37 | reported | 3440 | 0.05274 | rs74151252 |  |  |  |  |  |  |  |  |  |  |  |  |  |  |  |
| 0.6461 | 10 | igd | 0.0655 | G | \|\| id:ebi-a-GCST90001491 | ebi-a-GCST90001491 | TRUE | T | 97824758 | 4.20E-34 | reported | 3437 | 0.05249 | rs74151252 |  |  |  |  |  |  |  |  |  |  |  |  |  |  |  |
| 0.7499 | 10 | igd | 0.0655 | G | \|\| id:ebi-a-GCST90001496 | ebi-a-GCST90001496 | TRUE | T | 97824758 | 1.73E-44 | reported | 3437 | 0.05283 | rs74151252 |  |  |  |  |  |  |  |  |  |  |  |  |  |  |  |
| 0.7204 | 10 | igd | 0.0655 | G | \|\| id:ebi-a-GCST90001497 | ebi-a-GCST90001497 | TRUE | T | 97824758 | 3.91E-42 | reported | 3437 | 0.05225 | rs74151252 |  |  |  |  |  |  |  |  |  |  |  |  |  |  |  |
| -0.4239 | 22 | igd | 0.0655 | C | \|\| id:ebi-a-GCST90001702 | ebi-a-GCST90001702 | TRUE | G | 39375623 | 5.74E-16 | reported | 3657 | 0.05212 | rs9611061 |  |  |  |  |  |  |  |  |  |  |  |  |  |  |  |
| -0.4325 | 22 | igd | 0.0655 | C | \|\| id:ebi-a-GCST90001703 | ebi-a-GCST90001703 | TRUE | G | 39375623 | 1.41E-16 | reported | 3657 | 0.05209 | rs9611061 |  |  |  |  |  |  |  |  |  |  |  |  |  |  |  |
| -0.431 | 22 | igd | 0.0655 | C | \|\| id:ebi-a-GCST90001704 | ebi-a-GCST90001704 | TRUE | G | 39375623 | 1.63E-16 | reported | 3657 | 0.05202 | rs9611061 |  |  |  |  |  |  |  |  |  |  |  |  |  |  |  |
| -0.3848 | 22 | igd | 0.0655 | C | \|\| id:ebi-a-GCST90001710 | ebi-a-GCST90001710 | TRUE | G | 39375623 | 1.81E-13 | reported | 3657 | 0.05207 | rs9611061 |  |  |  |  |  |  |  |  |  |  |  |  |  |  |  |
| -0.4005 | 22 | igd | 0.0655 | C | \|\| id:ebi-a-GCST90001711 | ebi-a-GCST90001711 | TRUE | G | 39375623 | 1.51E-14 | reported | 3657 | 0.05189 | rs9611061 |  |  |  |  |  |  |  |  |  |  |  |  |  |  |  |
| -0.3527 | 1 | igd | 0.0655 | A | \|\| id:ebi-a-GCST90001989 | ebi-a-GCST90001989 | TRUE | G | 161925353 | 6.56E-12 | reported | 3621 | 0.05119 | rs138966305 |  |  |  |  |  |  |  |  |  |  |  |  |  |  |  |
| 0.3814 | 6 | igd | 0.0655 | C | \|\| id:ebi-a-GCST90002105 | ebi-a-GCST90002105 | TRUE | G | 28264928 | 4.75E-10 | reported | 2872 | 0.06103 | rs3800327 |  |  |  |  |  |  |  |  |  |  |  |  |  |  |  |
| 0.7801 | 10 | igd | 0.0654 | G | \|\| id:ebi-a-GCST90001660 | ebi-a-GCST90001660 | TRUE | T | 97824758 | 3.72E-48 | reported | 3408 | 0.05266 | rs74151252 |  |  |  |  |  |  |  |  |  |  |  |  |  |  |  |
| 0.7096 | 10 | igd | 0.0654 | G | \|\| id:ebi-a-GCST90001672 | ebi-a-GCST90001672 | TRUE | T | 97824758 | 3.54E-39 | reported | 3408 | 0.05351 | rs74151252 |  |  |  |  |  |  |  |  |  |  |  |  |  |  |  |
| 0.3441 | 10 | igd | 0.0654 | T | \|\| id:ebi-a-GCST90002030 | ebi-a-GCST90002030 | TRUE | C | 93109231 | 1.17E-09 | reported | 2920 | 0.05638 | rs142692221 |  |  |  |  |  |  |  |  |  |  |  |  |  |  |  |
| 0.342 | 10 | igd | 0.0654 | T | \|\| id:ebi-a-GCST90002031 | ebi-a-GCST90002031 | TRUE | C | 93109231 | 2.33E-09 | reported | 2920 | 0.05708 | rs142692221 |  |  |  |  |  |  |  |  |  |  |  |  |  |  |  |
| 0.3416 | 10 | igd | 0.0654 | T | \|\| id:ebi-a-GCST90002032 | ebi-a-GCST90002032 | TRUE | C | 93109231 | 2.50E-09 | reported | 2920 | 0.05713 | rs142692221 |  |  |  |  |  |  |  |  |  |  |  |  |  |  |  |
| 0.2846 | 10 | igd | 0.0653 | A | \|\| id:ebi-a-GCST90001484 | ebi-a-GCST90001484 | TRUE | C | 95256200 | 2.14E-08 | reported | 3437 | 0.0507 | rs3740373 |  |  |  |  |  |  |  |  |  |  |  |  |  |  |  |
| 0.6245 | 10 | igd | 0.0653 | G | \|\| id:ebi-a-GCST90001489 | ebi-a-GCST90001489 | TRUE | T | 97824758 | 4.58E-31 | reported | 3405 | 0.05335 | rs74151252 |  |  |  |  |  |  |  |  |  |  |  |  |  |  |  |
| 0.6959 | 10 | igd | 0.0653 | G | \|\| id:ebi-a-GCST90001495 | ebi-a-GCST90001495 | TRUE | T | 97824758 | 1.42E-37 | reported | 3405 | 0.05366 | rs74151252 |  |  |  |  |  |  |  |  |  |  |  |  |  |  |  |
| 0.2924 | 10 | igd | 0.0653 | A | \|\| id:ebi-a-GCST90001659 | ebi-a-GCST90001659 | TRUE | C | 95256200 | 5.31E-09 | reported | 3440 | 0.04996 | rs3740373 |  |  |  |  |  |  |  |  |  |  |  |  |  |  |  |
| -0.8033 | 19 | igd | 0.0652 | T | \|\| id:ebi-a-GCST90001951 | ebi-a-GCST90001951 | TRUE | G | 51685133 | 1.42E-25 | reported | 1464 | 0.07541 | rs116960573 |  |  |  |  |  |  |  |  |  |  |  |  |  |  |  |
| -0.432 | 6 | igd | 0.0651 | G | \|\| id:ebi-a-GCST90001766 | ebi-a-GCST90001766 | TRUE | A | 107164076 | 2.66E-17 | reported | 3638 | 0.05081 | rs9400058 |  |  |  |  |  |  |  |  |  |  |  |  |  |  |  |
| -0.5307 | 6 | igd | 0.0651 | G | \|\| id:ebi-a-GCST90001769 | ebi-a-GCST90001769 | TRUE | A | 107164076 | 2.21E-25 | reported | 3648 | 0.05059 | rs9400058 |  |  |  |  |  |  |  |  |  |  |  |  |  |  |  |
| -0.7377 | 6 | igd | 0.065 | G | \|\| id:ebi-a-GCST90001765 | ebi-a-GCST90001765 | TRUE | A | 107164076 | 1.16E-49 | reported | 3655 | 0.04904 | rs9400058 |  |  |  |  |  |  |  |  |  |  |  |  |  |  |  |
| -0.6739 | 6 | igd | 0.065 | G | \|\| id:ebi-a-GCST90001772 | ebi-a-GCST90001772 | TRUE | A | 107164076 | 3.93E-41 | reported | 3655 | 0.04955 | rs9400058 |  |  |  |  |  |  |  |  |  |  |  |  |  |  |  |
| -0.3311 | 22 | igd | 0.0648 | T | \|\| id:ebi-a-GCST90001702 | ebi-a-GCST90001702 | TRUE | C | 37748690 | 8.65E-11 | reported | 3657 | 0.05088 | rs12170003 |  |  |  |  |  |  |  |  |  |  |  |  |  |  |  |
| -0.322 | 22 | igd | 0.0648 | T | \|\| id:ebi-a-GCST90001703 | ebi-a-GCST90001703 | TRUE | C | 37748690 | 2.80E-10 | reported | 3657 | 0.05089 | rs12170003 |  |  |  |  |  |  |  |  |  |  |  |  |  |  |  |
| -0.3208 | 22 | igd | 0.0648 | T | \|\| id:ebi-a-GCST90001704 | ebi-a-GCST90001704 | TRUE | C | 37748690 | 3.08E-10 | reported | 3657 | 0.05083 | rs12170003 |  |  |  |  |  |  |  |  |  |  |  |  |  |  |  |
| -0.322 | 22 | igd | 0.0648 | T | \|\| id:ebi-a-GCST90001710 | ebi-a-GCST90001710 | TRUE | C | 37748690 | 2.49E-10 | reported | 3657 | 0.05075 | rs12170003 |  |  |  |  |  |  |  |  |  |  |  |  |  |  |  |
| -0.3114 | 22 | igd | 0.0648 | T | \|\| id:ebi-a-GCST90001711 | ebi-a-GCST90001711 | TRUE | C | 37748690 | 8.72E-10 | reported | 3657 | 0.05065 | rs12170003 |  |  |  |  |  |  |  |  |  |  |  |  |  |  |  |
| 0.3559 | 10 | igd | 0.0647 | A | \|\| id:ebi-a-GCST90001961 | ebi-a-GCST90001961 | TRUE | G | 97265004 | 3.13E-10 | reported | 2920 | 0.05637 | rs75632508 |  |  |  |  |  |  |  |  |  |  |  |  |  |  |  |
| -0.5935 | 10 | igd | 0.0647 | A | \|\| id:ebi-a-GCST90002030 | ebi-a-GCST90002030 | TRUE | G | 97265004 | 1.32E-26 | reported | 2920 | 0.05504 | rs75632508 |  |  |  |  |  |  |  |  |  |  |  |  |  |  |  |
| -0.5029 | 10 | igd | 0.0647 | A | \|\| id:ebi-a-GCST90002031 | ebi-a-GCST90002031 | TRUE | G | 97265004 | 1.16E-18 | reported | 2920 | 0.05664 | rs75632508 |  |  |  |  |  |  |  |  |  |  |  |  |  |  |  |
| -0.4932 | 10 | igd | 0.0647 | A | \|\| id:ebi-a-GCST90002032 | ebi-a-GCST90002032 | TRUE | G | 97265004 | 5.16E-18 | reported | 2920 | 0.05664 | rs75632508 |  |  |  |  |  |  |  |  |  |  |  |  |  |  |  |
| -0.3994 | 22 | igd | 0.0643 | T | \|\| id:ebi-a-GCST90001702 | ebi-a-GCST90001702 | TRUE | C | 41892836 | 3.90E-14 | reported | 3657 | 0.0526 | rs73178613 |  |  |  |  |  |  |  |  |  |  |  |  |  |  |  |
| -0.3792 | 22 | igd | 0.0643 | T | \|\| id:ebi-a-GCST90001703 | ebi-a-GCST90001703 | TRUE | C | 41892836 | 6.91E-13 | reported | 3657 | 0.05261 | rs73178613 |  |  |  |  |  |  |  |  |  |  |  |  |  |  |  |
| -0.2956 | 22 | igd | 0.0643 | T | \|\| id:ebi-a-GCST90001704 | ebi-a-GCST90001704 | TRUE | C | 41892836 | 2.15E-08 | reported | 3657 | 0.05267 | rs73178613 |  |  |  |  |  |  |  |  |  |  |  |  |  |  |  |
| -0.3761 | 22 | igd | 0.0643 | T | \|\| id:ebi-a-GCST90001710 | ebi-a-GCST90001710 | TRUE | C | 41892836 | 9.71E-13 | reported | 3657 | 0.05253 | rs73178613 |  |  |  |  |  |  |  |  |  |  |  |  |  |  |  |
| -0.3734 | 22 | igd | 0.0643 | T | \|\| id:ebi-a-GCST90001711 | ebi-a-GCST90001711 | TRUE | C | 41892836 | 1.18E-12 | reported | 3657 | 0.05234 | rs73178613 |  |  |  |  |  |  |  |  |  |  |  |  |  |  |  |
| -0.3245 | 2 | igd | 0.0643 | G | \|\| id:ebi-a-GCST90002098 | ebi-a-GCST90002098 | TRUE | T | 38698112 | 1.19E-09 | reported | 2910 | 0.05318 | rs7605193 |  |  |  |  |  |  |  |  |  |  |  |  |  |  |  |
| -0.3104 | 6 | igd | 0.0642 | T | \|\| id:ebi-a-GCST90001896 | ebi-a-GCST90001896 | TRUE | C | 91015039 | 3.68E-08 | reported | 2920 | 0.05622 | rs12529935 |  |  |  |  |  |  |  |  |  |  |  |  |  |  |  |
| 0.3794 | 22 | igd | 0.0641 | A | \|\| id:ebi-a-GCST90001711 | ebi-a-GCST90001711 | TRUE | G | 42392091 | 4.84E-13 | reported | 3657 | 0.05229 | rs55758879 |  |  |  |  |  |  |  |  |  |  |  |  |  |  |  |
| 0.3301 | 20 | igd | 0.0641 | C | \|\| id:ebi-a-GCST90001989 | ebi-a-GCST90001989 | TRUE | G | 43972682 | 1.02E-10 | reported | 3621 | 0.05091 | rs2251358 |  |  |  |  |  |  |  |  |  |  |  |  |  |  |  |
| -0.317 | 10 | igd | 0.064 | C | \|\| id:ebi-a-GCST90001660 | ebi-a-GCST90001660 | TRUE | A | 99452494 | 2.99E-09 | reported | 3408 | 0.0533 | rs146426185 |  |  |  |  |  |  |  |  |  |  |  |  |  |  |  |
| 0.4813 | 3 | igd | 0.064 | A | \|\| id:ebi-a-GCST90001801 | ebi-a-GCST90001801 | TRUE | T | 33772819 | 1.25E-11 | reported | 1836 | 0.0706 | rs78328252 |  |  |  |  |  |  |  |  |  |  |  |  |  |  |  |
| -0.3264 | 10 | igd | 0.0639 | C | \|\| id:ebi-a-GCST90001489 | ebi-a-GCST90001489 | TRUE | A | 99452494 | 1.19E-09 | reported | 3405 | 0.05353 | rs146426185 |  |  |  |  |  |  |  |  |  |  |  |  |  |  |  |
| -0.3052 | 10 | igd | 0.0639 | C | \|\| id:ebi-a-GCST90001495 | ebi-a-GCST90001495 | TRUE | A | 99452494 | 1.75E-08 | reported | 3405 | 0.05404 | rs146426185 |  |  |  |  |  |  |  |  |  |  |  |  |  |  |  |
| 0.3777 | 22 | igd | 0.0639 | A | \|\| id:ebi-a-GCST90001711 | ebi-a-GCST90001711 | TRUE | G | 42659964 | 7.77E-13 | reported | 3657 | 0.05252 | rs4822099 |  |  |  |  |  |  |  |  |  |  |  |  |  |  |  |
| 0.3298 | 1 | igd | 0.0639 | A | \|\| id:ebi-a-GCST90001854 | ebi-a-GCST90001854 | TRUE | G | 167499290 | 2.78E-09 | reported | 2919 | 0.05531 | rs10918708 |  |  |  |  |  |  |  |  |  |  |  |  |  |  |  |
| 0.307 | 1 | igd | 0.0639 | A | \|\| id:ebi-a-GCST90001856 | ebi-a-GCST90001856 | TRUE | G | 167499290 | 4.39E-08 | reported | 2919 | 0.05592 | rs10918708 |  |  |  |  |  |  |  |  |  |  |  |  |  |  |  |
| 0.2986 | 20 | igd | 0.0639 | C | \|\| id:ebi-a-GCST90001985 | ebi-a-GCST90001985 | TRUE | G | 43972682 | 4.87E-09 | reported | 3629 | 0.0509 | rs2251358 |  |  |  |  |  |  |  |  |  |  |  |  |  |  |  |
| 0.4755 | 10 | igd | 0.0639 | T | \|\| id:ebi-a-GCST90002030 | ebi-a-GCST90002030 | TRUE | C | 97989507 | 1.50E-17 | reported | 2920 | 0.05541 | rs72811025 |  |  |  |  |  |  |  |  |  |  |  |  |  |  |  |
| 0.4357 | 10 | igd | 0.0639 | T | \|\| id:ebi-a-GCST90002031 | ebi-a-GCST90002031 | TRUE | C | 97989507 | 2.43E-14 | reported | 2920 | 0.05684 | rs72811025 |  |  |  |  |  |  |  |  |  |  |  |  |  |  |  |
| 0.4845 | 10 | igd | 0.0639 | T | \|\| id:ebi-a-GCST90002032 | ebi-a-GCST90002032 | TRUE | C | 97989507 | 1.99E-17 | reported | 2920 | 0.05668 | rs72811025 |  |  |  |  |  |  |  |  |  |  |  |  |  |  |  |
| -0.4707 | 10 | igd | 0.0637 | A | \|\| id:ebi-a-GCST90001672 | ebi-a-GCST90001672 | TRUE | G | 97265004 | 1.59E-18 | reported | 3408 | 0.05328 | rs75632508 |  |  |  |  |  |  |  |  |  |  |  |  |  |  |  |
| -0.3344 | 10 | igd | 0.0636 | A | \|\| id:ebi-a-GCST90001483 | ebi-a-GCST90001483 | TRUE | G | 97265004 | 1.19E-10 | reported | 3405 | 0.05175 | rs75632508 |  |  |  |  |  |  |  |  |  |  |  |  |  |  |  |
| 0.315 | 22 | igd | 0.0636 | A | \|\| id:ebi-a-GCST90001702 | ebi-a-GCST90001702 | TRUE | G | 42247984 | 6.91E-10 | reported | 3657 | 0.05093 | rs117181659 |  |  |  |  |  |  |  |  |  |  |  |  |  |  |  |
| 0.3155 | 22 | igd | 0.0636 | A | \|\| id:ebi-a-GCST90001703 | ebi-a-GCST90001703 | TRUE | G | 42247984 | 6.45E-10 | reported | 3657 | 0.05092 | rs117181659 |  |  |  |  |  |  |  |  |  |  |  |  |  |  |  |
| 0.2864 | 22 | igd | 0.0636 | A | \|\| id:ebi-a-GCST90001704 | ebi-a-GCST90001704 | TRUE | G | 42247984 | 1.98E-08 | reported | 3657 | 0.0509 | rs117181659 |  |  |  |  |  |  |  |  |  |  |  |  |  |  |  |
| 0.2799 | 22 | igd | 0.0636 | A | \|\| id:ebi-a-GCST90001710 | ebi-a-GCST90001710 | TRUE | G | 42247984 | 3.93E-08 | reported | 3657 | 0.05084 | rs117181659 |  |  |  |  |  |  |  |  |  |  |  |  |  |  |  |
| 0.2819 | 22 | igd | 0.0636 | A | \|\| id:ebi-a-GCST90001711 | ebi-a-GCST90001711 | TRUE | G | 42247984 | 2.91E-08 | reported | 3657 | 0.05072 | rs117181659 |  |  |  |  |  |  |  |  |  |  |  |  |  |  |  |
| -0.3294 | 10 | igd | 0.0635 | C | \|\| id:ebi-a-GCST90001658 | ebi-a-GCST90001658 | TRUE | A | 99452494 | 1.11E-10 | reported | 3440 | 0.05091 | rs146426185 |  |  |  |  |  |  |  |  |  |  |  |  |  |  |  |
| -0.4787 | 10 | igd | 0.0635 | A | \|\| id:ebi-a-GCST90001658 | ebi-a-GCST90001658 | TRUE | G | 97265004 | 3.11E-21 | reported | 3440 | 0.05028 | rs75632508 |  |  |  |  |  |  |  |  |  |  |  |  |  |  |  |
| -0.317 | 10 | igd | 0.0635 | C | \|\| id:ebi-a-GCST90001659 | ebi-a-GCST90001659 | TRUE | A | 99452494 | 5.48E-10 | reported | 3440 | 0.05094 | rs146426185 |  |  |  |  |  |  |  |  |  |  |  |  |  |  |  |
| -0.5082 | 10 | igd | 0.0635 | A | \|\| id:ebi-a-GCST90001659 | ebi-a-GCST90001659 | TRUE | G | 97265004 | 9.27E-24 | reported | 3440 | 0.0502 | rs75632508 |  |  |  |  |  |  |  |  |  |  |  |  |  |  |  |
| -0.485 | 10 | igd | 0.0635 | A | \|\| id:ebi-a-GCST90001670 | ebi-a-GCST90001670 | TRUE | G | 97265004 | 3.43E-20 | reported | 3440 | 0.05237 | rs75632508 |  |  |  |  |  |  |  |  |  |  |  |  |  |  |  |
| -0.3008 | 10 | igd | 0.0635 | C | \|\| id:ebi-a-GCST90001671 | ebi-a-GCST90001671 | TRUE | A | 99452494 | 1.64E-08 | reported | 3440 | 0.05314 | rs146426185 |  |  |  |  |  |  |  |  |  |  |  |  |  |  |  |
| -0.4703 | 10 | igd | 0.0635 | A | \|\| id:ebi-a-GCST90001671 | ebi-a-GCST90001671 | TRUE | G | 97265004 | 5.13E-19 | reported | 3440 | 0.05248 | rs75632508 |  |  |  |  |  |  |  |  |  |  |  |  |  |  |  |
| 0.3992 | 6 | igd | 0.0635 | A | \|\| id:ebi-a-GCST90002105 | ebi-a-GCST90002105 | TRUE | G | 31013351 | 6.84E-12 | reported | 2872 | 0.05793 | rs17190106 |  |  |  |  |  |  |  |  |  |  |  |  |  |  |  |
| -0.3475 | 10 | igd | 0.0634 | A | \|\| id:ebi-a-GCST90001485 | ebi-a-GCST90001485 | TRUE | G | 97265004 | 1.51E-11 | reported | 3437 | 0.05133 | rs75632508 |  |  |  |  |  |  |  |  |  |  |  |  |  |  |  |
| -0.4461 | 10 | igd | 0.0634 | A | \|\| id:ebi-a-GCST90001491 | ebi-a-GCST90001491 | TRUE | G | 97265004 | 1.92E-17 | reported | 3437 | 0.05222 | rs75632508 |  |  |  |  |  |  |  |  |  |  |  |  |  |  |  |
| -0.3303 | 10 | igd | 0.0634 | C | \|\| id:ebi-a-GCST90001491 | ebi-a-GCST90001491 | TRUE | A | 99452494 | 4.45E-10 | reported | 3437 | 0.0528 | rs146426185 |  |  |  |  |  |  |  |  |  |  |  |  |  |  |  |
| -0.3085 | 10 | igd | 0.0634 | C | \|\| id:ebi-a-GCST90001496 | ebi-a-GCST90001496 | TRUE | A | 99452494 | 9.17E-09 | reported | 3437 | 0.05356 | rs146426185 |  |  |  |  |  |  |  |  |  |  |  |  |  |  |  |
| -0.3048 | 10 | igd | 0.0634 | C | \|\| id:ebi-a-GCST90001497 | ebi-a-GCST90001497 | TRUE | A | 99452494 | 8.96E-09 | reported | 3437 | 0.05289 | rs146426185 |  |  |  |  |  |  |  |  |  |  |  |  |  |  |  |
| 0.3187 | 22 | igd | 0.0634 | C | \|\| id:ebi-a-GCST90001702 | ebi-a-GCST90001702 | TRUE | G | 41993179 | 4.42E-10 | reported | 3657 | 0.05095 | rs73161310 |  |  |  |  |  |  |  |  |  |  |  |  |  |  |  |
| 0.3201 | 22 | igd | 0.0634 | C | \|\| id:ebi-a-GCST90001703 | ebi-a-GCST90001703 | TRUE | G | 41993179 | 3.67E-10 | reported | 3657 | 0.05094 | rs73161310 |  |  |  |  |  |  |  |  |  |  |  |  |  |  |  |
| 0.2908 | 22 | igd | 0.0634 | C | \|\| id:ebi-a-GCST90001704 | ebi-a-GCST90001704 | TRUE | G | 41993179 | 1.21E-08 | reported | 3657 | 0.05092 | rs73161310 |  |  |  |  |  |  |  |  |  |  |  |  |  |  |  |
| 0.2859 | 22 | igd | 0.0634 | C | \|\| id:ebi-a-GCST90001710 | ebi-a-GCST90001710 | TRUE | G | 41993179 | 2.03E-08 | reported | 3657 | 0.05086 | rs73161310 |  |  |  |  |  |  |  |  |  |  |  |  |  |  |  |
| 0.2891 | 22 | igd | 0.0634 | C | \|\| id:ebi-a-GCST90001711 | ebi-a-GCST90001711 | TRUE | G | 41993179 | 1.31E-08 | reported | 3657 | 0.05073 | rs73161310 |  |  |  |  |  |  |  |  |  |  |  |  |  |  |  |
| 0.3225 | 22 | igd | 0.0633 | C | \|\| id:ebi-a-GCST90001702 | ebi-a-GCST90001702 | TRUE | T | 42113165 | 2.95E-10 | reported | 3657 | 0.05103 | rs73161359 |  |  |  |  |  |  |  |  |  |  |  |  |  |  |  |
| 0.3238 | 22 | igd | 0.0633 | C | \|\| id:ebi-a-GCST90001703 | ebi-a-GCST90001703 | TRUE | T | 42113165 | 2.46E-10 | reported | 3657 | 0.05102 | rs73161359 |  |  |  |  |  |  |  |  |  |  |  |  |  |  |  |
| 0.2941 | 22 | igd | 0.0633 | C | \|\| id:ebi-a-GCST90001704 | ebi-a-GCST90001704 | TRUE | T | 42113165 | 8.78E-09 | reported | 3657 | 0.051 | rs73161359 |  |  |  |  |  |  |  |  |  |  |  |  |  |  |  |
| 0.2894 | 22 | igd | 0.0633 | C | \|\| id:ebi-a-GCST90001710 | ebi-a-GCST90001710 | TRUE | T | 42113165 | 1.45E-08 | reported | 3657 | 0.05094 | rs73161359 |  |  |  |  |  |  |  |  |  |  |  |  |  |  |  |
| -0.2976 | 10 | igd | 0.0632 | A | \|\| id:ebi-a-GCST90001660 | ebi-a-GCST90001660 | TRUE | G | 93372632 | 4.12E-08 | reported | 3408 | 0.05412 | rs73320142 |  |  |  |  |  |  |  |  |  |  |  |  |  |  |  |
| -0.304 | 10 | igd | 0.0632 | A | \|\| id:ebi-a-GCST90001672 | ebi-a-GCST90001672 | TRUE | G | 93372632 | 2.88E-08 | reported | 3408 | 0.05465 | rs73320142 |  |  |  |  |  |  |  |  |  |  |  |  |  |  |  |
| 0.2931 | 22 | igd | 0.0632 | C | \|\| id:ebi-a-GCST90001711 | ebi-a-GCST90001711 | TRUE | T | 42133567 | 9.27E-09 | reported | 3657 | 0.05092 | rs73161386 |  |  |  |  |  |  |  |  |  |  |  |  |  |  |  |
| 0.2896 | 20 | igd | 0.0632 | A | \|\| id:ebi-a-GCST90001989 | ebi-a-GCST90001989 | TRUE | G | 43339075 | 2.62E-08 | reported | 3621 | 0.05193 | rs62204573 |  |  |  |  |  |  |  |  |  |  |  |  |  |  |  |
| -0.6727 | 10 | igd | 0.0632 | T | \|\| id:ebi-a-GCST90002030 | ebi-a-GCST90002030 | TRUE | G | 97898846 | 1.68E-33 | reported | 2920 | 0.05507 | rs74453195 |  |  |  |  |  |  |  |  |  |  |  |  |  |  |  |
| -0.5455 | 10 | igd | 0.0632 | T | \|\| id:ebi-a-GCST90002031 | ebi-a-GCST90002031 | TRUE | G | 97898846 | 2.98E-21 | reported | 2920 | 0.05719 | rs74453195 |  |  |  |  |  |  |  |  |  |  |  |  |  |  |  |
| -0.5499 | 10 | igd | 0.0632 | T | \|\| id:ebi-a-GCST90002032 | ebi-a-GCST90002032 | TRUE | G | 97898846 | 1.26E-21 | reported | 2920 | 0.05711 | rs74453195 |  |  |  |  |  |  |  |  |  |  |  |  |  |  |  |
| -0.3371 | 10 | igd | 0.0632 | T | \|\| id:ebi-a-GCST90002061 | ebi-a-GCST90002061 | TRUE | G | 97898846 | 5.76E-09 | reported | 2920 | 0.05772 | rs74453195 |  |  |  |  |  |  |  |  |  |  |  |  |  |  |  |
| -0.4746 | 19 | igd | 0.0631 | C | \|\| id:ebi-a-GCST90001948 | ebi-a-GCST90001948 | TRUE | T | 52130337 | 1.56E-10 | reported | 1633 | 0.07368 | rs8105105 |  |  |  |  |  |  |  |  |  |  |  |  |  |  |  |
| 0.307 | 20 | igd | 0.0631 | A | \|\| id:ebi-a-GCST90001985 | ebi-a-GCST90001985 | TRUE | G | 43339075 | 3.41E-09 | reported | 3629 | 0.05182 | rs62204573 |  |  |  |  |  |  |  |  |  |  |  |  |  |  |  |
| -0.4556 | 19 | igd | 0.063 | C | \|\| id:ebi-a-GCST90001946 | ebi-a-GCST90001946 | TRUE | T | 52130337 | 9.95E-10 | reported | 1634 | 0.07412 | rs8105105 |  |  |  |  |  |  |  |  |  |  |  |  |  |  |  |
| 0.2847 | 20 | igd | 0.063 | A | \|\| id:ebi-a-GCST90001981 | ebi-a-GCST90001981 | TRUE | G | 43339075 | 3.64E-08 | reported | 3618 | 0.05159 | rs62204573 |  |  |  |  |  |  |  |  |  |  |  |  |  |  |  |
| 0.3696 | 3 | igd | 0.0629 | A | \|\| id:ebi-a-GCST90001798 | ebi-a-GCST90001798 | TRUE | T | 33772819 | 2.89E-12 | reported | 3657 | 0.05275 | rs78328252 |  |  |  |  |  |  |  |  |  |  |  |  |  |  |  |
| 0.3414 | 3 | igd | 0.0629 | A | \|\| id:ebi-a-GCST90001802 | ebi-a-GCST90001802 | TRUE | T | 33772819 | 1.71E-10 | reported | 3657 | 0.05331 | rs78328252 |  |  |  |  |  |  |  |  |  |  |  |  |  |  |  |
| 0.3048 | 3 | igd | 0.0629 | A | \|\| id:ebi-a-GCST90001805 | ebi-a-GCST90001805 | TRUE | T | 33772819 | 1.22E-08 | reported | 3657 | 0.05338 | rs78328252 |  |  |  |  |  |  |  |  |  |  |  |  |  |  |  |
| 0.3563 | 3 | igd | 0.0629 | A | \|\| id:ebi-a-GCST90001808 | ebi-a-GCST90001808 | TRUE | T | 33772819 | 2.47E-11 | reported | 3657 | 0.05321 | rs78328252 |  |  |  |  |  |  |  |  |  |  |  |  |  |  |  |
| 0.5003 | 1 | igd | 0.0629 | G | \|\| id:ebi-a-GCST90001858 | ebi-a-GCST90001858 | TRUE | T | 167376713 | 3.64E-19 | reported | 2919 | 0.05553 | rs61806126 |  |  |  |  |  |  |  |  |  |  |  |  |  |  |  |
| 0.4307 | 1 | igd | 0.0629 | G | \|\| id:ebi-a-GCST90001861 | ebi-a-GCST90001861 | TRUE | T | 167376713 | 8.27E-15 | reported | 2919 | 0.05519 | rs61806126 |  |  |  |  |  |  |  |  |  |  |  |  |  |  |  |
| 0.3192 | 1 | igd | 0.0629 | G | \|\| id:ebi-a-GCST90001863 | ebi-a-GCST90001863 | TRUE | T | 167376713 | 8.26E-09 | reported | 2919 | 0.05523 | rs61806126 |  |  |  |  |  |  |  |  |  |  |  |  |  |  |  |
| 0.4257 | 1 | igd | 0.0629 | G | \|\| id:ebi-a-GCST90001864 | ebi-a-GCST90001864 | TRUE | T | 167376713 | 3.08E-14 | reported | 2919 | 0.05577 | rs61806126 |  |  |  |  |  |  |  |  |  |  |  |  |  |  |  |
| 0.336 | 3 | igd | 0.0628 | A | \|\| id:ebi-a-GCST90001800 | ebi-a-GCST90001800 | TRUE | T | 33772819 | 3.98E-10 | reported | 3656 | 0.05358 | rs78328252 |  |  |  |  |  |  |  |  |  |  |  |  |  |  |  |
| 0.3667 | 3 | igd | 0.0628 | A | \|\| id:ebi-a-GCST90001806 | ebi-a-GCST90001806 | TRUE | T | 33772819 | 6.52E-12 | reported | 3656 | 0.05322 | rs78328252 |  |  |  |  |  |  |  |  |  |  |  |  |  |  |  |
| -0.3414 | 3 | igd | 0.0628 | C | \|\| id:ebi-a-GCST90002004 | ebi-a-GCST90002004 | TRUE | A | 46463884 | 2.07E-11 | reported | 3629 | 0.05079 | rs35118578 |  |  |  |  |  |  |  |  |  |  |  |  |  |  |  |
| -0.3408 | 3 | igd | 0.0628 | C | \|\| id:ebi-a-GCST90002008 | ebi-a-GCST90002008 | TRUE | A | 46463884 | 3.45E-11 | reported | 3629 | 0.05127 | rs35118578 |  |  |  |  |  |  |  |  |  |  |  |  |  |  |  |
| -0.3193 | 4 | igd | 0.0627 | C | \|\| id:ebi-a-GCST90001963 | ebi-a-GCST90001963 | TRUE | A | 42664508 | 3.67E-08 | reported | 2872 | 0.05783 | rs28424875 |  |  |  |  |  |  |  |  |  |  |  |  |  |  |  |
| -0.3114 | 2 | igd | 0.0627 | T | \|\| id:ebi-a-GCST90002119 | ebi-a-GCST90002119 | TRUE | C | 86859578 | 2.67E-08 | reported | 2920 | 0.05583 | rs150089781 |  |  |  |  |  |  |  |  |  |  |  |  |  |  |  |
| -0.4558 | 1 | igd | 0.0627 | C | \|\| id:ebi-a-GCST90002013 | ebi-a-GCST90002013 | TRUE | T | 161884403 | 1.61E-14 | reported | 2870 | 0.05905 | rs114676248 |  |  |  |  |  |  |  |  |  |  |  |  |  |  |  |
| -0.304 | 1 | igd | 0.0625 | C | \|\| id:ebi-a-GCST90001467 | ebi-a-GCST90001467 | TRUE | T | 161884403 | 1.92E-08 | reported | 3410 | 0.05398 | rs114676248 |  |  |  |  |  |  |  |  |  |  |  |  |  |  |  |
| -0.4743 | 1 | igd | 0.0625 | C | \|\| id:ebi-a-GCST90001474 | ebi-a-GCST90001474 | TRUE | T | 161884403 | 2.59E-18 | reported | 3410 | 0.05403 | rs114676248 |  |  |  |  |  |  |  |  |  |  |  |  |  |  |  |
| -0.4732 | 3 | igd | 0.0624 | G | \|\| id:ebi-a-GCST90002014 | ebi-a-GCST90002014 | TRUE | A | 46259296 | 1.64E-15 | reported | 2870 | 0.05907 | rs35587265 |  |  |  |  |  |  |  |  |  |  |  |  |  |  |  |
| -0.4223 | 3 | igd | 0.0624 | G | \|\| id:ebi-a-GCST90002013 | ebi-a-GCST90002013 | TRUE | A | 46259296 | 7.75E-13 | reported | 2870 | 0.05867 | rs35587265 |  |  |  |  |  |  |  |  |  |  |  |  |  |  |  |
| 0.4032 | 1 | igd | 0.0623 | G | \|\| id:ebi-a-GCST90001845 | ebi-a-GCST90001845 | TRUE | T | 167376713 | 9.22E-13 | reported | 2911 | 0.0562 | rs61806126 |  |  |  |  |  |  |  |  |  |  |  |  |  |  |  |
| 0.3627 | 6 | igd | 0.0622 | C | \|\| id:ebi-a-GCST90001896 | ebi-a-GCST90001896 | TRUE | T | 90749909 | 1.76E-10 | reported | 2920 | 0.05664 | rs117980071 |  |  |  |  |  |  |  |  |  |  |  |  |  |  |  |
| 0.4316 | 1 | igd | 0.0621 | C | \|\| id:ebi-a-GCST90001994 | ebi-a-GCST90001994 | TRUE | T | 161884403 | 1.23E-15 | reported | 3590 | 0.05369 | rs114676248 |  |  |  |  |  |  |  |  |  |  |  |  |  |  |  |
| 0.9303 | 6 | igd | 0.062 | A | \|\| id:ebi-a-GCST90002105 | ebi-a-GCST90002105 | TRUE | G | 32433384 | 1.06E-49 | reported | 2872 | 0.06157 | rs114433295 |  |  |  |  |  |  |  |  |  |  |  |  |  |  |  |
| -1.059 | 19 | igd | 0.0618 | T | \|\| id:ebi-a-GCST90001957 | ebi-a-GCST90001957 | TRUE | G | 51685133 | 1.80E-44 | reported | 1578 | 0.07333 | rs116960573 |  |  |  |  |  |  |  |  |  |  |  |  |  |  |  |
| -0.8239 | 6 | igd | 0.0617 | G | \|\| id:ebi-a-GCST90001767 | ebi-a-GCST90001767 | TRUE | A | 107164076 | 3.42E-30 | reported | 1832 | 0.07087 | rs9400058 |  |  |  |  |  |  |  |  |  |  |  |  |  |  |  |
| -1.049 | 19 | igd | 0.0617 | T | \|\| id:ebi-a-GCST90001956 | ebi-a-GCST90001956 | TRUE | G | 51685133 | 1.04E-43 | reported | 1579 | 0.07338 | rs116960573 |  |  |  |  |  |  |  |  |  |  |  |  |  |  |  |
| 0.3242 | 10 | igd | 0.0617 | G | \|\| id:ebi-a-GCST90002029 | ebi-a-GCST90002029 | TRUE | A | 97387248 | 1.13E-08 | reported | 2915 | 0.05661 | rs41291566 |  |  |  |  |  |  |  |  |  |  |  |  |  |  |  |
| -0.4146 | 1 | igd | 0.0617 | C | \|\| id:ebi-a-GCST90001999 | ebi-a-GCST90001999 | TRUE | T | 161884403 | 4.43E-14 | reported | 3621 | 0.05471 | rs114676248 |  |  |  |  |  |  |  |  |  |  |  |  |  |  |  |
| -0.3707 | 10 | igd | 0.0616 | G | \|\| id:ebi-a-GCST90001961 | ebi-a-GCST90001961 | TRUE | A | 97387248 | 1.72E-10 | reported | 2920 | 0.05786 | rs41291566 |  |  |  |  |  |  |  |  |  |  |  |  |  |  |  |
| 0.7032 | 10 | igd | 0.0616 | G | \|\| id:ebi-a-GCST90002030 | ebi-a-GCST90002030 | TRUE | A | 97387248 | 3.96E-35 | reported | 2920 | 0.05611 | rs41291566 |  |  |  |  |  |  |  |  |  |  |  |  |  |  |  |
| 0.6057 | 10 | igd | 0.0616 | G | \|\| id:ebi-a-GCST90002031 | ebi-a-GCST90002031 | TRUE | A | 97387248 | 3.37E-25 | reported | 2920 | 0.05786 | rs41291566 |  |  |  |  |  |  |  |  |  |  |  |  |  |  |  |
| 0.6789 | 10 | igd | 0.0616 | G | \|\| id:ebi-a-GCST90002032 | ebi-a-GCST90002032 | TRUE | A | 97387248 | 2.10E-31 | reported | 2920 | 0.05756 | rs41291566 |  |  |  |  |  |  |  |  |  |  |  |  |  |  |  |
| 0.3407 | 10 | igd | 0.0615 | T | \|\| id:ebi-a-GCST90001485 | ebi-a-GCST90001485 | TRUE | C | 97989507 | 5.32E-11 | reported | 3437 | 0.05175 | rs72811025 |  |  |  |  |  |  |  |  |  |  |  |  |  |  |  |
| -1.046 | 19 | igd | 0.0615 | T | \|\| id:ebi-a-GCST90001947 | ebi-a-GCST90001947 | TRUE | G | 51685133 | 4.11E-43 | reported | 1577 | 0.0737 | rs116960573 |  |  |  |  |  |  |  |  |  |  |  |  |  |  |  |
| -0.3894 | 22 | igd | 0.0614 | A | \|\| id:ebi-a-GCST90001702 | ebi-a-GCST90001702 | TRUE | G | 39487029 | 1.27E-13 | reported | 3657 | 0.05236 | rs111381709 |  |  |  |  |  |  |  |  |  |  |  |  |  |  |  |
| -0.39 | 22 | igd | 0.0614 | A | \|\| id:ebi-a-GCST90001703 | ebi-a-GCST90001703 | TRUE | G | 39487029 | 1.17E-13 | reported | 3657 | 0.05235 | rs111381709 |  |  |  |  |  |  |  |  |  |  |  |  |  |  |  |
| -0.3864 | 22 | igd | 0.0614 | A | \|\| id:ebi-a-GCST90001704 | ebi-a-GCST90001704 | TRUE | G | 39487029 | 1.81E-13 | reported | 3657 | 0.05229 | rs111381709 |  |  |  |  |  |  |  |  |  |  |  |  |  |  |  |
| -0.3878 | 22 | igd | 0.0614 | A | \|\| id:ebi-a-GCST90001710 | ebi-a-GCST90001710 | TRUE | G | 39487029 | 1.36E-13 | reported | 3657 | 0.05221 | rs111381709 |  |  |  |  |  |  |  |  |  |  |  |  |  |  |  |
| -0.404 | 22 | igd | 0.0614 | A | \|\| id:ebi-a-GCST90001711 | ebi-a-GCST90001711 | TRUE | G | 39487029 | 1.10E-14 | reported | 3657 | 0.05206 | rs111381709 |  |  |  |  |  |  |  |  |  |  |  |  |  |  |  |
| -0.5367 | 1 | igd | 0.0611 | C | \|\| id:ebi-a-GCST90001970 | ebi-a-GCST90001970 | TRUE | T | 161884403 | 9.22E-20 | reported | 2971 | 0.05858 | rs114676248 |  |  |  |  |  |  |  |  |  |  |  |  |  |  |  |
| -0.5569 | 1 | igd | 0.0611 | C | \|\| id:ebi-a-GCST90002077 | ebi-a-GCST90002077 | TRUE | T | 161884403 | 1.73E-21 | reported | 2971 | 0.05805 | rs114676248 |  |  |  |  |  |  |  |  |  |  |  |  |  |  |  |
| 0.5013 | 10 | igd | 0.061 | G | \|\| id:ebi-a-GCST90001484 | ebi-a-GCST90001484 | TRUE | A | 97387248 | 1.15E-21 | reported | 3437 | 0.05207 | rs41291566 |  |  |  |  |  |  |  |  |  |  |  |  |  |  |  |
| 0.4684 | 10 | igd | 0.061 | G | \|\| id:ebi-a-GCST90001485 | ebi-a-GCST90001485 | TRUE | A | 97387248 | 3.91E-19 | reported | 3437 | 0.05209 | rs41291566 |  |  |  |  |  |  |  |  |  |  |  |  |  |  |  |
| 0.4806 | 10 | igd | 0.061 | G | \|\| id:ebi-a-GCST90001491 | ebi-a-GCST90001491 | TRUE | A | 97387248 | 2.67E-19 | reported | 3437 | 0.05319 | rs41291566 |  |  |  |  |  |  |  |  |  |  |  |  |  |  |  |
| 0.6056 | 10 | igd | 0.061 | G | \|\| id:ebi-a-GCST90001496 | ebi-a-GCST90001496 | TRUE | A | 97387248 | 3.91E-29 | reported | 3437 | 0.05355 | rs41291566 |  |  |  |  |  |  |  |  |  |  |  |  |  |  |  |
| 0.6031 | 10 | igd | 0.061 | G | \|\| id:ebi-a-GCST90001497 | ebi-a-GCST90001497 | TRUE | A | 97387248 | 1.30E-29 | reported | 3437 | 0.05287 | rs41291566 |  |  |  |  |  |  |  |  |  |  |  |  |  |  |  |
| 0.5555 | 10 | igd | 0.0609 | G | \|\| id:ebi-a-GCST90001658 | ebi-a-GCST90001658 | TRUE | A | 97387248 | 4.73E-27 | reported | 3440 | 0.05113 | rs41291566 |  |  |  |  |  |  |  |  |  |  |  |  |  |  |  |
| 0.5933 | 10 | igd | 0.0609 | G | \|\| id:ebi-a-GCST90001659 | ebi-a-GCST90001659 | TRUE | A | 97387248 | 1.09E-30 | reported | 3440 | 0.05102 | rs41291566 |  |  |  |  |  |  |  |  |  |  |  |  |  |  |  |
| 0.4147 | 10 | igd | 0.0609 | G | \|\| id:ebi-a-GCST90001670 | ebi-a-GCST90001670 | TRUE | A | 97387248 | 1.43E-14 | reported | 3440 | 0.05367 | rs41291566 |  |  |  |  |  |  |  |  |  |  |  |  |  |  |  |
| 0.3837 | 10 | igd | 0.0609 | G | \|\| id:ebi-a-GCST90001671 | ebi-a-GCST90001671 | TRUE | A | 97387248 | 1.22E-12 | reported | 3440 | 0.05381 | rs41291566 |  |  |  |  |  |  |  |  |  |  |  |  |  |  |  |
| 0.281 | 1 | igd | 0.0609 | A | \|\| id:ebi-a-GCST90001725 | ebi-a-GCST90001725 | TRUE | G | 205241642 | 3.76E-08 | reported | 3656 | 0.05096 | rs79856590 |  |  |  |  |  |  |  |  |  |  |  |  |  |  |  |
| 0.292 | 1 | igd | 0.0609 | A | \|\| id:ebi-a-GCST90001737 | ebi-a-GCST90001737 | TRUE | G | 205241642 | 1.05E-08 | reported | 3653 | 0.05091 | rs79856590 |  |  |  |  |  |  |  |  |  |  |  |  |  |  |  |
| -0.283 | 14 | igd | 0.0609 | G | \|\| id:ebi-a-GCST90001820 | ebi-a-GCST90001820 | TRUE | A | 104798514 | 4.21E-08 | reported | 3656 | 0.05151 | rs77578883 |  |  |  |  |  |  |  |  |  |  |  |  |  |  |  |
| 0.4521 | 10 | igd | 0.0608 | G | \|\| id:ebi-a-GCST90001483 | ebi-a-GCST90001483 | TRUE | A | 97387248 | 1.53E-17 | reported | 3405 | 0.05274 | rs41291566 |  |  |  |  |  |  |  |  |  |  |  |  |  |  |  |
| 0.5029 | 10 | igd | 0.0608 | G | \|\| id:ebi-a-GCST90001489 | ebi-a-GCST90001489 | TRUE | A | 97387248 | 2.46E-20 | reported | 3405 | 0.05408 | rs41291566 |  |  |  |  |  |  |  |  |  |  |  |  |  |  |  |
| 0.6087 | 10 | igd | 0.0608 | G | \|\| id:ebi-a-GCST90001495 | ebi-a-GCST90001495 | TRUE | A | 97387248 | 1.10E-28 | reported | 3405 | 0.05428 | rs41291566 |  |  |  |  |  |  |  |  |  |  |  |  |  |  |  |
| 0.3659 | 22 | igd | 0.0608 | T | \|\| id:ebi-a-GCST90001711 | ebi-a-GCST90001711 | TRUE | C | 42542651 | 1.30E-11 | reported | 3657 | 0.05388 | rs149013899 |  |  |  |  |  |  |  |  |  |  |  |  |  |  |  |
| 0.3722 | 2 | igd | 0.0608 | A | \|\| id:ebi-a-GCST90002098 | ebi-a-GCST90002098 | TRUE | G | 39741498 | 5.56E-12 | reported | 2910 | 0.05379 | rs79291184 |  |  |  |  |  |  |  |  |  |  |  |  |  |  |  |
| 0.4007 | 2 | igd | 0.0608 | A | \|\| id:ebi-a-GCST90002102 | ebi-a-GCST90002102 | TRUE | G | 39741498 | 7.84E-14 | reported | 2919 | 0.05336 | rs79291184 |  |  |  |  |  |  |  |  |  |  |  |  |  |  |  |
| 0.5855 | 10 | igd | 0.0607 | G | \|\| id:ebi-a-GCST90001660 | ebi-a-GCST90001660 | TRUE | A | 97387248 | 3.25E-27 | reported | 3408 | 0.05372 | rs41291566 |  |  |  |  |  |  |  |  |  |  |  |  |  |  |  |
| 0.4347 | 10 | igd | 0.0607 | G | \|\| id:ebi-a-GCST90001672 | ebi-a-GCST90001672 | TRUE | A | 97387248 | 2.54E-15 | reported | 3408 | 0.05468 | rs41291566 |  |  |  |  |  |  |  |  |  |  |  |  |  |  |  |
| -0.3715 | 22 | igd | 0.0606 | G | \|\| id:ebi-a-GCST90001702 | ebi-a-GCST90001702 | TRUE | A | 40363433 | 7.89E-13 | reported | 3657 | 0.05168 | rs2958667 |  |  |  |  |  |  |  |  |  |  |  |  |  |  |  |
| -0.3676 | 22 | igd | 0.0606 | G | \|\| id:ebi-a-GCST90001703 | ebi-a-GCST90001703 | TRUE | A | 40363433 | 1.36E-12 | reported | 3657 | 0.05169 | rs2958667 |  |  |  |  |  |  |  |  |  |  |  |  |  |  |  |
| -0.3521 | 22 | igd | 0.0606 | G | \|\| id:ebi-a-GCST90001704 | ebi-a-GCST90001704 | TRUE | A | 40363433 | 1.08E-11 | reported | 3657 | 0.05165 | rs2958667 |  |  |  |  |  |  |  |  |  |  |  |  |  |  |  |
| -0.3439 | 22 | igd | 0.0606 | G | \|\| id:ebi-a-GCST90001710 | ebi-a-GCST90001710 | TRUE | A | 40363433 | 3.00E-11 | reported | 3657 | 0.05158 | rs2958667 |  |  |  |  |  |  |  |  |  |  |  |  |  |  |  |
| -0.362 | 22 | igd | 0.0606 | G | \|\| id:ebi-a-GCST90001711 | ebi-a-GCST90001711 | TRUE | A | 40363433 | 2.33E-12 | reported | 3657 | 0.05144 | rs2958667 |  |  |  |  |  |  |  |  |  |  |  |  |  |  |  |
| 0.5934 | 6 | igd | 0.0606 | T | \|\| id:ebi-a-GCST90002105 | ebi-a-GCST90002105 | TRUE | C | 32587279 | 2.54E-21 | reported | 2872 | 0.0621 | rs144934262 |  |  |  |  |  |  |  |  |  |  |  |  |  |  |  |
| 0.7137 | 6 | igd | 0.0605 | A | \|\| id:ebi-a-GCST90002108 | ebi-a-GCST90002108 | TRUE | G | 32587249 | 1.20E-18 | reported | 1579 | 0.07996 | rs146566004 |  |  |  |  |  |  |  |  |  |  |  |  |  |  |  |
| -0.3491 | 10 | igd | 0.0603 | A | \|\| id:ebi-a-GCST90002030 | ebi-a-GCST90002030 | TRUE | G | 92053108 | 1.00E-09 | reported | 2920 | 0.05696 | rs117482193 |  |  |  |  |  |  |  |  |  |  |  |  |  |  |  |
| -0.3765 | 10 | igd | 0.0602 | T | \|\| id:ebi-a-GCST90001483 | ebi-a-GCST90001483 | TRUE | G | 97898846 | 1.58E-12 | reported | 3405 | 0.05307 | rs74453195 |  |  |  |  |  |  |  |  |  |  |  |  |  |  |  |
| -0.4687 | 10 | igd | 0.0602 | T | \|\| id:ebi-a-GCST90001489 | ebi-a-GCST90001489 | TRUE | G | 97898846 | 9.51E-18 | reported | 3405 | 0.05433 | rs74453195 |  |  |  |  |  |  |  |  |  |  |  |  |  |  |  |
| -0.5212 | 10 | igd | 0.0602 | T | \|\| id:ebi-a-GCST90001495 | ebi-a-GCST90001495 | TRUE | G | 97898846 | 2.31E-21 | reported | 3405 | 0.05456 | rs74453195 |  |  |  |  |  |  |  |  |  |  |  |  |  |  |  |
| -0.5273 | 10 | igd | 0.0602 | T | \|\| id:ebi-a-GCST90001660 | ebi-a-GCST90001660 | TRUE | G | 97898846 | 2.31E-22 | reported | 3408 | 0.05383 | rs74453195 |  |  |  |  |  |  |  |  |  |  |  |  |  |  |  |
| -0.5276 | 10 | igd | 0.0602 | T | \|\| id:ebi-a-GCST90001672 | ebi-a-GCST90001672 | TRUE | G | 97898846 | 5.62E-22 | reported | 3408 | 0.05437 | rs74453195 |  |  |  |  |  |  |  |  |  |  |  |  |  |  |  |
| -0.4471 | 10 | igd | 0.0601 | T | \|\| id:ebi-a-GCST90001484 | ebi-a-GCST90001484 | TRUE | G | 97898846 | 3.09E-17 | reported | 3437 | 0.05267 | rs74453195 |  |  |  |  |  |  |  |  |  |  |  |  |  |  |  |
| -0.3769 | 10 | igd | 0.0601 | T | \|\| id:ebi-a-GCST90001485 | ebi-a-GCST90001485 | TRUE | G | 97898846 | 1.10E-12 | reported | 3437 | 0.05276 | rs74453195 |  |  |  |  |  |  |  |  |  |  |  |  |  |  |  |
| -0.489 | 10 | igd | 0.0601 | T | \|\| id:ebi-a-GCST90001491 | ebi-a-GCST90001491 | TRUE | G | 97898846 | 9.01E-20 | reported | 3437 | 0.05341 | rs74453195 |  |  |  |  |  |  |  |  |  |  |  |  |  |  |  |
| -0.534 | 10 | igd | 0.0601 | T | \|\| id:ebi-a-GCST90001496 | ebi-a-GCST90001496 | TRUE | G | 97898846 | 9.80E-23 | reported | 3437 | 0.05402 | rs74453195 |  |  |  |  |  |  |  |  |  |  |  |  |  |  |  |
| -0.5653 | 10 | igd | 0.0601 | T | \|\| id:ebi-a-GCST90001497 | ebi-a-GCST90001497 | TRUE | G | 97898846 | 6.00E-26 | reported | 3437 | 0.05323 | rs74453195 |  |  |  |  |  |  |  |  |  |  |  |  |  |  |  |
| -0.5638 | 10 | igd | 0.06 | T | \|\| id:ebi-a-GCST90001658 | ebi-a-GCST90001658 | TRUE | G | 97898846 | 1.17E-27 | reported | 3440 | 0.05128 | rs74453195 |  |  |  |  |  |  |  |  |  |  |  |  |  |  |  |
| -0.5717 | 10 | igd | 0.06 | T | \|\| id:ebi-a-GCST90001659 | ebi-a-GCST90001659 | TRUE | G | 97898846 | 2.11E-28 | reported | 3440 | 0.05126 | rs74453195 |  |  |  |  |  |  |  |  |  |  |  |  |  |  |  |
| -0.5412 | 10 | igd | 0.06 | T | \|\| id:ebi-a-GCST90001670 | ebi-a-GCST90001670 | TRUE | G | 97898846 | 1.16E-23 | reported | 3440 | 0.05358 | rs74453195 |  |  |  |  |  |  |  |  |  |  |  |  |  |  |  |
| -0.5568 | 10 | igd | 0.06 | T | \|\| id:ebi-a-GCST90001671 | ebi-a-GCST90001671 | TRUE | G | 97898846 | 5.90E-25 | reported | 3440 | 0.05355 | rs74453195 |  |  |  |  |  |  |  |  |  |  |  |  |  |  |  |
| -0.318 | 22 | igd | 0.06 | C | \|\| id:ebi-a-GCST90001702 | ebi-a-GCST90001702 | TRUE | T | 39095653 | 2.44E-09 | reported | 3657 | 0.05317 | rs149593440 |  |  |  |  |  |  |  |  |  |  |  |  |  |  |  |
| -0.3293 | 22 | igd | 0.06 | C | \|\| id:ebi-a-GCST90001703 | ebi-a-GCST90001703 | TRUE | T | 39095653 | 6.50E-10 | reported | 3657 | 0.05315 | rs149593440 |  |  |  |  |  |  |  |  |  |  |  |  |  |  |  |
| -0.3496 | 22 | igd | 0.06 | C | \|\| id:ebi-a-GCST90001704 | ebi-a-GCST90001704 | TRUE | T | 39095653 | 5.06E-11 | reported | 3657 | 0.05306 | rs149593440 |  |  |  |  |  |  |  |  |  |  |  |  |  |  |  |
| -0.2959 | 22 | igd | 0.06 | C | \|\| id:ebi-a-GCST90001710 | ebi-a-GCST90001710 | TRUE | T | 39095653 | 2.61E-08 | reported | 3657 | 0.05304 | rs149593440 |  |  |  |  |  |  |  |  |  |  |  |  |  |  |  |
| -0.3314 | 22 | igd | 0.06 | C | \|\| id:ebi-a-GCST90001711 | ebi-a-GCST90001711 | TRUE | T | 39095653 | 4.16E-10 | reported | 3657 | 0.05289 | rs149593440 |  |  |  |  |  |  |  |  |  |  |  |  |  |  |  |
| 0.3543 | 7 | igd | 0.0598 | T | \|\| id:ebi-a-GCST90001966 | ebi-a-GCST90001966 | TRUE | C | 92553149 | 7.22E-09 | reported | 2850 | 0.06106 | rs11768959 |  |  |  |  |  |  |  |  |  |  |  |  |  |  |  |
| 0.35 | 10 | igd | 0.0598 | T | \|\| id:ebi-a-GCST90002030 | ebi-a-GCST90002030 | TRUE | C | 101331215 | 2.36E-09 | reported | 2920 | 0.05843 | rs117246678 |  |  |  |  |  |  |  |  |  |  |  |  |  |  |  |
| 0.3822 | 7 | igd | 0.0598 | T | \|\| id:ebi-a-GCST90002078 | ebi-a-GCST90002078 | TRUE | C | 92553149 | 4.47E-11 | reported | 3112 | 0.0578 | rs11768959 |  |  |  |  |  |  |  |  |  |  |  |  |  |  |  |
| -0.3008 | 22 | igd | 0.0597 | T | \|\| id:ebi-a-GCST90001702 | ebi-a-GCST90001702 | TRUE | C | 37921434 | 2.09E-08 | reported | 3657 | 0.05355 | rs145530421 |  |  |  |  |  |  |  |  |  |  |  |  |  |  |  |
| -0.4153 | 10 | igd | 0.0595 | T | \|\| id:ebi-a-GCST90001483 | ebi-a-GCST90001483 | TRUE | C | 97252852 | 3.83E-15 | reported | 3405 | 0.0526 | rs146155044 |  |  |  |  |  |  |  |  |  |  |  |  |  |  |  |
| -0.4353 | 10 | igd | 0.0595 | T | \|\| id:ebi-a-GCST90001484 | ebi-a-GCST90001484 | TRUE | C | 97252852 | 9.26E-17 | reported | 3437 | 0.05209 | rs146155044 |  |  |  |  |  |  |  |  |  |  |  |  |  |  |  |
| -0.4128 | 10 | igd | 0.0595 | T | \|\| id:ebi-a-GCST90001485 | ebi-a-GCST90001485 | TRUE | C | 97252852 | 3.00E-15 | reported | 3437 | 0.05207 | rs146155044 |  |  |  |  |  |  |  |  |  |  |  |  |  |  |  |
| -0.5694 | 10 | igd | 0.0595 | T | \|\| id:ebi-a-GCST90001489 | ebi-a-GCST90001489 | TRUE | C | 97252852 | 6.44E-26 | reported | 3405 | 0.05364 | rs146155044 |  |  |  |  |  |  |  |  |  |  |  |  |  |  |  |
| -0.5666 | 10 | igd | 0.0595 | T | \|\| id:ebi-a-GCST90001491 | ebi-a-GCST90001491 | TRUE | C | 97252852 | 2.68E-26 | reported | 3437 | 0.05296 | rs146155044 |  |  |  |  |  |  |  |  |  |  |  |  |  |  |  |
| -0.5802 | 10 | igd | 0.0595 | T | \|\| id:ebi-a-GCST90001495 | ebi-a-GCST90001495 | TRUE | C | 97252852 | 2.69E-26 | reported | 3405 | 0.05423 | rs146155044 |  |  |  |  |  |  |  |  |  |  |  |  |  |  |  |
| -0.5639 | 10 | igd | 0.0595 | T | \|\| id:ebi-a-GCST90001496 | ebi-a-GCST90001496 | TRUE | C | 97252852 | 2.09E-25 | reported | 3437 | 0.0537 | rs146155044 |  |  |  |  |  |  |  |  |  |  |  |  |  |  |  |
| -0.5538 | 10 | igd | 0.0595 | T | \|\| id:ebi-a-GCST90001497 | ebi-a-GCST90001497 | TRUE | C | 97252852 | 3.75E-25 | reported | 3437 | 0.05304 | rs146155044 |  |  |  |  |  |  |  |  |  |  |  |  |  |  |  |
| -0.5832 | 10 | igd | 0.0594 | T | \|\| id:ebi-a-GCST90001658 | ebi-a-GCST90001658 | TRUE | C | 97252852 | 1.28E-29 | reported | 3440 | 0.05112 | rs146155044 |  |  |  |  |  |  |  |  |  |  |  |  |  |  |  |
| -0.5974 | 10 | igd | 0.0594 | T | \|\| id:ebi-a-GCST90001659 | ebi-a-GCST90001659 | TRUE | C | 97252852 | 5.14E-31 | reported | 3440 | 0.05108 | rs146155044 |  |  |  |  |  |  |  |  |  |  |  |  |  |  |  |
| -0.6431 | 10 | igd | 0.0594 | T | \|\| id:ebi-a-GCST90001660 | ebi-a-GCST90001660 | TRUE | C | 97252852 | 1.06E-32 | reported | 3408 | 0.05344 | rs146155044 |  |  |  |  |  |  |  |  |  |  |  |  |  |  |  |
| -0.5893 | 10 | igd | 0.0594 | T | \|\| id:ebi-a-GCST90001670 | ebi-a-GCST90001670 | TRUE | C | 97252852 | 4.85E-28 | reported | 3440 | 0.0532 | rs146155044 |  |  |  |  |  |  |  |  |  |  |  |  |  |  |  |
| -0.583 | 10 | igd | 0.0594 | T | \|\| id:ebi-a-GCST90001671 | ebi-a-GCST90001671 | TRUE | C | 97252852 | 2.45E-27 | reported | 3440 | 0.05336 | rs146155044 |  |  |  |  |  |  |  |  |  |  |  |  |  |  |  |
| -0.6236 | 10 | igd | 0.0594 | T | \|\| id:ebi-a-GCST90001672 | ebi-a-GCST90001672 | TRUE | C | 97252852 | 3.16E-30 | reported | 3408 | 0.05406 | rs146155044 |  |  |  |  |  |  |  |  |  |  |  |  |  |  |  |
| -0.35 | 10 | igd | 0.0593 | A | \|\| id:ebi-a-GCST90001483 | ebi-a-GCST90001483 | TRUE | G | 97451102 | 4.94E-11 | reported | 3405 | 0.05308 | rs113574745 |  |  |  |  |  |  |  |  |  |  |  |  |  |  |  |
| -1.001 | 19 | igd | 0.0593 | T | \|\| id:ebi-a-GCST90001952 | ebi-a-GCST90001952 | TRUE | G | 51685133 | 4.60E-37 | reported | 1569 | 0.07666 | rs116960573 |  |  |  |  |  |  |  |  |  |  |  |  |  |  |  |
| 0.496 | 2 | igd | 0.0593 | G | \|\| id:ebi-a-GCST90002098 | ebi-a-GCST90002098 | TRUE | T | 39342502 | 7.63E-19 | reported | 2910 | 0.05557 | rs78092438 |  |  |  |  |  |  |  |  |  |  |  |  |  |  |  |
| -0.4428 | 3 | igd | 0.0593 | T | \|\| id:ebi-a-GCST90002017 | ebi-a-GCST90002017 | TRUE | C | 46392976 | 4.69E-13 | reported | 2850 | 0.06093 | rs34473395 |  |  |  |  |  |  |  |  |  |  |  |  |  |  |  |
| -0.3525 | 10 | igd | 0.0592 | A | \|\| id:ebi-a-GCST90001485 | ebi-a-GCST90001485 | TRUE | G | 97451102 | 2.73E-11 | reported | 3437 | 0.05275 | rs113574745 |  |  |  |  |  |  |  |  |  |  |  |  |  |  |  |
| 0.4495 | 10 | igd | 0.0592 | T | \|\| id:ebi-a-GCST90001961 | ebi-a-GCST90001961 | TRUE | C | 97252852 | 1.22E-14 | reported | 2920 | 0.05798 | rs146155044 |  |  |  |  |  |  |  |  |  |  |  |  |  |  |  |
| -0.6244 | 10 | igd | 0.0592 | T | \|\| id:ebi-a-GCST90002030 | ebi-a-GCST90002030 | TRUE | C | 97252852 | 3.38E-27 | reported | 2920 | 0.05723 | rs146155044 |  |  |  |  |  |  |  |  |  |  |  |  |  |  |  |
| -0.51 | 10 | igd | 0.0592 | T | \|\| id:ebi-a-GCST90002031 | ebi-a-GCST90002031 | TRUE | C | 97252852 | 5.17E-18 | reported | 2920 | 0.05858 | rs146155044 |  |  |  |  |  |  |  |  |  |  |  |  |  |  |  |
| -0.446 | 10 | igd | 0.0592 | T | \|\| id:ebi-a-GCST90002032 | ebi-a-GCST90002032 | TRUE | C | 97252852 | 4.28E-14 | reported | 2920 | 0.05877 | rs146155044 |  |  |  |  |  |  |  |  |  |  |  |  |  |  |  |
| 0.3643 | 19 | igd | 0.05918 | C | \|\| id:prot-a-439 | prot-a-439 | TRUE | T | 51678482 | 1.05E-11 | reported | 3301 | 0.0536 | rs7254514 |  |  |  |  |  |  |  |  |  |  |  |  |  |  |  |
| -0.5075 | 22 | igd | 0.0591 | T | \|\| id:ebi-a-GCST90001702 | ebi-a-GCST90001702 | TRUE | C | 40590761 | 8.43E-22 | reported | 3657 | 0.05256 | rs142202860 |  |  |  |  |  |  |  |  |  |  |  |  |  |  |  |
| -0.5188 | 22 | igd | 0.0591 | T | \|\| id:ebi-a-GCST90001703 | ebi-a-GCST90001703 | TRUE | C | 40590761 | 9.90E-23 | reported | 3657 | 0.05252 | rs142202860 |  |  |  |  |  |  |  |  |  |  |  |  |  |  |  |
| -0.5556 | 22 | igd | 0.0591 | T | \|\| id:ebi-a-GCST90001704 | ebi-a-GCST90001704 | TRUE | C | 40590761 | 6.18E-26 | reported | 3657 | 0.05235 | rs142202860 |  |  |  |  |  |  |  |  |  |  |  |  |  |  |  |
| -0.4902 | 22 | igd | 0.0591 | T | \|\| id:ebi-a-GCST90001710 | ebi-a-GCST90001710 | TRUE | C | 40590761 | 1.54E-20 | reported | 3657 | 0.05245 | rs142202860 |  |  |  |  |  |  |  |  |  |  |  |  |  |  |  |
| -0.5087 | 22 | igd | 0.0591 | T | \|\| id:ebi-a-GCST90001711 | ebi-a-GCST90001711 | TRUE | C | 40590761 | 4.17E-22 | reported | 3657 | 0.05228 | rs142202860 |  |  |  |  |  |  |  |  |  |  |  |  |  |  |  |
| 0.4202 | 10 | igd | 0.0591 | T | \|\| id:ebi-a-GCST90002030 | ebi-a-GCST90002030 | TRUE | C | 97175465 | 5.78E-13 | reported | 2920 | 0.05805 | rs41315247 |  |  |  |  |  |  |  |  |  |  |  |  |  |  |  |
| 0.375 | 10 | igd | 0.0591 | T | \|\| id:ebi-a-GCST90002031 | ebi-a-GCST90002031 | TRUE | C | 97175465 | 3.50E-10 | reported | 2920 | 0.05956 | rs41315247 |  |  |  |  |  |  |  |  |  |  |  |  |  |  |  |
| 0.3318 | 10 | igd | 0.0591 | T | \|\| id:ebi-a-GCST90002032 | ebi-a-GCST90002032 | TRUE | C | 92681475 | 3.09E-08 | reported | 2920 | 0.05976 | rs3824739 |  |  |  |  |  |  |  |  |  |  |  |  |  |  |  |
| 0.3939 | 10 | igd | 0.0591 | T | \|\| id:ebi-a-GCST90002032 | ebi-a-GCST90002032 | TRUE | C | 97175465 | 4.19E-11 | reported | 2920 | 0.05948 | rs41315247 |  |  |  |  |  |  |  |  |  |  |  |  |  |  |  |
| 0.3054 | 10 | igd | 0.059 | A | \|\| id:ebi-a-GCST90001489 | ebi-a-GCST90001489 | TRUE | G | 101330656 | 3.66E-08 | reported | 3405 | 0.05533 | rs11190156 |  |  |  |  |  |  |  |  |  |  |  |  |  |  |  |
| 0.3285 | 10 | igd | 0.059 | A | \|\| id:ebi-a-GCST90001495 | ebi-a-GCST90001495 | TRUE | G | 101330656 | 4.66E-09 | reported | 3405 | 0.05593 | rs11190156 |  |  |  |  |  |  |  |  |  |  |  |  |  |  |  |
| 0.362 | 10 | igd | 0.059 | A | \|\| id:ebi-a-GCST90001660 | ebi-a-GCST90001660 | TRUE | G | 101330656 | 6.61E-11 | reported | 3408 | 0.05527 | rs11190156 |  |  |  |  |  |  |  |  |  |  |  |  |  |  |  |
| 0.4039 | 10 | igd | 0.059 | A | \|\| id:ebi-a-GCST90001672 | ebi-a-GCST90001672 | TRUE | G | 101330656 | 5.27E-13 | reported | 3408 | 0.05574 | rs11190156 |  |  |  |  |  |  |  |  |  |  |  |  |  |  |  |
| 0.4767 | 19 | igd | 0.05892 | A | \|\| id:prot-a-439 | prot-a-439 | TRUE | G | 51721244 | 8.32E-18 | reported | 3301 | 0.0555 | rs79254320 |  |  |  |  |  |  |  |  |  |  |  |  |  |  |  |
| 0.4474 | 10 | igd | 0.0589 | G | \|\| id:ebi-a-GCST90002030 | ebi-a-GCST90002030 | TRUE | A | 97285715 | 9.44E-15 | reported | 2920 | 0.05745 | rs72809757 |  |  |  |  |  |  |  |  |  |  |  |  |  |  |  |
| 0.4013 | 10 | igd | 0.0589 | G | \|\| id:ebi-a-GCST90002031 | ebi-a-GCST90002031 | TRUE | A | 97285715 | 1.19E-11 | reported | 2920 | 0.05895 | rs72809757 |  |  |  |  |  |  |  |  |  |  |  |  |  |  |  |
| -0.4895 | 3 | igd | 0.0589 | T | \|\| id:ebi-a-GCST90002014 | ebi-a-GCST90002014 | TRUE | C | 46381507 | 3.41E-15 | reported | 2870 | 0.06182 | rs71327053 |  |  |  |  |  |  |  |  |  |  |  |  |  |  |  |
| 0.5451 | 10 | igd | 0.0588 | G | \|\| id:ebi-a-GCST90001484 | ebi-a-GCST90001484 | TRUE | A | 97285715 | 7.07E-25 | reported | 3437 | 0.05251 | rs72809757 |  |  |  |  |  |  |  |  |  |  |  |  |  |  |  |
| 0.5051 | 10 | igd | 0.0588 | G | \|\| id:ebi-a-GCST90001485 | ebi-a-GCST90001485 | TRUE | A | 97285715 | 1.33E-21 | reported | 3437 | 0.05255 | rs72809757 |  |  |  |  |  |  |  |  |  |  |  |  |  |  |  |
| 0.4184 | 10 | igd | 0.0588 | G | \|\| id:ebi-a-GCST90001491 | ebi-a-GCST90001491 | TRUE | A | 97285715 | 9.94E-15 | reported | 3437 | 0.05382 | rs72809757 |  |  |  |  |  |  |  |  |  |  |  |  |  |  |  |
| 0.315 | 10 | igd | 0.0588 | A | \|\| id:ebi-a-GCST90001491 | ebi-a-GCST90001491 | TRUE | G | 101330656 | 8.98E-09 | reported | 3437 | 0.05466 | rs11190156 |  |  |  |  |  |  |  |  |  |  |  |  |  |  |  |
| 0.351 | 10 | igd | 0.0588 | A | \|\| id:ebi-a-GCST90001496 | ebi-a-GCST90001496 | TRUE | G | 101330656 | 2.54E-10 | reported | 3437 | 0.05534 | rs11190156 |  |  |  |  |  |  |  |  |  |  |  |  |  |  |  |
| 0.562 | 10 | igd | 0.0588 | G | \|\| id:ebi-a-GCST90001496 | ebi-a-GCST90001496 | TRUE | A | 97285715 | 7.59E-25 | reported | 3437 | 0.05417 | rs72809757 |  |  |  |  |  |  |  |  |  |  |  |  |  |  |  |
| 0.5322 | 10 | igd | 0.0588 | G | \|\| id:ebi-a-GCST90001497 | ebi-a-GCST90001497 | TRUE | A | 97285715 | 5.92E-23 | reported | 3437 | 0.05356 | rs72809757 |  |  |  |  |  |  |  |  |  |  |  |  |  |  |  |
| 0.3321 | 10 | igd | 0.0588 | A | \|\| id:ebi-a-GCST90001497 | ebi-a-GCST90001497 | TRUE | G | 101330656 | 1.38E-09 | reported | 3437 | 0.05467 | rs11190156 |  |  |  |  |  |  |  |  |  |  |  |  |  |  |  |
| -0.343 | 1 | igd | 0.0588 | C | \|\| id:ebi-a-GCST90001989 | ebi-a-GCST90001989 | TRUE | G | 161471320 | 1.03E-10 | reported | 3621 | 0.05292 | rs113727631 |  |  |  |  |  |  |  |  |  |  |  |  |  |  |  |
| -0.3027 | 10 | igd | 0.0587 | A | \|\| id:ebi-a-GCST90001495 | ebi-a-GCST90001495 | TRUE | G | 92053108 | 4.84E-08 | reported | 3405 | 0.05535 | rs117482193 |  |  |  |  |  |  |  |  |  |  |  |  |  |  |  |
| 0.4416 | 10 | igd | 0.0587 | G | \|\| id:ebi-a-GCST90001658 | ebi-a-GCST90001658 | TRUE | A | 97285715 | 2.58E-17 | reported | 3440 | 0.05189 | rs72809757 |  |  |  |  |  |  |  |  |  |  |  |  |  |  |  |
| 0.3091 | 10 | igd | 0.0587 | A | \|\| id:ebi-a-GCST90001658 | ebi-a-GCST90001658 | TRUE | G | 101330656 | 5.47E-09 | reported | 3440 | 0.05287 | rs11190156 |  |  |  |  |  |  |  |  |  |  |  |  |  |  |  |
| 0.3334 | 10 | igd | 0.0587 | A | \|\| id:ebi-a-GCST90001659 | ebi-a-GCST90001659 | TRUE | G | 101330656 | 3.16E-10 | reported | 3440 | 0.05283 | rs11190156 |  |  |  |  |  |  |  |  |  |  |  |  |  |  |  |
| 0.4701 | 10 | igd | 0.0587 | G | \|\| id:ebi-a-GCST90001659 | ebi-a-GCST90001659 | TRUE | A | 97285715 | 1.94E-19 | reported | 3440 | 0.05183 | rs72809757 |  |  |  |  |  |  |  |  |  |  |  |  |  |  |  |
| -0.3301 | 10 | igd | 0.0587 | A | \|\| id:ebi-a-GCST90001660 | ebi-a-GCST90001660 | TRUE | G | 92053108 | 1.64E-09 | reported | 3408 | 0.05458 | rs117482193 |  |  |  |  |  |  |  |  |  |  |  |  |  |  |  |
| 0.3747 | 10 | igd | 0.0587 | A | \|\| id:ebi-a-GCST90001670 | ebi-a-GCST90001670 | TRUE | G | 101330656 | 1.01E-11 | reported | 3440 | 0.05487 | rs11190156 |  |  |  |  |  |  |  |  |  |  |  |  |  |  |  |
| 0.3838 | 10 | igd | 0.0587 | G | \|\| id:ebi-a-GCST90001671 | ebi-a-GCST90001671 | TRUE | A | 97285715 | 1.84E-12 | reported | 3440 | 0.05426 | rs72809757 |  |  |  |  |  |  |  |  |  |  |  |  |  |  |  |
| 0.3616 | 10 | igd | 0.0587 | A | \|\| id:ebi-a-GCST90001671 | ebi-a-GCST90001671 | TRUE | G | 101330656 | 5.79E-11 | reported | 3440 | 0.05504 | rs11190156 |  |  |  |  |  |  |  |  |  |  |  |  |  |  |  |
| -0.4361 | 3 | igd | 0.0587 | T | \|\| id:ebi-a-GCST90002013 | ebi-a-GCST90002013 | TRUE | C | 46381772 | 1.09E-12 | reported | 2870 | 0.06098 | rs71327054 |  |  |  |  |  |  |  |  |  |  |  |  |  |  |  |
| 0.4827 | 10 | igd | 0.0586 | G | \|\| id:ebi-a-GCST90001483 | ebi-a-GCST90001483 | TRUE | A | 97285715 | 1.94E-19 | reported | 3405 | 0.05321 | rs72809757 |  |  |  |  |  |  |  |  |  |  |  |  |  |  |  |
| -0.7352 | 6 | igd | 0.0586 | G | \|\| id:ebi-a-GCST90001771 | ebi-a-GCST90001771 | TRUE | A | 107168477 | 6.21E-45 | reported | 3653 | 0.05156 | rs72948545 |  |  |  |  |  |  |  |  |  |  |  |  |  |  |  |
| -0.7073 | 6 | igd | 0.0586 | G | \|\| id:ebi-a-GCST90001773 | ebi-a-GCST90001773 | TRUE | A | 107168477 | 1.21E-40 | reported | 3652 | 0.05233 | rs72948545 |  |  |  |  |  |  |  |  |  |  |  |  |  |  |  |
| 0.3112 | 22 | igd | 0.0585 | A | \|\| id:ebi-a-GCST90001711 | ebi-a-GCST90001711 | TRUE | G | 40201703 | 8.16E-09 | reported | 3657 | 0.05386 | rs55713823 |  |  |  |  |  |  |  |  |  |  |  |  |  |  |  |
| 0.3221 | 20 | igd | 0.0584 | T | \|\| id:ebi-a-GCST90001804 | ebi-a-GCST90001804 | TRUE | C | 44834728 | 6.07E-10 | reported | 3657 | 0.0519 | rs138361462 |  |  |  |  |  |  |  |  |  |  |  |  |  |  |  |
| 0.3976 | 10 | igd | 0.0583 | G | \|\| id:ebi-a-GCST90001496 | ebi-a-GCST90001496 | TRUE | A | 97765208 | 8.18E-14 | reported | 3437 | 0.05303 | rs117561824 |  |  |  |  |  |  |  |  |  |  |  |  |  |  |  |
| 0.3977 | 10 | igd | 0.0583 | G | \|\| id:ebi-a-GCST90001497 | ebi-a-GCST90001497 | TRUE | A | 97765208 | 3.87E-14 | reported | 3437 | 0.05235 | rs117561824 |  |  |  |  |  |  |  |  |  |  |  |  |  |  |  |
| -0.3263 | 10 | igd | 0.0583 | A | \|\| id:ebi-a-GCST90001658 | ebi-a-GCST90001658 | TRUE | G | 92053108 | 4.48E-10 | reported | 3440 | 0.05217 | rs117482193 |  |  |  |  |  |  |  |  |  |  |  |  |  |  |  |
| -0.3289 | 10 | igd | 0.0583 | A | \|\| id:ebi-a-GCST90001659 | ebi-a-GCST90001659 | TRUE | G | 92053108 | 3.28E-10 | reported | 3440 | 0.05217 | rs117482193 |  |  |  |  |  |  |  |  |  |  |  |  |  |  |  |
| 0.3822 | 10 | igd | 0.0583 | G | \|\| id:ebi-a-GCST90001659 | ebi-a-GCST90001659 | TRUE | A | 97765208 | 5.01E-14 | reported | 3440 | 0.05053 | rs117561824 |  |  |  |  |  |  |  |  |  |  |  |  |  |  |  |
| -0.3098 | 10 | igd | 0.0583 | A | \|\| id:ebi-a-GCST90001670 | ebi-a-GCST90001670 | TRUE | G | 92053108 | 1.33E-08 | reported | 3440 | 0.05439 | rs117482193 |  |  |  |  |  |  |  |  |  |  |  |  |  |  |  |
| 0.4369 | 6 | igd | 0.0583 | T | \|\| id:ebi-a-GCST90002105 | ebi-a-GCST90002105 | TRUE | C | 26189305 | 2.66E-12 | reported | 2872 | 0.06218 | rs113589835 |  |  |  |  |  |  |  |  |  |  |  |  |  |  |  |
| -0.4952 | 22 | igd | 0.0582 | T | \|\| id:ebi-a-GCST90001702 | ebi-a-GCST90001702 | TRUE | C | 41782480 | 3.63E-19 | reported | 3657 | 0.05504 | rs148614978 |  |  |  |  |  |  |  |  |  |  |  |  |  |  |  |
| -0.4702 | 22 | igd | 0.0582 | T | \|\| id:ebi-a-GCST90001703 | ebi-a-GCST90001703 | TRUE | C | 41782480 | 1.96E-17 | reported | 3657 | 0.05507 | rs148614978 |  |  |  |  |  |  |  |  |  |  |  |  |  |  |  |
| -0.3826 | 22 | igd | 0.0582 | T | \|\| id:ebi-a-GCST90001704 | ebi-a-GCST90001704 | TRUE | C | 41782480 | 4.79E-12 | reported | 3657 | 0.05517 | rs148614978 |  |  |  |  |  |  |  |  |  |  |  |  |  |  |  |
| -0.4695 | 22 | igd | 0.0582 | T | \|\| id:ebi-a-GCST90001710 | ebi-a-GCST90001710 | TRUE | C | 41782480 | 1.93E-17 | reported | 3657 | 0.05498 | rs148614978 |  |  |  |  |  |  |  |  |  |  |  |  |  |  |  |
| -0.4627 | 22 | igd | 0.0582 | T | \|\| id:ebi-a-GCST90001711 | ebi-a-GCST90001711 | TRUE | C | 41782480 | 4.33E-17 | reported | 3657 | 0.05479 | rs148614978 |  |  |  |  |  |  |  |  |  |  |  |  |  |  |  |
| -1.013 | 19 | igd | 0.0582 | A | \|\| id:ebi-a-GCST90001955 | ebi-a-GCST90001955 | TRUE | C | 51692690 | 5.29E-38 | reported | 1554 | 0.07651 | rs71358815 |  |  |  |  |  |  |  |  |  |  |  |  |  |  |  |
| 0.5778 | 19 | igd | 0.0582 | A | \|\| id:ebi-a-GCST90002052 | ebi-a-GCST90002052 | TRUE | C | 51692690 | 6.05E-13 | reported | 1555 | 0.07957 | rs71358815 |  |  |  |  |  |  |  |  |  |  |  |  |  |  |  |
| -0.5211 | 22 | igd | 0.0578 | C | \|\| id:ebi-a-GCST90001702 | ebi-a-GCST90001702 | TRUE | G | 41155371 | 8.80E-21 | reported | 3657 | 0.0554 | rs35040955 |  |  |  |  |  |  |  |  |  |  |  |  |  |  |  |
| -0.5101 | 22 | igd | 0.0578 | C | \|\| id:ebi-a-GCST90001703 | ebi-a-GCST90001703 | TRUE | G | 41155371 | 5.53E-20 | reported | 3657 | 0.05541 | rs35040955 |  |  |  |  |  |  |  |  |  |  |  |  |  |  |  |
| -0.526 | 22 | igd | 0.0578 | C | \|\| id:ebi-a-GCST90001704 | ebi-a-GCST90001704 | TRUE | G | 41155371 | 3.25E-21 | reported | 3657 | 0.05529 | rs35040955 |  |  |  |  |  |  |  |  |  |  |  |  |  |  |  |
| -0.5327 | 22 | igd | 0.0578 | C | \|\| id:ebi-a-GCST90001710 | ebi-a-GCST90001710 | TRUE | G | 41155371 | 9.23E-22 | reported | 3657 | 0.05522 | rs35040955 |  |  |  |  |  |  |  |  |  |  |  |  |  |  |  |
| -0.535 | 22 | igd | 0.0578 | C | \|\| id:ebi-a-GCST90001711 | ebi-a-GCST90001711 | TRUE | G | 41155371 | 4.71E-22 | reported | 3657 | 0.05506 | rs35040955 |  |  |  |  |  |  |  |  |  |  |  |  |  |  |  |
| -1.018 | 19 | igd | 0.0578 | T | \|\| id:ebi-a-GCST90001953 | ebi-a-GCST90001953 | TRUE | G | 51685133 | 4.15E-38 | reported | 1558 | 0.07678 | rs116960573 |  |  |  |  |  |  |  |  |  |  |  |  |  |  |  |
| 1.19 | 6 | igd | 0.0578 | T | \|\| id:ebi-a-GCST90002105 | ebi-a-GCST90002105 | TRUE | C | 32683055 | 4.32E-89 | reported | 2872 | 0.05741 | rs3873448 |  |  |  |  |  |  |  |  |  |  |  |  |  |  |  |
| 0.3126 | 10 | igd | 0.0575 | T | \|\| id:ebi-a-GCST90001491 | ebi-a-GCST90001491 | TRUE | C | 97175465 | 1.78E-08 | reported | 3437 | 0.05536 | rs41315247 |  |  |  |  |  |  |  |  |  |  |  |  |  |  |  |
| 0.386 | 10 | igd | 0.0575 | T | \|\| id:ebi-a-GCST90001960 | ebi-a-GCST90001960 | TRUE | C | 6094697 | 1.42E-11 | reported | 2920 | 0.0569 | rs61839660 |  |  |  |  |  |  |  |  |  |  |  |  |  |  |  |
| -0.3352 | 10 | igd | 0.0575 | T | \|\| id:ebi-a-GCST90002032 | ebi-a-GCST90002032 | TRUE | C | 96784337 | 1.82E-08 | reported | 2920 | 0.05939 | rs185278477 |  |  |  |  |  |  |  |  |  |  |  |  |  |  |  |
| 0.8684 | 6 | igd | 0.0575 | A | \|\| id:ebi-a-GCST90002112 | ebi-a-GCST90002112 | TRUE | G | 32433384 | 3.33E-25 | reported | 1635 | 0.08234 | rs114433295 |  |  |  |  |  |  |  |  |  |  |  |  |  |  |  |
| 0.3263 | 10 | igd | 0.0574 | T | \|\| id:ebi-a-GCST90001658 | ebi-a-GCST90001658 | TRUE | C | 97175465 | 1.13E-09 | reported | 3440 | 0.05344 | rs41315247 |  |  |  |  |  |  |  |  |  |  |  |  |  |  |  |
| 0.3183 | 10 | igd | 0.0574 | T | \|\| id:ebi-a-GCST90001659 | ebi-a-GCST90001659 | TRUE | C | 97175465 | 2.87E-09 | reported | 3440 | 0.05346 | rs41315247 |  |  |  |  |  |  |  |  |  |  |  |  |  |  |  |
| 0.3441 | 20 | igd | 0.0573 | G | \|\| id:ebi-a-GCST90001989 | ebi-a-GCST90001989 | TRUE | C | 42333293 | 2.00E-10 | reported | 3621 | 0.05393 | rs6017147 |  |  |  |  |  |  |  |  |  |  |  |  |  |  |  |
| -0.3579 | 11 | igd | 0.0573 | T | \|\| id:ebi-a-GCST90002071 | ebi-a-GCST90002071 | TRUE | C | 4960640 | 3.38E-09 | reported | 2872 | 0.06035 | rs142456232 |  |  |  |  |  |  |  |  |  |  |  |  |  |  |  |
| -0.3341 | 11 | igd | 0.0573 | T | \|\| id:ebi-a-GCST90002072 | ebi-a-GCST90002072 | TRUE | C | 4960640 | 1.81E-08 | reported | 2872 | 0.05919 | rs142456232 |  |  |  |  |  |  |  |  |  |  |  |  |  |  |  |
| 0.3398 | 6 | igd | 0.0573 | T | \|\| id:ebi-a-GCST90002105 | ebi-a-GCST90002105 | TRUE | C | 30886729 | 2.86E-08 | reported | 2872 | 0.06105 | rs41273021 |  |  |  |  |  |  |  |  |  |  |  |  |  |  |  |
| -1.024 | 19 | igd | 0.0572 | T | \|\| id:ebi-a-GCST90001954 | ebi-a-GCST90001954 | TRUE | G | 51685133 | 3.95E-38 | reported | 1555 | 0.07721 | rs116960573 |  |  |  |  |  |  |  |  |  |  |  |  |  |  |  |
| -0.3287 | 11 | igd | 0.0572 | T | \|\| id:ebi-a-GCST90001967 | ebi-a-GCST90001967 | TRUE | C | 4960640 | 1.55E-08 | reported | 3112 | 0.05796 | rs142456232 |  |  |  |  |  |  |  |  |  |  |  |  |  |  |  |
| 0.3357 | 10 | igd | 0.0571 | T | \|\| id:ebi-a-GCST90001489 | ebi-a-GCST90001489 | TRUE | C | 97175465 | 2.77E-09 | reported | 3405 | 0.05632 | rs41315247 |  |  |  |  |  |  |  |  |  |  |  |  |  |  |  |
| 0.3469 | 10 | igd | 0.0571 | T | \|\| id:ebi-a-GCST90001495 | ebi-a-GCST90001495 | TRUE | C | 97175465 | 1.16E-09 | reported | 3405 | 0.05684 | rs41315247 |  |  |  |  |  |  |  |  |  |  |  |  |  |  |  |
| 0.4371 | 2 | igd | 0.057 | A | \|\| id:ebi-a-GCST90001896 | ebi-a-GCST90001896 | TRUE | G | 204812486 | 1.18E-12 | reported | 2920 | 0.06122 | rs80303378 |  |  |  |  |  |  |  |  |  |  |  |  |  |  |  |
| 0.3305 | 20 | igd | 0.057 | G | \|\| id:ebi-a-GCST90001989 | ebi-a-GCST90001989 | TRUE | T | 44531417 | 1.27E-09 | reported | 3621 | 0.0543 | rs11569649 |  |  |  |  |  |  |  |  |  |  |  |  |  |  |  |
| 0.3059 | 10 | igd | 0.0569 | G | \|\| id:ebi-a-GCST90001496 | ebi-a-GCST90001496 | TRUE | C | 94659515 | 2.29E-08 | reported | 3437 | 0.05461 | rs11187206 |  |  |  |  |  |  |  |  |  |  |  |  |  |  |  |
| 0.309 | 10 | igd | 0.0569 | G | \|\| id:ebi-a-GCST90001497 | ebi-a-GCST90001497 | TRUE | C | 94659515 | 1.09E-08 | reported | 3437 | 0.05392 | rs11187206 |  |  |  |  |  |  |  |  |  |  |  |  |  |  |  |
| 0.3258 | 20 | igd | 0.0569 | A | \|\| id:ebi-a-GCST90001802 | ebi-a-GCST90001802 | TRUE | T | 44818602 | 4.39E-09 | reported | 3657 | 0.05538 | rs1467401 |  |  |  |  |  |  |  |  |  |  |  |  |  |  |  |
| 0.3307 | 20 | igd | 0.0569 | A | \|\| id:ebi-a-GCST90001804 | ebi-a-GCST90001804 | TRUE | T | 44818602 | 1.45E-09 | reported | 3657 | 0.05453 | rs1467401 |  |  |  |  |  |  |  |  |  |  |  |  |  |  |  |
| 0.3103 | 20 | igd | 0.0569 | A | \|\| id:ebi-a-GCST90001805 | ebi-a-GCST90001805 | TRUE | T | 44818602 | 2.26E-08 | reported | 3657 | 0.05537 | rs1467401 |  |  |  |  |  |  |  |  |  |  |  |  |  |  |  |
| 0.3384 | 20 | igd | 0.0569 | A | \|\| id:ebi-a-GCST90001808 | ebi-a-GCST90001808 | TRUE | T | 44818602 | 9.82E-10 | reported | 3657 | 0.05522 | rs1467401 |  |  |  |  |  |  |  |  |  |  |  |  |  |  |  |
| 0.3709 | 6 | igd | 0.0568 | T | \|\| id:ebi-a-GCST90002105 | ebi-a-GCST90002105 | TRUE | C | 30783487 | 1.59E-09 | reported | 2872 | 0.06125 | rs142090224 |  |  |  |  |  |  |  |  |  |  |  |  |  |  |  |
| -0.5001 | 22 | igd | 0.0567 | C | \|\| id:ebi-a-GCST90001702 | ebi-a-GCST90001702 | TRUE | A | 40886744 | 1.22E-19 | reported | 3657 | 0.05484 | rs5750948 |  |  |  |  |  |  |  |  |  |  |  |  |  |  |  |
| -0.4919 | 22 | igd | 0.0567 | C | \|\| id:ebi-a-GCST90001703 | ebi-a-GCST90001703 | TRUE | A | 40886744 | 4.71E-19 | reported | 3657 | 0.05485 | rs5750948 |  |  |  |  |  |  |  |  |  |  |  |  |  |  |  |
| -0.5174 | 22 | igd | 0.0567 | C | \|\| id:ebi-a-GCST90001704 | ebi-a-GCST90001704 | TRUE | A | 40886744 | 5.45E-21 | reported | 3657 | 0.05471 | rs5750948 |  |  |  |  |  |  |  |  |  |  |  |  |  |  |  |
| 0.3184 | 22 | igd | 0.0567 | A | \|\| id:ebi-a-GCST90001710 | ebi-a-GCST90001710 | TRUE | G | 41959827 | 6.84E-09 | reported | 3657 | 0.05482 | rs140413410 |  |  |  |  |  |  |  |  |  |  |  |  |  |  |  |
| -0.5173 | 22 | igd | 0.0567 | C | \|\| id:ebi-a-GCST90001710 | ebi-a-GCST90001710 | TRUE | A | 40886744 | 5.04E-21 | reported | 3657 | 0.05465 | rs5750948 |  |  |  |  |  |  |  |  |  |  |  |  |  |  |  |
| -0.5113 | 22 | igd | 0.0567 | C | \|\| id:ebi-a-GCST90001711 | ebi-a-GCST90001711 | TRUE | A | 40886744 | 1.14E-20 | reported | 3657 | 0.05452 | rs5750948 |  |  |  |  |  |  |  |  |  |  |  |  |  |  |  |
| -0.3647 | 2 | igd | 0.0567 | G | \|\| id:ebi-a-GCST90001891 | ebi-a-GCST90001891 | TRUE | A | 204661621 | 9.35E-10 | reported | 2920 | 0.0594 | rs16840128 |  |  |  |  |  |  |  |  |  |  |  |  |  |  |  |
| -0.3359 | 2 | igd | 0.0567 | G | \|\| id:ebi-a-GCST90001894 | ebi-a-GCST90001894 | TRUE | A | 204661621 | 2.00E-08 | reported | 2920 | 0.05969 | rs16840128 |  |  |  |  |  |  |  |  |  |  |  |  |  |  |  |
| 0.4819 | 2 | igd | 0.0567 | C | \|\| id:ebi-a-GCST90001896 | ebi-a-GCST90001896 | TRUE | G | 204596768 | 5.64E-15 | reported | 2920 | 0.06136 | rs78307830 |  |  |  |  |  |  |  |  |  |  |  |  |  |  |  |
| -0.3511 | 2 | igd | 0.0567 | G | \|\| id:ebi-a-GCST90001898 | ebi-a-GCST90001898 | TRUE | A | 204661621 | 4.39E-09 | reported | 2920 | 0.05964 | rs16840128 |  |  |  |  |  |  |  |  |  |  |  |  |  |  |  |
| -0.3327 | 2 | igd | 0.0567 | G | \|\| id:ebi-a-GCST90001900 | ebi-a-GCST90001900 | TRUE | A | 204661621 | 2.82E-08 | reported | 2919 | 0.05976 | rs16840128 |  |  |  |  |  |  |  |  |  |  |  |  |  |  |  |
| 0.4398 | 3 | igd | 0.0566 | A | \|\| id:ebi-a-GCST90001801 | ebi-a-GCST90001801 | TRUE | G | 32848142 | 5.75E-09 | reported | 1836 | 0.07516 | rs140924680 |  |  |  |  |  |  |  |  |  |  |  |  |  |  |  |
| 0.4881 | 1 | igd | 0.0566 | A | \|\| id:ebi-a-GCST90001954 | ebi-a-GCST90001954 | TRUE | G | 247719769 | 6.30E-10 | reported | 1555 | 0.07845 | rs56043070 |  |  |  |  |  |  |  |  |  |  |  |  |  |  |  |
| 0.377 | 6 | igd | 0.0566 | T | \|\| id:ebi-a-GCST90002105 | ebi-a-GCST90002105 | TRUE | C | 29502128 | 1.33E-08 | reported | 2872 | 0.06616 | rs6456964 |  |  |  |  |  |  |  |  |  |  |  |  |  |  |  |
| 0.3319 | 10 | igd | 0.0565 | G | \|\| id:ebi-a-GCST90001660 | ebi-a-GCST90001660 | TRUE | A | 105786634 | 1.09E-09 | reported | 3408 | 0.0543 | rs45559432 |  |  |  |  |  |  |  |  |  |  |  |  |  |  |  |
| 43.94 | 1 | igd | 0.0565 | A | \|\| id:ebi-a-GCST90001698 | ebi-a-GCST90001698 | TRUE | G | 52644883 | 4.03E-08 | reported | 3408 | 7.987 | rs143940797 |  |  |  |  |  |  |  |  |  |  |  |  |  |  |  |
| 0.5006 | 1 | igd | 0.0565 | A | \|\| id:ebi-a-GCST90001953 | ebi-a-GCST90001953 | TRUE | G | 247719769 | 2.26E-10 | reported | 1558 | 0.0784 | rs56043070 |  |  |  |  |  |  |  |  |  |  |  |  |  |  |  |
| 0.9344 | 3 | igd | 0.0564 | C | \|\| id:ebi-a-GCST90001801 | ebi-a-GCST90001801 | TRUE | T | 32533246 | 1.08E-38 | reported | 1836 | 0.07016 | rs35574803 |  |  |  |  |  |  |  |  |  |  |  |  |  |  |  |
| -0.6102 | 22 | igd | 0.0563 | A | \|\| id:ebi-a-GCST90001702 | ebi-a-GCST90001702 | TRUE | G | 42870441 | 8.16E-30 | reported | 3657 | 0.05333 | rs187749054 |  |  |  |  |  |  |  |  |  |  |  |  |  |  |  |
| -0.3792 | 22 | igd | 0.0563 | A | \|\| id:ebi-a-GCST90001702 | ebi-a-GCST90001702 | TRUE | G | 44515711 | 5.71E-12 | reported | 3657 | 0.05489 | rs742550 |  |  |  |  |  |  |  |  |  |  |  |  |  |  |  |
| -0.6056 | 22 | igd | 0.0563 | A | \|\| id:ebi-a-GCST90001703 | ebi-a-GCST90001703 | TRUE | G | 42870441 | 2.20E-29 | reported | 3657 | 0.05334 | rs187749054 |  |  |  |  |  |  |  |  |  |  |  |  |  |  |  |
| -0.3842 | 22 | igd | 0.0563 | A | \|\| id:ebi-a-GCST90001703 | ebi-a-GCST90001703 | TRUE | G | 44515711 | 3.01E-12 | reported | 3657 | 0.05487 | rs742550 |  |  |  |  |  |  |  |  |  |  |  |  |  |  |  |
| -0.3771 | 22 | igd | 0.0563 | A | \|\| id:ebi-a-GCST90001704 | ebi-a-GCST90001704 | TRUE | G | 44515711 | 7.01E-12 | reported | 3657 | 0.05481 | rs742550 |  |  |  |  |  |  |  |  |  |  |  |  |  |  |  |
| -0.6166 | 22 | igd | 0.0563 | A | \|\| id:ebi-a-GCST90001704 | ebi-a-GCST90001704 | TRUE | G | 42870441 | 1.72E-30 | reported | 3657 | 0.05324 | rs187749054 |  |  |  |  |  |  |  |  |  |  |  |  |  |  |  |
| -0.5547 | 22 | igd | 0.0563 | A | \|\| id:ebi-a-GCST90001710 | ebi-a-GCST90001710 | TRUE | G | 42870441 | 5.41E-25 | reported | 3657 | 0.05333 | rs187749054 |  |  |  |  |  |  |  |  |  |  |  |  |  |  |  |
| -0.3706 | 22 | igd | 0.0563 | A | \|\| id:ebi-a-GCST90001710 | ebi-a-GCST90001710 | TRUE | G | 44515711 | 1.49E-11 | reported | 3657 | 0.05474 | rs742550 |  |  |  |  |  |  |  |  |  |  |  |  |  |  |  |
| -0.5522 | 22 | igd | 0.0563 | A | \|\| id:ebi-a-GCST90001711 | ebi-a-GCST90001711 | TRUE | G | 42870441 | 7.15E-25 | reported | 3657 | 0.05323 | rs187749054 |  |  |  |  |  |  |  |  |  |  |  |  |  |  |  |
| -0.3855 | 22 | igd | 0.0563 | A | \|\| id:ebi-a-GCST90001711 | ebi-a-GCST90001711 | TRUE | G | 44515711 | 1.96E-12 | reported | 3657 | 0.05459 | rs742550 |  |  |  |  |  |  |  |  |  |  |  |  |  |  |  |
| -0.3526 | 3 | igd | 0.0562 | C | \|\| id:ebi-a-GCST90001399 | ebi-a-GCST90001399 | TRUE | T | 32533246 | 5.92E-11 | reported | 3657 | 0.05371 | rs35574803 |  |  |  |  |  |  |  |  |  |  |  |  |  |  |  |
| -0.3044 | 3 | igd | 0.0562 | C | \|\| id:ebi-a-GCST90001433 | ebi-a-GCST90001433 | TRUE | T | 32533246 | 1.83E-08 | reported | 3656 | 0.05397 | rs35574803 |  |  |  |  |  |  |  |  |  |  |  |  |  |  |  |
| 0.7589 | 3 | igd | 0.0562 | C | \|\| id:ebi-a-GCST90001798 | ebi-a-GCST90001798 | TRUE | T | 32533246 | 3.63E-46 | reported | 3657 | 0.05246 | rs35574803 |  |  |  |  |  |  |  |  |  |  |  |  |  |  |  |
| 0.6071 | 3 | igd | 0.0562 | C | \|\| id:ebi-a-GCST90001802 | ebi-a-GCST90001802 | TRUE | T | 32533246 | 2.74E-29 | reported | 3657 | 0.05356 | rs35574803 |  |  |  |  |  |  |  |  |  |  |  |  |  |  |  |
| 0.6579 | 3 | igd | 0.0562 | C | \|\| id:ebi-a-GCST90001804 | ebi-a-GCST90001804 | TRUE | T | 32533246 | 3.42E-35 | reported | 3657 | 0.05259 | rs35574803 |  |  |  |  |  |  |  |  |  |  |  |  |  |  |  |
| 0.6708 | 3 | igd | 0.0562 | C | \|\| id:ebi-a-GCST90001805 | ebi-a-GCST90001805 | TRUE | T | 32533246 | 1.49E-35 | reported | 3657 | 0.05333 | rs35574803 |  |  |  |  |  |  |  |  |  |  |  |  |  |  |  |
| 0.6995 | 3 | igd | 0.0562 | C | \|\| id:ebi-a-GCST90001808 | ebi-a-GCST90001808 | TRUE | T | 32533246 | 1.02E-38 | reported | 3657 | 0.05313 | rs35574803 |  |  |  |  |  |  |  |  |  |  |  |  |  |  |  |
| 0.4979 | 10 | igd | 0.0562 | T | \|\| id:ebi-a-GCST90002031 | ebi-a-GCST90002031 | TRUE | C | 97496602 | 1.37E-16 | reported | 2920 | 0.05986 | rs79435780 |  |  |  |  |  |  |  |  |  |  |  |  |  |  |  |
| 0.3464 | 22 | igd | 0.0561 | C | \|\| id:ebi-a-GCST90001702 | ebi-a-GCST90001702 | TRUE | G | 41998660 | 5.72E-10 | reported | 3657 | 0.05573 | rs117430146 |  |  |  |  |  |  |  |  |  |  |  |  |  |  |  |
| 0.3453 | 22 | igd | 0.0561 | C | \|\| id:ebi-a-GCST90001703 | ebi-a-GCST90001703 | TRUE | G | 41998660 | 6.40E-10 | reported | 3657 | 0.05573 | rs117430146 |  |  |  |  |  |  |  |  |  |  |  |  |  |  |  |
| 0.3164 | 22 | igd | 0.0561 | C | \|\| id:ebi-a-GCST90001704 | ebi-a-GCST90001704 | TRUE | G | 41998660 | 1.45E-08 | reported | 3657 | 0.0557 | rs117430146 |  |  |  |  |  |  |  |  |  |  |  |  |  |  |  |
| 0.7463 | 3 | igd | 0.0561 | C | \|\| id:ebi-a-GCST90001800 | ebi-a-GCST90001800 | TRUE | T | 32533246 | 2.35E-43 | reported | 3656 | 0.05335 | rs35574803 |  |  |  |  |  |  |  |  |  |  |  |  |  |  |  |
| 0.7465 | 3 | igd | 0.0561 | C | \|\| id:ebi-a-GCST90001806 | ebi-a-GCST90001806 | TRUE | T | 32533246 | 8.02E-44 | reported | 3656 | 0.05306 | rs35574803 |  |  |  |  |  |  |  |  |  |  |  |  |  |  |  |
| 0.4277 | 10 | igd | 0.056 | T | \|\| id:ebi-a-GCST90001484 | ebi-a-GCST90001484 | TRUE | G | 97578750 | 3.88E-15 | reported | 3437 | 0.05417 | rs75284071 |  |  |  |  |  |  |  |  |  |  |  |  |  |  |  |
| 0.4023 | 10 | igd | 0.056 | C | \|\| id:ebi-a-GCST90001485 | ebi-a-GCST90001485 | TRUE | G | 97550580 | 1.31E-13 | reported | 3437 | 0.05411 | rs76464189 |  |  |  |  |  |  |  |  |  |  |  |  |  |  |  |
| 0.4652 | 10 | igd | 0.056 | T | \|\| id:ebi-a-GCST90001491 | ebi-a-GCST90001491 | TRUE | G | 97578750 | 5.55E-17 | reported | 3437 | 0.05526 | rs75284071 |  |  |  |  |  |  |  |  |  |  |  |  |  |  |  |
| 0.4637 | 10 | igd | 0.056 | A | \|\| id:ebi-a-GCST90001491 | ebi-a-GCST90001491 | TRUE | G | 97473779 | 6.56E-17 | reported | 3437 | 0.05522 | rs75696337 |  |  |  |  |  |  |  |  |  |  |  |  |  |  |  |
| 0.4504 | 10 | igd | 0.056 | T | \|\| id:ebi-a-GCST90001658 | ebi-a-GCST90001658 | TRUE | G | 97578750 | 4.78E-17 | reported | 3440 | 0.0534 | rs75284071 |  |  |  |  |  |  |  |  |  |  |  |  |  |  |  |
| 0.4507 | 10 | igd | 0.056 | A | \|\| id:ebi-a-GCST90001658 | ebi-a-GCST90001658 | TRUE | G | 97473779 | 4.31E-17 | reported | 3440 | 0.05335 | rs75696337 |  |  |  |  |  |  |  |  |  |  |  |  |  |  |  |
| 0.3898 | 10 | igd | 0.056 | T | \|\| id:ebi-a-GCST90001670 | ebi-a-GCST90001670 | TRUE | A | 97283689 | 3.85E-12 | reported | 3440 | 0.05595 | rs72824582 |  |  |  |  |  |  |  |  |  |  |  |  |  |  |  |
| -0.369 | 10 | igd | 0.056 | T | \|\| id:ebi-a-GCST90001961 | ebi-a-GCST90001961 | TRUE | G | 97283682 | 1.10E-09 | reported | 2920 | 0.06034 | rs72824580 |  |  |  |  |  |  |  |  |  |  |  |  |  |  |  |
| 0.6103 | 10 | igd | 0.056 | A | \|\| id:ebi-a-GCST90002030 | ebi-a-GCST90002030 | TRUE | C | 97511231 | 4.18E-25 | reported | 2920 | 0.05842 | rs138474301 |  |  |  |  |  |  |  |  |  |  |  |  |  |  |  |
| 0.5598 | 10 | igd | 0.056 | A | \|\| id:ebi-a-GCST90002032 | ebi-a-GCST90002032 | TRUE | C | 97511231 | 1.54E-20 | reported | 2920 | 0.05981 | rs138474301 |  |  |  |  |  |  |  |  |  |  |  |  |  |  |  |
| 0.4727 | 10 | igd | 0.0559 | A | \|\| id:ebi-a-GCST90001496 | ebi-a-GCST90001496 | TRUE | C | 97511231 | 5.05E-17 | reported | 3437 | 0.05608 | rs138474301 |  |  |  |  |  |  |  |  |  |  |  |  |  |  |  |
| 0.4633 | 10 | igd | 0.0559 | A | \|\| id:ebi-a-GCST90001497 | ebi-a-GCST90001497 | TRUE | C | 97511231 | 8.63E-17 | reported | 3437 | 0.05538 | rs138474301 |  |  |  |  |  |  |  |  |  |  |  |  |  |  |  |
| 0.3556 | 22 | igd | 0.0559 | C | \|\| id:ebi-a-GCST90001702 | ebi-a-GCST90001702 | TRUE | G | 41569496 | 1.28E-10 | reported | 3657 | 0.05514 | rs138878812 |  |  |  |  |  |  |  |  |  |  |  |  |  |  |  |
| 0.3402 | 22 | igd | 0.0559 | C | \|\| id:ebi-a-GCST90001703 | ebi-a-GCST90001703 | TRUE | A | 41570278 | 7.66E-10 | reported | 3657 | 0.05516 | rs144214275 |  |  |  |  |  |  |  |  |  |  |  |  |  |  |  |
| 0.3148 | 22 | igd | 0.0559 | C | \|\| id:ebi-a-GCST90001704 | ebi-a-GCST90001704 | TRUE | A | 41570278 | 1.22E-08 | reported | 3657 | 0.05513 | rs144214275 |  |  |  |  |  |  |  |  |  |  |  |  |  |  |  |
| 0.3372 | 22 | igd | 0.0559 | C | \|\| id:ebi-a-GCST90001710 | ebi-a-GCST90001710 | TRUE | A | 41570278 | 9.85E-10 | reported | 3657 | 0.05502 | rs144214275 |  |  |  |  |  |  |  |  |  |  |  |  |  |  |  |
| 0.3021 | 22 | igd | 0.0559 | C | \|\| id:ebi-a-GCST90001711 | ebi-a-GCST90001711 | TRUE | A | 41570278 | 4.11E-08 | reported | 3657 | 0.05495 | rs144214275 |  |  |  |  |  |  |  |  |  |  |  |  |  |  |  |
| -0.4459 | 6 | igd | 0.0559 | T | \|\| id:ebi-a-GCST90001774 | ebi-a-GCST90001774 | TRUE | C | 107159822 | 1.32E-16 | reported | 3656 | 0.05365 | rs12206866 |  |  |  |  |  |  |  |  |  |  |  |  |  |  |  |
| 0.42 | 10 | igd | 0.0558 | A | \|\| id:ebi-a-GCST90001659 | ebi-a-GCST90001659 | TRUE | C | 97511231 | 5.84E-15 | reported | 3440 | 0.05355 | rs138474301 |  |  |  |  |  |  |  |  |  |  |  |  |  |  |  |
| 0.3615 | 10 | igd | 0.0558 | A | \|\| id:ebi-a-GCST90001670 | ebi-a-GCST90001670 | TRUE | C | 97511231 | 1.09E-10 | reported | 3440 | 0.05584 | rs138474301 |  |  |  |  |  |  |  |  |  |  |  |  |  |  |  |
| 0.3821 | 10 | igd | 0.0558 | A | \|\| id:ebi-a-GCST90001671 | ebi-a-GCST90001671 | TRUE | C | 97511231 | 9.85E-12 | reported | 3440 | 0.05593 | rs138474301 |  |  |  |  |  |  |  |  |  |  |  |  |  |  |  |
| 0.4208 | 10 | igd | 0.0558 | T | \|\| id:ebi-a-GCST90001672 | ebi-a-GCST90001672 | TRUE | A | 97283689 | 1.79E-13 | reported | 3408 | 0.05691 | rs72824582 |  |  |  |  |  |  |  |  |  |  |  |  |  |  |  |
| -0.3089 | 1 | igd | 0.0558 | A | \|\| id:ebi-a-GCST90001854 | ebi-a-GCST90001854 | TRUE | G | 167431225 | 3.63E-08 | reported | 2919 | 0.05593 | rs12124119 |  |  |  |  |  |  |  |  |  |  |  |  |  |  |  |
| -0.3501 | 1 | igd | 0.0558 | A | \|\| id:ebi-a-GCST90001860 | ebi-a-GCST90001860 | TRUE | G | 167431225 | 6.35E-10 | reported | 2919 | 0.05645 | rs12124119 |  |  |  |  |  |  |  |  |  |  |  |  |  |  |  |
| 0.3679 | 10 | igd | 0.0557 | T | \|\| id:ebi-a-GCST90001483 | ebi-a-GCST90001483 | TRUE | G | 97578750 | 2.38E-11 | reported | 3405 | 0.05489 | rs75284071 |  |  |  |  |  |  |  |  |  |  |  |  |  |  |  |
| 0.4275 | 10 | igd | 0.0557 | T | \|\| id:ebi-a-GCST90001489 | ebi-a-GCST90001489 | TRUE | C | 97496602 | 3.70E-14 | reported | 3405 | 0.05623 | rs79435780 |  |  |  |  |  |  |  |  |  |  |  |  |  |  |  |
| 0.4309 | 10 | igd | 0.0557 | A | \|\| id:ebi-a-GCST90001495 | ebi-a-GCST90001495 | TRUE | G | 97473779 | 4.25E-14 | reported | 3405 | 0.05681 | rs75696337 |  |  |  |  |  |  |  |  |  |  |  |  |  |  |  |
| 0.4317 | 10 | igd | 0.0557 | T | \|\| id:ebi-a-GCST90001495 | ebi-a-GCST90001495 | TRUE | G | 97578750 | 4.00E-14 | reported | 3405 | 0.05686 | rs75284071 |  |  |  |  |  |  |  |  |  |  |  |  |  |  |  |
| -0.4091 | 1 | igd | 0.0557 | A | \|\| id:ebi-a-GCST90001845 | ebi-a-GCST90001845 | TRUE | G | 167431225 | 4.12E-13 | reported | 2911 | 0.05616 | rs12124119 |  |  |  |  |  |  |  |  |  |  |  |  |  |  |  |
| -0.3182 | 2 | igd | 0.0557 | T | \|\| id:ebi-a-GCST90002098 | ebi-a-GCST90002098 | TRUE | A | 39114128 | 2.60E-08 | reported | 2910 | 0.05702 | rs76073543 |  |  |  |  |  |  |  |  |  |  |  |  |  |  |  |
| 0.4142 | 10 | igd | 0.0556 | A | \|\| id:ebi-a-GCST90001660 | ebi-a-GCST90001660 | TRUE | G | 97473779 | 2.14E-13 | reported | 3408 | 0.05621 | rs75696337 |  |  |  |  |  |  |  |  |  |  |  |  |  |  |  |
| 0.415 | 10 | igd | 0.0556 | T | \|\| id:ebi-a-GCST90001660 | ebi-a-GCST90001660 | TRUE | G | 97578750 | 2.02E-13 | reported | 3408 | 0.05625 | rs75284071 |  |  |  |  |  |  |  |  |  |  |  |  |  |  |  |
| 0.3458 | 10 | igd | 0.0556 | A | \|\| id:ebi-a-GCST90001672 | ebi-a-GCST90001672 | TRUE | G | 97473779 | 1.36E-09 | reported | 3408 | 0.0569 | rs75696337 |  |  |  |  |  |  |  |  |  |  |  |  |  |  |  |
| 0.3467 | 10 | igd | 0.0556 | T | \|\| id:ebi-a-GCST90001672 | ebi-a-GCST90001672 | TRUE | G | 97578750 | 1.27E-09 | reported | 3408 | 0.05695 | rs75284071 |  |  |  |  |  |  |  |  |  |  |  |  |  |  |  |
| -0.4386 | 6 | igd | 0.0555 | G | \|\| id:ebi-a-GCST90001768 | ebi-a-GCST90001768 | TRUE | A | 107155687 | 1.28E-15 | reported | 3655 | 0.0546 | rs9398107 |  |  |  |  |  |  |  |  |  |  |  |  |  |  |  |
| 0.4241 | 2 | igd | 0.0555 | A | \|\| id:ebi-a-GCST90002098 | ebi-a-GCST90002098 | TRUE | G | 39384195 | 6.40E-14 | reported | 2910 | 0.05627 | rs116499452 |  |  |  |  |  |  |  |  |  |  |  |  |  |  |  |
| -0.3585 | 10 | igd | 0.0551 | A | \|\| id:ebi-a-GCST90001484 | ebi-a-GCST90001484 | TRUE | G | 97678177 | 4.60E-11 | reported | 3437 | 0.05429 | rs71482389 |  |  |  |  |  |  |  |  |  |  |  |  |  |  |  |
| -0.621 | 19 | igd | 0.0551 | T | \|\| id:ebi-a-GCST90001950 | ebi-a-GCST90001950 | TRUE | G | 51685133 | 1.17E-11 | reported | 1244 | 0.09068 | rs116960573 |  |  |  |  |  |  |  |  |  |  |  |  |  |  |  |
| -0.323 | 3 | igd | 0.055 | G | \|\| id:ebi-a-GCST90002008 | ebi-a-GCST90002008 | TRUE | A | 46338831 | 3.74E-09 | reported | 3629 | 0.05465 | rs35117954 |  |  |  |  |  |  |  |  |  |  |  |  |  |  |  |
| 0.4454 | 10 | igd | 0.0548 | A | \|\| id:ebi-a-GCST90001489 | ebi-a-GCST90001489 | TRUE | T | 97717995 | 3.76E-15 | reported | 3405 | 0.05639 | rs7917360 |  |  |  |  |  |  |  |  |  |  |  |  |  |  |  |
| -0.6177 | 19 | igd | 0.0548 | A | \|\| id:ebi-a-GCST90001946 | ebi-a-GCST90001946 | TRUE | C | 51542085 | 1.43E-14 | reported | 1634 | 0.07955 | rs62117667 |  |  |  |  |  |  |  |  |  |  |  |  |  |  |  |
| -0.6021 | 19 | igd | 0.0548 | A | \|\| id:ebi-a-GCST90001948 | ebi-a-GCST90001948 | TRUE | C | 51542085 | 4.26E-14 | reported | 1633 | 0.079 | rs62117667 |  |  |  |  |  |  |  |  |  |  |  |  |  |  |  |
| -0.5324 | 19 | igd | 0.0548 | A | \|\| id:ebi-a-GCST90001949 | ebi-a-GCST90001949 | TRUE | C | 51542085 | 2.46E-11 | reported | 1634 | 0.0792 | rs62117667 |  |  |  |  |  |  |  |  |  |  |  |  |  |  |  |
| -0.3427 | 10 | igd | 0.0548 | T | \|\| id:ebi-a-GCST90002030 | ebi-a-GCST90002030 | TRUE | C | 97168827 | 9.90E-09 | reported | 2920 | 0.05961 | rs11596570 |  |  |  |  |  |  |  |  |  |  |  |  |  |  |  |
| -0.3565 | 22 | igd | 0.0546 | T | \|\| id:ebi-a-GCST90001701 | ebi-a-GCST90001701 | TRUE | C | 42214719 | 7.11E-11 | reported | 3592 | 0.05453 | rs7292894 |  |  |  |  |  |  |  |  |  |  |  |  |  |  |  |
| -0.537 | 22 | igd | 0.0546 | G | \|\| id:ebi-a-GCST90001702 | ebi-a-GCST90001702 | TRUE | A | 40987104 | 2.76E-21 | reported | 3657 | 0.05635 | rs148646251 |  |  |  |  |  |  |  |  |  |  |  |  |  |  |  |
| -0.5273 | 22 | igd | 0.0546 | G | \|\| id:ebi-a-GCST90001703 | ebi-a-GCST90001703 | TRUE | A | 40987104 | 1.42E-20 | reported | 3657 | 0.05636 | rs148646251 |  |  |  |  |  |  |  |  |  |  |  |  |  |  |  |
| -0.5549 | 22 | igd | 0.0546 | G | \|\| id:ebi-a-GCST90001704 | ebi-a-GCST90001704 | TRUE | A | 40987104 | 1.06E-22 | reported | 3657 | 0.05621 | rs148646251 |  |  |  |  |  |  |  |  |  |  |  |  |  |  |  |
| -0.5604 | 22 | igd | 0.0546 | G | \|\| id:ebi-a-GCST90001710 | ebi-a-GCST90001710 | TRUE | A | 40987104 | 3.59E-23 | reported | 3657 | 0.05613 | rs148646251 |  |  |  |  |  |  |  |  |  |  |  |  |  |  |  |
| -0.5529 | 22 | igd | 0.0546 | G | \|\| id:ebi-a-GCST90001711 | ebi-a-GCST90001711 | TRUE | A | 40987104 | 1.04E-22 | reported | 3657 | 0.056 | rs148646251 |  |  |  |  |  |  |  |  |  |  |  |  |  |  |  |
| -0.3725 | 2 | igd | 0.0546 | T | \|\| id:ebi-a-GCST90002119 | ebi-a-GCST90002119 | TRUE | G | 86692529 | 4.01E-10 | reported | 2920 | 0.05937 | rs116009970 |  |  |  |  |  |  |  |  |  |  |  |  |  |  |  |
| -0.5971 | 2 | igd | 0.0546 | G | \|\| id:ebi-a-GCST90002119 | ebi-a-GCST90002119 | TRUE | C | 87078461 | 4.78E-25 | reported | 2920 | 0.05723 | rs62146884 |  |  |  |  |  |  |  |  |  |  |  |  |  |  |  |
| 0.3755 | 1 | igd | 0.0545 | T | \|\| id:ebi-a-GCST90001858 | ebi-a-GCST90001858 | TRUE | C | 167474825 | 1.72E-10 | reported | 2919 | 0.0586 | rs36108451 |  |  |  |  |  |  |  |  |  |  |  |  |  |  |  |
| -0.3799 | 10 | igd | 0.0545 | T | \|\| id:ebi-a-GCST90002030 | ebi-a-GCST90002030 | TRUE | C | 97064677 | 7.75E-10 | reported | 2920 | 0.06157 | rs79025152 |  |  |  |  |  |  |  |  |  |  |  |  |  |  |  |
| 0.5204 | 6 | igd | 0.0545 | A | \|\| id:ebi-a-GCST90002105 | ebi-a-GCST90002105 | TRUE | G | 30943138 | 1.03E-13 | reported | 2872 | 0.06963 | rs143764990 |  |  |  |  |  |  |  |  |  |  |  |  |  |  |  |
| -0.5272 | 6 | igd | 0.0544 | T | \|\| id:ebi-a-GCST90001743 | ebi-a-GCST90001743 | TRUE | C | 32623828 | 9.44E-09 | reported | 3660 | 0.09163 | rs147717587 |  |  |  |  |  |  |  |  |  |  |  |  |  |  |  |
| -0.6009 | 6 | igd | 0.0544 | T | \|\| id:ebi-a-GCST90001752 | ebi-a-GCST90001752 | TRUE | C | 32623828 | 6.14E-11 | reported | 3657 | 0.0916 | rs147717587 |  |  |  |  |  |  |  |  |  |  |  |  |  |  |  |
| -0.505 | 6 | igd | 0.0544 | T | \|\| id:ebi-a-GCST90001762 | ebi-a-GCST90001762 | TRUE | C | 32623828 | 3.96E-08 | reported | 3658 | 0.09175 | rs147717587 |  |  |  |  |  |  |  |  |  |  |  |  |  |  |  |
| 0.6113 | 10 | igd | 0.0544 | T | \|\| id:ebi-a-GCST90001933 | ebi-a-GCST90001933 | TRUE | C | 6094697 | 6.70E-28 | reported | 3435 | 0.05534 | rs61839660 |  |  |  |  |  |  |  |  |  |  |  |  |  |  |  |
| 0.464 | 6 | igd | 0.0544 | C | \|\| id:ebi-a-GCST90002111 | ebi-a-GCST90002111 | TRUE | T | 32668831 | 1.47E-08 | reported | 1635 | 0.08148 | rs9275382 |  |  |  |  |  |  |  |  |  |  |  |  |  |  |  |
| 0.9962 | 6 | igd | 0.0544 | T | \|\| id:ebi-a-GCST90002112 | ebi-a-GCST90002112 | TRUE | G | 32668846 | 7.23E-35 | reported | 1635 | 0.07899 | rs9275383 |  |  |  |  |  |  |  |  |  |  |  |  |  |  |  |
| 0.592 | 10 | igd | 0.0543 | T | \|\| id:ebi-a-GCST90001505 | ebi-a-GCST90001505 | TRUE | C | 6094697 | 1.64E-26 | reported | 3437 | 0.05509 | rs61839660 |  |  |  |  |  |  |  |  |  |  |  |  |  |  |  |
| 0.5156 | 10 | igd | 0.0543 | T | \|\| id:ebi-a-GCST90001506 | ebi-a-GCST90001506 | TRUE | C | 6094697 | 3.21E-20 | reported | 3437 | 0.05562 | rs61839660 |  |  |  |  |  |  |  |  |  |  |  |  |  |  |  |
| 0.6146 | 10 | igd | 0.0543 | T | \|\| id:ebi-a-GCST90001511 | ebi-a-GCST90001511 | TRUE | C | 6094697 | 2.78E-28 | reported | 3437 | 0.05523 | rs61839660 |  |  |  |  |  |  |  |  |  |  |  |  |  |  |  |
| 0.5758 | 10 | igd | 0.0543 | T | \|\| id:ebi-a-GCST90001512 | ebi-a-GCST90001512 | TRUE | C | 6094697 | 5.32E-25 | reported | 3437 | 0.05532 | rs61839660 |  |  |  |  |  |  |  |  |  |  |  |  |  |  |  |
| -0.3391 | 10 | igd | 0.0543 | T | \|\| id:ebi-a-GCST90001672 | ebi-a-GCST90001672 | TRUE | C | 97064677 | 7.74E-09 | reported | 3408 | 0.05859 | rs79025152 |  |  |  |  |  |  |  |  |  |  |  |  |  |  |  |
| -0.863 | 22 | igd | 0.0543 | T | \|\| id:ebi-a-GCST90001702 | ebi-a-GCST90001702 | TRUE | C | 42214719 | 4.82E-56 | reported | 3657 | 0.05378 | rs7292894 |  |  |  |  |  |  |  |  |  |  |  |  |  |  |  |
| -0.8544 | 22 | igd | 0.0543 | T | \|\| id:ebi-a-GCST90001703 | ebi-a-GCST90001703 | TRUE | C | 42214719 | 6.24E-55 | reported | 3657 | 0.05382 | rs7292894 |  |  |  |  |  |  |  |  |  |  |  |  |  |  |  |
| -0.8395 | 22 | igd | 0.0543 | T | \|\| id:ebi-a-GCST90001704 | ebi-a-GCST90001704 | TRUE | C | 42214719 | 3.57E-53 | reported | 3657 | 0.05381 | rs7292894 |  |  |  |  |  |  |  |  |  |  |  |  |  |  |  |
| -0.8348 | 22 | igd | 0.0543 | T | \|\| id:ebi-a-GCST90001710 | ebi-a-GCST90001710 | TRUE | C | 42214719 | 9.52E-53 | reported | 3657 | 0.05374 | rs7292894 |  |  |  |  |  |  |  |  |  |  |  |  |  |  |  |
| -0.8041 | 22 | igd | 0.0543 | T | \|\| id:ebi-a-GCST90001711 | ebi-a-GCST90001711 | TRUE | C | 42214719 | 3.70E-49 | reported | 3657 | 0.05375 | rs7292894 |  |  |  |  |  |  |  |  |  |  |  |  |  |  |  |
| -0.4694 | 10 | igd | 0.0543 | A | \|\| id:ebi-a-GCST90002030 | ebi-a-GCST90002030 | TRUE | G | 95569379 | 3.07E-14 | reported | 2920 | 0.06149 | rs78573011 |  |  |  |  |  |  |  |  |  |  |  |  |  |  |  |
| -0.415 | 10 | igd | 0.0543 | A | \|\| id:ebi-a-GCST90002031 | ebi-a-GCST90002031 | TRUE | G | 95569379 | 6.65E-11 | reported | 2920 | 0.06334 | rs78573011 |  |  |  |  |  |  |  |  |  |  |  |  |  |  |  |
| -0.4141 | 10 | igd | 0.0543 | A | \|\| id:ebi-a-GCST90002032 | ebi-a-GCST90002032 | TRUE | G | 95569379 | 7.00E-11 | reported | 2920 | 0.06327 | rs78573011 |  |  |  |  |  |  |  |  |  |  |  |  |  |  |  |
| -0.3251 | 3 | igd | 0.0543 | C | \|\| id:ebi-a-GCST90002004 | ebi-a-GCST90002004 | TRUE | T | 46288011 | 6.18E-09 | reported | 3629 | 0.05581 | rs13080757 |  |  |  |  |  |  |  |  |  |  |  |  |  |  |  |
| -0.3141 | 10 | igd | 0.0542 | T | \|\| id:ebi-a-GCST90001489 | ebi-a-GCST90001489 | TRUE | C | 97168827 | 3.60E-08 | reported | 3405 | 0.05688 | rs11596570 |  |  |  |  |  |  |  |  |  |  |  |  |  |  |  |
| 0.4625 | 10 | igd | 0.0542 | T | \|\| id:ebi-a-GCST90001504 | ebi-a-GCST90001504 | TRUE | C | 6094697 | 4.05E-16 | reported | 3405 | 0.05656 | rs61839660 |  |  |  |  |  |  |  |  |  |  |  |  |  |  |  |
| 0.5547 | 10 | igd | 0.0542 | T | \|\| id:ebi-a-GCST90001510 | ebi-a-GCST90001510 | TRUE | C | 6094697 | 2.40E-22 | reported | 3405 | 0.05665 | rs61839660 |  |  |  |  |  |  |  |  |  |  |  |  |  |  |  |
| -0.3117 | 10 | igd | 0.0541 | T | \|\| id:ebi-a-GCST90001491 | ebi-a-GCST90001491 | TRUE | C | 97168827 | 2.57E-08 | reported | 3437 | 0.05584 | rs11596570 |  |  |  |  |  |  |  |  |  |  |  |  |  |  |  |
| -0.3241 | 10 | igd | 0.0541 | T | \|\| id:ebi-a-GCST90001491 | ebi-a-GCST90001491 | TRUE | C | 97064677 | 1.71E-08 | reported | 3437 | 0.05734 | rs79025152 |  |  |  |  |  |  |  |  |  |  |  |  |  |  |  |
| -0.3489 | 10 | igd | 0.0541 | T | \|\| id:ebi-a-GCST90001496 | ebi-a-GCST90001496 | TRUE | C | 97064677 | 2.08E-09 | reported | 3437 | 0.05807 | rs79025152 |  |  |  |  |  |  |  |  |  |  |  |  |  |  |  |
| -0.3469 | 10 | igd | 0.0541 | T | \|\| id:ebi-a-GCST90001497 | ebi-a-GCST90001497 | TRUE | C | 97064677 | 1.60E-09 | reported | 3437 | 0.05734 | rs79025152 |  |  |  |  |  |  |  |  |  |  |  |  |  |  |  |
| -0.3203 | 10 | igd | 0.0541 | T | \|\| id:ebi-a-GCST90001671 | ebi-a-GCST90001671 | TRUE | C | 97064677 | 3.19E-08 | reported | 3440 | 0.05778 | rs79025152 |  |  |  |  |  |  |  |  |  |  |  |  |  |  |  |
| 0.3212 | 10 | igd | 0.0541 | T | \|\| id:ebi-a-GCST90001679 | ebi-a-GCST90001679 | TRUE | C | 6094697 | 3.41E-08 | reported | 3162 | 0.05805 | rs61839660 |  |  |  |  |  |  |  |  |  |  |  |  |  |  |  |
| -0.5002 | 22 | igd | 0.0539 | T | \|\| id:ebi-a-GCST90001702 | ebi-a-GCST90001702 | TRUE | A | 40377469 | 2.95E-19 | reported | 3657 | 0.05545 | rs139195105 |  |  |  |  |  |  |  |  |  |  |  |  |  |  |  |
| -0.5083 | 22 | igd | 0.0539 | T | \|\| id:ebi-a-GCST90001703 | ebi-a-GCST90001703 | TRUE | A | 40377469 | 7.74E-20 | reported | 3657 | 0.05543 | rs139195105 |  |  |  |  |  |  |  |  |  |  |  |  |  |  |  |
| -0.5521 | 22 | igd | 0.0539 | T | \|\| id:ebi-a-GCST90001704 | ebi-a-GCST90001704 | TRUE | A | 40377469 | 3.22E-23 | reported | 3657 | 0.05524 | rs139195105 |  |  |  |  |  |  |  |  |  |  |  |  |  |  |  |
| -0.4786 | 22 | igd | 0.0539 | T | \|\| id:ebi-a-GCST90001710 | ebi-a-GCST90001710 | TRUE | A | 40377469 | 7.72E-18 | reported | 3657 | 0.05535 | rs139195105 |  |  |  |  |  |  |  |  |  |  |  |  |  |  |  |
| -0.4962 | 22 | igd | 0.0539 | T | \|\| id:ebi-a-GCST90001711 | ebi-a-GCST90001711 | TRUE | A | 40377469 | 3.80E-19 | reported | 3657 | 0.05518 | rs139195105 |  |  |  |  |  |  |  |  |  |  |  |  |  |  |  |
| -0.4705 | 10 | igd | 0.0539 | T | \|\| id:ebi-a-GCST90002030 | ebi-a-GCST90002030 | TRUE | C | 96372939 | 6.55E-15 | reported | 2920 | 0.06006 | rs41291548 |  |  |  |  |  |  |  |  |  |  |  |  |  |  |  |
| 0.3795 | 6 | igd | 0.0538 | G | \|\| id:ebi-a-GCST90002105 | ebi-a-GCST90002105 | TRUE | C | 31834769 | 1.44E-08 | reported | 2872 | 0.06675 | rs76116290 |  |  |  |  |  |  |  |  |  |  |  |  |  |  |  |
| -1.386 | 22 | igd | 0.0536 | A | \|\| id:ebi-a-GCST90001702 | ebi-a-GCST90001702 | TRUE | G | 42144372 | 9.66E-148 | reported | 3657 | 0.05111 | rs17449595 |  |  |  |  |  |  |  |  |  |  |  |  |  |  |  |
| -1.386 | 22 | igd | 0.0536 | A | \|\| id:ebi-a-GCST90001703 | ebi-a-GCST90001703 | TRUE | G | 42144372 | 1.22E-147 | reported | 3657 | 0.05111 | rs17449595 |  |  |  |  |  |  |  |  |  |  |  |  |  |  |  |
| -1.396 | 22 | igd | 0.0536 | A | \|\| id:ebi-a-GCST90001704 | ebi-a-GCST90001704 | TRUE | G | 42144372 | 1.45E-150 | reported | 3657 | 0.05095 | rs17449595 |  |  |  |  |  |  |  |  |  |  |  |  |  |  |  |
| -1.325 | 22 | igd | 0.0536 | A | \|\| id:ebi-a-GCST90001710 | ebi-a-GCST90001710 | TRUE | G | 42144372 | 8.17E-135 | reported | 3657 | 0.05138 | rs17449595 |  |  |  |  |  |  |  |  |  |  |  |  |  |  |  |
| -1.351 | 22 | igd | 0.0536 | A | \|\| id:ebi-a-GCST90001711 | ebi-a-GCST90001711 | TRUE | G | 42144372 | 2.54E-141 | reported | 3657 | 0.05106 | rs17449595 |  |  |  |  |  |  |  |  |  |  |  |  |  |  |  |
| -0.4676 | 10 | igd | 0.0536 | T | \|\| id:ebi-a-GCST90002030 | ebi-a-GCST90002030 | TRUE | C | 96670061 | 1.30E-14 | reported | 2920 | 0.06037 | rs141856853 |  |  |  |  |  |  |  |  |  |  |  |  |  |  |  |
| 0.3779 | 2 | igd | 0.0536 | G | \|\| id:ebi-a-GCST90002118 | ebi-a-GCST90002118 | TRUE | A | 86972430 | 5.45E-10 | reported | 2920 | 0.0607 | rs11692611 |  |  |  |  |  |  |  |  |  |  |  |  |  |  |  |
| 0.3883 | 2 | igd | 0.0536 | G | \|\| id:ebi-a-GCST90002119 | ebi-a-GCST90002119 | TRUE | A | 86972430 | 8.39E-11 | reported | 2920 | 0.05958 | rs11692611 |  |  |  |  |  |  |  |  |  |  |  |  |  |  |  |
| 0.3351 | 2 | igd | 0.0536 | G | \|\| id:ebi-a-GCST90002120 | ebi-a-GCST90002120 | TRUE | A | 86972430 | 4.59E-08 | reported | 2920 | 0.06115 | rs11692611 |  |  |  |  |  |  |  |  |  |  |  |  |  |  |  |
| -0.5139 | 22 | igd | 0.0535 | T | \|\| id:ebi-a-GCST90001702 | ebi-a-GCST90001702 | TRUE | A | 41305795 | 2.39E-18 | reported | 3657 | 0.05851 | rs187315152 |  |  |  |  |  |  |  |  |  |  |  |  |  |  |  |
| 0.3362 | 22 | igd | 0.0535 | A | \|\| id:ebi-a-GCST90001702 | ebi-a-GCST90001702 | TRUE | G | 42162333 | 3.45E-09 | reported | 3657 | 0.05676 | rs139063190 |  |  |  |  |  |  |  |  |  |  |  |  |  |  |  |
| 0.3317 | 22 | igd | 0.0535 | A | \|\| id:ebi-a-GCST90001703 | ebi-a-GCST90001703 | TRUE | G | 42162333 | 5.58E-09 | reported | 3657 | 0.05676 | rs139063190 |  |  |  |  |  |  |  |  |  |  |  |  |  |  |  |
| -0.5025 | 22 | igd | 0.0535 | T | \|\| id:ebi-a-GCST90001703 | ebi-a-GCST90001703 | TRUE | A | 41305795 | 1.31E-17 | reported | 3657 | 0.05853 | rs187315152 |  |  |  |  |  |  |  |  |  |  |  |  |  |  |  |
| -0.5203 | 22 | igd | 0.0535 | T | \|\| id:ebi-a-GCST90001704 | ebi-a-GCST90001704 | TRUE | A | 41305796 | 8.00E-19 | reported | 3657 | 0.0584 | rs191405504 |  |  |  |  |  |  |  |  |  |  |  |  |  |  |  |
| -0.5316 | 22 | igd | 0.0535 | T | \|\| id:ebi-a-GCST90001710 | ebi-a-GCST90001710 | TRUE | A | 41305796 | 1.26E-19 | reported | 3657 | 0.05831 | rs191405504 |  |  |  |  |  |  |  |  |  |  |  |  |  |  |  |
| -0.5312 | 22 | igd | 0.0535 | T | \|\| id:ebi-a-GCST90001711 | ebi-a-GCST90001711 | TRUE | A | 41305796 | 1.07E-19 | reported | 3657 | 0.05816 | rs191405504 |  |  |  |  |  |  |  |  |  |  |  |  |  |  |  |
| 0.6371 | 6 | igd | 0.0535 | A | \|\| id:ebi-a-GCST90002108 | ebi-a-GCST90002108 | TRUE | G | 30943138 | 1.94E-13 | reported | 1579 | 0.08589 | rs143764990 |  |  |  |  |  |  |  |  |  |  |  |  |  |  |  |
| 0.3515 | 10 | igd | 0.0533 | A | \|\| id:ebi-a-GCST90001670 | ebi-a-GCST90001670 | TRUE | G | 93961353 | 9.61E-10 | reported | 3440 | 0.05732 | rs117962493 |  |  |  |  |  |  |  |  |  |  |  |  |  |  |  |
| -0.5182 | 22 | igd | 0.0533 | A | \|\| id:ebi-a-GCST90001701 | ebi-a-GCST90001701 | TRUE | G | 42144372 | 2.75E-21 | reported | 3592 | 0.05437 | rs17449595 |  |  |  |  |  |  |  |  |  |  |  |  |  |  |  |
| -0.5067 | 22 | igd | 0.0533 | G | \|\| id:ebi-a-GCST90001702 | ebi-a-GCST90001702 | TRUE | T | 40486993 | 8.81E-20 | reported | 3657 | 0.05535 | rs190339574 |  |  |  |  |  |  |  |  |  |  |  |  |  |  |  |
| -0.5175 | 22 | igd | 0.0533 | G | \|\| id:ebi-a-GCST90001703 | ebi-a-GCST90001703 | TRUE | T | 40486993 | 1.42E-20 | reported | 3657 | 0.05532 | rs190339574 |  |  |  |  |  |  |  |  |  |  |  |  |  |  |  |
| -0.5586 | 22 | igd | 0.0533 | G | \|\| id:ebi-a-GCST90001704 | ebi-a-GCST90001704 | TRUE | T | 40486993 | 8.17E-24 | reported | 3657 | 0.05513 | rs190339574 |  |  |  |  |  |  |  |  |  |  |  |  |  |  |  |
| -0.4876 | 22 | igd | 0.0533 | G | \|\| id:ebi-a-GCST90001710 | ebi-a-GCST90001710 | TRUE | T | 40486993 | 1.62E-18 | reported | 3657 | 0.05523 | rs190339574 |  |  |  |  |  |  |  |  |  |  |  |  |  |  |  |
| -0.5056 | 22 | igd | 0.0533 | G | \|\| id:ebi-a-GCST90001711 | ebi-a-GCST90001711 | TRUE | T | 40486993 | 6.97E-20 | reported | 3657 | 0.05507 | rs190339574 |  |  |  |  |  |  |  |  |  |  |  |  |  |  |  |
| 0.3369 | 22 | igd | 0.0532 | T | \|\| id:ebi-a-GCST90001702 | ebi-a-GCST90001702 | TRUE | C | 41814199 | 3.15E-09 | reported | 3657 | 0.05674 | rs79090302 |  |  |  |  |  |  |  |  |  |  |  |  |  |  |  |
| 0.3312 | 22 | igd | 0.0532 | T | \|\| id:ebi-a-GCST90001703 | ebi-a-GCST90001703 | TRUE | C | 41814199 | 5.79E-09 | reported | 3657 | 0.05674 | rs79090302 |  |  |  |  |  |  |  |  |  |  |  |  |  |  |  |
| 0.312 | 22 | igd | 0.0532 | T | \|\| id:ebi-a-GCST90001710 | ebi-a-GCST90001710 | TRUE | C | 41814199 | 3.83E-08 | reported | 3657 | 0.05662 | rs79090302 |  |  |  |  |  |  |  |  |  |  |  |  |  |  |  |
| -0.3156 | 10 | igd | 0.0531 | A | \|\| id:ebi-a-GCST90001658 | ebi-a-GCST90001658 | TRUE | G | 95569379 | 2.32E-08 | reported | 3440 | 0.05637 | rs78573011 |  |  |  |  |  |  |  |  |  |  |  |  |  |  |  |
| -0.3119 | 10 | igd | 0.0531 | A | \|\| id:ebi-a-GCST90001659 | ebi-a-GCST90001659 | TRUE | G | 95569379 | 3.42E-08 | reported | 3440 | 0.05639 | rs78573011 |  |  |  |  |  |  |  |  |  |  |  |  |  |  |  |
| -0.3432 | 10 | igd | 0.0531 | A | \|\| id:ebi-a-GCST90001670 | ebi-a-GCST90001670 | TRUE | G | 95569379 | 5.51E-09 | reported | 3440 | 0.0587 | rs78573011 |  |  |  |  |  |  |  |  |  |  |  |  |  |  |  |
| -0.356 | 10 | igd | 0.0531 | A | \|\| id:ebi-a-GCST90001671 | ebi-a-GCST90001671 | TRUE | G | 95569379 | 1.48E-09 | reported | 3440 | 0.05871 | rs78573011 |  |  |  |  |  |  |  |  |  |  |  |  |  |  |  |
| -0.3732 | 2 | igd | 0.0531 | C | \|\| id:ebi-a-GCST90001890 | ebi-a-GCST90001890 | TRUE | T | 203663746 | 2.73E-09 | reported | 2920 | 0.06255 | rs111895558 |  |  |  |  |  |  |  |  |  |  |  |  |  |  |  |
| -0.3774 | 2 | igd | 0.0531 | C | \|\| id:ebi-a-GCST90001891 | ebi-a-GCST90001891 | TRUE | T | 203663746 | 1.02E-09 | reported | 2920 | 0.06161 | rs111895558 |  |  |  |  |  |  |  |  |  |  |  |  |  |  |  |
| -0.3445 | 2 | igd | 0.0531 | C | \|\| id:ebi-a-GCST90001894 | ebi-a-GCST90001894 | TRUE | T | 203663746 | 2.76E-08 | reported | 2920 | 0.06183 | rs111895558 |  |  |  |  |  |  |  |  |  |  |  |  |  |  |  |
| -0.3442 | 2 | igd | 0.0531 | C | \|\| id:ebi-a-GCST90001898 | ebi-a-GCST90001898 | TRUE | T | 203663746 | 2.86E-08 | reported | 2920 | 0.06185 | rs111895558 |  |  |  |  |  |  |  |  |  |  |  |  |  |  |  |
| -0.4028 | 2 | igd | 0.0531 | C | \|\| id:ebi-a-GCST90001900 | ebi-a-GCST90001900 | TRUE | T | 203663746 | 8.19E-11 | reported | 2919 | 0.06176 | rs111895558 |  |  |  |  |  |  |  |  |  |  |  |  |  |  |  |
| -0.3267 | 10 | igd | 0.053 | A | \|\| id:ebi-a-GCST90001491 | ebi-a-GCST90001491 | TRUE | G | 95569379 | 2.38E-08 | reported | 3437 | 0.05839 | rs78573011 |  |  |  |  |  |  |  |  |  |  |  |  |  |  |  |
| -0.3434 | 10 | igd | 0.053 | A | \|\| id:ebi-a-GCST90001496 | ebi-a-GCST90001496 | TRUE | G | 95569379 | 7.03E-09 | reported | 3437 | 0.05916 | rs78573011 |  |  |  |  |  |  |  |  |  |  |  |  |  |  |  |
| -0.3536 | 10 | igd | 0.053 | T | \|\| id:ebi-a-GCST90001496 | ebi-a-GCST90001496 | TRUE | C | 96372939 | 7.16E-10 | reported | 3437 | 0.05722 | rs41291548 |  |  |  |  |  |  |  |  |  |  |  |  |  |  |  |
| -0.3521 | 10 | igd | 0.053 | A | \|\| id:ebi-a-GCST90001497 | ebi-a-GCST90001497 | TRUE | G | 95569379 | 1.81E-09 | reported | 3437 | 0.05839 | rs78573011 |  |  |  |  |  |  |  |  |  |  |  |  |  |  |  |
| -0.3415 | 10 | igd | 0.053 | T | \|\| id:ebi-a-GCST90001497 | ebi-a-GCST90001497 | TRUE | C | 96372939 | 1.67E-09 | reported | 3437 | 0.05651 | rs41291548 |  |  |  |  |  |  |  |  |  |  |  |  |  |  |  |
| -0.3216 | 10 | igd | 0.0529 | T | \|\| id:ebi-a-GCST90001489 | ebi-a-GCST90001489 | TRUE | C | 96372939 | 2.27E-08 | reported | 3405 | 0.0574 | rs41291548 |  |  |  |  |  |  |  |  |  |  |  |  |  |  |  |
| -0.3421 | 10 | igd | 0.0529 | T | \|\| id:ebi-a-GCST90001495 | ebi-a-GCST90001495 | TRUE | C | 96372939 | 3.72E-09 | reported | 3405 | 0.05787 | rs41291548 |  |  |  |  |  |  |  |  |  |  |  |  |  |  |  |
| -0.3078 | 22 | igd | 0.0529 | T | \|\| id:ebi-a-GCST90001702 | ebi-a-GCST90001702 | TRUE | C | 38659322 | 2.40E-08 | reported | 3657 | 0.05503 | rs113726896 |  |  |  |  |  |  |  |  |  |  |  |  |  |  |  |
| -0.3129 | 22 | igd | 0.0529 | T | \|\| id:ebi-a-GCST90001703 | ebi-a-GCST90001703 | TRUE | C | 38659322 | 1.39E-08 | reported | 3657 | 0.05502 | rs113726896 |  |  |  |  |  |  |  |  |  |  |  |  |  |  |  |
| -0.3086 | 22 | igd | 0.0529 | T | \|\| id:ebi-a-GCST90001710 | ebi-a-GCST90001710 | TRUE | C | 38659322 | 1.98E-08 | reported | 3657 | 0.05485 | rs113726896 |  |  |  |  |  |  |  |  |  |  |  |  |  |  |  |
| -0.3011 | 22 | igd | 0.0529 | T | \|\| id:ebi-a-GCST90001711 | ebi-a-GCST90001711 | TRUE | C | 38659322 | 4.13E-08 | reported | 3657 | 0.05477 | rs113726896 |  |  |  |  |  |  |  |  |  |  |  |  |  |  |  |
| 0.3476 | 22 | igd | 0.0528 | T | \|\| id:ebi-a-GCST90001702 | ebi-a-GCST90001702 | TRUE | C | 41208244 | 5.51E-10 | reported | 3657 | 0.05587 | rs112119625 |  |  |  |  |  |  |  |  |  |  |  |  |  |  |  |
| 0.3325 | 22 | igd | 0.0528 | T | \|\| id:ebi-a-GCST90001703 | ebi-a-GCST90001703 | TRUE | C | 41208244 | 2.97E-09 | reported | 3657 | 0.05589 | rs112119625 |  |  |  |  |  |  |  |  |  |  |  |  |  |  |  |
| 0.3186 | 22 | igd | 0.0528 | T | \|\| id:ebi-a-GCST90001704 | ebi-a-GCST90001704 | TRUE | C | 41208244 | 1.25E-08 | reported | 3657 | 0.05584 | rs112119625 |  |  |  |  |  |  |  |  |  |  |  |  |  |  |  |
| 0.3285 | 22 | igd | 0.0528 | T | \|\| id:ebi-a-GCST90001710 | ebi-a-GCST90001710 | TRUE | C | 41208244 | 4.15E-09 | reported | 3657 | 0.05575 | rs112119625 |  |  |  |  |  |  |  |  |  |  |  |  |  |  |  |
| 0.3147 | 22 | igd | 0.0528 | T | \|\| id:ebi-a-GCST90001711 | ebi-a-GCST90001711 | TRUE | C | 41208244 | 1.67E-08 | reported | 3657 | 0.05564 | rs112119625 |  |  |  |  |  |  |  |  |  |  |  |  |  |  |  |
| 0.4311 | 6 | igd | 0.0528 | T | \|\| id:ebi-a-GCST90002105 | ebi-a-GCST90002105 | TRUE | C | 31634971 | 1.69E-11 | reported | 2872 | 0.06379 | rs36048548 |  |  |  |  |  |  |  |  |  |  |  |  |  |  |  |
| 0.3596 | 10 | igd | 0.0527 | A | \|\| id:ebi-a-GCST90001672 | ebi-a-GCST90001672 | TRUE | G | 93961353 | 9.61E-10 | reported | 3408 | 0.05863 | rs117962493 |  |  |  |  |  |  |  |  |  |  |  |  |  |  |  |
| -0.5256 | 22 | igd | 0.0526 | G | \|\| id:ebi-a-GCST90001702 | ebi-a-GCST90001702 | TRUE | A | 40774205 | 9.05E-20 | reported | 3657 | 0.05743 | rs149806587 |  |  |  |  |  |  |  |  |  |  |  |  |  |  |  |
| -0.5165 | 22 | igd | 0.0526 | G | \|\| id:ebi-a-GCST90001703 | ebi-a-GCST90001703 | TRUE | A | 40774205 | 3.84E-19 | reported | 3657 | 0.05745 | rs149806587 |  |  |  |  |  |  |  |  |  |  |  |  |  |  |  |
| -0.5384 | 22 | igd | 0.0526 | G | \|\| id:ebi-a-GCST90001704 | ebi-a-GCST90001704 | TRUE | A | 40774205 | 9.77E-21 | reported | 3657 | 0.05731 | rs149806587 |  |  |  |  |  |  |  |  |  |  |  |  |  |  |  |
| -0.5402 | 22 | igd | 0.0526 | G | \|\| id:ebi-a-GCST90001710 | ebi-a-GCST90001710 | TRUE | A | 40774205 | 6.65E-21 | reported | 3657 | 0.05724 | rs149806587 |  |  |  |  |  |  |  |  |  |  |  |  |  |  |  |
| -0.5387 | 22 | igd | 0.0526 | G | \|\| id:ebi-a-GCST90001711 | ebi-a-GCST90001711 | TRUE | A | 40774205 | 6.70E-21 | reported | 3657 | 0.05709 | rs149806587 |  |  |  |  |  |  |  |  |  |  |  |  |  |  |  |
| 0.4946 | 6 | igd | 0.0526 | C | \|\| id:ebi-a-GCST90002108 | ebi-a-GCST90002108 | TRUE | T | 29495815 | 3.53E-08 | reported | 1579 | 0.08927 | rs6913746 |  |  |  |  |  |  |  |  |  |  |  |  |  |  |  |
| 0.5201 | 6 | igd | 0.0526 | C | \|\| id:ebi-a-GCST90002108 | ebi-a-GCST90002108 | TRUE | T | 29554829 | 5.19E-09 | reported | 1579 | 0.08855 | rs114383434 |  |  |  |  |  |  |  |  |  |  |  |  |  |  |  |
| -0.355 | 10 | igd | 0.0525 | T | \|\| id:ebi-a-GCST90001496 | ebi-a-GCST90001496 | TRUE | C | 96670061 | 7.76E-10 | reported | 3437 | 0.05756 | rs141856853 |  |  |  |  |  |  |  |  |  |  |  |  |  |  |  |
| -0.34 | 10 | igd | 0.0525 | T | \|\| id:ebi-a-GCST90001497 | ebi-a-GCST90001497 | TRUE | C | 96670061 | 2.46E-09 | reported | 3437 | 0.05685 | rs141856853 |  |  |  |  |  |  |  |  |  |  |  |  |  |  |  |
| -0.3296 | 10 | igd | 0.0524 | T | \|\| id:ebi-a-GCST90001489 | ebi-a-GCST90001489 | TRUE | C | 96670061 | 1.24E-08 | reported | 3405 | 0.05774 | rs141856853 |  |  |  |  |  |  |  |  |  |  |  |  |  |  |  |
| -0.3467 | 10 | igd | 0.0524 | T | \|\| id:ebi-a-GCST90001495 | ebi-a-GCST90001495 | TRUE | C | 96670061 | 2.85E-09 | reported | 3405 | 0.05821 | rs141856853 |  |  |  |  |  |  |  |  |  |  |  |  |  |  |  |
| -0.394 | 2 | igd | 0.0524 | G | \|\| id:ebi-a-GCST90001893 | ebi-a-GCST90001893 | TRUE | A | 204668145 | 2.36E-10 | reported | 2920 | 0.06197 | rs115472295 |  |  |  |  |  |  |  |  |  |  |  |  |  |  |  |
| -0.5585 | 19 | igd | 0.0523 | A | \|\| id:ebi-a-GCST90001952 | ebi-a-GCST90001952 | TRUE | C | 51542085 | 3.45E-11 | reported | 1569 | 0.08369 | rs62117667 |  |  |  |  |  |  |  |  |  |  |  |  |  |  |  |
| 0.3892 | 22 | igd | 0.0522 | T | \|\| id:ebi-a-GCST90001702 | ebi-a-GCST90001702 | TRUE | C | 42261360 | 4.96E-12 | reported | 3657 | 0.05616 | rs78302660 |  |  |  |  |  |  |  |  |  |  |  |  |  |  |  |
| 0.3737 | 22 | igd | 0.0522 | T | \|\| id:ebi-a-GCST90001703 | ebi-a-GCST90001703 | TRUE | C | 42261360 | 3.35E-11 | reported | 3657 | 0.05618 | rs78302660 |  |  |  |  |  |  |  |  |  |  |  |  |  |  |  |
| 0.3528 | 22 | igd | 0.0522 | T | \|\| id:ebi-a-GCST90001704 | ebi-a-GCST90001704 | TRUE | C | 42261360 | 3.71E-10 | reported | 3657 | 0.05615 | rs78302660 |  |  |  |  |  |  |  |  |  |  |  |  |  |  |  |
| 0.3629 | 22 | igd | 0.0522 | T | \|\| id:ebi-a-GCST90001710 | ebi-a-GCST90001710 | TRUE | C | 42261360 | 1.07E-10 | reported | 3657 | 0.05604 | rs78302660 |  |  |  |  |  |  |  |  |  |  |  |  |  |  |  |
| 0.3457 | 22 | igd | 0.0522 | T | \|\| id:ebi-a-GCST90001711 | ebi-a-GCST90001711 | TRUE | C | 42261360 | 7.22E-10 | reported | 3657 | 0.05595 | rs78302660 |  |  |  |  |  |  |  |  |  |  |  |  |  |  |  |
| -0.5761 | 19 | igd | 0.0521 | A | \|\| id:ebi-a-GCST90001954 | ebi-a-GCST90001954 | TRUE | C | 51542085 | 6.14E-12 | reported | 1555 | 0.08313 | rs62117667 |  |  |  |  |  |  |  |  |  |  |  |  |  |  |  |
| -0.5728 | 19 | igd | 0.0521 | A | \|\| id:ebi-a-GCST90001955 | ebi-a-GCST90001955 | TRUE | C | 51542085 | 9.50E-12 | reported | 1554 | 0.08342 | rs62117667 |  |  |  |  |  |  |  |  |  |  |  |  |  |  |  |
| 0.3593 | 6 | igd | 0.052 | A | \|\| id:ebi-a-GCST90001820 | ebi-a-GCST90001820 | TRUE | C | 43351686 | 5.51E-10 | reported | 3656 | 0.05776 | rs139084170 |  |  |  |  |  |  |  |  |  |  |  |  |  |  |  |
| -0.5752 | 19 | igd | 0.052 | A | \|\| id:ebi-a-GCST90001953 | ebi-a-GCST90001953 | TRUE | C | 51542085 | 6.67E-12 | reported | 1558 | 0.08315 | rs62117667 |  |  |  |  |  |  |  |  |  |  |  |  |  |  |  |
| -0.5754 | 19 | igd | 0.0519 | A | \|\| id:ebi-a-GCST90001956 | ebi-a-GCST90001956 | TRUE | C | 51542085 | 7.05E-12 | reported | 1579 | 0.08329 | rs62117667 |  |  |  |  |  |  |  |  |  |  |  |  |  |  |  |
| 0.3683 | 17 | igd | 0.0519 | T | \|\| id:ebi-a-GCST90001964 | ebi-a-GCST90001964 | TRUE | C | 63066415 | 3.33E-09 | reported | 2872 | 0.06208 | rs144843975 |  |  |  |  |  |  |  |  |  |  |  |  |  |  |  |
| -0.5591 | 19 | igd | 0.0517 | A | \|\| id:ebi-a-GCST90001947 | ebi-a-GCST90001947 | TRUE | C | 51542085 | 3.38E-11 | reported | 1577 | 0.08374 | rs62117667 |  |  |  |  |  |  |  |  |  |  |  |  |  |  |  |
| -0.5823 | 19 | igd | 0.0516 | A | \|\| id:ebi-a-GCST90001957 | ebi-a-GCST90001957 | TRUE | C | 51542085 | 4.42E-12 | reported | 1578 | 0.08346 | rs62117667 |  |  |  |  |  |  |  |  |  |  |  |  |  |  |  |
| -0.3443 | 10 | igd | 0.0515 | C | \|\| id:ebi-a-GCST90001483 | ebi-a-GCST90001483 | TRUE | T | 97974757 | 1.08E-09 | reported | 3405 | 0.05631 | rs79049066 |  |  |  |  |  |  |  |  |  |  |  |  |  |  |  |
| -0.3508 | 10 | igd | 0.0515 | C | \|\| id:ebi-a-GCST90001484 | ebi-a-GCST90001484 | TRUE | T | 97974757 | 3.69E-10 | reported | 3437 | 0.05582 | rs79049066 |  |  |  |  |  |  |  |  |  |  |  |  |  |  |  |
| -0.3441 | 10 | igd | 0.0515 | C | \|\| id:ebi-a-GCST90001485 | ebi-a-GCST90001485 | TRUE | T | 97974757 | 7.50E-10 | reported | 3437 | 0.05575 | rs79049066 |  |  |  |  |  |  |  |  |  |  |  |  |  |  |  |
| -0.4114 | 10 | igd | 0.0515 | C | \|\| id:ebi-a-GCST90001489 | ebi-a-GCST90001489 | TRUE | T | 97974757 | 1.26E-12 | reported | 3405 | 0.05774 | rs79049066 |  |  |  |  |  |  |  |  |  |  |  |  |  |  |  |
| -0.3646 | 10 | igd | 0.0515 | C | \|\| id:ebi-a-GCST90001491 | ebi-a-GCST90001491 | TRUE | T | 97974757 | 1.77E-10 | reported | 3437 | 0.05697 | rs79049066 |  |  |  |  |  |  |  |  |  |  |  |  |  |  |  |
| -0.4945 | 10 | igd | 0.0515 | C | \|\| id:ebi-a-GCST90001495 | ebi-a-GCST90001495 | TRUE | T | 97974757 | 2.63E-17 | reported | 3405 | 0.05813 | rs79049066 |  |  |  |  |  |  |  |  |  |  |  |  |  |  |  |
| -0.4716 | 10 | igd | 0.0515 | C | \|\| id:ebi-a-GCST90001496 | ebi-a-GCST90001496 | TRUE | T | 97974757 | 3.41E-16 | reported | 3437 | 0.05752 | rs79049066 |  |  |  |  |  |  |  |  |  |  |  |  |  |  |  |
| -0.4642 | 10 | igd | 0.0515 | C | \|\| id:ebi-a-GCST90001497 | ebi-a-GCST90001497 | TRUE | T | 97974757 | 4.23E-16 | reported | 3437 | 0.0568 | rs79049066 |  |  |  |  |  |  |  |  |  |  |  |  |  |  |  |
| -0.4645 | 10 | igd | 0.0515 | C | \|\| id:ebi-a-GCST90001658 | ebi-a-GCST90001658 | TRUE | T | 97974757 | 3.60E-17 | reported | 3440 | 0.05484 | rs79049066 |  |  |  |  |  |  |  |  |  |  |  |  |  |  |  |
| -0.463 | 10 | igd | 0.0515 | C | \|\| id:ebi-a-GCST90001659 | ebi-a-GCST90001659 | TRUE | T | 97974757 | 4.59E-17 | reported | 3440 | 0.05485 | rs79049066 |  |  |  |  |  |  |  |  |  |  |  |  |  |  |  |
| -0.5199 | 10 | igd | 0.0515 | C | \|\| id:ebi-a-GCST90001660 | ebi-a-GCST90001660 | TRUE | T | 97974757 | 2.19E-19 | reported | 3408 | 0.0574 | rs79049066 |  |  |  |  |  |  |  |  |  |  |  |  |  |  |  |
| -0.4606 | 10 | igd | 0.0515 | C | \|\| id:ebi-a-GCST90001670 | ebi-a-GCST90001670 | TRUE | T | 97974757 | 1.00E-15 | reported | 3440 | 0.05711 | rs79049066 |  |  |  |  |  |  |  |  |  |  |  |  |  |  |  |
| -0.4628 | 10 | igd | 0.0515 | C | \|\| id:ebi-a-GCST90001671 | ebi-a-GCST90001671 | TRUE | T | 97974757 | 8.26E-16 | reported | 3440 | 0.05721 | rs79049066 |  |  |  |  |  |  |  |  |  |  |  |  |  |  |  |
| -0.5003 | 10 | igd | 0.0515 | C | \|\| id:ebi-a-GCST90001672 | ebi-a-GCST90001672 | TRUE | T | 97974757 | 1.00E-17 | reported | 3408 | 0.05803 | rs79049066 |  |  |  |  |  |  |  |  |  |  |  |  |  |  |  |
| -0.5927 | 22 | igd | 0.0515 | A | \|\| id:ebi-a-GCST90001702 | ebi-a-GCST90001702 | TRUE | G | 42110234 | 1.06E-23 | reported | 3657 | 0.05865 | rs55638148 |  |  |  |  |  |  |  |  |  |  |  |  |  |  |  |
| 0.3394 | 22 | igd | 0.0515 | A | \|\| id:ebi-a-GCST90001702 | ebi-a-GCST90001702 | TRUE | G | 41499822 | 1.77E-09 | reported | 3657 | 0.05626 | rs17433014 |  |  |  |  |  |  |  |  |  |  |  |  |  |  |  |
| -0.5676 | 22 | igd | 0.0515 | A | \|\| id:ebi-a-GCST90001703 | ebi-a-GCST90001703 | TRUE | G | 42110234 | 7.21E-22 | reported | 3657 | 0.05869 | rs55638148 |  |  |  |  |  |  |  |  |  |  |  |  |  |  |  |
| 0.3273 | 22 | igd | 0.0515 | A | \|\| id:ebi-a-GCST90001703 | ebi-a-GCST90001703 | TRUE | G | 41499822 | 6.53E-09 | reported | 3657 | 0.05627 | rs17433014 |  |  |  |  |  |  |  |  |  |  |  |  |  |  |  |
| -0.4714 | 22 | igd | 0.0515 | A | \|\| id:ebi-a-GCST90001704 | ebi-a-GCST90001704 | TRUE | G | 42110234 | 1.47E-15 | reported | 3657 | 0.05882 | rs55638148 |  |  |  |  |  |  |  |  |  |  |  |  |  |  |  |
| -0.5681 | 22 | igd | 0.0515 | A | \|\| id:ebi-a-GCST90001710 | ebi-a-GCST90001710 | TRUE | G | 42110234 | 5.77E-22 | reported | 3657 | 0.05859 | rs55638148 |  |  |  |  |  |  |  |  |  |  |  |  |  |  |  |
| 0.3114 | 22 | igd | 0.0515 | A | \|\| id:ebi-a-GCST90001710 | ebi-a-GCST90001710 | TRUE | G | 41499822 | 3.12E-08 | reported | 3657 | 0.05614 | rs17433014 |  |  |  |  |  |  |  |  |  |  |  |  |  |  |  |
| -0.5588 | 22 | igd | 0.0515 | A | \|\| id:ebi-a-GCST90001711 | ebi-a-GCST90001711 | TRUE | G | 42110234 | 1.90E-21 | reported | 3657 | 0.05839 | rs55638148 |  |  |  |  |  |  |  |  |  |  |  |  |  |  |  |
| -0.383 | 10 | igd | 0.0515 | C | \|\| id:ebi-a-GCST90002031 | ebi-a-GCST90002031 | TRUE | G | 97128470 | 1.85E-09 | reported | 2920 | 0.06353 | rs142926792 |  |  |  |  |  |  |  |  |  |  |  |  |  |  |  |
| 0.3828 | 1 | igd | 0.0514 | T | \|\| id:ebi-a-GCST90001864 | ebi-a-GCST90001864 | TRUE | C | 166658530 | 2.04E-09 | reported | 2919 | 0.06366 | rs78365625 |  |  |  |  |  |  |  |  |  |  |  |  |  |  |  |
| -0.326 | 1 | igd | 0.0514 | T | \|\| id:ebi-a-GCST90001989 | ebi-a-GCST90001989 | TRUE | C | 161593831 | 1.25E-08 | reported | 3621 | 0.05713 | rs72702127 |  |  |  |  |  |  |  |  |  |  |  |  |  |  |  |
| -0.3544 | 22 | igd | 0.0513 | C | \|\| id:ebi-a-GCST90001830 | ebi-a-GCST90001830 | TRUE | G | 42064838 | 1.44E-10 | reported | 3648 | 0.05511 | rs117698911 |  |  |  |  |  |  |  |  |  |  |  |  |  |  |  |
| 0.5386 | 1 | igd | 0.0512 | C | \|\| id:ebi-a-GCST90001858 | ebi-a-GCST90001858 | TRUE | T | 167173140 | 1.51E-18 | reported | 2919 | 0.06087 | rs4657649 |  |  |  |  |  |  |  |  |  |  |  |  |  |  |  |
| 0.3573 | 1 | igd | 0.0512 | T | \|\| id:ebi-a-GCST90001858 | ebi-a-GCST90001858 | TRUE | C | 166663665 | 2.92E-08 | reported | 2919 | 0.06425 | rs11579223 |  |  |  |  |  |  |  |  |  |  |  |  |  |  |  |
| 0.4331 | 1 | igd | 0.0512 | C | \|\| id:ebi-a-GCST90001861 | ebi-a-GCST90001861 | TRUE | T | 167173140 | 1.12E-12 | reported | 2919 | 0.0606 | rs4657649 |  |  |  |  |  |  |  |  |  |  |  |  |  |  |  |
| 0.3372 | 1 | igd | 0.0512 | C | \|\| id:ebi-a-GCST90001863 | ebi-a-GCST90001863 | TRUE | T | 167173140 | 2.96E-08 | reported | 2919 | 0.06066 | rs4657649 |  |  |  |  |  |  |  |  |  |  |  |  |  |  |  |
| 0.447 | 1 | igd | 0.0512 | C | \|\| id:ebi-a-GCST90001864 | ebi-a-GCST90001864 | TRUE | T | 167173140 | 3.64E-13 | reported | 2919 | 0.06122 | rs4657649 |  |  |  |  |  |  |  |  |  |  |  |  |  |  |  |
| 0.3776 | 6 | igd | 0.0512 | A | \|\| id:ebi-a-GCST90002105 | ebi-a-GCST90002105 | TRUE | G | 28065038 | 3.55E-09 | reported | 2872 | 0.06376 | rs62401434 |  |  |  |  |  |  |  |  |  |  |  |  |  |  |  |
| -0.4738 | 6 | igd | 0.0512 | T | \|\| id:ebi-a-GCST90002105 | ebi-a-GCST90002105 | TRUE | A | 32381940 | 1.75E-13 | reported | 2872 | 0.06401 | rs28366194 |  |  |  |  |  |  |  |  |  |  |  |  |  |  |  |
| -0.3403 | 22 | igd | 0.0511 | T | \|\| id:ebi-a-GCST90001702 | ebi-a-GCST90001702 | TRUE | G | 45019262 | 6.21E-09 | reported | 3657 | 0.05843 | rs8135271 |  |  |  |  |  |  |  |  |  |  |  |  |  |  |  |
| -0.3272 | 22 | igd | 0.0511 | T | \|\| id:ebi-a-GCST90001703 | ebi-a-GCST90001703 | TRUE | G | 45019262 | 2.33E-08 | reported | 3657 | 0.05844 | rs8135271 |  |  |  |  |  |  |  |  |  |  |  |  |  |  |  |
| 0.3751 | 3 | igd | 0.0511 | A | \|\| id:ebi-a-GCST90001798 | ebi-a-GCST90001798 | TRUE | G | 32848142 | 3.05E-11 | reported | 3657 | 0.05628 | rs140924680 |  |  |  |  |  |  |  |  |  |  |  |  |  |  |  |
| 0.3569 | 3 | igd | 0.0511 | A | \|\| id:ebi-a-GCST90001800 | ebi-a-GCST90001800 | TRUE | G | 32848142 | 4.98E-10 | reported | 3656 | 0.05722 | rs140924680 |  |  |  |  |  |  |  |  |  |  |  |  |  |  |  |
| 0.3137 | 3 | igd | 0.0511 | A | \|\| id:ebi-a-GCST90001802 | ebi-a-GCST90001802 | TRUE | G | 32848142 | 4.05E-08 | reported | 3657 | 0.05704 | rs140924680 |  |  |  |  |  |  |  |  |  |  |  |  |  |  |  |
| 0.3266 | 3 | igd | 0.0511 | A | \|\| id:ebi-a-GCST90001805 | ebi-a-GCST90001805 | TRUE | G | 32848142 | 1.06E-08 | reported | 3657 | 0.05696 | rs140924680 |  |  |  |  |  |  |  |  |  |  |  |  |  |  |  |
| 0.4231 | 3 | igd | 0.0511 | A | \|\| id:ebi-a-GCST90001806 | ebi-a-GCST90001806 | TRUE | G | 32848142 | 1.20E-13 | reported | 3656 | 0.05683 | rs140924680 |  |  |  |  |  |  |  |  |  |  |  |  |  |  |  |
| 0.3699 | 3 | igd | 0.0511 | A | \|\| id:ebi-a-GCST90001808 | ebi-a-GCST90001808 | TRUE | G | 32848142 | 8.10E-11 | reported | 3657 | 0.05676 | rs140924680 |  |  |  |  |  |  |  |  |  |  |  |  |  |  |  |
| 0.4147 | 1 | igd | 0.051 | C | \|\| id:ebi-a-GCST90001845 | ebi-a-GCST90001845 | TRUE | T | 167173140 | 2.05E-11 | reported | 2911 | 0.06162 | rs4657649 |  |  |  |  |  |  |  |  |  |  |  |  |  |  |  |
| -0.3519 | 10 | igd | 0.051 | C | \|\| id:ebi-a-GCST90001961 | ebi-a-GCST90001961 | TRUE | T | 92733574 | 1.50E-08 | reported | 2920 | 0.06198 | rs74472826 |  |  |  |  |  |  |  |  |  |  |  |  |  |  |  |
| 0.414 | 10 | igd | 0.051 | C | \|\| id:ebi-a-GCST90001961 | ebi-a-GCST90001961 | TRUE | T | 97974757 | 3.23E-11 | reported | 2920 | 0.06215 | rs79049066 |  |  |  |  |  |  |  |  |  |  |  |  |  |  |  |
| -0.5992 | 10 | igd | 0.051 | G | \|\| id:ebi-a-GCST90002030 | ebi-a-GCST90002030 | TRUE | A | 97978518 | 3.07E-22 | reported | 2920 | 0.06128 | rs17232052 |  |  |  |  |  |  |  |  |  |  |  |  |  |  |  |
| -0.4492 | 10 | igd | 0.051 | A | \|\| id:ebi-a-GCST90002030 | ebi-a-GCST90002030 | TRUE | G | 96526812 | 3.85E-13 | reported | 2920 | 0.06159 | rs143622857 |  |  |  |  |  |  |  |  |  |  |  |  |  |  |  |
| -0.5451 | 10 | igd | 0.051 | C | \|\| id:ebi-a-GCST90002031 | ebi-a-GCST90002031 | TRUE | T | 97974757 | 5.14E-18 | reported | 2920 | 0.0626 | rs79049066 |  |  |  |  |  |  |  |  |  |  |  |  |  |  |  |
| -0.378 | 10 | igd | 0.051 | A | \|\| id:ebi-a-GCST90002031 | ebi-a-GCST90002031 | TRUE | G | 96526812 | 2.45E-09 | reported | 2920 | 0.06317 | rs143622857 |  |  |  |  |  |  |  |  |  |  |  |  |  |  |  |
| -0.3738 | 10 | igd | 0.051 | A | \|\| id:ebi-a-GCST90002032 | ebi-a-GCST90002032 | TRUE | G | 96526812 | 3.60E-09 | reported | 2920 | 0.06315 | rs143622857 |  |  |  |  |  |  |  |  |  |  |  |  |  |  |  |
| -0.5057 | 10 | igd | 0.051 | G | \|\| id:ebi-a-GCST90002032 | ebi-a-GCST90002032 | TRUE | A | 97978518 | 1.07E-15 | reported | 2920 | 0.06272 | rs17232052 |  |  |  |  |  |  |  |  |  |  |  |  |  |  |  |
| -0.3153 | 22 | igd | 0.0509 | A | \|\| id:ebi-a-GCST90001702 | ebi-a-GCST90001702 | TRUE | G | 39580427 | 3.90E-08 | reported | 3657 | 0.05726 | rs146264976 |  |  |  |  |  |  |  |  |  |  |  |  |  |  |  |
| -0.3225 | 22 | igd | 0.0509 | A | \|\| id:ebi-a-GCST90001703 | ebi-a-GCST90001703 | TRUE | G | 39580427 | 1.89E-08 | reported | 3657 | 0.05724 | rs146264976 |  |  |  |  |  |  |  |  |  |  |  |  |  |  |  |
| -0.3487 | 22 | igd | 0.0509 | A | \|\| id:ebi-a-GCST90001704 | ebi-a-GCST90001704 | TRUE | G | 39580427 | 1.14E-09 | reported | 3657 | 0.05713 | rs146264976 |  |  |  |  |  |  |  |  |  |  |  |  |  |  |  |
| -0.3193 | 22 | igd | 0.0509 | A | \|\| id:ebi-a-GCST90001711 | ebi-a-GCST90001711 | TRUE | G | 39580427 | 2.23E-08 | reported | 3657 | 0.05696 | rs146264976 |  |  |  |  |  |  |  |  |  |  |  |  |  |  |  |
| -0.5624 | 1 | igd | 0.0509 | C | \|\| id:ebi-a-GCST90001854 | ebi-a-GCST90001854 | TRUE | T | 167216803 | 9.08E-20 | reported | 2919 | 0.06136 | rs2036194 |  |  |  |  |  |  |  |  |  |  |  |  |  |  |  |
| -0.4568 | 1 | igd | 0.0509 | C | \|\| id:ebi-a-GCST90001856 | ebi-a-GCST90001856 | TRUE | T | 167216803 | 2.74E-13 | reported | 2919 | 0.06224 | rs2036194 |  |  |  |  |  |  |  |  |  |  |  |  |  |  |  |
| -0.5276 | 1 | igd | 0.0509 | C | \|\| id:ebi-a-GCST90001860 | ebi-a-GCST90001860 | TRUE | T | 167216803 | 3.40E-17 | reported | 2919 | 0.06219 | rs2036194 |  |  |  |  |  |  |  |  |  |  |  |  |  |  |  |
| -0.5317 | 10 | igd | 0.0507 | G | \|\| id:ebi-a-GCST90001961 | ebi-a-GCST90001961 | TRUE | A | 97941732 | 7.73E-16 | reported | 2920 | 0.06561 | rs144301498 |  |  |  |  |  |  |  |  |  |  |  |  |  |  |  |
| 0.9038 | 10 | igd | 0.0506 | C | \|\| id:ebi-a-GCST90001484 | ebi-a-GCST90001484 | TRUE | T | 97810637 | 1.23E-53 | reported | 3437 | 0.0576 | rs880518 |  |  |  |  |  |  |  |  |  |  |  |  |  |  |  |
| 0.858 | 10 | igd | 0.0506 | C | \|\| id:ebi-a-GCST90001485 | ebi-a-GCST90001485 | TRUE | T | 97810637 | 1.73E-48 | reported | 3437 | 0.05772 | rs880518 |  |  |  |  |  |  |  |  |  |  |  |  |  |  |  |
| -0.376 | 10 | igd | 0.0506 | C | \|\| id:ebi-a-GCST90001658 | ebi-a-GCST90001658 | TRUE | G | 97128470 | 2.39E-11 | reported | 3440 | 0.0561 | rs142926792 |  |  |  |  |  |  |  |  |  |  |  |  |  |  |  |
| -0.3773 | 10 | igd | 0.0506 | C | \|\| id:ebi-a-GCST90001659 | ebi-a-GCST90001659 | TRUE | G | 97128470 | 2.04E-11 | reported | 3440 | 0.0561 | rs142926792 |  |  |  |  |  |  |  |  |  |  |  |  |  |  |  |
| -0.3657 | 10 | igd | 0.0506 | C | \|\| id:ebi-a-GCST90001660 | ebi-a-GCST90001660 | TRUE | G | 97128470 | 6.12E-10 | reported | 3408 | 0.05893 | rs142926792 |  |  |  |  |  |  |  |  |  |  |  |  |  |  |  |
| -0.3918 | 10 | igd | 0.0506 | C | \|\| id:ebi-a-GCST90001670 | ebi-a-GCST90001670 | TRUE | G | 97128470 | 2.28E-11 | reported | 3440 | 0.05839 | rs142926792 |  |  |  |  |  |  |  |  |  |  |  |  |  |  |  |
| -0.3855 | 10 | igd | 0.0506 | C | \|\| id:ebi-a-GCST90001671 | ebi-a-GCST90001671 | TRUE | G | 97128470 | 5.00E-11 | reported | 3440 | 0.05848 | rs142926792 |  |  |  |  |  |  |  |  |  |  |  |  |  |  |  |
| -0.3886 | 10 | igd | 0.0506 | C | \|\| id:ebi-a-GCST90001672 | ebi-a-GCST90001672 | TRUE | G | 97128470 | 7.35E-11 | reported | 3408 | 0.05947 | rs142926792 |  |  |  |  |  |  |  |  |  |  |  |  |  |  |  |
| -0.3716 | 1 | igd | 0.0506 | G | \|\| id:ebi-a-GCST90001996 | ebi-a-GCST90001996 | TRUE | A | 161483843 | 1.40E-10 | reported | 3579 | 0.05775 | rs145947252 |  |  |  |  |  |  |  |  |  |  |  |  |  |  |  |
| 0.8302 | 10 | igd | 0.0505 | C | \|\| id:ebi-a-GCST90001483 | ebi-a-GCST90001483 | TRUE | T | 97810637 | 2.07E-44 | reported | 3405 | 0.05853 | rs880518 |  |  |  |  |  |  |  |  |  |  |  |  |  |  |  |
| 0.3863 | 22 | igd | 0.0505 | T | \|\| id:ebi-a-GCST90001702 | ebi-a-GCST90001702 | TRUE | C | 42039509 | 1.89E-11 | reported | 3657 | 0.05735 | rs112898596 |  |  |  |  |  |  |  |  |  |  |  |  |  |  |  |
| 0.3721 | 22 | igd | 0.0505 | T | \|\| id:ebi-a-GCST90001703 | ebi-a-GCST90001703 | TRUE | C | 42039509 | 9.99E-11 | reported | 3657 | 0.05737 | rs112898596 |  |  |  |  |  |  |  |  |  |  |  |  |  |  |  |
| 0.3435 | 22 | igd | 0.0505 | T | \|\| id:ebi-a-GCST90001704 | ebi-a-GCST90001704 | TRUE | C | 42039509 | 2.30E-09 | reported | 3657 | 0.05735 | rs112898596 |  |  |  |  |  |  |  |  |  |  |  |  |  |  |  |
| 0.3544 | 22 | igd | 0.0505 | T | \|\| id:ebi-a-GCST90001710 | ebi-a-GCST90001710 | TRUE | C | 42039509 | 6.62E-10 | reported | 3657 | 0.05724 | rs112898596 |  |  |  |  |  |  |  |  |  |  |  |  |  |  |  |
| 0.3397 | 22 | igd | 0.0505 | T | \|\| id:ebi-a-GCST90001711 | ebi-a-GCST90001711 | TRUE | C | 42039509 | 3.02E-09 | reported | 3657 | 0.05714 | rs112898596 |  |  |  |  |  |  |  |  |  |  |  |  |  |  |  |
| 0.385 | 10 | igd | 0.0504 | T | \|\| id:ebi-a-GCST90001670 | ebi-a-GCST90001670 | TRUE | A | 94119901 | 4.48E-11 | reported | 3440 | 0.05825 | rs150293382 |  |  |  |  |  |  |  |  |  |  |  |  |  |  |  |
| 0.3427 | 10 | igd | 0.0504 | T | \|\| id:ebi-a-GCST90001671 | ebi-a-GCST90001671 | TRUE | A | 94119901 | 4.89E-09 | reported | 3440 | 0.05843 | rs150293382 |  |  |  |  |  |  |  |  |  |  |  |  |  |  |  |
| -0.3096 | 22 | igd | 0.0504 | A | \|\| id:ebi-a-GCST90001830 | ebi-a-GCST90001830 | TRUE | C | 41939668 | 2.61E-08 | reported | 3648 | 0.0555 | rs78831364 |  |  |  |  |  |  |  |  |  |  |  |  |  |  |  |
| 0.5061 | 2 | igd | 0.0504 | T | \|\| id:ebi-a-GCST90002102 | ebi-a-GCST90002102 | TRUE | C | 39318970 | 4.64E-18 | reported | 2919 | 0.05804 | rs139649559 |  |  |  |  |  |  |  |  |  |  |  |  |  |  |  |
| 0.805 | 10 | igd | 0.0503 | G | \|\| id:ebi-a-GCST90001658 | ebi-a-GCST90001658 | TRUE | A | 97941732 | 3.70E-43 | reported | 3440 | 0.05763 | rs144301498 |  |  |  |  |  |  |  |  |  |  |  |  |  |  |  |
| 0.8358 | 10 | igd | 0.0503 | G | \|\| id:ebi-a-GCST90001659 | ebi-a-GCST90001659 | TRUE | A | 97941732 | 1.78E-46 | reported | 3440 | 0.05752 | rs144301498 |  |  |  |  |  |  |  |  |  |  |  |  |  |  |  |
| 0.7471 | 10 | igd | 0.0503 | G | \|\| id:ebi-a-GCST90001670 | ebi-a-GCST90001670 | TRUE | A | 97941732 | 1.50E-34 | reported | 3440 | 0.06027 | rs144301498 |  |  |  |  |  |  |  |  |  |  |  |  |  |  |  |
| 0.781 | 10 | igd | 0.0503 | G | \|\| id:ebi-a-GCST90001671 | ebi-a-GCST90001671 | TRUE | A | 97941732 | 1.70E-37 | reported | 3440 | 0.0603 | rs144301498 |  |  |  |  |  |  |  |  |  |  |  |  |  |  |  |
| 0.4397 | 10 | igd | 0.0503 | T | \|\| id:ebi-a-GCST90001888 | ebi-a-GCST90001888 | TRUE | C | 97910878 | 6.26E-13 | reported | 2920 | 0.06084 | rs77418133 |  |  |  |  |  |  |  |  |  |  |  |  |  |  |  |
| 0.5169 | 10 | igd | 0.0503 | T | \|\| id:ebi-a-GCST90001892 | ebi-a-GCST90001892 | TRUE | C | 97910878 | 8.62E-17 | reported | 2920 | 0.06173 | rs77418133 |  |  |  |  |  |  |  |  |  |  |  |  |  |  |  |
| 0.492 | 2 | igd | 0.0503 | T | \|\| id:ebi-a-GCST90002098 | ebi-a-GCST90002098 | TRUE | C | 39318970 | 6.09E-17 | reported | 2910 | 0.05847 | rs139649559 |  |  |  |  |  |  |  |  |  |  |  |  |  |  |  |
| 0.9158 | 10 | igd | 0.0502 | G | \|\| id:ebi-a-GCST90001484 | ebi-a-GCST90001484 | TRUE | A | 97941732 | 1.58E-54 | reported | 3437 | 0.05785 | rs144301498 |  |  |  |  |  |  |  |  |  |  |  |  |  |  |  |
| 0.8721 | 10 | igd | 0.0502 | G | \|\| id:ebi-a-GCST90001485 | ebi-a-GCST90001485 | TRUE | A | 97941732 | 1.35E-49 | reported | 3437 | 0.05796 | rs144301498 |  |  |  |  |  |  |  |  |  |  |  |  |  |  |  |
| 0.6842 | 10 | igd | 0.0502 | G | \|\| id:ebi-a-GCST90001491 | ebi-a-GCST90001491 | TRUE | A | 97941732 | 2.02E-29 | reported | 3437 | 0.06019 | rs144301498 |  |  |  |  |  |  |  |  |  |  |  |  |  |  |  |
| 0.8454 | 10 | igd | 0.0502 | G | \|\| id:ebi-a-GCST90001496 | ebi-a-GCST90001496 | TRUE | A | 97941732 | 2.78E-43 | reported | 3437 | 0.06044 | rs144301498 |  |  |  |  |  |  |  |  |  |  |  |  |  |  |  |
| 0.8043 | 10 | igd | 0.0502 | G | \|\| id:ebi-a-GCST90001497 | ebi-a-GCST90001497 | TRUE | A | 97941732 | 3.15E-40 | reported | 3437 | 0.0598 | rs144301498 |  |  |  |  |  |  |  |  |  |  |  |  |  |  |  |
| 0.8799 | 10 | igd | 0.0502 | G | \|\| id:ebi-a-GCST90001660 | ebi-a-GCST90001660 | TRUE | A | 97941732 | 7.78E-47 | reported | 3408 | 0.06029 | rs144301498 |  |  |  |  |  |  |  |  |  |  |  |  |  |  |  |
| 0.7903 | 10 | igd | 0.0502 | G | \|\| id:ebi-a-GCST90001672 | ebi-a-GCST90001672 | TRUE | A | 97941732 | 3.51E-37 | reported | 3408 | 0.06128 | rs144301498 |  |  |  |  |  |  |  |  |  |  |  |  |  |  |  |
| 0.4082 | 22 | igd | 0.0502 | T | \|\| id:ebi-a-GCST90001702 | ebi-a-GCST90001702 | TRUE | C | 42314392 | 3.10E-12 | reported | 3657 | 0.05833 | rs8140914 |  |  |  |  |  |  |  |  |  |  |  |  |  |  |  |
| 0.4143 | 22 | igd | 0.0502 | T | \|\| id:ebi-a-GCST90001703 | ebi-a-GCST90001703 | TRUE | C | 42314392 | 1.44E-12 | reported | 3657 | 0.05831 | rs8140914 |  |  |  |  |  |  |  |  |  |  |  |  |  |  |  |
| 0.3719 | 22 | igd | 0.0502 | T | \|\| id:ebi-a-GCST90001704 | ebi-a-GCST90001704 | TRUE | C | 42314392 | 2.01E-10 | reported | 3657 | 0.0583 | rs8140914 |  |  |  |  |  |  |  |  |  |  |  |  |  |  |  |
| 0.382 | 22 | igd | 0.0502 | T | \|\| id:ebi-a-GCST90001710 | ebi-a-GCST90001710 | TRUE | C | 42314392 | 6.13E-11 | reported | 3657 | 0.05823 | rs8140914 |  |  |  |  |  |  |  |  |  |  |  |  |  |  |  |
| -0.3788 | 1 | igd | 0.0502 | T | \|\| id:ebi-a-GCST90001858 | ebi-a-GCST90001858 | TRUE | C | 167426146 | 2.31E-10 | reported | 2919 | 0.05954 | rs2293325 |  |  |  |  |  |  |  |  |  |  |  |  |  |  |  |
| 0.8442 | 10 | igd | 0.0501 | G | \|\| id:ebi-a-GCST90001483 | ebi-a-GCST90001483 | TRUE | A | 97941732 | 1.82E-45 | reported | 3405 | 0.05877 | rs144301498 |  |  |  |  |  |  |  |  |  |  |  |  |  |  |  |
| 0.6597 | 10 | igd | 0.0501 | G | \|\| id:ebi-a-GCST90001489 | ebi-a-GCST90001489 | TRUE | A | 97941732 | 1.03E-26 | reported | 3405 | 0.06114 | rs144301498 |  |  |  |  |  |  |  |  |  |  |  |  |  |  |  |
| 0.7748 | 10 | igd | 0.0501 | G | \|\| id:ebi-a-GCST90001495 | ebi-a-GCST90001495 | TRUE | A | 97941732 | 1.20E-35 | reported | 3405 | 0.06146 | rs144301498 |  |  |  |  |  |  |  |  |  |  |  |  |  |  |  |
| 0.5717 | 6 | igd | 0.0501 | G | \|\| id:ebi-a-GCST90002109 | ebi-a-GCST90002109 | TRUE | C | 32667850 | 7.18E-10 | reported | 1578 | 0.0922 | rs9275356 |  |  |  |  |  |  |  |  |  |  |  |  |  |  |  |
| 0.3227 | 10 | igd | 0.05 | A | \|\| id:ebi-a-GCST90001659 | ebi-a-GCST90001659 | TRUE | G | 97137021 | 9.38E-09 | reported | 3440 | 0.05607 | rs77195353 |  |  |  |  |  |  |  |  |  |  |  |  |  |  |  |
| -0.3706 | 10 | igd | 0.05 | T | \|\| id:ebi-a-GCST90002030 | ebi-a-GCST90002030 | TRUE | C | 98019117 | 3.17E-09 | reported | 2920 | 0.06238 | rs1870170 |  |  |  |  |  |  |  |  |  |  |  |  |  |  |  |
| -0.3354 | 10 | igd | 0.0499 | A | \|\| id:ebi-a-GCST90001496 | ebi-a-GCST90001496 | TRUE | G | 96526812 | 1.28E-08 | reported | 3437 | 0.05881 | rs143622857 |  |  |  |  |  |  |  |  |  |  |  |  |  |  |  |
| 0.3919 | 10 | igd | 0.0499 | T | \|\| id:ebi-a-GCST90001672 | ebi-a-GCST90001672 | TRUE | A | 94119901 | 5.46E-11 | reported | 3408 | 0.05957 | rs150293382 |  |  |  |  |  |  |  |  |  |  |  |  |  |  |  |
| -0.6061 | 19 | igd | 0.0499 | A | \|\| id:ebi-a-GCST90001951 | ebi-a-GCST90001951 | TRUE | G | 51858911 | 4.02E-12 | reported | 1464 | 0.08664 | rs558625031 |  |  |  |  |  |  |  |  |  |  |  |  |  |  |  |
| -0.3266 | 10 | igd | 0.0498 | A | \|\| id:ebi-a-GCST90001495 | ebi-a-GCST90001495 | TRUE | G | 96526812 | 4.31E-08 | reported | 3405 | 0.05948 | rs143622857 |  |  |  |  |  |  |  |  |  |  |  |  |  |  |  |
| -0.3635 | 6 | igd | 0.0498 | G | \|\| id:ebi-a-GCST90001751 | ebi-a-GCST90001751 | TRUE | A | 15877615 | 9.94E-11 | reported | 3657 | 0.05604 | rs74475541 |  |  |  |  |  |  |  |  |  |  |  |  |  |  |  |
| -0.353 | 6 | igd | 0.0498 | G | \|\| id:ebi-a-GCST90001763 | ebi-a-GCST90001763 | TRUE | A | 15877615 | 5.25E-10 | reported | 3657 | 0.05668 | rs74475541 |  |  |  |  |  |  |  |  |  |  |  |  |  |  |  |
| -0.3573 | 10 | igd | 0.0498 | C | \|\| id:ebi-a-GCST90002061 | ebi-a-GCST90002061 | TRUE | T | 97293322 | 2.80E-08 | reported | 2920 | 0.06417 | rs190517052 |  |  |  |  |  |  |  |  |  |  |  |  |  |  |  |
| -0.4479 | 10 | igd | 0.0497 | T | \|\| id:ebi-a-GCST90002030 | ebi-a-GCST90002030 | TRUE | C | 98352281 | 1.09E-12 | reported | 2920 | 0.06264 | rs11595506 |  |  |  |  |  |  |  |  |  |  |  |  |  |  |  |
| -0.39 | 10 | igd | 0.0497 | T | \|\| id:ebi-a-GCST90002031 | ebi-a-GCST90002031 | TRUE | C | 98352281 | 1.47E-09 | reported | 2920 | 0.06429 | rs11595506 |  |  |  |  |  |  |  |  |  |  |  |  |  |  |  |
| -0.3439 | 10 | igd | 0.0495 | T | \|\| id:ebi-a-GCST90001484 | ebi-a-GCST90001484 | TRUE | C | 98352281 | 2.72E-09 | reported | 3437 | 0.05766 | rs11595506 |  |  |  |  |  |  |  |  |  |  |  |  |  |  |  |
| -0.3232 | 10 | igd | 0.0495 | T | \|\| id:ebi-a-GCST90001485 | ebi-a-GCST90001485 | TRUE | C | 98352281 | 2.19E-08 | reported | 3437 | 0.05761 | rs11595506 |  |  |  |  |  |  |  |  |  |  |  |  |  |  |  |
| -0.3863 | 10 | igd | 0.0495 | T | \|\| id:ebi-a-GCST90001489 | ebi-a-GCST90001489 | TRUE | C | 98352281 | 1.08E-10 | reported | 3405 | 0.05964 | rs11595506 |  |  |  |  |  |  |  |  |  |  |  |  |  |  |  |
| -0.3801 | 10 | igd | 0.0495 | T | \|\| id:ebi-a-GCST90001491 | ebi-a-GCST90001491 | TRUE | C | 98352281 | 1.12E-10 | reported | 3437 | 0.05875 | rs11595506 |  |  |  |  |  |  |  |  |  |  |  |  |  |  |  |
| -0.4047 | 10 | igd | 0.0495 | T | \|\| id:ebi-a-GCST90001495 | ebi-a-GCST90001495 | TRUE | C | 98352281 | 2.00E-11 | reported | 3405 | 0.06014 | rs11595506 |  |  |  |  |  |  |  |  |  |  |  |  |  |  |  |
| -0.4178 | 10 | igd | 0.0495 | T | \|\| id:ebi-a-GCST90001496 | ebi-a-GCST90001496 | TRUE | C | 98352281 | 2.57E-12 | reported | 3437 | 0.05947 | rs11595506 |  |  |  |  |  |  |  |  |  |  |  |  |  |  |  |
| -0.4091 | 10 | igd | 0.0495 | T | \|\| id:ebi-a-GCST90001497 | ebi-a-GCST90001497 | TRUE | C | 98352281 | 3.88E-12 | reported | 3437 | 0.05873 | rs11595506 |  |  |  |  |  |  |  |  |  |  |  |  |  |  |  |
| -0.4082 | 2 | igd | 0.0495 | T | \|\| id:ebi-a-GCST90001891 | ebi-a-GCST90001891 | TRUE | C | 203905135 | 2.56E-10 | reported | 2920 | 0.06432 | rs112492006 |  |  |  |  |  |  |  |  |  |  |  |  |  |  |  |
| -0.3765 | 2 | igd | 0.0495 | T | \|\| id:ebi-a-GCST90001893 | ebi-a-GCST90001893 | TRUE | C | 203905135 | 7.12E-09 | reported | 2920 | 0.06485 | rs112492006 |  |  |  |  |  |  |  |  |  |  |  |  |  |  |  |
| -0.3331 | 11 | igd | 0.0495 | T | \|\| id:ebi-a-GCST90001976 | ebi-a-GCST90001976 | TRUE | C | 11413255 | 4.55E-08 | reported | 3113 | 0.06077 | rs146765347 |  |  |  |  |  |  |  |  |  |  |  |  |  |  |  |
| -0.3761 | 10 | igd | 0.0494 | T | \|\| id:ebi-a-GCST90001658 | ebi-a-GCST90001658 | TRUE | C | 98352281 | 3.97E-11 | reported | 3440 | 0.05676 | rs11595506 |  |  |  |  |  |  |  |  |  |  |  |  |  |  |  |
| -0.3837 | 10 | igd | 0.0494 | T | \|\| id:ebi-a-GCST90001659 | ebi-a-GCST90001659 | TRUE | C | 98352281 | 1.61E-11 | reported | 3440 | 0.05675 | rs11595506 |  |  |  |  |  |  |  |  |  |  |  |  |  |  |  |
| -0.4288 | 10 | igd | 0.0494 | T | \|\| id:ebi-a-GCST90001660 | ebi-a-GCST90001660 | TRUE | C | 98352281 | 6.13E-13 | reported | 3408 | 0.05935 | rs11595506 |  |  |  |  |  |  |  |  |  |  |  |  |  |  |  |
| -0.3344 | 10 | igd | 0.0494 | T | \|\| id:ebi-a-GCST90001672 | ebi-a-GCST90001672 | TRUE | C | 98352281 | 2.86E-08 | reported | 3408 | 0.06012 | rs11595506 |  |  |  |  |  |  |  |  |  |  |  |  |  |  |  |
| -0.338 | 10 | igd | 0.0492 | T | \|\| id:ebi-a-GCST90001489 | ebi-a-GCST90001489 | TRUE | C | 98019117 | 1.07E-08 | reported | 3405 | 0.05895 | rs1870170 |  |  |  |  |  |  |  |  |  |  |  |  |  |  |  |
| -0.3323 | 10 | igd | 0.0492 | T | \|\| id:ebi-a-GCST90001495 | ebi-a-GCST90001495 | TRUE | C | 98019117 | 2.41E-08 | reported | 3405 | 0.05941 | rs1870170 |  |  |  |  |  |  |  |  |  |  |  |  |  |  |  |
| -0.3222 | 22 | igd | 0.0492 | C | \|\| id:ebi-a-GCST90001702 | ebi-a-GCST90001702 | TRUE | T | 44449165 | 2.94E-08 | reported | 3657 | 0.05798 | rs145815892 |  |  |  |  |  |  |  |  |  |  |  |  |  |  |  |
| -0.3223 | 22 | igd | 0.0492 | C | \|\| id:ebi-a-GCST90001704 | ebi-a-GCST90001704 | TRUE | T | 44449165 | 2.78E-08 | reported | 3657 | 0.05789 | rs145815892 |  |  |  |  |  |  |  |  |  |  |  |  |  |  |  |
| -0.3704 | 10 | igd | 0.0491 | T | \|\| id:ebi-a-GCST90001660 | ebi-a-GCST90001660 | TRUE | C | 98019117 | 2.99E-10 | reported | 3408 | 0.05863 | rs1870170 |  |  |  |  |  |  |  |  |  |  |  |  |  |  |  |
| 0.4265 | 10 | igd | 0.0491 | C | \|\| id:ebi-a-GCST90001670 | ebi-a-GCST90001670 | TRUE | T | 92733574 | 1.92E-13 | reported | 3440 | 0.05776 | rs74472826 |  |  |  |  |  |  |  |  |  |  |  |  |  |  |  |
| 0.3941 | 10 | igd | 0.0491 | C | \|\| id:ebi-a-GCST90001671 | ebi-a-GCST90001671 | TRUE | T | 92733574 | 1.13E-11 | reported | 3440 | 0.05785 | rs74472826 |  |  |  |  |  |  |  |  |  |  |  |  |  |  |  |
| 0.4164 | 10 | igd | 0.0491 | C | \|\| id:ebi-a-GCST90001672 | ebi-a-GCST90001672 | TRUE | T | 92733574 | 1.72E-12 | reported | 3408 | 0.0588 | rs74472826 |  |  |  |  |  |  |  |  |  |  |  |  |  |  |  |
| 0.4996 | 6 | igd | 0.0491 | G | \|\| id:ebi-a-GCST90002108 | ebi-a-GCST90002108 | TRUE | A | 31788479 | 3.07E-08 | reported | 1579 | 0.08977 | rs62395828 |  |  |  |  |  |  |  |  |  |  |  |  |  |  |  |
| -0.3847 | 10 | igd | 0.049 | T | \|\| id:ebi-a-GCST90001658 | ebi-a-GCST90001658 | TRUE | C | 98019117 | 7.37E-12 | reported | 3440 | 0.05596 | rs1870170 |  |  |  |  |  |  |  |  |  |  |  |  |  |  |  |
| -0.3833 | 10 | igd | 0.049 | T | \|\| id:ebi-a-GCST90001659 | ebi-a-GCST90001659 | TRUE | C | 98019117 | 8.85E-12 | reported | 3440 | 0.05597 | rs1870170 |  |  |  |  |  |  |  |  |  |  |  |  |  |  |  |
| -0.3595 | 1 | igd | 0.049 | G | \|\| id:ebi-a-GCST90001845 | ebi-a-GCST90001845 | TRUE | C | 165218634 | 1.30E-08 | reported | 2911 | 0.06304 | rs4656440 |  |  |  |  |  |  |  |  |  |  |  |  |  |  |  |
| -0.3709 | 1 | igd | 0.049 | G | \|\| id:ebi-a-GCST90001858 | ebi-a-GCST90001858 | TRUE | C | 165218634 | 4.18E-09 | reported | 2919 | 0.06292 | rs4656440 |  |  |  |  |  |  |  |  |  |  |  |  |  |  |  |
| 0.3301 | 10 | igd | 0.0488 | T | \|\| id:ebi-a-GCST90001483 | ebi-a-GCST90001483 | TRUE | C | 98503103 | 1.02E-08 | reported | 3405 | 0.05749 | rs11188900 |  |  |  |  |  |  |  |  |  |  |  |  |  |  |  |
| -0.3302 | 10 | igd | 0.0488 | A | \|\| id:ebi-a-GCST90001671 | ebi-a-GCST90001671 | TRUE | G | 98015470 | 1.66E-08 | reported | 3440 | 0.05836 | rs117791247 |  |  |  |  |  |  |  |  |  |  |  |  |  |  |  |
| -0.433 | 10 | igd | 0.0488 | G | \|\| id:ebi-a-GCST90002030 | ebi-a-GCST90002030 | TRUE | T | 95745528 | 4.45E-12 | reported | 2920 | 0.06228 | rs76689584 |  |  |  |  |  |  |  |  |  |  |  |  |  |  |  |
| -0.4095 | 10 | igd | 0.0488 | G | \|\| id:ebi-a-GCST90002031 | ebi-a-GCST90002031 | TRUE | T | 95745528 | 1.98E-10 | reported | 2920 | 0.06414 | rs76689584 |  |  |  |  |  |  |  |  |  |  |  |  |  |  |  |
| -0.3707 | 10 | igd | 0.0488 | G | \|\| id:ebi-a-GCST90002032 | ebi-a-GCST90002032 | TRUE | T | 95745528 | 8.36E-09 | reported | 2920 | 0.06415 | rs76689584 |  |  |  |  |  |  |  |  |  |  |  |  |  |  |  |
| 0.5667 | 6 | igd | 0.0488 | A | \|\| id:ebi-a-GCST90002108 | ebi-a-GCST90002108 | TRUE | T | 30365155 | 7.39E-10 | reported | 1579 | 0.09147 | rs78774442 |  |  |  |  |  |  |  |  |  |  |  |  |  |  |  |
| 0.3718 | 10 | igd | 0.0487 | T | \|\| id:ebi-a-GCST90001484 | ebi-a-GCST90001484 | TRUE | C | 98503103 | 7.89E-11 | reported | 3437 | 0.057 | rs11188900 |  |  |  |  |  |  |  |  |  |  |  |  |  |  |  |
| 0.3261 | 10 | igd | 0.0487 | T | \|\| id:ebi-a-GCST90001485 | ebi-a-GCST90001485 | TRUE | C | 98503103 | 1.16E-08 | reported | 3437 | 0.057 | rs11188900 |  |  |  |  |  |  |  |  |  |  |  |  |  |  |  |
| 0.31 | 10 | igd | 0.0487 | T | \|\| id:ebi-a-GCST90001659 | ebi-a-GCST90001659 | TRUE | C | 98503103 | 3.85E-08 | reported | 3440 | 0.05625 | rs11188900 |  |  |  |  |  |  |  |  |  |  |  |  |  |  |  |
| 0.3393 | 10 | igd | 0.0487 | T | \|\| id:ebi-a-GCST90001660 | ebi-a-GCST90001660 | TRUE | C | 98503103 | 9.19E-09 | reported | 3408 | 0.05891 | rs11188900 |  |  |  |  |  |  |  |  |  |  |  |  |  |  |  |
| 0.3439 | 3 | igd | 0.0487 | T | \|\| id:ebi-a-GCST90001800 | ebi-a-GCST90001800 | TRUE | C | 30550515 | 7.46E-09 | reported | 3656 | 0.05935 | rs79587478 |  |  |  |  |  |  |  |  |  |  |  |  |  |  |  |
| -0.3794 | 1 | igd | 0.0486 | T | \|\| id:ebi-a-GCST90001989 | ebi-a-GCST90001989 | TRUE | C | 161688248 | 3.49E-10 | reported | 3621 | 0.06029 | rs578250948 |  |  |  |  |  |  |  |  |  |  |  |  |  |  |  |
| -0.3694 | 2 | igd | 0.0486 | T | \|\| id:ebi-a-GCST90002098 | ebi-a-GCST90002098 | TRUE | C | 38887803 | 1.17E-09 | reported | 2910 | 0.06052 | rs77612867 |  |  |  |  |  |  |  |  |  |  |  |  |  |  |  |
| 0.4207 | 6 | igd | 0.0486 | G | \|\| id:ebi-a-GCST90002105 | ebi-a-GCST90002105 | TRUE | A | 27766519 | 1.01E-10 | reported | 2872 | 0.06482 | rs62401408 |  |  |  |  |  |  |  |  |  |  |  |  |  |  |  |
| 0.6459 | 10 | igd | 0.0483 | A | \|\| id:ebi-a-GCST90001670 | ebi-a-GCST90001670 | TRUE | G | 95845989 | 1.13E-27 | reported | 3440 | 0.05873 | rs72812685 |  |  |  |  |  |  |  |  |  |  |  |  |  |  |  |
| 0.6238 | 10 | igd | 0.0483 | A | \|\| id:ebi-a-GCST90001671 | ebi-a-GCST90001671 | TRUE | G | 95845989 | 7.75E-26 | reported | 3440 | 0.05887 | rs72812685 |  |  |  |  |  |  |  |  |  |  |  |  |  |  |  |
| -0.4351 | 10 | igd | 0.0482 | C | \|\| id:ebi-a-GCST90001484 | ebi-a-GCST90001484 | TRUE | T | 97293322 | 9.82E-14 | reported | 3437 | 0.05821 | rs190517052 |  |  |  |  |  |  |  |  |  |  |  |  |  |  |  |
| -0.4783 | 10 | igd | 0.0482 | C | \|\| id:ebi-a-GCST90001489 | ebi-a-GCST90001489 | TRUE | T | 97293322 | 2.34E-15 | reported | 3405 | 0.0601 | rs190517052 |  |  |  |  |  |  |  |  |  |  |  |  |  |  |  |
| -0.4872 | 10 | igd | 0.0482 | C | \|\| id:ebi-a-GCST90001495 | ebi-a-GCST90001495 | TRUE | T | 97293322 | 1.10E-15 | reported | 3405 | 0.06049 | rs190517052 |  |  |  |  |  |  |  |  |  |  |  |  |  |  |  |
| -0.5132 | 10 | igd | 0.0482 | C | \|\| id:ebi-a-GCST90001496 | ebi-a-GCST90001496 | TRUE | T | 97293322 | 1.39E-17 | reported | 3437 | 0.0598 | rs190517052 |  |  |  |  |  |  |  |  |  |  |  |  |  |  |  |
| -0.5243 | 10 | igd | 0.0482 | C | \|\| id:ebi-a-GCST90001497 | ebi-a-GCST90001497 | TRUE | T | 97293322 | 9.96E-19 | reported | 3437 | 0.059 | rs190517052 |  |  |  |  |  |  |  |  |  |  |  |  |  |  |  |
| -0.337 | 10 | igd | 0.0481 | G | \|\| id:ebi-a-GCST90001658 | ebi-a-GCST90001658 | TRUE | T | 95745528 | 3.09E-09 | reported | 3440 | 0.05672 | rs76689584 |  |  |  |  |  |  |  |  |  |  |  |  |  |  |  |
| -0.3344 | 10 | igd | 0.0481 | G | \|\| id:ebi-a-GCST90001659 | ebi-a-GCST90001659 | TRUE | T | 95745528 | 4.14E-09 | reported | 3440 | 0.05673 | rs76689584 |  |  |  |  |  |  |  |  |  |  |  |  |  |  |  |
| -0.4852 | 10 | igd | 0.0481 | C | \|\| id:ebi-a-GCST90001660 | ebi-a-GCST90001660 | TRUE | T | 97293322 | 6.59E-16 | reported | 3408 | 0.05978 | rs190517052 |  |  |  |  |  |  |  |  |  |  |  |  |  |  |  |
| 0.3505 | 10 | igd | 0.0481 | T | \|\| id:ebi-a-GCST90001670 | ebi-a-GCST90001670 | TRUE | C | 95489318 | 1.33E-09 | reported | 3440 | 0.05763 | rs34364288 |  |  |  |  |  |  |  |  |  |  |  |  |  |  |  |
| -0.3294 | 10 | igd | 0.0481 | G | \|\| id:ebi-a-GCST90001671 | ebi-a-GCST90001671 | TRUE | T | 95745528 | 2.76E-08 | reported | 3440 | 0.05916 | rs76689584 |  |  |  |  |  |  |  |  |  |  |  |  |  |  |  |
| 0.3396 | 10 | igd | 0.0481 | T | \|\| id:ebi-a-GCST90001671 | ebi-a-GCST90001671 | TRUE | C | 95489318 | 4.31E-09 | reported | 3440 | 0.05769 | rs34364288 |  |  |  |  |  |  |  |  |  |  |  |  |  |  |  |
| 0.6785 | 10 | igd | 0.0481 | A | \|\| id:ebi-a-GCST90001672 | ebi-a-GCST90001672 | TRUE | G | 95845989 | 1.49E-29 | reported | 3408 | 0.05954 | rs72812685 |  |  |  |  |  |  |  |  |  |  |  |  |  |  |  |
| 0.3886 | 22 | igd | 0.0481 | A | \|\| id:ebi-a-GCST90001711 | ebi-a-GCST90001711 | TRUE | G | 42262411 | 7.27E-11 | reported | 3657 | 0.05947 | rs143256776 |  |  |  |  |  |  |  |  |  |  |  |  |  |  |  |
| -0.5714 | 10 | igd | 0.0481 | A | \|\| id:ebi-a-GCST90001961 | ebi-a-GCST90001961 | TRUE | G | 95845989 | 8.41E-19 | reported | 2920 | 0.0641 | rs72812685 |  |  |  |  |  |  |  |  |  |  |  |  |  |  |  |
| 0.4288 | 10 | igd | 0.0481 | A | \|\| id:ebi-a-GCST90002061 | ebi-a-GCST90002061 | TRUE | G | 95845989 | 4.72E-11 | reported | 2920 | 0.06492 | rs72812685 |  |  |  |  |  |  |  |  |  |  |  |  |  |  |  |
| -0.3716 | 10 | igd | 0.048 | G | \|\| id:ebi-a-GCST90001496 | ebi-a-GCST90001496 | TRUE | T | 95745528 | 4.83E-10 | reported | 3437 | 0.05952 | rs76689584 |  |  |  |  |  |  |  |  |  |  |  |  |  |  |  |
| -0.3492 | 10 | igd | 0.048 | G | \|\| id:ebi-a-GCST90001497 | ebi-a-GCST90001497 | TRUE | T | 95745528 | 3.16E-09 | reported | 3437 | 0.0588 | rs76689584 |  |  |  |  |  |  |  |  |  |  |  |  |  |  |  |
| -0.3636 | 22 | igd | 0.048 | T | \|\| id:ebi-a-GCST90001702 | ebi-a-GCST90001702 | TRUE | C | 44226266 | 4.45E-10 | reported | 3657 | 0.05813 | rs73176418 |  |  |  |  |  |  |  |  |  |  |  |  |  |  |  |
| -0.3675 | 22 | igd | 0.048 | T | \|\| id:ebi-a-GCST90001703 | ebi-a-GCST90001703 | TRUE | C | 44226266 | 2.88E-10 | reported | 3657 | 0.05812 | rs73176418 |  |  |  |  |  |  |  |  |  |  |  |  |  |  |  |
| -0.3934 | 22 | igd | 0.048 | T | \|\| id:ebi-a-GCST90001704 | ebi-a-GCST90001704 | TRUE | C | 44226266 | 1.36E-11 | reported | 3657 | 0.058 | rs73176418 |  |  |  |  |  |  |  |  |  |  |  |  |  |  |  |
| -0.3792 | 22 | igd | 0.048 | T | \|\| id:ebi-a-GCST90001710 | ebi-a-GCST90001710 | TRUE | C | 44226266 | 6.76E-11 | reported | 3657 | 0.05793 | rs73176418 |  |  |  |  |  |  |  |  |  |  |  |  |  |  |  |
| -0.3642 | 22 | igd | 0.048 | T | \|\| id:ebi-a-GCST90001711 | ebi-a-GCST90001711 | TRUE | C | 44226266 | 3.38E-10 | reported | 3657 | 0.05784 | rs73176418 |  |  |  |  |  |  |  |  |  |  |  |  |  |  |  |
| 0.4182 | 2 | igd | 0.0479 | T | \|\| id:ebi-a-GCST90001508 | ebi-a-GCST90001508 | TRUE | C | 39318970 | 1.98E-12 | reported | 3437 | 0.05922 | rs139649559 |  |  |  |  |  |  |  |  |  |  |  |  |  |  |  |
| 0.3815 | 2 | igd | 0.0479 | T | \|\| id:ebi-a-GCST90001509 | ebi-a-GCST90001509 | TRUE | C | 39318970 | 1.38E-10 | reported | 3437 | 0.05926 | rs139649559 |  |  |  |  |  |  |  |  |  |  |  |  |  |  |  |
| 0.4649 | 6 | igd | 0.0479 | T | \|\| id:ebi-a-GCST90002105 | ebi-a-GCST90002105 | TRUE | C | 28692424 | 4.26E-12 | reported | 2872 | 0.06681 | rs142877486 |  |  |  |  |  |  |  |  |  |  |  |  |  |  |  |
| 0.4059 | 6 | igd | 0.0479 | G | \|\| id:ebi-a-GCST90002105 | ebi-a-GCST90002105 | TRUE | A | 27964529 | 5.89E-10 | reported | 2872 | 0.06532 | rs62401423 |  |  |  |  |  |  |  |  |  |  |  |  |  |  |  |
| -0.5725 | 1 | igd | 0.0479 | C | \|\| id:ebi-a-GCST90002035 | ebi-a-GCST90002035 | TRUE | A | 92842367 | 8.24E-19 | reported | 2871 | 0.0642 | rs139795227 |  |  |  |  |  |  |  |  |  |  |  |  |  |  |  |
| -0.5867 | 1 | igd | 0.0479 | C | \|\| id:ebi-a-GCST90002036 | ebi-a-GCST90002036 | TRUE | A | 92842367 | 1.36E-19 | reported | 2871 | 0.06432 | rs139795227 |  |  |  |  |  |  |  |  |  |  |  |  |  |  |  |
| -0.4338 | 22 | igd | 0.0477 | T | \|\| id:ebi-a-GCST90001702 | ebi-a-GCST90001702 | TRUE | C | 40102614 | 1.18E-13 | reported | 3657 | 0.05825 | rs12484428 |  |  |  |  |  |  |  |  |  |  |  |  |  |  |  |
| -0.4353 | 22 | igd | 0.0477 | T | \|\| id:ebi-a-GCST90001703 | ebi-a-GCST90001703 | TRUE | C | 40102614 | 9.75E-14 | reported | 3657 | 0.05824 | rs12484428 |  |  |  |  |  |  |  |  |  |  |  |  |  |  |  |
| -0.4578 | 22 | igd | 0.0477 | T | \|\| id:ebi-a-GCST90001704 | ebi-a-GCST90001704 | TRUE | C | 40102614 | 4.40E-15 | reported | 3657 | 0.05812 | rs12484428 |  |  |  |  |  |  |  |  |  |  |  |  |  |  |  |
| -0.4066 | 22 | igd | 0.0477 | T | \|\| id:ebi-a-GCST90001710 | ebi-a-GCST90001710 | TRUE | C | 40102614 | 3.11E-12 | reported | 3657 | 0.05812 | rs12484428 |  |  |  |  |  |  |  |  |  |  |  |  |  |  |  |
| -0.4314 | 22 | igd | 0.0477 | T | \|\| id:ebi-a-GCST90001711 | ebi-a-GCST90001711 | TRUE | C | 40102614 | 1.22E-13 | reported | 3657 | 0.05796 | rs12484428 |  |  |  |  |  |  |  |  |  |  |  |  |  |  |  |
| 0.4966 | 1 | igd | 0.0476 | A | \|\| id:ebi-a-GCST90001854 | ebi-a-GCST90001854 | TRUE | G | 167422599 | 2.10E-15 | reported | 2919 | 0.06223 | rs114473227 |  |  |  |  |  |  |  |  |  |  |  |  |  |  |  |
| 0.458 | 1 | igd | 0.0476 | A | \|\| id:ebi-a-GCST90001856 | ebi-a-GCST90001856 | TRUE | G | 167422599 | 4.71E-13 | reported | 2919 | 0.06302 | rs114473227 |  |  |  |  |  |  |  |  |  |  |  |  |  |  |  |
| -0.7134 | 19 | igd | 0.0476 | A | \|\| id:ebi-a-GCST90001947 | ebi-a-GCST90001947 | TRUE | G | 51858911 | 1.04E-16 | reported | 1577 | 0.085 | rs558625031 |  |  |  |  |  |  |  |  |  |  |  |  |  |  |  |
| 0.4484 | 10 | igd | 0.0476 | T | \|\| id:ebi-a-GCST90002030 | ebi-a-GCST90002030 | TRUE | G | 97054903 | 2.73E-12 | reported | 2920 | 0.06387 | rs35540768 |  |  |  |  |  |  |  |  |  |  |  |  |  |  |  |
| 0.4151 | 10 | igd | 0.0476 | T | \|\| id:ebi-a-GCST90002032 | ebi-a-GCST90002032 | TRUE | G | 97054903 | 3.34E-10 | reported | 2920 | 0.06585 | rs35540768 |  |  |  |  |  |  |  |  |  |  |  |  |  |  |  |
| -0.7095 | 19 | igd | 0.0475 | A | \|\| id:ebi-a-GCST90001956 | ebi-a-GCST90001956 | TRUE | G | 51858911 | 1.33E-16 | reported | 1579 | 0.08484 | rs558625031 |  |  |  |  |  |  |  |  |  |  |  |  |  |  |  |
| -0.7209 | 19 | igd | 0.0475 | A | \|\| id:ebi-a-GCST90001957 | ebi-a-GCST90001957 | TRUE | G | 51858911 | 4.46E-17 | reported | 1578 | 0.08485 | rs558625031 |  |  |  |  |  |  |  |  |  |  |  |  |  |  |  |
| 0.3606 | 10 | igd | 0.0474 | A | \|\| id:ebi-a-GCST90001672 | ebi-a-GCST90001672 | TRUE | G | 98458051 | 4.46E-09 | reported | 3408 | 0.06132 | rs12266911 |  |  |  |  |  |  |  |  |  |  |  |  |  |  |  |
| -0.3591 | 22 | igd | 0.0473 | G | \|\| id:ebi-a-GCST90001702 | ebi-a-GCST90001702 | TRUE | A | 42928924 | 7.57E-10 | reported | 3657 | 0.0582 | rs56306647 |  |  |  |  |  |  |  |  |  |  |  |  |  |  |  |
| -0.3558 | 22 | igd | 0.0473 | G | \|\| id:ebi-a-GCST90001703 | ebi-a-GCST90001703 | TRUE | A | 42928924 | 1.09E-09 | reported | 3657 | 0.0582 | rs56306647 |  |  |  |  |  |  |  |  |  |  |  |  |  |  |  |
| -0.3628 | 22 | igd | 0.0473 | G | \|\| id:ebi-a-GCST90001704 | ebi-a-GCST90001704 | TRUE | A | 42928924 | 4.81E-10 | reported | 3657 | 0.05812 | rs56306647 |  |  |  |  |  |  |  |  |  |  |  |  |  |  |  |
| -0.3433 | 22 | igd | 0.0473 | G | \|\| id:ebi-a-GCST90001710 | ebi-a-GCST90001710 | TRUE | A | 42928924 | 3.63E-09 | reported | 3657 | 0.05804 | rs56306647 |  |  |  |  |  |  |  |  |  |  |  |  |  |  |  |
| -0.3603 | 22 | igd | 0.0473 | G | \|\| id:ebi-a-GCST90001711 | ebi-a-GCST90001711 | TRUE | A | 42928924 | 5.48E-10 | reported | 3657 | 0.05791 | rs56306647 |  |  |  |  |  |  |  |  |  |  |  |  |  |  |  |
| 0.3317 | 10 | igd | 0.0472 | A | \|\| id:ebi-a-GCST90001670 | ebi-a-GCST90001670 | TRUE | G | 98458051 | 4.23E-08 | reported | 3440 | 0.06038 | rs12266911 |  |  |  |  |  |  |  |  |  |  |  |  |  |  |  |
| 0.3349 | 10 | igd | 0.0472 | A | \|\| id:ebi-a-GCST90001671 | ebi-a-GCST90001671 | TRUE | G | 98458051 | 3.20E-08 | reported | 3440 | 0.06042 | rs12266911 |  |  |  |  |  |  |  |  |  |  |  |  |  |  |  |
| 0.4171 | 20 | igd | 0.0472 | T | \|\| id:ebi-a-GCST90001989 | ebi-a-GCST90001989 | TRUE | A | 44076835 | 2.33E-12 | reported | 3621 | 0.05926 | rs6073738 |  |  |  |  |  |  |  |  |  |  |  |  |  |  |  |
| 0.3712 | 2 | igd | 0.0471 | T | \|\| id:ebi-a-GCST90001507 | ebi-a-GCST90001507 | TRUE | C | 39318970 | 9.39E-10 | reported | 3405 | 0.06049 | rs139649559 |  |  |  |  |  |  |  |  |  |  |  |  |  |  |  |
| 0.3395 | 20 | igd | 0.0471 | T | \|\| id:ebi-a-GCST90001981 | ebi-a-GCST90001981 | TRUE | A | 44076835 | 9.68E-09 | reported | 3618 | 0.05905 | rs6073738 |  |  |  |  |  |  |  |  |  |  |  |  |  |  |  |
| 0.3543 | 20 | igd | 0.0471 | T | \|\| id:ebi-a-GCST90001985 | ebi-a-GCST90001985 | TRUE | A | 44076835 | 2.57E-09 | reported | 3629 | 0.05932 | rs6073738 |  |  |  |  |  |  |  |  |  |  |  |  |  |  |  |
| -0.3852 | 6 | igd | 0.047 | G | \|\| id:ebi-a-GCST90002105 | ebi-a-GCST90002105 | TRUE | C | 31239506 | 1.74E-08 | reported | 2872 | 0.06816 | rs1050414 |  |  |  |  |  |  |  |  |  |  |  |  |  |  |  |
| -0.3847 | 10 | igd | 0.0469 | G | \|\| id:ebi-a-GCST90002031 | ebi-a-GCST90002031 | TRUE | A | 97961102 | 4.67E-09 | reported | 2920 | 0.06547 | rs11593308 |  |  |  |  |  |  |  |  |  |  |  |  |  |  |  |
| -0.3738 | 22 | igd | 0.0468 | T | \|\| id:ebi-a-GCST90001702 | ebi-a-GCST90001702 | TRUE | C | 39641412 | 3.16E-10 | reported | 3657 | 0.05926 | rs13058590 |  |  |  |  |  |  |  |  |  |  |  |  |  |  |  |
| -0.3801 | 22 | igd | 0.0468 | T | \|\| id:ebi-a-GCST90001703 | ebi-a-GCST90001703 | TRUE | C | 39641412 | 1.58E-10 | reported | 3657 | 0.05924 | rs13058590 |  |  |  |  |  |  |  |  |  |  |  |  |  |  |  |
| -0.4122 | 22 | igd | 0.0468 | T | \|\| id:ebi-a-GCST90001704 | ebi-a-GCST90001704 | TRUE | C | 39641412 | 3.64E-12 | reported | 3657 | 0.0591 | rs13058590 |  |  |  |  |  |  |  |  |  |  |  |  |  |  |  |
| -0.355 | 22 | igd | 0.0468 | T | \|\| id:ebi-a-GCST90001710 | ebi-a-GCST90001710 | TRUE | C | 39641412 | 2.10E-09 | reported | 3657 | 0.05912 | rs13058590 |  |  |  |  |  |  |  |  |  |  |  |  |  |  |  |
| -0.3797 | 22 | igd | 0.0468 | T | \|\| id:ebi-a-GCST90001711 | ebi-a-GCST90001711 | TRUE | C | 39641412 | 1.33E-10 | reported | 3657 | 0.05894 | rs13058590 |  |  |  |  |  |  |  |  |  |  |  |  |  |  |  |
| 0.4371 | 6 | igd | 0.0466 | C | \|\| id:ebi-a-GCST90001896 | ebi-a-GCST90001896 | TRUE | T | 90847879 | 3.48E-11 | reported | 2920 | 0.06573 | rs117570580 |  |  |  |  |  |  |  |  |  |  |  |  |  |  |  |
| 0.3601 | 10 | igd | 0.0464 | T | \|\| id:ebi-a-GCST90001483 | ebi-a-GCST90001483 | TRUE | C | 98014850 | 3.22E-09 | reported | 3405 | 0.06066 | rs35909109 |  |  |  |  |  |  |  |  |  |  |  |  |  |  |  |
| 0.6555 | 10 | igd | 0.0464 | A | \|\| id:ebi-a-GCST90001484 | ebi-a-GCST90001484 | TRUE | G | 98160835 | 5.35E-29 | reported | 3437 | 0.05812 | rs77520132 |  |  |  |  |  |  |  |  |  |  |  |  |  |  |  |
| 0.4014 | 10 | igd | 0.0464 | T | \|\| id:ebi-a-GCST90001484 | ebi-a-GCST90001484 | TRUE | C | 98014850 | 2.54E-11 | reported | 3437 | 0.05998 | rs35909109 |  |  |  |  |  |  |  |  |  |  |  |  |  |  |  |
| 0.6067 | 10 | igd | 0.0464 | A | \|\| id:ebi-a-GCST90001485 | ebi-a-GCST90001485 | TRUE | G | 98160835 | 4.44E-25 | reported | 3437 | 0.05819 | rs77520132 |  |  |  |  |  |  |  |  |  |  |  |  |  |  |  |
| 0.4503 | 10 | igd | 0.0464 | T | \|\| id:ebi-a-GCST90001489 | ebi-a-GCST90001489 | TRUE | C | 98014850 | 5.37E-13 | reported | 3405 | 0.06216 | rs35909109 |  |  |  |  |  |  |  |  |  |  |  |  |  |  |  |
| 0.5205 | 10 | igd | 0.0464 | A | \|\| id:ebi-a-GCST90001491 | ebi-a-GCST90001491 | TRUE | G | 98160835 | 3.83E-18 | reported | 3437 | 0.05961 | rs77520132 |  |  |  |  |  |  |  |  |  |  |  |  |  |  |  |
| 0.4399 | 10 | igd | 0.0464 | T | \|\| id:ebi-a-GCST90001491 | ebi-a-GCST90001491 | TRUE | C | 98014850 | 7.81E-13 | reported | 3437 | 0.06116 | rs35909109 |  |  |  |  |  |  |  |  |  |  |  |  |  |  |  |
| 0.4781 | 10 | igd | 0.0464 | T | \|\| id:ebi-a-GCST90001495 | ebi-a-GCST90001495 | TRUE | C | 98014850 | 3.20E-14 | reported | 3405 | 0.06273 | rs35909109 |  |  |  |  |  |  |  |  |  |  |  |  |  |  |  |
| 0.6113 | 10 | igd | 0.0464 | A | \|\| id:ebi-a-GCST90001496 | ebi-a-GCST90001496 | TRUE | G | 98160835 | 6.62E-24 | reported | 3437 | 0.06018 | rs77520132 |  |  |  |  |  |  |  |  |  |  |  |  |  |  |  |
| 0.4493 | 10 | igd | 0.0464 | T | \|\| id:ebi-a-GCST90001496 | ebi-a-GCST90001496 | TRUE | C | 98014850 | 5.12E-13 | reported | 3437 | 0.06197 | rs35909109 |  |  |  |  |  |  |  |  |  |  |  |  |  |  |  |
| 0.4638 | 10 | igd | 0.0464 | T | \|\| id:ebi-a-GCST90001497 | ebi-a-GCST90001497 | TRUE | C | 98014850 | 4.30E-14 | reported | 3437 | 0.06115 | rs35909109 |  |  |  |  |  |  |  |  |  |  |  |  |  |  |  |
| 0.5656 | 10 | igd | 0.0464 | A | \|\| id:ebi-a-GCST90001497 | ebi-a-GCST90001497 | TRUE | G | 98160835 | 3.79E-21 | reported | 3437 | 0.05953 | rs77520132 |  |  |  |  |  |  |  |  |  |  |  |  |  |  |  |
| 0.6182 | 10 | igd | 0.0464 | A | \|\| id:ebi-a-GCST90001658 | ebi-a-GCST90001658 | TRUE | G | 98160835 | 9.15E-27 | reported | 3440 | 0.05724 | rs77520132 |  |  |  |  |  |  |  |  |  |  |  |  |  |  |  |
| 0.371 | 10 | igd | 0.0464 | T | \|\| id:ebi-a-GCST90001658 | ebi-a-GCST90001658 | TRUE | C | 98014850 | 4.19E-10 | reported | 3440 | 0.05922 | rs35909109 |  |  |  |  |  |  |  |  |  |  |  |  |  |  |  |
| 0.3751 | 10 | igd | 0.0464 | T | \|\| id:ebi-a-GCST90001659 | ebi-a-GCST90001659 | TRUE | C | 98014850 | 2.71E-10 | reported | 3440 | 0.05923 | rs35909109 |  |  |  |  |  |  |  |  |  |  |  |  |  |  |  |
| 0.6599 | 10 | igd | 0.0464 | A | \|\| id:ebi-a-GCST90001659 | ebi-a-GCST90001659 | TRUE | G | 98160835 | 2.49E-30 | reported | 3440 | 0.05711 | rs77520132 |  |  |  |  |  |  |  |  |  |  |  |  |  |  |  |
| 0.4134 | 10 | igd | 0.0464 | T | \|\| id:ebi-a-GCST90001660 | ebi-a-GCST90001660 | TRUE | C | 98014850 | 3.29E-11 | reported | 3408 | 0.06212 | rs35909109 |  |  |  |  |  |  |  |  |  |  |  |  |  |  |  |
| 0.5924 | 10 | igd | 0.0464 | A | \|\| id:ebi-a-GCST90001670 | ebi-a-GCST90001670 | TRUE | G | 98160835 | 7.27E-23 | reported | 3440 | 0.05975 | rs77520132 |  |  |  |  |  |  |  |  |  |  |  |  |  |  |  |
| 0.6245 | 10 | igd | 0.0464 | A | \|\| id:ebi-a-GCST90001671 | ebi-a-GCST90001671 | TRUE | G | 98160835 | 3.26E-25 | reported | 3440 | 0.05972 | rs77520132 |  |  |  |  |  |  |  |  |  |  |  |  |  |  |  |
| 0.5437 | 10 | igd | 0.0464 | T | \|\| id:ebi-a-GCST90001961 | ebi-a-GCST90001961 | TRUE | C | 97327197 | 5.37E-17 | reported | 2920 | 0.0645 | rs116766485 |  |  |  |  |  |  |  |  |  |  |  |  |  |  |  |
| -0.6718 | 10 | igd | 0.0464 | T | \|\| id:ebi-a-GCST90002030 | ebi-a-GCST90002030 | TRUE | C | 97327197 | 3.36E-25 | reported | 2920 | 0.06418 | rs116766485 |  |  |  |  |  |  |  |  |  |  |  |  |  |  |  |
| -0.5061 | 10 | igd | 0.0464 | T | \|\| id:ebi-a-GCST90002031 | ebi-a-GCST90002031 | TRUE | C | 97327197 | 1.55E-14 | reported | 2920 | 0.06554 | rs116766485 |  |  |  |  |  |  |  |  |  |  |  |  |  |  |  |
| -0.4719 | 10 | igd | 0.0464 | T | \|\| id:ebi-a-GCST90002032 | ebi-a-GCST90002032 | TRUE | C | 97327197 | 8.37E-13 | reported | 2920 | 0.06566 | rs116766485 |  |  |  |  |  |  |  |  |  |  |  |  |  |  |  |
| 0.5173 | 2 | igd | 0.0464 | T | \|\| id:ebi-a-GCST90002098 | ebi-a-GCST90002098 | TRUE | C | 39446592 | 2.95E-17 | reported | 2910 | 0.06085 | rs184177111 |  |  |  |  |  |  |  |  |  |  |  |  |  |  |  |
| 0.5163 | 2 | igd | 0.0464 | T | \|\| id:ebi-a-GCST90002102 | ebi-a-GCST90002102 | TRUE | C | 39446592 | 2.08E-17 | reported | 2919 | 0.06044 | rs184177111 |  |  |  |  |  |  |  |  |  |  |  |  |  |  |  |
| 0.3916 | 10 | igd | 0.0463 | C | \|\| id:ebi-a-GCST90001496 | ebi-a-GCST90001496 | TRUE | T | 95633120 | 4.28E-10 | reported | 3437 | 0.06254 | rs12778618 |  |  |  |  |  |  |  |  |  |  |  |  |  |  |  |
| 0.4055 | 10 | igd | 0.0463 | C | \|\| id:ebi-a-GCST90001497 | ebi-a-GCST90001497 | TRUE | T | 95633120 | 5.80E-11 | reported | 3437 | 0.06172 | rs12778618 |  |  |  |  |  |  |  |  |  |  |  |  |  |  |  |
| -0.3519 | 22 | igd | 0.0462 | G | \|\| id:ebi-a-GCST90001702 | ebi-a-GCST90001702 | TRUE | A | 44349795 | 2.53E-09 | reported | 3657 | 0.05891 | rs139290596 |  |  |  |  |  |  |  |  |  |  |  |  |  |  |  |
| -0.345 | 22 | igd | 0.0462 | G | \|\| id:ebi-a-GCST90001703 | ebi-a-GCST90001703 | TRUE | A | 44349795 | 5.17E-09 | reported | 3657 | 0.05891 | rs139290596 |  |  |  |  |  |  |  |  |  |  |  |  |  |  |  |
| -0.3635 | 22 | igd | 0.0462 | G | \|\| id:ebi-a-GCST90001704 | ebi-a-GCST90001704 | TRUE | A | 44349795 | 7.05E-10 | reported | 3657 | 0.05881 | rs139290596 |  |  |  |  |  |  |  |  |  |  |  |  |  |  |  |
| -0.331 | 22 | igd | 0.0462 | G | \|\| id:ebi-a-GCST90001710 | ebi-a-GCST90001710 | TRUE | A | 44349795 | 1.91E-08 | reported | 3657 | 0.05877 | rs139290596 |  |  |  |  |  |  |  |  |  |  |  |  |  |  |  |
| -0.3385 | 22 | igd | 0.0462 | G | \|\| id:ebi-a-GCST90001711 | ebi-a-GCST90001711 | TRUE | A | 44349795 | 8.42E-09 | reported | 3657 | 0.05863 | rs139290596 |  |  |  |  |  |  |  |  |  |  |  |  |  |  |  |
| 0.438 | 6 | igd | 0.0461 | T | \|\| id:ebi-a-GCST90002105 | ebi-a-GCST90002105 | TRUE | C | 27610598 | 5.51E-11 | reported | 2872 | 0.06655 | rs62401395 |  |  |  |  |  |  |  |  |  |  |  |  |  |  |  |
| 0.5828 | 10 | igd | 0.046 | A | \|\| id:ebi-a-GCST90001483 | ebi-a-GCST90001483 | TRUE | G | 98160835 | 1.03E-22 | reported | 3405 | 0.05899 | rs77520132 |  |  |  |  |  |  |  |  |  |  |  |  |  |  |  |
| 0.4584 | 10 | igd | 0.046 | A | \|\| id:ebi-a-GCST90001489 | ebi-a-GCST90001489 | TRUE | G | 98160835 | 6.95E-14 | reported | 3405 | 0.06095 | rs77520132 |  |  |  |  |  |  |  |  |  |  |  |  |  |  |  |
| 0.5125 | 10 | igd | 0.046 | A | \|\| id:ebi-a-GCST90001495 | ebi-a-GCST90001495 | TRUE | G | 98160835 | 9.27E-17 | reported | 3405 | 0.06133 | rs77520132 |  |  |  |  |  |  |  |  |  |  |  |  |  |  |  |
| -0.3752 | 10 | igd | 0.046 | G | \|\| id:ebi-a-GCST90001495 | ebi-a-GCST90001495 | TRUE | A | 97961102 | 9.36E-10 | reported | 3405 | 0.06113 | rs11593308 |  |  |  |  |  |  |  |  |  |  |  |  |  |  |  |
| -0.336 | 10 | igd | 0.046 | G | \|\| id:ebi-a-GCST90001496 | ebi-a-GCST90001496 | TRUE | A | 97961102 | 3.10E-08 | reported | 3437 | 0.06055 | rs11593308 |  |  |  |  |  |  |  |  |  |  |  |  |  |  |  |
| -0.3626 | 10 | igd | 0.046 | G | \|\| id:ebi-a-GCST90001497 | ebi-a-GCST90001497 | TRUE | A | 97961102 | 1.42E-09 | reported | 3437 | 0.05974 | rs11593308 |  |  |  |  |  |  |  |  |  |  |  |  |  |  |  |
| 0.4311 | 19 | igd | 0.04599 | A | \|\| id:prot-a-439 | prot-a-439 | TRUE | G | 51782966 | 1.58E-12 | reported | 3301 | 0.061 | rs61291939 |  |  |  |  |  |  |  |  |  |  |  |  |  |  |  |
| -0.3889 | 10 | igd | 0.0459 | G | \|\| id:ebi-a-GCST90001658 | ebi-a-GCST90001658 | TRUE | A | 97961102 | 1.79E-11 | reported | 3440 | 0.05765 | rs11593308 |  |  |  |  |  |  |  |  |  |  |  |  |  |  |  |
| -0.3816 | 10 | igd | 0.0459 | G | \|\| id:ebi-a-GCST90001659 | ebi-a-GCST90001659 | TRUE | A | 97961102 | 4.25E-11 | reported | 3440 | 0.05767 | rs11593308 |  |  |  |  |  |  |  |  |  |  |  |  |  |  |  |
| -0.4066 | 10 | igd | 0.0459 | G | \|\| id:ebi-a-GCST90001660 | ebi-a-GCST90001660 | TRUE | A | 97961102 | 1.84E-11 | reported | 3408 | 0.06032 | rs11593308 |  |  |  |  |  |  |  |  |  |  |  |  |  |  |  |
| 0.6312 | 10 | igd | 0.0459 | A | \|\| id:ebi-a-GCST90001660 | ebi-a-GCST90001660 | TRUE | G | 98160835 | 2.59E-25 | reported | 3408 | 0.06024 | rs77520132 |  |  |  |  |  |  |  |  |  |  |  |  |  |  |  |
| -0.4117 | 10 | igd | 0.0459 | G | \|\| id:ebi-a-GCST90001670 | ebi-a-GCST90001670 | TRUE | A | 97961102 | 8.28E-12 | reported | 3440 | 0.06003 | rs11593308 |  |  |  |  |  |  |  |  |  |  |  |  |  |  |  |
| -0.3801 | 10 | igd | 0.0459 | G | \|\| id:ebi-a-GCST90001671 | ebi-a-GCST90001671 | TRUE | A | 97961102 | 2.93E-10 | reported | 3440 | 0.06014 | rs11593308 |  |  |  |  |  |  |  |  |  |  |  |  |  |  |  |
| -0.4251 | 10 | igd | 0.0459 | G | \|\| id:ebi-a-GCST90001672 | ebi-a-GCST90001672 | TRUE | A | 97961102 | 3.48E-12 | reported | 3408 | 0.06088 | rs11593308 |  |  |  |  |  |  |  |  |  |  |  |  |  |  |  |
| 0.5899 | 10 | igd | 0.0459 | A | \|\| id:ebi-a-GCST90001672 | ebi-a-GCST90001672 | TRUE | G | 98160835 | 7.27E-22 | reported | 3408 | 0.06097 | rs77520132 |  |  |  |  |  |  |  |  |  |  |  |  |  |  |  |
| -0.3562 | 10 | igd | 0.0458 | T | \|\| id:ebi-a-GCST90001483 | ebi-a-GCST90001483 | TRUE | C | 97347231 | 1.15E-09 | reported | 3405 | 0.05835 | rs7903143 |  |  |  |  |  |  |  |  |  |  |  |  |  |  |  |
| -0.3875 | 1 | igd | 0.0458 | G | \|\| id:ebi-a-GCST90001989 | ebi-a-GCST90001989 | TRUE | A | 161353349 | 4.82E-10 | reported | 3621 | 0.06209 | rs142405270 |  |  |  |  |  |  |  |  |  |  |  |  |  |  |  |
| -0.496 | 10 | igd | 0.0457 | T | \|\| id:ebi-a-GCST90001484 | ebi-a-GCST90001484 | TRUE | C | 97327197 | 3.91E-17 | reported | 3437 | 0.05862 | rs116766485 |  |  |  |  |  |  |  |  |  |  |  |  |  |  |  |
| -0.3763 | 10 | igd | 0.0457 | T | \|\| id:ebi-a-GCST90001484 | ebi-a-GCST90001484 | TRUE | C | 97347231 | 9.99E-11 | reported | 3437 | 0.058 | rs7903143 |  |  |  |  |  |  |  |  |  |  |  |  |  |  |  |
| -0.457 | 10 | igd | 0.0457 | T | \|\| id:ebi-a-GCST90001485 | ebi-a-GCST90001485 | TRUE | C | 97327197 | 8.50E-15 | reported | 3437 | 0.05863 | rs116766485 |  |  |  |  |  |  |  |  |  |  |  |  |  |  |  |
| -0.4191 | 10 | igd | 0.0457 | A | \|\| id:ebi-a-GCST90001489 | ebi-a-GCST90001489 | TRUE | G | 97578380 | 5.24E-12 | reported | 3405 | 0.06053 | rs149263258 |  |  |  |  |  |  |  |  |  |  |  |  |  |  |  |
| -0.6122 | 10 | igd | 0.0457 | T | \|\| id:ebi-a-GCST90001491 | ebi-a-GCST90001491 | TRUE | C | 97327197 | 3.40E-24 | reported | 3437 | 0.05987 | rs116766485 |  |  |  |  |  |  |  |  |  |  |  |  |  |  |  |
| -0.4063 | 10 | igd | 0.0457 | A | \|\| id:ebi-a-GCST90001495 | ebi-a-GCST90001495 | TRUE | G | 97578380 | 3.06E-11 | reported | 3405 | 0.06095 | rs149263258 |  |  |  |  |  |  |  |  |  |  |  |  |  |  |  |
| -0.6465 | 10 | igd | 0.0457 | T | \|\| id:ebi-a-GCST90001496 | ebi-a-GCST90001496 | TRUE | C | 97327197 | 3.65E-26 | reported | 3437 | 0.0606 | rs116766485 |  |  |  |  |  |  |  |  |  |  |  |  |  |  |  |
| -0.6554 | 10 | igd | 0.0457 | T | \|\| id:ebi-a-GCST90001497 | ebi-a-GCST90001497 | TRUE | C | 97327197 | 1.63E-27 | reported | 3437 | 0.05978 | rs116766485 |  |  |  |  |  |  |  |  |  |  |  |  |  |  |  |
| 0.4215 | 10 | igd | 0.0457 | C | \|\| id:ebi-a-GCST90001888 | ebi-a-GCST90001888 | TRUE | T | 95633120 | 8.83E-11 | reported | 2920 | 0.06475 | rs12778618 |  |  |  |  |  |  |  |  |  |  |  |  |  |  |  |
| 0.5196 | 10 | igd | 0.0457 | C | \|\| id:ebi-a-GCST90001892 | ebi-a-GCST90001892 | TRUE | T | 95633120 | 4.44E-15 | reported | 2920 | 0.06591 | rs12778618 |  |  |  |  |  |  |  |  |  |  |  |  |  |  |  |
| -0.6486 | 10 | igd | 0.0457 | C | \|\| id:ebi-a-GCST90001961 | ebi-a-GCST90001961 | TRUE | T | 95633120 | 3.04E-22 | reported | 2920 | 0.06633 | rs12778618 |  |  |  |  |  |  |  |  |  |  |  |  |  |  |  |
| 0.4198 | 10 | igd | 0.0457 | C | \|\| id:ebi-a-GCST90002030 | ebi-a-GCST90002030 | TRUE | T | 95633120 | 4.21E-10 | reported | 2920 | 0.06698 | rs12778618 |  |  |  |  |  |  |  |  |  |  |  |  |  |  |  |
| -0.3811 | 10 | igd | 0.0457 | T | \|\| id:ebi-a-GCST90002031 | ebi-a-GCST90002031 | TRUE | C | 97822433 | 1.01E-08 | reported | 2920 | 0.06632 | rs72823717 |  |  |  |  |  |  |  |  |  |  |  |  |  |  |  |
| 0.5908 | 10 | igd | 0.0457 | C | \|\| id:ebi-a-GCST90002061 | ebi-a-GCST90002061 | TRUE | T | 95633120 | 1.47E-18 | reported | 2920 | 0.06675 | rs12778618 |  |  |  |  |  |  |  |  |  |  |  |  |  |  |  |
| -0.6454 | 10 | igd | 0.0456 | T | \|\| id:ebi-a-GCST90001658 | ebi-a-GCST90001658 | TRUE | C | 97327197 | 1.88E-28 | reported | 3440 | 0.05781 | rs116766485 |  |  |  |  |  |  |  |  |  |  |  |  |  |  |  |
| -0.6663 | 10 | igd | 0.0456 | T | \|\| id:ebi-a-GCST90001659 | ebi-a-GCST90001659 | TRUE | C | 97327197 | 3.06E-30 | reported | 3440 | 0.05775 | rs116766485 |  |  |  |  |  |  |  |  |  |  |  |  |  |  |  |
| -0.6335 | 10 | igd | 0.0456 | T | \|\| id:ebi-a-GCST90001670 | ebi-a-GCST90001670 | TRUE | C | 97327197 | 1.53E-25 | reported | 3440 | 0.06016 | rs116766485 |  |  |  |  |  |  |  |  |  |  |  |  |  |  |  |
| -0.6238 | 10 | igd | 0.0456 | T | \|\| id:ebi-a-GCST90001671 | ebi-a-GCST90001671 | TRUE | C | 97327197 | 1.24E-24 | reported | 3440 | 0.06041 | rs116766485 |  |  |  |  |  |  |  |  |  |  |  |  |  |  |  |
| -0.466 | 10 | igd | 0.0456 | A | \|\| id:ebi-a-GCST90001672 | ebi-a-GCST90001672 | TRUE | G | 97578380 | 2.00E-14 | reported | 3408 | 0.06065 | rs149263258 |  |  |  |  |  |  |  |  |  |  |  |  |  |  |  |
| 0.4483 | 6 | igd | 0.0456 | T | \|\| id:ebi-a-GCST90002105 | ebi-a-GCST90002105 | TRUE | A | 30576155 | 8.33E-09 | reported | 2872 | 0.07757 | rs58525263 |  |  |  |  |  |  |  |  |  |  |  |  |  |  |  |
| -0.4609 | 10 | igd | 0.0455 | T | \|\| id:ebi-a-GCST90001483 | ebi-a-GCST90001483 | TRUE | C | 97327197 | 1.07E-14 | reported | 3405 | 0.05936 | rs116766485 |  |  |  |  |  |  |  |  |  |  |  |  |  |  |  |
| -0.4209 | 10 | igd | 0.0455 | A | \|\| id:ebi-a-GCST90001489 | ebi-a-GCST90001489 | TRUE | G | 97468434 | 4.46E-12 | reported | 3405 | 0.06058 | rs147259256 |  |  |  |  |  |  |  |  |  |  |  |  |  |  |  |
| -0.609 | 10 | igd | 0.0455 | T | \|\| id:ebi-a-GCST90001489 | ebi-a-GCST90001489 | TRUE | C | 97327197 | 2.01E-23 | reported | 3405 | 0.06062 | rs116766485 |  |  |  |  |  |  |  |  |  |  |  |  |  |  |  |
| -0.6568 | 10 | igd | 0.0455 | T | \|\| id:ebi-a-GCST90001495 | ebi-a-GCST90001495 | TRUE | C | 97327197 | 2.34E-26 | reported | 3405 | 0.06131 | rs116766485 |  |  |  |  |  |  |  |  |  |  |  |  |  |  |  |
| -0.4078 | 10 | igd | 0.0455 | A | \|\| id:ebi-a-GCST90001495 | ebi-a-GCST90001495 | TRUE | G | 97468434 | 2.67E-11 | reported | 3405 | 0.061 | rs147259256 |  |  |  |  |  |  |  |  |  |  |  |  |  |  |  |
| -0.6995 | 10 | igd | 0.0455 | T | \|\| id:ebi-a-GCST90001660 | ebi-a-GCST90001660 | TRUE | C | 97327197 | 2.78E-30 | reported | 3408 | 0.06058 | rs116766485 |  |  |  |  |  |  |  |  |  |  |  |  |  |  |  |
| -0.672 | 10 | igd | 0.0455 | T | \|\| id:ebi-a-GCST90001672 | ebi-a-GCST90001672 | TRUE | C | 97327197 | 1.64E-27 | reported | 3408 | 0.06129 | rs116766485 |  |  |  |  |  |  |  |  |  |  |  |  |  |  |  |
| 0.6466 | 10 | igd | 0.0455 | T | \|\| id:ebi-a-GCST90001888 | ebi-a-GCST90001888 | TRUE | C | 97491578 | 2.29E-24 | reported | 2920 | 0.06291 | rs7073436 |  |  |  |  |  |  |  |  |  |  |  |  |  |  |  |
| 0.423 | 10 | igd | 0.0455 | T | \|\| id:ebi-a-GCST90001901 | ebi-a-GCST90001901 | TRUE | G | 97665383 | 8.05E-11 | reported | 2650 | 0.06481 | rs148473024 |  |  |  |  |  |  |  |  |  |  |  |  |  |  |  |
| 0.495 | 10 | igd | 0.0455 | A | \|\| id:ebi-a-GCST90002030 | ebi-a-GCST90002030 | TRUE | G | 97282450 | 6.98E-14 | reported | 2920 | 0.06578 | rs140938703 |  |  |  |  |  |  |  |  |  |  |  |  |  |  |  |
| 0.4627 | 10 | igd | 0.0455 | A | \|\| id:ebi-a-GCST90002031 | ebi-a-GCST90002031 | TRUE | G | 97282450 | 7.16E-12 | reported | 2920 | 0.06723 | rs140938703 |  |  |  |  |  |  |  |  |  |  |  |  |  |  |  |
| 0.5196 | 10 | igd | 0.0455 | A | \|\| id:ebi-a-GCST90002032 | ebi-a-GCST90002032 | TRUE | G | 97282450 | 1.30E-14 | reported | 2920 | 0.06708 | rs140938703 |  |  |  |  |  |  |  |  |  |  |  |  |  |  |  |
| 0.5601 | 10 | igd | 0.0455 | T | \|\| id:ebi-a-GCST90002069 | ebi-a-GCST90002069 | TRUE | C | 97491578 | 5.88E-18 | reported | 2920 | 0.06445 | rs7073436 |  |  |  |  |  |  |  |  |  |  |  |  |  |  |  |
| 0.3501 | 10 | igd | 0.0454 | C | \|\| id:ebi-a-GCST90001491 | ebi-a-GCST90001491 | TRUE | G | 95606287 | 1.87E-08 | reported | 3437 | 0.0621 | rs71480887 |  |  |  |  |  |  |  |  |  |  |  |  |  |  |  |
| -0.4465 | 10 | igd | 0.0454 | C | \|\| id:ebi-a-GCST90001491 | ebi-a-GCST90001491 | TRUE | T | 97486277 | 9.56E-14 | reported | 3437 | 0.05971 | rs138153402 |  |  |  |  |  |  |  |  |  |  |  |  |  |  |  |
| -0.3375 | 1 | igd | 0.0453 | G | \|\| id:ebi-a-GCST90001474 | ebi-a-GCST90001474 | TRUE | A | 161114862 | 1.99E-08 | reported | 3410 | 0.05999 | rs192316193 |  |  |  |  |  |  |  |  |  |  |  |  |  |  |  |
| 0.5327 | 10 | igd | 0.0453 | C | \|\| id:ebi-a-GCST90001658 | ebi-a-GCST90001658 | TRUE | G | 95606287 | 7.15E-19 | reported | 3440 | 0.05969 | rs71480887 |  |  |  |  |  |  |  |  |  |  |  |  |  |  |  |
| 0.5475 | 10 | igd | 0.0453 | C | \|\| id:ebi-a-GCST90001659 | ebi-a-GCST90001659 | TRUE | G | 95606287 | 7.53E-20 | reported | 3440 | 0.05966 | rs71480887 |  |  |  |  |  |  |  |  |  |  |  |  |  |  |  |
| 0.8406 | 10 | igd | 0.0453 | C | \|\| id:ebi-a-GCST90001670 | ebi-a-GCST90001670 | TRUE | G | 95606287 | 6.07E-42 | reported | 3440 | 0.06112 | rs71480887 |  |  |  |  |  |  |  |  |  |  |  |  |  |  |  |
| -0.4649 | 10 | igd | 0.0453 | A | \|\| id:ebi-a-GCST90001670 | ebi-a-GCST90001670 | TRUE | G | 97578380 | 1.04E-14 | reported | 3440 | 0.05985 | rs149263258 |  |  |  |  |  |  |  |  |  |  |  |  |  |  |  |
| 0.8275 | 10 | igd | 0.0453 | C | \|\| id:ebi-a-GCST90001671 | ebi-a-GCST90001671 | TRUE | G | 95606287 | 1.83E-40 | reported | 3440 | 0.06133 | rs71480887 |  |  |  |  |  |  |  |  |  |  |  |  |  |  |  |
| -0.4698 | 10 | igd | 0.0453 | A | \|\| id:ebi-a-GCST90001671 | ebi-a-GCST90001671 | TRUE | G | 97592929 | 5.77E-15 | reported | 3440 | 0.05989 | rs148539761 |  |  |  |  |  |  |  |  |  |  |  |  |  |  |  |
| -0.3497 | 1 | igd | 0.0453 | C | \|\| id:ebi-a-GCST90001989 | ebi-a-GCST90001989 | TRUE | T | 161221713 | 2.69E-08 | reported | 3621 | 0.06275 | rs148765873 |  |  |  |  |  |  |  |  |  |  |  |  |  |  |  |
| 0.4052 | 20 | igd | 0.0453 | T | \|\| id:ebi-a-GCST90001989 | ebi-a-GCST90001989 | TRUE | C | 44999746 | 2.62E-11 | reported | 3621 | 0.06059 | rs13037628 |  |  |  |  |  |  |  |  |  |  |  |  |  |  |  |
| 0.7604 | 6 | igd | 0.0453 | T | \|\| id:ebi-a-GCST90002108 | ebi-a-GCST90002108 | TRUE | C | 32575432 | 1.16E-15 | reported | 1579 | 0.09397 | rs113742050 |  |  |  |  |  |  |  |  |  |  |  |  |  |  |  |
| 0.5365 | 6 | igd | 0.0453 | G | \|\| id:ebi-a-GCST90002108 | ebi-a-GCST90002108 | TRUE | A | 29458241 | 2.36E-08 | reported | 1579 | 0.0956 | rs1233493 |  |  |  |  |  |  |  |  |  |  |  |  |  |  |  |
| 0.525 | 6 | igd | 0.0453 | C | \|\| id:ebi-a-GCST90002108 | ebi-a-GCST90002108 | TRUE | T | 30038647 | 4.65E-08 | reported | 1579 | 0.09562 | rs9261290 |  |  |  |  |  |  |  |  |  |  |  |  |  |  |  |
| 0.7466 | 6 | igd | 0.0453 | G | \|\| id:ebi-a-GCST90002109 | ebi-a-GCST90002109 | TRUE | A | 32574537 | 5.89E-11 | reported | 1578 | 0.1133 | rs539705259 |  |  |  |  |  |  |  |  |  |  |  |  |  |  |  |
| 0.6474 | 6 | igd | 0.0453 | C | \|\| id:ebi-a-GCST90002111 | ebi-a-GCST90002111 | TRUE | T | 32571079 | 6.71E-09 | reported | 1635 | 0.1111 | rs9279789 |  |  |  |  |  |  |  |  |  |  |  |  |  |  |  |
| 0.7114 | 6 | igd | 0.0453 | C | \|\| id:ebi-a-GCST90002112 | ebi-a-GCST90002112 | TRUE | T | 32571079 | 2.87E-10 | reported | 1635 | 0.1121 | rs9279789 |  |  |  |  |  |  |  |  |  |  |  |  |  |  |  |
| 0.3767 | 10 | igd | 0.0452 | C | \|\| id:ebi-a-GCST90001489 | ebi-a-GCST90001489 | TRUE | G | 95606287 | 2.22E-09 | reported | 3405 | 0.06282 | rs71480887 |  |  |  |  |  |  |  |  |  |  |  |  |  |  |  |
| 0.422 | 10 | igd | 0.0452 | C | \|\| id:ebi-a-GCST90001495 | ebi-a-GCST90001495 | TRUE | G | 95606287 | 3.38E-11 | reported | 3405 | 0.06345 | rs71480887 |  |  |  |  |  |  |  |  |  |  |  |  |  |  |  |
| -0.4157 | 10 | igd | 0.0452 | G | \|\| id:ebi-a-GCST90001497 | ebi-a-GCST90001497 | TRUE | A | 97475858 | 4.09E-12 | reported | 3437 | 0.05973 | rs144022496 |  |  |  |  |  |  |  |  |  |  |  |  |  |  |  |
| -0.4679 | 10 | igd | 0.0452 | A | \|\| id:ebi-a-GCST90001658 | ebi-a-GCST90001658 | TRUE | G | 97468434 | 5.19E-16 | reported | 3440 | 0.05743 | rs147259256 |  |  |  |  |  |  |  |  |  |  |  |  |  |  |  |
| 0.5975 | 10 | igd | 0.0452 | C | \|\| id:ebi-a-GCST90001660 | ebi-a-GCST90001660 | TRUE | G | 95606287 | 1.78E-21 | reported | 3408 | 0.06236 | rs71480887 |  |  |  |  |  |  |  |  |  |  |  |  |  |  |  |
| -0.4674 | 10 | igd | 0.0452 | C | \|\| id:ebi-a-GCST90001660 | ebi-a-GCST90001660 | TRUE | T | 97628567 | 1.18E-14 | reported | 3408 | 0.0603 | rs151023368 |  |  |  |  |  |  |  |  |  |  |  |  |  |  |  |
| 0.4901 | 10 | igd | 0.0452 | A | \|\| id:ebi-a-GCST90001660 | ebi-a-GCST90001660 | TRUE | G | 97282450 | 3.67E-15 | reported | 3408 | 0.06202 | rs140938703 |  |  |  |  |  |  |  |  |  |  |  |  |  |  |  |
| 0.8574 | 10 | igd | 0.0452 | C | \|\| id:ebi-a-GCST90001672 | ebi-a-GCST90001672 | TRUE | G | 95606287 | 3.16E-42 | reported | 3408 | 0.0621 | rs71480887 |  |  |  |  |  |  |  |  |  |  |  |  |  |  |  |
| 0.3451 | 20 | igd | 0.0452 | T | \|\| id:ebi-a-GCST90001981 | ebi-a-GCST90001981 | TRUE | C | 44999746 | 1.10E-08 | reported | 3618 | 0.06024 | rs13037628 |  |  |  |  |  |  |  |  |  |  |  |  |  |  |  |
| 0.369 | 20 | igd | 0.0452 | T | \|\| id:ebi-a-GCST90001985 | ebi-a-GCST90001985 | TRUE | C | 44999746 | 1.19E-09 | reported | 3629 | 0.06051 | rs13037628 |  |  |  |  |  |  |  |  |  |  |  |  |  |  |  |
| 0.361 | 10 | igd | 0.0451 | A | \|\| id:ebi-a-GCST90001483 | ebi-a-GCST90001483 | TRUE | G | 97282450 | 3.18E-09 | reported | 3405 | 0.06079 | rs140938703 |  |  |  |  |  |  |  |  |  |  |  |  |  |  |  |
| 0.4341 | 10 | igd | 0.0451 | A | \|\| id:ebi-a-GCST90001489 | ebi-a-GCST90001489 | TRUE | G | 97282450 | 3.93E-12 | reported | 3405 | 0.06233 | rs140938703 |  |  |  |  |  |  |  |  |  |  |  |  |  |  |  |
| 0.5273 | 10 | igd | 0.0451 | A | \|\| id:ebi-a-GCST90001495 | ebi-a-GCST90001495 | TRUE | G | 97282450 | 6.56E-17 | reported | 3405 | 0.06279 | rs140938703 |  |  |  |  |  |  |  |  |  |  |  |  |  |  |  |
| 0.4493 | 10 | igd | 0.0451 | A | \|\| id:ebi-a-GCST90001658 | ebi-a-GCST90001658 | TRUE | G | 97282450 | 4.03E-14 | reported | 3440 | 0.05918 | rs140938703 |  |  |  |  |  |  |  |  |  |  |  |  |  |  |  |
| 0.4689 | 10 | igd | 0.0451 | A | \|\| id:ebi-a-GCST90001659 | ebi-a-GCST90001659 | TRUE | G | 97282450 | 2.98E-15 | reported | 3440 | 0.05915 | rs140938703 |  |  |  |  |  |  |  |  |  |  |  |  |  |  |  |
| 0.3928 | 10 | igd | 0.045 | A | \|\| id:ebi-a-GCST90001484 | ebi-a-GCST90001484 | TRUE | G | 97282450 | 7.82E-11 | reported | 3437 | 0.0602 | rs140938703 |  |  |  |  |  |  |  |  |  |  |  |  |  |  |  |
| -0.3552 | 10 | igd | 0.045 | C | \|\| id:ebi-a-GCST90001484 | ebi-a-GCST90001484 | TRUE | T | 97628567 | 2.03E-09 | reported | 3437 | 0.05909 | rs151023368 |  |  |  |  |  |  |  |  |  |  |  |  |  |  |  |
| 0.3653 | 10 | igd | 0.045 | A | \|\| id:ebi-a-GCST90001485 | ebi-a-GCST90001485 | TRUE | G | 97282450 | 1.40E-09 | reported | 3437 | 0.06017 | rs140938703 |  |  |  |  |  |  |  |  |  |  |  |  |  |  |  |
| -0.3269 | 10 | igd | 0.045 | C | \|\| id:ebi-a-GCST90001485 | ebi-a-GCST90001485 | TRUE | T | 97628567 | 3.33E-08 | reported | 3437 | 0.05905 | rs151023368 |  |  |  |  |  |  |  |  |  |  |  |  |  |  |  |
| 0.3922 | 10 | igd | 0.045 | A | \|\| id:ebi-a-GCST90001491 | ebi-a-GCST90001491 | TRUE | G | 97282450 | 2.07E-10 | reported | 3437 | 0.06152 | rs140938703 |  |  |  |  |  |  |  |  |  |  |  |  |  |  |  |
| -0.4507 | 10 | igd | 0.045 | C | \|\| id:ebi-a-GCST90001491 | ebi-a-GCST90001491 | TRUE | T | 97628567 | 6.22E-14 | reported | 3437 | 0.05981 | rs151023368 |  |  |  |  |  |  |  |  |  |  |  |  |  |  |  |
| -0.4371 | 10 | igd | 0.045 | C | \|\| id:ebi-a-GCST90001496 | ebi-a-GCST90001496 | TRUE | T | 97628567 | 7.09E-13 | reported | 3437 | 0.06066 | rs151023368 |  |  |  |  |  |  |  |  |  |  |  |  |  |  |  |
| 0.4991 | 10 | igd | 0.045 | A | \|\| id:ebi-a-GCST90001496 | ebi-a-GCST90001496 | TRUE | G | 97282450 | 1.30E-15 | reported | 3437 | 0.06213 | rs140938703 |  |  |  |  |  |  |  |  |  |  |  |  |  |  |  |
| -0.4232 | 10 | igd | 0.045 | C | \|\| id:ebi-a-GCST90001497 | ebi-a-GCST90001497 | TRUE | T | 97628567 | 1.95E-12 | reported | 3437 | 0.05992 | rs151023368 |  |  |  |  |  |  |  |  |  |  |  |  |  |  |  |
| -0.3506 | 10 | igd | 0.045 | T | \|\| id:ebi-a-GCST90001497 | ebi-a-GCST90001497 | TRUE | C | 97822433 | 7.27E-09 | reported | 3437 | 0.06045 | rs72823717 |  |  |  |  |  |  |  |  |  |  |  |  |  |  |  |
| 0.4977 | 10 | igd | 0.045 | A | \|\| id:ebi-a-GCST90001497 | ebi-a-GCST90001497 | TRUE | G | 97282450 | 6.76E-16 | reported | 3437 | 0.06134 | rs140938703 |  |  |  |  |  |  |  |  |  |  |  |  |  |  |  |
| -0.4592 | 10 | igd | 0.045 | A | \|\| id:ebi-a-GCST90001660 | ebi-a-GCST90001660 | TRUE | G | 97498148 | 5.64E-14 | reported | 3408 | 0.06084 | rs146487261 |  |  |  |  |  |  |  |  |  |  |  |  |  |  |  |
| 0.527 | 6 | igd | 0.045 | T | \|\| id:ebi-a-GCST90002108 | ebi-a-GCST90002108 | TRUE | C | 31683443 | 2.08E-08 | reported | 1579 | 0.09355 | rs11575849 |  |  |  |  |  |  |  |  |  |  |  |  |  |  |  |
| -0.3718 | 10 | igd | 0.0449 | T | \|\| id:ebi-a-GCST90001658 | ebi-a-GCST90001658 | TRUE | C | 97822433 | 2.05E-10 | reported | 3440 | 0.05831 | rs72823717 |  |  |  |  |  |  |  |  |  |  |  |  |  |  |  |
| -0.4702 | 10 | igd | 0.0449 | C | \|\| id:ebi-a-GCST90001658 | ebi-a-GCST90001658 | TRUE | T | 97628567 | 4.62E-16 | reported | 3440 | 0.05762 | rs151023368 |  |  |  |  |  |  |  |  |  |  |  |  |  |  |  |
| -0.3651 | 10 | igd | 0.0449 | T | \|\| id:ebi-a-GCST90001659 | ebi-a-GCST90001659 | TRUE | C | 97822433 | 4.32E-10 | reported | 3440 | 0.05833 | rs72823717 |  |  |  |  |  |  |  |  |  |  |  |  |  |  |  |
| -0.4835 | 10 | igd | 0.0449 | C | \|\| id:ebi-a-GCST90001659 | ebi-a-GCST90001659 | TRUE | T | 97628567 | 6.67E-17 | reported | 3440 | 0.05759 | rs151023368 |  |  |  |  |  |  |  |  |  |  |  |  |  |  |  |
| -0.4155 | 10 | igd | 0.0449 | T | \|\| id:ebi-a-GCST90001670 | ebi-a-GCST90001670 | TRUE | C | 97822433 | 8.94E-12 | reported | 3440 | 0.06068 | rs72823717 |  |  |  |  |  |  |  |  |  |  |  |  |  |  |  |
| -0.3801 | 10 | igd | 0.0449 | T | \|\| id:ebi-a-GCST90001671 | ebi-a-GCST90001671 | TRUE | C | 97822433 | 4.51E-10 | reported | 3440 | 0.06079 | rs72823717 |  |  |  |  |  |  |  |  |  |  |  |  |  |  |  |
| 0.4307 | 10 | igd | 0.0449 | T | \|\| id:ebi-a-GCST90001901 | ebi-a-GCST90001901 | TRUE | G | 97328735 | 4.65E-11 | reported | 2650 | 0.06516 | rs73325155 |  |  |  |  |  |  |  |  |  |  |  |  |  |  |  |
| 0.4455 | 10 | igd | 0.0449 | A | \|\| id:ebi-a-GCST90001901 | ebi-a-GCST90001901 | TRUE | G | 97551952 | 9.29E-12 | reported | 2650 | 0.06505 | rs117653391 |  |  |  |  |  |  |  |  |  |  |  |  |  |  |  |
| 0.3987 | 10 | igd | 0.0449 | A | \|\| id:ebi-a-GCST90002031 | ebi-a-GCST90002031 | TRUE | G | 98155678 | 2.27E-09 | reported | 2920 | 0.0665 | rs41291628 |  |  |  |  |  |  |  |  |  |  |  |  |  |  |  |
| -0.3568 | 10 | igd | 0.0448 | A | \|\| id:ebi-a-GCST90001484 | ebi-a-GCST90001484 | TRUE | G | 97498148 | 2.31E-09 | reported | 3437 | 0.05956 | rs146487261 |  |  |  |  |  |  |  |  |  |  |  |  |  |  |  |
| -0.3269 | 10 | igd | 0.0448 | A | \|\| id:ebi-a-GCST90001485 | ebi-a-GCST90001485 | TRUE | G | 97498148 | 4.25E-08 | reported | 3437 | 0.05952 | rs146487261 |  |  |  |  |  |  |  |  |  |  |  |  |  |  |  |
| -0.4343 | 10 | igd | 0.0448 | A | \|\| id:ebi-a-GCST90001496 | ebi-a-GCST90001496 | TRUE | G | 97498148 | 1.53E-12 | reported | 3437 | 0.06118 | rs146487261 |  |  |  |  |  |  |  |  |  |  |  |  |  |  |  |
| -0.4788 | 10 | igd | 0.0448 | A | \|\| id:ebi-a-GCST90001659 | ebi-a-GCST90001659 | TRUE | G | 97498148 | 2.42E-16 | reported | 3440 | 0.0581 | rs146487261 |  |  |  |  |  |  |  |  |  |  |  |  |  |  |  |
| -0.4619 | 22 | igd | 0.0448 | A | \|\| id:ebi-a-GCST90001702 | ebi-a-GCST90001702 | TRUE | G | 43646647 | 3.52E-14 | reported | 3657 | 0.06072 | rs8140084 |  |  |  |  |  |  |  |  |  |  |  |  |  |  |  |
| -0.4523 | 22 | igd | 0.0448 | A | \|\| id:ebi-a-GCST90001703 | ebi-a-GCST90001703 | TRUE | G | 43646647 | 1.16E-13 | reported | 3657 | 0.06072 | rs8140084 |  |  |  |  |  |  |  |  |  |  |  |  |  |  |  |
| -0.3998 | 22 | igd | 0.0448 | A | \|\| id:ebi-a-GCST90001704 | ebi-a-GCST90001704 | TRUE | G | 43646647 | 5.24E-11 | reported | 3657 | 0.06072 | rs8140084 |  |  |  |  |  |  |  |  |  |  |  |  |  |  |  |
| -0.4567 | 22 | igd | 0.0448 | A | \|\| id:ebi-a-GCST90001710 | ebi-a-GCST90001710 | TRUE | G | 43646647 | 6.00E-14 | reported | 3657 | 0.06058 | rs8140084 |  |  |  |  |  |  |  |  |  |  |  |  |  |  |  |
| -0.4437 | 22 | igd | 0.0448 | A | \|\| id:ebi-a-GCST90001711 | ebi-a-GCST90001711 | TRUE | G | 43646647 | 2.55E-13 | reported | 3657 | 0.06042 | rs8140084 |  |  |  |  |  |  |  |  |  |  |  |  |  |  |  |
| -0.3749 | 10 | igd | 0.0447 | C | \|\| id:ebi-a-GCST90001660 | ebi-a-GCST90001660 | TRUE | A | 99181662 | 6.11E-09 | reported | 3408 | 0.06433 | rs7914103 |  |  |  |  |  |  |  |  |  |  |  |  |  |  |  |
| 0.7462 | 10 | igd | 0.0447 | C | \|\| id:ebi-a-GCST90001892 | ebi-a-GCST90001892 | TRUE | T | 97654138 | 6.92E-31 | reported | 2920 | 0.06383 | rs144084574 |  |  |  |  |  |  |  |  |  |  |  |  |  |  |  |
| -0.4543 | 10 | igd | 0.0447 | T | \|\| id:ebi-a-GCST90001961 | ebi-a-GCST90001961 | TRUE | C | 98155912 | 6.81E-12 | reported | 2920 | 0.06594 | rs76192559 |  |  |  |  |  |  |  |  |  |  |  |  |  |  |  |
| 0.3906 | 10 | igd | 0.0447 | G | \|\| id:ebi-a-GCST90002030 | ebi-a-GCST90002030 | TRUE | C | 97289836 | 2.74E-09 | reported | 2920 | 0.06548 | rs61869977 |  |  |  |  |  |  |  |  |  |  |  |  |  |  |  |
| -0.5088 | 12 | igd | 0.0447 | T | \|\| id:ebi-a-GCST90002073 | ebi-a-GCST90002073 | TRUE | C | 70068379 | 1.85E-14 | reported | 2850 | 0.06608 | rs77947327 |  |  |  |  |  |  |  |  |  |  |  |  |  |  |  |
| 0.517 | 6 | igd | 0.0447 | G | \|\| id:ebi-a-GCST90002105 | ebi-a-GCST90002105 | TRUE | A | 32315754 | 5.27E-14 | reported | 2872 | 0.06835 | rs80197836 |  |  |  |  |  |  |  |  |  |  |  |  |  |  |  |
| -0.3942 | 10 | igd | 0.0446 | C | \|\| id:ebi-a-GCST90001489 | ebi-a-GCST90001489 | TRUE | A | 99181662 | 1.17E-09 | reported | 3405 | 0.06462 | rs7914103 |  |  |  |  |  |  |  |  |  |  |  |  |  |  |  |
| -0.3575 | 10 | igd | 0.0446 | C | \|\| id:ebi-a-GCST90001495 | ebi-a-GCST90001495 | TRUE | A | 97800656 | 9.22E-09 | reported | 3405 | 0.06208 | rs75726774 |  |  |  |  |  |  |  |  |  |  |  |  |  |  |  |
| -0.3794 | 10 | igd | 0.0446 | C | \|\| id:ebi-a-GCST90001495 | ebi-a-GCST90001495 | TRUE | A | 99181662 | 6.32E-09 | reported | 3405 | 0.06515 | rs7914103 |  |  |  |  |  |  |  |  |  |  |  |  |  |  |  |
| -0.3924 | 10 | igd | 0.0446 | C | \|\| id:ebi-a-GCST90001660 | ebi-a-GCST90001660 | TRUE | A | 97800656 | 1.69E-10 | reported | 3408 | 0.06125 | rs75726774 |  |  |  |  |  |  |  |  |  |  |  |  |  |  |  |
| -0.4257 | 10 | igd | 0.0446 | C | \|\| id:ebi-a-GCST90001672 | ebi-a-GCST90001672 | TRUE | A | 97800656 | 6.68E-12 | reported | 3408 | 0.0618 | rs75726774 |  |  |  |  |  |  |  |  |  |  |  |  |  |  |  |
| 0.6154 | 6 | igd | 0.0446 | T | \|\| id:ebi-a-GCST90002108 | ebi-a-GCST90002108 | TRUE | C | 31897298 | 5.19E-11 | reported | 1579 | 0.09308 | rs9332708 |  |  |  |  |  |  |  |  |  |  |  |  |  |  |  |
| 0.397 | 10 | igd | 0.0445 | T | \|\| id:ebi-a-GCST90001658 | ebi-a-GCST90001658 | TRUE | C | 97491578 | 9.57E-12 | reported | 3440 | 0.05807 | rs7073436 |  |  |  |  |  |  |  |  |  |  |  |  |  |  |  |
| -0.4146 | 10 | igd | 0.0445 | C | \|\| id:ebi-a-GCST90001658 | ebi-a-GCST90001658 | TRUE | A | 99181662 | 1.64E-11 | reported | 3440 | 0.06135 | rs7914103 |  |  |  |  |  |  |  |  |  |  |  |  |  |  |  |
| 0.369 | 10 | igd | 0.0445 | T | \|\| id:ebi-a-GCST90001659 | ebi-a-GCST90001659 | TRUE | C | 97491578 | 2.48E-10 | reported | 3440 | 0.05813 | rs7073436 |  |  |  |  |  |  |  |  |  |  |  |  |  |  |  |
| -0.3984 | 10 | igd | 0.0445 | C | \|\| id:ebi-a-GCST90001659 | ebi-a-GCST90001659 | TRUE | A | 99181662 | 9.85E-11 | reported | 3440 | 0.06139 | rs7914103 |  |  |  |  |  |  |  |  |  |  |  |  |  |  |  |
| 0.5736 | 10 | igd | 0.0445 | A | \|\| id:ebi-a-GCST90001961 | ebi-a-GCST90001961 | TRUE | G | 97590928 | 6.06E-18 | reported | 2920 | 0.06603 | rs72815720 |  |  |  |  |  |  |  |  |  |  |  |  |  |  |  |
| 0.5171 | 10 | igd | 0.0445 | A | \|\| id:ebi-a-GCST90002030 | ebi-a-GCST90002030 | TRUE | G | 98160835 | 2.99E-15 | reported | 2920 | 0.06517 | rs77520132 |  |  |  |  |  |  |  |  |  |  |  |  |  |  |  |
| -0.6895 | 10 | igd | 0.0445 | A | \|\| id:ebi-a-GCST90002030 | ebi-a-GCST90002030 | TRUE | G | 97590928 | 3.12E-25 | reported | 2920 | 0.06582 | rs72815720 |  |  |  |  |  |  |  |  |  |  |  |  |  |  |  |
| -0.5096 | 10 | igd | 0.0445 | A | \|\| id:ebi-a-GCST90002031 | ebi-a-GCST90002031 | TRUE | G | 97590928 | 4.45E-14 | reported | 2920 | 0.06719 | rs72815720 |  |  |  |  |  |  |  |  |  |  |  |  |  |  |  |
| -0.486 | 10 | igd | 0.0445 | A | \|\| id:ebi-a-GCST90002032 | ebi-a-GCST90002032 | TRUE | G | 97507873 | 6.83E-13 | reported | 2920 | 0.06735 | rs191140605 |  |  |  |  |  |  |  |  |  |  |  |  |  |  |  |
| 0.3834 | 2 | igd | 0.0445 | A | \|\| id:ebi-a-GCST90002098 | ebi-a-GCST90002098 | TRUE | G | 38829737 | 2.79E-09 | reported | 2910 | 0.06431 | rs34376380 |  |  |  |  |  |  |  |  |  |  |  |  |  |  |  |
| 0.3445 | 2 | igd | 0.0444 | G | \|\| id:ebi-a-GCST90001417 | ebi-a-GCST90001417 | TRUE | T | 73868977 | 1.94E-08 | reported | 3657 | 0.0612 | rs72635970 |  |  |  |  |  |  |  |  |  |  |  |  |  |  |  |
| -0.3465 | 10 | igd | 0.0444 | T | \|\| id:ebi-a-GCST90001485 | ebi-a-GCST90001485 | TRUE | C | 97659192 | 4.80E-09 | reported | 3437 | 0.05904 | rs182106827 |  |  |  |  |  |  |  |  |  |  |  |  |  |  |  |
| -0.3995 | 10 | igd | 0.0444 | C | \|\| id:ebi-a-GCST90001491 | ebi-a-GCST90001491 | TRUE | A | 99181662 | 3.86E-10 | reported | 3437 | 0.06364 | rs7914103 |  |  |  |  |  |  |  |  |  |  |  |  |  |  |  |
| -0.3891 | 10 | igd | 0.0444 | C | \|\| id:ebi-a-GCST90001496 | ebi-a-GCST90001496 | TRUE | A | 99181662 | 1.80E-09 | reported | 3437 | 0.06452 | rs7914103 |  |  |  |  |  |  |  |  |  |  |  |  |  |  |  |
| -0.3792 | 10 | igd | 0.0444 | C | \|\| id:ebi-a-GCST90001497 | ebi-a-GCST90001497 | TRUE | A | 99181662 | 2.94E-09 | reported | 3437 | 0.06372 | rs7914103 |  |  |  |  |  |  |  |  |  |  |  |  |  |  |  |
| 0.4214 | 2 | igd | 0.0444 | C | \|\| id:ebi-a-GCST90001508 | ebi-a-GCST90001508 | TRUE | T | 39435251 | 9.39E-12 | reported | 3437 | 0.06162 | rs181028240 |  |  |  |  |  |  |  |  |  |  |  |  |  |  |  |
| 0.3835 | 2 | igd | 0.0444 | C | \|\| id:ebi-a-GCST90001509 | ebi-a-GCST90001509 | TRUE | T | 39435251 | 5.59E-10 | reported | 3437 | 0.06166 | rs181028240 |  |  |  |  |  |  |  |  |  |  |  |  |  |  |  |
| 0.4643 | 14 | igd | 0.0444 | C | \|\| id:ebi-a-GCST90001820 | ebi-a-GCST90001820 | TRUE | T | 106660850 | 1.89E-10 | reported | 3656 | 0.07268 | rs7157807 |  |  |  |  |  |  |  |  |  |  |  |  |  |  |  |
| 0.4426 | 14 | igd | 0.0444 | C | \|\| id:ebi-a-GCST90001821 | ebi-a-GCST90001821 | TRUE | T | 106660850 | 1.17E-09 | reported | 3656 | 0.07255 | rs7157807 |  |  |  |  |  |  |  |  |  |  |  |  |  |  |  |
| 0.4062 | 10 | igd | 0.0444 | C | \|\| id:ebi-a-GCST90002121 | ebi-a-GCST90002121 | TRUE | T | 97673734 | 5.24E-10 | reported | 2915 | 0.06516 | rs116997635 |  |  |  |  |  |  |  |  |  |  |  |  |  |  |  |
| 0.5675 | 10 | igd | 0.0443 | A | \|\| id:ebi-a-GCST90001961 | ebi-a-GCST90001961 | TRUE | C | 97703338 | 2.44E-17 | reported | 2920 | 0.06658 | rs72813659 |  |  |  |  |  |  |  |  |  |  |  |  |  |  |  |
| -0.9709 | 10 | igd | 0.0443 | G | \|\| id:ebi-a-GCST90001961 | ebi-a-GCST90001961 | TRUE | A | 97675936 | 4.47E-50 | reported | 2920 | 0.06401 | rs117847109 |  |  |  |  |  |  |  |  |  |  |  |  |  |  |  |
| -0.6869 | 10 | igd | 0.0443 | A | \|\| id:ebi-a-GCST90002030 | ebi-a-GCST90002030 | TRUE | C | 97703338 | 1.18E-24 | reported | 2920 | 0.06641 | rs72813659 |  |  |  |  |  |  |  |  |  |  |  |  |  |  |  |
| -0.5195 | 10 | igd | 0.0443 | A | \|\| id:ebi-a-GCST90002031 | ebi-a-GCST90002031 | TRUE | C | 97703338 | 2.30E-14 | reported | 2920 | 0.06772 | rs72813659 |  |  |  |  |  |  |  |  |  |  |  |  |  |  |  |
| -0.4808 | 10 | igd | 0.0443 | A | \|\| id:ebi-a-GCST90002032 | ebi-a-GCST90002032 | TRUE | C | 97703338 | 1.74E-12 | reported | 2920 | 0.06786 | rs72813659 |  |  |  |  |  |  |  |  |  |  |  |  |  |  |  |
| 0.5543 | 10 | igd | 0.0443 | C | \|\| id:ebi-a-GCST90002069 | ebi-a-GCST90002069 | TRUE | A | 97331197 | 2.46E-17 | reported | 2920 | 0.06504 | rs7086374 |  |  |  |  |  |  |  |  |  |  |  |  |  |  |  |
| -0.9604 | 10 | igd | 0.0442 | T | \|\| id:ebi-a-GCST90001961 | ebi-a-GCST90001961 | TRUE | C | 97323294 | 2.68E-49 | reported | 2920 | 0.06386 | rs151095424 |  |  |  |  |  |  |  |  |  |  |  |  |  |  |  |
| 0.3679 | 10 | igd | 0.0442 | C | \|\| id:ebi-a-GCST90002030 | ebi-a-GCST90002030 | TRUE | T | 98865039 | 4.22E-08 | reported | 2920 | 0.06693 | rs71486106 |  |  |  |  |  |  |  |  |  |  |  |  |  |  |  |
| -0.3845 | 1 | igd | 0.044 | T | \|\| id:ebi-a-GCST90001856 | ebi-a-GCST90001856 | TRUE | C | 167425929 | 6.80E-09 | reported | 2919 | 0.06614 | rs72693681 |  |  |  |  |  |  |  |  |  |  |  |  |  |  |  |
| -0.5051 | 1 | igd | 0.044 | A | \|\| id:ebi-a-GCST90001858 | ebi-a-GCST90001858 | TRUE | G | 167200909 | 1.13E-14 | reported | 2919 | 0.06506 | rs61814913 |  |  |  |  |  |  |  |  |  |  |  |  |  |  |  |
| -0.4833 | 1 | igd | 0.044 | T | \|\| id:ebi-a-GCST90001860 | ebi-a-GCST90001860 | TRUE | C | 167425929 | 3.48E-13 | reported | 2919 | 0.06614 | rs72693681 |  |  |  |  |  |  |  |  |  |  |  |  |  |  |  |
| -0.4113 | 1 | igd | 0.044 | A | \|\| id:ebi-a-GCST90001861 | ebi-a-GCST90001861 | TRUE | G | 167200909 | 2.30E-10 | reported | 2919 | 0.06465 | rs61814913 |  |  |  |  |  |  |  |  |  |  |  |  |  |  |  |
| -0.377 | 1 | igd | 0.044 | A | \|\| id:ebi-a-GCST90001863 | ebi-a-GCST90001863 | TRUE | G | 167200909 | 5.39E-09 | reported | 2919 | 0.06441 | rs61814913 |  |  |  |  |  |  |  |  |  |  |  |  |  |  |  |
| -0.4605 | 1 | igd | 0.044 | A | \|\| id:ebi-a-GCST90001864 | ebi-a-GCST90001864 | TRUE | G | 167200909 | 2.02E-12 | reported | 2919 | 0.06519 | rs61814913 |  |  |  |  |  |  |  |  |  |  |  |  |  |  |  |
| 1.071 | 10 | igd | 0.044 | T | \|\| id:ebi-a-GCST90002061 | ebi-a-GCST90002061 | TRUE | C | 97609885 | 3.59E-60 | reported | 2920 | 0.06395 | rs116024139 |  |  |  |  |  |  |  |  |  |  |  |  |  |  |  |
| 0.556 | 10 | igd | 0.044 | A | \|\| id:ebi-a-GCST90002069 | ebi-a-GCST90002069 | TRUE | G | 97598233 | 2.28E-17 | reported | 2920 | 0.06517 | rs117590671 |  |  |  |  |  |  |  |  |  |  |  |  |  |  |  |
| -0.5204 | 12 | igd | 0.0439 | T | \|\| id:ebi-a-GCST90002074 | ebi-a-GCST90002074 | TRUE | C | 70068379 | 4.56E-16 | reported | 3112 | 0.06372 | rs77947327 |  |  |  |  |  |  |  |  |  |  |  |  |  |  |  |
| 0.5265 | 6 | igd | 0.0439 | T | \|\| id:ebi-a-GCST90002105 | ebi-a-GCST90002105 | TRUE | C | 31897298 | 4.49E-12 | reported | 2872 | 0.07575 | rs9332708 |  |  |  |  |  |  |  |  |  |  |  |  |  |  |  |
| 0.3608 | 2 | igd | 0.0438 | C | \|\| id:ebi-a-GCST90001507 | ebi-a-GCST90001507 | TRUE | T | 39435251 | 1.09E-08 | reported | 3405 | 0.06295 | rs181028240 |  |  |  |  |  |  |  |  |  |  |  |  |  |  |  |
| 0.7719 | 10 | igd | 0.0438 | C | \|\| id:ebi-a-GCST90001892 | ebi-a-GCST90001892 | TRUE | T | 97392564 | 1.85E-32 | reported | 2920 | 0.06428 | rs117197936 |  |  |  |  |  |  |  |  |  |  |  |  |  |  |  |
| 0.4341 | 10 | igd | 0.0438 | A | \|\| id:ebi-a-GCST90001901 | ebi-a-GCST90001901 | TRUE | G | 97442176 | 7.20E-11 | reported | 2650 | 0.06634 | rs141293370 |  |  |  |  |  |  |  |  |  |  |  |  |  |  |  |
| -0.9945 | 10 | igd | 0.0438 | T | \|\| id:ebi-a-GCST90001961 | ebi-a-GCST90001961 | TRUE | C | 97575726 | 1.45E-52 | reported | 2920 | 0.06388 | rs114111042 |  |  |  |  |  |  |  |  |  |  |  |  |  |  |  |
| -0.4432 | 10 | igd | 0.0438 | C | \|\| id:ebi-a-GCST90002030 | ebi-a-GCST90002030 | TRUE | A | 99181662 | 1.98E-10 | reported | 2920 | 0.06942 | rs7914103 |  |  |  |  |  |  |  |  |  |  |  |  |  |  |  |
| -0.394 | 10 | igd | 0.0438 | C | \|\| id:ebi-a-GCST90002031 | ebi-a-GCST90002031 | TRUE | A | 99181662 | 3.33E-08 | reported | 2920 | 0.07115 | rs7914103 |  |  |  |  |  |  |  |  |  |  |  |  |  |  |  |
| 1.078 | 10 | igd | 0.0438 | C | \|\| id:ebi-a-GCST90002061 | ebi-a-GCST90002061 | TRUE | T | 97503951 | 3.62E-60 | reported | 2920 | 0.06435 | rs116678302 |  |  |  |  |  |  |  |  |  |  |  |  |  |  |  |
| 0.3995 | 10 | igd | 0.0437 | T | \|\| id:ebi-a-GCST90001670 | ebi-a-GCST90001670 | TRUE | C | 99490436 | 1.09E-10 | reported | 3440 | 0.06171 | rs72835969 |  |  |  |  |  |  |  |  |  |  |  |  |  |  |  |
| 0.3999 | 10 | igd | 0.0437 | T | \|\| id:ebi-a-GCST90001671 | ebi-a-GCST90001671 | TRUE | C | 99490436 | 1.10E-10 | reported | 3440 | 0.06178 | rs72835969 |  |  |  |  |  |  |  |  |  |  |  |  |  |  |  |
| 0.406 | 10 | igd | 0.0437 | A | \|\| id:ebi-a-GCST90001672 | ebi-a-GCST90001672 | TRUE | G | 99487288 | 1.09E-10 | reported | 3408 | 0.06272 | rs143977651 |  |  |  |  |  |  |  |  |  |  |  |  |  |  |  |
| 0.6337 | 10 | igd | 0.0437 | C | \|\| id:ebi-a-GCST90001888 | ebi-a-GCST90001888 | TRUE | A | 97385025 | 7.72E-23 | reported | 2920 | 0.06387 | rs115728778 |  |  |  |  |  |  |  |  |  |  |  |  |  |  |  |
| 0.7896 | 10 | igd | 0.0437 | A | \|\| id:ebi-a-GCST90001892 | ebi-a-GCST90001892 | TRUE | G | 97551952 | 1.57E-33 | reported | 2920 | 0.06461 | rs117653391 |  |  |  |  |  |  |  |  |  |  |  |  |  |  |  |
| 0.4791 | 6 | igd | 0.0437 | C | \|\| id:ebi-a-GCST90002105 | ebi-a-GCST90002105 | TRUE | T | 29049070 | 9.58E-12 | reported | 2872 | 0.07004 | rs115348723 |  |  |  |  |  |  |  |  |  |  |  |  |  |  |  |
| -0.3509 | 4 | igd | 0.0436 | A | \|\| id:ebi-a-GCST90001811 | ebi-a-GCST90001811 | TRUE | G | 15615074 | 1.68E-08 | reported | 3656 | 0.06204 | rs114591102 |  |  |  |  |  |  |  |  |  |  |  |  |  |  |  |
| -0.3641 | 4 | igd | 0.0436 | A | \|\| id:ebi-a-GCST90001815 | ebi-a-GCST90001815 | TRUE | G | 15615074 | 1.80E-09 | reported | 3657 | 0.06038 | rs114591102 |  |  |  |  |  |  |  |  |  |  |  |  |  |  |  |
| -0.4975 | 10 | igd | 0.0435 | A | \|\| id:ebi-a-GCST90001484 | ebi-a-GCST90001484 | TRUE | G | 97507873 | 2.39E-16 | reported | 3437 | 0.06036 | rs191140605 |  |  |  |  |  |  |  |  |  |  |  |  |  |  |  |
| -0.4547 | 10 | igd | 0.0435 | A | \|\| id:ebi-a-GCST90001485 | ebi-a-GCST90001485 | TRUE | G | 97507873 | 6.37E-14 | reported | 3437 | 0.06037 | rs191140605 |  |  |  |  |  |  |  |  |  |  |  |  |  |  |  |
| -0.44 | 10 | igd | 0.0435 | A | \|\| id:ebi-a-GCST90001489 | ebi-a-GCST90001489 | TRUE | G | 97680042 | 1.22E-12 | reported | 3405 | 0.06171 | rs141398625 |  |  |  |  |  |  |  |  |  |  |  |  |  |  |  |
| -0.6066 | 10 | igd | 0.0435 | A | \|\| id:ebi-a-GCST90001491 | ebi-a-GCST90001491 | TRUE | G | 97590928 | 1.44E-22 | reported | 3437 | 0.06162 | rs72815720 |  |  |  |  |  |  |  |  |  |  |  |  |  |  |  |
| 0.4319 | 10 | igd | 0.0435 | C | \|\| id:ebi-a-GCST90001495 | ebi-a-GCST90001495 | TRUE | A | 97192443 | 2.00E-11 | reported | 3405 | 0.06419 | rs116060550 |  |  |  |  |  |  |  |  |  |  |  |  |  |  |  |
| -0.4258 | 10 | igd | 0.0435 | A | \|\| id:ebi-a-GCST90001495 | ebi-a-GCST90001495 | TRUE | G | 97680042 | 8.51E-12 | reported | 3405 | 0.06212 | rs141398625 |  |  |  |  |  |  |  |  |  |  |  |  |  |  |  |
| -0.6435 | 10 | igd | 0.0435 | A | \|\| id:ebi-a-GCST90001496 | ebi-a-GCST90001496 | TRUE | G | 97590928 | 1.32E-24 | reported | 3437 | 0.06236 | rs72815720 |  |  |  |  |  |  |  |  |  |  |  |  |  |  |  |
| -0.6478 | 10 | igd | 0.0435 | A | \|\| id:ebi-a-GCST90001497 | ebi-a-GCST90001497 | TRUE | G | 97590928 | 1.59E-25 | reported | 3437 | 0.06154 | rs72815720 |  |  |  |  |  |  |  |  |  |  |  |  |  |  |  |
| -0.642 | 10 | igd | 0.0435 | A | \|\| id:ebi-a-GCST90001658 | ebi-a-GCST90001658 | TRUE | G | 97590928 | 1.06E-26 | reported | 3440 | 0.05952 | rs72815720 |  |  |  |  |  |  |  |  |  |  |  |  |  |  |  |
| -0.6589 | 10 | igd | 0.0435 | A | \|\| id:ebi-a-GCST90001659 | ebi-a-GCST90001659 | TRUE | G | 97590928 | 4.67E-28 | reported | 3440 | 0.05947 | rs72815720 |  |  |  |  |  |  |  |  |  |  |  |  |  |  |  |
| -0.6382 | 10 | igd | 0.0435 | A | \|\| id:ebi-a-GCST90001670 | ebi-a-GCST90001670 | TRUE | G | 97507873 | 1.61E-24 | reported | 3440 | 0.06197 | rs191140605 |  |  |  |  |  |  |  |  |  |  |  |  |  |  |  |
| -0.6367 | 10 | igd | 0.0435 | A | \|\| id:ebi-a-GCST90001671 | ebi-a-GCST90001671 | TRUE | G | 97507873 | 3.04E-24 | reported | 3440 | 0.06221 | rs191140605 |  |  |  |  |  |  |  |  |  |  |  |  |  |  |  |
| 0.6424 | 10 | igd | 0.0435 | T | \|\| id:ebi-a-GCST90001888 | ebi-a-GCST90001888 | TRUE | C | 97615096 | 2.32E-23 | reported | 2920 | 0.06395 | rs144991674 |  |  |  |  |  |  |  |  |  |  |  |  |  |  |  |
| -0.4981 | 10 | igd | 0.0435 | A | \|\| id:ebi-a-GCST90002031 | ebi-a-GCST90002031 | TRUE | G | 97537876 | 1.63E-13 | reported | 2920 | 0.06721 | rs117242046 |  |  |  |  |  |  |  |  |  |  |  |  |  |  |  |
| 0.5073 | 6 | igd | 0.0435 | T | \|\| id:ebi-a-GCST90002105 | ebi-a-GCST90002105 | TRUE | G | 28432725 | 7.10E-12 | reported | 2872 | 0.07368 | rs116098657 |  |  |  |  |  |  |  |  |  |  |  |  |  |  |  |
| -0.6355 | 10 | igd | 0.0434 | A | \|\| id:ebi-a-GCST90001496 | ebi-a-GCST90001496 | TRUE | C | 97703338 | 1.03E-23 | reported | 3437 | 0.06284 | rs72813659 |  |  |  |  |  |  |  |  |  |  |  |  |  |  |  |
| -0.6381 | 10 | igd | 0.0434 | A | \|\| id:ebi-a-GCST90001497 | ebi-a-GCST90001497 | TRUE | C | 97703338 | 1.77E-24 | reported | 3437 | 0.06202 | rs72813659 |  |  |  |  |  |  |  |  |  |  |  |  |  |  |  |
| 0.5191 | 10 | igd | 0.0434 | C | \|\| id:ebi-a-GCST90001660 | ebi-a-GCST90001660 | TRUE | T | 97191362 | 3.12E-16 | reported | 3408 | 0.06323 | rs73321243 |  |  |  |  |  |  |  |  |  |  |  |  |  |  |  |
| -0.4457 | 10 | igd | 0.0434 | A | \|\| id:ebi-a-GCST90001672 | ebi-a-GCST90001672 | TRUE | G | 97680042 | 7.38E-13 | reported | 3408 | 0.0619 | rs141398625 |  |  |  |  |  |  |  |  |  |  |  |  |  |  |  |
| 0.3722 | 10 | igd | 0.0434 | A | \|\| id:ebi-a-GCST90002065 | ebi-a-GCST90002065 | TRUE | G | 97548303 | 4.86E-08 | reported | 2650 | 0.06802 | rs147763768 |  |  |  |  |  |  |  |  |  |  |  |  |  |  |  |
| 0.5156 | 6 | igd | 0.0434 | C | \|\| id:ebi-a-GCST90002112 | ebi-a-GCST90002112 | TRUE | G | 30984145 | 1.51E-08 | reported | 1635 | 0.09063 | rs142977195 |  |  |  |  |  |  |  |  |  |  |  |  |  |  |  |
| -0.4635 | 10 | igd | 0.0433 | A | \|\| id:ebi-a-GCST90001483 | ebi-a-GCST90001483 | TRUE | G | 97590928 | 4.07E-14 | reported | 3405 | 0.06106 | rs72815720 |  |  |  |  |  |  |  |  |  |  |  |  |  |  |  |
| -0.5968 | 10 | igd | 0.0433 | A | \|\| id:ebi-a-GCST90001489 | ebi-a-GCST90001489 | TRUE | G | 97590928 | 2.17E-21 | reported | 3405 | 0.06242 | rs72815720 |  |  |  |  |  |  |  |  |  |  |  |  |  |  |  |
| -0.6472 | 10 | igd | 0.0433 | A | \|\| id:ebi-a-GCST90001495 | ebi-a-GCST90001495 | TRUE | G | 97590928 | 2.64E-24 | reported | 3405 | 0.06313 | rs72815720 |  |  |  |  |  |  |  |  |  |  |  |  |  |  |  |
| -0.6346 | 10 | igd | 0.0433 | A | \|\| id:ebi-a-GCST90001658 | ebi-a-GCST90001658 | TRUE | C | 97703338 | 9.04E-26 | reported | 3440 | 0.05998 | rs72813659 |  |  |  |  |  |  |  |  |  |  |  |  |  |  |  |
| -0.6528 | 10 | igd | 0.0433 | A | \|\| id:ebi-a-GCST90001659 | ebi-a-GCST90001659 | TRUE | C | 97703338 | 3.48E-27 | reported | 3440 | 0.05993 | rs72813659 |  |  |  |  |  |  |  |  |  |  |  |  |  |  |  |
| -0.6959 | 10 | igd | 0.0433 | A | \|\| id:ebi-a-GCST90001660 | ebi-a-GCST90001660 | TRUE | G | 97590928 | 2.04E-28 | reported | 3408 | 0.06238 | rs72815720 |  |  |  |  |  |  |  |  |  |  |  |  |  |  |  |
| 0.3567 | 10 | igd | 0.0433 | T | \|\| id:ebi-a-GCST90001660 | ebi-a-GCST90001660 | TRUE | C | 97645361 | 6.06E-09 | reported | 3408 | 0.06119 | rs143275760 |  |  |  |  |  |  |  |  |  |  |  |  |  |  |  |
| -0.6378 | 10 | igd | 0.0433 | A | \|\| id:ebi-a-GCST90001670 | ebi-a-GCST90001670 | TRUE | C | 97703338 | 3.27E-24 | reported | 3440 | 0.06235 | rs72813659 |  |  |  |  |  |  |  |  |  |  |  |  |  |  |  |
| -0.453 | 10 | igd | 0.0433 | T | \|\| id:ebi-a-GCST90001671 | ebi-a-GCST90001671 | TRUE | C | 97706698 | 1.78E-13 | reported | 3440 | 0.06126 | rs149030330 |  |  |  |  |  |  |  |  |  |  |  |  |  |  |  |
| -0.6359 | 10 | igd | 0.0433 | A | \|\| id:ebi-a-GCST90001671 | ebi-a-GCST90001671 | TRUE | C | 97703338 | 6.50E-24 | reported | 3440 | 0.06259 | rs72813659 |  |  |  |  |  |  |  |  |  |  |  |  |  |  |  |
| -0.6739 | 10 | igd | 0.0433 | A | \|\| id:ebi-a-GCST90001672 | ebi-a-GCST90001672 | TRUE | G | 97590928 | 3.18E-26 | reported | 3408 | 0.06308 | rs72815720 |  |  |  |  |  |  |  |  |  |  |  |  |  |  |  |
| 47.59 | 4 | igd | 0.0433 | T | \|\| id:ebi-a-GCST90001698 | ebi-a-GCST90001698 | TRUE | G | 180201786 | 4.66E-08 | reported | 3408 | 8.691 | rs79888370 |  |  |  |  |  |  |  |  |  |  |  |  |  |  |  |
| 0.4506 | 6 | igd | 0.0433 | T | \|\| id:ebi-a-GCST90001896 | ebi-a-GCST90001896 | TRUE | C | 91039169 | 3.54E-11 | reported | 2920 | 0.06778 | rs79295708 |  |  |  |  |  |  |  |  |  |  |  |  |  |  |  |
| 0.7104 | 6 | igd | 0.0433 | C | \|\| id:ebi-a-GCST90002105 | ebi-a-GCST90002105 | TRUE | G | 30984145 | 3.19E-24 | reported | 2872 | 0.06934 | rs142977195 |  |  |  |  |  |  |  |  |  |  |  |  |  |  |  |
| -0.4635 | 10 | igd | 0.0432 | A | \|\| id:ebi-a-GCST90001483 | ebi-a-GCST90001483 | TRUE | C | 97703338 | 6.15E-14 | reported | 3405 | 0.0615 | rs72813659 |  |  |  |  |  |  |  |  |  |  |  |  |  |  |  |
| -0.6354 | 10 | igd | 0.0432 | A | \|\| id:ebi-a-GCST90001495 | ebi-a-GCST90001495 | TRUE | C | 97703338 | 3.65E-23 | reported | 3405 | 0.06363 | rs72813659 |  |  |  |  |  |  |  |  |  |  |  |  |  |  |  |
| 0.3664 | 10 | igd | 0.0432 | T | \|\| id:ebi-a-GCST90001659 | ebi-a-GCST90001659 | TRUE | C | 97645361 | 4.14E-10 | reported | 3440 | 0.05847 | rs143275760 |  |  |  |  |  |  |  |  |  |  |  |  |  |  |  |
| -0.4428 | 10 | igd | 0.0432 | A | \|\| id:ebi-a-GCST90001670 | ebi-a-GCST90001670 | TRUE | G | 97680042 | 5.17E-13 | reported | 3440 | 0.06109 | rs141398625 |  |  |  |  |  |  |  |  |  |  |  |  |  |  |  |
| 0.4003 | 22 | igd | 0.0432 | C | \|\| id:ebi-a-GCST90001702 | ebi-a-GCST90001702 | TRUE | T | 41922643 | 3.88E-10 | reported | 3657 | 0.06378 | rs146824396 |  |  |  |  |  |  |  |  |  |  |  |  |  |  |  |
| 0.4073 | 22 | igd | 0.0432 | C | \|\| id:ebi-a-GCST90001703 | ebi-a-GCST90001703 | TRUE | T | 41922643 | 1.89E-10 | reported | 3657 | 0.06375 | rs146824396 |  |  |  |  |  |  |  |  |  |  |  |  |  |  |  |
| 0.3641 | 22 | igd | 0.0432 | C | \|\| id:ebi-a-GCST90001704 | ebi-a-GCST90001704 | TRUE | T | 41922643 | 1.20E-08 | reported | 3657 | 0.06373 | rs146824396 |  |  |  |  |  |  |  |  |  |  |  |  |  |  |  |
| 0.3663 | 22 | igd | 0.0432 | C | \|\| id:ebi-a-GCST90001710 | ebi-a-GCST90001710 | TRUE | T | 41922643 | 9.52E-09 | reported | 3657 | 0.06368 | rs146824396 |  |  |  |  |  |  |  |  |  |  |  |  |  |  |  |
| 0.4048 | 22 | igd | 0.0432 | C | \|\| id:ebi-a-GCST90001711 | ebi-a-GCST90001711 | TRUE | T | 41922643 | 1.96E-10 | reported | 3657 | 0.06343 | rs146824396 |  |  |  |  |  |  |  |  |  |  |  |  |  |  |  |
| -0.5483 | 10 | igd | 0.0432 | C | \|\| id:ebi-a-GCST90002032 | ebi-a-GCST90002032 | TRUE | T | 97486277 | 5.58E-16 | reported | 2920 | 0.06732 | rs138153402 |  |  |  |  |  |  |  |  |  |  |  |  |  |  |  |
| -0.5479 | 10 | igd | 0.0432 | T | \|\| id:ebi-a-GCST90002032 | ebi-a-GCST90002032 | TRUE | C | 97602846 | 5.66E-16 | reported | 2920 | 0.06729 | rs141191901 |  |  |  |  |  |  |  |  |  |  |  |  |  |  |  |
| 0.5645 | 2 | igd | 0.0432 | A | \|\| id:ebi-a-GCST90002102 | ebi-a-GCST90002102 | TRUE | G | 39604703 | 4.14E-19 | reported | 2919 | 0.06275 | rs116483429 |  |  |  |  |  |  |  |  |  |  |  |  |  |  |  |
| 0.5587 | 6 | igd | 0.0432 | T | \|\| id:ebi-a-GCST90002105 | ebi-a-GCST90002105 | TRUE | C | 32209312 | 2.10E-14 | reported | 2872 | 0.07271 | rs143548785 |  |  |  |  |  |  |  |  |  |  |  |  |  |  |  |
| -0.6863 | 10 | igd | 0.0431 | A | \|\| id:ebi-a-GCST90001660 | ebi-a-GCST90001660 | TRUE | C | 97703338 | 2.71E-27 | reported | 3408 | 0.06287 | rs72813659 |  |  |  |  |  |  |  |  |  |  |  |  |  |  |  |
| -0.6736 | 10 | igd | 0.0431 | A | \|\| id:ebi-a-GCST90001672 | ebi-a-GCST90001672 | TRUE | C | 97703338 | 7.53E-26 | reported | 3408 | 0.06355 | rs72813659 |  |  |  |  |  |  |  |  |  |  |  |  |  |  |  |
| 0.5565 | 2 | igd | 0.0431 | A | \|\| id:ebi-a-GCST90002098 | ebi-a-GCST90002098 | TRUE | G | 39604703 | 2.22E-18 | reported | 2910 | 0.06321 | rs116483429 |  |  |  |  |  |  |  |  |  |  |  |  |  |  |  |
| 0.6179 | 6 | igd | 0.0431 | A | \|\| id:ebi-a-GCST90002108 | ebi-a-GCST90002108 | TRUE | T | 30788541 | 3.37E-10 | reported | 1579 | 0.09774 | rs57933581 |  |  |  |  |  |  |  |  |  |  |  |  |  |  |  |
| 0.4048 | 1 | igd | 0.043 | G | \|\| id:ebi-a-GCST90001832 | ebi-a-GCST90001832 | TRUE | A | 169722490 | 3.74E-10 | reported | 2872 | 0.06439 | rs61805844 |  |  |  |  |  |  |  |  |  |  |  |  |  |  |  |
| -0.6757 | 10 | igd | 0.043 | T | \|\| id:ebi-a-GCST90001961 | ebi-a-GCST90001961 | TRUE | C | 97932006 | 5.86E-24 | reported | 2920 | 0.06635 | rs117592294 |  |  |  |  |  |  |  |  |  |  |  |  |  |  |  |
| 0.7843 | 10 | igd | 0.043 | T | \|\| id:ebi-a-GCST90002061 | ebi-a-GCST90002061 | TRUE | C | 97932006 | 1.93E-31 | reported | 2920 | 0.06645 | rs117592294 |  |  |  |  |  |  |  |  |  |  |  |  |  |  |  |
| 0.4236 | 10 | igd | 0.043 | T | \|\| id:ebi-a-GCST90002069 | ebi-a-GCST90002069 | TRUE | C | 97932006 | 2.37E-10 | reported | 2920 | 0.06663 | rs117592294 |  |  |  |  |  |  |  |  |  |  |  |  |  |  |  |
| 0.3488 | 10 | igd | 0.0429 | C | \|\| id:ebi-a-GCST90001489 | ebi-a-GCST90001489 | TRUE | T | 98865039 | 4.73E-08 | reported | 3405 | 0.06372 | rs71486106 |  |  |  |  |  |  |  |  |  |  |  |  |  |  |  |
| 0.404 | 22 | igd | 0.0429 | A | \|\| id:ebi-a-GCST90001702 | ebi-a-GCST90001702 | TRUE | G | 41808162 | 3.31E-10 | reported | 3657 | 0.06412 | rs183413369 |  |  |  |  |  |  |  |  |  |  |  |  |  |  |  |
| 0.4117 | 22 | igd | 0.0429 | A | \|\| id:ebi-a-GCST90001703 | ebi-a-GCST90001703 | TRUE | G | 41808162 | 1.50E-10 | reported | 3657 | 0.06409 | rs183413369 |  |  |  |  |  |  |  |  |  |  |  |  |  |  |  |
| 0.3654 | 22 | igd | 0.0429 | A | \|\| id:ebi-a-GCST90001704 | ebi-a-GCST90001704 | TRUE | G | 41808162 | 1.27E-08 | reported | 3657 | 0.06407 | rs183413369 |  |  |  |  |  |  |  |  |  |  |  |  |  |  |  |
| 0.369 | 22 | igd | 0.0429 | A | \|\| id:ebi-a-GCST90001710 | ebi-a-GCST90001710 | TRUE | G | 41808162 | 8.85E-09 | reported | 3657 | 0.06402 | rs183413369 |  |  |  |  |  |  |  |  |  |  |  |  |  |  |  |
| 0.4072 | 22 | igd | 0.0429 | A | \|\| id:ebi-a-GCST90001711 | ebi-a-GCST90001711 | TRUE | G | 41808162 | 1.91E-10 | reported | 3657 | 0.06376 | rs183413369 |  |  |  |  |  |  |  |  |  |  |  |  |  |  |  |
| -0.3697 | 10 | igd | 0.0428 | G | \|\| id:ebi-a-GCST90001672 | ebi-a-GCST90001672 | TRUE | A | 94521742 | 6.50E-09 | reported | 3408 | 0.06355 | rs56046082 |  |  |  |  |  |  |  |  |  |  |  |  |  |  |  |
| 0.3773 | 6 | igd | 0.0428 | T | \|\| id:ebi-a-GCST90001820 | ebi-a-GCST90001820 | TRUE | G | 42975310 | 7.44E-10 | reported | 3656 | 0.06112 | rs141794401 |  |  |  |  |  |  |  |  |  |  |  |  |  |  |  |
| -1.022 | 10 | igd | 0.0428 | A | \|\| id:ebi-a-GCST90001961 | ebi-a-GCST90001961 | TRUE | G | 97442176 | 3.92E-53 | reported | 2920 | 0.06524 | rs141293370 |  |  |  |  |  |  |  |  |  |  |  |  |  |  |  |
| -0.6211 | 10 | igd | 0.0428 | C | \|\| id:ebi-a-GCST90002030 | ebi-a-GCST90002030 | TRUE | T | 97628567 | 4.95E-21 | reported | 2920 | 0.0655 | rs151023368 |  |  |  |  |  |  |  |  |  |  |  |  |  |  |  |
| -0.6225 | 10 | igd | 0.0428 | A | \|\| id:ebi-a-GCST90002030 | ebi-a-GCST90002030 | TRUE | G | 97498148 | 4.65E-21 | reported | 2920 | 0.0656 | rs146487261 |  |  |  |  |  |  |  |  |  |  |  |  |  |  |  |
| 0.4724 | 6 | igd | 0.0428 | T | \|\| id:ebi-a-GCST90002105 | ebi-a-GCST90002105 | TRUE | C | 31127037 | 2.08E-11 | reported | 2872 | 0.07022 | rs17190769 |  |  |  |  |  |  |  |  |  |  |  |  |  |  |  |
| 0.3595 | 20 | igd | 0.0427 | A | \|\| id:ebi-a-GCST90001981 | ebi-a-GCST90001981 | TRUE | G | 45318042 | 1.03E-08 | reported | 3618 | 0.06265 | rs2664523 |  |  |  |  |  |  |  |  |  |  |  |  |  |  |  |
| 0.4686 | 1 | igd | 0.0427 | T | \|\| id:ebi-a-GCST90001989 | ebi-a-GCST90001989 | TRUE | G | 161452866 | 2.64E-14 | reported | 3621 | 0.06129 | rs71639066 |  |  |  |  |  |  |  |  |  |  |  |  |  |  |  |
| 0.5455 | 6 | igd | 0.0427 | A | \|\| id:ebi-a-GCST90002105 | ebi-a-GCST90002105 | TRUE | G | 31930063 | 3.20E-14 | reported | 2872 | 0.0715 | rs115521166 |  |  |  |  |  |  |  |  |  |  |  |  |  |  |  |
| 0.4352 | 10 | igd | 0.0427 | A | \|\| id:ebi-a-GCST90002121 | ebi-a-GCST90002121 | TRUE | C | 97403248 | 7.57E-11 | reported | 2915 | 0.06661 | rs117519014 |  |  |  |  |  |  |  |  |  |  |  |  |  |  |  |
| 0.4357 | 10 | igd | 0.0427 | A | \|\| id:ebi-a-GCST90002121 | ebi-a-GCST90002121 | TRUE | G | 97569453 | 8.22E-11 | reported | 2915 | 0.06682 | rs142279413 |  |  |  |  |  |  |  |  |  |  |  |  |  |  |  |
| 0.3912 | 19 | igd | 0.04266 | G | \|\| id:prot-a-439 | prot-a-439 | TRUE | T | 51716784 | 7.94E-10 | reported | 3301 | 0.0637 | rs78901355 |  |  |  |  |  |  |  |  |  |  |  |  |  |  |  |
| -0.3442 | 10 | igd | 0.0426 | G | \|\| id:ebi-a-GCST90001671 | ebi-a-GCST90001671 | TRUE | A | 94521742 | 4.45E-08 | reported | 3440 | 0.06276 | rs56046082 |  |  |  |  |  |  |  |  |  |  |  |  |  |  |  |
| 0.3457 | 20 | igd | 0.0426 | A | \|\| id:ebi-a-GCST90001985 | ebi-a-GCST90001985 | TRUE | G | 45318042 | 4.39E-08 | reported | 3629 | 0.06302 | rs2664523 |  |  |  |  |  |  |  |  |  |  |  |  |  |  |  |
| 1.087 | 10 | igd | 0.0426 | C | \|\| id:ebi-a-GCST90002061 | ebi-a-GCST90002061 | TRUE | A | 97403216 | 7.57E-60 | reported | 2920 | 0.06513 | rs117958583 |  |  |  |  |  |  |  |  |  |  |  |  |  |  |  |
| -0.6166 | 2 | igd | 0.0426 | C | \|\| id:ebi-a-GCST90002119 | ebi-a-GCST90002119 | TRUE | A | 87008667 | 3.43E-20 | reported | 2920 | 0.0665 | rs115623211 |  |  |  |  |  |  |  |  |  |  |  |  |  |  |  |
| -0.5185 | 10 | igd | 0.0425 | A | \|\| id:ebi-a-GCST90001484 | ebi-a-GCST90001484 | TRUE | G | 97682867 | 6.74E-17 | reported | 3437 | 0.06177 | rs147991810 |  |  |  |  |  |  |  |  |  |  |  |  |  |  |  |
| -0.3326 | 10 | igd | 0.0425 | T | \|\| id:ebi-a-GCST90001484 | ebi-a-GCST90001484 | TRUE | C | 97574531 | 3.09E-08 | reported | 3437 | 0.05993 | rs117501902 |  |  |  |  |  |  |  |  |  |  |  |  |  |  |  |
| -0.3278 | 10 | igd | 0.0425 | G | \|\| id:ebi-a-GCST90001485 | ebi-a-GCST90001485 | TRUE | T | 97551132 | 4.79E-08 | reported | 3437 | 0.05991 | rs118020459 |  |  |  |  |  |  |  |  |  |  |  |  |  |  |  |
| -0.479 | 10 | igd | 0.0425 | A | \|\| id:ebi-a-GCST90001485 | ebi-a-GCST90001485 | TRUE | G | 97682867 | 1.16E-14 | reported | 3437 | 0.06177 | rs147991810 |  |  |  |  |  |  |  |  |  |  |  |  |  |  |  |
| 0.3605 | 10 | igd | 0.0425 | G | \|\| id:ebi-a-GCST90001660 | ebi-a-GCST90001660 | TRUE | C | 97474665 | 5.24E-09 | reported | 3408 | 0.06158 | rs115186884 |  |  |  |  |  |  |  |  |  |  |  |  |  |  |  |
| 1.192 | 10 | igd | 0.0425 | T | \|\| id:ebi-a-GCST90001672 | ebi-a-GCST90001672 | TRUE | C | 97609885 | 6.96E-85 | reported | 3408 | 0.05935 | rs116024139 |  |  |  |  |  |  |  |  |  |  |  |  |  |  |  |
| 1.191 | 10 | igd | 0.0425 | G | \|\| id:ebi-a-GCST90001672 | ebi-a-GCST90001672 | TRUE | C | 97474665 | 1.57E-85 | reported | 3408 | 0.05907 | rs115186884 |  |  |  |  |  |  |  |  |  |  |  |  |  |  |  |
| -0.3586 | 22 | igd | 0.0425 | T | \|\| id:ebi-a-GCST90001710 | ebi-a-GCST90001710 | TRUE | G | 42886952 | 6.43E-09 | reported | 3657 | 0.06162 | rs5758758 |  |  |  |  |  |  |  |  |  |  |  |  |  |  |  |
| -0.4081 | 11 | igd | 0.0425 | T | \|\| id:ebi-a-GCST90002071 | ebi-a-GCST90002071 | TRUE | C | 5488177 | 1.27E-08 | reported | 2872 | 0.07152 | rs80297185 |  |  |  |  |  |  |  |  |  |  |  |  |  |  |  |
| 0.5084 | 6 | igd | 0.0425 | A | \|\| id:ebi-a-GCST90002112 | ebi-a-GCST90002112 | TRUE | G | 32200235 | 4.72E-08 | reported | 1635 | 0.09264 | rs76161886 |  |  |  |  |  |  |  |  |  |  |  |  |  |  |  |
| 0.3944 | 10 | igd | 0.0424 | T | \|\| id:ebi-a-GCST90001658 | ebi-a-GCST90001658 | TRUE | C | 97615688 | 2.52E-11 | reported | 3440 | 0.05892 | rs138791076 |  |  |  |  |  |  |  |  |  |  |  |  |  |  |  |
| 1.259 | 10 | igd | 0.0424 | T | \|\| id:ebi-a-GCST90001670 | ebi-a-GCST90001670 | TRUE | C | 97615688 | 2.20E-98 | reported | 3440 | 0.05787 | rs138791076 |  |  |  |  |  |  |  |  |  |  |  |  |  |  |  |
| 1.261 | 10 | igd | 0.0424 | G | \|\| id:ebi-a-GCST90001670 | ebi-a-GCST90001670 | TRUE | C | 97474665 | 3.55E-99 | reported | 3440 | 0.05771 | rs115186884 |  |  |  |  |  |  |  |  |  |  |  |  |  |  |  |
| 1.211 | 10 | igd | 0.0424 | G | \|\| id:ebi-a-GCST90001671 | ebi-a-GCST90001671 | TRUE | C | 97474665 | 7.34E-91 | reported | 3440 | 0.05811 | rs115186884 |  |  |  |  |  |  |  |  |  |  |  |  |  |  |  |
| 1.21 | 10 | igd | 0.0424 | T | \|\| id:ebi-a-GCST90001671 | ebi-a-GCST90001671 | TRUE | C | 97615688 | 2.71E-90 | reported | 3440 | 0.05826 | rs138791076 |  |  |  |  |  |  |  |  |  |  |  |  |  |  |  |
| 0.8747 | 10 | igd | 0.0424 | T | \|\| id:ebi-a-GCST90001672 | ebi-a-GCST90001672 | TRUE | C | 97932006 | 1.14E-44 | reported | 3408 | 0.06147 | rs117592294 |  |  |  |  |  |  |  |  |  |  |  |  |  |  |  |
| 0.6845 | 6 | igd | 0.0424 | T | \|\| id:ebi-a-GCST90002108 | ebi-a-GCST90002108 | TRUE | G | 30589094 | 2.32E-10 | reported | 1579 | 0.1073 | rs183138129 |  |  |  |  |  |  |  |  |  |  |  |  |  |  |  |
| 0.9089 | 10 | igd | 0.0423 | T | \|\| id:ebi-a-GCST90001670 | ebi-a-GCST90001670 | TRUE | C | 97932006 | 1.11E-49 | reported | 3440 | 0.06036 | rs117592294 |  |  |  |  |  |  |  |  |  |  |  |  |  |  |  |
| 0.8553 | 10 | igd | 0.0423 | T | \|\| id:ebi-a-GCST90001671 | ebi-a-GCST90001671 | TRUE | C | 97932006 | 6.45E-44 | reported | 3440 | 0.06067 | rs117592294 |  |  |  |  |  |  |  |  |  |  |  |  |  |  |  |
| 0.3527 | 2 | igd | 0.0421 | G | \|\| id:ebi-a-GCST90001417 | ebi-a-GCST90001417 | TRUE | A | 73679013 | 1.71E-08 | reported | 3657 | 0.0624 | rs45608038 |  |  |  |  |  |  |  |  |  |  |  |  |  |  |  |
| 0.3422 | 10 | igd | 0.0421 | A | \|\| id:ebi-a-GCST90001660 | ebi-a-GCST90001660 | TRUE | G | 97373754 | 3.83E-08 | reported | 3408 | 0.0621 | rs11541780 |  |  |  |  |  |  |  |  |  |  |  |  |  |  |  |
| 0.4958 | 10 | igd | 0.0421 | C | \|\| id:ebi-a-GCST90001672 | ebi-a-GCST90001672 | TRUE | G | 97599537 | 9.94E-15 | reported | 3408 | 0.06377 | rs61731067 |  |  |  |  |  |  |  |  |  |  |  |  |  |  |  |
| 1.174 | 10 | igd | 0.0421 | A | \|\| id:ebi-a-GCST90001672 | ebi-a-GCST90001672 | TRUE | G | 97373754 | 1.23E-81 | reported | 3408 | 0.05969 | rs11541780 |  |  |  |  |  |  |  |  |  |  |  |  |  |  |  |
| 0.4965 | 10 | igd | 0.0421 | G | \|\| id:ebi-a-GCST90001672 | ebi-a-GCST90001672 | TRUE | C | 97495608 | 9.83E-15 | reported | 3408 | 0.06385 | rs11188461 |  |  |  |  |  |  |  |  |  |  |  |  |  |  |  |
| 0.4079 | 1 | igd | 0.0421 | T | \|\| id:ebi-a-GCST90001858 | ebi-a-GCST90001858 | TRUE | A | 167614149 | 9.35E-10 | reported | 2919 | 0.06643 | rs147807927 |  |  |  |  |  |  |  |  |  |  |  |  |  |  |  |
| 0.6483 | 10 | igd | 0.0421 | G | \|\| id:ebi-a-GCST90002031 | ebi-a-GCST90002031 | TRUE | A | 97617784 | 6.79E-21 | reported | 2920 | 0.06861 | rs12355114 |  |  |  |  |  |  |  |  |  |  |  |  |  |  |  |
| -0.6109 | 10 | igd | 0.042 | C | \|\| id:ebi-a-GCST90001491 | ebi-a-GCST90001491 | TRUE | T | 97716816 | 6.93E-22 | reported | 3437 | 0.06311 | rs72813666 |  |  |  |  |  |  |  |  |  |  |  |  |  |  |  |
| 0.3781 | 10 | igd | 0.042 | C | \|\| id:ebi-a-GCST90001658 | ebi-a-GCST90001658 | TRUE | A | 97385025 | 2.14E-10 | reported | 3440 | 0.05936 | rs115728778 |  |  |  |  |  |  |  |  |  |  |  |  |  |  |  |
| 0.353 | 10 | igd | 0.042 | C | \|\| id:ebi-a-GCST90001659 | ebi-a-GCST90001659 | TRUE | A | 97385025 | 3.11E-09 | reported | 3440 | 0.05941 | rs115728778 |  |  |  |  |  |  |  |  |  |  |  |  |  |  |  |
| 1.242 | 10 | igd | 0.042 | A | \|\| id:ebi-a-GCST90001670 | ebi-a-GCST90001670 | TRUE | G | 97373754 | 1.39E-94 | reported | 3440 | 0.05833 | rs11541780 |  |  |  |  |  |  |  |  |  |  |  |  |  |  |  |
| 1.19 | 10 | igd | 0.042 | A | \|\| id:ebi-a-GCST90001671 | ebi-a-GCST90001671 | TRUE | G | 97373754 | 3.32E-86 | reported | 3440 | 0.05875 | rs11541780 |  |  |  |  |  |  |  |  |  |  |  |  |  |  |  |
| -0.3921 | 10 | igd | 0.042 | A | \|\| id:ebi-a-GCST90001961 | ebi-a-GCST90001961 | TRUE | G | 97573405 | 8.54E-09 | reported | 2920 | 0.06791 | rs60144080 |  |  |  |  |  |  |  |  |  |  |  |  |  |  |  |
| 0.6936 | 10 | igd | 0.042 | T | \|\| id:ebi-a-GCST90002030 | ebi-a-GCST90002030 | TRUE | C | 97569492 | 1.36E-24 | reported | 2920 | 0.06715 | rs61503377 |  |  |  |  |  |  |  |  |  |  |  |  |  |  |  |
| 0.6394 | 10 | igd | 0.042 | A | \|\| id:ebi-a-GCST90002031 | ebi-a-GCST90002031 | TRUE | G | 97484659 | 1.38E-20 | reported | 2920 | 0.06823 | rs11188451 |  |  |  |  |  |  |  |  |  |  |  |  |  |  |  |
| 0.6152 | 10 | igd | 0.042 | T | \|\| id:ebi-a-GCST90002032 | ebi-a-GCST90002032 | TRUE | C | 97569492 | 4.26E-19 | reported | 2920 | 0.06841 | rs61503377 |  |  |  |  |  |  |  |  |  |  |  |  |  |  |  |
| 0.5138 | 10 | igd | 0.0419 | A | \|\| id:ebi-a-GCST90001483 | ebi-a-GCST90001483 | TRUE | C | 97513247 | 1.27E-16 | reported | 3405 | 0.06176 | rs41291576 |  |  |  |  |  |  |  |  |  |  |  |  |  |  |  |
| -0.6026 | 10 | igd | 0.0419 | C | \|\| id:ebi-a-GCST90001489 | ebi-a-GCST90001489 | TRUE | T | 97716816 | 7.74E-21 | reported | 3405 | 0.06394 | rs72813666 |  |  |  |  |  |  |  |  |  |  |  |  |  |  |  |
| 0.4835 | 10 | igd | 0.0419 | G | \|\| id:ebi-a-GCST90001491 | ebi-a-GCST90001491 | TRUE | C | 97495608 | 1.23E-14 | reported | 3437 | 0.06241 | rs11188461 |  |  |  |  |  |  |  |  |  |  |  |  |  |  |  |
| 0.5539 | 10 | igd | 0.0419 | A | \|\| id:ebi-a-GCST90001495 | ebi-a-GCST90001495 | TRUE | C | 97513247 | 8.24E-18 | reported | 3405 | 0.06408 | rs41291576 |  |  |  |  |  |  |  |  |  |  |  |  |  |  |  |
| 0.5314 | 10 | igd | 0.0419 | G | \|\| id:ebi-a-GCST90001496 | ebi-a-GCST90001496 | TRUE | C | 97495608 | 5.64E-17 | reported | 3437 | 0.06315 | rs11188461 |  |  |  |  |  |  |  |  |  |  |  |  |  |  |  |
| 0.5404 | 10 | igd | 0.0419 | G | \|\| id:ebi-a-GCST90001497 | ebi-a-GCST90001497 | TRUE | C | 97495608 | 6.44E-18 | reported | 3437 | 0.06232 | rs11188461 |  |  |  |  |  |  |  |  |  |  |  |  |  |  |  |
| 0.6063 | 10 | igd | 0.0419 | G | \|\| id:ebi-a-GCST90001659 | ebi-a-GCST90001659 | TRUE | C | 97495608 | 1.11E-23 | reported | 3440 | 0.06 | rs11188461 |  |  |  |  |  |  |  |  |  |  |  |  |  |  |  |
| 0.5141 | 10 | igd | 0.0419 | C | \|\| id:ebi-a-GCST90001670 | ebi-a-GCST90001670 | TRUE | T | 97584755 | 3.09E-16 | reported | 3440 | 0.06262 | rs58169787 |  |  |  |  |  |  |  |  |  |  |  |  |  |  |  |
| 0.5142 | 10 | igd | 0.0419 | A | \|\| id:ebi-a-GCST90001670 | ebi-a-GCST90001670 | TRUE | G | 97484659 | 3.05E-16 | reported | 3440 | 0.06262 | rs11188451 |  |  |  |  |  |  |  |  |  |  |  |  |  |  |  |
| 0.5102 | 10 | igd | 0.0419 | C | \|\| id:ebi-a-GCST90001671 | ebi-a-GCST90001671 | TRUE | T | 97618580 | 6.05E-16 | reported | 3440 | 0.06277 | rs3181121 |  |  |  |  |  |  |  |  |  |  |  |  |  |  |  |
| 0.5954 | 10 | igd | 0.0418 | A | \|\| id:ebi-a-GCST90001660 | ebi-a-GCST90001660 | TRUE | C | 97513247 | 9.44E-21 | reported | 3408 | 0.06332 | rs41291576 |  |  |  |  |  |  |  |  |  |  |  |  |  |  |  |
| 0.5147 | 10 | igd | 0.0417 | C | \|\| id:ebi-a-GCST90001483 | ebi-a-GCST90001483 | TRUE | T | 97624840 | 8.92E-17 | reported | 3405 | 0.06155 | rs2275760 |  |  |  |  |  |  |  |  |  |  |  |  |  |  |  |
| 0.5172 | 10 | igd | 0.0416 | A | \|\| id:ebi-a-GCST90001485 | ebi-a-GCST90001485 | TRUE | C | 97513247 | 4.23E-17 | reported | 3437 | 0.06121 | rs41291576 |  |  |  |  |  |  |  |  |  |  |  |  |  |  |  |
| 0.6205 | 10 | igd | 0.0416 | A | \|\| id:ebi-a-GCST90001658 | ebi-a-GCST90001658 | TRUE | C | 97513247 | 1.71E-24 | reported | 3440 | 0.06028 | rs41291576 |  |  |  |  |  |  |  |  |  |  |  |  |  |  |  |
| 0.5139 | 10 | igd | 0.0416 | A | \|\| id:ebi-a-GCST90001671 | ebi-a-GCST90001671 | TRUE | C | 97513247 | 5.75E-16 | reported | 3440 | 0.06319 | rs41291576 |  |  |  |  |  |  |  |  |  |  |  |  |  |  |  |
| 0.3795 | 10 | igd | 0.0416 | A | \|\| id:ebi-a-GCST90001794 | ebi-a-GCST90001794 | TRUE | C | 6191376 | 7.55E-10 | reported | 3658 | 0.0615 | rs147487472 |  |  |  |  |  |  |  |  |  |  |  |  |  |  |  |
| 0.4638 | 6 | igd | 0.0416 | C | \|\| id:ebi-a-GCST90002105 | ebi-a-GCST90002105 | TRUE | A | 30833788 | 1.15E-08 | reported | 2872 | 0.08102 | rs114697479 |  |  |  |  |  |  |  |  |  |  |  |  |  |  |  |
| 0.4239 | 10 | igd | 0.0415 | A | \|\| id:ebi-a-GCST90001775 | ebi-a-GCST90001775 | TRUE | C | 6191376 | 6.12E-12 | reported | 3660 | 0.06144 | rs147487472 |  |  |  |  |  |  |  |  |  |  |  |  |  |  |  |
| 0.4338 | 10 | igd | 0.0414 | A | \|\| id:ebi-a-GCST90001777 | ebi-a-GCST90001777 | TRUE | C | 6191376 | 6.71E-13 | reported | 3657 | 0.06016 | rs147487472 |  |  |  |  |  |  |  |  |  |  |  |  |  |  |  |
| 0.3572 | 10 | igd | 0.0414 | A | \|\| id:ebi-a-GCST90001778 | ebi-a-GCST90001778 | TRUE | C | 6191376 | 4.37E-09 | reported | 3657 | 0.06071 | rs147487472 |  |  |  |  |  |  |  |  |  |  |  |  |  |  |  |
| 0.4264 | 10 | igd | 0.0414 | A | \|\| id:ebi-a-GCST90001779 | ebi-a-GCST90001779 | TRUE | C | 6191376 | 4.00E-12 | reported | 3657 | 0.06126 | rs147487472 |  |  |  |  |  |  |  |  |  |  |  |  |  |  |  |
| 0.4191 | 10 | igd | 0.0414 | A | \|\| id:ebi-a-GCST90001784 | ebi-a-GCST90001784 | TRUE | C | 6191376 | 1.20E-11 | reported | 3657 | 0.06162 | rs147487472 |  |  |  |  |  |  |  |  |  |  |  |  |  |  |  |
| 0.3991 | 10 | igd | 0.0414 | A | \|\| id:ebi-a-GCST90001787 | ebi-a-GCST90001787 | TRUE | C | 6191376 | 5.96E-11 | reported | 3657 | 0.0608 | rs147487472 |  |  |  |  |  |  |  |  |  |  |  |  |  |  |  |
| 0.4586 | 10 | igd | 0.0414 | A | \|\| id:ebi-a-GCST90001789 | ebi-a-GCST90001789 | TRUE | C | 6191376 | 2.15E-14 | reported | 3657 | 0.05978 | rs147487472 |  |  |  |  |  |  |  |  |  |  |  |  |  |  |  |
| 0.448 | 10 | igd | 0.0414 | A | \|\| id:ebi-a-GCST90001790 | ebi-a-GCST90001790 | TRUE | C | 6191376 | 1.28E-13 | reported | 3657 | 0.06025 | rs147487472 |  |  |  |  |  |  |  |  |  |  |  |  |  |  |  |
| 0.4465 | 10 | igd | 0.0414 | A | \|\| id:ebi-a-GCST90001791 | ebi-a-GCST90001791 | TRUE | C | 6191376 | 5.60E-13 | reported | 3657 | 0.0617 | rs147487472 |  |  |  |  |  |  |  |  |  |  |  |  |  |  |  |
| 0.4095 | 10 | igd | 0.0414 | A | \|\| id:ebi-a-GCST90001792 | ebi-a-GCST90001792 | TRUE | C | 6191376 | 1.37E-11 | reported | 3657 | 0.06037 | rs147487472 |  |  |  |  |  |  |  |  |  |  |  |  |  |  |  |
| 0.4787 | 10 | igd | 0.0414 | A | \|\| id:ebi-a-GCST90001793 | ebi-a-GCST90001793 | TRUE | C | 6191376 | 3.75E-15 | reported | 3657 | 0.06062 | rs147487472 |  |  |  |  |  |  |  |  |  |  |  |  |  |  |  |
| -0.5715 | 10 | igd | 0.0413 | T | \|\| id:ebi-a-GCST90002032 | ebi-a-GCST90002032 | TRUE | C | 97706698 | 1.14E-16 | reported | 2920 | 0.06854 | rs149030330 |  |  |  |  |  |  |  |  |  |  |  |  |  |  |  |
| 0.4729 | 1 | igd | 0.0412 | A | \|\| id:ebi-a-GCST90001866 | ebi-a-GCST90001866 | TRUE | G | 167435358 | 2.70E-12 | reported | 2914 | 0.06734 | rs79903733 |  |  |  |  |  |  |  |  |  |  |  |  |  |  |  |
| 0.4284 | 3 | igd | 0.0412 | G | \|\| id:ebi-a-GCST90002017 | ebi-a-GCST90002017 | TRUE | A | 46354619 | 1.14E-09 | reported | 2850 | 0.07014 | rs140058837 |  |  |  |  |  |  |  |  |  |  |  |  |  |  |  |
| 0.5194 | 10 | igd | 0.0411 | T | \|\| id:ebi-a-GCST90001489 | ebi-a-GCST90001489 | TRUE | C | 97569547 | 7.70E-16 | reported | 3405 | 0.06414 | rs192072504 |  |  |  |  |  |  |  |  |  |  |  |  |  |  |  |
| 0.8489 | 1 | igd | 0.0411 | T | \|\| id:ebi-a-GCST90001858 | ebi-a-GCST90001858 | TRUE | C | 167427062 | 5.58E-37 | reported | 2919 | 0.06589 | rs17348881 |  |  |  |  |  |  |  |  |  |  |  |  |  |  |  |
| 0.5631 | 1 | igd | 0.0411 | T | \|\| id:ebi-a-GCST90001860 | ebi-a-GCST90001860 | TRUE | C | 167427062 | 1.21E-16 | reported | 2919 | 0.06759 | rs17348881 |  |  |  |  |  |  |  |  |  |  |  |  |  |  |  |
| 0.6388 | 1 | igd | 0.0411 | A | \|\| id:ebi-a-GCST90001861 | ebi-a-GCST90001861 | TRUE | G | 167435358 | 1.05E-21 | reported | 2919 | 0.0662 | rs79903733 |  |  |  |  |  |  |  |  |  |  |  |  |  |  |  |
| 0.5093 | 1 | igd | 0.0411 | T | \|\| id:ebi-a-GCST90001863 | ebi-a-GCST90001863 | TRUE | C | 167427062 | 2.38E-14 | reported | 2919 | 0.06643 | rs17348881 |  |  |  |  |  |  |  |  |  |  |  |  |  |  |  |
| 0.7411 | 1 | igd | 0.0411 | T | \|\| id:ebi-a-GCST90001864 | ebi-a-GCST90001864 | TRUE | C | 167427062 | 2.80E-28 | reported | 2919 | 0.0665 | rs17348881 |  |  |  |  |  |  |  |  |  |  |  |  |  |  |  |
| -0.5165 | 10 | igd | 0.0411 | T | \|\| id:ebi-a-GCST90002031 | ebi-a-GCST90002031 | TRUE | C | 97714035 | 8.70E-14 | reported | 2920 | 0.06891 | rs114574240 |  |  |  |  |  |  |  |  |  |  |  |  |  |  |  |
| -0.7335 | 12 | igd | 0.0411 | A | \|\| id:ebi-a-GCST90002073 | ebi-a-GCST90002073 | TRUE | G | 70219930 | 2.96E-25 | reported | 2850 | 0.06997 | rs76831874 |  |  |  |  |  |  |  |  |  |  |  |  |  |  |  |
| 0.5613 | 10 | igd | 0.041 | T | \|\| id:ebi-a-GCST90001484 | ebi-a-GCST90001484 | TRUE | C | 97628917 | 1.17E-19 | reported | 3437 | 0.06149 | rs73315224 |  |  |  |  |  |  |  |  |  |  |  |  |  |  |  |
| 0.528 | 10 | igd | 0.041 | T | \|\| id:ebi-a-GCST90001485 | ebi-a-GCST90001485 | TRUE | C | 97628917 | 1.34E-17 | reported | 3437 | 0.06149 | rs73315224 |  |  |  |  |  |  |  |  |  |  |  |  |  |  |  |
| -0.3431 | 10 | igd | 0.041 | A | \|\| id:ebi-a-GCST90001485 | ebi-a-GCST90001485 | TRUE | G | 97442176 | 2.79E-08 | reported | 3437 | 0.06163 | rs141293370 |  |  |  |  |  |  |  |  |  |  |  |  |  |  |  |
| -0.4498 | 6 | igd | 0.041 | G | \|\| id:ebi-a-GCST90001752 | ebi-a-GCST90001752 | TRUE | A | 32583261 | 3.07E-08 | reported | 3657 | 0.08105 | rs151212302 |  |  |  |  |  |  |  |  |  |  |  |  |  |  |  |
| 0.7712 | 10 | igd | 0.0409 | T | \|\| id:ebi-a-GCST90001497 | ebi-a-GCST90001497 | TRUE | G | 98139264 | 2.27E-32 | reported | 3437 | 0.06444 | rs111534725 |  |  |  |  |  |  |  |  |  |  |  |  |  |  |  |
| -0.3414 | 10 | igd | 0.0409 | T | \|\| id:ebi-a-GCST90001777 | ebi-a-GCST90001777 | TRUE | C | 6044517 | 2.59E-08 | reported | 3657 | 0.06118 | rs62626310 |  |  |  |  |  |  |  |  |  |  |  |  |  |  |  |
| -0.3446 | 10 | igd | 0.0409 | T | \|\| id:ebi-a-GCST90001778 | ebi-a-GCST90001778 | TRUE | C | 6044517 | 2.37E-08 | reported | 3657 | 0.06159 | rs62626310 |  |  |  |  |  |  |  |  |  |  |  |  |  |  |  |
| 0.6404 | 1 | igd | 0.0409 | T | \|\| id:ebi-a-GCST90001845 | ebi-a-GCST90001845 | TRUE | C | 167427062 | 3.53E-21 | reported | 2911 | 0.06728 | rs17348881 |  |  |  |  |  |  |  |  |  |  |  |  |  |  |  |
| 0.4289 | 10 | igd | 0.0409 | C | \|\| id:ebi-a-GCST90002032 | ebi-a-GCST90002032 | TRUE | A | 96667388 | 2.02E-09 | reported | 2920 | 0.0713 | rs79716071 |  |  |  |  |  |  |  |  |  |  |  |  |  |  |  |
| -0.4349 | 12 | igd | 0.0409 | G | \|\| id:ebi-a-GCST90002073 | ebi-a-GCST90002073 | TRUE | A | 70134433 | 1.84E-10 | reported | 2850 | 0.06798 | rs75180733 |  |  |  |  |  |  |  |  |  |  |  |  |  |  |  |
| 0.348 | 3 | igd | 0.0409 | G | \|\| id:ebi-a-GCST90002004 | ebi-a-GCST90002004 | TRUE | A | 46354619 | 1.97E-08 | reported | 3629 | 0.06185 | rs140058837 |  |  |  |  |  |  |  |  |  |  |  |  |  |  |  |
| 0.5645 | 10 | igd | 0.0408 | T | \|\| id:ebi-a-GCST90001489 | ebi-a-GCST90001489 | TRUE | G | 98139264 | 1.77E-17 | reported | 3405 | 0.066 | rs111534725 |  |  |  |  |  |  |  |  |  |  |  |  |  |  |  |
| 0.7109 | 10 | igd | 0.0408 | T | \|\| id:ebi-a-GCST90001495 | ebi-a-GCST90001495 | TRUE | G | 98139264 | 1.64E-26 | reported | 3405 | 0.06615 | rs111534725 |  |  |  |  |  |  |  |  |  |  |  |  |  |  |  |
| 0.3583 | 22 | igd | 0.0407 | A | \|\| id:ebi-a-GCST90001711 | ebi-a-GCST90001711 | TRUE | G | 39982756 | 9.29E-09 | reported | 3657 | 0.06225 | rs73169364 |  |  |  |  |  |  |  |  |  |  |  |  |  |  |  |
| 0.683 | 17 | igd | 0.0407 | C | \|\| id:ebi-a-GCST90001907 | ebi-a-GCST90001907 | TRUE | T | 38715186 | 3.72E-23 | reported | 2910 | 0.06832 | rs2228015 |  |  |  |  |  |  |  |  |  |  |  |  |  |  |  |
| 0.3833 | 17 | igd | 0.0407 | C | \|\| id:ebi-a-GCST90001908 | ebi-a-GCST90001908 | TRUE | T | 38715186 | 2.61E-08 | reported | 2910 | 0.06868 | rs2228015 |  |  |  |  |  |  |  |  |  |  |  |  |  |  |  |
| 0.423 | 2 | igd | 0.0406 | A | \|\| id:ebi-a-GCST90001508 | ebi-a-GCST90001508 | TRUE | G | 39604703 | 5.99E-11 | reported | 3437 | 0.06444 | rs116483429 |  |  |  |  |  |  |  |  |  |  |  |  |  |  |  |
| 0.3793 | 2 | igd | 0.0406 | A | \|\| id:ebi-a-GCST90001509 | ebi-a-GCST90001509 | TRUE | G | 39604703 | 4.41E-09 | reported | 3437 | 0.06448 | rs116483429 |  |  |  |  |  |  |  |  |  |  |  |  |  |  |  |
| 0.5004 | 10 | igd | 0.0402 | A | \|\| id:ebi-a-GCST90001483 | ebi-a-GCST90001483 | TRUE | G | 99297093 | 1.38E-14 | reported | 3405 | 0.06472 | rs78525581 |  |  |  |  |  |  |  |  |  |  |  |  |  |  |  |
| 0.5435 | 10 | igd | 0.0402 | A | \|\| id:ebi-a-GCST90001484 | ebi-a-GCST90001484 | TRUE | G | 99297093 | 3.51E-17 | reported | 3437 | 0.06414 | rs78525581 |  |  |  |  |  |  |  |  |  |  |  |  |  |  |  |
| 0.4796 | 10 | igd | 0.0402 | A | \|\| id:ebi-a-GCST90001485 | ebi-a-GCST90001485 | TRUE | G | 99297093 | 1.01E-13 | reported | 3437 | 0.0642 | rs78525581 |  |  |  |  |  |  |  |  |  |  |  |  |  |  |  |
| 0.4847 | 10 | igd | 0.0402 | A | \|\| id:ebi-a-GCST90001489 | ebi-a-GCST90001489 | TRUE | G | 99297093 | 4.02E-13 | reported | 3405 | 0.06654 | rs78525581 |  |  |  |  |  |  |  |  |  |  |  |  |  |  |  |
| 0.4271 | 10 | igd | 0.0402 | A | \|\| id:ebi-a-GCST90001491 | ebi-a-GCST90001491 | TRUE | G | 99297093 | 9.38E-11 | reported | 3437 | 0.06574 | rs78525581 |  |  |  |  |  |  |  |  |  |  |  |  |  |  |  |
| 0.5394 | 10 | igd | 0.0402 | A | \|\| id:ebi-a-GCST90001495 | ebi-a-GCST90001495 | TRUE | G | 99297093 | 1.20E-15 | reported | 3405 | 0.06707 | rs78525581 |  |  |  |  |  |  |  |  |  |  |  |  |  |  |  |
| 0.502 | 10 | igd | 0.0402 | A | \|\| id:ebi-a-GCST90001496 | ebi-a-GCST90001496 | TRUE | G | 99297093 | 5.53E-14 | reported | 3437 | 0.06648 | rs78525581 |  |  |  |  |  |  |  |  |  |  |  |  |  |  |  |
| 0.4796 | 10 | igd | 0.0402 | A | \|\| id:ebi-a-GCST90001497 | ebi-a-GCST90001497 | TRUE | G | 99297093 | 3.52E-13 | reported | 3437 | 0.06568 | rs78525581 |  |  |  |  |  |  |  |  |  |  |  |  |  |  |  |
| 0.6228 | 10 | igd | 0.0402 | A | \|\| id:ebi-a-GCST90001660 | ebi-a-GCST90001660 | TRUE | G | 99297093 | 7.13E-21 | reported | 3408 | 0.06602 | rs78525581 |  |  |  |  |  |  |  |  |  |  |  |  |  |  |  |
| 0.5399 | 10 | igd | 0.0402 | A | \|\| id:ebi-a-GCST90001672 | ebi-a-GCST90001672 | TRUE | G | 99297093 | 9.65E-16 | reported | 3408 | 0.0669 | rs78525581 |  |  |  |  |  |  |  |  |  |  |  |  |  |  |  |
| -0.3441 | 22 | igd | 0.0402 | C | \|\| id:ebi-a-GCST90001735 | ebi-a-GCST90001735 | TRUE | G | 43167446 | 3.77E-08 | reported | 3657 | 0.06242 | rs113612962 |  |  |  |  |  |  |  |  |  |  |  |  |  |  |  |
| 0.4395 | 10 | igd | 0.0402 | A | \|\| id:ebi-a-GCST90001892 | ebi-a-GCST90001892 | TRUE | G | 96073994 | 7.74E-10 | reported | 2920 | 0.07123 | rs61886343 |  |  |  |  |  |  |  |  |  |  |  |  |  |  |  |
| -0.6463 | 12 | igd | 0.0402 | A | \|\| id:ebi-a-GCST90002074 | ebi-a-GCST90002074 | TRUE | G | 70219930 | 2.55E-21 | reported | 3112 | 0.06767 | rs76831874 |  |  |  |  |  |  |  |  |  |  |  |  |  |  |  |
| 0.4413 | 19 | igd | 0.04014 | T | \|\| id:prot-a-439 | prot-a-439 | TRUE | G | 51858800 | 2.63E-12 | reported | 3301 | 0.0631 | rs139724399 |  |  |  |  |  |  |  |  |  |  |  |  |  |  |  |
| 0.4914 | 10 | igd | 0.0401 | A | \|\| id:ebi-a-GCST90001658 | ebi-a-GCST90001658 | TRUE | G | 99297093 | 1.10E-14 | reported | 3440 | 0.06331 | rs78525581 |  |  |  |  |  |  |  |  |  |  |  |  |  |  |  |
| 0.4959 | 10 | igd | 0.0401 | A | \|\| id:ebi-a-GCST90001659 | ebi-a-GCST90001659 | TRUE | G | 99297093 | 6.26E-15 | reported | 3440 | 0.06331 | rs78525581 |  |  |  |  |  |  |  |  |  |  |  |  |  |  |  |
| 0.4626 | 10 | igd | 0.0401 | A | \|\| id:ebi-a-GCST90001670 | ebi-a-GCST90001670 | TRUE | G | 99297093 | 2.87E-12 | reported | 3440 | 0.066 | rs78525581 |  |  |  |  |  |  |  |  |  |  |  |  |  |  |  |
| 0.4513 | 10 | igd | 0.0401 | A | \|\| id:ebi-a-GCST90001671 | ebi-a-GCST90001671 | TRUE | G | 99297093 | 1.03E-11 | reported | 3440 | 0.06612 | rs78525581 |  |  |  |  |  |  |  |  |  |  |  |  |  |  |  |
| -0.3895 | 22 | igd | 0.0401 | C | \|\| id:ebi-a-GCST90001702 | ebi-a-GCST90001702 | TRUE | T | 43498212 | 5.96E-10 | reported | 3657 | 0.06274 | rs135016 |  |  |  |  |  |  |  |  |  |  |  |  |  |  |  |
| -0.3989 | 22 | igd | 0.0401 | C | \|\| id:ebi-a-GCST90001703 | ebi-a-GCST90001703 | TRUE | T | 43498212 | 2.28E-10 | reported | 3657 | 0.06272 | rs135016 |  |  |  |  |  |  |  |  |  |  |  |  |  |  |  |
| -0.4129 | 22 | igd | 0.0401 | C | \|\| id:ebi-a-GCST90001704 | ebi-a-GCST90001704 | TRUE | T | 43498212 | 4.88E-11 | reported | 3657 | 0.06262 | rs135016 |  |  |  |  |  |  |  |  |  |  |  |  |  |  |  |
| -0.357 | 22 | igd | 0.0401 | C | \|\| id:ebi-a-GCST90001710 | ebi-a-GCST90001710 | TRUE | T | 43498212 | 1.28E-08 | reported | 3657 | 0.06261 | rs135016 |  |  |  |  |  |  |  |  |  |  |  |  |  |  |  |
| -0.3541 | 22 | igd | 0.0401 | C | \|\| id:ebi-a-GCST90001711 | ebi-a-GCST90001711 | TRUE | T | 43498212 | 1.57E-08 | reported | 3657 | 0.06248 | rs135016 |  |  |  |  |  |  |  |  |  |  |  |  |  |  |  |
| 0.5507 | 10 | igd | 0.0401 | A | \|\| id:ebi-a-GCST90002030 | ebi-a-GCST90002030 | TRUE | C | 97165421 | 2.60E-15 | reported | 2920 | 0.06925 | rs76052175 |  |  |  |  |  |  |  |  |  |  |  |  |  |  |  |
| 0.4697 | 10 | igd | 0.0401 | A | \|\| id:ebi-a-GCST90002031 | ebi-a-GCST90002031 | TRUE | C | 97165421 | 3.81E-11 | reported | 2920 | 0.07078 | rs76052175 |  |  |  |  |  |  |  |  |  |  |  |  |  |  |  |
| 0.511 | 10 | igd | 0.0401 | A | \|\| id:ebi-a-GCST90002032 | ebi-a-GCST90002032 | TRUE | C | 97165421 | 6.12E-13 | reported | 2920 | 0.07067 | rs76052175 |  |  |  |  |  |  |  |  |  |  |  |  |  |  |  |
| -0.4078 | 2 | igd | 0.0401 | T | \|\| id:ebi-a-GCST90002118 | ebi-a-GCST90002118 | TRUE | C | 87529233 | 2.35E-09 | reported | 2920 | 0.06808 | rs141441729 |  |  |  |  |  |  |  |  |  |  |  |  |  |  |  |
| -0.6536 | 2 | igd | 0.0401 | T | \|\| id:ebi-a-GCST90002119 | ebi-a-GCST90002119 | TRUE | C | 87529233 | 1.10E-22 | reported | 2920 | 0.06613 | rs141441729 |  |  |  |  |  |  |  |  |  |  |  |  |  |  |  |
| 0.3476 | 16 | igd | 0.0401 | T | \|\| id:ebi-a-GCST90001992 | ebi-a-GCST90001992 | TRUE | C | 58607804 | 2.29E-08 | reported | 3618 | 0.06205 | rs77640671 |  |  |  |  |  |  |  |  |  |  |  |  |  |  |  |
| 0.4012 | 2 | igd | 0.04 | G | \|\| id:ebi-a-GCST90002098 | ebi-a-GCST90002098 | TRUE | T | 40998619 | 1.62E-09 | reported | 2910 | 0.0663 | rs115007187 |  |  |  |  |  |  |  |  |  |  |  |  |  |  |  |
| -0.3545 | 10 | igd | 0.0399 | C | \|\| id:ebi-a-GCST90001483 | ebi-a-GCST90001483 | TRUE | T | 97561642 | 2.39E-08 | reported | 3405 | 0.06336 | rs149060699 |  |  |  |  |  |  |  |  |  |  |  |  |  |  |  |
| 0.4322 | 1 | igd | 0.0398 | T | \|\| id:ebi-a-GCST90001852 | ebi-a-GCST90001852 | TRUE | C | 167427062 | 1.57E-09 | reported | 2649 | 0.07134 | rs17348881 |  |  |  |  |  |  |  |  |  |  |  |  |  |  |  |
| 0.4106 | 2 | igd | 0.0397 | C | \|\| id:ebi-a-GCST90002098 | ebi-a-GCST90002098 | TRUE | G | 40884370 | 9.47E-10 | reported | 2910 | 0.06689 | rs114500846 |  |  |  |  |  |  |  |  |  |  |  |  |  |  |  |
| 0.3877 | 2 | igd | 0.0395 | A | \|\| id:ebi-a-GCST90002098 | ebi-a-GCST90002098 | TRUE | G | 42985311 | 6.20E-09 | reported | 2910 | 0.06651 | rs114576271 |  |  |  |  |  |  |  |  |  |  |  |  |  |  |  |
| -0.8386 | 6 | igd | 0.0395 | T | \|\| id:ebi-a-GCST90002105 | ebi-a-GCST90002105 | TRUE | C | 32528431 | 1.34E-10 | reported | 2872 | 0.1301 | rs71536556 |  |  |  |  |  |  |  |  |  |  |  |  |  |  |  |
| -0.5958 | 1 | igd | 0.0394 | A | \|\| id:ebi-a-GCST90001858 | ebi-a-GCST90001858 | TRUE | G | 167267029 | 7.00E-17 | reported | 2919 | 0.07095 | rs72693622 |  |  |  |  |  |  |  |  |  |  |  |  |  |  |  |
| -0.3932 | 1 | igd | 0.0394 | A | \|\| id:ebi-a-GCST90001861 | ebi-a-GCST90001861 | TRUE | G | 167267029 | 2.89E-08 | reported | 2919 | 0.07069 | rs72693622 |  |  |  |  |  |  |  |  |  |  |  |  |  |  |  |
| -0.4249 | 1 | igd | 0.0394 | A | \|\| id:ebi-a-GCST90001863 | ebi-a-GCST90001863 | TRUE | G | 167267029 | 1.56E-09 | reported | 2919 | 0.07015 | rs72693622 |  |  |  |  |  |  |  |  |  |  |  |  |  |  |  |
| -0.563 | 1 | igd | 0.0394 | A | \|\| id:ebi-a-GCST90001864 | ebi-a-GCST90001864 | TRUE | G | 167267029 | 3.04E-15 | reported | 2919 | 0.07098 | rs72693622 |  |  |  |  |  |  |  |  |  |  |  |  |  |  |  |
| 0.4339 | 10 | igd | 0.0393 | A | \|\| id:ebi-a-GCST90001491 | ebi-a-GCST90001491 | TRUE | C | 97165421 | 4.30E-11 | reported | 3437 | 0.06559 | rs76052175 |  |  |  |  |  |  |  |  |  |  |  |  |  |  |  |
| 0.4275 | 10 | igd | 0.0393 | A | \|\| id:ebi-a-GCST90001496 | ebi-a-GCST90001496 | TRUE | C | 97165421 | 1.47E-10 | reported | 3437 | 0.0665 | rs76052175 |  |  |  |  |  |  |  |  |  |  |  |  |  |  |  |
| 0.4083 | 10 | igd | 0.0393 | A | \|\| id:ebi-a-GCST90001497 | ebi-a-GCST90001497 | TRUE | C | 97165421 | 5.71E-10 | reported | 3437 | 0.06569 | rs76052175 |  |  |  |  |  |  |  |  |  |  |  |  |  |  |  |
| 0.3812 | 10 | igd | 0.0393 | A | \|\| id:ebi-a-GCST90001672 | ebi-a-GCST90001672 | TRUE | G | 96073994 | 2.21E-08 | reported | 3408 | 0.06797 | rs61886343 |  |  |  |  |  |  |  |  |  |  |  |  |  |  |  |
| -0.427 | 1 | igd | 0.0393 | A | \|\| id:ebi-a-GCST90001845 | ebi-a-GCST90001845 | TRUE | G | 167267029 | 2.69E-09 | reported | 2911 | 0.07154 | rs72693622 |  |  |  |  |  |  |  |  |  |  |  |  |  |  |  |
| 0.4181 | 10 | igd | 0.0392 | A | \|\| id:ebi-a-GCST90001658 | ebi-a-GCST90001658 | TRUE | C | 97165421 | 5.02E-11 | reported | 3440 | 0.06343 | rs76052175 |  |  |  |  |  |  |  |  |  |  |  |  |  |  |  |
| 0.3797 | 10 | igd | 0.0392 | A | \|\| id:ebi-a-GCST90001659 | ebi-a-GCST90001659 | TRUE | C | 97165421 | 2.48E-09 | reported | 3440 | 0.06351 | rs76052175 |  |  |  |  |  |  |  |  |  |  |  |  |  |  |  |
| 0.3956 | 10 | igd | 0.0392 | A | \|\| id:ebi-a-GCST90001670 | ebi-a-GCST90001670 | TRUE | G | 96073994 | 3.62E-09 | reported | 3440 | 0.06686 | rs61886343 |  |  |  |  |  |  |  |  |  |  |  |  |  |  |  |
| 0.3798 | 10 | igd | 0.0392 | A | \|\| id:ebi-a-GCST90001671 | ebi-a-GCST90001671 | TRUE | G | 96073994 | 1.52E-08 | reported | 3440 | 0.06695 | rs61886343 |  |  |  |  |  |  |  |  |  |  |  |  |  |  |  |
| 0.3632 | 10 | igd | 0.0392 | A | \|\| id:ebi-a-GCST90001671 | ebi-a-GCST90001671 | TRUE | C | 97165421 | 4.51E-08 | reported | 3440 | 0.06625 | rs76052175 |  |  |  |  |  |  |  |  |  |  |  |  |  |  |  |
| 0.4093 | 10 | igd | 0.0392 | T | \|\| id:ebi-a-GCST90001672 | ebi-a-GCST90001672 | TRUE | C | 97711014 | 2.51E-09 | reported | 3408 | 0.06849 | rs75584343 |  |  |  |  |  |  |  |  |  |  |  |  |  |  |  |
| 0.4198 | 10 | igd | 0.0391 | A | \|\| id:ebi-a-GCST90001489 | ebi-a-GCST90001489 | TRUE | C | 97165421 | 3.59E-10 | reported | 3405 | 0.06675 | rs76052175 |  |  |  |  |  |  |  |  |  |  |  |  |  |  |  |
| 0.4033 | 10 | igd | 0.0391 | A | \|\| id:ebi-a-GCST90001495 | ebi-a-GCST90001495 | TRUE | C | 97165421 | 2.55E-09 | reported | 3405 | 0.0675 | rs76052175 |  |  |  |  |  |  |  |  |  |  |  |  |  |  |  |
| 0.3827 | 10 | igd | 0.039 | A | \|\| id:ebi-a-GCST90001660 | ebi-a-GCST90001660 | TRUE | C | 97165421 | 1.09E-08 | reported | 3408 | 0.06679 | rs76052175 |  |  |  |  |  |  |  |  |  |  |  |  |  |  |  |
| 0.4303 | 10 | igd | 0.039 | T | \|\| id:ebi-a-GCST90001670 | ebi-a-GCST90001670 | TRUE | C | 97711014 | 1.81E-10 | reported | 3440 | 0.06727 | rs75584343 |  |  |  |  |  |  |  |  |  |  |  |  |  |  |  |
| 0.3926 | 10 | igd | 0.0389 | A | \|\| id:ebi-a-GCST90002030 | ebi-a-GCST90002030 | TRUE | G | 99297093 | 4.15E-08 | reported | 2920 | 0.0714 | rs78525581 |  |  |  |  |  |  |  |  |  |  |  |  |  |  |  |
| 0.7915 | 10 | igd | 0.0388 | T | \|\| id:ebi-a-GCST90001483 | ebi-a-GCST90001483 | TRUE | C | 98103215 | 1.77E-33 | reported | 3405 | 0.06494 | rs113594239 |  |  |  |  |  |  |  |  |  |  |  |  |  |  |  |
| 0.8808 | 10 | igd | 0.0388 | T | \|\| id:ebi-a-GCST90001484 | ebi-a-GCST90001484 | TRUE | C | 98103215 | 7.44E-42 | reported | 3437 | 0.06411 | rs113594239 |  |  |  |  |  |  |  |  |  |  |  |  |  |  |  |
| 0.829 | 10 | igd | 0.0388 | T | \|\| id:ebi-a-GCST90001485 | ebi-a-GCST90001485 | TRUE | C | 98103215 | 2.87E-37 | reported | 3437 | 0.06422 | rs113594239 |  |  |  |  |  |  |  |  |  |  |  |  |  |  |  |
| 0.5997 | 10 | igd | 0.0388 | T | \|\| id:ebi-a-GCST90001491 | ebi-a-GCST90001491 | TRUE | C | 98103215 | 2.60E-19 | reported | 3437 | 0.06635 | rs113594239 |  |  |  |  |  |  |  |  |  |  |  |  |  |  |  |
| 0.8328 | 10 | igd | 0.0388 | T | \|\| id:ebi-a-GCST90001496 | ebi-a-GCST90001496 | TRUE | C | 98103215 | 3.53E-35 | reported | 3437 | 0.06654 | rs113594239 |  |  |  |  |  |  |  |  |  |  |  |  |  |  |  |
| 0.8148 | 10 | igd | 0.0388 | T | \|\| id:ebi-a-GCST90001658 | ebi-a-GCST90001658 | TRUE | C | 98103215 | 4.72E-37 | reported | 3440 | 0.06331 | rs113594239 |  |  |  |  |  |  |  |  |  |  |  |  |  |  |  |
| 0.8338 | 10 | igd | 0.0388 | T | \|\| id:ebi-a-GCST90001659 | ebi-a-GCST90001659 | TRUE | C | 98103215 | 9.56E-39 | reported | 3440 | 0.06325 | rs113594239 |  |  |  |  |  |  |  |  |  |  |  |  |  |  |  |
| 0.772 | 10 | igd | 0.0388 | T | \|\| id:ebi-a-GCST90001670 | ebi-a-GCST90001670 | TRUE | C | 98103215 | 7.28E-31 | reported | 3440 | 0.06618 | rs113594239 |  |  |  |  |  |  |  |  |  |  |  |  |  |  |  |
| 0.8063 | 10 | igd | 0.0388 | T | \|\| id:ebi-a-GCST90001671 | ebi-a-GCST90001671 | TRUE | C | 98103215 | 1.74E-33 | reported | 3440 | 0.06615 | rs113594239 |  |  |  |  |  |  |  |  |  |  |  |  |  |  |  |
| 0.8355 | 10 | igd | 0.0387 | T | \|\| id:ebi-a-GCST90001660 | ebi-a-GCST90001660 | TRUE | C | 98103215 | 1.65E-35 | reported | 3408 | 0.06642 | rs113594239 |  |  |  |  |  |  |  |  |  |  |  |  |  |  |  |
| 0.7793 | 10 | igd | 0.0387 | T | \|\| id:ebi-a-GCST90001672 | ebi-a-GCST90001672 | TRUE | C | 98103215 | 1.92E-30 | reported | 3408 | 0.0673 | rs113594239 |  |  |  |  |  |  |  |  |  |  |  |  |  |  |  |
| -0.4265 | 10 | igd | 0.0387 | G | \|\| id:ebi-a-GCST90002031 | ebi-a-GCST90002031 | TRUE | A | 98455449 | 7.75E-09 | reported | 2920 | 0.07365 | rs117031756 |  |  |  |  |  |  |  |  |  |  |  |  |  |  |  |
| -1.392 | 19 | igd | 0.0386 | A | \|\| id:ebi-a-GCST90001946 | ebi-a-GCST90001946 | TRUE | G | 51770292 | 2.18E-50 | reported | 1634 | 0.09004 | rs11668077 |  |  |  |  |  |  |  |  |  |  |  |  |  |  |  |
| -1.222 | 19 | igd | 0.0386 | A | \|\| id:ebi-a-GCST90001949 | ebi-a-GCST90001949 | TRUE | G | 51770292 | 3.31E-39 | reported | 1634 | 0.09085 | rs11668077 |  |  |  |  |  |  |  |  |  |  |  |  |  |  |  |
| -1.086 | 19 | igd | 0.0386 | A | \|\| id:ebi-a-GCST90001951 | ebi-a-GCST90001951 | TRUE | G | 51770292 | 1.23E-27 | reported | 1464 | 0.09766 | rs11668077 |  |  |  |  |  |  |  |  |  |  |  |  |  |  |  |
| 0.604 | 10 | igd | 0.0383 | G | \|\| id:ebi-a-GCST90001489 | ebi-a-GCST90001489 | TRUE | A | 97410872 | 1.75E-19 | reported | 3405 | 0.06649 | rs146469548 |  |  |  |  |  |  |  |  |  |  |  |  |  |  |  |
| 0.3972 | 19 | igd | 0.03826 | G | \|\| id:prot-a-439 | prot-a-439 | TRUE | A | 51677700 | 6.92E-10 | reported | 3301 | 0.0644 | rs17728644 |  |  |  |  |  |  |  |  |  |  |  |  |  |  |  |
| 0.404 | 3 | igd | 0.0381 | A | \|\| id:ebi-a-GCST90002017 | ebi-a-GCST90002017 | TRUE | G | 46364860 | 3.52E-08 | reported | 2850 | 0.07308 | rs112313229 |  |  |  |  |  |  |  |  |  |  |  |  |  |  |  |
| 0.4555 | 10 | igd | 0.038 | G | \|\| id:ebi-a-GCST90001485 | ebi-a-GCST90001485 | TRUE | A | 97410872 | 2.40E-12 | reported | 3437 | 0.06475 | rs146469548 |  |  |  |  |  |  |  |  |  |  |  |  |  |  |  |
| -0.3845 | 2 | igd | 0.038 | T | \|\| id:ebi-a-GCST90001886 | ebi-a-GCST90001886 | TRUE | C | 204455383 | 2.18E-08 | reported | 2920 | 0.06851 | rs113830927 |  |  |  |  |  |  |  |  |  |  |  |  |  |  |  |
| -0.4209 | 2 | igd | 0.038 | T | \|\| id:ebi-a-GCST90001887 | ebi-a-GCST90001887 | TRUE | C | 204455383 | 3.00E-09 | reported | 2920 | 0.07073 | rs113830927 |  |  |  |  |  |  |  |  |  |  |  |  |  |  |  |
| -0.4524 | 2 | igd | 0.038 | T | \|\| id:ebi-a-GCST90001889 | ebi-a-GCST90001889 | TRUE | C | 204455383 | 6.72E-11 | reported | 2920 | 0.06906 | rs113830927 |  |  |  |  |  |  |  |  |  |  |  |  |  |  |  |
| -0.4697 | 2 | igd | 0.038 | T | \|\| id:ebi-a-GCST90001890 | ebi-a-GCST90001890 | TRUE | C | 204455383 | 8.35E-11 | reported | 2920 | 0.07206 | rs113830927 |  |  |  |  |  |  |  |  |  |  |  |  |  |  |  |
| -0.4812 | 2 | igd | 0.038 | T | \|\| id:ebi-a-GCST90001891 | ebi-a-GCST90001891 | TRUE | C | 204455383 | 1.49E-11 | reported | 2920 | 0.07101 | rs113830927 |  |  |  |  |  |  |  |  |  |  |  |  |  |  |  |
| -0.3933 | 2 | igd | 0.038 | T | \|\| id:ebi-a-GCST90001892 | ebi-a-GCST90001892 | TRUE | C | 204455383 | 3.70E-08 | reported | 2920 | 0.07125 | rs113830927 |  |  |  |  |  |  |  |  |  |  |  |  |  |  |  |
| -0.409 | 2 | igd | 0.038 | T | \|\| id:ebi-a-GCST90001893 | ebi-a-GCST90001893 | TRUE | C | 204455383 | 1.26E-08 | reported | 2920 | 0.07166 | rs113830927 |  |  |  |  |  |  |  |  |  |  |  |  |  |  |  |
| -0.4045 | 2 | igd | 0.038 | T | \|\| id:ebi-a-GCST90001894 | ebi-a-GCST90001894 | TRUE | C | 204455383 | 1.55E-08 | reported | 2920 | 0.07133 | rs113830927 |  |  |  |  |  |  |  |  |  |  |  |  |  |  |  |
| -0.422 | 2 | igd | 0.038 | T | \|\| id:ebi-a-GCST90001898 | ebi-a-GCST90001898 | TRUE | C | 204455383 | 3.68E-09 | reported | 2920 | 0.07133 | rs113830927 |  |  |  |  |  |  |  |  |  |  |  |  |  |  |  |
| -0.5106 | 2 | igd | 0.038 | T | \|\| id:ebi-a-GCST90001900 | ebi-a-GCST90001900 | TRUE | C | 204455383 | 8.95E-13 | reported | 2919 | 0.07114 | rs113830927 |  |  |  |  |  |  |  |  |  |  |  |  |  |  |  |
| -0.4171 | 2 | igd | 0.038 | T | \|\| id:ebi-a-GCST90001902 | ebi-a-GCST90001902 | TRUE | C | 204455383 | 1.01E-09 | reported | 2920 | 0.06807 | rs113830927 |  |  |  |  |  |  |  |  |  |  |  |  |  |  |  |
| -1.347 | 19 | igd | 0.038 | A | \|\| id:ebi-a-GCST90001948 | ebi-a-GCST90001948 | TRUE | G | 51770292 | 3.21E-46 | reported | 1633 | 0.09143 | rs11668077 |  |  |  |  |  |  |  |  |  |  |  |  |  |  |  |
| 0.4252 | 6 | igd | 0.038 | G | \|\| id:ebi-a-GCST90002105 | ebi-a-GCST90002105 | TRUE | A | 32373529 | 7.29E-09 | reported | 2872 | 0.07328 | rs10947264 |  |  |  |  |  |  |  |  |  |  |  |  |  |  |  |
| 0.3985 | 10 | igd | 0.0379 | G | \|\| id:ebi-a-GCST90001483 | ebi-a-GCST90001483 | TRUE | A | 97554402 | 2.67E-10 | reported | 3405 | 0.06289 | rs11818540 |  |  |  |  |  |  |  |  |  |  |  |  |  |  |  |
| 0.4273 | 10 | igd | 0.0379 | G | \|\| id:ebi-a-GCST90001489 | ebi-a-GCST90001489 | TRUE | A | 97554402 | 4.21E-11 | reported | 3405 | 0.06457 | rs11818540 |  |  |  |  |  |  |  |  |  |  |  |  |  |  |  |
| 0.4429 | 10 | igd | 0.0379 | C | \|\| id:ebi-a-GCST90001495 | ebi-a-GCST90001495 | TRUE | T | 97562296 | 1.04E-11 | reported | 3405 | 0.06489 | rs145097648 |  |  |  |  |  |  |  |  |  |  |  |  |  |  |  |
| 0.4293 | 10 | igd | 0.0379 | C | \|\| id:ebi-a-GCST90001660 | ebi-a-GCST90001660 | TRUE | T | 97562296 | 2.40E-11 | reported | 3408 | 0.06405 | rs145097648 |  |  |  |  |  |  |  |  |  |  |  |  |  |  |  |
| 0.4749 | 10 | igd | 0.0378 | T | \|\| id:ebi-a-GCST90001484 | ebi-a-GCST90001484 | TRUE | C | 97578026 | 3.61E-14 | reported | 3437 | 0.06243 | rs7088952 |  |  |  |  |  |  |  |  |  |  |  |  |  |  |  |
| 0.4359 | 10 | igd | 0.0378 | G | \|\| id:ebi-a-GCST90001485 | ebi-a-GCST90001485 | TRUE | A | 97554402 | 3.44E-12 | reported | 3437 | 0.06242 | rs11818540 |  |  |  |  |  |  |  |  |  |  |  |  |  |  |  |
| 0.4832 | 10 | igd | 0.0378 | C | \|\| id:ebi-a-GCST90001491 | ebi-a-GCST90001491 | TRUE | G | 97482986 | 2.92E-14 | reported | 3437 | 0.06329 | rs138520208 |  |  |  |  |  |  |  |  |  |  |  |  |  |  |  |
| 0.5194 | 10 | igd | 0.0378 | G | \|\| id:ebi-a-GCST90001496 | ebi-a-GCST90001496 | TRUE | A | 97554402 | 7.29E-16 | reported | 3437 | 0.06409 | rs11818540 |  |  |  |  |  |  |  |  |  |  |  |  |  |  |  |
| 0.5086 | 10 | igd | 0.0378 | G | \|\| id:ebi-a-GCST90001497 | ebi-a-GCST90001497 | TRUE | A | 97554402 | 1.26E-15 | reported | 3437 | 0.06329 | rs11818540 |  |  |  |  |  |  |  |  |  |  |  |  |  |  |  |
| 0.4405 | 10 | igd | 0.0378 | G | \|\| id:ebi-a-GCST90001658 | ebi-a-GCST90001658 | TRUE | A | 97554402 | 7.17E-13 | reported | 3440 | 0.06115 | rs11818540 |  |  |  |  |  |  |  |  |  |  |  |  |  |  |  |
| 0.467 | 10 | igd | 0.0378 | G | \|\| id:ebi-a-GCST90001659 | ebi-a-GCST90001659 | TRUE | A | 97554402 | 2.74E-14 | reported | 3440 | 0.0611 | rs11818540 |  |  |  |  |  |  |  |  |  |  |  |  |  |  |  |
| 0.3611 | 10 | igd | 0.0378 | G | \|\| id:ebi-a-GCST90001670 | ebi-a-GCST90001670 | TRUE | A | 97554402 | 1.74E-08 | reported | 3440 | 0.06392 | rs11818540 |  |  |  |  |  |  |  |  |  |  |  |  |  |  |  |
| 0.5317 | 10 | igd | 0.0378 | T | \|\| id:ebi-a-GCST90002030 | ebi-a-GCST90002030 | TRUE | C | 97057460 | 1.11E-13 | reported | 2920 | 0.07124 | rs137948887 |  |  |  |  |  |  |  |  |  |  |  |  |  |  |  |
| 0.4831 | 10 | igd | 0.0378 | T | \|\| id:ebi-a-GCST90002031 | ebi-a-GCST90002031 | TRUE | C | 97057460 | 4.65E-11 | reported | 2920 | 0.07313 | rs137948887 |  |  |  |  |  |  |  |  |  |  |  |  |  |  |  |
| 0.4949 | 10 | igd | 0.0378 | T | \|\| id:ebi-a-GCST90002032 | ebi-a-GCST90002032 | TRUE | C | 97057460 | 1.50E-11 | reported | 2920 | 0.07305 | rs137948887 |  |  |  |  |  |  |  |  |  |  |  |  |  |  |  |
| -0.4386 | 11 | igd | 0.0378 | G | \|\| id:ebi-a-GCST90002071 | ebi-a-GCST90002071 | TRUE | T | 5544477 | 5.27E-09 | reported | 2872 | 0.0749 | rs78736654 |  |  |  |  |  |  |  |  |  |  |  |  |  |  |  |
| -0.4036 | 11 | igd | 0.0378 | G | \|\| id:ebi-a-GCST90002072 | ebi-a-GCST90002072 | TRUE | T | 5544477 | 4.41E-08 | reported | 2872 | 0.07353 | rs78736654 |  |  |  |  |  |  |  |  |  |  |  |  |  |  |  |
| 0.3869 | 2 | igd | 0.0377 | A | \|\| id:ebi-a-GCST90002102 | ebi-a-GCST90002102 | TRUE | T | 38422426 | 2.07E-08 | reported | 2919 | 0.06883 | rs73926334 |  |  |  |  |  |  |  |  |  |  |  |  |  |  |  |
| 0.5948 | 2 | igd | 0.0377 | T | \|\| id:ebi-a-GCST90002102 | ebi-a-GCST90002102 | TRUE | C | 39164453 | 5.08E-18 | reported | 2919 | 0.06831 | rs183176024 |  |  |  |  |  |  |  |  |  |  |  |  |  |  |  |
| 0.7027 | 2 | igd | 0.0377 | T | \|\| id:ebi-a-GCST90002102 | ebi-a-GCST90002102 | TRUE | C | 38596674 | 1.90E-23 | reported | 2919 | 0.06982 | rs145947216 |  |  |  |  |  |  |  |  |  |  |  |  |  |  |  |
| 0.5783 | 3 | igd | 0.0376 | A | \|\| id:ebi-a-GCST90001801 | ebi-a-GCST90001801 | TRUE | G | 32945761 | 3.41E-10 | reported | 1836 | 0.0916 | rs75918255 |  |  |  |  |  |  |  |  |  |  |  |  |  |  |  |
| 0.371 | 1 | igd | 0.0376 | A | \|\| id:ebi-a-GCST90001981 | ebi-a-GCST90001981 | TRUE | G | 160808413 | 2.76E-08 | reported | 3618 | 0.06663 | rs111332135 |  |  |  |  |  |  |  |  |  |  |  |  |  |  |  |
| 0.3861 | 1 | igd | 0.0376 | A | \|\| id:ebi-a-GCST90001989 | ebi-a-GCST90001989 | TRUE | G | 160808413 | 9.95E-09 | reported | 3621 | 0.06721 | rs111332135 |  |  |  |  |  |  |  |  |  |  |  |  |  |  |  |
| 0.3947 | 1 | igd | 0.0376 | A | \|\| id:ebi-a-GCST90002087 | ebi-a-GCST90002087 | TRUE | C | 19786101 | 3.57E-08 | reported | 2872 | 0.07143 | rs78520150 |  |  |  |  |  |  |  |  |  |  |  |  |  |  |  |
| 0.5767 | 2 | igd | 0.0376 | T | \|\| id:ebi-a-GCST90002098 | ebi-a-GCST90002098 | TRUE | C | 39164453 | 8.55E-17 | reported | 2910 | 0.06887 | rs183176024 |  |  |  |  |  |  |  |  |  |  |  |  |  |  |  |
| 0.398 | 2 | igd | 0.0376 | T | \|\| id:ebi-a-GCST90002100 | ebi-a-GCST90002100 | TRUE | C | 39164453 | 2.81E-08 | reported | 2910 | 0.07147 | rs183176024 |  |  |  |  |  |  |  |  |  |  |  |  |  |  |  |
| -0.401 | 10 | igd | 0.0375 | G | \|\| id:ebi-a-GCST90001961 | ebi-a-GCST90001961 | TRUE | A | 92011043 | 3.66E-08 | reported | 2920 | 0.07263 | rs370666890 |  |  |  |  |  |  |  |  |  |  |  |  |  |  |  |
| 0.3697 | 1 | igd | 0.0375 | A | \|\| id:ebi-a-GCST90001985 | ebi-a-GCST90001985 | TRUE | G | 160808413 | 3.81E-08 | reported | 3629 | 0.06709 | rs111332135 |  |  |  |  |  |  |  |  |  |  |  |  |  |  |  |
| 0.4452 | 10 | igd | 0.0375 | A | \|\| id:ebi-a-GCST90002030 | ebi-a-GCST90002030 | TRUE | G | 98222551 | 1.21E-09 | reported | 2920 | 0.07301 | rs12780850 |  |  |  |  |  |  |  |  |  |  |  |  |  |  |  |
| 0.4421 | 10 | igd | 0.0375 | A | \|\| id:ebi-a-GCST90002032 | ebi-a-GCST90002032 | TRUE | G | 98222551 | 3.45E-09 | reported | 2920 | 0.07459 | rs12780850 |  |  |  |  |  |  |  |  |  |  |  |  |  |  |  |
| 0.7147 | 2 | igd | 0.0375 | T | \|\| id:ebi-a-GCST90002098 | ebi-a-GCST90002098 | TRUE | C | 38596674 | 9.98E-24 | reported | 2910 | 0.07055 | rs145947216 |  |  |  |  |  |  |  |  |  |  |  |  |  |  |  |
| 0.4331 | 2 | igd | 0.0375 | T | \|\| id:ebi-a-GCST90002100 | ebi-a-GCST90002100 | TRUE | C | 38596674 | 3.93E-09 | reported | 2910 | 0.07335 | rs145947216 |  |  |  |  |  |  |  |  |  |  |  |  |  |  |  |
| 0.491 | 6 | igd | 0.0374 | T | \|\| id:ebi-a-GCST90002105 | ebi-a-GCST90002105 | TRUE | G | 26460862 | 2.07E-11 | reported | 2872 | 0.07298 | rs2393668 |  |  |  |  |  |  |  |  |  |  |  |  |  |  |  |
| 0.4295 | 10 | igd | 0.0372 | T | \|\| id:ebi-a-GCST90001484 | ebi-a-GCST90001484 | TRUE | C | 97057460 | 1.14E-10 | reported | 3437 | 0.06641 | rs137948887 |  |  |  |  |  |  |  |  |  |  |  |  |  |  |  |
| 0.3933 | 10 | igd | 0.0372 | T | \|\| id:ebi-a-GCST90001485 | ebi-a-GCST90001485 | TRUE | C | 97057460 | 3.44E-09 | reported | 3437 | 0.06638 | rs137948887 |  |  |  |  |  |  |  |  |  |  |  |  |  |  |  |
| 0.3974 | 10 | igd | 0.0372 | T | \|\| id:ebi-a-GCST90001491 | ebi-a-GCST90001491 | TRUE | C | 97057460 | 4.89E-09 | reported | 3437 | 0.06775 | rs137948887 |  |  |  |  |  |  |  |  |  |  |  |  |  |  |  |
| 0.3772 | 10 | igd | 0.0372 | T | \|\| id:ebi-a-GCST90001496 | ebi-a-GCST90001496 | TRUE | C | 96529804 | 4.96E-08 | reported | 3437 | 0.06901 | rs61886221 |  |  |  |  |  |  |  |  |  |  |  |  |  |  |  |
| 0.3939 | 10 | igd | 0.0372 | C | \|\| id:ebi-a-GCST90001496 | ebi-a-GCST90001496 | TRUE | A | 94770488 | 5.39E-09 | reported | 3437 | 0.06734 | rs17108042 |  |  |  |  |  |  |  |  |  |  |  |  |  |  |  |
| 0.4136 | 10 | igd | 0.0372 | T | \|\| id:ebi-a-GCST90001496 | ebi-a-GCST90001496 | TRUE | C | 97057460 | 1.87E-09 | reported | 3437 | 0.06864 | rs137948887 |  |  |  |  |  |  |  |  |  |  |  |  |  |  |  |
| 0.404 | 10 | igd | 0.0372 | T | \|\| id:ebi-a-GCST90001497 | ebi-a-GCST90001497 | TRUE | C | 97057460 | 2.79E-09 | reported | 3437 | 0.06779 | rs137948887 |  |  |  |  |  |  |  |  |  |  |  |  |  |  |  |
| 0.385 | 10 | igd | 0.0372 | C | \|\| id:ebi-a-GCST90001497 | ebi-a-GCST90001497 | TRUE | A | 94770488 | 7.68E-09 | reported | 3437 | 0.0665 | rs17108042 |  |  |  |  |  |  |  |  |  |  |  |  |  |  |  |
| 0.3723 | 10 | igd | 0.0372 | T | \|\| id:ebi-a-GCST90001497 | ebi-a-GCST90001497 | TRUE | C | 96529804 | 4.99E-08 | reported | 3437 | 0.06815 | rs61886221 |  |  |  |  |  |  |  |  |  |  |  |  |  |  |  |
| -0.5404 | 19 | igd | 0.0372 | A | \|\| id:ebi-a-GCST90001531 | ebi-a-GCST90001531 | TRUE | G | 51770292 | 2.32E-09 | reported | 1856 | 0.09003 | rs11668077 |  |  |  |  |  |  |  |  |  |  |  |  |  |  |  |
| -0.717 | 19 | igd | 0.0372 | A | \|\| id:ebi-a-GCST90001533 | ebi-a-GCST90001533 | TRUE | G | 51770292 | 6.52E-16 | reported | 1857 | 0.08796 | rs11668077 |  |  |  |  |  |  |  |  |  |  |  |  |  |  |  |
| 0.372 | 10 | igd | 0.0372 | T | \|\| id:ebi-a-GCST90001658 | ebi-a-GCST90001658 | TRUE | C | 97057460 | 1.45E-08 | reported | 3440 | 0.06549 | rs137948887 |  |  |  |  |  |  |  |  |  |  |  |  |  |  |  |
| 0.4729 | 10 | igd | 0.0372 | G | \|\| id:ebi-a-GCST90002030 | ebi-a-GCST90002030 | TRUE | T | 96735730 | 1.02E-10 | reported | 2920 | 0.07288 | rs9332179 |  |  |  |  |  |  |  |  |  |  |  |  |  |  |  |
| 0.6781 | 10 | igd | 0.0372 | C | \|\| id:ebi-a-GCST90002030 | ebi-a-GCST90002030 | TRUE | T | 97562296 | 8.25E-23 | reported | 2920 | 0.0684 | rs145097648 |  |  |  |  |  |  |  |  |  |  |  |  |  |  |  |
| 0.5908 | 10 | igd | 0.0372 | C | \|\| id:ebi-a-GCST90002031 | ebi-a-GCST90002031 | TRUE | T | 97562296 | 8.92E-17 | reported | 2920 | 0.0706 | rs145097648 |  |  |  |  |  |  |  |  |  |  |  |  |  |  |  |
| 0.6232 | 10 | igd | 0.0372 | C | \|\| id:ebi-a-GCST90002032 | ebi-a-GCST90002032 | TRUE | G | 97482986 | 1.46E-18 | reported | 2920 | 0.07041 | rs138520208 |  |  |  |  |  |  |  |  |  |  |  |  |  |  |  |
| -0.4889 | 19 | igd | 0.0371 | A | \|\| id:ebi-a-GCST90001517 | ebi-a-GCST90001517 | TRUE | G | 51770292 | 3.74E-08 | reported | 1858 | 0.08848 | rs11668077 |  |  |  |  |  |  |  |  |  |  |  |  |  |  |  |
| -0.5056 | 19 | igd | 0.0371 | A | \|\| id:ebi-a-GCST90001518 | ebi-a-GCST90001518 | TRUE | G | 51770292 | 1.87E-08 | reported | 1858 | 0.08951 | rs11668077 |  |  |  |  |  |  |  |  |  |  |  |  |  |  |  |
| 0.5828 | 10 | igd | 0.037 | T | \|\| id:ebi-a-GCST90001495 | ebi-a-GCST90001495 | TRUE | C | 97482709 | 1.86E-18 | reported | 3405 | 0.0661 | rs142393920 |  |  |  |  |  |  |  |  |  |  |  |  |  |  |  |
| 0.3987 | 10 | igd | 0.037 | A | \|\| id:ebi-a-GCST90001496 | ebi-a-GCST90001496 | TRUE | G | 98222551 | 1.54E-08 | reported | 3437 | 0.07031 | rs12780850 |  |  |  |  |  |  |  |  |  |  |  |  |  |  |  |
| 0.3864 | 10 | igd | 0.037 | A | \|\| id:ebi-a-GCST90001497 | ebi-a-GCST90001497 | TRUE | G | 98222551 | 2.82E-08 | reported | 3437 | 0.06944 | rs12780850 |  |  |  |  |  |  |  |  |  |  |  |  |  |  |  |
| 0.5929 | 10 | igd | 0.037 | T | \|\| id:ebi-a-GCST90001660 | ebi-a-GCST90001660 | TRUE | C | 97482709 | 1.62E-19 | reported | 3408 | 0.06521 | rs142393920 |  |  |  |  |  |  |  |  |  |  |  |  |  |  |  |
| 0.5238 | 10 | igd | 0.037 | T | \|\| id:ebi-a-GCST90001672 | ebi-a-GCST90001672 | TRUE | C | 97482709 | 2.93E-15 | reported | 3408 | 0.06604 | rs142393920 |  |  |  |  |  |  |  |  |  |  |  |  |  |  |  |
| -0.5381 | 17 | igd | 0.037 | C | \|\| id:ebi-a-GCST90001926 | ebi-a-GCST90001926 | TRUE | T | 56744055 | 8.45E-14 | reported | 2920 | 0.07174 | rs144426810 |  |  |  |  |  |  |  |  |  |  |  |  |  |  |  |
| -0.6028 | 10 | igd | 0.037 | T | \|\| id:ebi-a-GCST90001961 | ebi-a-GCST90001961 | TRUE | C | 98103215 | 2.54E-16 | reported | 2920 | 0.07314 | rs113594239 |  |  |  |  |  |  |  |  |  |  |  |  |  |  |  |
| 0.7759 | 10 | igd | 0.037 | T | \|\| id:ebi-a-GCST90002030 | ebi-a-GCST90002030 | TRUE | C | 98103215 | 1.12E-26 | reported | 2920 | 0.07186 | rs113594239 |  |  |  |  |  |  |  |  |  |  |  |  |  |  |  |
| 0.6586 | 10 | igd | 0.037 | T | \|\| id:ebi-a-GCST90002031 | ebi-a-GCST90002031 | TRUE | C | 98103215 | 8.14E-19 | reported | 2920 | 0.07384 | rs113594239 |  |  |  |  |  |  |  |  |  |  |  |  |  |  |  |
| 0.4166 | 10 | igd | 0.037 | T | \|\| id:ebi-a-GCST90002032 | ebi-a-GCST90002032 | TRUE | C | 98103215 | 2.36E-08 | reported | 2920 | 0.07442 | rs113594239 |  |  |  |  |  |  |  |  |  |  |  |  |  |  |  |
| 0.3814 | 10 | igd | 0.0369 | A | \|\| id:ebi-a-GCST90001658 | ebi-a-GCST90001658 | TRUE | G | 98222551 | 1.43E-08 | reported | 3440 | 0.06712 | rs12780850 |  |  |  |  |  |  |  |  |  |  |  |  |  |  |  |
| -0.3659 | 22 | igd | 0.0368 | C | \|\| id:ebi-a-GCST90001704 | ebi-a-GCST90001704 | TRUE | T | 44581286 | 3.31E-08 | reported | 3657 | 0.0661 | rs117704411 |  |  |  |  |  |  |  |  |  |  |  |  |  |  |  |
| -0.3611 | 22 | igd | 0.0368 | C | \|\| id:ebi-a-GCST90001711 | ebi-a-GCST90001711 | TRUE | T | 44581286 | 4.45E-08 | reported | 3657 | 0.06586 | rs117704411 |  |  |  |  |  |  |  |  |  |  |  |  |  |  |  |
| 0.4249 | 10 | igd | 0.0367 | G | \|\| id:ebi-a-GCST90001484 | ebi-a-GCST90001484 | TRUE | C | 98948911 | 2.03E-10 | reported | 3437 | 0.06662 | rs701804 |  |  |  |  |  |  |  |  |  |  |  |  |  |  |  |
| 0.4363 | 10 | igd | 0.0367 | T | \|\| id:ebi-a-GCST90001484 | ebi-a-GCST90001484 | TRUE | C | 97482709 | 1.14E-11 | reported | 3437 | 0.06406 | rs142393920 |  |  |  |  |  |  |  |  |  |  |  |  |  |  |  |
| 0.3662 | 10 | igd | 0.0367 | G | \|\| id:ebi-a-GCST90001485 | ebi-a-GCST90001485 | TRUE | C | 98948911 | 4.18E-08 | reported | 3437 | 0.06663 | rs701804 |  |  |  |  |  |  |  |  |  |  |  |  |  |  |  |
| 0.5712 | 10 | igd | 0.0367 | T | \|\| id:ebi-a-GCST90001491 | ebi-a-GCST90001491 | TRUE | C | 97482709 | 1.69E-18 | reported | 3437 | 0.06472 | rs142393920 |  |  |  |  |  |  |  |  |  |  |  |  |  |  |  |
| 0.5619 | 10 | igd | 0.0367 | T | \|\| id:ebi-a-GCST90001496 | ebi-a-GCST90001496 | TRUE | C | 97482709 | 1.66E-17 | reported | 3437 | 0.06563 | rs142393920 |  |  |  |  |  |  |  |  |  |  |  |  |  |  |  |
| 0.5546 | 10 | igd | 0.0367 | T | \|\| id:ebi-a-GCST90001497 | ebi-a-GCST90001497 | TRUE | C | 97482709 | 1.72E-17 | reported | 3437 | 0.06481 | rs142393920 |  |  |  |  |  |  |  |  |  |  |  |  |  |  |  |
| 0.5436 | 10 | igd | 0.0366 | T | \|\| id:ebi-a-GCST90001658 | ebi-a-GCST90001658 | TRUE | C | 97482709 | 5.02E-18 | reported | 3440 | 0.06247 | rs142393920 |  |  |  |  |  |  |  |  |  |  |  |  |  |  |  |
| 0.5554 | 10 | igd | 0.0366 | T | \|\| id:ebi-a-GCST90001659 | ebi-a-GCST90001659 | TRUE | C | 97482709 | 9.46E-19 | reported | 3440 | 0.06245 | rs142393920 |  |  |  |  |  |  |  |  |  |  |  |  |  |  |  |
| 0.4042 | 10 | igd | 0.0366 | G | \|\| id:ebi-a-GCST90001659 | ebi-a-GCST90001659 | TRUE | C | 98948911 | 6.40E-10 | reported | 3440 | 0.06521 | rs701804 |  |  |  |  |  |  |  |  |  |  |  |  |  |  |  |
| 0.4931 | 10 | igd | 0.0366 | T | \|\| id:ebi-a-GCST90001670 | ebi-a-GCST90001670 | TRUE | C | 97482709 | 5.37E-14 | reported | 3440 | 0.06528 | rs142393920 |  |  |  |  |  |  |  |  |  |  |  |  |  |  |  |
| 0.4915 | 10 | igd | 0.0366 | T | \|\| id:ebi-a-GCST90001671 | ebi-a-GCST90001671 | TRUE | C | 97482709 | 6.61E-14 | reported | 3440 | 0.0653 | rs142393920 |  |  |  |  |  |  |  |  |  |  |  |  |  |  |  |
| -0.4896 | 10 | igd | 0.0366 | A | \|\| id:ebi-a-GCST90001961 | ebi-a-GCST90001961 | TRUE | G | 95539210 | 5.44E-11 | reported | 2920 | 0.07437 | rs187513283 |  |  |  |  |  |  |  |  |  |  |  |  |  |  |  |
| 0.4312 | 10 | igd | 0.0366 | A | \|\| id:ebi-a-GCST90002030 | ebi-a-GCST90002030 | TRUE | G | 95539210 | 5.50E-09 | reported | 2920 | 0.07372 | rs187513283 |  |  |  |  |  |  |  |  |  |  |  |  |  |  |  |
| 4.551 | 2 | igd | 0.0365 | A | \|\| id:ebi-a-GCST90001696 | ebi-a-GCST90001696 | TRUE | G | 40275338 | 4.80E-08 | reported | 3425 | 0.8319 | rs17024974 |  |  |  |  |  |  |  |  |  |  |  |  |  |  |  |
| -0.7397 | 22 | igd | 0.0365 | A | \|\| id:ebi-a-GCST90001702 | ebi-a-GCST90001702 | TRUE | G | 41957510 | 2.22E-29 | reported | 3657 | 0.06516 | rs117705111 |  |  |  |  |  |  |  |  |  |  |  |  |  |  |  |
| -0.731 | 22 | igd | 0.0365 | A | \|\| id:ebi-a-GCST90001703 | ebi-a-GCST90001703 | TRUE | G | 41957510 | 1.02E-28 | reported | 3657 | 0.06518 | rs117705111 |  |  |  |  |  |  |  |  |  |  |  |  |  |  |  |
| -0.763 | 22 | igd | 0.0365 | A | \|\| id:ebi-a-GCST90001704 | ebi-a-GCST90001704 | TRUE | G | 41957510 | 2.97E-31 | reported | 3657 | 0.065 | rs117705111 |  |  |  |  |  |  |  |  |  |  |  |  |  |  |  |
| -0.6939 | 22 | igd | 0.0365 | A | \|\| id:ebi-a-GCST90001710 | ebi-a-GCST90001710 | TRUE | G | 41957510 | 3.78E-26 | reported | 3657 | 0.06509 | rs117705111 |  |  |  |  |  |  |  |  |  |  |  |  |  |  |  |
| -0.699 | 22 | igd | 0.0365 | A | \|\| id:ebi-a-GCST90001711 | ebi-a-GCST90001711 | TRUE | G | 41957510 | 1.30E-26 | reported | 3657 | 0.06495 | rs117705111 |  |  |  |  |  |  |  |  |  |  |  |  |  |  |  |
| 0.4002 | 1 | igd | 0.0365 | C | \|\| id:ebi-a-GCST90001989 | ebi-a-GCST90001989 | TRUE | A | 161468609 | 1.83E-09 | reported | 3621 | 0.06639 | rs74124991 |  |  |  |  |  |  |  |  |  |  |  |  |  |  |  |
| 0.3971 | 10 | igd | 0.0364 | G | \|\| id:ebi-a-GCST90001483 | ebi-a-GCST90001483 | TRUE | C | 98948911 | 3.99E-09 | reported | 3405 | 0.06731 | rs701804 |  |  |  |  |  |  |  |  |  |  |  |  |  |  |  |
| 0.444 | 10 | igd | 0.0364 | A | \|\| id:ebi-a-GCST90001484 | ebi-a-GCST90001484 | TRUE | G | 95539210 | 3.28E-11 | reported | 3437 | 0.06671 | rs187513283 |  |  |  |  |  |  |  |  |  |  |  |  |  |  |  |
| 0.4381 | 10 | igd | 0.0364 | A | \|\| id:ebi-a-GCST90001485 | ebi-a-GCST90001485 | TRUE | G | 95539210 | 5.62E-11 | reported | 3437 | 0.06663 | rs187513283 |  |  |  |  |  |  |  |  |  |  |  |  |  |  |  |
| 0.4463 | 10 | igd | 0.0364 | A | \|\| id:ebi-a-GCST90001491 | ebi-a-GCST90001491 | TRUE | G | 95539210 | 5.83E-11 | reported | 3437 | 0.06794 | rs187513283 |  |  |  |  |  |  |  |  |  |  |  |  |  |  |  |
| 0.4019 | 10 | igd | 0.0364 | G | \|\| id:ebi-a-GCST90001495 | ebi-a-GCST90001495 | TRUE | C | 98948911 | 8.19E-09 | reported | 3405 | 0.06954 | rs701804 |  |  |  |  |  |  |  |  |  |  |  |  |  |  |  |
| 0.5266 | 10 | igd | 0.0364 | A | \|\| id:ebi-a-GCST90001496 | ebi-a-GCST90001496 | TRUE | G | 95539210 | 2.32E-14 | reported | 3437 | 0.0687 | rs187513283 |  |  |  |  |  |  |  |  |  |  |  |  |  |  |  |
| 0.505 | 10 | igd | 0.0364 | A | \|\| id:ebi-a-GCST90001497 | ebi-a-GCST90001497 | TRUE | G | 95539210 | 1.27E-13 | reported | 3437 | 0.06787 | rs187513283 |  |  |  |  |  |  |  |  |  |  |  |  |  |  |  |
| 0.5152 | 10 | igd | 0.0364 | G | \|\| id:ebi-a-GCST90001660 | ebi-a-GCST90001660 | TRUE | C | 98948911 | 6.56E-14 | reported | 3408 | 0.06843 | rs701804 |  |  |  |  |  |  |  |  |  |  |  |  |  |  |  |
| 0.4164 | 10 | igd | 0.0364 | G | \|\| id:ebi-a-GCST90001672 | ebi-a-GCST90001672 | TRUE | C | 98948911 | 2.09E-09 | reported | 3408 | 0.06931 | rs701804 |  |  |  |  |  |  |  |  |  |  |  |  |  |  |  |
| -0.8993 | 22 | igd | 0.0364 | A | \|\| id:ebi-a-GCST90001702 | ebi-a-GCST90001702 | TRUE | G | 42855698 | 7.99E-41 | reported | 3657 | 0.06639 | rs111993863 |  |  |  |  |  |  |  |  |  |  |  |  |  |  |  |
| -0.9077 | 22 | igd | 0.0364 | A | \|\| id:ebi-a-GCST90001703 | ebi-a-GCST90001703 | TRUE | G | 42855698 | 1.38E-41 | reported | 3657 | 0.06635 | rs111993863 |  |  |  |  |  |  |  |  |  |  |  |  |  |  |  |
| -0.9174 | 22 | igd | 0.0364 | A | \|\| id:ebi-a-GCST90001704 | ebi-a-GCST90001704 | TRUE | G | 42855698 | 1.43E-42 | reported | 3657 | 0.06623 | rs111993863 |  |  |  |  |  |  |  |  |  |  |  |  |  |  |  |
| -0.8723 | 22 | igd | 0.0364 | A | \|\| id:ebi-a-GCST90001710 | ebi-a-GCST90001710 | TRUE | G | 42855698 | 1.12E-38 | reported | 3657 | 0.06629 | rs111993863 |  |  |  |  |  |  |  |  |  |  |  |  |  |  |  |
| -0.877 | 22 | igd | 0.0364 | A | \|\| id:ebi-a-GCST90001711 | ebi-a-GCST90001711 | TRUE | G | 42855698 | 3.00E-39 | reported | 3657 | 0.06612 | rs111993863 |  |  |  |  |  |  |  |  |  |  |  |  |  |  |  |
| 0.3867 | 10 | igd | 0.0363 | G | \|\| id:ebi-a-GCST90001495 | ebi-a-GCST90001495 | TRUE | T | 96735730 | 4.43E-08 | reported | 3405 | 0.07051 | rs9332179 |  |  |  |  |  |  |  |  |  |  |  |  |  |  |  |
| 0.5198 | 10 | igd | 0.0363 | A | \|\| id:ebi-a-GCST90001658 | ebi-a-GCST90001658 | TRUE | G | 95539210 | 2.46E-15 | reported | 3440 | 0.06536 | rs187513283 |  |  |  |  |  |  |  |  |  |  |  |  |  |  |  |
| 0.5521 | 10 | igd | 0.0363 | A | \|\| id:ebi-a-GCST90001659 | ebi-a-GCST90001659 | TRUE | G | 95539210 | 4.07E-17 | reported | 3440 | 0.06529 | rs187513283 |  |  |  |  |  |  |  |  |  |  |  |  |  |  |  |
| 0.7078 | 10 | igd | 0.0363 | A | \|\| id:ebi-a-GCST90001670 | ebi-a-GCST90001670 | TRUE | G | 95539210 | 2.92E-25 | reported | 3440 | 0.06762 | rs187513283 |  |  |  |  |  |  |  |  |  |  |  |  |  |  |  |
| 0.6738 | 10 | igd | 0.0363 | A | \|\| id:ebi-a-GCST90001671 | ebi-a-GCST90001671 | TRUE | G | 95539210 | 5.64E-23 | reported | 3440 | 0.06778 | rs187513283 |  |  |  |  |  |  |  |  |  |  |  |  |  |  |  |
| 0.4459 | 10 | igd | 0.0363 | C | \|\| id:ebi-a-GCST90002030 | ebi-a-GCST90002030 | TRUE | T | 96384689 | 1.81E-09 | reported | 2920 | 0.0739 | rs72816614 |  |  |  |  |  |  |  |  |  |  |  |  |  |  |  |
| 0.7064 | 10 | igd | 0.0363 | T | \|\| id:ebi-a-GCST90002030 | ebi-a-GCST90002030 | TRUE | C | 97482709 | 5.06E-24 | reported | 2920 | 0.06927 | rs142393920 |  |  |  |  |  |  |  |  |  |  |  |  |  |  |  |
| 0.6343 | 10 | igd | 0.0363 | T | \|\| id:ebi-a-GCST90002031 | ebi-a-GCST90002031 | TRUE | C | 97482709 | 1.19E-18 | reported | 2920 | 0.07146 | rs142393920 |  |  |  |  |  |  |  |  |  |  |  |  |  |  |  |
| 0.4184 | 10 | igd | 0.0363 | C | \|\| id:ebi-a-GCST90002031 | ebi-a-GCST90002031 | TRUE | T | 96384689 | 3.57E-08 | reported | 2920 | 0.07571 | rs72816614 |  |  |  |  |  |  |  |  |  |  |  |  |  |  |  |
| 0.6432 | 10 | igd | 0.0363 | T | \|\| id:ebi-a-GCST90002032 | ebi-a-GCST90002032 | TRUE | C | 97482709 | 3.51E-19 | reported | 2920 | 0.07135 | rs142393920 |  |  |  |  |  |  |  |  |  |  |  |  |  |  |  |
| 0.4963 | 10 | igd | 0.0363 | C | \|\| id:ebi-a-GCST90002032 | ebi-a-GCST90002032 | TRUE | T | 96384689 | 5.80E-11 | reported | 2920 | 0.07551 | rs72816614 |  |  |  |  |  |  |  |  |  |  |  |  |  |  |  |
| 0.4152 | 10 | igd | 0.0361 | A | \|\| id:ebi-a-GCST90001483 | ebi-a-GCST90001483 | TRUE | G | 95539210 | 8.22E-10 | reported | 3405 | 0.06742 | rs187513283 |  |  |  |  |  |  |  |  |  |  |  |  |  |  |  |
| 0.4471 | 10 | igd | 0.0361 | A | \|\| id:ebi-a-GCST90001489 | ebi-a-GCST90001489 | TRUE | G | 95539210 | 1.21E-10 | reported | 3405 | 0.06922 | rs187513283 |  |  |  |  |  |  |  |  |  |  |  |  |  |  |  |
| 0.5056 | 10 | igd | 0.0361 | A | \|\| id:ebi-a-GCST90001495 | ebi-a-GCST90001495 | TRUE | G | 95539210 | 4.87E-13 | reported | 3405 | 0.06966 | rs187513283 |  |  |  |  |  |  |  |  |  |  |  |  |  |  |  |
| 0.6052 | 10 | igd | 0.0361 | A | \|\| id:ebi-a-GCST90001660 | ebi-a-GCST90001660 | TRUE | G | 95539210 | 1.60E-18 | reported | 3408 | 0.06851 | rs187513283 |  |  |  |  |  |  |  |  |  |  |  |  |  |  |  |
| 0.7513 | 10 | igd | 0.0361 | A | \|\| id:ebi-a-GCST90001672 | ebi-a-GCST90001672 | TRUE | G | 95539210 | 2.52E-27 | reported | 3408 | 0.06878 | rs187513283 |  |  |  |  |  |  |  |  |  |  |  |  |  |  |  |
| -0.4313 | 2 | igd | 0.036 | T | \|\| id:ebi-a-GCST90001893 | ebi-a-GCST90001893 | TRUE | C | 204479766 | 5.63E-09 | reported | 2920 | 0.07379 | rs151070587 |  |  |  |  |  |  |  |  |  |  |  |  |  |  |  |
| 0.5803 | 6 | igd | 0.036 | G | \|\| id:ebi-a-GCST90002105 | ebi-a-GCST90002105 | TRUE | A | 29182355 | 1.00E-13 | reported | 2872 | 0.07761 | rs115094999 |  |  |  |  |  |  |  |  |  |  |  |  |  |  |  |
| 0.4901 | 2 | igd | 0.0359 | T | \|\| id:ebi-a-GCST90001508 | ebi-a-GCST90001508 | TRUE | C | 39164453 | 2.27E-12 | reported | 3437 | 0.06959 | rs183176024 |  |  |  |  |  |  |  |  |  |  |  |  |  |  |  |
| 0.5425 | 2 | igd | 0.0359 | T | \|\| id:ebi-a-GCST90001508 | ebi-a-GCST90001508 | TRUE | C | 38596674 | 3.75E-14 | reported | 3437 | 0.07137 | rs145947216 |  |  |  |  |  |  |  |  |  |  |  |  |  |  |  |
| 0.5155 | 2 | igd | 0.0359 | T | \|\| id:ebi-a-GCST90001509 | ebi-a-GCST90001509 | TRUE | C | 38596674 | 6.30E-13 | reported | 3437 | 0.07139 | rs145947216 |  |  |  |  |  |  |  |  |  |  |  |  |  |  |  |
| 0.4499 | 2 | igd | 0.0359 | T | \|\| id:ebi-a-GCST90001509 | ebi-a-GCST90001509 | TRUE | C | 39164453 | 1.18E-10 | reported | 3437 | 0.06963 | rs183176024 |  |  |  |  |  |  |  |  |  |  |  |  |  |  |  |
| 0.3702 | 10 | igd | 0.0358 | G | \|\| id:ebi-a-GCST90001497 | ebi-a-GCST90001497 | TRUE | A | 92011043 | 4.98E-08 | reported | 3437 | 0.06776 | rs370666890 |  |  |  |  |  |  |  |  |  |  |  |  |  |  |  |
| 0.3912 | 10 | igd | 0.0358 | G | \|\| id:ebi-a-GCST90001659 | ebi-a-GCST90001659 | TRUE | A | 92011043 | 2.39E-09 | reported | 3440 | 0.06537 | rs370666890 |  |  |  |  |  |  |  |  |  |  |  |  |  |  |  |
| 0.374 | 6 | igd | 0.0358 | G | \|\| id:ebi-a-GCST90001989 | ebi-a-GCST90001989 | TRUE | C | 22713558 | 2.29E-08 | reported | 3621 | 0.06677 | rs77774351 |  |  |  |  |  |  |  |  |  |  |  |  |  |  |  |
| -0.4244 | 10 | igd | 0.0358 | G | \|\| id:ebi-a-GCST90002030 | ebi-a-GCST90002030 | TRUE | A | 98751438 | 6.54E-09 | reported | 2920 | 0.07292 | rs74529649 |  |  |  |  |  |  |  |  |  |  |  |  |  |  |  |
| 0.4547 | 10 | igd | 0.0358 | C | \|\| id:ebi-a-GCST90002030 | ebi-a-GCST90002030 | TRUE | T | 98447228 | 4.18E-10 | reported | 2920 | 0.07253 | rs143190403 |  |  |  |  |  |  |  |  |  |  |  |  |  |  |  |
| 0.4424 | 10 | igd | 0.0358 | C | \|\| id:ebi-a-GCST90002032 | ebi-a-GCST90002032 | TRUE | T | 98447228 | 3.10E-09 | reported | 2920 | 0.07443 | rs143190403 |  |  |  |  |  |  |  |  |  |  |  |  |  |  |  |
| 0.4533 | 2 | igd | 0.0357 | T | \|\| id:ebi-a-GCST90001507 | ebi-a-GCST90001507 | TRUE | C | 38596674 | 5.59E-10 | reported | 3405 | 0.07288 | rs145947216 |  |  |  |  |  |  |  |  |  |  |  |  |  |  |  |
| 0.5102 | 1 | igd | 0.0357 | T | \|\| id:ebi-a-GCST90001911 | ebi-a-GCST90001911 | TRUE | C | 25076059 | 6.88E-12 | reported | 3113 | 0.07408 | rs183053322 |  |  |  |  |  |  |  |  |  |  |  |  |  |  |  |
| 0.4073 | 10 | igd | 0.0356 | G | \|\| id:ebi-a-GCST90001484 | ebi-a-GCST90001484 | TRUE | T | 93293038 | 2.70E-09 | reported | 3437 | 0.06828 | rs560463716 |  |  |  |  |  |  |  |  |  |  |  |  |  |  |  |
| 0.368 | 10 | igd | 0.0356 | G | \|\| id:ebi-a-GCST90001659 | ebi-a-GCST90001659 | TRUE | A | 92573140 | 3.20E-08 | reported | 3440 | 0.06639 | rs77141476 |  |  |  |  |  |  |  |  |  |  |  |  |  |  |  |
| -0.4155 | 10 | igd | 0.0355 | T | \|\| id:ebi-a-GCST90001489 | ebi-a-GCST90001489 | TRUE | C | 97544553 | 9.21E-10 | reported | 3405 | 0.06767 | rs35360884 |  |  |  |  |  |  |  |  |  |  |  |  |  |  |  |
| -0.4706 | 10 | igd | 0.0355 | T | \|\| id:ebi-a-GCST90001495 | ebi-a-GCST90001495 | TRUE | C | 97544553 | 5.98E-12 | reported | 3405 | 0.06816 | rs35360884 |  |  |  |  |  |  |  |  |  |  |  |  |  |  |  |
| -0.477 | 10 | igd | 0.0355 | T | \|\| id:ebi-a-GCST90001660 | ebi-a-GCST90001660 | TRUE | C | 97544553 | 1.73E-12 | reported | 3408 | 0.06736 | rs35360884 |  |  |  |  |  |  |  |  |  |  |  |  |  |  |  |
| -0.4495 | 10 | igd | 0.0355 | T | \|\| id:ebi-a-GCST90001672 | ebi-a-GCST90001672 | TRUE | C | 97544553 | 4.68E-11 | reported | 3408 | 0.06809 | rs35360884 |  |  |  |  |  |  |  |  |  |  |  |  |  |  |  |
| -0.5301 | 19 | igd | 0.0355 | C | \|\| id:ebi-a-GCST90001946 | ebi-a-GCST90001946 | TRUE | G | 50051648 | 3.57E-08 | reported | 1634 | 0.09573 | rs147195142 |  |  |  |  |  |  |  |  |  |  |  |  |  |  |  |
| -1.137 | 6 | igd | 0.0355 | T | \|\| id:ebi-a-GCST90002108 | ebi-a-GCST90002108 | TRUE | C | 32528431 | 4.38E-10 | reported | 1579 | 0.1811 | rs71536556 |  |  |  |  |  |  |  |  |  |  |  |  |  |  |  |
| -0.377 | 10 | igd | 0.0354 | T | \|\| id:ebi-a-GCST90001484 | ebi-a-GCST90001484 | TRUE | C | 97544553 | 9.25E-09 | reported | 3437 | 0.06547 | rs35360884 |  |  |  |  |  |  |  |  |  |  |  |  |  |  |  |
| -0.3606 | 10 | igd | 0.0354 | T | \|\| id:ebi-a-GCST90001485 | ebi-a-GCST90001485 | TRUE | C | 97544553 | 3.77E-08 | reported | 3437 | 0.06541 | rs35360884 |  |  |  |  |  |  |  |  |  |  |  |  |  |  |  |
| 0.3875 | 10 | igd | 0.0354 | A | \|\| id:ebi-a-GCST90001489 | ebi-a-GCST90001489 | TRUE | G | 92414536 | 4.88E-08 | reported | 3405 | 0.07086 | rs6583720 |  |  |  |  |  |  |  |  |  |  |  |  |  |  |  |
| -0.4227 | 10 | igd | 0.0354 | T | \|\| id:ebi-a-GCST90001491 | ebi-a-GCST90001491 | TRUE | C | 97544553 | 2.53E-10 | reported | 3437 | 0.06663 | rs35360884 |  |  |  |  |  |  |  |  |  |  |  |  |  |  |  |
| -0.513 | 10 | igd | 0.0354 | T | \|\| id:ebi-a-GCST90001496 | ebi-a-GCST90001496 | TRUE | C | 97544553 | 3.33E-14 | reported | 3437 | 0.06735 | rs35360884 |  |  |  |  |  |  |  |  |  |  |  |  |  |  |  |
| -0.5031 | 10 | igd | 0.0354 | T | \|\| id:ebi-a-GCST90001497 | ebi-a-GCST90001497 | TRUE | C | 97544553 | 4.98E-14 | reported | 3437 | 0.06651 | rs35360884 |  |  |  |  |  |  |  |  |  |  |  |  |  |  |  |
| 0.4391 | 2 | igd | 0.0354 | T | \|\| id:ebi-a-GCST90001507 | ebi-a-GCST90001507 | TRUE | C | 39164453 | 7.17E-10 | reported | 3405 | 0.07106 | rs183176024 |  |  |  |  |  |  |  |  |  |  |  |  |  |  |  |
| 0.3973 | 10 | igd | 0.0354 | A | \|\| id:ebi-a-GCST90001672 | ebi-a-GCST90001672 | TRUE | G | 98210668 | 1.73E-08 | reported | 3408 | 0.07032 | rs145977486 |  |  |  |  |  |  |  |  |  |  |  |  |  |  |  |
| -0.4879 | 10 | igd | 0.0353 | T | \|\| id:ebi-a-GCST90001658 | ebi-a-GCST90001658 | TRUE | C | 97544553 | 3.89E-14 | reported | 3440 | 0.06423 | rs35360884 |  |  |  |  |  |  |  |  |  |  |  |  |  |  |  |
| 0.4886 | 10 | igd | 0.0353 | A | \|\| id:ebi-a-GCST90001659 | ebi-a-GCST90001659 | TRUE | G | 98210668 | 1.98E-13 | reported | 3440 | 0.06621 | rs145977486 |  |  |  |  |  |  |  |  |  |  |  |  |  |  |  |
| -0.4988 | 10 | igd | 0.0353 | T | \|\| id:ebi-a-GCST90001659 | ebi-a-GCST90001659 | TRUE | C | 97544553 | 1.05E-14 | reported | 3440 | 0.06421 | rs35360884 |  |  |  |  |  |  |  |  |  |  |  |  |  |  |  |
| -0.467 | 10 | igd | 0.0353 | T | \|\| id:ebi-a-GCST90001670 | ebi-a-GCST90001670 | TRUE | C | 97544553 | 3.59E-12 | reported | 3440 | 0.06693 | rs35360884 |  |  |  |  |  |  |  |  |  |  |  |  |  |  |  |
| -0.4613 | 10 | igd | 0.0353 | T | \|\| id:ebi-a-GCST90001671 | ebi-a-GCST90001671 | TRUE | C | 97544553 | 7.03E-12 | reported | 3440 | 0.06704 | rs35360884 |  |  |  |  |  |  |  |  |  |  |  |  |  |  |  |
| -0.6385 | 6 | igd | 0.0353 | G | \|\| id:ebi-a-GCST90001773 | ebi-a-GCST90001773 | TRUE | A | 107185544 | 1.49E-21 | reported | 3652 | 0.06654 | rs62427566 |  |  |  |  |  |  |  |  |  |  |  |  |  |  |  |
| 0.6905 | 2 | igd | 0.0353 | A | \|\| id:ebi-a-GCST90002102 | ebi-a-GCST90002102 | TRUE | G | 38713988 | 1.98E-22 | reported | 2919 | 0.07029 | rs62142582 |  |  |  |  |  |  |  |  |  |  |  |  |  |  |  |
| 0.392 | 10 | igd | 0.0352 | A | \|\| id:ebi-a-GCST90001489 | ebi-a-GCST90001489 | TRUE | G | 98210668 | 2.22E-08 | reported | 3405 | 0.0699 | rs145977486 |  |  |  |  |  |  |  |  |  |  |  |  |  |  |  |
| 0.382 | 10 | igd | 0.0352 | A | \|\| id:ebi-a-GCST90001491 | ebi-a-GCST90001491 | TRUE | G | 98210668 | 3.11E-08 | reported | 3437 | 0.06886 | rs145977486 |  |  |  |  |  |  |  |  |  |  |  |  |  |  |  |
| 0.4741 | 10 | igd | 0.0352 | A | \|\| id:ebi-a-GCST90001495 | ebi-a-GCST90001495 | TRUE | G | 98210668 | 1.96E-11 | reported | 3405 | 0.07043 | rs145977486 |  |  |  |  |  |  |  |  |  |  |  |  |  |  |  |
| 0.4473 | 10 | igd | 0.0352 | C | \|\| id:ebi-a-GCST90001495 | ebi-a-GCST90001495 | TRUE | T | 98447228 | 2.32E-10 | reported | 3405 | 0.07036 | rs143190403 |  |  |  |  |  |  |  |  |  |  |  |  |  |  |  |
| 0.4632 | 10 | igd | 0.0352 | A | \|\| id:ebi-a-GCST90001496 | ebi-a-GCST90001496 | TRUE | G | 98210668 | 3.38E-11 | reported | 3437 | 0.06965 | rs145977486 |  |  |  |  |  |  |  |  |  |  |  |  |  |  |  |
| 0.4808 | 10 | igd | 0.0352 | A | \|\| id:ebi-a-GCST90001497 | ebi-a-GCST90001497 | TRUE | G | 98210668 | 3.15E-12 | reported | 3437 | 0.06873 | rs145977486 |  |  |  |  |  |  |  |  |  |  |  |  |  |  |  |
| 0.423 | 10 | igd | 0.0352 | C | \|\| id:ebi-a-GCST90001658 | ebi-a-GCST90001658 | TRUE | A | 98166522 | 2.16E-10 | reported | 3440 | 0.06642 | rs140492060 |  |  |  |  |  |  |  |  |  |  |  |  |  |  |  |
| 0.4777 | 10 | igd | 0.0352 | C | \|\| id:ebi-a-GCST90001660 | ebi-a-GCST90001660 | TRUE | A | 98166522 | 7.79E-12 | reported | 3408 | 0.06958 | rs140492060 |  |  |  |  |  |  |  |  |  |  |  |  |  |  |  |
| -1.015 | 6 | igd | 0.0352 | T | \|\| id:ebi-a-GCST90002112 | ebi-a-GCST90002112 | TRUE | C | 32528431 | 1.63E-08 | reported | 1635 | 0.1789 | rs71536556 |  |  |  |  |  |  |  |  |  |  |  |  |  |  |  |
| 0.418 | 10 | igd | 0.0351 | C | \|\| id:ebi-a-GCST90001484 | ebi-a-GCST90001484 | TRUE | A | 98166522 | 6.71E-10 | reported | 3437 | 0.06752 | rs140492060 |  |  |  |  |  |  |  |  |  |  |  |  |  |  |  |
| -0.4472 | 22 | igd | 0.0351 | T | \|\| id:ebi-a-GCST90001702 | ebi-a-GCST90001702 | TRUE | C | 44827293 | 3.53E-11 | reported | 3657 | 0.06733 | rs115860798 |  |  |  |  |  |  |  |  |  |  |  |  |  |  |  |
| -0.463 | 22 | igd | 0.0351 | T | \|\| id:ebi-a-GCST90001703 | ebi-a-GCST90001703 | TRUE | C | 44827293 | 6.99E-12 | reported | 3657 | 0.0673 | rs115860798 |  |  |  |  |  |  |  |  |  |  |  |  |  |  |  |
| 0.6602 | 2 | igd | 0.0351 | G | \|\| id:ebi-a-GCST90002098 | ebi-a-GCST90002098 | TRUE | C | 38955419 | 2.53E-20 | reported | 2910 | 0.07094 | rs182240059 |  |  |  |  |  |  |  |  |  |  |  |  |  |  |  |
| 0.6625 | 2 | igd | 0.0351 | A | \|\| id:ebi-a-GCST90002098 | ebi-a-GCST90002098 | TRUE | G | 38713988 | 2.44E-20 | reported | 2910 | 0.07116 | rs62142582 |  |  |  |  |  |  |  |  |  |  |  |  |  |  |  |
| 0.4462 | 2 | igd | 0.0351 | G | \|\| id:ebi-a-GCST90002100 | ebi-a-GCST90002100 | TRUE | C | 38955419 | 1.60E-09 | reported | 2910 | 0.07371 | rs182240059 |  |  |  |  |  |  |  |  |  |  |  |  |  |  |  |
| 0.4099 | 2 | igd | 0.0351 | A | \|\| id:ebi-a-GCST90002100 | ebi-a-GCST90002100 | TRUE | G | 38713988 | 3.28E-08 | reported | 2910 | 0.07397 | rs62142582 |  |  |  |  |  |  |  |  |  |  |  |  |  |  |  |
| 0.6855 | 2 | igd | 0.0351 | G | \|\| id:ebi-a-GCST90002102 | ebi-a-GCST90002102 | TRUE | C | 38955419 | 3.90E-22 | reported | 2919 | 0.07029 | rs182240059 |  |  |  |  |  |  |  |  |  |  |  |  |  |  |  |
| 0.5011 | 3 | igd | 0.035 | A | \|\| id:ebi-a-GCST90001798 | ebi-a-GCST90001798 | TRUE | G | 32945761 | 1.07E-13 | reported | 3657 | 0.06717 | rs75918255 |  |  |  |  |  |  |  |  |  |  |  |  |  |  |  |
| 0.4946 | 3 | igd | 0.035 | A | \|\| id:ebi-a-GCST90001800 | ebi-a-GCST90001800 | TRUE | G | 32945761 | 5.54E-13 | reported | 3656 | 0.06834 | rs75918255 |  |  |  |  |  |  |  |  |  |  |  |  |  |  |  |
| 0.4158 | 3 | igd | 0.035 | A | \|\| id:ebi-a-GCST90001802 | ebi-a-GCST90001802 | TRUE | G | 32945761 | 1.20E-09 | reported | 3657 | 0.06821 | rs75918255 |  |  |  |  |  |  |  |  |  |  |  |  |  |  |  |
| 0.4177 | 3 | igd | 0.035 | A | \|\| id:ebi-a-GCST90001804 | ebi-a-GCST90001804 | TRUE | G | 32945761 | 4.84E-10 | reported | 3657 | 0.06693 | rs75918255 |  |  |  |  |  |  |  |  |  |  |  |  |  |  |  |
| 0.4366 | 3 | igd | 0.035 | A | \|\| id:ebi-a-GCST90001805 | ebi-a-GCST90001805 | TRUE | G | 32945761 | 1.57E-10 | reported | 3657 | 0.06803 | rs75918255 |  |  |  |  |  |  |  |  |  |  |  |  |  |  |  |
| 0.5086 | 3 | igd | 0.035 | A | \|\| id:ebi-a-GCST90001806 | ebi-a-GCST90001806 | TRUE | G | 32945761 | 9.62E-14 | reported | 3656 | 0.06804 | rs75918255 |  |  |  |  |  |  |  |  |  |  |  |  |  |  |  |
| 0.478 | 3 | igd | 0.035 | A | \|\| id:ebi-a-GCST90001808 | ebi-a-GCST90001808 | TRUE | G | 32945761 | 2.09E-12 | reported | 3657 | 0.06777 | rs75918255 |  |  |  |  |  |  |  |  |  |  |  |  |  |  |  |
| 0.4585 | 10 | igd | 0.0349 | C | \|\| id:ebi-a-GCST90001496 | ebi-a-GCST90001496 | TRUE | T | 96384689 | 8.87E-11 | reported | 3437 | 0.07048 | rs72816614 |  |  |  |  |  |  |  |  |  |  |  |  |  |  |  |
| 0.4441 | 10 | igd | 0.0349 | C | \|\| id:ebi-a-GCST90001497 | ebi-a-GCST90001497 | TRUE | T | 96384689 | 2.02E-10 | reported | 3437 | 0.06961 | rs72816614 |  |  |  |  |  |  |  |  |  |  |  |  |  |  |  |
| -0.4618 | 22 | igd | 0.0349 | A | \|\| id:ebi-a-GCST90001702 | ebi-a-GCST90001702 | TRUE | G | 42176283 | 1.15E-11 | reported | 3657 | 0.06783 | rs117886891 |  |  |  |  |  |  |  |  |  |  |  |  |  |  |  |
| -0.4471 | 22 | igd | 0.0349 | A | \|\| id:ebi-a-GCST90001703 | ebi-a-GCST90001703 | TRUE | G | 42176283 | 5.03E-11 | reported | 3657 | 0.06785 | rs117886891 |  |  |  |  |  |  |  |  |  |  |  |  |  |  |  |
| -0.4855 | 22 | igd | 0.0349 | T | \|\| id:ebi-a-GCST90001704 | ebi-a-GCST90001704 | TRUE | C | 44829954 | 7.38E-13 | reported | 3657 | 0.06745 | rs115848830 |  |  |  |  |  |  |  |  |  |  |  |  |  |  |  |
| -0.3914 | 22 | igd | 0.0349 | A | \|\| id:ebi-a-GCST90001704 | ebi-a-GCST90001704 | TRUE | G | 42176283 | 8.72E-09 | reported | 3657 | 0.06786 | rs117886891 |  |  |  |  |  |  |  |  |  |  |  |  |  |  |  |
| -0.4611 | 22 | igd | 0.0349 | T | \|\| id:ebi-a-GCST90001710 | ebi-a-GCST90001710 | TRUE | C | 44829954 | 8.95E-12 | reported | 3657 | 0.06737 | rs115848830 |  |  |  |  |  |  |  |  |  |  |  |  |  |  |  |
| -0.447 | 22 | igd | 0.0349 | A | \|\| id:ebi-a-GCST90001710 | ebi-a-GCST90001710 | TRUE | G | 42176283 | 4.49E-11 | reported | 3657 | 0.06766 | rs117886891 |  |  |  |  |  |  |  |  |  |  |  |  |  |  |  |
| -0.4857 | 22 | igd | 0.0349 | T | \|\| id:ebi-a-GCST90001711 | ebi-a-GCST90001711 | TRUE | C | 44829954 | 5.93E-13 | reported | 3657 | 0.0672 | rs115848830 |  |  |  |  |  |  |  |  |  |  |  |  |  |  |  |
| -0.4279 | 22 | igd | 0.0349 | A | \|\| id:ebi-a-GCST90001711 | ebi-a-GCST90001711 | TRUE | G | 42176283 | 2.66E-10 | reported | 3657 | 0.06754 | rs117886891 |  |  |  |  |  |  |  |  |  |  |  |  |  |  |  |
| 0.5857 | 10 | igd | 0.0349 | A | \|\| id:ebi-a-GCST90002030 | ebi-a-GCST90002030 | TRUE | G | 98366462 | 1.32E-15 | reported | 2920 | 0.07288 | rs139070116 |  |  |  |  |  |  |  |  |  |  |  |  |  |  |  |
| 0.5188 | 10 | igd | 0.0346 | A | \|\| id:ebi-a-GCST90002030 | ebi-a-GCST90002030 | TRUE | G | 98210668 | 3.07E-12 | reported | 2920 | 0.07407 | rs145977486 |  |  |  |  |  |  |  |  |  |  |  |  |  |  |  |
| 0.4535 | 10 | igd | 0.0346 | A | \|\| id:ebi-a-GCST90002031 | ebi-a-GCST90002031 | TRUE | G | 98210668 | 2.74E-09 | reported | 2920 | 0.07603 | rs145977486 |  |  |  |  |  |  |  |  |  |  |  |  |  |  |  |
| 0.4603 | 10 | igd | 0.0346 | A | \|\| id:ebi-a-GCST90002032 | ebi-a-GCST90002032 | TRUE | G | 98210668 | 1.55E-09 | reported | 2920 | 0.07597 | rs145977486 |  |  |  |  |  |  |  |  |  |  |  |  |  |  |  |
| -0.7147 | 19 | igd | 0.03456 | G | \|\| id:prot-a-439 | prot-a-439 | TRUE | A | 51691888 | 2.51E-21 | reported | 3301 | 0.0754 | rs80237306 |  |  |  |  |  |  |  |  |  |  |  |  |  |  |  |
| 0.4409 | 10 | igd | 0.0345 | C | \|\| id:ebi-a-GCST90001484 | ebi-a-GCST90001484 | TRUE | T | 92120184 | 7.61E-11 | reported | 3437 | 0.06753 | rs1274300 |  |  |  |  |  |  |  |  |  |  |  |  |  |  |  |
| 0.4069 | 10 | igd | 0.0345 | C | \|\| id:ebi-a-GCST90001485 | ebi-a-GCST90001485 | TRUE | T | 92120184 | 1.83E-09 | reported | 3437 | 0.0675 | rs1274300 |  |  |  |  |  |  |  |  |  |  |  |  |  |  |  |
| 0.5091 | 10 | igd | 0.0345 | C | \|\| id:ebi-a-GCST90001489 | ebi-a-GCST90001489 | TRUE | T | 98288920 | 3.04E-13 | reported | 3405 | 0.06953 | rs145910026 |  |  |  |  |  |  |  |  |  |  |  |  |  |  |  |
| 0.4778 | 10 | igd | 0.0345 | C | \|\| id:ebi-a-GCST90001495 | ebi-a-GCST90001495 | TRUE | T | 98288920 | 1.21E-11 | reported | 3405 | 0.07024 | rs145910026 |  |  |  |  |  |  |  |  |  |  |  |  |  |  |  |
| 0.4784 | 10 | igd | 0.0345 | C | \|\| id:ebi-a-GCST90001660 | ebi-a-GCST90001660 | TRUE | T | 98288920 | 6.63E-12 | reported | 3408 | 0.06943 | rs145910026 |  |  |  |  |  |  |  |  |  |  |  |  |  |  |  |
| 0.4196 | 10 | igd | 0.0345 | C | \|\| id:ebi-a-GCST90001672 | ebi-a-GCST90001672 | TRUE | T | 98288920 | 2.55E-09 | reported | 3408 | 0.07024 | rs145910026 |  |  |  |  |  |  |  |  |  |  |  |  |  |  |  |
| 0.3859 | 2 | igd | 0.0345 | T | \|\| id:ebi-a-GCST90002098 | ebi-a-GCST90002098 | TRUE | C | 34554065 | 4.95E-08 | reported | 2910 | 0.07057 | rs77418694 |  |  |  |  |  |  |  |  |  |  |  |  |  |  |  |
| 0.3962 | 10 | igd | 0.0343 | C | \|\| id:ebi-a-GCST90001484 | ebi-a-GCST90001484 | TRUE | T | 98288920 | 4.67E-09 | reported | 3437 | 0.06746 | rs145910026 |  |  |  |  |  |  |  |  |  |  |  |  |  |  |  |
| 0.496 | 10 | igd | 0.0343 | C | \|\| id:ebi-a-GCST90001491 | ebi-a-GCST90001491 | TRUE | T | 98288920 | 5.92E-13 | reported | 3437 | 0.06861 | rs145910026 |  |  |  |  |  |  |  |  |  |  |  |  |  |  |  |
| 0.4195 | 10 | igd | 0.0343 | C | \|\| id:ebi-a-GCST90001496 | ebi-a-GCST90001496 | TRUE | T | 98288920 | 1.93E-09 | reported | 3437 | 0.06969 | rs145910026 |  |  |  |  |  |  |  |  |  |  |  |  |  |  |  |
| 0.4146 | 10 | igd | 0.0343 | C | \|\| id:ebi-a-GCST90001497 | ebi-a-GCST90001497 | TRUE | T | 98288920 | 1.86E-09 | reported | 3437 | 0.06881 | rs145910026 |  |  |  |  |  |  |  |  |  |  |  |  |  |  |  |
| 0.5195 | 10 | igd | 0.0343 | C | \|\| id:ebi-a-GCST90001658 | ebi-a-GCST90001658 | TRUE | T | 98288920 | 5.73E-15 | reported | 3440 | 0.06622 | rs145910026 |  |  |  |  |  |  |  |  |  |  |  |  |  |  |  |
| 0.5198 | 10 | igd | 0.0343 | C | \|\| id:ebi-a-GCST90001659 | ebi-a-GCST90001659 | TRUE | T | 98288920 | 5.56E-15 | reported | 3440 | 0.06623 | rs145910026 |  |  |  |  |  |  |  |  |  |  |  |  |  |  |  |
| 0.4287 | 10 | igd | 0.0343 | C | \|\| id:ebi-a-GCST90001670 | ebi-a-GCST90001670 | TRUE | T | 98288920 | 6.32E-10 | reported | 3440 | 0.06914 | rs145910026 |  |  |  |  |  |  |  |  |  |  |  |  |  |  |  |
| 0.4091 | 10 | igd | 0.0343 | C | \|\| id:ebi-a-GCST90001671 | ebi-a-GCST90001671 | TRUE | T | 98288920 | 3.86E-09 | reported | 3440 | 0.06928 | rs145910026 |  |  |  |  |  |  |  |  |  |  |  |  |  |  |  |
| -0.5672 | 19 | igd | 0.0343 | A | \|\| id:ebi-a-GCST90001949 | ebi-a-GCST90001949 | TRUE | G | 52115646 | 2.41E-08 | reported | 1634 | 0.1011 | rs61736483 |  |  |  |  |  |  |  |  |  |  |  |  |  |  |  |
| 0.3856 | 10 | igd | 0.0341 | C | \|\| id:ebi-a-GCST90001483 | ebi-a-GCST90001483 | TRUE | T | 92120184 | 2.02E-08 | reported | 3405 | 0.06858 | rs1274300 |  |  |  |  |  |  |  |  |  |  |  |  |  |  |  |
| -0.3884 | 10 | igd | 0.0341 | A | \|\| id:ebi-a-GCST90001495 | ebi-a-GCST90001495 | TRUE | G | 97370768 | 2.59E-08 | reported | 3405 | 0.0696 | rs185394326 |  |  |  |  |  |  |  |  |  |  |  |  |  |  |  |
| 0.491 | 17 | igd | 0.0341 | A | \|\| id:ebi-a-GCST90001964 | ebi-a-GCST90001964 | TRUE | G | 62368885 | 1.22E-10 | reported | 2872 | 0.07599 | rs117402740 |  |  |  |  |  |  |  |  |  |  |  |  |  |  |  |
| -0.3676 | 10 | igd | 0.034 | A | \|\| id:ebi-a-GCST90001484 | ebi-a-GCST90001484 | TRUE | G | 97370768 | 4.22E-08 | reported | 3437 | 0.06691 | rs185394326 |  |  |  |  |  |  |  |  |  |  |  |  |  |  |  |
| -0.3829 | 10 | igd | 0.034 | G | \|\| id:ebi-a-GCST90001491 | ebi-a-GCST90001491 | TRUE | A | 97394685 | 1.81E-08 | reported | 3437 | 0.06785 | rs118183340 |  |  |  |  |  |  |  |  |  |  |  |  |  |  |  |
| -0.433 | 10 | igd | 0.034 | A | \|\| id:ebi-a-GCST90001496 | ebi-a-GCST90001496 | TRUE | G | 97370768 | 3.30E-10 | reported | 3437 | 0.0687 | rs185394326 |  |  |  |  |  |  |  |  |  |  |  |  |  |  |  |
| -0.4145 | 10 | igd | 0.034 | A | \|\| id:ebi-a-GCST90001497 | ebi-a-GCST90001497 | TRUE | G | 97370768 | 1.12E-09 | reported | 3437 | 0.06786 | rs185394326 |  |  |  |  |  |  |  |  |  |  |  |  |  |  |  |
| -0.4085 | 10 | igd | 0.034 | A | \|\| id:ebi-a-GCST90001658 | ebi-a-GCST90001658 | TRUE | G | 97370768 | 4.80E-10 | reported | 3440 | 0.06543 | rs185394326 |  |  |  |  |  |  |  |  |  |  |  |  |  |  |  |
| -0.4122 | 10 | igd | 0.034 | A | \|\| id:ebi-a-GCST90001659 | ebi-a-GCST90001659 | TRUE | G | 97370768 | 3.37E-10 | reported | 3440 | 0.06543 | rs185394326 |  |  |  |  |  |  |  |  |  |  |  |  |  |  |  |
| -0.4128 | 10 | igd | 0.034 | A | \|\| id:ebi-a-GCST90001660 | ebi-a-GCST90001660 | TRUE | G | 97370768 | 2.05E-09 | reported | 3408 | 0.06868 | rs185394326 |  |  |  |  |  |  |  |  |  |  |  |  |  |  |  |
| -0.3821 | 10 | igd | 0.034 | A | \|\| id:ebi-a-GCST90001670 | ebi-a-GCST90001670 | TRUE | G | 97370768 | 2.34E-08 | reported | 3440 | 0.06826 | rs185394326 |  |  |  |  |  |  |  |  |  |  |  |  |  |  |  |
| -0.3924 | 10 | igd | 0.034 | A | \|\| id:ebi-a-GCST90001672 | ebi-a-GCST90001672 | TRUE | G | 97370768 | 1.69E-08 | reported | 3408 | 0.06939 | rs185394326 |  |  |  |  |  |  |  |  |  |  |  |  |  |  |  |
| -0.8831 | 19 | igd | 0.034 | A | \|\| id:ebi-a-GCST90001946 | ebi-a-GCST90001946 | TRUE | T | 51732079 | 1.16E-19 | reported | 1634 | 0.09609 | rs149201050 |  |  |  |  |  |  |  |  |  |  |  |  |  |  |  |
| -0.8808 | 19 | igd | 0.034 | A | \|\| id:ebi-a-GCST90001948 | ebi-a-GCST90001948 | TRUE | T | 51732079 | 7.33E-20 | reported | 1633 | 0.09531 | rs149201050 |  |  |  |  |  |  |  |  |  |  |  |  |  |  |  |
| -0.8535 | 19 | igd | 0.034 | A | \|\| id:ebi-a-GCST90001949 | ebi-a-GCST90001949 | TRUE | T | 51732079 | 9.11E-19 | reported | 1634 | 0.09532 | rs149201050 |  |  |  |  |  |  |  |  |  |  |  |  |  |  |  |
| 0.4119 | 20 | igd | 0.034 | T | \|\| id:ebi-a-GCST90001989 | ebi-a-GCST90001989 | TRUE | C | 42701487 | 2.63E-09 | reported | 3621 | 0.06902 | rs111436326 |  |  |  |  |  |  |  |  |  |  |  |  |  |  |  |
| -0.5361 | 10 | igd | 0.0339 | T | \|\| id:ebi-a-GCST90002030 | ebi-a-GCST90002030 | TRUE | C | 97544553 | 1.72E-13 | reported | 2920 | 0.0724 | rs35360884 |  |  |  |  |  |  |  |  |  |  |  |  |  |  |  |
| -0.4301 | 10 | igd | 0.0339 | T | \|\| id:ebi-a-GCST90002031 | ebi-a-GCST90002031 | TRUE | C | 97544553 | 8.39E-09 | reported | 2920 | 0.07444 | rs35360884 |  |  |  |  |  |  |  |  |  |  |  |  |  |  |  |
| -0.454 | 10 | igd | 0.0339 | T | \|\| id:ebi-a-GCST90002032 | ebi-a-GCST90002032 | TRUE | C | 97544553 | 1.14E-09 | reported | 2920 | 0.07433 | rs35360884 |  |  |  |  |  |  |  |  |  |  |  |  |  |  |  |
| -0.5832 | 12 | igd | 0.0339 | C | \|\| id:ebi-a-GCST90002073 | ebi-a-GCST90002073 | TRUE | T | 70906075 | 8.69E-15 | reported | 2850 | 0.07478 | rs61930264 |  |  |  |  |  |  |  |  |  |  |  |  |  |  |  |
| 0.4884 | 2 | igd | 0.0336 | A | \|\| id:ebi-a-GCST90001508 | ebi-a-GCST90001508 | TRUE | G | 38713988 | 1.23E-11 | reported | 3437 | 0.07182 | rs62142582 |  |  |  |  |  |  |  |  |  |  |  |  |  |  |  |
| 0.445 | 2 | igd | 0.0336 | A | \|\| id:ebi-a-GCST90001509 | ebi-a-GCST90001509 | TRUE | G | 38713988 | 6.60E-10 | reported | 3437 | 0.07186 | rs62142582 |  |  |  |  |  |  |  |  |  |  |  |  |  |  |  |
| 0.4998 | 10 | igd | 0.0336 | C | \|\| id:ebi-a-GCST90001658 | ebi-a-GCST90001658 | TRUE | T | 98406362 | 4.85E-13 | reported | 3440 | 0.06887 | rs78573405 |  |  |  |  |  |  |  |  |  |  |  |  |  |  |  |
| 0.516 | 10 | igd | 0.0336 | C | \|\| id:ebi-a-GCST90001659 | ebi-a-GCST90001659 | TRUE | T | 98406362 | 8.34E-14 | reported | 3440 | 0.06884 | rs78573405 |  |  |  |  |  |  |  |  |  |  |  |  |  |  |  |
| 0.5652 | 10 | igd | 0.0336 | C | \|\| id:ebi-a-GCST90001660 | ebi-a-GCST90001660 | TRUE | T | 98406362 | 5.39E-15 | reported | 3408 | 0.07197 | rs78573405 |  |  |  |  |  |  |  |  |  |  |  |  |  |  |  |
| 0.4076 | 10 | igd | 0.0336 | C | \|\| id:ebi-a-GCST90001670 | ebi-a-GCST90001670 | TRUE | T | 98406362 | 1.50E-08 | reported | 3440 | 0.07182 | rs78573405 |  |  |  |  |  |  |  |  |  |  |  |  |  |  |  |
| 0.4461 | 10 | igd | 0.0336 | C | \|\| id:ebi-a-GCST90001671 | ebi-a-GCST90001671 | TRUE | T | 98406362 | 6.22E-10 | reported | 3440 | 0.07193 | rs78573405 |  |  |  |  |  |  |  |  |  |  |  |  |  |  |  |
| 0.4547 | 10 | igd | 0.0336 | C | \|\| id:ebi-a-GCST90001672 | ebi-a-GCST90001672 | TRUE | T | 98406362 | 5.05E-10 | reported | 3408 | 0.07291 | rs78573405 |  |  |  |  |  |  |  |  |  |  |  |  |  |  |  |
| -0.4049 | 22 | igd | 0.0336 | G | \|\| id:ebi-a-GCST90001702 | ebi-a-GCST90001702 | TRUE | A | 38091241 | 1.04E-08 | reported | 3657 | 0.07057 | rs188662274 |  |  |  |  |  |  |  |  |  |  |  |  |  |  |  |
| -0.3945 | 22 | igd | 0.0336 | G | \|\| id:ebi-a-GCST90001703 | ebi-a-GCST90001703 | TRUE | A | 38091241 | 2.44E-08 | reported | 3657 | 0.07057 | rs188662274 |  |  |  |  |  |  |  |  |  |  |  |  |  |  |  |
| -0.4082 | 22 | igd | 0.0336 | G | \|\| id:ebi-a-GCST90001710 | ebi-a-GCST90001710 | TRUE | A | 38091241 | 7.16E-09 | reported | 3657 | 0.07037 | rs188662274 |  |  |  |  |  |  |  |  |  |  |  |  |  |  |  |
| -0.4034 | 22 | igd | 0.0336 | G | \|\| id:ebi-a-GCST90001711 | ebi-a-GCST90001711 | TRUE | A | 38091241 | 9.85E-09 | reported | 3657 | 0.0702 | rs188662274 |  |  |  |  |  |  |  |  |  |  |  |  |  |  |  |
| -0.6032 | 19 | igd | 0.0336 | A | \|\| id:ebi-a-GCST90001947 | ebi-a-GCST90001947 | TRUE | G | 52115646 | 1.68E-08 | reported | 1577 | 0.1063 | rs61736483 |  |  |  |  |  |  |  |  |  |  |  |  |  |  |  |
| -0.6047 | 19 | igd | 0.0336 | A | \|\| id:ebi-a-GCST90001956 | ebi-a-GCST90001956 | TRUE | G | 52115646 | 1.49E-08 | reported | 1579 | 0.1062 | rs61736483 |  |  |  |  |  |  |  |  |  |  |  |  |  |  |  |
| -0.6121 | 19 | igd | 0.0336 | A | \|\| id:ebi-a-GCST90001957 | ebi-a-GCST90001957 | TRUE | G | 52115646 | 1.02E-08 | reported | 1578 | 0.1063 | rs61736483 |  |  |  |  |  |  |  |  |  |  |  |  |  |  |  |
| 0.5319 | 10 | igd | 0.0336 | C | \|\| id:ebi-a-GCST90002031 | ebi-a-GCST90002031 | TRUE | T | 98288920 | 4.21E-12 | reported | 2920 | 0.07643 | rs145910026 |  |  |  |  |  |  |  |  |  |  |  |  |  |  |  |
| 0.5523 | 10 | igd | 0.0336 | C | \|\| id:ebi-a-GCST90002032 | ebi-a-GCST90002032 | TRUE | T | 98288920 | 6.04E-13 | reported | 2920 | 0.07637 | rs145910026 |  |  |  |  |  |  |  |  |  |  |  |  |  |  |  |
| -0.4165 | 3 | igd | 0.0336 | T | \|\| id:ebi-a-GCST90002004 | ebi-a-GCST90002004 | TRUE | G | 46880193 | 1.02E-09 | reported | 3629 | 0.06803 | rs79825014 |  |  |  |  |  |  |  |  |  |  |  |  |  |  |  |
| -0.4085 | 3 | igd | 0.0336 | C | \|\| id:ebi-a-GCST90002008 | ebi-a-GCST90002008 | TRUE | A | 46882338 | 3.13E-09 | reported | 3629 | 0.06877 | rs79641389 |  |  |  |  |  |  |  |  |  |  |  |  |  |  |  |
| 0.5396 | 10 | igd | 0.0335 | C | \|\| id:ebi-a-GCST90001483 | ebi-a-GCST90001483 | TRUE | T | 98406362 | 1.84E-14 | reported | 3405 | 0.07012 | rs78573405 |  |  |  |  |  |  |  |  |  |  |  |  |  |  |  |
| 0.6321 | 10 | igd | 0.0335 | C | \|\| id:ebi-a-GCST90001484 | ebi-a-GCST90001484 | TRUE | T | 98406362 | 1.46E-19 | reported | 3437 | 0.06944 | rs78573405 |  |  |  |  |  |  |  |  |  |  |  |  |  |  |  |
| 0.5503 | 10 | igd | 0.0335 | C | \|\| id:ebi-a-GCST90001485 | ebi-a-GCST90001485 | TRUE | T | 98406362 | 3.37E-15 | reported | 3437 | 0.06954 | rs78573405 |  |  |  |  |  |  |  |  |  |  |  |  |  |  |  |
| 0.5005 | 10 | igd | 0.0335 | C | \|\| id:ebi-a-GCST90001496 | ebi-a-GCST90001496 | TRUE | T | 98406362 | 5.48E-12 | reported | 3437 | 0.07235 | rs78573405 |  |  |  |  |  |  |  |  |  |  |  |  |  |  |  |
| 0.4847 | 10 | igd | 0.0335 | C | \|\| id:ebi-a-GCST90001497 | ebi-a-GCST90001497 | TRUE | T | 98406362 | 1.39E-11 | reported | 3437 | 0.07147 | rs78573405 |  |  |  |  |  |  |  |  |  |  |  |  |  |  |  |
| -0.7778 | 19 | igd | 0.0335 | A | \|\| id:ebi-a-GCST90001951 | ebi-a-GCST90001951 | TRUE | T | 51732079 | 9.81E-14 | reported | 1464 | 0.1035 | rs149201050 |  |  |  |  |  |  |  |  |  |  |  |  |  |  |  |
| -0.4145 | 1 | igd | 0.0334 | C | \|\| id:ebi-a-GCST90001856 | ebi-a-GCST90001856 | TRUE | T | 167436326 | 3.00E-08 | reported | 2919 | 0.0746 | rs112413378 |  |  |  |  |  |  |  |  |  |  |  |  |  |  |  |
| -0.4879 | 1 | igd | 0.0334 | C | \|\| id:ebi-a-GCST90001864 | ebi-a-GCST90001864 | TRUE | T | 167436326 | 3.87E-11 | reported | 2919 | 0.07354 | rs112413378 |  |  |  |  |  |  |  |  |  |  |  |  |  |  |  |
| -0.4473 | 10 | igd | 0.0334 | C | \|\| id:ebi-a-GCST90002030 | ebi-a-GCST90002030 | TRUE | G | 90688122 | 2.57E-08 | reported | 2920 | 0.08011 | rs75799019 |  |  |  |  |  |  |  |  |  |  |  |  |  |  |  |
| -0.6348 | 10 | igd | 0.0334 | A | \|\| id:ebi-a-GCST90002030 | ebi-a-GCST90002030 | TRUE | G | 97370768 | 4.43E-18 | reported | 2920 | 0.07277 | rs185394326 |  |  |  |  |  |  |  |  |  |  |  |  |  |  |  |
| -0.4167 | 10 | igd | 0.0334 | A | \|\| id:ebi-a-GCST90002031 | ebi-a-GCST90002031 | TRUE | C | 97683080 | 3.17E-08 | reported | 2920 | 0.07513 | rs139847910 |  |  |  |  |  |  |  |  |  |  |  |  |  |  |  |
| -0.5956 | 10 | igd | 0.0334 | A | \|\| id:ebi-a-GCST90002031 | ebi-a-GCST90002031 | TRUE | G | 97370768 | 3.18E-15 | reported | 2920 | 0.07514 | rs185394326 |  |  |  |  |  |  |  |  |  |  |  |  |  |  |  |
| -0.6308 | 10 | igd | 0.0334 | A | \|\| id:ebi-a-GCST90002032 | ebi-a-GCST90002032 | TRUE | G | 97370768 | 5.84E-17 | reported | 2920 | 0.07493 | rs185394326 |  |  |  |  |  |  |  |  |  |  |  |  |  |  |  |
| -0.4112 | 3 | igd | 0.0334 | A | \|\| id:ebi-a-GCST90001996 | ebi-a-GCST90001996 | TRUE | G | 39318192 | 3.11E-09 | reported | 3579 | 0.06922 | rs56039226 |  |  |  |  |  |  |  |  |  |  |  |  |  |  |  |
| 0.5668 | 10 | igd | 0.0333 | C | \|\| id:ebi-a-GCST90001658 | ebi-a-GCST90001658 | TRUE | G | 98776436 | 5.37E-16 | reported | 3440 | 0.06962 | rs61864161 |  |  |  |  |  |  |  |  |  |  |  |  |  |  |  |
| 0.6052 | 10 | igd | 0.0333 | C | \|\| id:ebi-a-GCST90001659 | ebi-a-GCST90001659 | TRUE | G | 98776436 | 4.87E-18 | reported | 3440 | 0.06953 | rs61864161 |  |  |  |  |  |  |  |  |  |  |  |  |  |  |  |
| 0.7082 | 10 | igd | 0.0333 | C | \|\| id:ebi-a-GCST90001660 | ebi-a-GCST90001660 | TRUE | G | 98776436 | 3.18E-22 | reported | 3408 | 0.07255 | rs61864161 |  |  |  |  |  |  |  |  |  |  |  |  |  |  |  |
| 0.4822 | 10 | igd | 0.0333 | C | \|\| id:ebi-a-GCST90001670 | ebi-a-GCST90001670 | TRUE | G | 98776436 | 3.87E-11 | reported | 3440 | 0.07272 | rs61864161 |  |  |  |  |  |  |  |  |  |  |  |  |  |  |  |
| 0.5034 | 10 | igd | 0.0333 | C | \|\| id:ebi-a-GCST90001671 | ebi-a-GCST90001671 | TRUE | G | 98776436 | 5.40E-12 | reported | 3440 | 0.07275 | rs61864161 |  |  |  |  |  |  |  |  |  |  |  |  |  |  |  |
| 0.5678 | 10 | igd | 0.0333 | C | \|\| id:ebi-a-GCST90001672 | ebi-a-GCST90001672 | TRUE | G | 98776436 | 1.64E-14 | reported | 3408 | 0.07364 | rs61864161 |  |  |  |  |  |  |  |  |  |  |  |  |  |  |  |
| -0.7194 | 12 | igd | 0.0333 | G | \|\| id:ebi-a-GCST90002073 | ebi-a-GCST90002073 | TRUE | T | 69363349 | 7.13E-21 | reported | 2850 | 0.07616 | rs116987207 |  |  |  |  |  |  |  |  |  |  |  |  |  |  |  |
| -0.5995 | 12 | igd | 0.0333 | G | \|\| id:ebi-a-GCST90002074 | ebi-a-GCST90002074 | TRUE | T | 69363349 | 5.15E-16 | reported | 3112 | 0.07355 | rs116987207 |  |  |  |  |  |  |  |  |  |  |  |  |  |  |  |
| -0.4824 | 3 | igd | 0.0333 | A | \|\| id:ebi-a-GCST90001997 | ebi-a-GCST90001997 | TRUE | G | 39318192 | 5.44E-12 | reported | 3590 | 0.06975 | rs56039226 |  |  |  |  |  |  |  |  |  |  |  |  |  |  |  |
| -0.4652 | 3 | igd | 0.0333 | A | \|\| id:ebi-a-GCST90001995 | ebi-a-GCST90001995 | TRUE | G | 39318192 | 2.19E-11 | reported | 3590 | 0.06928 | rs56039226 |  |  |  |  |  |  |  |  |  |  |  |  |  |  |  |
| 0.5936 | 10 | igd | 0.0332 | C | \|\| id:ebi-a-GCST90001483 | ebi-a-GCST90001483 | TRUE | G | 98776436 | 1.17E-16 | reported | 3405 | 0.07127 | rs61864161 |  |  |  |  |  |  |  |  |  |  |  |  |  |  |  |
| 0.6216 | 10 | igd | 0.0332 | C | \|\| id:ebi-a-GCST90001484 | ebi-a-GCST90001484 | TRUE | G | 98776436 | 2.40E-18 | reported | 3437 | 0.07075 | rs61864161 |  |  |  |  |  |  |  |  |  |  |  |  |  |  |  |
| 0.5439 | 10 | igd | 0.0332 | C | \|\| id:ebi-a-GCST90001485 | ebi-a-GCST90001485 | TRUE | G | 98776436 | 2.09E-14 | reported | 3437 | 0.07083 | rs61864161 |  |  |  |  |  |  |  |  |  |  |  |  |  |  |  |
| 0.4557 | 10 | igd | 0.0332 | C | \|\| id:ebi-a-GCST90001489 | ebi-a-GCST90001489 | TRUE | G | 98776436 | 6.47E-10 | reported | 3405 | 0.07354 | rs61864161 |  |  |  |  |  |  |  |  |  |  |  |  |  |  |  |
| 0.5802 | 10 | igd | 0.0332 | C | \|\| id:ebi-a-GCST90001495 | ebi-a-GCST90001495 | TRUE | G | 98776436 | 5.36E-15 | reported | 3405 | 0.07387 | rs61864161 |  |  |  |  |  |  |  |  |  |  |  |  |  |  |  |
| 0.5314 | 10 | igd | 0.0332 | C | \|\| id:ebi-a-GCST90001496 | ebi-a-GCST90001496 | TRUE | G | 98776436 | 5.06E-13 | reported | 3437 | 0.07327 | rs61864161 |  |  |  |  |  |  |  |  |  |  |  |  |  |  |  |
| 0.5124 | 10 | igd | 0.0332 | C | \|\| id:ebi-a-GCST90001497 | ebi-a-GCST90001497 | TRUE | G | 98776436 | 1.74E-12 | reported | 3437 | 0.07238 | rs61864161 |  |  |  |  |  |  |  |  |  |  |  |  |  |  |  |
| 0.4325 | 2 | igd | 0.0332 | A | \|\| id:ebi-a-GCST90001507 | ebi-a-GCST90001507 | TRUE | G | 38713988 | 4.01E-09 | reported | 3405 | 0.07332 | rs62142582 |  |  |  |  |  |  |  |  |  |  |  |  |  |  |  |
| -0.4785 | 2 | igd | 0.0332 | G | \|\| id:ebi-a-GCST90002118 | ebi-a-GCST90002118 | TRUE | A | 86750489 | 2.95E-10 | reported | 2920 | 0.07567 | rs151209719 |  |  |  |  |  |  |  |  |  |  |  |  |  |  |  |
| -0.7324 | 2 | igd | 0.0332 | A | \|\| id:ebi-a-GCST90002119 | ebi-a-GCST90002119 | TRUE | G | 86777416 | 5.38E-23 | reported | 2920 | 0.07354 | rs146864932 |  |  |  |  |  |  |  |  |  |  |  |  |  |  |  |
| -1.028 | 19 | igd | 0.033 | A | \|\| id:ebi-a-GCST90001950 | ebi-a-GCST90001950 | TRUE | G | 51770292 | 9.84E-19 | reported | 1244 | 0.1145 | rs11668077 |  |  |  |  |  |  |  |  |  |  |  |  |  |  |  |
| 0.5052 | 3 | igd | 0.033 | C | \|\| id:ebi-a-GCST90001997 | ebi-a-GCST90001997 | TRUE | G | 39283094 | 6.07E-13 | reported | 3590 | 0.06992 | rs12637578 |  |  |  |  |  |  |  |  |  |  |  |  |  |  |  |
| 0.3843 | 3 | igd | 0.033 | C | \|\| id:ebi-a-GCST90001995 | ebi-a-GCST90001995 | TRUE | T | 39286884 | 3.69E-08 | reported | 3590 | 0.06965 | rs12639303 |  |  |  |  |  |  |  |  |  |  |  |  |  |  |  |
| 0.4908 | 2 | igd | 0.0328 | C | \|\| id:ebi-a-GCST90002098 | ebi-a-GCST90002098 | TRUE | T | 34796785 | 7.12E-12 | reported | 2910 | 0.0713 | rs72790816 |  |  |  |  |  |  |  |  |  |  |  |  |  |  |  |
| 0.5352 | 2 | igd | 0.0327 | G | \|\| id:ebi-a-GCST90001508 | ebi-a-GCST90001508 | TRUE | C | 38955419 | 2.16E-13 | reported | 3437 | 0.07263 | rs182240059 |  |  |  |  |  |  |  |  |  |  |  |  |  |  |  |
| 0.4916 | 2 | igd | 0.0327 | G | \|\| id:ebi-a-GCST90001509 | ebi-a-GCST90001509 | TRUE | C | 38955419 | 1.58E-11 | reported | 3437 | 0.07268 | rs182240059 |  |  |  |  |  |  |  |  |  |  |  |  |  |  |  |
| -0.4706 | 2 | igd | 0.0327 | T | \|\| id:ebi-a-GCST90002118 | ebi-a-GCST90002118 | TRUE | C | 86583806 | 6.85E-10 | reported | 2920 | 0.07603 | rs150649551 |  |  |  |  |  |  |  |  |  |  |  |  |  |  |  |
| -0.7292 | 2 | igd | 0.0327 | T | \|\| id:ebi-a-GCST90002119 | ebi-a-GCST90002119 | TRUE | C | 86583806 | 1.32E-22 | reported | 2920 | 0.07391 | rs150649551 |  |  |  |  |  |  |  |  |  |  |  |  |  |  |  |
| -0.5487 | 12 | igd | 0.0326 | C | \|\| id:ebi-a-GCST90002074 | ebi-a-GCST90002074 | TRUE | T | 70906075 | 5.58E-14 | reported | 3112 | 0.07266 | rs61930264 |  |  |  |  |  |  |  |  |  |  |  |  |  |  |  |
| 0.6018 | 2 | igd | 0.0326 | A | \|\| id:ebi-a-GCST90002098 | ebi-a-GCST90002098 | TRUE | G | 38849557 | 7.66E-17 | reported | 2910 | 0.07175 | rs150437123 |  |  |  |  |  |  |  |  |  |  |  |  |  |  |  |
| -1.304 | 19 | igd | 0.0325 | A | \|\| id:ebi-a-GCST90001952 | ebi-a-GCST90001952 | TRUE | G | 51770292 | 1.90E-36 | reported | 1569 | 0.1008 | rs11668077 |  |  |  |  |  |  |  |  |  |  |  |  |  |  |  |
| 0.4783 | 7 | igd | 0.0325 | A | \|\| id:ebi-a-GCST90002078 | ebi-a-GCST90002078 | TRUE | G | 92239892 | 2.21E-10 | reported | 3112 | 0.07511 | rs182090955 |  |  |  |  |  |  |  |  |  |  |  |  |  |  |  |
| 0.4242 | 2 | igd | 0.0325 | A | \|\| id:ebi-a-GCST90002102 | ebi-a-GCST90002102 | TRUE | G | 38849557 | 3.57E-09 | reported | 2919 | 0.07163 | rs150437123 |  |  |  |  |  |  |  |  |  |  |  |  |  |  |  |
| -0.4135 | 22 | igd | 0.0324 | G | \|\| id:ebi-a-GCST90001702 | ebi-a-GCST90001702 | TRUE | A | 40031206 | 4.26E-09 | reported | 3657 | 0.07022 | rs117655683 |  |  |  |  |  |  |  |  |  |  |  |  |  |  |  |
| -0.9511 | 22 | igd | 0.0324 | G | \|\| id:ebi-a-GCST90001702 | ebi-a-GCST90001702 | TRUE | T | 43080791 | 4.15E-42 | reported | 3657 | 0.06906 | rs140725954 |  |  |  |  |  |  |  |  |  |  |  |  |  |  |  |
| -0.9408 | 22 | igd | 0.0324 | G | \|\| id:ebi-a-GCST90001703 | ebi-a-GCST90001703 | TRUE | T | 43080791 | 3.13E-41 | reported | 3657 | 0.06908 | rs140725954 |  |  |  |  |  |  |  |  |  |  |  |  |  |  |  |
| -0.4155 | 22 | igd | 0.0324 | G | \|\| id:ebi-a-GCST90001703 | ebi-a-GCST90001703 | TRUE | A | 40031206 | 3.58E-09 | reported | 3657 | 0.07022 | rs117655683 |  |  |  |  |  |  |  |  |  |  |  |  |  |  |  |
| -0.4186 | 22 | igd | 0.0324 | G | \|\| id:ebi-a-GCST90001704 | ebi-a-GCST90001704 | TRUE | A | 40031206 | 2.62E-09 | reported | 3657 | 0.07013 | rs117655683 |  |  |  |  |  |  |  |  |  |  |  |  |  |  |  |
| -0.9374 | 22 | igd | 0.0324 | G | \|\| id:ebi-a-GCST90001704 | ebi-a-GCST90001704 | TRUE | T | 43080791 | 4.76E-41 | reported | 3657 | 0.069 | rs140725954 |  |  |  |  |  |  |  |  |  |  |  |  |  |  |  |
| -0.9028 | 22 | igd | 0.0324 | G | \|\| id:ebi-a-GCST90001710 | ebi-a-GCST90001710 | TRUE | T | 43080791 | 3.17E-38 | reported | 3657 | 0.06903 | rs140725954 |  |  |  |  |  |  |  |  |  |  |  |  |  |  |  |
| -0.3941 | 22 | igd | 0.0324 | G | \|\| id:ebi-a-GCST90001711 | ebi-a-GCST90001711 | TRUE | A | 40031206 | 1.86E-08 | reported | 3657 | 0.06991 | rs117655683 |  |  |  |  |  |  |  |  |  |  |  |  |  |  |  |
| -0.9284 | 22 | igd | 0.0324 | G | \|\| id:ebi-a-GCST90001711 | ebi-a-GCST90001711 | TRUE | T | 43080791 | 1.44E-40 | reported | 3657 | 0.06877 | rs140725954 |  |  |  |  |  |  |  |  |  |  |  |  |  |  |  |
| 0.4798 | 2 | igd | 0.0323 | G | \|\| id:ebi-a-GCST90001507 | ebi-a-GCST90001507 | TRUE | C | 38955419 | 1.13E-10 | reported | 3405 | 0.07417 | rs182240059 |  |  |  |  |  |  |  |  |  |  |  |  |  |  |  |
| -0.3985 | 10 | igd | 0.0323 | C | \|\| id:ebi-a-GCST90001658 | ebi-a-GCST90001658 | TRUE | G | 90688122 | 4.75E-08 | reported | 3440 | 0.07282 | rs75799019 |  |  |  |  |  |  |  |  |  |  |  |  |  |  |  |
| -0.4067 | 10 | igd | 0.0323 | C | \|\| id:ebi-a-GCST90001659 | ebi-a-GCST90001659 | TRUE | G | 90688122 | 2.51E-08 | reported | 3440 | 0.07282 | rs75799019 |  |  |  |  |  |  |  |  |  |  |  |  |  |  |  |
| -0.4281 | 10 | igd | 0.0323 | C | \|\| id:ebi-a-GCST90001670 | ebi-a-GCST90001670 | TRUE | G | 90688122 | 1.61E-08 | reported | 3440 | 0.07559 | rs75799019 |  |  |  |  |  |  |  |  |  |  |  |  |  |  |  |
| -0.4409 | 10 | igd | 0.0323 | C | \|\| id:ebi-a-GCST90001671 | ebi-a-GCST90001671 | TRUE | G | 90688122 | 6.74E-09 | reported | 3440 | 0.07586 | rs75799019 |  |  |  |  |  |  |  |  |  |  |  |  |  |  |  |
| -0.772 | 1 | igd | 0.0322 | G | \|\| id:ebi-a-GCST90001858 | ebi-a-GCST90001858 | TRUE | C | 167413486 | 1.91E-23 | reported | 2919 | 0.07671 | rs35240301 |  |  |  |  |  |  |  |  |  |  |  |  |  |  |  |
| -0.5673 | 1 | igd | 0.0322 | G | \|\| id:ebi-a-GCST90001861 | ebi-a-GCST90001861 | TRUE | C | 167413486 | 1.51E-13 | reported | 2919 | 0.07645 | rs35240301 |  |  |  |  |  |  |  |  |  |  |  |  |  |  |  |
| -0.6205 | 1 | igd | 0.0322 | G | \|\| id:ebi-a-GCST90001863 | ebi-a-GCST90001863 | TRUE | C | 167413486 | 3.48E-16 | reported | 2919 | 0.07565 | rs35240301 |  |  |  |  |  |  |  |  |  |  |  |  |  |  |  |
| -0.8173 | 1 | igd | 0.0322 | G | \|\| id:ebi-a-GCST90001864 | ebi-a-GCST90001864 | TRUE | C | 167413486 | 3.12E-26 | reported | 2919 | 0.07638 | rs35240301 |  |  |  |  |  |  |  |  |  |  |  |  |  |  |  |
| -0.4635 | 4 | igd | 0.0321 | A | \|\| id:ebi-a-GCST90001815 | ebi-a-GCST90001815 | TRUE | G | 15037408 | 9.22E-12 | reported | 3657 | 0.06776 | rs76354299 |  |  |  |  |  |  |  |  |  |  |  |  |  |  |  |
| 0.5063 | 10 | igd | 0.032 | T | \|\| id:ebi-a-GCST90002030 | ebi-a-GCST90002030 | TRUE | A | 95387682 | 9.94E-11 | reported | 2920 | 0.078 | rs192831774 |  |  |  |  |  |  |  |  |  |  |  |  |  |  |  |
| -0.5296 | 1 | igd | 0.0319 | G | \|\| id:ebi-a-GCST90001845 | ebi-a-GCST90001845 | TRUE | C | 167413486 | 1.16E-11 | reported | 2911 | 0.07774 | rs35240301 |  |  |  |  |  |  |  |  |  |  |  |  |  |  |  |
| 59.98 | 10 | igd | 0.0318 | A | \|\| id:ebi-a-GCST90001698 | ebi-a-GCST90001698 | TRUE | G | 132596864 | 6.47E-09 | reported | 3408 | 10.31 | rs138948609 |  |  |  |  |  |  |  |  |  |  |  |  |  |  |  |
| -0.4525 | 10 | igd | 0.0317 | T | \|\| id:ebi-a-GCST90001489 | ebi-a-GCST90001489 | TRUE | C | 97819462 | 5.30E-10 | reported | 3405 | 0.07265 | rs117301955 |  |  |  |  |  |  |  |  |  |  |  |  |  |  |  |
| -0.5028 | 10 | igd | 0.0317 | T | \|\| id:ebi-a-GCST90001495 | ebi-a-GCST90001495 | TRUE | C | 97819462 | 7.31E-12 | reported | 3405 | 0.07312 | rs117301955 |  |  |  |  |  |  |  |  |  |  |  |  |  |  |  |
| -0.5077 | 10 | igd | 0.0317 | T | \|\| id:ebi-a-GCST90001660 | ebi-a-GCST90001660 | TRUE | C | 97819462 | 2.49E-12 | reported | 3408 | 0.07222 | rs117301955 |  |  |  |  |  |  |  |  |  |  |  |  |  |  |  |
| -0.4603 | 10 | igd | 0.0317 | T | \|\| id:ebi-a-GCST90001672 | ebi-a-GCST90001672 | TRUE | C | 97819462 | 3.32E-10 | reported | 3408 | 0.07304 | rs117301955 |  |  |  |  |  |  |  |  |  |  |  |  |  |  |  |
| -0.8873 | 19 | igd | 0.0317 | A | \|\| id:ebi-a-GCST90001947 | ebi-a-GCST90001947 | TRUE | T | 51732079 | 4.59E-18 | reported | 1577 | 0.1012 | rs149201050 |  |  |  |  |  |  |  |  |  |  |  |  |  |  |  |
| -0.8912 | 19 | igd | 0.0317 | A | \|\| id:ebi-a-GCST90001956 | ebi-a-GCST90001956 | TRUE | T | 51732079 | 2.73E-18 | reported | 1579 | 0.1009 | rs149201050 |  |  |  |  |  |  |  |  |  |  |  |  |  |  |  |
| -1.33 | 19 | igd | 0.0317 | A | \|\| id:ebi-a-GCST90001956 | ebi-a-GCST90001956 | TRUE | G | 51770292 | 1.60E-37 | reported | 1579 | 0.1012 | rs11668077 |  |  |  |  |  |  |  |  |  |  |  |  |  |  |  |
| -0.9076 | 19 | igd | 0.0317 | A | \|\| id:ebi-a-GCST90001957 | ebi-a-GCST90001957 | TRUE | T | 51732079 | 5.98E-19 | reported | 1578 | 0.1008 | rs149201050 |  |  |  |  |  |  |  |  |  |  |  |  |  |  |  |
| -0.4909 | 5 | igd | 0.0317 | G | \|\| id:prot-a-427 | prot-a-427 | TRUE | A | 40972211 | 2.09E-11 | reported | 3301 | 0.0733 | rs74480769 |  |  |  |  |  |  |  |  |  |  |  |  |  |  |  |
| -0.9661 | 19 | igd | 0.0315 | A | \|\| id:ebi-a-GCST90001952 | ebi-a-GCST90001952 | TRUE | T | 51732079 | 4.34E-21 | reported | 1569 | 0.101 | rs149201050 |  |  |  |  |  |  |  |  |  |  |  |  |  |  |  |
| -1.308 | 19 | igd | 0.0315 | A | \|\| id:ebi-a-GCST90001953 | ebi-a-GCST90001953 | TRUE | G | 51770292 | 4.66E-36 | reported | 1558 | 0.1017 | rs11668077 |  |  |  |  |  |  |  |  |  |  |  |  |  |  |  |
| -1.232 | 19 | igd | 0.0315 | A | \|\| id:ebi-a-GCST90001955 | ebi-a-GCST90001955 | TRUE | G | 51770292 | 7.16E-32 | reported | 1554 | 0.1026 | rs11668077 |  |  |  |  |  |  |  |  |  |  |  |  |  |  |  |
| 0.6804 | 19 | igd | 0.0315 | A | \|\| id:ebi-a-GCST90002052 | ebi-a-GCST90002052 | TRUE | G | 51770292 | 2.22E-10 | reported | 1555 | 0.1065 | rs11668077 |  |  |  |  |  |  |  |  |  |  |  |  |  |  |  |
| -0.4019 | 10 | igd | 0.0314 | T | \|\| id:ebi-a-GCST90001484 | ebi-a-GCST90001484 | TRUE | C | 97819462 | 1.28E-08 | reported | 3437 | 0.07049 | rs117301955 |  |  |  |  |  |  |  |  |  |  |  |  |  |  |  |
| -0.3862 | 10 | igd | 0.0314 | T | \|\| id:ebi-a-GCST90001485 | ebi-a-GCST90001485 | TRUE | C | 97819462 | 4.42E-08 | reported | 3437 | 0.07041 | rs117301955 |  |  |  |  |  |  |  |  |  |  |  |  |  |  |  |
| -0.4549 | 10 | igd | 0.0314 | T | \|\| id:ebi-a-GCST90001491 | ebi-a-GCST90001491 | TRUE | C | 97819462 | 2.39E-10 | reported | 3437 | 0.07161 | rs117301955 |  |  |  |  |  |  |  |  |  |  |  |  |  |  |  |
| -0.5495 | 10 | igd | 0.0314 | T | \|\| id:ebi-a-GCST90001496 | ebi-a-GCST90001496 | TRUE | C | 97819462 | 4.07E-14 | reported | 3437 | 0.07239 | rs117301955 |  |  |  |  |  |  |  |  |  |  |  |  |  |  |  |
| -0.5282 | 10 | igd | 0.0314 | T | \|\| id:ebi-a-GCST90001497 | ebi-a-GCST90001497 | TRUE | C | 97819462 | 1.91E-13 | reported | 3437 | 0.07152 | rs117301955 |  |  |  |  |  |  |  |  |  |  |  |  |  |  |  |
| -0.51 | 10 | igd | 0.0314 | T | \|\| id:ebi-a-GCST90001658 | ebi-a-GCST90001658 | TRUE | C | 97819462 | 1.86E-13 | reported | 3440 | 0.06903 | rs117301955 |  |  |  |  |  |  |  |  |  |  |  |  |  |  |  |
| -0.5162 | 10 | igd | 0.0314 | T | \|\| id:ebi-a-GCST90001659 | ebi-a-GCST90001659 | TRUE | C | 97819462 | 9.46E-14 | reported | 3440 | 0.06903 | rs117301955 |  |  |  |  |  |  |  |  |  |  |  |  |  |  |  |
| -0.4673 | 10 | igd | 0.0314 | T | \|\| id:ebi-a-GCST90001670 | ebi-a-GCST90001670 | TRUE | C | 97819462 | 9.84E-11 | reported | 3440 | 0.07201 | rs117301955 |  |  |  |  |  |  |  |  |  |  |  |  |  |  |  |
| -0.4469 | 10 | igd | 0.0314 | T | \|\| id:ebi-a-GCST90001671 | ebi-a-GCST90001671 | TRUE | C | 97819462 | 6.44E-10 | reported | 3440 | 0.07212 | rs117301955 |  |  |  |  |  |  |  |  |  |  |  |  |  |  |  |
| -1.328 | 19 | igd | 0.0314 | A | \|\| id:ebi-a-GCST90001957 | ebi-a-GCST90001957 | TRUE | G | 51770292 | 4.34E-37 | reported | 1578 | 0.1017 | rs11668077 |  |  |  |  |  |  |  |  |  |  |  |  |  |  |  |
| 0.5858 | 10 | igd | 0.0313 | T | \|\| id:ebi-a-GCST90001484 | ebi-a-GCST90001484 | TRUE | A | 95387682 | 3.07E-16 | reported | 3437 | 0.07135 | rs192831774 |  |  |  |  |  |  |  |  |  |  |  |  |  |  |  |
| 0.5247 | 10 | igd | 0.0313 | T | \|\| id:ebi-a-GCST90001485 | ebi-a-GCST90001485 | TRUE | A | 95387682 | 2.46E-13 | reported | 3437 | 0.07138 | rs192831774 |  |  |  |  |  |  |  |  |  |  |  |  |  |  |  |
| 0.4138 | 10 | igd | 0.0313 | T | \|\| id:ebi-a-GCST90001491 | ebi-a-GCST90001491 | TRUE | A | 95387682 | 1.91E-08 | reported | 3437 | 0.07345 | rs192831774 |  |  |  |  |  |  |  |  |  |  |  |  |  |  |  |
| 0.6179 | 10 | igd | 0.0313 | T | \|\| id:ebi-a-GCST90001496 | ebi-a-GCST90001496 | TRUE | A | 95387682 | 1.01E-16 | reported | 3437 | 0.07403 | rs192831774 |  |  |  |  |  |  |  |  |  |  |  |  |  |  |  |
| 0.6013 | 10 | igd | 0.0313 | T | \|\| id:ebi-a-GCST90001497 | ebi-a-GCST90001497 | TRUE | A | 95387682 | 2.79E-16 | reported | 3437 | 0.07313 | rs192831774 |  |  |  |  |  |  |  |  |  |  |  |  |  |  |  |
| 0.53 | 10 | igd | 0.0312 | T | \|\| id:ebi-a-GCST90001658 | ebi-a-GCST90001658 | TRUE | A | 95387682 | 9.17E-14 | reported | 3440 | 0.07082 | rs192831774 |  |  |  |  |  |  |  |  |  |  |  |  |  |  |  |
| 0.5749 | 10 | igd | 0.0312 | T | \|\| id:ebi-a-GCST90001659 | ebi-a-GCST90001659 | TRUE | A | 95387682 | 6.02E-16 | reported | 3440 | 0.07073 | rs192831774 |  |  |  |  |  |  |  |  |  |  |  |  |  |  |  |
| 0.449 | 6 | igd | 0.0312 | G | \|\| id:ebi-a-GCST90001661 | ebi-a-GCST90001661 | TRUE | A | 102294761 | 2.28E-09 | reported | 3440 | 0.07493 | rs139249541 |  |  |  |  |  |  |  |  |  |  |  |  |  |  |  |
| 0.4744 | 10 | igd | 0.0312 | T | \|\| id:ebi-a-GCST90001670 | ebi-a-GCST90001670 | TRUE | A | 95387682 | 1.44E-10 | reported | 3440 | 0.07377 | rs192831774 |  |  |  |  |  |  |  |  |  |  |  |  |  |  |  |
| 0.4548 | 10 | igd | 0.0312 | T | \|\| id:ebi-a-GCST90001671 | ebi-a-GCST90001671 | TRUE | A | 95387682 | 8.88E-10 | reported | 3440 | 0.07401 | rs192831774 |  |  |  |  |  |  |  |  |  |  |  |  |  |  |  |
| 0.4291 | 6 | igd | 0.0312 | G | \|\| id:ebi-a-GCST90001694 | ebi-a-GCST90001694 | TRUE | A | 102294761 | 1.48E-08 | reported | 3440 | 0.07557 | rs139249541 |  |  |  |  |  |  |  |  |  |  |  |  |  |  |  |
| -1.328 | 19 | igd | 0.0312 | A | \|\| id:ebi-a-GCST90001954 | ebi-a-GCST90001954 | TRUE | G | 51770292 | 9.26E-37 | reported | 1555 | 0.1021 | rs11668077 |  |  |  |  |  |  |  |  |  |  |  |  |  |  |  |
| -0.5734 | 10 | igd | 0.0312 | T | \|\| id:ebi-a-GCST90002030 | ebi-a-GCST90002030 | TRUE | C | 97819462 | 1.30E-13 | reported | 2920 | 0.07706 | rs117301955 |  |  |  |  |  |  |  |  |  |  |  |  |  |  |  |
| 0.4845 | 10 | igd | 0.0312 | T | \|\| id:ebi-a-GCST90002030 | ebi-a-GCST90002030 | TRUE | C | 96919331 | 7.74E-11 | reported | 2920 | 0.07419 | rs149287350 |  |  |  |  |  |  |  |  |  |  |  |  |  |  |  |
| 0.478 | 10 | igd | 0.0312 | A | \|\| id:ebi-a-GCST90002030 | ebi-a-GCST90002030 | TRUE | C | 97054318 | 1.71E-10 | reported | 2920 | 0.0746 | rs80002058 |  |  |  |  |  |  |  |  |  |  |  |  |  |  |  |
| -0.4873 | 10 | igd | 0.0312 | T | \|\| id:ebi-a-GCST90002031 | ebi-a-GCST90002031 | TRUE | C | 97819462 | 8.60E-10 | reported | 2920 | 0.07919 | rs117301955 |  |  |  |  |  |  |  |  |  |  |  |  |  |  |  |
| -0.5199 | 10 | igd | 0.0312 | T | \|\| id:ebi-a-GCST90002032 | ebi-a-GCST90002032 | TRUE | C | 97819462 | 5.64E-11 | reported | 2920 | 0.07905 | rs117301955 |  |  |  |  |  |  |  |  |  |  |  |  |  |  |  |
| -0.5335 | 1 | igd | 0.0312 | T | \|\| id:ebi-a-GCST90002035 | ebi-a-GCST90002035 | TRUE | C | 92315896 | 2.85E-11 | reported | 2871 | 0.07987 | rs17575497 |  |  |  |  |  |  |  |  |  |  |  |  |  |  |  |
| -0.578 | 1 | igd | 0.0312 | T | \|\| id:ebi-a-GCST90002036 | ebi-a-GCST90002036 | TRUE | C | 92315896 | 6.00E-13 | reported | 2871 | 0.07991 | rs17575497 |  |  |  |  |  |  |  |  |  |  |  |  |  |  |  |
| 0.4945 | 10 | igd | 0.0311 | T | \|\| id:ebi-a-GCST90001483 | ebi-a-GCST90001483 | TRUE | A | 95387682 | 1.00E-11 | reported | 3405 | 0.0724 | rs192831774 |  |  |  |  |  |  |  |  |  |  |  |  |  |  |  |
| 0.5637 | 10 | igd | 0.0311 | T | \|\| id:ebi-a-GCST90001495 | ebi-a-GCST90001495 | TRUE | A | 95387682 | 8.00E-14 | reported | 3405 | 0.07514 | rs192831774 |  |  |  |  |  |  |  |  |  |  |  |  |  |  |  |
| -0.407 | 10 | igd | 0.0311 | A | \|\| id:ebi-a-GCST90001496 | ebi-a-GCST90001496 | TRUE | G | 97850092 | 1.81E-08 | reported | 3437 | 0.07213 | rs114614929 |  |  |  |  |  |  |  |  |  |  |  |  |  |  |  |
| -0.3967 | 10 | igd | 0.0311 | A | \|\| id:ebi-a-GCST90001497 | ebi-a-GCST90001497 | TRUE | G | 97850092 | 2.76E-08 | reported | 3437 | 0.07124 | rs114614929 |  |  |  |  |  |  |  |  |  |  |  |  |  |  |  |
| 0.5778 | 10 | igd | 0.0311 | T | \|\| id:ebi-a-GCST90001660 | ebi-a-GCST90001660 | TRUE | A | 95387682 | 9.91E-15 | reported | 3408 | 0.07431 | rs192831774 |  |  |  |  |  |  |  |  |  |  |  |  |  |  |  |
| 0.5219 | 10 | igd | 0.0311 | T | \|\| id:ebi-a-GCST90001672 | ebi-a-GCST90001672 | TRUE | A | 95387682 | 4.61E-12 | reported | 3408 | 0.07518 | rs192831774 |  |  |  |  |  |  |  |  |  |  |  |  |  |  |  |
| -1.317 | 19 | igd | 0.0311 | A | \|\| id:ebi-a-GCST90001947 | ebi-a-GCST90001947 | TRUE | G | 51770292 | 6.43E-36 | reported | 1577 | 0.1026 | rs11668077 |  |  |  |  |  |  |  |  |  |  |  |  |  |  |  |
| -0.4811 | 1 | igd | 0.031 | C | \|\| id:ebi-a-GCST90001858 | ebi-a-GCST90001858 | TRUE | T | 168360127 | 6.97E-10 | reported | 2919 | 0.07776 | rs68156293 |  |  |  |  |  |  |  |  |  |  |  |  |  |  |  |
| 0.4947 | 10 | igd | 0.031 | A | \|\| id:ebi-a-GCST90002030 | ebi-a-GCST90002030 | TRUE | G | 95481738 | 5.47E-10 | reported | 2920 | 0.07946 | rs118059432 |  |  |  |  |  |  |  |  |  |  |  |  |  |  |  |
| 0.4633 | 10 | igd | 0.031 | A | \|\| id:ebi-a-GCST90002032 | ebi-a-GCST90002032 | TRUE | G | 95481738 | 1.44E-08 | reported | 2920 | 0.0815 | rs118059432 |  |  |  |  |  |  |  |  |  |  |  |  |  |  |  |
| -0.4791 | 1 | igd | 0.031 | C | \|\| id:ebi-a-GCST90002035 | ebi-a-GCST90002035 | TRUE | G | 92171673 | 1.48E-09 | reported | 2871 | 0.07897 | rs114425738 |  |  |  |  |  |  |  |  |  |  |  |  |  |  |  |
| -0.4965 | 1 | igd | 0.031 | C | \|\| id:ebi-a-GCST90002036 | ebi-a-GCST90002036 | TRUE | G | 92171673 | 3.91E-10 | reported | 2871 | 0.07906 | rs114425738 |  |  |  |  |  |  |  |  |  |  |  |  |  |  |  |
| -0.5725 | 19 | igd | 0.0309 | G | \|\| id:ebi-a-GCST90001949 | ebi-a-GCST90001949 | TRUE | A | 51910411 | 4.63E-08 | reported | 1634 | 0.1043 | rs367719840 |  |  |  |  |  |  |  |  |  |  |  |  |  |  |  |
| 0.387 | 10 | igd | 0.0308 | T | \|\| id:ebi-a-GCST90001496 | ebi-a-GCST90001496 | TRUE | C | 96919331 | 4.36E-08 | reported | 3437 | 0.07052 | rs149287350 |  |  |  |  |  |  |  |  |  |  |  |  |  |  |  |
| 0.3834 | 10 | igd | 0.0308 | T | \|\| id:ebi-a-GCST90001497 | ebi-a-GCST90001497 | TRUE | C | 96919331 | 3.93E-08 | reported | 3437 | 0.06963 | rs149287350 |  |  |  |  |  |  |  |  |  |  |  |  |  |  |  |
| 0.3945 | 10 | igd | 0.0308 | T | \|\| id:ebi-a-GCST90001658 | ebi-a-GCST90001658 | TRUE | C | 96919331 | 4.42E-09 | reported | 3440 | 0.06705 | rs149287350 |  |  |  |  |  |  |  |  |  |  |  |  |  |  |  |
| 0.394 | 10 | igd | 0.0308 | T | \|\| id:ebi-a-GCST90001659 | ebi-a-GCST90001659 | TRUE | C | 96919331 | 4.62E-09 | reported | 3440 | 0.06706 | rs149287350 |  |  |  |  |  |  |  |  |  |  |  |  |  |  |  |
| 0.433 | 6 | igd | 0.0308 | G | \|\| id:ebi-a-GCST90001661 | ebi-a-GCST90001661 | TRUE | A | 102173117 | 1.71E-08 | reported | 3440 | 0.07661 | rs117913408 |  |  |  |  |  |  |  |  |  |  |  |  |  |  |  |
| -0.8639 | 19 | igd | 0.0308 | A | \|\| id:ebi-a-GCST90001953 | ebi-a-GCST90001953 | TRUE | T | 51732079 | 1.19E-16 | reported | 1558 | 0.1031 | rs149201050 |  |  |  |  |  |  |  |  |  |  |  |  |  |  |  |
| -0.4024 | 10 | igd | 0.0307 | A | \|\| id:ebi-a-GCST90001495 | ebi-a-GCST90001495 | TRUE | G | 97850092 | 4.66E-08 | reported | 3405 | 0.07348 | rs114614929 |  |  |  |  |  |  |  |  |  |  |  |  |  |  |  |
| -0.5379 | 2 | igd | 0.0307 | A | \|\| id:ebi-a-GCST90001516 | ebi-a-GCST90001516 | TRUE | C | 176970247 | 2.92E-08 | reported | 1857 | 0.09656 | rs72923436 |  |  |  |  |  |  |  |  |  |  |  |  |  |  |  |
| -0.3976 | 10 | igd | 0.0307 | A | \|\| id:ebi-a-GCST90001660 | ebi-a-GCST90001660 | TRUE | G | 97850092 | 4.56E-08 | reported | 3408 | 0.07255 | rs114614929 |  |  |  |  |  |  |  |  |  |  |  |  |  |  |  |
| -0.5355 | 10 | igd | 0.0307 | G | \|\| id:ebi-a-GCST90002030 | ebi-a-GCST90002030 | TRUE | A | 97546627 | 3.40E-11 | reported | 2920 | 0.08049 | rs35748815 |  |  |  |  |  |  |  |  |  |  |  |  |  |  |  |
| -0.465 | 10 | igd | 0.0307 | G | \|\| id:ebi-a-GCST90002031 | ebi-a-GCST90002031 | TRUE | A | 97546627 | 1.82E-08 | reported | 2920 | 0.08239 | rs35748815 |  |  |  |  |  |  |  |  |  |  |  |  |  |  |  |
| -0.5394 | 10 | igd | 0.0307 | G | \|\| id:ebi-a-GCST90002032 | ebi-a-GCST90002032 | TRUE | A | 97546627 | 6.28E-11 | reported | 2920 | 0.0822 | rs35748815 |  |  |  |  |  |  |  |  |  |  |  |  |  |  |  |
| -0.6467 | 2 | igd | 0.0307 | A | \|\| id:ebi-a-GCST90002118 | ebi-a-GCST90002118 | TRUE | G | 87111890 | 6.38E-16 | reported | 2920 | 0.07956 | rs191478693 |  |  |  |  |  |  |  |  |  |  |  |  |  |  |  |
| -0.8611 | 19 | igd | 0.0306 | A | \|\| id:ebi-a-GCST90001955 | ebi-a-GCST90001955 | TRUE | T | 51732079 | 2.61E-16 | reported | 1554 | 0.104 | rs149201050 |  |  |  |  |  |  |  |  |  |  |  |  |  |  |  |
| -0.9057 | 19 | igd | 0.0305 | A | \|\| id:ebi-a-GCST90001954 | ebi-a-GCST90001954 | TRUE | T | 51732079 | 4.73E-18 | reported | 1555 | 0.1033 | rs149201050 |  |  |  |  |  |  |  |  |  |  |  |  |  |  |  |
| -0.4093 | 20 | igd | 0.0304 | C | \|\| id:ebi-a-GCST90001805 | ebi-a-GCST90001805 | TRUE | T | 45314435 | 1.77E-08 | reported | 3657 | 0.0725 | rs72485816 |  |  |  |  |  |  |  |  |  |  |  |  |  |  |  |
| 0.3955 | 10 | igd | 0.0303 | A | \|\| id:ebi-a-GCST90001496 | ebi-a-GCST90001496 | TRUE | C | 97054318 | 3.92E-08 | reported | 3437 | 0.07181 | rs80002058 |  |  |  |  |  |  |  |  |  |  |  |  |  |  |  |
| 0.4032 | 10 | igd | 0.0303 | A | \|\| id:ebi-a-GCST90001497 | ebi-a-GCST90001497 | TRUE | C | 97054318 | 1.40E-08 | reported | 3437 | 0.07089 | rs80002058 |  |  |  |  |  |  |  |  |  |  |  |  |  |  |  |
| -0.4756 | 1 | igd | 0.0303 | C | \|\| id:ebi-a-GCST90001854 | ebi-a-GCST90001854 | TRUE | T | 165092633 | 2.59E-09 | reported | 2919 | 0.07961 | rs17396840 |  |  |  |  |  |  |  |  |  |  |  |  |  |  |  |
| -0.4872 | 1 | igd | 0.0303 | C | \|\| id:ebi-a-GCST90001858 | ebi-a-GCST90001858 | TRUE | T | 165092633 | 1.43E-09 | reported | 2919 | 0.08024 | rs17396840 |  |  |  |  |  |  |  |  |  |  |  |  |  |  |  |
| -0.5127 | 1 | igd | 0.0303 | C | \|\| id:ebi-a-GCST90001860 | ebi-a-GCST90001860 | TRUE | T | 165092633 | 2.22E-10 | reported | 2919 | 0.08051 | rs17396840 |  |  |  |  |  |  |  |  |  |  |  |  |  |  |  |
| -0.4417 | 1 | igd | 0.0303 | C | \|\| id:ebi-a-GCST90001863 | ebi-a-GCST90001863 | TRUE | T | 165092633 | 2.35E-08 | reported | 2919 | 0.07888 | rs17396840 |  |  |  |  |  |  |  |  |  |  |  |  |  |  |  |
| -0.5012 | 1 | igd | 0.0303 | C | \|\| id:ebi-a-GCST90001864 | ebi-a-GCST90001864 | TRUE | T | 165092633 | 4.52E-10 | reported | 2919 | 0.08011 | rs17396840 |  |  |  |  |  |  |  |  |  |  |  |  |  |  |  |
| -0.6737 | 17 | igd | 0.0303 | T | \|\| id:ebi-a-GCST90001926 | ebi-a-GCST90001926 | TRUE | C | 56603493 | 3.73E-17 | reported | 2920 | 0.07951 | rs148548662 |  |  |  |  |  |  |  |  |  |  |  |  |  |  |  |
| -0.6328 | 10 | igd | 0.0303 | A | \|\| id:ebi-a-GCST90002030 | ebi-a-GCST90002030 | TRUE | G | 97850092 | 6.14E-16 | reported | 2920 | 0.0778 | rs114614929 |  |  |  |  |  |  |  |  |  |  |  |  |  |  |  |
| -0.6431 | 10 | igd | 0.0303 | T | \|\| id:ebi-a-GCST90002030 | ebi-a-GCST90002030 | TRUE | C | 98411629 | 2.12E-15 | reported | 2920 | 0.08061 | rs114696821 |  |  |  |  |  |  |  |  |  |  |  |  |  |  |  |
| -0.6129 | 10 | igd | 0.0303 | T | \|\| id:ebi-a-GCST90002031 | ebi-a-GCST90002031 | TRUE | C | 98411629 | 1.55E-13 | reported | 2920 | 0.08262 | rs114696821 |  |  |  |  |  |  |  |  |  |  |  |  |  |  |  |
| -0.5807 | 10 | igd | 0.0303 | A | \|\| id:ebi-a-GCST90002031 | ebi-a-GCST90002031 | TRUE | G | 97850092 | 5.07E-13 | reported | 2920 | 0.08002 | rs114614929 |  |  |  |  |  |  |  |  |  |  |  |  |  |  |  |
| -0.5433 | 10 | igd | 0.0303 | T | \|\| id:ebi-a-GCST90002032 | ebi-a-GCST90002032 | TRUE | C | 98411629 | 6.12E-11 | reported | 2920 | 0.08275 | rs114696821 |  |  |  |  |  |  |  |  |  |  |  |  |  |  |  |
| -0.5828 | 10 | igd | 0.0303 | A | \|\| id:ebi-a-GCST90002032 | ebi-a-GCST90002032 | TRUE | G | 97850092 | 3.95E-13 | reported | 2920 | 0.07994 | rs114614929 |  |  |  |  |  |  |  |  |  |  |  |  |  |  |  |
| -0.7446 | 2 | igd | 0.0303 | C | \|\| id:ebi-a-GCST90002119 | ebi-a-GCST90002119 | TRUE | G | 86391633 | 3.98E-21 | reported | 2920 | 0.07833 | rs141133946 |  |  |  |  |  |  |  |  |  |  |  |  |  |  |  |
| -0.4125 | 22 | igd | 0.0302 | T | \|\| id:ebi-a-GCST90001702 | ebi-a-GCST90001702 | TRUE | C | 39481950 | 2.04E-08 | reported | 3657 | 0.07339 | rs117309320 |  |  |  |  |  |  |  |  |  |  |  |  |  |  |  |
| -0.4392 | 22 | igd | 0.0302 | T | \|\| id:ebi-a-GCST90001703 | ebi-a-GCST90001703 | TRUE | C | 39481950 | 2.32E-09 | reported | 3657 | 0.07333 | rs117309320 |  |  |  |  |  |  |  |  |  |  |  |  |  |  |  |
| -0.4244 | 22 | igd | 0.0302 | T | \|\| id:ebi-a-GCST90001704 | ebi-a-GCST90001704 | TRUE | C | 39481950 | 7.50E-09 | reported | 3657 | 0.07326 | rs117309320 |  |  |  |  |  |  |  |  |  |  |  |  |  |  |  |
| -0.4063 | 22 | igd | 0.0302 | T | \|\| id:ebi-a-GCST90001711 | ebi-a-GCST90001711 | TRUE | C | 39481950 | 2.81E-08 | reported | 3657 | 0.07302 | rs117309320 |  |  |  |  |  |  |  |  |  |  |  |  |  |  |  |
| -0.5239 | 22 | igd | 0.0301 | A | \|\| id:ebi-a-GCST90001702 | ebi-a-GCST90001702 | TRUE | G | 41893131 | 1.41E-12 | reported | 3657 | 0.0737 | rs138420756 |  |  |  |  |  |  |  |  |  |  |  |  |  |  |  |
| -0.5007 | 22 | igd | 0.0301 | A | \|\| id:ebi-a-GCST90001703 | ebi-a-GCST90001703 | TRUE | G | 41893131 | 1.30E-11 | reported | 3657 | 0.07374 | rs138420756 |  |  |  |  |  |  |  |  |  |  |  |  |  |  |  |
| -0.4426 | 22 | igd | 0.0301 | A | \|\| id:ebi-a-GCST90001704 | ebi-a-GCST90001704 | TRUE | G | 41893131 | 2.13E-09 | reported | 3657 | 0.07374 | rs138420756 |  |  |  |  |  |  |  |  |  |  |  |  |  |  |  |
| -0.513 | 22 | igd | 0.0301 | A | \|\| id:ebi-a-GCST90001710 | ebi-a-GCST90001710 | TRUE | G | 41893131 | 3.53E-12 | reported | 3657 | 0.07352 | rs138420756 |  |  |  |  |  |  |  |  |  |  |  |  |  |  |  |
| -0.4893 | 22 | igd | 0.0301 | A | \|\| id:ebi-a-GCST90001711 | ebi-a-GCST90001711 | TRUE | G | 41893131 | 2.98E-11 | reported | 3657 | 0.07338 | rs138420756 |  |  |  |  |  |  |  |  |  |  |  |  |  |  |  |
| 0.4818 | 10 | igd | 0.0301 | T | \|\| id:ebi-a-GCST90002030 | ebi-a-GCST90002030 | TRUE | G | 96749456 | 3.37E-09 | reported | 2920 | 0.08124 | rs142997475 |  |  |  |  |  |  |  |  |  |  |  |  |  |  |  |
| -0.7237 | 19 | igd | 0.03 | C | \|\| id:ebi-a-GCST90001946 | ebi-a-GCST90001946 | TRUE | T | 51750043 | 1.86E-11 | reported | 1634 | 0.107 | rs145323276 |  |  |  |  |  |  |  |  |  |  |  |  |  |  |  |
| -0.7394 | 19 | igd | 0.03 | C | \|\| id:ebi-a-GCST90001948 | ebi-a-GCST90001948 | TRUE | T | 51750043 | 6.57E-12 | reported | 1633 | 0.1069 | rs145323276 |  |  |  |  |  |  |  |  |  |  |  |  |  |  |  |
| -0.7134 | 19 | igd | 0.03 | C | \|\| id:ebi-a-GCST90001949 | ebi-a-GCST90001949 | TRUE | T | 51750043 | 3.02E-11 | reported | 1634 | 0.1066 | rs145323276 |  |  |  |  |  |  |  |  |  |  |  |  |  |  |  |
| -0.4505 | 10 | igd | 0.03 | T | \|\| id:ebi-a-GCST90001961 | ebi-a-GCST90001961 | TRUE | G | 96677921 | 3.31E-08 | reported | 2920 | 0.08133 | rs116878692 |  |  |  |  |  |  |  |  |  |  |  |  |  |  |  |
| -0.5987 | 6 | igd | 0.0299 | C | \|\| id:ebi-a-GCST90002105 | ebi-a-GCST90002105 | TRUE | A | 32427365 | 1.09E-13 | reported | 2872 | 0.08018 | rs28732228 |  |  |  |  |  |  |  |  |  |  |  |  |  |  |  |
| -0.479 | 1 | igd | 0.0298 | G | \|\| id:ebi-a-GCST90001858 | ebi-a-GCST90001858 | TRUE | T | 165191695 | 3.04E-09 | reported | 2919 | 0.08053 | rs78132817 |  |  |  |  |  |  |  |  |  |  |  |  |  |  |  |
| 0.4799 | 17 | igd | 0.0298 | T | \|\| id:ebi-a-GCST90001964 | ebi-a-GCST90001964 | TRUE | C | 62601428 | 7.13E-09 | reported | 2872 | 0.08266 | rs144830625 |  |  |  |  |  |  |  |  |  |  |  |  |  |  |  |
| -0.5752 | 22 | igd | 0.0297 | A | \|\| id:ebi-a-GCST90001702 | ebi-a-GCST90001702 | TRUE | T | 42752015 | 4.07E-15 | reported | 3657 | 0.07294 | rs142731119 |  |  |  |  |  |  |  |  |  |  |  |  |  |  |  |
| -0.5577 | 22 | igd | 0.0297 | A | \|\| id:ebi-a-GCST90001703 | ebi-a-GCST90001703 | TRUE | T | 42752015 | 2.70E-14 | reported | 3657 | 0.07297 | rs142731119 |  |  |  |  |  |  |  |  |  |  |  |  |  |  |  |
| -0.5476 | 22 | igd | 0.0297 | A | \|\| id:ebi-a-GCST90001704 | ebi-a-GCST90001704 | TRUE | T | 42752015 | 7.30E-14 | reported | 3657 | 0.0729 | rs142731119 |  |  |  |  |  |  |  |  |  |  |  |  |  |  |  |
| -0.5377 | 22 | igd | 0.0297 | A | \|\| id:ebi-a-GCST90001710 | ebi-a-GCST90001710 | TRUE | T | 42752015 | 1.87E-13 | reported | 3657 | 0.0728 | rs142731119 |  |  |  |  |  |  |  |  |  |  |  |  |  |  |  |
| -0.5262 | 22 | igd | 0.0297 | A | \|\| id:ebi-a-GCST90001711 | ebi-a-GCST90001711 | TRUE | T | 42752015 | 5.46E-13 | reported | 3657 | 0.07268 | rs142731119 |  |  |  |  |  |  |  |  |  |  |  |  |  |  |  |
| -0.4512 | 2 | igd | 0.0296 | C | \|\| id:ebi-a-GCST90001893 | ebi-a-GCST90001893 | TRUE | A | 204332458 | 3.09E-08 | reported | 2920 | 0.08128 | rs114171143 |  |  |  |  |  |  |  |  |  |  |  |  |  |  |  |
| -0.6034 | 19 | igd | 0.02958 | A | \|\| id:prot-a-439 | prot-a-439 | TRUE | G | 51806336 | 7.59E-14 | reported | 3301 | 0.0807 | rs117496758 |  |  |  |  |  |  |  |  |  |  |  |  |  |  |  |
| 0.5173 | 10 | igd | 0.0295 | T | \|\| id:ebi-a-GCST90001483 | ebi-a-GCST90001483 | TRUE | G | 96749456 | 7.19E-12 | reported | 3405 | 0.0752 | rs142997475 |  |  |  |  |  |  |  |  |  |  |  |  |  |  |  |
| 0.615 | 10 | igd | 0.0295 | T | \|\| id:ebi-a-GCST90001484 | ebi-a-GCST90001484 | TRUE | G | 96749456 | 1.65E-16 | reported | 3437 | 0.07422 | rs142997475 |  |  |  |  |  |  |  |  |  |  |  |  |  |  |  |
| 0.5567 | 10 | igd | 0.0295 | T | \|\| id:ebi-a-GCST90001485 | ebi-a-GCST90001485 | TRUE | G | 96749456 | 8.17E-14 | reported | 3437 | 0.07424 | rs142997475 |  |  |  |  |  |  |  |  |  |  |  |  |  |  |  |
| 0.4287 | 10 | igd | 0.0295 | T | \|\| id:ebi-a-GCST90001491 | ebi-a-GCST90001491 | TRUE | G | 96749456 | 2.22E-08 | reported | 3437 | 0.07646 | rs142997475 |  |  |  |  |  |  |  |  |  |  |  |  |  |  |  |
| 0.5967 | 10 | igd | 0.0295 | T | \|\| id:ebi-a-GCST90001495 | ebi-a-GCST90001495 | TRUE | G | 96749456 | 2.73E-14 | reported | 3405 | 0.07807 | rs142997475 |  |  |  |  |  |  |  |  |  |  |  |  |  |  |  |
| 0.6271 | 10 | igd | 0.0295 | T | \|\| id:ebi-a-GCST90001496 | ebi-a-GCST90001496 | TRUE | G | 96749456 | 5.77E-16 | reported | 3437 | 0.0771 | rs142997475 |  |  |  |  |  |  |  |  |  |  |  |  |  |  |  |
| 0.65 | 10 | igd | 0.0295 | T | \|\| id:ebi-a-GCST90001497 | ebi-a-GCST90001497 | TRUE | G | 96749456 | 1.90E-17 | reported | 3437 | 0.07606 | rs142997475 |  |  |  |  |  |  |  |  |  |  |  |  |  |  |  |
| 0.5396 | 10 | igd | 0.0295 | T | \|\| id:ebi-a-GCST90001658 | ebi-a-GCST90001658 | TRUE | G | 96749456 | 3.14E-13 | reported | 3440 | 0.07375 | rs142997475 |  |  |  |  |  |  |  |  |  |  |  |  |  |  |  |
| 0.5936 | 10 | igd | 0.0295 | T | \|\| id:ebi-a-GCST90001659 | ebi-a-GCST90001659 | TRUE | G | 96749456 | 1.03E-15 | reported | 3440 | 0.07364 | rs142997475 |  |  |  |  |  |  |  |  |  |  |  |  |  |  |  |
| 0.6424 | 10 | igd | 0.0295 | T | \|\| id:ebi-a-GCST90001660 | ebi-a-GCST90001660 | TRUE | G | 96749456 | 1.19E-16 | reported | 3408 | 0.07715 | rs142997475 |  |  |  |  |  |  |  |  |  |  |  |  |  |  |  |
| -0.4421 | 22 | igd | 0.0295 | T | \|\| id:ebi-a-GCST90001702 | ebi-a-GCST90001702 | TRUE | G | 37516789 | 1.15E-09 | reported | 3657 | 0.07243 | rs116921229 |  |  |  |  |  |  |  |  |  |  |  |  |  |  |  |
| -0.439 | 22 | igd | 0.0295 | T | \|\| id:ebi-a-GCST90001703 | ebi-a-GCST90001703 | TRUE | G | 37516789 | 1.49E-09 | reported | 3657 | 0.07243 | rs116921229 |  |  |  |  |  |  |  |  |  |  |  |  |  |  |  |
| -0.4153 | 22 | igd | 0.0295 | T | \|\| id:ebi-a-GCST90001704 | ebi-a-GCST90001704 | TRUE | G | 37516789 | 1.04E-08 | reported | 3657 | 0.07238 | rs116921229 |  |  |  |  |  |  |  |  |  |  |  |  |  |  |  |
| -0.4464 | 22 | igd | 0.0295 | T | \|\| id:ebi-a-GCST90001710 | ebi-a-GCST90001710 | TRUE | G | 37516789 | 7.02E-10 | reported | 3657 | 0.07221 | rs116921229 |  |  |  |  |  |  |  |  |  |  |  |  |  |  |  |
| -0.424 | 22 | igd | 0.0295 | T | \|\| id:ebi-a-GCST90001711 | ebi-a-GCST90001711 | TRUE | G | 37516789 | 4.46E-09 | reported | 3657 | 0.0721 | rs116921229 |  |  |  |  |  |  |  |  |  |  |  |  |  |  |  |
| -0.4663 | 10 | igd | 0.0294 | T | \|\| id:ebi-a-GCST90001489 | ebi-a-GCST90001489 | TRUE | C | 97254135 | 3.37E-09 | reported | 3405 | 0.07866 | rs76075467 |  |  |  |  |  |  |  |  |  |  |  |  |  |  |  |
| -0.5176 | 10 | igd | 0.0294 | T | \|\| id:ebi-a-GCST90001495 | ebi-a-GCST90001495 | TRUE | C | 97254135 | 8.36E-11 | reported | 3405 | 0.07946 | rs76075467 |  |  |  |  |  |  |  |  |  |  |  |  |  |  |  |
| 0.4904 | 10 | igd | 0.0294 | T | \|\| id:ebi-a-GCST90001670 | ebi-a-GCST90001670 | TRUE | G | 96677921 | 2.03E-10 | reported | 3440 | 0.07689 | rs116878692 |  |  |  |  |  |  |  |  |  |  |  |  |  |  |  |
| 0.492 | 10 | igd | 0.0294 | T | \|\| id:ebi-a-GCST90001671 | ebi-a-GCST90001671 | TRUE | G | 96677921 | 2.01E-10 | reported | 3440 | 0.07711 | rs116878692 |  |  |  |  |  |  |  |  |  |  |  |  |  |  |  |
| 0.5453 | 3 | igd | 0.0294 | A | \|\| id:ebi-a-GCST90001801 | ebi-a-GCST90001801 | TRUE | G | 32924804 | 4.44E-08 | reported | 1836 | 0.09922 | rs56237381 |  |  |  |  |  |  |  |  |  |  |  |  |  |  |  |
| -0.5573 | 10 | igd | 0.0293 | T | \|\| id:ebi-a-GCST90001660 | ebi-a-GCST90001660 | TRUE | C | 97254135 | 1.54E-12 | reported | 3408 | 0.07853 | rs76075467 |  |  |  |  |  |  |  |  |  |  |  |  |  |  |  |
| -0.5038 | 10 | igd | 0.0293 | T | \|\| id:ebi-a-GCST90001672 | ebi-a-GCST90001672 | TRUE | C | 97254135 | 2.54E-10 | reported | 3408 | 0.07942 | rs76075467 |  |  |  |  |  |  |  |  |  |  |  |  |  |  |  |
| 0.5446 | 10 | igd | 0.0293 | T | \|\| id:ebi-a-GCST90001672 | ebi-a-GCST90001672 | TRUE | G | 96677921 | 4.08E-12 | reported | 3408 | 0.07825 | rs116878692 |  |  |  |  |  |  |  |  |  |  |  |  |  |  |  |
| 0.4412 | 10 | igd | 0.0293 | G | \|\| id:ebi-a-GCST90002030 | ebi-a-GCST90002030 | TRUE | A | 97869375 | 1.24E-08 | reported | 2920 | 0.07727 | rs138709306 |  |  |  |  |  |  |  |  |  |  |  |  |  |  |  |
| 0.4706 | 10 | igd | 0.0293 | G | \|\| id:ebi-a-GCST90002031 | ebi-a-GCST90002031 | TRUE | A | 97869375 | 3.81E-09 | reported | 2920 | 0.07962 | rs138709306 |  |  |  |  |  |  |  |  |  |  |  |  |  |  |  |
| -0.4206 | 10 | igd | 0.0292 | T | \|\| id:ebi-a-GCST90001484 | ebi-a-GCST90001484 | TRUE | C | 97254135 | 3.34E-08 | reported | 3437 | 0.07598 | rs76075467 |  |  |  |  |  |  |  |  |  |  |  |  |  |  |  |
| -0.4814 | 10 | igd | 0.0292 | T | \|\| id:ebi-a-GCST90001491 | ebi-a-GCST90001491 | TRUE | C | 97254135 | 6.30E-10 | reported | 3437 | 0.07764 | rs76075467 |  |  |  |  |  |  |  |  |  |  |  |  |  |  |  |
| -0.4705 | 10 | igd | 0.0292 | T | \|\| id:ebi-a-GCST90001495 | ebi-a-GCST90001495 | TRUE | C | 98411629 | 1.29E-09 | reported | 3405 | 0.07732 | rs114696821 |  |  |  |  |  |  |  |  |  |  |  |  |  |  |  |
| -0.5269 | 10 | igd | 0.0292 | T | \|\| id:ebi-a-GCST90001496 | ebi-a-GCST90001496 | TRUE | C | 97254135 | 2.38E-11 | reported | 3437 | 0.07861 | rs76075467 |  |  |  |  |  |  |  |  |  |  |  |  |  |  |  |
| -0.4634 | 10 | igd | 0.0292 | T | \|\| id:ebi-a-GCST90001496 | ebi-a-GCST90001496 | TRUE | C | 98411629 | 1.51E-09 | reported | 3437 | 0.07648 | rs114696821 |  |  |  |  |  |  |  |  |  |  |  |  |  |  |  |
| -0.5431 | 10 | igd | 0.0292 | T | \|\| id:ebi-a-GCST90001497 | ebi-a-GCST90001497 | TRUE | C | 97254135 | 3.05E-12 | reported | 3437 | 0.07758 | rs76075467 |  |  |  |  |  |  |  |  |  |  |  |  |  |  |  |
| -0.4743 | 10 | igd | 0.0292 | T | \|\| id:ebi-a-GCST90001497 | ebi-a-GCST90001497 | TRUE | C | 98411629 | 3.72E-10 | reported | 3437 | 0.07549 | rs114696821 |  |  |  |  |  |  |  |  |  |  |  |  |  |  |  |
| -0.4265 | 10 | igd | 0.0292 | T | \|\| id:ebi-a-GCST90001658 | ebi-a-GCST90001658 | TRUE | C | 98411629 | 5.30E-09 | reported | 3440 | 0.07288 | rs114696821 |  |  |  |  |  |  |  |  |  |  |  |  |  |  |  |
| -0.5643 | 10 | igd | 0.0292 | T | \|\| id:ebi-a-GCST90001658 | ebi-a-GCST90001658 | TRUE | C | 97254135 | 6.26E-14 | reported | 3440 | 0.0749 | rs76075467 |  |  |  |  |  |  |  |  |  |  |  |  |  |  |  |
| -0.5709 | 10 | igd | 0.0292 | T | \|\| id:ebi-a-GCST90001659 | ebi-a-GCST90001659 | TRUE | C | 97254135 | 3.21E-14 | reported | 3440 | 0.0749 | rs76075467 |  |  |  |  |  |  |  |  |  |  |  |  |  |  |  |
| -0.4254 | 10 | igd | 0.0292 | T | \|\| id:ebi-a-GCST90001660 | ebi-a-GCST90001660 | TRUE | C | 98411629 | 2.83E-08 | reported | 3408 | 0.07644 | rs114696821 |  |  |  |  |  |  |  |  |  |  |  |  |  |  |  |
| -0.5194 | 10 | igd | 0.0292 | T | \|\| id:ebi-a-GCST90001670 | ebi-a-GCST90001670 | TRUE | C | 97254135 | 3.26E-11 | reported | 3440 | 0.07802 | rs76075467 |  |  |  |  |  |  |  |  |  |  |  |  |  |  |  |
| -0.4256 | 10 | igd | 0.0292 | T | \|\| id:ebi-a-GCST90001670 | ebi-a-GCST90001670 | TRUE | C | 98411629 | 2.27E-08 | reported | 3440 | 0.07596 | rs114696821 |  |  |  |  |  |  |  |  |  |  |  |  |  |  |  |
| -0.5317 | 10 | igd | 0.0292 | T | \|\| id:ebi-a-GCST90001671 | ebi-a-GCST90001671 | TRUE | C | 97254135 | 1.23E-11 | reported | 3440 | 0.07819 | rs76075467 |  |  |  |  |  |  |  |  |  |  |  |  |  |  |  |
| -0.4495 | 10 | igd | 0.0292 | T | \|\| id:ebi-a-GCST90001671 | ebi-a-GCST90001671 | TRUE | C | 98411629 | 3.57E-09 | reported | 3440 | 0.07596 | rs114696821 |  |  |  |  |  |  |  |  |  |  |  |  |  |  |  |
| -0.4592 | 10 | igd | 0.0292 | T | \|\| id:ebi-a-GCST90001672 | ebi-a-GCST90001672 | TRUE | C | 98411629 | 2.90E-09 | reported | 3408 | 0.07714 | rs114696821 |  |  |  |  |  |  |  |  |  |  |  |  |  |  |  |
| -0.4539 | 1 | igd | 0.0291 | G | \|\| id:ebi-a-GCST90001860 | ebi-a-GCST90001860 | TRUE | A | 167374213 | 2.85E-08 | reported | 2919 | 0.08155 | rs114838580 |  |  |  |  |  |  |  |  |  |  |  |  |  |  |  |
| 0.4433 | 10 | igd | 0.029 | T | \|\| id:ebi-a-GCST90001672 | ebi-a-GCST90001672 | TRUE | C | 101525037 | 1.25E-08 | reported | 3408 | 0.07767 | rs143176769 |  |  |  |  |  |  |  |  |  |  |  |  |  |  |  |
| -0.431 | 10 | igd | 0.0289 | G | \|\| id:ebi-a-GCST90001658 | ebi-a-GCST90001658 | TRUE | A | 97546627 | 9.01E-09 | reported | 3440 | 0.07478 | rs35748815 |  |  |  |  |  |  |  |  |  |  |  |  |  |  |  |
| 0.4337 | 6 | igd | 0.0289 | G | \|\| id:ebi-a-GCST90001661 | ebi-a-GCST90001661 | TRUE | A | 101593157 | 1.87E-08 | reported | 3440 | 0.07694 | rs79122093 |  |  |  |  |  |  |  |  |  |  |  |  |  |  |  |
| 0.4833 | 10 | igd | 0.0289 | A | \|\| id:ebi-a-GCST90001892 | ebi-a-GCST90001892 | TRUE | G | 96228883 | 3.99E-09 | reported | 2920 | 0.08188 | rs138352489 |  |  |  |  |  |  |  |  |  |  |  |  |  |  |  |
| -0.6393 | 10 | igd | 0.0289 | A | \|\| id:ebi-a-GCST90001961 | ebi-a-GCST90001961 | TRUE | G | 96293057 | 8.90E-15 | reported | 2920 | 0.08201 | rs117917573 |  |  |  |  |  |  |  |  |  |  |  |  |  |  |  |
| 0.5417 | 10 | igd | 0.0289 | A | \|\| id:ebi-a-GCST90002061 | ebi-a-GCST90002061 | TRUE | G | 96293057 | 7.78E-11 | reported | 2920 | 0.08297 | rs117917573 |  |  |  |  |  |  |  |  |  |  |  |  |  |  |  |
| -0.4365 | 10 | igd | 0.0288 | G | \|\| id:ebi-a-GCST90001495 | ebi-a-GCST90001495 | TRUE | A | 97546627 | 4.19E-08 | reported | 3405 | 0.07943 | rs35748815 |  |  |  |  |  |  |  |  |  |  |  |  |  |  |  |
| 0.7139 | 10 | igd | 0.0288 | A | \|\| id:ebi-a-GCST90001670 | ebi-a-GCST90001670 | TRUE | G | 96293057 | 1.85E-21 | reported | 3440 | 0.07454 | rs117917573 |  |  |  |  |  |  |  |  |  |  |  |  |  |  |  |
| 0.6631 | 10 | igd | 0.0288 | A | \|\| id:ebi-a-GCST90001671 | ebi-a-GCST90001671 | TRUE | G | 96293057 | 1.07E-18 | reported | 3440 | 0.07468 | rs117917573 |  |  |  |  |  |  |  |  |  |  |  |  |  |  |  |
| -0.4339 | 10 | igd | 0.0287 | G | \|\| id:ebi-a-GCST90001497 | ebi-a-GCST90001497 | TRUE | A | 97546627 | 2.53E-08 | reported | 3437 | 0.0777 | rs35748815 |  |  |  |  |  |  |  |  |  |  |  |  |  |  |  |
| 0.6017 | 6 | igd | 0.0287 | T | \|\| id:ebi-a-GCST90002105 | ebi-a-GCST90002105 | TRUE | C | 28315210 | 7.33E-13 | reported | 2872 | 0.08349 | rs117434283 |  |  |  |  |  |  |  |  |  |  |  |  |  |  |  |
| 0.7418 | 10 | igd | 0.0286 | A | \|\| id:ebi-a-GCST90001672 | ebi-a-GCST90001672 | TRUE | G | 96293057 | 2.54E-22 | reported | 3408 | 0.0758 | rs117917573 |  |  |  |  |  |  |  |  |  |  |  |  |  |  |  |
| -1.0727 | 19 | igd | 0.02858 | C | \|\| id:prot-a-439 | prot-a-439 | TRUE | G | 51761591 | 1.58E-41 | reported | 3301 | 0.0795 | rs62115998 |  |  |  |  |  |  |  |  |  |  |  |  |  |  |  |
| 0.4752 | 10 | igd | 0.0284 | T | \|\| id:ebi-a-GCST90001892 | ebi-a-GCST90001892 | TRUE | C | 96028656 | 1.04E-08 | reported | 2920 | 0.08277 | rs142022516 |  |  |  |  |  |  |  |  |  |  |  |  |  |  |  |
| -0.631 | 10 | igd | 0.0284 | T | \|\| id:ebi-a-GCST90001961 | ebi-a-GCST90001961 | TRUE | C | 96028656 | 4.01E-14 | reported | 2920 | 0.08304 | rs142022516 |  |  |  |  |  |  |  |  |  |  |  |  |  |  |  |
| 0.5385 | 10 | igd | 0.0284 | T | \|\| id:ebi-a-GCST90002061 | ebi-a-GCST90002061 | TRUE | C | 96028656 | 1.67E-10 | reported | 2920 | 0.08399 | rs142022516 |  |  |  |  |  |  |  |  |  |  |  |  |  |  |  |
| 0.7124 | 10 | igd | 0.0283 | T | \|\| id:ebi-a-GCST90001670 | ebi-a-GCST90001670 | TRUE | C | 96028656 | 5.60E-21 | reported | 3440 | 0.07531 | rs142022516 |  |  |  |  |  |  |  |  |  |  |  |  |  |  |  |
| 0.665 | 10 | igd | 0.0283 | T | \|\| id:ebi-a-GCST90001671 | ebi-a-GCST90001671 | TRUE | C | 96028656 | 1.86E-18 | reported | 3440 | 0.07544 | rs142022516 |  |  |  |  |  |  |  |  |  |  |  |  |  |  |  |
| 0.4138 | 20 | igd | 0.0283 | A | \|\| id:ebi-a-GCST90001798 | ebi-a-GCST90001798 | TRUE | G | 44895358 | 2.48E-08 | reported | 3657 | 0.07406 | rs77802711 |  |  |  |  |  |  |  |  |  |  |  |  |  |  |  |
| 0.4148 | 20 | igd | 0.0283 | A | \|\| id:ebi-a-GCST90001802 | ebi-a-GCST90001802 | TRUE | G | 44895358 | 3.23E-08 | reported | 3657 | 0.07487 | rs77802711 |  |  |  |  |  |  |  |  |  |  |  |  |  |  |  |
| 0.4628 | 20 | igd | 0.0283 | A | \|\| id:ebi-a-GCST90001804 | ebi-a-GCST90001804 | TRUE | G | 44895358 | 3.67E-10 | reported | 3657 | 0.07363 | rs77802711 |  |  |  |  |  |  |  |  |  |  |  |  |  |  |  |
| 0.4296 | 20 | igd | 0.0283 | A | \|\| id:ebi-a-GCST90001808 | ebi-a-GCST90001808 | TRUE | G | 44895358 | 9.32E-09 | reported | 3657 | 0.07464 | rs77802711 |  |  |  |  |  |  |  |  |  |  |  |  |  |  |  |
| -0.7118 | 17 | igd | 0.0283 | A | \|\| id:ebi-a-GCST90001926 | ebi-a-GCST90001926 | TRUE | G | 57241299 | 1.93E-18 | reported | 2920 | 0.08071 | rs140956807 |  |  |  |  |  |  |  |  |  |  |  |  |  |  |  |
| -0.7573 | 19 | igd | 0.0283 | C | \|\| id:ebi-a-GCST90001951 | ebi-a-GCST90001951 | TRUE | T | 51750043 | 9.41E-11 | reported | 1464 | 0.1161 | rs145323276 |  |  |  |  |  |  |  |  |  |  |  |  |  |  |  |
| -0.5186 | 10 | igd | 0.0283 | T | \|\| id:ebi-a-GCST90002030 | ebi-a-GCST90002030 | TRUE | C | 97254135 | 7.03E-10 | reported | 2920 | 0.08384 | rs76075467 |  |  |  |  |  |  |  |  |  |  |  |  |  |  |  |
| 0.7398 | 10 | igd | 0.0282 | T | \|\| id:ebi-a-GCST90001672 | ebi-a-GCST90001672 | TRUE | C | 96028656 | 8.51E-22 | reported | 3408 | 0.07659 | rs142022516 |  |  |  |  |  |  |  |  |  |  |  |  |  |  |  |
| -0.5243 | 6 | igd | 0.0282 | C | \|\| id:ebi-a-GCST90002105 | ebi-a-GCST90002105 | TRUE | A | 32848941 | 9.69E-10 | reported | 2872 | 0.08547 | rs9276902 |  |  |  |  |  |  |  |  |  |  |  |  |  |  |  |
| 0.6125 | 6 | igd | 0.0282 | C | \|\| id:ebi-a-GCST90002105 | ebi-a-GCST90002105 | TRUE | T | 28201103 | 4.90E-13 | reported | 2872 | 0.08434 | rs76437128 |  |  |  |  |  |  |  |  |  |  |  |  |  |  |  |
| 0.5237 | 7 | igd | 0.028 | A | \|\| id:ebi-a-GCST90002078 | ebi-a-GCST90002078 | TRUE | G | 92418979 | 9.41E-11 | reported | 3112 | 0.08059 | rs79912251 |  |  |  |  |  |  |  |  |  |  |  |  |  |  |  |
| -0.442 | 2 | igd | 0.028 | A | \|\| id:ebi-a-GCST90002087 | ebi-a-GCST90002087 | TRUE | G | 59592691 | 4.65E-08 | reported | 2872 | 0.08067 | rs75841521 |  |  |  |  |  |  |  |  |  |  |  |  |  |  |  |
| 0.665 | 6 | igd | 0.028 | G | \|\| id:ebi-a-GCST90002105 | ebi-a-GCST90002105 | TRUE | A | 28559487 | 2.72E-14 | reported | 2872 | 0.08693 | rs118025975 |  |  |  |  |  |  |  |  |  |  |  |  |  |  |  |
| 0.7685 | 10 | igd | 0.0279 | C | \|\| id:ebi-a-GCST90001672 | ebi-a-GCST90001672 | TRUE | A | 95931343 | 3.84E-23 | reported | 3408 | 0.077 | rs72814608 |  |  |  |  |  |  |  |  |  |  |  |  |  |  |  |
| -0.4345 | 6 | igd | 0.0279 | A | \|\| id:ebi-a-GCST90001765 | ebi-a-GCST90001765 | TRUE | G | 106936153 | 6.06E-09 | reported | 3655 | 0.07455 | rs137996519 |  |  |  |  |  |  |  |  |  |  |  |  |  |  |  |
| -0.4516 | 6 | igd | 0.0279 | A | \|\| id:ebi-a-GCST90001771 | ebi-a-GCST90001771 | TRUE | G | 106936153 | 1.65E-09 | reported | 3653 | 0.07472 | rs137996519 |  |  |  |  |  |  |  |  |  |  |  |  |  |  |  |
| -0.4758 | 6 | igd | 0.0279 | A | \|\| id:ebi-a-GCST90001773 | ebi-a-GCST90001773 | TRUE | G | 106936153 | 3.52E-10 | reported | 3652 | 0.07562 | rs137996519 |  |  |  |  |  |  |  |  |  |  |  |  |  |  |  |
| -0.7122 | 17 | igd | 0.0279 | A | \|\| id:ebi-a-GCST90001926 | ebi-a-GCST90001926 | TRUE | G | 56973826 | 2.64E-18 | reported | 2920 | 0.08108 | rs184451683 |  |  |  |  |  |  |  |  |  |  |  |  |  |  |  |
| 0.761 | 10 | igd | 0.0278 | C | \|\| id:ebi-a-GCST90001670 | ebi-a-GCST90001670 | TRUE | A | 95931343 | 2.62E-23 | reported | 3440 | 0.07595 | rs72814608 |  |  |  |  |  |  |  |  |  |  |  |  |  |  |  |
| 0.7063 | 10 | igd | 0.0278 | C | \|\| id:ebi-a-GCST90001671 | ebi-a-GCST90001671 | TRUE | A | 95931343 | 3.28E-20 | reported | 3440 | 0.07622 | rs72814608 |  |  |  |  |  |  |  |  |  |  |  |  |  |  |  |
| -0.5834 | 19 | igd | 0.0278 | G | \|\| id:ebi-a-GCST90001946 | ebi-a-GCST90001946 | TRUE | A | 48582916 | 4.18E-08 | reported | 1634 | 0.1059 | rs73051934 |  |  |  |  |  |  |  |  |  |  |  |  |  |  |  |
| -0.6094 | 19 | igd | 0.0278 | G | \|\| id:ebi-a-GCST90001949 | ebi-a-GCST90001949 | TRUE | A | 48582916 | 7.88E-09 | reported | 1634 | 0.105 | rs73051934 |  |  |  |  |  |  |  |  |  |  |  |  |  |  |  |
| -0.7524 | 17 | igd | 0.0277 | T | \|\| id:ebi-a-GCST90001926 | ebi-a-GCST90001926 | TRUE | C | 56385573 | 3.17E-20 | reported | 2920 | 0.08106 | rs138284624 |  |  |  |  |  |  |  |  |  |  |  |  |  |  |  |
| 0.6026 | 10 | igd | 0.0277 | G | \|\| id:ebi-a-GCST90002030 | ebi-a-GCST90002030 | TRUE | T | 97580324 | 4.66E-12 | reported | 2920 | 0.08677 | rs180727168 |  |  |  |  |  |  |  |  |  |  |  |  |  |  |  |
| 0.5548 | 10 | igd | 0.0277 | G | \|\| id:ebi-a-GCST90002031 | ebi-a-GCST90002031 | TRUE | T | 97580324 | 3.78E-10 | reported | 2920 | 0.08829 | rs180727168 |  |  |  |  |  |  |  |  |  |  |  |  |  |  |  |
| 0.5887 | 10 | igd | 0.0277 | G | \|\| id:ebi-a-GCST90002032 | ebi-a-GCST90002032 | TRUE | T | 97580324 | 3.03E-11 | reported | 2920 | 0.08825 | rs180727168 |  |  |  |  |  |  |  |  |  |  |  |  |  |  |  |
| -1.267 | 22 | igd | 0.0275 | A | \|\| id:ebi-a-GCST90001702 | ebi-a-GCST90001702 | TRUE | G | 41951320 | 2.97E-63 | reported | 3657 | 0.07402 | rs149414807 |  |  |  |  |  |  |  |  |  |  |  |  |  |  |  |
| -1.273 | 22 | igd | 0.0275 | A | \|\| id:ebi-a-GCST90001703 | ebi-a-GCST90001703 | TRUE | G | 41951320 | 6.84E-64 | reported | 3657 | 0.07398 | rs149414807 |  |  |  |  |  |  |  |  |  |  |  |  |  |  |  |
| -0.4244 | 22 | igd | 0.0275 | A | \|\| id:ebi-a-GCST90001704 | ebi-a-GCST90001704 | TRUE | C | 38664269 | 4.66E-08 | reported | 3657 | 0.0775 | rs140781542 |  |  |  |  |  |  |  |  |  |  |  |  |  |  |  |
| -1.298 | 22 | igd | 0.0275 | A | \|\| id:ebi-a-GCST90001704 | ebi-a-GCST90001704 | TRUE | G | 41951320 | 1.40E-66 | reported | 3657 | 0.07376 | rs149414807 |  |  |  |  |  |  |  |  |  |  |  |  |  |  |  |
| -1.244 | 22 | igd | 0.0275 | A | \|\| id:ebi-a-GCST90001710 | ebi-a-GCST90001710 | TRUE | G | 41951320 | 2.54E-61 | reported | 3657 | 0.0739 | rs149414807 |  |  |  |  |  |  |  |  |  |  |  |  |  |  |  |
| -1.261 | 22 | igd | 0.0275 | A | \|\| id:ebi-a-GCST90001711 | ebi-a-GCST90001711 | TRUE | G | 41951320 | 2.96E-63 | reported | 3657 | 0.07364 | rs149414807 |  |  |  |  |  |  |  |  |  |  |  |  |  |  |  |
| 0.6218 | 19 | igd | 0.0275 | G | \|\| id:ebi-a-GCST90001949 | ebi-a-GCST90001949 | TRUE | A | 52198419 | 3.39E-08 | reported | 1634 | 0.1121 | rs75614187 |  |  |  |  |  |  |  |  |  |  |  |  |  |  |  |
| 0.9085 | 6 | igd | 0.0275 | A | \|\| id:ebi-a-GCST90002108 | ebi-a-GCST90002108 | TRUE | G | 32653156 | 4.18E-09 | reported | 1579 | 0.1537 | rs34447121 |  |  |  |  |  |  |  |  |  |  |  |  |  |  |  |
| -0.5589 | 22 | igd | 0.0274 | A | \|\| id:ebi-a-GCST90001701 | ebi-a-GCST90001701 | TRUE | C | 41906827 | 1.04E-13 | reported | 3592 | 0.07486 | rs76120255 |  |  |  |  |  |  |  |  |  |  |  |  |  |  |  |
| 0.5084 | 10 | igd | 0.0274 | C | \|\| id:ebi-a-GCST90001892 | ebi-a-GCST90001892 | TRUE | A | 95931343 | 9.76E-10 | reported | 2920 | 0.08289 | rs72814608 |  |  |  |  |  |  |  |  |  |  |  |  |  |  |  |
| -0.6587 | 19 | igd | 0.0274 | C | \|\| id:ebi-a-GCST90001952 | ebi-a-GCST90001952 | TRUE | T | 51750043 | 6.21E-09 | reported | 1569 | 0.1127 | rs145323276 |  |  |  |  |  |  |  |  |  |  |  |  |  |  |  |
| -0.6523 | 10 | igd | 0.0274 | C | \|\| id:ebi-a-GCST90001961 | ebi-a-GCST90001961 | TRUE | A | 95931343 | 6.97E-15 | reported | 2920 | 0.08335 | rs72814608 |  |  |  |  |  |  |  |  |  |  |  |  |  |  |  |
| 0.6285 | 10 | igd | 0.0274 | C | \|\| id:ebi-a-GCST90002061 | ebi-a-GCST90002061 | TRUE | A | 95931343 | 9.29E-14 | reported | 2920 | 0.08394 | rs72814608 |  |  |  |  |  |  |  |  |  |  |  |  |  |  |  |
| 0.4819 | 6 | igd | 0.0273 | G | \|\| id:ebi-a-GCST90001661 | ebi-a-GCST90001661 | TRUE | A | 101980039 | 1.47E-09 | reported | 3440 | 0.07946 | rs117147408 |  |  |  |  |  |  |  |  |  |  |  |  |  |  |  |
| -0.5863 | 22 | igd | 0.0273 | T | \|\| id:ebi-a-GCST90001703 | ebi-a-GCST90001703 | TRUE | C | 43610730 | 3.72E-14 | reported | 3657 | 0.07714 | rs145628748 |  |  |  |  |  |  |  |  |  |  |  |  |  |  |  |
| -0.6139 | 22 | igd | 0.0273 | T | \|\| id:ebi-a-GCST90001704 | ebi-a-GCST90001704 | TRUE | C | 43610730 | 2.03E-15 | reported | 3657 | 0.07698 | rs145628748 |  |  |  |  |  |  |  |  |  |  |  |  |  |  |  |
| -0.5668 | 22 | igd | 0.0273 | T | \|\| id:ebi-a-GCST90001711 | ebi-a-GCST90001711 | TRUE | C | 43610730 | 1.94E-13 | reported | 3657 | 0.07679 | rs145628748 |  |  |  |  |  |  |  |  |  |  |  |  |  |  |  |
| -0.7368 | 19 | igd | 0.0273 | A | \|\| id:ebi-a-GCST90001950 | ebi-a-GCST90001950 | TRUE | T | 51732079 | 3.33E-09 | reported | 1244 | 0.1237 | rs149201050 |  |  |  |  |  |  |  |  |  |  |  |  |  |  |  |
| 1.176 | 10 | igd | 0.0272 | A | \|\| id:ebi-a-GCST90001484 | ebi-a-GCST90001484 | TRUE | G | 97688985 | 1.42E-52 | reported | 3437 | 0.07577 | rs138593770 |  |  |  |  |  |  |  |  |  |  |  |  |  |  |  |
| 1.193 | 10 | igd | 0.0272 | A | \|\| id:ebi-a-GCST90001484 | ebi-a-GCST90001484 | TRUE | G | 97569685 | 7.03E-54 | reported | 3437 | 0.07585 | rs138394023 |  |  |  |  |  |  |  |  |  |  |  |  |  |  |  |
| 1.104 | 10 | igd | 0.0272 | C | \|\| id:ebi-a-GCST90001485 | ebi-a-GCST90001485 | TRUE | G | 97566321 | 2.58E-46 | reported | 3437 | 0.07613 | rs10786238 |  |  |  |  |  |  |  |  |  |  |  |  |  |  |  |
| 1.098 | 10 | igd | 0.0272 | A | \|\| id:ebi-a-GCST90001485 | ebi-a-GCST90001485 | TRUE | G | 97688985 | 6.12E-46 | reported | 3437 | 0.076 | rs138593770 |  |  |  |  |  |  |  |  |  |  |  |  |  |  |  |
| 0.7773 | 10 | igd | 0.0272 | A | \|\| id:ebi-a-GCST90001491 | ebi-a-GCST90001491 | TRUE | G | 97569685 | 1.99E-22 | reported | 3437 | 0.07923 | rs138394023 |  |  |  |  |  |  |  |  |  |  |  |  |  |  |  |
| 0.7374 | 10 | igd | 0.0272 | A | \|\| id:ebi-a-GCST90001491 | ebi-a-GCST90001491 | TRUE | G | 97688985 | 2.12E-20 | reported | 3437 | 0.07916 | rs138593770 |  |  |  |  |  |  |  |  |  |  |  |  |  |  |  |
| 1.082 | 10 | igd | 0.0272 | A | \|\| id:ebi-a-GCST90001496 | ebi-a-GCST90001496 | TRUE | G | 97569685 | 2.33E-41 | reported | 3437 | 0.07929 | rs138394023 |  |  |  |  |  |  |  |  |  |  |  |  |  |  |  |
| 1.045 | 10 | igd | 0.0272 | A | \|\| id:ebi-a-GCST90001496 | ebi-a-GCST90001496 | TRUE | G | 97688985 | 8.87E-39 | reported | 3437 | 0.07925 | rs138593770 |  |  |  |  |  |  |  |  |  |  |  |  |  |  |  |
| 0.9742 | 10 | igd | 0.0272 | A | \|\| id:ebi-a-GCST90001497 | ebi-a-GCST90001497 | TRUE | G | 97688985 | 1.18E-34 | reported | 3437 | 0.07847 | rs138593770 |  |  |  |  |  |  |  |  |  |  |  |  |  |  |  |
| 1.011 | 10 | igd | 0.0272 | A | \|\| id:ebi-a-GCST90001497 | ebi-a-GCST90001497 | TRUE | G | 97569685 | 4.51E-37 | reported | 3437 | 0.07852 | rs138394023 |  |  |  |  |  |  |  |  |  |  |  |  |  |  |  |
| 1.132 | 10 | igd | 0.0272 | A | \|\| id:ebi-a-GCST90001658 | ebi-a-GCST90001658 | TRUE | G | 97569685 | 1.42E-49 | reported | 3440 | 0.07522 | rs138394023 |  |  |  |  |  |  |  |  |  |  |  |  |  |  |  |
| 1.103 | 10 | igd | 0.0272 | C | \|\| id:ebi-a-GCST90001658 | ebi-a-GCST90001658 | TRUE | G | 97719086 | 2.47E-47 | reported | 3440 | 0.07514 | rs77335265 |  |  |  |  |  |  |  |  |  |  |  |  |  |  |  |
| 1.143 | 10 | igd | 0.0272 | C | \|\| id:ebi-a-GCST90001659 | ebi-a-GCST90001659 | TRUE | G | 97718352 | 7.76E-51 | reported | 3440 | 0.07498 | rs116734143 |  |  |  |  |  |  |  |  |  |  |  |  |  |  |  |
| 1.175 | 10 | igd | 0.0272 | A | \|\| id:ebi-a-GCST90001659 | ebi-a-GCST90001659 | TRUE | G | 97569685 | 1.83E-53 | reported | 3440 | 0.07504 | rs138394023 |  |  |  |  |  |  |  |  |  |  |  |  |  |  |  |
| 1.025 | 10 | igd | 0.0272 | A | \|\| id:ebi-a-GCST90001670 | ebi-a-GCST90001670 | TRUE | G | 97688985 | 6.60E-38 | reported | 3440 | 0.07866 | rs138593770 |  |  |  |  |  |  |  |  |  |  |  |  |  |  |  |
| 1.051 | 10 | igd | 0.0272 | A | \|\| id:ebi-a-GCST90001670 | ebi-a-GCST90001670 | TRUE | G | 97569685 | 1.10E-39 | reported | 3440 | 0.07874 | rs138394023 |  |  |  |  |  |  |  |  |  |  |  |  |  |  |  |
| 1.052 | 10 | igd | 0.0272 | C | \|\| id:ebi-a-GCST90001671 | ebi-a-GCST90001671 | TRUE | G | 97718352 | 1.05E-39 | reported | 3440 | 0.07873 | rs116734143 |  |  |  |  |  |  |  |  |  |  |  |  |  |  |  |
| 1.076 | 10 | igd | 0.0272 | A | \|\| id:ebi-a-GCST90001671 | ebi-a-GCST90001671 | TRUE | G | 97569685 | 2.51E-41 | reported | 3440 | 0.07884 | rs138394023 |  |  |  |  |  |  |  |  |  |  |  |  |  |  |  |
| 0.4519 | 3 | igd | 0.0272 | A | \|\| id:ebi-a-GCST90001800 | ebi-a-GCST90001800 | TRUE | G | 32924804 | 5.82E-09 | reported | 3656 | 0.07743 | rs56237381 |  |  |  |  |  |  |  |  |  |  |  |  |  |  |  |
| 0.4286 | 3 | igd | 0.0272 | A | \|\| id:ebi-a-GCST90001805 | ebi-a-GCST90001805 | TRUE | G | 32924804 | 2.95E-08 | reported | 3657 | 0.07713 | rs56237381 |  |  |  |  |  |  |  |  |  |  |  |  |  |  |  |
| 0.4379 | 3 | igd | 0.0272 | A | \|\| id:ebi-a-GCST90001806 | ebi-a-GCST90001806 | TRUE | G | 32924804 | 1.39E-08 | reported | 3656 | 0.077 | rs56237381 |  |  |  |  |  |  |  |  |  |  |  |  |  |  |  |
| -0.4685 | 1 | igd | 0.0272 | A | \|\| id:ebi-a-GCST90001863 | ebi-a-GCST90001863 | TRUE | G | 165449377 | 1.91E-08 | reported | 2919 | 0.08313 | rs10800103 |  |  |  |  |  |  |  |  |  |  |  |  |  |  |  |
| -0.5738 | 1 | igd | 0.0272 | A | \|\| id:ebi-a-GCST90001864 | ebi-a-GCST90001864 | TRUE | G | 165449377 | 1.32E-11 | reported | 2919 | 0.08447 | rs10800103 |  |  |  |  |  |  |  |  |  |  |  |  |  |  |  |
| 0.9251 | 6 | igd | 0.0272 | A | \|\| id:ebi-a-GCST90002110 | ebi-a-GCST90002110 | TRUE | G | 32653156 | 1.99E-09 | reported | 1634 | 0.1533 | rs34447121 |  |  |  |  |  |  |  |  |  |  |  |  |  |  |  |
| 0.9151 | 6 | igd | 0.0272 | A | \|\| id:ebi-a-GCST90002111 | ebi-a-GCST90002111 | TRUE | G | 32653156 | 1.75E-09 | reported | 1635 | 0.1512 | rs34447121 |  |  |  |  |  |  |  |  |  |  |  |  |  |  |  |
| 0.8614 | 6 | igd | 0.0272 | A | \|\| id:ebi-a-GCST90002112 | ebi-a-GCST90002112 | TRUE | G | 32653156 | 2.15E-08 | reported | 1635 | 0.1531 | rs34447121 |  |  |  |  |  |  |  |  |  |  |  |  |  |  |  |
| -0.4791 | 22 | igd | 0.0271 | G | \|\| id:ebi-a-GCST90001702 | ebi-a-GCST90001702 | TRUE | A | 44688211 | 1.05E-09 | reported | 3657 | 0.07831 | rs151125173 |  |  |  |  |  |  |  |  |  |  |  |  |  |  |  |
| -0.4816 | 22 | igd | 0.0271 | G | \|\| id:ebi-a-GCST90001703 | ebi-a-GCST90001703 | TRUE | A | 44688211 | 8.52E-10 | reported | 3657 | 0.0783 | rs151125173 |  |  |  |  |  |  |  |  |  |  |  |  |  |  |  |
| -0.4955 | 22 | igd | 0.0271 | G | \|\| id:ebi-a-GCST90001704 | ebi-a-GCST90001704 | TRUE | A | 44688211 | 2.60E-10 | reported | 3657 | 0.07817 | rs151125173 |  |  |  |  |  |  |  |  |  |  |  |  |  |  |  |
| -0.4746 | 22 | igd | 0.0271 | G | \|\| id:ebi-a-GCST90001710 | ebi-a-GCST90001710 | TRUE | A | 44688211 | 1.35E-09 | reported | 3657 | 0.07809 | rs151125173 |  |  |  |  |  |  |  |  |  |  |  |  |  |  |  |
| -0.49 | 22 | igd | 0.0271 | G | \|\| id:ebi-a-GCST90001711 | ebi-a-GCST90001711 | TRUE | A | 44688211 | 3.54E-10 | reported | 3657 | 0.07789 | rs151125173 |  |  |  |  |  |  |  |  |  |  |  |  |  |  |  |
| -0.502 | 1 | igd | 0.0271 | A | \|\| id:ebi-a-GCST90001845 | ebi-a-GCST90001845 | TRUE | G | 165449377 | 3.58E-09 | reported | 2911 | 0.08479 | rs10800103 |  |  |  |  |  |  |  |  |  |  |  |  |  |  |  |
| -0.4868 | 10 | igd | 0.0271 | T | \|\| id:ebi-a-GCST90002032 | ebi-a-GCST90002032 | TRUE | C | 99016770 | 2.27E-08 | reported | 2920 | 0.08683 | rs147848988 |  |  |  |  |  |  |  |  |  |  |  |  |  |  |  |
| 0.4453 | 2 | igd | 0.0271 | T | \|\| id:ebi-a-GCST90002102 | ebi-a-GCST90002102 | TRUE | G | 40064621 | 2.96E-08 | reported | 2919 | 0.0801 | rs55928361 |  |  |  |  |  |  |  |  |  |  |  |  |  |  |  |
| 1.064 | 10 | igd | 0.027 | A | \|\| id:ebi-a-GCST90001483 | ebi-a-GCST90001483 | TRUE | G | 97688985 | 2.90E-42 | reported | 3405 | 0.07705 | rs138593770 |  |  |  |  |  |  |  |  |  |  |  |  |  |  |  |
| 1.068 | 10 | igd | 0.027 | A | \|\| id:ebi-a-GCST90001483 | ebi-a-GCST90001483 | TRUE | G | 97569685 | 1.99E-42 | reported | 3405 | 0.07719 | rs138394023 |  |  |  |  |  |  |  |  |  |  |  |  |  |  |  |
| 0.7588 | 10 | igd | 0.027 | T | \|\| id:ebi-a-GCST90001489 | ebi-a-GCST90001489 | TRUE | G | 97464047 | 3.78E-20 | reported | 3405 | 0.08201 | rs11188441 |  |  |  |  |  |  |  |  |  |  |  |  |  |  |  |
| 0.7145 | 10 | igd | 0.027 | A | \|\| id:ebi-a-GCST90001489 | ebi-a-GCST90001489 | TRUE | G | 97688985 | 1.01E-18 | reported | 3405 | 0.08041 | rs138593770 |  |  |  |  |  |  |  |  |  |  |  |  |  |  |  |
| 0.9122 | 10 | igd | 0.027 | A | \|\| id:ebi-a-GCST90001495 | ebi-a-GCST90001495 | TRUE | G | 97688985 | 4.11E-29 | reported | 3405 | 0.08069 | rs138593770 |  |  |  |  |  |  |  |  |  |  |  |  |  |  |  |
| 0.9424 | 10 | igd | 0.027 | A | \|\| id:ebi-a-GCST90001495 | ebi-a-GCST90001495 | TRUE | G | 97569685 | 7.34E-31 | reported | 3405 | 0.08078 | rs138394023 |  |  |  |  |  |  |  |  |  |  |  |  |  |  |  |
| 0.4375 | 10 | igd | 0.027 | G | \|\| id:ebi-a-GCST90001495 | ebi-a-GCST90001495 | TRUE | C | 97188433 | 1.30E-08 | reported | 3405 | 0.07677 | rs111455423 |  |  |  |  |  |  |  |  |  |  |  |  |  |  |  |
| 0.4262 | 10 | igd | 0.027 | G | \|\| id:ebi-a-GCST90001660 | ebi-a-GCST90001660 | TRUE | C | 97188433 | 2.06E-08 | reported | 3408 | 0.07583 | rs111455423 |  |  |  |  |  |  |  |  |  |  |  |  |  |  |  |
| 1.198 | 10 | igd | 0.027 | A | \|\| id:ebi-a-GCST90001660 | ebi-a-GCST90001660 | TRUE | G | 97569685 | 1.78E-50 | reported | 3408 | 0.07886 | rs138394023 |  |  |  |  |  |  |  |  |  |  |  |  |  |  |  |
| 1.159 | 10 | igd | 0.027 | G | \|\| id:ebi-a-GCST90001660 | ebi-a-GCST90001660 | TRUE | C | 97698274 | 1.84E-47 | reported | 3408 | 0.07883 | rs115526095 |  |  |  |  |  |  |  |  |  |  |  |  |  |  |  |
| 1.1 | 10 | igd | 0.027 | A | \|\| id:ebi-a-GCST90001672 | ebi-a-GCST90001672 | TRUE | G | 97569685 | 8.80E-42 | reported | 3408 | 0.0801 | rs138394023 |  |  |  |  |  |  |  |  |  |  |  |  |  |  |  |
| 1.067 | 10 | igd | 0.027 | A | \|\| id:ebi-a-GCST90001672 | ebi-a-GCST90001672 | TRUE | G | 97688985 | 1.45E-39 | reported | 3408 | 0.08003 | rs138593770 |  |  |  |  |  |  |  |  |  |  |  |  |  |  |  |
| 0.5012 | 6 | igd | 0.027 | C | \|\| id:ebi-a-GCST90002105 | ebi-a-GCST90002105 | TRUE | T | 27097731 | 5.85E-09 | reported | 2872 | 0.08585 | rs11755517 |  |  |  |  |  |  |  |  |  |  |  |  |  |  |  |
| 0.5018 | 6 | igd | 0.027 | C | \|\| id:ebi-a-GCST90002105 | ebi-a-GCST90002105 | TRUE | T | 26640615 | 5.77E-09 | reported | 2872 | 0.08591 | rs11752310 |  |  |  |  |  |  |  |  |  |  |  |  |  |  |  |
| 0.505 | 6 | igd | 0.027 | A | \|\| id:ebi-a-GCST90002105 | ebi-a-GCST90002105 | TRUE | G | 26802790 | 5.08E-09 | reported | 2872 | 0.08614 | rs139509968 |  |  |  |  |  |  |  |  |  |  |  |  |  |  |  |
| -0.5127 | 10 | igd | 0.0269 | C | \|\| id:ebi-a-GCST90001961 | ebi-a-GCST90001961 | TRUE | T | 96890004 | 2.20E-09 | reported | 2920 | 0.08543 | rs143132810 |  |  |  |  |  |  |  |  |  |  |  |  |  |  |  |
| 0.5823 | 10 | igd | 0.0269 | C | \|\| id:ebi-a-GCST90002030 | ebi-a-GCST90002030 | TRUE | T | 96890004 | 9.54E-12 | reported | 2920 | 0.08511 | rs143132810 |  |  |  |  |  |  |  |  |  |  |  |  |  |  |  |
| 0.5491 | 10 | igd | 0.0268 | G | \|\| id:ebi-a-GCST90001484 | ebi-a-GCST90001484 | TRUE | C | 97188433 | 1.25E-13 | reported | 3437 | 0.07378 | rs111455423 |  |  |  |  |  |  |  |  |  |  |  |  |  |  |  |
| 0.443 | 10 | igd | 0.0268 | G | \|\| id:ebi-a-GCST90001485 | ebi-a-GCST90001485 | TRUE | C | 97188433 | 2.24E-09 | reported | 3437 | 0.07389 | rs111455423 |  |  |  |  |  |  |  |  |  |  |  |  |  |  |  |
| 0.4485 | 10 | igd | 0.0268 | G | \|\| id:ebi-a-GCST90001491 | ebi-a-GCST90001491 | TRUE | C | 97188433 | 2.67E-09 | reported | 3437 | 0.07516 | rs111455423 |  |  |  |  |  |  |  |  |  |  |  |  |  |  |  |
| 0.5082 | 10 | igd | 0.0268 | G | \|\| id:ebi-a-GCST90001496 | ebi-a-GCST90001496 | TRUE | C | 97188433 | 2.76E-11 | reported | 3437 | 0.07607 | rs111455423 |  |  |  |  |  |  |  |  |  |  |  |  |  |  |  |
| 0.5621 | 10 | igd | 0.0268 | G | \|\| id:ebi-a-GCST90001497 | ebi-a-GCST90001497 | TRUE | C | 97188433 | 8.30E-14 | reported | 3437 | 0.07499 | rs111455423 |  |  |  |  |  |  |  |  |  |  |  |  |  |  |  |
| -0.5343 | 1 | igd | 0.0267 | T | \|\| id:ebi-a-GCST90001474 | ebi-a-GCST90001474 | TRUE | C | 161675994 | 1.91E-12 | reported | 3410 | 0.07561 | rs72704075 |  |  |  |  |  |  |  |  |  |  |  |  |  |  |  |
| 0.4382 | 10 | igd | 0.0267 | C | \|\| id:ebi-a-GCST90001658 | ebi-a-GCST90001658 | TRUE | A | 96752148 | 7.28E-09 | reported | 3440 | 0.07557 | rs61886805 |  |  |  |  |  |  |  |  |  |  |  |  |  |  |  |
| 0.4378 | 10 | igd | 0.0267 | G | \|\| id:ebi-a-GCST90001658 | ebi-a-GCST90001658 | TRUE | C | 97188433 | 1.75E-09 | reported | 3440 | 0.07253 | rs111455423 |  |  |  |  |  |  |  |  |  |  |  |  |  |  |  |
| 0.4431 | 10 | igd | 0.0267 | C | \|\| id:ebi-a-GCST90001659 | ebi-a-GCST90001659 | TRUE | A | 96752148 | 4.95E-09 | reported | 3440 | 0.07557 | rs61886805 |  |  |  |  |  |  |  |  |  |  |  |  |  |  |  |
| 0.4522 | 10 | igd | 0.0267 | G | \|\| id:ebi-a-GCST90001659 | ebi-a-GCST90001659 | TRUE | C | 97188433 | 5.04E-10 | reported | 3440 | 0.07252 | rs111455423 |  |  |  |  |  |  |  |  |  |  |  |  |  |  |  |
| -0.8545 | 10 | igd | 0.0267 | A | \|\| id:ebi-a-GCST90001961 | ebi-a-GCST90001961 | TRUE | G | 97569685 | 7.37E-23 | reported | 2920 | 0.08609 | rs138394023 |  |  |  |  |  |  |  |  |  |  |  |  |  |  |  |
| -0.837 | 10 | igd | 0.0267 | T | \|\| id:ebi-a-GCST90001961 | ebi-a-GCST90001961 | TRUE | C | 97725896 | 4.49E-22 | reported | 2920 | 0.08595 | rs7912552 |  |  |  |  |  |  |  |  |  |  |  |  |  |  |  |
| 0.7754 | 10 | igd | 0.0267 | A | \|\| id:ebi-a-GCST90002030 | ebi-a-GCST90002030 | TRUE | G | 97569685 | 3.34E-19 | reported | 2920 | 0.08597 | rs138394023 |  |  |  |  |  |  |  |  |  |  |  |  |  |  |  |
| 0.7469 | 10 | igd | 0.0267 | C | \|\| id:ebi-a-GCST90002030 | ebi-a-GCST90002030 | TRUE | A | 97721764 | 5.57E-18 | reported | 2920 | 0.08588 | rs2901895 |  |  |  |  |  |  |  |  |  |  |  |  |  |  |  |
| 0.5553 | 10 | igd | 0.0267 | G | \|\| id:ebi-a-GCST90002031 | ebi-a-GCST90002031 | TRUE | C | 97698274 | 3.18E-10 | reported | 2920 | 0.08798 | rs115526095 |  |  |  |  |  |  |  |  |  |  |  |  |  |  |  |
| 0.5783 | 10 | igd | 0.0267 | A | \|\| id:ebi-a-GCST90002031 | ebi-a-GCST90002031 | TRUE | G | 97569685 | 5.93E-11 | reported | 2920 | 0.08802 | rs138394023 |  |  |  |  |  |  |  |  |  |  |  |  |  |  |  |
| -0.7346 | 19 | igd | 0.0266 | C | \|\| id:ebi-a-GCST90001947 | ebi-a-GCST90001947 | TRUE | T | 51750043 | 1.94E-10 | reported | 1577 | 0.1146 | rs145323276 |  |  |  |  |  |  |  |  |  |  |  |  |  |  |  |
| -0.7516 | 19 | igd | 0.0266 | C | \|\| id:ebi-a-GCST90001956 | ebi-a-GCST90001956 | TRUE | T | 51750043 | 7.14E-11 | reported | 1579 | 0.1145 | rs145323276 |  |  |  |  |  |  |  |  |  |  |  |  |  |  |  |
| 0.4676 | 12 | igd | 0.0266 | A | \|\| id:ebi-a-GCST90002071 | ebi-a-GCST90002071 | TRUE | G | 25434182 | 2.89E-08 | reported | 2872 | 0.08405 | rs139947059 |  |  |  |  |  |  |  |  |  |  |  |  |  |  |  |
| 0.5214 | 10 | igd | 0.0265 | T | \|\| id:ebi-a-GCST90001888 | ebi-a-GCST90001888 | TRUE | C | 97260725 | 9.12E-10 | reported | 2920 | 0.08486 | rs142105763 |  |  |  |  |  |  |  |  |  |  |  |  |  |  |  |
| 0.5761 | 10 | igd | 0.0265 | T | \|\| id:ebi-a-GCST90001892 | ebi-a-GCST90001892 | TRUE | C | 97260725 | 3.09E-11 | reported | 2920 | 0.0864 | rs142105763 |  |  |  |  |  |  |  |  |  |  |  |  |  |  |  |
| -0.7779 | 10 | igd | 0.0265 | T | \|\| id:ebi-a-GCST90001961 | ebi-a-GCST90001961 | TRUE | C | 97260725 | 5.46E-19 | reported | 2920 | 0.08677 | rs142105763 |  |  |  |  |  |  |  |  |  |  |  |  |  |  |  |
| 0.8394 | 10 | igd | 0.0265 | T | \|\| id:ebi-a-GCST90002061 | ebi-a-GCST90002061 | TRUE | C | 97260725 | 1.12E-21 | reported | 2920 | 0.08706 | rs142105763 |  |  |  |  |  |  |  |  |  |  |  |  |  |  |  |
| 0.9945 | 6 | igd | 0.0265 | A | \|\| id:ebi-a-GCST90002105 | ebi-a-GCST90002105 | TRUE | G | 32653156 | 6.65E-16 | reported | 2872 | 0.1224 | rs34447121 |  |  |  |  |  |  |  |  |  |  |  |  |  |  |  |
| -0.5812 | 1 | igd | 0.0264 | T | \|\| id:ebi-a-GCST90001970 | ebi-a-GCST90001970 | TRUE | C | 161675994 | 4.24E-12 | reported | 2971 | 0.08353 | rs72704075 |  |  |  |  |  |  |  |  |  |  |  |  |  |  |  |
| -0.4757 | 1 | igd | 0.0264 | A | \|\| id:ebi-a-GCST90001970 | ebi-a-GCST90001970 | TRUE | G | 161908877 | 1.73E-08 | reported | 2971 | 0.08416 | rs72708099 |  |  |  |  |  |  |  |  |  |  |  |  |  |  |  |
| -0.4844 | 1 | igd | 0.0264 | A | \|\| id:ebi-a-GCST90002077 | ebi-a-GCST90002077 | TRUE | G | 161908877 | 8.14E-09 | reported | 2971 | 0.08377 | rs72708099 |  |  |  |  |  |  |  |  |  |  |  |  |  |  |  |
| -0.5694 | 1 | igd | 0.0264 | T | \|\| id:ebi-a-GCST90002077 | ebi-a-GCST90002077 | TRUE | C | 161675994 | 9.32E-12 | reported | 2971 | 0.0832 | rs72704075 |  |  |  |  |  |  |  |  |  |  |  |  |  |  |  |
| 0.6358 | 10 | igd | 0.0263 | C | \|\| id:ebi-a-GCST90001483 | ebi-a-GCST90001483 | TRUE | T | 96890004 | 1.07E-15 | reported | 3405 | 0.0789 | rs143132810 |  |  |  |  |  |  |  |  |  |  |  |  |  |  |  |
| 0.7376 | 10 | igd | 0.0263 | C | \|\| id:ebi-a-GCST90001484 | ebi-a-GCST90001484 | TRUE | T | 96890004 | 4.91E-21 | reported | 3437 | 0.07787 | rs143132810 |  |  |  |  |  |  |  |  |  |  |  |  |  |  |  |
| 0.6572 | 10 | igd | 0.0263 | C | \|\| id:ebi-a-GCST90001485 | ebi-a-GCST90001485 | TRUE | T | 96890004 | 5.02E-17 | reported | 3437 | 0.07797 | rs143132810 |  |  |  |  |  |  |  |  |  |  |  |  |  |  |  |
| 0.5409 | 10 | igd | 0.0263 | C | \|\| id:ebi-a-GCST90001489 | ebi-a-GCST90001489 | TRUE | T | 96890004 | 3.31E-11 | reported | 3405 | 0.08129 | rs143132810 |  |  |  |  |  |  |  |  |  |  |  |  |  |  |  |
| 0.5497 | 10 | igd | 0.0263 | C | \|\| id:ebi-a-GCST90001491 | ebi-a-GCST90001491 | TRUE | T | 96890004 | 8.08E-12 | reported | 3437 | 0.08012 | rs143132810 |  |  |  |  |  |  |  |  |  |  |  |  |  |  |  |
| 0.6882 | 10 | igd | 0.0263 | C | \|\| id:ebi-a-GCST90001495 | ebi-a-GCST90001495 | TRUE | T | 96890004 | 6.13E-17 | reported | 3405 | 0.08186 | rs143132810 |  |  |  |  |  |  |  |  |  |  |  |  |  |  |  |
| 0.718 | 10 | igd | 0.0263 | C | \|\| id:ebi-a-GCST90001496 | ebi-a-GCST90001496 | TRUE | T | 96890004 | 1.03E-18 | reported | 3437 | 0.08083 | rs143132810 |  |  |  |  |  |  |  |  |  |  |  |  |  |  |  |
| 0.7283 | 10 | igd | 0.0263 | C | \|\| id:ebi-a-GCST90001497 | ebi-a-GCST90001497 | TRUE | T | 96890004 | 1.14E-19 | reported | 3437 | 0.07976 | rs143132810 |  |  |  |  |  |  |  |  |  |  |  |  |  |  |  |
| 0.6299 | 10 | igd | 0.0263 | C | \|\| id:ebi-a-GCST90001658 | ebi-a-GCST90001658 | TRUE | T | 96890004 | 4.87E-16 | reported | 3440 | 0.07725 | rs143132810 |  |  |  |  |  |  |  |  |  |  |  |  |  |  |  |
| 0.6855 | 10 | igd | 0.0263 | C | \|\| id:ebi-a-GCST90001659 | ebi-a-GCST90001659 | TRUE | T | 96890004 | 9.80E-19 | reported | 3440 | 0.07712 | rs143132810 |  |  |  |  |  |  |  |  |  |  |  |  |  |  |  |
| 0.753 | 10 | igd | 0.0263 | C | \|\| id:ebi-a-GCST90001660 | ebi-a-GCST90001660 | TRUE | T | 96890004 | 2.01E-20 | reported | 3408 | 0.08079 | rs143132810 |  |  |  |  |  |  |  |  |  |  |  |  |  |  |  |
| 0.574 | 10 | igd | 0.0263 | C | \|\| id:ebi-a-GCST90001670 | ebi-a-GCST90001670 | TRUE | T | 96890004 | 1.22E-12 | reported | 3440 | 0.08051 | rs143132810 |  |  |  |  |  |  |  |  |  |  |  |  |  |  |  |
| 0.5744 | 10 | igd | 0.0263 | C | \|\| id:ebi-a-GCST90001671 | ebi-a-GCST90001671 | TRUE | T | 96890004 | 1.34E-12 | reported | 3440 | 0.0807 | rs143132810 |  |  |  |  |  |  |  |  |  |  |  |  |  |  |  |
| 0.8065 | 10 | igd | 0.0263 | T | \|\| id:ebi-a-GCST90001672 | ebi-a-GCST90001672 | TRUE | C | 97260725 | 1.06E-22 | reported | 3408 | 0.08166 | rs142105763 |  |  |  |  |  |  |  |  |  |  |  |  |  |  |  |
| 0.6468 | 10 | igd | 0.0263 | C | \|\| id:ebi-a-GCST90001672 | ebi-a-GCST90001672 | TRUE | T | 96890004 | 3.71E-15 | reported | 3408 | 0.08187 | rs143132810 |  |  |  |  |  |  |  |  |  |  |  |  |  |  |  |
| -0.7475 | 19 | igd | 0.0263 | C | \|\| id:ebi-a-GCST90001957 | ebi-a-GCST90001957 | TRUE | T | 51750043 | 1.00E-10 | reported | 1578 | 0.1148 | rs145323276 |  |  |  |  |  |  |  |  |  |  |  |  |  |  |  |
| 0.4639 | 2 | igd | 0.0263 | A | \|\| id:ebi-a-GCST90002098 | ebi-a-GCST90002098 | TRUE | C | 38420441 | 5.80E-09 | reported | 2910 | 0.07943 | rs116006547 |  |  |  |  |  |  |  |  |  |  |  |  |  |  |  |
| -0.5881 | 19 | igd | 0.02625 | C | \|\| id:prot-a-439 | prot-a-439 | TRUE | A | 51737053 | 1.45E-14 | reported | 3301 | 0.0765 | rs113464261 |  |  |  |  |  |  |  |  |  |  |  |  |  |  |  |
| -0.4401 | 1 | igd | 0.0262 | A | \|\| id:ebi-a-GCST90001474 | ebi-a-GCST90001474 | TRUE | G | 161908877 | 1.07E-08 | reported | 3410 | 0.07676 | rs72708099 |  |  |  |  |  |  |  |  |  |  |  |  |  |  |  |
| 0.4594 | 10 | igd | 0.0262 | G | \|\| id:ebi-a-GCST90001496 | ebi-a-GCST90001496 | TRUE | T | 95924521 | 2.63E-08 | reported | 3437 | 0.08235 | rs147121076 |  |  |  |  |  |  |  |  |  |  |  |  |  |  |  |
| 0.4681 | 10 | igd | 0.0262 | G | \|\| id:ebi-a-GCST90001497 | ebi-a-GCST90001497 | TRUE | T | 95924521 | 9.29E-09 | reported | 3437 | 0.0813 | rs147121076 |  |  |  |  |  |  |  |  |  |  |  |  |  |  |  |
| 0.8135 | 10 | igd | 0.0262 | T | \|\| id:ebi-a-GCST90001670 | ebi-a-GCST90001670 | TRUE | C | 97260725 | 1.07E-23 | reported | 3440 | 0.08047 | rs142105763 |  |  |  |  |  |  |  |  |  |  |  |  |  |  |  |
| 0.801 | 10 | igd | 0.0262 | T | \|\| id:ebi-a-GCST90001671 | ebi-a-GCST90001671 | TRUE | C | 97260725 | 6.27E-23 | reported | 3440 | 0.08067 | rs142105763 |  |  |  |  |  |  |  |  |  |  |  |  |  |  |  |
| -0.4594 | 10 | igd | 0.0261 | T | \|\| id:ebi-a-GCST90001489 | ebi-a-GCST90001489 | TRUE | C | 99651421 | 5.93E-09 | reported | 3405 | 0.07875 | rs7916771 |  |  |  |  |  |  |  |  |  |  |  |  |  |  |  |
| -0.4614 | 10 | igd | 0.0261 | T | \|\| id:ebi-a-GCST90001660 | ebi-a-GCST90001660 | TRUE | C | 99651421 | 3.84E-09 | reported | 3408 | 0.07812 | rs7916771 |  |  |  |  |  |  |  |  |  |  |  |  |  |  |  |
| -0.7112 | 22 | igd | 0.0261 | A | \|\| id:ebi-a-GCST90001702 | ebi-a-GCST90001702 | TRUE | G | 40175781 | 1.83E-19 | reported | 3657 | 0.07837 | rs150612106 |  |  |  |  |  |  |  |  |  |  |  |  |  |  |  |
| -0.708 | 22 | igd | 0.0261 | A | \|\| id:ebi-a-GCST90001703 | ebi-a-GCST90001703 | TRUE | G | 40175781 | 2.62E-19 | reported | 3657 | 0.07837 | rs150612106 |  |  |  |  |  |  |  |  |  |  |  |  |  |  |  |
| -0.7379 | 22 | igd | 0.0261 | A | \|\| id:ebi-a-GCST90001704 | ebi-a-GCST90001704 | TRUE | G | 40175781 | 6.58E-21 | reported | 3657 | 0.07819 | rs150612106 |  |  |  |  |  |  |  |  |  |  |  |  |  |  |  |
| -0.7252 | 22 | igd | 0.0261 | A | \|\| id:ebi-a-GCST90001710 | ebi-a-GCST90001710 | TRUE | G | 40175781 | 2.74E-20 | reported | 3657 | 0.07811 | rs150612106 |  |  |  |  |  |  |  |  |  |  |  |  |  |  |  |
| -0.725 | 22 | igd | 0.0261 | A | \|\| id:ebi-a-GCST90001711 | ebi-a-GCST90001711 | TRUE | G | 40175781 | 2.29E-20 | reported | 3657 | 0.07793 | rs150612106 |  |  |  |  |  |  |  |  |  |  |  |  |  |  |  |
| 0.4633 | 10 | igd | 0.0258 | G | \|\| id:ebi-a-GCST90001660 | ebi-a-GCST90001660 | TRUE | T | 97580324 | 4.96E-08 | reported | 3408 | 0.08478 | rs180727168 |  |  |  |  |  |  |  |  |  |  |  |  |  |  |  |
| 0.4934 | 6 | igd | 0.0258 | A | \|\| id:ebi-a-GCST90002105 | ebi-a-GCST90002105 | TRUE | G | 30227206 | 2.16E-08 | reported | 2872 | 0.08788 | rs17188303 |  |  |  |  |  |  |  |  |  |  |  |  |  |  |  |
| -0.5263 | 3 | igd | 0.0258 | C | \|\| id:ebi-a-GCST90001997 | ebi-a-GCST90001997 | TRUE | T | 39603035 | 4.17E-11 | reported | 3590 | 0.07952 | rs144569262 |  |  |  |  |  |  |  |  |  |  |  |  |  |  |  |
| -0.4844 | 3 | igd | 0.0258 | C | \|\| id:ebi-a-GCST90001995 | ebi-a-GCST90001995 | TRUE | T | 39603035 | 9.80E-10 | reported | 3590 | 0.07904 | rs144569262 |  |  |  |  |  |  |  |  |  |  |  |  |  |  |  |
| 0.4658 | 10 | igd | 0.0257 | G | \|\| id:ebi-a-GCST90001483 | ebi-a-GCST90001483 | TRUE | T | 97580324 | 1.90E-08 | reported | 3405 | 0.08268 | rs180727168 |  |  |  |  |  |  |  |  |  |  |  |  |  |  |  |
| 0.5161 | 10 | igd | 0.0257 | G | \|\| id:ebi-a-GCST90001489 | ebi-a-GCST90001489 | TRUE | T | 97580324 | 1.32E-09 | reported | 3405 | 0.08487 | rs180727168 |  |  |  |  |  |  |  |  |  |  |  |  |  |  |  |
| 0.5467 | 10 | igd | 0.0257 | G | \|\| id:ebi-a-GCST90001495 | ebi-a-GCST90001495 | TRUE | T | 97580324 | 2.15E-10 | reported | 3405 | 0.08583 | rs180727168 |  |  |  |  |  |  |  |  |  |  |  |  |  |  |  |
| 0.5246 | 10 | igd | 0.0257 | C | \|\| id:ebi-a-GCST90001888 | ebi-a-GCST90001888 | TRUE | T | 97092931 | 9.70E-10 | reported | 2920 | 0.08552 | rs143640912 |  |  |  |  |  |  |  |  |  |  |  |  |  |  |  |
| 0.5973 | 10 | igd | 0.0257 | C | \|\| id:ebi-a-GCST90001892 | ebi-a-GCST90001892 | TRUE | T | 97092931 | 7.84E-12 | reported | 2920 | 0.08695 | rs143640912 |  |  |  |  |  |  |  |  |  |  |  |  |  |  |  |
| -0.833 | 10 | igd | 0.0257 | C | \|\| id:ebi-a-GCST90001961 | ebi-a-GCST90001961 | TRUE | T | 97092931 | 2.42E-21 | reported | 2920 | 0.08714 | rs143640912 |  |  |  |  |  |  |  |  |  |  |  |  |  |  |  |
| 0.7752 | 10 | igd | 0.0257 | G | \|\| id:ebi-a-GCST90002030 | ebi-a-GCST90002030 | TRUE | C | 97188433 | 1.16E-20 | reported | 2920 | 0.08255 | rs111455423 |  |  |  |  |  |  |  |  |  |  |  |  |  |  |  |
| 0.6765 | 10 | igd | 0.0257 | G | \|\| id:ebi-a-GCST90002031 | ebi-a-GCST90002031 | TRUE | C | 97188433 | 3.22E-15 | reported | 2920 | 0.08537 | rs111455423 |  |  |  |  |  |  |  |  |  |  |  |  |  |  |  |
| 0.7281 | 10 | igd | 0.0257 | G | \|\| id:ebi-a-GCST90002032 | ebi-a-GCST90002032 | TRUE | C | 97188433 | 1.86E-17 | reported | 2920 | 0.0851 | rs111455423 |  |  |  |  |  |  |  |  |  |  |  |  |  |  |  |
| 0.8406 | 10 | igd | 0.0257 | C | \|\| id:ebi-a-GCST90002061 | ebi-a-GCST90002061 | TRUE | T | 97092931 | 1.86E-21 | reported | 2920 | 0.08768 | rs143640912 |  |  |  |  |  |  |  |  |  |  |  |  |  |  |  |
| 0.4775 | 10 | igd | 0.0256 | G | \|\| id:ebi-a-GCST90001484 | ebi-a-GCST90001484 | TRUE | T | 97580324 | 6.23E-09 | reported | 3437 | 0.08198 | rs180727168 |  |  |  |  |  |  |  |  |  |  |  |  |  |  |  |
| 0.4898 | 10 | igd | 0.0256 | G | \|\| id:ebi-a-GCST90001485 | ebi-a-GCST90001485 | TRUE | T | 97580324 | 2.39E-09 | reported | 3437 | 0.08184 | rs180727168 |  |  |  |  |  |  |  |  |  |  |  |  |  |  |  |
| 0.5364 | 10 | igd | 0.0256 | G | \|\| id:ebi-a-GCST90001491 | ebi-a-GCST90001491 | TRUE | T | 97580324 | 1.79E-10 | reported | 3437 | 0.08385 | rs180727168 |  |  |  |  |  |  |  |  |  |  |  |  |  |  |  |
| 0.5534 | 10 | igd | 0.0256 | G | \|\| id:ebi-a-GCST90001496 | ebi-a-GCST90001496 | TRUE | T | 97580324 | 8.40E-11 | reported | 3437 | 0.08496 | rs180727168 |  |  |  |  |  |  |  |  |  |  |  |  |  |  |  |
| 0.5621 | 10 | igd | 0.0256 | G | \|\| id:ebi-a-GCST90001497 | ebi-a-GCST90001497 | TRUE | T | 97580324 | 2.38E-11 | reported | 3437 | 0.08386 | rs180727168 |  |  |  |  |  |  |  |  |  |  |  |  |  |  |  |
| 0.5858 | 10 | igd | 0.0254 | T | \|\| id:ebi-a-GCST90001489 | ebi-a-GCST90001489 | TRUE | G | 97730285 | 4.25E-13 | reported | 3405 | 0.08052 | rs77856122 |  |  |  |  |  |  |  |  |  |  |  |  |  |  |  |
| 0.6232 | 10 | igd | 0.0254 | T | \|\| id:ebi-a-GCST90001495 | ebi-a-GCST90001495 | TRUE | G | 97730285 | 1.52E-14 | reported | 3405 | 0.08073 | rs77856122 |  |  |  |  |  |  |  |  |  |  |  |  |  |  |  |
| 0.6102 | 10 | igd | 0.0254 | T | \|\| id:ebi-a-GCST90001660 | ebi-a-GCST90001660 | TRUE | G | 97730285 | 2.35E-14 | reported | 3408 | 0.07962 | rs77856122 |  |  |  |  |  |  |  |  |  |  |  |  |  |  |  |
| 0.569 | 10 | igd | 0.0254 | T | \|\| id:ebi-a-GCST90001672 | ebi-a-GCST90001672 | TRUE | G | 97730285 | 1.91E-12 | reported | 3408 | 0.08051 | rs77856122 |  |  |  |  |  |  |  |  |  |  |  |  |  |  |  |
| -0.5353 | 6 | igd | 0.0253 | C | \|\| id:ebi-a-GCST90001752 | ebi-a-GCST90001752 | TRUE | T | 32611122 | 1.79E-09 | reported | 3657 | 0.08875 | rs62404112 |  |  |  |  |  |  |  |  |  |  |  |  |  |  |  |
| 0.4706 | 10 | igd | 0.0252 | C | \|\| id:ebi-a-GCST90001484 | ebi-a-GCST90001484 | TRUE | G | 95602445 | 8.04E-10 | reported | 3437 | 0.07638 | rs80320234 |  |  |  |  |  |  |  |  |  |  |  |  |  |  |  |
| 0.5734 | 10 | igd | 0.0252 | T | \|\| id:ebi-a-GCST90001491 | ebi-a-GCST90001491 | TRUE | G | 97730285 | 5.28E-13 | reported | 3437 | 0.07913 | rs77856122 |  |  |  |  |  |  |  |  |  |  |  |  |  |  |  |
| -0.4164 | 1 | igd | 0.0252 | C | \|\| id:ebi-a-GCST90001669 | ebi-a-GCST90001669 | TRUE | T | 198636596 | 1.23E-08 | reported | 3408 | 0.07294 | rs79641904 |  |  |  |  |  |  |  |  |  |  |  |  |  |  |  |
| -0.5752 | 1 | igd | 0.0252 | C | \|\| id:ebi-a-GCST90001693 | ebi-a-GCST90001693 | TRUE | T | 198636596 | 2.86E-12 | reported | 3408 | 0.08205 | rs79641904 |  |  |  |  |  |  |  |  |  |  |  |  |  |  |  |
| -0.6484 | 10 | igd | 0.0252 | T | \|\| id:ebi-a-GCST90002030 | ebi-a-GCST90002030 | TRUE | A | 95790290 | 2.34E-14 | reported | 2920 | 0.08454 | rs182078825 |  |  |  |  |  |  |  |  |  |  |  |  |  |  |  |
| -0.5649 | 10 | igd | 0.0252 | T | \|\| id:ebi-a-GCST90002031 | ebi-a-GCST90002031 | TRUE | A | 95790290 | 1.02E-10 | reported | 2920 | 0.08706 | rs182078825 |  |  |  |  |  |  |  |  |  |  |  |  |  |  |  |
| -0.6567 | 10 | igd | 0.0252 | T | \|\| id:ebi-a-GCST90002032 | ebi-a-GCST90002032 | TRUE | A | 95790290 | 4.98E-14 | reported | 2920 | 0.08675 | rs182078825 |  |  |  |  |  |  |  |  |  |  |  |  |  |  |  |
| -0.4861 | 6 | igd | 0.0252 | C | \|\| id:ebi-a-GCST90002105 | ebi-a-GCST90002105 | TRUE | A | 25256220 | 9.98E-09 | reported | 2872 | 0.08457 | rs74334317 |  |  |  |  |  |  |  |  |  |  |  |  |  |  |  |
| 0.5762 | 10 | igd | 0.0251 | T | \|\| id:ebi-a-GCST90001658 | ebi-a-GCST90001658 | TRUE | G | 97730285 | 5.17E-14 | reported | 3440 | 0.07623 | rs77856122 |  |  |  |  |  |  |  |  |  |  |  |  |  |  |  |
| 0.4176 | 10 | igd | 0.0251 | C | \|\| id:ebi-a-GCST90001659 | ebi-a-GCST90001659 | TRUE | G | 95602445 | 2.82E-08 | reported | 3440 | 0.07504 | rs80320234 |  |  |  |  |  |  |  |  |  |  |  |  |  |  |  |
| 0.5065 | 1 | igd | 0.0251 | C | \|\| id:ebi-a-GCST90001664 | ebi-a-GCST90001664 | TRUE | T | 198636596 | 1.83E-11 | reported | 3440 | 0.07513 | rs79641904 |  |  |  |  |  |  |  |  |  |  |  |  |  |  |  |
| 0.5496 | 10 | igd | 0.0251 | T | \|\| id:ebi-a-GCST90001670 | ebi-a-GCST90001670 | TRUE | G | 97730285 | 6.11E-12 | reported | 3440 | 0.07963 | rs77856122 |  |  |  |  |  |  |  |  |  |  |  |  |  |  |  |
| 0.577 | 10 | igd | 0.0251 | T | \|\| id:ebi-a-GCST90001671 | ebi-a-GCST90001671 | TRUE | G | 97730285 | 4.87E-13 | reported | 3440 | 0.0795 | rs77856122 |  |  |  |  |  |  |  |  |  |  |  |  |  |  |  |
| 0.8492 | 10 | igd | 0.0251 | C | \|\| id:ebi-a-GCST90001672 | ebi-a-GCST90001672 | TRUE | T | 97092931 | 1.63E-24 | reported | 3408 | 0.08246 | rs143640912 |  |  |  |  |  |  |  |  |  |  |  |  |  |  |  |
| -0.5286 | 1 | igd | 0.0251 | C | \|\| id:ebi-a-GCST90001691 | ebi-a-GCST90001691 | TRUE | T | 198636596 | 1.11E-10 | reported | 3440 | 0.08169 | rs79641904 |  |  |  |  |  |  |  |  |  |  |  |  |  |  |  |
| -0.4722 | 1 | igd | 0.0251 | C | \|\| id:ebi-a-GCST90001692 | ebi-a-GCST90001692 | TRUE | T | 198636596 | 5.64E-09 | reported | 3440 | 0.08083 | rs79641904 |  |  |  |  |  |  |  |  |  |  |  |  |  |  |  |
| 76.42 | 1 | igd | 0.0251 | C | \|\| id:ebi-a-GCST90001698 | ebi-a-GCST90001698 | TRUE | T | 81455274 | 1.55E-11 | reported | 3408 | 11.29 | rs12130486 |  |  |  |  |  |  |  |  |  |  |  |  |  |  |  |
| 0.8754 | 10 | igd | 0.025 | C | \|\| id:ebi-a-GCST90001670 | ebi-a-GCST90001670 | TRUE | T | 97092931 | 1.24E-26 | reported | 3440 | 0.08127 | rs143640912 |  |  |  |  |  |  |  |  |  |  |  |  |  |  |  |
| 0.8403 | 10 | igd | 0.025 | C | \|\| id:ebi-a-GCST90001671 | ebi-a-GCST90001671 | TRUE | T | 97092931 | 1.42E-24 | reported | 3440 | 0.0815 | rs143640912 |  |  |  |  |  |  |  |  |  |  |  |  |  |  |  |
| 0.5014 | 10 | igd | 0.025 | A | \|\| id:ebi-a-GCST90001888 | ebi-a-GCST90001888 | TRUE | G | 96597621 | 4.94E-09 | reported | 2920 | 0.08547 | rs117957417 |  |  |  |  |  |  |  |  |  |  |  |  |  |  |  |
| 0.6297 | 10 | igd | 0.025 | A | \|\| id:ebi-a-GCST90001892 | ebi-a-GCST90001892 | TRUE | G | 96597621 | 5.71E-13 | reported | 2920 | 0.08698 | rs117957417 |  |  |  |  |  |  |  |  |  |  |  |  |  |  |  |
| -0.8229 | 10 | igd | 0.025 | A | \|\| id:ebi-a-GCST90001961 | ebi-a-GCST90001961 | TRUE | G | 96597621 | 1.00E-20 | reported | 2920 | 0.08748 | rs117957417 |  |  |  |  |  |  |  |  |  |  |  |  |  |  |  |
| 0.8168 | 10 | igd | 0.025 | T | \|\| id:ebi-a-GCST90002030 | ebi-a-GCST90002030 | TRUE | G | 97730285 | 7.17E-22 | reported | 2920 | 0.0843 | rs77856122 |  |  |  |  |  |  |  |  |  |  |  |  |  |  |  |
| 0.7564 | 10 | igd | 0.025 | T | \|\| id:ebi-a-GCST90002031 | ebi-a-GCST90002031 | TRUE | G | 97730285 | 7.59E-18 | reported | 2920 | 0.08733 | rs77856122 |  |  |  |  |  |  |  |  |  |  |  |  |  |  |  |
| 0.7407 | 10 | igd | 0.025 | T | \|\| id:ebi-a-GCST90002032 | ebi-a-GCST90002032 | TRUE | G | 97730285 | 3.16E-17 | reported | 2920 | 0.08722 | rs77856122 |  |  |  |  |  |  |  |  |  |  |  |  |  |  |  |
| 0.794 | 10 | igd | 0.025 | A | \|\| id:ebi-a-GCST90002061 | ebi-a-GCST90002061 | TRUE | G | 96597621 | 2.99E-19 | reported | 2920 | 0.08791 | rs117957417 |  |  |  |  |  |  |  |  |  |  |  |  |  |  |  |
| -0.4599 | 10 | igd | 0.0249 | T | \|\| id:ebi-a-GCST90001497 | ebi-a-GCST90001497 | TRUE | A | 95790290 | 9.40E-09 | reported | 3437 | 0.07991 | rs182078825 |  |  |  |  |  |  |  |  |  |  |  |  |  |  |  |
| 0.9178 | 10 | igd | 0.0249 | A | \|\| id:ebi-a-GCST90001670 | ebi-a-GCST90001670 | TRUE | G | 96597621 | 9.51E-30 | reported | 3440 | 0.08026 | rs117957417 |  |  |  |  |  |  |  |  |  |  |  |  |  |  |  |
| 0.8684 | 10 | igd | 0.0249 | A | \|\| id:ebi-a-GCST90001671 | ebi-a-GCST90001671 | TRUE | G | 96597621 | 1.24E-26 | reported | 3440 | 0.08061 | rs117957417 |  |  |  |  |  |  |  |  |  |  |  |  |  |  |  |
| 0.9199 | 10 | igd | 0.0248 | A | \|\| id:ebi-a-GCST90001672 | ebi-a-GCST90001672 | TRUE | G | 96597621 | 6.79E-29 | reported | 3408 | 0.08171 | rs117957417 |  |  |  |  |  |  |  |  |  |  |  |  |  |  |  |
| -0.8825 | 19 | igd | 0.0248 | C | \|\| id:ebi-a-GCST90001946 | ebi-a-GCST90001946 | TRUE | A | 51737053 | 4.93E-15 | reported | 1634 | 0.1117 | rs113464261 |  |  |  |  |  |  |  |  |  |  |  |  |  |  |  |
| -0.8613 | 19 | igd | 0.0248 | C | \|\| id:ebi-a-GCST90001948 | ebi-a-GCST90001948 | TRUE | A | 51737053 | 1.42E-14 | reported | 1633 | 0.1109 | rs113464261 |  |  |  |  |  |  |  |  |  |  |  |  |  |  |  |
| -0.8145 | 19 | igd | 0.0248 | C | \|\| id:ebi-a-GCST90001949 | ebi-a-GCST90001949 | TRUE | A | 51737053 | 3.26E-13 | reported | 1634 | 0.1109 | rs113464261 |  |  |  |  |  |  |  |  |  |  |  |  |  |  |  |
| -0.5219 | 10 | igd | 0.0247 | C | \|\| id:ebi-a-GCST90001489 | ebi-a-GCST90001489 | TRUE | T | 97553483 | 3.67E-09 | reported | 3405 | 0.08825 | rs147773939 |  |  |  |  |  |  |  |  |  |  |  |  |  |  |  |
| -0.5855 | 10 | igd | 0.0247 | C | \|\| id:ebi-a-GCST90001495 | ebi-a-GCST90001495 | TRUE | T | 97553483 | 5.93E-11 | reported | 3405 | 0.08916 | rs147773939 |  |  |  |  |  |  |  |  |  |  |  |  |  |  |  |
| -0.5275 | 10 | igd | 0.0246 | C | \|\| id:ebi-a-GCST90001491 | ebi-a-GCST90001491 | TRUE | T | 97553483 | 1.56E-09 | reported | 3437 | 0.08711 | rs147773939 |  |  |  |  |  |  |  |  |  |  |  |  |  |  |  |
| -0.5778 | 10 | igd | 0.0246 | C | \|\| id:ebi-a-GCST90001497 | ebi-a-GCST90001497 | TRUE | T | 97553483 | 3.76E-11 | reported | 3437 | 0.08708 | rs147773939 |  |  |  |  |  |  |  |  |  |  |  |  |  |  |  |
| -0.6429 | 10 | igd | 0.0246 | C | \|\| id:ebi-a-GCST90001658 | ebi-a-GCST90001658 | TRUE | T | 97553483 | 2.52E-14 | reported | 3440 | 0.084 | rs147773939 |  |  |  |  |  |  |  |  |  |  |  |  |  |  |  |
| -0.6815 | 22 | igd | 0.0246 | T | \|\| id:ebi-a-GCST90001702 | ebi-a-GCST90001702 | TRUE | C | 39780097 | 1.55E-17 | reported | 3657 | 0.07956 | rs55706518 |  |  |  |  |  |  |  |  |  |  |  |  |  |  |  |
| -0.6932 | 22 | igd | 0.0246 | T | \|\| id:ebi-a-GCST90001703 | ebi-a-GCST90001703 | TRUE | C | 39780097 | 4.28E-18 | reported | 3657 | 0.07953 | rs55706518 |  |  |  |  |  |  |  |  |  |  |  |  |  |  |  |
| -0.7138 | 22 | igd | 0.0246 | T | \|\| id:ebi-a-GCST90001704 | ebi-a-GCST90001704 | TRUE | C | 39780097 | 3.82E-19 | reported | 3657 | 0.07938 | rs55706518 |  |  |  |  |  |  |  |  |  |  |  |  |  |  |  |
| -0.647 | 22 | igd | 0.0246 | T | \|\| id:ebi-a-GCST90001710 | ebi-a-GCST90001710 | TRUE | C | 39780097 | 4.99E-16 | reported | 3657 | 0.07939 | rs55706518 |  |  |  |  |  |  |  |  |  |  |  |  |  |  |  |
| -0.6741 | 22 | igd | 0.0246 | T | \|\| id:ebi-a-GCST90001711 | ebi-a-GCST90001711 | TRUE | C | 39780097 | 2.42E-17 | reported | 3657 | 0.07918 | rs55706518 |  |  |  |  |  |  |  |  |  |  |  |  |  |  |  |
| 0.4571 | 1 | igd | 0.0246 | A | \|\| id:ebi-a-GCST90001725 | ebi-a-GCST90001725 | TRUE | G | 206842241 | 7.38E-09 | reported | 3656 | 0.07887 | rs75320486 |  |  |  |  |  |  |  |  |  |  |  |  |  |  |  |
| 0.5238 | 1 | igd | 0.0246 | A | \|\| id:ebi-a-GCST90001729 | ebi-a-GCST90001729 | TRUE | G | 206842241 | 6.75E-11 | reported | 3657 | 0.08002 | rs75320486 |  |  |  |  |  |  |  |  |  |  |  |  |  |  |  |
| 0.4745 | 1 | igd | 0.0246 | A | \|\| id:ebi-a-GCST90001730 | ebi-a-GCST90001730 | TRUE | G | 206842241 | 2.31E-09 | reported | 3657 | 0.07922 | rs75320486 |  |  |  |  |  |  |  |  |  |  |  |  |  |  |  |
| 0.4393 | 1 | igd | 0.0246 | A | \|\| id:ebi-a-GCST90001737 | ebi-a-GCST90001737 | TRUE | G | 206842241 | 2.72E-08 | reported | 3653 | 0.07886 | rs75320486 |  |  |  |  |  |  |  |  |  |  |  |  |  |  |  |
| -0.4434 | 6 | igd | 0.0246 | G | \|\| id:ebi-a-GCST90001766 | ebi-a-GCST90001766 | TRUE | A | 107196452 | 3.37E-08 | reported | 3638 | 0.08013 | rs9486458 |  |  |  |  |  |  |  |  |  |  |  |  |  |  |  |
| 0.6089 | 2 | igd | 0.0246 | T | \|\| id:ebi-a-GCST90002098 | ebi-a-GCST90002098 | TRUE | C | 39864242 | 6.32E-13 | reported | 2910 | 0.08426 | rs147692482 |  |  |  |  |  |  |  |  |  |  |  |  |  |  |  |
| 0.6282 | 10 | igd | 0.0245 | T | \|\| id:ebi-a-GCST90001660 | ebi-a-GCST90001660 | TRUE | C | 97441169 | 3.70E-14 | reported | 3408 | 0.08262 | rs74805311 |  |  |  |  |  |  |  |  |  |  |  |  |  |  |  |
| 0.4684 | 10 | igd | 0.0245 | T | \|\| id:ebi-a-GCST90001672 | ebi-a-GCST90001672 | TRUE | C | 97441169 | 2.40E-08 | reported | 3408 | 0.08375 | rs74805311 |  |  |  |  |  |  |  |  |  |  |  |  |  |  |  |
| -0.7212 | 6 | igd | 0.0245 | G | \|\| id:ebi-a-GCST90001765 | ebi-a-GCST90001765 | TRUE | A | 107196452 | 5.58E-20 | reported | 3655 | 0.07834 | rs9486458 |  |  |  |  |  |  |  |  |  |  |  |  |  |  |  |
| -0.6239 | 6 | igd | 0.0245 | G | \|\| id:ebi-a-GCST90001769 | ebi-a-GCST90001769 | TRUE | A | 107196452 | 7.07E-15 | reported | 3648 | 0.07982 | rs9486458 |  |  |  |  |  |  |  |  |  |  |  |  |  |  |  |
| -0.7118 | 6 | igd | 0.0245 | G | \|\| id:ebi-a-GCST90001771 | ebi-a-GCST90001771 | TRUE | A | 107196452 | 2.04E-19 | reported | 3653 | 0.07855 | rs9486458 |  |  |  |  |  |  |  |  |  |  |  |  |  |  |  |
| -0.6126 | 6 | igd | 0.0245 | G | \|\| id:ebi-a-GCST90001772 | ebi-a-GCST90001772 | TRUE | A | 107196452 | 1.14E-14 | reported | 3655 | 0.07899 | rs9486458 |  |  |  |  |  |  |  |  |  |  |  |  |  |  |  |
| 0.5213 | 6 | igd | 0.0245 | T | \|\| id:ebi-a-GCST90002105 | ebi-a-GCST90002105 | TRUE | G | 30900435 | 1.15E-08 | reported | 2872 | 0.09109 | rs12525616 |  |  |  |  |  |  |  |  |  |  |  |  |  |  |  |
| 0.6033 | 10 | igd | 0.0244 | T | \|\| id:ebi-a-GCST90001489 | ebi-a-GCST90001489 | TRUE | C | 97441169 | 5.64E-13 | reported | 3405 | 0.08336 | rs74805311 |  |  |  |  |  |  |  |  |  |  |  |  |  |  |  |
| 0.5941 | 10 | igd | 0.0244 | T | \|\| id:ebi-a-GCST90001495 | ebi-a-GCST90001495 | TRUE | C | 97441169 | 1.66E-12 | reported | 3405 | 0.08384 | rs74805311 |  |  |  |  |  |  |  |  |  |  |  |  |  |  |  |
| 0.5996 | 10 | igd | 0.0243 | T | \|\| id:ebi-a-GCST90001658 | ebi-a-GCST90001658 | TRUE | C | 97441169 | 4.35E-14 | reported | 3440 | 0.07907 | rs74805311 |  |  |  |  |  |  |  |  |  |  |  |  |  |  |  |
| 0.5949 | 10 | igd | 0.0243 | T | \|\| id:ebi-a-GCST90001659 | ebi-a-GCST90001659 | TRUE | C | 97441169 | 6.88E-14 | reported | 3440 | 0.0791 | rs74805311 |  |  |  |  |  |  |  |  |  |  |  |  |  |  |  |
| 0.4627 | 10 | igd | 0.0243 | T | \|\| id:ebi-a-GCST90001671 | ebi-a-GCST90001671 | TRUE | C | 97441169 | 2.42E-08 | reported | 3440 | 0.08273 | rs74805311 |  |  |  |  |  |  |  |  |  |  |  |  |  |  |  |
| -0.5493 | 12 | igd | 0.0243 | T | \|\| id:ebi-a-GCST90002074 | ebi-a-GCST90002074 | TRUE | C | 68819783 | 1.66E-10 | reported | 3112 | 0.08569 | rs75615128 |  |  |  |  |  |  |  |  |  |  |  |  |  |  |  |
| 0.5548 | 6 | igd | 0.0242 | A | \|\| id:ebi-a-GCST90002105 | ebi-a-GCST90002105 | TRUE | C | 33021130 | 5.69E-10 | reported | 2872 | 0.08919 | rs113119046 |  |  |  |  |  |  |  |  |  |  |  |  |  |  |  |
| 0.4543 | 10 | igd | 0.0241 | T | \|\| id:ebi-a-GCST90001484 | ebi-a-GCST90001484 | TRUE | C | 97441169 | 2.38E-08 | reported | 3437 | 0.0812 | rs74805311 |  |  |  |  |  |  |  |  |  |  |  |  |  |  |  |
| 0.5926 | 10 | igd | 0.0241 | T | \|\| id:ebi-a-GCST90001491 | ebi-a-GCST90001491 | TRUE | C | 97441169 | 6.39E-13 | reported | 3437 | 0.08209 | rs74805311 |  |  |  |  |  |  |  |  |  |  |  |  |  |  |  |
| 0.5719 | 10 | igd | 0.0241 | T | \|\| id:ebi-a-GCST90001496 | ebi-a-GCST90001496 | TRUE | C | 97441169 | 7.61E-12 | reported | 3437 | 0.08325 | rs74805311 |  |  |  |  |  |  |  |  |  |  |  |  |  |  |  |
| 0.6038 | 10 | igd | 0.0241 | T | \|\| id:ebi-a-GCST90001497 | ebi-a-GCST90001497 | TRUE | C | 97441169 | 2.41E-13 | reported | 3437 | 0.08212 | rs74805311 |  |  |  |  |  |  |  |  |  |  |  |  |  |  |  |
| -0.4752 | 22 | igd | 0.0241 | A | \|\| id:ebi-a-GCST90001701 | ebi-a-GCST90001701 | TRUE | G | 41986143 | 2.84E-09 | reported | 3592 | 0.07979 | rs138378981 |  |  |  |  |  |  |  |  |  |  |  |  |  |  |  |
| -1.34 | 22 | igd | 0.0241 | A | \|\| id:ebi-a-GCST90001702 | ebi-a-GCST90001702 | TRUE | G | 41986143 | 3.76E-64 | reported | 3657 | 0.07769 | rs138378981 |  |  |  |  |  |  |  |  |  |  |  |  |  |  |  |
| -0.5423 | 22 | igd | 0.0241 | G | \|\| id:ebi-a-GCST90001702 | ebi-a-GCST90001702 | TRUE | C | 43641046 | 2.36E-11 | reported | 3657 | 0.0809 | rs143332213 |  |  |  |  |  |  |  |  |  |  |  |  |  |  |  |
| -0.5179 | 22 | igd | 0.0241 | G | \|\| id:ebi-a-GCST90001703 | ebi-a-GCST90001703 | TRUE | C | 43641046 | 1.77E-10 | reported | 3657 | 0.08095 | rs143332213 |  |  |  |  |  |  |  |  |  |  |  |  |  |  |  |
| -1.339 | 22 | igd | 0.0241 | A | \|\| id:ebi-a-GCST90001703 | ebi-a-GCST90001703 | TRUE | G | 41986143 | 4.76E-64 | reported | 3657 | 0.07769 | rs138378981 |  |  |  |  |  |  |  |  |  |  |  |  |  |  |  |
| -1.356 | 22 | igd | 0.0241 | A | \|\| id:ebi-a-GCST90001704 | ebi-a-GCST90001704 | TRUE | G | 41986143 | 6.44E-66 | reported | 3657 | 0.07751 | rs138378981 |  |  |  |  |  |  |  |  |  |  |  |  |  |  |  |
| -0.5316 | 22 | igd | 0.0241 | G | \|\| id:ebi-a-GCST90001710 | ebi-a-GCST90001710 | TRUE | C | 43641046 | 5.00E-11 | reported | 3657 | 0.08066 | rs143332213 |  |  |  |  |  |  |  |  |  |  |  |  |  |  |  |
| -1.274 | 22 | igd | 0.0241 | A | \|\| id:ebi-a-GCST90001710 | ebi-a-GCST90001710 | TRUE | G | 41986143 | 2.74E-58 | reported | 3657 | 0.07775 | rs138378981 |  |  |  |  |  |  |  |  |  |  |  |  |  |  |  |
| -0.5071 | 22 | igd | 0.0241 | G | \|\| id:ebi-a-GCST90001711 | ebi-a-GCST90001711 | TRUE | C | 43641046 | 3.49E-10 | reported | 3657 | 0.08058 | rs143332213 |  |  |  |  |  |  |  |  |  |  |  |  |  |  |  |
| -1.267 | 22 | igd | 0.0241 | A | \|\| id:ebi-a-GCST90001711 | ebi-a-GCST90001711 | TRUE | G | 41986143 | 6.82E-58 | reported | 3657 | 0.07761 | rs138378981 |  |  |  |  |  |  |  |  |  |  |  |  |  |  |  |
| 0.498 | 10 | igd | 0.0241 | C | \|\| id:ebi-a-GCST90002030 | ebi-a-GCST90002030 | TRUE | T | 92843848 | 4.25E-08 | reported | 2920 | 0.09063 | rs79595377 |  |  |  |  |  |  |  |  |  |  |  |  |  |  |  |
| 0.5341 | 6 | igd | 0.024 | A | \|\| id:ebi-a-GCST90002118 | ebi-a-GCST90002118 | TRUE | G | 30584133 | 5.65E-09 | reported | 2920 | 0.09139 | rs55777621 |  |  |  |  |  |  |  |  |  |  |  |  |  |  |  |
| -0.4501 | 1 | igd | 0.0239 | A | \|\| id:ebi-a-GCST90001798 | ebi-a-GCST90001798 | TRUE | G | 31211894 | 1.45E-08 | reported | 3657 | 0.07925 | rs34101571 |  |  |  |  |  |  |  |  |  |  |  |  |  |  |  |
| -0.534 | 1 | igd | 0.0239 | A | \|\| id:ebi-a-GCST90001804 | ebi-a-GCST90001804 | TRUE | G | 31211894 | 1.38E-11 | reported | 3657 | 0.07874 | rs34101571 |  |  |  |  |  |  |  |  |  |  |  |  |  |  |  |
| -0.5359 | 1 | igd | 0.0239 | A | \|\| id:ebi-a-GCST90001808 | ebi-a-GCST90001808 | TRUE | G | 31211894 | 2.10E-11 | reported | 3657 | 0.07974 | rs34101571 |  |  |  |  |  |  |  |  |  |  |  |  |  |  |  |
| -0.5823 | 3 | igd | 0.0239 | T | \|\| id:ebi-a-GCST90002015 | ebi-a-GCST90002015 | TRUE | C | 46301608 | 1.10E-10 | reported | 2871 | 0.08991 | rs140597534 |  |  |  |  |  |  |  |  |  |  |  |  |  |  |  |
| -0.5846 | 3 | igd | 0.0239 | T | \|\| id:ebi-a-GCST90002016 | ebi-a-GCST90002016 | TRUE | C | 46301608 | 8.63E-11 | reported | 2871 | 0.08975 | rs140597534 |  |  |  |  |  |  |  |  |  |  |  |  |  |  |  |
| -0.8766 | 12 | igd | 0.0239 | C | \|\| id:ebi-a-GCST90002073 | ebi-a-GCST90002073 | TRUE | G | 70360198 | 3.80E-23 | reported | 2850 | 0.08769 | rs148146302 |  |  |  |  |  |  |  |  |  |  |  |  |  |  |  |
| -0.5017 | 22 | igd | 0.0238 | A | \|\| id:ebi-a-GCST90001701 | ebi-a-GCST90001701 | TRUE | G | 42139665 | 5.70E-10 | reported | 3592 | 0.08072 | rs76198486 |  |  |  |  |  |  |  |  |  |  |  |  |  |  |  |
| -1.368 | 22 | igd | 0.0238 | A | \|\| id:ebi-a-GCST90001702 | ebi-a-GCST90001702 | TRUE | G | 42139665 | 2.10E-65 | reported | 3657 | 0.0785 | rs76198486 |  |  |  |  |  |  |  |  |  |  |  |  |  |  |  |
| -1.364 | 22 | igd | 0.0238 | A | \|\| id:ebi-a-GCST90001703 | ebi-a-GCST90001703 | TRUE | G | 42139665 | 5.33E-65 | reported | 3657 | 0.07852 | rs76198486 |  |  |  |  |  |  |  |  |  |  |  |  |  |  |  |
| -1.396 | 22 | igd | 0.0238 | A | \|\| id:ebi-a-GCST90001704 | ebi-a-GCST90001704 | TRUE | G | 42139665 | 2.85E-68 | reported | 3657 | 0.07826 | rs76198486 |  |  |  |  |  |  |  |  |  |  |  |  |  |  |  |
| -1.303 | 22 | igd | 0.0238 | A | \|\| id:ebi-a-GCST90001710 | ebi-a-GCST90001710 | TRUE | G | 42139665 | 1.24E-59 | reported | 3657 | 0.07855 | rs76198486 |  |  |  |  |  |  |  |  |  |  |  |  |  |  |  |
| -1.29 | 22 | igd | 0.0238 | A | \|\| id:ebi-a-GCST90001711 | ebi-a-GCST90001711 | TRUE | G | 42139665 | 1.10E-58 | reported | 3657 | 0.07845 | rs76198486 |  |  |  |  |  |  |  |  |  |  |  |  |  |  |  |
| -0.5703 | 10 | igd | 0.0238 | G | \|\| id:ebi-a-GCST90002030 | ebi-a-GCST90002030 | TRUE | A | 98337197 | 6.21E-11 | reported | 2920 | 0.08689 | rs75395894 |  |  |  |  |  |  |  |  |  |  |  |  |  |  |  |
| -0.5314 | 10 | igd | 0.0238 | G | \|\| id:ebi-a-GCST90002031 | ebi-a-GCST90002031 | TRUE | A | 98337197 | 3.90E-09 | reported | 2920 | 0.08998 | rs75395894 |  |  |  |  |  |  |  |  |  |  |  |  |  |  |  |
| -0.5475 | 10 | igd | 0.0238 | G | \|\| id:ebi-a-GCST90002032 | ebi-a-GCST90002032 | TRUE | A | 98337197 | 1.21E-09 | reported | 2920 | 0.08977 | rs75395894 |  |  |  |  |  |  |  |  |  |  |  |  |  |  |  |
| -0.4738 | 22 | igd | 0.0237 | T | \|\| id:ebi-a-GCST90001704 | ebi-a-GCST90001704 | TRUE | G | 38840146 | 1.01E-08 | reported | 3657 | 0.08251 | rs139592873 |  |  |  |  |  |  |  |  |  |  |  |  |  |  |  |
| -0.4513 | 6 | igd | 0.0237 | T | \|\| id:ebi-a-GCST90001774 | ebi-a-GCST90001774 | TRUE | G | 107188250 | 3.04E-08 | reported | 3656 | 0.0813 | rs732290 |  |  |  |  |  |  |  |  |  |  |  |  |  |  |  |
| 0.5534 | 2 | igd | 0.0237 | G | \|\| id:ebi-a-GCST90002098 | ebi-a-GCST90002098 | TRUE | A | 38052601 | 5.24E-11 | reported | 2910 | 0.08398 | rs80253251 |  |  |  |  |  |  |  |  |  |  |  |  |  |  |  |
| 0.7845 | 10 | igd | 0.0236 | T | \|\| id:ebi-a-GCST90002030 | ebi-a-GCST90002030 | TRUE | C | 97441169 | 1.04E-18 | reported | 2920 | 0.08825 | rs74805311 |  |  |  |  |  |  |  |  |  |  |  |  |  |  |  |
| 0.6528 | 10 | igd | 0.0236 | T | \|\| id:ebi-a-GCST90002031 | ebi-a-GCST90002031 | TRUE | C | 97441169 | 1.01E-12 | reported | 2920 | 0.09116 | rs74805311 |  |  |  |  |  |  |  |  |  |  |  |  |  |  |  |
| 0.7142 | 10 | igd | 0.0236 | T | \|\| id:ebi-a-GCST90002032 | ebi-a-GCST90002032 | TRUE | C | 97441169 | 5.44E-15 | reported | 2920 | 0.09089 | rs74805311 |  |  |  |  |  |  |  |  |  |  |  |  |  |  |  |
| 0.4573 | 5 | igd | 0.0236 | T | \|\| id:ebi-a-GCST90002003 | ebi-a-GCST90002003 | TRUE | G | 159009803 | 8.02E-09 | reported | 3629 | 0.07909 | rs77465361 |  |  |  |  |  |  |  |  |  |  |  |  |  |  |  |
| -0.6147 | 10 | igd | 0.0235 | C | \|\| id:ebi-a-GCST90002030 | ebi-a-GCST90002030 | TRUE | T | 97553483 | 9.49E-11 | reported | 2920 | 0.09458 | rs147773939 |  |  |  |  |  |  |  |  |  |  |  |  |  |  |  |
| -0.5778 | 22 | igd | 0.0234 | T | \|\| id:ebi-a-GCST90001702 | ebi-a-GCST90001702 | TRUE | C | 38823838 | 1.63E-12 | reported | 3657 | 0.08152 | rs79928737 |  |  |  |  |  |  |  |  |  |  |  |  |  |  |  |
| -0.5847 | 22 | igd | 0.0234 | T | \|\| id:ebi-a-GCST90001703 | ebi-a-GCST90001703 | TRUE | C | 38823838 | 8.77E-13 | reported | 3657 | 0.08151 | rs79928737 |  |  |  |  |  |  |  |  |  |  |  |  |  |  |  |
| -0.6017 | 22 | igd | 0.0234 | T | \|\| id:ebi-a-GCST90001704 | ebi-a-GCST90001704 | TRUE | C | 38823838 | 1.76E-13 | reported | 3657 | 0.08137 | rs79928737 |  |  |  |  |  |  |  |  |  |  |  |  |  |  |  |
| -0.5882 | 22 | igd | 0.0234 | A | \|\| id:ebi-a-GCST90001710 | ebi-a-GCST90001710 | TRUE | C | 38815265 | 5.49E-13 | reported | 3657 | 0.08126 | rs190817865 |  |  |  |  |  |  |  |  |  |  |  |  |  |  |  |
| -0.5912 | 22 | igd | 0.0234 | A | \|\| id:ebi-a-GCST90001711 | ebi-a-GCST90001711 | TRUE | C | 38815265 | 3.77E-13 | reported | 3657 | 0.08109 | rs190817865 |  |  |  |  |  |  |  |  |  |  |  |  |  |  |  |
| -0.6289 | 3 | igd | 0.0233 | T | \|\| id:ebi-a-GCST90002015 | ebi-a-GCST90002015 | TRUE | G | 46320863 | 2.08E-11 | reported | 2871 | 0.09348 | rs116271348 |  |  |  |  |  |  |  |  |  |  |  |  |  |  |  |
| -0.6263 | 3 | igd | 0.0233 | T | \|\| id:ebi-a-GCST90002016 | ebi-a-GCST90002016 | TRUE | G | 46320863 | 2.31E-11 | reported | 2871 | 0.09332 | rs116271348 |  |  |  |  |  |  |  |  |  |  |  |  |  |  |  |
| -0.498 | 1 | igd | 0.0232 | T | \|\| id:ebi-a-GCST90001474 | ebi-a-GCST90001474 | TRUE | C | 160483800 | 5.69E-09 | reported | 3410 | 0.08527 | rs35161722 |  |  |  |  |  |  |  |  |  |  |  |  |  |  |  |
| -0.4854 | 22 | igd | 0.0232 | A | \|\| id:ebi-a-GCST90001702 | ebi-a-GCST90001702 | TRUE | C | 38857451 | 5.94E-09 | reported | 3657 | 0.08323 | rs143128622 |  |  |  |  |  |  |  |  |  |  |  |  |  |  |  |
| -0.4494 | 22 | igd | 0.0232 | G | \|\| id:ebi-a-GCST90001702 | ebi-a-GCST90001702 | TRUE | A | 39383555 | 1.81E-08 | reported | 3657 | 0.07966 | rs11703312 |  |  |  |  |  |  |  |  |  |  |  |  |  |  |  |
| -0.4791 | 22 | igd | 0.0232 | A | \|\| id:ebi-a-GCST90001703 | ebi-a-GCST90001703 | TRUE | C | 38857451 | 9.33E-09 | reported | 3657 | 0.08323 | rs143128622 |  |  |  |  |  |  |  |  |  |  |  |  |  |  |  |
| -0.4367 | 22 | igd | 0.0232 | G | \|\| id:ebi-a-GCST90001703 | ebi-a-GCST90001703 | TRUE | A | 39383555 | 4.52E-08 | reported | 3657 | 0.07968 | rs11703312 |  |  |  |  |  |  |  |  |  |  |  |  |  |  |  |
| -0.4361 | 22 | igd | 0.0232 | G | \|\| id:ebi-a-GCST90001710 | ebi-a-GCST90001710 | TRUE | A | 39383555 | 4.27E-08 | reported | 3657 | 0.07943 | rs11703312 |  |  |  |  |  |  |  |  |  |  |  |  |  |  |  |
| 0.6413 | 17 | igd | 0.0232 | G | \|\| id:ebi-a-GCST90001964 | ebi-a-GCST90001964 | TRUE | A | 62128083 | 7.97E-13 | reported | 2872 | 0.08914 | rs117067704 |  |  |  |  |  |  |  |  |  |  |  |  |  |  |  |
| 0.566 | 6 | igd | 0.0232 | T | \|\| id:ebi-a-GCST90002105 | ebi-a-GCST90002105 | TRUE | C | 29764041 | 7.66E-09 | reported | 2872 | 0.09771 | rs11757990 |  |  |  |  |  |  |  |  |  |  |  |  |  |  |  |
| -0.5359 | 3 | igd | 0.0232 | C | \|\| id:ebi-a-GCST90002014 | ebi-a-GCST90002014 | TRUE | G | 46039473 | 1.61E-08 | reported | 2870 | 0.0946 | rs115046204 |  |  |  |  |  |  |  |  |  |  |  |  |  |  |  |
| 0.4769 | 10 | igd | 0.0231 | T | \|\| id:ebi-a-GCST90001497 | ebi-a-GCST90001497 | TRUE | C | 95621414 | 4.89E-08 | reported | 3437 | 0.08722 | rs7915058 |  |  |  |  |  |  |  |  |  |  |  |  |  |  |  |
| 0.4754 | 7 | igd | 0.0231 | C | \|\| id:ebi-a-GCST90001933 | ebi-a-GCST90001933 | TRUE | T | 35158967 | 2.44E-08 | reported | 3435 | 0.08504 | rs184919955 |  |  |  |  |  |  |  |  |  |  |  |  |  |  |  |
| 0.5057 | 10 | igd | 0.023 | C | \|\| id:ebi-a-GCST90001658 | ebi-a-GCST90001658 | TRUE | T | 92843848 | 9.28E-10 | reported | 3440 | 0.08238 | rs79595377 |  |  |  |  |  |  |  |  |  |  |  |  |  |  |  |
| 0.496 | 10 | igd | 0.023 | C | \|\| id:ebi-a-GCST90001659 | ebi-a-GCST90001659 | TRUE | T | 92843848 | 1.94E-09 | reported | 3440 | 0.08241 | rs79595377 |  |  |  |  |  |  |  |  |  |  |  |  |  |  |  |
| 0.4837 | 7 | igd | 0.023 | A | \|\| id:ebi-a-GCST90001933 | ebi-a-GCST90001933 | TRUE | G | 34988133 | 1.34E-08 | reported | 3435 | 0.08492 | rs116943369 |  |  |  |  |  |  |  |  |  |  |  |  |  |  |  |
| -0.5702 | 12 | igd | 0.023 | T | \|\| id:ebi-a-GCST90002073 | ebi-a-GCST90002073 | TRUE | C | 68826898 | 7.20E-10 | reported | 2850 | 0.09222 | rs141296675 |  |  |  |  |  |  |  |  |  |  |  |  |  |  |  |
| -0.8012 | 12 | igd | 0.023 | C | \|\| id:ebi-a-GCST90002074 | ebi-a-GCST90002074 | TRUE | G | 70360198 | 1.65E-20 | reported | 3112 | 0.08571 | rs148146302 |  |  |  |  |  |  |  |  |  |  |  |  |  |  |  |
| -0.4682 | 22 | igd | 0.0228 | A | \|\| id:ebi-a-GCST90001702 | ebi-a-GCST90001702 | TRUE | G | 38313552 | 3.43E-08 | reported | 3657 | 0.08467 | rs9607500 |  |  |  |  |  |  |  |  |  |  |  |  |  |  |  |
| -0.4746 | 22 | igd | 0.0228 | A | \|\| id:ebi-a-GCST90001703 | ebi-a-GCST90001703 | TRUE | G | 38313552 | 2.21E-08 | reported | 3657 | 0.08465 | rs9607500 |  |  |  |  |  |  |  |  |  |  |  |  |  |  |  |
| -0.4799 | 22 | igd | 0.0228 | A | \|\| id:ebi-a-GCST90001704 | ebi-a-GCST90001704 | TRUE | G | 38313552 | 1.47E-08 | reported | 3657 | 0.08452 | rs9607500 |  |  |  |  |  |  |  |  |  |  |  |  |  |  |  |
| -0.6872 | 22 | igd | 0.0227 | A | \|\| id:ebi-a-GCST90001702 | ebi-a-GCST90001702 | TRUE | C | 44757870 | 3.80E-16 | reported | 3657 | 0.08398 | rs78434681 |  |  |  |  |  |  |  |  |  |  |  |  |  |  |  |
| -0.6905 | 22 | igd | 0.0227 | A | \|\| id:ebi-a-GCST90001703 | ebi-a-GCST90001703 | TRUE | C | 44757870 | 2.74E-16 | reported | 3657 | 0.08398 | rs78434681 |  |  |  |  |  |  |  |  |  |  |  |  |  |  |  |
| -0.7197 | 22 | igd | 0.0227 | A | \|\| id:ebi-a-GCST90001704 | ebi-a-GCST90001704 | TRUE | C | 44757870 | 1.30E-17 | reported | 3657 | 0.08381 | rs78434681 |  |  |  |  |  |  |  |  |  |  |  |  |  |  |  |
| -0.6652 | 22 | igd | 0.0227 | A | \|\| id:ebi-a-GCST90001710 | ebi-a-GCST90001710 | TRUE | C | 44757870 | 2.65E-15 | reported | 3657 | 0.08377 | rs78434681 |  |  |  |  |  |  |  |  |  |  |  |  |  |  |  |
| -0.6938 | 22 | igd | 0.0227 | A | \|\| id:ebi-a-GCST90001711 | ebi-a-GCST90001711 | TRUE | C | 44757870 | 1.40E-16 | reported | 3657 | 0.08355 | rs78434681 |  |  |  |  |  |  |  |  |  |  |  |  |  |  |  |
| 0.5257 | 10 | igd | 0.0226 | C | \|\| id:ebi-a-GCST90001489 | ebi-a-GCST90001489 | TRUE | T | 92843848 | 1.90E-09 | reported | 3405 | 0.08728 | rs79595377 |  |  |  |  |  |  |  |  |  |  |  |  |  |  |  |
| 0.4899 | 10 | igd | 0.0226 | C | \|\| id:ebi-a-GCST90001495 | ebi-a-GCST90001495 | TRUE | T | 92843848 | 3.06E-08 | reported | 3405 | 0.08825 | rs79595377 |  |  |  |  |  |  |  |  |  |  |  |  |  |  |  |
| 0.4915 | 10 | igd | 0.0226 | C | \|\| id:ebi-a-GCST90001660 | ebi-a-GCST90001660 | TRUE | T | 92843848 | 1.97E-08 | reported | 3408 | 0.08734 | rs79595377 |  |  |  |  |  |  |  |  |  |  |  |  |  |  |  |
| 66.78 | 10 | igd | 0.0226 | A | \|\| id:ebi-a-GCST90001698 | ebi-a-GCST90001698 | TRUE | G | 21789927 | 3.56E-08 | reported | 3408 | 12.09 | rs76756668 |  |  |  |  |  |  |  |  |  |  |  |  |  |  |  |
| -0.4709 | 1 | igd | 0.0226 | G | \|\| id:ebi-a-GCST90001989 | ebi-a-GCST90001989 | TRUE | C | 161475114 | 2.60E-08 | reported | 3621 | 0.08441 | rs72717028 |  |  |  |  |  |  |  |  |  |  |  |  |  |  |  |
| 0.482 | 1 | igd | 0.0226 | A | \|\| id:ebi-a-GCST90002080 | ebi-a-GCST90002080 | TRUE | G | 205560814 | 1.47E-08 | reported | 3113 | 0.08487 | rs115752428 |  |  |  |  |  |  |  |  |  |  |  |  |  |  |  |
| 0.4677 | 10 | igd | 0.0225 | G | \|\| id:ebi-a-GCST90001670 | ebi-a-GCST90001670 | TRUE | A | 99958280 | 2.93E-08 | reported | 3440 | 0.08415 | rs4919186 |  |  |  |  |  |  |  |  |  |  |  |  |  |  |  |
| 0.4871 | 10 | igd | 0.0225 | G | \|\| id:ebi-a-GCST90001671 | ebi-a-GCST90001671 | TRUE | A | 99958280 | 7.50E-09 | reported | 3440 | 0.08408 | rs4919186 |  |  |  |  |  |  |  |  |  |  |  |  |  |  |  |
| -0.5509 | 3 | igd | 0.0225 | G | \|\| id:ebi-a-GCST90002015 | ebi-a-GCST90002015 | TRUE | A | 45647096 | 1.56E-09 | reported | 2871 | 0.09095 | rs116641116 |  |  |  |  |  |  |  |  |  |  |  |  |  |  |  |
| -0.5515 | 3 | igd | 0.0225 | G | \|\| id:ebi-a-GCST90002016 | ebi-a-GCST90002016 | TRUE | A | 45647096 | 1.43E-09 | reported | 2871 | 0.09084 | rs116641116 |  |  |  |  |  |  |  |  |  |  |  |  |  |  |  |
| 50.84 | 6 | igd | 0.0224 | T | \|\| id:ebi-a-GCST90001695 | ebi-a-GCST90001695 | TRUE | C | 67268882 | 6.47E-09 | reported | 3408 | 8.738 | rs79236020 |  |  |  |  |  |  |  |  |  |  |  |  |  |  |  |
| 75.87 | 5 | igd | 0.0224 | G | \|\| id:ebi-a-GCST90001698 | ebi-a-GCST90001698 | TRUE | A | 81200772 | 2.71E-10 | reported | 3408 | 11.98 | rs11738987 |  |  |  |  |  |  |  |  |  |  |  |  |  |  |  |
| 0.6567 | 1 | igd | 0.0224 | A | \|\| id:ebi-a-GCST90001728 | ebi-a-GCST90001728 | TRUE | G | 207018206 | 1.65E-08 | reported | 1833 | 0.1158 | rs144743804 |  |  |  |  |  |  |  |  |  |  |  |  |  |  |  |
| 0.473 | 10 | igd | 0.0223 | G | \|\| id:ebi-a-GCST90001672 | ebi-a-GCST90001672 | TRUE | A | 99958280 | 3.44E-08 | reported | 3408 | 0.08553 | rs4919186 |  |  |  |  |  |  |  |  |  |  |  |  |  |  |  |
| 0.5735 | 3 | igd | 0.0223 | C | \|\| id:ebi-a-GCST90001800 | ebi-a-GCST90001800 | TRUE | A | 31134534 | 5.04E-11 | reported | 3656 | 0.08703 | rs76929865 |  |  |  |  |  |  |  |  |  |  |  |  |  |  |  |
| 0.4995 | 7 | igd | 0.0223 | A | \|\| id:ebi-a-GCST90001933 | ebi-a-GCST90001933 | TRUE | T | 35264731 | 8.30E-09 | reported | 3435 | 0.08646 | rs181516190 |  |  |  |  |  |  |  |  |  |  |  |  |  |  |  |
| 0.4773 | 1 | igd | 0.0223 | C | \|\| id:ebi-a-GCST90001985 | ebi-a-GCST90001985 | TRUE | A | 161383559 | 2.91E-08 | reported | 3629 | 0.08586 | rs143135133 |  |  |  |  |  |  |  |  |  |  |  |  |  |  |  |
| -0.5163 | 3 | igd | 0.0223 | C | \|\| id:ebi-a-GCST90002015 | ebi-a-GCST90002015 | TRUE | T | 45757826 | 3.23E-08 | reported | 2871 | 0.09313 | rs77559692 |  |  |  |  |  |  |  |  |  |  |  |  |  |  |  |
| -0.5176 | 3 | igd | 0.0223 | C | \|\| id:ebi-a-GCST90002016 | ebi-a-GCST90002016 | TRUE | T | 45757826 | 2.86E-08 | reported | 2871 | 0.093 | rs77559692 |  |  |  |  |  |  |  |  |  |  |  |  |  |  |  |
| -0.6566 | 12 | igd | 0.0223 | G | \|\| id:ebi-a-GCST90002073 | ebi-a-GCST90002073 | TRUE | A | 70378617 | 3.10E-11 | reported | 2850 | 0.09848 | rs117696306 |  |  |  |  |  |  |  |  |  |  |  |  |  |  |  |
| -0.598 | 12 | igd | 0.0223 | G | \|\| id:ebi-a-GCST90002074 | ebi-a-GCST90002074 | TRUE | A | 70378617 | 3.91E-10 | reported | 3112 | 0.09525 | rs117696306 |  |  |  |  |  |  |  |  |  |  |  |  |  |  |  |
| -0.5601 | 6 | igd | 0.0223 | G | \|\| id:ebi-a-GCST90002105 | ebi-a-GCST90002105 | TRUE | C | 31813576 | 1.73E-08 | reported | 2872 | 0.09907 | rs9469063 |  |  |  |  |  |  |  |  |  |  |  |  |  |  |  |
| 0.4919 | 10 | igd | 0.0221 | C | \|\| id:ebi-a-GCST90001484 | ebi-a-GCST90001484 | TRUE | T | 97683907 | 6.95E-09 | reported | 3437 | 0.0847 | rs74503287 |  |  |  |  |  |  |  |  |  |  |  |  |  |  |  |
| 0.494 | 10 | igd | 0.0221 | C | \|\| id:ebi-a-GCST90001485 | ebi-a-GCST90001485 | TRUE | T | 97683907 | 5.70E-09 | reported | 3437 | 0.08458 | rs74503287 |  |  |  |  |  |  |  |  |  |  |  |  |  |  |  |
| 0.4768 | 10 | igd | 0.0221 | C | \|\| id:ebi-a-GCST90001491 | ebi-a-GCST90001491 | TRUE | T | 97683907 | 3.46E-08 | reported | 3437 | 0.08624 | rs74503287 |  |  |  |  |  |  |  |  |  |  |  |  |  |  |  |
| 0.5887 | 10 | igd | 0.0221 | C | \|\| id:ebi-a-GCST90001496 | ebi-a-GCST90001496 | TRUE | T | 97683907 | 1.72E-11 | reported | 3437 | 0.08721 | rs74503287 |  |  |  |  |  |  |  |  |  |  |  |  |  |  |  |
| 0.561 | 10 | igd | 0.0221 | C | \|\| id:ebi-a-GCST90001497 | ebi-a-GCST90001497 | TRUE | T | 97683907 | 8.47E-11 | reported | 3437 | 0.08615 | rs74503287 |  |  |  |  |  |  |  |  |  |  |  |  |  |  |  |
| 0.5547 | 10 | igd | 0.0221 | C | \|\| id:ebi-a-GCST90001658 | ebi-a-GCST90001658 | TRUE | T | 97683907 | 2.91E-11 | reported | 3440 | 0.08313 | rs74503287 |  |  |  |  |  |  |  |  |  |  |  |  |  |  |  |
| 0.7928 | 10 | igd | 0.0221 | C | \|\| id:ebi-a-GCST90002030 | ebi-a-GCST90002030 | TRUE | T | 97683907 | 1.19E-17 | reported | 2920 | 0.09209 | rs74503287 |  |  |  |  |  |  |  |  |  |  |  |  |  |  |  |
| 0.6642 | 10 | igd | 0.0221 | C | \|\| id:ebi-a-GCST90002031 | ebi-a-GCST90002031 | TRUE | T | 97683907 | 2.84E-12 | reported | 2920 | 0.09467 | rs74503287 |  |  |  |  |  |  |  |  |  |  |  |  |  |  |  |
| 0.7433 | 10 | igd | 0.0221 | C | \|\| id:ebi-a-GCST90002032 | ebi-a-GCST90002032 | TRUE | T | 97683907 | 4.84E-15 | reported | 2920 | 0.09441 | rs74503287 |  |  |  |  |  |  |  |  |  |  |  |  |  |  |  |
| 0.5439 | 6 | igd | 0.0221 | A | \|\| id:ebi-a-GCST90002105 | ebi-a-GCST90002105 | TRUE | G | 32115876 | 9.21E-09 | reported | 2872 | 0.0944 | rs114620154 |  |  |  |  |  |  |  |  |  |  |  |  |  |  |  |
| 0.496 | 10 | igd | 0.022 | C | \|\| id:ebi-a-GCST90001483 | ebi-a-GCST90001483 | TRUE | T | 97683907 | 7.32E-09 | reported | 3405 | 0.08555 | rs74503287 |  |  |  |  |  |  |  |  |  |  |  |  |  |  |  |
| 0.6174 | 10 | igd | 0.022 | C | \|\| id:ebi-a-GCST90001495 | ebi-a-GCST90001495 | TRUE | T | 97683907 | 3.37E-12 | reported | 3405 | 0.08837 | rs74503287 |  |  |  |  |  |  |  |  |  |  |  |  |  |  |  |
| 0.5768 | 10 | igd | 0.022 | C | \|\| id:ebi-a-GCST90001660 | ebi-a-GCST90001660 | TRUE | T | 97683907 | 4.79E-11 | reported | 3408 | 0.0874 | rs74503287 |  |  |  |  |  |  |  |  |  |  |  |  |  |  |  |
| -0.5174 | 1 | igd | 0.022 | T | \|\| id:ebi-a-GCST90002077 | ebi-a-GCST90002077 | TRUE | C | 160483800 | 3.34E-08 | reported | 2971 | 0.09344 | rs35161722 |  |  |  |  |  |  |  |  |  |  |  |  |  |  |  |
| 0.5092 | 10 | igd | 0.0219 | A | \|\| id:ebi-a-GCST90001483 | ebi-a-GCST90001483 | TRUE | G | 96132500 | 2.25E-09 | reported | 3405 | 0.08493 | rs76736637 |  |  |  |  |  |  |  |  |  |  |  |  |  |  |  |
| 0.4773 | 10 | igd | 0.0219 | A | \|\| id:ebi-a-GCST90001489 | ebi-a-GCST90001489 | TRUE | G | 96132500 | 4.92E-08 | reported | 3405 | 0.08732 | rs76736637 |  |  |  |  |  |  |  |  |  |  |  |  |  |  |  |
| 0.5564 | 10 | igd | 0.0219 | A | \|\| id:ebi-a-GCST90001660 | ebi-a-GCST90001660 | TRUE | G | 96132500 | 1.69E-10 | reported | 3408 | 0.08684 | rs76736637 |  |  |  |  |  |  |  |  |  |  |  |  |  |  |  |
| 0.5559 | 3 | igd | 0.0219 | G | \|\| id:ebi-a-GCST90001800 | ebi-a-GCST90001800 | TRUE | C | 31003613 | 3.57E-10 | reported | 3656 | 0.08839 | rs140362691 |  |  |  |  |  |  |  |  |  |  |  |  |  |  |  |
| -0.9169 | 19 | igd | 0.0219 | C | \|\| id:ebi-a-GCST90001947 | ebi-a-GCST90001947 | TRUE | A | 51737053 | 1.14E-13 | reported | 1577 | 0.1224 | rs113464261 |  |  |  |  |  |  |  |  |  |  |  |  |  |  |  |
| -0.9284 | 19 | igd | 0.0219 | C | \|\| id:ebi-a-GCST90001954 | ebi-a-GCST90001954 | TRUE | A | 51737053 | 4.98E-14 | reported | 1555 | 0.1221 | rs113464261 |  |  |  |  |  |  |  |  |  |  |  |  |  |  |  |
| -0.8784 | 19 | igd | 0.0219 | C | \|\| id:ebi-a-GCST90001955 | ebi-a-GCST90001955 | TRUE | A | 51737053 | 1.25E-12 | reported | 1554 | 0.1227 | rs113464261 |  |  |  |  |  |  |  |  |  |  |  |  |  |  |  |
| -0.8814 | 19 | igd | 0.0219 | C | \|\| id:ebi-a-GCST90001957 | ebi-a-GCST90001957 | TRUE | A | 51737053 | 8.63E-13 | reported | 1578 | 0.1222 | rs113464261 |  |  |  |  |  |  |  |  |  |  |  |  |  |  |  |
| 0.5845 | 10 | igd | 0.0219 | A | \|\| id:ebi-a-GCST90002030 | ebi-a-GCST90002030 | TRUE | G | 96132500 | 2.60E-10 | reported | 2920 | 0.09215 | rs76736637 |  |  |  |  |  |  |  |  |  |  |  |  |  |  |  |
| -0.5505 | 12 | igd | 0.0219 | G | \|\| id:ebi-a-GCST90002073 | ebi-a-GCST90002073 | TRUE | A | 69303704 | 4.07E-09 | reported | 2850 | 0.09331 | rs138455208 |  |  |  |  |  |  |  |  |  |  |  |  |  |  |  |
| 0.4835 | 10 | igd | 0.0218 | A | \|\| id:ebi-a-GCST90001484 | ebi-a-GCST90001484 | TRUE | G | 96132500 | 1.10E-08 | reported | 3437 | 0.08441 | rs76736637 |  |  |  |  |  |  |  |  |  |  |  |  |  |  |  |
| 0.5106 | 10 | igd | 0.0218 | A | \|\| id:ebi-a-GCST90001485 | ebi-a-GCST90001485 | TRUE | G | 96132500 | 1.50E-09 | reported | 3437 | 0.08424 | rs76736637 |  |  |  |  |  |  |  |  |  |  |  |  |  |  |  |
| 0.4796 | 10 | igd | 0.0218 | A | \|\| id:ebi-a-GCST90001496 | ebi-a-GCST90001496 | TRUE | G | 96132500 | 3.97E-08 | reported | 3437 | 0.08712 | rs76736637 |  |  |  |  |  |  |  |  |  |  |  |  |  |  |  |
| 0.617 | 10 | igd | 0.0218 | A | \|\| id:ebi-a-GCST90001658 | ebi-a-GCST90001658 | TRUE | G | 96132500 | 1.10E-13 | reported | 3440 | 0.08272 | rs76736637 |  |  |  |  |  |  |  |  |  |  |  |  |  |  |  |
| 0.5963 | 10 | igd | 0.0218 | A | \|\| id:ebi-a-GCST90001659 | ebi-a-GCST90001659 | TRUE | G | 96132500 | 7.14E-13 | reported | 3440 | 0.08277 | rs76736637 |  |  |  |  |  |  |  |  |  |  |  |  |  |  |  |
| 0.5157 | 10 | igd | 0.0218 | A | \|\| id:ebi-a-GCST90001670 | ebi-a-GCST90001670 | TRUE | G | 96132500 | 2.61E-09 | reported | 3440 | 0.08638 | rs76736637 |  |  |  |  |  |  |  |  |  |  |  |  |  |  |  |
| -0.9133 | 19 | igd | 0.0218 | C | \|\| id:ebi-a-GCST90001953 | ebi-a-GCST90001953 | TRUE | A | 51737053 | 1.26E-13 | reported | 1558 | 0.1221 | rs113464261 |  |  |  |  |  |  |  |  |  |  |  |  |  |  |  |
| -0.8876 | 19 | igd | 0.0218 | C | \|\| id:ebi-a-GCST90001956 | ebi-a-GCST90001956 | TRUE | A | 51737053 | 6.11E-13 | reported | 1579 | 0.1223 | rs113464261 |  |  |  |  |  |  |  |  |  |  |  |  |  |  |  |
| 76.4 | 4 | igd | 0.0217 | T | \|\| id:ebi-a-GCST90001698 | ebi-a-GCST90001698 | TRUE | C | 14229184 | 2.03E-10 | reported | 3408 | 11.98 | rs137976632 |  |  |  |  |  |  |  |  |  |  |  |  |  |  |  |
| 0.5525 | 10 | igd | 0.0216 | A | \|\| id:ebi-a-GCST90001489 | ebi-a-GCST90001489 | TRUE | G | 97367813 | 1.01E-09 | reported | 3405 | 0.0902 | rs112194607 |  |  |  |  |  |  |  |  |  |  |  |  |  |  |  |
| 0.5483 | 10 | igd | 0.0216 | G | \|\| id:ebi-a-GCST90001489 | ebi-a-GCST90001489 | TRUE | A | 97496679 | 1.82E-09 | reported | 3405 | 0.09094 | rs17553185 |  |  |  |  |  |  |  |  |  |  |  |  |  |  |  |
| 0.6103 | 10 | igd | 0.0216 | G | \|\| id:ebi-a-GCST90001495 | ebi-a-GCST90001495 | TRUE | A | 97496679 | 3.39E-11 | reported | 3405 | 0.09177 | rs17553185 |  |  |  |  |  |  |  |  |  |  |  |  |  |  |  |
| 0.5194 | 10 | igd | 0.0216 | A | \|\| id:ebi-a-GCST90001495 | ebi-a-GCST90001495 | TRUE | G | 97367813 | 1.38E-08 | reported | 3405 | 0.09129 | rs112194607 |  |  |  |  |  |  |  |  |  |  |  |  |  |  |  |
| 0.5032 | 10 | igd | 0.0216 | G | \|\| id:ebi-a-GCST90001660 | ebi-a-GCST90001660 | TRUE | A | 97496679 | 3.26E-08 | reported | 3408 | 0.09082 | rs17553185 |  |  |  |  |  |  |  |  |  |  |  |  |  |  |  |
| 0.5784 | 10 | igd | 0.0216 | A | \|\| id:ebi-a-GCST90001660 | ebi-a-GCST90001660 | TRUE | G | 97367813 | 1.70E-10 | reported | 3408 | 0.09029 | rs112194607 |  |  |  |  |  |  |  |  |  |  |  |  |  |  |  |
| 76.74 | 2 | igd | 0.0216 | A | \|\| id:ebi-a-GCST90001698 | ebi-a-GCST90001698 | TRUE | T | 81191156 | 1.93E-10 | reported | 3408 | 12.02 | rs78530932 |  |  |  |  |  |  |  |  |  |  |  |  |  |  |  |
| 0.5786 | 6 | igd | 0.0216 | A | \|\| id:ebi-a-GCST90002118 | ebi-a-GCST90002118 | TRUE | G | 30751379 | 2.02E-09 | reported | 2920 | 0.09618 | rs62408691 |  |  |  |  |  |  |  |  |  |  |  |  |  |  |  |
| 0.7181 | 1 | igd | 0.0215 | G | \|\| id:ebi-a-GCST90001728 | ebi-a-GCST90001728 | TRUE | A | 207276336 | 7.39E-10 | reported | 1833 | 0.116 | rs113812519 |  |  |  |  |  |  |  |  |  |  |  |  |  |  |  |
| -0.5283 | 12 | igd | 0.0215 | G | \|\| id:ebi-a-GCST90002074 | ebi-a-GCST90002074 | TRUE | A | 69303704 | 5.52E-09 | reported | 3112 | 0.09035 | rs138455208 |  |  |  |  |  |  |  |  |  |  |  |  |  |  |  |
| 0.5036 | 10 | igd | 0.0214 | A | \|\| id:ebi-a-GCST90001484 | ebi-a-GCST90001484 | TRUE | G | 97367813 | 8.57E-09 | reported | 3437 | 0.08727 | rs112194607 |  |  |  |  |  |  |  |  |  |  |  |  |  |  |  |
| 0.5348 | 10 | igd | 0.0214 | A | \|\| id:ebi-a-GCST90001491 | ebi-a-GCST90001491 | TRUE | G | 97367813 | 2.26E-09 | reported | 3437 | 0.08922 | rs112194607 |  |  |  |  |  |  |  |  |  |  |  |  |  |  |  |
| 0.5305 | 10 | igd | 0.0214 | G | \|\| id:ebi-a-GCST90001491 | ebi-a-GCST90001491 | TRUE | A | 97496679 | 4.03E-09 | reported | 3437 | 0.08995 | rs17553185 |  |  |  |  |  |  |  |  |  |  |  |  |  |  |  |
| 0.5103 | 10 | igd | 0.0214 | A | \|\| id:ebi-a-GCST90001496 | ebi-a-GCST90001496 | TRUE | G | 97367813 | 1.84E-08 | reported | 3437 | 0.09047 | rs112194607 |  |  |  |  |  |  |  |  |  |  |  |  |  |  |  |
| 0.6095 | 10 | igd | 0.0214 | G | \|\| id:ebi-a-GCST90001496 | ebi-a-GCST90001496 | TRUE | A | 97496679 | 2.49E-11 | reported | 3437 | 0.09102 | rs17553185 |  |  |  |  |  |  |  |  |  |  |  |  |  |  |  |
| 0.511 | 10 | igd | 0.0214 | A | \|\| id:ebi-a-GCST90001497 | ebi-a-GCST90001497 | TRUE | G | 97367813 | 1.15E-08 | reported | 3437 | 0.08933 | rs112194607 |  |  |  |  |  |  |  |  |  |  |  |  |  |  |  |
| 0.5878 | 10 | igd | 0.0214 | G | \|\| id:ebi-a-GCST90001497 | ebi-a-GCST90001497 | TRUE | A | 97496679 | 7.14E-11 | reported | 3437 | 0.08991 | rs17553185 |  |  |  |  |  |  |  |  |  |  |  |  |  |  |  |
| 0.4772 | 10 | igd | 0.0214 | G | \|\| id:ebi-a-GCST90001658 | ebi-a-GCST90001658 | TRUE | A | 97496679 | 4.30E-08 | reported | 3440 | 0.08691 | rs17553185 |  |  |  |  |  |  |  |  |  |  |  |  |  |  |  |
| 0.6503 | 10 | igd | 0.0214 | A | \|\| id:ebi-a-GCST90001658 | ebi-a-GCST90001658 | TRUE | G | 97367813 | 5.19E-14 | reported | 3440 | 0.08603 | rs112194607 |  |  |  |  |  |  |  |  |  |  |  |  |  |  |  |
| 0.6171 | 10 | igd | 0.0214 | A | \|\| id:ebi-a-GCST90001659 | ebi-a-GCST90001659 | TRUE | G | 97367813 | 9.42E-13 | reported | 3440 | 0.08611 | rs112194607 |  |  |  |  |  |  |  |  |  |  |  |  |  |  |  |
| 0.5027 | 10 | igd | 0.0214 | A | \|\| id:ebi-a-GCST90001670 | ebi-a-GCST90001670 | TRUE | G | 97367813 | 2.33E-08 | reported | 3440 | 0.0898 | rs112194607 |  |  |  |  |  |  |  |  |  |  |  |  |  |  |  |
| 0.5349 | 10 | igd | 0.0214 | A | \|\| id:ebi-a-GCST90001671 | ebi-a-GCST90001671 | TRUE | G | 97367813 | 3.02E-09 | reported | 3440 | 0.08995 | rs112194607 |  |  |  |  |  |  |  |  |  |  |  |  |  |  |  |
| -0.4829 | 22 | igd | 0.0214 | T | \|\| id:ebi-a-GCST90001701 | ebi-a-GCST90001701 | TRUE | C | 39903477 | 1.40E-08 | reported | 3592 | 0.08492 | rs146514405 |  |  |  |  |  |  |  |  |  |  |  |  |  |  |  |
| 0.5264 | 1 | igd | 0.0214 | T | \|\| id:ebi-a-GCST90001856 | ebi-a-GCST90001856 | TRUE | C | 166035343 | 4.79E-08 | reported | 2919 | 0.09617 | rs76540213 |  |  |  |  |  |  |  |  |  |  |  |  |  |  |  |
| -0.853 | 19 | igd | 0.0214 | C | \|\| id:ebi-a-GCST90001952 | ebi-a-GCST90001952 | TRUE | A | 51737053 | 1.15E-11 | reported | 1569 | 0.1248 | rs113464261 |  |  |  |  |  |  |  |  |  |  |  |  |  |  |  |
| 0.5587 | 10 | igd | 0.0214 | A | \|\| id:ebi-a-GCST90002029 | ebi-a-GCST90002029 | TRUE | G | 97367813 | 2.65E-09 | reported | 2915 | 0.09358 | rs112194607 |  |  |  |  |  |  |  |  |  |  |  |  |  |  |  |
| 0.7529 | 10 | igd | 0.0214 | A | \|\| id:ebi-a-GCST90002030 | ebi-a-GCST90002030 | TRUE | G | 97367813 | 1.13E-14 | reported | 2920 | 0.09697 | rs112194607 |  |  |  |  |  |  |  |  |  |  |  |  |  |  |  |
| 0.7735 | 10 | igd | 0.0214 | A | \|\| id:ebi-a-GCST90002031 | ebi-a-GCST90002031 | TRUE | G | 97367813 | 4.31E-15 | reported | 2920 | 0.09806 | rs112194607 |  |  |  |  |  |  |  |  |  |  |  |  |  |  |  |
| 0.7492 | 10 | igd | 0.0214 | A | \|\| id:ebi-a-GCST90002032 | ebi-a-GCST90002032 | TRUE | G | 97367813 | 3.21E-14 | reported | 2920 | 0.09822 | rs112194607 |  |  |  |  |  |  |  |  |  |  |  |  |  |  |  |
| -0.6368 | 12 | igd | 0.0214 | T | \|\| id:ebi-a-GCST90002074 | ebi-a-GCST90002074 | TRUE | C | 69953596 | 3.70E-12 | reported | 3112 | 0.09129 | rs532669 |  |  |  |  |  |  |  |  |  |  |  |  |  |  |  |
| -0.5543 | 6 | igd | 0.0214 | T | \|\| id:ebi-a-GCST90002105 | ebi-a-GCST90002105 | TRUE | C | 31974014 | 3.39E-08 | reported | 2872 | 0.1002 | rs139010734 |  |  |  |  |  |  |  |  |  |  |  |  |  |  |  |
| 75.83 | 6 | igd | 0.0213 | A | \|\| id:ebi-a-GCST90001698 | ebi-a-GCST90001698 | TRUE | G | 20219649 | 7.69E-10 | reported | 3408 | 12.29 | rs35901634 |  |  |  |  |  |  |  |  |  |  |  |  |  |  |  |
| -0.9707 | 22 | igd | 0.0213 | T | \|\| id:ebi-a-GCST90001702 | ebi-a-GCST90001702 | TRUE | C | 39903477 | 2.52E-29 | reported | 3657 | 0.08559 | rs146514405 |  |  |  |  |  |  |  |  |  |  |  |  |  |  |  |
| 0.4814 | 10 | igd | 0.0212 | A | \|\| id:ebi-a-GCST90001658 | ebi-a-GCST90001658 | TRUE | G | 95830221 | 2.29E-08 | reported | 3440 | 0.08593 | rs61886297 |  |  |  |  |  |  |  |  |  |  |  |  |  |  |  |
| 0.5934 | 10 | igd | 0.0212 | A | \|\| id:ebi-a-GCST90001659 | ebi-a-GCST90001659 | TRUE | G | 97696820 | 3.26E-12 | reported | 3440 | 0.08487 | rs80150604 |  |  |  |  |  |  |  |  |  |  |  |  |  |  |  |
| -0.5154 | 1 | igd | 0.0212 | T | \|\| id:ebi-a-GCST90001691 | ebi-a-GCST90001691 | TRUE | C | 193505625 | 3.04E-09 | reported | 3440 | 0.0867 | rs60220946 |  |  |  |  |  |  |  |  |  |  |  |  |  |  |  |
| 0.5818 | 10 | igd | 0.0212 | T | \|\| id:ebi-a-GCST90002030 | ebi-a-GCST90002030 | TRUE | G | 96311553 | 5.72E-10 | reported | 2920 | 0.09356 | rs185474383 |  |  |  |  |  |  |  |  |  |  |  |  |  |  |  |
| 0.5403 | 10 | igd | 0.0212 | T | \|\| id:ebi-a-GCST90002031 | ebi-a-GCST90002031 | TRUE | G | 96311553 | 1.79E-08 | reported | 2920 | 0.09569 | rs185474383 |  |  |  |  |  |  |  |  |  |  |  |  |  |  |  |
| -0.5777 | 12 | igd | 0.0212 | C | \|\| id:ebi-a-GCST90002073 | ebi-a-GCST90002073 | TRUE | T | 67839087 | 4.35E-09 | reported | 2850 | 0.0981 | rs80216229 |  |  |  |  |  |  |  |  |  |  |  |  |  |  |  |
| 0.7929 | 10 | igd | 0.0211 | G | \|\| id:ebi-a-GCST90001484 | ebi-a-GCST90001484 | TRUE | A | 95850055 | 1.24E-19 | reported | 3437 | 0.08694 | rs145557266 |  |  |  |  |  |  |  |  |  |  |  |  |  |  |  |
| 0.6978 | 10 | igd | 0.0211 | G | \|\| id:ebi-a-GCST90001485 | ebi-a-GCST90001485 | TRUE | A | 95850055 | 1.49E-15 | reported | 3437 | 0.08705 | rs145557266 |  |  |  |  |  |  |  |  |  |  |  |  |  |  |  |
| 0.5371 | 10 | igd | 0.0211 | A | \|\| id:ebi-a-GCST90001489 | ebi-a-GCST90001489 | TRUE | G | 97696820 | 2.42E-09 | reported | 3405 | 0.08976 | rs80150604 |  |  |  |  |  |  |  |  |  |  |  |  |  |  |  |
| 0.6071 | 10 | igd | 0.0211 | G | \|\| id:ebi-a-GCST90001491 | ebi-a-GCST90001491 | TRUE | A | 95850055 | 1.37E-11 | reported | 3437 | 0.08948 | rs145557266 |  |  |  |  |  |  |  |  |  |  |  |  |  |  |  |
| 0.7359 | 10 | igd | 0.0211 | G | \|\| id:ebi-a-GCST90001496 | ebi-a-GCST90001496 | TRUE | A | 95850055 | 5.55E-16 | reported | 3437 | 0.09042 | rs145557266 |  |  |  |  |  |  |  |  |  |  |  |  |  |  |  |
| 0.7299 | 10 | igd | 0.0211 | G | \|\| id:ebi-a-GCST90001497 | ebi-a-GCST90001497 | TRUE | A | 95850055 | 4.09E-16 | reported | 3437 | 0.08928 | rs145557266 |  |  |  |  |  |  |  |  |  |  |  |  |  |  |  |
| 0.6746 | 10 | igd | 0.0211 | G | \|\| id:ebi-a-GCST90001658 | ebi-a-GCST90001658 | TRUE | A | 95850055 | 7.61E-15 | reported | 3440 | 0.08639 | rs145557266 |  |  |  |  |  |  |  |  |  |  |  |  |  |  |  |
| 0.7452 | 10 | igd | 0.0211 | G | \|\| id:ebi-a-GCST90001659 | ebi-a-GCST90001659 | TRUE | A | 95850055 | 8.40E-18 | reported | 3440 | 0.08624 | rs145557266 |  |  |  |  |  |  |  |  |  |  |  |  |  |  |  |
| 69.9 | 19 | igd | 0.0211 | T | \|\| id:ebi-a-GCST90001698 | ebi-a-GCST90001698 | TRUE | C | 50001170 | 7.48E-09 | reported | 3408 | 12.06 | rs140800343 |  |  |  |  |  |  |  |  |  |  |  |  |  |  |  |
| 0.569 | 10 | igd | 0.0211 | G | \|\| id:ebi-a-GCST90002030 | ebi-a-GCST90002030 | TRUE | A | 95850055 | 3.84E-09 | reported | 2920 | 0.09629 | rs145557266 |  |  |  |  |  |  |  |  |  |  |  |  |  |  |  |
| 0.6669 | 10 | igd | 0.021 | G | \|\| id:ebi-a-GCST90001483 | ebi-a-GCST90001483 | TRUE | A | 95850055 | 4.85E-14 | reported | 3405 | 0.08812 | rs145557266 |  |  |  |  |  |  |  |  |  |  |  |  |  |  |  |
| 0.5292 | 10 | igd | 0.021 | T | \|\| id:ebi-a-GCST90001483 | ebi-a-GCST90001483 | TRUE | G | 96311553 | 1.10E-09 | reported | 3405 | 0.08658 | rs185474383 |  |  |  |  |  |  |  |  |  |  |  |  |  |  |  |
| 0.558 | 10 | igd | 0.021 | G | \|\| id:ebi-a-GCST90001489 | ebi-a-GCST90001489 | TRUE | A | 95850055 | 8.85E-10 | reported | 3405 | 0.09078 | rs145557266 |  |  |  |  |  |  |  |  |  |  |  |  |  |  |  |
| 0.6755 | 10 | igd | 0.021 | G | \|\| id:ebi-a-GCST90001495 | ebi-a-GCST90001495 | TRUE | A | 95850055 | 2.05E-13 | reported | 3405 | 0.09159 | rs145557266 |  |  |  |  |  |  |  |  |  |  |  |  |  |  |  |
| 0.5673 | 10 | igd | 0.021 | T | \|\| id:ebi-a-GCST90001660 | ebi-a-GCST90001660 | TRUE | G | 96311553 | 1.75E-10 | reported | 3408 | 0.08862 | rs185474383 |  |  |  |  |  |  |  |  |  |  |  |  |  |  |  |
| 0.7936 | 10 | igd | 0.021 | G | \|\| id:ebi-a-GCST90001660 | ebi-a-GCST90001660 | TRUE | A | 95850055 | 2.46E-18 | reported | 3408 | 0.09034 | rs145557266 |  |  |  |  |  |  |  |  |  |  |  |  |  |  |  |
| 70.95 | 15 | igd | 0.021 | A | \|\| id:ebi-a-GCST90001698 | ebi-a-GCST90001698 | TRUE | G | 32413895 | 5.15E-09 | reported | 3408 | 12.11 | rs117638796 |  |  |  |  |  |  |  |  |  |  |  |  |  |  |  |
| 0.4997 | 10 | igd | 0.0209 | T | \|\| id:ebi-a-GCST90001484 | ebi-a-GCST90001484 | TRUE | G | 96311553 | 6.83E-09 | reported | 3437 | 0.08601 | rs185474383 |  |  |  |  |  |  |  |  |  |  |  |  |  |  |  |
| 0.5279 | 10 | igd | 0.0209 | T | \|\| id:ebi-a-GCST90001485 | ebi-a-GCST90001485 | TRUE | G | 96311553 | 8.62E-10 | reported | 3437 | 0.08583 | rs185474383 |  |  |  |  |  |  |  |  |  |  |  |  |  |  |  |
| 0.6145 | 10 | igd | 0.0209 | T | \|\| id:ebi-a-GCST90001658 | ebi-a-GCST90001658 | TRUE | G | 96311553 | 4.17E-13 | reported | 3440 | 0.08443 | rs185474383 |  |  |  |  |  |  |  |  |  |  |  |  |  |  |  |
| 0.5974 | 10 | igd | 0.0209 | T | \|\| id:ebi-a-GCST90001659 | ebi-a-GCST90001659 | TRUE | G | 96311553 | 1.85E-12 | reported | 3440 | 0.08448 | rs185474383 |  |  |  |  |  |  |  |  |  |  |  |  |  |  |  |
| 0.4978 | 10 | igd | 0.0209 | T | \|\| id:ebi-a-GCST90001670 | ebi-a-GCST90001670 | TRUE | G | 96311553 | 1.77E-08 | reported | 3440 | 0.08816 | rs185474383 |  |  |  |  |  |  |  |  |  |  |  |  |  |  |  |
| 0.5175 | 10 | igd | 0.0209 | T | \|\| id:ebi-a-GCST90001671 | ebi-a-GCST90001671 | TRUE | G | 96311553 | 4.93E-09 | reported | 3440 | 0.08824 | rs185474383 |  |  |  |  |  |  |  |  |  |  |  |  |  |  |  |
| 0.4664 | 10 | igd | 0.0208 | G | \|\| id:ebi-a-GCST90001485 | ebi-a-GCST90001485 | TRUE | A | 97958434 | 4.26E-08 | reported | 3437 | 0.08492 | rs74430332 |  |  |  |  |  |  |  |  |  |  |  |  |  |  |  |
| 72.54 | 4 | igd | 0.0207 | G | \|\| id:ebi-a-GCST90001698 | ebi-a-GCST90001698 | TRUE | T | 4128043 | 2.10E-08 | reported | 3408 | 12.91 | rs189420173 |  |  |  |  |  |  |  |  |  |  |  |  |  |  |  |
| 69.5 | 10 | igd | 0.0207 | T | \|\| id:ebi-a-GCST90001698 | ebi-a-GCST90001698 | TRUE | C | 21894518 | 3.30E-08 | reported | 3408 | 12.55 | rs74722793 |  |  |  |  |  |  |  |  |  |  |  |  |  |  |  |
| 77.03 | 10 | igd | 0.0207 | A | \|\| id:ebi-a-GCST90001698 | ebi-a-GCST90001698 | TRUE | G | 22411454 | 6.37E-09 | reported | 3408 | 13.23 | rs145021762 |  |  |  |  |  |  |  |  |  |  |  |  |  |  |  |
| -0.5664 | 12 | igd | 0.0207 | G | \|\| id:ebi-a-GCST90002074 | ebi-a-GCST90002074 | TRUE | T | 69316340 | 2.33E-09 | reported | 3112 | 0.09454 | rs74099496 |  |  |  |  |  |  |  |  |  |  |  |  |  |  |  |
| 0.6037 | 6 | igd | 0.0207 | T | \|\| id:ebi-a-GCST90002105 | ebi-a-GCST90002105 | TRUE | C | 30724198 | 7.22E-09 | reported | 2872 | 0.104 | rs77645489 |  |  |  |  |  |  |  |  |  |  |  |  |  |  |  |
| 0.4892 | 13 | igd | 0.0206 | G | \|\| id:ebi-a-GCST90001417 | ebi-a-GCST90001417 | TRUE | A | 50147180 | 3.03E-08 | reported | 3657 | 0.08811 | rs76477280 |  |  |  |  |  |  |  |  |  |  |  |  |  |  |  |
| 70.72 | 1 | igd | 0.0205 | T | \|\| id:ebi-a-GCST90001698 | ebi-a-GCST90001698 | TRUE | C | 100072380 | 2.30E-08 | reported | 3408 | 12.63 | rs4908331 |  |  |  |  |  |  |  |  |  |  |  |  |  |  |  |
| 0.7788 | 1 | igd | 0.0205 | G | \|\| id:ebi-a-GCST90001728 | ebi-a-GCST90001728 | TRUE | A | 206902437 | 9.18E-11 | reported | 1833 | 0.1195 | rs55712453 |  |  |  |  |  |  |  |  |  |  |  |  |  |  |  |
| -0.5067 | 6 | igd | 0.0205 | T | \|\| id:ebi-a-GCST90001765 | ebi-a-GCST90001765 | TRUE | C | 101424225 | 9.14E-09 | reported | 3655 | 0.08798 | rs138478119 |  |  |  |  |  |  |  |  |  |  |  |  |  |  |  |
| -0.5101 | 6 | igd | 0.0205 | T | \|\| id:ebi-a-GCST90001771 | ebi-a-GCST90001771 | TRUE | C | 101424225 | 7.88E-09 | reported | 3653 | 0.08819 | rs138478119 |  |  |  |  |  |  |  |  |  |  |  |  |  |  |  |
| 0.5111 | 10 | igd | 0.0204 | T | \|\| id:ebi-a-GCST90001489 | ebi-a-GCST90001489 | TRUE | C | 99903526 | 4.41E-08 | reported | 3405 | 0.09317 | rs72826245 |  |  |  |  |  |  |  |  |  |  |  |  |  |  |  |
| 0.5723 | 10 | igd | 0.0204 | T | \|\| id:ebi-a-GCST90001495 | ebi-a-GCST90001495 | TRUE | C | 99903526 | 1.28E-09 | reported | 3405 | 0.09403 | rs72826245 |  |  |  |  |  |  |  |  |  |  |  |  |  |  |  |
| 0.6048 | 10 | igd | 0.0204 | T | \|\| id:ebi-a-GCST90001660 | ebi-a-GCST90001660 | TRUE | C | 99903526 | 8.69E-11 | reported | 3408 | 0.09292 | rs72826245 |  |  |  |  |  |  |  |  |  |  |  |  |  |  |  |
| 0.4948 | 22 | igd | 0.0204 | A | \|\| id:ebi-a-GCST90001702 | ebi-a-GCST90001702 | TRUE | G | 43126236 | 2.75E-08 | reported | 3657 | 0.08885 | rs75737464 |  |  |  |  |  |  |  |  |  |  |  |  |  |  |  |
| -0.9902 | 22 | igd | 0.0204 | G | \|\| id:ebi-a-GCST90001703 | ebi-a-GCST90001703 | TRUE | A | 39997710 | 1.97E-29 | reported | 3657 | 0.08714 | rs713767 |  |  |  |  |  |  |  |  |  |  |  |  |  |  |  |
| 0.4895 | 22 | igd | 0.0204 | A | \|\| id:ebi-a-GCST90001703 | ebi-a-GCST90001703 | TRUE | G | 43126236 | 3.85E-08 | reported | 3657 | 0.08885 | rs75737464 |  |  |  |  |  |  |  |  |  |  |  |  |  |  |  |
| 0.5303 | 22 | igd | 0.0204 | A | \|\| id:ebi-a-GCST90001704 | ebi-a-GCST90001704 | TRUE | G | 43126236 | 2.45E-09 | reported | 3657 | 0.08867 | rs75737464 |  |  |  |  |  |  |  |  |  |  |  |  |  |  |  |
| -1.017 | 22 | igd | 0.0204 | G | \|\| id:ebi-a-GCST90001704 | ebi-a-GCST90001704 | TRUE | A | 39997710 | 4.70E-31 | reported | 3657 | 0.08694 | rs713767 |  |  |  |  |  |  |  |  |  |  |  |  |  |  |  |
| -0.9894 | 22 | igd | 0.0204 | G | \|\| id:ebi-a-GCST90001710 | ebi-a-GCST90001710 | TRUE | A | 39997710 | 1.52E-29 | reported | 3657 | 0.08689 | rs713767 |  |  |  |  |  |  |  |  |  |  |  |  |  |  |  |
| 0.5124 | 22 | igd | 0.0204 | A | \|\| id:ebi-a-GCST90001710 | ebi-a-GCST90001710 | TRUE | G | 43126236 | 7.83E-09 | reported | 3657 | 0.08857 | rs75737464 |  |  |  |  |  |  |  |  |  |  |  |  |  |  |  |
| 0.499 | 22 | igd | 0.0204 | A | \|\| id:ebi-a-GCST90001711 | ebi-a-GCST90001711 | TRUE | G | 43126236 | 1.78E-08 | reported | 3657 | 0.08839 | rs75737464 |  |  |  |  |  |  |  |  |  |  |  |  |  |  |  |
| -0.9893 | 22 | igd | 0.0204 | G | \|\| id:ebi-a-GCST90001711 | ebi-a-GCST90001711 | TRUE | A | 39997710 | 1.17E-29 | reported | 3657 | 0.0867 | rs713767 |  |  |  |  |  |  |  |  |  |  |  |  |  |  |  |
| -0.7678 | 12 | igd | 0.0204 | T | \|\| id:ebi-a-GCST90002073 | ebi-a-GCST90002073 | TRUE | C | 69953596 | 2.79E-15 | reported | 2850 | 0.09665 | rs532669 |  |  |  |  |  |  |  |  |  |  |  |  |  |  |  |
| -0.7306 | 12 | igd | 0.0204 | G | \|\| id:ebi-a-GCST90002073 | ebi-a-GCST90002073 | TRUE | T | 69316340 | 2.93E-13 | reported | 2850 | 0.09964 | rs74099496 |  |  |  |  |  |  |  |  |  |  |  |  |  |  |  |
| -0.5421 | 1 | igd | 0.0202 | T | \|\| id:ebi-a-GCST90001970 | ebi-a-GCST90001970 | TRUE | C | 161181134 | 2.10E-08 | reported | 2971 | 0.09648 | rs76265753 |  |  |  |  |  |  |  |  |  |  |  |  |  |  |  |
| 0.483 | 1 | igd | 0.0201 | T | \|\| id:ebi-a-GCST90001415 | ebi-a-GCST90001415 | TRUE | C | 8009985 | 3.86E-08 | reported | 3657 | 0.08768 | rs75766272 |  |  |  |  |  |  |  |  |  |  |  |  |  |  |  |
| -0.4865 | 1 | igd | 0.0201 | T | \|\| id:ebi-a-GCST90001417 | ebi-a-GCST90001417 | TRUE | C | 8009985 | 2.05E-08 | reported | 3657 | 0.08656 | rs75766272 |  |  |  |  |  |  |  |  |  |  |  |  |  |  |  |
| 0.5474 | 1 | igd | 0.0201 | A | \|\| id:ebi-a-GCST90001723 | ebi-a-GCST90001723 | TRUE | G | 206855483 | 9.28E-10 | reported | 3656 | 0.08918 | rs112417310 |  |  |  |  |  |  |  |  |  |  |  |  |  |  |  |
| 0.5738 | 1 | igd | 0.0201 | G | \|\| id:ebi-a-GCST90001724 | ebi-a-GCST90001724 | TRUE | A | 206902437 | 2.31E-10 | reported | 3656 | 0.09026 | rs55712453 |  |  |  |  |  |  |  |  |  |  |  |  |  |  |  |
| 0.5237 | 1 | igd | 0.0201 | A | \|\| id:ebi-a-GCST90001725 | ebi-a-GCST90001725 | TRUE | G | 206855483 | 3.80E-09 | reported | 3656 | 0.08866 | rs112417310 |  |  |  |  |  |  |  |  |  |  |  |  |  |  |  |
| 0.6504 | 1 | igd | 0.0201 | A | \|\| id:ebi-a-GCST90001729 | ebi-a-GCST90001729 | TRUE | G | 206855483 | 5.43E-13 | reported | 3657 | 0.08983 | rs112417310 |  |  |  |  |  |  |  |  |  |  |  |  |  |  |  |
| 0.5594 | 1 | igd | 0.0201 | A | \|\| id:ebi-a-GCST90001730 | ebi-a-GCST90001730 | TRUE | G | 206855483 | 3.65E-10 | reported | 3657 | 0.089 | rs112417310 |  |  |  |  |  |  |  |  |  |  |  |  |  |  |  |
| 0.5373 | 1 | igd | 0.0201 | A | \|\| id:ebi-a-GCST90001735 | ebi-a-GCST90001735 | TRUE | G | 206855483 | 1.70E-09 | reported | 3657 | 0.08896 | rs112417310 |  |  |  |  |  |  |  |  |  |  |  |  |  |  |  |
| 0.5116 | 1 | igd | 0.0201 | A | \|\| id:ebi-a-GCST90001736 | ebi-a-GCST90001736 | TRUE | G | 206855483 | 1.12E-08 | reported | 3657 | 0.08936 | rs112417310 |  |  |  |  |  |  |  |  |  |  |  |  |  |  |  |
| 0.4895 | 1 | igd | 0.0201 | A | \|\| id:ebi-a-GCST90001737 | ebi-a-GCST90001737 | TRUE | G | 206855483 | 3.62E-08 | reported | 3653 | 0.08867 | rs112417310 |  |  |  |  |  |  |  |  |  |  |  |  |  |  |  |
| 0.5735 | 1 | igd | 0.0201 | G | \|\| id:ebi-a-GCST90001738 | ebi-a-GCST90001738 | TRUE | A | 206902437 | 2.02E-10 | reported | 3656 | 0.08992 | rs55712453 |  |  |  |  |  |  |  |  |  |  |  |  |  |  |  |
| 0.4952 | 1 | igd | 0.0201 | A | \|\| id:ebi-a-GCST90001740 | ebi-a-GCST90001740 | TRUE | G | 206855483 | 3.38E-08 | reported | 3657 | 0.0895 | rs112417310 |  |  |  |  |  |  |  |  |  |  |  |  |  |  |  |
| 0.5195 | 1 | igd | 0.0201 | A | \|\| id:ebi-a-GCST90001741 | ebi-a-GCST90001741 | TRUE | G | 206855483 | 5.79E-09 | reported | 3658 | 0.08901 | rs112417310 |  |  |  |  |  |  |  |  |  |  |  |  |  |  |  |
| 0.5582 | 1 | igd | 0.0201 | G | \|\| id:ebi-a-GCST90001742 | ebi-a-GCST90001742 | TRUE | A | 206902437 | 8.34E-10 | reported | 3657 | 0.0907 | rs55712453 |  |  |  |  |  |  |  |  |  |  |  |  |  |  |  |
| 0.5404 | 10 | igd | 0.0201 | T | \|\| id:ebi-a-GCST90002029 | ebi-a-GCST90002029 | TRUE | C | 97535558 | 2.22E-08 | reported | 2915 | 0.09634 | rs182242903 |  |  |  |  |  |  |  |  |  |  |  |  |  |  |  |
| 0.5771 | 2 | igd | 0.0201 | C | \|\| id:ebi-a-GCST90002098 | ebi-a-GCST90002098 | TRUE | T | 38401167 | 4.47E-10 | reported | 2910 | 0.09222 | rs2432677 |  |  |  |  |  |  |  |  |  |  |  |  |  |  |  |
| 0.5315 | 2 | igd | 0.0201 | A | \|\| id:ebi-a-GCST90002098 | ebi-a-GCST90002098 | TRUE | C | 41607266 | 8.80E-09 | reported | 2910 | 0.09213 | rs141997643 |  |  |  |  |  |  |  |  |  |  |  |  |  |  |  |
| 0.5056 | 13 | igd | 0.02 | T | \|\| id:ebi-a-GCST90001442 | ebi-a-GCST90001442 | TRUE | C | 50212885 | 8.75E-09 | reported | 3656 | 0.08768 | rs73192651 |  |  |  |  |  |  |  |  |  |  |  |  |  |  |  |
| 0.6128 | 2 | igd | 0.02 | C | \|\| id:ebi-a-GCST90002102 | ebi-a-GCST90002102 | TRUE | T | 38401167 | 2.71E-11 | reported | 2919 | 0.09164 | rs2432677 |  |  |  |  |  |  |  |  |  |  |  |  |  |  |  |
| -0.497 | 3 | igd | 0.02 | A | \|\| id:ebi-a-GCST90002004 | ebi-a-GCST90002004 | TRUE | G | 47552103 | 1.31E-08 | reported | 3629 | 0.08722 | rs77923150 |  |  |  |  |  |  |  |  |  |  |  |  |  |  |  |
| -0.5143 | 10 | igd | 0.0199 | C | \|\| id:ebi-a-GCST90001484 | ebi-a-GCST90001484 | TRUE | G | 97533613 | 1.30E-08 | reported | 3437 | 0.09022 | rs144839174 |  |  |  |  |  |  |  |  |  |  |  |  |  |  |  |
| -0.5374 | 10 | igd | 0.0199 | A | \|\| id:ebi-a-GCST90001491 | ebi-a-GCST90001491 | TRUE | C | 97509429 | 4.97E-09 | reported | 3437 | 0.09165 | rs151177407 |  |  |  |  |  |  |  |  |  |  |  |  |  |  |  |
| -0.6175 | 10 | igd | 0.0199 | A | \|\| id:ebi-a-GCST90001497 | ebi-a-GCST90001497 | TRUE | C | 97509429 | 1.81E-11 | reported | 3437 | 0.09157 | rs151177407 |  |  |  |  |  |  |  |  |  |  |  |  |  |  |  |
| -0.5474 | 10 | igd | 0.0199 | A | \|\| id:ebi-a-GCST90001658 | ebi-a-GCST90001658 | TRUE | C | 97509429 | 6.58E-10 | reported | 3440 | 0.08839 | rs151177407 |  |  |  |  |  |  |  |  |  |  |  |  |  |  |  |
| -0.5411 | 10 | igd | 0.0198 | A | \|\| id:ebi-a-GCST90001489 | ebi-a-GCST90001489 | TRUE | C | 97509429 | 7.77E-09 | reported | 3405 | 0.09349 | rs151177407 |  |  |  |  |  |  |  |  |  |  |  |  |  |  |  |
| -0.5741 | 10 | igd | 0.0198 | A | \|\| id:ebi-a-GCST90001495 | ebi-a-GCST90001495 | TRUE | C | 97509429 | 1.15E-09 | reported | 3405 | 0.09404 | rs151177407 |  |  |  |  |  |  |  |  |  |  |  |  |  |  |  |
| 0.6138 | 1 | igd | 0.0197 | A | \|\| id:ebi-a-GCST90001729 | ebi-a-GCST90001729 | TRUE | G | 207018206 | 1.07E-11 | reported | 3657 | 0.09002 | rs144743804 |  |  |  |  |  |  |  |  |  |  |  |  |  |  |  |
| 0.5079 | 1 | igd | 0.0197 | A | \|\| id:ebi-a-GCST90001730 | ebi-a-GCST90001730 | TRUE | G | 207018206 | 1.34E-08 | reported | 3657 | 0.0892 | rs144743804 |  |  |  |  |  |  |  |  |  |  |  |  |  |  |  |
| 0.4919 | 1 | igd | 0.0197 | A | \|\| id:ebi-a-GCST90001738 | ebi-a-GCST90001738 | TRUE | G | 207018206 | 3.95E-08 | reported | 3656 | 0.08935 | rs144743804 |  |  |  |  |  |  |  |  |  |  |  |  |  |  |  |
| 0.539 | 1 | igd | 0.0197 | A | \|\| id:ebi-a-GCST90001742 | ebi-a-GCST90001742 | TRUE | G | 207018206 | 2.36E-09 | reported | 3657 | 0.09004 | rs144743804 |  |  |  |  |  |  |  |  |  |  |  |  |  |  |  |
| -0.5756 | 10 | igd | 0.0197 | A | \|\| id:ebi-a-GCST90002030 | ebi-a-GCST90002030 | TRUE | C | 97509429 | 7.47E-09 | reported | 2920 | 0.09929 | rs151177407 |  |  |  |  |  |  |  |  |  |  |  |  |  |  |  |
| 0.6644 | 6 | igd | 0.0197 | A | \|\| id:ebi-a-GCST90002105 | ebi-a-GCST90002105 | TRUE | G | 29819252 | 3.28E-10 | reported | 2872 | 0.1053 | rs144450403 |  |  |  |  |  |  |  |  |  |  |  |  |  |  |  |
| -0.499 | 3 | igd | 0.0197 | C | \|\| id:ebi-a-GCST90002004 | ebi-a-GCST90002004 | TRUE | G | 47054609 | 1.67E-08 | reported | 3629 | 0.08823 | rs114694813 |  |  |  |  |  |  |  |  |  |  |  |  |  |  |  |
| 0.4939 | 1 | igd | 0.0196 | T | \|\| id:ebi-a-GCST90001485 | ebi-a-GCST90001485 | TRUE | C | 198029657 | 2.52E-08 | reported | 3437 | 0.08843 | rs74230223 |  |  |  |  |  |  |  |  |  |  |  |  |  |  |  |
| 0.6028 | 1 | igd | 0.0196 | T | \|\| id:ebi-a-GCST90001509 | ebi-a-GCST90001509 | TRUE | C | 198026905 | 3.35E-11 | reported | 3437 | 0.09062 | rs77634072 |  |  |  |  |  |  |  |  |  |  |  |  |  |  |  |
| -0.6131 | 1 | igd | 0.0196 | G | \|\| id:ebi-a-GCST90001691 | ebi-a-GCST90001691 | TRUE | A | 198029422 | 1.46E-11 | reported | 3440 | 0.0905 | rs78231382 |  |  |  |  |  |  |  |  |  |  |  |  |  |  |  |
| 0.5549 | 2 | igd | 0.0196 | C | \|\| id:ebi-a-GCST90002098 | ebi-a-GCST90002098 | TRUE | A | 42177501 | 3.82E-09 | reported | 2910 | 0.09389 | rs116191705 |  |  |  |  |  |  |  |  |  |  |  |  |  |  |  |
| 0.5407 | 10 | igd | 0.0195 | G | \|\| id:ebi-a-GCST90001489 | ebi-a-GCST90001489 | TRUE | A | 104555688 | 1.92E-08 | reported | 3405 | 0.09601 | rs74371703 |  |  |  |  |  |  |  |  |  |  |  |  |  |  |  |
| 0.6353 | 10 | igd | 0.0195 | G | \|\| id:ebi-a-GCST90001495 | ebi-a-GCST90001495 | TRUE | A | 104555688 | 8.07E-11 | reported | 3405 | 0.09744 | rs74371703 |  |  |  |  |  |  |  |  |  |  |  |  |  |  |  |
| 0.5575 | 10 | igd | 0.0195 | G | \|\| id:ebi-a-GCST90001496 | ebi-a-GCST90001496 | TRUE | A | 104555688 | 8.31E-09 | reported | 3437 | 0.09651 | rs74371703 |  |  |  |  |  |  |  |  |  |  |  |  |  |  |  |
| 0.5264 | 10 | igd | 0.0195 | G | \|\| id:ebi-a-GCST90001497 | ebi-a-GCST90001497 | TRUE | A | 104555688 | 3.61E-08 | reported | 3437 | 0.09534 | rs74371703 |  |  |  |  |  |  |  |  |  |  |  |  |  |  |  |
| 0.5131 | 10 | igd | 0.0195 | G | \|\| id:ebi-a-GCST90001658 | ebi-a-GCST90001658 | TRUE | A | 104555688 | 2.87E-08 | reported | 3440 | 0.09225 | rs74371703 |  |  |  |  |  |  |  |  |  |  |  |  |  |  |  |
| -0.5107 | 10 | igd | 0.0195 | C | \|\| id:ebi-a-GCST90001660 | ebi-a-GCST90001660 | TRUE | A | 97997497 | 2.13E-08 | reported | 3408 | 0.09097 | rs76756973 |  |  |  |  |  |  |  |  |  |  |  |  |  |  |  |
| 0.6768 | 10 | igd | 0.0195 | G | \|\| id:ebi-a-GCST90001660 | ebi-a-GCST90001660 | TRUE | A | 104555688 | 2.67E-12 | reported | 3408 | 0.09641 | rs74371703 |  |  |  |  |  |  |  |  |  |  |  |  |  |  |  |
| 0.5485 | 10 | igd | 0.0195 | A | \|\| id:ebi-a-GCST90001670 | ebi-a-GCST90001670 | TRUE | G | 99843767 | 1.32E-09 | reported | 3440 | 0.09019 | rs117134709 |  |  |  |  |  |  |  |  |  |  |  |  |  |  |  |
| 0.5678 | 10 | igd | 0.0195 | A | \|\| id:ebi-a-GCST90001671 | ebi-a-GCST90001671 | TRUE | G | 99843767 | 3.41E-10 | reported | 3440 | 0.09017 | rs117134709 |  |  |  |  |  |  |  |  |  |  |  |  |  |  |  |
| -0.5166 | 10 | igd | 0.0195 | C | \|\| id:ebi-a-GCST90001672 | ebi-a-GCST90001672 | TRUE | A | 97997497 | 2.02E-08 | reported | 3408 | 0.09186 | rs76756973 |  |  |  |  |  |  |  |  |  |  |  |  |  |  |  |
| 0.5472 | 10 | igd | 0.0195 | A | \|\| id:ebi-a-GCST90001672 | ebi-a-GCST90001672 | TRUE | G | 99843767 | 2.38E-09 | reported | 3408 | 0.09142 | rs117134709 |  |  |  |  |  |  |  |  |  |  |  |  |  |  |  |
| -0.6259 | 17 | igd | 0.0195 | T | \|\| id:ebi-a-GCST90001926 | ebi-a-GCST90001926 | TRUE | C | 56358429 | 6.18E-10 | reported | 2920 | 0.1008 | rs34097845 |  |  |  |  |  |  |  |  |  |  |  |  |  |  |  |
| -0.8118 | 12 | igd | 0.0195 | C | \|\| id:ebi-a-GCST90002073 | ebi-a-GCST90002073 | TRUE | G | 70957193 | 4.50E-16 | reported | 2850 | 0.09934 | rs2717442 |  |  |  |  |  |  |  |  |  |  |  |  |  |  |  |
| 0.5112 | 1 | igd | 0.0194 | T | \|\| id:ebi-a-GCST90001483 | ebi-a-GCST90001483 | TRUE | C | 198029657 | 1.27E-08 | reported | 3405 | 0.0896 | rs74230223 |  |  |  |  |  |  |  |  |  |  |  |  |  |  |  |
| 0.6375 | 1 | igd | 0.0194 | T | \|\| id:ebi-a-GCST90001507 | ebi-a-GCST90001507 | TRUE | C | 198026905 | 6.39E-12 | reported | 3405 | 0.09246 | rs77634072 |  |  |  |  |  |  |  |  |  |  |  |  |  |  |  |
| -0.8532 | 22 | igd | 0.0194 | A | \|\| id:ebi-a-GCST90001702 | ebi-a-GCST90001702 | TRUE | G | 40305664 | 1.17E-20 | reported | 3657 | 0.091 | rs116996173 |  |  |  |  |  |  |  |  |  |  |  |  |  |  |  |
| -0.5345 | 22 | igd | 0.0194 | G | \|\| id:ebi-a-GCST90001702 | ebi-a-GCST90001702 | TRUE | A | 44355693 | 4.19E-09 | reported | 3657 | 0.09074 | rs76015644 |  |  |  |  |  |  |  |  |  |  |  |  |  |  |  |
| -0.856 | 22 | igd | 0.0194 | A | \|\| id:ebi-a-GCST90001703 | ebi-a-GCST90001703 | TRUE | G | 40305664 | 8.76E-21 | reported | 3657 | 0.09099 | rs116996173 |  |  |  |  |  |  |  |  |  |  |  |  |  |  |  |
| -0.5506 | 22 | igd | 0.0194 | G | \|\| id:ebi-a-GCST90001703 | ebi-a-GCST90001703 | TRUE | A | 44355693 | 1.41E-09 | reported | 3657 | 0.09071 | rs76015644 |  |  |  |  |  |  |  |  |  |  |  |  |  |  |  |
| -0.8376 | 22 | igd | 0.0194 | A | \|\| id:ebi-a-GCST90001704 | ebi-a-GCST90001704 | TRUE | G | 40305664 | 5.24E-20 | reported | 3657 | 0.09093 | rs116996173 |  |  |  |  |  |  |  |  |  |  |  |  |  |  |  |
| -0.8121 | 22 | igd | 0.0194 | A | \|\| id:ebi-a-GCST90001710 | ebi-a-GCST90001710 | TRUE | G | 40305664 | 5.95E-19 | reported | 3657 | 0.09083 | rs116996173 |  |  |  |  |  |  |  |  |  |  |  |  |  |  |  |
| -0.5137 | 22 | igd | 0.0194 | G | \|\| id:ebi-a-GCST90001710 | ebi-a-GCST90001710 | TRUE | A | 44355693 | 1.48E-08 | reported | 3657 | 0.0905 | rs76015644 |  |  |  |  |  |  |  |  |  |  |  |  |  |  |  |
| -0.5039 | 22 | igd | 0.0194 | G | \|\| id:ebi-a-GCST90001711 | ebi-a-GCST90001711 | TRUE | A | 44355693 | 2.61E-08 | reported | 3657 | 0.09034 | rs76015644 |  |  |  |  |  |  |  |  |  |  |  |  |  |  |  |
| -0.8282 | 22 | igd | 0.0194 | A | \|\| id:ebi-a-GCST90001711 | ebi-a-GCST90001711 | TRUE | G | 40305664 | 1.01E-19 | reported | 3657 | 0.09061 | rs116996173 |  |  |  |  |  |  |  |  |  |  |  |  |  |  |  |
| -0.7826 | 4 | igd | 0.0194 | A | \|\| id:ebi-a-GCST90001811 | ebi-a-GCST90001811 | TRUE | G | 15915069 | 4.58E-18 | reported | 3656 | 0.08987 | rs76112278 |  |  |  |  |  |  |  |  |  |  |  |  |  |  |  |
| -0.9249 | 4 | igd | 0.0194 | A | \|\| id:ebi-a-GCST90001815 | ebi-a-GCST90001815 | TRUE | G | 15915069 | 5.61E-26 | reported | 3657 | 0.08708 | rs76112278 |  |  |  |  |  |  |  |  |  |  |  |  |  |  |  |
| -0.6509 | 12 | igd | 0.0194 | C | \|\| id:ebi-a-GCST90002074 | ebi-a-GCST90002074 | TRUE | G | 70957193 | 1.55E-11 | reported | 3112 | 0.09617 | rs2717442 |  |  |  |  |  |  |  |  |  |  |  |  |  |  |  |
| 0.5635 | 20 | igd | 0.0193 | A | \|\| id:ebi-a-GCST90001980 | ebi-a-GCST90001980 | TRUE | G | 44494860 | 1.16E-10 | reported | 3629 | 0.08718 | rs113927526 |  |  |  |  |  |  |  |  |  |  |  |  |  |  |  |
| 0.6239 | 20 | igd | 0.0193 | A | \|\| id:ebi-a-GCST90001981 | ebi-a-GCST90001981 | TRUE | T | 44484097 | 8.51E-13 | reported | 3618 | 0.08691 | rs113775752 |  |  |  |  |  |  |  |  |  |  |  |  |  |  |  |
| 0.6774 | 20 | igd | 0.0193 | A | \|\| id:ebi-a-GCST90001985 | ebi-a-GCST90001985 | TRUE | G | 44494860 | 1.01E-14 | reported | 3629 | 0.08718 | rs113927526 |  |  |  |  |  |  |  |  |  |  |  |  |  |  |  |
| 0.6641 | 20 | igd | 0.0193 | A | \|\| id:ebi-a-GCST90001989 | ebi-a-GCST90001989 | TRUE | T | 44484097 | 2.10E-14 | reported | 3621 | 0.08652 | rs113775752 |  |  |  |  |  |  |  |  |  |  |  |  |  |  |  |
| 0.4024 | 6 | igd | 0.0192 | G | \|\| id:ebi-a-GCST90001688 | ebi-a-GCST90001688 | TRUE | A | 31360602 | 3.97E-09 | reported | 3440 | 0.06819 | rs116562707 |  |  |  |  |  |  |  |  |  |  |  |  |  |  |  |
| 0.8759 | 10 | igd | 0.019 | A | \|\| id:ebi-a-GCST90001670 | ebi-a-GCST90001670 | TRUE | G | 96773382 | 6.64E-20 | reported | 3440 | 0.09531 | rs117458836 |  |  |  |  |  |  |  |  |  |  |  |  |  |  |  |
| 0.8279 | 10 | igd | 0.019 | A | \|\| id:ebi-a-GCST90001671 | ebi-a-GCST90001671 | TRUE | G | 96773382 | 7.37E-18 | reported | 3440 | 0.09564 | rs117458836 |  |  |  |  |  |  |  |  |  |  |  |  |  |  |  |
| 0.5554 | 10 | igd | 0.0189 | C | \|\| id:ebi-a-GCST90001489 | ebi-a-GCST90001489 | TRUE | T | 97380342 | 1.36E-08 | reported | 3405 | 0.09756 | rs111722849 |  |  |  |  |  |  |  |  |  |  |  |  |  |  |  |
| 0.6158 | 10 | igd | 0.0189 | C | \|\| id:ebi-a-GCST90001495 | ebi-a-GCST90001495 | TRUE | T | 97380342 | 4.28E-10 | reported | 3405 | 0.09834 | rs111722849 |  |  |  |  |  |  |  |  |  |  |  |  |  |  |  |
| 0.9116 | 10 | igd | 0.0189 | A | \|\| id:ebi-a-GCST90001672 | ebi-a-GCST90001672 | TRUE | G | 96773382 | 1.03E-20 | reported | 3408 | 0.09705 | rs117458836 |  |  |  |  |  |  |  |  |  |  |  |  |  |  |  |
| 76.2 | 18 | igd | 0.0189 | G | \|\| id:ebi-a-GCST90001698 | ebi-a-GCST90001698 | TRUE | A | 34022780 | 2.34E-09 | reported | 3408 | 12.72 | rs116938017 |  |  |  |  |  |  |  |  |  |  |  |  |  |  |  |
| 80.23 | 21 | igd | 0.0189 | A | \|\| id:ebi-a-GCST90001698 | ebi-a-GCST90001698 | TRUE | G | 35824714 | 3.39E-10 | reported | 3408 | 12.74 | rs7276763 |  |  |  |  |  |  |  |  |  |  |  |  |  |  |  |
| 0.5535 | 10 | igd | 0.0188 | C | \|\| id:ebi-a-GCST90001491 | ebi-a-GCST90001491 | TRUE | T | 97380342 | 1.01E-08 | reported | 3437 | 0.09638 | rs111722849 |  |  |  |  |  |  |  |  |  |  |  |  |  |  |  |
| 0.6416 | 10 | igd | 0.0188 | C | \|\| id:ebi-a-GCST90001496 | ebi-a-GCST90001496 | TRUE | T | 97380342 | 5.47E-11 | reported | 3437 | 0.09753 | rs111722849 |  |  |  |  |  |  |  |  |  |  |  |  |  |  |  |
| 0.6107 | 10 | igd | 0.0188 | C | \|\| id:ebi-a-GCST90001497 | ebi-a-GCST90001497 | TRUE | T | 97380342 | 2.63E-10 | reported | 3437 | 0.09635 | rs111722849 |  |  |  |  |  |  |  |  |  |  |  |  |  |  |  |
| -0.5872 | 10 | igd | 0.0188 | T | \|\| id:ebi-a-GCST90002032 | ebi-a-GCST90002032 | TRUE | C | 100309478 | 4.17E-08 | reported | 2920 | 0.1068 | rs117207419 |  |  |  |  |  |  |  |  |  |  |  |  |  |  |  |
| 0.6736 | 6 | igd | 0.0188 | A | \|\| id:ebi-a-GCST90002105 | ebi-a-GCST90002105 | TRUE | T | 30628957 | 6.21E-11 | reported | 2872 | 0.1026 | rs74917683 |  |  |  |  |  |  |  |  |  |  |  |  |  |  |  |
| 0.8078 | 6 | igd | 0.0188 | T | \|\| id:ebi-a-GCST90002105 | ebi-a-GCST90002105 | TRUE | C | 33056962 | 7.92E-16 | reported | 2872 | 0.0997 | rs3097649 |  |  |  |  |  |  |  |  |  |  |  |  |  |  |  |
| -0.5608 | 19 | igd | 0.01874 | C | \|\| id:prot-a-439 | prot-a-439 | TRUE | T | 51809973 | 2.09E-09 | reported | 3301 | 0.0936 | rs73062986 |  |  |  |  |  |  |  |  |  |  |  |  |  |  |  |
| 0.6927 | 10 | igd | 0.0187 | A | \|\| id:ebi-a-GCST90001892 | ebi-a-GCST90001892 | TRUE | G | 96773382 | 3.45E-11 | reported | 2920 | 0.1041 | rs117458836 |  |  |  |  |  |  |  |  |  |  |  |  |  |  |  |
| -0.8416 | 10 | igd | 0.0187 | A | \|\| id:ebi-a-GCST90001961 | ebi-a-GCST90001961 | TRUE | G | 96773382 | 1.46E-15 | reported | 2920 | 0.1049 | rs117458836 |  |  |  |  |  |  |  |  |  |  |  |  |  |  |  |
| 0.632 | 10 | igd | 0.0187 | C | \|\| id:ebi-a-GCST90002030 | ebi-a-GCST90002030 | TRUE | T | 97914511 | 3.87E-10 | reported | 2920 | 0.1006 | rs75042907 |  |  |  |  |  |  |  |  |  |  |  |  |  |  |  |
| 0.5792 | 10 | igd | 0.0187 | C | \|\| id:ebi-a-GCST90002032 | ebi-a-GCST90002032 | TRUE | T | 97914511 | 2.34E-08 | reported | 2920 | 0.1034 | rs75042907 |  |  |  |  |  |  |  |  |  |  |  |  |  |  |  |
| 0.8979 | 10 | igd | 0.0187 | A | \|\| id:ebi-a-GCST90002061 | ebi-a-GCST90002061 | TRUE | G | 96773382 | 2.34E-17 | reported | 2920 | 0.1053 | rs117458836 |  |  |  |  |  |  |  |  |  |  |  |  |  |  |  |
| 84.59 | 4 | igd | 0.0185 | G | \|\| id:ebi-a-GCST90001698 | ebi-a-GCST90001698 | TRUE | A | 32690871 | 5.08E-10 | reported | 3408 | 13.57 | rs13104493 |  |  |  |  |  |  |  |  |  |  |  |  |  |  |  |
| 0.5546 | 1 | igd | 0.0185 | G | \|\| id:ebi-a-GCST90001724 | ebi-a-GCST90001724 | TRUE | A | 207276336 | 1.92E-09 | reported | 3656 | 0.09212 | rs113812519 |  |  |  |  |  |  |  |  |  |  |  |  |  |  |  |
| 0.5314 | 1 | igd | 0.0185 | G | \|\| id:ebi-a-GCST90001725 | ebi-a-GCST90001725 | TRUE | A | 207276336 | 6.42E-09 | reported | 3656 | 0.09132 | rs113812519 |  |  |  |  |  |  |  |  |  |  |  |  |  |  |  |
| 0.6788 | 1 | igd | 0.0185 | G | \|\| id:ebi-a-GCST90001729 | ebi-a-GCST90001729 | TRUE | A | 207276336 | 2.55E-13 | reported | 3657 | 0.09244 | rs113812519 |  |  |  |  |  |  |  |  |  |  |  |  |  |  |  |
| 0.5583 | 1 | igd | 0.0185 | G | \|\| id:ebi-a-GCST90001730 | ebi-a-GCST90001730 | TRUE | A | 207276336 | 1.22E-09 | reported | 3657 | 0.09163 | rs113812519 |  |  |  |  |  |  |  |  |  |  |  |  |  |  |  |
| 0.5404 | 1 | igd | 0.0185 | G | \|\| id:ebi-a-GCST90001738 | ebi-a-GCST90001738 | TRUE | A | 207276336 | 4.29E-09 | reported | 3656 | 0.0918 | rs113812519 |  |  |  |  |  |  |  |  |  |  |  |  |  |  |  |
| 0.5095 | 1 | igd | 0.0185 | G | \|\| id:ebi-a-GCST90001741 | ebi-a-GCST90001741 | TRUE | A | 207276336 | 2.91E-08 | reported | 3658 | 0.09165 | rs113812519 |  |  |  |  |  |  |  |  |  |  |  |  |  |  |  |
| 0.5863 | 1 | igd | 0.0185 | G | \|\| id:ebi-a-GCST90001742 | ebi-a-GCST90001742 | TRUE | A | 207276336 | 2.61E-10 | reported | 3657 | 0.09251 | rs113812519 |  |  |  |  |  |  |  |  |  |  |  |  |  |  |  |
| -0.7043 | 1 | igd | 0.0185 | A | \|\| id:ebi-a-GCST90001858 | ebi-a-GCST90001858 | TRUE | G | 167647194 | 6.75E-12 | reported | 2919 | 0.1022 | rs147145243 |  |  |  |  |  |  |  |  |  |  |  |  |  |  |  |
| -0.6054 | 1 | igd | 0.0185 | A | \|\| id:ebi-a-GCST90001860 | ebi-a-GCST90001860 | TRUE | G | 167647194 | 3.99E-09 | reported | 2919 | 0.1026 | rs147145243 |  |  |  |  |  |  |  |  |  |  |  |  |  |  |  |
| -0.5775 | 1 | igd | 0.0185 | A | \|\| id:ebi-a-GCST90001861 | ebi-a-GCST90001861 | TRUE | G | 167647194 | 1.30E-08 | reported | 2919 | 0.1013 | rs147145243 |  |  |  |  |  |  |  |  |  |  |  |  |  |  |  |
| -0.5645 | 1 | igd | 0.0185 | A | \|\| id:ebi-a-GCST90001863 | ebi-a-GCST90001863 | TRUE | G | 167647194 | 1.99E-08 | reported | 2919 | 0.1003 | rs147145243 |  |  |  |  |  |  |  |  |  |  |  |  |  |  |  |
| -0.7752 | 1 | igd | 0.0185 | A | \|\| id:ebi-a-GCST90001864 | ebi-a-GCST90001864 | TRUE | G | 167647194 | 3.37E-14 | reported | 2919 | 0.1017 | rs147145243 |  |  |  |  |  |  |  |  |  |  |  |  |  |  |  |
| -0.602 | 1 | igd | 0.0184 | A | \|\| id:ebi-a-GCST90001845 | ebi-a-GCST90001845 | TRUE | G | 167647194 | 4.55E-09 | reported | 2911 | 0.1024 | rs147145243 |  |  |  |  |  |  |  |  |  |  |  |  |  |  |  |
| 0.6017 | 10 | igd | 0.0183 | A | \|\| id:ebi-a-GCST90001671 | ebi-a-GCST90001671 | TRUE | G | 96084585 | 2.73E-10 | reported | 3440 | 0.09502 | rs139126232 |  |  |  |  |  |  |  |  |  |  |  |  |  |  |  |
| 0.547 | 10 | igd | 0.0183 | A | \|\| id:ebi-a-GCST90001672 | ebi-a-GCST90001672 | TRUE | G | 96084585 | 1.57E-08 | reported | 3408 | 0.09652 | rs139126232 |  |  |  |  |  |  |  |  |  |  |  |  |  |  |  |
| 0.5267 | 10 | igd | 0.018 | C | \|\| id:ebi-a-GCST90001484 | ebi-a-GCST90001484 | TRUE | T | 97914511 | 1.45E-08 | reported | 3437 | 0.09273 | rs75042907 |  |  |  |  |  |  |  |  |  |  |  |  |  |  |  |
| 0.5521 | 10 | igd | 0.018 | C | \|\| id:ebi-a-GCST90001491 | ebi-a-GCST90001491 | TRUE | T | 97914511 | 4.49E-09 | reported | 3437 | 0.09389 | rs75042907 |  |  |  |  |  |  |  |  |  |  |  |  |  |  |  |
| 0.5651 | 10 | igd | 0.018 | C | \|\| id:ebi-a-GCST90001496 | ebi-a-GCST90001496 | TRUE | T | 97914511 | 3.15E-09 | reported | 3437 | 0.09515 | rs75042907 |  |  |  |  |  |  |  |  |  |  |  |  |  |  |  |
| 0.5877 | 10 | igd | 0.018 | C | \|\| id:ebi-a-GCST90001497 | ebi-a-GCST90001497 | TRUE | T | 97914511 | 4.34E-10 | reported | 3437 | 0.0939 | rs75042907 |  |  |  |  |  |  |  |  |  |  |  |  |  |  |  |
| 0.4984 | 10 | igd | 0.018 | C | \|\| id:ebi-a-GCST90001658 | ebi-a-GCST90001658 | TRUE | T | 97914511 | 4.05E-08 | reported | 3440 | 0.09059 | rs75042907 |  |  |  |  |  |  |  |  |  |  |  |  |  |  |  |
| 0.5563 | 10 | igd | 0.018 | C | \|\| id:ebi-a-GCST90001659 | ebi-a-GCST90001659 | TRUE | T | 97914511 | 8.82E-10 | reported | 3440 | 0.09051 | rs75042907 |  |  |  |  |  |  |  |  |  |  |  |  |  |  |  |
| 0.5703 | 10 | igd | 0.018 | C | \|\| id:ebi-a-GCST90001670 | ebi-a-GCST90001670 | TRUE | T | 97914511 | 1.69E-09 | reported | 3440 | 0.09439 | rs75042907 |  |  |  |  |  |  |  |  |  |  |  |  |  |  |  |
| 0.5552 | 20 | igd | 0.018 | T | \|\| id:ebi-a-GCST90001798 | ebi-a-GCST90001798 | TRUE | C | 44647851 | 4.74E-09 | reported | 3657 | 0.09458 | rs144858460 |  |  |  |  |  |  |  |  |  |  |  |  |  |  |  |
| -0.6092 | 1 | igd | 0.018 | A | \|\| id:ebi-a-GCST90001854 | ebi-a-GCST90001854 | TRUE | G | 166939596 | 3.59E-09 | reported | 2919 | 0.1029 | rs150822649 |  |  |  |  |  |  |  |  |  |  |  |  |  |  |  |
| -0.5835 | 1 | igd | 0.018 | A | \|\| id:ebi-a-GCST90001860 | ebi-a-GCST90001860 | TRUE | G | 166939596 | 2.33E-08 | reported | 2919 | 0.1042 | rs150822649 |  |  |  |  |  |  |  |  |  |  |  |  |  |  |  |
| 0.5613 | 10 | igd | 0.0179 | C | \|\| id:ebi-a-GCST90001495 | ebi-a-GCST90001495 | TRUE | T | 97914511 | 6.36E-09 | reported | 3405 | 0.09642 | rs75042907 |  |  |  |  |  |  |  |  |  |  |  |  |  |  |  |
| 0.6168 | 10 | igd | 0.0179 | C | \|\| id:ebi-a-GCST90001672 | ebi-a-GCST90001672 | TRUE | T | 97914511 | 1.46E-10 | reported | 3408 | 0.09594 | rs75042907 |  |  |  |  |  |  |  |  |  |  |  |  |  |  |  |
| 0.8802 | 6 | igd | 0.0179 | C | \|\| id:ebi-a-GCST90002105 | ebi-a-GCST90002105 | TRUE | T | 32786882 | 2.95E-17 | reported | 2872 | 0.1035 | rs3763355 |  |  |  |  |  |  |  |  |  |  |  |  |  |  |  |
| 0.5416 | 1 | igd | 0.0178 | G | \|\| id:ebi-a-GCST90001981 | ebi-a-GCST90001981 | TRUE | C | 161450098 | 8.48E-09 | reported | 3618 | 0.09383 | rs140797462 |  |  |  |  |  |  |  |  |  |  |  |  |  |  |  |
| 75.73 | 15 | igd | 0.0176 | A | \|\| id:ebi-a-GCST90001698 | ebi-a-GCST90001698 | TRUE | C | 56814907 | 3.46E-08 | reported | 3408 | 13.7 | rs113370154 |  |  |  |  |  |  |  |  |  |  |  |  |  |  |  |
| -0.6561 | 9 | igd | 0.0175 | A | \|\| id:ebi-a-GCST90001527 | ebi-a-GCST90001527 | TRUE | G | 107953420 | 3.83E-08 | reported | 1858 | 0.1188 | rs185048341 |  |  |  |  |  |  |  |  |  |  |  |  |  |  |  |
| 0.5045 | 1 | igd | 0.0175 | C | \|\| id:ebi-a-GCST90001666 | ebi-a-GCST90001666 | TRUE | T | 199193075 | 2.60E-09 | reported | 3408 | 0.08449 | rs116393041 |  |  |  |  |  |  |  |  |  |  |  |  |  |  |  |
| 55.39 | 4 | igd | 0.0175 | C | \|\| id:ebi-a-GCST90001695 | ebi-a-GCST90001695 | TRUE | A | 45244488 | 2.87E-08 | reported | 3408 | 9.959 | rs148799115 |  |  |  |  |  |  |  |  |  |  |  |  |  |  |  |
| -0.8453 | 22 | igd | 0.0175 | C | \|\| id:ebi-a-GCST90001702 | ebi-a-GCST90001702 | TRUE | G | 44601093 | 2.29E-18 | reported | 3657 | 0.09618 | rs79328498 |  |  |  |  |  |  |  |  |  |  |  |  |  |  |  |
| -0.8626 | 22 | igd | 0.0175 | C | \|\| id:ebi-a-GCST90001703 | ebi-a-GCST90001703 | TRUE | G | 44601093 | 4.49E-19 | reported | 3657 | 0.09612 | rs79328498 |  |  |  |  |  |  |  |  |  |  |  |  |  |  |  |
| -0.8866 | 22 | igd | 0.0175 | C | \|\| id:ebi-a-GCST90001704 | ebi-a-GCST90001704 | TRUE | G | 44601093 | 4.03E-20 | reported | 3657 | 0.09594 | rs79328498 |  |  |  |  |  |  |  |  |  |  |  |  |  |  |  |
| -0.8444 | 22 | igd | 0.0175 | C | \|\| id:ebi-a-GCST90001710 | ebi-a-GCST90001710 | TRUE | G | 44601093 | 1.99E-18 | reported | 3657 | 0.0959 | rs79328498 |  |  |  |  |  |  |  |  |  |  |  |  |  |  |  |
| -0.8509 | 22 | igd | 0.0175 | C | \|\| id:ebi-a-GCST90001711 | ebi-a-GCST90001711 | TRUE | G | 44601093 | 9.07E-19 | reported | 3657 | 0.09566 | rs79328498 |  |  |  |  |  |  |  |  |  |  |  |  |  |  |  |
| -0.6236 | 1 | igd | 0.0175 | A | \|\| id:ebi-a-GCST90001864 | ebi-a-GCST90001864 | TRUE | G | 168308120 | 2.07E-09 | reported | 2919 | 0.1037 | rs72701770 |  |  |  |  |  |  |  |  |  |  |  |  |  |  |  |
| 0.5503 | 20 | igd | 0.0175 | G | \|\| id:ebi-a-GCST90001980 | ebi-a-GCST90001980 | TRUE | A | 45333273 | 1.29E-08 | reported | 3629 | 0.09653 | rs117295081 |  |  |  |  |  |  |  |  |  |  |  |  |  |  |  |
| 0.5419 | 1 | igd | 0.0173 | C | \|\| id:ebi-a-GCST90001664 | ebi-a-GCST90001664 | TRUE | T | 199193075 | 1.08E-09 | reported | 3440 | 0.08863 | rs116393041 |  |  |  |  |  |  |  |  |  |  |  |  |  |  |  |
| 0.5638 | 10 | igd | 0.0173 | T | \|\| id:ebi-a-GCST90001670 | ebi-a-GCST90001670 | TRUE | C | 95374050 | 9.00E-09 | reported | 3440 | 0.09784 | rs150985132 |  |  |  |  |  |  |  |  |  |  |  |  |  |  |  |
| 0.5426 | 10 | igd | 0.0173 | T | \|\| id:ebi-a-GCST90001671 | ebi-a-GCST90001671 | TRUE | C | 95374050 | 3.36E-08 | reported | 3440 | 0.09805 | rs150985132 |  |  |  |  |  |  |  |  |  |  |  |  |  |  |  |
| 95.49 | 11 | igd | 0.0173 | T | \|\| id:ebi-a-GCST90001698 | ebi-a-GCST90001698 | TRUE | C | 12065087 | 3.28E-12 | reported | 3408 | 13.66 | rs1106305 |  |  |  |  |  |  |  |  |  |  |  |  |  |  |  |
| 82 | 12 | igd | 0.0173 | T | \|\| id:ebi-a-GCST90001698 | ebi-a-GCST90001698 | TRUE | C | 106241673 | 4.67E-09 | reported | 3408 | 13.96 | rs11112760 |  |  |  |  |  |  |  |  |  |  |  |  |  |  |  |
| -0.5824 | 1 | igd | 0.0173 | A | \|\| id:ebi-a-GCST90001860 | ebi-a-GCST90001860 | TRUE | G | 168306279 | 3.24E-08 | reported | 2919 | 0.1051 | rs114672530 |  |  |  |  |  |  |  |  |  |  |  |  |  |  |  |
| 0.6109 | 10 | igd | 0.0172 | A | \|\| id:ebi-a-GCST90001484 | ebi-a-GCST90001484 | TRUE | T | 95495162 | 3.33E-10 | reported | 3437 | 0.09695 | rs57091493 |  |  |  |  |  |  |  |  |  |  |  |  |  |  |  |
| 0.5513 | 10 | igd | 0.0172 | A | \|\| id:ebi-a-GCST90001496 | ebi-a-GCST90001496 | TRUE | T | 95495162 | 3.84E-08 | reported | 3437 | 0.1001 | rs57091493 |  |  |  |  |  |  |  |  |  |  |  |  |  |  |  |
| 0.563 | 10 | igd | 0.0172 | A | \|\| id:ebi-a-GCST90001497 | ebi-a-GCST90001497 | TRUE | T | 95495162 | 1.30E-08 | reported | 3437 | 0.09877 | rs57091493 |  |  |  |  |  |  |  |  |  |  |  |  |  |  |  |
| 0.6056 | 10 | igd | 0.0172 | A | \|\| id:ebi-a-GCST90001658 | ebi-a-GCST90001658 | TRUE | T | 95495162 | 2.28E-10 | reported | 3440 | 0.09521 | rs57091493 |  |  |  |  |  |  |  |  |  |  |  |  |  |  |  |
| 0.6096 | 10 | igd | 0.0172 | A | \|\| id:ebi-a-GCST90001659 | ebi-a-GCST90001659 | TRUE | T | 95495162 | 1.73E-10 | reported | 3440 | 0.09521 | rs57091493 |  |  |  |  |  |  |  |  |  |  |  |  |  |  |  |
| -0.6769 | 22 | igd | 0.0172 | T | \|\| id:ebi-a-GCST90001702 | ebi-a-GCST90001702 | TRUE | G | 41553917 | 5.37E-12 | reported | 3657 | 0.09784 | rs117018441 |  |  |  |  |  |  |  |  |  |  |  |  |  |  |  |
| -0.671 | 22 | igd | 0.0172 | T | \|\| id:ebi-a-GCST90001703 | ebi-a-GCST90001703 | TRUE | G | 41553917 | 8.19E-12 | reported | 3657 | 0.09785 | rs117018441 |  |  |  |  |  |  |  |  |  |  |  |  |  |  |  |
| -0.5432 | 22 | igd | 0.0172 | T | \|\| id:ebi-a-GCST90001704 | ebi-a-GCST90001704 | TRUE | G | 41553917 | 3.13E-08 | reported | 3657 | 0.09794 | rs117018441 |  |  |  |  |  |  |  |  |  |  |  |  |  |  |  |
| -0.5945 | 22 | igd | 0.0172 | T | \|\| id:ebi-a-GCST90001710 | ebi-a-GCST90001710 | TRUE | G | 41553917 | 1.29E-09 | reported | 3657 | 0.0977 | rs117018441 |  |  |  |  |  |  |  |  |  |  |  |  |  |  |  |
| -0.5757 | 22 | igd | 0.0172 | T | \|\| id:ebi-a-GCST90001711 | ebi-a-GCST90001711 | TRUE | G | 41553917 | 3.88E-09 | reported | 3657 | 0.09753 | rs117018441 |  |  |  |  |  |  |  |  |  |  |  |  |  |  |  |
| -0.8234 | 12 | igd | 0.0172 | G | \|\| id:ebi-a-GCST90002073 | ebi-a-GCST90002073 | TRUE | A | 69247557 | 7.27E-15 | reported | 2850 | 0.1053 | rs1152928 |  |  |  |  |  |  |  |  |  |  |  |  |  |  |  |
| -0.5435 | 22 | igd | 0.0171 | G | \|\| id:ebi-a-GCST90001702 | ebi-a-GCST90001702 | TRUE | A | 38146976 | 3.15E-08 | reported | 3657 | 0.09801 | rs117723114 |  |  |  |  |  |  |  |  |  |  |  |  |  |  |  |
| -0.5359 | 22 | igd | 0.0171 | G | \|\| id:ebi-a-GCST90001703 | ebi-a-GCST90001703 | TRUE | A | 38146976 | 4.84E-08 | reported | 3657 | 0.09799 | rs117723114 |  |  |  |  |  |  |  |  |  |  |  |  |  |  |  |
| -0.545 | 22 | igd | 0.0171 | G | \|\| id:ebi-a-GCST90001704 | ebi-a-GCST90001704 | TRUE | A | 38146976 | 2.72E-08 | reported | 3657 | 0.09784 | rs117723114 |  |  |  |  |  |  |  |  |  |  |  |  |  |  |  |
| 0.5868 | 10 | igd | 0.017 | T | \|\| id:ebi-a-GCST90001658 | ebi-a-GCST90001658 | TRUE | G | 96611102 | 1.87E-09 | reported | 3440 | 0.09739 | rs141019273 |  |  |  |  |  |  |  |  |  |  |  |  |  |  |  |
| 0.5706 | 10 | igd | 0.017 | T | \|\| id:ebi-a-GCST90001659 | ebi-a-GCST90001659 | TRUE | G | 96611102 | 5.17E-09 | reported | 3440 | 0.09744 | rs141019273 |  |  |  |  |  |  |  |  |  |  |  |  |  |  |  |
| 0.5689 | 10 | igd | 0.017 | A | \|\| id:ebi-a-GCST90001660 | ebi-a-GCST90001660 | TRUE | T | 95495162 | 1.42E-08 | reported | 3408 | 0.1001 | rs57091493 |  |  |  |  |  |  |  |  |  |  |  |  |  |  |  |
| -0.7769 | 10 | igd | 0.017 | A | \|\| id:ebi-a-GCST90001961 | ebi-a-GCST90001961 | TRUE | G | 97189011 | 9.49E-14 | reported | 2920 | 0.1038 | rs143965674 |  |  |  |  |  |  |  |  |  |  |  |  |  |  |  |
| 0.6076 | 2 | igd | 0.017 | T | \|\| id:ebi-a-GCST90002102 | ebi-a-GCST90002102 | TRUE | C | 37723876 | 6.82E-09 | reported | 2919 | 0.1045 | rs116816240 |  |  |  |  |  |  |  |  |  |  |  |  |  |  |  |
| 0.5993 | 17 | igd | 0.017 | T | \|\| id:ebi-a-GCST90001907 | ebi-a-GCST90001907 | TRUE | C | 38723925 | 2.09E-08 | reported | 2910 | 0.1066 | rs75983909 |  |  |  |  |  |  |  |  |  |  |  |  |  |  |  |
| 0.6158 | 3 | igd | 0.017 | A | \|\| id:ebi-a-GCST90001997 | ebi-a-GCST90001997 | TRUE | G | 39557771 | 8.40E-10 | reported | 3590 | 0.1001 | rs116640761 |  |  |  |  |  |  |  |  |  |  |  |  |  |  |  |
| -0.5657 | 10 | igd | 0.0169 | A | \|\| id:ebi-a-GCST90001672 | ebi-a-GCST90001672 | TRUE | G | 96147923 | 1.35E-08 | reported | 3408 | 0.09936 | rs150066446 |  |  |  |  |  |  |  |  |  |  |  |  |  |  |  |
| -0.5593 | 10 | igd | 0.0169 | T | \|\| id:ebi-a-GCST90001672 | ebi-a-GCST90001672 | TRUE | G | 96561181 | 2.24E-08 | reported | 3408 | 0.09977 | rs141932959 |  |  |  |  |  |  |  |  |  |  |  |  |  |  |  |
| -0.9479 | 19 | igd | 0.01685 | G | \|\| id:prot-a-439 | prot-a-439 | TRUE | A | 51731875 | 5.37E-19 | reported | 3301 | 0.1064 | rs145402896 |  |  |  |  |  |  |  |  |  |  |  |  |  |  |  |
| -0.6407 | 12 | igd | 0.0168 | G | \|\| id:ebi-a-GCST90002073 | ebi-a-GCST90002073 | TRUE | A | 68925296 | 2.62E-09 | reported | 2850 | 0.1073 | rs182069564 |  |  |  |  |  |  |  |  |  |  |  |  |  |  |  |
| -0.7304 | 12 | igd | 0.0168 | T | \|\| id:ebi-a-GCST90002073 | ebi-a-GCST90002073 | TRUE | G | 69832279 | 8.76E-12 | reported | 2850 | 0.1066 | rs151020523 |  |  |  |  |  |  |  |  |  |  |  |  |  |  |  |
| 0.7678 | 10 | igd | 0.0167 | A | \|\| id:ebi-a-GCST90001672 | ebi-a-GCST90001672 | TRUE | G | 97189011 | 3.11E-15 | reported | 3408 | 0.0969 | rs143965674 |  |  |  |  |  |  |  |  |  |  |  |  |  |  |  |
| 75.62 | 11 | igd | 0.0167 | C | \|\| id:ebi-a-GCST90001698 | ebi-a-GCST90001698 | TRUE | T | 130368054 | 3.50E-08 | reported | 3408 | 13.68 | rs146863207 |  |  |  |  |  |  |  |  |  |  |  |  |  |  |  |
| -0.5574 | 7 | igd | 0.0167 | C | \|\| id:ebi-a-GCST90002002 | ebi-a-GCST90002002 | TRUE | A | 152162064 | 1.16E-08 | reported | 3629 | 0.09747 | rs117195373 |  |  |  |  |  |  |  |  |  |  |  |  |  |  |  |
| 0.8833 | 10 | igd | 0.0166 | A | \|\| id:ebi-a-GCST90001670 | ebi-a-GCST90001670 | TRUE | G | 97189011 | 3.58E-20 | reported | 3440 | 0.09541 | rs143965674 |  |  |  |  |  |  |  |  |  |  |  |  |  |  |  |
| 0.8952 | 10 | igd | 0.0166 | A | \|\| id:ebi-a-GCST90001671 | ebi-a-GCST90001671 | TRUE | G | 97189011 | 1.14E-20 | reported | 3440 | 0.09541 | rs143965674 |  |  |  |  |  |  |  |  |  |  |  |  |  |  |  |
| -0.7519 | 12 | igd | 0.0165 | G | \|\| id:ebi-a-GCST90002074 | ebi-a-GCST90002074 | TRUE | A | 69247557 | 2.48E-13 | reported | 3112 | 0.1023 | rs1152928 |  |  |  |  |  |  |  |  |  |  |  |  |  |  |  |
| 0.5877 | 10 | igd | 0.0164 | A | \|\| id:ebi-a-GCST90001491 | ebi-a-GCST90001491 | TRUE | G | 98584210 | 1.62E-08 | reported | 3437 | 0.1038 | rs12257954 |  |  |  |  |  |  |  |  |  |  |  |  |  |  |  |
| -0.575 | 6 | igd | 0.0164 | G | \|\| id:ebi-a-GCST90001765 | ebi-a-GCST90001765 | TRUE | A | 101582531 | 9.26E-09 | reported | 3655 | 0.09988 | rs148432091 |  |  |  |  |  |  |  |  |  |  |  |  |  |  |  |
| 0.5742 | 10 | igd | 0.0163 | T | \|\| id:ebi-a-GCST90001489 | ebi-a-GCST90001489 | TRUE | C | 97883062 | 8.09E-09 | reported | 3405 | 0.09932 | rs78130758 |  |  |  |  |  |  |  |  |  |  |  |  |  |  |  |
| 0.5986 | 10 | igd | 0.0163 | T | \|\| id:ebi-a-GCST90001660 | ebi-a-GCST90001660 | TRUE | C | 97883062 | 1.28E-09 | reported | 3408 | 0.09834 | rs78130758 |  |  |  |  |  |  |  |  |  |  |  |  |  |  |  |
| -0.5881 | 7 | igd | 0.0163 | G | \|\| id:ebi-a-GCST90002002 | ebi-a-GCST90002002 | TRUE | C | 151910695 | 4.15E-09 | reported | 3629 | 0.0998 | rs147810622 |  |  |  |  |  |  |  |  |  |  |  |  |  |  |  |
| 85.79 | 6 | igd | 0.0161 | T | \|\| id:ebi-a-GCST90001698 | ebi-a-GCST90001698 | TRUE | C | 39196392 | 2.00E-09 | reported | 3408 | 14.27 | rs9471016 |  |  |  |  |  |  |  |  |  |  |  |  |  |  |  |
| 94.21 | 5 | igd | 0.016 | C | \|\| id:ebi-a-GCST90001698 | ebi-a-GCST90001698 | TRUE | T | 82017337 | 1.36E-11 | reported | 3408 | 13.88 | rs77713156 |  |  |  |  |  |  |  |  |  |  |  |  |  |  |  |
| -0.6444 | 22 | igd | 0.0159 | A | \|\| id:ebi-a-GCST90001702 | ebi-a-GCST90001702 | TRUE | G | 40999221 | 1.35E-09 | reported | 3657 | 0.106 | rs80270068 |  |  |  |  |  |  |  |  |  |  |  |  |  |  |  |
| -0.6175 | 22 | igd | 0.0159 | A | \|\| id:ebi-a-GCST90001703 | ebi-a-GCST90001703 | TRUE | C | 41084852 | 2.90E-09 | reported | 3657 | 0.1037 | rs192539655 |  |  |  |  |  |  |  |  |  |  |  |  |  |  |  |
| -0.6129 | 22 | igd | 0.0159 | A | \|\| id:ebi-a-GCST90001710 | ebi-a-GCST90001710 | TRUE | G | 40999221 | 7.45E-09 | reported | 3657 | 0.1058 | rs80270068 |  |  |  |  |  |  |  |  |  |  |  |  |  |  |  |
| -0.5875 | 22 | igd | 0.0159 | A | \|\| id:ebi-a-GCST90001711 | ebi-a-GCST90001711 | TRUE | G | 40999221 | 2.84E-08 | reported | 3657 | 0.1056 | rs80270068 |  |  |  |  |  |  |  |  |  |  |  |  |  |  |  |
| 0.5383 | 10 | igd | 0.0158 | A | \|\| id:ebi-a-GCST90001659 | ebi-a-GCST90001659 | TRUE | C | 97404972 | 2.91E-08 | reported | 3440 | 0.09682 | rs139620561 |  |  |  |  |  |  |  |  |  |  |  |  |  |  |  |
| 0.6069 | 10 | igd | 0.0156 | A | \|\| id:ebi-a-GCST90001489 | ebi-a-GCST90001489 | TRUE | G | 98554178 | 4.43E-08 | reported | 3405 | 0.1106 | rs11596351 |  |  |  |  |  |  |  |  |  |  |  |  |  |  |  |
| 96.61 | 9 | igd | 0.0156 | A | \|\| id:ebi-a-GCST90001698 | ebi-a-GCST90001698 | TRUE | C | 45362596 | 1.17E-09 | reported | 3408 | 15.83 | rs559632285 |  |  |  |  |  |  |  |  |  |  |  |  |  |  |  |
| 0.6102 | 10 | igd | 0.0154 | C | \|\| id:ebi-a-GCST90001670 | ebi-a-GCST90001670 | TRUE | T | 97812812 | 1.64E-09 | reported | 3440 | 0.1009 | rs144144036 |  |  |  |  |  |  |  |  |  |  |  |  |  |  |  |
| 0.5633 | 10 | igd | 0.0154 | C | \|\| id:ebi-a-GCST90001672 | ebi-a-GCST90001672 | TRUE | T | 97812812 | 4.73E-08 | reported | 3408 | 0.1029 | rs144144036 |  |  |  |  |  |  |  |  |  |  |  |  |  |  |  |
| -0.6475 | 1 | igd | 0.0154 | G | \|\| id:ebi-a-GCST90001858 | ebi-a-GCST90001858 | TRUE | A | 168605042 | 4.15E-09 | reported | 2919 | 0.1098 | rs12752576 |  |  |  |  |  |  |  |  |  |  |  |  |  |  |  |
| -0.645 | 1 | igd | 0.0154 | G | \|\| id:ebi-a-GCST90001860 | ebi-a-GCST90001860 | TRUE | A | 168605042 | 6.02E-09 | reported | 2919 | 0.1106 | rs12752576 |  |  |  |  |  |  |  |  |  |  |  |  |  |  |  |
| -0.6698 | 1 | igd | 0.0154 | G | \|\| id:ebi-a-GCST90001864 | ebi-a-GCST90001864 | TRUE | A | 168605042 | 1.20E-09 | reported | 2919 | 0.1098 | rs12752576 |  |  |  |  |  |  |  |  |  |  |  |  |  |  |  |
| -0.7265 | 17 | igd | 0.0154 | G | \|\| id:ebi-a-GCST90001926 | ebi-a-GCST90001926 | TRUE | A | 59130160 | 3.45E-10 | reported | 2920 | 0.1153 | rs142824579 |  |  |  |  |  |  |  |  |  |  |  |  |  |  |  |
| -0.7512 | 12 | igd | 0.0154 | A | \|\| id:ebi-a-GCST90002073 | ebi-a-GCST90002073 | TRUE | G | 69243706 | 1.54E-11 | reported | 2850 | 0.1109 | rs145035636 |  |  |  |  |  |  |  |  |  |  |  |  |  |  |  |
| 0.7861 | 10 | igd | 0.0151 | T | \|\| id:ebi-a-GCST90002030 | ebi-a-GCST90002030 | TRUE | A | 97615623 | 4.59E-13 | reported | 2920 | 0.1081 | rs11188510 |  |  |  |  |  |  |  |  |  |  |  |  |  |  |  |
| 0.6341 | 10 | igd | 0.0151 | T | \|\| id:ebi-a-GCST90002031 | ebi-a-GCST90002031 | TRUE | A | 97615623 | 1.48E-08 | reported | 2920 | 0.1116 | rs11188510 |  |  |  |  |  |  |  |  |  |  |  |  |  |  |  |
| 0.6713 | 10 | igd | 0.0151 | T | \|\| id:ebi-a-GCST90002032 | ebi-a-GCST90002032 | TRUE | A | 97615623 | 1.90E-09 | reported | 2920 | 0.1114 | rs11188510 |  |  |  |  |  |  |  |  |  |  |  |  |  |  |  |
| 0.5726 | 10 | igd | 0.015 | T | \|\| id:ebi-a-GCST90001484 | ebi-a-GCST90001484 | TRUE | A | 97615623 | 1.95E-08 | reported | 3437 | 0.1017 | rs11188510 |  |  |  |  |  |  |  |  |  |  |  |  |  |  |  |
| 0.5953 | 10 | igd | 0.015 | T | \|\| id:ebi-a-GCST90001495 | ebi-a-GCST90001495 | TRUE | A | 97615623 | 2.10E-08 | reported | 3405 | 0.106 | rs11188510 |  |  |  |  |  |  |  |  |  |  |  |  |  |  |  |
| 0.6582 | 10 | igd | 0.015 | T | \|\| id:ebi-a-GCST90001496 | ebi-a-GCST90001496 | TRUE | A | 97615623 | 3.64E-10 | reported | 3437 | 0.1047 | rs11188510 |  |  |  |  |  |  |  |  |  |  |  |  |  |  |  |
| 0.6486 | 10 | igd | 0.015 | T | \|\| id:ebi-a-GCST90001497 | ebi-a-GCST90001497 | TRUE | A | 97615623 | 3.95E-10 | reported | 3437 | 0.1034 | rs11188510 |  |  |  |  |  |  |  |  |  |  |  |  |  |  |  |
| 0.584 | 10 | igd | 0.015 | T | \|\| id:ebi-a-GCST90001658 | ebi-a-GCST90001658 | TRUE | A | 97615623 | 5.28E-09 | reported | 3440 | 0.09978 | rs11188510 |  |  |  |  |  |  |  |  |  |  |  |  |  |  |  |
| 0.6013 | 10 | igd | 0.015 | T | \|\| id:ebi-a-GCST90001659 | ebi-a-GCST90001659 | TRUE | A | 97615623 | 1.85E-09 | reported | 3440 | 0.09977 | rs11188510 |  |  |  |  |  |  |  |  |  |  |  |  |  |  |  |
| 96.99 | 5 | igd | 0.015 | G | \|\| id:ebi-a-GCST90001698 | ebi-a-GCST90001698 | TRUE | A | 80176162 | 3.91E-10 | reported | 3408 | 15.45 | rs116601877 |  |  |  |  |  |  |  |  |  |  |  |  |  |  |  |
| -0.7507 | 22 | igd | 0.015 | C | \|\| id:ebi-a-GCST90001702 | ebi-a-GCST90001702 | TRUE | A | 40727723 | 8.87E-13 | reported | 3657 | 0.1047 | rs118137006 |  |  |  |  |  |  |  |  |  |  |  |  |  |  |  |
| -0.7279 | 22 | igd | 0.015 | T | \|\| id:ebi-a-GCST90001702 | ebi-a-GCST90001702 | TRUE | C | 41195121 | 2.91E-12 | reported | 3657 | 0.1039 | rs118166233 |  |  |  |  |  |  |  |  |  |  |  |  |  |  |  |
| -0.7206 | 22 | igd | 0.015 | T | \|\| id:ebi-a-GCST90001703 | ebi-a-GCST90001703 | TRUE | C | 41195121 | 4.83E-12 | reported | 3657 | 0.1039 | rs118166233 |  |  |  |  |  |  |  |  |  |  |  |  |  |  |  |
| -0.7396 | 22 | igd | 0.015 | C | \|\| id:ebi-a-GCST90001703 | ebi-a-GCST90001703 | TRUE | A | 40727723 | 1.92E-12 | reported | 3657 | 0.1047 | rs118137006 |  |  |  |  |  |  |  |  |  |  |  |  |  |  |  |
| -0.6158 | 22 | igd | 0.015 | C | \|\| id:ebi-a-GCST90001704 | ebi-a-GCST90001704 | TRUE | A | 40727723 | 4.54E-09 | reported | 3657 | 0.1048 | rs118137006 |  |  |  |  |  |  |  |  |  |  |  |  |  |  |  |
| -0.5972 | 22 | igd | 0.015 | T | \|\| id:ebi-a-GCST90001704 | ebi-a-GCST90001704 | TRUE | C | 41195121 | 1.01E-08 | reported | 3657 | 0.104 | rs118166233 |  |  |  |  |  |  |  |  |  |  |  |  |  |  |  |
| -0.7062 | 22 | igd | 0.015 | C | \|\| id:ebi-a-GCST90001710 | ebi-a-GCST90001710 | TRUE | A | 40727723 | 1.58E-11 | reported | 3657 | 0.1044 | rs118137006 |  |  |  |  |  |  |  |  |  |  |  |  |  |  |  |
| -0.674 | 22 | igd | 0.015 | T | \|\| id:ebi-a-GCST90001710 | ebi-a-GCST90001710 | TRUE | C | 41195121 | 9.13E-11 | reported | 3657 | 0.1037 | rs118166233 |  |  |  |  |  |  |  |  |  |  |  |  |  |  |  |
| -0.6428 | 22 | igd | 0.015 | T | \|\| id:ebi-a-GCST90001711 | ebi-a-GCST90001711 | TRUE | C | 41195121 | 6.00E-10 | reported | 3657 | 0.1036 | rs118166233 |  |  |  |  |  |  |  |  |  |  |  |  |  |  |  |
| -0.6722 | 22 | igd | 0.015 | C | \|\| id:ebi-a-GCST90001711 | ebi-a-GCST90001711 | TRUE | A | 40727723 | 1.31E-10 | reported | 3657 | 0.1043 | rs118137006 |  |  |  |  |  |  |  |  |  |  |  |  |  |  |  |
| -0.7701 | 12 | igd | 0.0149 | A | \|\| id:ebi-a-GCST90002073 | ebi-a-GCST90002073 | TRUE | G | 69114122 | 1.56E-11 | reported | 2850 | 0.1138 | rs149466784 |  |  |  |  |  |  |  |  |  |  |  |  |  |  |  |
| 0.698 | 2 | igd | 0.0149 | C | \|\| id:ebi-a-GCST90002102 | ebi-a-GCST90002102 | TRUE | A | 40773763 | 3.00E-10 | reported | 2919 | 0.1104 | rs78689347 |  |  |  |  |  |  |  |  |  |  |  |  |  |  |  |
| 0.6001 | 10 | igd | 0.0148 | G | \|\| id:ebi-a-GCST90001671 | ebi-a-GCST90001671 | TRUE | A | 98927415 | 2.05E-08 | reported | 3440 | 0.1068 | rs72821763 |  |  |  |  |  |  |  |  |  |  |  |  |  |  |  |
| 90.03 | 2 | igd | 0.0148 | A | \|\| id:ebi-a-GCST90001698 | ebi-a-GCST90001698 | TRUE | G | 81365670 | 7.31E-10 | reported | 3408 | 14.57 | rs146627868 |  |  |  |  |  |  |  |  |  |  |  |  |  |  |  |
| -0.9465 | 6 | igd | 0.0148 | T | \|\| id:ebi-a-GCST90002105 | ebi-a-GCST90002105 | TRUE | C | 32455040 | 9.87E-13 | reported | 2872 | 0.1321 | rs73401335 |  |  |  |  |  |  |  |  |  |  |  |  |  |  |  |
| 0.657 | 10 | igd | 0.0147 | G | \|\| id:ebi-a-GCST90001660 | ebi-a-GCST90001660 | TRUE | A | 96455679 | 6.13E-09 | reported | 3408 | 0.1127 | rs145712520 |  |  |  |  |  |  |  |  |  |  |  |  |  |  |  |
| 71.36 | 12 | igd | 0.0147 | T | \|\| id:ebi-a-GCST90001695 | ebi-a-GCST90001695 | TRUE | C | 47679643 | 9.24E-11 | reported | 3408 | 10.98 | rs139457426 |  |  |  |  |  |  |  |  |  |  |  |  |  |  |  |
| -0.5993 | 3 | igd | 0.0146 | G | \|\| id:ebi-a-GCST90002004 | ebi-a-GCST90002004 | TRUE | C | 53544015 | 5.80E-09 | reported | 3629 | 0.1027 | rs141411805 |  |  |  |  |  |  |  |  |  |  |  |  |  |  |  |
| 0.6318 | 1 | igd | 0.0145 | G | \|\| id:ebi-a-GCST90001508 | ebi-a-GCST90001508 | TRUE | A | 188691469 | 4.42E-09 | reported | 3437 | 0.1074 | rs61836370 |  |  |  |  |  |  |  |  |  |  |  |  |  |  |  |
| 0.6084 | 1 | igd | 0.0145 | G | \|\| id:ebi-a-GCST90001509 | ebi-a-GCST90001509 | TRUE | A | 188691469 | 1.58E-08 | reported | 3437 | 0.1074 | rs61836370 |  |  |  |  |  |  |  |  |  |  |  |  |  |  |  |
| 0.6466 | 10 | igd | 0.0145 | G | \|\| id:ebi-a-GCST90001658 | ebi-a-GCST90001658 | TRUE | A | 96455679 | 2.27E-09 | reported | 3440 | 0.1079 | rs145712520 |  |  |  |  |  |  |  |  |  |  |  |  |  |  |  |
| 0.5938 | 10 | igd | 0.0145 | G | \|\| id:ebi-a-GCST90001659 | ebi-a-GCST90001659 | TRUE | A | 96455679 | 4.09E-08 | reported | 3440 | 0.108 | rs145712520 |  |  |  |  |  |  |  |  |  |  |  |  |  |  |  |
| 110.8 | 5 | igd | 0.0145 | A | \|\| id:ebi-a-GCST90001698 | ebi-a-GCST90001698 | TRUE | G | 79224940 | 3.34E-13 | reported | 3408 | 15.16 | rs78374377 |  |  |  |  |  |  |  |  |  |  |  |  |  |  |  |
| -0.6629 | 10 | igd | 0.0144 | T | \|\| id:ebi-a-GCST90001496 | ebi-a-GCST90001496 | TRUE | G | 97320916 | 3.51E-10 | reported | 3437 | 0.1053 | rs141886579 |  |  |  |  |  |  |  |  |  |  |  |  |  |  |  |
| -0.6483 | 10 | igd | 0.0144 | T | \|\| id:ebi-a-GCST90001497 | ebi-a-GCST90001497 | TRUE | G | 97320916 | 5.17E-10 | reported | 3437 | 0.104 | rs141886579 |  |  |  |  |  |  |  |  |  |  |  |  |  |  |  |
| -0.8103 | 17 | igd | 0.0144 | A | \|\| id:ebi-a-GCST90001926 | ebi-a-GCST90001926 | TRUE | G | 58777014 | 5.08E-12 | reported | 2920 | 0.1169 | rs142587417 |  |  |  |  |  |  |  |  |  |  |  |  |  |  |  |
| -0.6824 | 10 | igd | 0.0144 | T | \|\| id:ebi-a-GCST90002030 | ebi-a-GCST90002030 | TRUE | G | 97320916 | 2.18E-09 | reported | 2920 | 0.1137 | rs141886579 |  |  |  |  |  |  |  |  |  |  |  |  |  |  |  |
| -0.659 | 2 | igd | 0.0144 | A | \|\| id:ebi-a-GCST90002105 | ebi-a-GCST90002105 | TRUE | G | 179315726 | 3.40E-08 | reported | 2872 | 0.1191 | rs62176112 |  |  |  |  |  |  |  |  |  |  |  |  |  |  |  |
| 0.8912 | 6 | igd | 0.0144 | A | \|\| id:ebi-a-GCST90002105 | ebi-a-GCST90002105 | TRUE | G | 30354706 | 4.63E-14 | reported | 2872 | 0.1176 | rs12664075 |  |  |  |  |  |  |  |  |  |  |  |  |  |  |  |
| 0.6506 | 10 | igd | 0.0143 | T | \|\| id:ebi-a-GCST90001484 | ebi-a-GCST90001484 | TRUE | C | 97643729 | 3.53E-10 | reported | 3437 | 0.1034 | rs61871073 |  |  |  |  |  |  |  |  |  |  |  |  |  |  |  |
| 0.6128 | 10 | igd | 0.0143 | T | \|\| id:ebi-a-GCST90001485 | ebi-a-GCST90001485 | TRUE | C | 97643729 | 3.32E-09 | reported | 3437 | 0.1033 | rs61871073 |  |  |  |  |  |  |  |  |  |  |  |  |  |  |  |
| 0.7261 | 10 | igd | 0.0143 | T | \|\| id:ebi-a-GCST90001496 | ebi-a-GCST90001496 | TRUE | C | 97643729 | 7.96E-12 | reported | 3437 | 0.1058 | rs61871073 |  |  |  |  |  |  |  |  |  |  |  |  |  |  |  |
| 0.7182 | 10 | igd | 0.0143 | T | \|\| id:ebi-a-GCST90001497 | ebi-a-GCST90001497 | TRUE | C | 97643729 | 7.33E-12 | reported | 3437 | 0.1045 | rs61871073 |  |  |  |  |  |  |  |  |  |  |  |  |  |  |  |
| -0.7686 | 17 | igd | 0.0142 | T | \|\| id:ebi-a-GCST90001926 | ebi-a-GCST90001926 | TRUE | C | 58986927 | 7.68E-11 | reported | 2920 | 0.1177 | rs148952084 |  |  |  |  |  |  |  |  |  |  |  |  |  |  |  |
| 61.29 | 14 | igd | 0.0141 | C | \|\| id:ebi-a-GCST90001695 | ebi-a-GCST90001695 | TRUE | T | 43151367 | 4.77E-08 | reported | 3408 | 11.2 | rs150383571 |  |  |  |  |  |  |  |  |  |  |  |  |  |  |  |
| -0.6436 | 10 | igd | 0.014 | C | \|\| id:ebi-a-GCST90001496 | ebi-a-GCST90001496 | TRUE | T | 100792938 | 1.35E-08 | reported | 3437 | 0.113 | rs117882530 |  |  |  |  |  |  |  |  |  |  |  |  |  |  |  |
| 0.7193 | 10 | igd | 0.014 | T | \|\| id:ebi-a-GCST90001670 | ebi-a-GCST90001670 | TRUE | G | 95638595 | 4.52E-11 | reported | 3440 | 0.1089 | rs71480890 |  |  |  |  |  |  |  |  |  |  |  |  |  |  |  |
| 0.6704 | 10 | igd | 0.014 | T | \|\| id:ebi-a-GCST90001671 | ebi-a-GCST90001671 | TRUE | G | 95638595 | 8.43E-10 | reported | 3440 | 0.1089 | rs71480890 |  |  |  |  |  |  |  |  |  |  |  |  |  |  |  |
| -0.659 | 17 | igd | 0.014 | T | \|\| id:ebi-a-GCST90001926 | ebi-a-GCST90001926 | TRUE | C | 55643849 | 9.40E-09 | reported | 2920 | 0.1144 | rs139132726 |  |  |  |  |  |  |  |  |  |  |  |  |  |  |  |
| -0.6435 | 12 | igd | 0.014 | T | \|\| id:ebi-a-GCST90002074 | ebi-a-GCST90002074 | TRUE | C | 69506844 | 1.65E-08 | reported | 3112 | 0.1137 | rs11837612 |  |  |  |  |  |  |  |  |  |  |  |  |  |  |  |
| 0.6077 | 10 | igd | 0.0139 | T | \|\| id:ebi-a-GCST90001672 | ebi-a-GCST90001672 | TRUE | G | 95638595 | 4.50E-08 | reported | 3408 | 0.1108 | rs71480890 |  |  |  |  |  |  |  |  |  |  |  |  |  |  |  |
| 69.53 | 6 | igd | 0.0139 | A | \|\| id:ebi-a-GCST90001695 | ebi-a-GCST90001695 | TRUE | T | 65783601 | 1.87E-09 | reported | 3408 | 11.54 | rs115485721 |  |  |  |  |  |  |  |  |  |  |  |  |  |  |  |
| -0.7006 | 22 | igd | 0.0139 | A | \|\| id:ebi-a-GCST90001702 | ebi-a-GCST90001702 | TRUE | G | 39719566 | 2.41E-10 | reported | 3657 | 0.1103 | rs144712270 |  |  |  |  |  |  |  |  |  |  |  |  |  |  |  |
| -0.6843 | 22 | igd | 0.0139 | A | \|\| id:ebi-a-GCST90001703 | ebi-a-GCST90001703 | TRUE | G | 39719566 | 6.23E-10 | reported | 3657 | 0.1103 | rs144712270 |  |  |  |  |  |  |  |  |  |  |  |  |  |  |  |
| -0.6715 | 22 | igd | 0.0139 | A | \|\| id:ebi-a-GCST90001710 | ebi-a-GCST90001710 | TRUE | G | 39719566 | 1.16E-09 | reported | 3657 | 0.11 | rs144712270 |  |  |  |  |  |  |  |  |  |  |  |  |  |  |  |
| -0.6445 | 22 | igd | 0.0139 | A | \|\| id:ebi-a-GCST90001711 | ebi-a-GCST90001711 | TRUE | G | 39719566 | 4.86E-09 | reported | 3657 | 0.1099 | rs144712270 |  |  |  |  |  |  |  |  |  |  |  |  |  |  |  |
| 0.9061 | 10 | igd | 0.0139 | T | \|\| id:ebi-a-GCST90002030 | ebi-a-GCST90002030 | TRUE | C | 97643729 | 1.48E-15 | reported | 2920 | 0.1129 | rs61871073 |  |  |  |  |  |  |  |  |  |  |  |  |  |  |  |
| 0.8122 | 10 | igd | 0.0139 | T | \|\| id:ebi-a-GCST90002031 | ebi-a-GCST90002031 | TRUE | C | 97643729 | 5.10E-12 | reported | 2920 | 0.1172 | rs61871073 |  |  |  |  |  |  |  |  |  |  |  |  |  |  |  |
| 0.8985 | 10 | igd | 0.0139 | T | \|\| id:ebi-a-GCST90002032 | ebi-a-GCST90002032 | TRUE | C | 97643729 | 1.88E-14 | reported | 2920 | 0.1167 | rs61871073 |  |  |  |  |  |  |  |  |  |  |  |  |  |  |  |
| 0.7195 | 2 | igd | 0.0139 | C | \|\| id:ebi-a-GCST90002098 | ebi-a-GCST90002098 | TRUE | T | 40807940 | 1.10E-09 | reported | 2910 | 0.1177 | rs141658096 |  |  |  |  |  |  |  |  |  |  |  |  |  |  |  |
| 0.6234 | 10 | igd | 0.0138 | C | \|\| id:ebi-a-GCST90001660 | ebi-a-GCST90001660 | TRUE | A | 104067646 | 3.88E-08 | reported | 3408 | 0.1132 | rs145702817 |  |  |  |  |  |  |  |  |  |  |  |  |  |  |  |
| 105.7 | 5 | igd | 0.0138 | T | \|\| id:ebi-a-GCST90001698 | ebi-a-GCST90001698 | TRUE | C | 80642243 | 7.44E-12 | reported | 3408 | 15.37 | rs72771180 |  |  |  |  |  |  |  |  |  |  |  |  |  |  |  |
| -0.6329 | 22 | igd | 0.0138 | A | \|\| id:ebi-a-GCST90001702 | ebi-a-GCST90001702 | TRUE | T | 44698304 | 7.26E-09 | reported | 3657 | 0.1092 | rs189503542 |  |  |  |  |  |  |  |  |  |  |  |  |  |  |  |
| -0.6337 | 22 | igd | 0.0138 | A | \|\| id:ebi-a-GCST90001703 | ebi-a-GCST90001703 | TRUE | T | 44698304 | 6.96E-09 | reported | 3657 | 0.1091 | rs189503542 |  |  |  |  |  |  |  |  |  |  |  |  |  |  |  |
| -0.6776 | 22 | igd | 0.0138 | A | \|\| id:ebi-a-GCST90001704 | ebi-a-GCST90001704 | TRUE | T | 44698304 | 5.52E-10 | reported | 3657 | 0.1089 | rs189503542 |  |  |  |  |  |  |  |  |  |  |  |  |  |  |  |
| 0.6427 | 10 | igd | 0.0137 | A | \|\| id:ebi-a-GCST90001495 | ebi-a-GCST90001495 | TRUE | G | 98768220 | 3.46E-08 | reported | 3405 | 0.1162 | rs12245404 |  |  |  |  |  |  |  |  |  |  |  |  |  |  |  |
| -0.6456 | 10 | igd | 0.0137 | A | \|\| id:ebi-a-GCST90001496 | ebi-a-GCST90001496 | TRUE | G | 100616579 | 1.77E-08 | reported | 3437 | 0.1143 | rs75742656 |  |  |  |  |  |  |  |  |  |  |  |  |  |  |  |
| 0.7814 | 10 | igd | 0.0137 | C | \|\| id:ebi-a-GCST90002031 | ebi-a-GCST90002031 | TRUE | G | 96140978 | 1.65E-10 | reported | 2920 | 0.1218 | rs145170141 |  |  |  |  |  |  |  |  |  |  |  |  |  |  |  |
| 0.7923 | 10 | igd | 0.0137 | C | \|\| id:ebi-a-GCST90002032 | ebi-a-GCST90002032 | TRUE | G | 96140978 | 9.40E-11 | reported | 2920 | 0.1219 | rs145170141 |  |  |  |  |  |  |  |  |  |  |  |  |  |  |  |
| -0.8217 | 12 | igd | 0.0137 | T | \|\| id:ebi-a-GCST90002073 | ebi-a-GCST90002073 | TRUE | C | 69506844 | 5.03E-12 | reported | 2850 | 0.1185 | rs11837612 |  |  |  |  |  |  |  |  |  |  |  |  |  |  |  |
| -0.611 | 10 | igd | 0.0135 | T | \|\| id:ebi-a-GCST90001495 | ebi-a-GCST90001495 | TRUE | A | 95396375 | 2.32E-08 | reported | 3405 | 0.1091 | rs117418927 |  |  |  |  |  |  |  |  |  |  |  |  |  |  |  |
| -0.6731 | 10 | igd | 0.0135 | T | \|\| id:ebi-a-GCST90001496 | ebi-a-GCST90001496 | TRUE | A | 95396375 | 4.97E-10 | reported | 3437 | 0.1079 | rs117418927 |  |  |  |  |  |  |  |  |  |  |  |  |  |  |  |
| 0.6482 | 10 | igd | 0.0135 | A | \|\| id:ebi-a-GCST90001496 | ebi-a-GCST90001496 | TRUE | G | 98768220 | 2.06E-08 | reported | 3437 | 0.1153 | rs12245404 |  |  |  |  |  |  |  |  |  |  |  |  |  |  |  |
| -0.6637 | 10 | igd | 0.0135 | T | \|\| id:ebi-a-GCST90001497 | ebi-a-GCST90001497 | TRUE | A | 95396375 | 5.25E-10 | reported | 3437 | 0.1065 | rs117418927 |  |  |  |  |  |  |  |  |  |  |  |  |  |  |  |
| -0.5643 | 10 | igd | 0.0135 | T | \|\| id:ebi-a-GCST90001659 | ebi-a-GCST90001659 | TRUE | A | 95396375 | 4.28E-08 | reported | 3440 | 0.1028 | rs117418927 |  |  |  |  |  |  |  |  |  |  |  |  |  |  |  |
| -0.5977 | 10 | igd | 0.0135 | T | \|\| id:ebi-a-GCST90001660 | ebi-a-GCST90001660 | TRUE | A | 95396375 | 2.99E-08 | reported | 3408 | 0.1076 | rs117418927 |  |  |  |  |  |  |  |  |  |  |  |  |  |  |  |
| 95.82 | 10 | igd | 0.0135 | A | \|\| id:ebi-a-GCST90001698 | ebi-a-GCST90001698 | TRUE | C | 33544216 | 1.92E-10 | reported | 3408 | 15 | rs7096016 |  |  |  |  |  |  |  |  |  |  |  |  |  |  |  |
| 93.79 | 18 | igd | 0.0135 | A | \|\| id:ebi-a-GCST90001698 | ebi-a-GCST90001698 | TRUE | T | 38662297 | 5.14E-10 | reported | 3408 | 15.05 | rs150635355 |  |  |  |  |  |  |  |  |  |  |  |  |  |  |  |
| -0.5805 | 16 | igd | 0.0135 | T | \|\| id:ebi-a-GCST90001724 | ebi-a-GCST90001724 | TRUE | C | 28979854 | 3.14E-08 | reported | 3656 | 0.1047 | rs62037424 |  |  |  |  |  |  |  |  |  |  |  |  |  |  |  |
| -0.605 | 16 | igd | 0.0135 | T | \|\| id:ebi-a-GCST90001729 | ebi-a-GCST90001729 | TRUE | C | 28979854 | 1.05E-08 | reported | 3657 | 0.1055 | rs62037424 |  |  |  |  |  |  |  |  |  |  |  |  |  |  |  |
| 88.95 | 6 | igd | 0.0134 | A | \|\| id:ebi-a-GCST90001698 | ebi-a-GCST90001698 | TRUE | C | 159259567 | 1.07E-08 | reported | 3408 | 15.51 | rs141291585 |  |  |  |  |  |  |  |  |  |  |  |  |  |  |  |
| -1.208 | 22 | igd | 0.0134 | T | \|\| id:ebi-a-GCST90001702 | ebi-a-GCST90001702 | TRUE | C | 39691045 | 2.40E-28 | reported | 3657 | 0.1085 | rs80132412 |  |  |  |  |  |  |  |  |  |  |  |  |  |  |  |
| -1.218 | 22 | igd | 0.0134 | T | \|\| id:ebi-a-GCST90001703 | ebi-a-GCST90001703 | TRUE | C | 39691045 | 9.19E-29 | reported | 3657 | 0.1085 | rs80132412 |  |  |  |  |  |  |  |  |  |  |  |  |  |  |  |
| -1.219 | 22 | igd | 0.0134 | T | \|\| id:ebi-a-GCST90001704 | ebi-a-GCST90001704 | TRUE | C | 39691045 | 6.67E-29 | reported | 3657 | 0.1083 | rs80132412 |  |  |  |  |  |  |  |  |  |  |  |  |  |  |  |
| -1.139 | 22 | igd | 0.0134 | T | \|\| id:ebi-a-GCST90001710 | ebi-a-GCST90001710 | TRUE | C | 39691045 | 1.91E-25 | reported | 3657 | 0.1084 | rs80132412 |  |  |  |  |  |  |  |  |  |  |  |  |  |  |  |
| -1.176 | 22 | igd | 0.0134 | T | \|\| id:ebi-a-GCST90001711 | ebi-a-GCST90001711 | TRUE | C | 39691045 | 3.70E-27 | reported | 3657 | 0.1081 | rs80132412 |  |  |  |  |  |  |  |  |  |  |  |  |  |  |  |
| 0.8697 | 10 | igd | 0.0134 | T | \|\| id:ebi-a-GCST90002030 | ebi-a-GCST90002030 | TRUE | C | 97783706 | 2.00E-13 | reported | 2920 | 0.1178 | rs116882651 |  |  |  |  |  |  |  |  |  |  |  |  |  |  |  |
| 0.8344 | 10 | igd | 0.0134 | T | \|\| id:ebi-a-GCST90002031 | ebi-a-GCST90002031 | TRUE | C | 97783706 | 7.24E-12 | reported | 2920 | 0.1213 | rs116882651 |  |  |  |  |  |  |  |  |  |  |  |  |  |  |  |
| 0.8885 | 10 | igd | 0.0134 | T | \|\| id:ebi-a-GCST90002032 | ebi-a-GCST90002032 | TRUE | C | 97783706 | 2.69E-13 | reported | 2920 | 0.121 | rs116882651 |  |  |  |  |  |  |  |  |  |  |  |  |  |  |  |
| 0.7268 | 2 | igd | 0.0134 | A | \|\| id:ebi-a-GCST90002098 | ebi-a-GCST90002098 | TRUE | G | 41319875 | 6.17E-10 | reported | 2910 | 0.1171 | rs116064674 |  |  |  |  |  |  |  |  |  |  |  |  |  |  |  |
| 0.7055 | 2 | igd | 0.0134 | G | \|\| id:ebi-a-GCST90002098 | ebi-a-GCST90002098 | TRUE | T | 40941990 | 2.82E-09 | reported | 2910 | 0.1184 | rs116193467 |  |  |  |  |  |  |  |  |  |  |  |  |  |  |  |
| 0.722 | 2 | igd | 0.0134 | C | \|\| id:ebi-a-GCST90002102 | ebi-a-GCST90002102 | TRUE | T | 41377007 | 5.78E-10 | reported | 2919 | 0.1161 | rs72791360 |  |  |  |  |  |  |  |  |  |  |  |  |  |  |  |
| 0.7012 | 2 | igd | 0.0134 | G | \|\| id:ebi-a-GCST90002102 | ebi-a-GCST90002102 | TRUE | T | 40941990 | 2.74E-09 | reported | 2919 | 0.1176 | rs116193467 |  |  |  |  |  |  |  |  |  |  |  |  |  |  |  |
| 0.6455 | 10 | igd | 0.0132 | T | \|\| id:ebi-a-GCST90001484 | ebi-a-GCST90001484 | TRUE | C | 97783706 | 2.57E-09 | reported | 3437 | 0.1081 | rs116882651 |  |  |  |  |  |  |  |  |  |  |  |  |  |  |  |
| 0.5999 | 10 | igd | 0.0132 | T | \|\| id:ebi-a-GCST90001485 | ebi-a-GCST90001485 | TRUE | C | 97783706 | 2.98E-08 | reported | 3437 | 0.108 | rs116882651 |  |  |  |  |  |  |  |  |  |  |  |  |  |  |  |
| 0.661 | 10 | igd | 0.0132 | T | \|\| id:ebi-a-GCST90001496 | ebi-a-GCST90001496 | TRUE | C | 97783706 | 2.87E-09 | reported | 3437 | 0.111 | rs116882651 |  |  |  |  |  |  |  |  |  |  |  |  |  |  |  |
| 0.6618 | 10 | igd | 0.0132 | T | \|\| id:ebi-a-GCST90001497 | ebi-a-GCST90001497 | TRUE | C | 97783706 | 1.72E-09 | reported | 3437 | 0.1096 | rs116882651 |  |  |  |  |  |  |  |  |  |  |  |  |  |  |  |
| 0.6476 | 10 | igd | 0.0132 | C | \|\| id:ebi-a-GCST90001660 | ebi-a-GCST90001660 | TRUE | T | 104204626 | 1.53E-08 | reported | 3408 | 0.1142 | rs139310175 |  |  |  |  |  |  |  |  |  |  |  |  |  |  |  |
| 107.5 | 5 | igd | 0.0132 | G | \|\| id:ebi-a-GCST90001698 | ebi-a-GCST90001698 | TRUE | A | 82086616 | 6.36E-12 | reported | 3408 | 15.59 | rs115077566 |  |  |  |  |  |  |  |  |  |  |  |  |  |  |  |
| 102.6 | 19 | igd | 0.0132 | A | \|\| id:ebi-a-GCST90001698 | ebi-a-GCST90001698 | TRUE | G | 31931387 | 2.73E-11 | reported | 3408 | 15.35 | rs75287501 |  |  |  |  |  |  |  |  |  |  |  |  |  |  |  |
| 0.7387 | 2 | igd | 0.0132 | T | \|\| id:ebi-a-GCST90002098 | ebi-a-GCST90002098 | TRUE | C | 41198855 | 1.03E-09 | reported | 2910 | 0.1206 | rs115716183 |  |  |  |  |  |  |  |  |  |  |  |  |  |  |  |
| 0.7447 | 2 | igd | 0.0132 | T | \|\| id:ebi-a-GCST90002102 | ebi-a-GCST90002102 | TRUE | C | 41198855 | 5.70E-10 | reported | 2919 | 0.1197 | rs115716183 |  |  |  |  |  |  |  |  |  |  |  |  |  |  |  |
| 0.6099 | 10 | igd | 0.0131 | T | \|\| id:ebi-a-GCST90001660 | ebi-a-GCST90001660 | TRUE | C | 102435664 | 4.79E-08 | reported | 3408 | 0.1115 | rs34912720 |  |  |  |  |  |  |  |  |  |  |  |  |  |  |  |
| 97.12 | 16 | igd | 0.0131 | T | \|\| id:ebi-a-GCST90001698 | ebi-a-GCST90001698 | TRUE | C | 7465748 | 1.17E-09 | reported | 3408 | 15.92 | rs4786166 |  |  |  |  |  |  |  |  |  |  |  |  |  |  |  |
| -0.7089 | 22 | igd | 0.013 | T | \|\| id:ebi-a-GCST90001702 | ebi-a-GCST90001702 | TRUE | G | 37565404 | 1.08E-10 | reported | 3657 | 0.1095 | rs142894868 |  |  |  |  |  |  |  |  |  |  |  |  |  |  |  |
| -0.7028 | 22 | igd | 0.013 | G | \|\| id:ebi-a-GCST90001702 | ebi-a-GCST90001702 | TRUE | A | 44524022 | 1.26E-09 | reported | 3657 | 0.1154 | rs150453698 |  |  |  |  |  |  |  |  |  |  |  |  |  |  |  |
| -0.6857 | 22 | igd | 0.013 | G | \|\| id:ebi-a-GCST90001703 | ebi-a-GCST90001703 | TRUE | A | 44524022 | 3.09E-09 | reported | 3657 | 0.1154 | rs150453698 |  |  |  |  |  |  |  |  |  |  |  |  |  |  |  |
| -0.7196 | 22 | igd | 0.013 | T | \|\| id:ebi-a-GCST90001703 | ebi-a-GCST90001703 | TRUE | G | 37565404 | 5.62E-11 | reported | 3657 | 0.1095 | rs142894868 |  |  |  |  |  |  |  |  |  |  |  |  |  |  |  |
| -0.721 | 22 | igd | 0.013 | T | \|\| id:ebi-a-GCST90001704 | ebi-a-GCST90001704 | TRUE | G | 37565404 | 4.86E-11 | reported | 3657 | 0.1093 | rs142894868 |  |  |  |  |  |  |  |  |  |  |  |  |  |  |  |
| -0.603 | 22 | igd | 0.013 | A | \|\| id:ebi-a-GCST90001704 | ebi-a-GCST90001704 | TRUE | G | 38979154 | 4.32E-08 | reported | 3657 | 0.1099 | rs112861194 |  |  |  |  |  |  |  |  |  |  |  |  |  |  |  |
| -0.7313 | 22 | igd | 0.013 | G | \|\| id:ebi-a-GCST90001704 | ebi-a-GCST90001704 | TRUE | A | 44524022 | 2.42E-10 | reported | 3657 | 0.1152 | rs150453698 |  |  |  |  |  |  |  |  |  |  |  |  |  |  |  |
| -0.7526 | 22 | igd | 0.013 | T | \|\| id:ebi-a-GCST90001710 | ebi-a-GCST90001710 | TRUE | G | 37565404 | 6.14E-12 | reported | 3657 | 0.1091 | rs142894868 |  |  |  |  |  |  |  |  |  |  |  |  |  |  |  |
| -0.6639 | 22 | igd | 0.013 | G | \|\| id:ebi-a-GCST90001710 | ebi-a-GCST90001710 | TRUE | A | 44524022 | 9.01E-09 | reported | 3657 | 0.1152 | rs150453698 |  |  |  |  |  |  |  |  |  |  |  |  |  |  |  |
| -0.6773 | 22 | igd | 0.013 | G | \|\| id:ebi-a-GCST90001711 | ebi-a-GCST90001711 | TRUE | A | 44524022 | 4.00E-09 | reported | 3657 | 0.1148 | rs150453698 |  |  |  |  |  |  |  |  |  |  |  |  |  |  |  |
| -0.7267 | 22 | igd | 0.013 | T | \|\| id:ebi-a-GCST90001711 | ebi-a-GCST90001711 | TRUE | G | 37565404 | 2.89E-11 | reported | 3657 | 0.1089 | rs142894868 |  |  |  |  |  |  |  |  |  |  |  |  |  |  |  |
| 0.7457 | 10 | igd | 0.013 | A | \|\| id:ebi-a-GCST90002030 | ebi-a-GCST90002030 | TRUE | G | 96823042 | 1.07E-09 | reported | 2920 | 0.1219 | rs113376608 |  |  |  |  |  |  |  |  |  |  |  |  |  |  |  |
| 0.7242 | 10 | igd | 0.013 | A | \|\| id:ebi-a-GCST90002031 | ebi-a-GCST90002031 | TRUE | G | 96823042 | 4.76E-09 | reported | 2920 | 0.1233 | rs113376608 |  |  |  |  |  |  |  |  |  |  |  |  |  |  |  |
| 0.7229 | 10 | igd | 0.013 | A | \|\| id:ebi-a-GCST90002032 | ebi-a-GCST90002032 | TRUE | G | 96823042 | 5.25E-09 | reported | 2920 | 0.1234 | rs113376608 |  |  |  |  |  |  |  |  |  |  |  |  |  |  |  |
| 0.6266 | 10 | igd | 0.0129 | A | \|\| id:ebi-a-GCST90001658 | ebi-a-GCST90001658 | TRUE | G | 96823042 | 1.01E-08 | reported | 3440 | 0.1091 | rs113376608 |  |  |  |  |  |  |  |  |  |  |  |  |  |  |  |
| 0.6332 | 10 | igd | 0.0129 | A | \|\| id:ebi-a-GCST90001671 | ebi-a-GCST90001671 | TRUE | G | 96823042 | 2.79E-08 | reported | 3440 | 0.1137 | rs113376608 |  |  |  |  |  |  |  |  |  |  |  |  |  |  |  |
| -0.996 | 22 | igd | 0.0129 | A | \|\| id:ebi-a-GCST90001702 | ebi-a-GCST90001702 | TRUE | G | 38163121 | 7.62E-20 | reported | 3657 | 0.1086 | rs190529651 |  |  |  |  |  |  |  |  |  |  |  |  |  |  |  |
| -1.005 | 22 | igd | 0.0129 | A | \|\| id:ebi-a-GCST90001703 | ebi-a-GCST90001703 | TRUE | G | 38163121 | 3.59E-20 | reported | 3657 | 0.1086 | rs190529651 |  |  |  |  |  |  |  |  |  |  |  |  |  |  |  |
| -1.028 | 22 | igd | 0.0129 | A | \|\| id:ebi-a-GCST90001704 | ebi-a-GCST90001704 | TRUE | G | 38163121 | 4.01E-21 | reported | 3657 | 0.1084 | rs190529651 |  |  |  |  |  |  |  |  |  |  |  |  |  |  |  |
| -1.002 | 22 | igd | 0.0129 | A | \|\| id:ebi-a-GCST90001710 | ebi-a-GCST90001710 | TRUE | G | 38163121 | 3.61E-20 | reported | 3657 | 0.1083 | rs190529651 |  |  |  |  |  |  |  |  |  |  |  |  |  |  |  |
| -1.002 | 22 | igd | 0.0129 | A | \|\| id:ebi-a-GCST90001711 | ebi-a-GCST90001711 | TRUE | G | 38163121 | 3.04E-20 | reported | 3657 | 0.108 | rs190529651 |  |  |  |  |  |  |  |  |  |  |  |  |  |  |  |
| 97.68 | 10 | igd | 0.0128 | C | \|\| id:ebi-a-GCST90001698 | ebi-a-GCST90001698 | TRUE | T | 33359425 | 1.30E-10 | reported | 3408 | 15.15 | rs78622624 |  |  |  |  |  |  |  |  |  |  |  |  |  |  |  |
| -0.6808 | 1 | igd | 0.0128 | T | \|\| id:ebi-a-GCST90002077 | ebi-a-GCST90002077 | TRUE | C | 157425761 | 3.93E-08 | reported | 2971 | 0.1236 | rs143012283 |  |  |  |  |  |  |  |  |  |  |  |  |  |  |  |
| -0.8258 | 22 | igd | 0.0127 | A | \|\| id:ebi-a-GCST90001702 | ebi-a-GCST90001702 | TRUE | G | 40044235 | 2.96E-13 | reported | 3657 | 0.1128 | rs77273762 |  |  |  |  |  |  |  |  |  |  |  |  |  |  |  |
| -0.8118 | 22 | igd | 0.0127 | A | \|\| id:ebi-a-GCST90001703 | ebi-a-GCST90001703 | TRUE | G | 40044235 | 7.37E-13 | reported | 3657 | 0.1128 | rs77273762 |  |  |  |  |  |  |  |  |  |  |  |  |  |  |  |
| -0.6875 | 22 | igd | 0.0127 | A | \|\| id:ebi-a-GCST90001704 | ebi-a-GCST90001704 | TRUE | G | 40044235 | 1.23E-09 | reported | 3657 | 0.1129 | rs77273762 |  |  |  |  |  |  |  |  |  |  |  |  |  |  |  |
| -0.7901 | 22 | igd | 0.0127 | A | \|\| id:ebi-a-GCST90001710 | ebi-a-GCST90001710 | TRUE | G | 40044235 | 2.58E-12 | reported | 3657 | 0.1125 | rs77273762 |  |  |  |  |  |  |  |  |  |  |  |  |  |  |  |
| -0.7609 | 22 | igd | 0.0127 | A | \|\| id:ebi-a-GCST90001711 | ebi-a-GCST90001711 | TRUE | G | 40044235 | 1.45E-11 | reported | 3657 | 0.1123 | rs77273762 |  |  |  |  |  |  |  |  |  |  |  |  |  |  |  |
| 0.6341 | 2 | igd | 0.0127 | T | \|\| id:ebi-a-GCST90002098 | ebi-a-GCST90002098 | TRUE | C | 37634429 | 2.78E-08 | reported | 2910 | 0.1138 | rs62133303 |  |  |  |  |  |  |  |  |  |  |  |  |  |  |  |
| 0.6944 | 10 | igd | 0.0126 | T | \|\| id:ebi-a-GCST90001495 | ebi-a-GCST90001495 | TRUE | A | 104624083 | 7.29E-09 | reported | 3405 | 0.1197 | rs142871406 |  |  |  |  |  |  |  |  |  |  |  |  |  |  |  |
| 0.7169 | 10 | igd | 0.0126 | T | \|\| id:ebi-a-GCST90001660 | ebi-a-GCST90001660 | TRUE | A | 104624083 | 1.53E-09 | reported | 3408 | 0.1184 | rs142871406 |  |  |  |  |  |  |  |  |  |  |  |  |  |  |  |
| 93.82 | 5 | igd | 0.0126 | A | \|\| id:ebi-a-GCST90001698 | ebi-a-GCST90001698 | TRUE | T | 180502672 | 1.12E-08 | reported | 3408 | 16.39 | rs13360609 |  |  |  |  |  |  |  |  |  |  |  |  |  |  |  |
| 0.682 | 10 | igd | 0.0125 | T | \|\| id:ebi-a-GCST90001660 | ebi-a-GCST90001660 | TRUE | C | 104312260 | 9.80E-09 | reported | 3408 | 0.1186 | rs147071786 |  |  |  |  |  |  |  |  |  |  |  |  |  |  |  |
| 74.28 | 6 | igd | 0.0125 | C | \|\| id:ebi-a-GCST90001695 | ebi-a-GCST90001695 | TRUE | A | 67374008 | 1.29E-10 | reported | 3408 | 11.52 | rs78939697 |  |  |  |  |  |  |  |  |  |  |  |  |  |  |  |
| -0.7606 | 12 | igd | 0.0125 | T | \|\| id:ebi-a-GCST90002073 | ebi-a-GCST90002073 | TRUE | C | 67990063 | 3.11E-09 | reported | 2850 | 0.1279 | rs76869634 |  |  |  |  |  |  |  |  |  |  |  |  |  |  |  |
| 0.6569 | 2 | igd | 0.0125 | G | \|\| id:ebi-a-GCST90002102 | ebi-a-GCST90002102 | TRUE | A | 37461691 | 1.28E-08 | reported | 2919 | 0.1151 | rs144589267 |  |  |  |  |  |  |  |  |  |  |  |  |  |  |  |
| 0.6536 | 10 | igd | 0.0124 | T | \|\| id:ebi-a-GCST90001658 | ebi-a-GCST90001658 | TRUE | C | 96986836 | 5.39E-09 | reported | 3440 | 0.1117 | rs140389196 |  |  |  |  |  |  |  |  |  |  |  |  |  |  |  |
| 0.6611 | 10 | igd | 0.0124 | T | \|\| id:ebi-a-GCST90001671 | ebi-a-GCST90001671 | TRUE | C | 96986836 | 1.50E-08 | reported | 3440 | 0.1165 | rs140389196 |  |  |  |  |  |  |  |  |  |  |  |  |  |  |  |
| -0.7164 | 6 | igd | 0.0124 | T | \|\| id:ebi-a-GCST90002105 | ebi-a-GCST90002105 | TRUE | A | 32862910 | 1.87E-08 | reported | 2872 | 0.127 | rs12195238 |  |  |  |  |  |  |  |  |  |  |  |  |  |  |  |
| 0.6784 | 10 | igd | 0.0123 | T | \|\| id:ebi-a-GCST90001660 | ebi-a-GCST90001660 | TRUE | C | 104444481 | 1.49E-08 | reported | 3408 | 0.1195 | rs181937854 |  |  |  |  |  |  |  |  |  |  |  |  |  |  |  |
| 120 | 5 | igd | 0.0123 | G | \|\| id:ebi-a-GCST90001698 | ebi-a-GCST90001698 | TRUE | C | 81877269 | 4.79E-14 | reported | 3408 | 15.85 | rs147896110 |  |  |  |  |  |  |  |  |  |  |  |  |  |  |  |
| 0.7439 | 10 | igd | 0.0123 | T | \|\| id:ebi-a-GCST90002030 | ebi-a-GCST90002030 | TRUE | C | 96986836 | 2.87E-09 | reported | 2920 | 0.1249 | rs140389196 |  |  |  |  |  |  |  |  |  |  |  |  |  |  |  |
| 0.741 | 10 | igd | 0.0123 | T | \|\| id:ebi-a-GCST90002031 | ebi-a-GCST90002031 | TRUE | C | 96986836 | 5.03E-09 | reported | 2920 | 0.1264 | rs140389196 |  |  |  |  |  |  |  |  |  |  |  |  |  |  |  |
| 0.7201 | 10 | igd | 0.0123 | T | \|\| id:ebi-a-GCST90002032 | ebi-a-GCST90002032 | TRUE | C | 96986836 | 1.38E-08 | reported | 2920 | 0.1265 | rs140389196 |  |  |  |  |  |  |  |  |  |  |  |  |  |  |  |
| -0.7413 | 1 | igd | 0.0122 | T | \|\| id:ebi-a-GCST90001693 | ebi-a-GCST90001693 | TRUE | C | 199808849 | 3.98E-10 | reported | 3408 | 0.1182 | rs11589644 |  |  |  |  |  |  |  |  |  |  |  |  |  |  |  |
| 90.23 | 11 | igd | 0.0122 | A | \|\| id:ebi-a-GCST90001698 | ebi-a-GCST90001698 | TRUE | G | 29902098 | 3.21E-08 | reported | 3408 | 16.28 | rs74540476 |  |  |  |  |  |  |  |  |  |  |  |  |  |  |  |
| 126 | 13 | igd | 0.0122 | C | \|\| id:ebi-a-GCST90001698 | ebi-a-GCST90001698 | TRUE | T | 114557883 | 3.05E-15 | reported | 3408 | 15.9 | rs59615051 |  |  |  |  |  |  |  |  |  |  |  |  |  |  |  |
| -0.7636 | 1 | igd | 0.0121 | T | \|\| id:ebi-a-GCST90001691 | ebi-a-GCST90001691 | TRUE | C | 199808849 | 1.00E-10 | reported | 3440 | 0.1177 | rs11589644 |  |  |  |  |  |  |  |  |  |  |  |  |  |  |  |
| 0.6631 | 10 | igd | 0.012 | C | \|\| id:ebi-a-GCST90001489 | ebi-a-GCST90001489 | TRUE | T | 103927711 | 4.77E-08 | reported | 3405 | 0.1212 | rs142835941 |  |  |  |  |  |  |  |  |  |  |  |  |  |  |  |
| 0.6902 | 10 | igd | 0.012 | C | \|\| id:ebi-a-GCST90001495 | ebi-a-GCST90001495 | TRUE | T | 103927711 | 1.74E-08 | reported | 3405 | 0.1222 | rs142835941 |  |  |  |  |  |  |  |  |  |  |  |  |  |  |  |
| 0.7527 | 10 | igd | 0.012 | C | \|\| id:ebi-a-GCST90001660 | ebi-a-GCST90001660 | TRUE | T | 103927711 | 4.85E-10 | reported | 3408 | 0.1206 | rs142835941 |  |  |  |  |  |  |  |  |  |  |  |  |  |  |  |
| -0.9373 | 22 | igd | 0.012 | A | \|\| id:ebi-a-GCST90001702 | ebi-a-GCST90001702 | TRUE | C | 40157631 | 7.50E-16 | reported | 3657 | 0.1157 | rs117119882 |  |  |  |  |  |  |  |  |  |  |  |  |  |  |  |
| -0.9235 | 22 | igd | 0.012 | A | \|\| id:ebi-a-GCST90001703 | ebi-a-GCST90001703 | TRUE | C | 40157631 | 1.98E-15 | reported | 3657 | 0.1158 | rs117119882 |  |  |  |  |  |  |  |  |  |  |  |  |  |  |  |
| -0.7817 | 22 | igd | 0.012 | A | \|\| id:ebi-a-GCST90001704 | ebi-a-GCST90001704 | TRUE | C | 40157631 | 1.78E-11 | reported | 3657 | 0.1159 | rs117119882 |  |  |  |  |  |  |  |  |  |  |  |  |  |  |  |
| -0.9009 | 22 | igd | 0.012 | A | \|\| id:ebi-a-GCST90001710 | ebi-a-GCST90001710 | TRUE | C | 40157631 | 7.93E-15 | reported | 3657 | 0.1155 | rs117119882 |  |  |  |  |  |  |  |  |  |  |  |  |  |  |  |
| -0.8608 | 22 | igd | 0.012 | A | \|\| id:ebi-a-GCST90001711 | ebi-a-GCST90001711 | TRUE | C | 40157631 | 1.04E-13 | reported | 3657 | 0.1153 | rs117119882 |  |  |  |  |  |  |  |  |  |  |  |  |  |  |  |
| 0.9407 | 10 | igd | 0.012 | A | \|\| id:ebi-a-GCST90002061 | ebi-a-GCST90002061 | TRUE | G | 97255916 | 3.11E-14 | reported | 2920 | 0.1233 | rs189723298 |  |  |  |  |  |  |  |  |  |  |  |  |  |  |  |
| 0.714 | 6 | igd | 0.012 | T | \|\| id:ebi-a-GCST90002105 | ebi-a-GCST90002105 | TRUE | C | 24422292 | 4.21E-08 | reported | 2872 | 0.1299 | rs111608618 |  |  |  |  |  |  |  |  |  |  |  |  |  |  |  |
| 0.6556 | 1 | igd | 0.0119 | T | \|\| id:ebi-a-GCST90001485 | ebi-a-GCST90001485 | TRUE | C | 199808849 | 1.39E-08 | reported | 3437 | 0.1153 | rs11589644 |  |  |  |  |  |  |  |  |  |  |  |  |  |  |  |
| 0.6921 | 1 | igd | 0.0119 | T | \|\| id:ebi-a-GCST90001508 | ebi-a-GCST90001508 | TRUE | C | 199808849 | 6.88E-09 | reported | 3437 | 0.1191 | rs11589644 |  |  |  |  |  |  |  |  |  |  |  |  |  |  |  |
| 0.6755 | 1 | igd | 0.0119 | T | \|\| id:ebi-a-GCST90001509 | ebi-a-GCST90001509 | TRUE | C | 199808849 | 1.53E-08 | reported | 3437 | 0.1191 | rs11589644 |  |  |  |  |  |  |  |  |  |  |  |  |  |  |  |
| -0.7196 | 10 | igd | 0.0116 | T | \|\| id:ebi-a-GCST90001496 | ebi-a-GCST90001496 | TRUE | C | 97907924 | 3.38E-09 | reported | 3437 | 0.1214 | rs150160533 |  |  |  |  |  |  |  |  |  |  |  |  |  |  |  |
| -0.695 | 10 | igd | 0.0116 | T | \|\| id:ebi-a-GCST90001497 | ebi-a-GCST90001497 | TRUE | C | 97907924 | 7.39E-09 | reported | 3437 | 0.1199 | rs150160533 |  |  |  |  |  |  |  |  |  |  |  |  |  |  |  |
| -0.7682 | 10 | igd | 0.0116 | C | \|\| id:ebi-a-GCST90001672 | ebi-a-GCST90001672 | TRUE | G | 92822294 | 3.53E-10 | reported | 3408 | 0.1221 | rs79802990 |  |  |  |  |  |  |  |  |  |  |  |  |  |  |  |
| 72.93 | 6 | igd | 0.0116 | G | \|\| id:ebi-a-GCST90001695 | ebi-a-GCST90001695 | TRUE | A | 70784408 | 1.02E-08 | reported | 3408 | 12.7 | rs12192395 |  |  |  |  |  |  |  |  |  |  |  |  |  |  |  |
| 130.7 | 7 | igd | 0.0116 | A | \|\| id:ebi-a-GCST90001698 | ebi-a-GCST90001698 | TRUE | G | 113679246 | 5.64E-14 | reported | 3408 | 17.31 | rs73206236 |  |  |  |  |  |  |  |  |  |  |  |  |  |  |  |
| 99.34 | 17 | igd | 0.0116 | A | \|\| id:ebi-a-GCST90001698 | ebi-a-GCST90001698 | TRUE | G | 65896989 | 5.33E-09 | reported | 3408 | 16.98 | rs79757812 |  |  |  |  |  |  |  |  |  |  |  |  |  |  |  |
| -0.6961 | 4 | igd | 0.0116 | A | \|\| id:ebi-a-GCST90001811 | ebi-a-GCST90001811 | TRUE | G | 15967933 | 7.17E-09 | reported | 3656 | 0.12 | rs115832613 |  |  |  |  |  |  |  |  |  |  |  |  |  |  |  |
| -0.8923 | 4 | igd | 0.0116 | A | \|\| id:ebi-a-GCST90001815 | ebi-a-GCST90001815 | TRUE | G | 15967933 | 2.44E-14 | reported | 3657 | 0.1165 | rs115832613 |  |  |  |  |  |  |  |  |  |  |  |  |  |  |  |
| -0.6899 | 10 | igd | 0.0115 | T | \|\| id:ebi-a-GCST90001495 | ebi-a-GCST90001495 | TRUE | C | 97907924 | 2.37E-08 | reported | 3405 | 0.1233 | rs150160533 |  |  |  |  |  |  |  |  |  |  |  |  |  |  |  |
| -0.6664 | 10 | igd | 0.0115 | C | \|\| id:ebi-a-GCST90001658 | ebi-a-GCST90001658 | TRUE | G | 92822294 | 9.14E-09 | reported | 3440 | 0.1157 | rs79802990 |  |  |  |  |  |  |  |  |  |  |  |  |  |  |  |
| -0.6559 | 10 | igd | 0.0115 | C | \|\| id:ebi-a-GCST90001659 | ebi-a-GCST90001659 | TRUE | G | 92822294 | 1.56E-08 | reported | 3440 | 0.1157 | rs79802990 |  |  |  |  |  |  |  |  |  |  |  |  |  |  |  |
| -0.7928 | 10 | igd | 0.0115 | C | \|\| id:ebi-a-GCST90001670 | ebi-a-GCST90001670 | TRUE | G | 92822294 | 5.10E-11 | reported | 3440 | 0.1203 | rs79802990 |  |  |  |  |  |  |  |  |  |  |  |  |  |  |  |
| -0.7682 | 10 | igd | 0.0115 | C | \|\| id:ebi-a-GCST90001671 | ebi-a-GCST90001671 | TRUE | G | 92822294 | 2.04E-10 | reported | 3440 | 0.1205 | rs79802990 |  |  |  |  |  |  |  |  |  |  |  |  |  |  |  |
| 0.6562 | 10 | igd | 0.0114 | A | \|\| id:ebi-a-GCST90001672 | ebi-a-GCST90001672 | TRUE | C | 98037332 | 3.30E-08 | reported | 3408 | 0.1185 | rs80254417 |  |  |  |  |  |  |  |  |  |  |  |  |  |  |  |
| 74.57 | 6 | igd | 0.0114 | T | \|\| id:ebi-a-GCST90001695 | ebi-a-GCST90001695 | TRUE | C | 66337344 | 9.44E-09 | reported | 3408 | 12.96 | rs139174916 |  |  |  |  |  |  |  |  |  |  |  |  |  |  |  |
| 114 | 4 | igd | 0.0114 | T | \|\| id:ebi-a-GCST90001698 | ebi-a-GCST90001698 | TRUE | C | 49518806 | 5.42E-09 | reported | 3408 | 19.49 | rs541823325 |  |  |  |  |  |  |  |  |  |  |  |  |  |  |  |
| 95.85 | 10 | igd | 0.0114 | A | \|\| id:ebi-a-GCST90001698 | ebi-a-GCST90001698 | TRUE | G | 91013835 | 7.06E-09 | reported | 3408 | 16.51 | rs112517700 |  |  |  |  |  |  |  |  |  |  |  |  |  |  |  |
| 0.7228 | 10 | igd | 0.0113 | C | \|\| id:ebi-a-GCST90001660 | ebi-a-GCST90001660 | TRUE | T | 104775045 | 1.25E-08 | reported | 3408 | 0.1267 | rs149619000 |  |  |  |  |  |  |  |  |  |  |  |  |  |  |  |
| 0.7652 | 10 | igd | 0.0113 | A | \|\| id:ebi-a-GCST90001670 | ebi-a-GCST90001670 | TRUE | C | 98037332 | 7.02E-11 | reported | 3440 | 0.117 | rs80254417 |  |  |  |  |  |  |  |  |  |  |  |  |  |  |  |
| 0.7142 | 10 | igd | 0.0113 | A | \|\| id:ebi-a-GCST90001671 | ebi-a-GCST90001671 | TRUE | C | 98037332 | 1.13E-09 | reported | 3440 | 0.117 | rs80254417 |  |  |  |  |  |  |  |  |  |  |  |  |  |  |  |
| 98.72 | 2 | igd | 0.0113 | G | \|\| id:ebi-a-GCST90001698 | ebi-a-GCST90001698 | TRUE | A | 42107980 | 2.08E-08 | reported | 3408 | 17.57 | rs13010105 |  |  |  |  |  |  |  |  |  |  |  |  |  |  |  |
| 110.2 | 5 | igd | 0.0113 | T | \|\| id:ebi-a-GCST90001698 | ebi-a-GCST90001698 | TRUE | C | 120420356 | 6.99E-11 | reported | 3408 | 16.85 | rs140976870 |  |  |  |  |  |  |  |  |  |  |  |  |  |  |  |
| 95.63 | 5 | igd | 0.0112 | G | \|\| id:ebi-a-GCST90001698 | ebi-a-GCST90001698 | TRUE | T | 180055849 | 1.46E-08 | reported | 3408 | 16.84 | rs75361132 |  |  |  |  |  |  |  |  |  |  |  |  |  |  |  |
| 101.5 | 11 | igd | 0.0112 | A | \|\| id:ebi-a-GCST90001698 | ebi-a-GCST90001698 | TRUE | G | 110616516 | 2.07E-09 | reported | 3408 | 16.89 | rs117343355 |  |  |  |  |  |  |  |  |  |  |  |  |  |  |  |
| -1.076 | 22 | igd | 0.0111 | C | \|\| id:ebi-a-GCST90001702 | ebi-a-GCST90001702 | TRUE | G | 40567889 | 3.10E-18 | reported | 3657 | 0.1229 | rs117003628 |  |  |  |  |  |  |  |  |  |  |  |  |  |  |  |
| -1.056 | 22 | igd | 0.0111 | C | \|\| id:ebi-a-GCST90001703 | ebi-a-GCST90001703 | TRUE | G | 40567889 | 1.31E-17 | reported | 3657 | 0.123 | rs117003628 |  |  |  |  |  |  |  |  |  |  |  |  |  |  |  |
| -0.8987 | 22 | igd | 0.0111 | C | \|\| id:ebi-a-GCST90001704 | ebi-a-GCST90001704 | TRUE | G | 40567889 | 3.58E-13 | reported | 3657 | 0.1231 | rs117003628 |  |  |  |  |  |  |  |  |  |  |  |  |  |  |  |
| -1.005 | 22 | igd | 0.0111 | A | \|\| id:ebi-a-GCST90001710 | ebi-a-GCST90001710 | TRUE | G | 40568020 | 3.54E-16 | reported | 3657 | 0.1227 | rs528009599 |  |  |  |  |  |  |  |  |  |  |  |  |  |  |  |
| -0.9648 | 22 | igd | 0.0111 | C | \|\| id:ebi-a-GCST90001711 | ebi-a-GCST90001711 | TRUE | G | 40567889 | 4.54E-15 | reported | 3657 | 0.1226 | rs117003628 |  |  |  |  |  |  |  |  |  |  |  |  |  |  |  |
| 0.7478 | 10 | igd | 0.011 | A | \|\| id:ebi-a-GCST90001495 | ebi-a-GCST90001495 | TRUE | T | 104958176 | 2.48E-08 | reported | 3405 | 0.1338 | rs531275752 |  |  |  |  |  |  |  |  |  |  |  |  |  |  |  |
| 0.776 | 10 | igd | 0.011 | T | \|\| id:ebi-a-GCST90001660 | ebi-a-GCST90001660 | TRUE | C | 104958173 | 4.81E-09 | reported | 3408 | 0.1322 | rs551935239 |  |  |  |  |  |  |  |  |  |  |  |  |  |  |  |
| -0.9619 | 10 | igd | 0.011 | C | \|\| id:ebi-a-GCST90001961 | ebi-a-GCST90001961 | TRUE | G | 97687049 | 1.43E-13 | reported | 2920 | 0.1295 | rs41291582 |  |  |  |  |  |  |  |  |  |  |  |  |  |  |  |
| 0.8817 | 10 | igd | 0.011 | C | \|\| id:ebi-a-GCST90002061 | ebi-a-GCST90002061 | TRUE | G | 97687049 | 1.99E-11 | reported | 2920 | 0.131 | rs41291582 |  |  |  |  |  |  |  |  |  |  |  |  |  |  |  |
| 146.6 | 5 | igd | 0.0109 | G | \|\| id:ebi-a-GCST90001698 | ebi-a-GCST90001698 | TRUE | A | 81624749 | 1.65E-17 | reported | 3408 | 17.12 | rs115574834 |  |  |  |  |  |  |  |  |  |  |  |  |  |  |  |
| -0.7651 | 10 | igd | 0.0108 | A | \|\| id:ebi-a-GCST90001496 | ebi-a-GCST90001496 | TRUE | G | 98805782 | 1.32E-09 | reported | 3437 | 0.1258 | rs117257558 |  |  |  |  |  |  |  |  |  |  |  |  |  |  |  |
| -0.7069 | 10 | igd | 0.0108 | A | \|\| id:ebi-a-GCST90001497 | ebi-a-GCST90001497 | TRUE | G | 98805782 | 1.40E-08 | reported | 3437 | 0.1243 | rs117257558 |  |  |  |  |  |  |  |  |  |  |  |  |  |  |  |
| 147 | 5 | igd | 0.0107 | T | \|\| id:ebi-a-GCST90001698 | ebi-a-GCST90001698 | TRUE | C | 81461758 | 3.45E-17 | reported | 3408 | 17.34 | rs114802738 |  |  |  |  |  |  |  |  |  |  |  |  |  |  |  |
| 107.7 | 16 | igd | 0.0107 | T | \|\| id:ebi-a-GCST90001698 | ebi-a-GCST90001698 | TRUE | C | 7218981 | 7.35E-10 | reported | 3408 | 17.44 | rs59673378 |  |  |  |  |  |  |  |  |  |  |  |  |  |  |  |
| 106.4 | 11 | igd | 0.0106 | A | \|\| id:ebi-a-GCST90001698 | ebi-a-GCST90001698 | TRUE | T | 43037421 | 1.07E-09 | reported | 3408 | 17.4 | rs201928503 |  |  |  |  |  |  |  |  |  |  |  |  |  |  |  |
| 116.2 | 16 | igd | 0.0106 | C | \|\| id:ebi-a-GCST90001698 | ebi-a-GCST90001698 | TRUE | G | 25931790 | 1.25E-10 | reported | 3408 | 18 | rs34265859 |  |  |  |  |  |  |  |  |  |  |  |  |  |  |  |
| 0.7412 | 1 | igd | 0.0105 | T | \|\| id:ebi-a-GCST90002098 | ebi-a-GCST90002098 | TRUE | G | 199039653 | 2.45E-09 | reported | 2910 | 0.1239 | rs111992639 |  |  |  |  |  |  |  |  |  |  |  |  |  |  |  |
| 0.6799 | 1 | igd | 0.0104 | T | \|\| id:ebi-a-GCST90001483 | ebi-a-GCST90001483 | TRUE | G | 199039653 | 1.31E-08 | reported | 3405 | 0.1193 | rs111992639 |  |  |  |  |  |  |  |  |  |  |  |  |  |  |  |
| 0.6123 | 1 | igd | 0.0104 | T | \|\| id:ebi-a-GCST90001666 | ebi-a-GCST90001666 | TRUE | G | 199039653 | 1.49E-08 | reported | 3408 | 0.1079 | rs111992639 |  |  |  |  |  |  |  |  |  |  |  |  |  |  |  |
| -0.6604 | 1 | igd | 0.0104 | T | \|\| id:ebi-a-GCST90001669 | ebi-a-GCST90001669 | TRUE | G | 199039653 | 1.69E-09 | reported | 3408 | 0.1093 | rs111992639 |  |  |  |  |  |  |  |  |  |  |  |  |  |  |  |
| -0.6925 | 1 | igd | 0.0104 | T | \|\| id:ebi-a-GCST90001693 | ebi-a-GCST90001693 | TRUE | G | 199039653 | 2.13E-08 | reported | 3408 | 0.1233 | rs111992639 |  |  |  |  |  |  |  |  |  |  |  |  |  |  |  |
| 101.3 | 5 | igd | 0.0104 | C | \|\| id:ebi-a-GCST90001698 | ebi-a-GCST90001698 | TRUE | T | 158110928 | 1.51E-08 | reported | 3408 | 17.85 | rs182750448 |  |  |  |  |  |  |  |  |  |  |  |  |  |  |  |
| 101.5 | 13 | igd | 0.0104 | C | \|\| id:ebi-a-GCST90001698 | ebi-a-GCST90001698 | TRUE | T | 113631761 | 1.05E-08 | reported | 3408 | 17.69 | rs75177063 |  |  |  |  |  |  |  |  |  |  |  |  |  |  |  |
| 0.7877 | 1 | igd | 0.0104 | T | \|\| id:ebi-a-GCST90001832 | ebi-a-GCST90001832 | TRUE | A | 169712675 | 2.24E-10 | reported | 2872 | 0.1237 | rs114934710 |  |  |  |  |  |  |  |  |  |  |  |  |  |  |  |
| 0.7964 | 1 | igd | 0.0103 | T | \|\| id:ebi-a-GCST90001485 | ebi-a-GCST90001485 | TRUE | G | 199039653 | 2.36E-11 | reported | 3437 | 0.1188 | rs111992639 |  |  |  |  |  |  |  |  |  |  |  |  |  |  |  |
| 0.7415 | 1 | igd | 0.0103 | T | \|\| id:ebi-a-GCST90001508 | ebi-a-GCST90001508 | TRUE | G | 199039653 | 2.40E-09 | reported | 3437 | 0.1239 | rs111992639 |  |  |  |  |  |  |  |  |  |  |  |  |  |  |  |
| 0.728 | 1 | igd | 0.0103 | T | \|\| id:ebi-a-GCST90001509 | ebi-a-GCST90001509 | TRUE | G | 199039653 | 4.56E-09 | reported | 3437 | 0.1239 | rs111992639 |  |  |  |  |  |  |  |  |  |  |  |  |  |  |  |
| 0.7461 | 1 | igd | 0.0103 | T | \|\| id:ebi-a-GCST90001664 | ebi-a-GCST90001664 | TRUE | G | 199039653 | 4.62E-11 | reported | 3440 | 0.113 | rs111992639 |  |  |  |  |  |  |  |  |  |  |  |  |  |  |  |
| 0.6717 | 1 | igd | 0.0103 | T | \|\| id:ebi-a-GCST90001665 | ebi-a-GCST90001665 | TRUE | G | 199039653 | 2.83E-08 | reported | 3440 | 0.1207 | rs111992639 |  |  |  |  |  |  |  |  |  |  |  |  |  |  |  |
| -0.6682 | 1 | igd | 0.0103 | T | \|\| id:ebi-a-GCST90001668 | ebi-a-GCST90001668 | TRUE | G | 199039653 | 3.15E-08 | reported | 3440 | 0.1205 | rs111992639 |  |  |  |  |  |  |  |  |  |  |  |  |  |  |  |
| -0.7447 | 2 | igd | 0.0103 | A | \|\| id:ebi-a-GCST90001680 | ebi-a-GCST90001680 | TRUE | G | 54970478 | 3.00E-09 | reported | 3162 | 0.1252 | rs72915529 |  |  |  |  |  |  |  |  |  |  |  |  |  |  |  |
| 100.8 | 2 | igd | 0.0103 | T | \|\| id:ebi-a-GCST90001698 | ebi-a-GCST90001698 | TRUE | C | 39184018 | 1.27E-08 | reported | 3408 | 17.68 | rs75054940 |  |  |  |  |  |  |  |  |  |  |  |  |  |  |  |
| 101.4 | 6 | igd | 0.0103 | A | \|\| id:ebi-a-GCST90001698 | ebi-a-GCST90001698 | TRUE | G | 90900544 | 7.68E-09 | reported | 3408 | 17.52 | rs78275221 |  |  |  |  |  |  |  |  |  |  |  |  |  |  |  |
| 118.5 | 14 | igd | 0.0103 | T | \|\| id:ebi-a-GCST90001698 | ebi-a-GCST90001698 | TRUE | C | 99653123 | 1.86E-11 | reported | 3408 | 17.59 | rs10484068 |  |  |  |  |  |  |  |  |  |  |  |  |  |  |  |
| 0.7791 | 1 | igd | 0.0103 | A | \|\| id:ebi-a-GCST90001832 | ebi-a-GCST90001832 | TRUE | G | 169865920 | 6.00E-10 | reported | 2872 | 0.1254 | rs114190066 |  |  |  |  |  |  |  |  |  |  |  |  |  |  |  |
| 0.7846 | 6 | igd | 0.0103 | T | \|\| id:ebi-a-GCST90002105 | ebi-a-GCST90002105 | TRUE | C | 31219650 | 5.20E-09 | reported | 2872 | 0.1339 | rs116227766 |  |  |  |  |  |  |  |  |  |  |  |  |  |  |  |
| -0.9308 | 10 | igd | 0.0102 | T | \|\| id:ebi-a-GCST90001496 | ebi-a-GCST90001496 | TRUE | C | 97771420 | 3.86E-10 | reported | 3437 | 0.1483 | rs117048108 |  |  |  |  |  |  |  |  |  |  |  |  |  |  |  |
| -0.9068 | 10 | igd | 0.0102 | T | \|\| id:ebi-a-GCST90001497 | ebi-a-GCST90001497 | TRUE | C | 97771420 | 6.61E-10 | reported | 3437 | 0.1464 | rs117048108 |  |  |  |  |  |  |  |  |  |  |  |  |  |  |  |
| 1.178 | 10 | igd | 0.0102 | C | \|\| id:ebi-a-GCST90001670 | ebi-a-GCST90001670 | TRUE | G | 97687049 | 2.42E-22 | reported | 3440 | 0.1204 | rs41291582 |  |  |  |  |  |  |  |  |  |  |  |  |  |  |  |
| 1.168 | 10 | igd | 0.0102 | C | \|\| id:ebi-a-GCST90001671 | ebi-a-GCST90001671 | TRUE | G | 97687049 | 5.07E-22 | reported | 3440 | 0.1202 | rs41291582 |  |  |  |  |  |  |  |  |  |  |  |  |  |  |  |
| 1.102 | 10 | igd | 0.0101 | C | \|\| id:ebi-a-GCST90001672 | ebi-a-GCST90001672 | TRUE | G | 97687049 | 4.96E-19 | reported | 3408 | 0.1229 | rs41291582 |  |  |  |  |  |  |  |  |  |  |  |  |  |  |  |
| -0.9155 | 10 | igd | 0.01 | T | \|\| id:ebi-a-GCST90001495 | ebi-a-GCST90001495 | TRUE | C | 97771420 | 1.31E-09 | reported | 3405 | 0.1505 | rs117048108 |  |  |  |  |  |  |  |  |  |  |  |  |  |  |  |
| -0.8488 | 10 | igd | 0.01 | T | \|\| id:ebi-a-GCST90001660 | ebi-a-GCST90001660 | TRUE | C | 97771420 | 1.17E-08 | reported | 3408 | 0.1485 | rs117048108 |  |  |  |  |  |  |  |  |  |  |  |  |  |  |  |
| 0.6482 | 1 | igd | 0.01 | A | \|\| id:ebi-a-GCST90001664 | ebi-a-GCST90001664 | TRUE | G | 199471227 | 1.60E-08 | reported | 3440 | 0.1144 | rs190023756 |  |  |  |  |  |  |  |  |  |  |  |  |  |  |  |
| 159.5 | 5 | igd | 0.01 | C | \|\| id:ebi-a-GCST90001698 | ebi-a-GCST90001698 | TRUE | T | 81311763 | 4.20E-18 | reported | 3408 | 18.29 | rs144613377 |  |  |  |  |  |  |  |  |  |  |  |  |  |  |  |
| 122.6 | 11 | igd | 0.01 | T | \|\| id:ebi-a-GCST90001698 | ebi-a-GCST90001698 | TRUE | C | 109281106 | 1.42E-11 | reported | 3408 | 18.08 | rs189796120 |  |  |  |  |  |  |  |  |  |  |  |  |  |  |  |
| 4.47 | 11 | igd | 0.0099 | T | \|\| id:ebi-a-GCST90001700 | ebi-a-GCST90001700 | TRUE | C | 109281106 | 5.14E-09 | reported | 3425 | 0.763 | rs189796120 |  |  |  |  |  |  |  |  |  |  |  |  |  |  |  |
| -0.788 | 6 | igd | 0.0099 | T | \|\| id:ebi-a-GCST90002105 | ebi-a-GCST90002105 | TRUE | A | 30658508 | 3.16E-08 | reported | 2872 | 0.142 | rs114089471 |  |  |  |  |  |  |  |  |  |  |  |  |  |  |  |
| 75.05 | 8 | igd | 0.0098 | A | \|\| id:ebi-a-GCST90001695 | ebi-a-GCST90001695 | TRUE | G | 142607179 | 8.29E-09 | reported | 3408 | 12.99 | rs62513410 |  |  |  |  |  |  |  |  |  |  |  |  |  |  |  |
| -1.001 | 19 | igd | 0.0098 | G | \|\| id:ebi-a-GCST90001948 | ebi-a-GCST90001948 | TRUE | A | 51572403 | 1.69E-08 | reported | 1633 | 0.1765 | rs79321884 |  |  |  |  |  |  |  |  |  |  |  |  |  |  |  |
| -0.9978 | 19 | igd | 0.0098 | G | \|\| id:ebi-a-GCST90001949 | ebi-a-GCST90001949 | TRUE | A | 51572403 | 1.79E-08 | reported | 1634 | 0.1763 | rs79321884 |  |  |  |  |  |  |  |  |  |  |  |  |  |  |  |
| 132.5 | 7 | igd | 0.0097 | A | \|\| id:ebi-a-GCST90001698 | ebi-a-GCST90001698 | TRUE | G | 158334355 | 2.61E-14 | reported | 3408 | 17.32 | rs78470553 |  |  |  |  |  |  |  |  |  |  |  |  |  |  |  |
| 0.788 | 10 | igd | 0.0096 | A | \|\| id:ebi-a-GCST90001484 | ebi-a-GCST90001484 | TRUE | C | 97681928 | 1.11E-08 | reported | 3437 | 0.1376 | rs77824252 |  |  |  |  |  |  |  |  |  |  |  |  |  |  |  |
| 0.7962 | 10 | igd | 0.0096 | A | \|\| id:ebi-a-GCST90001496 | ebi-a-GCST90001496 | TRUE | C | 97681928 | 1.74E-08 | reported | 3437 | 0.1409 | rs77824252 |  |  |  |  |  |  |  |  |  |  |  |  |  |  |  |
| 0.8874 | 10 | igd | 0.0096 | A | \|\| id:ebi-a-GCST90001497 | ebi-a-GCST90001497 | TRUE | C | 97681928 | 1.96E-10 | reported | 3437 | 0.139 | rs77824252 |  |  |  |  |  |  |  |  |  |  |  |  |  |  |  |
| 0.7787 | 10 | igd | 0.0096 | A | \|\| id:ebi-a-GCST90001658 | ebi-a-GCST90001658 | TRUE | C | 97681928 | 6.76E-09 | reported | 3440 | 0.134 | rs77824252 |  |  |  |  |  |  |  |  |  |  |  |  |  |  |  |
| 0.7491 | 10 | igd | 0.0096 | A | \|\| id:ebi-a-GCST90001659 | ebi-a-GCST90001659 | TRUE | C | 97681928 | 2.48E-08 | reported | 3440 | 0.1341 | rs77824252 |  |  |  |  |  |  |  |  |  |  |  |  |  |  |  |
| -0.7401 | 2 | igd | 0.0096 | A | \|\| id:ebi-a-GCST90001679 | ebi-a-GCST90001679 | TRUE | G | 54957709 | 1.37E-08 | reported | 3162 | 0.13 | rs78667279 |  |  |  |  |  |  |  |  |  |  |  |  |  |  |  |
| -0.7492 | 2 | igd | 0.0096 | A | \|\| id:ebi-a-GCST90001681 | ebi-a-GCST90001681 | TRUE | G | 54957709 | 1.32E-08 | reported | 3130 | 0.1315 | rs78667279 |  |  |  |  |  |  |  |  |  |  |  |  |  |  |  |
| -0.8641 | 22 | igd | 0.0096 | A | \|\| id:ebi-a-GCST90001702 | ebi-a-GCST90001702 | TRUE | G | 39821998 | 3.77E-10 | reported | 3657 | 0.1376 | rs9607646 |  |  |  |  |  |  |  |  |  |  |  |  |  |  |  |
| -0.8465 | 22 | igd | 0.0096 | A | \|\| id:ebi-a-GCST90001703 | ebi-a-GCST90001703 | TRUE | G | 39821998 | 8.50E-10 | reported | 3657 | 0.1376 | rs9607646 |  |  |  |  |  |  |  |  |  |  |  |  |  |  |  |
| -0.8449 | 22 | igd | 0.0096 | A | \|\| id:ebi-a-GCST90001710 | ebi-a-GCST90001710 | TRUE | G | 39821998 | 8.21E-10 | reported | 3657 | 0.1372 | rs9607646 |  |  |  |  |  |  |  |  |  |  |  |  |  |  |  |
| -0.8153 | 22 | igd | 0.0096 | A | \|\| id:ebi-a-GCST90001711 | ebi-a-GCST90001711 | TRUE | G | 39821998 | 2.89E-09 | reported | 3657 | 0.137 | rs9607646 |  |  |  |  |  |  |  |  |  |  |  |  |  |  |  |
| 0.866 | 10 | igd | 0.0096 | A | \|\| id:ebi-a-GCST90002030 | ebi-a-GCST90002030 | TRUE | C | 97681928 | 5.83E-09 | reported | 2920 | 0.1483 | rs77824252 |  |  |  |  |  |  |  |  |  |  |  |  |  |  |  |
| 0.7292 | 10 | igd | 0.0095 | G | \|\| id:ebi-a-GCST90001496 | ebi-a-GCST90001496 | TRUE | A | 97204949 | 2.35E-08 | reported | 3437 | 0.1303 | rs118112011 |  |  |  |  |  |  |  |  |  |  |  |  |  |  |  |
| 104.8 | 1 | igd | 0.0095 | C | \|\| id:ebi-a-GCST90001698 | ebi-a-GCST90001698 | TRUE | T | 35892939 | 1.97E-08 | reported | 3408 | 18.61 | rs75255396 |  |  |  |  |  |  |  |  |  |  |  |  |  |  |  |
| -0.7593 | 10 | igd | 0.0094 | A | \|\| id:ebi-a-GCST90001672 | ebi-a-GCST90001672 | TRUE | G | 92029720 | 2.54E-08 | reported | 3408 | 0.136 | rs111954194 |  |  |  |  |  |  |  |  |  |  |  |  |  |  |  |
| 105.4 | 1 | igd | 0.0094 | T | \|\| id:ebi-a-GCST90001698 | ebi-a-GCST90001698 | TRUE | C | 36001984 | 2.05E-08 | reported | 3408 | 18.75 | rs114350985 |  |  |  |  |  |  |  |  |  |  |  |  |  |  |  |
| 114.2 | 7 | igd | 0.0094 | T | \|\| id:ebi-a-GCST90001698 | ebi-a-GCST90001698 | TRUE | C | 37590076 | 4.11E-10 | reported | 3408 | 18.22 | rs147681963 |  |  |  |  |  |  |  |  |  |  |  |  |  |  |  |
| 105.3 | 11 | igd | 0.0094 | A | \|\| id:ebi-a-GCST90001698 | ebi-a-GCST90001698 | TRUE | G | 33121166 | 1.18E-08 | reported | 3408 | 18.42 | rs78085398 |  |  |  |  |  |  |  |  |  |  |  |  |  |  |  |
| 99.95 | 20 | igd | 0.0094 | A | \|\| id:ebi-a-GCST90001698 | ebi-a-GCST90001698 | TRUE | G | 47823538 | 4.03E-08 | reported | 3408 | 18.17 | rs148827835 |  |  |  |  |  |  |  |  |  |  |  |  |  |  |  |
| -0.7283 | 10 | igd | 0.0093 | A | \|\| id:ebi-a-GCST90001658 | ebi-a-GCST90001658 | TRUE | G | 92029720 | 1.69E-08 | reported | 3440 | 0.1288 | rs111954194 |  |  |  |  |  |  |  |  |  |  |  |  |  |  |  |
| -0.811 | 10 | igd | 0.0093 | A | \|\| id:ebi-a-GCST90001670 | ebi-a-GCST90001670 | TRUE | G | 92029720 | 1.57E-09 | reported | 3440 | 0.134 | rs111954194 |  |  |  |  |  |  |  |  |  |  |  |  |  |  |  |
| -0.7851 | 10 | igd | 0.0093 | A | \|\| id:ebi-a-GCST90001671 | ebi-a-GCST90001671 | TRUE | G | 92029720 | 5.36E-09 | reported | 3440 | 0.1342 | rs111954194 |  |  |  |  |  |  |  |  |  |  |  |  |  |  |  |
| 0.8402 | 1 | igd | 0.0093 | C | \|\| id:ebi-a-GCST90002098 | ebi-a-GCST90002098 | TRUE | T | 199163362 | 3.66E-10 | reported | 2910 | 0.1336 | rs112009416 |  |  |  |  |  |  |  |  |  |  |  |  |  |  |  |
| 124.1 | 10 | igd | 0.0092 | C | \|\| id:ebi-a-GCST90001698 | ebi-a-GCST90001698 | TRUE | T | 16995517 | 2.08E-10 | reported | 3408 | 19.47 | rs145743664 |  |  |  |  |  |  |  |  |  |  |  |  |  |  |  |
| 111.8 | 19 | igd | 0.0092 | G | \|\| id:ebi-a-GCST90001698 | ebi-a-GCST90001698 | TRUE | A | 1191667 | 6.12E-10 | reported | 3408 | 18.01 | rs143871291 |  |  |  |  |  |  |  |  |  |  |  |  |  |  |  |
| 4.521 | 6 | igd | 0.0092 | G | \|\| id:ebi-a-GCST90001700 | ebi-a-GCST90001700 | TRUE | A | 165166578 | 2.04E-08 | reported | 3425 | 0.8043 | rs375356899 |  |  |  |  |  |  |  |  |  |  |  |  |  |  |  |
| -1.072 | 17 | igd | 0.0092 | T | \|\| id:ebi-a-GCST90001926 | ebi-a-GCST90001926 | TRUE | C | 55921141 | 6.60E-14 | reported | 2920 | 0.1424 | rs139095969 |  |  |  |  |  |  |  |  |  |  |  |  |  |  |  |
| 0.803 | 6 | igd | 0.0092 | G | \|\| id:ebi-a-GCST90002105 | ebi-a-GCST90002105 | TRUE | A | 32704266 | 5.86E-09 | reported | 2872 | 0.1375 | rs7762228 |  |  |  |  |  |  |  |  |  |  |  |  |  |  |  |
| -0.8838 | 1 | igd | 0.0091 | C | \|\| id:ebi-a-GCST90001669 | ebi-a-GCST90001669 | TRUE | T | 199163362 | 1.07E-13 | reported | 3408 | 0.1184 | rs112009416 |  |  |  |  |  |  |  |  |  |  |  |  |  |  |  |
| -0.8134 | 1 | igd | 0.0091 | C | \|\| id:ebi-a-GCST90001693 | ebi-a-GCST90001693 | TRUE | T | 199163362 | 1.38E-09 | reported | 3408 | 0.1339 | rs112009416 |  |  |  |  |  |  |  |  |  |  |  |  |  |  |  |
| 103.6 | 2 | igd | 0.0091 | G | \|\| id:ebi-a-GCST90001698 | ebi-a-GCST90001698 | TRUE | C | 29209073 | 8.27E-09 | reported | 3408 | 17.93 | rs79679109 |  |  |  |  |  |  |  |  |  |  |  |  |  |  |  |
| 150.2 | 5 | igd | 0.0091 | C | \|\| id:ebi-a-GCST90001698 | ebi-a-GCST90001698 | TRUE | T | 148699825 | 4.15E-16 | reported | 3408 | 18.37 | rs17642570 |  |  |  |  |  |  |  |  |  |  |  |  |  |  |  |
| 132.9 | 7 | igd | 0.0091 | T | \|\| id:ebi-a-GCST90001698 | ebi-a-GCST90001698 | TRUE | G | 158183340 | 1.72E-13 | reported | 3408 | 17.97 | rs73178203 |  |  |  |  |  |  |  |  |  |  |  |  |  |  |  |
| 0.8054 | 1 | igd | 0.0091 | C | \|\| id:ebi-a-GCST90002102 | ebi-a-GCST90002102 | TRUE | T | 199163362 | 2.86E-09 | reported | 2919 | 0.1352 | rs112009416 |  |  |  |  |  |  |  |  |  |  |  |  |  |  |  |
| 0.7799 | 1 | igd | 0.009 | C | \|\| id:ebi-a-GCST90001483 | ebi-a-GCST90001483 | TRUE | T | 199163362 | 3.04E-09 | reported | 3405 | 0.1312 | rs112009416 |  |  |  |  |  |  |  |  |  |  |  |  |  |  |  |
| 0.8565 | 1 | igd | 0.009 | C | \|\| id:ebi-a-GCST90001483 | ebi-a-GCST90001483 | TRUE | T | 195919849 | 4.29E-10 | reported | 3405 | 0.1368 | rs146899923 |  |  |  |  |  |  |  |  |  |  |  |  |  |  |  |
| 0.7337 | 1 | igd | 0.009 | C | \|\| id:ebi-a-GCST90001665 | ebi-a-GCST90001665 | TRUE | T | 199163362 | 2.17E-08 | reported | 3440 | 0.1308 | rs112009416 |  |  |  |  |  |  |  |  |  |  |  |  |  |  |  |
| -0.8723 | 1 | igd | 0.009 | C | \|\| id:ebi-a-GCST90001667 | ebi-a-GCST90001667 | TRUE | T | 199163362 | 2.71E-12 | reported | 3440 | 0.1243 | rs112009416 |  |  |  |  |  |  |  |  |  |  |  |  |  |  |  |
| -0.8514 | 1 | igd | 0.009 | C | \|\| id:ebi-a-GCST90001668 | ebi-a-GCST90001668 | TRUE | T | 199163362 | 7.41E-11 | reported | 3440 | 0.1303 | rs112009416 |  |  |  |  |  |  |  |  |  |  |  |  |  |  |  |
| -0.7981 | 1 | igd | 0.009 | C | \|\| id:ebi-a-GCST90001691 | ebi-a-GCST90001691 | TRUE | T | 199163362 | 2.33E-09 | reported | 3440 | 0.1333 | rs112009416 |  |  |  |  |  |  |  |  |  |  |  |  |  |  |  |
| -0.7443 | 1 | igd | 0.009 | C | \|\| id:ebi-a-GCST90001692 | ebi-a-GCST90001692 | TRUE | T | 199163362 | 1.97E-08 | reported | 3440 | 0.1322 | rs112009416 |  |  |  |  |  |  |  |  |  |  |  |  |  |  |  |
| 0.9128 | 1 | igd | 0.0089 | C | \|\| id:ebi-a-GCST90001485 | ebi-a-GCST90001485 | TRUE | T | 199163362 | 3.13E-12 | reported | 3437 | 0.1305 | rs112009416 |  |  |  |  |  |  |  |  |  |  |  |  |  |  |  |
| 0.9069 | 1 | igd | 0.0089 | C | \|\| id:ebi-a-GCST90001485 | ebi-a-GCST90001485 | TRUE | T | 195919849 | 3.14E-11 | reported | 3437 | 0.1361 | rs146899923 |  |  |  |  |  |  |  |  |  |  |  |  |  |  |  |
| 0.7546 | 1 | igd | 0.0089 | C | \|\| id:ebi-a-GCST90001508 | ebi-a-GCST90001508 | TRUE | T | 199163362 | 2.95E-08 | reported | 3437 | 0.1358 | rs112009416 |  |  |  |  |  |  |  |  |  |  |  |  |  |  |  |
| 0.6318 | 1 | igd | 0.0089 | C | \|\| id:ebi-a-GCST90001561 | ebi-a-GCST90001561 | TRUE | T | 199163362 | 3.05E-08 | reported | 3427 | 0.1138 | rs112009416 |  |  |  |  |  |  |  |  |  |  |  |  |  |  |  |
| 0.7516 | 1 | igd | 0.0089 | C | \|\| id:ebi-a-GCST90001665 | ebi-a-GCST90001665 | TRUE | T | 195919849 | 4.52E-08 | reported | 3440 | 0.1371 | rs146899923 |  |  |  |  |  |  |  |  |  |  |  |  |  |  |  |
| 105.7 | 11 | igd | 0.0089 | C | \|\| id:ebi-a-GCST90001698 | ebi-a-GCST90001698 | TRUE | A | 42856719 | 6.21E-09 | reported | 3408 | 18.15 | rs11601011 |  |  |  |  |  |  |  |  |  |  |  |  |  |  |  |
| 106.8 | 1 | igd | 0.0088 | A | \|\| id:ebi-a-GCST90001698 | ebi-a-GCST90001698 | TRUE | C | 99542806 | 4.27E-08 | reported | 3408 | 19.44 | rs76636736 |  |  |  |  |  |  |  |  |  |  |  |  |  |  |  |
| 104.4 | 1 | igd | 0.0088 | A | \|\| id:ebi-a-GCST90001698 | ebi-a-GCST90001698 | TRUE | G | 35747418 | 4.11E-08 | reported | 3408 | 18.99 | rs79448993 |  |  |  |  |  |  |  |  |  |  |  |  |  |  |  |
| 122.5 | 5 | igd | 0.0088 | C | \|\| id:ebi-a-GCST90001698 | ebi-a-GCST90001698 | TRUE | T | 180540926 | 2.37E-10 | reported | 3408 | 19.28 | rs56126793 |  |  |  |  |  |  |  |  |  |  |  |  |  |  |  |
| 115.3 | 10 | igd | 0.0087 | A | \|\| id:ebi-a-GCST90001698 | ebi-a-GCST90001698 | TRUE | G | 54898644 | 5.52E-10 | reported | 3408 | 18.53 | rs11597865 |  |  |  |  |  |  |  |  |  |  |  |  |  |  |  |
| 120.5 | 8 | igd | 0.0085 | G | \|\| id:ebi-a-GCST90001698 | ebi-a-GCST90001698 | TRUE | A | 116705188 | 7.18E-10 | reported | 3408 | 19.5 | rs183038581 |  |  |  |  |  |  |  |  |  |  |  |  |  |  |  |
| 137 | 8 | igd | 0.0085 | T | \|\| id:ebi-a-GCST90001698 | ebi-a-GCST90001698 | TRUE | C | 117040660 | 1.26E-11 | reported | 3408 | 20.15 | rs181660070 |  |  |  |  |  |  |  |  |  |  |  |  |  |  |  |
| 128.8 | 19 | igd | 0.0084 | T | \|\| id:ebi-a-GCST90001698 | ebi-a-GCST90001698 | TRUE | C | 13419796 | 2.53E-11 | reported | 3408 | 19.23 | rs116924509 |  |  |  |  |  |  |  |  |  |  |  |  |  |  |  |
| 1.246 | 6 | igd | 0.0084 | G | \|\| id:ebi-a-GCST90002105 | ebi-a-GCST90002105 | TRUE | C | 24144026 | 2.02E-13 | reported | 2872 | 0.1687 | rs74411153 |  |  |  |  |  |  |  |  |  |  |  |  |  |  |  |
| 120.4 | 7 | igd | 0.0082 | G | \|\| id:ebi-a-GCST90001698 | ebi-a-GCST90001698 | TRUE | A | 68314983 | 5.28E-10 | reported | 3408 | 19.33 | rs113799183 |  |  |  |  |  |  |  |  |  |  |  |  |  |  |  |
| 120.6 | 10 | igd | 0.0082 | C | \|\| id:ebi-a-GCST90001698 | ebi-a-GCST90001698 | TRUE | T | 53667102 | 1.52E-09 | reported | 3408 | 19.91 | rs117010194 |  |  |  |  |  |  |  |  |  |  |  |  |  |  |  |
| 0.9978 | 6 | igd | 0.008 | G | \|\| id:ebi-a-GCST90002105 | ebi-a-GCST90002105 | TRUE | A | 24693940 | 6.60E-10 | reported | 2872 | 0.161 | rs60707509 |  |  |  |  |  |  |  |  |  |  |  |  |  |  |  |
| 1.04 | 6 | igd | 0.008 | A | \|\| id:ebi-a-GCST90002105 | ebi-a-GCST90002105 | TRUE | G | 24329095 | 2.04E-10 | reported | 2872 | 0.1629 | rs115629436 |  |  |  |  |  |  |  |  |  |  |  |  |  |  |  |
| 141 | 6 | igd | 0.0079 | C | \|\| id:ebi-a-GCST90001698 | ebi-a-GCST90001698 | TRUE | T | 86346609 | 3.50E-12 | reported | 3408 | 20.2 | rs117822024 |  |  |  |  |  |  |  |  |  |  |  |  |  |  |  |
| 121.1 | 6 | igd | 0.0079 | T | \|\| id:ebi-a-GCST90001698 | ebi-a-GCST90001698 | TRUE | A | 11805124 | 2.34E-10 | reported | 3408 | 19.05 | rs75424381 |  |  |  |  |  |  |  |  |  |  |  |  |  |  |  |
| 123.5 | 8 | igd | 0.0079 | A | \|\| id:ebi-a-GCST90001698 | ebi-a-GCST90001698 | TRUE | G | 124197307 | 7.32E-10 | reported | 3408 | 20 | rs147959386 |  |  |  |  |  |  |  |  |  |  |  |  |  |  |  |
| 130.9 | 9 | igd | 0.0079 | A | \|\| id:ebi-a-GCST90001698 | ebi-a-GCST90001698 | TRUE | G | 122201008 | 3.35E-10 | reported | 3408 | 20.78 | rs76543451 |  |  |  |  |  |  |  |  |  |  |  |  |  |  |  |
| 143.3 | 9 | igd | 0.0079 | G | \|\| id:ebi-a-GCST90001698 | ebi-a-GCST90001698 | TRUE | A | 100968226 | 1.89E-12 | reported | 3408 | 20.28 | rs7850783 |  |  |  |  |  |  |  |  |  |  |  |  |  |  |  |
| -0.9089 | 17 | igd | 0.0079 | A | \|\| id:ebi-a-GCST90001926 | ebi-a-GCST90001926 | TRUE | G | 61576413 | 4.89E-09 | reported | 2920 | 0.1549 | rs146130460 |  |  |  |  |  |  |  |  |  |  |  |  |  |  |  |
| 0.832 | 1 | igd | 0.0078 | C | \|\| id:ebi-a-GCST90001483 | ebi-a-GCST90001483 | TRUE | T | 200021804 | 2.47E-09 | reported | 3405 | 0.1391 | rs180839256 |  |  |  |  |  |  |  |  |  |  |  |  |  |  |  |
| 0.8047 | 1 | igd | 0.0078 | C | \|\| id:ebi-a-GCST90001666 | ebi-a-GCST90001666 | TRUE | T | 200021804 | 1.56E-10 | reported | 3408 | 0.1254 | rs180839256 |  |  |  |  |  |  |  |  |  |  |  |  |  |  |  |
| 133.8 | 11 | igd | 0.0078 | T | \|\| id:ebi-a-GCST90001698 | ebi-a-GCST90001698 | TRUE | C | 109965739 | 1.05E-11 | reported | 3408 | 19.61 | rs117104919 |  |  |  |  |  |  |  |  |  |  |  |  |  |  |  |
| 0.9076 | 1 | igd | 0.0077 | C | \|\| id:ebi-a-GCST90001485 | ebi-a-GCST90001485 | TRUE | T | 200021804 | 6.67E-11 | reported | 3437 | 0.1386 | rs180839256 |  |  |  |  |  |  |  |  |  |  |  |  |  |  |  |
| 0.7026 | 1 | igd | 0.0077 | C | \|\| id:ebi-a-GCST90001561 | ebi-a-GCST90001561 | TRUE | T | 200021804 | 7.70E-09 | reported | 3427 | 0.1214 | rs180839256 |  |  |  |  |  |  |  |  |  |  |  |  |  |  |  |
| 0.8984 | 1 | igd | 0.0077 | C | \|\| id:ebi-a-GCST90001664 | ebi-a-GCST90001664 | TRUE | T | 200021804 | 9.37E-12 | reported | 3440 | 0.1314 | rs180839256 |  |  |  |  |  |  |  |  |  |  |  |  |  |  |  |
| -0.821 | 1 | igd | 0.0077 | C | \|\| id:ebi-a-GCST90001668 | ebi-a-GCST90001668 | TRUE | T | 200021804 | 4.89E-09 | reported | 3440 | 0.14 | rs180839256 |  |  |  |  |  |  |  |  |  |  |  |  |  |  |  |
| 0.8507 | 5 | igd | 0.0077 | A | \|\| id:ebi-a-GCST90001928 | ebi-a-GCST90001928 | TRUE | G | 174310054 | 1.37E-08 | reported | 2920 | 0.1494 | rs116423144 |  |  |  |  |  |  |  |  |  |  |  |  |  |  |  |
| 87.63 | 15 | igd | 0.0076 | T | \|\| id:ebi-a-GCST90001695 | ebi-a-GCST90001695 | TRUE | C | 27818525 | 9.60E-09 | reported | 3408 | 15.24 | rs112568609 |  |  |  |  |  |  |  |  |  |  |  |  |  |  |  |
| 137.7 | 6 | igd | 0.0076 | G | \|\| id:ebi-a-GCST90001698 | ebi-a-GCST90001698 | TRUE | T | 95586945 | 3.12E-11 | reported | 3408 | 20.67 | rs149260403 |  |  |  |  |  |  |  |  |  |  |  |  |  |  |  |
| 125.6 | 9 | igd | 0.0076 | T | \|\| id:ebi-a-GCST90001698 | ebi-a-GCST90001698 | TRUE | C | 135625608 | 1.16E-10 | reported | 3408 | 19.43 | rs74336249 |  |  |  |  |  |  |  |  |  |  |  |  |  |  |  |
| 0.9087 | 1 | igd | 0.0076 | C | \|\| id:ebi-a-GCST90002098 | ebi-a-GCST90002098 | TRUE | T | 200021804 | 7.72E-10 | reported | 2910 | 0.1472 | rs180839256 |  |  |  |  |  |  |  |  |  |  |  |  |  |  |  |
| 84.27 | 20 | igd | 0.0075 | G | \|\| id:ebi-a-GCST90001695 | ebi-a-GCST90001695 | TRUE | A | 3521072 | 4.04E-08 | reported | 3408 | 15.32 | rs117036387 |  |  |  |  |  |  |  |  |  |  |  |  |  |  |  |
| 113 | 8 | igd | 0.0075 | C | \|\| id:ebi-a-GCST90001698 | ebi-a-GCST90001698 | TRUE | A | 124129212 | 2.29E-08 | reported | 3408 | 20.18 | rs116862272 |  |  |  |  |  |  |  |  |  |  |  |  |  |  |  |
| 132.7 | 11 | igd | 0.0075 | A | \|\| id:ebi-a-GCST90001698 | ebi-a-GCST90001698 | TRUE | G | 109402068 | 1.09E-10 | reported | 3408 | 20.5 | rs116853880 |  |  |  |  |  |  |  |  |  |  |  |  |  |  |  |
| 124.8 | 15 | igd | 0.0075 | A | \|\| id:ebi-a-GCST90001698 | ebi-a-GCST90001698 | TRUE | G | 52274894 | 6.93E-10 | reported | 3408 | 20.18 | rs2414121 |  |  |  |  |  |  |  |  |  |  |  |  |  |  |  |
| -0.8986 | 17 | igd | 0.0074 | A | \|\| id:ebi-a-GCST90001926 | ebi-a-GCST90001926 | TRUE | G | 61993406 | 2.74E-08 | reported | 2920 | 0.1613 | rs186581898 |  |  |  |  |  |  |  |  |  |  |  |  |  |  |  |
| -1.096 | 17 | igd | 0.0074 | T | \|\| id:ebi-a-GCST90001926 | ebi-a-GCST90001926 | TRUE | G | 59945942 | 1.12E-11 | reported | 2920 | 0.1607 | rs147173440 |  |  |  |  |  |  |  |  |  |  |  |  |  |  |  |
| 88.88 | 18 | igd | 0.0073 | T | \|\| id:ebi-a-GCST90001695 | ebi-a-GCST90001695 | TRUE | C | 7862061 | 2.66E-08 | reported | 3408 | 15.94 | rs75346347 |  |  |  |  |  |  |  |  |  |  |  |  |  |  |  |
| 163.3 | 2 | igd | 0.0073 | T | \|\| id:ebi-a-GCST90001698 | ebi-a-GCST90001698 | TRUE | C | 72979172 | 7.76E-15 | reported | 3408 | 20.92 | rs1508064 |  |  |  |  |  |  |  |  |  |  |  |  |  |  |  |
| 118 | 3 | igd | 0.0073 | G | \|\| id:ebi-a-GCST90001698 | ebi-a-GCST90001698 | TRUE | C | 166778156 | 3.17E-08 | reported | 3408 | 21.29 | rs141337845 |  |  |  |  |  |  |  |  |  |  |  |  |  |  |  |
| 138.3 | 3 | igd | 0.0073 | C | \|\| id:ebi-a-GCST90001698 | ebi-a-GCST90001698 | TRUE | T | 78528460 | 1.36E-11 | reported | 3408 | 20.39 | rs143458243 |  |  |  |  |  |  |  |  |  |  |  |  |  |  |  |
| 116.1 | 4 | igd | 0.0073 | C | \|\| id:ebi-a-GCST90001698 | ebi-a-GCST90001698 | TRUE | T | 95896442 | 1.87E-08 | reported | 3408 | 20.59 | rs112233105 |  |  |  |  |  |  |  |  |  |  |  |  |  |  |  |
| 126.5 | 7 | igd | 0.0073 | A | \|\| id:ebi-a-GCST90001698 | ebi-a-GCST90001698 | TRUE | G | 69003154 | 2.78E-09 | reported | 3408 | 21.22 | rs117075001 |  |  |  |  |  |  |  |  |  |  |  |  |  |  |  |
| 141.4 | 11 | igd | 0.0073 | A | \|\| id:ebi-a-GCST90001698 | ebi-a-GCST90001698 | TRUE | G | 109660227 | 8.60E-12 | reported | 3408 | 20.64 | rs79741386 |  |  |  |  |  |  |  |  |  |  |  |  |  |  |  |
| -1.233 | 6 | igd | 0.0073 | A | \|\| id:ebi-a-GCST90002105 | ebi-a-GCST90002105 | TRUE | C | 32650631 | 1.28E-10 | reported | 2872 | 0.1911 | rs17212055 |  |  |  |  |  |  |  |  |  |  |  |  |  |  |  |
| 109 | 14 | igd | 0.0072 | G | \|\| id:ebi-a-GCST90001695 | ebi-a-GCST90001695 | TRUE | T | 76846837 | 4.64E-12 | reported | 3408 | 15.71 | rs77243672 |  |  |  |  |  |  |  |  |  |  |  |  |  |  |  |
| 4.44 | 10 | igd | 0.0072 | A | \|\| id:ebi-a-GCST90001697 | ebi-a-GCST90001697 | TRUE | G | 70543706 | 2.66E-09 | reported | 3425 | 0.7441 | rs143930177 |  |  |  |  |  |  |  |  |  |  |  |  |  |  |  |
| 120.7 | 4 | igd | 0.0072 | G | \|\| id:ebi-a-GCST90001698 | ebi-a-GCST90001698 | TRUE | C | 11942411 | 7.97E-09 | reported | 3408 | 20.87 | rs75196907 |  |  |  |  |  |  |  |  |  |  |  |  |  |  |  |
| 127.1 | 5 | igd | 0.0072 | C | \|\| id:ebi-a-GCST90001698 | ebi-a-GCST90001698 | TRUE | A | 145713781 | 3.00E-09 | reported | 3408 | 21.37 | rs142922198 |  |  |  |  |  |  |  |  |  |  |  |  |  |  |  |
| 120.3 | 6 | igd | 0.0072 | T | \|\| id:ebi-a-GCST90001698 | ebi-a-GCST90001698 | TRUE | C | 129871947 | 1.76E-08 | reported | 3408 | 21.3 | rs77729615 |  |  |  |  |  |  |  |  |  |  |  |  |  |  |  |
| 134.7 | 11 | igd | 0.0072 | A | \|\| id:ebi-a-GCST90001698 | ebi-a-GCST90001698 | TRUE | G | 109281956 | 1.78E-10 | reported | 3408 | 21.05 | rs118168955 |  |  |  |  |  |  |  |  |  |  |  |  |  |  |  |
| 4.991 | 19 | igd | 0.0072 | A | \|\| id:ebi-a-GCST90001700 | ebi-a-GCST90001700 | TRUE | G | 18858754 | 1.62E-08 | reported | 3425 | 0.8815 | rs191923221 |  |  |  |  |  |  |  |  |  |  |  |  |  |  |  |
| -1.145 | 16 | igd | 0.0072 | T | \|\| id:ebi-a-GCST90002028 | ebi-a-GCST90002028 | TRUE | C | 32611053 | 4.60E-09 | reported | 3112 | 0.1948 | rs112285207 |  |  |  |  |  |  |  |  |  |  |  |  |  |  |  |
| 0.9239 | 1 | igd | 0.0072 | C | \|\| id:ebi-a-GCST90002102 | ebi-a-GCST90002102 | TRUE | T | 200021804 | 9.02E-10 | reported | 2919 | 0.1503 | rs180839256 |  |  |  |  |  |  |  |  |  |  |  |  |  |  |  |
| 0.8321 | 10 | igd | 0.007 | T | \|\| id:ebi-a-GCST90001484 | ebi-a-GCST90001484 | TRUE | G | 97211637 | 3.07E-08 | reported | 3437 | 0.1499 | rs116888462 |  |  |  |  |  |  |  |  |  |  |  |  |  |  |  |
| 0.8418 | 10 | igd | 0.007 | T | \|\| id:ebi-a-GCST90001485 | ebi-a-GCST90001485 | TRUE | G | 97211637 | 2.02E-08 | reported | 3437 | 0.1497 | rs116888462 |  |  |  |  |  |  |  |  |  |  |  |  |  |  |  |
| 0.9429 | 10 | igd | 0.007 | T | \|\| id:ebi-a-GCST90001658 | ebi-a-GCST90001658 | TRUE | G | 97211637 | 2.39E-10 | reported | 3440 | 0.1484 | rs116888462 |  |  |  |  |  |  |  |  |  |  |  |  |  |  |  |
| 0.9457 | 10 | igd | 0.007 | T | \|\| id:ebi-a-GCST90001659 | ebi-a-GCST90001659 | TRUE | G | 97211637 | 2.12E-10 | reported | 3440 | 0.1484 | rs116888462 |  |  |  |  |  |  |  |  |  |  |  |  |  |  |  |
| 0.9778 | 10 | igd | 0.007 | T | \|\| id:ebi-a-GCST90001670 | ebi-a-GCST90001670 | TRUE | G | 97211637 | 2.60E-10 | reported | 3440 | 0.1542 | rs116888462 |  |  |  |  |  |  |  |  |  |  |  |  |  |  |  |
| 0.959 | 10 | igd | 0.007 | T | \|\| id:ebi-a-GCST90001671 | ebi-a-GCST90001671 | TRUE | G | 97211637 | 6.42E-10 | reported | 3440 | 0.1547 | rs116888462 |  |  |  |  |  |  |  |  |  |  |  |  |  |  |  |
| 154.4 | 5 | igd | 0.007 | T | \|\| id:ebi-a-GCST90001698 | ebi-a-GCST90001698 | TRUE | A | 180054853 | 4.62E-13 | reported | 3408 | 21.25 | rs3822406 |  |  |  |  |  |  |  |  |  |  |  |  |  |  |  |
| 134.1 | 8 | igd | 0.007 | A | \|\| id:ebi-a-GCST90001698 | ebi-a-GCST90001698 | TRUE | G | 8107029 | 2.19E-11 | reported | 3408 | 19.97 | rs113986067 |  |  |  |  |  |  |  |  |  |  |  |  |  |  |  |
| 121.9 | 17 | igd | 0.007 | T | \|\| id:ebi-a-GCST90001698 | ebi-a-GCST90001698 | TRUE | C | 41836082 | 8.32E-09 | reported | 3408 | 21.11 | rs17882143 |  |  |  |  |  |  |  |  |  |  |  |  |  |  |  |
| -0.85 | 16 | igd | 0.007 | A | \|\| id:ebi-a-GCST90001729 | ebi-a-GCST90001729 | TRUE | C | 34579231 | 2.02E-08 | reported | 3657 | 0.1512 | rs17723384 |  |  |  |  |  |  |  |  |  |  |  |  |  |  |  |
| -0.8293 | 16 | igd | 0.007 | A | \|\| id:ebi-a-GCST90001735 | ebi-a-GCST90001735 | TRUE | C | 34579231 | 2.77E-08 | reported | 3657 | 0.1489 | rs17723384 |  |  |  |  |  |  |  |  |  |  |  |  |  |  |  |
| 123.1 | 1 | igd | 0.0069 | G | \|\| id:ebi-a-GCST90001698 | ebi-a-GCST90001698 | TRUE | A | 10140691 | 1.49E-08 | reported | 3408 | 21.69 | rs187566373 |  |  |  |  |  |  |  |  |  |  |  |  |  |  |  |
| 120.1 | 3 | igd | 0.0069 | A | \|\| id:ebi-a-GCST90001698 | ebi-a-GCST90001698 | TRUE | T | 126149695 | 5.53E-09 | reported | 3408 | 20.54 | rs75343794 |  |  |  |  |  |  |  |  |  |  |  |  |  |  |  |
| 119.1 | 4 | igd | 0.0069 | T | \|\| id:ebi-a-GCST90001698 | ebi-a-GCST90001698 | TRUE | C | 96110827 | 1.62E-08 | reported | 3408 | 21.04 | rs189378517 |  |  |  |  |  |  |  |  |  |  |  |  |  |  |  |
| 131.9 | 6 | igd | 0.0069 | A | \|\| id:ebi-a-GCST90001698 | ebi-a-GCST90001698 | TRUE | G | 38410053 | 6.88E-10 | reported | 3408 | 21.32 | rs141943967 |  |  |  |  |  |  |  |  |  |  |  |  |  |  |  |
| 172.1 | 7 | igd | 0.0069 | C | \|\| id:ebi-a-GCST90001698 | ebi-a-GCST90001698 | TRUE | T | 109711861 | 3.31E-15 | reported | 3408 | 21.74 | rs9649329 |  |  |  |  |  |  |  |  |  |  |  |  |  |  |  |
| 124.2 | 8 | igd | 0.0069 | G | \|\| id:ebi-a-GCST90001698 | ebi-a-GCST90001698 | TRUE | T | 8211858 | 3.63E-09 | reported | 3408 | 20.99 | rs56396250 |  |  |  |  |  |  |  |  |  |  |  |  |  |  |  |
| 149.1 | 19 | igd | 0.0069 | A | \|\| id:ebi-a-GCST90001698 | ebi-a-GCST90001698 | TRUE | G | 34075835 | 2.36E-12 | reported | 3408 | 21.18 | rs59745713 |  |  |  |  |  |  |  |  |  |  |  |  |  |  |  |
| 0.9113 | 10 | igd | 0.0068 | T | \|\| id:ebi-a-GCST90001483 | ebi-a-GCST90001483 | TRUE | G | 97211637 | 3.38E-09 | reported | 3405 | 0.1537 | rs116888462 |  |  |  |  |  |  |  |  |  |  |  |  |  |  |  |
| -0.9117 | 10 | igd | 0.0068 | A | \|\| id:ebi-a-GCST90001961 | ebi-a-GCST90001961 | TRUE | T | 98505365 | 3.09E-08 | reported | 2920 | 0.1642 | rs11188901 |  |  |  |  |  |  |  |  |  |  |  |  |  |  |  |
| -0.9619 | 12 | igd | 0.0068 | T | \|\| id:ebi-a-GCST90002073 | ebi-a-GCST90002073 | TRUE | C | 68425985 | 1.97E-08 | reported | 2850 | 0.1708 | rs77928745 |  |  |  |  |  |  |  |  |  |  |  |  |  |  |  |
| 0.9149 | 10 | igd | 0.0067 | T | \|\| id:ebi-a-GCST90001660 | ebi-a-GCST90001660 | TRUE | G | 97211637 | 7.97E-09 | reported | 3408 | 0.1582 | rs116888462 |  |  |  |  |  |  |  |  |  |  |  |  |  |  |  |
| 0.9699 | 10 | igd | 0.0067 | T | \|\| id:ebi-a-GCST90001672 | ebi-a-GCST90001672 | TRUE | G | 97211637 | 1.38E-09 | reported | 3408 | 0.1597 | rs116888462 |  |  |  |  |  |  |  |  |  |  |  |  |  |  |  |
| 142.5 | 7 | igd | 0.0067 | T | \|\| id:ebi-a-GCST90001698 | ebi-a-GCST90001698 | TRUE | C | 69178260 | 4.65E-10 | reported | 3408 | 22.81 | rs117848605 |  |  |  |  |  |  |  |  |  |  |  |  |  |  |  |
| -1.055 | 16 | igd | 0.0067 | T | \|\| id:ebi-a-GCST90001729 | ebi-a-GCST90001729 | TRUE | C | 32611053 | 3.30E-09 | reported | 3657 | 0.1779 | rs112285207 |  |  |  |  |  |  |  |  |  |  |  |  |  |  |  |
| 0.9505 | 10 | igd | 0.0067 | A | \|\| id:ebi-a-GCST90002030 | ebi-a-GCST90002030 | TRUE | G | 97898692 | 1.82E-08 | reported | 2920 | 0.1684 | rs587668661 |  |  |  |  |  |  |  |  |  |  |  |  |  |  |  |
| 1.326 | 6 | igd | 0.0067 | G | \|\| id:ebi-a-GCST90002112 | ebi-a-GCST90002112 | TRUE | A | 31299958 | 2.60E-08 | reported | 1635 | 0.237 | rs34972021 |  |  |  |  |  |  |  |  |  |  |  |  |  |  |  |
| -0.8348 | 14 | igd | 0.0066 | C | \|\| id:ebi-a-GCST90001422 | ebi-a-GCST90001422 | TRUE | T | 31993501 | 2.48E-08 | reported | 3659 | 0.1494 | rs117308259 |  |  |  |  |  |  |  |  |  |  |  |  |  |  |  |
| 0.9157 | 1 | igd | 0.0066 | G | \|\| id:ebi-a-GCST90001483 | ebi-a-GCST90001483 | TRUE | A | 199432953 | 2.91E-09 | reported | 3405 | 0.1538 | rs111758901 |  |  |  |  |  |  |  |  |  |  |  |  |  |  |  |
| 0.8927 | 1 | igd | 0.0066 | G | \|\| id:ebi-a-GCST90001507 | ebi-a-GCST90001507 | TRUE | A | 199432953 | 2.94E-08 | reported | 3405 | 0.1606 | rs111758901 |  |  |  |  |  |  |  |  |  |  |  |  |  |  |  |
| 0.8663 | 1 | igd | 0.0066 | G | \|\| id:ebi-a-GCST90001561 | ebi-a-GCST90001561 | TRUE | A | 199432953 | 1.23E-10 | reported | 3427 | 0.1342 | rs111758901 |  |  |  |  |  |  |  |  |  |  |  |  |  |  |  |
| 143.2 | 5 | igd | 0.0066 | G | \|\| id:ebi-a-GCST90001698 | ebi-a-GCST90001698 | TRUE | A | 2084803 | 2.01E-11 | reported | 3408 | 21.29 | rs10067057 |  |  |  |  |  |  |  |  |  |  |  |  |  |  |  |
| 126.9 | 7 | igd | 0.0066 | T | \|\| id:ebi-a-GCST90001698 | ebi-a-GCST90001698 | TRUE | C | 158756428 | 3.58E-09 | reported | 3408 | 21.44 | rs141521458 |  |  |  |  |  |  |  |  |  |  |  |  |  |  |  |
| 124.3 | 7 | igd | 0.0066 | T | \|\| id:ebi-a-GCST90001698 | ebi-a-GCST90001698 | TRUE | C | 83499414 | 1.08E-08 | reported | 3408 | 21.69 | rs184379743 |  |  |  |  |  |  |  |  |  |  |  |  |  |  |  |
| 164.3 | 8 | igd | 0.0066 | T | \|\| id:ebi-a-GCST90001698 | ebi-a-GCST90001698 | TRUE | C | 25574426 | 3.68E-14 | reported | 3408 | 21.61 | rs34000508 |  |  |  |  |  |  |  |  |  |  |  |  |  |  |  |
| 116.6 | 8 | igd | 0.0066 | T | \|\| id:ebi-a-GCST90001698 | ebi-a-GCST90001698 | TRUE | C | 4499026 | 3.19E-08 | reported | 3408 | 21.02 | rs77009235 |  |  |  |  |  |  |  |  |  |  |  |  |  |  |  |
| 156.1 | 10 | igd | 0.0066 | G | \|\| id:ebi-a-GCST90001698 | ebi-a-GCST90001698 | TRUE | A | 36442592 | 2.15E-12 | reported | 3408 | 22.14 | rs2384564 |  |  |  |  |  |  |  |  |  |  |  |  |  |  |  |
| 163.9 | 13 | igd | 0.0066 | G | \|\| id:ebi-a-GCST90001698 | ebi-a-GCST90001698 | TRUE | A | 20312298 | 4.39E-14 | reported | 3408 | 21.62 | rs117703192 |  |  |  |  |  |  |  |  |  |  |  |  |  |  |  |
| 123.8 | 20 | igd | 0.0066 | T | \|\| id:ebi-a-GCST90001698 | ebi-a-GCST90001698 | TRUE | C | 14461922 | 3.41E-08 | reported | 3408 | 22.38 | rs17227006 |  |  |  |  |  |  |  |  |  |  |  |  |  |  |  |
| 1.034 | 1 | igd | 0.0065 | G | \|\| id:ebi-a-GCST90001485 | ebi-a-GCST90001485 | TRUE | A | 199432953 | 1.73E-11 | reported | 3437 | 0.1532 | rs111758901 |  |  |  |  |  |  |  |  |  |  |  |  |  |  |  |
| 0.947 | 1 | igd | 0.0065 | G | \|\| id:ebi-a-GCST90001508 | ebi-a-GCST90001508 | TRUE | A | 199432953 | 3.32E-09 | reported | 3437 | 0.1597 | rs111758901 |  |  |  |  |  |  |  |  |  |  |  |  |  |  |  |
| 0.9085 | 1 | igd | 0.0065 | G | \|\| id:ebi-a-GCST90001509 | ebi-a-GCST90001509 | TRUE | A | 199432953 | 1.37E-08 | reported | 3437 | 0.1597 | rs111758901 |  |  |  |  |  |  |  |  |  |  |  |  |  |  |  |
| -0.8884 | 1 | igd | 0.0065 | G | \|\| id:ebi-a-GCST90001511 | ebi-a-GCST90001511 | TRUE | A | 199432953 | 2.58E-08 | reported | 3437 | 0.1592 | rs111758901 |  |  |  |  |  |  |  |  |  |  |  |  |  |  |  |
| -0.9501 | 1 | igd | 0.0065 | G | \|\| id:ebi-a-GCST90001691 | ebi-a-GCST90001691 | TRUE | A | 199432953 | 1.90E-09 | reported | 3440 | 0.1578 | rs111758901 |  |  |  |  |  |  |  |  |  |  |  |  |  |  |  |
| -0.9271 | 1 | igd | 0.0065 | G | \|\| id:ebi-a-GCST90001692 | ebi-a-GCST90001692 | TRUE | A | 199432953 | 3.72E-09 | reported | 3440 | 0.1568 | rs111758901 |  |  |  |  |  |  |  |  |  |  |  |  |  |  |  |
| 144 | 2 | igd | 0.0065 | A | \|\| id:ebi-a-GCST90001698 | ebi-a-GCST90001698 | TRUE | G | 99679099 | 1.13E-09 | reported | 3408 | 23.58 | rs62156361 |  |  |  |  |  |  |  |  |  |  |  |  |  |  |  |
| 156.3 | 3 | igd | 0.0065 | C | \|\| id:ebi-a-GCST90001698 | ebi-a-GCST90001698 | TRUE | T | 81799701 | 5.65E-11 | reported | 3408 | 23.78 | rs184977394 |  |  |  |  |  |  |  |  |  |  |  |  |  |  |  |
| 168 | 5 | igd | 0.0065 | C | \|\| id:ebi-a-GCST90001698 | ebi-a-GCST90001698 | TRUE | G | 176286100 | 3.77E-14 | reported | 3408 | 22.1 | rs541985955 |  |  |  |  |  |  |  |  |  |  |  |  |  |  |  |
| 133.3 | 7 | igd | 0.0065 | T | \|\| id:ebi-a-GCST90001698 | ebi-a-GCST90001698 | TRUE | C | 69370570 | 6.44E-09 | reported | 3408 | 22.9 | rs117307453 |  |  |  |  |  |  |  |  |  |  |  |  |  |  |  |
| 126.2 | 7 | igd | 0.0065 | T | \|\| id:ebi-a-GCST90001698 | ebi-a-GCST90001698 | TRUE | C | 153895185 | 5.99E-09 | reported | 3408 | 21.64 | rs116921833 |  |  |  |  |  |  |  |  |  |  |  |  |  |  |  |
| 131.5 | 13 | igd | 0.0065 | T | \|\| id:ebi-a-GCST90001698 | ebi-a-GCST90001698 | TRUE | C | 98295423 | 4.61E-08 | reported | 3408 | 24.01 | rs140627381 |  |  |  |  |  |  |  |  |  |  |  |  |  |  |  |
| 1.129 | 1 | igd | 0.0065 | C | \|\| id:ebi-a-GCST90002098 | ebi-a-GCST90002098 | TRUE | T | 196183282 | 5.85E-11 | reported | 2910 | 0.1718 | rs115186385 |  |  |  |  |  |  |  |  |  |  |  |  |  |  |  |
| 1.275 | 1 | igd | 0.0063 | C | \|\| id:ebi-a-GCST90001483 | ebi-a-GCST90001483 | TRUE | T | 196183282 | 1.18E-14 | reported | 3405 | 0.1645 | rs115186385 |  |  |  |  |  |  |  |  |  |  |  |  |  |  |  |
| 1.288 | 1 | igd | 0.0063 | C | \|\| id:ebi-a-GCST90001485 | ebi-a-GCST90001485 | TRUE | T | 196183282 | 5.47E-15 | reported | 3437 | 0.1641 | rs115186385 |  |  |  |  |  |  |  |  |  |  |  |  |  |  |  |
| 1.346 | 1 | igd | 0.0063 | C | \|\| id:ebi-a-GCST90001508 | ebi-a-GCST90001508 | TRUE | T | 196183282 | 4.88E-15 | reported | 3437 | 0.1711 | rs115186385 |  |  |  |  |  |  |  |  |  |  |  |  |  |  |  |
| 1.276 | 1 | igd | 0.0063 | C | \|\| id:ebi-a-GCST90001509 | ebi-a-GCST90001509 | TRUE | T | 196183282 | 1.14E-13 | reported | 3437 | 0.1712 | rs115186385 |  |  |  |  |  |  |  |  |  |  |  |  |  |  |  |
| 1.063 | 1 | igd | 0.0063 | C | \|\| id:ebi-a-GCST90001664 | ebi-a-GCST90001664 | TRUE | T | 196183282 | 1.30E-11 | reported | 3440 | 0.1565 | rs115186385 |  |  |  |  |  |  |  |  |  |  |  |  |  |  |  |
| 1.063 | 1 | igd | 0.0063 | C | \|\| id:ebi-a-GCST90001665 | ebi-a-GCST90001665 | TRUE | T | 196183282 | 2.27E-10 | reported | 3440 | 0.1672 | rs115186385 |  |  |  |  |  |  |  |  |  |  |  |  |  |  |  |
| 1.021 | 1 | igd | 0.0063 | C | \|\| id:ebi-a-GCST90001666 | ebi-a-GCST90001666 | TRUE | T | 196183282 | 9.40E-12 | reported | 3408 | 0.1493 | rs115186385 |  |  |  |  |  |  |  |  |  |  |  |  |  |  |  |
| -1.004 | 1 | igd | 0.0063 | C | \|\| id:ebi-a-GCST90001667 | ebi-a-GCST90001667 | TRUE | T | 196183282 | 2.85E-10 | reported | 3440 | 0.1588 | rs115186385 |  |  |  |  |  |  |  |  |  |  |  |  |  |  |  |
| -0.8611 | 1 | igd | 0.0063 | C | \|\| id:ebi-a-GCST90001669 | ebi-a-GCST90001669 | TRUE | T | 196183282 | 1.46E-08 | reported | 3408 | 0.1516 | rs115186385 |  |  |  |  |  |  |  |  |  |  |  |  |  |  |  |
| 147.6 | 1 | igd | 0.0063 | G | \|\| id:ebi-a-GCST90001698 | ebi-a-GCST90001698 | TRUE | A | 81185936 | 2.82E-10 | reported | 3408 | 23.33 | rs2152857 |  |  |  |  |  |  |  |  |  |  |  |  |  |  |  |
| 134.6 | 1 | igd | 0.0063 | C | \|\| id:ebi-a-GCST90001698 | ebi-a-GCST90001698 | TRUE | T | 180881632 | 1.16E-09 | reported | 3408 | 22.06 | rs76736944 |  |  |  |  |  |  |  |  |  |  |  |  |  |  |  |
| 159.3 | 7 | igd | 0.0063 | T | \|\| id:ebi-a-GCST90001698 | ebi-a-GCST90001698 | TRUE | C | 79203452 | 3.68E-13 | reported | 3408 | 21.83 | rs187429425 |  |  |  |  |  |  |  |  |  |  |  |  |  |  |  |
| 144.7 | 1 | igd | 0.0062 | C | \|\| id:ebi-a-GCST90001698 | ebi-a-GCST90001698 | TRUE | T | 80927199 | 2.60E-09 | reported | 3408 | 24.22 | rs79587638 |  |  |  |  |  |  |  |  |  |  |  |  |  |  |  |
| 144.1 | 12 | igd | 0.0062 | A | \|\| id:ebi-a-GCST90001698 | ebi-a-GCST90001698 | TRUE | G | 53402638 | 1.30E-08 | reported | 3408 | 25.27 | rs144682449 |  |  |  |  |  |  |  |  |  |  |  |  |  |  |  |
| 181.8 | 19 | igd | 0.0062 | A | \|\| id:ebi-a-GCST90001698 | ebi-a-GCST90001698 | TRUE | G | 17726676 | 1.30E-15 | reported | 3408 | 22.63 | rs150063907 |  |  |  |  |  |  |  |  |  |  |  |  |  |  |  |
| -0.8842 | 22 | igd | 0.0062 | T | \|\| id:ebi-a-GCST90001702 | ebi-a-GCST90001702 | TRUE | C | 41502451 | 1.95E-08 | reported | 3657 | 0.1571 | rs76331127 |  |  |  |  |  |  |  |  |  |  |  |  |  |  |  |
| -0.9093 | 22 | igd | 0.0062 | T | \|\| id:ebi-a-GCST90001703 | ebi-a-GCST90001703 | TRUE | C | 41502451 | 7.65E-09 | reported | 3657 | 0.1571 | rs76331127 |  |  |  |  |  |  |  |  |  |  |  |  |  |  |  |
| -0.8943 | 22 | igd | 0.0062 | T | \|\| id:ebi-a-GCST90001704 | ebi-a-GCST90001704 | TRUE | C | 41502451 | 1.30E-08 | reported | 3657 | 0.1569 | rs76331127 |  |  |  |  |  |  |  |  |  |  |  |  |  |  |  |
| -0.8637 | 22 | igd | 0.0062 | T | \|\| id:ebi-a-GCST90001711 | ebi-a-GCST90001711 | TRUE | C | 41502451 | 3.55E-08 | reported | 3657 | 0.1564 | rs76331127 |  |  |  |  |  |  |  |  |  |  |  |  |  |  |  |
| -0.9269 | 7 | igd | 0.0062 | A | \|\| id:ebi-a-GCST90001784 | ebi-a-GCST90001784 | TRUE | G | 47871972 | 6.27E-09 | reported | 3657 | 0.1592 | rs149662107 |  |  |  |  |  |  |  |  |  |  |  |  |  |  |  |
| 1.198 | 1 | igd | 0.0062 | C | \|\| id:ebi-a-GCST90002102 | ebi-a-GCST90002102 | TRUE | T | 196183282 | 1.58E-11 | reported | 2919 | 0.177 | rs115186385 |  |  |  |  |  |  |  |  |  |  |  |  |  |  |  |
| -0.8623 | 1 | igd | 0.0061 | C | \|\| id:ebi-a-GCST90001536 | ebi-a-GCST90001536 | TRUE | A | 196046115 | 1.22E-08 | reported | 3427 | 0.151 | rs79157236 |  |  |  |  |  |  |  |  |  |  |  |  |  |  |  |
| -1.248 | 16 | igd | 0.0061 | G | \|\| id:ebi-a-GCST90002028 | ebi-a-GCST90002028 | TRUE | A | 32963441 | 2.07E-09 | reported | 3112 | 0.2077 | rs550649515 |  |  |  |  |  |  |  |  |  |  |  |  |  |  |  |
| -1.385 | 6 | igd | 0.0061 | C | \|\| id:ebi-a-GCST90002112 | ebi-a-GCST90002112 | TRUE | G | 32437013 | 1.06E-08 | reported | 1635 | 0.2409 | rs202109233 |  |  |  |  |  |  |  |  |  |  |  |  |  |  |  |
| 96.39 | 17 | igd | 0.006 | T | \|\| id:ebi-a-GCST90001695 | ebi-a-GCST90001695 | TRUE | C | 65645024 | 1.14E-08 | reported | 3408 | 16.84 | rs117941087 |  |  |  |  |  |  |  |  |  |  |  |  |  |  |  |
| 179.8 | 6 | igd | 0.006 | A | \|\| id:ebi-a-GCST90001698 | ebi-a-GCST90001698 | TRUE | G | 3349658 | 2.48E-15 | reported | 3408 | 22.61 | rs75865965 |  |  |  |  |  |  |  |  |  |  |  |  |  |  |  |
| 163.2 | 7 | igd | 0.006 | C | \|\| id:ebi-a-GCST90001698 | ebi-a-GCST90001698 | TRUE | T | 69510130 | 6.03E-12 | reported | 3408 | 23.63 | rs117079032 |  |  |  |  |  |  |  |  |  |  |  |  |  |  |  |
| 129.4 | 10 | igd | 0.006 | A | \|\| id:ebi-a-GCST90001698 | ebi-a-GCST90001698 | TRUE | G | 92224804 | 2.23E-08 | reported | 3408 | 23.09 | rs189333054 |  |  |  |  |  |  |  |  |  |  |  |  |  |  |  |
| 145.3 | 11 | igd | 0.006 | T | \|\| id:ebi-a-GCST90001698 | ebi-a-GCST90001698 | TRUE | G | 130332877 | 1.91E-10 | reported | 3408 | 22.75 | rs76666017 |  |  |  |  |  |  |  |  |  |  |  |  |  |  |  |
| 179.1 | 13 | igd | 0.006 | G | \|\| id:ebi-a-GCST90001698 | ebi-a-GCST90001698 | TRUE | A | 20110927 | 5.43E-15 | reported | 3408 | 22.8 | rs150862589 |  |  |  |  |  |  |  |  |  |  |  |  |  |  |  |
| 1.203 | 1 | igd | 0.006 | A | \|\| id:ebi-a-GCST90002103 | ebi-a-GCST90002103 | TRUE | T | 196210418 | 2.40E-09 | reported | 2650 | 0.2008 | rs79663932 |  |  |  |  |  |  |  |  |  |  |  |  |  |  |  |
| 1.105 | 1 | igd | 0.006 | C | \|\| id:ebi-a-GCST90002103 | ebi-a-GCST90002103 | TRUE | T | 196059008 | 2.50E-09 | reported | 2650 | 0.1847 | rs79001184 |  |  |  |  |  |  |  |  |  |  |  |  |  |  |  |
| 1.207 | 1 | igd | 0.006 | A | \|\| id:ebi-a-GCST90002103 | ebi-a-GCST90002103 | TRUE | G | 196490208 | 1.95E-09 | reported | 2650 | 0.2003 | rs6669247 |  |  |  |  |  |  |  |  |  |  |  |  |  |  |  |
| 1.138 | 1 | igd | 0.0059 | C | \|\| id:ebi-a-GCST90001483 | ebi-a-GCST90001483 | TRUE | T | 197472286 | 3.85E-13 | reported | 3405 | 0.1562 | rs114866384 |  |  |  |  |  |  |  |  |  |  |  |  |  |  |  |
| 1.35 | 1 | igd | 0.0059 | A | \|\| id:ebi-a-GCST90001507 | ebi-a-GCST90001507 | TRUE | T | 196210418 | 5.61E-14 | reported | 3405 | 0.1789 | rs79663932 |  |  |  |  |  |  |  |  |  |  |  |  |  |  |  |
| 1.11 | 1 | igd | 0.0059 | C | \|\| id:ebi-a-GCST90001507 | ebi-a-GCST90001507 | TRUE | T | 197472286 | 1.21E-11 | reported | 3405 | 0.1631 | rs114866384 |  |  |  |  |  |  |  |  |  |  |  |  |  |  |  |
| 0.8597 | 1 | igd | 0.0059 | C | \|\| id:ebi-a-GCST90001666 | ebi-a-GCST90001666 | TRUE | T | 197472286 | 1.39E-09 | reported | 3408 | 0.1416 | rs114866384 |  |  |  |  |  |  |  |  |  |  |  |  |  |  |  |
| -0.8232 | 1 | igd | 0.0059 | C | \|\| id:ebi-a-GCST90001669 | ebi-a-GCST90001669 | TRUE | T | 197472286 | 1.07E-08 | reported | 3408 | 0.1436 | rs114866384 |  |  |  |  |  |  |  |  |  |  |  |  |  |  |  |
| -0.8917 | 1 | igd | 0.0059 | C | \|\| id:ebi-a-GCST90001693 | ebi-a-GCST90001693 | TRUE | T | 197472286 | 3.93E-08 | reported | 3408 | 0.1619 | rs114866384 |  |  |  |  |  |  |  |  |  |  |  |  |  |  |  |
| 139.3 | 1 | igd | 0.0059 | T | \|\| id:ebi-a-GCST90001698 | ebi-a-GCST90001698 | TRUE | C | 81668305 | 3.67E-08 | reported | 3408 | 25.24 | rs114791501 |  |  |  |  |  |  |  |  |  |  |  |  |  |  |  |
| 128 | 2 | igd | 0.0059 | G | \|\| id:ebi-a-GCST90001698 | ebi-a-GCST90001698 | TRUE | A | 66305842 | 3.81E-08 | reported | 3408 | 23.23 | rs79660525 |  |  |  |  |  |  |  |  |  |  |  |  |  |  |  |
| 214.3 | 18 | igd | 0.0059 | G | \|\| id:ebi-a-GCST90001698 | ebi-a-GCST90001698 | TRUE | C | 7850610 | 2.40E-20 | reported | 3408 | 23.04 | rs17473530 |  |  |  |  |  |  |  |  |  |  |  |  |  |  |  |
| 1.163 | 1 | igd | 0.0058 | C | \|\| id:ebi-a-GCST90001485 | ebi-a-GCST90001485 | TRUE | T | 197472286 | 1.03E-13 | reported | 3437 | 0.1557 | rs114866384 |  |  |  |  |  |  |  |  |  |  |  |  |  |  |  |
| 1.179 | 1 | igd | 0.0058 | C | \|\| id:ebi-a-GCST90001508 | ebi-a-GCST90001508 | TRUE | T | 197472286 | 4.41E-13 | reported | 3437 | 0.1622 | rs114866384 |  |  |  |  |  |  |  |  |  |  |  |  |  |  |  |
| 1.177 | 1 | igd | 0.0058 | C | \|\| id:ebi-a-GCST90001509 | ebi-a-GCST90001509 | TRUE | T | 197472286 | 4.66E-13 | reported | 3437 | 0.1621 | rs114866384 |  |  |  |  |  |  |  |  |  |  |  |  |  |  |  |
| -0.9041 | 1 | igd | 0.0058 | A | \|\| id:ebi-a-GCST90001535 | ebi-a-GCST90001535 | TRUE | T | 196210418 | 3.51E-08 | reported | 3427 | 0.1636 | rs79663932 |  |  |  |  |  |  |  |  |  |  |  |  |  |  |  |
| 0.8396 | 1 | igd | 0.0058 | C | \|\| id:ebi-a-GCST90001561 | ebi-a-GCST90001561 | TRUE | T | 197472286 | 9.46E-10 | reported | 3427 | 0.1368 | rs114866384 |  |  |  |  |  |  |  |  |  |  |  |  |  |  |  |
| 0.9494 | 1 | igd | 0.0058 | A | \|\| id:ebi-a-GCST90001561 | ebi-a-GCST90001561 | TRUE | T | 196210418 | 3.03E-10 | reported | 3427 | 0.1503 | rs79663932 |  |  |  |  |  |  |  |  |  |  |  |  |  |  |  |
| 0.9568 | 1 | igd | 0.0058 | C | \|\| id:ebi-a-GCST90001664 | ebi-a-GCST90001664 | TRUE | T | 197472286 | 1.23E-10 | reported | 3440 | 0.1482 | rs114866384 |  |  |  |  |  |  |  |  |  |  |  |  |  |  |  |
| 1.065 | 1 | igd | 0.0058 | C | \|\| id:ebi-a-GCST90001665 | ebi-a-GCST90001665 | TRUE | T | 197472286 | 1.88E-11 | reported | 3440 | 0.1581 | rs114866384 |  |  |  |  |  |  |  |  |  |  |  |  |  |  |  |
| -0.8638 | 1 | igd | 0.0058 | C | \|\| id:ebi-a-GCST90001667 | ebi-a-GCST90001667 | TRUE | T | 197472286 | 1.03E-08 | reported | 3440 | 0.1505 | rs114866384 |  |  |  |  |  |  |  |  |  |  |  |  |  |  |  |
| -1.095 | 1 | igd | 0.0058 | A | \|\| id:ebi-a-GCST90001668 | ebi-a-GCST90001668 | TRUE | T | 196210418 | 3.19E-10 | reported | 3440 | 0.1736 | rs79663932 |  |  |  |  |  |  |  |  |  |  |  |  |  |  |  |
| -0.9991 | 1 | igd | 0.0058 | C | \|\| id:ebi-a-GCST90001691 | ebi-a-GCST90001691 | TRUE | T | 197472286 | 5.72E-10 | reported | 3440 | 0.1607 | rs114866384 |  |  |  |  |  |  |  |  |  |  |  |  |  |  |  |
| -1.129 | 1 | igd | 0.0058 | A | \|\| id:ebi-a-GCST90001691 | ebi-a-GCST90001691 | TRUE | T | 196210418 | 1.81E-10 | reported | 3440 | 0.1765 | rs79663932 |  |  |  |  |  |  |  |  |  |  |  |  |  |  |  |
| -1.172 | 1 | igd | 0.0058 | A | \|\| id:ebi-a-GCST90001692 | ebi-a-GCST90001692 | TRUE | T | 196210418 | 2.81E-11 | reported | 3440 | 0.1755 | rs79663932 |  |  |  |  |  |  |  |  |  |  |  |  |  |  |  |
| 1.104 | 1 | igd | 0.0058 | C | \|\| id:ebi-a-GCST90002098 | ebi-a-GCST90002098 | TRUE | T | 196059008 | 1.25E-10 | reported | 2910 | 0.1709 | rs79001184 |  |  |  |  |  |  |  |  |  |  |  |  |  |  |  |
| 1.324 | 1 | igd | 0.0058 | A | \|\| id:ebi-a-GCST90002098 | ebi-a-GCST90002098 | TRUE | G | 196490208 | 8.95E-13 | reported | 2910 | 0.1844 | rs6669247 |  |  |  |  |  |  |  |  |  |  |  |  |  |  |  |
| 1.119 | 1 | igd | 0.0058 | G | \|\| id:ebi-a-GCST90002103 | ebi-a-GCST90002103 | TRUE | C | 196915125 | 4.65E-09 | reported | 2650 | 0.1903 | rs573537686 |  |  |  |  |  |  |  |  |  |  |  |  |  |  |  |
| -1.276 | 6 | igd | 0.0058 | T | \|\| id:ebi-a-GCST90002112 | ebi-a-GCST90002112 | TRUE | G | 32303474 | 2.94E-08 | reported | 1635 | 0.2291 | rs28732191 |  |  |  |  |  |  |  |  |  |  |  |  |  |  |  |
| 1.178 | 1 | igd | 0.0057 | A | \|\| id:ebi-a-GCST90001483 | ebi-a-GCST90001483 | TRUE | T | 197816763 | 3.07E-13 | reported | 3405 | 0.1609 | rs114156678 |  |  |  |  |  |  |  |  |  |  |  |  |  |  |  |
| 1.228 | 1 | igd | 0.0057 | A | \|\| id:ebi-a-GCST90001485 | ebi-a-GCST90001485 | TRUE | T | 197816763 | 2.36E-14 | reported | 3437 | 0.1603 | rs114156678 |  |  |  |  |  |  |  |  |  |  |  |  |  |  |  |
| 1.177 | 1 | igd | 0.0057 | A | \|\| id:ebi-a-GCST90001507 | ebi-a-GCST90001507 | TRUE | T | 197816763 | 2.82E-12 | reported | 3405 | 0.1679 | rs114156678 |  |  |  |  |  |  |  |  |  |  |  |  |  |  |  |
| 1.282 | 1 | igd | 0.0057 | A | \|\| id:ebi-a-GCST90001508 | ebi-a-GCST90001508 | TRUE | T | 197816763 | 1.81E-14 | reported | 3437 | 0.1666 | rs114156678 |  |  |  |  |  |  |  |  |  |  |  |  |  |  |  |
| 1.277 | 1 | igd | 0.0057 | A | \|\| id:ebi-a-GCST90001509 | ebi-a-GCST90001509 | TRUE | T | 197816763 | 2.27E-14 | reported | 3437 | 0.1665 | rs114156678 |  |  |  |  |  |  |  |  |  |  |  |  |  |  |  |
| 1.244 | 5 | igd | 0.0057 | T | \|\| id:ebi-a-GCST90001521 | ebi-a-GCST90001521 | TRUE | C | 112408670 | 3.04E-08 | reported | 1858 | 0.2236 | rs111231787 |  |  |  |  |  |  |  |  |  |  |  |  |  |  |  |
| 0.9084 | 1 | igd | 0.0057 | A | \|\| id:ebi-a-GCST90001561 | ebi-a-GCST90001561 | TRUE | T | 197816763 | 1.20E-10 | reported | 3427 | 0.1406 | rs114156678 |  |  |  |  |  |  |  |  |  |  |  |  |  |  |  |
| 1.152 | 1 | igd | 0.0057 | A | \|\| id:ebi-a-GCST90001664 | ebi-a-GCST90001664 | TRUE | T | 197816763 | 4.66E-14 | reported | 3440 | 0.1522 | rs114156678 |  |  |  |  |  |  |  |  |  |  |  |  |  |  |  |
| 1.214 | 1 | igd | 0.0057 | A | \|\| id:ebi-a-GCST90001665 | ebi-a-GCST90001665 | TRUE | T | 197816763 | 9.02E-14 | reported | 3440 | 0.1622 | rs114156678 |  |  |  |  |  |  |  |  |  |  |  |  |  |  |  |
| 1.011 | 1 | igd | 0.0057 | A | \|\| id:ebi-a-GCST90001666 | ebi-a-GCST90001666 | TRUE | T | 197816763 | 4.14E-12 | reported | 3408 | 0.1452 | rs114156678 |  |  |  |  |  |  |  |  |  |  |  |  |  |  |  |
| -0.9699 | 1 | igd | 0.0057 | A | \|\| id:ebi-a-GCST90001667 | ebi-a-GCST90001667 | TRUE | T | 197816763 | 4.04E-10 | reported | 3440 | 0.1547 | rs114156678 |  |  |  |  |  |  |  |  |  |  |  |  |  |  |  |
| -0.9213 | 1 | igd | 0.0057 | A | \|\| id:ebi-a-GCST90001668 | ebi-a-GCST90001668 | TRUE | T | 197816763 | 1.55E-08 | reported | 3440 | 0.1625 | rs114156678 |  |  |  |  |  |  |  |  |  |  |  |  |  |  |  |
| -0.9659 | 1 | igd | 0.0057 | A | \|\| id:ebi-a-GCST90001669 | ebi-a-GCST90001669 | TRUE | T | 197816763 | 6.66E-11 | reported | 3408 | 0.1475 | rs114156678 |  |  |  |  |  |  |  |  |  |  |  |  |  |  |  |
| -1.119 | 1 | igd | 0.0057 | A | \|\| id:ebi-a-GCST90001691 | ebi-a-GCST90001691 | TRUE | T | 197816763 | 1.53E-11 | reported | 3440 | 0.1653 | rs114156678 |  |  |  |  |  |  |  |  |  |  |  |  |  |  |  |
| -0.9003 | 1 | igd | 0.0057 | A | \|\| id:ebi-a-GCST90001692 | ebi-a-GCST90001692 | TRUE | T | 197816763 | 4.74E-08 | reported | 3440 | 0.1645 | rs114156678 |  |  |  |  |  |  |  |  |  |  |  |  |  |  |  |
| -1.053 | 1 | igd | 0.0057 | A | \|\| id:ebi-a-GCST90001693 | ebi-a-GCST90001693 | TRUE | T | 197816763 | 2.82E-10 | reported | 3408 | 0.1664 | rs114156678 |  |  |  |  |  |  |  |  |  |  |  |  |  |  |  |
| 118.8 | 16 | igd | 0.0057 | G | \|\| id:ebi-a-GCST90001695 | ebi-a-GCST90001695 | TRUE | A | 75062625 | 2.27E-12 | reported | 3408 | 16.87 | rs149716976 |  |  |  |  |  |  |  |  |  |  |  |  |  |  |  |
| 4.577 | 16 | igd | 0.0057 | G | \|\| id:ebi-a-GCST90001697 | ebi-a-GCST90001697 | TRUE | A | 75062625 | 7.53E-09 | reported | 3425 | 0.79 | rs149716976 |  |  |  |  |  |  |  |  |  |  |  |  |  |  |  |
| 161.1 | 4 | igd | 0.0057 | T | \|\| id:ebi-a-GCST90001698 | ebi-a-GCST90001698 | TRUE | C | 72089575 | 1.61E-10 | reported | 3408 | 25.11 | rs149160429 |  |  |  |  |  |  |  |  |  |  |  |  |  |  |  |
| 129.7 | 4 | igd | 0.0057 | A | \|\| id:ebi-a-GCST90001698 | ebi-a-GCST90001698 | TRUE | C | 33082406 | 4.77E-08 | reported | 3408 | 23.7 | rs78500482 |  |  |  |  |  |  |  |  |  |  |  |  |  |  |  |
| 169.6 | 6 | igd | 0.0057 | A | \|\| id:ebi-a-GCST90001698 | ebi-a-GCST90001698 | TRUE | G | 40616989 | 4.38E-13 | reported | 3408 | 23.32 | rs12332976 |  |  |  |  |  |  |  |  |  |  |  |  |  |  |  |
| 183.9 | 10 | igd | 0.0057 | G | \|\| id:ebi-a-GCST90001698 | ebi-a-GCST90001698 | TRUE | A | 54704473 | 2.32E-16 | reported | 3408 | 22.31 | rs147968748 |  |  |  |  |  |  |  |  |  |  |  |  |  |  |  |
| 139.6 | 18 | igd | 0.0057 | A | \|\| id:ebi-a-GCST90001698 | ebi-a-GCST90001698 | TRUE | G | 55183945 | 7.22E-09 | reported | 3408 | 24.06 | rs17685554 |  |  |  |  |  |  |  |  |  |  |  |  |  |  |  |
| -1.129 | 22 | igd | 0.0057 | G | \|\| id:ebi-a-GCST90001702 | ebi-a-GCST90001702 | TRUE | A | 41943950 | 1.48E-11 | reported | 3657 | 0.1668 | rs148407669 |  |  |  |  |  |  |  |  |  |  |  |  |  |  |  |
| -0.902 | 22 | igd | 0.0057 | T | \|\| id:ebi-a-GCST90001702 | ebi-a-GCST90001702 | TRUE | C | 41610240 | 2.98E-08 | reported | 3657 | 0.1624 | rs112391039 |  |  |  |  |  |  |  |  |  |  |  |  |  |  |  |
| -0.9156 | 22 | igd | 0.0057 | T | \|\| id:ebi-a-GCST90001703 | ebi-a-GCST90001703 | TRUE | C | 41610240 | 1.84E-08 | reported | 3657 | 0.1624 | rs112391039 |  |  |  |  |  |  |  |  |  |  |  |  |  |  |  |
| -1.122 | 22 | igd | 0.0057 | G | \|\| id:ebi-a-GCST90001703 | ebi-a-GCST90001703 | TRUE | A | 41943950 | 1.99E-11 | reported | 3657 | 0.1668 | rs148407669 |  |  |  |  |  |  |  |  |  |  |  |  |  |  |  |
| -1.125 | 22 | igd | 0.0057 | G | \|\| id:ebi-a-GCST90001704 | ebi-a-GCST90001704 | TRUE | A | 41943950 | 1.65E-11 | reported | 3657 | 0.1666 | rs148407669 |  |  |  |  |  |  |  |  |  |  |  |  |  |  |  |
| -0.9132 | 22 | igd | 0.0057 | T | \|\| id:ebi-a-GCST90001704 | ebi-a-GCST90001704 | TRUE | C | 41610240 | 1.93E-08 | reported | 3657 | 0.1622 | rs112391039 |  |  |  |  |  |  |  |  |  |  |  |  |  |  |  |
| -1.109 | 22 | igd | 0.0057 | G | \|\| id:ebi-a-GCST90001710 | ebi-a-GCST90001710 | TRUE | A | 41943950 | 3.00E-11 | reported | 3657 | 0.1663 | rs148407669 |  |  |  |  |  |  |  |  |  |  |  |  |  |  |  |
| -1.112 | 22 | igd | 0.0057 | G | \|\| id:ebi-a-GCST90001711 | ebi-a-GCST90001711 | TRUE | A | 41943950 | 2.36E-11 | reported | 3657 | 0.166 | rs148407669 |  |  |  |  |  |  |  |  |  |  |  |  |  |  |  |
| -0.886 | 22 | igd | 0.0057 | T | \|\| id:ebi-a-GCST90001711 | ebi-a-GCST90001711 | TRUE | C | 41610240 | 4.49E-08 | reported | 3657 | 0.1616 | rs112391039 |  |  |  |  |  |  |  |  |  |  |  |  |  |  |  |
| -1.032 | 16 | igd | 0.0057 | G | \|\| id:ebi-a-GCST90001729 | ebi-a-GCST90001729 | TRUE | A | 32963441 | 3.45E-08 | reported | 3657 | 0.1866 | rs550649515 |  |  |  |  |  |  |  |  |  |  |  |  |  |  |  |
| 1.094 | 1 | igd | 0.0057 | C | \|\| id:ebi-a-GCST90002098 | ebi-a-GCST90002098 | TRUE | T | 197472286 | 4.56E-11 | reported | 2910 | 0.1655 | rs114866384 |  |  |  |  |  |  |  |  |  |  |  |  |  |  |  |
| 1.199 | 1 | igd | 0.0057 | G | \|\| id:ebi-a-GCST90002098 | ebi-a-GCST90002098 | TRUE | C | 196915125 | 1.28E-11 | reported | 2910 | 0.1763 | rs573537686 |  |  |  |  |  |  |  |  |  |  |  |  |  |  |  |
| 1.189 | 1 | igd | 0.0057 | A | \|\| id:ebi-a-GCST90002098 | ebi-a-GCST90002098 | TRUE | T | 197816763 | 2.26E-12 | reported | 2910 | 0.1686 | rs114156678 |  |  |  |  |  |  |  |  |  |  |  |  |  |  |  |
| 1.289 | 1 | igd | 0.0057 | G | \|\| id:ebi-a-GCST90002103 | ebi-a-GCST90002103 | TRUE | A | 197141261 | 4.60E-10 | reported | 2650 | 0.206 | rs45512792 |  |  |  |  |  |  |  |  |  |  |  |  |  |  |  |
| 181.6 | 2 | igd | 0.0056 | T | \|\| id:ebi-a-GCST90001698 | ebi-a-GCST90001698 | TRUE | G | 107911125 | 1.00E-15 | reported | 3408 | 22.52 | rs567695407 |  |  |  |  |  |  |  |  |  |  |  |  |  |  |  |
| 152.8 | 12 | igd | 0.0056 | T | \|\| id:ebi-a-GCST90001698 | ebi-a-GCST90001698 | TRUE | C | 100494123 | 1.58E-10 | reported | 3408 | 23.81 | rs146661200 |  |  |  |  |  |  |  |  |  |  |  |  |  |  |  |
| 1.437 | 1 | igd | 0.0055 | G | \|\| id:ebi-a-GCST90002098 | ebi-a-GCST90002098 | TRUE | A | 197141261 | 4.40E-14 | reported | 2910 | 0.1894 | rs45512792 |  |  |  |  |  |  |  |  |  |  |  |  |  |  |  |
| 1.369 | 1 | igd | 0.0055 | A | \|\| id:ebi-a-GCST90002102 | ebi-a-GCST90002102 | TRUE | G | 196490208 | 8.80E-13 | reported | 2919 | 0.1907 | rs6669247 |  |  |  |  |  |  |  |  |  |  |  |  |  |  |  |
| 1.117 | 1 | igd | 0.0055 | C | \|\| id:ebi-a-GCST90002102 | ebi-a-GCST90002102 | TRUE | T | 196059008 | 2.58E-10 | reported | 2919 | 0.176 | rs79001184 |  |  |  |  |  |  |  |  |  |  |  |  |  |  |  |
| 1.571 | 1 | igd | 0.0054 | A | \|\| id:ebi-a-GCST90001483 | ebi-a-GCST90001483 | TRUE | G | 196490208 | 6.85E-18 | reported | 3405 | 0.1813 | rs6669247 |  |  |  |  |  |  |  |  |  |  |  |  |  |  |  |
| 1.312 | 1 | igd | 0.0054 | C | \|\| id:ebi-a-GCST90001483 | ebi-a-GCST90001483 | TRUE | T | 196059008 | 9.86E-15 | reported | 3405 | 0.1688 | rs79001184 |  |  |  |  |  |  |  |  |  |  |  |  |  |  |  |
| 1.051 | 1 | igd | 0.0054 | A | \|\| id:ebi-a-GCST90001484 | ebi-a-GCST90001484 | TRUE | G | 196490208 | 8.90E-09 | reported | 3437 | 0.1822 | rs6669247 |  |  |  |  |  |  |  |  |  |  |  |  |  |  |  |
| 1.645 | 1 | igd | 0.0054 | A | \|\| id:ebi-a-GCST90001485 | ebi-a-GCST90001485 | TRUE | G | 196490208 | 1.45E-19 | reported | 3437 | 0.1807 | rs6669247 |  |  |  |  |  |  |  |  |  |  |  |  |  |  |  |
| 1.322 | 1 | igd | 0.0054 | C | \|\| id:ebi-a-GCST90001485 | ebi-a-GCST90001485 | TRUE | T | 196059008 | 5.23E-15 | reported | 3437 | 0.1683 | rs79001184 |  |  |  |  |  |  |  |  |  |  |  |  |  |  |  |
| 1.401 | 1 | igd | 0.0054 | C | \|\| id:ebi-a-GCST90001507 | ebi-a-GCST90001507 | TRUE | T | 196059008 | 2.41E-15 | reported | 3405 | 0.1761 | rs79001184 |  |  |  |  |  |  |  |  |  |  |  |  |  |  |  |
| 1.493 | 1 | igd | 0.0054 | A | \|\| id:ebi-a-GCST90001507 | ebi-a-GCST90001507 | TRUE | G | 196490208 | 4.82E-15 | reported | 3405 | 0.1898 | rs6669247 |  |  |  |  |  |  |  |  |  |  |  |  |  |  |  |
| 1.56 | 1 | igd | 0.0054 | A | \|\| id:ebi-a-GCST90001508 | ebi-a-GCST90001508 | TRUE | G | 196490208 | 1.93E-16 | reported | 3437 | 0.1886 | rs6669247 |  |  |  |  |  |  |  |  |  |  |  |  |  |  |  |
| 1.404 | 1 | igd | 0.0054 | C | \|\| id:ebi-a-GCST90001508 | ebi-a-GCST90001508 | TRUE | T | 196059008 | 1.47E-15 | reported | 3437 | 0.1752 | rs79001184 |  |  |  |  |  |  |  |  |  |  |  |  |  |  |  |
| 1.323 | 1 | igd | 0.0054 | C | \|\| id:ebi-a-GCST90001509 | ebi-a-GCST90001509 | TRUE | T | 196059008 | 5.68E-14 | reported | 3437 | 0.1752 | rs79001184 |  |  |  |  |  |  |  |  |  |  |  |  |  |  |  |
| 1.461 | 1 | igd | 0.0054 | A | \|\| id:ebi-a-GCST90001509 | ebi-a-GCST90001509 | TRUE | G | 196490208 | 1.30E-14 | reported | 3437 | 0.1888 | rs6669247 |  |  |  |  |  |  |  |  |  |  |  |  |  |  |  |
| -1.18 | 1 | igd | 0.0054 | A | \|\| id:ebi-a-GCST90001511 | ebi-a-GCST90001511 | TRUE | G | 196490208 | 4.51E-10 | reported | 3437 | 0.1887 | rs6669247 |  |  |  |  |  |  |  |  |  |  |  |  |  |  |  |
| -0.9894 | 1 | igd | 0.0054 | C | \|\| id:ebi-a-GCST90001511 | ebi-a-GCST90001511 | TRUE | T | 196059008 | 1.81E-08 | reported | 3437 | 0.1753 | rs79001184 |  |  |  |  |  |  |  |  |  |  |  |  |  |  |  |
| -1.107 | 1 | igd | 0.0054 | A | \|\| id:ebi-a-GCST90001512 | ebi-a-GCST90001512 | TRUE | G | 196490208 | 4.91E-09 | reported | 3437 | 0.1887 | rs6669247 |  |  |  |  |  |  |  |  |  |  |  |  |  |  |  |
| -0.9936 | 1 | igd | 0.0054 | A | \|\| id:ebi-a-GCST90001535 | ebi-a-GCST90001535 | TRUE | G | 196490208 | 1.14E-08 | reported | 3427 | 0.1736 | rs6669247 |  |  |  |  |  |  |  |  |  |  |  |  |  |  |  |
| -0.9278 | 1 | igd | 0.0054 | C | \|\| id:ebi-a-GCST90001535 | ebi-a-GCST90001535 | TRUE | T | 196059008 | 9.42E-09 | reported | 3427 | 0.1612 | rs79001184 |  |  |  |  |  |  |  |  |  |  |  |  |  |  |  |
| -0.9929 | 1 | igd | 0.0054 | A | \|\| id:ebi-a-GCST90001536 | ebi-a-GCST90001536 | TRUE | G | 196490208 | 1.76E-08 | reported | 3427 | 0.1758 | rs6669247 |  |  |  |  |  |  |  |  |  |  |  |  |  |  |  |
| 0.9132 | 1 | igd | 0.0054 | C | \|\| id:ebi-a-GCST90001561 | ebi-a-GCST90001561 | TRUE | T | 196059008 | 7.74E-10 | reported | 3427 | 0.1481 | rs79001184 |  |  |  |  |  |  |  |  |  |  |  |  |  |  |  |
| 1.09 | 1 | igd | 0.0054 | A | \|\| id:ebi-a-GCST90001561 | ebi-a-GCST90001561 | TRUE | G | 196490208 | 9.12E-12 | reported | 3427 | 0.1593 | rs6669247 |  |  |  |  |  |  |  |  |  |  |  |  |  |  |  |
| 1.359 | 1 | igd | 0.0054 | A | \|\| id:ebi-a-GCST90001664 | ebi-a-GCST90001664 | TRUE | G | 196490208 | 4.22E-15 | reported | 3440 | 0.1723 | rs6669247 |  |  |  |  |  |  |  |  |  |  |  |  |  |  |  |
| 1.039 | 1 | igd | 0.0054 | C | \|\| id:ebi-a-GCST90001664 | ebi-a-GCST90001664 | TRUE | T | 196059008 | 1.09E-10 | reported | 3440 | 0.1604 | rs79001184 |  |  |  |  |  |  |  |  |  |  |  |  |  |  |  |
| 1.436 | 1 | igd | 0.0054 | A | \|\| id:ebi-a-GCST90001665 | ebi-a-GCST90001665 | TRUE | G | 196490208 | 7.31E-15 | reported | 3440 | 0.1838 | rs6669247 |  |  |  |  |  |  |  |  |  |  |  |  |  |  |  |
| 1.109 | 1 | igd | 0.0054 | C | \|\| id:ebi-a-GCST90001665 | ebi-a-GCST90001665 | TRUE | T | 196059008 | 1.03E-10 | reported | 3440 | 0.1711 | rs79001184 |  |  |  |  |  |  |  |  |  |  |  |  |  |  |  |
| 1.031 | 1 | igd | 0.0054 | C | \|\| id:ebi-a-GCST90001666 | ebi-a-GCST90001666 | TRUE | T | 196059008 | 1.84E-11 | reported | 3408 | 0.1529 | rs79001184 |  |  |  |  |  |  |  |  |  |  |  |  |  |  |  |
| 1.275 | 1 | igd | 0.0054 | A | \|\| id:ebi-a-GCST90001666 | ebi-a-GCST90001666 | TRUE | G | 196490208 | 1.17E-14 | reported | 3408 | 0.1644 | rs6669247 |  |  |  |  |  |  |  |  |  |  |  |  |  |  |  |
| -1.27 | 1 | igd | 0.0054 | A | \|\| id:ebi-a-GCST90001667 | ebi-a-GCST90001667 | TRUE | G | 196490208 | 4.79E-13 | reported | 3440 | 0.175 | rs6669247 |  |  |  |  |  |  |  |  |  |  |  |  |  |  |  |
| -1.118 | 1 | igd | 0.0054 | C | \|\| id:ebi-a-GCST90001667 | ebi-a-GCST90001667 | TRUE | T | 196059008 | 7.21E-12 | reported | 3440 | 0.1625 | rs79001184 |  |  |  |  |  |  |  |  |  |  |  |  |  |  |  |
| -1.179 | 1 | igd | 0.0054 | A | \|\| id:ebi-a-GCST90001668 | ebi-a-GCST90001668 | TRUE | G | 196490208 | 1.67E-10 | reported | 3440 | 0.184 | rs6669247 |  |  |  |  |  |  |  |  |  |  |  |  |  |  |  |
| -1.043 | 1 | igd | 0.0054 | C | \|\| id:ebi-a-GCST90001668 | ebi-a-GCST90001668 | TRUE | T | 196059008 | 1.17E-09 | reported | 3440 | 0.1709 | rs79001184 |  |  |  |  |  |  |  |  |  |  |  |  |  |  |  |
| -1.12 | 1 | igd | 0.0054 | A | \|\| id:ebi-a-GCST90001669 | ebi-a-GCST90001669 | TRUE | G | 196490208 | 2.42E-11 | reported | 3408 | 0.1671 | rs6669247 |  |  |  |  |  |  |  |  |  |  |  |  |  |  |  |
| -0.9227 | 1 | igd | 0.0054 | C | \|\| id:ebi-a-GCST90001669 | ebi-a-GCST90001669 | TRUE | T | 196059008 | 3.10E-09 | reported | 3408 | 0.1553 | rs79001184 |  |  |  |  |  |  |  |  |  |  |  |  |  |  |  |
| -1.08 | 1 | igd | 0.0054 | C | \|\| id:ebi-a-GCST90001691 | ebi-a-GCST90001691 | TRUE | T | 196059008 | 6.13E-10 | reported | 3440 | 0.174 | rs79001184 |  |  |  |  |  |  |  |  |  |  |  |  |  |  |  |
| -1.194 | 1 | igd | 0.0054 | A | \|\| id:ebi-a-GCST90001691 | ebi-a-GCST90001691 | TRUE | G | 196490208 | 2.11E-10 | reported | 3440 | 0.1874 | rs6669247 |  |  |  |  |  |  |  |  |  |  |  |  |  |  |  |
| -1.28 | 1 | igd | 0.0054 | A | \|\| id:ebi-a-GCST90001692 | ebi-a-GCST90001692 | TRUE | G | 196490208 | 7.19E-12 | reported | 3440 | 0.1861 | rs6669247 |  |  |  |  |  |  |  |  |  |  |  |  |  |  |  |
| -1.162 | 1 | igd | 0.0054 | C | \|\| id:ebi-a-GCST90001692 | ebi-a-GCST90001692 | TRUE | T | 196059008 | 1.99E-11 | reported | 3440 | 0.1727 | rs79001184 |  |  |  |  |  |  |  |  |  |  |  |  |  |  |  |
| 139.5 | 3 | igd | 0.0054 | A | \|\| id:ebi-a-GCST90001698 | ebi-a-GCST90001698 | TRUE | G | 166256176 | 3.08E-08 | reported | 3408 | 25.13 | rs138401052 |  |  |  |  |  |  |  |  |  |  |  |  |  |  |  |
| 160.1 | 3 | igd | 0.0054 | T | \|\| id:ebi-a-GCST90001698 | ebi-a-GCST90001698 | TRUE | C | 64543612 | 1.21E-11 | reported | 3408 | 23.53 | rs115040341 |  |  |  |  |  |  |  |  |  |  |  |  |  |  |  |
| 137.1 | 12 | igd | 0.0054 | T | \|\| id:ebi-a-GCST90001698 | ebi-a-GCST90001698 | TRUE | C | 98582780 | 4.28E-09 | reported | 3408 | 23.28 | rs187229204 |  |  |  |  |  |  |  |  |  |  |  |  |  |  |  |
| 1.485 | 1 | igd | 0.0053 | G | \|\| id:ebi-a-GCST90001483 | ebi-a-GCST90001483 | TRUE | C | 196915125 | 1.81E-17 | reported | 3405 | 0.1737 | rs573537686 |  |  |  |  |  |  |  |  |  |  |  |  |  |  |  |
| 1.373 | 1 | igd | 0.0053 | G | \|\| id:ebi-a-GCST90001507 | ebi-a-GCST90001507 | TRUE | C | 196915125 | 5.60E-14 | reported | 3405 | 0.1819 | rs573537686 |  |  |  |  |  |  |  |  |  |  |  |  |  |  |  |
| -0.9109 | 1 | igd | 0.0053 | G | \|\| id:ebi-a-GCST90001535 | ebi-a-GCST90001535 | TRUE | C | 196915125 | 4.66E-08 | reported | 3427 | 0.1663 | rs573537686 |  |  |  |  |  |  |  |  |  |  |  |  |  |  |  |
| -0.9339 | 1 | igd | 0.0053 | G | \|\| id:ebi-a-GCST90001536 | ebi-a-GCST90001536 | TRUE | C | 196915125 | 3.14E-08 | reported | 3427 | 0.1684 | rs573537686 |  |  |  |  |  |  |  |  |  |  |  |  |  |  |  |
| 0.9799 | 1 | igd | 0.0053 | G | \|\| id:ebi-a-GCST90001561 | ebi-a-GCST90001561 | TRUE | C | 196915125 | 1.57E-10 | reported | 3427 | 0.1527 | rs573537686 |  |  |  |  |  |  |  |  |  |  |  |  |  |  |  |
| 1.157 | 1 | igd | 0.0053 | G | \|\| id:ebi-a-GCST90001666 | ebi-a-GCST90001666 | TRUE | C | 196915125 | 2.57E-13 | reported | 3408 | 0.1576 | rs573537686 |  |  |  |  |  |  |  |  |  |  |  |  |  |  |  |
| -0.9846 | 1 | igd | 0.0053 | G | \|\| id:ebi-a-GCST90001669 | ebi-a-GCST90001669 | TRUE | C | 196915125 | 8.87E-10 | reported | 3408 | 0.1602 | rs573537686 |  |  |  |  |  |  |  |  |  |  |  |  |  |  |  |
| 101.4 | 12 | igd | 0.0053 | G | \|\| id:ebi-a-GCST90001695 | ebi-a-GCST90001695 | TRUE | A | 13243168 | 3.25E-08 | reported | 3408 | 18.3 | rs17212998 |  |  |  |  |  |  |  |  |  |  |  |  |  |  |  |
| 143.9 | 12 | igd | 0.0053 | T | \|\| id:ebi-a-GCST90001698 | ebi-a-GCST90001698 | TRUE | C | 79375516 | 5.64E-09 | reported | 3408 | 24.63 | rs148788593 |  |  |  |  |  |  |  |  |  |  |  |  |  |  |  |
| 145.4 | 13 | igd | 0.0053 | C | \|\| id:ebi-a-GCST90001698 | ebi-a-GCST90001698 | TRUE | A | 109351448 | 1.20E-09 | reported | 3408 | 23.84 | rs575342165 |  |  |  |  |  |  |  |  |  |  |  |  |  |  |  |
| 163.6 | 16 | igd | 0.0053 | C | \|\| id:ebi-a-GCST90001698 | ebi-a-GCST90001698 | TRUE | T | 11614838 | 8.71E-09 | reported | 3408 | 28.37 | rs79548047 |  |  |  |  |  |  |  |  |  |  |  |  |  |  |  |
| 161.6 | 16 | igd | 0.0053 | G | \|\| id:ebi-a-GCST90001698 | ebi-a-GCST90001698 | TRUE | A | 69023689 | 8.64E-11 | reported | 3408 | 24.83 | rs143946135 |  |  |  |  |  |  |  |  |  |  |  |  |  |  |  |
| 1.23 | 1 | igd | 0.0053 | G | \|\| id:ebi-a-GCST90002102 | ebi-a-GCST90002102 | TRUE | C | 196915125 | 1.59E-11 | reported | 2919 | 0.1818 | rs573537686 |  |  |  |  |  |  |  |  |  |  |  |  |  |  |  |
| 1.287 | 1 | igd | 0.0053 | A | \|\| id:ebi-a-GCST90002102 | ebi-a-GCST90002102 | TRUE | T | 197816763 | 1.31E-13 | reported | 2919 | 0.1729 | rs114156678 |  |  |  |  |  |  |  |  |  |  |  |  |  |  |  |
| 1.165 | 1 | igd | 0.0053 | C | \|\| id:ebi-a-GCST90002102 | ebi-a-GCST90002102 | TRUE | T | 197472286 | 8.44E-12 | reported | 2919 | 0.1699 | rs114866384 |  |  |  |  |  |  |  |  |  |  |  |  |  |  |  |
| 1.064 | 1 | igd | 0.0053 | A | \|\| id:ebi-a-GCST90002103 | ebi-a-GCST90002103 | TRUE | T | 197816763 | 3.88E-08 | reported | 2650 | 0.193 | rs114156678 |  |  |  |  |  |  |  |  |  |  |  |  |  |  |  |
| 1.025 | 1 | igd | 0.0052 | G | \|\| id:ebi-a-GCST90001484 | ebi-a-GCST90001484 | TRUE | C | 196915125 | 4.65E-09 | reported | 3437 | 0.1745 | rs573537686 |  |  |  |  |  |  |  |  |  |  |  |  |  |  |  |
| 1.565 | 1 | igd | 0.0052 | G | \|\| id:ebi-a-GCST90001485 | ebi-a-GCST90001485 | TRUE | C | 196915125 | 2.45E-19 | reported | 3437 | 0.1731 | rs573537686 |  |  |  |  |  |  |  |  |  |  |  |  |  |  |  |
| 1.434 | 1 | igd | 0.0052 | G | \|\| id:ebi-a-GCST90001508 | ebi-a-GCST90001508 | TRUE | C | 196915125 | 2.79E-15 | reported | 3437 | 0.1807 | rs573537686 |  |  |  |  |  |  |  |  |  |  |  |  |  |  |  |
| 1.337 | 1 | igd | 0.0052 | G | \|\| id:ebi-a-GCST90001509 | ebi-a-GCST90001509 | TRUE | C | 196915125 | 1.81E-13 | reported | 3437 | 0.1809 | rs573537686 |  |  |  |  |  |  |  |  |  |  |  |  |  |  |  |
| -1.082 | 1 | igd | 0.0052 | G | \|\| id:ebi-a-GCST90001511 | ebi-a-GCST90001511 | TRUE | C | 196915125 | 2.37E-09 | reported | 3437 | 0.1808 | rs573537686 |  |  |  |  |  |  |  |  |  |  |  |  |  |  |  |
| -1.031 | 1 | igd | 0.0052 | G | \|\| id:ebi-a-GCST90001512 | ebi-a-GCST90001512 | TRUE | C | 196915125 | 1.28E-08 | reported | 3437 | 0.1808 | rs573537686 |  |  |  |  |  |  |  |  |  |  |  |  |  |  |  |
| 1.231 | 1 | igd | 0.0052 | G | \|\| id:ebi-a-GCST90001664 | ebi-a-GCST90001664 | TRUE | C | 196915125 | 1.13E-13 | reported | 3440 | 0.1652 | rs573537686 |  |  |  |  |  |  |  |  |  |  |  |  |  |  |  |
| 1.281 | 1 | igd | 0.0052 | G | \|\| id:ebi-a-GCST90001665 | ebi-a-GCST90001665 | TRUE | C | 196915125 | 4.30E-13 | reported | 3440 | 0.1762 | rs573537686 |  |  |  |  |  |  |  |  |  |  |  |  |  |  |  |
| -1.122 | 1 | igd | 0.0052 | G | \|\| id:ebi-a-GCST90001667 | ebi-a-GCST90001667 | TRUE | C | 196915125 | 2.57E-11 | reported | 3440 | 0.1677 | rs573537686 |  |  |  |  |  |  |  |  |  |  |  |  |  |  |  |
| -1.061 | 1 | igd | 0.0052 | G | \|\| id:ebi-a-GCST90001668 | ebi-a-GCST90001668 | TRUE | C | 196915125 | 1.94E-09 | reported | 3440 | 0.1763 | rs573537686 |  |  |  |  |  |  |  |  |  |  |  |  |  |  |  |
| -1.069 | 1 | igd | 0.0052 | G | \|\| id:ebi-a-GCST90001691 | ebi-a-GCST90001691 | TRUE | C | 196915125 | 2.89E-09 | reported | 3440 | 0.1795 | rs573537686 |  |  |  |  |  |  |  |  |  |  |  |  |  |  |  |
| -1.168 | 1 | igd | 0.0052 | G | \|\| id:ebi-a-GCST90001692 | ebi-a-GCST90001692 | TRUE | C | 196915125 | 6.58E-11 | reported | 3440 | 0.1782 | rs573537686 |  |  |  |  |  |  |  |  |  |  |  |  |  |  |  |
| -1.133 | 6 | igd | 0.0052 | T | \|\| id:ebi-a-GCST90001753 | ebi-a-GCST90001753 | TRUE | A | 32575340 | 5.17E-09 | reported | 3657 | 0.1935 | rs73410643 |  |  |  |  |  |  |  |  |  |  |  |  |  |  |  |
| 1.302 | 1 | igd | 0.0052 | C | \|\| id:ebi-a-GCST90002098 | ebi-a-GCST90002098 | TRUE | T | 199594111 | 4.72E-14 | reported | 2910 | 0.1718 | rs147198392 |  |  |  |  |  |  |  |  |  |  |  |  |  |  |  |
| 1.227 | 1 | igd | 0.0051 | C | \|\| id:ebi-a-GCST90001483 | ebi-a-GCST90001483 | TRUE | T | 199594111 | 2.46E-13 | reported | 3405 | 0.1669 | rs147198392 |  |  |  |  |  |  |  |  |  |  |  |  |  |  |  |
| 1.676 | 1 | igd | 0.0051 | G | \|\| id:ebi-a-GCST90001483 | ebi-a-GCST90001483 | TRUE | A | 197141261 | 2.24E-19 | reported | 3405 | 0.1851 | rs45512792 |  |  |  |  |  |  |  |  |  |  |  |  |  |  |  |
| 1.16 | 1 | igd | 0.0051 | G | \|\| id:ebi-a-GCST90001484 | ebi-a-GCST90001484 | TRUE | A | 197141261 | 5.18E-10 | reported | 3437 | 0.1861 | rs45512792 |  |  |  |  |  |  |  |  |  |  |  |  |  |  |  |
| 1.782 | 1 | igd | 0.0051 | G | \|\| id:ebi-a-GCST90001485 | ebi-a-GCST90001485 | TRUE | A | 197141261 | 8.02E-22 | reported | 3437 | 0.1844 | rs45512792 |  |  |  |  |  |  |  |  |  |  |  |  |  |  |  |
| 1.376 | 1 | igd | 0.0051 | C | \|\| id:ebi-a-GCST90001485 | ebi-a-GCST90001485 | TRUE | T | 199594111 | 1.57E-16 | reported | 3437 | 0.1659 | rs147198392 |  |  |  |  |  |  |  |  |  |  |  |  |  |  |  |
| 1.01 | 1 | igd | 0.0051 | C | \|\| id:ebi-a-GCST90001507 | ebi-a-GCST90001507 | TRUE | T | 199594111 | 7.73E-09 | reported | 3405 | 0.1744 | rs147198392 |  |  |  |  |  |  |  |  |  |  |  |  |  |  |  |
| 1.573 | 1 | igd | 0.0051 | G | \|\| id:ebi-a-GCST90001507 | ebi-a-GCST90001507 | TRUE | A | 197141261 | 6.75E-16 | reported | 3405 | 0.1938 | rs45512792 |  |  |  |  |  |  |  |  |  |  |  |  |  |  |  |
| 1.681 | 1 | igd | 0.0051 | G | \|\| id:ebi-a-GCST90001508 | ebi-a-GCST90001508 | TRUE | A | 197141261 | 3.79E-18 | reported | 3437 | 0.1925 | rs45512792 |  |  |  |  |  |  |  |  |  |  |  |  |  |  |  |
| 1.122 | 1 | igd | 0.0051 | C | \|\| id:ebi-a-GCST90001508 | ebi-a-GCST90001508 | TRUE | T | 199594111 | 1.01E-10 | reported | 3437 | 0.1731 | rs147198392 |  |  |  |  |  |  |  |  |  |  |  |  |  |  |  |
| 1.586 | 1 | igd | 0.0051 | G | \|\| id:ebi-a-GCST90001509 | ebi-a-GCST90001509 | TRUE | A | 197141261 | 2.53E-16 | reported | 3437 | 0.1926 | rs45512792 |  |  |  |  |  |  |  |  |  |  |  |  |  |  |  |
| 1.059 | 1 | igd | 0.0051 | C | \|\| id:ebi-a-GCST90001509 | ebi-a-GCST90001509 | TRUE | T | 199594111 | 1.04E-09 | reported | 3437 | 0.1731 | rs147198392 |  |  |  |  |  |  |  |  |  |  |  |  |  |  |  |
| -1.022 | 1 | igd | 0.0051 | C | \|\| id:ebi-a-GCST90001511 | ebi-a-GCST90001511 | TRUE | T | 199594111 | 3.46E-09 | reported | 3437 | 0.1726 | rs147198392 |  |  |  |  |  |  |  |  |  |  |  |  |  |  |  |
| -1.208 | 1 | igd | 0.0051 | G | \|\| id:ebi-a-GCST90001511 | ebi-a-GCST90001511 | TRUE | A | 197141261 | 4.23E-10 | reported | 3437 | 0.1928 | rs45512792 |  |  |  |  |  |  |  |  |  |  |  |  |  |  |  |
| -1.131 | 1 | igd | 0.0051 | G | \|\| id:ebi-a-GCST90001512 | ebi-a-GCST90001512 | TRUE | A | 197141261 | 4.97E-09 | reported | 3437 | 0.1929 | rs45512792 |  |  |  |  |  |  |  |  |  |  |  |  |  |  |  |
| -0.8827 | 1 | igd | 0.0051 | C | \|\| id:ebi-a-GCST90001535 | ebi-a-GCST90001535 | TRUE | T | 199594111 | 2.95E-08 | reported | 3427 | 0.1588 | rs147198392 |  |  |  |  |  |  |  |  |  |  |  |  |  |  |  |
| -1.076 | 1 | igd | 0.0051 | G | \|\| id:ebi-a-GCST90001535 | ebi-a-GCST90001535 | TRUE | A | 197141261 | 1.45E-09 | reported | 3427 | 0.1773 | rs45512792 |  |  |  |  |  |  |  |  |  |  |  |  |  |  |  |
| -1.068 | 1 | igd | 0.0051 | G | \|\| id:ebi-a-GCST90001536 | ebi-a-GCST90001536 | TRUE | A | 197141261 | 3.05E-09 | reported | 3427 | 0.1796 | rs45512792 |  |  |  |  |  |  |  |  |  |  |  |  |  |  |  |
| 1.148 | 1 | igd | 0.0051 | G | \|\| id:ebi-a-GCST90001561 | ebi-a-GCST90001561 | TRUE | A | 197141261 | 2.10E-12 | reported | 3427 | 0.1627 | rs45512792 |  |  |  |  |  |  |  |  |  |  |  |  |  |  |  |
| 1.017 | 1 | igd | 0.0051 | C | \|\| id:ebi-a-GCST90001561 | ebi-a-GCST90001561 | TRUE | T | 199594111 | 3.24E-12 | reported | 3427 | 0.1454 | rs147198392 |  |  |  |  |  |  |  |  |  |  |  |  |  |  |  |
| 1.261 | 1 | igd | 0.0051 | C | \|\| id:ebi-a-GCST90001664 | ebi-a-GCST90001664 | TRUE | T | 199594111 | 1.49E-15 | reported | 3440 | 0.1573 | rs147198392 |  |  |  |  |  |  |  |  |  |  |  |  |  |  |  |
| 1.406 | 1 | igd | 0.0051 | G | \|\| id:ebi-a-GCST90001664 | ebi-a-GCST90001664 | TRUE | A | 197141261 | 1.85E-15 | reported | 3440 | 0.1761 | rs45512792 |  |  |  |  |  |  |  |  |  |  |  |  |  |  |  |
| 1.473 | 1 | igd | 0.0051 | G | \|\| id:ebi-a-GCST90001665 | ebi-a-GCST90001665 | TRUE | A | 197141261 | 5.69E-15 | reported | 3440 | 0.1878 | rs45512792 |  |  |  |  |  |  |  |  |  |  |  |  |  |  |  |
| 1.018 | 1 | igd | 0.0051 | C | \|\| id:ebi-a-GCST90001665 | ebi-a-GCST90001665 | TRUE | T | 199594111 | 1.64E-09 | reported | 3440 | 0.1683 | rs147198392 |  |  |  |  |  |  |  |  |  |  |  |  |  |  |  |
| 1.278 | 1 | igd | 0.0051 | G | \|\| id:ebi-a-GCST90001666 | ebi-a-GCST90001666 | TRUE | A | 197141261 | 3.68E-14 | reported | 3408 | 0.1681 | rs45512792 |  |  |  |  |  |  |  |  |  |  |  |  |  |  |  |
| 1.09 | 1 | igd | 0.0051 | C | \|\| id:ebi-a-GCST90001666 | ebi-a-GCST90001666 | TRUE | T | 199594111 | 4.76E-13 | reported | 3408 | 0.1502 | rs147198392 |  |  |  |  |  |  |  |  |  |  |  |  |  |  |  |
| -1.309 | 1 | igd | 0.0051 | G | \|\| id:ebi-a-GCST90001667 | ebi-a-GCST90001667 | TRUE | A | 197141261 | 3.04E-13 | reported | 3440 | 0.1788 | rs45512792 |  |  |  |  |  |  |  |  |  |  |  |  |  |  |  |
| -1.014 | 1 | igd | 0.0051 | C | \|\| id:ebi-a-GCST90001668 | ebi-a-GCST90001668 | TRUE | T | 199594111 | 1.76E-09 | reported | 3440 | 0.168 | rs147198392 |  |  |  |  |  |  |  |  |  |  |  |  |  |  |  |
| -1.223 | 1 | igd | 0.0051 | G | \|\| id:ebi-a-GCST90001668 | ebi-a-GCST90001668 | TRUE | A | 197141261 | 8.75E-11 | reported | 3440 | 0.188 | rs45512792 |  |  |  |  |  |  |  |  |  |  |  |  |  |  |  |
| -1.2 | 1 | igd | 0.0051 | G | \|\| id:ebi-a-GCST90001669 | ebi-a-GCST90001669 | TRUE | A | 197141261 | 2.51E-12 | reported | 3408 | 0.1707 | rs45512792 |  |  |  |  |  |  |  |  |  |  |  |  |  |  |  |
| -1.238 | 1 | igd | 0.0051 | G | \|\| id:ebi-a-GCST90001691 | ebi-a-GCST90001691 | TRUE | A | 197141261 | 1.13E-10 | reported | 3440 | 0.1914 | rs45512792 |  |  |  |  |  |  |  |  |  |  |  |  |  |  |  |
| -1.105 | 1 | igd | 0.0051 | C | \|\| id:ebi-a-GCST90001691 | ebi-a-GCST90001691 | TRUE | T | 199594111 | 1.20E-10 | reported | 3440 | 0.1711 | rs147198392 |  |  |  |  |  |  |  |  |  |  |  |  |  |  |  |
| -0.9743 | 1 | igd | 0.0051 | C | \|\| id:ebi-a-GCST90001692 | ebi-a-GCST90001692 | TRUE | T | 199594111 | 1.12E-08 | reported | 3440 | 0.1701 | rs147198392 |  |  |  |  |  |  |  |  |  |  |  |  |  |  |  |
| -1.338 | 1 | igd | 0.0051 | G | \|\| id:ebi-a-GCST90001692 | ebi-a-GCST90001692 | TRUE | A | 197141261 | 2.33E-12 | reported | 3440 | 0.19 | rs45512792 |  |  |  |  |  |  |  |  |  |  |  |  |  |  |  |
| -1.058 | 1 | igd | 0.0051 | G | \|\| id:ebi-a-GCST90001693 | ebi-a-GCST90001693 | TRUE | A | 197141261 | 4.57E-08 | reported | 3408 | 0.193 | rs45512792 |  |  |  |  |  |  |  |  |  |  |  |  |  |  |  |
| -1.05 | 1 | igd | 0.0051 | C | \|\| id:ebi-a-GCST90001693 | ebi-a-GCST90001693 | TRUE | T | 199594111 | 1.18E-09 | reported | 3408 | 0.1722 | rs147198392 |  |  |  |  |  |  |  |  |  |  |  |  |  |  |  |
| 165.1 | 6 | igd | 0.0051 | A | \|\| id:ebi-a-GCST90001698 | ebi-a-GCST90001698 | TRUE | T | 84561060 | 5.04E-12 | reported | 3408 | 23.83 | rs7748677 |  |  |  |  |  |  |  |  |  |  |  |  |  |  |  |
| 145.9 | 12 | igd | 0.0051 | C | \|\| id:ebi-a-GCST90001698 | ebi-a-GCST90001698 | TRUE | T | 79481302 | 4.37E-09 | reported | 3408 | 24.8 | rs75260481 |  |  |  |  |  |  |  |  |  |  |  |  |  |  |  |
| 152.3 | 12 | igd | 0.0051 | T | \|\| id:ebi-a-GCST90001698 | ebi-a-GCST90001698 | TRUE | C | 79593516 | 1.45E-09 | reported | 3408 | 25.11 | rs77217392 |  |  |  |  |  |  |  |  |  |  |  |  |  |  |  |
| 148.5 | 13 | igd | 0.0051 | C | \|\| id:ebi-a-GCST90001698 | ebi-a-GCST90001698 | TRUE | T | 97407406 | 1.20E-09 | reported | 3408 | 24.36 | rs7319629 |  |  |  |  |  |  |  |  |  |  |  |  |  |  |  |
| 1.462 | 1 | igd | 0.0051 | G | \|\| id:ebi-a-GCST90002102 | ebi-a-GCST90002102 | TRUE | A | 197141261 | 1.30E-13 | reported | 2919 | 0.1965 | rs45512792 |  |  |  |  |  |  |  |  |  |  |  |  |  |  |  |
| 143.9 | 2 | igd | 0.005 | C | \|\| id:ebi-a-GCST90001698 | ebi-a-GCST90001698 | TRUE | T | 41343706 | 3.78E-08 | reported | 3408 | 26.11 | rs146217030 |  |  |  |  |  |  |  |  |  |  |  |  |  |  |  |
| 151.3 | 7 | igd | 0.005 | T | \|\| id:ebi-a-GCST90001698 | ebi-a-GCST90001698 | TRUE | C | 57089053 | 2.82E-09 | reported | 3408 | 25.4 | rs189858213 |  |  |  |  |  |  |  |  |  |  |  |  |  |  |  |
| 195.2 | 13 | igd | 0.005 | G | \|\| id:ebi-a-GCST90001698 | ebi-a-GCST90001698 | TRUE | T | 20546409 | 6.09E-16 | reported | 3408 | 24.02 | rs142978530 |  |  |  |  |  |  |  |  |  |  |  |  |  |  |  |
| 145.7 | 14 | igd | 0.005 | A | \|\| id:ebi-a-GCST90001698 | ebi-a-GCST90001698 | TRUE | G | 33154139 | 1.38E-09 | reported | 3408 | 23.98 | rs117221169 |  |  |  |  |  |  |  |  |  |  |  |  |  |  |  |
| 202.5 | 19 | igd | 0.005 | G | \|\| id:ebi-a-GCST90001698 | ebi-a-GCST90001698 | TRUE | C | 34111992 | 2.68E-16 | reported | 3408 | 24.61 | rs145410896 |  |  |  |  |  |  |  |  |  |  |  |  |  |  |  |
| 202.1 | 21 | igd | 0.005 | T | \|\| id:ebi-a-GCST90001698 | ebi-a-GCST90001698 | TRUE | C | 19551017 | 2.36E-15 | reported | 3408 | 25.4 | rs9981720 |  |  |  |  |  |  |  |  |  |  |  |  |  |  |  |
| -0.9923 | 1 | igd | 0.0049 | A | \|\| id:ebi-a-GCST90001667 | ebi-a-GCST90001667 | TRUE | G | 189981116 | 1.45E-08 | reported | 3440 | 0.1747 | rs116759767 |  |  |  |  |  |  |  |  |  |  |  |  |  |  |  |
| 111 | 1 | igd | 0.0048 | T | \|\| id:ebi-a-GCST90001695 | ebi-a-GCST90001695 | TRUE | C | 161526187 | 1.89E-08 | reported | 3408 | 19.7 | rs138010330 |  |  |  |  |  |  |  |  |  |  |  |  |  |  |  |
| 109.4 | 1 | igd | 0.0048 | A | \|\| id:ebi-a-GCST90001695 | ebi-a-GCST90001695 | TRUE | T | 91139978 | 2.80E-09 | reported | 3408 | 18.35 | rs78003778 |  |  |  |  |  |  |  |  |  |  |  |  |  |  |  |
| 147.5 | 4 | igd | 0.0048 | A | \|\| id:ebi-a-GCST90001698 | ebi-a-GCST90001698 | TRUE | G | 34247795 | 1.67E-08 | reported | 3408 | 26.08 | rs111371663 |  |  |  |  |  |  |  |  |  |  |  |  |  |  |  |
| 148.4 | 4 | igd | 0.0048 | G | \|\| id:ebi-a-GCST90001698 | ebi-a-GCST90001698 | TRUE | A | 34012400 | 1.53E-08 | reported | 3408 | 26.16 | rs112722102 |  |  |  |  |  |  |  |  |  |  |  |  |  |  |  |
| 153.6 | 13 | igd | 0.0048 | A | \|\| id:ebi-a-GCST90001698 | ebi-a-GCST90001698 | TRUE | G | 98313824 | 7.89E-10 | reported | 3408 | 24.92 | rs7981825 |  |  |  |  |  |  |  |  |  |  |  |  |  |  |  |
| -1.133 | 16 | igd | 0.0048 | G | \|\| id:ebi-a-GCST90001723 | ebi-a-GCST90001723 | TRUE | A | 33381931 | 1.29E-08 | reported | 3656 | 0.1987 | rs556576369 |  |  |  |  |  |  |  |  |  |  |  |  |  |  |  |
| -1.109 | 16 | igd | 0.0048 | G | \|\| id:ebi-a-GCST90001724 | ebi-a-GCST90001724 | TRUE | A | 33381931 | 2.94E-08 | reported | 3656 | 0.1995 | rs556576369 |  |  |  |  |  |  |  |  |  |  |  |  |  |  |  |
| -1.239 | 16 | igd | 0.0048 | G | \|\| id:ebi-a-GCST90001729 | ebi-a-GCST90001729 | TRUE | A | 33381931 | 7.58E-10 | reported | 3657 | 0.2008 | rs556576369 |  |  |  |  |  |  |  |  |  |  |  |  |  |  |  |
| -1.091 | 16 | igd | 0.0048 | G | \|\| id:ebi-a-GCST90001730 | ebi-a-GCST90001730 | TRUE | A | 33381931 | 4.31E-08 | reported | 3657 | 0.1988 | rs556576369 |  |  |  |  |  |  |  |  |  |  |  |  |  |  |  |
| -1.15 | 16 | igd | 0.0048 | G | \|\| id:ebi-a-GCST90001735 | ebi-a-GCST90001735 | TRUE | A | 33381931 | 6.85E-09 | reported | 3657 | 0.1979 | rs556576369 |  |  |  |  |  |  |  |  |  |  |  |  |  |  |  |
| -1.122 | 16 | igd | 0.0048 | G | \|\| id:ebi-a-GCST90001742 | ebi-a-GCST90001742 | TRUE | A | 33381931 | 2.42E-08 | reported | 3657 | 0.2007 | rs556576369 |  |  |  |  |  |  |  |  |  |  |  |  |  |  |  |
| 1.21 | 1 | igd | 0.0048 | C | \|\| id:ebi-a-GCST90002102 | ebi-a-GCST90002102 | TRUE | T | 199594111 | 1.01E-11 | reported | 2919 | 0.1771 | rs147198392 |  |  |  |  |  |  |  |  |  |  |  |  |  |  |  |
| 1.062 | 1 | igd | 0.0047 | C | \|\| id:ebi-a-GCST90001484 | ebi-a-GCST90001484 | TRUE | T | 198830942 | 6.08E-10 | reported | 3437 | 0.1712 | rs113116201 |  |  |  |  |  |  |  |  |  |  |  |  |  |  |  |
| -1.057 | 1 | igd | 0.0047 | C | \|\| id:ebi-a-GCST90001510 | ebi-a-GCST90001510 | TRUE | T | 198830942 | 4.92E-09 | reported | 3405 | 0.1802 | rs113116201 |  |  |  |  |  |  |  |  |  |  |  |  |  |  |  |
| -1.133 | 1 | igd | 0.0047 | C | \|\| id:ebi-a-GCST90001534 | ebi-a-GCST90001534 | TRUE | T | 198830942 | 3.72E-10 | reported | 3395 | 0.1804 | rs113116201 |  |  |  |  |  |  |  |  |  |  |  |  |  |  |  |
| -1.073 | 1 | igd | 0.0047 | C | \|\| id:ebi-a-GCST90001536 | ebi-a-GCST90001536 | TRUE | T | 198830942 | 8.34E-11 | reported | 3427 | 0.1648 | rs113116201 |  |  |  |  |  |  |  |  |  |  |  |  |  |  |  |
| 1.668 | 1 | igd | 0.0047 | C | \|\| id:ebi-a-GCST90001664 | ebi-a-GCST90001664 | TRUE | T | 198830942 | 1.21E-24 | reported | 3440 | 0.1615 | rs113116201 |  |  |  |  |  |  |  |  |  |  |  |  |  |  |  |
| 1.611 | 1 | igd | 0.0047 | C | \|\| id:ebi-a-GCST90001665 | ebi-a-GCST90001665 | TRUE | T | 198830942 | 2.69E-20 | reported | 3440 | 0.1734 | rs113116201 |  |  |  |  |  |  |  |  |  |  |  |  |  |  |  |
| 1.429 | 1 | igd | 0.0047 | C | \|\| id:ebi-a-GCST90001666 | ebi-a-GCST90001666 | TRUE | T | 198830942 | 5.05E-20 | reported | 3408 | 0.155 | rs113116201 |  |  |  |  |  |  |  |  |  |  |  |  |  |  |  |
| -1.296 | 1 | igd | 0.0047 | C | \|\| id:ebi-a-GCST90001669 | ebi-a-GCST90001669 | TRUE | T | 198830942 | 2.10E-16 | reported | 3408 | 0.157 | rs113116201 |  |  |  |  |  |  |  |  |  |  |  |  |  |  |  |
| 5.388 | 5 | igd | 0.0047 | A | \|\| id:ebi-a-GCST90001697 | ebi-a-GCST90001697 | TRUE | C | 72679056 | 1.95E-09 | reported | 3425 | 0.8953 | rs12656604 |  |  |  |  |  |  |  |  |  |  |  |  |  |  |  |
| 164 | 3 | igd | 0.0047 | T | \|\| id:ebi-a-GCST90001698 | ebi-a-GCST90001698 | TRUE | C | 166969119 | 8.21E-11 | reported | 3408 | 25.16 | rs139329941 |  |  |  |  |  |  |  |  |  |  |  |  |  |  |  |
| 165.4 | 6 | igd | 0.0047 | T | \|\| id:ebi-a-GCST90001698 | ebi-a-GCST90001698 | TRUE | G | 119126703 | 1.01E-08 | reported | 3408 | 28.8 | rs6905813 |  |  |  |  |  |  |  |  |  |  |  |  |  |  |  |
| 145.9 | 6 | igd | 0.0047 | A | \|\| id:ebi-a-GCST90001698 | ebi-a-GCST90001698 | TRUE | G | 67064897 | 2.35E-08 | reported | 3408 | 26.06 | rs138707019 |  |  |  |  |  |  |  |  |  |  |  |  |  |  |  |
| 162.2 | 8 | igd | 0.0047 | T | \|\| id:ebi-a-GCST90001698 | ebi-a-GCST90001698 | TRUE | C | 12621142 | 3.10E-11 | reported | 3408 | 24.35 | rs62486441 |  |  |  |  |  |  |  |  |  |  |  |  |  |  |  |
| 206 | 10 | igd | 0.0047 | T | \|\| id:ebi-a-GCST90001698 | ebi-a-GCST90001698 | TRUE | A | 17605945 | 1.92E-16 | reported | 3408 | 24.92 | rs72780729 |  |  |  |  |  |  |  |  |  |  |  |  |  |  |  |
| 152.1 | 15 | igd | 0.0047 | A | \|\| id:ebi-a-GCST90001698 | ebi-a-GCST90001698 | TRUE | G | 38397413 | 3.13E-09 | reported | 3408 | 25.6 | rs142262063 |  |  |  |  |  |  |  |  |  |  |  |  |  |  |  |
| 161.4 | 19 | igd | 0.0047 | T | \|\| id:ebi-a-GCST90001698 | ebi-a-GCST90001698 | TRUE | C | 364005 | 6.21E-11 | reported | 3408 | 24.6 | rs116891054 |  |  |  |  |  |  |  |  |  |  |  |  |  |  |  |
| -1.046 | 22 | igd | 0.0046 | C | \|\| id:ebi-a-GCST90001704 | ebi-a-GCST90001704 | TRUE | A | 44689355 | 2.78E-08 | reported | 3657 | 0.1879 | rs13056800 |  |  |  |  |  |  |  |  |  |  |  |  |  |  |  |
| 1.066 | 1 | igd | 0.0046 | C | \|\| id:ebi-a-GCST90002098 | ebi-a-GCST90002098 | TRUE | T | 194010208 | 2.71E-08 | reported | 2910 | 0.1913 | rs116794175 |  |  |  |  |  |  |  |  |  |  |  |  |  |  |  |
| 1.635 | 1 | igd | 0.0046 | T | \|\| id:ebi-a-GCST90002098 | ebi-a-GCST90002098 | TRUE | C | 199363317 | 2.92E-16 | reported | 2910 | 0.1988 | rs149290361 |  |  |  |  |  |  |  |  |  |  |  |  |  |  |  |
| 1.693 | 1 | igd | 0.0045 | T | \|\| id:ebi-a-GCST90001664 | ebi-a-GCST90001664 | TRUE | C | 199363317 | 1.23E-20 | reported | 3440 | 0.1806 | rs149290361 |  |  |  |  |  |  |  |  |  |  |  |  |  |  |  |
| 1.538 | 1 | igd | 0.0045 | T | \|\| id:ebi-a-GCST90001665 | ebi-a-GCST90001665 | TRUE | C | 199363317 | 2.33E-15 | reported | 3440 | 0.1932 | rs149290361 |  |  |  |  |  |  |  |  |  |  |  |  |  |  |  |
| 1.478 | 1 | igd | 0.0045 | T | \|\| id:ebi-a-GCST90001666 | ebi-a-GCST90001666 | TRUE | C | 199363317 | 1.53E-17 | reported | 3408 | 0.1725 | rs149290361 |  |  |  |  |  |  |  |  |  |  |  |  |  |  |  |
| 156.1 | 1 | igd | 0.0045 | G | \|\| id:ebi-a-GCST90001698 | ebi-a-GCST90001698 | TRUE | A | 95606937 | 1.60E-08 | reported | 3408 | 27.56 | rs76993077 |  |  |  |  |  |  |  |  |  |  |  |  |  |  |  |
| 160.4 | 4 | igd | 0.0045 | C | \|\| id:ebi-a-GCST90001698 | ebi-a-GCST90001698 | TRUE | G | 33761520 | 1.40E-08 | reported | 3408 | 28.2 | rs113733627 |  |  |  |  |  |  |  |  |  |  |  |  |  |  |  |
| 153.9 | 7 | igd | 0.0045 | A | \|\| id:ebi-a-GCST90001698 | ebi-a-GCST90001698 | TRUE | C | 157693151 | 3.04E-09 | reported | 3408 | 25.89 | rs143992242 |  |  |  |  |  |  |  |  |  |  |  |  |  |  |  |
| 168.8 | 8 | igd | 0.0045 | C | \|\| id:ebi-a-GCST90001698 | ebi-a-GCST90001698 | TRUE | G | 23682802 | 3.43E-11 | reported | 3408 | 25.4 | rs142111513 |  |  |  |  |  |  |  |  |  |  |  |  |  |  |  |
| 178.1 | 9 | igd | 0.0045 | T | \|\| id:ebi-a-GCST90001698 | ebi-a-GCST90001698 | TRUE | A | 69822644 | 8.80E-09 | reported | 3408 | 30.88 | rs62560287 |  |  |  |  |  |  |  |  |  |  |  |  |  |  |  |
| 1.037 | 20 | igd | 0.0045 | A | \|\| id:ebi-a-GCST90001980 | ebi-a-GCST90001980 | TRUE | G | 44730041 | 7.95E-09 | reported | 3629 | 0.1793 | rs182282247 |  |  |  |  |  |  |  |  |  |  |  |  |  |  |  |
| 1.058 | 20 | igd | 0.0045 | A | \|\| id:ebi-a-GCST90001985 | ebi-a-GCST90001985 | TRUE | G | 44730041 | 4.11E-09 | reported | 3629 | 0.1796 | rs182282247 |  |  |  |  |  |  |  |  |  |  |  |  |  |  |  |
| -1.275 | 16 | igd | 0.0045 | G | \|\| id:ebi-a-GCST90002028 | ebi-a-GCST90002028 | TRUE | A | 33381931 | 1.83E-08 | reported | 3112 | 0.226 | rs556576369 |  |  |  |  |  |  |  |  |  |  |  |  |  |  |  |
| 1.394 | 1 | igd | 0.0045 | A | \|\| id:ebi-a-GCST90002098 | ebi-a-GCST90002098 | TRUE | G | 199999121 | 3.79E-13 | reported | 2910 | 0.191 | rs11579717 |  |  |  |  |  |  |  |  |  |  |  |  |  |  |  |
| 1.537 | 1 | igd | 0.0044 | C | \|\| id:ebi-a-GCST90001483 | ebi-a-GCST90001483 | TRUE | A | 199278900 | 2.21E-16 | reported | 3405 | 0.1862 | rs140372175 |  |  |  |  |  |  |  |  |  |  |  |  |  |  |  |
| 1.608 | 1 | igd | 0.0044 | A | \|\| id:ebi-a-GCST90001483 | ebi-a-GCST90001483 | TRUE | G | 199999121 | 4.41E-18 | reported | 3405 | 0.1845 | rs11579717 |  |  |  |  |  |  |  |  |  |  |  |  |  |  |  |
| 1.128 | 1 | igd | 0.0044 | A | \|\| id:ebi-a-GCST90001484 | ebi-a-GCST90001484 | TRUE | G | 199999121 | 1.23E-09 | reported | 3437 | 0.1851 | rs11579717 |  |  |  |  |  |  |  |  |  |  |  |  |  |  |  |
| 1.116 | 1 | igd | 0.0044 | A | \|\| id:ebi-a-GCST90001485 | ebi-a-GCST90001485 | TRUE | G | 190444745 | 1.47E-08 | reported | 3437 | 0.1965 | rs142274302 |  |  |  |  |  |  |  |  |  |  |  |  |  |  |  |
| 1.118 | 1 | igd | 0.0044 | A | \|\| id:ebi-a-GCST90001485 | ebi-a-GCST90001485 | TRUE | G | 190341116 | 1.65E-08 | reported | 3437 | 0.1975 | rs114454943 |  |  |  |  |  |  |  |  |  |  |  |  |  |  |  |
| 1.658 | 1 | igd | 0.0044 | C | \|\| id:ebi-a-GCST90001485 | ebi-a-GCST90001485 | TRUE | A | 199278900 | 5.24E-19 | reported | 3437 | 0.1851 | rs140372175 |  |  |  |  |  |  |  |  |  |  |  |  |  |  |  |
| 1.718 | 1 | igd | 0.0044 | A | \|\| id:ebi-a-GCST90001485 | ebi-a-GCST90001485 | TRUE | G | 199999121 | 1.35E-20 | reported | 3437 | 0.1835 | rs11579717 |  |  |  |  |  |  |  |  |  |  |  |  |  |  |  |
| 1.342 | 1 | igd | 0.0044 | A | \|\| id:ebi-a-GCST90001507 | ebi-a-GCST90001507 | TRUE | G | 199999121 | 4.23E-12 | reported | 3405 | 0.1929 | rs11579717 |  |  |  |  |  |  |  |  |  |  |  |  |  |  |  |
| 1.34 | 1 | igd | 0.0044 | C | \|\| id:ebi-a-GCST90001507 | ebi-a-GCST90001507 | TRUE | A | 199278900 | 6.42E-12 | reported | 3405 | 0.1944 | rs140372175 |  |  |  |  |  |  |  |  |  |  |  |  |  |  |  |
| 1.418 | 1 | igd | 0.0044 | A | \|\| id:ebi-a-GCST90001508 | ebi-a-GCST90001508 | TRUE | G | 199999121 | 1.55E-13 | reported | 3437 | 0.1913 | rs11579717 |  |  |  |  |  |  |  |  |  |  |  |  |  |  |  |
| 1.441 | 1 | igd | 0.0044 | C | \|\| id:ebi-a-GCST90001508 | ebi-a-GCST90001508 | TRUE | A | 199278900 | 9.39E-14 | reported | 3437 | 0.1926 | rs140372175 |  |  |  |  |  |  |  |  |  |  |  |  |  |  |  |
| 1.373 | 1 | igd | 0.0044 | C | \|\| id:ebi-a-GCST90001509 | ebi-a-GCST90001509 | TRUE | A | 199278900 | 1.24E-12 | reported | 3437 | 0.1926 | rs140372175 |  |  |  |  |  |  |  |  |  |  |  |  |  |  |  |
| 1.391 | 1 | igd | 0.0044 | A | \|\| id:ebi-a-GCST90001509 | ebi-a-GCST90001509 | TRUE | G | 199999121 | 4.29E-13 | reported | 3437 | 0.1912 | rs11579717 |  |  |  |  |  |  |  |  |  |  |  |  |  |  |  |
| -1.069 | 1 | igd | 0.0044 | C | \|\| id:ebi-a-GCST90001510 | ebi-a-GCST90001510 | TRUE | A | 199278900 | 4.71E-08 | reported | 3405 | 0.1952 | rs140372175 |  |  |  |  |  |  |  |  |  |  |  |  |  |  |  |
| -1.2 | 1 | igd | 0.0044 | C | \|\| id:ebi-a-GCST90001511 | ebi-a-GCST90001511 | TRUE | A | 199278900 | 4.93E-10 | reported | 3437 | 0.1923 | rs140372175 |  |  |  |  |  |  |  |  |  |  |  |  |  |  |  |
| -1.241 | 1 | igd | 0.0044 | A | \|\| id:ebi-a-GCST90001511 | ebi-a-GCST90001511 | TRUE | G | 199999121 | 9.23E-11 | reported | 3437 | 0.1909 | rs11579717 |  |  |  |  |  |  |  |  |  |  |  |  |  |  |  |
| -1.064 | 1 | igd | 0.0044 | C | \|\| id:ebi-a-GCST90001512 | ebi-a-GCST90001512 | TRUE | A | 199278900 | 3.46E-08 | reported | 3437 | 0.1925 | rs140372175 |  |  |  |  |  |  |  |  |  |  |  |  |  |  |  |
| -1.078 | 1 | igd | 0.0044 | A | \|\| id:ebi-a-GCST90001512 | ebi-a-GCST90001512 | TRUE | G | 199999121 | 1.83E-08 | reported | 3437 | 0.1911 | rs11579717 |  |  |  |  |  |  |  |  |  |  |  |  |  |  |  |
| -1.081 | 1 | igd | 0.0044 | A | \|\| id:ebi-a-GCST90001535 | ebi-a-GCST90001535 | TRUE | G | 199999121 | 8.87E-10 | reported | 3427 | 0.1759 | rs11579717 |  |  |  |  |  |  |  |  |  |  |  |  |  |  |  |
| -1.02 | 1 | igd | 0.0044 | C | \|\| id:ebi-a-GCST90001535 | ebi-a-GCST90001535 | TRUE | A | 199278900 | 9.65E-09 | reported | 3427 | 0.1773 | rs140372175 |  |  |  |  |  |  |  |  |  |  |  |  |  |  |  |
| 1.279 | 1 | igd | 0.0044 | C | \|\| id:ebi-a-GCST90001561 | ebi-a-GCST90001561 | TRUE | A | 199278900 | 3.48E-15 | reported | 3427 | 0.1617 | rs140372175 |  |  |  |  |  |  |  |  |  |  |  |  |  |  |  |
| 1.168 | 1 | igd | 0.0044 | A | \|\| id:ebi-a-GCST90001561 | ebi-a-GCST90001561 | TRUE | G | 199999121 | 4.59E-13 | reported | 3427 | 0.1608 | rs11579717 |  |  |  |  |  |  |  |  |  |  |  |  |  |  |  |
| 1.303 | 1 | igd | 0.0044 | A | \|\| id:ebi-a-GCST90001664 | ebi-a-GCST90001664 | TRUE | G | 199999121 | 1.02E-13 | reported | 3440 | 0.1744 | rs11579717 |  |  |  |  |  |  |  |  |  |  |  |  |  |  |  |
| 1.307 | 1 | igd | 0.0044 | A | \|\| id:ebi-a-GCST90001665 | ebi-a-GCST90001665 | TRUE | G | 199999121 | 2.37E-12 | reported | 3440 | 0.1858 | rs11579717 |  |  |  |  |  |  |  |  |  |  |  |  |  |  |  |
| 1.176 | 1 | igd | 0.0044 | A | \|\| id:ebi-a-GCST90001666 | ebi-a-GCST90001666 | TRUE | G | 199999121 | 1.77E-12 | reported | 3408 | 0.1662 | rs11579717 |  |  |  |  |  |  |  |  |  |  |  |  |  |  |  |
| -1.201 | 1 | igd | 0.0044 | A | \|\| id:ebi-a-GCST90001667 | ebi-a-GCST90001667 | TRUE | G | 199999121 | 1.42E-11 | reported | 3440 | 0.1771 | rs11579717 |  |  |  |  |  |  |  |  |  |  |  |  |  |  |  |
| -1.038 | 1 | igd | 0.0044 | A | \|\| id:ebi-a-GCST90001667 | ebi-a-GCST90001667 | TRUE | G | 190341116 | 4.03E-08 | reported | 3440 | 0.1887 | rs114454943 |  |  |  |  |  |  |  |  |  |  |  |  |  |  |  |
| -1.032 | 1 | igd | 0.0044 | A | \|\| id:ebi-a-GCST90001667 | ebi-a-GCST90001667 | TRUE | G | 190444745 | 4.23E-08 | reported | 3440 | 0.1878 | rs142274302 |  |  |  |  |  |  |  |  |  |  |  |  |  |  |  |
| -1.192 | 1 | igd | 0.0044 | C | \|\| id:ebi-a-GCST90001667 | ebi-a-GCST90001667 | TRUE | A | 199278900 | 2.74E-11 | reported | 3440 | 0.1784 | rs140372175 |  |  |  |  |  |  |  |  |  |  |  |  |  |  |  |
| -1.217 | 1 | igd | 0.0044 | A | \|\| id:ebi-a-GCST90001668 | ebi-a-GCST90001668 | TRUE | G | 199999121 | 6.36E-11 | reported | 3440 | 0.1857 | rs11579717 |  |  |  |  |  |  |  |  |  |  |  |  |  |  |  |
| -1.33 | 1 | igd | 0.0044 | C | \|\| id:ebi-a-GCST90001668 | ebi-a-GCST90001668 | TRUE | A | 199278900 | 1.30E-12 | reported | 3440 | 0.1868 | rs140372175 |  |  |  |  |  |  |  |  |  |  |  |  |  |  |  |
| -1.125 | 1 | igd | 0.0044 | A | \|\| id:ebi-a-GCST90001669 | ebi-a-GCST90001669 | TRUE | G | 199999121 | 3.18E-11 | reported | 3408 | 0.169 | rs11579717 |  |  |  |  |  |  |  |  |  |  |  |  |  |  |  |
| -1.149 | 1 | igd | 0.0044 | C | \|\| id:ebi-a-GCST90001669 | ebi-a-GCST90001669 | TRUE | A | 199278900 | 1.70E-11 | reported | 3408 | 0.1702 | rs140372175 |  |  |  |  |  |  |  |  |  |  |  |  |  |  |  |
| -1.406 | 1 | igd | 0.0044 | A | \|\| id:ebi-a-GCST90001691 | ebi-a-GCST90001691 | TRUE | G | 199999121 | 1.39E-13 | reported | 3440 | 0.1893 | rs11579717 |  |  |  |  |  |  |  |  |  |  |  |  |  |  |  |
| -1.443 | 1 | igd | 0.0044 | C | \|\| id:ebi-a-GCST90001691 | ebi-a-GCST90001691 | TRUE | A | 199278900 | 4.83E-14 | reported | 3440 | 0.1906 | rs140372175 |  |  |  |  |  |  |  |  |  |  |  |  |  |  |  |
| -1.345 | 1 | igd | 0.0044 | C | \|\| id:ebi-a-GCST90001692 | ebi-a-GCST90001692 | TRUE | A | 199278900 | 1.42E-12 | reported | 3440 | 0.1892 | rs140372175 |  |  |  |  |  |  |  |  |  |  |  |  |  |  |  |
| -1.249 | 1 | igd | 0.0044 | A | \|\| id:ebi-a-GCST90001692 | ebi-a-GCST90001692 | TRUE | G | 199999121 | 3.55E-11 | reported | 3440 | 0.188 | rs11579717 |  |  |  |  |  |  |  |  |  |  |  |  |  |  |  |
| -1.253 | 1 | igd | 0.0044 | A | \|\| id:ebi-a-GCST90001693 | ebi-a-GCST90001693 | TRUE | G | 199999121 | 5.53E-11 | reported | 3408 | 0.1905 | rs11579717 |  |  |  |  |  |  |  |  |  |  |  |  |  |  |  |
| -1.332 | 1 | igd | 0.0044 | C | \|\| id:ebi-a-GCST90001693 | ebi-a-GCST90001693 | TRUE | A | 199278900 | 4.45E-12 | reported | 3408 | 0.1918 | rs140372175 |  |  |  |  |  |  |  |  |  |  |  |  |  |  |  |
| 149.3 | 9 | igd | 0.0044 | T | \|\| id:ebi-a-GCST90001698 | ebi-a-GCST90001698 | TRUE | C | 28759554 | 1.64E-08 | reported | 3408 | 26.38 | rs148380088 |  |  |  |  |  |  |  |  |  |  |  |  |  |  |  |
| 1.214 | 1 | igd | 0.0044 | C | \|\| id:ebi-a-GCST90001941 | ebi-a-GCST90001941 | TRUE | T | 198830942 | 8.43E-11 | reported | 3435 | 0.1863 | rs113116201 |  |  |  |  |  |  |  |  |  |  |  |  |  |  |  |
| 1.189 | 1 | igd | 0.0044 | C | \|\| id:ebi-a-GCST90001942 | ebi-a-GCST90001942 | TRUE | T | 198830942 | 1.82E-10 | reported | 3435 | 0.186 | rs113116201 |  |  |  |  |  |  |  |  |  |  |  |  |  |  |  |
| 1.068 | 1 | igd | 0.0044 | C | \|\| id:ebi-a-GCST90001943 | ebi-a-GCST90001943 | TRUE | T | 198830942 | 8.02E-09 | reported | 3435 | 0.1846 | rs113116201 |  |  |  |  |  |  |  |  |  |  |  |  |  |  |  |
| 1.189 | 1 | igd | 0.0043 | C | \|\| id:ebi-a-GCST90001483 | ebi-a-GCST90001483 | TRUE | T | 194010208 | 6.05E-10 | reported | 3405 | 0.1915 | rs116794175 |  |  |  |  |  |  |  |  |  |  |  |  |  |  |  |
| 1.018 | 1 | igd | 0.0043 | C | \|\| id:ebi-a-GCST90001666 | ebi-a-GCST90001666 | TRUE | T | 194010208 | 3.23E-09 | reported | 3408 | 0.1715 | rs116794175 |  |  |  |  |  |  |  |  |  |  |  |  |  |  |  |
| -1.044 | 1 | igd | 0.0043 | A | \|\| id:ebi-a-GCST90001669 | ebi-a-GCST90001669 | TRUE | G | 190444745 | 7.83E-09 | reported | 3408 | 0.1805 | rs142274302 |  |  |  |  |  |  |  |  |  |  |  |  |  |  |  |
| -1.051 | 1 | igd | 0.0043 | A | \|\| id:ebi-a-GCST90001669 | ebi-a-GCST90001669 | TRUE | G | 190341116 | 7.45E-09 | reported | 3408 | 0.1814 | rs114454943 |  |  |  |  |  |  |  |  |  |  |  |  |  |  |  |
| 124 | 1 | igd | 0.0043 | A | \|\| id:ebi-a-GCST90001695 | ebi-a-GCST90001695 | TRUE | G | 18070672 | 2.75E-09 | reported | 3408 | 20.79 | rs140434581 |  |  |  |  |  |  |  |  |  |  |  |  |  |  |  |
| 123.8 | 1 | igd | 0.0043 | C | \|\| id:ebi-a-GCST90001695 | ebi-a-GCST90001695 | TRUE | T | 21025910 | 3.21E-10 | reported | 3408 | 19.64 | rs111978771 |  |  |  |  |  |  |  |  |  |  |  |  |  |  |  |
| 168.6 | 4 | igd | 0.0043 | T | \|\| id:ebi-a-GCST90001698 | ebi-a-GCST90001698 | TRUE | C | 109566866 | 4.71E-10 | reported | 3408 | 26.99 | rs80331575 |  |  |  |  |  |  |  |  |  |  |  |  |  |  |  |
| 159.2 | 6 | igd | 0.0043 | C | \|\| id:ebi-a-GCST90001698 | ebi-a-GCST90001698 | TRUE | T | 66416572 | 2.05E-08 | reported | 3408 | 28.32 | rs116431337 |  |  |  |  |  |  |  |  |  |  |  |  |  |  |  |
| 212.3 | 11 | igd | 0.0043 | A | \|\| id:ebi-a-GCST90001698 | ebi-a-GCST90001698 | TRUE | T | 9178127 | 4.26E-15 | reported | 3408 | 26.93 | rs144909360 |  |  |  |  |  |  |  |  |  |  |  |  |  |  |  |
| 161.3 | 12 | igd | 0.0043 | T | \|\| id:ebi-a-GCST90001698 | ebi-a-GCST90001698 | TRUE | A | 109036912 | 1.23E-08 | reported | 3408 | 28.25 | rs139898536 |  |  |  |  |  |  |  |  |  |  |  |  |  |  |  |
| 171.4 | 13 | igd | 0.0043 | C | \|\| id:ebi-a-GCST90001698 | ebi-a-GCST90001698 | TRUE | T | 113746760 | 9.66E-09 | reported | 3408 | 29.81 | rs561977461 |  |  |  |  |  |  |  |  |  |  |  |  |  |  |  |
| 1.872 | 1 | igd | 0.0043 | T | \|\| id:ebi-a-GCST90002098 | ebi-a-GCST90002098 | TRUE | C | 196321168 | 2.62E-17 | reported | 2910 | 0.2198 | rs191753228 |  |  |  |  |  |  |  |  |  |  |  |  |  |  |  |
| 1.649 | 1 | igd | 0.0043 | T | \|\| id:ebi-a-GCST90002102 | ebi-a-GCST90002102 | TRUE | C | 199363317 | 2.26E-15 | reported | 2919 | 0.2069 | rs149290361 |  |  |  |  |  |  |  |  |  |  |  |  |  |  |  |
| 1.583 | 1 | igd | 0.0043 | T | \|\| id:ebi-a-GCST90002103 | ebi-a-GCST90002103 | TRUE | C | 196321168 | 8.93E-11 | reported | 2650 | 0.2431 | rs191753228 |  |  |  |  |  |  |  |  |  |  |  |  |  |  |  |
| 1.311 | 1 | igd | 0.0042 | C | \|\| id:ebi-a-GCST90001485 | ebi-a-GCST90001485 | TRUE | T | 194010208 | 6.98E-12 | reported | 3437 | 0.1905 | rs116794175 |  |  |  |  |  |  |  |  |  |  |  |  |  |  |  |
| 1.112 | 1 | igd | 0.0042 | C | \|\| id:ebi-a-GCST90001508 | ebi-a-GCST90001508 | TRUE | T | 194010208 | 1.98E-08 | reported | 3437 | 0.1976 | rs116794175 |  |  |  |  |  |  |  |  |  |  |  |  |  |  |  |
| 1.11 | 1 | igd | 0.0042 | C | \|\| id:ebi-a-GCST90001509 | ebi-a-GCST90001509 | TRUE | T | 194010208 | 2.08E-08 | reported | 3437 | 0.1975 | rs116794175 |  |  |  |  |  |  |  |  |  |  |  |  |  |  |  |
| -1.101 | 1 | igd | 0.0042 | C | \|\| id:ebi-a-GCST90001511 | ebi-a-GCST90001511 | TRUE | T | 194010208 | 2.46E-08 | reported | 3437 | 0.1969 | rs116794175 |  |  |  |  |  |  |  |  |  |  |  |  |  |  |  |
| 1.164 | 1 | igd | 0.0042 | C | \|\| id:ebi-a-GCST90001664 | ebi-a-GCST90001664 | TRUE | T | 194010208 | 1.30E-10 | reported | 3440 | 0.1805 | rs116794175 |  |  |  |  |  |  |  |  |  |  |  |  |  |  |  |
| 1.191 | 1 | igd | 0.0042 | C | \|\| id:ebi-a-GCST90001665 | ebi-a-GCST90001665 | TRUE | T | 194010208 | 5.92E-10 | reported | 3440 | 0.1918 | rs116794175 |  |  |  |  |  |  |  |  |  |  |  |  |  |  |  |
| -1.073 | 1 | igd | 0.0042 | C | \|\| id:ebi-a-GCST90001691 | ebi-a-GCST90001691 | TRUE | T | 194010208 | 4.99E-08 | reported | 3440 | 0.1963 | rs116794175 |  |  |  |  |  |  |  |  |  |  |  |  |  |  |  |
| -1.088 | 22 | igd | 0.0042 | C | \|\| id:ebi-a-GCST90001702 | ebi-a-GCST90001702 | TRUE | G | 37514022 | 3.75E-08 | reported | 3657 | 0.1972 | rs78996523 |  |  |  |  |  |  |  |  |  |  |  |  |  |  |  |
| -1.109 | 22 | igd | 0.0042 | C | \|\| id:ebi-a-GCST90001703 | ebi-a-GCST90001703 | TRUE | G | 37514022 | 2.03E-08 | reported | 3657 | 0.1972 | rs78996523 |  |  |  |  |  |  |  |  |  |  |  |  |  |  |  |
| -1.146 | 22 | igd | 0.0042 | C | \|\| id:ebi-a-GCST90001704 | ebi-a-GCST90001704 | TRUE | G | 37514022 | 6.42E-09 | reported | 3657 | 0.197 | rs78996523 |  |  |  |  |  |  |  |  |  |  |  |  |  |  |  |
| 1.978 | 1 | igd | 0.0041 | T | \|\| id:ebi-a-GCST90001483 | ebi-a-GCST90001483 | TRUE | C | 196321168 | 1.11E-19 | reported | 3405 | 0.2166 | rs191753228 |  |  |  |  |  |  |  |  |  |  |  |  |  |  |  |
| 1.611 | 1 | igd | 0.0041 | T | \|\| id:ebi-a-GCST90001483 | ebi-a-GCST90001483 | TRUE | C | 198922068 | 1.79E-18 | reported | 3405 | 0.1826 | rs116517602 |  |  |  |  |  |  |  |  |  |  |  |  |  |  |  |
| 1.366 | 1 | igd | 0.0041 | T | \|\| id:ebi-a-GCST90001484 | ebi-a-GCST90001484 | TRUE | C | 196321168 | 3.85E-10 | reported | 3437 | 0.2176 | rs191753228 |  |  |  |  |  |  |  |  |  |  |  |  |  |  |  |
| 2.072 | 1 | igd | 0.0041 | T | \|\| id:ebi-a-GCST90001485 | ebi-a-GCST90001485 | TRUE | C | 196321168 | 1.38E-21 | reported | 3437 | 0.2156 | rs191753228 |  |  |  |  |  |  |  |  |  |  |  |  |  |  |  |
| 1.787 | 1 | igd | 0.0041 | T | \|\| id:ebi-a-GCST90001485 | ebi-a-GCST90001485 | TRUE | C | 198922068 | 1.84E-22 | reported | 3437 | 0.182 | rs116517602 |  |  |  |  |  |  |  |  |  |  |  |  |  |  |  |
| 1.722 | 1 | igd | 0.0041 | T | \|\| id:ebi-a-GCST90001507 | ebi-a-GCST90001507 | TRUE | C | 198922068 | 3.67E-19 | reported | 3405 | 0.1913 | rs116517602 |  |  |  |  |  |  |  |  |  |  |  |  |  |  |  |
| 1.941 | 1 | igd | 0.0041 | T | \|\| id:ebi-a-GCST90001507 | ebi-a-GCST90001507 | TRUE | C | 196321168 | 1.43E-17 | reported | 3405 | 0.2263 | rs191753228 |  |  |  |  |  |  |  |  |  |  |  |  |  |  |  |
| 1.941 | 1 | igd | 0.0041 | T | \|\| id:ebi-a-GCST90001508 | ebi-a-GCST90001508 | TRUE | C | 198922068 | 4.76E-24 | reported | 3437 | 0.1905 | rs116517602 |  |  |  |  |  |  |  |  |  |  |  |  |  |  |  |
| 2.034 | 1 | igd | 0.0041 | T | \|\| id:ebi-a-GCST90001508 | ebi-a-GCST90001508 | TRUE | C | 196321168 | 2.18E-19 | reported | 3437 | 0.2246 | rs191753228 |  |  |  |  |  |  |  |  |  |  |  |  |  |  |  |
| 1.928 | 1 | igd | 0.0041 | T | \|\| id:ebi-a-GCST90001509 | ebi-a-GCST90001509 | TRUE | C | 196321168 | 1.40E-17 | reported | 3437 | 0.2247 | rs191753228 |  |  |  |  |  |  |  |  |  |  |  |  |  |  |  |
| 1.875 | 1 | igd | 0.0041 | T | \|\| id:ebi-a-GCST90001509 | ebi-a-GCST90001509 | TRUE | C | 198922068 | 1.50E-22 | reported | 3437 | 0.1906 | rs116517602 |  |  |  |  |  |  |  |  |  |  |  |  |  |  |  |
| -1.274 | 1 | igd | 0.0041 | T | \|\| id:ebi-a-GCST90001510 | ebi-a-GCST90001510 | TRUE | C | 196321168 | 2.58E-08 | reported | 3405 | 0.2283 | rs191753228 |  |  |  |  |  |  |  |  |  |  |  |  |  |  |  |
| -1.122 | 1 | igd | 0.0041 | T | \|\| id:ebi-a-GCST90001511 | ebi-a-GCST90001511 | TRUE | C | 198922068 | 5.20E-09 | reported | 3437 | 0.1917 | rs116517602 |  |  |  |  |  |  |  |  |  |  |  |  |  |  |  |
| -1.501 | 1 | igd | 0.0041 | T | \|\| id:ebi-a-GCST90001511 | ebi-a-GCST90001511 | TRUE | C | 196321168 | 2.87E-11 | reported | 3437 | 0.2249 | rs191753228 |  |  |  |  |  |  |  |  |  |  |  |  |  |  |  |
| -1.382 | 1 | igd | 0.0041 | T | \|\| id:ebi-a-GCST90001512 | ebi-a-GCST90001512 | TRUE | C | 196321168 | 9.03E-10 | reported | 3437 | 0.225 | rs191753228 |  |  |  |  |  |  |  |  |  |  |  |  |  |  |  |
| -1.264 | 1 | igd | 0.0041 | T | \|\| id:ebi-a-GCST90001535 | ebi-a-GCST90001535 | TRUE | C | 198922068 | 5.78E-13 | reported | 3427 | 0.1748 | rs116517602 |  |  |  |  |  |  |  |  |  |  |  |  |  |  |  |
| -1.272 | 1 | igd | 0.0041 | T | \|\| id:ebi-a-GCST90001535 | ebi-a-GCST90001535 | TRUE | C | 196321168 | 9.44E-10 | reported | 3427 | 0.2073 | rs191753228 |  |  |  |  |  |  |  |  |  |  |  |  |  |  |  |
| -1.231 | 1 | igd | 0.0041 | T | \|\| id:ebi-a-GCST90001536 | ebi-a-GCST90001536 | TRUE | C | 196321168 | 5.16E-09 | reported | 3427 | 0.2102 | rs191753228 |  |  |  |  |  |  |  |  |  |  |  |  |  |  |  |
| 1.205 | 1 | igd | 0.0041 | T | \|\| id:ebi-a-GCST90001561 | ebi-a-GCST90001561 | TRUE | C | 198922068 | 1.07E-13 | reported | 3427 | 0.1615 | rs116517602 |  |  |  |  |  |  |  |  |  |  |  |  |  |  |  |
| 1.51 | 1 | igd | 0.0041 | T | \|\| id:ebi-a-GCST90001561 | ebi-a-GCST90001561 | TRUE | C | 196321168 | 2.26E-15 | reported | 3427 | 0.1896 | rs191753228 |  |  |  |  |  |  |  |  |  |  |  |  |  |  |  |
| 1.116 | 1 | igd | 0.0041 | T | \|\| id:ebi-a-GCST90001562 | ebi-a-GCST90001562 | TRUE | C | 196321168 | 1.75E-08 | reported | 3427 | 0.1975 | rs191753228 |  |  |  |  |  |  |  |  |  |  |  |  |  |  |  |
| 1.789 | 1 | igd | 0.0041 | T | \|\| id:ebi-a-GCST90001664 | ebi-a-GCST90001664 | TRUE | C | 196321168 | 4.53E-18 | reported | 3440 | 0.2053 | rs191753228 |  |  |  |  |  |  |  |  |  |  |  |  |  |  |  |
| 1.875 | 1 | igd | 0.0041 | T | \|\| id:ebi-a-GCST90001665 | ebi-a-GCST90001665 | TRUE | C | 196321168 | 1.51E-17 | reported | 3440 | 0.2188 | rs191753228 |  |  |  |  |  |  |  |  |  |  |  |  |  |  |  |
| 1.64 | 1 | igd | 0.0041 | T | \|\| id:ebi-a-GCST90001666 | ebi-a-GCST90001666 | TRUE | C | 196321168 | 8.27E-17 | reported | 3408 | 0.1959 | rs191753228 |  |  |  |  |  |  |  |  |  |  |  |  |  |  |  |
| -1.333 | 1 | igd | 0.0041 | T | \|\| id:ebi-a-GCST90001667 | ebi-a-GCST90001667 | TRUE | C | 198922068 | 6.83E-14 | reported | 3440 | 0.1772 | rs116517602 |  |  |  |  |  |  |  |  |  |  |  |  |  |  |  |
| -1.692 | 1 | igd | 0.0041 | T | \|\| id:ebi-a-GCST90001667 | ebi-a-GCST90001667 | TRUE | C | 196321168 | 6.67E-16 | reported | 3440 | 0.2084 | rs191753228 |  |  |  |  |  |  |  |  |  |  |  |  |  |  |  |
| -1.571 | 1 | igd | 0.0041 | T | \|\| id:ebi-a-GCST90001668 | ebi-a-GCST90001668 | TRUE | C | 196321168 | 8.98E-13 | reported | 3440 | 0.2191 | rs191753228 |  |  |  |  |  |  |  |  |  |  |  |  |  |  |  |
| -1.401 | 1 | igd | 0.0041 | T | \|\| id:ebi-a-GCST90001668 | ebi-a-GCST90001668 | TRUE | C | 198922068 | 8.07E-14 | reported | 3440 | 0.1867 | rs116517602 |  |  |  |  |  |  |  |  |  |  |  |  |  |  |  |
| -1.545 | 1 | igd | 0.0041 | T | \|\| id:ebi-a-GCST90001669 | ebi-a-GCST90001669 | TRUE | C | 196321168 | 1.15E-14 | reported | 3408 | 0.1991 | rs191753228 |  |  |  |  |  |  |  |  |  |  |  |  |  |  |  |
| 1.072 | 10 | igd | 0.0041 | A | \|\| id:ebi-a-GCST90001670 | ebi-a-GCST90001670 | TRUE | C | 95773554 | 1.96E-08 | reported | 3440 | 0.1905 | rs139933525 |  |  |  |  |  |  |  |  |  |  |  |  |  |  |  |
| 1.094 | 10 | igd | 0.0041 | A | \|\| id:ebi-a-GCST90001671 | ebi-a-GCST90001671 | TRUE | C | 95773554 | 9.59E-09 | reported | 3440 | 0.1903 | rs139933525 |  |  |  |  |  |  |  |  |  |  |  |  |  |  |  |
| 1.054 | 10 | igd | 0.0041 | A | \|\| id:ebi-a-GCST90001672 | ebi-a-GCST90001672 | TRUE | C | 95773554 | 4.72E-08 | reported | 3408 | 0.1926 | rs139933525 |  |  |  |  |  |  |  |  |  |  |  |  |  |  |  |
| -1.654 | 1 | igd | 0.0041 | T | \|\| id:ebi-a-GCST90001691 | ebi-a-GCST90001691 | TRUE | C | 198922068 | 2.58E-18 | reported | 3440 | 0.1885 | rs116517602 |  |  |  |  |  |  |  |  |  |  |  |  |  |  |  |
| -1.702 | 1 | igd | 0.0041 | T | \|\| id:ebi-a-GCST90001691 | ebi-a-GCST90001691 | TRUE | C | 196321168 | 3.06E-14 | reported | 3440 | 0.2232 | rs191753228 |  |  |  |  |  |  |  |  |  |  |  |  |  |  |  |
| -1.571 | 1 | igd | 0.0041 | T | \|\| id:ebi-a-GCST90001692 | ebi-a-GCST90001692 | TRUE | C | 198922068 | 1.03E-16 | reported | 3440 | 0.1882 | rs116517602 |  |  |  |  |  |  |  |  |  |  |  |  |  |  |  |
| -1.719 | 1 | igd | 0.0041 | T | \|\| id:ebi-a-GCST90001692 | ebi-a-GCST90001692 | TRUE | C | 196321168 | 1.12E-14 | reported | 3440 | 0.2215 | rs191753228 |  |  |  |  |  |  |  |  |  |  |  |  |  |  |  |
| -1.632 | 1 | igd | 0.0041 | T | \|\| id:ebi-a-GCST90001693 | ebi-a-GCST90001693 | TRUE | C | 198922068 | 1.33E-17 | reported | 3408 | 0.19 | rs116517602 |  |  |  |  |  |  |  |  |  |  |  |  |  |  |  |
| -1.448 | 1 | igd | 0.0041 | T | \|\| id:ebi-a-GCST90001693 | ebi-a-GCST90001693 | TRUE | C | 196321168 | 1.46E-10 | reported | 3408 | 0.2252 | rs191753228 |  |  |  |  |  |  |  |  |  |  |  |  |  |  |  |
| 157 | 1 | igd | 0.0041 | G | \|\| id:ebi-a-GCST90001698 | ebi-a-GCST90001698 | TRUE | C | 30851229 | 2.16E-08 | reported | 3408 | 27.97 | rs150501994 |  |  |  |  |  |  |  |  |  |  |  |  |  |  |  |
| 152.6 | 2 | igd | 0.0041 | C | \|\| id:ebi-a-GCST90001698 | ebi-a-GCST90001698 | TRUE | T | 18361173 | 1.82E-08 | reported | 3408 | 27.04 | rs114157137 |  |  |  |  |  |  |  |  |  |  |  |  |  |  |  |
| 166.4 | 2 | igd | 0.0041 | T | \|\| id:ebi-a-GCST90001698 | ebi-a-GCST90001698 | TRUE | C | 107184424 | 1.46E-09 | reported | 3408 | 27.44 | rs7426047 |  |  |  |  |  |  |  |  |  |  |  |  |  |  |  |
| 225.5 | 3 | igd | 0.0041 | C | \|\| id:ebi-a-GCST90001698 | ebi-a-GCST90001698 | TRUE | T | 187980426 | 1.75E-16 | reported | 3408 | 27.24 | rs149082401 |  |  |  |  |  |  |  |  |  |  |  |  |  |  |  |
| 192.2 | 7 | igd | 0.0041 | G | \|\| id:ebi-a-GCST90001698 | ebi-a-GCST90001698 | TRUE | T | 56724250 | 1.88E-11 | reported | 3408 | 28.52 | rs115221944 |  |  |  |  |  |  |  |  |  |  |  |  |  |  |  |
| 223.2 | 7 | igd | 0.0041 | T | \|\| id:ebi-a-GCST90001698 | ebi-a-GCST90001698 | TRUE | C | 158902149 | 1.01E-12 | reported | 3408 | 31.19 | rs117276320 |  |  |  |  |  |  |  |  |  |  |  |  |  |  |  |
| 159.1 | 12 | igd | 0.0041 | A | \|\| id:ebi-a-GCST90001698 | ebi-a-GCST90001698 | TRUE | G | 109146504 | 1.79E-08 | reported | 3408 | 28.18 | rs183962585 |  |  |  |  |  |  |  |  |  |  |  |  |  |  |  |
| 167.7 | 14 | igd | 0.0041 | G | \|\| id:ebi-a-GCST90001698 | ebi-a-GCST90001698 | TRUE | A | 55666624 | 3.29E-09 | reported | 3408 | 28.28 | rs139431704 |  |  |  |  |  |  |  |  |  |  |  |  |  |  |  |
| 160.7 | 18 | igd | 0.0041 | T | \|\| id:ebi-a-GCST90001698 | ebi-a-GCST90001698 | TRUE | A | 73901698 | 1.16E-08 | reported | 3408 | 28.09 | rs147136626 |  |  |  |  |  |  |  |  |  |  |  |  |  |  |  |
| -1.443 | 16 | igd | 0.0041 | C | \|\| id:ebi-a-GCST90001723 | ebi-a-GCST90001723 | TRUE | G | 32422808 | 3.15E-11 | reported | 3656 | 0.2168 | rs138920008 |  |  |  |  |  |  |  |  |  |  |  |  |  |  |  |
| -1.326 | 16 | igd | 0.0041 | C | \|\| id:ebi-a-GCST90001724 | ebi-a-GCST90001724 | TRUE | G | 32422808 | 1.26E-09 | reported | 3656 | 0.2178 | rs138920008 |  |  |  |  |  |  |  |  |  |  |  |  |  |  |  |
| -1.188 | 16 | igd | 0.0041 | C | \|\| id:ebi-a-GCST90001725 | ebi-a-GCST90001725 | TRUE | G | 32422808 | 4.50E-08 | reported | 3656 | 0.2168 | rs138920008 |  |  |  |  |  |  |  |  |  |  |  |  |  |  |  |
| -1.438 | 16 | igd | 0.0041 | C | \|\| id:ebi-a-GCST90001729 | ebi-a-GCST90001729 | TRUE | G | 32422808 | 6.50E-11 | reported | 3657 | 0.2195 | rs138920008 |  |  |  |  |  |  |  |  |  |  |  |  |  |  |  |
| -1.234 | 16 | igd | 0.0041 | C | \|\| id:ebi-a-GCST90001730 | ebi-a-GCST90001730 | TRUE | G | 32422808 | 1.50E-08 | reported | 3657 | 0.2174 | rs138920008 |  |  |  |  |  |  |  |  |  |  |  |  |  |  |  |
| -1.197 | 16 | igd | 0.0041 | C | \|\| id:ebi-a-GCST90001733 | ebi-a-GCST90001733 | TRUE | G | 32422808 | 4.23E-08 | reported | 3657 | 0.2179 | rs138920008 |  |  |  |  |  |  |  |  |  |  |  |  |  |  |  |
| -1.514 | 16 | igd | 0.0041 | C | \|\| id:ebi-a-GCST90001735 | ebi-a-GCST90001735 | TRUE | G | 32422808 | 2.63E-12 | reported | 3657 | 0.2157 | rs138920008 |  |  |  |  |  |  |  |  |  |  |  |  |  |  |  |
| -1.277 | 16 | igd | 0.0041 | C | \|\| id:ebi-a-GCST90001736 | ebi-a-GCST90001736 | TRUE | G | 32422808 | 4.85E-09 | reported | 3657 | 0.2176 | rs138920008 |  |  |  |  |  |  |  |  |  |  |  |  |  |  |  |
| -1.283 | 16 | igd | 0.0041 | C | \|\| id:ebi-a-GCST90001738 | ebi-a-GCST90001738 | TRUE | G | 32422808 | 3.96E-09 | reported | 3656 | 0.2175 | rs138920008 |  |  |  |  |  |  |  |  |  |  |  |  |  |  |  |
| -1.364 | 16 | igd | 0.0041 | C | \|\| id:ebi-a-GCST90001740 | ebi-a-GCST90001740 | TRUE | G | 32422808 | 3.69E-10 | reported | 3657 | 0.2171 | rs138920008 |  |  |  |  |  |  |  |  |  |  |  |  |  |  |  |
| -1.3 | 16 | igd | 0.0041 | C | \|\| id:ebi-a-GCST90001742 | ebi-a-GCST90001742 | TRUE | G | 32422808 | 3.41E-09 | reported | 3657 | 0.2194 | rs138920008 |  |  |  |  |  |  |  |  |  |  |  |  |  |  |  |
| 1.788 | 1 | igd | 0.0041 | T | \|\| id:ebi-a-GCST90002098 | ebi-a-GCST90002098 | TRUE | C | 198922068 | 8.35E-21 | reported | 2910 | 0.1897 | rs116517602 |  |  |  |  |  |  |  |  |  |  |  |  |  |  |  |
| 1.147 | 1 | igd | 0.0041 | T | \|\| id:ebi-a-GCST90002100 | ebi-a-GCST90002100 | TRUE | C | 198922068 | 1.29E-08 | reported | 2910 | 0.201 | rs116517602 |  |  |  |  |  |  |  |  |  |  |  |  |  |  |  |
| 1.165 | 1 | igd | 0.0041 | A | \|\| id:ebi-a-GCST90002102 | ebi-a-GCST90002102 | TRUE | G | 190341116 | 3.24E-08 | reported | 2919 | 0.2103 | rs114454943 |  |  |  |  |  |  |  |  |  |  |  |  |  |  |  |
| 1.499 | 1 | igd | 0.0041 | A | \|\| id:ebi-a-GCST90002102 | ebi-a-GCST90002102 | TRUE | G | 199999121 | 4.74E-14 | reported | 2919 | 0.1979 | rs11579717 |  |  |  |  |  |  |  |  |  |  |  |  |  |  |  |
| 1.164 | 1 | igd | 0.0041 | A | \|\| id:ebi-a-GCST90002102 | ebi-a-GCST90002102 | TRUE | G | 190444745 | 3.05E-08 | reported | 2919 | 0.2096 | rs142274302 |  |  |  |  |  |  |  |  |  |  |  |  |  |  |  |
| 120.9 | 1 | igd | 0.004 | T | \|\| id:ebi-a-GCST90001695 | ebi-a-GCST90001695 | TRUE | C | 17259563 | 1.37E-08 | reported | 3408 | 21.25 | rs186674046 |  |  |  |  |  |  |  |  |  |  |  |  |  |  |  |
| 123.2 | 12 | igd | 0.004 | T | \|\| id:ebi-a-GCST90001695 | ebi-a-GCST90001695 | TRUE | C | 59066617 | 1.42E-08 | reported | 3408 | 21.68 | rs74902462 |  |  |  |  |  |  |  |  |  |  |  |  |  |  |  |
| 113 | 16 | igd | 0.004 | T | \|\| id:ebi-a-GCST90001695 | ebi-a-GCST90001695 | TRUE | C | 80860190 | 4.19E-08 | reported | 3408 | 20.57 | rs2549733 |  |  |  |  |  |  |  |  |  |  |  |  |  |  |  |
| 170.1 | 1 | igd | 0.004 | C | \|\| id:ebi-a-GCST90001698 | ebi-a-GCST90001698 | TRUE | T | 184687251 | 1.15E-09 | reported | 3408 | 27.87 | rs75420102 |  |  |  |  |  |  |  |  |  |  |  |  |  |  |  |
| 166.7 | 2 | igd | 0.004 | A | \|\| id:ebi-a-GCST90001698 | ebi-a-GCST90001698 | TRUE | G | 147743716 | 2.53E-09 | reported | 3408 | 27.9 | rs114859965 |  |  |  |  |  |  |  |  |  |  |  |  |  |  |  |
| 171.1 | 2 | igd | 0.004 | A | \|\| id:ebi-a-GCST90001698 | ebi-a-GCST90001698 | TRUE | C | 40115478 | 2.20E-09 | reported | 3408 | 28.52 | rs72789132 |  |  |  |  |  |  |  |  |  |  |  |  |  |  |  |
| 178 | 4 | igd | 0.004 | G | \|\| id:ebi-a-GCST90001698 | ebi-a-GCST90001698 | TRUE | C | 97323594 | 9.61E-11 | reported | 3408 | 27.41 | rs181416491 |  |  |  |  |  |  |  |  |  |  |  |  |  |  |  |
| 189.6 | 5 | igd | 0.004 | A | \|\| id:ebi-a-GCST90001698 | ebi-a-GCST90001698 | TRUE | G | 135639453 | 1.20E-10 | reported | 3408 | 29.36 | rs192556004 |  |  |  |  |  |  |  |  |  |  |  |  |  |  |  |
| 176.3 | 6 | igd | 0.004 | T | \|\| id:ebi-a-GCST90001698 | ebi-a-GCST90001698 | TRUE | C | 168136303 | 1.74E-10 | reported | 3408 | 27.54 | rs140087548 |  |  |  |  |  |  |  |  |  |  |  |  |  |  |  |
| 204.6 | 7 | igd | 0.004 | G | \|\| id:ebi-a-GCST90001698 | ebi-a-GCST90001698 | TRUE | T | 46417626 | 4.93E-14 | reported | 3408 | 27.04 | rs73116391 |  |  |  |  |  |  |  |  |  |  |  |  |  |  |  |
| 183.3 | 7 | igd | 0.004 | T | \|\| id:ebi-a-GCST90001698 | ebi-a-GCST90001698 | TRUE | C | 112589900 | 3.96E-11 | reported | 3408 | 27.66 | rs62491100 |  |  |  |  |  |  |  |  |  |  |  |  |  |  |  |
| 178.4 | 7 | igd | 0.004 | T | \|\| id:ebi-a-GCST90001698 | ebi-a-GCST90001698 | TRUE | C | 6017749 | 3.41E-09 | reported | 3408 | 30.1 | rs193218059 |  |  |  |  |  |  |  |  |  |  |  |  |  |  |  |
| 159.8 | 8 | igd | 0.004 | C | \|\| id:ebi-a-GCST90001698 | ebi-a-GCST90001698 | TRUE | T | 16615390 | 1.07E-08 | reported | 3408 | 27.87 | rs7823439 |  |  |  |  |  |  |  |  |  |  |  |  |  |  |  |
| 177.1 | 10 | igd | 0.004 | C | \|\| id:ebi-a-GCST90001698 | ebi-a-GCST90001698 | TRUE | A | 120573449 | 2.42E-08 | reported | 3408 | 31.66 | rs191325818 |  |  |  |  |  |  |  |  |  |  |  |  |  |  |  |
| 152.4 | 11 | igd | 0.004 | T | \|\| id:ebi-a-GCST90001698 | ebi-a-GCST90001698 | TRUE | A | 92081132 | 1.54E-08 | reported | 3408 | 26.87 | rs186370342 |  |  |  |  |  |  |  |  |  |  |  |  |  |  |  |
| 248.6 | 11 | igd | 0.004 | A | \|\| id:ebi-a-GCST90001698 | ebi-a-GCST90001698 | TRUE | G | 3830358 | 4.08E-18 | reported | 3408 | 28.49 | rs118014538 |  |  |  |  |  |  |  |  |  |  |  |  |  |  |  |
| 161.3 | 12 | igd | 0.004 | A | \|\| id:ebi-a-GCST90001698 | ebi-a-GCST90001698 | TRUE | C | 12221257 | 5.29E-09 | reported | 3408 | 27.56 | rs139733089 |  |  |  |  |  |  |  |  |  |  |  |  |  |  |  |
| 164.3 | 13 | igd | 0.004 | G | \|\| id:ebi-a-GCST90001698 | ebi-a-GCST90001698 | TRUE | A | 70597692 | 3.99E-09 | reported | 3408 | 27.85 | rs116860298 |  |  |  |  |  |  |  |  |  |  |  |  |  |  |  |
| 174.2 | 13 | igd | 0.004 | C | \|\| id:ebi-a-GCST90001698 | ebi-a-GCST90001698 | TRUE | T | 97913305 | 2.72E-10 | reported | 3408 | 27.5 | rs73555775 |  |  |  |  |  |  |  |  |  |  |  |  |  |  |  |
| 174.9 | 13 | igd | 0.004 | C | \|\| id:ebi-a-GCST90001698 | ebi-a-GCST90001698 | TRUE | T | 98181976 | 8.51E-11 | reported | 3408 | 26.86 | rs9556733 |  |  |  |  |  |  |  |  |  |  |  |  |  |  |  |
| 176.2 | 15 | igd | 0.004 | C | \|\| id:ebi-a-GCST90001698 | ebi-a-GCST90001698 | TRUE | A | 29298378 | 3.65E-09 | reported | 3408 | 29.79 | rs146259677 |  |  |  |  |  |  |  |  |  |  |  |  |  |  |  |
| 159.7 | 15 | igd | 0.004 | G | \|\| id:ebi-a-GCST90001698 | ebi-a-GCST90001698 | TRUE | A | 30234373 | 1.82E-08 | reported | 3408 | 28.3 | rs149438077 |  |  |  |  |  |  |  |  |  |  |  |  |  |  |  |
| 188.1 | 20 | igd | 0.004 | G | \|\| id:ebi-a-GCST90001698 | ebi-a-GCST90001698 | TRUE | C | 42202097 | 1.86E-11 | reported | 3408 | 27.91 | rs140941472 |  |  |  |  |  |  |  |  |  |  |  |  |  |  |  |
| 1.053 | 1 | igd | 0.0039 | A | \|\| id:ebi-a-GCST90001664 | ebi-a-GCST90001664 | TRUE | G | 191648600 | 1.53E-08 | reported | 3440 | 0.1857 | rs147071297 |  |  |  |  |  |  |  |  |  |  |  |  |  |  |  |
| 1.077 | 10 | igd | 0.0039 | T | \|\| id:ebi-a-GCST90001670 | ebi-a-GCST90001670 | TRUE | C | 95991311 | 2.03E-08 | reported | 3440 | 0.1915 | rs184480114 |  |  |  |  |  |  |  |  |  |  |  |  |  |  |  |
| 1.894 | 1 | igd | 0.0039 | T | \|\| id:ebi-a-GCST90002102 | ebi-a-GCST90002102 | TRUE | C | 196321168 | 3.48E-16 | reported | 2919 | 0.2309 | rs191753228 |  |  |  |  |  |  |  |  |  |  |  |  |  |  |  |
| 155.1 | 2 | igd | 0.0038 | A | \|\| id:ebi-a-GCST90001698 | ebi-a-GCST90001698 | TRUE | G | 8152821 | 3.76E-08 | reported | 3408 | 28.13 | rs138567976 |  |  |  |  |  |  |  |  |  |  |  |  |  |  |  |
| 156.8 | 2 | igd | 0.0038 | C | \|\| id:ebi-a-GCST90001698 | ebi-a-GCST90001698 | TRUE | T | 118874672 | 3.56E-08 | reported | 3408 | 28.38 | rs71422564 |  |  |  |  |  |  |  |  |  |  |  |  |  |  |  |
| 172.3 | 5 | igd | 0.0038 | T | \|\| id:ebi-a-GCST90001698 | ebi-a-GCST90001698 | TRUE | C | 176911575 | 1.93E-09 | reported | 3408 | 28.61 | rs114425563 |  |  |  |  |  |  |  |  |  |  |  |  |  |  |  |
| 175.5 | 6 | igd | 0.0038 | T | \|\| id:ebi-a-GCST90001698 | ebi-a-GCST90001698 | TRUE | G | 119363401 | 5.27E-09 | reported | 3408 | 29.98 | rs73767230 |  |  |  |  |  |  |  |  |  |  |  |  |  |  |  |
| 153.5 | 7 | igd | 0.0038 | T | \|\| id:ebi-a-GCST90001698 | ebi-a-GCST90001698 | TRUE | A | 46569794 | 3.96E-08 | reported | 3408 | 27.88 | rs73119694 |  |  |  |  |  |  |  |  |  |  |  |  |  |  |  |
| 196.4 | 9 | igd | 0.0038 | C | \|\| id:ebi-a-GCST90001698 | ebi-a-GCST90001698 | TRUE | A | 504491 | 4.89E-12 | reported | 3408 | 28.32 | rs374964617 |  |  |  |  |  |  |  |  |  |  |  |  |  |  |  |
| 151 | 12 | igd | 0.0038 | T | \|\| id:ebi-a-GCST90001698 | ebi-a-GCST90001698 | TRUE | A | 63097090 | 4.06E-08 | reported | 3408 | 27.45 | rs143936985 |  |  |  |  |  |  |  |  |  |  |  |  |  |  |  |
| 162.1 | 14 | igd | 0.0038 | A | \|\| id:ebi-a-GCST90001698 | ebi-a-GCST90001698 | TRUE | T | 63841100 | 1.72E-08 | reported | 3408 | 28.69 | rs118022841 |  |  |  |  |  |  |  |  |  |  |  |  |  |  |  |
| 166.7 | 20 | igd | 0.0038 | A | \|\| id:ebi-a-GCST90001698 | ebi-a-GCST90001698 | TRUE | G | 37477054 | 4.13E-09 | reported | 3408 | 28.27 | rs142718677 |  |  |  |  |  |  |  |  |  |  |  |  |  |  |  |
| 1.756 | 1 | igd | 0.0038 | T | \|\| id:ebi-a-GCST90002102 | ebi-a-GCST90002102 | TRUE | C | 198922068 | 1.44E-18 | reported | 2919 | 0.1983 | rs116517602 |  |  |  |  |  |  |  |  |  |  |  |  |  |  |  |
| 1.517 | 1 | igd | 0.0037 | T | \|\| id:ebi-a-GCST90001483 | ebi-a-GCST90001483 | TRUE | C | 197574661 | 1.67E-14 | reported | 3405 | 0.1968 | rs145970654 |  |  |  |  |  |  |  |  |  |  |  |  |  |  |  |
| 1.515 | 1 | igd | 0.0037 | C | \|\| id:ebi-a-GCST90001483 | ebi-a-GCST90001483 | TRUE | T | 197687255 | 1.74E-14 | reported | 3405 | 0.1967 | rs115549264 |  |  |  |  |  |  |  |  |  |  |  |  |  |  |  |
| 1.561 | 1 | igd | 0.0037 | T | \|\| id:ebi-a-GCST90001507 | ebi-a-GCST90001507 | TRUE | C | 197574661 | 4.13E-14 | reported | 3405 | 0.2056 | rs145970654 |  |  |  |  |  |  |  |  |  |  |  |  |  |  |  |
| 1.559 | 1 | igd | 0.0037 | C | \|\| id:ebi-a-GCST90001507 | ebi-a-GCST90001507 | TRUE | T | 197687255 | 4.20E-14 | reported | 3405 | 0.2056 | rs115549264 |  |  |  |  |  |  |  |  |  |  |  |  |  |  |  |
| 1.37 | 1 | igd | 0.0037 | T | \|\| id:ebi-a-GCST90001666 | ebi-a-GCST90001666 | TRUE | C | 197574661 | 2.05E-14 | reported | 3408 | 0.1784 | rs145970654 |  |  |  |  |  |  |  |  |  |  |  |  |  |  |  |
| 1.369 | 1 | igd | 0.0037 | C | \|\| id:ebi-a-GCST90001666 | ebi-a-GCST90001666 | TRUE | T | 197687255 | 2.08E-14 | reported | 3408 | 0.1783 | rs115549264 |  |  |  |  |  |  |  |  |  |  |  |  |  |  |  |
| -1.254 | 1 | igd | 0.0037 | T | \|\| id:ebi-a-GCST90001669 | ebi-a-GCST90001669 | TRUE | C | 197574661 | 5.03E-12 | reported | 3408 | 0.181 | rs145970654 |  |  |  |  |  |  |  |  |  |  |  |  |  |  |  |
| -1.253 | 1 | igd | 0.0037 | C | \|\| id:ebi-a-GCST90001669 | ebi-a-GCST90001669 | TRUE | T | 197687255 | 5.11E-12 | reported | 3408 | 0.1809 | rs115549264 |  |  |  |  |  |  |  |  |  |  |  |  |  |  |  |
| -1.387 | 1 | igd | 0.0037 | T | \|\| id:ebi-a-GCST90001693 | ebi-a-GCST90001693 | TRUE | C | 197574661 | 1.30E-11 | reported | 3408 | 0.2042 | rs145970654 |  |  |  |  |  |  |  |  |  |  |  |  |  |  |  |
| -1.386 | 1 | igd | 0.0037 | C | \|\| id:ebi-a-GCST90001693 | ebi-a-GCST90001693 | TRUE | T | 197687255 | 1.30E-11 | reported | 3408 | 0.2041 | rs115549264 |  |  |  |  |  |  |  |  |  |  |  |  |  |  |  |
| 142.3 | 4 | igd | 0.0037 | T | \|\| id:ebi-a-GCST90001695 | ebi-a-GCST90001695 | TRUE | C | 8174723 | 2.87E-10 | reported | 3408 | 22.5 | rs140311178 |  |  |  |  |  |  |  |  |  |  |  |  |  |  |  |
| 138.5 | 10 | igd | 0.0037 | A | \|\| id:ebi-a-GCST90001695 | ebi-a-GCST90001695 | TRUE | C | 89153882 | 1.80E-10 | reported | 3408 | 21.65 | rs189683893 |  |  |  |  |  |  |  |  |  |  |  |  |  |  |  |
| 164.5 | 1 | igd | 0.0037 | A | \|\| id:ebi-a-GCST90001698 | ebi-a-GCST90001698 | TRUE | G | 207521506 | 1.22E-08 | reported | 3408 | 28.8 | rs28739007 |  |  |  |  |  |  |  |  |  |  |  |  |  |  |  |
| 189.8 | 1 | igd | 0.0037 | G | \|\| id:ebi-a-GCST90001698 | ebi-a-GCST90001698 | TRUE | A | 207678128 | 7.67E-11 | reported | 3408 | 29.08 | rs113698814 |  |  |  |  |  |  |  |  |  |  |  |  |  |  |  |
| 160 | 3 | igd | 0.0037 | C | \|\| id:ebi-a-GCST90001698 | ebi-a-GCST90001698 | TRUE | G | 23633832 | 4.31E-08 | reported | 3408 | 29.14 | rs147774047 |  |  |  |  |  |  |  |  |  |  |  |  |  |  |  |
| 160.9 | 5 | igd | 0.0037 | G | \|\| id:ebi-a-GCST90001698 | ebi-a-GCST90001698 | TRUE | A | 180495180 | 1.47E-08 | reported | 3408 | 28.33 | rs58243030 |  |  |  |  |  |  |  |  |  |  |  |  |  |  |  |
| 203.4 | 9 | igd | 0.0037 | G | \|\| id:ebi-a-GCST90001698 | ebi-a-GCST90001698 | TRUE | C | 102347204 | 1.49E-11 | reported | 3408 | 30.04 | rs9408992 |  |  |  |  |  |  |  |  |  |  |  |  |  |  |  |
| 188.4 | 9 | igd | 0.0037 | T | \|\| id:ebi-a-GCST90001698 | ebi-a-GCST90001698 | TRUE | C | 140235501 | 2.46E-09 | reported | 3408 | 31.51 | rs77733172 |  |  |  |  |  |  |  |  |  |  |  |  |  |  |  |
| 167 | 10 | igd | 0.0037 | T | \|\| id:ebi-a-GCST90001698 | ebi-a-GCST90001698 | TRUE | A | 87670003 | 1.25E-08 | reported | 3408 | 29.27 | rs78888005 |  |  |  |  |  |  |  |  |  |  |  |  |  |  |  |
| 252.9 | 13 | igd | 0.0037 | G | \|\| id:ebi-a-GCST90001698 | ebi-a-GCST90001698 | TRUE | C | 19869255 | 2.05E-18 | reported | 3408 | 28.72 | rs117075995 |  |  |  |  |  |  |  |  |  |  |  |  |  |  |  |
| 219 | 13 | igd | 0.0037 | A | \|\| id:ebi-a-GCST90001698 | ebi-a-GCST90001698 | TRUE | G | 98908125 | 8.21E-15 | reported | 3408 | 28.08 | rs10492710 |  |  |  |  |  |  |  |  |  |  |  |  |  |  |  |
| 185 | 15 | igd | 0.0037 | A | \|\| id:ebi-a-GCST90001698 | ebi-a-GCST90001698 | TRUE | G | 39668760 | 6.24E-10 | reported | 3408 | 29.82 | rs75669615 |  |  |  |  |  |  |  |  |  |  |  |  |  |  |  |
| 181.5 | 18 | igd | 0.0037 | A | \|\| id:ebi-a-GCST90001698 | ebi-a-GCST90001698 | TRUE | C | 7461184 | 6.06E-10 | reported | 3408 | 29.24 | rs186394234 |  |  |  |  |  |  |  |  |  |  |  |  |  |  |  |
| 182 | 20 | igd | 0.0037 | A | \|\| id:ebi-a-GCST90001698 | ebi-a-GCST90001698 | TRUE | G | 38150081 | 5.29E-09 | reported | 3408 | 31.1 | rs149644685 |  |  |  |  |  |  |  |  |  |  |  |  |  |  |  |
| -1.165 | 22 | igd | 0.0037 | G | \|\| id:ebi-a-GCST90001704 | ebi-a-GCST90001704 | TRUE | A | 41271935 | 1.53E-08 | reported | 3657 | 0.2054 | rs142837365 |  |  |  |  |  |  |  |  |  |  |  |  |  |  |  |
| 1.259 | 1 | igd | 0.0036 | A | \|\| id:ebi-a-GCST90001484 | ebi-a-GCST90001484 | TRUE | G | 197921246 | 2.82E-10 | reported | 3437 | 0.199 | rs115328872 |  |  |  |  |  |  |  |  |  |  |  |  |  |  |  |
| 1.589 | 1 | igd | 0.0036 | C | \|\| id:ebi-a-GCST90001485 | ebi-a-GCST90001485 | TRUE | T | 197687255 | 7.31E-16 | reported | 3437 | 0.1961 | rs115549264 |  |  |  |  |  |  |  |  |  |  |  |  |  |  |  |
| 1.591 | 1 | igd | 0.0036 | T | \|\| id:ebi-a-GCST90001485 | ebi-a-GCST90001485 | TRUE | C | 197574661 | 6.93E-16 | reported | 3437 | 0.1962 | rs145970654 |  |  |  |  |  |  |  |  |  |  |  |  |  |  |  |
| 1.604 | 1 | igd | 0.0036 | T | \|\| id:ebi-a-GCST90001508 | ebi-a-GCST90001508 | TRUE | C | 197574661 | 5.95E-15 | reported | 3437 | 0.2046 | rs145970654 |  |  |  |  |  |  |  |  |  |  |  |  |  |  |  |
| 1.603 | 1 | igd | 0.0036 | C | \|\| id:ebi-a-GCST90001508 | ebi-a-GCST90001508 | TRUE | T | 197687255 | 6.18E-15 | reported | 3437 | 0.2046 | rs115549264 |  |  |  |  |  |  |  |  |  |  |  |  |  |  |  |
| 1.578 | 1 | igd | 0.0036 | T | \|\| id:ebi-a-GCST90001509 | ebi-a-GCST90001509 | TRUE | C | 197574661 | 1.61E-14 | reported | 3437 | 0.2046 | rs145970654 |  |  |  |  |  |  |  |  |  |  |  |  |  |  |  |
| 1.576 | 1 | igd | 0.0036 | C | \|\| id:ebi-a-GCST90001509 | ebi-a-GCST90001509 | TRUE | T | 197687255 | 1.66E-14 | reported | 3437 | 0.2045 | rs115549264 |  |  |  |  |  |  |  |  |  |  |  |  |  |  |  |
| -1.286 | 1 | igd | 0.0036 | C | \|\| id:ebi-a-GCST90001511 | ebi-a-GCST90001511 | TRUE | T | 197674188 | 3.56E-10 | reported | 3437 | 0.2044 | rs114780135 |  |  |  |  |  |  |  |  |  |  |  |  |  |  |  |
| -1.098 | 1 | igd | 0.0036 | C | \|\| id:ebi-a-GCST90001535 | ebi-a-GCST90001535 | TRUE | T | 197687255 | 5.66E-09 | reported | 3427 | 0.1879 | rs115549264 |  |  |  |  |  |  |  |  |  |  |  |  |  |  |  |
| -1.098 | 1 | igd | 0.0036 | T | \|\| id:ebi-a-GCST90001535 | ebi-a-GCST90001535 | TRUE | C | 197574661 | 5.57E-09 | reported | 3427 | 0.1879 | rs145970654 |  |  |  |  |  |  |  |  |  |  |  |  |  |  |  |
| 1.283 | 1 | igd | 0.0036 | C | \|\| id:ebi-a-GCST90001561 | ebi-a-GCST90001561 | TRUE | T | 197687255 | 1.26E-13 | reported | 3427 | 0.1724 | rs115549264 |  |  |  |  |  |  |  |  |  |  |  |  |  |  |  |
| 1.284 | 1 | igd | 0.0036 | T | \|\| id:ebi-a-GCST90001561 | ebi-a-GCST90001561 | TRUE | C | 197574661 | 1.21E-13 | reported | 3427 | 0.1724 | rs145970654 |  |  |  |  |  |  |  |  |  |  |  |  |  |  |  |
| 1.474 | 1 | igd | 0.0036 | T | \|\| id:ebi-a-GCST90001664 | ebi-a-GCST90001664 | TRUE | C | 197574661 | 3.90E-15 | reported | 3440 | 0.1867 | rs145970654 |  |  |  |  |  |  |  |  |  |  |  |  |  |  |  |
| 1.473 | 1 | igd | 0.0036 | C | \|\| id:ebi-a-GCST90001664 | ebi-a-GCST90001664 | TRUE | T | 197687255 | 4.01E-15 | reported | 3440 | 0.1866 | rs115549264 |  |  |  |  |  |  |  |  |  |  |  |  |  |  |  |
| 1.491 | 1 | igd | 0.0036 | T | \|\| id:ebi-a-GCST90001665 | ebi-a-GCST90001665 | TRUE | C | 197574661 | 9.95E-14 | reported | 3440 | 0.1995 | rs145970654 |  |  |  |  |  |  |  |  |  |  |  |  |  |  |  |
| 1.489 | 1 | igd | 0.0036 | C | \|\| id:ebi-a-GCST90001665 | ebi-a-GCST90001665 | TRUE | T | 197687255 | 1.02E-13 | reported | 3440 | 0.1994 | rs115549264 |  |  |  |  |  |  |  |  |  |  |  |  |  |  |  |
| -1.361 | 1 | igd | 0.0036 | C | \|\| id:ebi-a-GCST90001667 | ebi-a-GCST90001667 | TRUE | T | 197687255 | 8.33E-13 | reported | 3440 | 0.1895 | rs115549264 |  |  |  |  |  |  |  |  |  |  |  |  |  |  |  |
| -1.362 | 1 | igd | 0.0036 | T | \|\| id:ebi-a-GCST90001667 | ebi-a-GCST90001667 | TRUE | C | 197574661 | 8.27E-13 | reported | 3440 | 0.1896 | rs145970654 |  |  |  |  |  |  |  |  |  |  |  |  |  |  |  |
| -1.415 | 1 | igd | 0.0036 | C | \|\| id:ebi-a-GCST90001668 | ebi-a-GCST90001668 | TRUE | T | 197674188 | 1.49E-12 | reported | 3440 | 0.1992 | rs114780135 |  |  |  |  |  |  |  |  |  |  |  |  |  |  |  |
| -1.634 | 1 | igd | 0.0036 | C | \|\| id:ebi-a-GCST90001691 | ebi-a-GCST90001691 | TRUE | T | 197674188 | 8.62E-16 | reported | 3440 | 0.2021 | rs114780135 |  |  |  |  |  |  |  |  |  |  |  |  |  |  |  |
| -1.387 | 1 | igd | 0.0036 | T | \|\| id:ebi-a-GCST90001692 | ebi-a-GCST90001692 | TRUE | C | 197574661 | 7.21E-12 | reported | 3440 | 0.2016 | rs145970654 |  |  |  |  |  |  |  |  |  |  |  |  |  |  |  |
| -1.386 | 1 | igd | 0.0036 | C | \|\| id:ebi-a-GCST90001692 | ebi-a-GCST90001692 | TRUE | T | 197687255 | 7.36E-12 | reported | 3440 | 0.2016 | rs115549264 |  |  |  |  |  |  |  |  |  |  |  |  |  |  |  |
| 5.866 | 5 | igd | 0.0036 | G | \|\| id:ebi-a-GCST90001697 | ebi-a-GCST90001697 | TRUE | A | 81918911 | 2.22E-08 | reported | 3425 | 1.046 | rs115348091 |  |  |  |  |  |  |  |  |  |  |  |  |  |  |  |
| 1.525 | 1 | igd | 0.0036 | A | \|\| id:ebi-a-GCST90002098 | ebi-a-GCST90002098 | TRUE | G | 191648600 | 9.25E-13 | reported | 2910 | 0.2126 | rs147071297 |  |  |  |  |  |  |  |  |  |  |  |  |  |  |  |
| 1.33 | 1 | igd | 0.0036 | A | \|\| id:ebi-a-GCST90002098 | ebi-a-GCST90002098 | TRUE | C | 190079170 | 6.19E-09 | reported | 2910 | 0.2281 | rs138915779 |  |  |  |  |  |  |  |  |  |  |  |  |  |  |  |
| 1.375 | 1 | igd | 0.0035 | A | \|\| id:ebi-a-GCST90001485 | ebi-a-GCST90001485 | TRUE | C | 190079170 | 1.20E-09 | reported | 3437 | 0.2256 | rs138915779 |  |  |  |  |  |  |  |  |  |  |  |  |  |  |  |
| 1.302 | 1 | igd | 0.0035 | A | \|\| id:ebi-a-GCST90001508 | ebi-a-GCST90001508 | TRUE | C | 190079170 | 2.82E-08 | reported | 3437 | 0.234 | rs138915779 |  |  |  |  |  |  |  |  |  |  |  |  |  |  |  |
| 147.3 | 1 | igd | 0.0035 | G | \|\| id:ebi-a-GCST90001695 | ebi-a-GCST90001695 | TRUE | T | 102745011 | 3.93E-10 | reported | 3408 | 23.48 | rs76595423 |  |  |  |  |  |  |  |  |  |  |  |  |  |  |  |
| 160.7 | 2 | igd | 0.0035 | G | \|\| id:ebi-a-GCST90001695 | ebi-a-GCST90001695 | TRUE | A | 101458549 | 2.34E-10 | reported | 3408 | 25.29 | rs62156096 |  |  |  |  |  |  |  |  |  |  |  |  |  |  |  |
| 137.7 | 3 | igd | 0.0035 | C | \|\| id:ebi-a-GCST90001695 | ebi-a-GCST90001695 | TRUE | T | 3710729 | 9.64E-10 | reported | 3408 | 22.45 | rs545794900 |  |  |  |  |  |  |  |  |  |  |  |  |  |  |  |
| 132.8 | 7 | igd | 0.0035 | A | \|\| id:ebi-a-GCST90001695 | ebi-a-GCST90001695 | TRUE | C | 79791344 | 1.24E-10 | reported | 3408 | 20.58 | rs117469911 |  |  |  |  |  |  |  |  |  |  |  |  |  |  |  |
| 171.3 | 1 | igd | 0.0035 | C | \|\| id:ebi-a-GCST90001698 | ebi-a-GCST90001698 | TRUE | G | 184790365 | 9.79E-09 | reported | 3408 | 29.8 | rs12129699 |  |  |  |  |  |  |  |  |  |  |  |  |  |  |  |
| 213.4 | 2 | igd | 0.0035 | A | \|\| id:ebi-a-GCST90001698 | ebi-a-GCST90001698 | TRUE | G | 67050839 | 4.65E-12 | reported | 3408 | 30.74 | rs116252790 |  |  |  |  |  |  |  |  |  |  |  |  |  |  |  |
| 158.9 | 2 | igd | 0.0035 | A | \|\| id:ebi-a-GCST90001698 | ebi-a-GCST90001698 | TRUE | G | 118664032 | 3.53E-08 | reported | 3408 | 28.76 | rs35948774 |  |  |  |  |  |  |  |  |  |  |  |  |  |  |  |
| 167.4 | 2 | igd | 0.0035 | G | \|\| id:ebi-a-GCST90001698 | ebi-a-GCST90001698 | TRUE | A | 71772859 | 3.39E-08 | reported | 3408 | 30.26 | rs77937453 |  |  |  |  |  |  |  |  |  |  |  |  |  |  |  |
| 196.5 | 3 | igd | 0.0035 | C | \|\| id:ebi-a-GCST90001698 | ebi-a-GCST90001698 | TRUE | T | 67860216 | 6.06E-11 | reported | 3408 | 29.94 | rs111745963 |  |  |  |  |  |  |  |  |  |  |  |  |  |  |  |
| 272.5 | 7 | igd | 0.0035 | A | \|\| id:ebi-a-GCST90001698 | ebi-a-GCST90001698 | TRUE | C | 79791344 | 1.31E-22 | reported | 3408 | 27.65 | rs117469911 |  |  |  |  |  |  |  |  |  |  |  |  |  |  |  |
| 168.7 | 8 | igd | 0.0035 | A | \|\| id:ebi-a-GCST90001698 | ebi-a-GCST90001698 | TRUE | G | 31416078 | 1.56E-08 | reported | 3408 | 29.76 | rs117807786 |  |  |  |  |  |  |  |  |  |  |  |  |  |  |  |
| 172.5 | 8 | igd | 0.0035 | A | \|\| id:ebi-a-GCST90001698 | ebi-a-GCST90001698 | TRUE | G | 57056704 | 4.95E-09 | reported | 3408 | 29.41 | rs139584477 |  |  |  |  |  |  |  |  |  |  |  |  |  |  |  |
| 192.4 | 10 | igd | 0.0035 | T | \|\| id:ebi-a-GCST90001698 | ebi-a-GCST90001698 | TRUE | C | 52735936 | 1.68E-11 | reported | 3408 | 28.49 | rs2816832 |  |  |  |  |  |  |  |  |  |  |  |  |  |  |  |
| 194.3 | 10 | igd | 0.0035 | A | \|\| id:ebi-a-GCST90001698 | ebi-a-GCST90001698 | TRUE | G | 124406240 | 9.89E-11 | reported | 3408 | 29.94 | rs117003718 |  |  |  |  |  |  |  |  |  |  |  |  |  |  |  |
| 167.3 | 11 | igd | 0.0035 | T | \|\| id:ebi-a-GCST90001698 | ebi-a-GCST90001698 | TRUE | C | 5475505 | 1.37E-08 | reported | 3408 | 29.4 | rs75620804 |  |  |  |  |  |  |  |  |  |  |  |  |  |  |  |
| 175.8 | 15 | igd | 0.0035 | G | \|\| id:ebi-a-GCST90001698 | ebi-a-GCST90001698 | TRUE | A | 51068272 | 2.72E-08 | reported | 3408 | 31.56 | rs34270462 |  |  |  |  |  |  |  |  |  |  |  |  |  |  |  |
| 190.5 | 15 | igd | 0.0035 | T | \|\| id:ebi-a-GCST90001698 | ebi-a-GCST90001698 | TRUE | A | 50131225 | 5.21E-10 | reported | 3408 | 30.58 | rs56265851 |  |  |  |  |  |  |  |  |  |  |  |  |  |  |  |
| -1.578 | 16 | igd | 0.0035 | C | \|\| id:ebi-a-GCST90002028 | ebi-a-GCST90002028 | TRUE | G | 32422808 | 8.42E-10 | reported | 3112 | 0.2564 | rs138920008 |  |  |  |  |  |  |  |  |  |  |  |  |  |  |  |
| -1.512 | 1 | igd | 0.0034 | A | \|\| id:ebi-a-GCST90001510 | ebi-a-GCST90001510 | TRUE | G | 197997953 | 2.05E-12 | reported | 3405 | 0.2142 | rs4497244 |  |  |  |  |  |  |  |  |  |  |  |  |  |  |  |
| -1.344 | 1 | igd | 0.0034 | A | \|\| id:ebi-a-GCST90001534 | ebi-a-GCST90001534 | TRUE | G | 197997953 | 5.04E-10 | reported | 3395 | 0.2155 | rs4497244 |  |  |  |  |  |  |  |  |  |  |  |  |  |  |  |
| -1.438 | 1 | igd | 0.0034 | A | \|\| id:ebi-a-GCST90001535 | ebi-a-GCST90001535 | TRUE | G | 197997953 | 1.94E-13 | reported | 3427 | 0.1949 | rs4497244 |  |  |  |  |  |  |  |  |  |  |  |  |  |  |  |
| -1.351 | 1 | igd | 0.0034 | A | \|\| id:ebi-a-GCST90001536 | ebi-a-GCST90001536 | TRUE | G | 197997953 | 1.02E-11 | reported | 3427 | 0.1979 | rs4497244 |  |  |  |  |  |  |  |  |  |  |  |  |  |  |  |
| 1.669 | 1 | igd | 0.0034 | A | \|\| id:ebi-a-GCST90001666 | ebi-a-GCST90001666 | TRUE | G | 197997953 | 1.83E-19 | reported | 3408 | 0.1839 | rs4497244 |  |  |  |  |  |  |  |  |  |  |  |  |  |  |  |
| -1.785 | 1 | igd | 0.0034 | A | \|\| id:ebi-a-GCST90001669 | ebi-a-GCST90001669 | TRUE | G | 197997953 | 1.87E-21 | reported | 3408 | 0.1864 | rs4497244 |  |  |  |  |  |  |  |  |  |  |  |  |  |  |  |
| -1.228 | 1 | igd | 0.0034 | A | \|\| id:ebi-a-GCST90001669 | ebi-a-GCST90001669 | TRUE | C | 190079170 | 3.74E-09 | reported | 3408 | 0.2078 | rs138915779 |  |  |  |  |  |  |  |  |  |  |  |  |  |  |  |
| -1.867 | 1 | igd | 0.0034 | A | \|\| id:ebi-a-GCST90001693 | ebi-a-GCST90001693 | TRUE | G | 197997953 | 1.18E-18 | reported | 3408 | 0.2106 | rs4497244 |  |  |  |  |  |  |  |  |  |  |  |  |  |  |  |
| 128.8 | 13 | igd | 0.0034 | T | \|\| id:ebi-a-GCST90001695 | ebi-a-GCST90001695 | TRUE | C | 110903174 | 6.54E-09 | reported | 3408 | 22.13 | rs80142250 |  |  |  |  |  |  |  |  |  |  |  |  |  |  |  |
| 214.1 | 1 | igd | 0.0034 | A | \|\| id:ebi-a-GCST90001698 | ebi-a-GCST90001698 | TRUE | G | 195769309 | 3.93E-11 | reported | 3408 | 32.31 | rs146138581 |  |  |  |  |  |  |  |  |  |  |  |  |  |  |  |
| 232.9 | 1 | igd | 0.0034 | T | \|\| id:ebi-a-GCST90001698 | ebi-a-GCST90001698 | TRUE | A | 102669487 | 3.28E-13 | reported | 3408 | 31.86 | rs115470507 |  |  |  |  |  |  |  |  |  |  |  |  |  |  |  |
| 200.7 | 1 | igd | 0.0034 | A | \|\| id:ebi-a-GCST90001698 | ebi-a-GCST90001698 | TRUE | G | 5236853 | 9.35E-10 | reported | 3408 | 32.7 | rs138822361 |  |  |  |  |  |  |  |  |  |  |  |  |  |  |  |
| 188.4 | 2 | igd | 0.0034 | G | \|\| id:ebi-a-GCST90001698 | ebi-a-GCST90001698 | TRUE | T | 40232997 | 3.05E-10 | reported | 3408 | 29.84 | rs72791010 |  |  |  |  |  |  |  |  |  |  |  |  |  |  |  |
| 177.5 | 2 | igd | 0.0034 | C | \|\| id:ebi-a-GCST90001698 | ebi-a-GCST90001698 | TRUE | T | 170436614 | 1.01E-08 | reported | 3408 | 30.9 | rs115517310 |  |  |  |  |  |  |  |  |  |  |  |  |  |  |  |
| 192.7 | 3 | igd | 0.0034 | T | \|\| id:ebi-a-GCST90001698 | ebi-a-GCST90001698 | TRUE | G | 67983792 | 3.32E-10 | reported | 3408 | 30.58 | rs113303971 |  |  |  |  |  |  |  |  |  |  |  |  |  |  |  |
| 165.5 | 3 | igd | 0.0034 | T | \|\| id:ebi-a-GCST90001698 | ebi-a-GCST90001698 | TRUE | G | 155470721 | 4.71E-08 | reported | 3408 | 30.23 | rs74976773 |  |  |  |  |  |  |  |  |  |  |  |  |  |  |  |
| 166.1 | 3 | igd | 0.0034 | A | \|\| id:ebi-a-GCST90001698 | ebi-a-GCST90001698 | TRUE | C | 155625697 | 4.38E-08 | reported | 3408 | 30.26 | rs113855452 |  |  |  |  |  |  |  |  |  |  |  |  |  |  |  |
| 178.7 | 5 | igd | 0.0034 | T | \|\| id:ebi-a-GCST90001698 | ebi-a-GCST90001698 | TRUE | C | 178628264 | 1.29E-09 | reported | 3408 | 29.37 | rs72818624 |  |  |  |  |  |  |  |  |  |  |  |  |  |  |  |
| 178.9 | 7 | igd | 0.0034 | A | \|\| id:ebi-a-GCST90001698 | ebi-a-GCST90001698 | TRUE | G | 57955796 | 1.20E-08 | reported | 3408 | 31.32 | rs535726421 |  |  |  |  |  |  |  |  |  |  |  |  |  |  |  |
| 178.6 | 7 | igd | 0.0034 | A | \|\| id:ebi-a-GCST90001698 | ebi-a-GCST90001698 | TRUE | T | 103411102 | 1.38E-08 | reported | 3408 | 31.4 | rs373800564 |  |  |  |  |  |  |  |  |  |  |  |  |  |  |  |
| 178.6 | 9 | igd | 0.0034 | T | \|\| id:ebi-a-GCST90001698 | ebi-a-GCST90001698 | TRUE | C | 25286345 | 3.02E-09 | reported | 3408 | 30.03 | rs189016063 |  |  |  |  |  |  |  |  |  |  |  |  |  |  |  |
| 215.9 | 12 | igd | 0.0034 | A | \|\| id:ebi-a-GCST90001698 | ebi-a-GCST90001698 | TRUE | G | 77297125 | 1.95E-12 | reported | 3408 | 30.57 | rs73129374 |  |  |  |  |  |  |  |  |  |  |  |  |  |  |  |
| 224.8 | 15 | igd | 0.0034 | T | \|\| id:ebi-a-GCST90001698 | ebi-a-GCST90001698 | TRUE | C | 29421113 | 7.31E-13 | reported | 3408 | 31.22 | rs117801457 |  |  |  |  |  |  |  |  |  |  |  |  |  |  |  |
| 203.4 | 15 | igd | 0.0034 | C | \|\| id:ebi-a-GCST90001698 | ebi-a-GCST90001698 | TRUE | T | 63236912 | 3.06E-10 | reported | 3408 | 32.21 | rs146060900 |  |  |  |  |  |  |  |  |  |  |  |  |  |  |  |
| 197.3 | 20 | igd | 0.0034 | G | \|\| id:ebi-a-GCST90001698 | ebi-a-GCST90001698 | TRUE | T | 15614140 | 2.34E-11 | reported | 3408 | 29.42 | rs459056 |  |  |  |  |  |  |  |  |  |  |  |  |  |  |  |
| 7.455 | 1 | igd | 0.0034 | T | \|\| id:ebi-a-GCST90001700 | ebi-a-GCST90001700 | TRUE | A | 102669487 | 3.09E-08 | reported | 3425 | 1.343 | rs115470507 |  |  |  |  |  |  |  |  |  |  |  |  |  |  |  |
| 7.355 | 1 | igd | 0.0034 | T | \|\| id:ebi-a-GCST90001700 | ebi-a-GCST90001700 | TRUE | C | 102443839 | 4.09E-08 | reported | 3425 | 1.337 | rs80193483 |  |  |  |  |  |  |  |  |  |  |  |  |  |  |  |
| 1.647 | 1 | igd | 0.0034 | T | \|\| id:ebi-a-GCST90002098 | ebi-a-GCST90002098 | TRUE | C | 197574661 | 8.91E-15 | reported | 2910 | 0.2113 | rs145970654 |  |  |  |  |  |  |  |  |  |  |  |  |  |  |  |
| 1.645 | 1 | igd | 0.0034 | C | \|\| id:ebi-a-GCST90002098 | ebi-a-GCST90002098 | TRUE | T | 197687255 | 9.40E-15 | reported | 2910 | 0.2112 | rs115549264 |  |  |  |  |  |  |  |  |  |  |  |  |  |  |  |
| 2.022 | 1 | igd | 0.0033 | A | \|\| id:ebi-a-GCST90001508 | ebi-a-GCST90001508 | TRUE | G | 197997953 | 1.75E-21 | reported | 3437 | 0.211 | rs4497244 |  |  |  |  |  |  |  |  |  |  |  |  |  |  |  |
| -1.396 | 1 | igd | 0.0033 | A | \|\| id:ebi-a-GCST90001512 | ebi-a-GCST90001512 | TRUE | G | 197997953 | 4.86E-11 | reported | 3437 | 0.2116 | rs4497244 |  |  |  |  |  |  |  |  |  |  |  |  |  |  |  |
| 1.942 | 1 | igd | 0.0033 | A | \|\| id:ebi-a-GCST90001664 | ebi-a-GCST90001664 | TRUE | G | 197997953 | 1.28E-23 | reported | 3440 | 0.1925 | rs4497244 |  |  |  |  |  |  |  |  |  |  |  |  |  |  |  |
| 1.972 | 1 | igd | 0.0033 | A | \|\| id:ebi-a-GCST90001665 | ebi-a-GCST90001665 | TRUE | G | 197997953 | 1.47E-21 | reported | 3440 | 0.2054 | rs4497244 |  |  |  |  |  |  |  |  |  |  |  |  |  |  |  |
| -1.779 | 1 | igd | 0.0033 | A | \|\| id:ebi-a-GCST90001667 | ebi-a-GCST90001667 | TRUE | G | 197997953 | 1.58E-19 | reported | 3440 | 0.1957 | rs4497244 |  |  |  |  |  |  |  |  |  |  |  |  |  |  |  |
| -1.763 | 1 | igd | 0.0033 | A | \|\| id:ebi-a-GCST90001668 | ebi-a-GCST90001668 | TRUE | G | 197997953 | 1.47E-17 | reported | 3440 | 0.2055 | rs4497244 |  |  |  |  |  |  |  |  |  |  |  |  |  |  |  |
| -1.816 | 1 | igd | 0.0033 | A | \|\| id:ebi-a-GCST90001692 | ebi-a-GCST90001692 | TRUE | G | 197997953 | 3.85E-18 | reported | 3440 | 0.208 | rs4497244 |  |  |  |  |  |  |  |  |  |  |  |  |  |  |  |
| 2.118 | 1 | igd | 0.0033 | G | \|\| id:ebi-a-GCST90002098 | ebi-a-GCST90002098 | TRUE | A | 198151688 | 1.55E-20 | reported | 2910 | 0.2263 | rs113435341 |  |  |  |  |  |  |  |  |  |  |  |  |  |  |  |
| 2.654 | 1 | igd | 0.0033 | T | \|\| id:ebi-a-GCST90002098 | ebi-a-GCST90002098 | TRUE | G | 198524264 | 2.65E-20 | reported | 2910 | 0.2853 | rs146620586 |  |  |  |  |  |  |  |  |  |  |  |  |  |  |  |
| 2.452 | 1 | igd | 0.0033 | A | \|\| id:ebi-a-GCST90002098 | ebi-a-GCST90002098 | TRUE | G | 198283058 | 1.02E-20 | reported | 2910 | 0.2607 | rs541367304 |  |  |  |  |  |  |  |  |  |  |  |  |  |  |  |
| 1.586 | 1 | igd | 0.0033 | A | \|\| id:ebi-a-GCST90002102 | ebi-a-GCST90002102 | TRUE | C | 190079170 | 4.28E-11 | reported | 2919 | 0.2396 | rs138915779 |  |  |  |  |  |  |  |  |  |  |  |  |  |  |  |
| 1.37 | 1 | igd | 0.0033 | A | \|\| id:ebi-a-GCST90002102 | ebi-a-GCST90002102 | TRUE | G | 191648600 | 8.42E-10 | reported | 2919 | 0.2226 | rs147071297 |  |  |  |  |  |  |  |  |  |  |  |  |  |  |  |
| 2.54 | 1 | igd | 0.0032 | T | \|\| id:ebi-a-GCST90001483 | ebi-a-GCST90001483 | TRUE | G | 198524264 | 1.21E-19 | reported | 3405 | 0.2783 | rs146620586 |  |  |  |  |  |  |  |  |  |  |  |  |  |  |  |
| 2.296 | 1 | igd | 0.0032 | A | \|\| id:ebi-a-GCST90001483 | ebi-a-GCST90001483 | TRUE | G | 198283058 | 2.14E-19 | reported | 3405 | 0.2534 | rs541367304 |  |  |  |  |  |  |  |  |  |  |  |  |  |  |  |
| 2.005 | 1 | igd | 0.0032 | G | \|\| id:ebi-a-GCST90001483 | ebi-a-GCST90001483 | TRUE | A | 198151688 | 7.82E-20 | reported | 3405 | 0.2186 | rs113435341 |  |  |  |  |  |  |  |  |  |  |  |  |  |  |  |
| 1.568 | 1 | igd | 0.0032 | A | \|\| id:ebi-a-GCST90001484 | ebi-a-GCST90001484 | TRUE | G | 198283058 | 7.86E-10 | reported | 3437 | 0.2543 | rs541367304 |  |  |  |  |  |  |  |  |  |  |  |  |  |  |  |
| 1.382 | 1 | igd | 0.0032 | G | \|\| id:ebi-a-GCST90001484 | ebi-a-GCST90001484 | TRUE | A | 198151688 | 3.43E-10 | reported | 3437 | 0.2195 | rs113435341 |  |  |  |  |  |  |  |  |  |  |  |  |  |  |  |
| 1.743 | 1 | igd | 0.0032 | T | \|\| id:ebi-a-GCST90001484 | ebi-a-GCST90001484 | TRUE | G | 198524264 | 4.97E-10 | reported | 3437 | 0.2794 | rs146620586 |  |  |  |  |  |  |  |  |  |  |  |  |  |  |  |
| 2.601 | 1 | igd | 0.0032 | A | \|\| id:ebi-a-GCST90001485 | ebi-a-GCST90001485 | TRUE | G | 198283058 | 1.03E-24 | reported | 3437 | 0.2515 | rs541367304 |  |  |  |  |  |  |  |  |  |  |  |  |  |  |  |
| 2.877 | 1 | igd | 0.0032 | T | \|\| id:ebi-a-GCST90001485 | ebi-a-GCST90001485 | TRUE | G | 198524264 | 5.11E-25 | reported | 3437 | 0.2763 | rs146620586 |  |  |  |  |  |  |  |  |  |  |  |  |  |  |  |
| 2.267 | 1 | igd | 0.0032 | G | \|\| id:ebi-a-GCST90001485 | ebi-a-GCST90001485 | TRUE | A | 198151688 | 3.79E-25 | reported | 3437 | 0.2171 | rs113435341 |  |  |  |  |  |  |  |  |  |  |  |  |  |  |  |
| 1.947 | 1 | igd | 0.0032 | G | \|\| id:ebi-a-GCST90001507 | ebi-a-GCST90001507 | TRUE | A | 198151688 | 2.13E-17 | reported | 3405 | 0.2282 | rs113435341 |  |  |  |  |  |  |  |  |  |  |  |  |  |  |  |
| 2.499 | 1 | igd | 0.0032 | T | \|\| id:ebi-a-GCST90001507 | ebi-a-GCST90001507 | TRUE | G | 198524264 | 1.10E-17 | reported | 3405 | 0.2903 | rs146620586 |  |  |  |  |  |  |  |  |  |  |  |  |  |  |  |
| 2.292 | 1 | igd | 0.0032 | A | \|\| id:ebi-a-GCST90001507 | ebi-a-GCST90001507 | TRUE | G | 198283058 | 6.25E-18 | reported | 3405 | 0.2642 | rs541367304 |  |  |  |  |  |  |  |  |  |  |  |  |  |  |  |
| 2.618 | 1 | igd | 0.0032 | A | \|\| id:ebi-a-GCST90001508 | ebi-a-GCST90001508 | TRUE | G | 198283058 | 2.97E-23 | reported | 3437 | 0.2616 | rs541367304 |  |  |  |  |  |  |  |  |  |  |  |  |  |  |  |
| 2.84 | 1 | igd | 0.0032 | T | \|\| id:ebi-a-GCST90001508 | ebi-a-GCST90001508 | TRUE | G | 198524264 | 1.03E-22 | reported | 3437 | 0.2875 | rs146620586 |  |  |  |  |  |  |  |  |  |  |  |  |  |  |  |
| 2.227 | 1 | igd | 0.0032 | G | \|\| id:ebi-a-GCST90001508 | ebi-a-GCST90001508 | TRUE | A | 198151688 | 1.36E-22 | reported | 3437 | 0.2261 | rs113435341 |  |  |  |  |  |  |  |  |  |  |  |  |  |  |  |
| 2.689 | 1 | igd | 0.0032 | T | \|\| id:ebi-a-GCST90001509 | ebi-a-GCST90001509 | TRUE | G | 198524264 | 1.61E-20 | reported | 3437 | 0.2877 | rs146620586 |  |  |  |  |  |  |  |  |  |  |  |  |  |  |  |
| 2.485 | 1 | igd | 0.0032 | A | \|\| id:ebi-a-GCST90001509 | ebi-a-GCST90001509 | TRUE | G | 198283058 | 4.06E-21 | reported | 3437 | 0.2618 | rs541367304 |  |  |  |  |  |  |  |  |  |  |  |  |  |  |  |
| 2.115 | 1 | igd | 0.0032 | G | \|\| id:ebi-a-GCST90001509 | ebi-a-GCST90001509 | TRUE | A | 198151688 | 1.58E-20 | reported | 3437 | 0.2263 | rs113435341 |  |  |  |  |  |  |  |  |  |  |  |  |  |  |  |
| -1.875 | 1 | igd | 0.0032 | A | \|\| id:ebi-a-GCST90001510 | ebi-a-GCST90001510 | TRUE | G | 198283058 | 2.11E-12 | reported | 3405 | 0.2658 | rs541367304 |  |  |  |  |  |  |  |  |  |  |  |  |  |  |  |
| -2.081 | 1 | igd | 0.0032 | T | \|\| id:ebi-a-GCST90001510 | ebi-a-GCST90001510 | TRUE | G | 198524264 | 1.24E-12 | reported | 3405 | 0.292 | rs146620586 |  |  |  |  |  |  |  |  |  |  |  |  |  |  |  |
| -1.681 | 1 | igd | 0.0032 | G | \|\| id:ebi-a-GCST90001510 | ebi-a-GCST90001510 | TRUE | A | 198151688 | 3.03E-13 | reported | 3405 | 0.2295 | rs113435341 |  |  |  |  |  |  |  |  |  |  |  |  |  |  |  |
| -1.786 | 1 | igd | 0.0032 | A | \|\| id:ebi-a-GCST90001511 | ebi-a-GCST90001511 | TRUE | G | 198283058 | 1.23E-11 | reported | 3437 | 0.2626 | rs541367304 |  |  |  |  |  |  |  |  |  |  |  |  |  |  |  |
| -1.621 | 1 | igd | 0.0032 | G | \|\| id:ebi-a-GCST90001511 | ebi-a-GCST90001511 | TRUE | A | 198151688 | 1.08E-12 | reported | 3437 | 0.2268 | rs113435341 |  |  |  |  |  |  |  |  |  |  |  |  |  |  |  |
| -2.02 | 1 | igd | 0.0032 | T | \|\| id:ebi-a-GCST90001511 | ebi-a-GCST90001511 | TRUE | G | 198524264 | 2.97E-12 | reported | 3437 | 0.2884 | rs146620586 |  |  |  |  |  |  |  |  |  |  |  |  |  |  |  |
| -1.883 | 1 | igd | 0.0032 | T | \|\| id:ebi-a-GCST90001512 | ebi-a-GCST90001512 | TRUE | G | 198524264 | 7.75E-11 | reported | 3437 | 0.2885 | rs146620586 |  |  |  |  |  |  |  |  |  |  |  |  |  |  |  |
| -1.492 | 1 | igd | 0.0032 | G | \|\| id:ebi-a-GCST90001512 | ebi-a-GCST90001512 | TRUE | A | 198151688 | 5.58E-11 | reported | 3437 | 0.2269 | rs113435341 |  |  |  |  |  |  |  |  |  |  |  |  |  |  |  |
| -1.652 | 1 | igd | 0.0032 | A | \|\| id:ebi-a-GCST90001512 | ebi-a-GCST90001512 | TRUE | G | 198283058 | 3.61E-10 | reported | 3437 | 0.2627 | rs541367304 |  |  |  |  |  |  |  |  |  |  |  |  |  |  |  |
| -1.575 | 1 | igd | 0.0032 | G | \|\| id:ebi-a-GCST90001534 | ebi-a-GCST90001534 | TRUE | A | 198151688 | 1.00E-11 | reported | 3395 | 0.2306 | rs113435341 |  |  |  |  |  |  |  |  |  |  |  |  |  |  |  |
| -2.006 | 1 | igd | 0.0032 | T | \|\| id:ebi-a-GCST90001534 | ebi-a-GCST90001534 | TRUE | G | 198524264 | 9.58E-12 | reported | 3395 | 0.2934 | rs146620586 |  |  |  |  |  |  |  |  |  |  |  |  |  |  |  |
| -1.857 | 1 | igd | 0.0032 | A | \|\| id:ebi-a-GCST90001534 | ebi-a-GCST90001534 | TRUE | G | 198283058 | 4.24E-12 | reported | 3395 | 0.267 | rs541367304 |  |  |  |  |  |  |  |  |  |  |  |  |  |  |  |
| -1.568 | 1 | igd | 0.0032 | G | \|\| id:ebi-a-GCST90001535 | ebi-a-GCST90001535 | TRUE | A | 198151688 | 7.25E-14 | reported | 3427 | 0.2086 | rs113435341 |  |  |  |  |  |  |  |  |  |  |  |  |  |  |  |
| -2.021 | 1 | igd | 0.0032 | T | \|\| id:ebi-a-GCST90001535 | ebi-a-GCST90001535 | TRUE | G | 198524264 | 3.46E-14 | reported | 3427 | 0.2655 | rs146620586 |  |  |  |  |  |  |  |  |  |  |  |  |  |  |  |
| -1.837 | 1 | igd | 0.0032 | A | \|\| id:ebi-a-GCST90001535 | ebi-a-GCST90001535 | TRUE | G | 198283058 | 3.71E-14 | reported | 3427 | 0.2416 | rs541367304 |  |  |  |  |  |  |  |  |  |  |  |  |  |  |  |
| -1.788 | 1 | igd | 0.0032 | A | \|\| id:ebi-a-GCST90001536 | ebi-a-GCST90001536 | TRUE | G | 198283058 | 3.67E-13 | reported | 3427 | 0.2451 | rs541367304 |  |  |  |  |  |  |  |  |  |  |  |  |  |  |  |
| -1.508 | 1 | igd | 0.0032 | G | \|\| id:ebi-a-GCST90001536 | ebi-a-GCST90001536 | TRUE | A | 198151688 | 1.28E-12 | reported | 3427 | 0.2116 | rs113435341 |  |  |  |  |  |  |  |  |  |  |  |  |  |  |  |
| -1.991 | 1 | igd | 0.0032 | T | \|\| id:ebi-a-GCST90001536 | ebi-a-GCST90001536 | TRUE | G | 198524264 | 1.78E-13 | reported | 3427 | 0.2693 | rs146620586 |  |  |  |  |  |  |  |  |  |  |  |  |  |  |  |
| 1.867 | 1 | igd | 0.0032 | A | \|\| id:ebi-a-GCST90001561 | ebi-a-GCST90001561 | TRUE | G | 198283058 | 4.52E-17 | reported | 3427 | 0.2212 | rs541367304 |  |  |  |  |  |  |  |  |  |  |  |  |  |  |  |
| 1.61 | 1 | igd | 0.0032 | G | \|\| id:ebi-a-GCST90001561 | ebi-a-GCST90001561 | TRUE | A | 198151688 | 5.21E-17 | reported | 3427 | 0.1911 | rs113435341 |  |  |  |  |  |  |  |  |  |  |  |  |  |  |  |
| 2.034 | 1 | igd | 0.0032 | T | \|\| id:ebi-a-GCST90001561 | ebi-a-GCST90001561 | TRUE | G | 198524264 | 8.26E-17 | reported | 3427 | 0.243 | rs146620586 |  |  |  |  |  |  |  |  |  |  |  |  |  |  |  |
| 1.312 | 1 | igd | 0.0032 | A | \|\| id:ebi-a-GCST90001562 | ebi-a-GCST90001562 | TRUE | G | 198283058 | 1.36E-08 | reported | 3427 | 0.2306 | rs541367304 |  |  |  |  |  |  |  |  |  |  |  |  |  |  |  |
| 1.455 | 1 | igd | 0.0032 | T | \|\| id:ebi-a-GCST90001562 | ebi-a-GCST90001562 | TRUE | G | 198524264 | 1.00E-08 | reported | 3427 | 0.2533 | rs146620586 |  |  |  |  |  |  |  |  |  |  |  |  |  |  |  |
| 1.115 | 1 | igd | 0.0032 | G | \|\| id:ebi-a-GCST90001562 | ebi-a-GCST90001562 | TRUE | A | 198151688 | 2.40E-08 | reported | 3427 | 0.1993 | rs113435341 |  |  |  |  |  |  |  |  |  |  |  |  |  |  |  |
| 2.157 | 1 | igd | 0.0032 | G | \|\| id:ebi-a-GCST90001664 | ebi-a-GCST90001664 | TRUE | A | 198151688 | 2.96E-25 | reported | 3440 | 0.2061 | rs113435341 |  |  |  |  |  |  |  |  |  |  |  |  |  |  |  |
| 2.523 | 1 | igd | 0.0032 | A | \|\| id:ebi-a-GCST90001664 | ebi-a-GCST90001664 | TRUE | G | 198283058 | 9.14E-26 | reported | 3440 | 0.2385 | rs541367304 |  |  |  |  |  |  |  |  |  |  |  |  |  |  |  |
| 2.73 | 1 | igd | 0.0032 | T | \|\| id:ebi-a-GCST90001664 | ebi-a-GCST90001664 | TRUE | G | 198524264 | 5.12E-25 | reported | 3440 | 0.2622 | rs146620586 |  |  |  |  |  |  |  |  |  |  |  |  |  |  |  |
| 2.776 | 1 | igd | 0.0032 | T | \|\| id:ebi-a-GCST90001665 | ebi-a-GCST90001665 | TRUE | G | 198524264 | 6.75E-23 | reported | 3440 | 0.2798 | rs146620586 |  |  |  |  |  |  |  |  |  |  |  |  |  |  |  |
| 2.175 | 1 | igd | 0.0032 | G | \|\| id:ebi-a-GCST90001665 | ebi-a-GCST90001665 | TRUE | A | 198151688 | 1.03E-22 | reported | 3440 | 0.2201 | rs113435341 |  |  |  |  |  |  |  |  |  |  |  |  |  |  |  |
| 2.559 | 1 | igd | 0.0032 | A | \|\| id:ebi-a-GCST90001665 | ebi-a-GCST90001665 | TRUE | G | 198283058 | 1.96E-23 | reported | 3440 | 0.2546 | rs541367304 |  |  |  |  |  |  |  |  |  |  |  |  |  |  |  |
| 1.818 | 1 | igd | 0.0032 | G | \|\| id:ebi-a-GCST90001666 | ebi-a-GCST90001666 | TRUE | A | 198151688 | 4.99E-20 | reported | 3408 | 0.1972 | rs113435341 |  |  |  |  |  |  |  |  |  |  |  |  |  |  |  |
| 2.334 | 1 | igd | 0.0032 | T | \|\| id:ebi-a-GCST90001666 | ebi-a-GCST90001666 | TRUE | G | 198524264 | 2.12E-20 | reported | 3408 | 0.2506 | rs146620586 |  |  |  |  |  |  |  |  |  |  |  |  |  |  |  |
| 2.141 | 1 | igd | 0.0032 | A | \|\| id:ebi-a-GCST90001666 | ebi-a-GCST90001666 | TRUE | G | 198283058 | 1.10E-20 | reported | 3408 | 0.2281 | rs541367304 |  |  |  |  |  |  |  |  |  |  |  |  |  |  |  |
| -1.901 | 1 | igd | 0.0032 | G | \|\| id:ebi-a-GCST90001667 | ebi-a-GCST90001667 | TRUE | A | 198151688 | 2.12E-19 | reported | 3440 | 0.2098 | rs113435341 |  |  |  |  |  |  |  |  |  |  |  |  |  |  |  |
| -2.445 | 1 | igd | 0.0032 | T | \|\| id:ebi-a-GCST90001667 | ebi-a-GCST90001667 | TRUE | G | 198524264 | 8.19E-20 | reported | 3440 | 0.2667 | rs146620586 |  |  |  |  |  |  |  |  |  |  |  |  |  |  |  |
| -2.266 | 1 | igd | 0.0032 | A | \|\| id:ebi-a-GCST90001667 | ebi-a-GCST90001667 | TRUE | G | 198283058 | 1.74E-20 | reported | 3440 | 0.2427 | rs541367304 |  |  |  |  |  |  |  |  |  |  |  |  |  |  |  |
| -2.245 | 1 | igd | 0.0032 | A | \|\| id:ebi-a-GCST90001668 | ebi-a-GCST90001668 | TRUE | G | 198283058 | 2.09E-18 | reported | 3440 | 0.255 | rs541367304 |  |  |  |  |  |  |  |  |  |  |  |  |  |  |  |
| -1.884 | 1 | igd | 0.0032 | G | \|\| id:ebi-a-GCST90001668 | ebi-a-GCST90001668 | TRUE | A | 198151688 | 1.94E-17 | reported | 3440 | 0.2205 | rs113435341 |  |  |  |  |  |  |  |  |  |  |  |  |  |  |  |
| -2.425 | 1 | igd | 0.0032 | T | \|\| id:ebi-a-GCST90001668 | ebi-a-GCST90001668 | TRUE | G | 198524264 | 7.44E-18 | reported | 3440 | 0.2802 | rs146620586 |  |  |  |  |  |  |  |  |  |  |  |  |  |  |  |
| -1.945 | 1 | igd | 0.0032 | G | \|\| id:ebi-a-GCST90001669 | ebi-a-GCST90001669 | TRUE | A | 198151688 | 3.97E-22 | reported | 3408 | 0.1997 | rs113435341 |  |  |  |  |  |  |  |  |  |  |  |  |  |  |  |
| -2.476 | 1 | igd | 0.0032 | T | \|\| id:ebi-a-GCST90001669 | ebi-a-GCST90001669 | TRUE | G | 198524264 | 3.56E-22 | reported | 3408 | 0.254 | rs146620586 |  |  |  |  |  |  |  |  |  |  |  |  |  |  |  |
| -2.319 | 1 | igd | 0.0032 | A | \|\| id:ebi-a-GCST90001669 | ebi-a-GCST90001669 | TRUE | G | 198283058 | 2.18E-23 | reported | 3408 | 0.231 | rs541367304 |  |  |  |  |  |  |  |  |  |  |  |  |  |  |  |
| -2.427 | 1 | igd | 0.0032 | A | \|\| id:ebi-a-GCST90001691 | ebi-a-GCST90001691 | TRUE | G | 198283058 | 1.55E-20 | reported | 3440 | 0.2596 | rs541367304 |  |  |  |  |  |  |  |  |  |  |  |  |  |  |  |
| -2.085 | 1 | igd | 0.0032 | G | \|\| id:ebi-a-GCST90001691 | ebi-a-GCST90001691 | TRUE | A | 198151688 | 2.48E-20 | reported | 3440 | 0.2242 | rs113435341 |  |  |  |  |  |  |  |  |  |  |  |  |  |  |  |
| -2.647 | 1 | igd | 0.0032 | T | \|\| id:ebi-a-GCST90001691 | ebi-a-GCST90001691 | TRUE | G | 198524264 | 2.92E-20 | reported | 3440 | 0.2853 | rs146620586 |  |  |  |  |  |  |  |  |  |  |  |  |  |  |  |
| -2.596 | 1 | igd | 0.0032 | T | \|\| id:ebi-a-GCST90001692 | ebi-a-GCST90001692 | TRUE | G | 198524264 | 8.26E-20 | reported | 3440 | 0.2832 | rs146620586 |  |  |  |  |  |  |  |  |  |  |  |  |  |  |  |
| -2.361 | 1 | igd | 0.0032 | A | \|\| id:ebi-a-GCST90001692 | ebi-a-GCST90001692 | TRUE | G | 198283058 | 9.02E-20 | reported | 3440 | 0.2579 | rs541367304 |  |  |  |  |  |  |  |  |  |  |  |  |  |  |  |
| -2.011 | 1 | igd | 0.0032 | G | \|\| id:ebi-a-GCST90001692 | ebi-a-GCST90001692 | TRUE | A | 198151688 | 2.98E-19 | reported | 3440 | 0.2229 | rs113435341 |  |  |  |  |  |  |  |  |  |  |  |  |  |  |  |
| -2.048 | 1 | igd | 0.0032 | G | \|\| id:ebi-a-GCST90001693 | ebi-a-GCST90001693 | TRUE | A | 198151688 | 1.83E-19 | reported | 3408 | 0.2256 | rs113435341 |  |  |  |  |  |  |  |  |  |  |  |  |  |  |  |
| -2.393 | 1 | igd | 0.0032 | A | \|\| id:ebi-a-GCST90001693 | ebi-a-GCST90001693 | TRUE | G | 198283058 | 8.39E-20 | reported | 3408 | 0.2611 | rs541367304 |  |  |  |  |  |  |  |  |  |  |  |  |  |  |  |
| -2.585 | 1 | igd | 0.0032 | T | \|\| id:ebi-a-GCST90001693 | ebi-a-GCST90001693 | TRUE | G | 198524264 | 3.36E-19 | reported | 3408 | 0.2869 | rs146620586 |  |  |  |  |  |  |  |  |  |  |  |  |  |  |  |
| 130.9 | 4 | igd | 0.0032 | A | \|\| id:ebi-a-GCST90001695 | ebi-a-GCST90001695 | TRUE | G | 188776830 | 2.90E-08 | reported | 3408 | 23.54 | rs72714668 |  |  |  |  |  |  |  |  |  |  |  |  |  |  |  |
| 154.5 | 4 | igd | 0.0032 | G | \|\| id:ebi-a-GCST90001695 | ebi-a-GCST90001695 | TRUE | A | 187981624 | 8.23E-12 | reported | 3408 | 22.53 | rs76506268 |  |  |  |  |  |  |  |  |  |  |  |  |  |  |  |
| 6.353 | 4 | igd | 0.0032 | G | \|\| id:ebi-a-GCST90001697 | ebi-a-GCST90001697 | TRUE | A | 187981624 | 1.52E-09 | reported | 3425 | 1.049 | rs76506268 |  |  |  |  |  |  |  |  |  |  |  |  |  |  |  |
| 252.6 | 1 | igd | 0.0032 | C | \|\| id:ebi-a-GCST90001698 | ebi-a-GCST90001698 | TRUE | T | 99407037 | 5.74E-15 | reported | 3408 | 32.2 | rs150073927 |  |  |  |  |  |  |  |  |  |  |  |  |  |  |  |
| 177.1 | 2 | igd | 0.0032 | T | \|\| id:ebi-a-GCST90001698 | ebi-a-GCST90001698 | TRUE | C | 84361833 | 1.19E-08 | reported | 3408 | 30.98 | rs138103139 |  |  |  |  |  |  |  |  |  |  |  |  |  |  |  |
| 196.1 | 2 | igd | 0.0032 | G | \|\| id:ebi-a-GCST90001698 | ebi-a-GCST90001698 | TRUE | C | 104557240 | 4.22E-09 | reported | 3408 | 33.29 | rs145503403 |  |  |  |  |  |  |  |  |  |  |  |  |  |  |  |
| 255.1 | 3 | igd | 0.0032 | A | \|\| id:ebi-a-GCST90001698 | ebi-a-GCST90001698 | TRUE | G | 64390467 | 1.40E-17 | reported | 3408 | 29.73 | rs62247421 |  |  |  |  |  |  |  |  |  |  |  |  |  |  |  |
| 181.2 | 4 | igd | 0.0032 | T | \|\| id:ebi-a-GCST90001698 | ebi-a-GCST90001698 | TRUE | C | 100588891 | 4.82E-08 | reported | 3408 | 33.13 | rs147026949 |  |  |  |  |  |  |  |  |  |  |  |  |  |  |  |
| 184.2 | 4 | igd | 0.0032 | T | \|\| id:ebi-a-GCST90001698 | ebi-a-GCST90001698 | TRUE | C | 176991524 | 9.60E-10 | reported | 3408 | 30.04 | rs113388342 |  |  |  |  |  |  |  |  |  |  |  |  |  |  |  |
| 175.4 | 6 | igd | 0.0032 | T | \|\| id:ebi-a-GCST90001698 | ebi-a-GCST90001698 | TRUE | C | 167014418 | 2.59E-08 | reported | 3408 | 31.44 | rs189131343 |  |  |  |  |  |  |  |  |  |  |  |  |  |  |  |
| 184.9 | 7 | igd | 0.0032 | G | \|\| id:ebi-a-GCST90001698 | ebi-a-GCST90001698 | TRUE | T | 131413341 | 2.77E-09 | reported | 3408 | 31.03 | rs74842782 |  |  |  |  |  |  |  |  |  |  |  |  |  |  |  |
| 181.1 | 7 | igd | 0.0032 | T | \|\| id:ebi-a-GCST90001698 | ebi-a-GCST90001698 | TRUE | C | 83368658 | 5.04E-09 | reported | 3408 | 30.9 | rs184401135 |  |  |  |  |  |  |  |  |  |  |  |  |  |  |  |
| 165.8 | 7 | igd | 0.0032 | C | \|\| id:ebi-a-GCST90001698 | ebi-a-GCST90001698 | TRUE | T | 83674280 | 3.11E-08 | reported | 3408 | 29.88 | rs112368105 |  |  |  |  |  |  |  |  |  |  |  |  |  |  |  |
| 180.4 | 8 | igd | 0.0032 | A | \|\| id:ebi-a-GCST90001698 | ebi-a-GCST90001698 | TRUE | G | 31315428 | 5.91E-09 | reported | 3408 | 30.92 | rs117127715 |  |  |  |  |  |  |  |  |  |  |  |  |  |  |  |
| 204.4 | 10 | igd | 0.0032 | A | \|\| id:ebi-a-GCST90001698 | ebi-a-GCST90001698 | TRUE | G | 1765793 | 1.58E-08 | reported | 3408 | 36.08 | rs140322068 |  |  |  |  |  |  |  |  |  |  |  |  |  |  |  |
| 167 | 10 | igd | 0.0032 | T | \|\| id:ebi-a-GCST90001698 | ebi-a-GCST90001698 | TRUE | C | 1729255 | 4.24E-08 | reported | 3408 | 30.41 | rs140167443 |  |  |  |  |  |  |  |  |  |  |  |  |  |  |  |
| 186 | 11 | igd | 0.0032 | C | \|\| id:ebi-a-GCST90001698 | ebi-a-GCST90001698 | TRUE | A | 60694375 | 2.73E-09 | reported | 3408 | 31.2 | rs117270364 |  |  |  |  |  |  |  |  |  |  |  |  |  |  |  |
| 231.7 | 12 | igd | 0.0032 | A | \|\| id:ebi-a-GCST90001698 | ebi-a-GCST90001698 | TRUE | G | 78656357 | 1.84E-12 | reported | 3408 | 32.76 | rs142055441 |  |  |  |  |  |  |  |  |  |  |  |  |  |  |  |
| 246 | 15 | igd | 0.0032 | A | \|\| id:ebi-a-GCST90001698 | ebi-a-GCST90001698 | TRUE | G | 29114742 | 1.13E-11 | reported | 3408 | 36.11 | rs188087675 |  |  |  |  |  |  |  |  |  |  |  |  |  |  |  |
| 185.9 | 15 | igd | 0.0032 | T | \|\| id:ebi-a-GCST90001698 | ebi-a-GCST90001698 | TRUE | C | 51406779 | 8.71E-09 | reported | 3408 | 32.23 | rs12917273 |  |  |  |  |  |  |  |  |  |  |  |  |  |  |  |
| 203.7 | 15 | igd | 0.0032 | C | \|\| id:ebi-a-GCST90001698 | ebi-a-GCST90001698 | TRUE | G | 50008133 | 1.41E-10 | reported | 3408 | 31.66 | rs56041702 |  |  |  |  |  |  |  |  |  |  |  |  |  |  |  |
| 180.4 | 18 | igd | 0.0032 | T | \|\| id:ebi-a-GCST90001698 | ebi-a-GCST90001698 | TRUE | C | 59422486 | 3.47E-08 | reported | 3408 | 32.64 | rs59985987 |  |  |  |  |  |  |  |  |  |  |  |  |  |  |  |
| 201.7 | 20 | igd | 0.0032 | G | \|\| id:ebi-a-GCST90001698 | ebi-a-GCST90001698 | TRUE | C | 37819906 | 4.77E-10 | reported | 3408 | 32.3 | rs142680317 |  |  |  |  |  |  |  |  |  |  |  |  |  |  |  |
| 172.1 | 21 | igd | 0.0032 | T | \|\| id:ebi-a-GCST90001698 | ebi-a-GCST90001698 | TRUE | C | 27289871 | 4.77E-08 | reported | 3408 | 31.46 | rs117922572 |  |  |  |  |  |  |  |  |  |  |  |  |  |  |  |
| 1.724 | 1 | igd | 0.0032 | A | \|\| id:ebi-a-GCST90002103 | ebi-a-GCST90002103 | TRUE | G | 198283058 | 5.44E-09 | reported | 2650 | 0.2946 | rs541367304 |  |  |  |  |  |  |  |  |  |  |  |  |  |  |  |
| 1.829 | 1 | igd | 0.0032 | T | \|\| id:ebi-a-GCST90002103 | ebi-a-GCST90002103 | TRUE | G | 198524264 | 1.34E-08 | reported | 2650 | 0.321 | rs146620586 |  |  |  |  |  |  |  |  |  |  |  |  |  |  |  |
| 1.461 | 1 | igd | 0.0032 | G | \|\| id:ebi-a-GCST90002103 | ebi-a-GCST90002103 | TRUE | A | 198151688 | 1.20E-08 | reported | 2650 | 0.2555 | rs113435341 |  |  |  |  |  |  |  |  |  |  |  |  |  |  |  |
| 141.9 | 14 | igd | 0.0031 | C | \|\| id:ebi-a-GCST90001695 | ebi-a-GCST90001695 | TRUE | T | 68224381 | 1.88E-09 | reported | 3408 | 23.56 | rs529047505 |  |  |  |  |  |  |  |  |  |  |  |  |  |  |  |
| 177.2 | 1 | igd | 0.0031 | C | \|\| id:ebi-a-GCST90001698 | ebi-a-GCST90001698 | TRUE | G | 193430502 | 3.21E-08 | reported | 3408 | 31.97 | rs145647597 |  |  |  |  |  |  |  |  |  |  |  |  |  |  |  |
| 194.8 | 3 | igd | 0.0031 | A | \|\| id:ebi-a-GCST90001698 | ebi-a-GCST90001698 | TRUE | T | 77131697 | 1.02E-09 | reported | 3408 | 31.82 | rs112461654 |  |  |  |  |  |  |  |  |  |  |  |  |  |  |  |
| 193.7 | 6 | igd | 0.0031 | G | \|\| id:ebi-a-GCST90001698 | ebi-a-GCST90001698 | TRUE | A | 151992984 | 3.18E-09 | reported | 3408 | 32.62 | rs9479093 |  |  |  |  |  |  |  |  |  |  |  |  |  |  |  |
| 196.3 | 6 | igd | 0.0031 | G | \|\| id:ebi-a-GCST90001698 | ebi-a-GCST90001698 | TRUE | A | 64775927 | 4.51E-09 | reported | 3408 | 33.38 | rs79635150 |  |  |  |  |  |  |  |  |  |  |  |  |  |  |  |
| 185.9 | 9 | igd | 0.0031 | G | \|\| id:ebi-a-GCST90001698 | ebi-a-GCST90001698 | TRUE | A | 106801353 | 2.28E-08 | reported | 3408 | 33.18 | rs145563511 |  |  |  |  |  |  |  |  |  |  |  |  |  |  |  |
| 186.8 | 9 | igd | 0.0031 | A | \|\| id:ebi-a-GCST90001698 | ebi-a-GCST90001698 | TRUE | C | 85888772 | 9.47E-09 | reported | 3408 | 32.47 | rs148534514 |  |  |  |  |  |  |  |  |  |  |  |  |  |  |  |
| 216.9 | 12 | igd | 0.0031 | G | \|\| id:ebi-a-GCST90001698 | ebi-a-GCST90001698 | TRUE | A | 129773605 | 2.49E-12 | reported | 3408 | 30.85 | rs11060236 |  |  |  |  |  |  |  |  |  |  |  |  |  |  |  |
| 202.4 | 13 | igd | 0.0031 | C | \|\| id:ebi-a-GCST90001698 | ebi-a-GCST90001698 | TRUE | T | 43404037 | 7.31E-10 | reported | 3408 | 32.76 | rs145720280 |  |  |  |  |  |  |  |  |  |  |  |  |  |  |  |
| 179 | 20 | igd | 0.0031 | C | \|\| id:ebi-a-GCST90001698 | ebi-a-GCST90001698 | TRUE | G | 33236696 | 2.75E-08 | reported | 3408 | 32.14 | rs56238684 |  |  |  |  |  |  |  |  |  |  |  |  |  |  |  |
| 214.6 | 20 | igd | 0.0031 | T | \|\| id:ebi-a-GCST90001698 | ebi-a-GCST90001698 | TRUE | C | 43743769 | 7.27E-11 | reported | 3408 | 32.83 | rs73910118 |  |  |  |  |  |  |  |  |  |  |  |  |  |  |  |
| 1.334 | 1 | igd | 0.0031 | A | \|\| id:ebi-a-GCST90001941 | ebi-a-GCST90001941 | TRUE | G | 197997953 | 2.60E-09 | reported | 3435 | 0.2234 | rs4497244 |  |  |  |  |  |  |  |  |  |  |  |  |  |  |  |
| 1.226 | 1 | igd | 0.0031 | A | \|\| id:ebi-a-GCST90001942 | ebi-a-GCST90001942 | TRUE | G | 197997953 | 4.03E-08 | reported | 3435 | 0.2228 | rs4497244 |  |  |  |  |  |  |  |  |  |  |  |  |  |  |  |
| 1.832 | 1 | igd | 0.0031 | C | \|\| id:ebi-a-GCST90002102 | ebi-a-GCST90002102 | TRUE | T | 197687255 | 1.82E-16 | reported | 2919 | 0.2212 | rs115549264 |  |  |  |  |  |  |  |  |  |  |  |  |  |  |  |
| 1.834 | 1 | igd | 0.0031 | T | \|\| id:ebi-a-GCST90002102 | ebi-a-GCST90002102 | TRUE | C | 197574661 | 1.72E-16 | reported | 2919 | 0.2213 | rs145970654 |  |  |  |  |  |  |  |  |  |  |  |  |  |  |  |
| 2.134 | 1 | igd | 0.0029 | A | \|\| id:ebi-a-GCST90001483 | ebi-a-GCST90001483 | TRUE | G | 197924770 | 2.17E-21 | reported | 3405 | 0.2233 | rs17583875 |  |  |  |  |  |  |  |  |  |  |  |  |  |  |  |
| 2.289 | 1 | igd | 0.0029 | A | \|\| id:ebi-a-GCST90001485 | ebi-a-GCST90001485 | TRUE | G | 197924770 | 1.33E-24 | reported | 3437 | 0.2218 | rs17583875 |  |  |  |  |  |  |  |  |  |  |  |  |  |  |  |
| 2.038 | 1 | igd | 0.0029 | A | \|\| id:ebi-a-GCST90001507 | ebi-a-GCST90001507 | TRUE | G | 197924770 | 3.37E-18 | reported | 3405 | 0.233 | rs17583875 |  |  |  |  |  |  |  |  |  |  |  |  |  |  |  |
| 2.133 | 1 | igd | 0.0029 | A | \|\| id:ebi-a-GCST90001509 | ebi-a-GCST90001509 | TRUE | G | 197924770 | 4.10E-20 | reported | 3437 | 0.2308 | rs17583875 |  |  |  |  |  |  |  |  |  |  |  |  |  |  |  |
| -1.737 | 1 | igd | 0.0029 | A | \|\| id:ebi-a-GCST90001511 | ebi-a-GCST90001511 | TRUE | G | 197924770 | 7.08E-14 | reported | 3437 | 0.2311 | rs17583875 |  |  |  |  |  |  |  |  |  |  |  |  |  |  |  |
| 1.755 | 1 | igd | 0.0029 | A | \|\| id:ebi-a-GCST90001561 | ebi-a-GCST90001561 | TRUE | G | 197924770 | 3.06E-19 | reported | 3427 | 0.1946 | rs17583875 |  |  |  |  |  |  |  |  |  |  |  |  |  |  |  |
| 1.27 | 10 | igd | 0.0029 | T | \|\| id:ebi-a-GCST90001670 | ebi-a-GCST90001670 | TRUE | C | 98816434 | 2.14E-08 | reported | 3440 | 0.2262 | rs117648214 |  |  |  |  |  |  |  |  |  |  |  |  |  |  |  |
| 1.245 | 10 | igd | 0.0029 | T | \|\| id:ebi-a-GCST90001671 | ebi-a-GCST90001671 | TRUE | C | 98816434 | 3.85E-08 | reported | 3440 | 0.226 | rs117648214 |  |  |  |  |  |  |  |  |  |  |  |  |  |  |  |
| 0.9979 | 1 | igd | 0.0029 | A | \|\| id:ebi-a-GCST90001689 | ebi-a-GCST90001689 | TRUE | G | 197924770 | 8.82E-09 | reported | 3440 | 0.1731 | rs17583875 |  |  |  |  |  |  |  |  |  |  |  |  |  |  |  |
| -2.154 | 1 | igd | 0.0029 | A | \|\| id:ebi-a-GCST90001691 | ebi-a-GCST90001691 | TRUE | G | 197924770 | 8.60E-21 | reported | 3440 | 0.2288 | rs17583875 |  |  |  |  |  |  |  |  |  |  |  |  |  |  |  |
| 141.3 | 4 | igd | 0.0029 | T | \|\| id:ebi-a-GCST90001695 | ebi-a-GCST90001695 | TRUE | G | 187962855 | 9.49E-09 | reported | 3408 | 24.56 | rs77239996 |  |  |  |  |  |  |  |  |  |  |  |  |  |  |  |
| 14.88 | 14 | igd | 0.0029 | T | \|\| id:ebi-a-GCST90001696 | ebi-a-GCST90001696 | TRUE | C | 68217724 | 3.49E-08 | reported | 3425 | 2.692 | rs742867 |  |  |  |  |  |  |  |  |  |  |  |  |  |  |  |
| 7.048 | 14 | igd | 0.0029 | T | \|\| id:ebi-a-GCST90001697 | ebi-a-GCST90001697 | TRUE | C | 68217724 | 1.57E-10 | reported | 3425 | 1.098 | rs742867 |  |  |  |  |  |  |  |  |  |  |  |  |  |  |  |
| 176.7 | 2 | igd | 0.0029 | G | \|\| id:ebi-a-GCST90001698 | ebi-a-GCST90001698 | TRUE | A | 206257759 | 3.28E-08 | reported | 3408 | 31.91 | rs72946029 |  |  |  |  |  |  |  |  |  |  |  |  |  |  |  |
| 203 | 2 | igd | 0.0029 | T | \|\| id:ebi-a-GCST90001698 | ebi-a-GCST90001698 | TRUE | C | 1592272 | 1.41E-08 | reported | 3408 | 35.7 | rs76568972 |  |  |  |  |  |  |  |  |  |  |  |  |  |  |  |
| 202.3 | 3 | igd | 0.0029 | G | \|\| id:ebi-a-GCST90001698 | ebi-a-GCST90001698 | TRUE | A | 183061652 | 6.63E-09 | reported | 3408 | 34.78 | rs192693069 |  |  |  |  |  |  |  |  |  |  |  |  |  |  |  |
| 255.9 | 4 | igd | 0.0029 | A | \|\| id:ebi-a-GCST90001698 | ebi-a-GCST90001698 | TRUE | G | 181272381 | 8.89E-18 | reported | 3408 | 29.64 | rs17069255 |  |  |  |  |  |  |  |  |  |  |  |  |  |  |  |
| 218.4 | 6 | igd | 0.0029 | C | \|\| id:ebi-a-GCST90001698 | ebi-a-GCST90001698 | TRUE | T | 152137377 | 5.41E-11 | reported | 3408 | 33.18 | rs73780868 |  |  |  |  |  |  |  |  |  |  |  |  |  |  |  |
| 191.6 | 7 | igd | 0.0029 | G | \|\| id:ebi-a-GCST90001698 | ebi-a-GCST90001698 | TRUE | T | 116026485 | 1.05E-09 | reported | 3408 | 31.32 | rs142483421 |  |  |  |  |  |  |  |  |  |  |  |  |  |  |  |
| 247.6 | 7 | igd | 0.0029 | T | \|\| id:ebi-a-GCST90001698 | ebi-a-GCST90001698 | TRUE | C | 138060697 | 2.98E-13 | reported | 3408 | 33.81 | rs145421571 |  |  |  |  |  |  |  |  |  |  |  |  |  |  |  |
| 254.2 | 7 | igd | 0.0029 | C | \|\| id:ebi-a-GCST90001698 | ebi-a-GCST90001698 | TRUE | T | 56879331 | 1.97E-14 | reported | 3408 | 33.07 | rs560527730 |  |  |  |  |  |  |  |  |  |  |  |  |  |  |  |
| 216.1 | 7 | igd | 0.0029 | C | \|\| id:ebi-a-GCST90001698 | ebi-a-GCST90001698 | TRUE | A | 57699019 | 1.99E-10 | reported | 3408 | 33.87 | rs537258273 |  |  |  |  |  |  |  |  |  |  |  |  |  |  |  |
| 180.5 | 8 | igd | 0.0029 | T | \|\| id:ebi-a-GCST90001698 | ebi-a-GCST90001698 | TRUE | C | 144718749 | 1.47E-08 | reported | 3408 | 31.78 | rs149005143 |  |  |  |  |  |  |  |  |  |  |  |  |  |  |  |
| 185.2 | 8 | igd | 0.0029 | T | \|\| id:ebi-a-GCST90001698 | ebi-a-GCST90001698 | TRUE | G | 29165023 | 1.82E-08 | reported | 3408 | 32.82 | rs367824960 |  |  |  |  |  |  |  |  |  |  |  |  |  |  |  |
| 263.6 | 11 | igd | 0.0029 | T | \|\| id:ebi-a-GCST90001698 | ebi-a-GCST90001698 | TRUE | C | 25534610 | 1.02E-16 | reported | 3408 | 31.58 | rs78922116 |  |  |  |  |  |  |  |  |  |  |  |  |  |  |  |
| 183 | 11 | igd | 0.0029 | G | \|\| id:ebi-a-GCST90001698 | ebi-a-GCST90001698 | TRUE | T | 94284134 | 3.42E-08 | reported | 3408 | 33.09 | rs78524989 |  |  |  |  |  |  |  |  |  |  |  |  |  |  |  |
| 187 | 11 | igd | 0.0029 | A | \|\| id:ebi-a-GCST90001698 | ebi-a-GCST90001698 | TRUE | G | 76470121 | 3.98E-09 | reported | 3408 | 31.68 | rs138259561 |  |  |  |  |  |  |  |  |  |  |  |  |  |  |  |
| 188 | 11 | igd | 0.0029 | A | \|\| id:ebi-a-GCST90001698 | ebi-a-GCST90001698 | TRUE | G | 1149087 | 2.71E-08 | reported | 3408 | 33.74 | rs117573224 |  |  |  |  |  |  |  |  |  |  |  |  |  |  |  |
| 207 | 13 | igd | 0.0029 | T | \|\| id:ebi-a-GCST90001698 | ebi-a-GCST90001698 | TRUE | C | 112088993 | 9.17E-10 | reported | 3408 | 33.71 | rs75550713 |  |  |  |  |  |  |  |  |  |  |  |  |  |  |  |
| 211.5 | 13 | igd | 0.0029 | A | \|\| id:ebi-a-GCST90001698 | ebi-a-GCST90001698 | TRUE | G | 24658539 | 8.21E-11 | reported | 3408 | 32.45 | rs76942041 |  |  |  |  |  |  |  |  |  |  |  |  |  |  |  |
| 217.1 | 14 | igd | 0.0029 | G | \|\| id:ebi-a-GCST90001698 | ebi-a-GCST90001698 | TRUE | C | 55526313 | 8.42E-11 | reported | 3408 | 33.33 | rs147158544 |  |  |  |  |  |  |  |  |  |  |  |  |  |  |  |
| 191.9 | 14 | igd | 0.0029 | C | \|\| id:ebi-a-GCST90001698 | ebi-a-GCST90001698 | TRUE | T | 58704708 | 3.39E-09 | reported | 3408 | 32.38 | rs72716934 |  |  |  |  |  |  |  |  |  |  |  |  |  |  |  |
| 181.9 | 16 | igd | 0.0029 | A | \|\| id:ebi-a-GCST90001698 | ebi-a-GCST90001698 | TRUE | G | 5628078 | 2.90E-08 | reported | 3408 | 32.71 | rs142076427 |  |  |  |  |  |  |  |  |  |  |  |  |  |  |  |
| 214 | 20 | igd | 0.0029 | T | \|\| id:ebi-a-GCST90001698 | ebi-a-GCST90001698 | TRUE | C | 14932245 | 1.43E-09 | reported | 3408 | 35.26 | rs143279177 |  |  |  |  |  |  |  |  |  |  |  |  |  |  |  |
| 199 | 21 | igd | 0.0029 | A | \|\| id:ebi-a-GCST90001698 | ebi-a-GCST90001698 | TRUE | T | 35532237 | 1.26E-08 | reported | 3408 | 34.88 | rs111714479 |  |  |  |  |  |  |  |  |  |  |  |  |  |  |  |
| 1.489 | 1 | igd | 0.0029 | G | \|\| id:ebi-a-GCST90001941 | ebi-a-GCST90001941 | TRUE | A | 198151688 | 7.34E-10 | reported | 3435 | 0.241 | rs113435341 |  |  |  |  |  |  |  |  |  |  |  |  |  |  |  |
| 1.802 | 1 | igd | 0.0029 | A | \|\| id:ebi-a-GCST90001941 | ebi-a-GCST90001941 | TRUE | G | 198283058 | 1.28E-10 | reported | 3435 | 0.2794 | rs541367304 |  |  |  |  |  |  |  |  |  |  |  |  |  |  |  |
| 1.932 | 1 | igd | 0.0029 | T | \|\| id:ebi-a-GCST90001941 | ebi-a-GCST90001941 | TRUE | G | 198524264 | 3.05E-10 | reported | 3435 | 0.3059 | rs146620586 |  |  |  |  |  |  |  |  |  |  |  |  |  |  |  |
| 1.661 | 1 | igd | 0.0029 | A | \|\| id:ebi-a-GCST90001942 | ebi-a-GCST90001942 | TRUE | G | 198283058 | 2.81E-09 | reported | 3435 | 0.2788 | rs541367304 |  |  |  |  |  |  |  |  |  |  |  |  |  |  |  |
| 1.367 | 1 | igd | 0.0029 | G | \|\| id:ebi-a-GCST90001942 | ebi-a-GCST90001942 | TRUE | A | 198151688 | 1.43E-08 | reported | 3435 | 0.2405 | rs113435341 |  |  |  |  |  |  |  |  |  |  |  |  |  |  |  |
| 1.766 | 1 | igd | 0.0029 | T | \|\| id:ebi-a-GCST90001942 | ebi-a-GCST90001942 | TRUE | G | 198524264 | 7.79E-09 | reported | 3435 | 0.3052 | rs146620586 |  |  |  |  |  |  |  |  |  |  |  |  |  |  |  |
| 2.212 | 1 | igd | 0.0029 | A | \|\| id:ebi-a-GCST90002098 | ebi-a-GCST90002098 | TRUE | G | 197924770 | 2.83E-21 | reported | 2910 | 0.2318 | rs17583875 |  |  |  |  |  |  |  |  |  |  |  |  |  |  |  |
| 2.31 | 1 | igd | 0.0029 | G | \|\| id:ebi-a-GCST90002102 | ebi-a-GCST90002102 | TRUE | A | 198151688 | 6.19E-22 | reported | 2919 | 0.238 | rs113435341 |  |  |  |  |  |  |  |  |  |  |  |  |  |  |  |
| 2.889 | 1 | igd | 0.0029 | T | \|\| id:ebi-a-GCST90002102 | ebi-a-GCST90002102 | TRUE | G | 198524264 | 9.73E-22 | reported | 2919 | 0.2992 | rs146620586 |  |  |  |  |  |  |  |  |  |  |  |  |  |  |  |
| 2.693 | 1 | igd | 0.0029 | A | \|\| id:ebi-a-GCST90002102 | ebi-a-GCST90002102 | TRUE | G | 198283058 | 2.12E-22 | reported | 2919 | 0.2744 | rs541367304 |  |  |  |  |  |  |  |  |  |  |  |  |  |  |  |
| 150.1 | 15 | igd | 0.0028 | T | \|\| id:ebi-a-GCST90001695 | ebi-a-GCST90001695 | TRUE | G | 96363039 | 2.20E-09 | reported | 3408 | 25.02 | rs139857965 |  |  |  |  |  |  |  |  |  |  |  |  |  |  |  |
| 151.8 | 16 | igd | 0.0028 | C | \|\| id:ebi-a-GCST90001695 | ebi-a-GCST90001695 | TRUE | T | 17341866 | 9.76E-10 | reported | 3408 | 24.76 | rs140134394 |  |  |  |  |  |  |  |  |  |  |  |  |  |  |  |
| 198.7 | 1 | igd | 0.0028 | C | \|\| id:ebi-a-GCST90001698 | ebi-a-GCST90001698 | TRUE | T | 203800675 | 8.75E-09 | reported | 3408 | 34.45 | rs61827268 |  |  |  |  |  |  |  |  |  |  |  |  |  |  |  |
| 267.7 | 1 | igd | 0.0028 | T | \|\| id:ebi-a-GCST90001698 | ebi-a-GCST90001698 | TRUE | G | 102519585 | 1.47E-14 | reported | 3408 | 34.66 | rs77557517 |  |  |  |  |  |  |  |  |  |  |  |  |  |  |  |
| 225.9 | 1 | igd | 0.0028 | A | \|\| id:ebi-a-GCST90001698 | ebi-a-GCST90001698 | TRUE | G | 227475703 | 3.61E-12 | reported | 3408 | 32.38 | rs113415332 |  |  |  |  |  |  |  |  |  |  |  |  |  |  |  |
| 223.1 | 2 | igd | 0.0028 | A | \|\| id:ebi-a-GCST90001698 | ebi-a-GCST90001698 | TRUE | G | 241020317 | 7.25E-11 | reported | 3408 | 34.13 | rs184258293 |  |  |  |  |  |  |  |  |  |  |  |  |  |  |  |
| 193.9 | 3 | igd | 0.0028 | T | \|\| id:ebi-a-GCST90001698 | ebi-a-GCST90001698 | TRUE | C | 67519105 | 6.54E-09 | reported | 3408 | 33.32 | rs75000537 |  |  |  |  |  |  |  |  |  |  |  |  |  |  |  |
| 235.6 | 3 | igd | 0.0028 | A | \|\| id:ebi-a-GCST90001698 | ebi-a-GCST90001698 | TRUE | G | 3132754 | 8.14E-10 | reported | 3408 | 38.25 | rs17878699 |  |  |  |  |  |  |  |  |  |  |  |  |  |  |  |
| 184.6 | 3 | igd | 0.0028 | T | \|\| id:ebi-a-GCST90001698 | ebi-a-GCST90001698 | TRUE | C | 152823388 | 2.02E-08 | reported | 3408 | 32.82 | rs74755787 |  |  |  |  |  |  |  |  |  |  |  |  |  |  |  |
| 195.3 | 4 | igd | 0.0028 | G | \|\| id:ebi-a-GCST90001698 | ebi-a-GCST90001698 | TRUE | C | 186198542 | 7.83E-09 | reported | 3408 | 33.75 | rs13143236 |  |  |  |  |  |  |  |  |  |  |  |  |  |  |  |
| 203.9 | 6 | igd | 0.0028 | G | \|\| id:ebi-a-GCST90001698 | ebi-a-GCST90001698 | TRUE | A | 162457335 | 2.60E-08 | reported | 3408 | 36.54 | rs149801155 |  |  |  |  |  |  |  |  |  |  |  |  |  |  |  |
| 208.3 | 6 | igd | 0.0028 | T | \|\| id:ebi-a-GCST90001698 | ebi-a-GCST90001698 | TRUE | C | 119514715 | 8.96E-11 | reported | 3408 | 32.03 | rs117185099 |  |  |  |  |  |  |  |  |  |  |  |  |  |  |  |
| 216.8 | 6 | igd | 0.0028 | C | \|\| id:ebi-a-GCST90001698 | ebi-a-GCST90001698 | TRUE | T | 127536446 | 2.49E-10 | reported | 3408 | 34.16 | rs141049042 |  |  |  |  |  |  |  |  |  |  |  |  |  |  |  |
| 219.9 | 7 | igd | 0.0028 | C | \|\| id:ebi-a-GCST90001698 | ebi-a-GCST90001698 | TRUE | T | 115706613 | 8.78E-11 | reported | 3408 | 33.8 | rs137997289 |  |  |  |  |  |  |  |  |  |  |  |  |  |  |  |
| 228.2 | 7 | igd | 0.0028 | C | \|\| id:ebi-a-GCST90001698 | ebi-a-GCST90001698 | TRUE | T | 101754392 | 4.17E-11 | reported | 3408 | 34.47 | rs554574739 |  |  |  |  |  |  |  |  |  |  |  |  |  |  |  |
| 224 | 9 | igd | 0.0028 | C | \|\| id:ebi-a-GCST90001698 | ebi-a-GCST90001698 | TRUE | T | 29764580 | 3.06E-12 | reported | 3408 | 32 | rs117871241 |  |  |  |  |  |  |  |  |  |  |  |  |  |  |  |
| 224.1 | 9 | igd | 0.0028 | T | \|\| id:ebi-a-GCST90001698 | ebi-a-GCST90001698 | TRUE | A | 29889453 | 3.03E-12 | reported | 3408 | 32 | rs118062750 |  |  |  |  |  |  |  |  |  |  |  |  |  |  |  |
| 223.7 | 12 | igd | 0.0028 | A | \|\| id:ebi-a-GCST90001698 | ebi-a-GCST90001698 | TRUE | G | 79764351 | 5.64E-11 | reported | 3408 | 34.03 | rs148181242 |  |  |  |  |  |  |  |  |  |  |  |  |  |  |  |
| 255.1 | 13 | igd | 0.0028 | T | \|\| id:ebi-a-GCST90001698 | ebi-a-GCST90001698 | TRUE | C | 77081029 | 2.52E-14 | reported | 3408 | 33.32 | rs117177255 |  |  |  |  |  |  |  |  |  |  |  |  |  |  |  |
| 221.3 | 14 | igd | 0.0028 | A | \|\| id:ebi-a-GCST90001698 | ebi-a-GCST90001698 | TRUE | G | 57252645 | 1.29E-09 | reported | 3408 | 36.37 | rs74566408 |  |  |  |  |  |  |  |  |  |  |  |  |  |  |  |
| 268.2 | 15 | igd | 0.0028 | A | \|\| id:ebi-a-GCST90001698 | ebi-a-GCST90001698 | TRUE | C | 54488410 | 9.51E-14 | reported | 3408 | 35.86 | rs77969678 |  |  |  |  |  |  |  |  |  |  |  |  |  |  |  |
| 189.1 | 17 | igd | 0.0028 | T | \|\| id:ebi-a-GCST90001698 | ebi-a-GCST90001698 | TRUE | C | 30967385 | 1.84E-08 | reported | 3408 | 33.53 | rs143393137 |  |  |  |  |  |  |  |  |  |  |  |  |  |  |  |
| 182.2 | 21 | igd | 0.0028 | T | \|\| id:ebi-a-GCST90001698 | ebi-a-GCST90001698 | TRUE | C | 37446078 | 4.51E-08 | reported | 3408 | 33.24 | rs41562512 |  |  |  |  |  |  |  |  |  |  |  |  |  |  |  |
| 1.489 | 1 | igd | 0.0028 | A | \|\| id:ebi-a-GCST90002103 | ebi-a-GCST90002103 | TRUE | G | 197924770 | 1.29E-08 | reported | 2650 | 0.2609 | rs17583875 |  |  |  |  |  |  |  |  |  |  |  |  |  |  |  |
| 148.2 | 6 | igd | 0.0026 | T | \|\| id:ebi-a-GCST90001695 | ebi-a-GCST90001695 | TRUE | C | 78745175 | 4.22E-09 | reported | 3408 | 25.16 | rs145336406 |  |  |  |  |  |  |  |  |  |  |  |  |  |  |  |
| 147.6 | 16 | igd | 0.0026 | T | \|\| id:ebi-a-GCST90001695 | ebi-a-GCST90001695 | TRUE | C | 75249375 | 5.36E-09 | reported | 3408 | 25.23 | rs148200695 |  |  |  |  |  |  |  |  |  |  |  |  |  |  |  |
| 188.7 | 1 | igd | 0.0026 | C | \|\| id:ebi-a-GCST90001698 | ebi-a-GCST90001698 | TRUE | T | 237836250 | 1.83E-08 | reported | 3408 | 33.44 | rs115355154 |  |  |  |  |  |  |  |  |  |  |  |  |  |  |  |
| 245.9 | 1 | igd | 0.0026 | G | \|\| id:ebi-a-GCST90001698 | ebi-a-GCST90001698 | TRUE | A | 4759830 | 5.14E-12 | reported | 3408 | 35.5 | rs76235097 |  |  |  |  |  |  |  |  |  |  |  |  |  |  |  |
| 216.5 | 2 | igd | 0.0026 | C | \|\| id:ebi-a-GCST90001698 | ebi-a-GCST90001698 | TRUE | A | 87503702 | 1.00E-09 | reported | 3408 | 35.34 | rs142604048 |  |  |  |  |  |  |  |  |  |  |  |  |  |  |  |
| 206.8 | 2 | igd | 0.0026 | A | \|\| id:ebi-a-GCST90001698 | ebi-a-GCST90001698 | TRUE | G | 127831527 | 2.03E-10 | reported | 3408 | 32.42 | rs112785730 |  |  |  |  |  |  |  |  |  |  |  |  |  |  |  |
| 230.6 | 2 | igd | 0.0026 | T | \|\| id:ebi-a-GCST90001698 | ebi-a-GCST90001698 | TRUE | C | 22576943 | 1.02E-11 | reported | 3408 | 33.78 | rs183895386 |  |  |  |  |  |  |  |  |  |  |  |  |  |  |  |
| 289 | 2 | igd | 0.0026 | G | \|\| id:ebi-a-GCST90001698 | ebi-a-GCST90001698 | TRUE | A | 243057445 | 5.61E-09 | reported | 3408 | 49.46 | rs191734212 |  |  |  |  |  |  |  |  |  |  |  |  |  |  |  |
| 210.6 | 2 | igd | 0.0026 | T | \|\| id:ebi-a-GCST90001698 | ebi-a-GCST90001698 | TRUE | C | 169297185 | 4.98E-10 | reported | 3408 | 33.76 | rs140929582 |  |  |  |  |  |  |  |  |  |  |  |  |  |  |  |
| 199.4 | 2 | igd | 0.0026 | A | \|\| id:ebi-a-GCST90001698 | ebi-a-GCST90001698 | TRUE | C | 195788562 | 9.74E-09 | reported | 3408 | 34.68 | rs140173523 |  |  |  |  |  |  |  |  |  |  |  |  |  |  |  |
| 207.4 | 2 | igd | 0.0026 | A | \|\| id:ebi-a-GCST90001698 | ebi-a-GCST90001698 | TRUE | T | 169189038 | 1.18E-08 | reported | 3408 | 36.27 | rs114253343 |  |  |  |  |  |  |  |  |  |  |  |  |  |  |  |
| 265.3 | 4 | igd | 0.0026 | G | \|\| id:ebi-a-GCST90001698 | ebi-a-GCST90001698 | TRUE | A | 85975191 | 7.98E-12 | reported | 3408 | 38.66 | rs150133604 |  |  |  |  |  |  |  |  |  |  |  |  |  |  |  |
| 186.1 | 6 | igd | 0.0026 | T | \|\| id:ebi-a-GCST90001698 | ebi-a-GCST90001698 | TRUE | C | 78745175 | 4.06E-08 | reported | 3408 | 33.82 | rs145336406 |  |  |  |  |  |  |  |  |  |  |  |  |  |  |  |
| 206.9 | 6 | igd | 0.0026 | A | \|\| id:ebi-a-GCST90001698 | ebi-a-GCST90001698 | TRUE | G | 159962229 | 2.34E-09 | reported | 3408 | 34.55 | rs111867376 |  |  |  |  |  |  |  |  |  |  |  |  |  |  |  |
| 213 | 6 | igd | 0.0026 | A | \|\| id:ebi-a-GCST90001698 | ebi-a-GCST90001698 | TRUE | G | 152427849 | 6.31E-10 | reported | 3408 | 34.35 | rs149300552 |  |  |  |  |  |  |  |  |  |  |  |  |  |  |  |
| 209 | 7 | igd | 0.0026 | G | \|\| id:ebi-a-GCST90001698 | ebi-a-GCST90001698 | TRUE | C | 154979218 | 1.94E-09 | reported | 3408 | 34.72 | rs62472120 |  |  |  |  |  |  |  |  |  |  |  |  |  |  |  |
| 206.4 | 8 | igd | 0.0026 | A | \|\| id:ebi-a-GCST90001698 | ebi-a-GCST90001698 | TRUE | G | 73079210 | 3.85E-08 | reported | 3408 | 37.47 | rs117808048 |  |  |  |  |  |  |  |  |  |  |  |  |  |  |  |
| 219.6 | 9 | igd | 0.0026 | A | \|\| id:ebi-a-GCST90001698 | ebi-a-GCST90001698 | TRUE | T | 107332377 | 5.57E-11 | reported | 3408 | 33.39 | rs76017116 |  |  |  |  |  |  |  |  |  |  |  |  |  |  |  |
| 200.1 | 12 | igd | 0.0026 | G | \|\| id:ebi-a-GCST90001698 | ebi-a-GCST90001698 | TRUE | A | 96394364 | 1.99E-09 | reported | 3408 | 33.27 | rs147948747 |  |  |  |  |  |  |  |  |  |  |  |  |  |  |  |
| 237.5 | 12 | igd | 0.0026 | T | \|\| id:ebi-a-GCST90001698 | ebi-a-GCST90001698 | TRUE | C | 80464739 | 6.69E-10 | reported | 3408 | 38.36 | rs78547506 |  |  |  |  |  |  |  |  |  |  |  |  |  |  |  |
| 218.2 | 14 | igd | 0.0026 | A | \|\| id:ebi-a-GCST90001698 | ebi-a-GCST90001698 | TRUE | G | 58912880 | 1.29E-10 | reported | 3408 | 33.84 | rs72717000 |  |  |  |  |  |  |  |  |  |  |  |  |  |  |  |
| 192.6 | 16 | igd | 0.0026 | A | \|\| id:ebi-a-GCST90001698 | ebi-a-GCST90001698 | TRUE | C | 23355581 | 4.96E-08 | reported | 3408 | 35.24 | rs150935172 |  |  |  |  |  |  |  |  |  |  |  |  |  |  |  |
| 221 | 16 | igd | 0.0026 | G | \|\| id:ebi-a-GCST90001698 | ebi-a-GCST90001698 | TRUE | C | 50471284 | 2.91E-09 | reported | 3408 | 37.13 | rs117898578 |  |  |  |  |  |  |  |  |  |  |  |  |  |  |  |
| 211.7 | 17 | igd | 0.0026 | A | \|\| id:ebi-a-GCST90001698 | ebi-a-GCST90001698 | TRUE | G | 39959627 | 2.42E-10 | reported | 3408 | 33.34 | rs34539402 |  |  |  |  |  |  |  |  |  |  |  |  |  |  |  |
| 192.2 | 18 | igd | 0.0026 | C | \|\| id:ebi-a-GCST90001698 | ebi-a-GCST90001698 | TRUE | G | 57661776 | 1.83E-08 | reported | 3408 | 34.07 | rs150073722 |  |  |  |  |  |  |  |  |  |  |  |  |  |  |  |
| 220.8 | 18 | igd | 0.0026 | C | \|\| id:ebi-a-GCST90001698 | ebi-a-GCST90001698 | TRUE | A | 26275367 | 1.54E-09 | reported | 3408 | 36.46 | rs148153860 |  |  |  |  |  |  |  |  |  |  |  |  |  |  |  |
| 187.4 | 19 | igd | 0.0026 | G | \|\| id:ebi-a-GCST90001698 | ebi-a-GCST90001698 | TRUE | C | 45630360 | 3.51E-08 | reported | 3408 | 33.91 | rs146989801 |  |  |  |  |  |  |  |  |  |  |  |  |  |  |  |
| 222.6 | 19 | igd | 0.0026 | T | \|\| id:ebi-a-GCST90001698 | ebi-a-GCST90001698 | TRUE | C | 22191029 | 4.19E-10 | reported | 3408 | 35.54 | rs189012866 |  |  |  |  |  |  |  |  |  |  |  |  |  |  |  |
| 219.5 | 19 | igd | 0.0026 | T | \|\| id:ebi-a-GCST90001698 | ebi-a-GCST90001698 | TRUE | C | 18473968 | 1.04E-09 | reported | 3408 | 35.86 | rs139824473 |  |  |  |  |  |  |  |  |  |  |  |  |  |  |  |
| 272.8 | 19 | igd | 0.0026 | T | \|\| id:ebi-a-GCST90001698 | ebi-a-GCST90001698 | TRUE | C | 27993861 | 4.20E-12 | reported | 3408 | 39.23 | rs143756329 |  |  |  |  |  |  |  |  |  |  |  |  |  |  |  |
| 198.5 | 19 | igd | 0.0026 | A | \|\| id:ebi-a-GCST90001698 | ebi-a-GCST90001698 | TRUE | G | 29372503 | 3.85E-09 | reported | 3408 | 33.62 | rs77577257 |  |  |  |  |  |  |  |  |  |  |  |  |  |  |  |
| 191.1 | 20 | igd | 0.0026 | T | \|\| id:ebi-a-GCST90001698 | ebi-a-GCST90001698 | TRUE | A | 32794982 | 1.25E-08 | reported | 3408 | 33.48 | rs76327094 |  |  |  |  |  |  |  |  |  |  |  |  |  |  |  |
| 2.32 | 1 | igd | 0.0026 | A | \|\| id:ebi-a-GCST90002102 | ebi-a-GCST90002102 | TRUE | G | 197924770 | 4.86E-21 | reported | 2919 | 0.2446 | rs17583875 |  |  |  |  |  |  |  |  |  |  |  |  |  |  |  |
| 159.7 | 3 | igd | 0.0025 | G | \|\| id:ebi-a-GCST90001695 | ebi-a-GCST90001695 | TRUE | A | 29402908 | 2.42E-09 | reported | 3408 | 26.69 | rs11716645 |  |  |  |  |  |  |  |  |  |  |  |  |  |  |  |
| 137.6 | 14 | igd | 0.0025 | G | \|\| id:ebi-a-GCST90001695 | ebi-a-GCST90001695 | TRUE | A | 84194540 | 1.82E-08 | reported | 3408 | 24.38 | rs111953852 |  |  |  |  |  |  |  |  |  |  |  |  |  |  |  |
| 161.7 | 16 | igd | 0.0025 | T | \|\| id:ebi-a-GCST90001695 | ebi-a-GCST90001695 | TRUE | C | 68607748 | 1.33E-09 | reported | 3408 | 26.59 | rs140605634 |  |  |  |  |  |  |  |  |  |  |  |  |  |  |  |
| 210.7 | 1 | igd | 0.0025 | T | \|\| id:ebi-a-GCST90001698 | ebi-a-GCST90001698 | TRUE | C | 111842326 | 4.25E-09 | reported | 3408 | 35.78 | rs144942838 |  |  |  |  |  |  |  |  |  |  |  |  |  |  |  |
| 237.5 | 1 | igd | 0.0025 | T | \|\| id:ebi-a-GCST90001698 | ebi-a-GCST90001698 | TRUE | C | 247552838 | 6.53E-09 | reported | 3408 | 40.82 | rs9651010 |  |  |  |  |  |  |  |  |  |  |  |  |  |  |  |
| 201 | 3 | igd | 0.0025 | T | \|\| id:ebi-a-GCST90001698 | ebi-a-GCST90001698 | TRUE | C | 186991832 | 1.26E-08 | reported | 3408 | 35.23 | rs148301040 |  |  |  |  |  |  |  |  |  |  |  |  |  |  |  |
| 200.5 | 3 | igd | 0.0025 | A | \|\| id:ebi-a-GCST90001698 | ebi-a-GCST90001698 | TRUE | G | 78262281 | 1.09E-08 | reported | 3408 | 35 | rs6766455 |  |  |  |  |  |  |  |  |  |  |  |  |  |  |  |
| 202.1 | 3 | igd | 0.0025 | T | \|\| id:ebi-a-GCST90001698 | ebi-a-GCST90001698 | TRUE | C | 138645438 | 6.60E-09 | reported | 3408 | 34.75 | rs188215927 |  |  |  |  |  |  |  |  |  |  |  |  |  |  |  |
| 203.5 | 4 | igd | 0.0025 | A | \|\| id:ebi-a-GCST90001698 | ebi-a-GCST90001698 | TRUE | G | 54854600 | 2.21E-08 | reported | 3408 | 36.28 | rs113577058 |  |  |  |  |  |  |  |  |  |  |  |  |  |  |  |
| 230.6 | 6 | igd | 0.0025 | A | \|\| id:ebi-a-GCST90001698 | ebi-a-GCST90001698 | TRUE | G | 21345473 | 1.23E-09 | reported | 3408 | 37.84 | rs144246120 |  |  |  |  |  |  |  |  |  |  |  |  |  |  |  |
| 294.3 | 6 | igd | 0.0025 | A | \|\| id:ebi-a-GCST90001698 | ebi-a-GCST90001698 | TRUE | G | 84704240 | 5.23E-20 | reported | 3408 | 31.93 | rs147650194 |  |  |  |  |  |  |  |  |  |  |  |  |  |  |  |
| 210.8 | 7 | igd | 0.0025 | A | \|\| id:ebi-a-GCST90001698 | ebi-a-GCST90001698 | TRUE | T | 115500463 | 1.98E-10 | reported | 3408 | 33.03 | rs7455442 |  |  |  |  |  |  |  |  |  |  |  |  |  |  |  |
| 206.7 | 7 | igd | 0.0025 | A | \|\| id:ebi-a-GCST90001698 | ebi-a-GCST90001698 | TRUE | G | 88787842 | 4.18E-09 | reported | 3408 | 35.08 | rs13230395 |  |  |  |  |  |  |  |  |  |  |  |  |  |  |  |
| 300.6 | 7 | igd | 0.0025 | G | \|\| id:ebi-a-GCST90001698 | ebi-a-GCST90001698 | TRUE | A | 62917010 | 4.15E-16 | reported | 3408 | 36.78 | rs185754925 |  |  |  |  |  |  |  |  |  |  |  |  |  |  |  |
| 219.8 | 7 | igd | 0.0025 | C | \|\| id:ebi-a-GCST90001698 | ebi-a-GCST90001698 | TRUE | G | 49481239 | 5.36E-10 | reported | 3408 | 35.3 | rs17392885 |  |  |  |  |  |  |  |  |  |  |  |  |  |  |  |
| 198.1 | 8 | igd | 0.0025 | T | \|\| id:ebi-a-GCST90001698 | ebi-a-GCST90001698 | TRUE | C | 31108124 | 1.08E-08 | reported | 3408 | 34.57 | rs149897686 |  |  |  |  |  |  |  |  |  |  |  |  |  |  |  |
| 245.4 | 9 | igd | 0.0025 | A | \|\| id:ebi-a-GCST90001698 | ebi-a-GCST90001698 | TRUE | G | 79057643 | 3.79E-11 | reported | 3408 | 36.99 | rs12378893 |  |  |  |  |  |  |  |  |  |  |  |  |  |  |  |
| 226.6 | 11 | igd | 0.0025 | C | \|\| id:ebi-a-GCST90001698 | ebi-a-GCST90001698 | TRUE | T | 6405935 | 4.15E-11 | reported | 3408 | 34.22 | rs186313806 |  |  |  |  |  |  |  |  |  |  |  |  |  |  |  |
| 262.4 | 14 | igd | 0.0025 | A | \|\| id:ebi-a-GCST90001698 | ebi-a-GCST90001698 | TRUE | G | 55295865 | 1.91E-12 | reported | 3408 | 37.13 | rs150055480 |  |  |  |  |  |  |  |  |  |  |  |  |  |  |  |
| 286.8 | 18 | igd | 0.0025 | T | \|\| id:ebi-a-GCST90001698 | ebi-a-GCST90001698 | TRUE | C | 63832285 | 8.99E-14 | reported | 3408 | 38.31 | rs536434085 |  |  |  |  |  |  |  |  |  |  |  |  |  |  |  |
| 242.3 | 19 | igd | 0.0025 | C | \|\| id:ebi-a-GCST90001698 | ebi-a-GCST90001698 | TRUE | T | 24118546 | 1.30E-11 | reported | 3408 | 35.68 | rs74820396 |  |  |  |  |  |  |  |  |  |  |  |  |  |  |  |
| 231.8 | 20 | igd | 0.0025 | T | \|\| id:ebi-a-GCST90001698 | ebi-a-GCST90001698 | TRUE | C | 33642396 | 3.63E-10 | reported | 3408 | 36.87 | rs62213709 |  |  |  |  |  |  |  |  |  |  |  |  |  |  |  |
| 8.527 | 7 | igd | 0.0025 | G | \|\| id:ebi-a-GCST90001700 | ebi-a-GCST90001700 | TRUE | A | 62917010 | 4.90E-08 | reported | 3425 | 1.56 | rs185754925 |  |  |  |  |  |  |  |  |  |  |  |  |  |  |  |
| 178.9 | 8 | igd | 0.0023 | C | \|\| id:ebi-a-GCST90001695 | ebi-a-GCST90001695 | TRUE | T | 42077996 | 9.07E-11 | reported | 3408 | 27.52 | rs59236867 |  |  |  |  |  |  |  |  |  |  |  |  |  |  |  |
| 184.6 | 16 | igd | 0.0023 | G | \|\| id:ebi-a-GCST90001695 | ebi-a-GCST90001695 | TRUE | C | 80791733 | 2.06E-11 | reported | 3408 | 27.45 | rs116964140 |  |  |  |  |  |  |  |  |  |  |  |  |  |  |  |
| 205.4 | 1 | igd | 0.0023 | C | \|\| id:ebi-a-GCST90001698 | ebi-a-GCST90001698 | TRUE | G | 30792221 | 2.06E-08 | reported | 3408 | 36.55 | rs144182418 |  |  |  |  |  |  |  |  |  |  |  |  |  |  |  |
| 324.4 | 1 | igd | 0.0023 | A | \|\| id:ebi-a-GCST90001698 | ebi-a-GCST90001698 | TRUE | G | 102796337 | 1.01E-17 | reported | 3408 | 37.63 | rs114230155 |  |  |  |  |  |  |  |  |  |  |  |  |  |  |  |
| 205.2 | 2 | igd | 0.0023 | G | \|\| id:ebi-a-GCST90001698 | ebi-a-GCST90001698 | TRUE | A | 110257372 | 2.55E-08 | reported | 3408 | 36.76 | rs75443298 |  |  |  |  |  |  |  |  |  |  |  |  |  |  |  |
| 207.9 | 3 | igd | 0.0023 | A | \|\| id:ebi-a-GCST90001698 | ebi-a-GCST90001698 | TRUE | C | 195531004 | 1.15E-08 | reported | 3408 | 36.33 | rs115660429 |  |  |  |  |  |  |  |  |  |  |  |  |  |  |  |
| 212.5 | 3 | igd | 0.0023 | C | \|\| id:ebi-a-GCST90001698 | ebi-a-GCST90001698 | TRUE | T | 182409985 | 5.23E-09 | reported | 3408 | 36.29 | rs78741146 |  |  |  |  |  |  |  |  |  |  |  |  |  |  |  |
| 221 | 3 | igd | 0.0023 | T | \|\| id:ebi-a-GCST90001698 | ebi-a-GCST90001698 | TRUE | A | 130074113 | 2.75E-10 | reported | 3408 | 34.9 | rs77521823 |  |  |  |  |  |  |  |  |  |  |  |  |  |  |  |
| 240.6 | 5 | igd | 0.0023 | A | \|\| id:ebi-a-GCST90001698 | ebi-a-GCST90001698 | TRUE | G | 10691926 | 6.91E-11 | reported | 3408 | 36.78 | rs78625591 |  |  |  |  |  |  |  |  |  |  |  |  |  |  |  |
| 267.4 | 7 | igd | 0.0023 | G | \|\| id:ebi-a-GCST90001698 | ebi-a-GCST90001698 | TRUE | A | 21581550 | 7.48E-10 | reported | 3408 | 43.31 | rs72655959 |  |  |  |  |  |  |  |  |  |  |  |  |  |  |  |
| 224.7 | 7 | igd | 0.0023 | A | \|\| id:ebi-a-GCST90001698 | ebi-a-GCST90001698 | TRUE | G | 115314096 | 4.35E-11 | reported | 3408 | 33.98 | rs78451817 |  |  |  |  |  |  |  |  |  |  |  |  |  |  |  |
| 212.2 | 8 | igd | 0.0023 | T | \|\| id:ebi-a-GCST90001698 | ebi-a-GCST90001698 | TRUE | C | 30306775 | 2.52E-09 | reported | 3408 | 35.51 | rs113492924 |  |  |  |  |  |  |  |  |  |  |  |  |  |  |  |
| 228.9 | 8 | igd | 0.0023 | T | \|\| id:ebi-a-GCST90001698 | ebi-a-GCST90001698 | TRUE | C | 143605650 | 8.03E-09 | reported | 3408 | 39.59 | rs112471383 |  |  |  |  |  |  |  |  |  |  |  |  |  |  |  |
| 196 | 8 | igd | 0.0023 | G | \|\| id:ebi-a-GCST90001698 | ebi-a-GCST90001698 | TRUE | C | 69864162 | 3.68E-08 | reported | 3408 | 35.52 | rs117626443 |  |  |  |  |  |  |  |  |  |  |  |  |  |  |  |
| 252.6 | 9 | igd | 0.0023 | T | \|\| id:ebi-a-GCST90001698 | ebi-a-GCST90001698 | TRUE | C | 14983311 | 3.77E-11 | reported | 3408 | 38.08 | rs75067089 |  |  |  |  |  |  |  |  |  |  |  |  |  |  |  |
| 211.5 | 14 | igd | 0.0023 | T | \|\| id:ebi-a-GCST90001698 | ebi-a-GCST90001698 | TRUE | C | 72431599 | 4.80E-09 | reported | 3408 | 36.04 | rs61755651 |  |  |  |  |  |  |  |  |  |  |  |  |  |  |  |
| 234 | 19 | igd | 0.0023 | G | \|\| id:ebi-a-GCST90001698 | ebi-a-GCST90001698 | TRUE | T | 10500229 | 3.64E-10 | reported | 3408 | 37.22 | rs28382806 |  |  |  |  |  |  |  |  |  |  |  |  |  |  |  |
| 217.6 | 19 | igd | 0.0023 | G | \|\| id:ebi-a-GCST90001698 | ebi-a-GCST90001698 | TRUE | T | 17307102 | 4.72E-09 | reported | 3408 | 37.05 | rs77955766 |  |  |  |  |  |  |  |  |  |  |  |  |  |  |  |
| 230 | 19 | igd | 0.0023 | T | \|\| id:ebi-a-GCST90001698 | ebi-a-GCST90001698 | TRUE | C | 28276821 | 1.37E-10 | reported | 3408 | 35.71 | rs76424002 |  |  |  |  |  |  |  |  |  |  |  |  |  |  |  |
| 241.9 | 20 | igd | 0.0023 | G | \|\| id:ebi-a-GCST90001698 | ebi-a-GCST90001698 | TRUE | A | 37972398 | 6.69E-11 | reported | 3408 | 36.94 | rs76604626 |  |  |  |  |  |  |  |  |  |  |  |  |  |  |  |
| 221.5 | 20 | igd | 0.0023 | A | \|\| id:ebi-a-GCST90001698 | ebi-a-GCST90001698 | TRUE | G | 50004560 | 1.36E-10 | reported | 3408 | 34.4 | rs11538695 |  |  |  |  |  |  |  |  |  |  |  |  |  |  |  |
| 224.2 | 20 | igd | 0.0023 | G | \|\| id:ebi-a-GCST90001698 | ebi-a-GCST90001698 | TRUE | A | 18108907 | 2.58E-10 | reported | 3408 | 35.36 | rs146021667 |  |  |  |  |  |  |  |  |  |  |  |  |  |  |  |
| 9.265 | 1 | igd | 0.0023 | A | \|\| id:ebi-a-GCST90001700 | ebi-a-GCST90001700 | TRUE | G | 102796337 | 5.47E-09 | reported | 3425 | 1.585 | rs114230155 |  |  |  |  |  |  |  |  |  |  |  |  |  |  |  |
| 176.7 | 1 | igd | 0.0022 | A | \|\| id:ebi-a-GCST90001695 | ebi-a-GCST90001695 | TRUE | G | 21892266 | 1.92E-09 | reported | 3408 | 29.35 | rs114507999 |  |  |  |  |  |  |  |  |  |  |  |  |  |  |  |
| 185.1 | 2 | igd | 0.0022 | C | \|\| id:ebi-a-GCST90001695 | ebi-a-GCST90001695 | TRUE | T | 80415786 | 6.76E-12 | reported | 3408 | 26.88 | rs72815709 |  |  |  |  |  |  |  |  |  |  |  |  |  |  |  |
| 192.8 | 8 | igd | 0.0022 | A | \|\| id:ebi-a-GCST90001695 | ebi-a-GCST90001695 | TRUE | G | 112881674 | 1.63E-08 | reported | 3408 | 34.06 | rs117271894 |  |  |  |  |  |  |  |  |  |  |  |  |  |  |  |
| 172.7 | 8 | igd | 0.0022 | G | \|\| id:ebi-a-GCST90001695 | ebi-a-GCST90001695 | TRUE | T | 53929771 | 1.12E-09 | reported | 3408 | 28.26 | rs116959045 |  |  |  |  |  |  |  |  |  |  |  |  |  |  |  |
| 186.4 | 15 | igd | 0.0022 | A | \|\| id:ebi-a-GCST90001695 | ebi-a-GCST90001695 | TRUE | G | 95776793 | 2.26E-11 | reported | 3408 | 27.77 | rs75884646 |  |  |  |  |  |  |  |  |  |  |  |  |  |  |  |
| 18.44 | 2 | igd | 0.0022 | C | \|\| id:ebi-a-GCST90001696 | ebi-a-GCST90001696 | TRUE | T | 80415786 | 2.16E-09 | reported | 3425 | 3.073 | rs72815709 |  |  |  |  |  |  |  |  |  |  |  |  |  |  |  |
| 234.6 | 1 | igd | 0.0022 | A | \|\| id:ebi-a-GCST90001698 | ebi-a-GCST90001698 | TRUE | G | 21892266 | 3.60E-09 | reported | 3408 | 39.65 | rs114507999 |  |  |  |  |  |  |  |  |  |  |  |  |  |  |  |
| 288.2 | 1 | igd | 0.0022 | A | \|\| id:ebi-a-GCST90001698 | ebi-a-GCST90001698 | TRUE | G | 110381691 | 7.53E-14 | reported | 3408 | 38.37 | rs34060107 |  |  |  |  |  |  |  |  |  |  |  |  |  |  |  |
| 297 | 1 | igd | 0.0022 | C | \|\| id:ebi-a-GCST90001698 | ebi-a-GCST90001698 | TRUE | A | 195636177 | 2.47E-14 | reported | 3408 | 38.79 | rs116510159 |  |  |  |  |  |  |  |  |  |  |  |  |  |  |  |
| 304.4 | 2 | igd | 0.0022 | G | \|\| id:ebi-a-GCST90001698 | ebi-a-GCST90001698 | TRUE | A | 146174597 | 3.32E-16 | reported | 3408 | 37.12 | rs76498040 |  |  |  |  |  |  |  |  |  |  |  |  |  |  |  |
| 232.5 | 2 | igd | 0.0022 | A | \|\| id:ebi-a-GCST90001698 | ebi-a-GCST90001698 | TRUE | T | 28527777 | 7.20E-11 | reported | 3408 | 35.57 | rs181281260 |  |  |  |  |  |  |  |  |  |  |  |  |  |  |  |
| 257.4 | 2 | igd | 0.0022 | T | \|\| id:ebi-a-GCST90001698 | ebi-a-GCST90001698 | TRUE | C | 10326418 | 1.20E-12 | reported | 3408 | 36.09 | rs148947489 |  |  |  |  |  |  |  |  |  |  |  |  |  |  |  |
| 331.8 | 2 | igd | 0.0022 | C | \|\| id:ebi-a-GCST90001698 | ebi-a-GCST90001698 | TRUE | T | 146371302 | 2.06E-17 | reported | 3408 | 38.87 | rs79027575 |  |  |  |  |  |  |  |  |  |  |  |  |  |  |  |
| 237.4 | 2 | igd | 0.0022 | A | \|\| id:ebi-a-GCST90001698 | ebi-a-GCST90001698 | TRUE | G | 29081586 | 7.05E-11 | reported | 3408 | 36.3 | rs183787103 |  |  |  |  |  |  |  |  |  |  |  |  |  |  |  |
| 207.9 | 2 | igd | 0.0022 | G | \|\| id:ebi-a-GCST90001698 | ebi-a-GCST90001698 | TRUE | T | 211324936 | 4.34E-08 | reported | 3408 | 37.88 | rs72930513 |  |  |  |  |  |  |  |  |  |  |  |  |  |  |  |
| 297.4 | 2 | igd | 0.0022 | A | \|\| id:ebi-a-GCST90001698 | ebi-a-GCST90001698 | TRUE | C | 151265438 | 1.40E-14 | reported | 3408 | 38.47 | rs76732535 |  |  |  |  |  |  |  |  |  |  |  |  |  |  |  |
| 225.4 | 3 | igd | 0.0022 | T | \|\| id:ebi-a-GCST90001698 | ebi-a-GCST90001698 | TRUE | A | 152065838 | 9.72E-09 | reported | 3408 | 39.21 | rs62272737 |  |  |  |  |  |  |  |  |  |  |  |  |  |  |  |
| 212.6 | 3 | igd | 0.0022 | T | \|\| id:ebi-a-GCST90001698 | ebi-a-GCST90001698 | TRUE | C | 185766414 | 5.30E-09 | reported | 3408 | 36.32 | rs55787444 |  |  |  |  |  |  |  |  |  |  |  |  |  |  |  |
| 209.4 | 3 | igd | 0.0022 | T | \|\| id:ebi-a-GCST90001698 | ebi-a-GCST90001698 | TRUE | C | 75412248 | 1.35E-08 | reported | 3408 | 36.78 | rs111769097 |  |  |  |  |  |  |  |  |  |  |  |  |  |  |  |
| 228.2 | 3 | igd | 0.0022 | T | \|\| id:ebi-a-GCST90001698 | ebi-a-GCST90001698 | TRUE | C | 76086471 | 6.97E-10 | reported | 3408 | 36.91 | rs13097663 |  |  |  |  |  |  |  |  |  |  |  |  |  |  |  |
| 340.7 | 4 | igd | 0.0022 | A | \|\| id:ebi-a-GCST90001698 | ebi-a-GCST90001698 | TRUE | C | 30657979 | 1.61E-12 | reported | 3408 | 48.05 | rs181879723 |  |  |  |  |  |  |  |  |  |  |  |  |  |  |  |
| 235.6 | 5 | igd | 0.0022 | G | \|\| id:ebi-a-GCST90001698 | ebi-a-GCST90001698 | TRUE | T | 1957403 | 2.18E-10 | reported | 3408 | 37 | rs62337106 |  |  |  |  |  |  |  |  |  |  |  |  |  |  |  |
| 223.5 | 5 | igd | 0.0022 | G | \|\| id:ebi-a-GCST90001698 | ebi-a-GCST90001698 | TRUE | C | 151998899 | 4.06E-08 | reported | 3408 | 40.63 | rs72797239 |  |  |  |  |  |  |  |  |  |  |  |  |  |  |  |
| 241 | 6 | igd | 0.0022 | T | \|\| id:ebi-a-GCST90001698 | ebi-a-GCST90001698 | TRUE | C | 7216130 | 2.61E-10 | reported | 3408 | 38.01 | rs149764219 |  |  |  |  |  |  |  |  |  |  |  |  |  |  |  |
| 203.1 | 6 | igd | 0.0022 | C | \|\| id:ebi-a-GCST90001698 | ebi-a-GCST90001698 | TRUE | G | 30958409 | 2.44E-08 | reported | 3408 | 36.33 | rs149136965 |  |  |  |  |  |  |  |  |  |  |  |  |  |  |  |
| 239.6 | 6 | igd | 0.0022 | C | \|\| id:ebi-a-GCST90001698 | ebi-a-GCST90001698 | TRUE | T | 64401131 | 5.54E-11 | reported | 3408 | 36.44 | rs75510626 |  |  |  |  |  |  |  |  |  |  |  |  |  |  |  |
| 339.6 | 6 | igd | 0.0022 | T | \|\| id:ebi-a-GCST90001698 | ebi-a-GCST90001698 | TRUE | C | 84898123 | 2.09E-23 | reported | 3408 | 33.81 | rs149828559 |  |  |  |  |  |  |  |  |  |  |  |  |  |  |  |
| 287.5 | 8 | igd | 0.0022 | A | \|\| id:ebi-a-GCST90001698 | ebi-a-GCST90001698 | TRUE | G | 112881674 | 4.63E-10 | reported | 3408 | 46.01 | rs117271894 |  |  |  |  |  |  |  |  |  |  |  |  |  |  |  |
| 269.2 | 8 | igd | 0.0022 | A | \|\| id:ebi-a-GCST90001698 | ebi-a-GCST90001698 | TRUE | T | 142897670 | 2.28E-12 | reported | 3408 | 38.23 | rs117892032 |  |  |  |  |  |  |  |  |  |  |  |  |  |  |  |
| 248.5 | 8 | igd | 0.0022 | A | \|\| id:ebi-a-GCST90001698 | ebi-a-GCST90001698 | TRUE | G | 144562245 | 2.41E-11 | reported | 3408 | 37.09 | rs118076041 |  |  |  |  |  |  |  |  |  |  |  |  |  |  |  |
| 196.1 | 8 | igd | 0.0022 | C | \|\| id:ebi-a-GCST90001698 | ebi-a-GCST90001698 | TRUE | T | 82400020 | 4.08E-08 | reported | 3408 | 35.66 | rs117448261 |  |  |  |  |  |  |  |  |  |  |  |  |  |  |  |
| 260.8 | 9 | igd | 0.0022 | T | \|\| id:ebi-a-GCST90001698 | ebi-a-GCST90001698 | TRUE | C | 132172017 | 4.42E-11 | reported | 3408 | 39.44 | rs145933881 |  |  |  |  |  |  |  |  |  |  |  |  |  |  |  |
| 212.8 | 10 | igd | 0.0022 | C | \|\| id:ebi-a-GCST90001698 | ebi-a-GCST90001698 | TRUE | G | 53794261 | 2.84E-09 | reported | 3408 | 35.72 | rs140589168 |  |  |  |  |  |  |  |  |  |  |  |  |  |  |  |
| 302.9 | 10 | igd | 0.0022 | T | \|\| id:ebi-a-GCST90001698 | ebi-a-GCST90001698 | TRUE | C | 59188637 | 4.04E-14 | reported | 3408 | 39.9 | rs117240457 |  |  |  |  |  |  |  |  |  |  |  |  |  |  |  |
| 212.8 | 11 | igd | 0.0022 | A | \|\| id:ebi-a-GCST90001698 | ebi-a-GCST90001698 | TRUE | G | 95180478 | 2.00E-08 | reported | 3408 | 37.83 | rs75157411 |  |  |  |  |  |  |  |  |  |  |  |  |  |  |  |
| 224.3 | 12 | igd | 0.0022 | C | \|\| id:ebi-a-GCST90001698 | ebi-a-GCST90001698 | TRUE | A | 132501996 | 3.03E-09 | reported | 3408 | 37.72 | rs3923761 |  |  |  |  |  |  |  |  |  |  |  |  |  |  |  |
| 218 | 12 | igd | 0.0022 | C | \|\| id:ebi-a-GCST90001698 | ebi-a-GCST90001698 | TRUE | T | 13466189 | 1.08E-09 | reported | 3408 | 35.65 | rs142326649 |  |  |  |  |  |  |  |  |  |  |  |  |  |  |  |
| 247.8 | 12 | igd | 0.0022 | G | \|\| id:ebi-a-GCST90001698 | ebi-a-GCST90001698 | TRUE | A | 3889066 | 1.66E-10 | reported | 3408 | 38.65 | rs140622705 |  |  |  |  |  |  |  |  |  |  |  |  |  |  |  |
| 231.5 | 16 | igd | 0.0022 | T | \|\| id:ebi-a-GCST90001698 | ebi-a-GCST90001698 | TRUE | C | 21576888 | 1.96E-09 | reported | 3408 | 38.47 | rs370319809 |  |  |  |  |  |  |  |  |  |  |  |  |  |  |  |
| 214.7 | 19 | igd | 0.0022 | C | \|\| id:ebi-a-GCST90001698 | ebi-a-GCST90001698 | TRUE | T | 55017690 | 1.94E-08 | reported | 3408 | 38.13 | rs57121931 |  |  |  |  |  |  |  |  |  |  |  |  |  |  |  |
| 9.036 | 2 | igd | 0.0022 | C | \|\| id:ebi-a-GCST90001700 | ebi-a-GCST90001700 | TRUE | T | 146371302 | 4.84E-08 | reported | 3425 | 1.652 | rs79027575 |  |  |  |  |  |  |  |  |  |  |  |  |  |  |  |
| 8.874 | 2 | igd | 0.0022 | G | \|\| id:ebi-a-GCST90001700 | ebi-a-GCST90001700 | TRUE | A | 235962017 | 1.51E-08 | reported | 3425 | 1.564 | rs114581990 |  |  |  |  |  |  |  |  |  |  |  |  |  |  |  |
| -1.367 | 22 | igd | 0.0022 | T | \|\| id:ebi-a-GCST90001702 | ebi-a-GCST90001702 | TRUE | G | 41514702 | 2.65E-08 | reported | 3657 | 0.2452 | rs140745259 |  |  |  |  |  |  |  |  |  |  |  |  |  |  |  |
| -1.406 | 22 | igd | 0.0022 | T | \|\| id:ebi-a-GCST90001703 | ebi-a-GCST90001703 | TRUE | G | 41514702 | 1.05E-08 | reported | 3657 | 0.2451 | rs140745259 |  |  |  |  |  |  |  |  |  |  |  |  |  |  |  |
| -1.392 | 22 | igd | 0.0022 | T | \|\| id:ebi-a-GCST90001704 | ebi-a-GCST90001704 | TRUE | G | 41514702 | 1.40E-08 | reported | 3657 | 0.2448 | rs140745259 |  |  |  |  |  |  |  |  |  |  |  |  |  |  |  |
| -1.668 | 18 | igd | 0.0022 | T | \|\| id:ebi-a-GCST90001897 | ebi-a-GCST90001897 | TRUE | C | 39778311 | 2.96E-09 | reported | 2914 | 0.2802 | rs75380201 |  |  |  |  |  |  |  |  |  |  |  |  |  |  |  |
| 267.8 | 1 | igd | 0.0021 | G | \|\| id:ebi-a-GCST90001698 | ebi-a-GCST90001698 | TRUE | C | 4591565 | 2.07E-12 | reported | 3408 | 37.95 | rs114672498 |  |  |  |  |  |  |  |  |  |  |  |  |  |  |  |
| 225.7 | 1 | igd | 0.0021 | A | \|\| id:ebi-a-GCST90001698 | ebi-a-GCST90001698 | TRUE | G | 7934382 | 1.38E-09 | reported | 3408 | 37.15 | rs72633042 |  |  |  |  |  |  |  |  |  |  |  |  |  |  |  |
| 262.1 | 1 | igd | 0.0021 | A | \|\| id:ebi-a-GCST90001698 | ebi-a-GCST90001698 | TRUE | G | 204459946 | 1.63E-11 | reported | 3408 | 38.78 | rs148484526 |  |  |  |  |  |  |  |  |  |  |  |  |  |  |  |
| 222.7 | 1 | igd | 0.0021 | G | \|\| id:ebi-a-GCST90001698 | ebi-a-GCST90001698 | TRUE | A | 218059620 | 4.50E-09 | reported | 3408 | 37.88 | rs11117965 |  |  |  |  |  |  |  |  |  |  |  |  |  |  |  |
| 244 | 2 | igd | 0.0021 | G | \|\| id:ebi-a-GCST90001698 | ebi-a-GCST90001698 | TRUE | A | 40546780 | 4.30E-11 | reported | 3408 | 36.89 | rs138152896 |  |  |  |  |  |  |  |  |  |  |  |  |  |  |  |
| 245 | 2 | igd | 0.0021 | C | \|\| id:ebi-a-GCST90001698 | ebi-a-GCST90001698 | TRUE | T | 235591412 | 2.96E-11 | reported | 3408 | 36.72 | rs79205523 |  |  |  |  |  |  |  |  |  |  |  |  |  |  |  |
| 282.9 | 2 | igd | 0.0021 | C | \|\| id:ebi-a-GCST90001698 | ebi-a-GCST90001698 | TRUE | A | 150956800 | 9.60E-14 | reported | 3408 | 37.84 | rs180716798 |  |  |  |  |  |  |  |  |  |  |  |  |  |  |  |
| 237.6 | 3 | igd | 0.0021 | G | \|\| id:ebi-a-GCST90001698 | ebi-a-GCST90001698 | TRUE | A | 14459099 | 9.39E-10 | reported | 3408 | 38.71 | rs111902471 |  |  |  |  |  |  |  |  |  |  |  |  |  |  |  |
| 256.8 | 3 | igd | 0.0021 | C | \|\| id:ebi-a-GCST90001698 | ebi-a-GCST90001698 | TRUE | T | 169040594 | 2.96E-09 | reported | 3408 | 43.16 | rs142666853 |  |  |  |  |  |  |  |  |  |  |  |  |  |  |  |
| 256.6 | 3 | igd | 0.0021 | A | \|\| id:ebi-a-GCST90001698 | ebi-a-GCST90001698 | TRUE | G | 122037000 | 2.57E-12 | reported | 3408 | 36.53 | rs115078330 |  |  |  |  |  |  |  |  |  |  |  |  |  |  |  |
| 219.8 | 4 | igd | 0.0021 | C | \|\| id:ebi-a-GCST90001698 | ebi-a-GCST90001698 | TRUE | A | 29997865 | 5.48E-09 | reported | 3408 | 37.59 | rs140920654 |  |  |  |  |  |  |  |  |  |  |  |  |  |  |  |
| 426.8 | 4 | igd | 0.0021 | A | \|\| id:ebi-a-GCST90001698 | ebi-a-GCST90001698 | TRUE | G | 181744208 | 1.01E-31 | reported | 3408 | 36.05 | rs139578065 |  |  |  |  |  |  |  |  |  |  |  |  |  |  |  |
| 245.7 | 5 | igd | 0.0021 | G | \|\| id:ebi-a-GCST90001698 | ebi-a-GCST90001698 | TRUE | C | 134113453 | 8.80E-09 | reported | 3408 | 42.61 | rs139332323 |  |  |  |  |  |  |  |  |  |  |  |  |  |  |  |
| 260.1 | 5 | igd | 0.0021 | T | \|\| id:ebi-a-GCST90001698 | ebi-a-GCST90001698 | TRUE | C | 178718371 | 1.95E-11 | reported | 3408 | 38.64 | rs116681650 |  |  |  |  |  |  |  |  |  |  |  |  |  |  |  |
| 235 | 6 | igd | 0.0021 | C | \|\| id:ebi-a-GCST90001698 | ebi-a-GCST90001698 | TRUE | G | 118624587 | 8.87E-09 | reported | 3408 | 40.75 | rs117142908 |  |  |  |  |  |  |  |  |  |  |  |  |  |  |  |
| 239.5 | 7 | igd | 0.0021 | G | \|\| id:ebi-a-GCST90001698 | ebi-a-GCST90001698 | TRUE | T | 149552333 | 9.97E-10 | reported | 3408 | 39.09 | rs111743964 |  |  |  |  |  |  |  |  |  |  |  |  |  |  |  |
| 260.9 | 8 | igd | 0.0021 | A | \|\| id:ebi-a-GCST90001698 | ebi-a-GCST90001698 | TRUE | G | 112234506 | 1.43E-10 | reported | 3408 | 40.57 | rs117741105 |  |  |  |  |  |  |  |  |  |  |  |  |  |  |  |
| 229.3 | 8 | igd | 0.0021 | C | \|\| id:ebi-a-GCST90001698 | ebi-a-GCST90001698 | TRUE | G | 129915383 | 4.76E-09 | reported | 3408 | 39.06 | rs117123362 |  |  |  |  |  |  |  |  |  |  |  |  |  |  |  |
| 253.3 | 8 | igd | 0.0021 | A | \|\| id:ebi-a-GCST90001698 | ebi-a-GCST90001698 | TRUE | G | 30627573 | 5.64E-11 | reported | 3408 | 38.53 | rs183280627 |  |  |  |  |  |  |  |  |  |  |  |  |  |  |  |
| 225.6 | 9 | igd | 0.0021 | C | \|\| id:ebi-a-GCST90001698 | ebi-a-GCST90001698 | TRUE | G | 115853052 | 8.26E-09 | reported | 3408 | 39.06 | rs151186194 |  |  |  |  |  |  |  |  |  |  |  |  |  |  |  |
| 215.2 | 9 | igd | 0.0021 | G | \|\| id:ebi-a-GCST90001698 | ebi-a-GCST90001698 | TRUE | A | 83435830 | 4.46E-08 | reported | 3408 | 39.24 | rs117702997 |  |  |  |  |  |  |  |  |  |  |  |  |  |  |  |
| 340.9 | 12 | igd | 0.0021 | G | \|\| id:ebi-a-GCST90001698 | ebi-a-GCST90001698 | TRUE | A | 1692305 | 1.65E-15 | reported | 3408 | 42.59 | rs192351072 |  |  |  |  |  |  |  |  |  |  |  |  |  |  |  |
| 279.2 | 12 | igd | 0.0021 | A | \|\| id:ebi-a-GCST90001698 | ebi-a-GCST90001698 | TRUE | G | 129545747 | 5.42E-14 | reported | 3408 | 36.97 | rs143435133 |  |  |  |  |  |  |  |  |  |  |  |  |  |  |  |
| 257 | 13 | igd | 0.0021 | G | \|\| id:ebi-a-GCST90001698 | ebi-a-GCST90001698 | TRUE | A | 99034552 | 1.78E-11 | reported | 3408 | 38.1 | rs3848018 |  |  |  |  |  |  |  |  |  |  |  |  |  |  |  |
| 214.3 | 17 | igd | 0.0021 | A | \|\| id:ebi-a-GCST90001698 | ebi-a-GCST90001698 | TRUE | C | 31685258 | 2.41E-08 | reported | 3408 | 38.32 | rs146807102 |  |  |  |  |  |  |  |  |  |  |  |  |  |  |  |
| 338.4 | 19 | igd | 0.0021 | A | \|\| id:ebi-a-GCST90001698 | ebi-a-GCST90001698 | TRUE | G | 22454852 | 5.64E-16 | reported | 3408 | 41.59 | rs146650864 |  |  |  |  |  |  |  |  |  |  |  |  |  |  |  |
| 260.3 | 20 | igd | 0.0021 | A | \|\| id:ebi-a-GCST90001698 | ebi-a-GCST90001698 | TRUE | C | 40384344 | 5.06E-11 | reported | 3408 | 39.49 | rs112014600 |  |  |  |  |  |  |  |  |  |  |  |  |  |  |  |
| 214.8 | 20 | igd | 0.0021 | T | \|\| id:ebi-a-GCST90001698 | ebi-a-GCST90001698 | TRUE | C | 21420694 | 1.10E-08 | reported | 3408 | 37.5 | rs113369914 |  |  |  |  |  |  |  |  |  |  |  |  |  |  |  |
| 222.2 | 20 | igd | 0.0021 | T | \|\| id:ebi-a-GCST90001698 | ebi-a-GCST90001698 | TRUE | C | 30658042 | 1.64E-08 | reported | 3408 | 39.26 | rs79541727 |  |  |  |  |  |  |  |  |  |  |  |  |  |  |  |
| 296 | 20 | igd | 0.0021 | A | \|\| id:ebi-a-GCST90001698 | ebi-a-GCST90001698 | TRUE | G | 37708329 | 1.92E-13 | reported | 3408 | 40.08 | rs141848350 |  |  |  |  |  |  |  |  |  |  |  |  |  |  |  |
| 223.1 | 22 | igd | 0.0021 | T | \|\| id:ebi-a-GCST90001698 | ebi-a-GCST90001698 | TRUE | G | 34529587 | 3.90E-08 | reported | 3408 | 40.5 | rs190539465 |  |  |  |  |  |  |  |  |  |  |  |  |  |  |  |
| 246.8 | 22 | igd | 0.0021 | A | \|\| id:ebi-a-GCST90001698 | ebi-a-GCST90001698 | TRUE | C | 36506460 | 7.63E-11 | reported | 3408 | 37.81 | rs117840149 |  |  |  |  |  |  |  |  |  |  |  |  |  |  |  |
| 210.2 | 16 | igd | 0.0019 | A | \|\| id:ebi-a-GCST90001695 | ebi-a-GCST90001695 | TRUE | G | 79757046 | 3.44E-12 | reported | 3408 | 30.1 | rs116941636 |  |  |  |  |  |  |  |  |  |  |  |  |  |  |  |
| 8.79 | 2 | igd | 0.0019 | A | \|\| id:ebi-a-GCST90001697 | ebi-a-GCST90001697 | TRUE | T | 12784208 | 3.28E-10 | reported | 3425 | 1.394 | rs150146567 |  |  |  |  |  |  |  |  |  |  |  |  |  |  |  |
| 277.6 | 1 | igd | 0.0019 | A | \|\| id:ebi-a-GCST90001698 | ebi-a-GCST90001698 | TRUE | C | 3566631 | 6.13E-12 | reported | 3408 | 40.22 | rs375432272 |  |  |  |  |  |  |  |  |  |  |  |  |  |  |  |
| 274.9 | 1 | igd | 0.0019 | C | \|\| id:ebi-a-GCST90001698 | ebi-a-GCST90001698 | TRUE | T | 110414036 | 5.05E-12 | reported | 3408 | 39.67 | rs12085671 |  |  |  |  |  |  |  |  |  |  |  |  |  |  |  |
| 276 | 1 | igd | 0.0019 | T | \|\| id:ebi-a-GCST90001698 | ebi-a-GCST90001698 | TRUE | C | 47203126 | 6.73E-12 | reported | 3408 | 40.08 | rs12027410 |  |  |  |  |  |  |  |  |  |  |  |  |  |  |  |
| 342.3 | 1 | igd | 0.0019 | A | \|\| id:ebi-a-GCST90001698 | ebi-a-GCST90001698 | TRUE | G | 100459655 | 1.66E-12 | reported | 3408 | 48.3 | rs115175419 |  |  |  |  |  |  |  |  |  |  |  |  |  |  |  |
| 260.9 | 1 | igd | 0.0019 | G | \|\| id:ebi-a-GCST90001698 | ebi-a-GCST90001698 | TRUE | A | 247511918 | 8.87E-10 | reported | 3408 | 42.46 | rs141709616 |  |  |  |  |  |  |  |  |  |  |  |  |  |  |  |
| 219.9 | 1 | igd | 0.0019 | T | \|\| id:ebi-a-GCST90001698 | ebi-a-GCST90001698 | TRUE | C | 186774402 | 2.94E-08 | reported | 3408 | 39.58 | rs144685135 |  |  |  |  |  |  |  |  |  |  |  |  |  |  |  |
| 311.8 | 2 | igd | 0.0019 | A | \|\| id:ebi-a-GCST90001698 | ebi-a-GCST90001698 | TRUE | T | 225713694 | 6.35E-14 | reported | 3408 | 41.4 | rs116012594 |  |  |  |  |  |  |  |  |  |  |  |  |  |  |  |
| 353.7 | 2 | igd | 0.0019 | G | \|\| id:ebi-a-GCST90001698 | ebi-a-GCST90001698 | TRUE | T | 75148081 | 1.02E-18 | reported | 3408 | 39.81 | rs4853078 |  |  |  |  |  |  |  |  |  |  |  |  |  |  |  |
| 236.4 | 2 | igd | 0.0019 | A | \|\| id:ebi-a-GCST90001698 | ebi-a-GCST90001698 | TRUE | C | 145532139 | 5.59E-09 | reported | 3408 | 40.45 | rs143938461 |  |  |  |  |  |  |  |  |  |  |  |  |  |  |  |
| 292.3 | 2 | igd | 0.0019 | C | \|\| id:ebi-a-GCST90001698 | ebi-a-GCST90001698 | TRUE | T | 41540149 | 6.43E-13 | reported | 3408 | 40.49 | rs112137766 |  |  |  |  |  |  |  |  |  |  |  |  |  |  |  |
| 254.2 | 3 | igd | 0.0019 | A | \|\| id:ebi-a-GCST90001698 | ebi-a-GCST90001698 | TRUE | T | 105559499 | 6.65E-09 | reported | 3408 | 43.72 | rs144575081 |  |  |  |  |  |  |  |  |  |  |  |  |  |  |  |
| 217.3 | 3 | igd | 0.0019 | T | \|\| id:ebi-a-GCST90001698 | ebi-a-GCST90001698 | TRUE | C | 84637309 | 1.14E-08 | reported | 3408 | 37.98 | rs114065818 |  |  |  |  |  |  |  |  |  |  |  |  |  |  |  |
| 246.7 | 3 | igd | 0.0019 | A | \|\| id:ebi-a-GCST90001698 | ebi-a-GCST90001698 | TRUE | T | 186195530 | 5.47E-11 | reported | 3408 | 37.5 | rs78193714 |  |  |  |  |  |  |  |  |  |  |  |  |  |  |  |
| 326.2 | 4 | igd | 0.0019 | T | \|\| id:ebi-a-GCST90001698 | ebi-a-GCST90001698 | TRUE | C | 176618043 | 3.23E-17 | reported | 3408 | 38.45 | rs17657144 |  |  |  |  |  |  |  |  |  |  |  |  |  |  |  |
| 322.4 | 4 | igd | 0.0019 | T | \|\| id:ebi-a-GCST90001698 | ebi-a-GCST90001698 | TRUE | C | 130934266 | 2.24E-16 | reported | 3408 | 39.08 | rs147841321 |  |  |  |  |  |  |  |  |  |  |  |  |  |  |  |
| 232.2 | 4 | igd | 0.0019 | A | \|\| id:ebi-a-GCST90001698 | ebi-a-GCST90001698 | TRUE | T | 132202830 | 3.99E-10 | reported | 3408 | 37.01 | rs76274371 |  |  |  |  |  |  |  |  |  |  |  |  |  |  |  |
| 245.2 | 4 | igd | 0.0019 | T | \|\| id:ebi-a-GCST90001698 | ebi-a-GCST90001698 | TRUE | C | 87102249 | 1.85E-10 | reported | 3408 | 38.35 | rs1460765 |  |  |  |  |  |  |  |  |  |  |  |  |  |  |  |
| 248.3 | 4 | igd | 0.0019 | T | \|\| id:ebi-a-GCST90001698 | ebi-a-GCST90001698 | TRUE | C | 176251569 | 1.99E-10 | reported | 3408 | 38.91 | rs79742404 |  |  |  |  |  |  |  |  |  |  |  |  |  |  |  |
| 231.3 | 5 | igd | 0.0019 | A | \|\| id:ebi-a-GCST90001698 | ebi-a-GCST90001698 | TRUE | G | 29518230 | 3.90E-09 | reported | 3408 | 39.18 | rs62354927 |  |  |  |  |  |  |  |  |  |  |  |  |  |  |  |
| 239.1 | 5 | igd | 0.0019 | G | \|\| id:ebi-a-GCST90001698 | ebi-a-GCST90001698 | TRUE | T | 24165437 | 2.24E-09 | reported | 3408 | 39.88 | rs114871166 |  |  |  |  |  |  |  |  |  |  |  |  |  |  |  |
| 263.3 | 6 | igd | 0.0019 | A | \|\| id:ebi-a-GCST90001698 | ebi-a-GCST90001698 | TRUE | G | 1765867 | 6.20E-11 | reported | 3408 | 40.13 | rs72841914 |  |  |  |  |  |  |  |  |  |  |  |  |  |  |  |
| 264.9 | 7 | igd | 0.0019 | A | \|\| id:ebi-a-GCST90001698 | ebi-a-GCST90001698 | TRUE | G | 6934892 | 1.67E-11 | reported | 3408 | 39.21 | rs117433099 |  |  |  |  |  |  |  |  |  |  |  |  |  |  |  |
| 305.8 | 7 | igd | 0.0019 | A | \|\| id:ebi-a-GCST90001698 | ebi-a-GCST90001698 | TRUE | G | 84609804 | 2.64E-13 | reported | 3408 | 41.66 | rs73179823 |  |  |  |  |  |  |  |  |  |  |  |  |  |  |  |
| 395.7 | 8 | igd | 0.0019 | A | \|\| id:ebi-a-GCST90001698 | ebi-a-GCST90001698 | TRUE | C | 12500832 | 4.89E-19 | reported | 3408 | 44.12 | rs34039708 |  |  |  |  |  |  |  |  |  |  |  |  |  |  |  |
| 345 | 8 | igd | 0.0019 | T | \|\| id:ebi-a-GCST90001698 | ebi-a-GCST90001698 | TRUE | C | 144101087 | 6.11E-16 | reported | 3408 | 42.46 | rs117809561 |  |  |  |  |  |  |  |  |  |  |  |  |  |  |  |
| 220.6 | 9 | igd | 0.0019 | T | \|\| id:ebi-a-GCST90001698 | ebi-a-GCST90001698 | TRUE | C | 134761700 | 3.26E-08 | reported | 3408 | 39.83 | rs78937059 |  |  |  |  |  |  |  |  |  |  |  |  |  |  |  |
| 275 | 10 | igd | 0.0019 | T | \|\| id:ebi-a-GCST90001698 | ebi-a-GCST90001698 | TRUE | C | 119779310 | 1.11E-11 | reported | 3408 | 40.35 | rs144684742 |  |  |  |  |  |  |  |  |  |  |  |  |  |  |  |
| 221.2 | 10 | igd | 0.0019 | A | \|\| id:ebi-a-GCST90001698 | ebi-a-GCST90001698 | TRUE | G | 82437388 | 4.44E-08 | reported | 3408 | 40.33 | rs184899141 |  |  |  |  |  |  |  |  |  |  |  |  |  |  |  |
| 407.5 | 10 | igd | 0.0019 | T | \|\| id:ebi-a-GCST90001698 | ebi-a-GCST90001698 | TRUE | G | 53915868 | 2.65E-23 | reported | 3408 | 40.68 | rs10824158 |  |  |  |  |  |  |  |  |  |  |  |  |  |  |  |
| 383.1 | 10 | igd | 0.0019 | G | \|\| id:ebi-a-GCST90001698 | ebi-a-GCST90001698 | TRUE | A | 119966169 | 5.34E-22 | reported | 3408 | 39.46 | rs575794055 |  |  |  |  |  |  |  |  |  |  |  |  |  |  |  |
| 218.4 | 12 | igd | 0.0019 | G | \|\| id:ebi-a-GCST90001698 | ebi-a-GCST90001698 | TRUE | T | 17156413 | 3.92E-08 | reported | 3408 | 39.67 | rs533748626 |  |  |  |  |  |  |  |  |  |  |  |  |  |  |  |
| 271.1 | 12 | igd | 0.0019 | A | \|\| id:ebi-a-GCST90001698 | ebi-a-GCST90001698 | TRUE | G | 117089403 | 2.02E-11 | reported | 3408 | 40.3 | rs184910025 |  |  |  |  |  |  |  |  |  |  |  |  |  |  |  |
| 249.4 | 12 | igd | 0.0019 | C | \|\| id:ebi-a-GCST90001698 | ebi-a-GCST90001698 | TRUE | T | 119037578 | 5.14E-09 | reported | 3408 | 42.57 | rs112121527 |  |  |  |  |  |  |  |  |  |  |  |  |  |  |  |
| 268.7 | 12 | igd | 0.0019 | T | \|\| id:ebi-a-GCST90001698 | ebi-a-GCST90001698 | TRUE | C | 14480598 | 7.94E-12 | reported | 3408 | 39.15 | rs111733718 |  |  |  |  |  |  |  |  |  |  |  |  |  |  |  |
| 254.8 | 13 | igd | 0.0019 | A | \|\| id:ebi-a-GCST90001698 | ebi-a-GCST90001698 | TRUE | G | 76613080 | 5.02E-10 | reported | 3408 | 40.85 | rs139114055 |  |  |  |  |  |  |  |  |  |  |  |  |  |  |  |
| 260.8 | 14 | igd | 0.0019 | T | \|\| id:ebi-a-GCST90001698 | ebi-a-GCST90001698 | TRUE | A | 33586273 | 6.23E-11 | reported | 3408 | 39.75 | rs117862145 |  |  |  |  |  |  |  |  |  |  |  |  |  |  |  |
| 228.4 | 15 | igd | 0.0019 | T | \|\| id:ebi-a-GCST90001698 | ebi-a-GCST90001698 | TRUE | C | 33948485 | 2.91E-09 | reported | 3408 | 38.37 | rs143110935 |  |  |  |  |  |  |  |  |  |  |  |  |  |  |  |
| 274.8 | 16 | igd | 0.0019 | A | \|\| id:ebi-a-GCST90001698 | ebi-a-GCST90001698 | TRUE | C | 1071217 | 1.69E-12 | reported | 3408 | 38.79 | rs142151887 |  |  |  |  |  |  |  |  |  |  |  |  |  |  |  |
| 273.6 | 20 | igd | 0.0019 | A | \|\| id:ebi-a-GCST90001698 | ebi-a-GCST90001698 | TRUE | G | 33943897 | 2.34E-12 | reported | 3408 | 38.88 | rs62211528 |  |  |  |  |  |  |  |  |  |  |  |  |  |  |  |
| 229.2 | 20 | igd | 0.0019 | G | \|\| id:ebi-a-GCST90001698 | ebi-a-GCST90001698 | TRUE | A | 25018520 | 1.03E-08 | reported | 3408 | 39.93 | rs139665738 |  |  |  |  |  |  |  |  |  |  |  |  |  |  |  |
| 9.274 | 2 | igd | 0.0019 | G | \|\| id:ebi-a-GCST90001700 | ebi-a-GCST90001700 | TRUE | T | 75148081 | 3.74E-08 | reported | 3425 | 1.681 | rs4853078 |  |  |  |  |  |  |  |  |  |  |  |  |  |  |  |
| 12.51 | 8 | igd | 0.0019 | A | \|\| id:ebi-a-GCST90001700 | ebi-a-GCST90001700 | TRUE | C | 12500832 | 1.58E-11 | reported | 3425 | 1.85 | rs34039708 |  |  |  |  |  |  |  |  |  |  |  |  |  |  |  |
| 9.768 | 10 | igd | 0.0019 | T | \|\| id:ebi-a-GCST90001700 | ebi-a-GCST90001700 | TRUE | G | 53915868 | 1.62E-08 | reported | 3425 | 1.725 | rs10824158 |  |  |  |  |  |  |  |  |  |  |  |  |  |  |  |
| 224.8 | 5 | igd | 0.0018 | C | \|\| id:ebi-a-GCST90001695 | ebi-a-GCST90001695 | TRUE | T | 81353920 | 2.83E-12 | reported | 3408 | 32.07 | rs76343453 |  |  |  |  |  |  |  |  |  |  |  |  |  |  |  |
| 201.5 | 9 | igd | 0.0018 | A | \|\| id:ebi-a-GCST90001695 | ebi-a-GCST90001695 | TRUE | G | 83853182 | 4.11E-10 | reported | 3408 | 32.14 | rs117196114 |  |  |  |  |  |  |  |  |  |  |  |  |  |  |  |
| 10.98 | 5 | igd | 0.0018 | C | \|\| id:ebi-a-GCST90001697 | ebi-a-GCST90001697 | TRUE | T | 81353920 | 2.98E-13 | reported | 3425 | 1.499 | rs76343453 |  |  |  |  |  |  |  |  |  |  |  |  |  |  |  |
| 270.7 | 1 | igd | 0.0018 | T | \|\| id:ebi-a-GCST90001698 | ebi-a-GCST90001698 | TRUE | C | 112269677 | 7.32E-11 | reported | 3408 | 41.43 | rs34772471 |  |  |  |  |  |  |  |  |  |  |  |  |  |  |  |
| 264 | 1 | igd | 0.0018 | G | \|\| id:ebi-a-GCST90001698 | ebi-a-GCST90001698 | TRUE | A | 110742985 | 8.16E-11 | reported | 3408 | 40.5 | rs77538429 |  |  |  |  |  |  |  |  |  |  |  |  |  |  |  |
| 241.7 | 2 | igd | 0.0018 | A | \|\| id:ebi-a-GCST90001698 | ebi-a-GCST90001698 | TRUE | G | 31343284 | 1.38E-09 | reported | 3408 | 39.78 | rs112320586 |  |  |  |  |  |  |  |  |  |  |  |  |  |  |  |
| 366 | 2 | igd | 0.0018 | C | \|\| id:ebi-a-GCST90001698 | ebi-a-GCST90001698 | TRUE | A | 17719245 | 7.76E-15 | reported | 3408 | 46.88 | rs548679517 |  |  |  |  |  |  |  |  |  |  |  |  |  |  |  |
| 262.1 | 2 | igd | 0.0018 | G | \|\| id:ebi-a-GCST90001698 | ebi-a-GCST90001698 | TRUE | A | 122142931 | 4.23E-10 | reported | 3408 | 41.84 | rs146750948 |  |  |  |  |  |  |  |  |  |  |  |  |  |  |  |
| 278.8 | 2 | igd | 0.0018 | A | \|\| id:ebi-a-GCST90001698 | ebi-a-GCST90001698 | TRUE | C | 104590904 | 7.23E-11 | reported | 3408 | 42.64 | rs77156603 |  |  |  |  |  |  |  |  |  |  |  |  |  |  |  |
| 313.5 | 3 | igd | 0.0018 | T | \|\| id:ebi-a-GCST90001698 | ebi-a-GCST90001698 | TRUE | C | 76660068 | 7.66E-14 | reported | 3408 | 41.76 | rs146756366 |  |  |  |  |  |  |  |  |  |  |  |  |  |  |  |
| 274.8 | 3 | igd | 0.0018 | T | \|\| id:ebi-a-GCST90001698 | ebi-a-GCST90001698 | TRUE | C | 74527776 | 8.39E-12 | reported | 3408 | 40.08 | rs115212782 |  |  |  |  |  |  |  |  |  |  |  |  |  |  |  |
| 243.1 | 3 | igd | 0.0018 | A | \|\| id:ebi-a-GCST90001698 | ebi-a-GCST90001698 | TRUE | G | 168867417 | 1.98E-08 | reported | 3408 | 43.21 | rs34281122 |  |  |  |  |  |  |  |  |  |  |  |  |  |  |  |
| 225.9 | 3 | igd | 0.0018 | T | \|\| id:ebi-a-GCST90001698 | ebi-a-GCST90001698 | TRUE | C | 11075184 | 3.37E-08 | reported | 3408 | 40.82 | rs78313301 |  |  |  |  |  |  |  |  |  |  |  |  |  |  |  |
| 338.9 | 4 | igd | 0.0018 | C | \|\| id:ebi-a-GCST90001698 | ebi-a-GCST90001698 | TRUE | A | 131041099 | 7.89E-17 | reported | 3408 | 40.47 | rs77814979 |  |  |  |  |  |  |  |  |  |  |  |  |  |  |  |
| 470.3 | 4 | igd | 0.0018 | G | \|\| id:ebi-a-GCST90001698 | ebi-a-GCST90001698 | TRUE | A | 180746796 | 1.10E-33 | reported | 3408 | 38.46 | rs144034514 |  |  |  |  |  |  |  |  |  |  |  |  |  |  |  |
| 264.7 | 4 | igd | 0.0018 | G | \|\| id:ebi-a-GCST90001698 | ebi-a-GCST90001698 | TRUE | T | 133809883 | 1.38E-09 | reported | 3408 | 43.58 | rs147248733 |  |  |  |  |  |  |  |  |  |  |  |  |  |  |  |
| 225.4 | 5 | igd | 0.0018 | C | \|\| id:ebi-a-GCST90001698 | ebi-a-GCST90001698 | TRUE | A | 141469416 | 3.49E-08 | reported | 3408 | 40.79 | rs146314225 |  |  |  |  |  |  |  |  |  |  |  |  |  |  |  |
| 295 | 5 | igd | 0.0018 | A | \|\| id:ebi-a-GCST90001698 | ebi-a-GCST90001698 | TRUE | G | 152120667 | 2.80E-10 | reported | 3408 | 46.61 | rs190724856 |  |  |  |  |  |  |  |  |  |  |  |  |  |  |  |
| 308.9 | 5 | igd | 0.0018 | A | \|\| id:ebi-a-GCST90001698 | ebi-a-GCST90001698 | TRUE | G | 3635021 | 4.29E-12 | reported | 3408 | 44.43 | rs77587134 |  |  |  |  |  |  |  |  |  |  |  |  |  |  |  |
| 232.9 | 6 | igd | 0.0018 | A | \|\| id:ebi-a-GCST90001698 | ebi-a-GCST90001698 | TRUE | T | 4783496 | 1.42E-08 | reported | 3408 | 40.97 | rs9504251 |  |  |  |  |  |  |  |  |  |  |  |  |  |  |  |
| 264.5 | 7 | igd | 0.0018 | C | \|\| id:ebi-a-GCST90001698 | ebi-a-GCST90001698 | TRUE | T | 130015968 | 2.11E-10 | reported | 3408 | 41.51 | rs140950248 |  |  |  |  |  |  |  |  |  |  |  |  |  |  |  |
| 299.6 | 7 | igd | 0.0018 | T | \|\| id:ebi-a-GCST90001698 | ebi-a-GCST90001698 | TRUE | C | 136855654 | 4.15E-13 | reported | 3408 | 41.15 | rs150400236 |  |  |  |  |  |  |  |  |  |  |  |  |  |  |  |
| 316.1 | 7 | igd | 0.0018 | C | \|\| id:ebi-a-GCST90001698 | ebi-a-GCST90001698 | TRUE | T | 77661026 | 2.01E-13 | reported | 3408 | 42.84 | rs62461160 |  |  |  |  |  |  |  |  |  |  |  |  |  |  |  |
| 301.1 | 8 | igd | 0.0018 | G | \|\| id:ebi-a-GCST90001698 | ebi-a-GCST90001698 | TRUE | A | 42060072 | 2.29E-12 | reported | 3408 | 42.76 | rs8178689 |  |  |  |  |  |  |  |  |  |  |  |  |  |  |  |
| 310 | 9 | igd | 0.0018 | A | \|\| id:ebi-a-GCST90001698 | ebi-a-GCST90001698 | TRUE | T | 25887570 | 3.59E-13 | reported | 3408 | 42.47 | rs151035277 |  |  |  |  |  |  |  |  |  |  |  |  |  |  |  |
| 240.5 | 9 | igd | 0.0018 | G | \|\| id:ebi-a-GCST90001698 | ebi-a-GCST90001698 | TRUE | A | 6764265 | 1.95E-08 | reported | 3408 | 42.72 | rs146141101 |  |  |  |  |  |  |  |  |  |  |  |  |  |  |  |
| 378.5 | 11 | igd | 0.0018 | T | \|\| id:ebi-a-GCST90001698 | ebi-a-GCST90001698 | TRUE | G | 86306354 | 2.06E-19 | reported | 3408 | 41.75 | rs564907878 |  |  |  |  |  |  |  |  |  |  |  |  |  |  |  |
| 269.8 | 11 | igd | 0.0018 | A | \|\| id:ebi-a-GCST90001698 | ebi-a-GCST90001698 | TRUE | T | 70599488 | 8.23E-11 | reported | 3408 | 41.4 | rs77695406 |  |  |  |  |  |  |  |  |  |  |  |  |  |  |  |
| 353.5 | 13 | igd | 0.0018 | A | \|\| id:ebi-a-GCST90001698 | ebi-a-GCST90001698 | TRUE | G | 114441229 | 8.66E-17 | reported | 3408 | 42.26 | rs188618717 |  |  |  |  |  |  |  |  |  |  |  |  |  |  |  |
| 289.8 | 13 | igd | 0.0018 | A | \|\| id:ebi-a-GCST90001698 | ebi-a-GCST90001698 | TRUE | T | 40914557 | 3.67E-12 | reported | 3408 | 41.55 | rs113007178 |  |  |  |  |  |  |  |  |  |  |  |  |  |  |  |
| 286 | 13 | igd | 0.0018 | C | \|\| id:ebi-a-GCST90001698 | ebi-a-GCST90001698 | TRUE | T | 106788553 | 9.79E-13 | reported | 3408 | 39.94 | rs76039100 |  |  |  |  |  |  |  |  |  |  |  |  |  |  |  |
| 399.5 | 14 | igd | 0.0018 | G | \|\| id:ebi-a-GCST90001698 | ebi-a-GCST90001698 | TRUE | T | 46740502 | 1.07E-13 | reported | 3408 | 53.53 | rs146937045 |  |  |  |  |  |  |  |  |  |  |  |  |  |  |  |
| 234.7 | 15 | igd | 0.0018 | G | \|\| id:ebi-a-GCST90001698 | ebi-a-GCST90001698 | TRUE | C | 45262839 | 5.66E-09 | reported | 3408 | 40.18 | rs115746888 |  |  |  |  |  |  |  |  |  |  |  |  |  |  |  |
| 271.8 | 15 | igd | 0.0018 | A | \|\| id:ebi-a-GCST90001698 | ebi-a-GCST90001698 | TRUE | C | 60441655 | 7.25E-11 | reported | 3408 | 41.58 | rs117247453 |  |  |  |  |  |  |  |  |  |  |  |  |  |  |  |
| 306.4 | 18 | igd | 0.0018 | C | \|\| id:ebi-a-GCST90001698 | ebi-a-GCST90001698 | TRUE | G | 73678862 | 6.88E-13 | reported | 3408 | 42.5 | rs139733728 |  |  |  |  |  |  |  |  |  |  |  |  |  |  |  |
| 362.8 | 18 | igd | 0.0018 | T | \|\| id:ebi-a-GCST90001698 | ebi-a-GCST90001698 | TRUE | A | 65092573 | 5.51E-15 | reported | 3408 | 46.21 | rs73966235 |  |  |  |  |  |  |  |  |  |  |  |  |  |  |  |
| 246.6 | 18 | igd | 0.0018 | C | \|\| id:ebi-a-GCST90001698 | ebi-a-GCST90001698 | TRUE | T | 49292661 | 2.27E-09 | reported | 3408 | 41.15 | rs144945962 |  |  |  |  |  |  |  |  |  |  |  |  |  |  |  |
| 516.5 | 19 | igd | 0.0018 | T | \|\| id:ebi-a-GCST90001698 | ebi-a-GCST90001698 | TRUE | C | 24477916 | 1.25E-22 | reported | 3408 | 52.39 | rs118089863 |  |  |  |  |  |  |  |  |  |  |  |  |  |  |  |
| 238.6 | 19 | igd | 0.0018 | T | \|\| id:ebi-a-GCST90001698 | ebi-a-GCST90001698 | TRUE | G | 27781976 | 3.53E-08 | reported | 3408 | 43.17 | rs75608802 |  |  |  |  |  |  |  |  |  |  |  |  |  |  |  |
| 241.7 | 20 | igd | 0.0018 | A | \|\| id:ebi-a-GCST90001698 | ebi-a-GCST90001698 | TRUE | G | 31038623 | 4.58E-09 | reported | 3408 | 41.13 | rs370726978 |  |  |  |  |  |  |  |  |  |  |  |  |  |  |  |
| 267.2 | 20 | igd | 0.0018 | A | \|\| id:ebi-a-GCST90001698 | ebi-a-GCST90001698 | TRUE | T | 18880832 | 4.68E-11 | reported | 3408 | 40.47 | rs184911123 |  |  |  |  |  |  |  |  |  |  |  |  |  |  |  |
| 303.1 | 20 | igd | 0.0018 | A | \|\| id:ebi-a-GCST90001698 | ebi-a-GCST90001698 | TRUE | G | 24785625 | 1.68E-13 | reported | 3408 | 40.95 | rs117418627 |  |  |  |  |  |  |  |  |  |  |  |  |  |  |  |
| 318.2 | 20 | igd | 0.0018 | G | \|\| id:ebi-a-GCST90001698 | ebi-a-GCST90001698 | TRUE | A | 31298488 | 4.09E-13 | reported | 3408 | 43.7 | rs143802121 |  |  |  |  |  |  |  |  |  |  |  |  |  |  |  |
| 282.1 | 20 | igd | 0.0018 | T | \|\| id:ebi-a-GCST90001698 | ebi-a-GCST90001698 | TRUE | C | 61268143 | 9.20E-11 | reported | 3408 | 43.4 | rs148513824 |  |  |  |  |  |  |  |  |  |  |  |  |  |  |  |
| 332.6 | 21 | igd | 0.0018 | C | \|\| id:ebi-a-GCST90001698 | ebi-a-GCST90001698 | TRUE | T | 14574280 | 1.34E-10 | reported | 3408 | 51.63 | rs149097929 |  |  |  |  |  |  |  |  |  |  |  |  |  |  |  |
| 267.8 | 22 | igd | 0.0018 | T | \|\| id:ebi-a-GCST90001698 | ebi-a-GCST90001698 | TRUE | C | 35958024 | 1.30E-09 | reported | 3408 | 44.02 | rs118111037 |  |  |  |  |  |  |  |  |  |  |  |  |  |  |  |
| 12.62 | 19 | igd | 0.0018 | T | \|\| id:ebi-a-GCST90001700 | ebi-a-GCST90001700 | TRUE | C | 24477916 | 1.98E-08 | reported | 3425 | 2.243 | rs118089863 |  |  |  |  |  |  |  |  |  |  |  |  |  |  |  |
| 2.431 | 1 | igd | 0.0016 | T | \|\| id:ebi-a-GCST90001531 | ebi-a-GCST90001531 | TRUE | C | 185852065 | 9.41E-09 | reported | 1856 | 0.4216 | rs114590598 |  |  |  |  |  |  |  |  |  |  |  |  |  |  |  |
| 187 | 1 | igd | 0.0016 | A | \|\| id:ebi-a-GCST90001695 | ebi-a-GCST90001695 | TRUE | G | 221000197 | 1.74E-08 | reported | 3408 | 33.11 | rs76732756 |  |  |  |  |  |  |  |  |  |  |  |  |  |  |  |
| 301 | 1 | igd | 0.0016 | G | \|\| id:ebi-a-GCST90001698 | ebi-a-GCST90001698 | TRUE | A | 47595103 | 5.38E-12 | reported | 3408 | 43.5 | rs147591823 |  |  |  |  |  |  |  |  |  |  |  |  |  |  |  |
| 312.5 | 1 | igd | 0.0016 | T | \|\| id:ebi-a-GCST90001698 | ebi-a-GCST90001698 | TRUE | G | 246645088 | 2.16E-12 | reported | 3408 | 44.32 | rs567145695 |  |  |  |  |  |  |  |  |  |  |  |  |  |  |  |
| 325.9 | 2 | igd | 0.0016 | G | \|\| id:ebi-a-GCST90001698 | ebi-a-GCST90001698 | TRUE | A | 27386488 | 4.49E-13 | reported | 3408 | 44.84 | rs140246558 |  |  |  |  |  |  |  |  |  |  |  |  |  |  |  |
| 318.6 | 3 | igd | 0.0016 | A | \|\| id:ebi-a-GCST90001698 | ebi-a-GCST90001698 | TRUE | G | 128554746 | 1.16E-11 | reported | 3408 | 46.8 | rs115002116 |  |  |  |  |  |  |  |  |  |  |  |  |  |  |  |
| 314.7 | 4 | igd | 0.0016 | G | \|\| id:ebi-a-GCST90001698 | ebi-a-GCST90001698 | TRUE | C | 181659780 | 5.69E-12 | reported | 3408 | 45.54 | rs74289069 |  |  |  |  |  |  |  |  |  |  |  |  |  |  |  |
| 315.7 | 4 | igd | 0.0016 | T | \|\| id:ebi-a-GCST90001698 | ebi-a-GCST90001698 | TRUE | C | 167555345 | 7.06E-14 | reported | 3408 | 41.99 | rs115677012 |  |  |  |  |  |  |  |  |  |  |  |  |  |  |  |
| 235.3 | 5 | igd | 0.0016 | T | \|\| id:ebi-a-GCST90001698 | ebi-a-GCST90001698 | TRUE | C | 154015729 | 3.85E-08 | reported | 3408 | 42.7 | rs146135726 |  |  |  |  |  |  |  |  |  |  |  |  |  |  |  |
| 348.1 | 5 | igd | 0.0016 | A | \|\| id:ebi-a-GCST90001698 | ebi-a-GCST90001698 | TRUE | G | 10850589 | 2.50E-14 | reported | 3408 | 45.47 | rs75760272 |  |  |  |  |  |  |  |  |  |  |  |  |  |  |  |
| 263 | 6 | igd | 0.0016 | A | \|\| id:ebi-a-GCST90001698 | ebi-a-GCST90001698 | TRUE | G | 118769058 | 1.99E-09 | reported | 3408 | 43.72 | rs62424171 |  |  |  |  |  |  |  |  |  |  |  |  |  |  |  |
| 335.1 | 7 | igd | 0.0016 | C | \|\| id:ebi-a-GCST90001698 | ebi-a-GCST90001698 | TRUE | G | 84326785 | 1.09E-14 | reported | 3408 | 43.17 | rs73175497 |  |  |  |  |  |  |  |  |  |  |  |  |  |  |  |
| 322 | 7 | igd | 0.0016 | C | \|\| id:ebi-a-GCST90001698 | ebi-a-GCST90001698 | TRUE | T | 20974789 | 3.89E-12 | reported | 3408 | 46.22 | rs145689986 |  |  |  |  |  |  |  |  |  |  |  |  |  |  |  |
| 249.9 | 9 | igd | 0.0016 | A | \|\| id:ebi-a-GCST90001698 | ebi-a-GCST90001698 | TRUE | G | 93695603 | 3.72E-09 | reported | 3408 | 42.27 | rs111710268 |  |  |  |  |  |  |  |  |  |  |  |  |  |  |  |
| 289 | 10 | igd | 0.0016 | A | \|\| id:ebi-a-GCST90001698 | ebi-a-GCST90001698 | TRUE | G | 6003716 | 2.56E-11 | reported | 3408 | 43.18 | rs8177783 |  |  |  |  |  |  |  |  |  |  |  |  |  |  |  |
| 335 | 11 | igd | 0.0016 | T | \|\| id:ebi-a-GCST90001698 | ebi-a-GCST90001698 | TRUE | C | 10620991 | 4.92E-14 | reported | 3408 | 44.28 | rs76385522 |  |  |  |  |  |  |  |  |  |  |  |  |  |  |  |
| 297 | 11 | igd | 0.0016 | C | \|\| id:ebi-a-GCST90001698 | ebi-a-GCST90001698 | TRUE | A | 127881478 | 1.07E-11 | reported | 3408 | 43.55 | rs78881177 |  |  |  |  |  |  |  |  |  |  |  |  |  |  |  |
| 322 | 12 | igd | 0.0016 | T | \|\| id:ebi-a-GCST90001698 | ebi-a-GCST90001698 | TRUE | G | 116077199 | 1.29E-14 | reported | 3408 | 41.59 | rs77577050 |  |  |  |  |  |  |  |  |  |  |  |  |  |  |  |
| 361 | 12 | igd | 0.0016 | C | \|\| id:ebi-a-GCST90001698 | ebi-a-GCST90001698 | TRUE | T | 131702931 | 8.54E-15 | reported | 3408 | 46.32 | rs145923523 |  |  |  |  |  |  |  |  |  |  |  |  |  |  |  |
| 303.6 | 12 | igd | 0.0016 | C | \|\| id:ebi-a-GCST90001698 | ebi-a-GCST90001698 | TRUE | T | 71181930 | 3.79E-11 | reported | 3408 | 45.76 | rs78773852 |  |  |  |  |  |  |  |  |  |  |  |  |  |  |  |
| 321.7 | 14 | igd | 0.0016 | G | \|\| id:ebi-a-GCST90001698 | ebi-a-GCST90001698 | TRUE | A | 35132040 | 4.80E-13 | reported | 3408 | 44.31 | rs12050105 |  |  |  |  |  |  |  |  |  |  |  |  |  |  |  |
| 263.7 | 14 | igd | 0.0016 | A | \|\| id:ebi-a-GCST90001698 | ebi-a-GCST90001698 | TRUE | C | 44921417 | 9.34E-09 | reported | 3408 | 45.81 | rs75767240 |  |  |  |  |  |  |  |  |  |  |  |  |  |  |  |
| 416.3 | 15 | igd | 0.0016 | T | \|\| id:ebi-a-GCST90001698 | ebi-a-GCST90001698 | TRUE | C | 54344919 | 1.31E-19 | reported | 3408 | 45.67 | rs149908314 |  |  |  |  |  |  |  |  |  |  |  |  |  |  |  |
| 268.4 | 17 | igd | 0.0016 | T | \|\| id:ebi-a-GCST90001698 | ebi-a-GCST90001698 | TRUE | C | 55639212 | 1.35E-10 | reported | 3408 | 41.66 | rs77725246 |  |  |  |  |  |  |  |  |  |  |  |  |  |  |  |
| 250.2 | 18 | igd | 0.0016 | T | \|\| id:ebi-a-GCST90001698 | ebi-a-GCST90001698 | TRUE | C | 63349806 | 5.79E-09 | reported | 3408 | 42.87 | rs57834818 |  |  |  |  |  |  |  |  |  |  |  |  |  |  |  |
| 319.6 | 19 | igd | 0.0016 | A | \|\| id:ebi-a-GCST90001698 | ebi-a-GCST90001698 | TRUE | G | 46387941 | 7.07E-13 | reported | 3408 | 44.35 | rs200198801 |  |  |  |  |  |  |  |  |  |  |  |  |  |  |  |
| 314.7 | 19 | igd | 0.0016 | T | \|\| id:ebi-a-GCST90001698 | ebi-a-GCST90001698 | TRUE | C | 29131218 | 2.43E-13 | reported | 3408 | 42.8 | rs78775565 |  |  |  |  |  |  |  |  |  |  |  |  |  |  |  |
| 250 | 19 | igd | 0.0016 | T | \|\| id:ebi-a-GCST90001698 | ebi-a-GCST90001698 | TRUE | C | 44937435 | 1.20E-08 | reported | 3408 | 43.75 | rs73034498 |  |  |  |  |  |  |  |  |  |  |  |  |  |  |  |
| 262.1 | 20 | igd | 0.0016 | A | \|\| id:ebi-a-GCST90001698 | ebi-a-GCST90001698 | TRUE | G | 54228961 | 2.38E-09 | reported | 3408 | 43.79 | rs138246394 |  |  |  |  |  |  |  |  |  |  |  |  |  |  |  |
| 339.5 | 20 | igd | 0.0016 | G | \|\| id:ebi-a-GCST90001698 | ebi-a-GCST90001698 | TRUE | C | 6679181 | 5.36E-13 | reported | 3408 | 46.87 | rs7274360 |  |  |  |  |  |  |  |  |  |  |  |  |  |  |  |
| 311.1 | 20 | igd | 0.0016 | T | \|\| id:ebi-a-GCST90001698 | ebi-a-GCST90001698 | TRUE | C | 36955628 | 3.00E-13 | reported | 3408 | 42.48 | rs141407114 |  |  |  |  |  |  |  |  |  |  |  |  |  |  |  |
| 310.8 | 21 | igd | 0.0016 | T | \|\| id:ebi-a-GCST90001698 | ebi-a-GCST90001698 | TRUE | C | 41237243 | 1.61E-12 | reported | 3408 | 43.83 | rs144680887 |  |  |  |  |  |  |  |  |  |  |  |  |  |  |  |
| 10.65 | 18 | igd | 0.0016 | T | \|\| id:ebi-a-GCST90001700 | ebi-a-GCST90001700 | TRUE | C | 62442252 | 2.50E-09 | reported | 3425 | 1.782 | rs116915023 |  |  |  |  |  |  |  |  |  |  |  |  |  |  |  |
| 213 | 2 | igd | 0.0015 | A | \|\| id:ebi-a-GCST90001695 | ebi-a-GCST90001695 | TRUE | G | 174896997 | 7.23E-09 | reported | 3408 | 36.73 | rs115954619 |  |  |  |  |  |  |  |  |  |  |  |  |  |  |  |
| 189.7 | 2 | igd | 0.0015 | A | \|\| id:ebi-a-GCST90001695 | ebi-a-GCST90001695 | TRUE | G | 176542288 | 4.09E-08 | reported | 3408 | 34.49 | rs12464465 |  |  |  |  |  |  |  |  |  |  |  |  |  |  |  |
| 195.7 | 16 | igd | 0.0015 | A | \|\| id:ebi-a-GCST90001695 | ebi-a-GCST90001695 | TRUE | T | 17388418 | 3.69E-08 | reported | 3408 | 35.46 | rs62033204 |  |  |  |  |  |  |  |  |  |  |  |  |  |  |  |
| 186.1 | 16 | igd | 0.0015 | C | \|\| id:ebi-a-GCST90001695 | ebi-a-GCST90001695 | TRUE | T | 73783555 | 2.88E-09 | reported | 3408 | 31.25 | rs72803141 |  |  |  |  |  |  |  |  |  |  |  |  |  |  |  |
| 9.757 | 2 | igd | 0.0015 | A | \|\| id:ebi-a-GCST90001697 | ebi-a-GCST90001697 | TRUE | G | 174896997 | 2.15E-08 | reported | 3425 | 1.738 | rs115954619 |  |  |  |  |  |  |  |  |  |  |  |  |  |  |  |
| 9.254 | 8 | igd | 0.0015 | T | \|\| id:ebi-a-GCST90001697 | ebi-a-GCST90001697 | TRUE | C | 88503714 | 1.08E-08 | reported | 3425 | 1.615 | rs2977034 |  |  |  |  |  |  |  |  |  |  |  |  |  |  |  |
| 360.9 | 1 | igd | 0.0015 | C | \|\| id:ebi-a-GCST90001698 | ebi-a-GCST90001698 | TRUE | T | 46796909 | 1.62E-15 | reported | 3408 | 45.09 | rs61784602 |  |  |  |  |  |  |  |  |  |  |  |  |  |  |  |
| 279.5 | 1 | igd | 0.0015 | C | \|\| id:ebi-a-GCST90001698 | ebi-a-GCST90001698 | TRUE | T | 88737271 | 1.08E-10 | reported | 3408 | 43.15 | rs148704742 |  |  |  |  |  |  |  |  |  |  |  |  |  |  |  |
| 263.8 | 1 | igd | 0.0015 | G | \|\| id:ebi-a-GCST90001698 | ebi-a-GCST90001698 | TRUE | T | 228993248 | 3.24E-08 | reported | 3408 | 47.61 | rs11590127 |  |  |  |  |  |  |  |  |  |  |  |  |  |  |  |
| 271.6 | 2 | igd | 0.0015 | G | \|\| id:ebi-a-GCST90001698 | ebi-a-GCST90001698 | TRUE | C | 172953167 | 2.11E-09 | reported | 3408 | 45.23 | rs3821186 |  |  |  |  |  |  |  |  |  |  |  |  |  |  |  |
| 347.6 | 4 | igd | 0.0015 | G | \|\| id:ebi-a-GCST90001698 | ebi-a-GCST90001698 | TRUE | C | 97639809 | 4.77E-14 | reported | 3408 | 45.92 | rs114454771 |  |  |  |  |  |  |  |  |  |  |  |  |  |  |  |
| 361.5 | 4 | igd | 0.0015 | A | \|\| id:ebi-a-GCST90001698 | ebi-a-GCST90001698 | TRUE | T | 186181360 | 3.71E-14 | reported | 3408 | 47.54 | rs13102570 |  |  |  |  |  |  |  |  |  |  |  |  |  |  |  |
| 257.5 | 4 | igd | 0.0015 | A | \|\| id:ebi-a-GCST90001698 | ebi-a-GCST90001698 | TRUE | G | 114293931 | 9.76E-09 | reported | 3408 | 44.78 | rs138943622 |  |  |  |  |  |  |  |  |  |  |  |  |  |  |  |
| 472.4 | 4 | igd | 0.0015 | A | \|\| id:ebi-a-GCST90001698 | ebi-a-GCST90001698 | TRUE | G | 97075402 | 4.36E-24 | reported | 3408 | 46.31 | rs189025847 |  |  |  |  |  |  |  |  |  |  |  |  |  |  |  |
| 268.3 | 4 | igd | 0.0015 | A | \|\| id:ebi-a-GCST90001698 | ebi-a-GCST90001698 | TRUE | G | 183314518 | 1.40E-09 | reported | 3408 | 44.19 | rs116498781 |  |  |  |  |  |  |  |  |  |  |  |  |  |  |  |
| 262.7 | 4 | igd | 0.0015 | A | \|\| id:ebi-a-GCST90001698 | ebi-a-GCST90001698 | TRUE | G | 187560601 | 4.22E-09 | reported | 3408 | 44.6 | rs148590919 |  |  |  |  |  |  |  |  |  |  |  |  |  |  |  |
| 268.3 | 5 | igd | 0.0015 | C | \|\| id:ebi-a-GCST90001698 | ebi-a-GCST90001698 | TRUE | G | 150408639 | 1.34E-09 | reported | 3408 | 44.14 | rs8177454 |  |  |  |  |  |  |  |  |  |  |  |  |  |  |  |
| 301.9 | 6 | igd | 0.0015 | C | \|\| id:ebi-a-GCST90001698 | ebi-a-GCST90001698 | TRUE | A | 20965785 | 2.12E-11 | reported | 3408 | 44.92 | rs180745907 |  |  |  |  |  |  |  |  |  |  |  |  |  |  |  |
| 312.7 | 6 | igd | 0.0015 | A | \|\| id:ebi-a-GCST90001698 | ebi-a-GCST90001698 | TRUE | G | 130091902 | 1.30E-11 | reported | 3408 | 46.04 | rs112880431 |  |  |  |  |  |  |  |  |  |  |  |  |  |  |  |
| 413.6 | 7 | igd | 0.0015 | G | \|\| id:ebi-a-GCST90001698 | ebi-a-GCST90001698 | TRUE | A | 47504039 | 1.66E-19 | reported | 3408 | 45.5 | rs77465871 |  |  |  |  |  |  |  |  |  |  |  |  |  |  |  |
| 412.5 | 7 | igd | 0.0015 | T | \|\| id:ebi-a-GCST90001698 | ebi-a-GCST90001698 | TRUE | C | 84877396 | 1.91E-17 | reported | 3408 | 48.28 | rs147036741 |  |  |  |  |  |  |  |  |  |  |  |  |  |  |  |
| 303 | 7 | igd | 0.0015 | T | \|\| id:ebi-a-GCST90001698 | ebi-a-GCST90001698 | TRUE | G | 146550168 | 3.75E-12 | reported | 3408 | 43.46 | rs111399817 |  |  |  |  |  |  |  |  |  |  |  |  |  |  |  |
| 334.2 | 8 | igd | 0.0015 | A | \|\| id:ebi-a-GCST90001698 | ebi-a-GCST90001698 | TRUE | G | 143666124 | 1.15E-13 | reported | 3408 | 44.83 | rs111519300 |  |  |  |  |  |  |  |  |  |  |  |  |  |  |  |
| 269.8 | 8 | igd | 0.0015 | A | \|\| id:ebi-a-GCST90001698 | ebi-a-GCST90001698 | TRUE | G | 9202609 | 1.39E-09 | reported | 3408 | 44.42 | rs75361870 |  |  |  |  |  |  |  |  |  |  |  |  |  |  |  |
| 277.5 | 9 | igd | 0.0015 | A | \|\| id:ebi-a-GCST90001698 | ebi-a-GCST90001698 | TRUE | G | 138254577 | 1.10E-10 | reported | 3408 | 42.87 | rs75789063 |  |  |  |  |  |  |  |  |  |  |  |  |  |  |  |
| 336.9 | 10 | igd | 0.0015 | A | \|\| id:ebi-a-GCST90001698 | ebi-a-GCST90001698 | TRUE | C | 4688192 | 2.36E-13 | reported | 3408 | 45.79 | rs77390603 |  |  |  |  |  |  |  |  |  |  |  |  |  |  |  |
| 297.7 | 10 | igd | 0.0015 | T | \|\| id:ebi-a-GCST90001698 | ebi-a-GCST90001698 | TRUE | A | 3005035 | 6.07E-11 | reported | 3408 | 45.36 | rs113966442 |  |  |  |  |  |  |  |  |  |  |  |  |  |  |  |
| 321.7 | 11 | igd | 0.0015 | G | \|\| id:ebi-a-GCST90001698 | ebi-a-GCST90001698 | TRUE | A | 127735836 | 1.16E-12 | reported | 3408 | 45.08 | rs77806002 |  |  |  |  |  |  |  |  |  |  |  |  |  |  |  |
| 342.1 | 11 | igd | 0.0015 | A | \|\| id:ebi-a-GCST90001698 | ebi-a-GCST90001698 | TRUE | G | 59336329 | 2.29E-13 | reported | 3408 | 46.48 | rs7130130 |  |  |  |  |  |  |  |  |  |  |  |  |  |  |  |
| 265.5 | 11 | igd | 0.0015 | G | \|\| id:ebi-a-GCST90001698 | ebi-a-GCST90001698 | TRUE | A | 5252284 | 8.02E-09 | reported | 3408 | 45.92 | rs112102097 |  |  |  |  |  |  |  |  |  |  |  |  |  |  |  |
| 287.4 | 11 | igd | 0.0015 | A | \|\| id:ebi-a-GCST90001698 | ebi-a-GCST90001698 | TRUE | C | 129187484 | 6.96E-11 | reported | 3408 | 43.92 | rs77485746 |  |  |  |  |  |  |  |  |  |  |  |  |  |  |  |
| 345.5 | 12 | igd | 0.0015 | T | \|\| id:ebi-a-GCST90001698 | ebi-a-GCST90001698 | TRUE | C | 129340399 | 8.74E-16 | reported | 3408 | 42.75 | rs112529428 |  |  |  |  |  |  |  |  |  |  |  |  |  |  |  |
| 273.3 | 12 | igd | 0.0015 | T | \|\| id:ebi-a-GCST90001698 | ebi-a-GCST90001698 | TRUE | C | 95005964 | 4.26E-10 | reported | 3408 | 43.65 | rs138404875 |  |  |  |  |  |  |  |  |  |  |  |  |  |  |  |
| 397 | 12 | igd | 0.0015 | T | \|\| id:ebi-a-GCST90001698 | ebi-a-GCST90001698 | TRUE | C | 82065434 | 2.74E-16 | reported | 3408 | 48.27 | rs71466029 |  |  |  |  |  |  |  |  |  |  |  |  |  |  |  |
| 250.3 | 12 | igd | 0.0015 | A | \|\| id:ebi-a-GCST90001698 | ebi-a-GCST90001698 | TRUE | G | 93540934 | 3.01E-08 | reported | 3408 | 45.06 | rs117613430 |  |  |  |  |  |  |  |  |  |  |  |  |  |  |  |
| 391.9 | 13 | igd | 0.0015 | C | \|\| id:ebi-a-GCST90001698 | ebi-a-GCST90001698 | TRUE | G | 113264513 | 6.53E-17 | reported | 3408 | 46.66 | rs80133207 |  |  |  |  |  |  |  |  |  |  |  |  |  |  |  |
| 308.6 | 13 | igd | 0.0015 | A | \|\| id:ebi-a-GCST90001698 | ebi-a-GCST90001698 | TRUE | G | 28485345 | 1.04E-12 | reported | 3408 | 43.15 | rs111648746 |  |  |  |  |  |  |  |  |  |  |  |  |  |  |  |
| 297.2 | 13 | igd | 0.0015 | T | \|\| id:ebi-a-GCST90001698 | ebi-a-GCST90001698 | TRUE | C | 76775102 | 2.78E-11 | reported | 3408 | 44.49 | rs80128954 |  |  |  |  |  |  |  |  |  |  |  |  |  |  |  |
| 337.1 | 14 | igd | 0.0015 | A | \|\| id:ebi-a-GCST90001698 | ebi-a-GCST90001698 | TRUE | G | 58571589 | 9.91E-15 | reported | 3408 | 43.36 | rs113699161 |  |  |  |  |  |  |  |  |  |  |  |  |  |  |  |
| 311.5 | 14 | igd | 0.0015 | T | \|\| id:ebi-a-GCST90001698 | ebi-a-GCST90001698 | TRUE | C | 33828647 | 6.69E-12 | reported | 3408 | 45.22 | rs79624763 |  |  |  |  |  |  |  |  |  |  |  |  |  |  |  |
| 331.7 | 16 | igd | 0.0015 | A | \|\| id:ebi-a-GCST90001698 | ebi-a-GCST90001698 | TRUE | G | 23373955 | 1.46E-13 | reported | 3408 | 44.7 | rs151246819 |  |  |  |  |  |  |  |  |  |  |  |  |  |  |  |
| 352 | 17 | igd | 0.0015 | A | \|\| id:ebi-a-GCST90001698 | ebi-a-GCST90001698 | TRUE | G | 8298873 | 6.36E-15 | reported | 3408 | 44.95 | rs78100215 |  |  |  |  |  |  |  |  |  |  |  |  |  |  |  |
| 311 | 18 | igd | 0.0015 | A | \|\| id:ebi-a-GCST90001698 | ebi-a-GCST90001698 | TRUE | G | 70586990 | 7.72E-11 | reported | 3408 | 47.64 | rs150905742 |  |  |  |  |  |  |  |  |  |  |  |  |  |  |  |
| 11.19 | 4 | igd | 0.0015 | A | \|\| id:ebi-a-GCST90001700 | ebi-a-GCST90001700 | TRUE | G | 97075402 | 1.56E-08 | reported | 3425 | 1.975 | rs189025847 |  |  |  |  |  |  |  |  |  |  |  |  |  |  |  |
| 13.03 | 7 | igd | 0.0015 | G | \|\| id:ebi-a-GCST90001700 | ebi-a-GCST90001700 | TRUE | A | 47504039 | 2.13E-11 | reported | 3425 | 1.94 | rs77465871 |  |  |  |  |  |  |  |  |  |  |  |  |  |  |  |
| 263.3 | 1 | igd | 0.0013 | A | \|\| id:ebi-a-GCST90001695 | ebi-a-GCST90001695 | TRUE | G | 166905650 | 1.89E-12 | reported | 3408 | 37.24 | rs78656354 |  |  |  |  |  |  |  |  |  |  |  |  |  |  |  |
| 221.3 | 1 | igd | 0.0013 | A | \|\| id:ebi-a-GCST90001695 | ebi-a-GCST90001695 | TRUE | G | 221221416 | 2.42E-08 | reported | 3408 | 39.57 | rs140732999 |  |  |  |  |  |  |  |  |  |  |  |  |  |  |  |
| 196.3 | 11 | igd | 0.0013 | T | \|\| id:ebi-a-GCST90001695 | ebi-a-GCST90001695 | TRUE | C | 65647885 | 1.56E-08 | reported | 3408 | 34.64 | rs145650095 |  |  |  |  |  |  |  |  |  |  |  |  |  |  |  |
| 216.7 | 11 | igd | 0.0013 | C | \|\| id:ebi-a-GCST90001695 | ebi-a-GCST90001695 | TRUE | G | 65259911 | 2.33E-09 | reported | 3408 | 36.18 | rs17676161 |  |  |  |  |  |  |  |  |  |  |  |  |  |  |  |
| 234.7 | 16 | igd | 0.0013 | T | \|\| id:ebi-a-GCST90001695 | ebi-a-GCST90001695 | TRUE | G | 74078663 | 1.23E-11 | reported | 3408 | 34.51 | rs72789106 |  |  |  |  |  |  |  |  |  |  |  |  |  |  |  |
| 270.5 | 16 | igd | 0.0013 | T | \|\| id:ebi-a-GCST90001695 | ebi-a-GCST90001695 | TRUE | C | 5991815 | 1.55E-11 | reported | 3408 | 39.98 | rs117262195 |  |  |  |  |  |  |  |  |  |  |  |  |  |  |  |
| 648.7 | 1 | igd | 0.0013 | C | \|\| id:ebi-a-GCST90001698 | ebi-a-GCST90001698 | TRUE | G | 160679804 | 6.82E-26 | reported | 3408 | 61.14 | rs149050426 |  |  |  |  |  |  |  |  |  |  |  |  |  |  |  |
| 317.1 | 1 | igd | 0.0013 | A | \|\| id:ebi-a-GCST90001698 | ebi-a-GCST90001698 | TRUE | G | 186023973 | 1.19E-10 | reported | 3408 | 49.08 | rs78085370 |  |  |  |  |  |  |  |  |  |  |  |  |  |  |  |
| 287 | 1 | igd | 0.0013 | A | \|\| id:ebi-a-GCST90001698 | ebi-a-GCST90001698 | TRUE | G | 166905650 | 1.60E-08 | reported | 3408 | 50.66 | rs78656354 |  |  |  |  |  |  |  |  |  |  |  |  |  |  |  |
| 371 | 1 | igd | 0.0013 | C | \|\| id:ebi-a-GCST90001698 | ebi-a-GCST90001698 | TRUE | G | 226235714 | 1.12E-14 | reported | 3408 | 47.82 | rs72757046 |  |  |  |  |  |  |  |  |  |  |  |  |  |  |  |
| 266.3 | 2 | igd | 0.0013 | C | \|\| id:ebi-a-GCST90001698 | ebi-a-GCST90001698 | TRUE | G | 240726495 | 2.41E-08 | reported | 3408 | 47.62 | rs184230902 |  |  |  |  |  |  |  |  |  |  |  |  |  |  |  |
| 419.8 | 2 | igd | 0.0013 | G | \|\| id:ebi-a-GCST90001698 | ebi-a-GCST90001698 | TRUE | T | 97762985 | 1.09E-14 | reported | 3408 | 54.08 | rs377446032 |  |  |  |  |  |  |  |  |  |  |  |  |  |  |  |
| 353 | 2 | igd | 0.0013 | T | \|\| id:ebi-a-GCST90001698 | ebi-a-GCST90001698 | TRUE | C | 199542954 | 5.07E-14 | reported | 3408 | 46.68 | rs75561723 |  |  |  |  |  |  |  |  |  |  |  |  |  |  |  |
| 449.3 | 2 | igd | 0.0013 | C | \|\| id:ebi-a-GCST90001698 | ebi-a-GCST90001698 | TRUE | T | 203830976 | 3.39E-17 | reported | 3408 | 53 | rs143679542 |  |  |  |  |  |  |  |  |  |  |  |  |  |  |  |
| 354.3 | 3 | igd | 0.0013 | A | \|\| id:ebi-a-GCST90001698 | ebi-a-GCST90001698 | TRUE | G | 126619549 | 7.47E-13 | reported | 3408 | 49.22 | rs572820113 |  |  |  |  |  |  |  |  |  |  |  |  |  |  |  |
| 281.3 | 3 | igd | 0.0013 | C | \|\| id:ebi-a-GCST90001698 | ebi-a-GCST90001698 | TRUE | T | 141999791 | 4.83E-08 | reported | 3408 | 51.43 | rs35268412 |  |  |  |  |  |  |  |  |  |  |  |  |  |  |  |
| 281.5 | 3 | igd | 0.0013 | T | \|\| id:ebi-a-GCST90001698 | ebi-a-GCST90001698 | TRUE | A | 190504703 | 2.21E-08 | reported | 3408 | 50.2 | rs114057146 |  |  |  |  |  |  |  |  |  |  |  |  |  |  |  |
| 525.8 | 4 | igd | 0.0013 | A | \|\| id:ebi-a-GCST90001698 | ebi-a-GCST90001698 | TRUE | G | 180665663 | 7.08E-36 | reported | 3408 | 41.57 | rs78999408 |  |  |  |  |  |  |  |  |  |  |  |  |  |  |  |
| 620.5 | 4 | igd | 0.0013 | A | \|\| id:ebi-a-GCST90001698 | ebi-a-GCST90001698 | TRUE | G | 180565400 | 1.11E-45 | reported | 3408 | 43.09 | rs12501785 |  |  |  |  |  |  |  |  |  |  |  |  |  |  |  |
| 262.2 | 5 | igd | 0.0013 | G | \|\| id:ebi-a-GCST90001698 | ebi-a-GCST90001698 | TRUE | A | 12372451 | 1.94E-08 | reported | 3408 | 46.56 | rs112290739 |  |  |  |  |  |  |  |  |  |  |  |  |  |  |  |
| 358.2 | 5 | igd | 0.0013 | T | \|\| id:ebi-a-GCST90001698 | ebi-a-GCST90001698 | TRUE | C | 142967016 | 1.67E-14 | reported | 3408 | 46.47 | rs189369117 |  |  |  |  |  |  |  |  |  |  |  |  |  |  |  |
| 266.7 | 5 | igd | 0.0013 | G | \|\| id:ebi-a-GCST90001698 | ebi-a-GCST90001698 | TRUE | A | 12480500 | 1.34E-08 | reported | 3408 | 46.83 | rs148939501 |  |  |  |  |  |  |  |  |  |  |  |  |  |  |  |
| 258.3 | 5 | igd | 0.0013 | C | \|\| id:ebi-a-GCST90001698 | ebi-a-GCST90001698 | TRUE | T | 12269594 | 2.62E-08 | reported | 3408 | 46.3 | rs113776377 |  |  |  |  |  |  |  |  |  |  |  |  |  |  |  |
| 308.2 | 6 | igd | 0.0013 | A | \|\| id:ebi-a-GCST90001698 | ebi-a-GCST90001698 | TRUE | C | 149283507 | 3.31E-09 | reported | 3408 | 51.96 | rs139289963 |  |  |  |  |  |  |  |  |  |  |  |  |  |  |  |
| 378.1 | 6 | igd | 0.0013 | T | \|\| id:ebi-a-GCST90001698 | ebi-a-GCST90001698 | TRUE | C | 119632972 | 4.68E-11 | reported | 3408 | 57.27 | rs117248086 |  |  |  |  |  |  |  |  |  |  |  |  |  |  |  |
| 278.1 | 6 | igd | 0.0013 | A | \|\| id:ebi-a-GCST90001698 | ebi-a-GCST90001698 | TRUE | G | 48192452 | 1.29E-08 | reported | 3408 | 48.77 | rs76025137 |  |  |  |  |  |  |  |  |  |  |  |  |  |  |  |
| 281.7 | 6 | igd | 0.0013 | A | \|\| id:ebi-a-GCST90001698 | ebi-a-GCST90001698 | TRUE | G | 130199006 | 3.59E-08 | reported | 3408 | 51.01 | rs111883537 |  |  |  |  |  |  |  |  |  |  |  |  |  |  |  |
| 325.6 | 7 | igd | 0.0013 | G | \|\| id:ebi-a-GCST90001698 | ebi-a-GCST90001698 | TRUE | T | 105961923 | 2.24E-11 | reported | 3408 | 48.51 | rs149882069 |  |  |  |  |  |  |  |  |  |  |  |  |  |  |  |
| 361.9 | 9 | igd | 0.0013 | G | \|\| id:ebi-a-GCST90001698 | ebi-a-GCST90001698 | TRUE | T | 7228994 | 3.71E-15 | reported | 3408 | 45.81 | rs76533941 |  |  |  |  |  |  |  |  |  |  |  |  |  |  |  |
| 424.9 | 9 | igd | 0.0013 | G | \|\| id:ebi-a-GCST90001698 | ebi-a-GCST90001698 | TRUE | A | 10774031 | 7.01E-18 | reported | 3408 | 49.05 | rs72704856 |  |  |  |  |  |  |  |  |  |  |  |  |  |  |  |
| 374.9 | 10 | igd | 0.0013 | T | \|\| id:ebi-a-GCST90001698 | ebi-a-GCST90001698 | TRUE | C | 121905612 | 1.30E-15 | reported | 3408 | 46.66 | rs72828601 |  |  |  |  |  |  |  |  |  |  |  |  |  |  |  |
| 453.6 | 10 | igd | 0.0013 | T | \|\| id:ebi-a-GCST90001698 | ebi-a-GCST90001698 | TRUE | C | 31026572 | 3.55E-21 | reported | 3408 | 47.7 | rs149860203 |  |  |  |  |  |  |  |  |  |  |  |  |  |  |  |
| 394.5 | 11 | igd | 0.0013 | T | \|\| id:ebi-a-GCST90001698 | ebi-a-GCST90001698 | TRUE | C | 87291175 | 1.55E-14 | reported | 3408 | 51.12 | rs142869493 |  |  |  |  |  |  |  |  |  |  |  |  |  |  |  |
| 339.3 | 11 | igd | 0.0013 | C | \|\| id:ebi-a-GCST90001698 | ebi-a-GCST90001698 | TRUE | T | 86050877 | 1.08E-12 | reported | 3408 | 47.47 | rs144162903 |  |  |  |  |  |  |  |  |  |  |  |  |  |  |  |
| 350.8 | 12 | igd | 0.0013 | T | \|\| id:ebi-a-GCST90001698 | ebi-a-GCST90001698 | TRUE | C | 133004766 | 1.13E-12 | reported | 3408 | 49.12 | rs76409114 |  |  |  |  |  |  |  |  |  |  |  |  |  |  |  |
| 403.5 | 12 | igd | 0.0013 | T | \|\| id:ebi-a-GCST90001698 | ebi-a-GCST90001698 | TRUE | C | 132674299 | 4.78E-15 | reported | 3408 | 51.28 | rs192624111 |  |  |  |  |  |  |  |  |  |  |  |  |  |  |  |
| 335 | 14 | igd | 0.0013 | A | \|\| id:ebi-a-GCST90001698 | ebi-a-GCST90001698 | TRUE | T | 33869413 | 1.67E-12 | reported | 3408 | 47.28 | rs192174372 |  |  |  |  |  |  |  |  |  |  |  |  |  |  |  |
| 313.8 | 14 | igd | 0.0013 | T | \|\| id:ebi-a-GCST90001698 | ebi-a-GCST90001698 | TRUE | A | 90692356 | 3.54E-11 | reported | 3408 | 47.23 | rs142503544 |  |  |  |  |  |  |  |  |  |  |  |  |  |  |  |
| 306.1 | 14 | igd | 0.0013 | T | \|\| id:ebi-a-GCST90001698 | ebi-a-GCST90001698 | TRUE | G | 45055780 | 3.39E-10 | reported | 3408 | 48.59 | rs184184520 |  |  |  |  |  |  |  |  |  |  |  |  |  |  |  |
| 316 | 18 | igd | 0.0013 | T | \|\| id:ebi-a-GCST90001698 | ebi-a-GCST90001698 | TRUE | C | 56708792 | 1.59E-10 | reported | 3408 | 49.25 | rs150994087 |  |  |  |  |  |  |  |  |  |  |  |  |  |  |  |
| 351 | 21 | igd | 0.0013 | A | \|\| id:ebi-a-GCST90001698 | ebi-a-GCST90001698 | TRUE | G | 14784547 | 5.05E-12 | reported | 3408 | 50.66 | rs117658011 |  |  |  |  |  |  |  |  |  |  |  |  |  |  |  |
| 452.4 | 21 | igd | 0.0013 | T | \|\| id:ebi-a-GCST90001698 | ebi-a-GCST90001698 | TRUE | C | 14448330 | 1.08E-11 | reported | 3408 | 66.34 | rs201239055 |  |  |  |  |  |  |  |  |  |  |  |  |  |  |  |
| 355.7 | 22 | igd | 0.0013 | A | \|\| id:ebi-a-GCST90001698 | ebi-a-GCST90001698 | TRUE | G | 23709958 | 1.26E-11 | reported | 3408 | 52.34 | rs146461857 |  |  |  |  |  |  |  |  |  |  |  |  |  |  |  |
| 10.8 | 4 | igd | 0.0013 | A | \|\| id:ebi-a-GCST90001700 | ebi-a-GCST90001700 | TRUE | G | 180565400 | 1.00E-08 | reported | 3425 | 1.881 | rs12501785 |  |  |  |  |  |  |  |  |  |  |  |  |  |  |  |
| 220.3 | 8 | igd | 0.0012 | A | \|\| id:ebi-a-GCST90001695 | ebi-a-GCST90001695 | TRUE | G | 28700432 | 9.99E-09 | reported | 3408 | 38.35 | rs147564030 |  |  |  |  |  |  |  |  |  |  |  |  |  |  |  |
| 216.6 | 8 | igd | 0.0012 | G | \|\| id:ebi-a-GCST90001695 | ebi-a-GCST90001695 | TRUE | A | 28941831 | 1.39E-08 | reported | 3408 | 38.08 | rs150665187 |  |  |  |  |  |  |  |  |  |  |  |  |  |  |  |
| 218.9 | 8 | igd | 0.0012 | C | \|\| id:ebi-a-GCST90001695 | ebi-a-GCST90001695 | TRUE | T | 28804989 | 1.13E-08 | reported | 3408 | 38.25 | rs149334275 |  |  |  |  |  |  |  |  |  |  |  |  |  |  |  |
| 214.1 | 11 | igd | 0.0012 | T | \|\| id:ebi-a-GCST90001695 | ebi-a-GCST90001695 | TRUE | G | 64593188 | 1.15E-08 | reported | 3408 | 37.43 | rs117154185 |  |  |  |  |  |  |  |  |  |  |  |  |  |  |  |
| 278.5 | 19 | igd | 0.0012 | G | \|\| id:ebi-a-GCST90001695 | ebi-a-GCST90001695 | TRUE | T | 24522507 | 1.04E-11 | reported | 3408 | 40.8 | rs112362737 |  |  |  |  |  |  |  |  |  |  |  |  |  |  |  |
| 285.3 | 20 | igd | 0.0012 | G | \|\| id:ebi-a-GCST90001695 | ebi-a-GCST90001695 | TRUE | C | 37580670 | 7.09E-13 | reported | 3408 | 39.59 | rs41276984 |  |  |  |  |  |  |  |  |  |  |  |  |  |  |  |
| 10.56 | 2 | igd | 0.0012 | G | \|\| id:ebi-a-GCST90001697 | ebi-a-GCST90001697 | TRUE | T | 151197759 | 1.64E-08 | reported | 3425 | 1.865 | rs79190516 |  |  |  |  |  |  |  |  |  |  |  |  |  |  |  |
| 11.17 | 2 | igd | 0.0012 | A | \|\| id:ebi-a-GCST90001697 | ebi-a-GCST90001697 | TRUE | G | 151046523 | 1.91E-08 | reported | 3425 | 1.983 | rs77275661 |  |  |  |  |  |  |  |  |  |  |  |  |  |  |  |
| 370.4 | 1 | igd | 0.0012 | T | \|\| id:ebi-a-GCST90001698 | ebi-a-GCST90001698 | TRUE | A | 182075634 | 6.30E-14 | reported | 3408 | 49.17 | rs7529502 |  |  |  |  |  |  |  |  |  |  |  |  |  |  |  |
| 361.2 | 1 | igd | 0.0012 | T | \|\| id:ebi-a-GCST90001698 | ebi-a-GCST90001698 | TRUE | C | 220974171 | 9.17E-13 | reported | 3408 | 50.37 | rs72472310 |  |  |  |  |  |  |  |  |  |  |  |  |  |  |  |
| 313.5 | 1 | igd | 0.0012 | A | \|\| id:ebi-a-GCST90001698 | ebi-a-GCST90001698 | TRUE | G | 234694848 | 2.51E-08 | reported | 3408 | 56.13 | rs150862550 |  |  |  |  |  |  |  |  |  |  |  |  |  |  |  |
| 361.2 | 1 | igd | 0.0012 | G | \|\| id:ebi-a-GCST90001698 | ebi-a-GCST90001698 | TRUE | A | 221083373 | 9.32E-13 | reported | 3408 | 50.4 | rs74880146 |  |  |  |  |  |  |  |  |  |  |  |  |  |  |  |
| 330.2 | 2 | igd | 0.0012 | T | \|\| id:ebi-a-GCST90001698 | ebi-a-GCST90001698 | TRUE | C | 159969605 | 4.27E-08 | reported | 3408 | 60.13 | rs12616335 |  |  |  |  |  |  |  |  |  |  |  |  |  |  |  |
| 320.1 | 2 | igd | 0.0012 | T | \|\| id:ebi-a-GCST90001698 | ebi-a-GCST90001698 | TRUE | C | 240644273 | 2.29E-11 | reported | 3408 | 47.71 | rs151250363 |  |  |  |  |  |  |  |  |  |  |  |  |  |  |  |
| 339 | 3 | igd | 0.0012 | C | \|\| id:ebi-a-GCST90001698 | ebi-a-GCST90001698 | TRUE | T | 89721008 | 1.68E-10 | reported | 3408 | 52.89 | rs147847587 |  |  |  |  |  |  |  |  |  |  |  |  |  |  |  |
| 420 | 3 | igd | 0.0012 | T | \|\| id:ebi-a-GCST90001698 | ebi-a-GCST90001698 | TRUE | G | 130201546 | 2.10E-11 | reported | 3408 | 62.49 | rs116719160 |  |  |  |  |  |  |  |  |  |  |  |  |  |  |  |
| 358.1 | 3 | igd | 0.0012 | T | \|\| id:ebi-a-GCST90001698 | ebi-a-GCST90001698 | TRUE | C | 16578446 | 5.81E-09 | reported | 3408 | 61.35 | rs115721431 |  |  |  |  |  |  |  |  |  |  |  |  |  |  |  |
| 267.9 | 3 | igd | 0.0012 | T | \|\| id:ebi-a-GCST90001698 | ebi-a-GCST90001698 | TRUE | G | 98166935 | 2.05E-08 | reported | 3408 | 47.66 | rs116256350 |  |  |  |  |  |  |  |  |  |  |  |  |  |  |  |
| 398.9 | 4 | igd | 0.0012 | A | \|\| id:ebi-a-GCST90001698 | ebi-a-GCST90001698 | TRUE | G | 86225214 | 1.24E-16 | reported | 3408 | 47.93 | rs142133433 |  |  |  |  |  |  |  |  |  |  |  |  |  |  |  |
| 387.3 | 4 | igd | 0.0012 | A | \|\| id:ebi-a-GCST90001698 | ebi-a-GCST90001698 | TRUE | C | 31456124 | 2.22E-14 | reported | 3408 | 50.49 | rs139236907 |  |  |  |  |  |  |  |  |  |  |  |  |  |  |  |
| 547.6 | 4 | igd | 0.0012 | T | \|\| id:ebi-a-GCST90001698 | ebi-a-GCST90001698 | TRUE | A | 180881450 | 1.30E-36 | reported | 3408 | 42.82 | rs115378596 |  |  |  |  |  |  |  |  |  |  |  |  |  |  |  |
| 460.1 | 4 | igd | 0.0012 | C | \|\| id:ebi-a-GCST90001698 | ebi-a-GCST90001698 | TRUE | T | 131786765 | 1.21E-19 | reported | 3408 | 50.43 | rs140367496 |  |  |  |  |  |  |  |  |  |  |  |  |  |  |  |
| 438.9 | 4 | igd | 0.0012 | A | \|\| id:ebi-a-GCST90001698 | ebi-a-GCST90001698 | TRUE | G | 22140311 | 5.06E-17 | reported | 3408 | 52.07 | rs77434296 |  |  |  |  |  |  |  |  |  |  |  |  |  |  |  |
| 312.3 | 4 | igd | 0.0012 | A | \|\| id:ebi-a-GCST90001698 | ebi-a-GCST90001698 | TRUE | G | 166260369 | 1.03E-08 | reported | 3408 | 54.42 | rs28622225 |  |  |  |  |  |  |  |  |  |  |  |  |  |  |  |
| 381.2 | 4 | igd | 0.0012 | C | \|\| id:ebi-a-GCST90001698 | ebi-a-GCST90001698 | TRUE | T | 146773005 | 6.19E-11 | reported | 3408 | 58.11 | rs72723889 |  |  |  |  |  |  |  |  |  |  |  |  |  |  |  |
| 475.9 | 4 | igd | 0.0012 | G | \|\| id:ebi-a-GCST90001698 | ebi-a-GCST90001698 | TRUE | A | 108096534 | 6.51E-19 | reported | 3408 | 53.26 | rs77146551 |  |  |  |  |  |  |  |  |  |  |  |  |  |  |  |
| 386.6 | 5 | igd | 0.0012 | G | \|\| id:ebi-a-GCST90001698 | ebi-a-GCST90001698 | TRUE | A | 128365521 | 4.77E-14 | reported | 3408 | 51.06 | rs112973846 |  |  |  |  |  |  |  |  |  |  |  |  |  |  |  |
| 337.2 | 6 | igd | 0.0012 | T | \|\| id:ebi-a-GCST90001698 | ebi-a-GCST90001698 | TRUE | C | 82300125 | 2.61E-11 | reported | 3408 | 50.4 | rs76602542 |  |  |  |  |  |  |  |  |  |  |  |  |  |  |  |
| 276.4 | 7 | igd | 0.0012 | G | \|\| id:ebi-a-GCST90001698 | ebi-a-GCST90001698 | TRUE | A | 131030913 | 7.63E-09 | reported | 3408 | 47.73 | rs76869550 |  |  |  |  |  |  |  |  |  |  |  |  |  |  |  |
| 447.3 | 7 | igd | 0.0012 | G | \|\| id:ebi-a-GCST90001698 | ebi-a-GCST90001698 | TRUE | A | 78248378 | 2.32E-19 | reported | 3408 | 49.41 | rs73143179 |  |  |  |  |  |  |  |  |  |  |  |  |  |  |  |
| 350.7 | 8 | igd | 0.0012 | G | \|\| id:ebi-a-GCST90001698 | ebi-a-GCST90001698 | TRUE | A | 143036141 | 4.69E-13 | reported | 3408 | 48.29 | rs113544591 |  |  |  |  |  |  |  |  |  |  |  |  |  |  |  |
| 494.7 | 9 | igd | 0.0012 | T | \|\| id:ebi-a-GCST90001698 | ebi-a-GCST90001698 | TRUE | G | 77966779 | 1.04E-18 | reported | 3408 | 55.7 | rs144206851 |  |  |  |  |  |  |  |  |  |  |  |  |  |  |  |
| 359.1 | 9 | igd | 0.0012 | A | \|\| id:ebi-a-GCST90001698 | ebi-a-GCST90001698 | TRUE | G | 99511751 | 3.58E-13 | reported | 3408 | 49.2 | rs10122523 |  |  |  |  |  |  |  |  |  |  |  |  |  |  |  |
| 338.6 | 11 | igd | 0.0012 | G | \|\| id:ebi-a-GCST90001698 | ebi-a-GCST90001698 | TRUE | A | 3810665 | 7.38E-11 | reported | 3408 | 51.82 | rs78658327 |  |  |  |  |  |  |  |  |  |  |  |  |  |  |  |
| 416.6 | 12 | igd | 0.0012 | T | \|\| id:ebi-a-GCST90001698 | ebi-a-GCST90001698 | TRUE | C | 133446245 | 7.89E-16 | reported | 3408 | 51.47 | rs75391939 |  |  |  |  |  |  |  |  |  |  |  |  |  |  |  |
| 432.3 | 13 | igd | 0.0012 | A | \|\| id:ebi-a-GCST90001698 | ebi-a-GCST90001698 | TRUE | G | 113289664 | 1.92E-16 | reported | 3408 | 52.29 | rs150476409 |  |  |  |  |  |  |  |  |  |  |  |  |  |  |  |
| 391.1 | 14 | igd | 0.0012 | G | \|\| id:ebi-a-GCST90001698 | ebi-a-GCST90001698 | TRUE | A | 33998519 | 6.43E-15 | reported | 3408 | 49.95 | rs80334718 |  |  |  |  |  |  |  |  |  |  |  |  |  |  |  |
| 402.6 | 14 | igd | 0.0012 | T | \|\| id:ebi-a-GCST90001698 | ebi-a-GCST90001698 | TRUE | C | 34140441 | 8.49E-15 | reported | 3408 | 51.64 | rs112223740 |  |  |  |  |  |  |  |  |  |  |  |  |  |  |  |
| 311.3 | 14 | igd | 0.0012 | G | \|\| id:ebi-a-GCST90001698 | ebi-a-GCST90001698 | TRUE | A | 79235275 | 2.99E-09 | reported | 3408 | 52.33 | rs72685437 |  |  |  |  |  |  |  |  |  |  |  |  |  |  |  |
| 396.5 | 14 | igd | 0.0012 | C | \|\| id:ebi-a-GCST90001698 | ebi-a-GCST90001698 | TRUE | T | 33709145 | 1.28E-14 | reported | 3408 | 51.22 | rs138955903 |  |  |  |  |  |  |  |  |  |  |  |  |  |  |  |
| 335.4 | 14 | igd | 0.0012 | C | \|\| id:ebi-a-GCST90001698 | ebi-a-GCST90001698 | TRUE | T | 35486479 | 8.81E-10 | reported | 3408 | 54.56 | rs138230916 |  |  |  |  |  |  |  |  |  |  |  |  |  |  |  |
| 366.1 | 16 | igd | 0.0012 | G | \|\| id:ebi-a-GCST90001698 | ebi-a-GCST90001698 | TRUE | C | 31402773 | 6.56E-14 | reported | 3408 | 48.63 | rs572954905 |  |  |  |  |  |  |  |  |  |  |  |  |  |  |  |
| 397 | 17 | igd | 0.0012 | G | \|\| id:ebi-a-GCST90001698 | ebi-a-GCST90001698 | TRUE | A | 29891369 | 1.22E-15 | reported | 3408 | 49.38 | rs80108006 |  |  |  |  |  |  |  |  |  |  |  |  |  |  |  |
| 359.2 | 17 | igd | 0.0012 | G | \|\| id:ebi-a-GCST90001698 | ebi-a-GCST90001698 | TRUE | A | 67774399 | 9.71E-14 | reported | 3408 | 48.05 | rs143391757 |  |  |  |  |  |  |  |  |  |  |  |  |  |  |  |
| 285.1 | 18 | igd | 0.0012 | A | \|\| id:ebi-a-GCST90001698 | ebi-a-GCST90001698 | TRUE | T | 1699838 | 3.80E-09 | reported | 3408 | 48.25 | rs184040186 |  |  |  |  |  |  |  |  |  |  |  |  |  |  |  |
| 367.4 | 19 | igd | 0.0012 | A | \|\| id:ebi-a-GCST90001698 | ebi-a-GCST90001698 | TRUE | G | 43789908 | 5.92E-12 | reported | 3408 | 53.2 | rs58572634 |  |  |  |  |  |  |  |  |  |  |  |  |  |  |  |
| 609.3 | 20 | igd | 0.0012 | G | \|\| id:ebi-a-GCST90001698 | ebi-a-GCST90001698 | TRUE | C | 37580670 | 6.17E-30 | reported | 3408 | 53.09 | rs41276984 |  |  |  |  |  |  |  |  |  |  |  |  |  |  |  |
| 378.7 | 20 | igd | 0.0012 | C | \|\| id:ebi-a-GCST90001698 | ebi-a-GCST90001698 | TRUE | G | 15245578 | 1.18E-14 | reported | 3408 | 48.86 | rs145250558 |  |  |  |  |  |  |  |  |  |  |  |  |  |  |  |
| 322.5 | 20 | igd | 0.0012 | C | \|\| id:ebi-a-GCST90001698 | ebi-a-GCST90001698 | TRUE | T | 20630777 | 3.29E-10 | reported | 3408 | 51.17 | rs77785558 |  |  |  |  |  |  |  |  |  |  |  |  |  |  |  |
| 417.3 | 22 | igd | 0.0012 | T | \|\| id:ebi-a-GCST90001698 | ebi-a-GCST90001698 | TRUE | C | 36610967 | 6.59E-16 | reported | 3408 | 51.4 | rs132751 |  |  |  |  |  |  |  |  |  |  |  |  |  |  |  |
| 15.14 | 20 | igd | 0.0012 | G | \|\| id:ebi-a-GCST90001700 | ebi-a-GCST90001700 | TRUE | C | 37580670 | 2.20E-11 | reported | 3425 | 2.254 | rs41276984 |  |  |  |  |  |  |  |  |  |  |  |  |  |  |  |
| 239.7 | 3 | igd | 0.001 | A | \|\| id:ebi-a-GCST90001695 | ebi-a-GCST90001695 | TRUE | G | 39505173 | 1.26E-08 | reported | 3408 | 42.01 | rs79489889 |  |  |  |  |  |  |  |  |  |  |  |  |  |  |  |
| 251.6 | 5 | igd | 0.001 | A | \|\| id:ebi-a-GCST90001695 | ebi-a-GCST90001695 | TRUE | G | 97959184 | 2.78E-09 | reported | 3408 | 42.22 | rs62369101 |  |  |  |  |  |  |  |  |  |  |  |  |  |  |  |
| 251.2 | 8 | igd | 0.001 | T | \|\| id:ebi-a-GCST90001695 | ebi-a-GCST90001695 | TRUE | C | 28561116 | 1.15E-09 | reported | 3408 | 41.16 | rs143825022 |  |  |  |  |  |  |  |  |  |  |  |  |  |  |  |
| 232.5 | 12 | igd | 0.001 | T | \|\| id:ebi-a-GCST90001695 | ebi-a-GCST90001695 | TRUE | G | 132283582 | 3.63E-08 | reported | 3408 | 42.11 | rs117785622 |  |  |  |  |  |  |  |  |  |  |  |  |  |  |  |
| 223.6 | 16 | igd | 0.001 | A | \|\| id:ebi-a-GCST90001695 | ebi-a-GCST90001695 | TRUE | G | 71406147 | 2.26E-08 | reported | 3408 | 39.9 | rs148260825 |  |  |  |  |  |  |  |  |  |  |  |  |  |  |  |
| 379.6 | 16 | igd | 0.001 | C | \|\| id:ebi-a-GCST90001695 | ebi-a-GCST90001695 | TRUE | T | 78643920 | 4.53E-19 | reported | 3408 | 42.29 | rs112039735 |  |  |  |  |  |  |  |  |  |  |  |  |  |  |  |
| 379.8 | 16 | igd | 0.001 | A | \|\| id:ebi-a-GCST90001695 | ebi-a-GCST90001695 | TRUE | T | 81757975 | 6.44E-18 | reported | 3408 | 43.79 | rs143279234 |  |  |  |  |  |  |  |  |  |  |  |  |  |  |  |
| 367.9 | 16 | igd | 0.001 | A | \|\| id:ebi-a-GCST90001695 | ebi-a-GCST90001695 | TRUE | G | 81625215 | 8.71E-20 | reported | 3408 | 40.16 | rs190312656 |  |  |  |  |  |  |  |  |  |  |  |  |  |  |  |
| 25.44 | 15 | igd | 0.001 | C | \|\| id:ebi-a-GCST90001696 | ebi-a-GCST90001696 | TRUE | G | 69137961 | 4.93E-08 | reported | 3425 | 4.654 | rs62010321 |  |  |  |  |  |  |  |  |  |  |  |  |  |  |  |
| 26.32 | 16 | igd | 0.001 | A | \|\| id:ebi-a-GCST90001696 | ebi-a-GCST90001696 | TRUE | G | 81625215 | 1.42E-08 | reported | 3425 | 4.629 | rs190312656 |  |  |  |  |  |  |  |  |  |  |  |  |  |  |  |
| 11.36 | 1 | igd | 0.001 | T | \|\| id:ebi-a-GCST90001697 | ebi-a-GCST90001697 | TRUE | C | 117061778 | 1.67E-09 | reported | 3425 | 1.879 | rs55971473 |  |  |  |  |  |  |  |  |  |  |  |  |  |  |  |
| 10.42 | 16 | igd | 0.001 | A | \|\| id:ebi-a-GCST90001697 | ebi-a-GCST90001697 | TRUE | G | 81625215 | 3.91E-08 | reported | 3425 | 1.892 | rs190312656 |  |  |  |  |  |  |  |  |  |  |  |  |  |  |  |
| 11.54 | 16 | igd | 0.001 | A | \|\| id:ebi-a-GCST90001697 | ebi-a-GCST90001697 | TRUE | T | 81757975 | 2.33E-08 | reported | 3425 | 2.061 | rs143279234 |  |  |  |  |  |  |  |  |  |  |  |  |  |  |  |
| 465.8 | 1 | igd | 0.001 | C | \|\| id:ebi-a-GCST90001698 | ebi-a-GCST90001698 | TRUE | T | 66490103 | 1.42E-19 | reported | 3408 | 51.16 | rs145583539 |  |  |  |  |  |  |  |  |  |  |  |  |  |  |  |
| 363.6 | 2 | igd | 0.001 | T | \|\| id:ebi-a-GCST90001698 | ebi-a-GCST90001698 | TRUE | A | 36162712 | 1.28E-13 | reported | 3408 | 48.88 | rs74620549 |  |  |  |  |  |  |  |  |  |  |  |  |  |  |  |
| 332.2 | 2 | igd | 0.001 | C | \|\| id:ebi-a-GCST90001698 | ebi-a-GCST90001698 | TRUE | G | 234725994 | 6.35E-10 | reported | 3408 | 53.59 | rs149131245 |  |  |  |  |  |  |  |  |  |  |  |  |  |  |  |
| 320.5 | 2 | igd | 0.001 | C | \|\| id:ebi-a-GCST90001698 | ebi-a-GCST90001698 | TRUE | G | 208755904 | 1.33E-08 | reported | 3408 | 56.26 | rs77678807 |  |  |  |  |  |  |  |  |  |  |  |  |  |  |  |
| 300.3 | 3 | igd | 0.001 | T | \|\| id:ebi-a-GCST90001698 | ebi-a-GCST90001698 | TRUE | G | 96355429 | 6.67E-09 | reported | 3408 | 51.66 | rs78014003 |  |  |  |  |  |  |  |  |  |  |  |  |  |  |  |
| 581.1 | 4 | igd | 0.001 | C | \|\| id:ebi-a-GCST90001698 | ebi-a-GCST90001698 | TRUE | T | 120352238 | 6.72E-19 | reported | 3408 | 65.06 | rs199797711 |  |  |  |  |  |  |  |  |  |  |  |  |  |  |  |
| 466.1 | 6 | igd | 0.001 | T | \|\| id:ebi-a-GCST90001698 | ebi-a-GCST90001698 | TRUE | C | 5734886 | 2.51E-18 | reported | 3408 | 53.08 | rs6916896 |  |  |  |  |  |  |  |  |  |  |  |  |  |  |  |
| 475 | 7 | igd | 0.001 | T | \|\| id:ebi-a-GCST90001698 | ebi-a-GCST90001698 | TRUE | C | 109248384 | 1.21E-17 | reported | 3408 | 55.24 | rs35730839 |  |  |  |  |  |  |  |  |  |  |  |  |  |  |  |
| 403.5 | 8 | igd | 0.001 | G | \|\| id:ebi-a-GCST90001698 | ebi-a-GCST90001698 | TRUE | C | 142876352 | 3.95E-15 | reported | 3408 | 51.12 | rs113122796 |  |  |  |  |  |  |  |  |  |  |  |  |  |  |  |
| 311 | 8 | igd | 0.001 | A | \|\| id:ebi-a-GCST90001698 | ebi-a-GCST90001698 | TRUE | G | 98360805 | 2.47E-08 | reported | 3408 | 55.65 | rs74397834 |  |  |  |  |  |  |  |  |  |  |  |  |  |  |  |
| 318.9 | 8 | igd | 0.001 | A | \|\| id:ebi-a-GCST90001698 | ebi-a-GCST90001698 | TRUE | C | 2821818 | 1.66E-09 | reported | 3408 | 52.77 | rs188963780 |  |  |  |  |  |  |  |  |  |  |  |  |  |  |  |
| 328.1 | 10 | igd | 0.001 | T | \|\| id:ebi-a-GCST90001698 | ebi-a-GCST90001698 | TRUE | C | 101494118 | 4.02E-10 | reported | 3408 | 52.32 | rs143772909 |  |  |  |  |  |  |  |  |  |  |  |  |  |  |  |
| 387.4 | 10 | igd | 0.001 | T | \|\| id:ebi-a-GCST90001698 | ebi-a-GCST90001698 | TRUE | G | 89500755 | 4.58E-09 | reported | 3408 | 65.92 | rs11202534 |  |  |  |  |  |  |  |  |  |  |  |  |  |  |  |
| 454.9 | 10 | igd | 0.001 | T | \|\| id:ebi-a-GCST90001698 | ebi-a-GCST90001698 | TRUE | C | 121973769 | 5.58E-17 | reported | 3408 | 54.04 | rs74157741 |  |  |  |  |  |  |  |  |  |  |  |  |  |  |  |
| 346.3 | 11 | igd | 0.001 | T | \|\| id:ebi-a-GCST90001698 | ebi-a-GCST90001698 | TRUE | G | 83227574 | 6.77E-12 | reported | 3408 | 50.29 | rs192165769 |  |  |  |  |  |  |  |  |  |  |  |  |  |  |  |
| 344.4 | 11 | igd | 0.001 | A | \|\| id:ebi-a-GCST90001698 | ebi-a-GCST90001698 | TRUE | T | 31631366 | 3.96E-10 | reported | 3408 | 54.89 | rs117420444 |  |  |  |  |  |  |  |  |  |  |  |  |  |  |  |
| 573 | 12 | igd | 0.001 | T | \|\| id:ebi-a-GCST90001698 | ebi-a-GCST90001698 | TRUE | G | 132283582 | 7.11E-23 | reported | 3408 | 57.77 | rs117785622 |  |  |  |  |  |  |  |  |  |  |  |  |  |  |  |
| 516.8 | 12 | igd | 0.001 | G | \|\| id:ebi-a-GCST90001698 | ebi-a-GCST90001698 | TRUE | A | 102082782 | 7.67E-19 | reported | 3408 | 57.96 | rs139075636 |  |  |  |  |  |  |  |  |  |  |  |  |  |  |  |
| 521.7 | 12 | igd | 0.001 | A | \|\| id:ebi-a-GCST90001698 | ebi-a-GCST90001698 | TRUE | G | 131571288 | 6.26E-20 | reported | 3408 | 56.73 | rs74653052 |  |  |  |  |  |  |  |  |  |  |  |  |  |  |  |
| 542.5 | 12 | igd | 0.001 | G | \|\| id:ebi-a-GCST90001698 | ebi-a-GCST90001698 | TRUE | A | 88561479 | 9.78E-23 | reported | 3408 | 54.89 | rs35275693 |  |  |  |  |  |  |  |  |  |  |  |  |  |  |  |
| 419.2 | 13 | igd | 0.001 | T | \|\| id:ebi-a-GCST90001698 | ebi-a-GCST90001698 | TRUE | C | 103352454 | 1.50E-11 | reported | 3408 | 61.92 | rs113394325 |  |  |  |  |  |  |  |  |  |  |  |  |  |  |  |
| 386.5 | 13 | igd | 0.001 | A | \|\| id:ebi-a-GCST90001698 | ebi-a-GCST90001698 | TRUE | T | 31805158 | 1.91E-13 | reported | 3408 | 52.34 | rs186400376 |  |  |  |  |  |  |  |  |  |  |  |  |  |  |  |
| 442.9 | 14 | igd | 0.001 | T | \|\| id:ebi-a-GCST90001698 | ebi-a-GCST90001698 | TRUE | G | 89207147 | 8.69E-16 | reported | 3408 | 54.79 | rs74565504 |  |  |  |  |  |  |  |  |  |  |  |  |  |  |  |
| 308.3 | 14 | igd | 0.001 | G | \|\| id:ebi-a-GCST90001698 | ebi-a-GCST90001698 | TRUE | C | 76826605 | 1.88E-08 | reported | 3408 | 54.7 | rs192467351 |  |  |  |  |  |  |  |  |  |  |  |  |  |  |  |
| 447.6 | 15 | igd | 0.001 | A | \|\| id:ebi-a-GCST90001698 | ebi-a-GCST90001698 | TRUE | G | 51649599 | 1.71E-16 | reported | 3408 | 54.04 | rs117211386 |  |  |  |  |  |  |  |  |  |  |  |  |  |  |  |
| 414.6 | 16 | igd | 0.001 | C | \|\| id:ebi-a-GCST90001698 | ebi-a-GCST90001698 | TRUE | A | 13128176 | 1.41E-14 | reported | 3408 | 53.64 | rs117555426 |  |  |  |  |  |  |  |  |  |  |  |  |  |  |  |
| 322.2 | 17 | igd | 0.001 | C | \|\| id:ebi-a-GCST90001698 | ebi-a-GCST90001698 | TRUE | G | 79246592 | 6.08E-10 | reported | 3408 | 51.91 | rs72850608 |  |  |  |  |  |  |  |  |  |  |  |  |  |  |  |
| 443.1 | 17 | igd | 0.001 | T | \|\| id:ebi-a-GCST90001698 | ebi-a-GCST90001698 | TRUE | C | 67625638 | 2.25E-17 | reported | 3408 | 51.97 | rs139736375 |  |  |  |  |  |  |  |  |  |  |  |  |  |  |  |
| 447.6 | 17 | igd | 0.001 | A | \|\| id:ebi-a-GCST90001698 | ebi-a-GCST90001698 | TRUE | G | 80344923 | 2.09E-17 | reported | 3408 | 52.44 | rs568349042 |  |  |  |  |  |  |  |  |  |  |  |  |  |  |  |
| 364.4 | 18 | igd | 0.001 | C | \|\| id:ebi-a-GCST90001698 | ebi-a-GCST90001698 | TRUE | A | 22735939 | 2.74E-11 | reported | 3408 | 54.53 | rs76808079 |  |  |  |  |  |  |  |  |  |  |  |  |  |  |  |
| 469.1 | 18 | igd | 0.001 | T | \|\| id:ebi-a-GCST90001698 | ebi-a-GCST90001698 | TRUE | C | 38279589 | 1.14E-18 | reported | 3408 | 52.88 | rs116940971 |  |  |  |  |  |  |  |  |  |  |  |  |  |  |  |
| 481.7 | 19 | igd | 0.001 | A | \|\| id:ebi-a-GCST90001698 | ebi-a-GCST90001698 | TRUE | G | 45431674 | 5.42E-18 | reported | 3408 | 55.41 | rs118060185 |  |  |  |  |  |  |  |  |  |  |  |  |  |  |  |
| 300.7 | 19 | igd | 0.001 | A | \|\| id:ebi-a-GCST90001698 | ebi-a-GCST90001698 | TRUE | G | 8487210 | 4.56E-08 | reported | 3408 | 54.87 | rs138771381 |  |  |  |  |  |  |  |  |  |  |  |  |  |  |  |
| 489.9 | 20 | igd | 0.001 | A | \|\| id:ebi-a-GCST90001698 | ebi-a-GCST90001698 | TRUE | G | 24457030 | 8.85E-22 | reported | 3408 | 50.73 | rs180972485 |  |  |  |  |  |  |  |  |  |  |  |  |  |  |  |
| 456.5 | 20 | igd | 0.001 | G | \|\| id:ebi-a-GCST90001698 | ebi-a-GCST90001698 | TRUE | C | 41552583 | 2.75E-17 | reported | 3408 | 53.7 | rs142564480 |  |  |  |  |  |  |  |  |  |  |  |  |  |  |  |
| 534.9 | 20 | igd | 0.001 | C | \|\| id:ebi-a-GCST90001698 | ebi-a-GCST90001698 | TRUE | T | 37027035 | 6.75E-22 | reported | 3408 | 55.24 | rs118180479 |  |  |  |  |  |  |  |  |  |  |  |  |  |  |  |
| 37.99 | 4 | igd | 0.001 | C | \|\| id:ebi-a-GCST90001699 | ebi-a-GCST90001699 | TRUE | T | 120352238 | 5.20E-09 | reported | 3425 | 6.487 | rs199797711 |  |  |  |  |  |  |  |  |  |  |  |  |  |  |  |
| 20.29 | 4 | igd | 0.001 | C | \|\| id:ebi-a-GCST90001700 | ebi-a-GCST90001700 | TRUE | T | 120352238 | 3.16E-13 | reported | 3425 | 2.774 | rs199797711 |  |  |  |  |  |  |  |  |  |  |  |  |  |  |  |
| 12.83 | 12 | igd | 0.001 | G | \|\| id:ebi-a-GCST90001700 | ebi-a-GCST90001700 | TRUE | A | 88561479 | 4.51E-08 | reported | 3425 | 2.34 | rs35275693 |  |  |  |  |  |  |  |  |  |  |  |  |  |  |  |
| 12.36 | 20 | igd | 0.001 | A | \|\| id:ebi-a-GCST90001700 | ebi-a-GCST90001700 | TRUE | G | 24457030 | 2.22E-08 | reported | 3425 | 2.205 | rs180972485 |  |  |  |  |  |  |  |  |  |  |  |  |  |  |  |
| 232.3 | 1 | igd | 9.00E-04 | A | \|\| id:ebi-a-GCST90001695 | ebi-a-GCST90001695 | TRUE | G | 177793844 | 4.06E-08 | reported | 3408 | 42.22 | rs116218713 |  |  |  |  |  |  |  |  |  |  |  |  |  |  |  |
| 281.9 | 3 | igd | 9.00E-04 | G | \|\| id:ebi-a-GCST90001695 | ebi-a-GCST90001695 | TRUE | A | 194347376 | 5.10E-10 | reported | 3408 | 45.23 | rs115879183 |  |  |  |  |  |  |  |  |  |  |  |  |  |  |  |
| 281.9 | 6 | igd | 9.00E-04 | A | \|\| id:ebi-a-GCST90001695 | ebi-a-GCST90001695 | TRUE | G | 70855865 | 2.80E-09 | reported | 3408 | 47.31 | rs145613913 |  |  |  |  |  |  |  |  |  |  |  |  |  |  |  |
| 286.9 | 6 | igd | 9.00E-04 | T | \|\| id:ebi-a-GCST90001695 | ebi-a-GCST90001695 | TRUE | C | 148462118 | 2.84E-10 | reported | 3408 | 45.35 | rs112857714 |  |  |  |  |  |  |  |  |  |  |  |  |  |  |  |
| 256.7 | 7 | igd | 9.00E-04 | C | \|\| id:ebi-a-GCST90001695 | ebi-a-GCST90001695 | TRUE | T | 57279055 | 1.91E-08 | reported | 3408 | 45.57 | rs34592474 |  |  |  |  |  |  |  |  |  |  |  |  |  |  |  |
| 237.4 | 8 | igd | 9.00E-04 | T | \|\| id:ebi-a-GCST90001695 | ebi-a-GCST90001695 | TRUE | C | 52839926 | 3.80E-08 | reported | 3408 | 43.07 | rs140086187 |  |  |  |  |  |  |  |  |  |  |  |  |  |  |  |
| 259.6 | 13 | igd | 9.00E-04 | T | \|\| id:ebi-a-GCST90001695 | ebi-a-GCST90001695 | TRUE | C | 73498369 | 2.11E-09 | reported | 3408 | 43.23 | rs142470414 |  |  |  |  |  |  |  |  |  |  |  |  |  |  |  |
| 257.2 | 13 | igd | 9.00E-04 | T | \|\| id:ebi-a-GCST90001695 | ebi-a-GCST90001695 | TRUE | A | 73364029 | 2.76E-09 | reported | 3408 | 43.15 | rs149774755 |  |  |  |  |  |  |  |  |  |  |  |  |  |  |  |
| 233.8 | 15 | igd | 9.00E-04 | A | \|\| id:ebi-a-GCST90001695 | ebi-a-GCST90001695 | TRUE | G | 94910184 | 4.31E-08 | reported | 3408 | 42.59 | rs139217681 |  |  |  |  |  |  |  |  |  |  |  |  |  |  |  |
| 416.3 | 16 | igd | 9.00E-04 | C | \|\| id:ebi-a-GCST90001695 | ebi-a-GCST90001695 | TRUE | A | 78214566 | 2.82E-21 | reported | 3408 | 43.68 | rs151237482 |  |  |  |  |  |  |  |  |  |  |  |  |  |  |  |
| 267.8 | 16 | igd | 9.00E-04 | T | \|\| id:ebi-a-GCST90001695 | ebi-a-GCST90001695 | TRUE | C | 32379367 | 2.79E-08 | reported | 3408 | 48.1 | rs541390197 |  |  |  |  |  |  |  |  |  |  |  |  |  |  |  |
| 317.4 | 16 | igd | 9.00E-04 | T | \|\| id:ebi-a-GCST90001695 | ebi-a-GCST90001695 | TRUE | C | 85685140 | 2.34E-13 | reported | 3408 | 43.13 | rs192302673 |  |  |  |  |  |  |  |  |  |  |  |  |  |  |  |
| 262.3 | 17 | igd | 9.00E-04 | G | \|\| id:ebi-a-GCST90001695 | ebi-a-GCST90001695 | TRUE | A | 76005958 | 5.71E-09 | reported | 3408 | 44.92 | rs146164275 |  |  |  |  |  |  |  |  |  |  |  |  |  |  |  |
| 289.9 | 20 | igd | 9.00E-04 | T | \|\| id:ebi-a-GCST90001695 | ebi-a-GCST90001695 | TRUE | G | 38313269 | 2.59E-11 | reported | 3408 | 43.33 | rs118010773 |  |  |  |  |  |  |  |  |  |  |  |  |  |  |  |
| 28.75 | 15 | igd | 9.00E-04 | G | \|\| id:ebi-a-GCST90001696 | ebi-a-GCST90001696 | TRUE | A | 69903187 | 3.59E-08 | reported | 3425 | 5.206 | rs140385926 |  |  |  |  |  |  |  |  |  |  |  |  |  |  |  |
| 11.85 | 16 | igd | 9.00E-04 | C | \|\| id:ebi-a-GCST90001697 | ebi-a-GCST90001697 | TRUE | A | 78214566 | 9.68E-09 | reported | 3425 | 2.061 | rs151237482 |  |  |  |  |  |  |  |  |  |  |  |  |  |  |  |
| 12.63 | 16 | igd | 9.00E-04 | T | \|\| id:ebi-a-GCST90001697 | ebi-a-GCST90001697 | TRUE | C | 85685140 | 4.39E-10 | reported | 3425 | 2.018 | rs192302673 |  |  |  |  |  |  |  |  |  |  |  |  |  |  |  |
| 326.1 | 1 | igd | 9.00E-04 | T | \|\| id:ebi-a-GCST90001698 | ebi-a-GCST90001698 | TRUE | C | 197334740 | 1.19E-08 | reported | 3408 | 57.06 | rs571348134 |  |  |  |  |  |  |  |  |  |  |  |  |  |  |  |
| 495.5 | 1 | igd | 9.00E-04 | G | \|\| id:ebi-a-GCST90001698 | ebi-a-GCST90001698 | TRUE | T | 185411696 | 3.66E-18 | reported | 3408 | 56.71 | rs75390358 |  |  |  |  |  |  |  |  |  |  |  |  |  |  |  |
| 381.7 | 1 | igd | 9.00E-04 | A | \|\| id:ebi-a-GCST90001698 | ebi-a-GCST90001698 | TRUE | G | 236238830 | 8.73E-13 | reported | 3408 | 53.18 | rs139423647 |  |  |  |  |  |  |  |  |  |  |  |  |  |  |  |
| 636.7 | 1 | igd | 9.00E-04 | A | \|\| id:ebi-a-GCST90001698 | ebi-a-GCST90001698 | TRUE | G | 220781210 | 5.51E-22 | reported | 3408 | 65.61 | rs141644068 |  |  |  |  |  |  |  |  |  |  |  |  |  |  |  |
| 505.9 | 2 | igd | 9.00E-04 | G | \|\| id:ebi-a-GCST90001698 | ebi-a-GCST90001698 | TRUE | C | 129405810 | 4.85E-17 | reported | 3408 | 59.97 | rs192583129 |  |  |  |  |  |  |  |  |  |  |  |  |  |  |  |
| 501.2 | 2 | igd | 9.00E-04 | C | \|\| id:ebi-a-GCST90001698 | ebi-a-GCST90001698 | TRUE | A | 198985983 | 3.57E-19 | reported | 3408 | 55.67 | rs142711096 |  |  |  |  |  |  |  |  |  |  |  |  |  |  |  |
| 426.4 | 2 | igd | 9.00E-04 | A | \|\| id:ebi-a-GCST90001698 | ebi-a-GCST90001698 | TRUE | G | 24501710 | 4.89E-13 | reported | 3408 | 58.76 | rs187500876 |  |  |  |  |  |  |  |  |  |  |  |  |  |  |  |
| 381.8 | 2 | igd | 9.00E-04 | G | \|\| id:ebi-a-GCST90001698 | ebi-a-GCST90001698 | TRUE | A | 24625376 | 6.77E-12 | reported | 3408 | 55.45 | rs146911193 |  |  |  |  |  |  |  |  |  |  |  |  |  |  |  |
| 378.4 | 3 | igd | 9.00E-04 | G | \|\| id:ebi-a-GCST90001698 | ebi-a-GCST90001698 | TRUE | C | 128385376 | 8.71E-11 | reported | 3408 | 58.14 | rs150305408 |  |  |  |  |  |  |  |  |  |  |  |  |  |  |  |
| 337.3 | 3 | igd | 9.00E-04 | G | \|\| id:ebi-a-GCST90001698 | ebi-a-GCST90001698 | TRUE | A | 96914205 | 1.13E-09 | reported | 3408 | 55.22 | rs145714288 |  |  |  |  |  |  |  |  |  |  |  |  |  |  |  |
| 324.3 | 3 | igd | 9.00E-04 | C | \|\| id:ebi-a-GCST90001698 | ebi-a-GCST90001698 | TRUE | T | 62448768 | 1.11E-08 | reported | 3408 | 56.62 | rs139414070 |  |  |  |  |  |  |  |  |  |  |  |  |  |  |  |
| 358.8 | 3 | igd | 9.00E-04 | G | \|\| id:ebi-a-GCST90001698 | ebi-a-GCST90001698 | TRUE | A | 168644376 | 6.39E-11 | reported | 3408 | 54.73 | rs75551427 |  |  |  |  |  |  |  |  |  |  |  |  |  |  |  |
| 380.8 | 3 | igd | 9.00E-04 | T | \|\| id:ebi-a-GCST90001698 | ebi-a-GCST90001698 | TRUE | C | 129334353 | 3.27E-11 | reported | 3408 | 57.21 | rs193051234 |  |  |  |  |  |  |  |  |  |  |  |  |  |  |  |
| 571.9 | 3 | igd | 9.00E-04 | G | \|\| id:ebi-a-GCST90001698 | ebi-a-GCST90001698 | TRUE | A | 194347376 | 1.66E-20 | reported | 3408 | 61.21 | rs115879183 |  |  |  |  |  |  |  |  |  |  |  |  |  |  |  |
| 428.3 | 3 | igd | 9.00E-04 | G | \|\| id:ebi-a-GCST90001698 | ebi-a-GCST90001698 | TRUE | A | 144835290 | 9.36E-13 | reported | 3408 | 59.76 | rs187990734 |  |  |  |  |  |  |  |  |  |  |  |  |  |  |  |
| 538.4 | 3 | igd | 9.00E-04 | C | \|\| id:ebi-a-GCST90001698 | ebi-a-GCST90001698 | TRUE | T | 112721963 | 4.34E-20 | reported | 3408 | 58.29 | rs150062416 |  |  |  |  |  |  |  |  |  |  |  |  |  |  |  |
| 433.1 | 6 | igd | 9.00E-04 | G | \|\| id:ebi-a-GCST90001698 | ebi-a-GCST90001698 | TRUE | C | 82142072 | 9.88E-13 | reported | 3408 | 60.5 | rs143911258 |  |  |  |  |  |  |  |  |  |  |  |  |  |  |  |
| 415.4 | 6 | igd | 9.00E-04 | T | \|\| id:ebi-a-GCST90001698 | ebi-a-GCST90001698 | TRUE | C | 82633290 | 1.29E-12 | reported | 3408 | 58.33 | rs77778233 |  |  |  |  |  |  |  |  |  |  |  |  |  |  |  |
| 436.5 | 6 | igd | 9.00E-04 | T | \|\| id:ebi-a-GCST90001698 | ebi-a-GCST90001698 | TRUE | C | 81984275 | 8.35E-13 | reported | 3408 | 60.77 | rs192397067 |  |  |  |  |  |  |  |  |  |  |  |  |  |  |  |
| 336.7 | 7 | igd | 9.00E-04 | T | \|\| id:ebi-a-GCST90001698 | ebi-a-GCST90001698 | TRUE | C | 131191874 | 3.31E-10 | reported | 3408 | 53.43 | rs35706199 |  |  |  |  |  |  |  |  |  |  |  |  |  |  |  |
| 375.1 | 8 | igd | 9.00E-04 | C | \|\| id:ebi-a-GCST90001698 | ebi-a-GCST90001698 | TRUE | T | 98513319 | 2.43E-09 | reported | 3408 | 62.7 | rs117440863 |  |  |  |  |  |  |  |  |  |  |  |  |  |  |  |
| 410.9 | 9 | igd | 9.00E-04 | A | \|\| id:ebi-a-GCST90001698 | ebi-a-GCST90001698 | TRUE | G | 6501225 | 5.00E-13 | reported | 3408 | 56.65 | rs80096444 |  |  |  |  |  |  |  |  |  |  |  |  |  |  |  |
| 412 | 9 | igd | 9.00E-04 | A | \|\| id:ebi-a-GCST90001698 | ebi-a-GCST90001698 | TRUE | C | 6391190 | 4.77E-13 | reported | 3408 | 56.75 | rs147999173 |  |  |  |  |  |  |  |  |  |  |  |  |  |  |  |
| 526.2 | 10 | igd | 9.00E-04 | G | \|\| id:ebi-a-GCST90001698 | ebi-a-GCST90001698 | TRUE | C | 1734511 | 6.51E-22 | reported | 3408 | 54.32 | rs11596188 |  |  |  |  |  |  |  |  |  |  |  |  |  |  |  |
| 549.6 | 10 | igd | 9.00E-04 | T | \|\| id:ebi-a-GCST90001698 | ebi-a-GCST90001698 | TRUE | C | 5377215 | 1.16E-21 | reported | 3408 | 57.08 | rs147563624 |  |  |  |  |  |  |  |  |  |  |  |  |  |  |  |
| 440.3 | 11 | igd | 9.00E-04 | T | \|\| id:ebi-a-GCST90001698 | ebi-a-GCST90001698 | TRUE | G | 1468243 | 8.25E-15 | reported | 3408 | 56.46 | rs147394922 |  |  |  |  |  |  |  |  |  |  |  |  |  |  |  |
| 429.8 | 11 | igd | 9.00E-04 | T | \|\| id:ebi-a-GCST90001698 | ebi-a-GCST90001698 | TRUE | C | 82932495 | 3.93E-15 | reported | 3408 | 54.45 | rs116952577 |  |  |  |  |  |  |  |  |  |  |  |  |  |  |  |
| 422.8 | 11 | igd | 9.00E-04 | C | \|\| id:ebi-a-GCST90001698 | ebi-a-GCST90001698 | TRUE | T | 1576057 | 2.10E-14 | reported | 3408 | 55.07 | rs148749783 |  |  |  |  |  |  |  |  |  |  |  |  |  |  |  |
| 642.9 | 12 | igd | 9.00E-04 | T | \|\| id:ebi-a-GCST90001698 | ebi-a-GCST90001698 | TRUE | A | 130060949 | 1.66E-31 | reported | 3408 | 54.5 | rs146822590 |  |  |  |  |  |  |  |  |  |  |  |  |  |  |  |
| 515.7 | 12 | igd | 9.00E-04 | A | \|\| id:ebi-a-GCST90001698 | ebi-a-GCST90001698 | TRUE | C | 10963475 | 5.40E-20 | reported | 3408 | 55.97 | rs137943198 |  |  |  |  |  |  |  |  |  |  |  |  |  |  |  |
| 546.1 | 12 | igd | 9.00E-04 | G | \|\| id:ebi-a-GCST90001698 | ebi-a-GCST90001698 | TRUE | C | 129086998 | 9.57E-20 | reported | 3408 | 59.68 | rs192175945 |  |  |  |  |  |  |  |  |  |  |  |  |  |  |  |
| 496.1 | 17 | igd | 9.00E-04 | T | \|\| id:ebi-a-GCST90001698 | ebi-a-GCST90001698 | TRUE | C | 80299376 | 5.81E-19 | reported | 3408 | 55.44 | rs150053624 |  |  |  |  |  |  |  |  |  |  |  |  |  |  |  |
| 302.6 | 17 | igd | 9.00E-04 | T | \|\| id:ebi-a-GCST90001698 | ebi-a-GCST90001698 | TRUE | C | 743825 | 2.79E-08 | reported | 3408 | 54.36 | rs142234058 |  |  |  |  |  |  |  |  |  |  |  |  |  |  |  |
| 345.9 | 19 | igd | 9.00E-04 | G | \|\| id:ebi-a-GCST90001698 | ebi-a-GCST90001698 | TRUE | C | 50566868 | 1.01E-08 | reported | 3408 | 60.24 | rs413628 |  |  |  |  |  |  |  |  |  |  |  |  |  |  |  |
| 598.5 | 20 | igd | 9.00E-04 | T | \|\| id:ebi-a-GCST90001698 | ebi-a-GCST90001698 | TRUE | G | 38313269 | 4.40E-25 | reported | 3408 | 57.4 | rs118010773 |  |  |  |  |  |  |  |  |  |  |  |  |  |  |  |
| 559.6 | 20 | igd | 9.00E-04 | G | \|\| id:ebi-a-GCST90001698 | ebi-a-GCST90001698 | TRUE | T | 40197928 | 1.61E-22 | reported | 3408 | 56.91 | rs75600477 |  |  |  |  |  |  |  |  |  |  |  |  |  |  |  |
| 493.5 | 21 | igd | 9.00E-04 | A | \|\| id:ebi-a-GCST90001698 | ebi-a-GCST90001698 | TRUE | C | 24279231 | 5.35E-18 | reported | 3408 | 56.77 | rs79998237 |  |  |  |  |  |  |  |  |  |  |  |  |  |  |  |
| 524 | 21 | igd | 9.00E-04 | C | \|\| id:ebi-a-GCST90001698 | ebi-a-GCST90001698 | TRUE | A | 24382637 | 8.10E-19 | reported | 3408 | 58.81 | rs117577961 |  |  |  |  |  |  |  |  |  |  |  |  |  |  |  |
| 14.19 | 12 | igd | 9.00E-04 | T | \|\| id:ebi-a-GCST90001700 | ebi-a-GCST90001700 | TRUE | A | 130060949 | 2.68E-09 | reported | 3425 | 2.379 | rs146822590 |  |  |  |  |  |  |  |  |  |  |  |  |  |  |  |
| 259.1 | 2 | igd | 7.00E-04 | C | \|\| id:ebi-a-GCST90001695 | ebi-a-GCST90001695 | TRUE | T | 146534594 | 9.70E-09 | reported | 3408 | 45.06 | rs138962348 |  |  |  |  |  |  |  |  |  |  |  |  |  |  |  |
| 283 | 2 | igd | 7.00E-04 | A | \|\| id:ebi-a-GCST90001695 | ebi-a-GCST90001695 | TRUE | G | 144233823 | 1.55E-09 | reported | 3408 | 46.74 | rs75828981 |  |  |  |  |  |  |  |  |  |  |  |  |  |  |  |
| 293.3 | 4 | igd | 7.00E-04 | T | \|\| id:ebi-a-GCST90001695 | ebi-a-GCST90001695 | TRUE | C | 114347303 | 2.16E-09 | reported | 3408 | 48.87 | rs192553272 |  |  |  |  |  |  |  |  |  |  |  |  |  |  |  |
| 305.4 | 5 | igd | 7.00E-04 | T | \|\| id:ebi-a-GCST90001695 | ebi-a-GCST90001695 | TRUE | C | 20785820 | 2.93E-09 | reported | 3408 | 51.32 | rs141855504 |  |  |  |  |  |  |  |  |  |  |  |  |  |  |  |
| 271.2 | 7 | igd | 7.00E-04 | C | \|\| id:ebi-a-GCST90001695 | ebi-a-GCST90001695 | TRUE | G | 84434549 | 2.39E-08 | reported | 3408 | 48.48 | rs143845801 |  |  |  |  |  |  |  |  |  |  |  |  |  |  |  |
| 375.7 | 8 | igd | 7.00E-04 | C | \|\| id:ebi-a-GCST90001695 | ebi-a-GCST90001695 | TRUE | T | 127054086 | 2.64E-10 | reported | 3408 | 59.27 | rs72728362 |  |  |  |  |  |  |  |  |  |  |  |  |  |  |  |
| 352.4 | 11 | igd | 7.00E-04 | A | \|\| id:ebi-a-GCST90001695 | ebi-a-GCST90001695 | TRUE | G | 118189351 | 1.09E-12 | reported | 3408 | 49.32 | rs150427323 |  |  |  |  |  |  |  |  |  |  |  |  |  |  |  |
| 308 | 12 | igd | 7.00E-04 | A | \|\| id:ebi-a-GCST90001695 | ebi-a-GCST90001695 | TRUE | G | 734001 | 4.56E-10 | reported | 3408 | 49.26 | rs34499829 |  |  |  |  |  |  |  |  |  |  |  |  |  |  |  |
| 296.2 | 14 | igd | 7.00E-04 | C | \|\| id:ebi-a-GCST90001695 | ebi-a-GCST90001695 | TRUE | T | 86640070 | 3.93E-09 | reported | 3408 | 50.18 | rs61988500 |  |  |  |  |  |  |  |  |  |  |  |  |  |  |  |
| 285.3 | 15 | igd | 7.00E-04 | T | \|\| id:ebi-a-GCST90001695 | ebi-a-GCST90001695 | TRUE | G | 94946993 | 1.66E-09 | reported | 3408 | 47.21 | rs146940094 |  |  |  |  |  |  |  |  |  |  |  |  |  |  |  |
| 337.2 | 16 | igd | 7.00E-04 | T | \|\| id:ebi-a-GCST90001695 | ebi-a-GCST90001695 | TRUE | C | 26527473 | 3.73E-10 | reported | 3408 | 53.66 | rs150856260 |  |  |  |  |  |  |  |  |  |  |  |  |  |  |  |
| 450.3 | 1 | igd | 7.00E-04 | C | \|\| id:ebi-a-GCST90001698 | ebi-a-GCST90001698 | TRUE | T | 207801545 | 4.12E-10 | reported | 3408 | 71.85 | rs11580627 |  |  |  |  |  |  |  |  |  |  |  |  |  |  |  |
| 617.1 | 1 | igd | 7.00E-04 | C | \|\| id:ebi-a-GCST90001698 | ebi-a-GCST90001698 | TRUE | G | 113653257 | 3.76E-23 | reported | 3408 | 61.82 | rs41299575 |  |  |  |  |  |  |  |  |  |  |  |  |  |  |  |
| 542.8 | 1 | igd | 7.00E-04 | T | \|\| id:ebi-a-GCST90001698 | ebi-a-GCST90001698 | TRUE | G | 188273967 | 1.60E-18 | reported | 3408 | 61.45 | rs182068311 |  |  |  |  |  |  |  |  |  |  |  |  |  |  |  |
| 585.1 | 1 | igd | 7.00E-04 | C | \|\| id:ebi-a-GCST90001698 | ebi-a-GCST90001698 | TRUE | G | 46898789 | 2.53E-22 | reported | 3408 | 59.78 | rs114021246 |  |  |  |  |  |  |  |  |  |  |  |  |  |  |  |
| 621.6 | 1 | igd | 7.00E-04 | G | \|\| id:ebi-a-GCST90001698 | ebi-a-GCST90001698 | TRUE | C | 187795006 | 1.28E-23 | reported | 3408 | 61.6 | rs116446467 |  |  |  |  |  |  |  |  |  |  |  |  |  |  |  |
| 350 | 1 | igd | 7.00E-04 | A | \|\| id:ebi-a-GCST90001698 | ebi-a-GCST90001698 | TRUE | T | 207698225 | 1.85E-08 | reported | 3408 | 62.07 | rs186998459 |  |  |  |  |  |  |  |  |  |  |  |  |  |  |  |
| 605.2 | 1 | igd | 7.00E-04 | T | \|\| id:ebi-a-GCST90001698 | ebi-a-GCST90001698 | TRUE | C | 181280885 | 1.15E-21 | reported | 3408 | 62.86 | rs75641434 |  |  |  |  |  |  |  |  |  |  |  |  |  |  |  |
| 413.3 | 2 | igd | 7.00E-04 | T | \|\| id:ebi-a-GCST90001698 | ebi-a-GCST90001698 | TRUE | C | 162960872 | 4.25E-11 | reported | 3408 | 62.46 | rs188292706 |  |  |  |  |  |  |  |  |  |  |  |  |  |  |  |
| 562.4 | 2 | igd | 7.00E-04 | C | \|\| id:ebi-a-GCST90001698 | ebi-a-GCST90001698 | TRUE | T | 24167677 | 8.73E-17 | reported | 3408 | 67.24 | rs149876155 |  |  |  |  |  |  |  |  |  |  |  |  |  |  |  |
| 406.7 | 3 | igd | 7.00E-04 | T | \|\| id:ebi-a-GCST90001698 | ebi-a-GCST90001698 | TRUE | C | 94081481 | 1.62E-11 | reported | 3408 | 60.17 | rs116226749 |  |  |  |  |  |  |  |  |  |  |  |  |  |  |  |
| 445.4 | 3 | igd | 7.00E-04 | C | \|\| id:ebi-a-GCST90001698 | ebi-a-GCST90001698 | TRUE | T | 96737521 | 9.12E-13 | reported | 3408 | 62.12 | rs190518971 |  |  |  |  |  |  |  |  |  |  |  |  |  |  |  |
| 450.9 | 3 | igd | 7.00E-04 | T | \|\| id:ebi-a-GCST90001698 | ebi-a-GCST90001698 | TRUE | A | 162562015 | 5.82E-13 | reported | 3408 | 62.34 | rs141295857 |  |  |  |  |  |  |  |  |  |  |  |  |  |  |  |
| 402.6 | 3 | igd | 7.00E-04 | A | \|\| id:ebi-a-GCST90001698 | ebi-a-GCST90001698 | TRUE | G | 89852507 | 2.35E-11 | reported | 3408 | 60.05 | rs186592830 |  |  |  |  |  |  |  |  |  |  |  |  |  |  |  |
| 424.1 | 3 | igd | 7.00E-04 | A | \|\| id:ebi-a-GCST90001698 | ebi-a-GCST90001698 | TRUE | G | 96511991 | 5.11E-12 | reported | 3408 | 61.23 | rs114861660 |  |  |  |  |  |  |  |  |  |  |  |  |  |  |  |
| 451.4 | 3 | igd | 7.00E-04 | C | \|\| id:ebi-a-GCST90001698 | ebi-a-GCST90001698 | TRUE | A | 162446104 | 5.69E-13 | reported | 3408 | 62.39 | rs147196001 |  |  |  |  |  |  |  |  |  |  |  |  |  |  |  |
| 466.9 | 3 | igd | 7.00E-04 | A | \|\| id:ebi-a-GCST90001698 | ebi-a-GCST90001698 | TRUE | G | 93585214 | 2.74E-13 | reported | 3408 | 63.65 | rs115609319 |  |  |  |  |  |  |  |  |  |  |  |  |  |  |  |
| 388.3 | 4 | igd | 7.00E-04 | G | \|\| id:ebi-a-GCST90001698 | ebi-a-GCST90001698 | TRUE | A | 93955349 | 6.10E-10 | reported | 3408 | 62.57 | rs80071510 |  |  |  |  |  |  |  |  |  |  |  |  |  |  |  |
| 653.3 | 4 | igd | 7.00E-04 | T | \|\| id:ebi-a-GCST90001698 | ebi-a-GCST90001698 | TRUE | A | 175833277 | 5.04E-23 | reported | 3408 | 65.64 | rs116116252 |  |  |  |  |  |  |  |  |  |  |  |  |  |  |  |
| 580.3 | 4 | igd | 7.00E-04 | A | \|\| id:ebi-a-GCST90001698 | ebi-a-GCST90001698 | TRUE | C | 123414413 | 2.16E-21 | reported | 3408 | 60.69 | rs191607214 |  |  |  |  |  |  |  |  |  |  |  |  |  |  |  |
| 438.9 | 5 | igd | 7.00E-04 | G | \|\| id:ebi-a-GCST90001698 | ebi-a-GCST90001698 | TRUE | A | 71107850 | 8.69E-12 | reported | 3408 | 64.07 | rs148816938 |  |  |  |  |  |  |  |  |  |  |  |  |  |  |  |
| 942.7 | 5 | igd | 7.00E-04 | T | \|\| id:ebi-a-GCST90001698 | ebi-a-GCST90001698 | TRUE | C | 94553542 | 1.49E-32 | reported | 3408 | 78.52 | rs143693103 |  |  |  |  |  |  |  |  |  |  |  |  |  |  |  |
| 487.3 | 6 | igd | 7.00E-04 | C | \|\| id:ebi-a-GCST90001698 | ebi-a-GCST90001698 | TRUE | G | 125051261 | 9.16E-19 | reported | 3408 | 54.77 | rs150287087 |  |  |  |  |  |  |  |  |  |  |  |  |  |  |  |
| 796.1 | 6 | igd | 7.00E-04 | C | \|\| id:ebi-a-GCST90001698 | ebi-a-GCST90001698 | TRUE | T | 10198189 | 2.18E-30 | reported | 3408 | 68.82 | rs77217387 |  |  |  |  |  |  |  |  |  |  |  |  |  |  |  |
| 412.6 | 6 | igd | 7.00E-04 | G | \|\| id:ebi-a-GCST90001698 | ebi-a-GCST90001698 | TRUE | A | 10321345 | 1.02E-08 | reported | 3408 | 71.86 | rs185682202 |  |  |  |  |  |  |  |  |  |  |  |  |  |  |  |
| 515.3 | 6 | igd | 7.00E-04 | C | \|\| id:ebi-a-GCST90001698 | ebi-a-GCST90001698 | TRUE | T | 2728734 | 1.90E-15 | reported | 3408 | 64.53 | rs141609647 |  |  |  |  |  |  |  |  |  |  |  |  |  |  |  |
| 487.3 | 6 | igd | 7.00E-04 | A | \|\| id:ebi-a-GCST90001698 | ebi-a-GCST90001698 | TRUE | G | 131690870 | 7.34E-14 | reported | 3408 | 64.86 | rs13196699 |  |  |  |  |  |  |  |  |  |  |  |  |  |  |  |
| 767.7 | 7 | igd | 7.00E-04 | C | \|\| id:ebi-a-GCST90001698 | ebi-a-GCST90001698 | TRUE | G | 84434549 | 2.77E-32 | reported | 3408 | 64.23 | rs143845801 |  |  |  |  |  |  |  |  |  |  |  |  |  |  |  |
| 454.3 | 7 | igd | 7.00E-04 | A | \|\| id:ebi-a-GCST90001698 | ebi-a-GCST90001698 | TRUE | T | 141411124 | 1.26E-13 | reported | 3408 | 61.06 | rs117961284 |  |  |  |  |  |  |  |  |  |  |  |  |  |  |  |
| 404.7 | 7 | igd | 7.00E-04 | G | \|\| id:ebi-a-GCST90001698 | ebi-a-GCST90001698 | TRUE | T | 34681601 | 8.89E-11 | reported | 3408 | 62.22 | rs80305889 |  |  |  |  |  |  |  |  |  |  |  |  |  |  |  |
| 750.9 | 7 | igd | 7.00E-04 | C | \|\| id:ebi-a-GCST90001698 | ebi-a-GCST90001698 | TRUE | A | 13558580 | 1.70E-27 | reported | 3408 | 68.52 | rs186251573 |  |  |  |  |  |  |  |  |  |  |  |  |  |  |  |
| 404.4 | 8 | igd | 7.00E-04 | C | \|\| id:ebi-a-GCST90001698 | ebi-a-GCST90001698 | TRUE | T | 57917248 | 7.99E-11 | reported | 3408 | 62.01 | rs77370289 |  |  |  |  |  |  |  |  |  |  |  |  |  |  |  |
| 329.5 | 8 | igd | 7.00E-04 | A | \|\| id:ebi-a-GCST90001698 | ebi-a-GCST90001698 | TRUE | G | 62733106 | 4.55E-08 | reported | 3408 | 60.12 | rs141663545 |  |  |  |  |  |  |  |  |  |  |  |  |  |  |  |
| 469.2 | 8 | igd | 7.00E-04 | A | \|\| id:ebi-a-GCST90001698 | ebi-a-GCST90001698 | TRUE | G | 144517333 | 2.55E-12 | reported | 3408 | 66.78 | rs144615355 |  |  |  |  |  |  |  |  |  |  |  |  |  |  |  |
| 828.9 | 8 | igd | 7.00E-04 | C | \|\| id:ebi-a-GCST90001698 | ebi-a-GCST90001698 | TRUE | G | 143859934 | 3.17E-31 | reported | 3408 | 70.61 | rs79218577 |  |  |  |  |  |  |  |  |  |  |  |  |  |  |  |
| 369.2 | 8 | igd | 7.00E-04 | G | \|\| id:ebi-a-GCST90001698 | ebi-a-GCST90001698 | TRUE | A | 2859400 | 2.79E-09 | reported | 3408 | 61.96 | rs144258173 |  |  |  |  |  |  |  |  |  |  |  |  |  |  |  |
| 583 | 8 | igd | 7.00E-04 | G | \|\| id:ebi-a-GCST90001698 | ebi-a-GCST90001698 | TRUE | C | 143262415 | 3.30E-22 | reported | 3408 | 59.74 | rs117887959 |  |  |  |  |  |  |  |  |  |  |  |  |  |  |  |
| 708.1 | 9 | igd | 7.00E-04 | T | \|\| id:ebi-a-GCST90001698 | ebi-a-GCST90001698 | TRUE | C | 10889621 | 3.47E-28 | reported | 3408 | 63.75 | rs149649979 |  |  |  |  |  |  |  |  |  |  |  |  |  |  |  |
| 520 | 9 | igd | 7.00E-04 | A | \|\| id:ebi-a-GCST90001698 | ebi-a-GCST90001698 | TRUE | G | 137149408 | 6.19E-18 | reported | 3408 | 59.93 | rs72768736 |  |  |  |  |  |  |  |  |  |  |  |  |  |  |  |
| 378.8 | 10 | igd | 7.00E-04 | C | \|\| id:ebi-a-GCST90001698 | ebi-a-GCST90001698 | TRUE | T | 51023864 | 2.15E-08 | reported | 3408 | 67.49 | rs117850415 |  |  |  |  |  |  |  |  |  |  |  |  |  |  |  |
| 699.1 | 11 | igd | 7.00E-04 | T | \|\| id:ebi-a-GCST90001698 | ebi-a-GCST90001698 | TRUE | C | 84269972 | 1.90E-23 | reported | 3408 | 69.55 | rs12279408 |  |  |  |  |  |  |  |  |  |  |  |  |  |  |  |
| 398.3 | 12 | igd | 7.00E-04 | G | \|\| id:ebi-a-GCST90001698 | ebi-a-GCST90001698 | TRUE | A | 46009960 | 4.25E-11 | reported | 3408 | 60.2 | rs58817739 |  |  |  |  |  |  |  |  |  |  |  |  |  |  |  |
| 440.2 | 12 | igd | 7.00E-04 | C | \|\| id:ebi-a-GCST90001698 | ebi-a-GCST90001698 | TRUE | T | 29095512 | 9.46E-12 | reported | 3408 | 64.38 | rs142686680 |  |  |  |  |  |  |  |  |  |  |  |  |  |  |  |
| 606.7 | 14 | igd | 7.00E-04 | T | \|\| id:ebi-a-GCST90001698 | ebi-a-GCST90001698 | TRUE | C | 41636680 | 2.95E-21 | reported | 3408 | 63.67 | rs183436787 |  |  |  |  |  |  |  |  |  |  |  |  |  |  |  |
| 330.6 | 17 | igd | 7.00E-04 | A | \|\| id:ebi-a-GCST90001698 | ebi-a-GCST90001698 | TRUE | G | 79094162 | 3.68E-08 | reported | 3408 | 59.91 | rs55856613 |  |  |  |  |  |  |  |  |  |  |  |  |  |  |  |
| 417.2 | 19 | igd | 7.00E-04 | A | \|\| id:ebi-a-GCST90001698 | ebi-a-GCST90001698 | TRUE | C | 6753000 | 2.64E-11 | reported | 3408 | 62.38 | rs146101423 |  |  |  |  |  |  |  |  |  |  |  |  |  |  |  |
| 826.8 | 19 | igd | 7.00E-04 | T | \|\| id:ebi-a-GCST90001698 | ebi-a-GCST90001698 | TRUE | C | 49416171 | 7.54E-32 | reported | 3408 | 69.68 | rs147572731 |  |  |  |  |  |  |  |  |  |  |  |  |  |  |  |
| 694 | 20 | igd | 7.00E-04 | A | \|\| id:ebi-a-GCST90001698 | ebi-a-GCST90001698 | TRUE | G | 39941038 | 7.18E-28 | reported | 3408 | 62.86 | rs112468167 |  |  |  |  |  |  |  |  |  |  |  |  |  |  |  |
| 16.4 | 6 | igd | 7.00E-04 | C | \|\| id:ebi-a-GCST90001700 | ebi-a-GCST90001700 | TRUE | T | 10198189 | 1.93E-08 | reported | 3425 | 2.911 | rs77217387 |  |  |  |  |  |  |  |  |  |  |  |  |  |  |  |
| 18.54 | 7 | igd | 7.00E-04 | C | \|\| id:ebi-a-GCST90001700 | ebi-a-GCST90001700 | TRUE | G | 84434549 | 1.90E-11 | reported | 3425 | 2.753 | rs143845801 |  |  |  |  |  |  |  |  |  |  |  |  |  |  |  |
| 16 | 11 | igd | 7.00E-04 | T | \|\| id:ebi-a-GCST90001700 | ebi-a-GCST90001700 | TRUE | C | 84269972 | 4.60E-08 | reported | 3425 | 2.92 | rs12279408 |  |  |  |  |  |  |  |  |  |  |  |  |  |  |  |
| 16.14 | 20 | igd | 7.00E-04 | A | \|\| id:ebi-a-GCST90001700 | ebi-a-GCST90001700 | TRUE | G | 39941038 | 2.17E-09 | reported | 3425 | 2.69 | rs112468167 |  |  |  |  |  |  |  |  |  |  |  |  |  |  |  |
| 15.67 | 22 | igd | 7.00E-04 | A | \|\| id:ebi-a-GCST90001700 | ebi-a-GCST90001700 | TRUE | T | 23072586 | 4.65E-08 | reported | 3425 | 2.862 | rs62218442 |  |  |  |  |  |  |  |  |  |  |  |  |  |  |  |
| 372.8 | 1 | igd | 6.00E-04 | T | \|\| id:ebi-a-GCST90001695 | ebi-a-GCST90001695 | TRUE | C | 166694481 | 2.26E-08 | reported | 3408 | 66.52 | rs150351182 |  |  |  |  |  |  |  |  |  |  |  |  |  |  |  |
| 328.7 | 1 | igd | 6.00E-04 | T | \|\| id:ebi-a-GCST90001695 | ebi-a-GCST90001695 | TRUE | G | 18635367 | 5.40E-09 | reported | 3408 | 56.2 | rs182784388 |  |  |  |  |  |  |  |  |  |  |  |  |  |  |  |
| 358.6 | 1 | igd | 6.00E-04 | T | \|\| id:ebi-a-GCST90001695 | ebi-a-GCST90001695 | TRUE | A | 178035728 | 4.54E-12 | reported | 3408 | 51.64 | rs148620542 |  |  |  |  |  |  |  |  |  |  |  |  |  |  |  |
| 329.9 | 1 | igd | 6.00E-04 | G | \|\| id:ebi-a-GCST90001695 | ebi-a-GCST90001695 | TRUE | T | 86142451 | 3.42E-10 | reported | 3408 | 52.39 | rs61783744 |  |  |  |  |  |  |  |  |  |  |  |  |  |  |  |
| 307.6 | 1 | igd | 6.00E-04 | C | \|\| id:ebi-a-GCST90001695 | ebi-a-GCST90001695 | TRUE | G | 213124992 | 3.35E-08 | reported | 3408 | 55.58 | rs149124296 |  |  |  |  |  |  |  |  |  |  |  |  |  |  |  |
| 353 | 2 | igd | 6.00E-04 | A | \|\| id:ebi-a-GCST90001695 | ebi-a-GCST90001695 | TRUE | G | 80539481 | 9.89E-10 | reported | 3408 | 57.6 | rs116359296 |  |  |  |  |  |  |  |  |  |  |  |  |  |  |  |
| 285.1 | 4 | igd | 6.00E-04 | G | \|\| id:ebi-a-GCST90001695 | ebi-a-GCST90001695 | TRUE | A | 93935039 | 2.11E-08 | reported | 3408 | 50.77 | rs77152285 |  |  |  |  |  |  |  |  |  |  |  |  |  |  |  |
| 343.8 | 4 | igd | 6.00E-04 | G | \|\| id:ebi-a-GCST90001695 | ebi-a-GCST90001695 | TRUE | A | 113804516 | 1.71E-09 | reported | 3408 | 56.93 | rs113720879 |  |  |  |  |  |  |  |  |  |  |  |  |  |  |  |
| 347.8 | 4 | igd | 6.00E-04 | C | \|\| id:ebi-a-GCST90001695 | ebi-a-GCST90001695 | TRUE | A | 187679105 | 6.81E-10 | reported | 3408 | 56.2 | rs59951086 |  |  |  |  |  |  |  |  |  |  |  |  |  |  |  |
| 384.3 | 5 | igd | 6.00E-04 | C | \|\| id:ebi-a-GCST90001695 | ebi-a-GCST90001695 | TRUE | T | 29413612 | 4.14E-11 | reported | 3408 | 58.05 | rs116581718 |  |  |  |  |  |  |  |  |  |  |  |  |  |  |  |
| 349.7 | 5 | igd | 6.00E-04 | C | \|\| id:ebi-a-GCST90001695 | ebi-a-GCST90001695 | TRUE | T | 28612713 | 1.39E-10 | reported | 3408 | 54.32 | rs76019287 |  |  |  |  |  |  |  |  |  |  |  |  |  |  |  |
| 389.5 | 6 | igd | 6.00E-04 | A | \|\| id:ebi-a-GCST90001695 | ebi-a-GCST90001695 | TRUE | G | 382181 | 1.08E-09 | reported | 3408 | 63.7 | rs187115825 |  |  |  |  |  |  |  |  |  |  |  |  |  |  |  |
| 326.5 | 6 | igd | 6.00E-04 | G | \|\| id:ebi-a-GCST90001695 | ebi-a-GCST90001695 | TRUE | C | 143880059 | 5.13E-09 | reported | 3408 | 55.73 | rs147385696 |  |  |  |  |  |  |  |  |  |  |  |  |  |  |  |
| 332.4 | 7 | igd | 6.00E-04 | A | \|\| id:ebi-a-GCST90001695 | ebi-a-GCST90001695 | TRUE | G | 148905542 | 3.92E-09 | reported | 3408 | 56.32 | rs185778924 |  |  |  |  |  |  |  |  |  |  |  |  |  |  |  |
| 379.6 | 7 | igd | 6.00E-04 | A | \|\| id:ebi-a-GCST90001695 | ebi-a-GCST90001695 | TRUE | G | 21673701 | 5.76E-10 | reported | 3408 | 61.08 | rs77164608 |  |  |  |  |  |  |  |  |  |  |  |  |  |  |  |
| 429.7 | 7 | igd | 6.00E-04 | A | \|\| id:ebi-a-GCST90001695 | ebi-a-GCST90001695 | TRUE | G | 10682504 | 1.57E-11 | reported | 3408 | 63.52 | rs80046553 |  |  |  |  |  |  |  |  |  |  |  |  |  |  |  |
| 284.3 | 10 | igd | 6.00E-04 | C | \|\| id:ebi-a-GCST90001695 | ebi-a-GCST90001695 | TRUE | T | 133431823 | 3.69E-08 | reported | 3408 | 51.52 | rs61864030 |  |  |  |  |  |  |  |  |  |  |  |  |  |  |  |
| 303.3 | 11 | igd | 6.00E-04 | T | \|\| id:ebi-a-GCST90001695 | ebi-a-GCST90001695 | TRUE | C | 36584481 | 1.95E-08 | reported | 3408 | 53.88 | rs111417549 |  |  |  |  |  |  |  |  |  |  |  |  |  |  |  |
| 320.2 | 12 | igd | 6.00E-04 | A | \|\| id:ebi-a-GCST90001695 | ebi-a-GCST90001695 | TRUE | G | 114081812 | 1.42E-08 | reported | 3408 | 56.32 | rs142911819 |  |  |  |  |  |  |  |  |  |  |  |  |  |  |  |
| 314.9 | 13 | igd | 6.00E-04 | C | \|\| id:ebi-a-GCST90001695 | ebi-a-GCST90001695 | TRUE | G | 93702077 | 1.39E-08 | reported | 3408 | 55.36 | rs75350737 |  |  |  |  |  |  |  |  |  |  |  |  |  |  |  |
| 319.5 | 17 | igd | 6.00E-04 | C | \|\| id:ebi-a-GCST90001695 | ebi-a-GCST90001695 | TRUE | T | 71902183 | 1.87E-09 | reported | 3408 | 53.02 | rs72847598 |  |  |  |  |  |  |  |  |  |  |  |  |  |  |  |
| 351.9 | 19 | igd | 6.00E-04 | T | \|\| id:ebi-a-GCST90001695 | ebi-a-GCST90001695 | TRUE | C | 48379346 | 1.68E-09 | reported | 3408 | 58.23 | rs62531041 |  |  |  |  |  |  |  |  |  |  |  |  |  |  |  |
| 345.8 | 21 | igd | 6.00E-04 | T | \|\| id:ebi-a-GCST90001695 | ebi-a-GCST90001695 | TRUE | C | 30128360 | 1.48E-09 | reported | 3408 | 57.03 | rs113432575 |  |  |  |  |  |  |  |  |  |  |  |  |  |  |  |
| 18.56 | 8 | igd | 6.00E-04 | C | \|\| id:ebi-a-GCST90001697 | ebi-a-GCST90001697 | TRUE | T | 130903744 | 3.31E-11 | reported | 3425 | 2.789 | rs142586905 |  |  |  |  |  |  |  |  |  |  |  |  |  |  |  |
| 15.84 | 12 | igd | 6.00E-04 | T | \|\| id:ebi-a-GCST90001697 | ebi-a-GCST90001697 | TRUE | G | 52408142 | 3.65E-08 | reported | 3425 | 2.869 | rs117975109 |  |  |  |  |  |  |  |  |  |  |  |  |  |  |  |
| 794.2 | 1 | igd | 6.00E-04 | T | \|\| id:ebi-a-GCST90001698 | ebi-a-GCST90001698 | TRUE | C | 6118693 | 2.61E-29 | reported | 3408 | 70 | rs11576563 |  |  |  |  |  |  |  |  |  |  |  |  |  |  |  |
| 731.4 | 1 | igd | 6.00E-04 | A | \|\| id:ebi-a-GCST90001698 | ebi-a-GCST90001698 | TRUE | G | 223544915 | 9.38E-26 | reported | 3408 | 69.14 | rs151086171 |  |  |  |  |  |  |  |  |  |  |  |  |  |  |  |
| 734.3 | 1 | igd | 6.00E-04 | C | \|\| id:ebi-a-GCST90001698 | ebi-a-GCST90001698 | TRUE | G | 206572177 | 7.51E-27 | reported | 3408 | 67.86 | rs373989598 |  |  |  |  |  |  |  |  |  |  |  |  |  |  |  |
| 749.7 | 2 | igd | 6.00E-04 | T | \|\| id:ebi-a-GCST90001698 | ebi-a-GCST90001698 | TRUE | A | 67789879 | 1.35E-18 | reported | 3408 | 84.69 | rs188021268 |  |  |  |  |  |  |  |  |  |  |  |  |  |  |  |
| 545.8 | 3 | igd | 6.00E-04 | T | \|\| id:ebi-a-GCST90001698 | ebi-a-GCST90001698 | TRUE | C | 89982367 | 5.87E-16 | reported | 3408 | 67.12 | rs147995494 |  |  |  |  |  |  |  |  |  |  |  |  |  |  |  |
| 759.4 | 3 | igd | 6.00E-04 | G | \|\| id:ebi-a-GCST90001698 | ebi-a-GCST90001698 | TRUE | A | 115341107 | 6.21E-27 | reported | 3408 | 70.06 | rs115821332 |  |  |  |  |  |  |  |  |  |  |  |  |  |  |  |
| 548.1 | 3 | igd | 6.00E-04 | A | \|\| id:ebi-a-GCST90001698 | ebi-a-GCST90001698 | TRUE | C | 90216749 | 4.65E-16 | reported | 3408 | 67.17 | rs185034429 |  |  |  |  |  |  |  |  |  |  |  |  |  |  |  |
| 570.9 | 3 | igd | 6.00E-04 | C | \|\| id:ebi-a-GCST90001698 | ebi-a-GCST90001698 | TRUE | T | 162179186 | 6.12E-16 | reported | 3408 | 70.25 | rs138871895 |  |  |  |  |  |  |  |  |  |  |  |  |  |  |  |
| 887.3 | 3 | igd | 6.00E-04 | A | \|\| id:ebi-a-GCST90001698 | ebi-a-GCST90001698 | TRUE | G | 117384981 | 2.03E-35 | reported | 3408 | 70.63 | rs185587352 |  |  |  |  |  |  |  |  |  |  |  |  |  |  |  |
| 483.6 | 4 | igd | 6.00E-04 | T | \|\| id:ebi-a-GCST90001698 | ebi-a-GCST90001698 | TRUE | A | 167039787 | 5.75E-13 | reported | 3408 | 66.84 | rs112958567 |  |  |  |  |  |  |  |  |  |  |  |  |  |  |  |
| 889.9 | 4 | igd | 6.00E-04 | A | \|\| id:ebi-a-GCST90001698 | ebi-a-GCST90001698 | TRUE | G | 176754667 | 1.04E-32 | reported | 3408 | 73.93 | rs116399750 |  |  |  |  |  |  |  |  |  |  |  |  |  |  |  |
| 919 | 4 | igd | 6.00E-04 | C | \|\| id:ebi-a-GCST90001698 | ebi-a-GCST90001698 | TRUE | T | 98435066 | 4.56E-44 | reported | 3408 | 65.06 | rs17026741 |  |  |  |  |  |  |  |  |  |  |  |  |  |  |  |
| 473.7 | 4 | igd | 6.00E-04 | G | \|\| id:ebi-a-GCST90001698 | ebi-a-GCST90001698 | TRUE | A | 119826754 | 7.35E-12 | reported | 3408 | 68.91 | rs72671613 |  |  |  |  |  |  |  |  |  |  |  |  |  |  |  |
| 929.9 | 4 | igd | 6.00E-04 | G | \|\| id:ebi-a-GCST90001698 | ebi-a-GCST90001698 | TRUE | A | 176150621 | 5.57E-34 | reported | 3408 | 75.68 | rs79964765 |  |  |  |  |  |  |  |  |  |  |  |  |  |  |  |
| 725.8 | 4 | igd | 6.00E-04 | G | \|\| id:ebi-a-GCST90001698 | ebi-a-GCST90001698 | TRUE | A | 71875684 | 2.81E-27 | reported | 3408 | 66.51 | rs114619811 |  |  |  |  |  |  |  |  |  |  |  |  |  |  |  |
| 512.2 | 4 | igd | 6.00E-04 | A | \|\| id:ebi-a-GCST90001698 | ebi-a-GCST90001698 | TRUE | C | 73027861 | 2.61E-13 | reported | 3408 | 69.76 | rs561331466 |  |  |  |  |  |  |  |  |  |  |  |  |  |  |  |
| 935.9 | 4 | igd | 6.00E-04 | T | \|\| id:ebi-a-GCST90001698 | ebi-a-GCST90001698 | TRUE | G | 98899399 | 3.76E-45 | reported | 3408 | 65.4 | rs115515703 |  |  |  |  |  |  |  |  |  |  |  |  |  |  |  |
| 1584 | 4 | igd | 6.00E-04 | T | \|\| id:ebi-a-GCST90001698 | ebi-a-GCST90001698 | TRUE | C | 176348299 | 1.79E-57 | reported | 3408 | 97.29 | rs79395509 |  |  |  |  |  |  |  |  |  |  |  |  |  |  |  |
| 422.8 | 4 | igd | 6.00E-04 | G | \|\| id:ebi-a-GCST90001698 | ebi-a-GCST90001698 | TRUE | C | 119007638 | 7.87E-09 | reported | 3408 | 73.07 | rs72674145 |  |  |  |  |  |  |  |  |  |  |  |  |  |  |  |
| 614.7 | 4 | igd | 6.00E-04 | A | \|\| id:ebi-a-GCST90001698 | ebi-a-GCST90001698 | TRUE | G | 103535765 | 5.79E-20 | reported | 3408 | 66.78 | rs4648125 |  |  |  |  |  |  |  |  |  |  |  |  |  |  |  |
| 1129 | 4 | igd | 6.00E-04 | G | \|\| id:ebi-a-GCST90001698 | ebi-a-GCST90001698 | TRUE | A | 99029457 | 2.50E-53 | reported | 3408 | 72.17 | rs12499962 |  |  |  |  |  |  |  |  |  |  |  |  |  |  |  |
| 937.1 | 4 | igd | 6.00E-04 | A | \|\| id:ebi-a-GCST90001698 | ebi-a-GCST90001698 | TRUE | G | 99216768 | 2.60E-45 | reported | 3408 | 65.36 | rs12498745 |  |  |  |  |  |  |  |  |  |  |  |  |  |  |  |
| 607.8 | 4 | igd | 6.00E-04 | G | \|\| id:ebi-a-GCST90001698 | ebi-a-GCST90001698 | TRUE | A | 130098084 | 6.97E-16 | reported | 3408 | 74.94 | rs78088167 |  |  |  |  |  |  |  |  |  |  |  |  |  |  |  |
| 864.3 | 5 | igd | 6.00E-04 | C | \|\| id:ebi-a-GCST90001698 | ebi-a-GCST90001698 | TRUE | T | 28612713 | 2.18E-31 | reported | 3408 | 73.42 | rs76019287 |  |  |  |  |  |  |  |  |  |  |  |  |  |  |  |
| 1006 | 5 | igd | 6.00E-04 | C | \|\| id:ebi-a-GCST90001698 | ebi-a-GCST90001698 | TRUE | T | 29413612 | 2.29E-36 | reported | 3408 | 78.94 | rs116581718 |  |  |  |  |  |  |  |  |  |  |  |  |  |  |  |
| 419.3 | 7 | igd | 6.00E-04 | T | \|\| id:ebi-a-GCST90001698 | ebi-a-GCST90001698 | TRUE | C | 29025284 | 1.23E-10 | reported | 3408 | 64.95 | rs138449036 |  |  |  |  |  |  |  |  |  |  |  |  |  |  |  |
| 410.8 | 8 | igd | 6.00E-04 | A | \|\| id:ebi-a-GCST90001698 | ebi-a-GCST90001698 | TRUE | G | 47330299 | 2.77E-09 | reported | 3408 | 68.92 | rs115602951 |  |  |  |  |  |  |  |  |  |  |  |  |  |  |  |
| 414.3 | 8 | igd | 6.00E-04 | T | \|\| id:ebi-a-GCST90001698 | ebi-a-GCST90001698 | TRUE | C | 47021629 | 2.32E-09 | reported | 3408 | 69.16 | rs118140166 |  |  |  |  |  |  |  |  |  |  |  |  |  |  |  |
| 420.7 | 8 | igd | 6.00E-04 | G | \|\| id:ebi-a-GCST90001698 | ebi-a-GCST90001698 | TRUE | T | 43138280 | 1.73E-09 | reported | 3408 | 69.68 | rs146628758 |  |  |  |  |  |  |  |  |  |  |  |  |  |  |  |
| 501.5 | 9 | igd | 6.00E-04 | C | \|\| id:ebi-a-GCST90001698 | ebi-a-GCST90001698 | TRUE | G | 6258531 | 1.85E-14 | reported | 3408 | 65.17 | rs147790588 |  |  |  |  |  |  |  |  |  |  |  |  |  |  |  |
| 539 | 10 | igd | 6.00E-04 | A | \|\| id:ebi-a-GCST90001698 | ebi-a-GCST90001698 | TRUE | C | 49362940 | 5.34E-15 | reported | 3408 | 68.63 | rs188781836 |  |  |  |  |  |  |  |  |  |  |  |  |  |  |  |
| 665 | 11 | igd | 6.00E-04 | G | \|\| id:ebi-a-GCST90001698 | ebi-a-GCST90001698 | TRUE | A | 83079124 | 4.84E-23 | reported | 3408 | 66.79 | rs117911420 |  |  |  |  |  |  |  |  |  |  |  |  |  |  |  |
| 405.9 | 11 | igd | 6.00E-04 | G | \|\| id:ebi-a-GCST90001698 | ebi-a-GCST90001698 | TRUE | A | 124958100 | 1.19E-08 | reported | 3408 | 71.03 | rs146696496 |  |  |  |  |  |  |  |  |  |  |  |  |  |  |  |
| 834.4 | 11 | igd | 6.00E-04 | G | \|\| id:ebi-a-GCST90001698 | ebi-a-GCST90001698 | TRUE | A | 121746998 | 6.48E-25 | reported | 3408 | 80.31 | rs116916598 |  |  |  |  |  |  |  |  |  |  |  |  |  |  |  |
| 437.7 | 12 | igd | 6.00E-04 | T | \|\| id:ebi-a-GCST90001698 | ebi-a-GCST90001698 | TRUE | C | 52437438 | 2.64E-10 | reported | 3408 | 69.06 | rs117293367 |  |  |  |  |  |  |  |  |  |  |  |  |  |  |  |
| 559.9 | 13 | igd | 6.00E-04 | T | \|\| id:ebi-a-GCST90001698 | ebi-a-GCST90001698 | TRUE | G | 105831838 | 1.22E-15 | reported | 3408 | 69.63 | rs75592465 |  |  |  |  |  |  |  |  |  |  |  |  |  |  |  |
| 584.1 | 14 | igd | 6.00E-04 | A | \|\| id:ebi-a-GCST90001698 | ebi-a-GCST90001698 | TRUE | G | 34919717 | 5.22E-17 | reported | 3408 | 69.32 | rs71419987 |  |  |  |  |  |  |  |  |  |  |  |  |  |  |  |
| 628.3 | 15 | igd | 6.00E-04 | A | \|\| id:ebi-a-GCST90001698 | ebi-a-GCST90001698 | TRUE | G | 84950824 | 3.68E-22 | reported | 3408 | 64.46 | rs181228225 |  |  |  |  |  |  |  |  |  |  |  |  |  |  |  |
| 480.5 | 16 | igd | 6.00E-04 | A | \|\| id:ebi-a-GCST90001698 | ebi-a-GCST90001698 | TRUE | G | 22611639 | 1.16E-09 | reported | 3408 | 78.74 | rs529447294 |  |  |  |  |  |  |  |  |  |  |  |  |  |  |  |
| 434.2 | 16 | igd | 6.00E-04 | A | \|\| id:ebi-a-GCST90001698 | ebi-a-GCST90001698 | TRUE | C | 88701572 | 7.20E-12 | reported | 3408 | 63.13 | rs78888262 |  |  |  |  |  |  |  |  |  |  |  |  |  |  |  |
| 591.4 | 17 | igd | 6.00E-04 | A | \|\| id:ebi-a-GCST90001698 | ebi-a-GCST90001698 | TRUE | G | 5023557 | 7.80E-16 | reported | 3408 | 73.04 | rs144104650 |  |  |  |  |  |  |  |  |  |  |  |  |  |  |  |
| 785.2 | 17 | igd | 6.00E-04 | G | \|\| id:ebi-a-GCST90001698 | ebi-a-GCST90001698 | TRUE | A | 30028367 | 4.89E-28 | reported | 3408 | 70.89 | rs117922931 |  |  |  |  |  |  |  |  |  |  |  |  |  |  |  |
| 854.9 | 18 | igd | 6.00E-04 | C | \|\| id:ebi-a-GCST90001698 | ebi-a-GCST90001698 | TRUE | T | 3305228 | 3.13E-30 | reported | 3408 | 74.11 | rs117745825 |  |  |  |  |  |  |  |  |  |  |  |  |  |  |  |
| 643.8 | 19 | igd | 6.00E-04 | A | \|\| id:ebi-a-GCST90001698 | ebi-a-GCST90001698 | TRUE | G | 9246656 | 1.54E-15 | reported | 3408 | 80.36 | rs7254864 |  |  |  |  |  |  |  |  |  |  |  |  |  |  |  |
| 1000 | 20 | igd | 6.00E-04 | T | \|\| id:ebi-a-GCST90001698 | ebi-a-GCST90001698 | TRUE | G | 33104122 | 1.18E-41 | reported | 3408 | 72.99 | rs137942933 |  |  |  |  |  |  |  |  |  |  |  |  |  |  |  |
| 751.8 | 20 | igd | 6.00E-04 | C | \|\| id:ebi-a-GCST90001698 | ebi-a-GCST90001698 | TRUE | T | 17556587 | 1.00E-30 | reported | 3408 | 64.6 | rs17791728 |  |  |  |  |  |  |  |  |  |  |  |  |  |  |  |
| 769.4 | 21 | igd | 6.00E-04 | T | \|\| id:ebi-a-GCST90001698 | ebi-a-GCST90001698 | TRUE | C | 24506038 | 3.43E-27 | reported | 3408 | 70.62 | rs138911069 |  |  |  |  |  |  |  |  |  |  |  |  |  |  |  |
| 18.17 | 3 | igd | 6.00E-04 | A | \|\| id:ebi-a-GCST90001700 | ebi-a-GCST90001700 | TRUE | G | 117384981 | 3.62E-09 | reported | 3425 | 3.072 | rs185587352 |  |  |  |  |  |  |  |  |  |  |  |  |  |  |  |
| 22.18 | 4 | igd | 6.00E-04 | C | \|\| id:ebi-a-GCST90001700 | ebi-a-GCST90001700 | TRUE | T | 98435066 | 1.09E-14 | reported | 3425 | 2.857 | rs17026741 |  |  |  |  |  |  |  |  |  |  |  |  |  |  |  |
| 22.1 | 4 | igd | 6.00E-04 | A | \|\| id:ebi-a-GCST90001700 | ebi-a-GCST90001700 | TRUE | G | 103535765 | 2.13E-14 | reported | 3425 | 2.879 | rs4648125 |  |  |  |  |  |  |  |  |  |  |  |  |  |  |  |
| 17.96 | 4 | igd | 6.00E-04 | A | \|\| id:ebi-a-GCST90001700 | ebi-a-GCST90001700 | TRUE | G | 176754667 | 2.27E-08 | reported | 3425 | 3.205 | rs116399750 |  |  |  |  |  |  |  |  |  |  |  |  |  |  |  |
| 26.23 | 4 | igd | 6.00E-04 | T | \|\| id:ebi-a-GCST90001700 | ebi-a-GCST90001700 | TRUE | C | 176348299 | 4.38E-10 | reported | 3425 | 4.191 | rs79395509 |  |  |  |  |  |  |  |  |  |  |  |  |  |  |  |
| 22.76 | 4 | igd | 6.00E-04 | T | \|\| id:ebi-a-GCST90001700 | ebi-a-GCST90001700 | TRUE | G | 98899399 | 3.27E-15 | reported | 3425 | 2.875 | rs115515703 |  |  |  |  |  |  |  |  |  |  |  |  |  |  |  |
| 25.15 | 4 | igd | 6.00E-04 | G | \|\| id:ebi-a-GCST90001700 | ebi-a-GCST90001700 | TRUE | A | 99029457 | 1.59E-15 | reported | 3425 | 3.141 | rs12499962 |  |  |  |  |  |  |  |  |  |  |  |  |  |  |  |
| 22.86 | 4 | igd | 6.00E-04 | A | \|\| id:ebi-a-GCST90001700 | ebi-a-GCST90001700 | TRUE | G | 99216768 | 2.39E-15 | reported | 3425 | 2.873 | rs12498745 |  |  |  |  |  |  |  |  |  |  |  |  |  |  |  |
| 19.81 | 4 | igd | 6.00E-04 | G | \|\| id:ebi-a-GCST90001700 | ebi-a-GCST90001700 | TRUE | A | 130098084 | 8.99E-10 | reported | 3425 | 3.224 | rs78088167 |  |  |  |  |  |  |  |  |  |  |  |  |  |  |  |
| 17.53 | 4 | igd | 6.00E-04 | G | \|\| id:ebi-a-GCST90001700 | ebi-a-GCST90001700 | TRUE | A | 119826754 | 3.67E-09 | reported | 3425 | 2.964 | rs72671613 |  |  |  |  |  |  |  |  |  |  |  |  |  |  |  |
| 19.52 | 5 | igd | 6.00E-04 | C | \|\| id:ebi-a-GCST90001700 | ebi-a-GCST90001700 | TRUE | T | 28612713 | 2.98E-10 | reported | 3425 | 3.09 | rs76019287 |  |  |  |  |  |  |  |  |  |  |  |  |  |  |  |
| 22.4 | 5 | igd | 6.00E-04 | C | \|\| id:ebi-a-GCST90001700 | ebi-a-GCST90001700 | TRUE | T | 29413612 | 1.35E-11 | reported | 3425 | 3.301 | rs116581718 |  |  |  |  |  |  |  |  |  |  |  |  |  |  |  |
| 19.07 | 11 | igd | 6.00E-04 | G | \|\| id:ebi-a-GCST90001700 | ebi-a-GCST90001700 | TRUE | A | 121746998 | 2.33E-08 | reported | 3425 | 3.407 | rs116916598 |  |  |  |  |  |  |  |  |  |  |  |  |  |  |  |
| 19.65 | 13 | igd | 6.00E-04 | G | \|\| id:ebi-a-GCST90001700 | ebi-a-GCST90001700 | TRUE | A | 83713677 | 6.91E-11 | reported | 3425 | 3.003 | rs78848995 |  |  |  |  |  |  |  |  |  |  |  |  |  |  |  |
| 23.37 | 20 | igd | 6.00E-04 | T | \|\| id:ebi-a-GCST90001700 | ebi-a-GCST90001700 | TRUE | G | 33104122 | 1.58E-13 | reported | 3425 | 3.154 | rs137942933 |  |  |  |  |  |  |  |  |  |  |  |  |  |  |  |
| 15.79 | 20 | igd | 6.00E-04 | C | \|\| id:ebi-a-GCST90001700 | ebi-a-GCST90001700 | TRUE | T | 17556587 | 2.10E-08 | reported | 3425 | 2.811 | rs17791728 |  |  |  |  |  |  |  |  |  |  |  |  |  |  |  |
| -3.125 | 1 | igd | 5.00E-04 | C | \|\| id:ebi-a-GCST90001917 | ebi-a-GCST90001917 | TRUE | T | 42691989 | 3.93E-08 | reported | 3113 | 0.5674 | rs116030815 |  |  |  |  |  |  |  |  |  |  |  |  |  |  |  |
| 1.818 | 6 | igd | 4.00E-04 | T | \|\| id:ebi-a-GCST90001688 | ebi-a-GCST90001688 | TRUE | C | 148775706 | 1.17E-09 | reported | 3440 | 0.2979 | rs117475733 |  |  |  |  |  |  |  |  |  |  |  |  |  |  |  |
| 530.8 | 1 | igd | 4.00E-04 | C | \|\| id:ebi-a-GCST90001695 | ebi-a-GCST90001695 | TRUE | A | 83046200 | 1.81E-19 | reported | 3408 | 58.46 | rs150650566 |  |  |  |  |  |  |  |  |  |  |  |  |  |  |  |
| 465.1 | 1 | igd | 4.00E-04 | T | \|\| id:ebi-a-GCST90001695 | ebi-a-GCST90001695 | TRUE | A | 79294084 | 3.38E-15 | reported | 3408 | 58.77 | rs115976158 |  |  |  |  |  |  |  |  |  |  |  |  |  |  |  |
| 463.8 | 1 | igd | 4.00E-04 | A | \|\| id:ebi-a-GCST90001695 | ebi-a-GCST90001695 | TRUE | G | 79094990 | 2.46E-15 | reported | 3408 | 58.31 | rs138829583 |  |  |  |  |  |  |  |  |  |  |  |  |  |  |  |
| 405.6 | 2 | igd | 4.00E-04 | A | \|\| id:ebi-a-GCST90001695 | ebi-a-GCST90001695 | TRUE | G | 229222112 | 5.84E-11 | reported | 3408 | 61.74 | rs74932477 |  |  |  |  |  |  |  |  |  |  |  |  |  |  |  |
| 521.2 | 2 | igd | 4.00E-04 | A | \|\| id:ebi-a-GCST90001695 | ebi-a-GCST90001695 | TRUE | T | 106552757 | 3.40E-13 | reported | 3408 | 71.33 | rs151288286 |  |  |  |  |  |  |  |  |  |  |  |  |  |  |  |
| 641.1 | 2 | igd | 4.00E-04 | A | \|\| id:ebi-a-GCST90001695 | ebi-a-GCST90001695 | TRUE | G | 228411157 | 2.24E-21 | reported | 3408 | 67.08 | rs137978465 |  |  |  |  |  |  |  |  |  |  |  |  |  |  |  |
| 556.4 | 3 | igd | 4.00E-04 | A | \|\| id:ebi-a-GCST90001695 | ebi-a-GCST90001695 | TRUE | G | 15790899 | 4.51E-11 | reported | 3408 | 84.21 | rs141023610 |  |  |  |  |  |  |  |  |  |  |  |  |  |  |  |
| 548.7 | 4 | igd | 4.00E-04 | T | \|\| id:ebi-a-GCST90001695 | ebi-a-GCST90001695 | TRUE | C | 106862213 | 3.01E-08 | reported | 3408 | 98.8 | rs55830085 |  |  |  |  |  |  |  |  |  |  |  |  |  |  |  |
| 492.9 | 4 | igd | 4.00E-04 | C | \|\| id:ebi-a-GCST90001695 | ebi-a-GCST90001695 | TRUE | T | 115997346 | 3.51E-10 | reported | 3408 | 78.33 | rs138579687 |  |  |  |  |  |  |  |  |  |  |  |  |  |  |  |
| 397.6 | 4 | igd | 4.00E-04 | A | \|\| id:ebi-a-GCST90001695 | ebi-a-GCST90001695 | TRUE | G | 64142694 | 3.50E-08 | reported | 3408 | 71.92 | rs79870119 |  |  |  |  |  |  |  |  |  |  |  |  |  |  |  |
| 383.2 | 5 | igd | 4.00E-04 | T | \|\| id:ebi-a-GCST90001695 | ebi-a-GCST90001695 | TRUE | A | 159062854 | 7.72E-11 | reported | 3408 | 58.71 | rs115546994 |  |  |  |  |  |  |  |  |  |  |  |  |  |  |  |
| 521.4 | 7 | igd | 4.00E-04 | T | \|\| id:ebi-a-GCST90001695 | ebi-a-GCST90001695 | TRUE | C | 142438302 | 2.08E-09 | reported | 3408 | 86.78 | rs79647440 |  |  |  |  |  |  |  |  |  |  |  |  |  |  |  |
| 372.4 | 8 | igd | 4.00E-04 | T | \|\| id:ebi-a-GCST90001695 | ebi-a-GCST90001695 | TRUE | C | 131518018 | 6.07E-09 | reported | 3408 | 63.88 | rs149487182 |  |  |  |  |  |  |  |  |  |  |  |  |  |  |  |
| 402.2 | 9 | igd | 4.00E-04 | A | \|\| id:ebi-a-GCST90001695 | ebi-a-GCST90001695 | TRUE | G | 96711406 | 4.24E-11 | reported | 3408 | 60.78 | rs151120803 |  |  |  |  |  |  |  |  |  |  |  |  |  |  |  |
| 385.7 | 11 | igd | 4.00E-04 | G | \|\| id:ebi-a-GCST90001695 | ebi-a-GCST90001695 | TRUE | A | 44356396 | 2.31E-08 | reported | 3408 | 68.87 | rs117431224 |  |  |  |  |  |  |  |  |  |  |  |  |  |  |  |
| 364.8 | 11 | igd | 4.00E-04 | C | \|\| id:ebi-a-GCST90001695 | ebi-a-GCST90001695 | TRUE | T | 16290338 | 3.57E-08 | reported | 3408 | 66.04 | rs117413806 |  |  |  |  |  |  |  |  |  |  |  |  |  |  |  |
| 370 | 12 | igd | 4.00E-04 | C | \|\| id:ebi-a-GCST90001695 | ebi-a-GCST90001695 | TRUE | G | 2808166 | 4.28E-08 | reported | 3408 | 67.38 | rs61908026 |  |  |  |  |  |  |  |  |  |  |  |  |  |  |  |
| 442.7 | 13 | igd | 4.00E-04 | T | \|\| id:ebi-a-GCST90001695 | ebi-a-GCST90001695 | TRUE | C | 100586942 | 3.90E-12 | reported | 3408 | 63.56 | rs113819655 |  |  |  |  |  |  |  |  |  |  |  |  |  |  |  |
| 368.8 | 13 | igd | 4.00E-04 | T | \|\| id:ebi-a-GCST90001695 | ebi-a-GCST90001695 | TRUE | C | 44264686 | 2.59E-08 | reported | 3408 | 66.09 | rs117048727 |  |  |  |  |  |  |  |  |  |  |  |  |  |  |  |
| 368.8 | 13 | igd | 4.00E-04 | T | \|\| id:ebi-a-GCST90001695 | ebi-a-GCST90001695 | TRUE | G | 94341740 | 2.59E-08 | reported | 3408 | 66.09 | rs80012883 |  |  |  |  |  |  |  |  |  |  |  |  |  |  |  |
| 363.2 | 18 | igd | 4.00E-04 | C | \|\| id:ebi-a-GCST90001695 | ebi-a-GCST90001695 | TRUE | G | 3267975 | 1.59E-09 | reported | 3408 | 60.02 | rs148362510 |  |  |  |  |  |  |  |  |  |  |  |  |  |  |  |
| 472.2 | 19 | igd | 4.00E-04 | A | \|\| id:ebi-a-GCST90001695 | ebi-a-GCST90001695 | TRUE | C | 43422957 | 2.03E-11 | reported | 3408 | 70.2 | rs117428784 |  |  |  |  |  |  |  |  |  |  |  |  |  |  |  |
| 358.8 | 20 | igd | 4.00E-04 | C | \|\| id:ebi-a-GCST90001695 | ebi-a-GCST90001695 | TRUE | T | 20799344 | 2.28E-10 | reported | 3408 | 56.41 | rs77937827 |  |  |  |  |  |  |  |  |  |  |  |  |  |  |  |
| 15.77 | 5 | igd | 4.00E-04 | T | \|\| id:ebi-a-GCST90001697 | ebi-a-GCST90001697 | TRUE | A | 159062854 | 7.76E-09 | reported | 3425 | 2.724 | rs115546994 |  |  |  |  |  |  |  |  |  |  |  |  |  |  |  |
| 20.95 | 8 | igd | 4.00E-04 | T | \|\| id:ebi-a-GCST90001697 | ebi-a-GCST90001697 | TRUE | C | 131518018 | 2.46E-12 | reported | 3425 | 2.979 | rs149487182 |  |  |  |  |  |  |  |  |  |  |  |  |  |  |  |
| 16.44 | 10 | igd | 4.00E-04 | G | \|\| id:ebi-a-GCST90001697 | ebi-a-GCST90001697 | TRUE | A | 108143796 | 1.62E-09 | reported | 3425 | 2.719 | rs117006645 |  |  |  |  |  |  |  |  |  |  |  |  |  |  |  |
| 20.33 | 11 | igd | 4.00E-04 | C | \|\| id:ebi-a-GCST90001697 | ebi-a-GCST90001697 | TRUE | T | 54986405 | 3.68E-08 | reported | 3425 | 3.685 | rs146027781 |  |  |  |  |  |  |  |  |  |  |  |  |  |  |  |
| 19.12 | 12 | igd | 4.00E-04 | C | \|\| id:ebi-a-GCST90001697 | ebi-a-GCST90001697 | TRUE | G | 2808166 | 1.56E-09 | reported | 3425 | 3.159 | rs61908026 |  |  |  |  |  |  |  |  |  |  |  |  |  |  |  |
| 15.42 | 16 | igd | 4.00E-04 | C | \|\| id:ebi-a-GCST90001697 | ebi-a-GCST90001697 | TRUE | G | 1037963 | 8.83E-09 | reported | 3425 | 2.675 | rs151077900 |  |  |  |  |  |  |  |  |  |  |  |  |  |  |  |
| 16.37 | 19 | igd | 4.00E-04 | A | \|\| id:ebi-a-GCST90001697 | ebi-a-GCST90001697 | TRUE | G | 43539305 | 4.72E-09 | reported | 3425 | 2.788 | rs144764562 |  |  |  |  |  |  |  |  |  |  |  |  |  |  |  |
| 24.71 | 19 | igd | 4.00E-04 | A | \|\| id:ebi-a-GCST90001697 | ebi-a-GCST90001697 | TRUE | C | 43422957 | 4.54E-14 | reported | 3425 | 3.261 | rs117428784 |  |  |  |  |  |  |  |  |  |  |  |  |  |  |  |
| 701.2 | 1 | igd | 4.00E-04 | C | \|\| id:ebi-a-GCST90001698 | ebi-a-GCST90001698 | TRUE | G | 12228810 | 9.39E-20 | reported | 3408 | 76.62 | rs595254 |  |  |  |  |  |  |  |  |  |  |  |  |  |  |  |
| 1256 | 1 | igd | 4.00E-04 | A | \|\| id:ebi-a-GCST90001698 | ebi-a-GCST90001698 | TRUE | G | 234912820 | 2.73E-31 | reported | 3408 | 106.9 | rs554542958 |  |  |  |  |  |  |  |  |  |  |  |  |  |  |  |
| 584.2 | 2 | igd | 4.00E-04 | T | \|\| id:ebi-a-GCST90001698 | ebi-a-GCST90001698 | TRUE | C | 201091838 | 5.15E-15 | reported | 3408 | 74.33 | rs13001274 |  |  |  |  |  |  |  |  |  |  |  |  |  |  |  |
| 1573 | 2 | igd | 4.00E-04 | A | \|\| id:ebi-a-GCST90001698 | ebi-a-GCST90001698 | TRUE | T | 106552757 | 2.91E-61 | reported | 3408 | 93.35 | rs151288286 |  |  |  |  |  |  |  |  |  |  |  |  |  |  |  |
| 658.4 | 2 | igd | 4.00E-04 | A | \|\| id:ebi-a-GCST90001698 | ebi-a-GCST90001698 | TRUE | G | 229222112 | 2.02E-15 | reported | 3408 | 82.54 | rs74932477 |  |  |  |  |  |  |  |  |  |  |  |  |  |  |  |
| 568.9 | 2 | igd | 4.00E-04 | C | \|\| id:ebi-a-GCST90001698 | ebi-a-GCST90001698 | TRUE | T | 211172214 | 6.60E-13 | reported | 3408 | 78.84 | rs72996438 |  |  |  |  |  |  |  |  |  |  |  |  |  |  |  |
| 602.1 | 3 | igd | 4.00E-04 | C | \|\| id:ebi-a-GCST90001698 | ebi-a-GCST90001698 | TRUE | T | 87180533 | 9.30E-16 | reported | 3408 | 74.57 | rs144886167 |  |  |  |  |  |  |  |  |  |  |  |  |  |  |  |
| 632.9 | 3 | igd | 4.00E-04 | A | \|\| id:ebi-a-GCST90001698 | ebi-a-GCST90001698 | TRUE | G | 129232254 | 5.07E-17 | reported | 3408 | 75.09 | rs75660425 |  |  |  |  |  |  |  |  |  |  |  |  |  |  |  |
| 1007 | 3 | igd | 4.00E-04 | G | \|\| id:ebi-a-GCST90001698 | ebi-a-GCST90001698 | TRUE | A | 152281325 | 6.27E-35 | reported | 3408 | 80.78 | rs2700341 |  |  |  |  |  |  |  |  |  |  |  |  |  |  |  |
| 1083 | 3 | igd | 4.00E-04 | G | \|\| id:ebi-a-GCST90001698 | ebi-a-GCST90001698 | TRUE | C | 151051554 | 5.76E-41 | reported | 3408 | 79.72 | rs4680357 |  |  |  |  |  |  |  |  |  |  |  |  |  |  |  |
| 720.8 | 4 | igd | 4.00E-04 | A | \|\| id:ebi-a-GCST90001698 | ebi-a-GCST90001698 | TRUE | T | 85213802 | 4.27E-17 | reported | 3408 | 85.31 | rs114559319 |  |  |  |  |  |  |  |  |  |  |  |  |  |  |  |
| 836.1 | 4 | igd | 4.00E-04 | C | \|\| id:ebi-a-GCST90001698 | ebi-a-GCST90001698 | TRUE | T | 32184694 | 1.75E-29 | reported | 3408 | 73.46 | rs138954494 |  |  |  |  |  |  |  |  |  |  |  |  |  |  |  |
| 634.9 | 4 | igd | 4.00E-04 | T | \|\| id:ebi-a-GCST90001698 | ebi-a-GCST90001698 | TRUE | C | 129754843 | 5.64E-15 | reported | 3408 | 80.9 | rs76679968 |  |  |  |  |  |  |  |  |  |  |  |  |  |  |  |
| 1165 | 4 | igd | 4.00E-04 | A | \|\| id:ebi-a-GCST90001698 | ebi-a-GCST90001698 | TRUE | G | 85578601 | 6.83E-42 | reported | 3408 | 84.75 | rs146374482 |  |  |  |  |  |  |  |  |  |  |  |  |  |  |  |
| 662.4 | 4 | igd | 4.00E-04 | A | \|\| id:ebi-a-GCST90001698 | ebi-a-GCST90001698 | TRUE | G | 138349095 | 9.84E-16 | reported | 3408 | 82.11 | rs114685875 |  |  |  |  |  |  |  |  |  |  |  |  |  |  |  |
| 544.6 | 4 | igd | 4.00E-04 | T | \|\| id:ebi-a-GCST90001698 | ebi-a-GCST90001698 | TRUE | C | 129607171 | 5.28E-13 | reported | 3408 | 75.15 | rs78792541 |  |  |  |  |  |  |  |  |  |  |  |  |  |  |  |
| 1117 | 5 | igd | 4.00E-04 | C | \|\| id:ebi-a-GCST90001698 | ebi-a-GCST90001698 | TRUE | T | 94705605 | 3.23E-39 | reported | 3408 | 84.15 | rs79725470 |  |  |  |  |  |  |  |  |  |  |  |  |  |  |  |
| 988.1 | 5 | igd | 4.00E-04 | T | \|\| id:ebi-a-GCST90001698 | ebi-a-GCST90001698 | TRUE | C | 75564943 | 6.58E-35 | reported | 3408 | 79.27 | rs189412340 |  |  |  |  |  |  |  |  |  |  |  |  |  |  |  |
| 905.9 | 6 | igd | 4.00E-04 | T | \|\| id:ebi-a-GCST90001698 | ebi-a-GCST90001698 | TRUE | C | 8229697 | 6.58E-32 | reported | 3408 | 76.27 | rs72820159 |  |  |  |  |  |  |  |  |  |  |  |  |  |  |  |
| 774.8 | 7 | igd | 4.00E-04 | T | \|\| id:ebi-a-GCST90001698 | ebi-a-GCST90001698 | TRUE | C | 46985321 | 2.02E-19 | reported | 3408 | 85.45 | rs80018878 |  |  |  |  |  |  |  |  |  |  |  |  |  |  |  |
| 1574 | 7 | igd | 4.00E-04 | T | \|\| id:ebi-a-GCST90001698 | ebi-a-GCST90001698 | TRUE | C | 142438302 | 1.91E-42 | reported | 3408 | 113.7 | rs79647440 |  |  |  |  |  |  |  |  |  |  |  |  |  |  |  |
| 751.9 | 9 | igd | 4.00E-04 | T | \|\| id:ebi-a-GCST90001698 | ebi-a-GCST90001698 | TRUE | C | 10278984 | 1.83E-21 | reported | 3408 | 78.49 | rs148835086 |  |  |  |  |  |  |  |  |  |  |  |  |  |  |  |
| 584.7 | 10 | igd | 4.00E-04 | T | \|\| id:ebi-a-GCST90001698 | ebi-a-GCST90001698 | TRUE | C | 92600913 | 2.60E-14 | reported | 3408 | 76.44 | rs72810820 |  |  |  |  |  |  |  |  |  |  |  |  |  |  |  |
| 479.2 | 10 | igd | 4.00E-04 | G | \|\| id:ebi-a-GCST90001698 | ebi-a-GCST90001698 | TRUE | T | 66659421 | 2.19E-09 | reported | 3408 | 79.88 | rs145835734 |  |  |  |  |  |  |  |  |  |  |  |  |  |  |  |
| 492 | 10 | igd | 4.00E-04 | T | \|\| id:ebi-a-GCST90001698 | ebi-a-GCST90001698 | TRUE | C | 66557993 | 1.76E-09 | reported | 3408 | 81.52 | rs116917274 |  |  |  |  |  |  |  |  |  |  |  |  |  |  |  |
| 658.4 | 10 | igd | 4.00E-04 | G | \|\| id:ebi-a-GCST90001698 | ebi-a-GCST90001698 | TRUE | A | 85916001 | 2.61E-15 | reported | 3408 | 82.87 | rs74322543 |  |  |  |  |  |  |  |  |  |  |  |  |  |  |  |
| 1592 | 11 | igd | 4.00E-04 | C | \|\| id:ebi-a-GCST90001698 | ebi-a-GCST90001698 | TRUE | T | 83560886 | 6.43E-56 | reported | 3408 | 99.22 | rs78268719 |  |  |  |  |  |  |  |  |  |  |  |  |  |  |  |
| 985.5 | 12 | igd | 4.00E-04 | C | \|\| id:ebi-a-GCST90001698 | ebi-a-GCST90001698 | TRUE | A | 10043518 | 1.12E-35 | reported | 3408 | 78.14 | rs10505739 |  |  |  |  |  |  |  |  |  |  |  |  |  |  |  |
| 1015 | 19 | igd | 4.00E-04 | T | \|\| id:ebi-a-GCST90001698 | ebi-a-GCST90001698 | TRUE | C | 46587710 | 8.89E-35 | reported | 3408 | 81.57 | rs112604811 |  |  |  |  |  |  |  |  |  |  |  |  |  |  |  |
| 953.9 | 21 | igd | 4.00E-04 | A | \|\| id:ebi-a-GCST90001698 | ebi-a-GCST90001698 | TRUE | G | 24128149 | 3.10E-35 | reported | 3408 | 76.14 | rs118173242 |  |  |  |  |  |  |  |  |  |  |  |  |  |  |  |
| 35.17 | 2 | igd | 4.00E-04 | A | \|\| id:ebi-a-GCST90001700 | ebi-a-GCST90001700 | TRUE | T | 106552757 | 5.32E-18 | reported | 3425 | 4.045 | rs151288286 |  |  |  |  |  |  |  |  |  |  |  |  |  |  |  |
| 18.96 | 3 | igd | 4.00E-04 | T | \|\| id:ebi-a-GCST90001700 | ebi-a-GCST90001700 | TRUE | C | 72625988 | 4.09E-08 | reported | 3425 | 3.448 | rs148750394 |  |  |  |  |  |  |  |  |  |  |  |  |  |  |  |
| 19.84 | 3 | igd | 4.00E-04 | G | \|\| id:ebi-a-GCST90001700 | ebi-a-GCST90001700 | TRUE | C | 151051554 | 1.38E-08 | reported | 3425 | 3.487 | rs4680357 |  |  |  |  |  |  |  |  |  |  |  |  |  |  |  |
| 24.25 | 4 | igd | 4.00E-04 | T | \|\| id:ebi-a-GCST90001700 | ebi-a-GCST90001700 | TRUE | G | 55167665 | 3.39E-11 | reported | 3425 | 3.646 | rs115318535 |  |  |  |  |  |  |  |  |  |  |  |  |  |  |  |
| 37.38 | 7 | igd | 4.00E-04 | T | \|\| id:ebi-a-GCST90001700 | ebi-a-GCST90001700 | TRUE | C | 142438302 | 3.94E-14 | reported | 3425 | 4.922 | rs79647440 |  |  |  |  |  |  |  |  |  |  |  |  |  |  |  |
| 22.98 | 7 | igd | 4.00E-04 | T | \|\| id:ebi-a-GCST90001700 | ebi-a-GCST90001700 | TRUE | C | 137598761 | 7.13E-10 | reported | 3425 | 3.719 | rs35614905 |  |  |  |  |  |  |  |  |  |  |  |  |  |  |  |
| 27.01 | 11 | igd | 4.00E-04 | C | \|\| id:ebi-a-GCST90001700 | ebi-a-GCST90001700 | TRUE | T | 83560886 | 7.30E-10 | reported | 3425 | 4.372 | rs78268719 |  |  |  |  |  |  |  |  |  |  |  |  |  |  |  |
| 18.77 | 11 | igd | 4.00E-04 | A | \|\| id:ebi-a-GCST90001700 | ebi-a-GCST90001700 | TRUE | G | 24544063 | 2.10E-08 | reported | 3425 | 3.342 | rs117173207 |  |  |  |  |  |  |  |  |  |  |  |  |  |  |  |
| 20.04 | 11 | igd | 4.00E-04 | A | \|\| id:ebi-a-GCST90001700 | ebi-a-GCST90001700 | TRUE | C | 82042361 | 3.18E-08 | reported | 3425 | 3.615 | rs2138000 |  |  |  |  |  |  |  |  |  |  |  |  |  |  |  |
| 18.81 | 12 | igd | 4.00E-04 | C | \|\| id:ebi-a-GCST90001700 | ebi-a-GCST90001700 | TRUE | A | 10043518 | 3.21E-08 | reported | 3425 | 3.393 | rs10505739 |  |  |  |  |  |  |  |  |  |  |  |  |  |  |  |
| 3.098 | 1 | igd | 3.00E-04 | C | \|\| id:ebi-a-GCST90001561 | ebi-a-GCST90001561 | TRUE | T | 214625781 | 3.03E-08 | reported | 3427 | 0.5579 | rs150956189 |  |  |  |  |  |  |  |  |  |  |  |  |  |  |  |
| 3.341 | 3 | igd | 3.00E-04 | T | \|\| id:ebi-a-GCST90001666 | ebi-a-GCST90001666 | TRUE | C | 84799491 | 2.58E-08 | reported | 3408 | 0.5986 | rs74577787 |  |  |  |  |  |  |  |  |  |  |  |  |  |  |  |
| 3.323 | 3 | igd | 3.00E-04 | C | \|\| id:ebi-a-GCST90001666 | ebi-a-GCST90001666 | TRUE | T | 84455642 | 3.06E-08 | reported | 3408 | 0.5987 | rs116784631 |  |  |  |  |  |  |  |  |  |  |  |  |  |  |  |
| 3.334 | 6 | igd | 3.00E-04 | A | \|\| id:ebi-a-GCST90001666 | ebi-a-GCST90001666 | TRUE | C | 149114553 | 2.76E-08 | reported | 3408 | 0.5986 | rs112637846 |  |  |  |  |  |  |  |  |  |  |  |  |  |  |  |
| 420.4 | 1 | igd | 3.00E-04 | A | \|\| id:ebi-a-GCST90001695 | ebi-a-GCST90001695 | TRUE | G | 77340084 | 3.37E-08 | reported | 3408 | 75.98 | rs182911396 |  |  |  |  |  |  |  |  |  |  |  |  |  |  |  |
| 485.7 | 2 | igd | 3.00E-04 | T | \|\| id:ebi-a-GCST90001695 | ebi-a-GCST90001695 | TRUE | C | 200181613 | 3.29E-11 | reported | 3408 | 72.98 | rs72929459 |  |  |  |  |  |  |  |  |  |  |  |  |  |  |  |
| 490.7 | 2 | igd | 3.00E-04 | A | \|\| id:ebi-a-GCST90001695 | ebi-a-GCST90001695 | TRUE | G | 9944052 | 7.72E-10 | reported | 3408 | 79.55 | rs190272290 |  |  |  |  |  |  |  |  |  |  |  |  |  |  |  |
| 566.5 | 2 | igd | 3.00E-04 | C | \|\| id:ebi-a-GCST90001695 | ebi-a-GCST90001695 | TRUE | T | 8930530 | 2.23E-13 | reported | 3408 | 76.93 | rs114135956 |  |  |  |  |  |  |  |  |  |  |  |  |  |  |  |
| 444.1 | 2 | igd | 3.00E-04 | A | \|\| id:ebi-a-GCST90001695 | ebi-a-GCST90001695 | TRUE | G | 238422350 | 7.31E-09 | reported | 3408 | 76.59 | rs112839187 |  |  |  |  |  |  |  |  |  |  |  |  |  |  |  |
| 429 | 2 | igd | 3.00E-04 | T | \|\| id:ebi-a-GCST90001695 | ebi-a-GCST90001695 | TRUE | C | 145836600 | 1.06E-08 | reported | 3408 | 74.81 | rs144711723 |  |  |  |  |  |  |  |  |  |  |  |  |  |  |  |
| 484.1 | 2 | igd | 3.00E-04 | A | \|\| id:ebi-a-GCST90001695 | ebi-a-GCST90001695 | TRUE | G | 199934892 | 3.89E-11 | reported | 3408 | 73.02 | rs115945729 |  |  |  |  |  |  |  |  |  |  |  |  |  |  |  |
| 522.5 | 2 | igd | 3.00E-04 | G | \|\| id:ebi-a-GCST90001695 | ebi-a-GCST90001695 | TRUE | A | 106438361 | 3.54E-13 | reported | 3408 | 71.56 | rs190665193 |  |  |  |  |  |  |  |  |  |  |  |  |  |  |  |
| 473.4 | 3 | igd | 3.00E-04 | A | \|\| id:ebi-a-GCST90001695 | ebi-a-GCST90001695 | TRUE | C | 18550864 | 6.10E-09 | reported | 3408 | 81.22 | rs149994650 |  |  |  |  |  |  |  |  |  |  |  |  |  |  |  |
| 1005 | 4 | igd | 3.00E-04 | C | \|\| id:ebi-a-GCST90001695 | ebi-a-GCST90001695 | TRUE | A | 116470715 | 1.39E-14 | reported | 3408 | 130 | rs183382760 |  |  |  |  |  |  |  |  |  |  |  |  |  |  |  |
| 436 | 6 | igd | 3.00E-04 | A | \|\| id:ebi-a-GCST90001695 | ebi-a-GCST90001695 | TRUE | C | 66316172 | 6.72E-09 | reported | 3408 | 75.02 | rs114801643 |  |  |  |  |  |  |  |  |  |  |  |  |  |  |  |
| 419.1 | 6 | igd | 3.00E-04 | T | \|\| id:ebi-a-GCST90001695 | ebi-a-GCST90001695 | TRUE | A | 24233134 | 1.60E-08 | reported | 3408 | 73.98 | rs74651043 |  |  |  |  |  |  |  |  |  |  |  |  |  |  |  |
| 446.8 | 6 | igd | 3.00E-04 | C | \|\| id:ebi-a-GCST90001695 | ebi-a-GCST90001695 | TRUE | G | 17677103 | 2.34E-08 | reported | 3408 | 79.82 | rs78425522 |  |  |  |  |  |  |  |  |  |  |  |  |  |  |  |
| 404.2 | 6 | igd | 3.00E-04 | A | \|\| id:ebi-a-GCST90001695 | ebi-a-GCST90001695 | TRUE | C | 79460848 | 2.09E-08 | reported | 3408 | 71.96 | rs58860560 |  |  |  |  |  |  |  |  |  |  |  |  |  |  |  |
| 416.5 | 6 | igd | 3.00E-04 | G | \|\| id:ebi-a-GCST90001695 | ebi-a-GCST90001695 | TRUE | A | 66445479 | 1.38E-08 | reported | 3408 | 73.2 | rs150883323 |  |  |  |  |  |  |  |  |  |  |  |  |  |  |  |
| 450.6 | 6 | igd | 3.00E-04 | A | \|\| id:ebi-a-GCST90001695 | ebi-a-GCST90001695 | TRUE | G | 18617129 | 7.79E-09 | reported | 3408 | 77.86 | rs115379408 |  |  |  |  |  |  |  |  |  |  |  |  |  |  |  |
| 595.1 | 7 | igd | 3.00E-04 | T | \|\| id:ebi-a-GCST90001695 | ebi-a-GCST90001695 | TRUE | C | 1084031 | 1.22E-16 | reported | 3408 | 71.5 | rs142522853 |  |  |  |  |  |  |  |  |  |  |  |  |  |  |  |
| 726.8 | 7 | igd | 3.00E-04 | T | \|\| id:ebi-a-GCST90001695 | ebi-a-GCST90001695 | TRUE | A | 194075 | 1.26E-18 | reported | 3408 | 82.02 | rs138358136 |  |  |  |  |  |  |  |  |  |  |  |  |  |  |  |
| 591.6 | 8 | igd | 3.00E-04 | T | \|\| id:ebi-a-GCST90001695 | ebi-a-GCST90001695 | TRUE | C | 7162695 | 3.56E-12 | reported | 3408 | 84.77 | rs62488802 |  |  |  |  |  |  |  |  |  |  |  |  |  |  |  |
| 420 | 9 | igd | 3.00E-04 | A | \|\| id:ebi-a-GCST90001695 | ebi-a-GCST90001695 | TRUE | G | 136915695 | 3.87E-09 | reported | 3408 | 71.13 | rs34609592 |  |  |  |  |  |  |  |  |  |  |  |  |  |  |  |
| 389.2 | 10 | igd | 3.00E-04 | A | \|\| id:ebi-a-GCST90001695 | ebi-a-GCST90001695 | TRUE | C | 28109090 | 4.14E-08 | reported | 3408 | 70.79 | rs79407975 |  |  |  |  |  |  |  |  |  |  |  |  |  |  |  |
| 416.6 | 10 | igd | 3.00E-04 | A | \|\| id:ebi-a-GCST90001695 | ebi-a-GCST90001695 | TRUE | G | 21304171 | 2.58E-08 | reported | 3408 | 74.65 | rs45507192 |  |  |  |  |  |  |  |  |  |  |  |  |  |  |  |
| 513.2 | 11 | igd | 3.00E-04 | T | \|\| id:ebi-a-GCST90001695 | ebi-a-GCST90001695 | TRUE | C | 64998378 | 5.95E-09 | reported | 3408 | 87.98 | rs138668741 |  |  |  |  |  |  |  |  |  |  |  |  |  |  |  |
| 514.7 | 15 | igd | 3.00E-04 | C | \|\| id:ebi-a-GCST90001695 | ebi-a-GCST90001695 | TRUE | T | 96888705 | 3.97E-10 | reported | 3408 | 82.05 | rs79888820 |  |  |  |  |  |  |  |  |  |  |  |  |  |  |  |
| 402.2 | 15 | igd | 3.00E-04 | A | \|\| id:ebi-a-GCST90001695 | ebi-a-GCST90001695 | TRUE | G | 78554710 | 2.39E-08 | reported | 3408 | 71.9 | rs186595909 |  |  |  |  |  |  |  |  |  |  |  |  |  |  |  |
| 732.2 | 16 | igd | 3.00E-04 | A | \|\| id:ebi-a-GCST90001695 | ebi-a-GCST90001695 | TRUE | G | 69966147 | 1.54E-08 | reported | 3408 | 129.1 | rs189280617 |  |  |  |  |  |  |  |  |  |  |  |  |  |  |  |
| 564.5 | 16 | igd | 3.00E-04 | T | \|\| id:ebi-a-GCST90001695 | ebi-a-GCST90001695 | TRUE | G | 32022251 | 2.18E-09 | reported | 3408 | 94.08 | rs147802671 |  |  |  |  |  |  |  |  |  |  |  |  |  |  |  |
| 654.7 | 16 | igd | 3.00E-04 | G | \|\| id:ebi-a-GCST90001695 | ebi-a-GCST90001695 | TRUE | T | 70343845 | 1.06E-08 | reported | 3408 | 114.1 | rs188394601 |  |  |  |  |  |  |  |  |  |  |  |  |  |  |  |
| 592.4 | 16 | igd | 3.00E-04 | A | \|\| id:ebi-a-GCST90001695 | ebi-a-GCST90001695 | TRUE | G | 70591907 | 1.06E-08 | reported | 3408 | 103.3 | rs149406145 |  |  |  |  |  |  |  |  |  |  |  |  |  |  |  |
| 538.9 | 18 | igd | 3.00E-04 | C | \|\| id:ebi-a-GCST90001695 | ebi-a-GCST90001695 | TRUE | T | 39828718 | 4.03E-13 | reported | 3408 | 73.99 | rs75420474 |  |  |  |  |  |  |  |  |  |  |  |  |  |  |  |
| 462.3 | 21 | igd | 3.00E-04 | C | \|\| id:ebi-a-GCST90001695 | ebi-a-GCST90001695 | TRUE | A | 26316207 | 2.71E-08 | reported | 3408 | 82.97 | rs117514047 |  |  |  |  |  |  |  |  |  |  |  |  |  |  |  |
| 53.93 | 2 | igd | 3.00E-04 | C | \|\| id:ebi-a-GCST90001696 | ebi-a-GCST90001696 | TRUE | T | 8930530 | 6.98E-10 | reported | 3425 | 8.721 | rs114135956 |  |  |  |  |  |  |  |  |  |  |  |  |  |  |  |
| 32 | 2 | igd | 3.00E-04 | C | \|\| id:ebi-a-GCST90001697 | ebi-a-GCST90001697 | TRUE | T | 8930530 | 2.61E-19 | reported | 3425 | 3.541 | rs114135956 |  |  |  |  |  |  |  |  |  |  |  |  |  |  |  |
| 24.63 | 2 | igd | 3.00E-04 | A | \|\| id:ebi-a-GCST90001697 | ebi-a-GCST90001697 | TRUE | G | 9944052 | 2.34E-11 | reported | 3425 | 3.673 | rs190272290 |  |  |  |  |  |  |  |  |  |  |  |  |  |  |  |
| 22.84 | 3 | igd | 3.00E-04 | A | \|\| id:ebi-a-GCST90001697 | ebi-a-GCST90001697 | TRUE | C | 18550864 | 1.47E-09 | reported | 3425 | 3.766 | rs149994650 |  |  |  |  |  |  |  |  |  |  |  |  |  |  |  |
| 18.92 | 7 | igd | 3.00E-04 | A | \|\| id:ebi-a-GCST90001697 | ebi-a-GCST90001697 | TRUE | G | 31473901 | 1.37E-08 | reported | 3425 | 3.324 | rs76938777 |  |  |  |  |  |  |  |  |  |  |  |  |  |  |  |
| 29.98 | 8 | igd | 3.00E-04 | T | \|\| id:ebi-a-GCST90001697 | ebi-a-GCST90001697 | TRUE | C | 7162695 | 2.31E-14 | reported | 3425 | 3.911 | rs62488802 |  |  |  |  |  |  |  |  |  |  |  |  |  |  |  |
| 23.77 | 11 | igd | 3.00E-04 | A | \|\| id:ebi-a-GCST90001697 | ebi-a-GCST90001697 | TRUE | G | 82732689 | 5.93E-11 | reported | 3425 | 3.62 | rs11233425 |  |  |  |  |  |  |  |  |  |  |  |  |  |  |  |
| 20.46 | 12 | igd | 3.00E-04 | C | \|\| id:ebi-a-GCST90001697 | ebi-a-GCST90001697 | TRUE | A | 97040021 | 9.41E-10 | reported | 3425 | 3.333 | rs538264691 |  |  |  |  |  |  |  |  |  |  |  |  |  |  |  |
| 18.44 | 14 | igd | 3.00E-04 | A | \|\| id:ebi-a-GCST90001697 | ebi-a-GCST90001697 | TRUE | G | 45526169 | 2.28E-08 | reported | 3425 | 3.292 | rs117551202 |  |  |  |  |  |  |  |  |  |  |  |  |  |  |  |
| 20.94 | 15 | igd | 3.00E-04 | G | \|\| id:ebi-a-GCST90001697 | ebi-a-GCST90001697 | TRUE | A | 84246825 | 2.66E-10 | reported | 3425 | 3.304 | rs117552343 |  |  |  |  |  |  |  |  |  |  |  |  |  |  |  |
| 20.97 | 15 | igd | 3.00E-04 | C | \|\| id:ebi-a-GCST90001697 | ebi-a-GCST90001697 | TRUE | T | 86484743 | 2.50E-10 | reported | 3425 | 3.305 | rs74707627 |  |  |  |  |  |  |  |  |  |  |  |  |  |  |  |
| 19.32 | 15 | igd | 3.00E-04 | A | \|\| id:ebi-a-GCST90001697 | ebi-a-GCST90001697 | TRUE | G | 78554710 | 7.72E-09 | reported | 3425 | 3.338 | rs186595909 |  |  |  |  |  |  |  |  |  |  |  |  |  |  |  |
| 18.3 | 16 | igd | 3.00E-04 | G | \|\| id:ebi-a-GCST90001697 | ebi-a-GCST90001697 | TRUE | A | 19193363 | 1.04E-08 | reported | 3425 | 3.188 | rs34375694 |  |  |  |  |  |  |  |  |  |  |  |  |  |  |  |
| 28.44 | 18 | igd | 3.00E-04 | C | \|\| id:ebi-a-GCST90001697 | ebi-a-GCST90001697 | TRUE | T | 39828718 | 1.57E-16 | reported | 3425 | 3.429 | rs75420474 |  |  |  |  |  |  |  |  |  |  |  |  |  |  |  |
| 895.1 | 2 | igd | 3.00E-04 | T | \|\| id:ebi-a-GCST90001698 | ebi-a-GCST90001698 | TRUE | C | 200181613 | 6.91E-20 | reported | 3408 | 97.45 | rs72929459 |  |  |  |  |  |  |  |  |  |  |  |  |  |  |  |
| 1581 | 2 | igd | 3.00E-04 | G | \|\| id:ebi-a-GCST90001698 | ebi-a-GCST90001698 | TRUE | A | 106438361 | 1.69E-61 | reported | 3408 | 93.65 | rs190665193 |  |  |  |  |  |  |  |  |  |  |  |  |  |  |  |
| 1003 | 2 | igd | 3.00E-04 | G | \|\| id:ebi-a-GCST90001698 | ebi-a-GCST90001698 | TRUE | A | 159316835 | 3.69E-24 | reported | 3408 | 98.17 | rs72931475 |  |  |  |  |  |  |  |  |  |  |  |  |  |  |  |
| 896.2 | 2 | igd | 3.00E-04 | A | \|\| id:ebi-a-GCST90001698 | ebi-a-GCST90001698 | TRUE | G | 199934892 | 6.49E-20 | reported | 3408 | 97.49 | rs115945729 |  |  |  |  |  |  |  |  |  |  |  |  |  |  |  |
| 1034 | 3 | igd | 3.00E-04 | G | \|\| id:ebi-a-GCST90001698 | ebi-a-GCST90001698 | TRUE | A | 126486072 | 4.15E-29 | reported | 3408 | 91.46 | rs115445664 |  |  |  |  |  |  |  |  |  |  |  |  |  |  |  |
| 961.3 | 3 | igd | 3.00E-04 | A | \|\| id:ebi-a-GCST90001698 | ebi-a-GCST90001698 | TRUE | C | 141886501 | 1.80E-24 | reported | 3408 | 93.43 | rs79418578 |  |  |  |  |  |  |  |  |  |  |  |  |  |  |  |
| 903.1 | 3 | igd | 3.00E-04 | G | \|\| id:ebi-a-GCST90001698 | ebi-a-GCST90001698 | TRUE | C | 141674361 | 8.40E-23 | reported | 3408 | 91.22 | rs143346058 |  |  |  |  |  |  |  |  |  |  |  |  |  |  |  |
| 623.6 | 3 | igd | 3.00E-04 | A | \|\| id:ebi-a-GCST90001698 | ebi-a-GCST90001698 | TRUE | G | 105859785 | 1.69E-12 | reported | 3408 | 88.02 | rs115135290 |  |  |  |  |  |  |  |  |  |  |  |  |  |  |  |
| 915.9 | 4 | igd | 3.00E-04 | A | \|\| id:ebi-a-GCST90001698 | ebi-a-GCST90001698 | TRUE | C | 16986930 | 4.51E-22 | reported | 3408 | 94.17 | rs114421150 |  |  |  |  |  |  |  |  |  |  |  |  |  |  |  |
| 771.6 | 5 | igd | 3.00E-04 | T | \|\| id:ebi-a-GCST90001698 | ebi-a-GCST90001698 | TRUE | C | 168914162 | 4.87E-17 | reported | 3408 | 91.49 | rs115755474 |  |  |  |  |  |  |  |  |  |  |  |  |  |  |  |
| 1285 | 6 | igd | 3.00E-04 | A | \|\| id:ebi-a-GCST90001698 | ebi-a-GCST90001698 | TRUE | G | 1730982 | 5.98E-46 | reported | 3408 | 88.96 | rs138327786 |  |  |  |  |  |  |  |  |  |  |  |  |  |  |  |
| 1445 | 6 | igd | 3.00E-04 | C | \|\| id:ebi-a-GCST90001698 | ebi-a-GCST90001698 | TRUE | A | 10017676 | 3.23E-52 | reported | 3408 | 93.44 | rs149208831 |  |  |  |  |  |  |  |  |  |  |  |  |  |  |  |
| 1994 | 6 | igd | 3.00E-04 | G | \|\| id:ebi-a-GCST90001698 | ebi-a-GCST90001698 | TRUE | A | 11491250 | 1.48E-71 | reported | 3408 | 108.9 | rs145604620 |  |  |  |  |  |  |  |  |  |  |  |  |  |  |  |
| 857.2 | 6 | igd | 3.00E-04 | C | \|\| id:ebi-a-GCST90001698 | ebi-a-GCST90001698 | TRUE | T | 28389143 | 1.53E-15 | reported | 3408 | 107 | rs79518488 |  |  |  |  |  |  |  |  |  |  |  |  |  |  |  |
| 863.3 | 6 | igd | 3.00E-04 | G | \|\| id:ebi-a-GCST90001698 | ebi-a-GCST90001698 | TRUE | A | 28628536 | 1.55E-15 | reported | 3408 | 107.8 | rs138045952 |  |  |  |  |  |  |  |  |  |  |  |  |  |  |  |
| 1008 | 9 | igd | 3.00E-04 | A | \|\| id:ebi-a-GCST90001698 | ebi-a-GCST90001698 | TRUE | G | 136915695 | 4.73E-27 | reported | 3408 | 92.78 | rs34609592 |  |  |  |  |  |  |  |  |  |  |  |  |  |  |  |
| 851.2 | 12 | igd | 3.00E-04 | T | \|\| id:ebi-a-GCST90001698 | ebi-a-GCST90001698 | TRUE | C | 48190275 | 6.37E-20 | reported | 3408 | 92.57 | rs147107195 |  |  |  |  |  |  |  |  |  |  |  |  |  |  |  |
| 829.8 | 14 | igd | 3.00E-04 | C | \|\| id:ebi-a-GCST90001698 | ebi-a-GCST90001698 | TRUE | T | 100822892 | 2.04E-15 | reported | 3408 | 104 | rs150889386 |  |  |  |  |  |  |  |  |  |  |  |  |  |  |  |
| 730 | 14 | igd | 3.00E-04 | G | \|\| id:ebi-a-GCST90001698 | ebi-a-GCST90001698 | TRUE | T | 34065832 | 4.65E-14 | reported | 3408 | 96.39 | rs144275943 |  |  |  |  |  |  |  |  |  |  |  |  |  |  |  |
| 812 | 14 | igd | 3.00E-04 | A | \|\| id:ebi-a-GCST90001698 | ebi-a-GCST90001698 | TRUE | G | 101208855 | 4.34E-15 | reported | 3408 | 103 | rs192994248 |  |  |  |  |  |  |  |  |  |  |  |  |  |  |  |
| 842.4 | 16 | igd | 3.00E-04 | T | \|\| id:ebi-a-GCST90001698 | ebi-a-GCST90001698 | TRUE | C | 51095561 | 1.34E-20 | reported | 3408 | 89.95 | rs138937927 |  |  |  |  |  |  |  |  |  |  |  |  |  |  |  |
| 508.4 | 17 | igd | 3.00E-04 | A | \|\| id:ebi-a-GCST90001698 | ebi-a-GCST90001698 | TRUE | G | 49002799 | 2.39E-08 | reported | 3408 | 90.88 | rs150885241 |  |  |  |  |  |  |  |  |  |  |  |  |  |  |  |
| 31.04 | 2 | igd | 3.00E-04 | G | \|\| id:ebi-a-GCST90001700 | ebi-a-GCST90001700 | TRUE | A | 159316835 | 7.30E-13 | reported | 3425 | 4.31 | rs72931475 |  |  |  |  |  |  |  |  |  |  |  |  |  |  |  |
| 29.23 | 2 | igd | 3.00E-04 | C | \|\| id:ebi-a-GCST90001700 | ebi-a-GCST90001700 | TRUE | G | 83631928 | 5.97E-11 | reported | 3425 | 4.452 | rs10176830 |  |  |  |  |  |  |  |  |  |  |  |  |  |  |  |
| 35.37 | 2 | igd | 3.00E-04 | G | \|\| id:ebi-a-GCST90001700 | ebi-a-GCST90001700 | TRUE | A | 106438361 | 4.44E-18 | reported | 3425 | 4.058 | rs190665193 |  |  |  |  |  |  |  |  |  |  |  |  |  |  |  |
| 26.82 | 3 | igd | 3.00E-04 | A | \|\| id:ebi-a-GCST90001700 | ebi-a-GCST90001700 | TRUE | G | 74016318 | 4.81E-10 | reported | 3425 | 4.296 | rs111747460 |  |  |  |  |  |  |  |  |  |  |  |  |  |  |  |
| 25 | 4 | igd | 3.00E-04 | A | \|\| id:ebi-a-GCST90001700 | ebi-a-GCST90001700 | TRUE | C | 16986930 | 1.00E-09 | reported | 3425 | 4.08 | rs114421150 |  |  |  |  |  |  |  |  |  |  |  |  |  |  |  |
| 34.45 | 6 | igd | 3.00E-04 | G | \|\| id:ebi-a-GCST90001700 | ebi-a-GCST90001700 | TRUE | A | 11491250 | 1.22E-12 | reported | 3425 | 4.832 | rs145604620 |  |  |  |  |  |  |  |  |  |  |  |  |  |  |  |
| 24.73 | 6 | igd | 3.00E-04 | G | \|\| id:ebi-a-GCST90001700 | ebi-a-GCST90001700 | TRUE | A | 28628536 | 4.38E-08 | reported | 3425 | 4.507 | rs138045952 |  |  |  |  |  |  |  |  |  |  |  |  |  |  |  |
| 24.55 | 6 | igd | 3.00E-04 | C | \|\| id:ebi-a-GCST90001700 | ebi-a-GCST90001700 | TRUE | T | 28389143 | 4.37E-08 | reported | 3425 | 4.474 | rs79518488 |  |  |  |  |  |  |  |  |  |  |  |  |  |  |  |
| 25.69 | 6 | igd | 3.00E-04 | C | \|\| id:ebi-a-GCST90001700 | ebi-a-GCST90001700 | TRUE | A | 10017676 | 3.97E-10 | reported | 3425 | 4.095 | rs149208831 |  |  |  |  |  |  |  |  |  |  |  |  |  |  |  |
| 21.5 | 6 | igd | 3.00E-04 | A | \|\| id:ebi-a-GCST90001700 | ebi-a-GCST90001700 | TRUE | G | 1730982 | 3.38E-08 | reported | 3425 | 3.886 | rs138327786 |  |  |  |  |  |  |  |  |  |  |  |  |  |  |  |
| 23.9 | 12 | igd | 3.00E-04 | T | \|\| id:ebi-a-GCST90001700 | ebi-a-GCST90001700 | TRUE | C | 48190275 | 3.92E-09 | reported | 3425 | 4.049 | rs147107195 |  |  |  |  |  |  |  |  |  |  |  |  |  |  |  |
| 42.31 | 13 | igd | 3.00E-04 | A | \|\| id:ebi-a-GCST90001700 | ebi-a-GCST90001700 | TRUE | G | 84232175 | 1.26E-17 | reported | 3425 | 4.924 | rs189511968 |  |  |  |  |  |  |  |  |  |  |  |  |  |  |  |
| 23.94 | 16 | igd | 3.00E-04 | T | \|\| id:ebi-a-GCST90001700 | ebi-a-GCST90001700 | TRUE | C | 51095561 | 9.81E-10 | reported | 3425 | 3.906 | rs138937927 |  |  |  |  |  |  |  |  |  |  |  |  |  |  |  |
| 31.1 | 17 | igd | 3.00E-04 | A | \|\| id:ebi-a-GCST90001700 | ebi-a-GCST90001700 | TRUE | G | 49002799 | 3.08E-15 | reported | 3425 | 3.924 | rs150885241 |  |  |  |  |  |  |  |  |  |  |  |  |  |  |  |
| 6.643 | 1 | igd | 1.00E-04 | G | \|\| id:ebi-a-GCST90001688 | ebi-a-GCST90001688 | TRUE | A | 216352525 | 3.22E-08 | reported | 3440 | 1.199 | rs12755474 |  |  |  |  |  |  |  |  |  |  |  |  |  |  |  |
| 3.783 | 1 | igd | 1.00E-04 | G | \|\| id:ebi-a-GCST90001688 | ebi-a-GCST90001688 | TRUE | A | 62672889 | 1.62E-08 | reported | 3440 | 0.6683 | rs142530179 |  |  |  |  |  |  |  |  |  |  |  |  |  |  |  |
| 4.52 | 1 | igd | 1.00E-04 | G | \|\| id:ebi-a-GCST90001688 | ebi-a-GCST90001688 | TRUE | A | 232754676 | 1.13E-08 | reported | 3440 | 0.7897 | rs149023182 |  |  |  |  |  |  |  |  |  |  |  |  |  |  |  |
| 6.493 | 3 | igd | 1.00E-04 | G | \|\| id:ebi-a-GCST90001688 | ebi-a-GCST90001688 | TRUE | A | 170169608 | 8.69E-09 | reported | 3440 | 1.126 | rs146985291 |  |  |  |  |  |  |  |  |  |  |  |  |  |  |  |
| 7.448 | 3 | igd | 1.00E-04 | C | \|\| id:ebi-a-GCST90001688 | ebi-a-GCST90001688 | TRUE | T | 170436082 | 1.84E-08 | reported | 3440 | 1.321 | rs142946974 |  |  |  |  |  |  |  |  |  |  |  |  |  |  |  |
| 3.867 | 4 | igd | 1.00E-04 | C | \|\| id:ebi-a-GCST90001688 | ebi-a-GCST90001688 | TRUE | A | 31397782 | 1.59E-08 | reported | 3440 | 0.6826 | rs76604940 |  |  |  |  |  |  |  |  |  |  |  |  |  |  |  |
| 4.272 | 4 | igd | 1.00E-04 | G | \|\| id:ebi-a-GCST90001688 | ebi-a-GCST90001688 | TRUE | T | 67836131 | 1.60E-08 | reported | 3440 | 0.7543 | rs115218435 |  |  |  |  |  |  |  |  |  |  |  |  |  |  |  |
| 3.974 | 5 | igd | 1.00E-04 | C | \|\| id:ebi-a-GCST90001688 | ebi-a-GCST90001688 | TRUE | T | 173002007 | 4.54E-08 | reported | 3440 | 0.7251 | rs137858581 |  |  |  |  |  |  |  |  |  |  |  |  |  |  |  |
| 3.897 | 6 | igd | 1.00E-04 | A | \|\| id:ebi-a-GCST90001688 | ebi-a-GCST90001688 | TRUE | C | 21472682 | 1.87E-08 | reported | 3440 | 0.6914 | rs72840806 |  |  |  |  |  |  |  |  |  |  |  |  |  |  |  |
| 4.025 | 7 | igd | 1.00E-04 | C | \|\| id:ebi-a-GCST90001688 | ebi-a-GCST90001688 | TRUE | T | 92791863 | 3.59E-10 | reported | 3440 | 0.6399 | rs184082746 |  |  |  |  |  |  |  |  |  |  |  |  |  |  |  |
| 4.848 | 7 | igd | 1.00E-04 | G | \|\| id:ebi-a-GCST90001688 | ebi-a-GCST90001688 | TRUE | A | 124954943 | 2.34E-09 | reported | 3440 | 0.8097 | rs182236332 |  |  |  |  |  |  |  |  |  |  |  |  |  |  |  |
| 3.787 | 7 | igd | 1.00E-04 | G | \|\| id:ebi-a-GCST90001688 | ebi-a-GCST90001688 | TRUE | A | 111704231 | 2.44E-08 | reported | 3440 | 0.6773 | rs112571466 |  |  |  |  |  |  |  |  |  |  |  |  |  |  |  |
| 3.717 | 8 | igd | 1.00E-04 | A | \|\| id:ebi-a-GCST90001688 | ebi-a-GCST90001688 | TRUE | C | 140342260 | 3.11E-08 | reported | 3440 | 0.67 | rs143498795 |  |  |  |  |  |  |  |  |  |  |  |  |  |  |  |
| 3.794 | 9 | igd | 1.00E-04 | C | \|\| id:ebi-a-GCST90001688 | ebi-a-GCST90001688 | TRUE | G | 12800264 | 1.44E-08 | reported | 3440 | 0.6676 | rs142967914 |  |  |  |  |  |  |  |  |  |  |  |  |  |  |  |
| 5.704 | 9 | igd | 1.00E-04 | T | \|\| id:ebi-a-GCST90001688 | ebi-a-GCST90001688 | TRUE | A | 33552507 | 1.35E-08 | reported | 3440 | 1.002 | rs192475369 |  |  |  |  |  |  |  |  |  |  |  |  |  |  |  |
| 4.877 | 9 | igd | 1.00E-04 | G | \|\| id:ebi-a-GCST90001688 | ebi-a-GCST90001688 | TRUE | A | 122676137 | 1.53E-08 | reported | 3440 | 0.8599 | rs76325389 |  |  |  |  |  |  |  |  |  |  |  |  |  |  |  |
| 4.765 | 9 | igd | 1.00E-04 | C | \|\| id:ebi-a-GCST90001688 | ebi-a-GCST90001688 | TRUE | T | 105346840 | 1.92E-08 | reported | 3440 | 0.8459 | rs113447229 |  |  |  |  |  |  |  |  |  |  |  |  |  |  |  |
| 4.68 | 10 | igd | 1.00E-04 | G | \|\| id:ebi-a-GCST90001688 | ebi-a-GCST90001688 | TRUE | T | 91332621 | 8.65E-09 | reported | 3440 | 0.8111 | rs80194890 |  |  |  |  |  |  |  |  |  |  |  |  |  |  |  |
| 4.11 | 10 | igd | 1.00E-04 | A | \|\| id:ebi-a-GCST90001688 | ebi-a-GCST90001688 | TRUE | C | 68336237 | 1.60E-08 | reported | 3440 | 0.7256 | rs189801254 |  |  |  |  |  |  |  |  |  |  |  |  |  |  |  |
| 3.783 | 12 | igd | 1.00E-04 | A | \|\| id:ebi-a-GCST90001688 | ebi-a-GCST90001688 | TRUE | G | 49315200 | 1.63E-08 | reported | 3440 | 0.6683 | rs117927481 |  |  |  |  |  |  |  |  |  |  |  |  |  |  |  |
| 3.436 | 12 | igd | 1.00E-04 | T | \|\| id:ebi-a-GCST90001688 | ebi-a-GCST90001688 | TRUE | C | 55672838 | 1.25E-08 | reported | 3440 | 0.6021 | rs112258643 |  |  |  |  |  |  |  |  |  |  |  |  |  |  |  |
| 3.97 | 12 | igd | 1.00E-04 | G | \|\| id:ebi-a-GCST90001688 | ebi-a-GCST90001688 | TRUE | C | 55885740 | 2.32E-08 | reported | 3440 | 0.709 | rs111739344 |  |  |  |  |  |  |  |  |  |  |  |  |  |  |  |
| 3.76 | 13 | igd | 1.00E-04 | T | \|\| id:ebi-a-GCST90001688 | ebi-a-GCST90001688 | TRUE | A | 66723738 | 1.99E-08 | reported | 3440 | 0.6683 | rs74981620 |  |  |  |  |  |  |  |  |  |  |  |  |  |  |  |
| 4.763 | 13 | igd | 1.00E-04 | C | \|\| id:ebi-a-GCST90001688 | ebi-a-GCST90001688 | TRUE | G | 67157607 | 1.65E-08 | reported | 3440 | 0.8417 | rs9592479 |  |  |  |  |  |  |  |  |  |  |  |  |  |  |  |
| 3.833 | 14 | igd | 1.00E-04 | G | \|\| id:ebi-a-GCST90001688 | ebi-a-GCST90001688 | TRUE | T | 28349764 | 1.63E-08 | reported | 3440 | 0.6771 | rs58471414 |  |  |  |  |  |  |  |  |  |  |  |  |  |  |  |
| 3.894 | 14 | igd | 1.00E-04 | A | \|\| id:ebi-a-GCST90001688 | ebi-a-GCST90001688 | TRUE | G | 56862154 | 1.94E-08 | reported | 3440 | 0.6914 | rs117494082 |  |  |  |  |  |  |  |  |  |  |  |  |  |  |  |
| 4.463 | 16 | igd | 1.00E-04 | C | \|\| id:ebi-a-GCST90001688 | ebi-a-GCST90001688 | TRUE | A | 79205197 | 1.90E-08 | reported | 3440 | 0.7922 | rs111283043 |  |  |  |  |  |  |  |  |  |  |  |  |  |  |  |
| 3.946 | 16 | igd | 1.00E-04 | G | \|\| id:ebi-a-GCST90001688 | ebi-a-GCST90001688 | TRUE | A | 59010741 | 1.60E-08 | reported | 3440 | 0.6967 | rs144591335 |  |  |  |  |  |  |  |  |  |  |  |  |  |  |  |
| 3.755 | 18 | igd | 1.00E-04 | G | \|\| id:ebi-a-GCST90001688 | ebi-a-GCST90001688 | TRUE | A | 68968361 | 1.72E-08 | reported | 3440 | 0.6644 | rs77935824 |  |  |  |  |  |  |  |  |  |  |  |  |  |  |  |
| 3.814 | 19 | igd | 1.00E-04 | T | \|\| id:ebi-a-GCST90001688 | ebi-a-GCST90001688 | TRUE | C | 51829114 | 8.68E-09 | reported | 3440 | 0.6612 | rs142208889 |  |  |  |  |  |  |  |  |  |  |  |  |  |  |  |
| 4.893 | 20 | igd | 1.00E-04 | T | \|\| id:ebi-a-GCST90001688 | ebi-a-GCST90001688 | TRUE | C | 57132122 | 5.91E-09 | reported | 3440 | 0.8386 | rs145827119 |  |  |  |  |  |  |  |  |  |  |  |  |  |  |  |
| 3.533 | 21 | igd | 1.00E-04 | A | \|\| id:ebi-a-GCST90001688 | ebi-a-GCST90001688 | TRUE | G | 41143748 | 3.12E-08 | reported | 3440 | 0.6368 | rs149926070 |  |  |  |  |  |  |  |  |  |  |  |  |  |  |  |
| 760.9 | 1 | igd | 1.00E-04 | T | \|\| id:ebi-a-GCST90001695 | ebi-a-GCST90001695 | TRUE | C | 24640467 | 4.68E-14 | reported | 3408 | 100.5 | rs140963286 |  |  |  |  |  |  |  |  |  |  |  |  |  |  |  |
| 725.9 | 1 | igd | 1.00E-04 | G | \|\| id:ebi-a-GCST90001695 | ebi-a-GCST90001695 | TRUE | A | 65133062 | 6.76E-13 | reported | 3408 | 100.6 | rs144571871 |  |  |  |  |  |  |  |  |  |  |  |  |  |  |  |
| 644.1 | 1 | igd | 1.00E-04 | C | \|\| id:ebi-a-GCST90001695 | ebi-a-GCST90001695 | TRUE | T | 80897562 | 1.37E-09 | reported | 3408 | 106 | rs61772921 |  |  |  |  |  |  |  |  |  |  |  |  |  |  |  |
| 721.2 | 1 | igd | 1.00E-04 | T | \|\| id:ebi-a-GCST90001695 | ebi-a-GCST90001695 | TRUE | G | 34724192 | 4.50E-15 | reported | 3408 | 91.57 | rs55881385 |  |  |  |  |  |  |  |  |  |  |  |  |  |  |  |
| 791.9 | 2 | igd | 1.00E-04 | T | \|\| id:ebi-a-GCST90001695 | ebi-a-GCST90001695 | TRUE | C | 9441279 | 1.17E-15 | reported | 3408 | 98.43 | rs138799468 |  |  |  |  |  |  |  |  |  |  |  |  |  |  |  |
| 979.6 | 2 | igd | 1.00E-04 | A | \|\| id:ebi-a-GCST90001695 | ebi-a-GCST90001695 | TRUE | C | 191319109 | 2.52E-09 | reported | 3408 | 163.9 | rs114763811 |  |  |  |  |  |  |  |  |  |  |  |  |  |  |  |
| 751.4 | 3 | igd | 1.00E-04 | T | \|\| id:ebi-a-GCST90001695 | ebi-a-GCST90001695 | TRUE | C | 153311399 | 7.82E-14 | reported | 3408 | 100.1 | rs148658406 |  |  |  |  |  |  |  |  |  |  |  |  |  |  |  |
| 550.9 | 3 | igd | 1.00E-04 | G | \|\| id:ebi-a-GCST90001695 | ebi-a-GCST90001695 | TRUE | T | 10035985 | 2.17E-08 | reported | 3408 | 98.17 | rs139772031 |  |  |  |  |  |  |  |  |  |  |  |  |  |  |  |
| 760.5 | 3 | igd | 1.00E-04 | A | \|\| id:ebi-a-GCST90001695 | ebi-a-GCST90001695 | TRUE | G | 152385976 | 5.25E-14 | reported | 3408 | 100.6 | rs16864501 |  |  |  |  |  |  |  |  |  |  |  |  |  |  |  |
| 610.4 | 4 | igd | 1.00E-04 | A | \|\| id:ebi-a-GCST90001695 | ebi-a-GCST90001695 | TRUE | G | 25412773 | 1.11E-08 | reported | 3408 | 106.6 | rs62409210 |  |  |  |  |  |  |  |  |  |  |  |  |  |  |  |
| 591 | 5 | igd | 1.00E-04 | T | \|\| id:ebi-a-GCST90001695 | ebi-a-GCST90001695 | TRUE | C | 140131486 | 1.16E-09 | reported | 3408 | 96.85 | rs114216104 |  |  |  |  |  |  |  |  |  |  |  |  |  |  |  |
| 913.1 | 6 | igd | 1.00E-04 | A | \|\| id:ebi-a-GCST90001695 | ebi-a-GCST90001695 | TRUE | G | 52375282 | 4.71E-20 | reported | 3408 | 98.95 | rs149957527 |  |  |  |  |  |  |  |  |  |  |  |  |  |  |  |
| 887.7 | 7 | igd | 1.00E-04 | C | \|\| id:ebi-a-GCST90001695 | ebi-a-GCST90001695 | TRUE | G | 115835641 | 1.33E-09 | reported | 3408 | 146 | rs140112233 |  |  |  |  |  |  |  |  |  |  |  |  |  |  |  |
| 557.6 | 8 | igd | 1.00E-04 | A | \|\| id:ebi-a-GCST90001695 | ebi-a-GCST90001695 | TRUE | G | 144233069 | 4.91E-08 | reported | 3408 | 102 | rs535005711 |  |  |  |  |  |  |  |  |  |  |  |  |  |  |  |
| 754.3 | 11 | igd | 1.00E-04 | T | \|\| id:ebi-a-GCST90001695 | ebi-a-GCST90001695 | TRUE | A | 91872187 | 4.02E-13 | reported | 3408 | 103.6 | rs141656121 |  |  |  |  |  |  |  |  |  |  |  |  |  |  |  |
| 591.3 | 12 | igd | 1.00E-04 | C | \|\| id:ebi-a-GCST90001695 | ebi-a-GCST90001695 | TRUE | A | 101714936 | 3.23E-09 | reported | 3408 | 99.63 | rs74603821 |  |  |  |  |  |  |  |  |  |  |  |  |  |  |  |
| 720.5 | 15 | igd | 1.00E-04 | T | \|\| id:ebi-a-GCST90001695 | ebi-a-GCST90001695 | TRUE | C | 54616572 | 4.88E-08 | reported | 3408 | 131.8 | rs28669135 |  |  |  |  |  |  |  |  |  |  |  |  |  |  |  |
| 1065 | 15 | igd | 1.00E-04 | T | \|\| id:ebi-a-GCST90001695 | ebi-a-GCST90001695 | TRUE | C | 50365389 | 1.78E-09 | reported | 3408 | 176.6 | rs62021713 |  |  |  |  |  |  |  |  |  |  |  |  |  |  |  |
| 611.9 | 16 | igd | 1.00E-04 | G | \|\| id:ebi-a-GCST90001695 | ebi-a-GCST90001695 | TRUE | C | 86442575 | 1.42E-09 | reported | 3408 | 100.8 | rs12597262 |  |  |  |  |  |  |  |  |  |  |  |  |  |  |  |
| 647.9 | 17 | igd | 1.00E-04 | C | \|\| id:ebi-a-GCST90001695 | ebi-a-GCST90001695 | TRUE | T | 16446446 | 6.57E-11 | reported | 3408 | 98.9 | rs116876186 |  |  |  |  |  |  |  |  |  |  |  |  |  |  |  |
| 613.8 | 18 | igd | 1.00E-04 | A | \|\| id:ebi-a-GCST90001695 | ebi-a-GCST90001695 | TRUE | G | 38375388 | 1.50E-11 | reported | 3408 | 90.65 | rs151145927 |  |  |  |  |  |  |  |  |  |  |  |  |  |  |  |
| 61.28 | 2 | igd | 1.00E-04 | T | \|\| id:ebi-a-GCST90001696 | ebi-a-GCST90001696 | TRUE | C | 9441279 | 4.37E-08 | reported | 3425 | 11.17 | rs138799468 |  |  |  |  |  |  |  |  |  |  |  |  |  |  |  |
| 25.81 | 1 | igd | 1.00E-04 | T | \|\| id:ebi-a-GCST90001697 | ebi-a-GCST90001697 | TRUE | G | 34724192 | 1.66E-09 | reported | 3425 | 4.27 | rs55881385 |  |  |  |  |  |  |  |  |  |  |  |  |  |  |  |
| 31.35 | 1 | igd | 1.00E-04 | C | \|\| id:ebi-a-GCST90001697 | ebi-a-GCST90001697 | TRUE | T | 80897562 | 1.95E-10 | reported | 3425 | 4.91 | rs61772921 |  |  |  |  |  |  |  |  |  |  |  |  |  |  |  |
| 47.98 | 2 | igd | 1.00E-04 | A | \|\| id:ebi-a-GCST90001697 | ebi-a-GCST90001697 | TRUE | C | 191319109 | 2.92E-10 | reported | 3425 | 7.59 | rs114763811 |  |  |  |  |  |  |  |  |  |  |  |  |  |  |  |
| 39.54 | 2 | igd | 1.00E-04 | T | \|\| id:ebi-a-GCST90001697 | ebi-a-GCST90001697 | TRUE | C | 9441279 | 4.13E-18 | reported | 3425 | 4.533 | rs138799468 |  |  |  |  |  |  |  |  |  |  |  |  |  |  |  |
| 29.68 | 4 | igd | 1.00E-04 | A | \|\| id:ebi-a-GCST90001697 | ebi-a-GCST90001697 | TRUE | G | 25412773 | 2.03E-09 | reported | 3425 | 4.936 | rs62409210 |  |  |  |  |  |  |  |  |  |  |  |  |  |  |  |
| 30.16 | 5 | igd | 1.00E-04 | T | \|\| id:ebi-a-GCST90001697 | ebi-a-GCST90001697 | TRUE | C | 140131486 | 2.09E-11 | reported | 3425 | 4.486 | rs114216104 |  |  |  |  |  |  |  |  |  |  |  |  |  |  |  |
| 43.28 | 7 | igd | 1.00E-04 | C | \|\| id:ebi-a-GCST90001697 | ebi-a-GCST90001697 | TRUE | G | 115835641 | 1.74E-10 | reported | 3425 | 6.761 | rs140112233 |  |  |  |  |  |  |  |  |  |  |  |  |  |  |  |
| 34.38 | 12 | igd | 1.00E-04 | G | \|\| id:ebi-a-GCST90001697 | ebi-a-GCST90001697 | TRUE | C | 31264096 | 1.02E-08 | reported | 3425 | 5.988 | rs143926433 |  |  |  |  |  |  |  |  |  |  |  |  |  |  |  |
| 26.26 | 12 | igd | 1.00E-04 | A | \|\| id:ebi-a-GCST90001697 | ebi-a-GCST90001697 | TRUE | G | 28246470 | 2.22E-08 | reported | 3425 | 4.684 | rs111388649 |  |  |  |  |  |  |  |  |  |  |  |  |  |  |  |
| 52.08 | 15 | igd | 1.00E-04 | T | \|\| id:ebi-a-GCST90001697 | ebi-a-GCST90001697 | TRUE | C | 50365389 | 2.17E-10 | reported | 3425 | 8.178 | rs62021713 |  |  |  |  |  |  |  |  |  |  |  |  |  |  |  |
| 29.78 | 16 | igd | 1.00E-04 | G | \|\| id:ebi-a-GCST90001697 | ebi-a-GCST90001697 | TRUE | C | 86442575 | 2.03E-10 | reported | 3425 | 4.668 | rs12597262 |  |  |  |  |  |  |  |  |  |  |  |  |  |  |  |
| 2896 | 1 | igd | 1.00E-04 | A | \|\| id:ebi-a-GCST90001698 | ebi-a-GCST90001698 | TRUE | G | 249084997 | 5.00E-107 | reported | 3408 | 127.1 | rs76444609 |  |  |  |  |  |  |  |  |  |  |  |  |  |  |  |
| 3252 | 1 | igd | 1.00E-04 | A | \|\| id:ebi-a-GCST90001698 | ebi-a-GCST90001698 | TRUE | C | 249213594 | 1.50E-106 | reported | 3408 | 143.1 | rs146605567 |  |  |  |  |  |  |  |  |  |  |  |  |  |  |  |
| 2073 | 1 | igd | 1.00E-04 | T | \|\| id:ebi-a-GCST90001698 | ebi-a-GCST90001698 | TRUE | C | 24640467 | 1.15E-56 | reported | 3408 | 128.3 | rs140963286 |  |  |  |  |  |  |  |  |  |  |  |  |  |  |  |
| 2073 | 3 | igd | 1.00E-04 | A | \|\| id:ebi-a-GCST90001698 | ebi-a-GCST90001698 | TRUE | G | 152385976 | 1.74E-56 | reported | 3408 | 128.5 | rs16864501 |  |  |  |  |  |  |  |  |  |  |  |  |  |  |  |
| 2047 | 3 | igd | 1.00E-04 | T | \|\| id:ebi-a-GCST90001698 | ebi-a-GCST90001698 | TRUE | C | 153311399 | 1.21E-55 | reported | 3408 | 127.9 | rs148658406 |  |  |  |  |  |  |  |  |  |  |  |  |  |  |  |
| 1757 | 4 | igd | 1.00E-04 | T | \|\| id:ebi-a-GCST90001698 | ebi-a-GCST90001698 | TRUE | C | 120605817 | 8.19E-41 | reported | 3408 | 129.6 | rs62321187 |  |  |  |  |  |  |  |  |  |  |  |  |  |  |  |
| 1826 | 8 | igd | 1.00E-04 | A | \|\| id:ebi-a-GCST90001698 | ebi-a-GCST90001698 | TRUE | G | 13716390 | 6.61E-42 | reported | 3408 | 132.8 | rs79851411 |  |  |  |  |  |  |  |  |  |  |  |  |  |  |  |
| 1232 | 10 | igd | 1.00E-04 | T | \|\| id:ebi-a-GCST90001698 | ebi-a-GCST90001698 | TRUE | C | 69536834 | 1.63E-09 | reported | 3408 | 203.7 | rs143395500 |  |  |  |  |  |  |  |  |  |  |  |  |  |  |  |
| 1783 | 11 | igd | 1.00E-04 | T | \|\| id:ebi-a-GCST90001698 | ebi-a-GCST90001698 | TRUE | A | 122918979 | 9.60E-41 | reported | 3408 | 131.7 | rs117284243 |  |  |  |  |  |  |  |  |  |  |  |  |  |  |  |
| 2721 | 11 | igd | 1.00E-04 | A | \|\| id:ebi-a-GCST90001698 | ebi-a-GCST90001698 | TRUE | G | 83433494 | 8.41E-101 | reported | 3408 | 123.4 | rs76552842 |  |  |  |  |  |  |  |  |  |  |  |  |  |  |  |
| 730.1 | 12 | igd | 1.00E-04 | G | \|\| id:ebi-a-GCST90001698 | ebi-a-GCST90001698 | TRUE | A | 83249816 | 2.27E-08 | reported | 3408 | 130.3 | rs77113660 |  |  |  |  |  |  |  |  |  |  |  |  |  |  |  |
| 1200 | 12 | igd | 1.00E-04 | A | \|\| id:ebi-a-GCST90001698 | ebi-a-GCST90001698 | TRUE | G | 19311236 | 1.70E-27 | reported | 3408 | 109.5 | rs188074116 |  |  |  |  |  |  |  |  |  |  |  |  |  |  |  |
| 733.4 | 14 | igd | 1.00E-04 | G | \|\| id:ebi-a-GCST90001698 | ebi-a-GCST90001698 | TRUE | T | 101798387 | 2.09E-08 | reported | 3408 | 130.6 | rs35068665 |  |  |  |  |  |  |  |  |  |  |  |  |  |  |  |
| 910.1 | 17 | igd | 1.00E-04 | A | \|\| id:ebi-a-GCST90001698 | ebi-a-GCST90001698 | TRUE | C | 22048470 | 7.70E-22 | reported | 3408 | 94.12 | rs138281296 |  |  |  |  |  |  |  |  |  |  |  |  |  |  |  |
| 939.1 | 18 | igd | 1.00E-04 | A | \|\| id:ebi-a-GCST90001698 | ebi-a-GCST90001698 | TRUE | G | 26017285 | 6.41E-13 | reported | 3408 | 130.1 | rs75438005 |  |  |  |  |  |  |  |  |  |  |  |  |  |  |  |
| 1057 | 19 | igd | 1.00E-04 | C | \|\| id:ebi-a-GCST90001698 | ebi-a-GCST90001698 | TRUE | T | 33953595 | 4.18E-15 | reported | 3408 | 134.1 | rs78703636 |  |  |  |  |  |  |  |  |  |  |  |  |  |  |  |
| 51.58 | 1 | igd | 1.00E-04 | A | \|\| id:ebi-a-GCST90001700 | ebi-a-GCST90001700 | TRUE | G | 249084997 | 4.82E-19 | reported | 3425 | 5.751 | rs76444609 |  |  |  |  |  |  |  |  |  |  |  |  |  |  |  |
| 57.78 | 1 | igd | 1.00E-04 | A | \|\| id:ebi-a-GCST90001700 | ebi-a-GCST90001700 | TRUE | C | 249213594 | 6.97E-19 | reported | 3425 | 6.472 | rs146605567 |  |  |  |  |  |  |  |  |  |  |  |  |  |  |  |
| 33.4 | 1 | igd | 1.00E-04 | T | \|\| id:ebi-a-GCST90001700 | ebi-a-GCST90001700 | TRUE | C | 24640467 | 6.30E-09 | reported | 3425 | 5.736 | rs140963286 |  |  |  |  |  |  |  |  |  |  |  |  |  |  |  |
| 33.31 | 3 | igd | 1.00E-04 | A | \|\| id:ebi-a-GCST90001700 | ebi-a-GCST90001700 | TRUE | G | 152385976 | 7.31E-09 | reported | 3425 | 5.744 | rs16864501 |  |  |  |  |  |  |  |  |  |  |  |  |  |  |  |
| 32.81 | 3 | igd | 1.00E-04 | T | \|\| id:ebi-a-GCST90001700 | ebi-a-GCST90001700 | TRUE | C | 153311399 | 1.02E-08 | reported | 3425 | 5.716 | rs148658406 |  |  |  |  |  |  |  |  |  |  |  |  |  |  |  |
| 38.22 | 4 | igd | 1.00E-04 | C | \|\| id:ebi-a-GCST90001700 | ebi-a-GCST90001700 | TRUE | T | 55305871 | 9.73E-16 | reported | 3425 | 4.737 | rs75993384 |  |  |  |  |  |  |  |  |  |  |  |  |  |  |  |
| 49 | 4 | igd | 1.00E-04 | T | \|\| id:ebi-a-GCST90001700 | ebi-a-GCST90001700 | TRUE | C | 120605817 | 1.26E-17 | reported | 3425 | 5.703 | rs62321187 |  |  |  |  |  |  |  |  |  |  |  |  |  |  |  |
| 33.95 | 7 | igd | 1.00E-04 | T | \|\| id:ebi-a-GCST90001700 | ebi-a-GCST90001700 | TRUE | G | 39587008 | 5.41E-09 | reported | 3425 | 5.804 | rs117692894 |  |  |  |  |  |  |  |  |  |  |  |  |  |  |  |
| 32.87 | 8 | igd | 1.00E-04 | T | \|\| id:ebi-a-GCST90001700 | ebi-a-GCST90001700 | TRUE | C | 56081028 | 1.53E-08 | reported | 3425 | 5.796 | rs117478414 |  |  |  |  |  |  |  |  |  |  |  |  |  |  |  |
| 36.56 | 8 | igd | 1.00E-04 | A | \|\| id:ebi-a-GCST90001700 | ebi-a-GCST90001700 | TRUE | G | 15699657 | 6.06E-09 | reported | 3425 | 6.272 | rs117867588 |  |  |  |  |  |  |  |  |  |  |  |  |  |  |  |
| 35.15 | 8 | igd | 1.00E-04 | A | \|\| id:ebi-a-GCST90001700 | ebi-a-GCST90001700 | TRUE | G | 13716390 | 1.44E-09 | reported | 3425 | 5.793 | rs79851411 |  |  |  |  |  |  |  |  |  |  |  |  |  |  |  |
| 51.17 | 10 | igd | 1.00E-04 | T | \|\| id:ebi-a-GCST90001700 | ebi-a-GCST90001700 | TRUE | C | 69536834 | 7.92E-09 | reported | 3425 | 8.845 | rs143395500 |  |  |  |  |  |  |  |  |  |  |  |  |  |  |  |
| 46.68 | 10 | igd | 1.00E-04 | G | \|\| id:ebi-a-GCST90001700 | ebi-a-GCST90001700 | TRUE | A | 9668124 | 5.07E-09 | reported | 3425 | 7.967 | rs78069205 |  |  |  |  |  |  |  |  |  |  |  |  |  |  |  |
| 49.65 | 11 | igd | 1.00E-04 | T | \|\| id:ebi-a-GCST90001700 | ebi-a-GCST90001700 | TRUE | A | 122918979 | 1.57E-17 | reported | 3425 | 5.795 | rs117284243 |  |  |  |  |  |  |  |  |  |  |  |  |  |  |  |
| 49.64 | 11 | igd | 1.00E-04 | A | \|\| id:ebi-a-GCST90001700 | ebi-a-GCST90001700 | TRUE | G | 83433494 | 6.60E-19 | reported | 3425 | 5.557 | rs76552842 |  |  |  |  |  |  |  |  |  |  |  |  |  |  |  |
| 52.91 | 12 | igd | 1.00E-04 | G | \|\| id:ebi-a-GCST90001700 | ebi-a-GCST90001700 | TRUE | A | 83249816 | 1.16E-20 | reported | 3425 | 5.64 | rs77113660 |  |  |  |  |  |  |  |  |  |  |  |  |  |  |  |
| 46.69 | 13 | igd | 1.00E-04 | T | \|\| id:ebi-a-GCST90001700 | ebi-a-GCST90001700 | TRUE | A | 84005540 | 3.69E-18 | reported | 3425 | 5.344 | rs77214871 |  |  |  |  |  |  |  |  |  |  |  |  |  |  |  |
| 53.41 | 14 | igd | 1.00E-04 | G | \|\| id:ebi-a-GCST90001700 | ebi-a-GCST90001700 | TRUE | T | 101798387 | 5.87E-21 | reported | 3425 | 5.65 | rs35068665 |  |  |  |  |  |  |  |  |  |  |  |  |  |  |  |
| 33.05 | 15 | igd | 1.00E-04 | C | \|\| id:ebi-a-GCST90001700 | ebi-a-GCST90001700 | TRUE | T | 69811049 | 5.19E-09 | reported | 3425 | 5.644 | rs72758450 |  |  |  |  |  |  |  |  |  |  |  |  |  |  |  |
| 44.6 | 15 | igd | 1.00E-04 | G | \|\| id:ebi-a-GCST90001700 | ebi-a-GCST90001700 | TRUE | A | 59013643 | 7.70E-09 | reported | 3425 | 7.703 | rs80331554 |  |  |  |  |  |  |  |  |  |  |  |  |  |  |  |
| 42.63 | 15 | igd | 1.00E-04 | T | \|\| id:ebi-a-GCST90001700 | ebi-a-GCST90001700 | TRUE | G | 81422902 | 9.65E-09 | reported | 3425 | 7.413 | rs147093663 |  |  |  |  |  |  |  |  |  |  |  |  |  |  |  |
| 34.38 | 16 | igd | 1.00E-04 | T | \|\| id:ebi-a-GCST90001700 | ebi-a-GCST90001700 | TRUE | C | 78662086 | 4.56E-09 | reported | 3425 | 5.85 | rs77467380 |  |  |  |  |  |  |  |  |  |  |  |  |  |  |  |
| 38.95 | 18 | igd | 1.00E-04 | A | \|\| id:ebi-a-GCST90001700 | ebi-a-GCST90001700 | TRUE | G | 26017285 | 9.31E-12 | reported | 3425 | 5.695 | rs75438005 |  |  |  |  |  |  |  |  |  |  |  |  |  |  |  |
| Supplementary Table 3: Causality between 178 leukocyte subsets and FGR. | | | | | | | | | | | | | | | | | | | | | |  |  |  |  |  |  |  |  |
| **Causality between 178 leukocyte subsets and FGR** | | | | | | | | | | | | | | | | | | | | | |  |  |  |  |  |  |  |  |
| **id.exposure** | **Trait** | **id.outcome** | **outcome** | **exposure** | **method** | **nsnp** | **b** | **se.x** | **pval.x** | **lo_ci** | **up_ci** | **or** | **or_lci95** | **or_uci95** | **P.FDR** | **Q** | **Q_df** | **Q_pval** | **egger_intercept** | **se.y** | **pval.y** |  |  |  |  |  |  |  |  |
| ebi-a-GCST90001982 | CCR2 on CD14- CD16+ monocyte | ukb-b-13378 | Birth weight \|\| id:ukb-b-13378 | \|\| id:ebi-a-GCST90001982 | IVW | 2 | -0.10357167 | 0.015954895 | 8.50E-11 | -0.134843263 | -0.072300076 | 0.901611402 | 0.873852866 | 0.930251707 | 5.77E-09 | 0.442097131 | 1 | 0.506111992 | NA | NA | NA |  |  |  |  |  |  |  |  |
| met-b-144 | CD27 on IgA+B | ukb-b-13378 | Birth weight \|\| id:ukb-b-13378 | \|\| id:met-b-144 | Wald ratio | 1 | -0.090261434 | 0.02110874 | 1.90E-05 | -0.131634565 | -0.048888304 | 0.913692283 | 0.876661299 | 0.952287491 | 2.47E-04 | NA | NA | NA | NA | NA | NA |  |  |  |  |  |  |  |  |
| ebi-a-GCST90001910 | CD45 on B cell | ieu-a-1083 | Birth weight \|\| id:ieu-a-1083 | \|\| id:ebi-a-GCST90001910 | IVW | 3 | -0.08706271 | 0.023933194 | 2.75E-04 | -0.133971772 | -0.040153649 | 0.916619612 | 0.874614753 | 0.960641826 | 3.07E-03 | 3.572835371 | 2 | 0.167559346 | 0.012598518 | 0.093712084 | 0.914923853 |  |  |  |  |  |  |  |  |
| met-b-146 | CD27 on IgG+B | ukb-b-13378 | Birth weight \|\| id:ukb-b-13378 | \|\| id:met-b-146 | Wald ratio | 1 | -0.085281631 | 0.019944152 | 1.90E-05 | -0.124372169 | -0.046191092 | 0.91825364 | 0.883051136 | 0.954859478 | 2.47E-04 | NA | NA | NA | NA | NA | NA |  |  |  |  |  |  |  |  |
| met-b-155 | CD27 on CD4 T | ukb-b-13378 | Birth weight \|\| id:ukb-b-13378 | \|\| id:met-b-155 | Wald ratio | 1 | -0.079731161 | 0.018646107 | 1.90E-05 | -0.116277531 | -0.043184791 | 0.923364549 | 0.890228123 | 0.957734393 | 2.47E-04 | NA | NA | NA | NA | NA | NA |  |  |  |  |  |  |  |  |
| ebi-a-GCST90001807 | CD27 on Plasma Blast-Plasma Cell | ukb-b-13378 | Birth weight \|\| id:ukb-b-13378 | \|\| id:ebi-a-GCST90001807 | Wald ratio | 1 | -0.078984501 | 0.018292116 | 1.57E-05 | -0.114837049 | -0.043131954 | 0.924054246 | 0.891511405 | 0.957784998 | 2.18E-04 | NA | NA | NA | NA | NA | NA |  |  |  |  |  |  |  |  |
| met-b-157 | CD27 on CD8 T | ukb-b-13378 | Birth weight \|\| id:ukb-b-13378 | \|\| id:met-b-157 | Wald ratio | 1 | -0.062243632 | 0.014556435 | 1.90E-05 | -0.090774244 | -0.03371302 | 0.939653929 | 0.913223854 | 0.966848931 | 2.47E-04 | NA | NA | NA | NA | NA | NA |  |  |  |  |  |  |  |  |
| ebi-a-GCST90001733 | CD19 on IgD- CD38- B cell | ukb-b-13378 | Birth weight \|\| id:ukb-b-13378 | \|\| id:ebi-a-GCST90001733 | Wald ratio | 1 | -0.057879916 | 0.01970537 | 3.31E-03 | -0.096502441 | -0.019257392 | 0.943763271 | 0.908007681 | 0.980926847 | 1.97E-02 | NA | NA | NA | NA | NA | NA |  |  |  |  |  |  |  |  |
| ebi-a-GCST90001992 | CCR2 on CD14+ CD16+ monocyte | ukb-b-13378 | Birth weight \|\| id:ukb-b-13378 | \|\| id:ebi-a-GCST90001992 | IVW (fixed) | 4 | -0.057608339 | 0.010741946 | 8.19E-08 | -0.078662553 | -0.036554125 | 0.944019611 | 0.924351792 | 0.96410591 | 2.48E-06 | NA | NA | NA | -0.016970767 | 0.022691401 | 0.532506371 |  |  |  |  |  |  |  |  |
| met-b-150 | CD32 on mDC | ukb-b-13378 | Birth weight \|\| id:ukb-b-13378 | \|\| id:met-b-150 | IVW | 3 | -0.05413579 | 0.013266575 | 4.49E-05 | -0.080138277 | -0.028133302 | 0.947303464 | 0.922988709 | 0.972258754 | 5.16E-04 | 1.217206114 | 2 | 0.54411043 | 0.042438089 | 0.038469123 | 0.468795142 |  |  |  |  |  |  |  |  |
| met-b-162 | CD32 on 11c+123+DC | ieu-a-1083 | Birth weight \|\| id:ieu-a-1083 | \|\| id:met-b-162 | Maximum likelihood | 3 | -0.047099824 | 0.018320726 | 1.01E-02 | -0.083008447 | -0.011191201 | 0.953992162 | 0.920343373 | 0.988871188 | 4.83E-02 | NA | NA | NA | -0.027983025 | 0.029478493 | 0.51656465 |  |  |  |  |  |  |  |  |
| ebi-a-GCST90001740 | CD19 on switched memory B cell | ukb-b-13378 | Birth weight \|\| id:ukb-b-13378 | \|\| id:ebi-a-GCST90001740 | IVW | 4 | -0.045075691 | 0.01400588 | 1.29E-03 | -0.072527215 | -0.017624167 | 0.955925124 | 0.930040435 | 0.98253023 | 9.31E-03 | 6.233688381 | 3 | 0.100778074 | -0.01478178 | 0.005955228 | 0.131130444 |  |  |  |  |  |  |  |  |
| ebi-a-GCST90001442 | CD24+ CD27+ B cell %lymphocyte | ieu-a-1083 | Birth weight \|\| id:ieu-a-1083 | \|\| id:ebi-a-GCST90001442 | IVW (random) | 4 | -0.043986395 | 0.004292678 | 1.22E-24 | -0.052400044 | -0.035572746 | 0.956966977 | 0.94894917 | 0.965052528 | 3.47E-22 | NA | NA | NA | 0.004015249 | 0.010704543 | 0.743630379 |  |  |  |  |  |  |  |  |
| ebi-a-GCST90002012 | CX3CR1 on CD14- CD16+ monocyte | ukb-b-13378 | Birth weight \|\| id:ukb-b-13378 | \|\| id:ebi-a-GCST90002012 | IVW | 3 | -0.040895527 | 0.009369929 | 1.27E-05 | -0.059260589 | -0.022530466 | 0.959929411 | 0.942461142 | 0.97772145 | 1.87E-04 | 1.42547052 | 2 | 0.49030126 | 0.068314003 | 0.064828262 | 0.483336735 |  |  |  |  |  |  |  |  |
| ebi-a-GCST90001439 | IgD+ CD24+ B cell %lymphocyte | ieu-a-1083 | Birth weight \|\| id:ieu-a-1083 | \|\| id:ebi-a-GCST90001439 | IVW (random) | 3 | -0.040737107 | 0.002372799 | 4.58E-66 | -0.045387792 | -0.036086421 | 0.960081496 | 0.955626825 | 0.964556932 | 3.25E-63 | NA | NA | NA | 0.000734953 | 0.014673612 | 0.968140443 |  |  |  |  |  |  |  |  |
| ebi-a-GCST90001723 | CD19 on CD24+ CD27+ B cell | ukb-b-13378 | Birth weight \|\| id:ukb-b-13378 | \|\| id:ebi-a-GCST90001723 | IVW (fixed) | 3 | -0.039008878 | 0.011348489 | 5.87E-04 | -0.061251917 | -0.016765839 | 0.961742171 | 0.94058626 | 0.983373925 | 5.01E-03 | NA | NA | NA | -0.014249111 | 0.005634549 | 0.239726769 |  |  |  |  |  |  |  |  |
| ebi-a-GCST90001799 | CD27 on T cell | ukb-b-13378 | Birth weight \|\| id:ukb-b-13378 | \|\| id:ebi-a-GCST90001799 | Wald ratio | 1 | -0.037243573 | 0.008709865 | 1.90E-05 | -0.054314909 | -0.020172238 | 0.963441438 | 0.947133799 | 0.980029861 | 2.47E-04 | NA | NA | NA | NA | NA | NA |  |  |  |  |  |  |  |  |
| ebi-a-GCST90002021 | CD14 on CD33dim HLA DR+ CD11b+ | ukb-b-13378 | Birth weight \|\| id:ukb-b-13378 | \|\| id:ebi-a-GCST90002021 | Wald ratio | 1 | -0.034300681 | 0.008021634 | 1.90E-05 | -0.050023083 | -0.018578279 | 0.966280919 | 0.951207468 | 0.981593234 | 2.47E-04 | NA | NA | NA | NA | NA | NA |  |  |  |  |  |  |  |  |
| ebi-a-GCST90002035 | CD80 on mDC | ukb-b-13378 | Birth weight \|\| id:ukb-b-13378 | \|\| id:ebi-a-GCST90002035 | IVW (fixed) | 15 | -0.03427772 | 0.004367423 | 4.21E-15 | -0.042837869 | -0.025717571 | 0.966303105 | 0.958066709 | 0.974610309 | 5.45E-13 | NA | NA | NA | -0.001982936 | 0.007643755 | 0.799374652 |  |  |  |  |  |  |  |  |
| ebi-a-GCST90001661 | CD28- CD8dim T cell %T cell | ieu-a-1083 | Birth weight \|\| id:ieu-a-1083 | \|\| id:ebi-a-GCST90001661 | IVW | 7 | -0.033735196 | 0.011833358 | 4.36E-03 | -0.056928578 | -0.010541814 | 0.966827491 | 0.944661537 | 0.989513557 | 2.65E-02 | 3.397472347 | 6 | 0.757556792 | 0.001188282 | 0.005944889 | 0.849447215 |  |  |  |  |  |  |  |  |
| ebi-a-GCST90002036 | CD80 on CD62L+ mDC | ieu-a-1083 | Birth weight \|\| id:ieu-a-1083 | \|\| id:ebi-a-GCST90002036 | IVW | 15 | -0.027282342 | 0.007696976 | 3.93E-04 | -0.042368415 | -0.012196269 | 0.973086459 | 0.958516584 | 0.987877804 | 3.93E-03 | 23.52727567 | 14 | 0.052212873 | 0.011694885 | 0.005573753 | 0.055994545 |  |  |  |  |  |  |  |  |
| ebi-a-GCST90001679 | CD25++ CD8+ T cell %T cell | ieu-a-1083 | Birth weight \|\| id:ieu-a-1083 | \|\| id:ebi-a-GCST90001679 | IVW | 2 | -0.026924027 | 0.010423341 | 9.79E-03 | -0.047353775 | -0.006494278 | 0.973435194 | 0.953749925 | 0.993526764 | 4.71E-02 | 0.000744331 | 1 | 0.978234473 | NA | NA | NA |  |  |  |  |  |  |  |  |
| ebi-a-GCST90001821 | IgD on IgD+ CD24- B cell | ieu-a-1083 | Birth weight \|\| id:ieu-a-1083 | \|\| id:ebi-a-GCST90001821 | IVW (fixed) | 17 | -0.026587151 | 0.005132999 | 2.22E-07 | -0.036647828 | -0.016526473 | 0.973763176 | 0.964015575 | 0.98360934 | 7.05E-06 | NA | NA | NA | -0.00036152 | 0.003778091 | 0.925034609 |  |  |  |  |  |  |  |  |
| ebi-a-GCST90002120 | CD8 on CD28- CD8+ T cell | ieu-a-1083 | Birth weight \|\| id:ieu-a-1083 | \|\| id:ebi-a-GCST90002120 | IVW | 11 | -0.024846295 | 0.007922826 | 1.71E-03 | -0.040375035 | -0.009317555 | 0.975459834 | 0.960429177 | 0.990725719 | 1.30E-02 | 11.65600808 | 10 | 0.308738003 | -0.00120244 | 0.009918543 | 0.906171235 |  |  |  |  |  |  |  |  |
| ebi-a-GCST90001774 | CD24 on transitional B cell | ieu-a-1083 | Birth weight \|\| id:ieu-a-1083 | \|\| id:ebi-a-GCST90001774 | IVW (random) | 4 | -0.024161469 | 0.004094036 | 3.60E-09 | -0.03218578 | -0.016137159 | 0.976128082 | 0.96832667 | 0.983992347 | 1.58E-07 | NA | NA | NA | 0.003206924 | 0.006694786 | 0.67918673 |  |  |  |  |  |  |  |  |
| ebi-a-GCST90001460 | pDC AC | ukb-b-13378 | Birth weight \|\| id:ukb-b-13378 | \|\| id:ebi-a-GCST90001460 | IVW | 6 | -0.023137824 | 0.00766753 | 2.55E-03 | -0.038166183 | -0.008109466 | 0.977127803 | 0.962552968 | 0.991923327 | 1.60E-02 | 4.7178806 | 5 | 0.451270937 | -0.015227039 | 0.009695345 | 0.191377813 |  |  |  |  |  |  |  |  |
| ebi-a-GCST90001763 | CD20 on transitional B cell | ukb-b-13378 | Birth weight \|\| id:ukb-b-13378 | \|\| id:ebi-a-GCST90001763 | IVW | 4 | -0.022026183 | 0.008540097 | 9.90E-03 | -0.038764774 | -0.005287593 | 0.978214622 | 0.961976965 | 0.994726362 | 4.37E-02 | 2.50016627 | 3 | 0.475261035 | -0.005665947 | 0.009400478 | 0.60792905 |  |  |  |  |  |  |  |  |
| ebi-a-GCST90002014 | CCR2 on CD62L+ mDC | ukb-b-13378 | Birth weight \|\| id:ukb-b-13378 | \|\| id:ebi-a-GCST90002014 | IVW (fixed) | 14 | -0.021857666 | 0.003543033 | 6.86E-10 | -0.028802011 | -0.014913321 | 0.978379482 | 0.971608814 | 0.985197332 | 3.15E-08 | NA | NA | NA | 0.002243416 | 0.00754512 | 0.771296376 |  |  |  |  |  |  |  |  |
| ebi-a-GCST90002045 | CD45 on CD33- HLA DR- | ukb-b-13378 | Birth weight \|\| id:ukb-b-13378 | \|\| id:ebi-a-GCST90002045 | IVW (random) | 2 | -0.021425214 | 0.003018267 | 1.26E-12 | -0.027341018 | -0.01550941 | 0.978802675 | 0.973029364 | 0.984610241 | 9.98E-11 | NA | NA | NA | NA | NA | NA |  |  |  |  |  |  |  |  |
| ebi-a-GCST90001401 | IgD- CD27- B cell AC | ieu-a-1083 | Birth weight \|\| id:ieu-a-1083 | \|\| id:ebi-a-GCST90001401 | IVW (random) | 2 | -0.021303217 | 0.002315206 | 3.53E-20 | -0.02584102 | -0.016765413 | 0.978922094 | 0.974490001 | 0.983374344 | 7.16E-18 | NA | NA | NA | NA | NA | NA |  |  |  |  |  |  |  |  |
| ebi-a-GCST90001820 | IgD on IgD+ CD24+ B cell | ukb-a-198 | Birth weight \|\| id:ukb-a-198 | \|\| id:ebi-a-GCST90001820 | IVW (fixed) | 37 | -0.019645116 | 0.004258408 | 3.96E-06 | -0.027991595 | -0.011298636 | 0.980546592 | 0.97239654 | 0.988764954 | 1.51E-04 | NA | NA | NA | -0.010819507 | 0.009925668 | 0.283137875 |  |  |  |  |  |  |  |  |
| ebi-a-GCST90002069 | CD4 on CD39+ secreting CD4 Treg | ukb-a-198 | Birth weight \|\| id:ukb-a-198 | \|\| id:ebi-a-GCST90002069 | Weighted median | 10 | -0.019633101 | 0.006860604 | 4.21E-03 | -0.033079886 | -0.006186317 | 0.980558373 | 0.96746127 | 0.993832779 | 3.42E-02 | NA | NA | NA | 0.009246283 | 0.005714263 | 0.144300721 |  |  |  |  |  |  |  |  |
| ebi-a-GCST90002087 | CD11c on mDC | ukb-a-198 | Birth weight \|\| id:ukb-a-198 | \|\| id:ebi-a-GCST90002087 | IVW | 13 | -0.019435848 | 0.005256517 | 2.18E-04 | -0.029738622 | -0.009133075 | 0.98075181 | 0.97069922 | 0.990908505 | 3.69E-03 | 12.33108688 | 12 | 0.419469292 | -0.005942412 | 0.004813822 | 0.242757468 |  |  |  |  |  |  |  |  |
| ebi-a-GCST90001768 | CD24 on IgD+ CD38+ B cell | ukb-a-198 | Birth weight \|\| id:ukb-a-198 | \|\| id:ebi-a-GCST90001768 | IVW (random) | 3 | -0.019360307 | 0.005919397 | 1.07E-03 | -0.030962325 | -0.007758289 | 0.9808259 | 0.969512099 | 0.992271729 | 1.23E-02 | NA | NA | NA | 0.002029435 | 0.007119939 | 0.823228521 |  |  |  |  |  |  |  |  |
| ebi-a-GCST90001996 | CX3CR1 on CD14+ CD16+ monocyte | ukb-b-13378 | Birth weight \|\| id:ukb-b-13378 | \|\| id:ebi-a-GCST90001996 | IVW (fixed) | 12 | -0.019030253 | 0.003606365 | 1.31E-07 | -0.026098729 | -0.011961777 | 0.981149679 | 0.974238899 | 0.988109481 | 3.75E-06 | NA | NA | NA | 0.005987175 | 0.00462798 | 0.224854046 |  |  |  |  |  |  |  |  |
| ebi-a-GCST90001748 | CD20 on IgD+ CD38- B cell | ukb-b-13378 | Birth weight \|\| id:ukb-b-13378 | \|\| id:ebi-a-GCST90001748 | IVW (random) | 5 | -0.018686058 | 0.006996089 | 7.56E-03 | -0.032398394 | -0.004973723 | 0.981487444 | 0.968120812 | 0.995038625 | 3.63E-02 | NA | NA | NA | -0.005884046 | 0.007747436 | 0.502790886 |  |  |  |  |  |  |  |  |
| ebi-a-GCST90002088 | CD11c on CD62L+ mDC | ukb-b-13378 | Birth weight \|\| id:ukb-b-13378 | \|\| id:ebi-a-GCST90002088 | IVW | 11 | -0.018459523 | 0.004118694 | 7.40E-06 | -0.026532162 | -0.010386883 | 0.981709811 | 0.973816723 | 0.989666874 | 1.22E-04 | 5.135686758 | 10 | 0.881931532 | 0.002465042 | 0.00405636 | 0.558407803 |  |  |  |  |  |  |  |  |
| ebi-a-GCST90001528 | CD33dim HLA DR+ CD11b- %CD33dim HLA DR+ | ieu-a-1083 | Birth weight \|\| id:ieu-a-1083 | \|\| id:ebi-a-GCST90001528 | IVW | 6 | -0.018281297 | 0.004290649 | 2.04E-05 | -0.026690969 | -0.009871625 | 0.981884792 | 0.973662086 | 0.99017694 | 3.40E-04 | 4.048488626 | 5 | 0.542456125 | 0.000485936 | 0.00704043 | 0.948285752 |  |  |  |  |  |  |  |  |
| ebi-a-GCST90001964 | FSC-A on pDC | ukb-b-13378 | Birth weight \|\| id:ukb-b-13378 | \|\| id:ebi-a-GCST90001964 | IVW | 7 | -0.016589363 | 0.006557102 | 1.14E-02 | -0.029441284 | -0.003737443 | 0.983547482 | 0.970987889 | 0.996269533 | 4.78E-02 | 3.16873502 | 6 | 0.787390343 | 0.000570479 | 0.004039587 | 0.893207946 |  |  |  |  |  |  |  |  |
| ebi-a-GCST90001830 | BAFF-R on CD20- B cell | ukb-b-13378 | Birth weight \|\| id:ukb-b-13378 | \|\| id:ebi-a-GCST90001830 | IVW (random) | 7 | -0.015624936 | 0.005439446 | 4.07E-03 | -0.02628625 | -0.004963621 | 0.984496501 | 0.974056226 | 0.995048678 | 2.31E-02 | NA | NA | NA | 0.001009519 | 0.004450251 | 0.829526773 |  |  |  |  |  |  |  |  |
| ebi-a-GCST90001701 | BAFF-R on CD20- CD38- B cell | ukb-b-13378 | Birth weight \|\| id:ukb-b-13378 | \|\| id:ebi-a-GCST90001701 | IVW (fixed) | 13 | -0.014691744 | 0.004684135 | 1.71E-03 | -0.023872649 | -0.005510839 | 0.985415653 | 0.976410049 | 0.994504318 | 1.17E-02 | NA | NA | NA | 0.002388629 | 0.003913208 | 0.553997445 |  |  |  |  |  |  |  |  |
| ebi-a-GCST90001745 | CD20 on CD24+ CD27+ B cell | ukb-b-13378 | Birth weight \|\| id:ukb-b-13378 | \|\| id:ebi-a-GCST90001745 | IVW | 14 | -0.013814419 | 0.005156901 | 7.39E-03 | -0.023921945 | -0.003706892 | 0.986280562 | 0.976361917 | 0.99629997 | 3.57E-02 | 11.83573886 | 13 | 0.541157179 | -0.001522135 | 0.004385944 | 0.734565303 |  |  |  |  |  |  |  |  |
| ebi-a-GCST90001761 | CD20 on switched memory B cell | ukb-b-13378 | Birth weight \|\| id:ukb-b-13378 | \|\| id:ebi-a-GCST90001761 | IVW (fixed) | 13 | -0.013277556 | 0.004752005 | 5.20E-03 | -0.022591486 | -0.003963625 | 0.986810202 | 0.977661791 | 0.996044219 | 2.76E-02 | NA | NA | NA | -0.002089204 | 0.007254464 | 0.778709075 |  |  |  |  |  |  |  |  |
| ebi-a-GCST90002052 | CD45 on IMDSCs | ieu-a-1083 | Birth weight \|\| id:ieu-a-1083 | \|\| id:ebi-a-GCST90002052 | IVW (random) | 5 | -0.012918293 | 0.00245024 | 1.35E-07 | -0.017720763 | -0.008115822 | 0.98716479 | 0.982435326 | 0.991917022 | 4.46E-06 | NA | NA | NA | -0.001060286 | 0.007019489 | 0.88952231 |  |  |  |  |  |  |  |  |
| ebi-a-GCST90001976 | FSC-A on NK T | ukb-b-13378 | Birth weight \|\| id:ukb-b-13378 | \|\| id:ebi-a-GCST90001976 | IVW (random) | 3 | -0.01277342 | 0.000890419 | 1.14E-46 | -0.014518641 | -0.011028198 | 0.987307814 | 0.985586246 | 0.98903239 | 1.62E-43 | NA | NA | NA | 0.000276442 | 0.016426789 | 0.989287498 |  |  |  |  |  |  |  |  |
| ebi-a-GCST90001995 | CX3CR1 on monocyte | ukb-b-13378 | Birth weight \|\| id:ukb-b-13378 | \|\| id:ebi-a-GCST90001995 | IVW (fixed) | 16 | -0.01266176 | 0.003330278 | 1.44E-04 | -0.019189105 | -0.006134414 | 0.987418063 | 0.980993834 | 0.993884363 | 1.47E-03 | NA | NA | NA | 0.003631788 | 0.006769039 | 0.600015386 |  |  |  |  |  |  |  |  |
| ebi-a-GCST90001516 | IMDSCs %CD33dim HLA DR- CD66b- | ukb-b-13378 | Birth weight \|\| id:ukb-b-13378 | \|\| id:ebi-a-GCST90001516 | IVW (random) | 2 | -0.012472015 | 0.002282627 | 4.66E-08 | -0.016945964 | -0.007998066 | 0.987605438 | 0.983196811 | 0.992033834 | 1.50E-06 | NA | NA | NA | NA | NA | NA |  |  |  |  |  |  |  |  |
| ebi-a-GCST90002118 | CD8 on CD28+ CD45RA- CD8+ T cell | ieu-a-1083 | Birth weight \|\| id:ieu-a-1083 | \|\| id:ebi-a-GCST90002118 | IVW | 37 | -0.01216944 | 0.003945476 | 2.04E-03 | -0.019902574 | -0.004436306 | 0.987904308 | 0.980294175 | 0.99557352 | 1.49E-02 | 38.83220426 | 36 | 0.343262185 | 0.003534756 | 0.002457115 | 0.159152665 |  |  |  |  |  |  |  |  |
| ebi-a-GCST90002084 | SSC-A on NK T | ukb-a-198 | Birth weight \|\| id:ukb-a-198 | \|\| id:ebi-a-GCST90002084 | IVW (random) | 2 | -0.01216051 | 0.004464635 | 6.45E-03 | -0.020911194 | -0.003409826 | 0.98791313 | 0.979305929 | 0.996595981 | 4.65E-02 | NA | NA | NA | NA | NA | NA |  |  |  |  |  |  |  |  |
| ebi-a-GCST90001665 | CD28+ CD45RA+ CD8dim T cell %CD8dim T cell | ieu-a-1083 | Birth weight \|\| id:ieu-a-1083 | \|\| id:ebi-a-GCST90001665 | MR Egger | 26 | -0.011748972 | 0.002877148 | 4.27E-04 | -0.017388182 | -0.006109763 | 0.988319777 | 0.98276212 | 0.993908864 | 4.14E-03 | 25.22935137 | 24 | 0.393384312 | 0.009151951 | 0.002550299 | 0.001479015 |  |  |  |  |  |  |  |  |
| ebi-a-GCST90001997 | CX3CR1 on CD14+ CD16- monocyte | ukb-b-13378 | Birth weight \|\| id:ukb-b-13378 | \|\| id:ebi-a-GCST90001997 | IVW (fixed) | 21 | -0.010263162 | 0.002788347 | 2.33E-04 | -0.015728323 | -0.004798002 | 0.989789324 | 0.984394721 | 0.99521349 | 2.25E-03 | NA | NA | NA | 0.003098084 | 0.005010409 | 0.543700165 |  |  |  |  |  |  |  |  |
| ebi-a-GCST90002102 | CD45RA on resting CD4 Treg | ieu-a-1083 | Birth weight \|\| id:ieu-a-1083 | \|\| id:ebi-a-GCST90002102 | MR Egger | 61 | -0.009898072 | 0.002241804 | 4.37E-05 | -0.014292008 | -0.005504135 | 0.990150753 | 0.985809637 | 0.994510985 | 6.71E-04 | 58.88883527 | 59 | 0.479586843 | 0.005127042 | 0.001458893 | 0.000853611 |  |  |  |  |  |  |  |  |
| ebi-a-GCST90001942 | CD25 on CD39+ secreting CD4 Treg | ieu-a-1083 | Birth weight \|\| id:ieu-a-1083 | \|\| id:ebi-a-GCST90001942 | IVW (random) | 5 | -0.009833127 | 0.00264562 | 2.02E-04 | -0.015018542 | -0.004647711 | 0.990215061 | 0.985093674 | 0.995363073 | 2.43E-03 | NA | NA | NA | -0.02944033 | 0.045676939 | 0.565121954 |  |  |  |  |  |  |  |  |
| ebi-a-GCST90002016 | CCR2 on CD62L+ pDC | ukb-b-13378 | Birth weight \|\| id:ukb-b-13378 | \|\| id:ebi-a-GCST90002016 | IVW (fixed) | 11 | -0.009629251 | 0.003715837 | 9.56E-03 | -0.016912291 | -0.00234621 | 0.990416962 | 0.983229919 | 0.99765654 | 4.26E-02 | NA | NA | NA | 0.008954064 | 0.00412041 | 0.057820813 |  |  |  |  |  |  |  |  |
| ebi-a-GCST90002015 | CCR2 on pDC | ukb-b-13378 | Birth weight \|\| id:ukb-b-13378 | \|\| id:ebi-a-GCST90002015 | IVW (fixed) | 11 | -0.009600036 | 0.003709777 | 9.66E-03 | -0.016871199 | -0.002328874 | 0.990445897 | 0.983270322 | 0.997673836 | 4.29E-02 | NA | NA | NA | 0.008932825 | 0.004132391 | 0.058908045 |  |  |  |  |  |  |  |  |
| ebi-a-GCST90001787 | CD25 on IgD- CD38- B cell | ukb-b-13378 | Birth weight \|\| id:ukb-b-13378 | \|\| id:ebi-a-GCST90001787 | IVW (random) | 12 | -0.009097139 | 0.003197365 | 4.44E-03 | -0.015363974 | -0.002830304 | 0.990944115 | 0.98475345 | 0.997173697 | 2.46E-02 | NA | NA | NA | 0.001236927 | 0.003532364 | 0.733474752 |  |  |  |  |  |  |  |  |
| ebi-a-GCST90001941 | CD25 on secreting CD4 Treg | ieu-a-1083 | Birth weight \|\| id:ieu-a-1083 | \|\| id:ebi-a-GCST90001941 | IVW (random) | 5 | -0.009075855 | 0.002497665 | 2.79E-04 | -0.013971279 | -0.00418043 | 0.990965207 | 0.986125866 | 0.995828295 | 3.11E-03 | NA | NA | NA | -0.027903498 | 0.041509837 | 0.549587495 |  |  |  |  |  |  |  |  |
| ebi-a-GCST90001664 | CD28+ CD45RA+ CD8dim T cell %T cell | ieu-a-1083 | Birth weight \|\| id:ieu-a-1083 | \|\| id:ebi-a-GCST90001664 | MR Egger | 37 | -0.008635264 | 0.002724005 | 3.16E-03 | -0.013974313 | -0.003296214 | 0.991401913 | 0.986122874 | 0.996709212 | 2.07E-02 | 38.98456576 | 35 | 0.295167983 | 0.005241695 | 0.001829331 | 0.007000578 |  |  |  |  |  |  |  |  |
| ebi-a-GCST90001782 | CD25 on IgD+ CD38- unswitched memory B cell | ukb-b-13378 | Birth weight \|\| id:ukb-b-13378 | \|\| id:ebi-a-GCST90001782 | IVW (random) | 9 | -0.008387325 | 0.003166007 | 8.07E-03 | -0.014592699 | -0.002181951 | 0.991647751 | 0.985513258 | 0.997820428 | 3.79E-02 | NA | NA | NA | 0.000410592 | 0.003217174 | 0.902034638 |  |  |  |  |  |  |  |  |
| ebi-a-GCST90002098 | CD45RA on naive CD4+ T cell | ieu-a-1083 | Birth weight \|\| id:ieu-a-1083 | \|\| id:ebi-a-GCST90002098 | MR Egger | 86 | -0.008336554 | 0.002314879 | 5.35E-04 | -1.29E-02 | -0.00379939 | 0.991698099 | 0.987208795 | 0.996207818 | 4.94E-03 | 82.04768253 | 84 | 0.539924653 | 0.004264205 | 0.001215907 | 0.000730242 |  |  |  |  |  |  |  |  |
| ebi-a-GCST90001794 | CD25 on IgD+ B cell | ukb-b-13378 | Birth weight \|\| id:ukb-b-13378 | \|\| id:ebi-a-GCST90001794 | IVW (random) | 12 | -7.98E-03 | 2.93E-03 | 6.48E-03 | -1.37E-02 | -2.23E-03 | 0.992053549 | 0.986371876 | 0.99776795 | 3.24E-02 | NA | NA | NA | 0.001470572 | 0.003006321 | 0.635279437 |  |  |  |  |  |  |  |  |
| ebi-a-GCST90001784 | CD25 on IgD+ CD38dim B cell | ukb-b-13378 | Birth weight \|\| id:ukb-b-13378 | \|\| id:ebi-a-GCST90001784 | IVW (random) | 13 | -0.007942928 | 0.002384795 | 8.66E-04 | -0.012617126 | -0.00326873 | 0.992088533 | 0.987462136 | 0.996736607 | 6.86E-03 | NA | NA | NA | 0.001462127 | 0.002503806 | 0.571026713 |  |  |  |  |  |  |  |  |
| ebi-a-GCST90001666 | CD28+ CD45RA+ CD8dim T cell AC | ukb-a-198 | Birth weight \|\| id:ukb-a-198 | \|\| id:ebi-a-GCST90001666 | MR Egger | 30 | -0.007816852 | 0.001682043 | 7.27E-05 | -0.011113656 | -0.004520047 | 0.992213621 | 0.988947873 | 0.995490153 | 1.47E-03 | 23.53508057 | 28 | 0.705820479 | 0.00596003 | 0.001533311 | 0.000568401 |  |  |  |  |  |  |  |  |
| ebi-a-GCST90001888 | CD28 on CD39+ secreting CD4 Treg | ukb-a-198 | Birth weight \|\| id:ukb-a-198 | \|\| id:ebi-a-GCST90001888 | IVW (random) | 18 | -0.007690761 | 0.002811894 | 6.24E-03 | -0.013202073 | -0.002179449 | 0.992338737 | 0.986884692 | 0.997822925 | 4.52E-02 | NA | NA | NA | 0.003197316 | 0.002685811 | 0.251234102 |  |  |  |  |  |  |  |  |
| ebi-a-GCST90001854 | CD3 on CD39+ activated CD4 Treg | ieu-a-1083 | Birth weight \|\| id:ieu-a-1083 | \|\| id:ebi-a-GCST90001854 | IVW (random) | 27 | -0.007426523 | 0.002684457 | 5.67E-03 | -0.012688058 | -0.002164988 | 0.992600985 | 0.987392096 | 0.997837353 | 3.22E-02 | NA | NA | NA | -0.003397326 | 0.002397984 | 0.168899591 |  |  |  |  |  |  |  |  |
| ebi-a-GCST90002119 | CD8 on CD28+ CD45RA+ CD8+ T cell | ieu-a-1083 | Birth weight \|\| id:ieu-a-1083 | \|\| id:ebi-a-GCST90002119 | IVW | 72 | -0.007035289 | 0.002393261 | 3.29E-03 | -0.01172608 | -0.002344497 | 0.992989401 | 0.988342403 | 0.997658249 | 2.13E-02 | 68.09126076 | 71 | 0.575868685 | 0.00311365 | 0.001618166 | 0.058397442 |  |  |  |  |  |  |  |  |
| ebi-a-GCST90001793 | CD25 on switched memory B cell | ukb-b-13378 | Birth weight \|\| id:ukb-b-13378 | \|\| id:ebi-a-GCST90001793 | IVW (random) | 12 | -0.006977587 | 0.002754551 | 1.13E-02 | -0.012376506 | -0.001578668 | 0.9930467 | 0.987699768 | 0.998422577 | 4.76E-02 | NA | NA | NA | 0.001130455 | 0.003386414 | 0.745409572 |  |  |  |  |  |  |  |  |
| ebi-a-GCST90001517 | CD33+ HLA DR+ AC | ukb-a-198 | Birth weight \|\| id:ukb-a-198 | \|\| id:ebi-a-GCST90001517 | IVW (random) | 2 | -0.00697443 | 0.000721918 | 4.42E-22 | -0.008389389 | -0.005559471 | 0.993049835 | 0.991645704 | 0.994455954 | 1.38E-19 | NA | NA | NA | NA | NA | NA |  |  |  |  |  |  |  |  |
| ebi-a-GCST90001518 | CD33+ HLA DR+ CD14- AC | ukb-a-198 | Birth weight \|\| id:ukb-a-198 | \|\| id:ebi-a-GCST90001518 | IVW (random) | 2 | -0.006832992 | 0.000672958 | 3.19E-24 | -0.008151991 | -0.005513994 | 0.993190299 | 0.991881147 | 0.99450118 | 1.24E-21 | NA | NA | NA | NA | NA | NA |  |  |  |  |  |  |  |  |
| ebi-a-GCST90001791 | CD25 on naive-mature B cell | ukb-b-13378 | Birth weight \|\| id:ukb-b-13378 | \|\| id:ebi-a-GCST90001791 | IVW (random) | 15 | -0.006693937 | 0.002596354 | 9.93E-03 | -0.011782791 | -0.001605083 | 0.993328417 | 0.988286354 | 0.998396204 | 4.37E-02 | NA | NA | NA | 0.001544237 | 0.002450225 | 0.539456593 |  |  |  |  |  |  |  |  |
| ebi-a-GCST90002109 | HLA DR on CD33+ HLA DR+ CD14dim | ukb-b-13378 | Birth weight \|\| id:ukb-b-13378 | \|\| id:ebi-a-GCST90002109 | IVW (fixed) | 36 | -0.00607668 | 0.002144893 | 4.61E-03 | -0.01028067 | -0.001872691 | 0.993941745 | 0.989771996 | 0.998129062 | 2.53E-02 | NA | NA | NA | -0.003597622 | 0.00390256 | 0.363096153 |  |  |  |  |  |  |  |  |
| ebi-a-GCST90002111 | HLA DR on CD33dim HLA DR+ CD11b- | ukb-b-13378 | Birth weight \|\| id:ukb-b-13378 | \|\| id:ebi-a-GCST90002111 | IVW (fixed) | 54 | -0.005554946 | 0.001687935 | 9.98E-04 | -0.008863298 | -0.002246594 | 0.994460454 | 0.991175866 | 0.997755928 | 7.65E-03 | NA | NA | NA | -0.003657564 | 0.00346761 | 0.296402368 |  |  |  |  |  |  |  |  |
| ebi-a-GCST90001485 | CD39+ resting CD4 Treg %CD4 Treg | ieu-a-1083 | Birth weight \|\| id:ieu-a-1083 | \|\| id:ebi-a-GCST90001485 | IVW | 143 | -0.005533575 | 0.001300499 | 2.09E-05 | -0.008082552 | -0.002984598 | 0.994481707 | 0.991950024 | 0.997019852 | 3.46E-04 | 165.715817 | 142 | 0.084592754 | -0.000650957 | 0.00104446 | 0.534128862 |  |  |  |  |  |  |  |  |
| ebi-a-GCST90001507 | CD25++ CD45RA+ CD4 not Treg AC | ieu-a-1083 | Birth weight \|\| id:ieu-a-1083 | \|\| id:ebi-a-GCST90001507 | IVW | 31 | -0.005479132 | 0.002021814 | 6.73E-03 | -0.009441888 | -0.001516376 | 0.994535852 | 0.990602547 | 0.998484774 | 3.63E-02 | 28.494779 | 30 | 0.544235035 | 0.003275213 | 0.002599743 | 0.217778287 |  |  |  |  |  |  |  |  |
| ebi-a-GCST90001536 | CD45RA- CD4+ T cell %T cell | ukb-b-13378 | Birth weight \|\| id:ukb-b-13378 | \|\| id:ebi-a-GCST90001536 | IVW (random) | 10 | -0.005104103 | 0.00165379 | 2.03E-03 | -0.00834553 | -0.001862675 | 0.994908901 | 0.991689197 | 0.998139059 | 1.34E-02 | NA | NA | NA | -0.004890533 | 0.003906725 | 0.245994399 |  |  |  |  |  |  |  |  |
| ebi-a-GCST90001508 | CD25++ CD45RA+ CD4 not Treg %CD4+ T cell | ieu-a-1083 | Birth weight \|\| id:ieu-a-1083 | \|\| id:ebi-a-GCST90001508 | IVW | 40 | -0.004927913 | 0.00188949 | 9.11E-03 | -0.008631313 | -0.001224514 | 0.995084209 | 0.99140583 | 0.998776236 | 4.47E-02 | 42.50459696 | 39 | 0.322561857 | 0.001662649 | 0.002119075 | 0.437546039 |  |  |  |  |  |  |  |  |
| ebi-a-GCST90001484 | CD39+ resting CD4 Treg %resting CD4 Treg | ieu-a-1083 | Birth weight \|\| id:ieu-a-1083 | \|\| id:ebi-a-GCST90001484 | IVW | 141 | -0.004784073 | 0.001468689 | 1.12E-03 | -0.007662704 | -0.001905442 | 0.995227353 | 0.99236658 | 0.998096373 | 9.31E-03 | 165.5573458 | 140 | 0.069106629 | -0.000778879 | 0.001293202 | 0.547964154 |  |  |  |  |  |  |  |  |
| ebi-a-GCST90001672 | CD39+ CD8+ T cell AC | ukb-b-13378 | Birth weight \|\| id:ukb-b-13378 | \|\| id:ebi-a-GCST90001672 | IVW (fixed) | 165 | -0.004781041 | 0.000929795 | 2.72E-07 | -0.006603438 | -0.002958643 | 0.99523037 | 0.993418316 | 0.99704573 | 6.65E-06 | NA | NA | NA | -0.001956244 | 0.001067039 | 0.068576287 |  |  |  |  |  |  |  |  |
| ebi-a-GCST90001489 | CD39+ activated CD4 Treg AC | ukb-b-13378 | Birth weight \|\| id:ukb-b-13378 | \|\| id:ebi-a-GCST90001489 | IVW (fixed) | 126 | -0.004341572 | 0.001127101 | 1.17E-04 | -0.006550691 | -0.002132453 | 0.995667839 | 0.993470718 | 0.997869819 | 1.23E-03 | NA | NA | NA | -0.002321522 | 0.001397044 | 0.099091351 |  |  |  |  |  |  |  |  |
| ebi-a-GCST90001658 | CD39+ CD4+ T cell %T cell | ukb-b-13378 | Birth weight \|\| id:ukb-b-13378 | \|\| id:ebi-a-GCST90001658 | IVW (fixed) | 163 | -0.004085807 | 0.000986233 | 3.43E-05 | -0.006018824 | -0.00215279 | 0.995922529 | 0.993999253 | 0.997849526 | 4.13E-04 | NA | NA | NA | -0.00153823 | 0.001190944 | 0.198345159 |  |  |  |  |  |  |  |  |
| ebi-a-GCST90001496 | CD39+ secreting CD4 Treg %secreting CD4 Treg | ukb-b-13378 | Birth weight \|\| id:ukb-b-13378 | \|\| id:ebi-a-GCST90001496 | IVW (fixed) | 160 | -0.004060546 | 0.000944963 | 1.73E-05 | -0.005912673 | -0.002208419 | 0.995947687 | 0.994104772 | 0.997794018 | 2.32E-04 | NA | NA | NA | -0.002214621 | 0.001133915 | 0.052577341 |  |  |  |  |  |  |  |  |
| ebi-a-GCST90001670 | CD39+ CD8+ T cell %T cell | ukb-b-13378 | Birth weight \|\| id:ukb-b-13378 | \|\| id:ebi-a-GCST90001670 | IVW (fixed) | 159 | -0.004020855 | 0.000935589 | 1.73E-05 | -0.00585461 | -0.0021871 | 0.995987218 | 0.994162495 | 0.99781529 | 2.32E-04 | NA | NA | NA | -0.001421274 | 0.00107298 | 0.187226952 |  |  |  |  |  |  |  |  |
| ebi-a-GCST90001659 | CD39+ CD4+ T cell %CD4+ T cell | ukb-b-13378 | Birth weight \|\| id:ukb-b-13378 | \|\| id:ebi-a-GCST90001659 | IVW (fixed) | 165 | -0.003832384 | 0.00096829 | 7.56E-05 | -0.005730233 | -0.001934536 | 0.99617495 | 0.994286154 | 0.998067334 | 8.31E-04 | NA | NA | NA | -0.001444237 | 0.00105922 | 0.174608074 |  |  |  |  |  |  |  |  |
| ebi-a-GCST90002112 | HLA DR on CD33- HLA DR+ | ukb-b-13378 | Birth weight \|\| id:ukb-b-13378 | \|\| id:ebi-a-GCST90002112 | IVW (fixed) | 67 | -0.003758775 | 0.001112563 | 7.29E-04 | -0.005939399 | -0.001578151 | 0.996248281 | 0.994078204 | 0.998423094 | 5.98E-03 | NA | NA | NA | -0.003426581 | 0.001793642 | 0.060494392 |  |  |  |  |  |  |  |  |
| ebi-a-GCST90001692 | CD28+ CD45RA- CD8+ T cell %CD8+ T cell | ukb-b-13378 | Birth weight \|\| id:ukb-b-13378 | \|\| id:ebi-a-GCST90001692 | IVW (fixed) | 35 | -0.003739603 | 0.00148724 | 1.19E-02 | -0.006654594 | -0.000824611 | 0.996267381 | 0.993367499 | 0.999175729 | 4.92E-02 | NA | NA | NA | -0.00177311 | 0.001423495 | 0.221682047 |  |  |  |  |  |  |  |  |
| ebi-a-GCST90001671 | CD39+ CD8+ T cell %CD8+ T cell | ukb-b-13378 | Birth weight \|\| id:ukb-b-13378 | \|\| id:ebi-a-GCST90001671 | IVW (fixed) | 155 | -0.003719688 | 0.00095444 | 9.73E-05 | -0.005590392 | -0.001848985 | 0.996287221 | 0.994425205 | 0.998152723 | 1.05E-03 | NA | NA | NA | -0.002193313 | 0.001129054 | 0.053899469 |  |  |  |  |  |  |  |  |
| ebi-a-GCST90001660 | CD39+ CD4+ T cell AC | ieu-a-1083 | Birth weight \|\| id:ieu-a-1083 | \|\| id:ebi-a-GCST90001660 | IVW (fixed) | 173 | -0.003638047 | 0.001345954 | 6.87E-03 | -0.006276117 | -0.000999978 | 0.996368562 | 0.993743537 | 0.999000522 | 3.68E-02 | NA | NA | NA | -0.001677895 | 0.001287469 | 0.194241192 |  |  |  |  |  |  |  |  |
| ebi-a-GCST90001512 | CD25++ CD45RA- CD4 not Treg %T cell | ukb-b-13378 | Birth weight \|\| id:ukb-b-13378 | \|\| id:ebi-a-GCST90001512 | IVW (random) | 10 | -0.003554272 | 0.001337022 | 7.85E-03 | -0.006174836 | -0.000933709 | 0.996452037 | 0.993844189 | 0.999066727 | 3.72E-02 | NA | NA | NA | 0.007489303 | 0.007477767 | 0.345891754 |  |  |  |  |  |  |  |  |
| ebi-a-GCST90002031 | CD39 on CD39+ secreting CD4 Treg | ukb-a-198 | Birth weight \|\| id:ukb-a-198 | \|\| id:ebi-a-GCST90002031 | IVW (fixed) | 127 | -0.003184443 | 0.001127269 | 4.73E-03 | -0.005393891 | -0.000974995 | 0.996820622 | 0.99462063 | 0.99902548 | 3.71E-02 | NA | NA | NA | -0.002253587 | 0.001316651 | 0.089449557 |  |  |  |  |  |  |  |  |
| ebi-a-GCST90001702 | BAFF-R on CD24+ CD27+ B cell | ukb-a-198 | Birth weight \|\| id:ukb-a-198 | \|\| id:ebi-a-GCST90001702 | IVW (fixed) | 197 | -0.003161274 | 0.001120471 | 4.78E-03 | -0.005357396 | -0.000965152 | 0.996843717 | 0.994656929 | 0.999035314 | 3.74E-02 | NA | NA | NA | 0.00183508 | 0.000937938 | 0.051833722 |  |  |  |  |  |  |  |  |
| ebi-a-GCST90001491 | CD39+ activated CD4 Treg %CD4 Treg | ukb-b-13378 | Birth weight \|\| id:ukb-b-13378 | \|\| id:ebi-a-GCST90001491 | IVW (fixed) | 128 | -0.003081804 | 0.001064442 | 3.79E-03 | -0.005168111 | -0.000995497 | 0.99692294 | 0.994845221 | 0.999004999 | 2.19E-02 | NA | NA | NA | -7.1334E-05 | 0.001166439 | 0.951332388 |  |  |  |  |  |  |  |  |
| ebi-a-GCST90001483 | CD39+ resting CD4 Treg AC | ukb-a-198 | Birth weight \|\| id:ukb-a-198 | \|\| id:ebi-a-GCST90001483 | IVW (fixed) | 123 | -0.003053882 | 0.001116688 | 6.24E-03 | -0.00524259 | -0.000865174 | 0.996950776 | 0.994771128 | 0.999135201 | 4.53E-02 | NA | NA | NA | -0.001596681 | 0.001019194 | 0.119817012 |  |  |  |  |  |  |  |  |
| ebi-a-GCST90002108 | HLA DR on CD33+ HLA DR+ CD14- | ukb-b-13378 | Birth weight \|\| id:ukb-b-13378 | \|\| id:ebi-a-GCST90002108 | IVW (fixed) | 102 | -0.002737624 | 0.001034378 | 8.13E-03 | -0.004765006 | -0.000710242 | 0.99726612 | 0.995246329 | 0.99929001 | 3.81E-02 | NA | NA | NA | -0.003014856 | 0.001925904 | 0.12064271 |  |  |  |  |  |  |  |  |
| ebi-a-GCST90002030 | CD39 on CD39+ activated CD4 Treg | ukb-b-13378 | Birth weight \|\| id:ukb-b-13378 | \|\| id:ebi-a-GCST90002030 | IVW (fixed) | 177 | -0.002245454 | 0.000844116 | 7.81E-03 | -0.003899921 | -0.000590987 | 0.997757065 | 0.996107674 | 0.999409188 | 3.71E-02 | NA | NA | NA | -0.00044948 | 0.001056216 | 0.670953436 |  |  |  |  |  |  |  |  |
| ebi-a-GCST90001699 | CD45RA+ CD28- CD8+ T cell %CD8+ T cell | ukb-b-13378 | Birth weight \|\| id:ukb-b-13378 | \|\| id:ebi-a-GCST90001699 | IVW | 2 | 0.002228697 | 0.000875633 | 1.09E-02 | 0.000512457 | 0.003944938 | 1.002231183 | 1.000512588 | 1.003952729 | 4.65E-02 | 0.074401564 | 1 | 0.785032712 | NA | NA | NA |  |  |  |  |  |  |  |  |
| ebi-a-GCST90001509 | CD25++ CD45RA+ CD4 not Treg %T cell | ukb-b-13378 | Birth weight \|\| id:ukb-b-13378 | \|\| id:ebi-a-GCST90001509 | IVW | 36 | 0.003355375 | 0.001334321 | 1.19E-02 | 0.000740106 | 0.005970644 | 1.003361011 | 1.00074038 | 1.005988504 | 4.92E-02 | 35.85298137 | 35 | 0.428276771 | 0.001055163 | 0.001443719 | 0.469868953 |  |  |  |  |  |  |  |  |
| ebi-a-GCST90001961 | CD25 on CD39+ CD4+ T cell | ukb-b-13378 | Birth weight \|\| id:ukb-b-13378 | \|\| id:ebi-a-GCST90001961 | IVW | 76 | 0.004139509 | 0.00128619 | 1.29E-03 | 0.001618577 | 0.006660441 | 1.004148089 | 1.001619888 | 1.006682672 | 9.31E-03 | 72.83919797 | 75 | 0.549165423 | -0.000362977 | 0.001125743 | 0.748033603 |  |  |  |  |  |  |  |  |
| ebi-a-GCST90002103 | CD45RA on CD39+ resting CD4 Treg | ukb-b-13378 | Birth weight \|\| id:ukb-b-13378 | \|\| id:ebi-a-GCST90002103 | IVW (random) | 10 | 0.004152943 | 0.000486482 | 1.38E-17 | 0.00319944 | 0.005106447 | 1.004161579 | 1.003204563 | 1.005119507 | 2.81E-15 | NA | NA | NA | 0.002855828 | 0.017654235 | 0.875502209 |  |  |  |  |  |  |  |  |
| ebi-a-GCST90002046 | CD45 on CD33- HLA DR+ | ukb-a-198 | Birth weight \|\| id:ukb-a-198 | \|\| id:ebi-a-GCST90002046 | IVW (random) | 2 | 0.004415403 | 0.001277548 | 5.48E-04 | 0.001911409 | 0.006919398 | 1.004425166 | 1.001913237 | 1.006943392 | 7.20E-03 | NA | NA | NA | NA | NA | NA |  |  |  |  |  |  |  |  |
| ebi-a-GCST90001736 | CD19 on memory B cell | ieu-a-1083 | Birth weight \|\| id:ieu-a-1083 | \|\| id:ebi-a-GCST90001736 | IVW (random) | 2 | 0.004449449 | 0.000400066 | 9.83E-29 | 0.00366532 | 0.005233577 | 1.004459362 | 1.003672046 | 1.005247296 | 3.49E-26 | NA | NA | NA | NA | NA | NA |  |  |  |  |  |  |  |  |
| ebi-a-GCST90001691 | CD28+ CD45RA- CD8+ T cell %T cell | ieu-a-1083 | Birth weight \|\| id:ieu-a-1083 | \|\| id:ebi-a-GCST90001691 | IVW (fixed) | 24 | 0.00561931 | 0.002105462 | 7.61E-03 | 0.001492604 | 0.009746017 | 1.005635128 | 1.001493718 | 1.009793664 | 3.94E-02 | NA | NA | NA | -0.008204224 | 0.007407882 | 0.280040457 |  |  |  |  |  |  |  |  |
| ebi-a-GCST90001989 | CD40 on CD14- CD16+ monocyte | ukb-b-13378 | Birth weight \|\| id:ukb-b-13378 | \|\| id:ebi-a-GCST90001989 | IVW (fixed) | 42 | 0.005904383 | 0.002016464 | 3.41E-03 | 0.001952113 | 0.009856652 | 1.005921848 | 1.00195402 | 1.009905389 | 2.01E-02 | NA | NA | NA | -0.00256936 | 0.002387784 | 0.288355301 |  |  |  |  |  |  |  |  |
| ebi-a-GCST90001953 | CD33 on CD33dim HLA DR- | ieu-a-1083 | Birth weight \|\| id:ieu-a-1083 | \|\| id:ebi-a-GCST90001953 | IVW | 38 | 0.006076351 | 0.002199874 | 5.74E-03 | 0.001764597 | 0.010388105 | 1.00609485 | 1.001766155 | 1.010442249 | 3.25E-02 | 39.81614187 | 37 | 0.34585565 | 0.001422194 | 0.001894923 | 0.457813996 |  |  |  |  |  |  |  |  |
| ebi-a-GCST90001954 | CD33 on basophil | ieu-a-1083 | Birth weight \|\| id:ieu-a-1083 | \|\| id:ebi-a-GCST90001954 | IVW | 42 | 0.006166197 | 0.002068501 | 2.87E-03 | 0.002111936 | 0.010220458 | 1.006185247 | 1.002114167 | 1.010272865 | 1.93E-02 | 40.30075598 | 41 | 0.501536688 | 0.000940457 | 0.001771212 | 0.598376712 |  |  |  |  |  |  |  |  |
[truncated: 197,943 more chars]
